# Phenotypic, histological and proteomic analyses reveal multiple differences associated with chloroplast development in yellow and variegated variants from *Camellia sinensis*

**DOI:** 10.1038/srep33369

**Published:** 2016-09-16

**Authors:** Chengying Ma, Junxi Cao, Jianke Li, Bo Zhou, Jinchi Tang, Aiqing Miao

**Affiliations:** 1 Tea Research Institute, Guangdong Academy of Agricultural Sciences, Guangzhou 510640, China; 2 Guangdong Provincial Key Laboratory of Tea Plant Resources Innovation & Utilization, Guangzhou 510640, China; 3 Institute of Apicultural Research/Key Laboratory of Pollinating Insect Biology, Ministry of Agriculture, Chinese Academy of Agricultural Sciences, Beijing 100093, China

## Abstract

Leaf colour variation is observed in several plants. We obtained two types of branches with yellow and variegated leaves from *Camellia sinensis*. To reveal the mechanisms that underlie the leaf colour variations, combined morphological, histological, ionomic and proteomic analyses were performed using leaves from abnormal branches (variants) and normal branches (CKs). The measurement of the CIE-Lab coordinates showed that the brightness and yellowness of the variants were more intense than the CKs. When chloroplast profiles were analysed, HY1 (branch with yellow leaves) and HY2 (branch with variegated leaves) displayed abnormal chloroplast structures and a reduced number and size compared with the CKs, indicating that the abnormal chloroplast development might be tightly linked to the leaf colour variations. Moreover, the concentration of elemental minerals was different between the variants and the CKs. Furthermore, DEPs (differentially expressed proteins) were identified in the variants and the CKs by a quantitative proteomics analysis using the label-free approach. The DEPs were significantly involved in photosynthesis and included PSI, PSII, cytochrome b6/f complex, photosynthetic electron transport, LHC and F-type ATPase. Our results suggested that a decrease in the abundance of photosynthetic proteins might be associated with the changes of leaf colours in tea plants.

The leaves of plants are the major photosynthetic organs that provide energy for plant development. The leaf colour, size, and shape directly affect photosynthesis, yield and quality. Generally, the normal leaf colour is green, which depends on stabilised chloroplast development, chlorophyll and the biosynthesis of other pigments. However, the leaf-colour variations, including chlorina, albino, and striata, are observed in many higher plant species and are applied in breeding, such as rice^1^,^2^, wheat^3^,^4^, oilseed rapa^5^ and *Camellia sinensis*^6^,^7^,^8^. These mutants mentioned above serve as a perfect material to reveal the underlying mechanism involved in chlorophyll biosynthesis^9^, chloroplast structure and function^1^, the regulation of chloroplast development, and photosynthesis^10^. Dissecting the underlying mechanism of the leaf colour variations is of great importance for theoretical significances and for broad application prospects.

The occurrence of the variations in leaf colour is mainly determined by genetic and environmental factors. These variants mainly confer a change from green to white and yellow colours according to their phenotypes. The formation of leaf colour depends on several processes, including chloroplast development, the number and size of chloroplasts, and chlorophyll biosynthesis. Thus, any defect in these processes can result in the loss of the green colour in the leaf. For the internal factors, a gene mutation, including nuclear genes and cytoplasmic genes and the restraining protein transport, can result in variations in leaf colour. To date, many genes involved in chloroplast development and chlorophyll biosynthesis have been identified through the leaf colour mutants^11^,^12^,^13^. Those genes directly or indirectly regulate the structure of chloroplasts, chlorophyll biosynthesis and several metabolic processes that affect the depth of leaf colour^14^,^15^. Additionally, changes in environmental factors, including temperature^6^,^16^, light^17^, and elemental minerals^18^, can also lead to the variations in leaf colour. Therefore, the variations in leaf colour are caused by one or/and more of the factors mentioned above, which leads to difficulty in studying the underlying mechanism of leaf colour.

The tea plant, *Camellia sinensis*, is an economically important genus cultivated in China, Japan, and Korea^19^. Among the diverse cultivars, many materials showing variations in leaf colour have been obtained to expand the germplasms. At present, two main types of variations in leaf colour were identified in tea plants, including albino and chlorina^6^,^7^. They exhibit highly improved economic value depending on changes in their biochemical composition^7^,^20^. Therefore, the elucidation of the molecular mechanism underlying colour formation is important for tea plants breeding with variable leaf colours. However, to date, only a few studies have reported the molecular mechanisms involved in the changes of leaf colour in tea plants^6^,^7^,^8^. These studies found that the differentially expressed genes and proteins involved in the metabolism of amino acids, nitrogen and sulfur, photosynthesis, flavonoid biosynthesis, and chlorophyll biosynthesis are the major driving forces for the leaf colour changes. Although some mechanisms related to leaf colour (chlorina) in tea plants have been reported, the materials used in the previous studies did not share the same genetic background^7^,^8^, which indicates that these materials were not optimum for studying the molecular mechanism. Thus, to gain a true mechanistic view into this issue, it is essential to obtain leaf colour variants with the same genetic background compared with the normal plants.

Proteomics has emerged as a powerful tool that facilitates the study of global protein expression and is widely used in plants to address specific biological responses^21^,^22^,^23^. Additionally, proteomic analyses were used in studies of the leaf colour of tea plants through two different approaches (2D gel electrophoresis and iTRAQ)^4^,^8^. Thus, large-scale proteomic data derived from the leaf colour variants in tea plants are the basic information for the underlying mechanism of variations in leaf colour. In this study, we adopted a label-free MS-based approach.

We obtained two types of branches showing yellow and variegated colours in leaves from a population of “Yinghongjiuhao”. “Yinghongjiuhao” is a tea cultivar that was selected from Yunnan Big leaf tea. Investigating these variants is helpful to explore the molecular mechanisms underpinning leaf colour formation. Here, the growth performances were observed, and the leaf colour was identified through the CIE-Lab model, which provides digitalisation and visualisation results. Then, the number and ultra-structure of the chloroplasts were analysed to find the relationship between leaf colour and the characteristics of the chloroplasts. Furthermore, the ionomics and proteomics were performed to explore the mechanism of the leaf colour variations. Our findings reveal the complex process of leaf colour formation, involving phenotype, ultra-structure, mineral ions and protein, which will improve our understanding of phenotype in the leaf colour variants.

## Results

### Characterisation of leaf colour variants and their corresponding CKs

**Figure 1:**
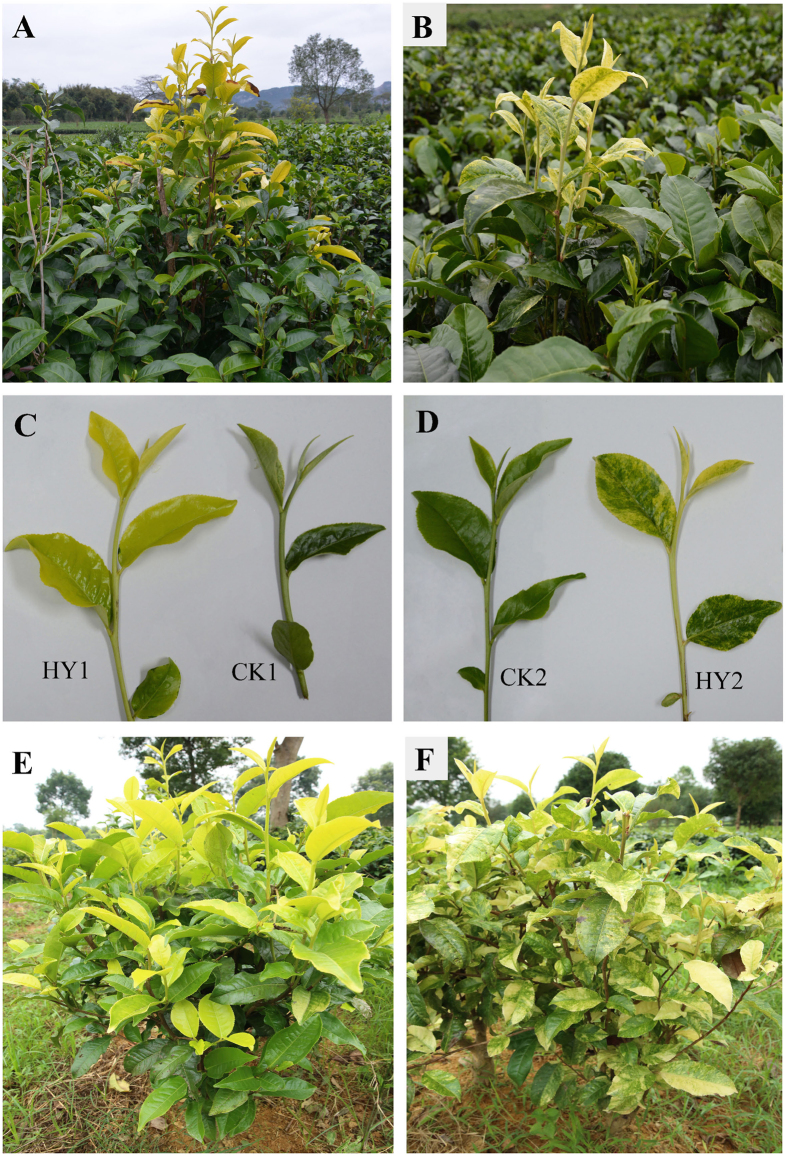
Phenotypes of leaves from the variants and the CKs of *Camellia Sinensis.* (**A**) Performance of HY1 (showing yellow leaf) and CK1 (showing green leaf) in the field; (**B**) Performance of HY2 (showing variegated leaf) and CK2 (showing green leaf) in the field; (**C**) a comparison of the characteristics of HY1 and its corresponding CK1; (**D**) a comparison of HY2 and its corresponding CK2; (**E**) the stable variation of HY1 in leaf colour through the grafting propagation; (**F**) the stable phenotype in leaf colour of HY2 through the grafting propagation.

Compared with CK1 and CK2, the leaves from HY1 (Fig. 1A,C) and HY2 (Fig. 1B,D) exhibited yellow and variegated colour at an early stage of leaf development, respectively, and then the leaves tended to revert to green along with increasing maturity. Additionally, the variations in leaf colour can be stably maintained through grafting propagation of variants (Fig. 1E,F).

To characterise the colour changes between the variants and the CKs during leaf development, the measurement of the CIE-Lab coordinates was carried out, and the average and standard deviation of the L*, a* and b* parameters were calculated (Table 1). Due to the variegated colour at the different scanned points, the standard deviation of L*, a* and b* in HY2 was high. The L* and b* colour parameters showed that the brightness and yellowness, respectively, of the variants were more intense than the CKs. Moreover, the lower a* during leaf development indicated that the leaves from HY1 and HY2 were gradually greening, and the greenness of the four-leaf from HY2 (−8.59) approximated CK2 (−8.96), and at the same time, HY1’s (−3.39) did not achieve the level of CK1 (−8.82).

### Profiles of chloroplasts in the variants and their CKs

**Figure 2:**
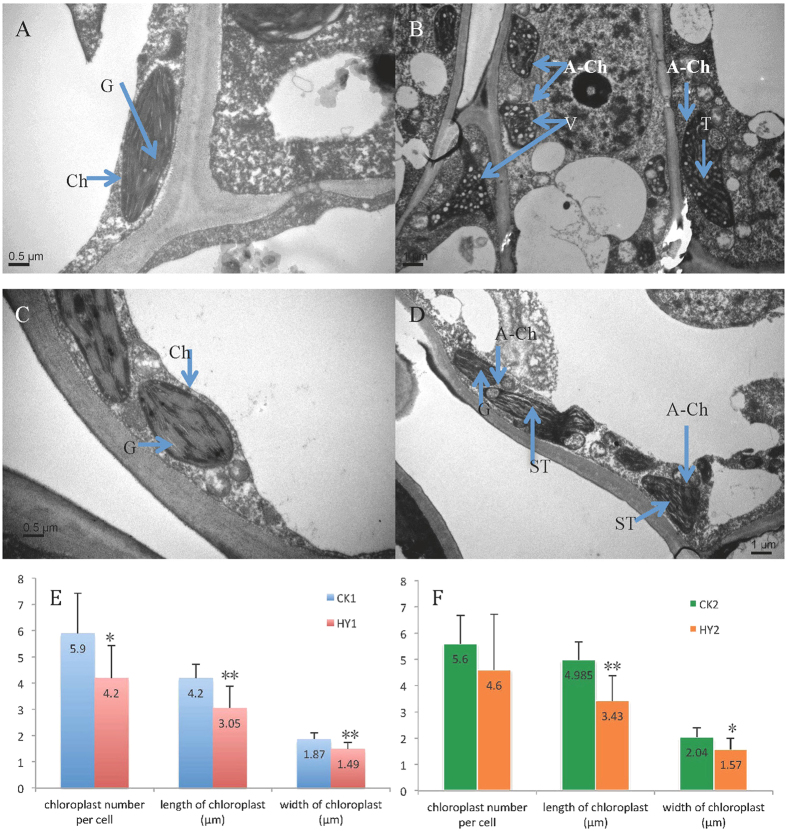
Chloroplast profiles of the variants and the CKs. (**A–D**) Chloroplast ultrastructure of the variants and the CKs; (**A,C**) chloroplast ultrastructure of CK1 and CK2, respectively (Bar = 0.5 μm); (**B,D**) Chloroplast ultrastructure of HY1 and HY2, respectively (Bar = 1 μm). (**E,F**) the difference of the number, length and width of the chloroplasts in the variants and the CKs; (**E**) a comparison of the number, length and width of the chloroplasts between HY1 and CK1; (**F**) a comparison of the number, length and width of the chloroplasts between HY2 and CK2. In these pictures, Ch refers to the chloroplast; **G** refers to the grana; A-Ch refers to an abnormal chloroplast; T refers to the thylakoid; ST refers to a swelling thylakoid; V refers to a vesicle; **indicates a significant difference (*P* < 0.01); *refers to a significant difference at the 0.05 level.

To further identify the relationship between the chloroplasts and leaf colour variations in our study, the number, size and ultrastructure of the chloroplasts were investigated using the second leaves from the one bud and four leaves stage. The chloroplasts showed well-developed membrane systems composed of grana connected by stroma lamellae in CK1 and CK2 (Fig. 2A,C). However, in HY1, the chloroplasts lacked well-structured thylakoid membranes, and some of the chloroplasts contained irregularly arranged vesicles, which led to a decrease of the number of thylakoids and the disappearance of the grana (Fig. 2B). In particular, a few chloroplasts in HY1 were almost completely filled with vesicles and almost no inner member structures (Fig. 2B). In the HY2, normal, or close normal chloroplasts, were observed; although, abnormal chloroplasts with swelling thylakoid and the disappearance of stacks of the thylakoid also existed (Fig. 2D). Moreover, the shape of the chloroplasts in CK1 and CK2 mainly displayed an ellipse, while those in HY1 and HY2 appeared as abnormal shapes (Fig. 2B,D). Additionally, there were significant differences in the number, length and width of the chloroplasts between HY1 and CK1, while in HY2 and CK2 insignificant differences in number were found (Fig. 2E,F).

As described above, we found that the leaves of HY1 and HY2 gradually turned green in colour with the maturity of the leaves. As shown in Fig. S1, the dysfunctional structures of the chloroplasts gradually improved in the leaves with different maturity in HY1 and HY2, containing an increased number of lamellar structured and a well-structured thylakoid, which is consistent with the change in leaf colour. For example, in the first leaf of HY1, the chloroplast displayed a swelling thylakoid, while stacks of well-structured thylakoids were observed in the fourth leaf (Fig. S1A and S1D).

### Ionomics on the leaves of the variants and the CKs

To identify the relationship between ion accumulation in leaves and leaf colour variation, ionome profiling was performed on the leaves of the variants and the CKs (Table 2). The concentrations of various macro- and microelements were observed in two comparisons. The Mn concentrations in two types of CKs were higher than in their corresponding variants. The concentrations of Na, K, Ca, Fe, As, Mo and Pb increased in two variants. The changes in the concentrations of Mg, Cr, Ni, Cu, Zn and Cd in the two comparisons showed a different tendency.

### Quantitative identification of proteins from leaves of the variants and the CKs

To gain a global view of the molecular responses to leaf colour variations, total proteins in leaves were extracted from the variants and the corresponding CK branches in three independent biological experiments, and the protein expression profiles were explored using the label-free MS-based technique. We have deposited the LC-MS/MS data to the ProteomeXchange Consortium via the PRIDE partner repository with the data set identifier PXD004750. Overall, 1,185, 1,006, 2,280 and 1,836 proteins were identified in HY1, CK1, HY2 and CH2, respectively (Tables S2–5). The numbers of peptides, the mass, the sequence coverage and the description of the proteins are also provided. Additionally, among identified proteins, the number of proteins identified by one unique peptide with only one spectrum was 97, 255, 142 and 361 in CK1, CK2, HY1 and HY2, respectively, and the annotated spectra corresponding to proteins were shown in Fig. S2-5.

**Figure 3:**
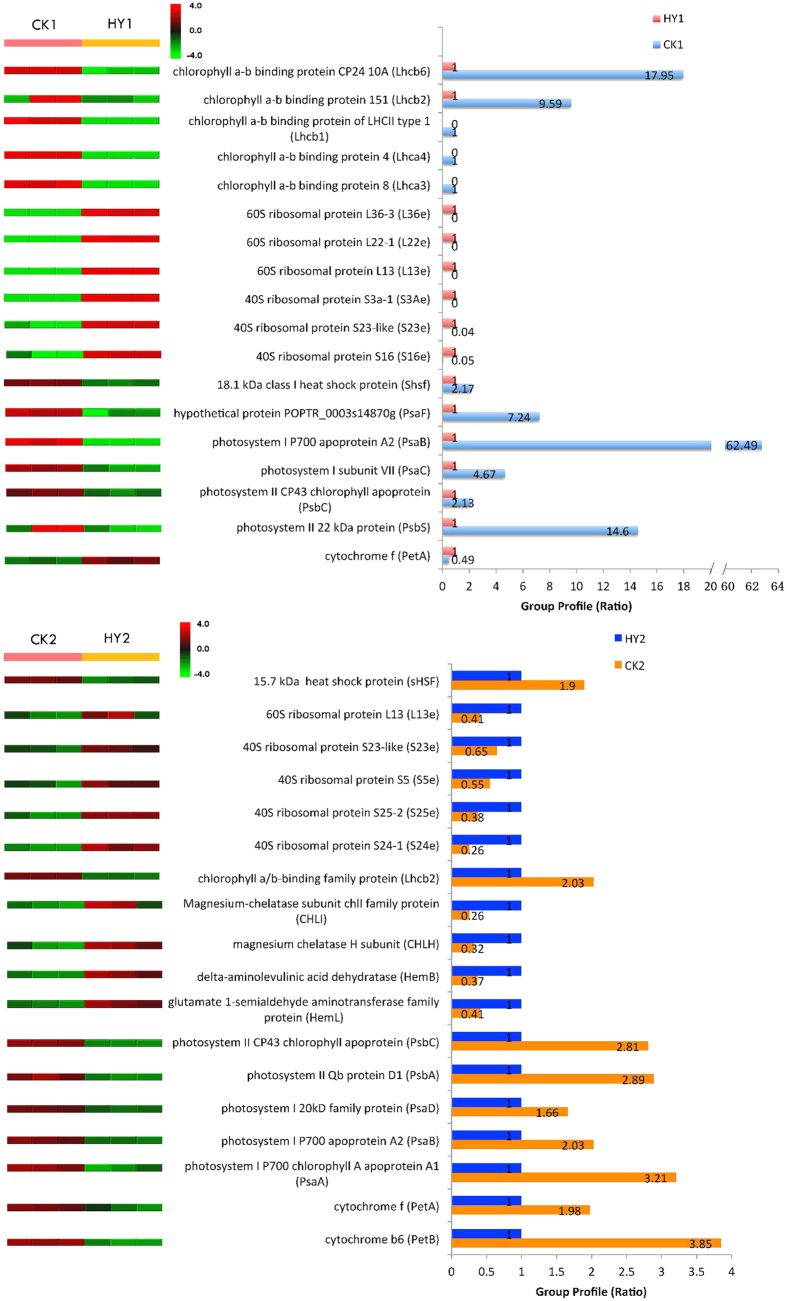
Quantitative comparison of several differentially expressed proteins between the variants and the CKs. The right panels represent the fold change of the individual protein, and the left panels represent the reproducibility in three independent biological experiments. The up- or down-regulated proteins are indicated by the red and green colour code, respectively. The colour intensity changes with the protein expressional level.

Changes in protein abundance in response to HY1 and HY2 variations were analysed, and 93 and 202 proteins were significantly different (Tables S6 and S7). Among the proteins identified, 45 and 32 proteins decreased their expression level to less than 0.67-fold with the yellow and variegation variations, respectively. However, the expression level of 48 and 170 proteins increased in HY1 and HY2, respectively (Tables S6 and S7). For example, as shown in Fig. 3, the expression of chlorophyll a-b binding proteins, heat shock proteins and proteins related to photosystem all decreased in HY1 and HY2, whereas the expression of ribosomal proteins in the two types of variants was significantly increased. However, cytochrome f protein showed a different expression pattern in the two comparisons, and four proteins related to chlorophyll synthesis, including CHLI, CHLH, HemB and HemL, were only found in the comparison between HY2 and CK2 (Fig. 3; Figs S6 and S7; Tables S6 and S7).

**Figure 4:**
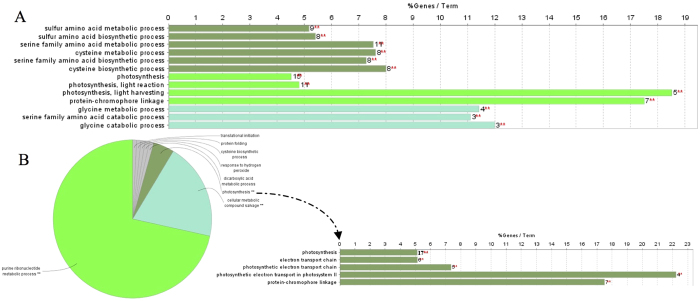
ClueGO analysis of differentially expressed proteins. Single (*) or double (**) asterisk indicate significant enriched GO terms at the p < 0.05 and p < 0.01 statistical levels, respectively. The numbers of corresponding genes associated with a specific term are indicated. The percentage of genes associated with a specific term is listed on the bars. (**A**) Enriched GO terms of differentially proteins identified from the comparison of HY1 and CK1. (**B**) The left pie chart indicates overview specific cluster of differentially expressed proteins between HY2 and CK2. The right represents specific Go terms related to photosynthesis.

To elucidate the possible different biological events behind the proteomic data, all the DEPs (differentially expressed proteins) in the two comparisons were translated into *Vitis vinifera* orthologues (Tables S8 and S9) and were analysed using ClueGO with the *Vitis vinifera* database to detect the significantly enriched GO terms. In the comparison of HY1 and CK1, serine family amino acid metabolic process, photosynthesis and glycine metabolic process were significantly enriched (Fig. 4A). In another comparison, the proteins were enriched in eight major functional groups, and among these, photosynthesis was also significantly enriched (Fig. 4B; Fig. S8).

**Figure 5:**
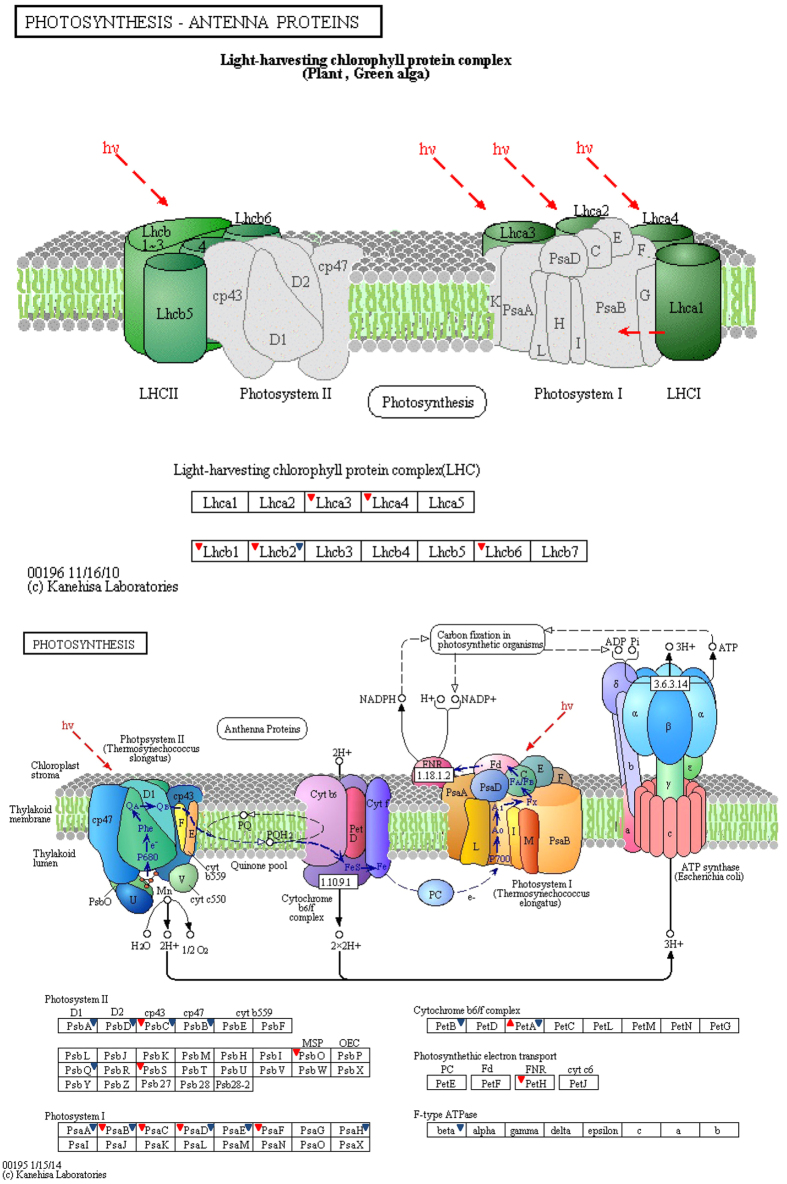
Mapping differentially expressed proteins with known Photosynthesis pathway. The images of known photosynthesis pathway were obtained from freely available KEGG database (www.kegg.jp). Regular triangle with red colour refers to higher expression in HY1 compared with CK1, while inverted triangles with red colour indicate the higher expression in CK1. Blue inverted triangles refer to lower expression of proteins in HY2 compared to CK2.

For a better understanding of the biological process of the DEPs, these proteins were further investigated using the KEGG database. The DEPs in the two comparisons were mapped to 50 and 63 KEGG pathways, respectively (Tables S10 and S11). The biological pathways involved in ribosome (16), photosynthesis (9) and photosynthesis – antenna proteins (5) were significantly enriched between HY1 and CK1 (Table 3). Moreover, four processes, including carbon metabolism (22), carbon fixation in photosynthetic organisms (11), photosynthesis (13) and glycolysis/gluconeogenesis (12), were significantly enriched between HY2 and CK2 (Table 3). Additionally, one protein derived from the DEPs between HY2 and CK2 was also annotated in photosynthesis – antenna proteins (Table S11). Moreover, almost all the proteins mapped in the photosynthesis pathway showed decreased expression in the two variants compared to their CKs, except for cytochrome f between CK1 and HY1 (Table 4). To survey the differences in photosynthesis between the variants and the CKs, we overlaid each protein profile onto a photosynthesis pathway (Fig. 5). The results showed that 14 differentially expressed proteins were associated with photosynthesis between HY1 and CK1, including 4 proteins in PSI, 3 proteins in PSII, 1 protein in the cytochrome b6/f complex, 1 protein in the photosynthetic electron transport, and 5 proteins in LHC; whereas between HY2 and CK2, 14 proteins, including 5 proteins in PSI, 5 proteins in PSII, 2 proteins in the cytochrome b6/f complex, 1 protein in the F-type ATPase, and 1 protein in LHC, were observed. These proteins may therefore be associated with the leaf colour variations.

### Transcriptional expression analysis of the differentially expressed proteins

**Figure 6:**
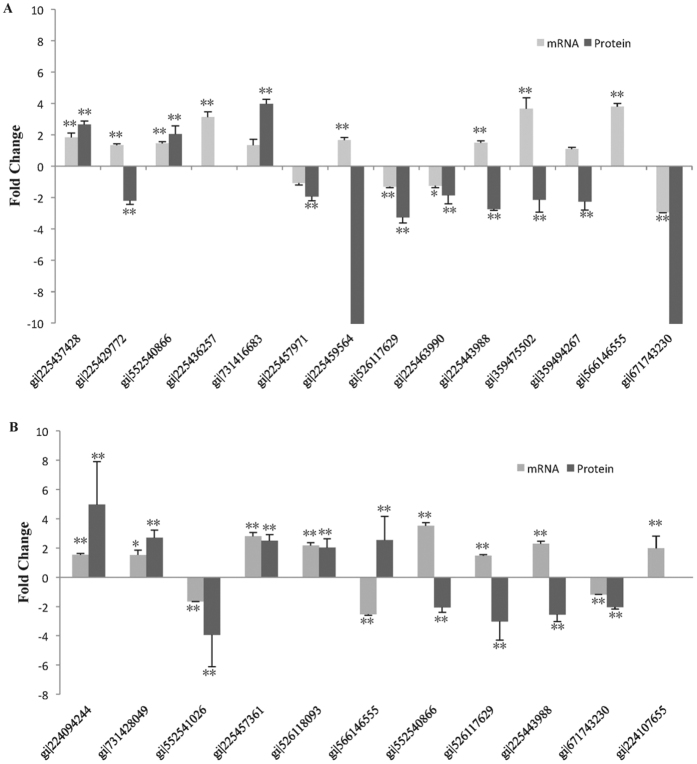
Quantitative real time PCR analyses of fourteen and eleven differentially expressed proteins at mRNA level, respectively. (**A**) The black and the gray bars represent fold changes of protein and mRNA between HY1 and CK1, respectively. The positive values indicate higher expression in the HY1 and negative values denote higher expression in CK1. (**B**) The black and the gray bars represent fold changes of protein and mRNA between HY2 and CK2, respectively. The positive values indicate higher expression in the HY2 and negative values denote higher expression in CK2. Error bar is standard deviation. *indicates significant difference (*P* < 0.05) at mRNA level, **refers to significant difference at 0.01 level at mRNA and protein level.

In order to assess the correlation of the expression levels between mRNA and protein, fourteen and eleven proteins were randomly selected, respectively, in the two comparisons and were analysed by quantitative RT-PCR (Fig. 6). Between HY1 and CK1, the expression of seven genes (gi|225437428, gi|552540866, gi|731416683, gi|225457971, gi|526117629, gi|225463990 and gi|671743230) is consistent with the corresponding proteins, while six genes (gi|224094244, gi|731428049, gi|552541026, gi|225457361, gi|526118093 and gi|671743230) showed similar protein and mRNA expression patterns in the comparison of HY2 and CK2 (Fig. 6; Tables S6 and S7). Additionally, the proteins (gi|225436257 and gi|566146555) were not detected in HY1 and CK1, respectively, while the protein (gi|224107655) was not detected in CK2. And the fold change in proteins expression of gi|225459564 and gi|671743230 more than ten. The QRT-PCR demonstrated that these genes displayed similar protein and mRNA expression patterns with divergent quantitative values. Additionally, other genes showed different expression patterns between the mRNAs and proteins, which might be a result of posttranscriptional and posttranslational regulatory processes.

## Discussion

Leaves with a green colour are the primary sites of photosynthesis and contribute to the biosynthesis of plant biomass and energy^24^,^25^. Currently, two types of abnormal branches showing yellow and variegated colour in leaves are observed from the tea cultivar “Yinghongjiuhao,” which leads to more uniform fermenting and a better presentation of tea in production; however, the mechanism of these variations is not fully understood. Accordingly, to reveal the mechanism, combined morphological, histological, ionomic and proteomic analyses were performed using leaves from the variants and their corresponding CKs. We found that the leaf colour in HY1 and HY2 both exhibited gradual greening along with increasing maturity. Moreover, abnormal chloroplast structures and a reduced number and size of chloroplasts were observed in the two variants. Finally, the difference in the concentration of elemental minerals and the protein expression between the variants and the CKs might be associated with the leaf colour changes.

### The present variants are suitable materials for analysing the mechanism of leaf colour variations

The mechanism of leaf colour variations is a complicated biological process. As an excellent model in such previous studies, leaf colour mutants have gained more and more attention. In the present study, two types of variants, showing yellow leaf and variegated leaves, were selected as the materials. They were suitable for mechanism exploration because they are of the same genetic background compared with the CK branches. Furthermore, the leaf colour variations in HY1 and HY2 might be caused by some unknown mutation(s) and might belong to bud mutation, because of the very low frequency, the random and the stability of leaf colour variations. Thus, in our opinion HY1 and HY2 might be mutants derived from the CKs. Moreover, leaf colour was traditionally identified by subjective judgements in previous studies of leaf colour variations. In this study, the leaf colour variations were quantified from colour parameters (CIE Lab) using a chromameter, which facilitated objective results and helped us to accurately distinguish the differences of leaf colour among the different samples, especially the samples from the different developmental stages. The above results indicate that suitable samples were prepared for mechanism exploration of leaf colour variations in tea plants.

### Leaf colour reflected the developmental characteristics of the chloroplast

Chloroplasts, composed of chloroplast membrane, thylakoid and matrix, is essential for carbon assimilation and amino acid synthesis^26^. The chloroplast profiles, including the structure, number and size are relatively stable, but they are apt to be influenced by genetic and environmental conditions^27^. Most affected plants with dysfunctional chloroplasts usually have leaves that lose their green colour. Thus, changes in leaf colour might reflect the abnormal development and function of the plastid. In our study, abnormal chloroplast structures and the improvement of abnormal chloroplasts structure were observed through lateral and vertical comparative assessments, respectively (Fig. 2; Fig. S1), which strongly suggests a relationship between the chloroplast and leaf colour. A previous study reported that abnormal chloroplasts might only be located on the variational positions, and our data showed that both normal and abnormal chloroplasts existed in the leaf of HY2, which could explain the phenotype of the variegated leaf. In addition, more types of abnormal chloroplasts were observed in the present variants than in those reported by Wang and Li^6^,^7^,^8^, and the number and size of the chloroplasts was firstly analysed in the leaf colour variants of the tea plants (Fig. 2). In HY1, a significantly decreased size of the chloroplast and a decreased number were observed, which indicates that the size and number of the chloroplasts are also related to leaf colour variations. These multiple comparisons provide more convincing results than in previous studies^6^,^7^,^8^. These findings demonstrate that the interruption of chloroplast development might be tightly linked to occurrence of the leaf colour variations. Additionally, the metabolism of ion might be influenced in leaves of variants, and several kinds of ions were associated with the chloroplast development. Based on the present data, the differences in the concentration of the elemental minerals in leaves were observed. Furthermore, the changes in concentration of Mn and Zn may be associated with the chloroplast development. For example, Zn plays a role in the formation of chloroplasts, and Mn is essential in the formation and maintenance of the normal structure of chloroplasts^28^.

### Proteins expression patterns and leaf colour variations

The present variants showed abnormal leaf colour and chloroplast profiles, while also displaying changes in protein expression patterns. Proteomic profiling provides information regarding quantitative changes in protein expression, which will advance our understanding of the role of proteins involved in leaf colour variations. For tea plants, there have been only two reports regarding a protein analysis for the changes in leaf colour. One of the studies identified twenty-six differentially expressed proteins in three developmental stages of the albino tea cultivar using a comparative proteomic approach based on two-dimensional electrophoresis and mass spectrometry. However, the disadvantage of the 2D gel technique limited its application to a comprehensive analysis of proteome changes^29^,^30^,^31^,^32^. Fortunately, MS-based high-resolution proteomic approaches are a powerful tool for large-scale protein identification and quantitation and are successfully utilized for the comprehensive characterisation of the proteome. Currently, two main types of relative quantification strategies for MS-based proteomics analysis exist, including label-based and label-free MS-based approaches^33^. In another study of leaf colour from tea plants, iTRAQ (label-based) was used to analyse the differentially expressed proteins, which overcame the disadvantages of the 2D gel technique and identified more proteins^8^. It is reported that MS-based label-free quantitative proteomics studies are reliable, versatile, and a cost-effective alternative to label-based quantitation^22^, and the label-free method allows for a qualitative analysis based on the number of identified proteins. Conversely, the iTRAQ methodology does not allow for the identification of unique proteins because the protein ratio is only calculated when the protein is present in both of the tested conditions^34^. Additionally, Latosinska *et al*.^35^ reported that the label-free strategy provided a higher protein sequence coverage and ultimately detected a higher number of significant changes compared to the iTRAQ experiment^35^. Therefore, in this study, we adopted a quantitative proteomic method using the label-free MS based system, and a total of 6307 proteins were identified in the leaves of the variants and the corresponding CKs (Tables S2–S5). In agreement with a previous study, several unique proteins were only detected in one sample, such as chlorophyll a-b binding protein of LHCII type 1 (gi|359483839) and 3-oxoacyl-[acyl-carrier-protein]reductase (gi|224107655), which were identified in HY1 and CK1 comparison and in HY2 and CK2 comparison, respectively (Tables S6 and S7). Thus, the greater number of proteins identified in our study will provide an effective information pool to identify the related proteins involved in leaf colour variations. In our study, two types of variants sharing the same genetic background were selected to analyse the differences in the proteins, which facilitated the identification of the proteins involved in leaf colour variations because of the common property between the yellow and variegated leaf.

The chloroplast profile assays demonstrated that an abnormal structure was observed in HY1 and HY2 compared with the CKs (Fig. 2). Thus, we speculate that changes in protein expression might be related to the chloroplast development in the two variants. The biogenesis of chloroplasts is a complex process that is tightly regulated by the coordinated expression of both nuclear and plastid genes and requires communication among the nucleus, cytoplasm, chloroplast, and mitochondrion^36^,^37^. To date, many genes involved in chloroplast development have been identified, such as the plastid-encoded genes *Lhcb, rbcL, rbcS, psaA* and *psbA*^38^,^39^. Several lines of evidence demonstrate that the chlorotic phenotype is closely associated with a reduced level of plastid genes^40^,^41^,^42^. For example, impaired grana stacking and a reduced number of chloroplasts in the mutants are a result of the significantly decreased level of LHC proteins^43^,^44^. In our study, the expression levels of the proteins corresponding to the above five plastid-encoded genes, chlorophyll a-b binding protein, ribulose bisphosphate carboxylase, photosystem I P700 chlorophyll A apoprotein A1, and photosystem II Qb protein D1, were remarkably repressed in the HY2 (Table 4). Additionally, heat shock protein also affects the chloroplast development by regulating protein folding and protein transport^45^,^46^. Consistent with the previous findings, reduced expression of the heat shock proteins in the variants was also observed in our study (Fig. 3; Tables S6 and S7). Thus, we speculated that the abnormal chloroplast profiles in the present variants might be associated with the downregulation of the above-mentioned proteins.

Photosynthesis takes place in the chloroplast. Thus, abnormal chloroplast profiles might be linked to a dramatic down-regulation of proteins related to the photosystem. Consistent with this theory, the DEPs were both significantly enriched in the photosynthesis pathway in the two comparisons. The expression of these DEPs, including PSI subunits, PSII subunits, antenna proteins, cytochrome b6/f complex, and the beta F-type ATPase, was decreased in HY1 and/or HY2 (Fig. 5). The relationship between the leaf colour variations and the expression patterns of proteins was revealed through the proteomic analysis, which facilitated the exploration of the mechanism of leaf colour formation.

## Conclusions

In conclusion, two types of leaf colour variations (yellow and variegated leaf colour) are observed from the tea cultivar “Yinghongjiuhao” in our study. To reveal the mechanisms, combined morphological, histological, ionomic and proteomic analyses were performed between variants and their corresponding CKs. The measurement of the CIE-Lab coordinates showed that HY1 (yellow colour in leaves) and HY2 (variegated colour in leaves) exhibited more brightness and yellowness according to the L* and b* colour parameters, and gradual greening was observed with increasing maturity. Moreover, the results of the chloroplast analyses indicated that abnormal chloroplast development was related to leaf colour variations. In addition, the changes in the concentration of elemental minerals may be associated with the leaf colour variations. Finally, a quantification of proteins with leaf colour variations was performed. A total of 93 and 202 DEPs in HY1 and HY2, respectively, were identified. Many pathways potentially associated with chloroplast development were revealed, such as photosynthesis. Further studies should mainly focus on the origin of the different observations showed in our present results between variants and CKs. We speculated that the ultimate cause of phenotype variations in our study must be a mutation (or mutations), which led to a series of changes including organelle and molecular levels and might ultimately regulate the formation of leaf colour in tea plants.

## Materials and Methods

### Plant Materials

The two leaf-colour variation branches of tea (*Camellia sinensis*) were derived from the cultivar “Yinghongjiuhao,” which was grown in the field of the Tea Research Institute at the Guangdong Academy of Agricultural Sciences in Yingde, China. The branches with yellow leaves and variegated leaves were named HY1 and HY2, respectively, and the remaining normal corresponding branches of the same plant were named CK1 and CK2 (See the details in Fig. 1). Additionally, the very low frequency of variations (approximately 0.001%) was observed in our tea plantation (about 800000 plants) based on three years statistics. All the leaves samples were harvested with three independent biological replicates, and the specific samples used for the different analyses were derived from the same sample pool.

### Leaf colour determination of the variants and the CKs

To better illustrate the leaf colour differences between the variants and the CKs, the leaves at the developmental stage of one bud and four leaves were harvested as samples. The colour coordinates (CIE Lab) of the sampled leaves were quantified by a portable spectrophotometer (Datacolor CHECK II) (Datacolor, US). The leaf colour variations were also detected using leaves from four leaf positions of the variants and the CKs, which illustrate the changes in the leaf colour corresponding with the developmental stages. To determine leaf colour exactly, the samples were scanned in triplicate at three different locations (sections) away from the main leaf vein. Following the measurement, the mean of nine values was calculated as the averaged colour data for each sample. The statistical analyses were performed by one-way ANOVA (SPSS version 19.0, SPSS, Inc.) using Duncan’s multiple-range test.

### Transmission electron microscopic (TEM) analysis of the chloroplasts

The ultrastructure of the chloroplasts from the leaves at the different developmental stages in the variants and the CKs was investigated via TEM, which clarified the basic explanation for the occurrence of varied characters. The samples were collected from the four leaf positions as described above. The differences between the variants and the CKs were elucidated using the second leaf. In addition, the profiles of the chloroplasts in the variants at different developmental stages were also analysed. The fresh samples were cut into 2 mm-wide pieces and were fixed with 2.5% glutaraldehyde overnight at 4 °C. Following a wash with phosphate buffer (0.1 M, pH 7.0, three times) for 15 min each time, the samples were incubated in 1% OsO4 for 2 h and washed again with 0.1 M phosphate buffer three times. Subsequently, the samples were subjected to a dehydration series in ascending concentrations of ethanol (30%, 50%, 70%, 80%, 90% and 95%), with each concentration step lasting 15 min, and were then soaked in 100% ethanol for 20 min. The dehydrated samples were drenched in acetone for 20 min followed by a drenching in epoxy resin and acetone (v/v = 1/1) for 1 h and epoxy resin and acetone (v/v = 3/1) for 3 h. The samples were finally embedded in a pure epoxy resin at 70 °C overnight. The thick sections (90 nm) were cut and stained with lead citrate and uranyl acetate in 50% ethanol for 10 min, respectively, and were visualised with a Hitachi H-7650 transmission electron microscope (Hitachi, Tokyo, Japan).

### Multi-element analysis

To identify the relationship between elemental minerals and leaf colour variation, ionomics were performed using one bud and two leaves from the variants and the CKs. The freeze-dried samples were digested with HNO_3_-H_2_O_2_, and the final volume was 50.0 ml. Then, the metal concentrations of the digests were measured using an Agilent 7700x ICP-MS (Agilent Technologies, Palo Alto, CA). The ICP-MS operating parameters for the analysis were as follows: RF power 1500 W; RF matching 1.8 V; carrier gas 1.08 L/min; plasma gas 14.97 L/min; nebulizer pump 0.1 rps; and sample depth 8 mm. All the samples and the blank solutions were measured in triplicate. The statistical significance of differences of concentration of ions within the comparison were analysed using one-way ANOVA (SPSS version 19.0, SPSS, Inc.).

### Protein Sample Preparation

Fresh leaves from the four materials (two variants and two CKs) in three independent biological replicates at the development stage of one bud and two leaves were collected for the proteomic analysis. Approximately 3 g of leaves from each biological replicate with 0.4 g of PVP (polyvinylpyrrolidone) and 0.1 g of DTT (dithiothreitol) were ground into a fine powder in liquid nitrogen and were suspended in 8 volumes of ice-cold acetone containing 10% (v/v) TCA (trichloroacetic acid) and 0.07% (v/v) β-Me (β-mercaptoethanol). After an intermittent ultrasonic treatment that was repeated 50 times (time for each treatment was 5 s, with a 15 s interval) on ice, the mixture was kept at −20 °C for 1 h and centrifuged at 15,000 x *g* for 15 min at 4 °C. Then, the supernatant was discarded, and the pellets were washed twice with 6 volumes of ice-cold acetone containing 0.07% β-Me, vacuum-dried and stored at −80 °C. The dried protein powders (50 mg of each sample) were re-suspended in 750 μL of lysis buffer (8 M urea, 30 mM DTT, 4% CHAPS, 2 M thiourea, and 20 mM Tris-base), vortexed, incubated at 37 °C for 2 h and centrifuged at 15,000 x *g* for 15 min at 4 °C. Four volumes of ice-cold acetone (containing 5 mM β-Me) were added to the above collected supernatant, and the mixture was kept at 4 °C for 2 h for protein precipitation and desalting. Subsequently, the mixture was centrifuged at 15,000 x *g* and 4 °C for 15 min. The supernatant was discarded, and the pellets were vacuum-dried. The pellets were dissolved in the lysis buffer described above and were incubated at 37 °C for 2 h. After centrifugation at 15,000 x *g* and 4 °C for 15 min, an aliquot of each supernatant was used to determine the protein concentration using the Bradford assay. A total of 1.5 mg of the dried pellets was dissolved thoroughly in 100 μL of 5 M urea buffer, and then 400 μL of a 40 mM NH_4_HCO_3_ buffer was added to the mixture. The proteins were reduced with 50 μL of 100 mM DTT and were then alkylated with 100 mM iodoacetamide. The proteins were digested using trypsin at a mass ratio of 1:50 (enzyme/protein) at 37 °C for 14 h. The enzymatic digestion was stopped by adding 1 μL of formic acid to the solution, and the samples were then vacuum-dried using a SpeedVac system (RVC 2-18, Marin Christ, Osterod, Germany). The samples were redissolved in 0.1% formic acid. The sample solution was centrifuged, and the supernatant was collected for the MS/MS analysis.

### Nano-LC−MS/MS Analysis

The analysis was performed according to a method described by Han, *et al*.^47^. Briefly, each of the biological replicates was analysed using a Q-Exactive mass spectrometer coupled to a two-column EASY-nLC 1000 nanoflow system (Thermo Fisher Scientific). The samples were loaded onto a 2-cm long, 100-μm inner diameter fused silica trap column containing 5.0 μm Aqua C18 beads (Thermo Fisher Scientific) for 2 min in buffer A (0.1% acetic acid) at a flow rate of 5 μL/min prior to the analytical separation. A 15-cm fused silica trap column (75 μm i.d.) filled with 3.0 μm Aqua C18 beads (Thermo Fisher Scientific) was selected as the stationary phase, while 0.1% formic acid (A) and 0.1% formic acid in 80% acetonitrile (B) was chosen as the binary mobile phase. The peptides were separated by the following gradient elution: from 0 to 8% solvent B for 5 min; from 8 to 20% solvent B for 55 min; from 20 to 30% solvent B for 10 min; from 30 to 100% solvent B for 10 min; and 100% solvent B for 15 min. The eluate from the column was analysed with a Q-Exactive mass spectrometer (Thermo Fisher Scientific) using an interface with an ESI ionisation source. The MS and MS/MS information was collected in data-dependent mode using the following settings: one full scan (resolution 70 000 at *m*/*z* 400; m/z300−1800) followed by the top 20 MS/MS scans using a high-energy collision dissociation in the linear ion trap mass spectrometer (resolution: 17 500, isolation window: 2 *m*/*z*, normalized collision energy: 27) using dynamic exclusion (charge exclusion: unassigned 1, > 8; peptide match: preferred; exclude isotopes: on; dynamic exclusion: 10 s).

### Protein Identification and Label-Free Abundance Quantitation

The raw peptide data were retrieved using Xcalibur software (version 2.2, Thermo Fisher Scientific) and were analysed and processed using the in-house PEAKS software (version 7.0, Bioinformatics Solutions). A composite database was downloaded with a total of 85,307 entities, containing 38,134 protein sequences of *Vitis vinifera*, 45,942 protein sequences of *Populus trichocarpa* and 1,231 sequences from *Camellia*. The search criteria were as follows: parent ion mass tolerance, 20.0 ppm; fragment ion mass tolerance, 0.05 Da; enzyme, trypsin; allowing a nonspecific cleavage at neither end of the peptide; maximum missed cleavages per peptide, 2; and maximum allowed variable post translational modification (PTM) per peptide, 3. A fusion target-decoy approach was used for the estimation of the false discovery rate (FDR) and was controlled at ≤1.0% (−10 log P ≥ 20.0) at both the protein and peptide level.

The relative quantification of the peptide abundance level was performed by the label-free approach in the PEAKS Q module. Feature detection was performed separately on each sample by using the expectation-maximization algorithm^48^. The features of the same peptide from different samples were aligned together using a high-performance retention time alignment algorithm^49^. The identification results were chosen so that they would attach as the last step of the label-free quantification. The peptide features and proteins were considered to be significantly changed between different samples using statistical p value < 0.01 and a fold change ≥ 1.5.

### Bioinformatics Analysis

To create an expressional profile of the DEPs, unsupervised hierarchical clustering was analysed (gene cluster 3.0) using an uncentered Pearson correlation and an average linkage and was visualised by Java Treeview software.

To better understand the biological implications of the DEPs, we assigned the *Vitis vinifera* orthologues of the tea DEPs a UniProt identifier by searching the UniProt database, due to the high homology of the amino acid sequences between the tea plant and *Vitis vinifera,* and the more effective database of *Vitis vinifera*. Subsequently, the unique identifiers of the *Vitis vinifera* orthologues of the tea DEPs were used as an input for functional analyses using CluGO (version 2.1.6), a Cytoscape plug-in^50^. The significantly enriched, functional gene ontology (GO) categories in biological processes were reported using a right-sided hypergeometric test. This test compares the background set of the GO annotations in the entire *Vitis vinifera* database and then assesses the FDR by a Bonferroni stepdown test to correct the probability value. The results are represented visually in graphical form.

Additionally, the DEPs were analysed by the KEGG Orthology - Based Annotation System (KOBAS 2.0, http://kobas.cbi.pku.edu.cn) to significantly enrich the identified proteins in biological pathways. The DEPs were firstly translated into the *Vitis vinifera* orthologues and were blasted against the *Vitis vinifera* database, and the pathway enrichment was then conducted by a hypergeometric statistical test. Only a corrected p value < 0.05 was considered a statistically significant enriched biological pathway.

### Quantitative Real-Time RT-PCR

Total RNA was extracted from one bud and two leaves of HY1, CK1, HY2 and CK2 using the RNAprep Pure Plant Kit (TIANGEN) and was converted to cDNA for qRT-PCR using the PrimeScript RT Reagent Kit (Takara). All the amplification reactions were performed with specific primers (Table S1) using 20-μl volumes of SYBR Green (Takara) and the ABI StepOne Real-Time PCR system. The 18 S ribosomal RNA gene was selected as a reference gene for normalisation^7^,^8^. Three biological replicates were included for each sample, and three technical replicates per each replicate were performed. And finally the fold change was calculated for each gene.

## Additional Information

**How to cite this article**: Ma, C. *et al*. Phenotypic, histological and proteomic analyses reveal multiple differences associated with chloroplast development in yellow and variegated variants from *Camellia sinensis. Sci. Rep.* **6**, 33369; doi: 10.1038/srep33369 (2016).

## Supplementary Material

Supplementary Information

## Figures and Tables

**Table 1:** Average data and standard deviation of the L*, a* and b* parameters of leaves from different leaf positions in the variants and the CKs.

Sample		L*	a*	b*
HY1	1^st^ leaf	66.04 ± 0.76a	1.67 ± 1.14a	56.78 ± 0.79b
	2^nd^ leaf	66.83 ± 1.43aE	3.33 ± 2.56aE	58.34 ± 1.83abE
	3^rd^ leaf	66.08 ± 1.43a	1.17 ± 3.19a	59.87 ± 2.15a
	4^th^ leaf	62.95 ± 0.96b	−3.39 ± 2.53b	53.69 ± 3.11c
CK1	1^st^ leaf	46.63 ± 1.23a	−7.67 ± 1.50ab	27.90 ± 2.98a
	2^nd^ leaf	41.15 ± 1.60bF	−7.13 ± 0.58 aF	24.26 ± 2.09bF
	3^rd^ leaf	39.87 ± 2.18bc	−8.00 ± 0.26bc	22.60 ± 2.73bc
	4^th^ leaf	39.26 ± 1.47c	−8.82 ± 0.61c	21.63 ± 1.39c
HY2	1^st^ leaf	67.49 ± 0.64a	−1.39 ± 0.74a	45.72 ± 4.39a
	2^nd^ leaf	64.31 ± 4.58aE	−3.53 ± 2.54bE	48.17 ± 5.06aE
	3^rd^ leaf	53.8 ± 4.00b	−6.17 ± 1.34c	35.70 ± 2.93b
	4^th^ leaf	52.18 ± 4.15b	−8.59 ± 0.41d	34.51 ± 6.14b
CK2	1^st^ leaf	41.49 ± 1.23a	−9.48 ± 0.42c	24.97 ± 2.11a
	2^nd^ leaf	40.75 ± 0.83abF	−8.01 ± 0.89 aF	24.19 ± 1.43 aF
	3^rd^ leaf	39.56 ± 1.39b	−8.61 ± 0.61ab	22.08 ± 1.69b
	4^th^ leaf	40.66 ± 1.97ab	−8.96 ± 0.73bc	23.91 ± 2.43ab

L*: brightness, 0% (no reflection) for black-coloured objects and 100% for white-coloured objects; a*: redness, with negative values for green and positive values for red; and b*: yellowness, with negative values for blue and positive values for yellow. Within the same columns, a, b, c, d refer to significant difference (*P* < 0.05) among the different developmental stages of the same sample. E, F in the same columns between HY1 and CK1, HY2 and CK2, respectively, indicate significant difference (*P* < 0.01) in the 2^nd^ leaf stage.

**Table 2:** Concentration of fourteen types of elemental minerals in the second leaves of CK1, HY1, CK2 and HY2.

	CK1	HY1	CK2	HY2
Na (g/kg)	0.09 ± 0.001B	0.13 ± 0.004A	0.03 ± 0.01	0.03 ± 0.006
Mg (g/kg)	1.85 ± 0.039B	2.2 ± 0.035A	2.37 ± 0.058	1.89 ± 0.227
K (g/kg)	18.33 ± 0.294B	21.36 ± 0.331A	20.6 ± 3.589	21.81 ± 2.95
Ca (g/kg)	2.37 ± 0.037B	2.81 ± 0.08A	3.63 ± 0.684	3.71 ± 0.509
Cr (mg/kg)	0.21 ± 0.066	0.28 ± 0.064	0.26 ± 0.023	0.2 ± 0.055
Mn (mg/kg)	678.61 ± 8.522	661.45 ± 7.215	464.4 ± 13.013	443.76 ± 39.674
Fe (mg/kg)	46.69 ± 0.947b	49.21 ± 0.177a	0.06 ± 0.012	0.07 ± 0.007
Ni (mg/kg)	3.83 ± 0.071	2.94 ± 0.036	3 ± 0.091	3.81 ± 0.514
Cu (mg/kg)	10.37 ± 0.248B	11.21 ± 0.147A	13.76 ± 0.427	13.63 ± 1.629
Zn (mg/kg)	22.89 ± 0.389A	20.93 ± 0.106B	14.84 ± 0.144B	36.78 ± 0.808A
As (mg/kg)	0.03 ± 0.004	0.04 ± 0.003	0.05 ± 0.015	0.06 ± 0.009
Mo (mg/kg)	0.02 ± 0.015B	0.08 ± 0.099A	0.02 ± 0.004	0.04 ± 0.02
Cd (mg/kg)	0.03 ± 0.006B	0.06 ± 0.004A	0.09 ± 0.016a	0.06 ± 0.009b
Pb (mg/kg)	0.37 ± 0.035b	0.48 ± 0.029a	0.31 ± 0.165	0.6 ± 0.114

The values, expressed as g/kg or mg/kg dry weight, are the mean ± SD of three independent experimental replicates. The different lowercase letters in the same row within the comparison (CK1 and HY1, CK2 and HY2, respectively) indicate a significant difference (*P* < 0.05), and the uppercase letters refer to a significant difference at the 0.01 level.

**Table 3:** Significantly enriched pathways among differentially expressed proteins.

	Pathway	Number of the mapped DEPs	Number of reference proteins	Corrected P value
HY1 VS. CK1	Ribosome	16	256	0.001225464
	Photosynthesis	9	84	0.001225464
	Photosynthesis- antenna proteins	5	19	0.001225464
HY2 VS. CK2	Carbon metabolism	22	156	0.001632539
	Carbon fixation in photosynthetic organisms	11	52	0.004375366
	Photosynthesis	13	84	0.009634559
	Glycolysis/Gluconeogenesis	12	74	0.009634559

**Table 4:** Identified differentially expressed proteins involved in photosynthesis.

Accession	Protein	Gene	CK1/HY1 (Ratio)	CK2/HY22 (Ratio)
gi|671743315	photosystem II CP43 chlorophyll apoprotein (chloroplast)	PsbC	2.13:1.00	2.81:1.00
gi|225459564	PREDICTED: photosystem II 22 kDa protein chloroplastic	PsbS	14.60:1.00	/
gi|224084209	O_2_ evolving complex 33kD family protein	PsbO	2.99:1.00	/
gi|552540956	photosystem II Qb protein D1 (chloroplast)	PsbA	/	2.89:1.00
gi|568244554	photosystem II protein D2 (plastid)	PsbD	/	2.02:1.00
gi|542688125	photosystem II p680 chlorophyll A apoprotein CP-47 (chloroplast)	PsbB	/	2.49:1.00
gi|224078826	Oxygen-evolving enhancer protein 3-1	PsbQ	/	1.50:1.00
gi|671743230	photosystem I P700 apoprotein A2 (chloroplast)	PsaB	62.49:1.00	2.03:1.00
gi|671743459	photosystem I subunit VII (chloroplast)	PsaC	4.67:1.00	/
gi|566184073	photosystem I 20kD family protein	PsaD	2.49:1.00	1.66:1.00
gi|566162704	hypothetical protein POPTR_0003s14870g	PsaF	7.24:1.00	/
gi|552541026	photosystem I P700 chlorophyll A apoprotein A1 (chloroplast)	PsaA	/	3.21:1.00
gi|225437898	PREDICTED: photosystem I reaction center subunit IV B chloroplastic-like	PsaE	/	2.08:1.00
gi|224073967	photosystem I 11 K family protein	PsaH	/	1.76:1.00
gi|552540866	Cytochrome f (chloroplast) [Camellia danzaiensis]	PetA	0.49:1.00	1.98:1.00
gi|359492254	PREDICTED: ferredoxin–NADP reductase leaf-type isozyme chloroplastic	PetH	1.84:1.00	/
gi|671743260	cytochrome b6 (chloroplast) [Camellia petelotii]	PetB	/	3.85:1.00
gi|225436257	PREDICTED: chlorophyll a-b binding protein 8 chloroplastic	Lhca3	1.00:0	/
gi|225457389	PREDICTED: chlorophyll a-b binding protein 4 chloroplastic	Lhca4	1.00:0	/
gi|359483839	PREDICTED: chlorophyll a-b binding protein of LHCII type 1	Lhcb1	1.00:0	/
gi|225447576	PREDICTED: chlorophyll a-b binding protein 151 chloroplastic	Lhcb2	9.59:1.00	2.03:1.00
gi|225447745	PREDICTED: chlorophyll a-b binding protein CP24 10A chloroplastic	Lhcb6	17.95:1.00	/
gi|542688129	ATP synthase CF1 beta subunit (chloroplast)	beta	/	1.61:1.00

“/” indicates not detect in the database.
