# Supplementary material for: Phenotypic, histological and proteomic analyses reveal multiple differences associated with chloroplast development in yellow and variegated variants from Camellia sinensis
Source: Sci Rep. 2016 Sep 16;6:33369. doi: 10.1038/srep33369 (PMC5025893; doi:10.1038/srep33369)
Supplement: Supplementary Information [file srep33369-s1.pdf]

Supplementary Information: Fig. S1-S8, Tables S1-S11

Phenotypic, histological and proteomic analyses reveal multiple differences associated with chloroplast development in yellow and variegated variants from *Camellia sinensis*

Chengying Ma[1], Junxi Cao[1, †], Jianke Li[2], Bo Zhou[1], Jinchi Tang[1], Aiqing Miao[1, †]

[1]Tea Research Institute, Guangdong Academy of Agricultural Sciences, Guangzhou 510640, China

[2] Institute of Apicultural Research/Key Laboratory of Pollinating Insect Biology, Ministry of Agriculture, Chinese Academy of Agricultural Sciences, Beijing 100093, China

[†] To whom correspondence should be addressed.

Aiqing Miao: miaoaiqing248@163.com,

Junxi Cao: junxic@126.com

Table S1 Specific primers for quantitative PCR analysis

Primer name	Forward sequence (5'-3')	Reverse sequence (5'-3')
gi|225437428	AGCGGGTTACAATGATGTGA	TCGATTTGTTTGGGCTGATA
gi|225429772	AGCATCAGCAACAGCAATTC	GCTGGTGTATTTCCAGAGCA
gi|552540866	TGACTAGAACTGTCACTGTTACTTGAA	TCGTAATGCAGAAACATTACCTG
gi|225436257	GGGCTGACCCATACACTCTT	TGGAACCTCCTGTGCTCTG
gi|731416683	AGATGGTCCAACCACTGTGA	ATGTGGTCCAGTTGACATGC
gi|225457971	AGCTCCGGTTTGATATGTCC	GTCGGGCCATCTTGAGTAGT
gi|225459564	CCTAAGCCGAAGGTTGAAAG	TGCTTCTCCCAACAAAGATG
gi|526117629	TTGCTTGTGTGGTGATGTTG	TGACCACCAGTCCTCAGGTA
gi|225463990	TATCCCAAACGTCCTCATCA	CCACCCATCATATCATTTGC
gi|225443988	TCCATCAGCAAACAGGAAAG	TATCTGCAAGGGTGAAACCA
gi|359475502	CTCCAATCCAACCCTCTCAT	TTTGTCGCTTCTCAGGTGAC
gi|359494267	ATGCTGGGAGGAGAATATGG	GTCGACATACCCGAATTCCT
gi|224094244	ACTGATGGTGCACTTGTGGT	GCTCAAGGAAGCACCTATCC
gi|731428049	GCTTGCATACTTCGCTGTGT	GTGCTGGAGCAGATGTTGTC
gi|552541026	CATGTATTTCCACGGTGCTC	TTTGTATTCCTCGGAAACCC
gi|225457361	ACGAGCACAACCAGACAGAG	TGTCACAACAGCACTCCTCA
gi|526118093	GCTGAGAAGCATCACATCGT	AACACCTCCCAAGTTTCGTC
gi|671743230	GCCGACTCCTACTGGGAATA	GAAGGCTGGATCGTAGGAAG
gi|566146555	GAGGCTACTGAGGAGGAACG	GAGCCTTCCTGAGGGTAATG
gi|224107655	GACCGACCAGCCTAGAGTGT	CCTCTTGCACGTAGCCATTA

Table S2 The identified proteins in CK1

Protein Group	Protein ID	Accession	-10lgP	Coverage (%)	#Peptides	#Unique	PTM	Avg. Mass	Description
8	6	gi|359495606	299.92	50	43	2	Carbamidomethylation; Oxidation (M)	80006	PREDICTED: heat shock cognate protein 80 [Vitis vinifera]
1	16386	gi|669100005	299.39	76	38	38	Carbamidomethylation	52635	ribulose bisphosphate carboxylase large chain (chloroplast) [Camellia crapnelliana]
11	7	gi|225462013	294.76	47	41	2	Carbamidomethylation	80849	PREDICTED: heat shock cognate protein 80-like [Vitis vinifera]
5	1	gi|225426385	285.18	72	29	1	Carbamidomethylation	50292	PREDICTED: tubulin beta-2 chain [Vitis vinifera]
10	5	gi|359486799	283.97	55	36	2	Carbamidomethylation; Oxidation (M)	71171	PREDICTED: heat shock cognate 70 kDa protein 2 [Vitis vinifera]
13	10	gi|225434984	282.15	56	36	1	Carbamidomethylation; Oxidation (M)	71235	PREDICTED: heat shock cognate 70 kDa protein 2 [Vitis vinifera]
29	16417	gi|224056837	279.01	42	38	2	Carbamidomethylation; Oxidation (M)	80026	hypothetical protein POPTR_0001s47020g [Populus trichocarpa]
33	16411	gi|224100971	278.05	57	34	1	Carbamidomethylation; Oxidation (M)	71117	heat shock protein 70 cognate [Populus trichocarpa]
18	8	gi|225426164	277.96	40	33	2	Carbamidomethylation	80852	PREDICTED: heat shock protein 83 [Vitis vinifera]
15	16395	gi|566195847	277.71	60	26	1	Carbamidomethylation	50164	tubulin beta chain family protein [Populus trichocarpa]
2	16405	gi|566225321	276.6	72	26	3	Carbamidomethylation; Oxidation (M)	41712	actin family protein [Populus trichocarpa]
17	16	gi|225440324	275.65	52	35	1	Carbamidomethylation; Oxidation (M)	71066	PREDICTED: heat shock cognate 70 kDa protein 2 [Vitis vinifera]
24	16410	gi|566191989	275.42	55	35	1	Carbamidomethylation; Oxidation (M)	71140	heat shock protein 70 cognate [Populus trichocarpa]
3	12	gi|731410014	274.04	70	25	1	Carbamidomethylation	41593	PREDICTED: actin-101 [Vitis vinifera]

44	18	gi|225452767	267.21	57	23	3	Carbamidomethylation	49622	PREDICTED: tubulin alpha chain [Vitis vinifera]
30	17	gi|225434722	258.32	58	25	2	Carbamidomethylation	50275	PREDICTED: tubulin beta-2 chain [Vitis vinifera]
57	16494	gi|566146990	245.53	32	21	1	Carbamidomethylation; Oxidation (M)	71882	heat shock protein 70 cognate [Populus trichocarpa]
54	45	gi|225435758	245.37	49	19	4	Carbamidomethylation	49669	PREDICTED: tubulin alpha-3 chain [Vitis vinifera]
68	36	gi|225426230	244.7	35	30	1	Carbamidomethylation; Oxidation (M)	73581	PREDICTED: luminal-binding protein 5 [Vitis vinifera]
50	16441	gi|224146039	241.8	33	25	1	Carbamidomethylation	85012	hypothetical protein POPTR_0019s05430g [Populus trichocarpa]
63	46	gi|225445820	239.37	30	19	1	Carbamidomethylation	71402	PREDICTED: heat shock 70 kDa protein [Vitis vinifera]
34	31	gi|225439223	237.79	31	24	4	Carbamidomethylation	84991	PREDICTED: 5-methyltetrahydropteroyltriglutamate--homocysteine methyltransferase [Vitis vinifera]
110	20	gi|225440045	234.42	44	29	4	Carbamidomethylation; Oxidation (M)	89589	PREDICTED: cell division cycle protein 48 homolog [Vitis vinifera]
56	16456	gi|566188157	227.78	27	20	3	Carbamidomethylation	89855	hypothetical protein POPTR_0009s15490g [Populus trichocarpa]
70	16510	gi|566146992	226.31	24	14	1	Carbamidomethylation	71904	heat shock protein 70 cognate [Populus trichocarpa]
130	16503	gi|224073124	225.48	34	20	1	Carbamidomethylation	64544	chaperonin precursor family protein [Populus trichocarpa]
74	26	gi|225456079	221.3	40	16	3	Carbamidomethylation	59180	PREDICTED: ATP synthase subunit beta mitochondrial-like [Vitis vinifera]
103	16444	gi|224094244	221.19	28	22	3	Carbamidomethylation	93953	elongation factor 2 family protein [Populus trichocarpa]
65	160	gi|359491599	221.14	39	13	2	Carbamidomethylation	36735	PREDICTED: glyceraldehyde-3-phosphate dehydrogenase cytosolic [Vitis vinifera]

111	41	gi|225456004	220.63	24	18	6	Carbamidomethylation	75635	PREDICTED: stromal 70 kDa heat shock-related protein chloroplastic [Vitis vinifera]
86	16496	gi|224085900	220.21	24	22	6		94047	hypothetical protein POPTR_0005s26260g [Populus trichocarpa]
102	42	gi|731393262	219.28	41	26	3	Carbamidomethylation	64608	PREDICTED: ruBisCO large subunit-binding protein subunit beta chloroplastic [Vitis vinifera]
99	78	gi|359478916	219.09	36	15	3	Carbamidomethylation	48674	PREDICTED: ribulose bisphosphate carboxylase/oxygenase activase chloroplastic isoform X2 [Vitis vinifera]
47	33	gi|225437708	218.08	47	14	1	Carbamidomethylation	42793	PREDICTED: S-adenosylmethionine synthase 5 [Vitis vinifera]
41	34	gi|225432155	216.68	51	15	1	Carbamidomethylation	43114	PREDICTED: S-adenosylmethionine synthase 2 [Vitis vinifera]
95	16511	gi|566196410	216.3	27	21	3	Oxidation (M)	73406	BiP isoform A family protein [Populus trichocarpa]
101	16475	gi|224099437	214.5	36	14	1	Carbamidomethylation	59970	H+-transporting two-sector ATPase family protein [Populus trichocarpa]
109	39	gi|225462164	212.98	26	21	3	Carbamidomethylation	93979	PREDICTED: elongation factor 2 [Vitis vinifera]
87	44	gi|359473642	210.62	21	13	3	Carbamidomethylation	90465	PREDICTED: heat shock protein 90-1 [Vitis vinifera]
155	120	gi|733214771	209.21	22	18	1		73139	mediator of RNA polymerase II transcription subunit 37a precursor [Vitis vinifera]
76	16500	gi|224136806	207.52	43	18	3	Carbamidomethylation	47928	hypothetical protein POPTR_0015s14380g [Populus trichocarpa]
161	16476	gi|566212215	206.06	39	19	4	Carbamidomethylation	53198	adenosylhomocysteinase family protein [Populus trichocarpa]

141	16468	gi|542688129	205.93	47	17	16		53694	ATP synthase CF1 beta subunit (chloroplast) [Camellia taliensis]
66	16466	gi|566199288	205.78	40	12	1	Carbamidomethylation	42361	NAD-dependent epimerase/dehydratase family protein [Populus trichocarpa]
114	67	gi|224365668	203.2	36	18	16		55143	ATPase subunit 1 (mitochondrion) [Vitis vinifera]
145	125	gi|731401345	202.48	31	13	1	Carbamidomethylation	47976	PREDICTED: ribulose bisphosphate carboxylase/oxygenase activase chloroplastic isoform X2 [Vitis vinifera]
90	100	gi|225457939	200.86	22	20	3	Carbamidomethylation	93220	PREDICTED: endoplasmin homolog [Vitis vinifera]
93	353	gi|359483839	199.58	52	9	1	Carbamidomethylation	27890	PREDICTED: chlorophyll a-b binding protein of LHCII type 1 [Vitis vinifera]
144	16518	gi|566204581	199.5	16	10	2	Carbamidomethylation	82947	hypothetical protein POPTR_0014s16280g [Populus trichocarpa]
79	110	gi|359490179	199.08	24	16	3	Carbamidomethylation	78856	PREDICTED: transketolase chloroplastic [Vitis vinifera]
61	102	gi|731393868	199.04	17	15	1	Carbamidomethylation	89385	PREDICTED: 5-methyltetrahydropteroyltriglutamate--homocysteine methyltransferase 2 [Vitis vinifera]
163	16612	gi|566182577	197.7	28	13	3	Carbamidomethylation	48316	hypothetical protein POPTR_0008s05870g [Populus trichocarpa]
83	58	gi|225449120	197.69	34	16	1	Carbamidomethylation	49376	PREDICTED: elongation factor 1-alpha [Vitis vinifera]
72	94	gi|225464999	197.08	36	13	4		42422	PREDICTED: phosphoglycerate kinase cytosolic [Vitis vinifera]
162	92	gi|359479362	196.27	29	16	3		61984	PREDICTED: ruBisCO large subunit-binding protein subunit alpha [Vitis vinifera]

134	16497	gi|224088798	195.88	38	15	14	Carbamidomethylation	46974	translation initiation factor eIF-4A family protein [Populus trichocarpa]
197	98	gi|731379109	195.81	17	12	3	Carbamidomethylation	110565	PREDICTED: puromycin-sensitive aminopeptidase isoform X1 [Vitis vinifera]
81	16526	gi|224063766	195.08	23	15	2	Carbamidomethylation	80716	transketolase family protein [Populus trichocarpa]
175	116	gi|731410136	194.76	14	11	2	Carbamidomethylation	95223	PREDICTED: heat shock protein 83 isoform X3 [Vitis vinifera]
119	17009	gi|224065861	193.89	52	10	5	Carbamidomethylation	18243	Peptidyl-prolyl cis-trans isomerase family protein [Populus trichocarpa]
154	104	gi|225456806	193.79	33	16	1	Carbamidomethylation	53079	PREDICTED: adenosylhomocysteinase-like [Vitis vinifera]
157	72	gi|225456880	193.68	30	12	4	Carbamidomethylation	52692	PREDICTED: elongation factor TuB chloroplastic [Vitis vinifera]
82	252	gi|526117553	193.64	33	9	1	Carbamidomethylation	39322	glutamine synthetase cytosolic isozyme 2 [Vitis vinifera]
69	76	gi|225432858	192.17	36	13	1	Carbamidomethylation	42449	PREDICTED: GDP-mannose 3 5-epimerase 2 isoform X2 [Vitis vinifera]
91	138	gi|731423140	191.86	31	15	1	Carbamidomethylation	55855	PREDICTED: enolase [Vitis vinifera]
108	16572	gi|566204141	190.41	38	14	8	Carbamidomethylation	42935	hypothetical protein POPTR_0014s13660g [Populus trichocarpa]
117	205	gi|526117591	189.44	28	11	1	Carbamidomethylation	49581	chalcone synthase [Vitis vinifera]
96	16607	gi|224109060	188.92	35	15	2		50212	PHOSPHOGLYCERATE KINASE 1 family protein [Populus trichocarpa]
92	145	gi|225464995	187.41	31	14	2		50083	PREDICTED: phosphoglycerate kinase chloroplastic [Vitis vinifera]

164	85	gi|225460328	186.64	18	13	1	Carbamidomethylation	74178	PREDICTED: stromal 70 kDa heat shock-related protein chloroplastic-like [Vitis vinifera]
104	159	gi|225462777	185.29	34	11	1	Carbamidomethylation	42597	PREDICTED: chalcone synthase 2 [Vitis vinifera]
113	16660	gi|566163210	183.72	28	11	1	Carbamidomethylation	42709	naregenin-chalcone synthase family protein [Populus trichocarpa]
170	130	gi|731402352	183.36	24	18	1	Carbamidomethylation	65010	PREDICTED: ruBisCO large subunit-binding protein subunit beta chloroplastic [Vitis vinifera]
150	16609	gi|224053971	183.2	30	11	2	Carbamidomethylation	52743	hypothetical protein POPTR_0001s08770g [Populus trichocarpa]
151	146	gi|731418890	183.2	29	11	1	Carbamidomethylation	52944	PREDICTED: elongation factor Tu chloroplastic-like [Vitis vinifera]
78	121	gi|225425884	183.14	35	14	1	Carbamidomethylation	36759	PREDICTED: glyceraldehyde-3-phosphate dehydrogenase cytosolic [Vitis vinifera]
209	16775	gi|566198898	183.04	15	11	1		73464	heat shock protein 70 cognate [Populus trichocarpa]
166	17040	gi|566184599	182.19	24	9	3		37085	glyceraldehyde 3-phosphate dehydrogenase family protein [Populus trichocarpa]
306	61	gi|731426977	182.07	18	13	11	Carbamidomethylation	102347	PREDICTED: ATP-dependent Clp protease ATP-binding subunit clpA homolog CD4B chloroplastic [Vitis vinifera]
319	136	gi|225456274	181.84	25	11	3	Carbamidomethylation	55180	PREDICTED: argininosuccinate synthase chloroplastic [Vitis vinifera]
168	66	gi|225460961	181.8	16	14	2	Carbamidomethylation	110056	PREDICTED: aconitate hydratase cytoplasmic [Vitis vinifera]

182	16545	gi|566168009	181.09	24	14	1		62041	RUBISCO SUBUNIT BINDING-protein ALPHA SUBUNIT [Populus trichocarpa]
179	166	gi|225433510	180.74	25	9	4	Carbamidomethylation	51906	PREDICTED: serine hydroxymethyltransferase 4 [Vitis vinifera]
186	16674	gi|542688125	180.65	25	9	9		56099	photosystem II p680 chlorophyll A apoprotein CP-47 (chloroplast) [Camellia taliensis]
274	16517	gi|224097420	179.16	22	12	12	Carbamidomethylation	68619	Vacuolar ATP synthase catalytic subunit A family protein [Populus trichocarpa]
147	203	gi|526117485	177.56	39	10	2	Carbamidomethylation	40813	naringenin 2-oxoglutarate 3-dioxygenase [Vitis vinifera]
226	256	gi|731418471	174.86	30	10	1	Carbamidomethylation	38687	PREDICTED: mitochondrial phosphate carrier protein 3 mitochondrial isoform X2 [Vitis vinifera]
214	16575	gi|224063263	174.61	25	13	7	Carbamidomethylation	58692	2-dehydro-3-deoxyphosphoheptonate aldolase family protein [Populus trichocarpa]
269	16939	gi|224109788	174.22	28	9	1	Carbamidomethylation	37547	putative adenosine kinase family protein [Populus trichocarpa]
250	200	gi|225427768	173.87	40	9	3	Carbamidomethylation	42948	PREDICTED: fructose-bisphosphate aldolase 1 chloroplastic [Vitis vinifera]
105	143	gi|731415862	173.38	30	14	6	Carbamidomethylation	49362	PREDICTED: calreticulin [Vitis vinifera]
225	16583	gi|224079726	173.03	29	13	13	Carbamidomethylation	46428	isocitrate dehydrogenase family protein [Populus trichocarpa]
321	128	gi|225430776	171.6	22	11	11	Carbamidomethylation	73659	PREDICTED: succinate dehydrogenase [ubiquinone] flavoprotein subunit mitochondrial [Vitis vinifera]
316	38	gi|225448483	171.24	8	12	1	Carbamidomethylation; Oxidation (M)	192967	PREDICTED: clathrin heavy chain 1 [Vitis vinifera]

320	16787	gi|224102193	168.59	25	9	3	Carbamidomethylation	35716	cytosolic malate dehydrogenase family protein [Populus trichocarpa]
301	16605	gi|224141947	167.64	23	10	2	Carbamidomethylation	53416	hypothetical protein POPTR_0018s02400g [Populus trichocarpa]
137	16865	gi|224101491	167.02	70	10	1	Carbamidomethylation	20563	ADP-ribosylation factor family protein [Populus trichocarpa]
305	89	gi|731402464	166.05	15	10	2	Carbamidomethylation	93319	PREDICTED: heat shock 70 kDa protein 14-like [Vitis vinifera]
206	248	gi|225433375	165.62	21	11	1	Carbamidomethylation	61370	PREDICTED: chaperonin CPN60-2 mitochondrial [Vitis vinifera]
270	17042	gi|566164883	165.35	21	10	3	Carbamidomethylation	43246	ketol-acid reductoisomerase family protein partial [Populus trichocarpa]
193	258	gi|731404310	165.34	26	8	7	Carbamidomethylation	33129	PREDICTED: putative lactoylglutathione lyase [Vitis vinifera]
369	101	gi|225429228	165.17	16	10	1		72770	PREDICTED: heat shock 70 kDa protein mitochondrial [Vitis vinifera]
88	16659	gi|224110772	163.97	27	10	1	Carbamidomethylation	37124	glyceraldehyde 3-phosphate dehydrogenase family protein [Populus trichocarpa]
614	17095	gi|224100953	163.58	28	8	2	Carbamidomethylation	39107	thymidine diphospho-glucose 4-6-dehydratase family protein [Populus trichocarpa]
159	16723	gi|224141865	163.4	36	8	8	Carbamidomethylation	25015	GTP-binding family protein [Populus trichocarpa]
127	16640	gi|671743230	162.38	12	8	7	Carbamidomethylation	82370	photosystem I P700 apoprotein A2 (chloroplast) [Camellia petelotii]
129	494	gi|225449541	161.89	24	7	2	Carbamidomethylation	27128	PREDICTED: triosephosphate isomerase cytosolic [Vitis vinifera]
469	17246	gi|566190515	161.76	25	6	5	Carbamidomethylation	42536	hypothetical protein POPTR_0010s12790g [Populus trichocarpa]

488	500	gi|225440011	160.27	12	5	1		77480	PREDICTED: phenylalanine ammonia-lyase [Vitis vinifera]
256	237	gi|225446579	160.24	14	10	3	Carbamidomethylation	63486	PREDICTED: ketol-acid reductoisomerase chloroplastic [Vitis vinifera]
124	338	gi|225427917	160.04	26	10	4	Carbamidomethylation	34675	PREDICTED: triosephosphate isomerase chloroplastic [Vitis vinifera]
233	16962	gi|224128376	159.82	22	9	4	Carbamidomethylation	39401	thioredoxin family protein [Populus trichocarpa]
310	141	gi|731419756	159.42	23	9	2	Carbamidomethylation	52962	PREDICTED: UDP-glucose 6-dehydrogenase 1 [Vitis vinifera]
135	16861	gi|224068638	158.41	70	11	2	Carbamidomethylation	20680	hypothetical protein POPTR_0002s19210g [Populus trichocarpa]
177	315	gi|225457971	157.7	33	9	1	Carbamidomethylation	40415	PREDICTED: flavanone 3-dioxygenase [Vitis vinifera]
218	295	gi|359480275	157.34	22	6	1		35505	PREDICTED: malate dehydrogenase cytoplasmic [Vitis vinifera]
156	17045	gi|224084209	157.15	18	7	2	Carbamidomethylation	35142	O2 evolving complex 33kD family protein [Populus trichocarpa]
300	16574	gi|224058852	156.83	14	11	3	Carbamidomethylation	94161	heat shock protein 70 [Populus trichocarpa]
247	16791	gi|566173570	156.56	19	8	2	Carbamidomethylation	44912	hypothetical protein POPTR_0005s27550g [Populus trichocarpa]
349	16713	gi|566213382	155.47	20	9	2	Carbamidomethylation	53743	phosphogluconate dehydrogenase family protein [Populus trichocarpa]
278	17023	gi|566202051	155.3	14	7	2	Carbamidomethylation	71118	hypothetical protein POPTR_0014s02550g [Populus trichocarpa]
287	269	gi|359476642	155.15	23	7	1	Carbamidomethylation	42804	PREDICTED: fructose-bisphosphate aldolase 1 chloroplastic-like [Vitis vinifera]
248	449	gi|731420830	154.79	31	7	7		31951	PREDICTED: prohibitin-1 mitochondrial [Vitis vinifera]

229	16634	gi|224059642	154.69	25	10	1		42100	adenine nucleotide translocator family protein [Populus trichocarpa]
375	16970	gi|568244554	154.25	25	6	6		39549	photosystem II protein D2 (plastid) [Camellia oleifera]
189	227	gi|224365636	153.72	13	8	8		69587	PSII 32 kDa protein (mitochondrion) [Vitis vinifera]
172	16968	gi|566210965	153.05	27	10	1		39217	glutamate-ammonia ligase family protein [Populus trichocarpa]
331	16636	gi|566181869	152.94	17	8	1	Carbamidomethylation	54252	hypothetical protein POPTR_0008s02110g [Populus trichocarpa]
393	16712	gi|224100783	152.88	11	5	1		77598	phenylalanine ammonia-lyase family protein [Populus trichocarpa]
195	16693	gi|566209405	152.05	24	9	1		44445	hypothetical protein POPTR_0016s08780g [Populus trichocarpa]
149	16625	gi|671743315	151.34	19	7	6		51822	photosystem II CP43 chlorophyll apoprotein (chloroplast) [Camellia pubicosta]
327	194	gi|225442018	151.08	29	8	1	Carbamidomethylation	33909	PREDICTED: 40S ribosomal protein SA [Vitis vinifera]
210	16635	gi|566178983	150.97	23	9	1		41070	hypothetical protein POPTR_0007s01010g [Populus trichocarpa]
235	149	gi|225435480	150.84	26	10	1		42838	PREDICTED: ADP ATP carrier protein mitochondrial [Vitis vinifera]
239	17104	gi|671743459	150.79	83	7	7	Carbamidomethylation	9038	photosystem I subunit VII (chloroplast) [Camellia reticulata]
262	163	gi|225456339	150.2	28	9	1	Carbamidomethylation	41981	PREDICTED: UDP-arabinopyranose mutase 1 isoform X2 [Vitis vinifera]
160	16797	gi|224109062	150.15	24	9	1		42679	cytosolic phosphoglycerate kinase family protein [Populus trichocarpa]

169	16871	gi|566205887	149.73	29	9	1	Carbamidomethylation; Oxidation (M)	38451	glutamine synthetase family protein [Populus trichocarpa]
367	304	gi|225425053	149.71	18	8	1	Carbamidomethylation	53492	PREDICTED: 6-phosphogluconate dehydrogenase decarboxylating 3 [Vitis vinifera]
438	16573	gi|566151627	149.57	13	9	1		73210	heat shock protein 70 [Populus trichocarpa]
252	16593	gi|552541036	149.32	21	10	8		55539	ATP synthase CF1 alpha subunit (chloroplast) [Camellia yunnanensis]
342	365	gi|225459918	149.32	27	6	2	Carbamidomethylation	30824	PREDICTED: chlorophyll a-b binding protein CP26 chloroplastic [Vitis vinifera]
241	16953	gi|224105301	149.18	24	7	1	Carbamidomethylation	42832	latex plastidic aldolase-like family protein [Populus trichocarpa]
497	16615	gi|224078391	147.78	15	6	1		54253	vacuolar ATP synthase subunit B family protein [Populus trichocarpa]
356	16506	gi|224065685	146.82	22	9	9	Carbamidomethylation	56639	catalase family protein [Populus trichocarpa]
211	16633	gi|566186668	146.49	22	9	1		41811	ADP family protein [Populus trichocarpa]
205	16784	gi|566175737	146.18	22	9	8	Carbamidomethylation	47050	Monodehydroascorbate reductase family protein [Populus trichocarpa]
146	313	gi|225458424	146.13	17	6	1	Carbamidomethylation	35120	PREDICTED: oxygen-evolving enhancer protein 1 chloroplastic [Vitis vinifera]
374	448	gi|526117711	145.71	21	6	2	Carbamidomethylation	36875	malate dehydrogenase [Vitis vinifera]
475	206	gi|731385022	145.29	13	10	4	Carbamidomethylation	114759	PREDICTED: glycine dehydrogenase (decarboxylating) mitochondrial [Vitis vinifera]
324	161	gi|731388175	144.77	13	8	8	Carbamidomethylation	65990	PREDICTED: ATP-citrate synthase beta chain protein 2 [Vitis vinifera]

284	17341	gi|566238007	144.54	20	5	1	Carbamidomethylation	30925	Chlorophyll a-b binding protein CP26 [Populus trichocarpa]
125	16937	gi|224098421	144.03	26	7	1	Carbamidomethylation	27259	Triosephosphate isomerase family protein [Populus trichocarpa]
512	344	gi|225428086	143.82	15	6	1		54287	PREDICTED: V-type proton ATPase subunit B 2 [Vitis vinifera]
238	389	gi|225441754	143.57	12	6	2		53382	PREDICTED: adenylosuccinate synthetase 2 chloroplastic [Vitis vinifera]
445	741	gi|731399769	142.25	28	6	2	Carbamidomethylation	30174	PREDICTED: 2-Cys peroxiredoxin isoform X1 [Vitis vinifera]
370	17076	gi|224143955	142.08	54	7	3	Carbamidomethylation	17511	eukaryotic translation initiation factor 5A isoform V family protein [Populus trichocarpa]
231	208	gi|359485034	141.57	18	7	7		53389	PREDICTED: xylose isomerase [Vitis vinifera]
249	139	gi|225468576	141.57	12	10	2	Carbamidomethylation	98187	PREDICTED: aconitate hydratase 1 [Vitis vinifera]
379	563	gi|731376167	141.31	31	6	2	Carbamidomethylation	29267	PREDICTED: proteasome subunit beta type-5 [Vitis vinifera]
295	16781	gi|566165396	141.25	15	6	3	Carbamidomethylation	51757	hypothetical protein POPTR_0004s07280g [Populus trichocarpa]
304	17069	gi|224077927	140.75	17	6	1	Carbamidomethylation	42796	latex plastidic aldolase-like family protein [Populus trichocarpa]
64	16867	gi|566147823	140.22	9	7	3	Oxidation (M)	83349	hypothetical protein POPTR_0001s07870g [Populus trichocarpa]
347	17140	gi|224075758	139.91	50	6	6	Carbamidomethylation	15268	hypothetical protein POPTR_0003s22120g [Populus trichocarpa]
191	17348	gi|566184073	139.66	43	8	3	Carbamidomethylation	22518	photosystem I 20kD family protein [Populus trichocarpa]
440	16872	gi|566170807	139.26	24	8	1	Carbamidomethylation	40534	Chain A family protein [Populus trichocarpa]

329	16748	gi|224060514	138.95	14	7	3	Carbamidomethylation	70476	polyadenylate-binding family protein [Populus trichocarpa]
259	228	gi|225451995	138.37	35	9	1		29540	PREDICTED: 14-3-3-like protein A [Vitis vinifera]
387	17251	gi|224080233	137.96	14	6	1	Carbamidomethylation	53053	UDP-glucose 6-dehydrogenase family protein [Populus trichocarpa]
178	16804	gi|224053300	137.55	47	9	2		17702	ubiquitin/ribosomal protein 27a [Populus trichocarpa]
422	16639	gi|224086120	137.25	16	7	1	Carbamidomethylation	67209	pyrophosphate-dependent phosphofructokinase alpha subunit family protein [Populus trichocarpa]
358	16999	gi|566161272	137.23	24	8	1	Carbamidomethylation	34289	hypothetical protein POPTR_0003s06880g [Populus trichocarpa]
341	16823	gi|224104269	136.16	32	7	1	Carbamidomethylation	22228	peptidyl-prolyl cis-trans isomerase family protein [Populus trichocarpa]
333	460	gi|359483716	136.07	23	7	1	Carbamidomethylation	29551	PREDICTED: 14-3-3-like protein D isoform X2 [Vitis vinifera]
425	335	gi|225445867	135.85	16	7	2	Carbamidomethylation	60743	PREDICTED: leucine aminopeptidase 1 [Vitis vinifera]
230	132	gi|731428778	135.65	17	8	1	Carbamidomethylation	56891	PREDICTED: serine hydroxymethyltransferase mitochondrial [Vitis vinifera]
336	16665	gi|224087339	135.57	19	9	1	Carbamidomethylation	57707	biotin carboxylase precursor family protein [Populus trichocarpa]
491	137	gi|526117707	134.76	16	6	2	Carbamidomethylation	53695	uncharacterized protein LOC100261274 [Vitis vinifera]
373	16958	gi|224099363	133.94	8	7	5		101194	alpha-xylosidase family protein [Populus trichocarpa]
318	83	gi|225430261	133.51	19	9	8	Carbamidomethylation	48395	PREDICTED: DEAD-box ATP-dependent RNA helicase 56 [Vitis vinifera]
283	316	gi|359480881	133.44	7	5	5	Carbamidomethylation	94936	PREDICTED: beta-galactosidase 10 [Vitis vinifera]

185	281	gi|225465030	133.17	47	8	1		17196	PREDICTED: ubiquitin-NEDD8-like protein RUB2 [Vitis vinifera]
280	17398	gi|224138586	132.96	15	4	2		27577	ascorbate peroxidase family protein [Populus trichocarpa]
180	165	gi|359493870	132.72	15	8	2	Carbamidomethylation	42815	PREDICTED: protochlorophyllide reductase chloroplastic [Vitis vinifera]
466	413	gi|225457075	132.64	17	7	1	Carbamidomethylation	58613	PREDICTED: aldehyde dehydrogenase family 2 member B4 mitochondrial [Vitis vinifera]
339	374	gi|225426162	132.43	36	6	2	Carbamidomethylation	20013	PREDICTED: 40S ribosomal protein S10 [Vitis vinifera]
586	17453	gi|224088276	132.14	14	5	1	Carbamidomethylation	50030	leucine aminopeptidase family protein [Populus trichocarpa]
394	627	gi|225464720	132.01	14	5	2	Carbamidomethylation	44946	PREDICTED: oxygen-dependent coproporphyrinogen-III oxidase chloroplastic-like [Vitis vinifera]
123	453	gi|225447576	131.99	29	5	1	Carbamidomethylation	28468	PREDICTED: chlorophyll a-b binding protein 151 chloroplastic [Vitis vinifera]
528	17070	gi|224064707	131.75	11	7	3	Carbamidomethylation	58836	mitochondrial aldehyde dehydrogenase family protein [Populus trichocarpa]
203	688	gi|225437028	131.58	28	6	1	Carbamidomethylation	22563	PREDICTED: photosystem I reaction center subunit II chloroplastic [Vitis vinifera]
410	585	gi|225444377	131.51	20	5	1	Carbamidomethylation	38105	PREDICTED: thiamine thiazole synthase 2 chloroplastic [Vitis vinifera]
279	728	gi|359485890	131.08	6	5	2	Carbamidomethylation	83365	PREDICTED: beta-xylosidase/alpha-L-arabinofuranosidase 2-like [Vitis vinifera]
181	170	gi|225446944	130.98	19	7	1	Carbamidomethylation	43340	PREDICTED: protochlorophyllide reductase [Vitis vinifera]

228	16942	gi|566178461	130.95	14	7	5		51019	aspartate aminotransferase 2 family protein [Populus trichocarpa]
355	16705	gi|566210462	130.93	11	7	2	Carbamidomethylation	67453	Methylenetetrahydrofolate reductase family protein [Populus trichocarpa]
892	18083	gi|566159452	130.88	9	5	2		75110	hypothetical protein POPTR_0002s22560g [Populus trichocarpa]
244	364	gi|225451146	130.86	15	8	4		57701	PREDICTED: bifunctional 3-dehydroquinate dehydratase/shikimate dehydrogenase chloroplastic [Vitis vinifera]
389	271	gi|225439064	130.85	14	6	3		61092	PREDICTED: 2 3-bisphosphoglycerate-independent phosphoglycerate mutase [Vitis vinifera]
348	16923	gi|552540866	130.6	27	7	5		35220	cytochrome f (chloroplast) [Camellia danzaiensis]
354	221	gi|225445664	130.45	21	9	1	Carbamidomethylation	57338	PREDICTED: biotin carboxylase 1 chloroplastic [Vitis vinifera]
447	17192	gi|566194916	130.44	44	6	5	Carbamidomethylation	18051	hypothetical protein POPTR_0011s11260g [Populus trichocarpa]
338	197	gi|731406213	130.17	11	9	3	Carbamidomethylation	92443	PREDICTED: sucrose synthase [Vitis vinifera]
584	111	gi|225432252	130.02	16	6	6		47374	PREDICTED: 26S protease regulatory subunit 6A homolog [Vitis vinifera]
403	16898	gi|224116086	129.93	20	5	1	Carbamidomethylation	37284	hypothetical protein POPTR_0011s00500g [Populus trichocarpa]
302	251	gi|225461654	129.52	27	8	2	Carbamidomethylation	28864	PREDICTED: 14-3-3-like protein D [Vitis vinifera]
454	17032	gi|224086697	129.51	63	6	6	Carbamidomethylation	16848	calmodulin 6 family protein [Populus trichocarpa]

460	818	gi|731396254	129.03	15	5	5	Carbamidomethylation	40707	PREDICTED: alcohol dehydrogenase class-3 [Vitis vinifera]
806	706	gi|225446693	129.03	9	4	1		74318	PREDICTED: ATP-dependent zinc metalloprotease FTSH 2 chloroplastic [Vitis vinifera]
579	17122	gi|224112138	128.92	14	6	1	Carbamidomethylation	52794	hypothetical protein POPTR_0010s16730g [Populus trichocarpa]
413	16922	gi|224058615	128.92	20	5	2	Carbamidomethylation	33817	hypothetical protein POPTR_0001s08570g [Populus trichocarpa]
437	254	gi|225430619	128.42	36	8	8	Carbamidomethylation	22781	PREDICTED: 40S ribosomal protein S5 [Vitis vinifera]
456	282	gi|731423943	127.32	38	6	2	Carbamidomethylation	19961	PREDICTED: 40S ribosomal protein S10 [Vitis vinifera]
762	520	gi|225431090	126.69	28	5	5	Carbamidomethylation	27195	PREDICTED: proteasome subunit alpha type-7 [Vitis vinifera]
267	16846	gi|552541026	126.68	11	10	9	Carbamidomethylation	83146	photosystem I P700 chlorophyll A apoprotein A1 (chloroplast) [Camellia yunnanensis]
268	214	gi|526117762	126.39	28	8	1		29376	14-3-3 protein [Vitis vinifera]
84	955	gi|225465423	126.36	9	6	6	Carbamidomethylation	53142	PREDICTED: uncharacterized protein LOC100251726 [Vitis vinifera]
153	17213	gi|224103243	126.34	18	5	1	Carbamidomethylation	33816	triosephosphate isomerase family protein [Populus trichocarpa]
303	1727	gi|225465837	126.18	6	3	2	Carbamidomethylation	46787	PREDICTED: protein ASPARTIC PROTEASE IN GUARD CELL 2 [Vitis vinifera]
265	17021	gi|224083020	126.1	38	6	1	Carbamidomethylation; Oxidation (M)	18138	Peptidyl-prolyl cis-trans isomerase family protein [Populus trichocarpa]
400	74	gi|731393844	125.85	10	9	7	Carbamidomethylation	101855	PREDICTED: protein argonaute 4 [Vitis vinifera]

350	16889	gi|566170533	125.16	12	5	5		47176	hypothetical protein POPTR_0005s10290g [Populus trichocarpa]
485	16530	gi|566214279	124.61	27	9	9	Carbamidomethylation	44682	hypothetical protein POPTR_0018s04900g [Populus trichocarpa]
402	108	gi|526117643	124.38	11	9	5		101329	heat shock protein 101 [Vitis vinifera]
201	217	gi|225437428	124.38	7	6	1	Carbamidomethylation	92483	PREDICTED: sucrose synthase 2 [Vitis vinifera]
543	932	gi|225450626	124.36	17	6	1	Carbamidomethylation	39269	PREDICTED: probable protein disulfide-isomerase A6 [Vitis vinifera]
647	1175	gi|359483879	123.97	15	5	1	Carbamidomethylation	43617	PREDICTED: LOW QUALITY PROTEIN: pyruvate dehydrogenase E1 component subunit beta-3 chloroplastic-like [Vitis vinifera]
659	17029	gi|566210440	123.75	15	5	5	Carbamidomethylation	49767	GDP dissociation inhibitor family protein [Populus trichocarpa]
458	333	gi|225447725	123.42	9	5	2	Carbamidomethylation	61438	PREDICTED: calnexin homolog [Vitis vinifera]
424	16940	gi|566206623	123.27	8	5	1	Carbamidomethylation	54072	aldehyde dehydrogenase 1 precursor family protein [Populus trichocarpa]
875	71040	gi|224072580	123.19	27	4	4	Carbamidomethylation	29016	chlorophyll a/b-binding protein type II precursor [Populus trichocarpa]
773	1012	gi|225430650	122.83	16	5	1	Carbamidomethylation; Oxidation (M)	43927	PREDICTED: pyruvate dehydrogenase E1 component subunit beta-3 chloroplastic [Vitis vinifera]
642	572	gi|731382516	122.67	14	4	4		37912	PREDICTED: ankyrin repeat domain-containing protein 2 isoform X2 [Vitis vinifera]
251	969	gi|225426753	122.67	9	6	2	Oxidation (M)	81719	PREDICTED: primary amine oxidase [Vitis vinifera]
273	255	gi|526117940	122.46	4	3	1		91805	phospholipase D alpha [Vitis vinifera]

237	633	gi|225444649	122.39	16	4	4		26396	PREDICTED: 20 kDa chaperonin chloroplastic [Vitis vinifera]
246	614	gi|359493457	122.29	15	5	1	Carbamidomethylation; Oxidation (M)	48311	PREDICTED: elongation factor 1-gamma [Vitis vinifera]
385	17541	gi|566196811	122.04	8	4	1	Carbamidomethylation	62410	hypothetical protein POPTR_0012s04670g [Populus trichocarpa]
388	229	gi|731427020	121.62	10	6	1	Carbamidomethylation; Oxidation (M)	76330	PREDICTED: glycine--tRNA ligase 1 mitochondrial [Vitis vinifera]
260	481	gi|225442079	121.41	23	5	5	Carbamidomethylation	27325	PREDICTED: proteasome subunit alpha type-6 [Vitis vinifera]
464	107	gi|225452950	121.33	8	6	4		109834	PREDICTED: chaperone protein ClpB3 chloroplastic [Vitis vinifera]
142	859	gi|225429614	121.05	26	3	2		17293	PREDICTED: 17.3 kDa class II heat shock protein [Vitis vinifera]
213	16790	gi|566191383	120.83	10	5	1	Carbamidomethylation	45066	hypothetical protein POPTR_0010s17930g [Populus trichocarpa]
368	301	gi|225452510	120.71	9	6	1	Carbamidomethylation	58057	PREDICTED: aldehyde dehydrogenase family 2 member B7 mitochondrial [Vitis vinifera]
510	598	gi|225454009	120.7	12	6	1	Carbamidomethylation	80666	PREDICTED: transketolase chloroplastic [Vitis vinifera]
428	16954	gi|224076908	120.28	19	7	1		40750	hypothetical protein POPTR_0004s06400g [Populus trichocarpa]
479	661	gi|225459564	119.66	13	3	3		28575	PREDICTED: photosystem II 22 kDa protein chloroplastic [Vitis vinifera]
240	17330	gi|566195299	119.15	39	4	1		13997	histone H2A family protein [Populus trichocarpa]
257	17363	gi|224109746	118.93	14	4	2	Carbamidomethylation	26377	putative chlorophyll a/b-binding family protein [Populus trichocarpa]

126	410	gi|359475042	118.51	34	6	1	Carbamidomethylation; Oxidation (M)	18313	PREDICTED: peptidyl-prolyl cis-trans isomerase [Vitis vinifera]
527	720	gi|359476926	118.3	18	3	1	Carbamidomethylation	18454	PREDICTED: photosystem I reaction center subunit N chloroplastic [Vitis vinifera]
703	1634	gi|731413091	118.16	25	7	1	Carbamidomethylation	38789	PREDICTED: UDP-glucuronic acid decarboxylase 5 [Vitis vinifera]
433	541	gi|225436900	117.88	19	7	1		47680	PREDICTED: glutamine synthetase leaf isozyme chloroplastic [Vitis vinifera]
340	664	gi|731403303	117.64	30	4	4	Carbamidomethylation	15026	PREDICTED: 40S ribosomal protein S12 [Vitis vinifera]
590	18948	gi|566197644	117.61	15	5	4	Carbamidomethylation	26318	hypothetical protein POPTR_0012s09540g [Populus trichocarpa]
559	629	gi|359492254	117.51	20	9	2	Carbamidomethylation	40876	PREDICTED: ferredoxin--NADP reductase leaf-type isozyme chloroplastic [Vitis vinifera]
496	337	gi|225423961	117.05	9	6	4	Carbamidomethylation	103148	PREDICTED: alpha-xylosidase 1 [Vitis vinifera]
530	16811	gi|566182073	116.85	30	6	6		15828	hypothetical protein POPTR_0008s03050g [Populus trichocarpa]
407	17819	gi|224131818	116.14	22	3	1		28513	chlorophyll a/b-binding family protein [Populus trichocarpa]
282	16754	gi|566176273	115.69	7	6	1		92143	sucrose synthase family protein [Populus trichocarpa]
550	156	gi|225441236	115.42	3	4	2		177678	PREDICTED: ferredoxin-dependent glutamate synthase chloroplastic [Vitis vinifera]
317	17234	gi|566207041	115.24	12	4	2	Carbamidomethylation	53652	glutamate 1-semialdehyde aminotransferase family protein [Populus trichocarpa]
421	18346	gi|566184549	115.23	26	6	2	Carbamidomethylation	29191	20S proteasome beta subunit family protein [Populus trichocarpa]

366	17320	gi|566189392	115.04	18	5	5		27258	hypothetical protein POPTR_0010s06320g [Populus trichocarpa]
346	663	gi|225449052	114.65	29	4	4		17824	PREDICTED: 60S ribosomal protein L12 [Vitis vinifera]
728	551	gi|731440696	114.43	10	6	3		64402	PREDICTED: hsp70-Hsp90 organizing protein 3-like [Vitis vinifera]
868	17422	gi|552540940	114.09	9	4	4		58361	acetyl-CoA carboxylase carboxyl transferase beta (chloroplast) [Camellia impressinervis]
556	16998	gi|566156890	114.02	13	5	2		44077	hypothetical protein POPTR_0002s08240g [Populus trichocarpa]
537	17442	gi|566152994	113.95	15	7	2	Carbamidomethylation	57135	hypothetical protein POPTR_0001s35790g [Populus trichocarpa]
311	216	gi|526117840	113.09	11	7	1		58698	3-deoxy-D-arabino-heptulosonate-7-phosphate synthase [Vitis vinifera]
297	602	gi|359476682	112.85	20	4	2	Carbamidomethylation; Oxidation (M)	24349	PREDICTED: soluble inorganic pyrophosphatase isoform X2 [Vitis vinifera]
455	17820	gi|566175251	112.65	7	3	3		34569	hypothetical protein POPTR_0006s08710g [Populus trichocarpa]
457	16901	gi|224102579	112.36	16	7	2		47747	glutamine synthetase family protein [Populus trichocarpa]
391	692	gi|731388721	112.17	23	4	2		18965	PREDICTED: translationally-controlled tumor protein homolog [Vitis vinifera]
216	18507	gi|566242200	112.12	14	4	2		19093	hypothetical protein POPTR_0019s12370g partial [Populus trichocarpa]
765	29329	gi|224141875	112.09	18	3	3	Carbamidomethylation	16908	zinc finger family protein [Populus trichocarpa]
449	1514	gi|225450791	111.48	20	3	1		22379	PREDICTED: translationally-controlled tumor protein homolog [Vitis vinifera]

442	17944	gi|224093408	111.26	17	3	1	Carbamidomethylation	18464	hypothetical protein POPTR_0007s04160g [Populus trichocarpa]
785	473	gi|225436253	111.14	9	5	1	Carbamidomethylation	66904	PREDICTED: probable methylenetetrahydrofolate reductase [Vitis vinifera]
630	17915	gi|224131676	110.82	38	4	2	Carbamidomethylation	11636	Ubiquitin-like family protein [Populus trichocarpa]
470	17056	gi|224110900	110.37	12	5	1	Carbamidomethylation	53476	hypothetical protein POPTR_0010s05530g [Populus trichocarpa]
255	17194	gi|566181525	109.84	13	6	1		42160	hypothetical protein POPTR_0008s00350g [Populus trichocarpa]
640	636	gi|731387895	109.81	9	4	1	Carbamidomethylation	51263	PREDICTED: UTP--glucose-1-phosphate uridylyltransferase isoform X2 [Vitis vinifera]
521	16879	gi|224059184	109.72	7	4	2		91823	phospholipase D family protein [Populus trichocarpa]
121	899	gi|225431269	108.6	10	3	2	Carbamidomethylation	36580	PREDICTED: peroxidase 72 [Vitis vinifera]
397	832	gi|225461287	108.14	20	3	3	Carbamidomethylation	24076	PREDICTED: cytochrome b6-f complex iron-sulfur subunit 1 chloroplastic [Vitis vinifera]
380	17232	gi|566169561	107.86	13	3	1		34208	hypothetical protein POPTR_0005s05040g [Populus trichocarpa]
360	17598	gi|566158065	107.84	11	4	1		37108	arginase family protein [Populus trichocarpa]
296	16672	gi|566203657	107.37	12	5	3	Carbamidomethylation	56866	hypothetical protein POPTR_0014s10800g [Populus trichocarpa]
436	746	gi|225447041	107.37	8	4	2	Carbamidomethylation	60008	PREDICTED: L-ascorbate oxidase homolog [Vitis vinifera]
564	1208	gi|225424530	106.81	22	5	5		36021	PREDICTED: eukaryotic translation initiation factor 3 subunit I [Vitis vinifera]

900	447	gi|359494836	106.76	6	3	3	Carbamidomethylation	61508	PREDICTED: pyrophosphate--fructose 6-phosphate 1-phosphotransferase subunit beta [Vitis vinifera]
332	829	gi|225436257	106.64	12	3	3		29530	PREDICTED: chlorophyll a-b binding protein 8 chloroplastic [Vitis vinifera]
730	785	gi|225459806	106.57	10	6	1	Carbamidomethylation	57322	PREDICTED: T-complex protein 1 subunit beta [Vitis vinifera]
330	17266	gi|224104631	106.48	13	3	1		27319	cytosolic ascorbate peroxidase family protein [Populus trichocarpa]
598	140	gi|359475304	106.4	6	5	5	Carbamidomethylation	98624	PREDICTED: coatomer subunit gamma-2 [Vitis vinifera]
691	16870	gi|224092216	106.17	7	3	3	Carbamidomethylation	57364	glycine hydroxymethyltransferase family protein [Populus trichocarpa]
700	17127	gi|566149354	106.06	21	5	5		26031	Proteasome subunit alpha type 5-1 family protein [Populus trichocarpa]
116	17575	gi|566170929	106.05	10	3	1	Carbamidomethylation	36394	hypothetical protein POPTR_0005s12070g [Populus trichocarpa]
173	17181	gi|566259890	105.93	11	8	3		48155	elongation factor 1-gamma 1 family protein [Populus trichocarpa]
223	238	gi|359481634	105.84	33	5	2		16594	PREDICTED: ubiquitin-conjugating enzyme E2 variant 1D [Vitis vinifera]
653	727	gi|225464809	105.45	9	4	1	Carbamidomethylation	56995	PREDICTED: gamma aminobutyrate transaminase 1 mitochondrial-like [Vitis vinifera]
509	18144	gi|224073967	105.33	13	3	3		15188	photosystem I 11K family protein [Populus trichocarpa]
788	411	gi|359497202	105.28	7	4	4	Carbamidomethylation	60614	PREDICTED: T-complex protein 1 subunit eta [Vitis vinifera]

1040	311	gi|225430390	105.09	9	3	3		57260	PREDICTED: threonine synthase chloroplastic [Vitis vinifera]
648	223	gi|225429530	105.07	8	4	4		54362	PREDICTED: UDP-sulfoquinovose synthase chloroplastic [Vitis vinifera]
351	1330	gi|225426512	104.96	14	3	1	Carbamidomethylation	24384	PREDICTED: soluble inorganic pyrophosphatase [Vitis vinifera]
541	17258	gi|224140177	104.87	7	5	2	Carbamidomethylation	53698	adenylosuccinate synthetase family protein [Populus trichocarpa]
636	321	gi|225457058	104.76	8	4	1	Carbamidomethylation	60477	PREDICTED: T-complex protein 1 subunit gamma [Vitis vinifera]
688	17046	gi|224085984	104.62	10	5	1	Carbamidomethylation	61382	multi-copper oxidase type 1 family protein [Populus trichocarpa]
71	736	gi|225455934	104.51	19	5	5	Carbamidomethylation; Oxidation (M)	20429	PREDICTED: ribulose bisphosphate carboxylase small chain chloroplastic [Vitis vinifera]
569	17223	gi|224139104	104.41	10	3	3	Carbamidomethylation	33044	pyridoxin biosynthesis PDX1-like protein 3 [Populus trichocarpa]
553	750	gi|225457502	104.23	7	2	2		35357	PREDICTED: probable succinyl-CoA ligase [ADP-forming] subunit alpha mitochondrial [Vitis vinifera]
357	658	gi|731392450	104.19	12	4	1	Carbamidomethylation	27557	PREDICTED: L-ascorbate peroxidase 2 cytosolic [Vitis vinifera]
392	2170	gi|225425388	104.13	30	2	2		13594	PREDICTED: nuclear transport factor 2 [Vitis vinifera]
677	17917	gi|566206287	103.95	15	4	1		35464	ATP synthase gamma chain family protein [Populus trichocarpa]
753	699	gi|225435309	103.87	6	5	2	Carbamidomethylation	82345	PREDICTED: 4-hydroxy-3-methylbut-2-en-1-yl diphosphate synthase chloroplastic [Vitis vinifera]

683	721	gi|225429209	103.76	7	4	3		53142	PREDICTED: serine carboxypeptidase-like 16 [Vitis vinifera]
378	811	gi|225456404	103.56	9	3	1	Carbamidomethylation	50437	PREDICTED: glutamate-1-semialdehyde 2 1-aminomutase chloroplastic [Vitis vinifera]
758	3000	gi|225453620	103.25	7	2	2	Carbamidomethylation; Oxidation (M)	47761	PREDICTED: pyruvate dehydrogenase E1 component subunit alpha-3 chloroplastic [Vitis vinifera]
605	1222	gi|225457219	102.87	13	4	1	Carbamidomethylation	35220	PREDICTED: ATP synthase subunit gamma mitochondrial [Vitis vinifera]
377	18002	gi|566185685	102.49	14	3	3	Carbamidomethylation	22408	hypothetical protein POPTR_0009s01050g [Populus trichocarpa]
524	16560	gi|566206267	102.44	5	5	1		100324	hypothetical protein POPTR_0015s06760g [Populus trichocarpa]
465	792	gi|225465764	102.43	13	4	4	Carbamidomethylation	43403	PREDICTED: UDP-D-apiose/UDP-D-xylose synthase 2 [Vitis vinifera]
476	293	gi|225455784	102.38	12	4	3	Carbamidomethylation	50558	PREDICTED: enolase 1 chloroplastic [Vitis vinifera]
467	18849	gi|224065507	102.35	19	2	1		15080	Photosystem I reaction center subunit IV A family protein [Populus trichocarpa]
506	218	gi|731395250	102.3	11	4	1	Carbamidomethylation	48931	PREDICTED: elongation factor Tu mitochondrial [Vitis vinifera]
832	19656	gi|566169467	102.2	17	2	1		13987	hypothetical protein POPTR_0005s04590g [Populus trichocarpa]
856	325	gi|225456051	102.08	9	4	2	Carbamidomethylation	57589	PREDICTED: T-complex protein 1 subunit delta [Vitis vinifera]
627	2319	gi|225452270	101.95	12	4	2		34598	PREDICTED: 28 kDa ribonucleoprotein chloroplastic-like [Vitis vinifera]

673	16821	gi|566215982	101.86	8	4	1	Carbamidomethylation	60519	hypothetical protein POPTR_0019s00210g [Populus trichocarpa]
408	547	gi|225456295	101.84	20	5	3		24961	PREDICTED: elongation factor 1-delta [Vitis vinifera]
419	953	gi|526118138	101.82	17	4	4		33840	uncharacterized protein LOC100264250 [Vitis vinifera]
337	17014	gi|224140653	101.8	7	4	1		61182	phosphoglycerate mutase family protein [Populus trichocarpa]
784	1243	gi|225438781	101.74	7	3	1	Carbamidomethylation	71362	PREDICTED: polyadenylate-binding protein 2 [Vitis vinifera]
477	962	gi|225447723	101.57	3	4	2		109436	PREDICTED: staphylococcal nuclease domain-containing protein 1 [Vitis vinifera]
322	703	gi|731388929	101.27	11	5	2	Carbamidomethylation	56541	PREDICTED: bifunctional 3-dehydroquinate dehydratase/shikimate dehydrogenase chloroplastic isoform X2 [Vitis vinifera]
600	1620	gi|225433918	101.14	6	2	1		35199	PREDICTED: probable fructokinase-4 [Vitis vinifera]
261	578	gi|225436841	100.94	38	4	1		14393	PREDICTED: histone H2AX [Vitis vinifera]
417	731	gi|225449971	100.9	4	4	4		121958	PREDICTED: protein transport protein SEC31 homolog B isoform X2 [Vitis vinifera]
602	979	gi|731416996	100.76	9	4	4		46827	PREDICTED: poly(rC)-binding protein 3 [Vitis vinifera]
502	1529	gi|225432466	100.68	17	2	2	Carbamidomethylation	18476	PREDICTED: peptide methionine sulfoxide reductase B5-like [Vitis vinifera]
609	17949	gi|224132216	100.42	31	3	1	Carbamidomethylation	11688	hypothetical protein POPTR_0014s18990g [Populus trichocarpa]
621	18654	gi|224053535	100.22	12	3	1		38585	pyruvate dehydrogenase family protein [Populus trichocarpa]
872	17257	gi|224108938	100.14	42	5	1	Carbamidomethylation	17381	hypothetical protein POPTR_0010s17020g [Populus trichocarpa]

551	18232	gi|566187006	99.99	8	3	2		50172	hypothetical protein POPTR_0009s08570g [Populus trichocarpa]
1064	17962	gi|359476900	99.98	10	3	1		40711	PREDICTED: isocitrate dehydrogenase [NAD] regulatory subunit 1 mitochondrial-like [Vitis vinifera]
869	17523	gi|224107106	99.81	5	2	2		79571	MULTIFUNCTIONAL family protein [Populus trichocarpa]
395	17243	gi|224100839	99.71	24	4	4		19919	ATP synthase D chain-related family protein [Populus trichocarpa]
849	17738	gi|224066507	99.61	9	4	3	Carbamidomethylation	43683	mRNA-binding family protein [Populus trichocarpa]
531	1029	gi|359473645	99.58	6	5	3	Carbamidomethylation	66079	PREDICTED: chaperonin 60 subunit beta 4 chloroplastic [Vitis vinifera]
733	231	gi|225450275	99.57	18	5	3	Carbamidomethylation	35771	PREDICTED: serine/threonine-protein phosphatase PP2A-2 catalytic subunit [Vitis vinifera]
308	17233	gi|224065421	99.5	6	4	1		64078	hypothetical protein POPTR_0002s24970g [Populus trichocarpa]
588	17873	gi|224058705	99.37	21	2	2	Carbamidomethylation	18641	cytochrome c oxidase-related family protein [Populus trichocarpa]
921	326	gi|359492510	99.32	7	3	1		41451	PREDICTED: probable L-ascorbate peroxidase 6 chloroplastic isoform X2 [Vitis vinifera]
694	312	gi|225428247	99.2	10	5	1	Carbamidomethylation	60225	PREDICTED: L-ascorbate oxidase homolog [Vitis vinifera]
429	30216	gi|566168757	99.13	11	4	2		42533	hypothetical protein POPTR_0005s01370g [Populus trichocarpa]
591	18711	gi|224128708	99.07	12	3	3		33828	xyloglucan endo-1 family protein [Populus trichocarpa]

500	17495	gi|224122692	98.94	16	4	4		25625	Proteasome subunit alpha type 2 family protein [Populus trichocarpa]
717	606	gi|731438689	98.88	13	5	5		44313	PREDICTED: FAM10 family protein At4g22670 [Vitis vinifera]
566	17264	gi|224121954	98.83	15	3	2		26774	hypothetical protein POPTR_0012s09720g [Populus trichocarpa]
382	887	gi|359479647	98.79	24	5	5	Carbamidomethylation	22491	PREDICTED: proteasome subunit beta type-2-A [Vitis vinifera]
990	875	gi|225452831	98.56	11	4	3		43750	PREDICTED: malate dehydrogenase chloroplastic-like [Vitis vinifera]
601	247	gi|225452974	98.32	11	5	3		58482	PREDICTED: probable mitochondrial-processing peptidase subunit beta [Vitis vinifera]
675	18119	gi|224063066	98.16	8	4	1	Carbamidomethylation	56188	disulfide-isomerase family protein [Populus trichocarpa]
315	17196	gi|224121218	98.14	12	4	4	Carbamidomethylation	36046	GTP-binding protein beta chain [Populus trichocarpa]
704	17528	gi|566162704	97.83	20	3	2		18629	hypothetical protein POPTR_0003s14870g [Populus trichocarpa]
526	17210	gi|224136368	97.77	4	4	2		107885	110 kDa 4SNc-Tudor domain family protein [Populus trichocarpa]
352	729	gi|731421236	97.65	11	4	1		37446	PREDICTED: arginase 1 mitochondrial [Vitis vinifera]
980	155	gi|225446449	97.53	7	5	2		97503	PREDICTED: 26S proteasome non-ATPase regulatory subunit 2 homolog A [Vitis vinifera]
782	545	gi|526117441	97.47	4	4	1		101670	lipoxygenase [Vitis vinifera]
616	762	gi|225448367	97.35	10	3	3		34281	PREDICTED: 60S acidic ribosomal protein P0 [Vitis vinifera]

643	363	gi|225464852	97.25	43	5	2		16577	PREDICTED: ubiquitin-conjugating enzyme E2 variant 1D [Vitis vinifera]
555	424	gi|225428772	97.24	3	3	1		121098	PREDICTED: presequence protease 2 chloroplastic/mitochondrial-like [Vitis vinifera]
511	18516	gi|224104953	97.04	5	3	1		68350	beta-D-glucan exohydrolase family protein [Populus trichocarpa]
468	1587	gi|225453350	96.64	21	3	2		16310	PREDICTED: nucleoside diphosphate kinase B [Vitis vinifera]
582	866	gi|225425304	96.52	44	3	3	Carbamidomethylation	9611	PREDICTED: 40S ribosomal protein S27-2 [Vitis vinifera]
618	192	gi|225434712	96.47	21	6	1		29576	PREDICTED: proliferating cell nuclear antigen [Vitis vinifera]
767	17277	gi|566170863	96.36	33	3	3		11409	histone H4 family protein [Populus trichocarpa]
578	16779	gi|566175799	96.1	10	6	3	Carbamidomethylation	57465	pyruvate kinase family protein [Populus trichocarpa]
813	18442	gi|224100797	95.84	12	4	2	Carbamidomethylation	26417	putative chlorophyll a/b-binding family protein [Populus trichocarpa]
289	807	gi|225461209	95.4	17	3	3	Oxidation (M)	21740	PREDICTED: NAD(P)H dehydrogenase (quinone) FQR1 [Vitis vinifera]
314	226	gi|359497288	95.35	7	4	1	Carbamidomethylation	64041	PREDICTED: peptidyl-prolyl cis-trans isomerase FKBP62 isoform X1 [Vitis vinifera]
420	1290	gi|225453909	94.85	17	3	3	Carbamidomethylation	24628	PREDICTED: proteasome subunit beta type-1 [Vitis vinifera]
943	593	gi|731412938	94.74	3	4	1	Carbamidomethylation	155024	PREDICTED: probable phosphoribosylformylglycinamidine synthase chloroplastic/mitochondrial [Vitis vinifera]

486	17370	gi|566192956	94.61	10	4	1	Carbamidomethylation	44225	hypothetical protein POPTR_0011s00650g [Populus trichocarpa]
328	17211	gi|224135485	94.61	6	4	1	Carbamidomethylation	58870	glycosyl hydrolase family 1 family protein [Populus trichocarpa]
935	17344	gi|224125422	94.36	6	3	2	Carbamidomethylation	56543	hypothetical protein POPTR_0013s03070g [Populus trichocarpa]
635	16938	gi|224145979	94.18	9	4	4	Carbamidomethylation	58735	hypothetical protein POPTR_0019s04980g [Populus trichocarpa]
538	1552	gi|359495476	94.05	23	5	1		29035	PREDICTED: chlorophyll a-b binding protein 13 chloroplastic [Vitis vinifera]
817	17391	gi|566182904	93.84	6	3	3		59760	hypothetical protein POPTR_0008s07890g [Populus trichocarpa]
617	1238	gi|225425166	93.83	12	3	1		39489	PREDICTED: pyruvate dehydrogenase E1 component subunit beta-1 mitochondrial [Vitis vinifera]
904	458	gi|359475502	93.8	11	4	2	Carbamidomethylation	37652	PREDICTED: malate dehydrogenase glyoxysomal [Vitis vinifera]
597	336	gi|225429870	93.39	9	6	6	Carbamidomethylation	60861	PREDICTED: aspartate--tRNA ligase cytoplasmic [Vitis vinifera]
561	685	gi|225459808	93.33	6	4	1		60137	PREDICTED: L-ascorbate oxidase homolog [Vitis vinifera]
650	1650	gi|225439520	93.25	6	4	2		65247	PREDICTED: pyruvate decarboxylase 2 [Vitis vinifera]
769	17256	gi|224125002	93.15	5	2	1	Carbamidomethylation	59410	importin alpha-1 subunit family protein [Populus trichocarpa]
652	17008	gi|566186274	92.41	17	6	1		29153	proliferating cell nuclear antigen family protein [Populus trichocarpa]

570	17814	gi|566177370	92.33	7	4	1		60158	DEAD box RNA helicase family protein [Populus trichocarpa]
764	1353	gi|225428047	92.29	24	4	3	Carbamidomethylation	17518	PREDICTED: SKP1-like protein 1A isoform X5 [Vitis vinifera]
523	417	gi|225429850	92.08	17	3	1		24915	PREDICTED: proteasome subunit beta type-6 [Vitis vinifera]
984	17804	gi|566168414	92.01	10	3	1		34495	quinone oxidoreductase-like family protein [Populus trichocarpa]
430	16793	gi|566186880	91.89	4	4	3	Carbamidomethylation	99572	ubiquitin activating enzyme 2 family protein [Populus trichocarpa]
701	17124	gi|566259876	91.78	14	3	1		23976	hypothetical protein POPTR_0022s00350g [Populus trichocarpa]
680	639	gi|731428049	91.68	9	3	2	Carbamidomethylation	47334	PREDICTED: delta-aminolevulinic acid dehydratase chloroplastic [Vitis vinifera]
1025	597	gi|225441924	91.22	7	2	1	Carbamidomethylation	51126	PREDICTED: eukaryotic translation initiation factor 3 subunit E [Vitis vinifera]
603	1324	gi|526117872	91.21	14	3	3		25310	manganese superoxide dismutase [Vitis vinifera]
490	566	gi|731407251	91.09	3	3	1		92866	PREDICTED: phospholipase D alpha 1-like [Vitis vinifera]
807	592	gi|225437537	90.97	18	3	3		22019	PREDICTED: GTP-binding protein SAR1A [Vitis vinifera]
361	902	gi|225461808	90.93	8	4	1		75747	PREDICTED: trifunctional UDP-glucose 4 6-dehydratase/UDP-4-keto-6-deoxy-D-glucose 3 5-epimerase/UDP-4-keto-L-rhamnose-reductase RHM1 [Vitis vinifera]
599	17503	gi|566165056	90.92	14	4	1	Carbamidomethylation	36365	hypothetical protein POPTR_0004s05340g [Populus trichocarpa]

698	16798	gi|566149322	90.9	4	4	1		105097	putative plasma membrane H+ ATPase family protein [Populus trichocarpa]
499	1322	gi|225424805	90.72	10	4	1	Carbamidomethylation	43702	PREDICTED: pyruvate dehydrogenase E1 component subunit alpha mitochondrial [Vitis vinifera]
842	17635	gi|224092266	90.59	6	3	3	Carbamidomethylation	63096	hypothetical protein POPTR_0006s25280g [Populus trichocarpa]
463	1011	gi|225435878	90.49	28	3	2		10567	PREDICTED: 10 kDa chaperonin [Vitis vinifera]
815	71046	gi|224129046	90.46	9	2	1	Carbamidomethylation	36161	1-aminocyclopropane-1-carboxylate oxidase family protein [Populus trichocarpa]
372	1653	gi|225429975	90.46	8	3	2		39098	PREDICTED: bark storage protein A [Vitis vinifera]
353	1023	gi|731392188	90.44	15	3	1	Carbamidomethylation	23071	PREDICTED: triosephosphate isomerase cytosolic isoform X2 [Vitis vinifera]
737	1518	gi|225440290	90.3	38	4	3	Carbamidomethylation	16532	PREDICTED: ubiquitin-conjugating enzyme E2-17 kDa [Vitis vinifera]
612	17490	gi|224137858	90.3	13	4	1		41555	hypothetical protein POPTR_0016s04600g [Populus trichocarpa]
481	987	gi|731411833	90.21	9	2	2	Carbamidomethylation	35859	PREDICTED: nitrilase-like protein 2 [Vitis vinifera]
501	804	gi|225460542	89.97	10	3	1		36331	PREDICTED: heterogeneous nuclear ribonucleoprotein 1 [Vitis vinifera]
655	17270	gi|566147298	89.75	9	4	1		41639	ENOYL-ACP REDUCTASE 1 family protein [Populus trichocarpa]
742	17315	gi|224079274	89.54	12	4	4		41124	ubiquitin family protein [Populus trichocarpa]
199	1532	gi|225460502	89.44	4	2	1	Carbamidomethylation	64083	PREDICTED: laccase-14 [Vitis vinifera]

991	17699	gi|566174515	89.16	4	3	3		80466	hypothetical protein POPTR_0006s04870g [Populus trichocarpa]
634	16961	gi|566166593	89.03	3	3	1		122467	hypothetical protein POPTR_0004s14960g [Populus trichocarpa]
633	17228	gi|224103569	88.92	4	3	1		112946	hypothetical protein POPTR_0009s10650g [Populus trichocarpa]
945	17475	gi|224107361	88.92	3	3	3		128813	ADP-forming family protein [Populus trichocarpa]
516	18185	gi|566211620	88.85	12	3	2		29136	hypothetical protein POPTR_0017s05770g [Populus trichocarpa]
554	17536	gi|566178862	88.82	7	2	2		34943	hypothetical protein POPTR_0007s00300g [Populus trichocarpa]
779	17297	gi|224141065	88.7	7	3	1		58604	mitochondrial processing peptidase beta subunit family protein [Populus trichocarpa]
604	17489	gi|224071429	88.58	5	2	1	Carbamidomethylation	44978	Phosphoribulokinase family protein [Populus trichocarpa]
882	596	gi|225466690	88.24	10	3	3		42525	PREDICTED: sedoheptulose-1 7-bisphosphatase chloroplastic [Vitis vinifera]
710	17574	gi|566178300	88.03	6	2	1	Carbamidomethylation	45519	hypothetical protein POPTR_0006s26630g [Populus trichocarpa]
472	540	gi|225449262	87.15	24	5	1		18173	PREDICTED: 18.1 kDa class I heat shock protein [Vitis vinifera]
563	667	gi|225470804	87.09	6	2	1		45224	PREDICTED: phosphoribulokinase chloroplastic [Vitis vinifera]
805	991	gi|359494079	86.99	5	2	2	Carbamidomethylation	65102	PREDICTED: LOW QUALITY PROTEIN: 65-kDa microtubule-associated protein 1-like [Vitis vinifera]

908	17613	gi|224109576	86.98	13	3	1	Carbamidomethylation	24014	hypothetical protein POPTR_0010s21600g [Populus trichocarpa]
841	1498	gi|225459760	86.77	11	4	4	Carbamidomethylation	33454	PREDICTED: thioredoxin-like protein CDSP32 chloroplastic [Vitis vinifera]
939	17448	gi|566149991	86.73	6	3	2	Carbamidomethylation	47475	hypothetical protein POPTR_0001s20510g [Populus trichocarpa]
901	16906	gi|224118744	86.64	6	3	1		57369	chaperonin family protein [Populus trichocarpa]
1067	1626	gi|359482440	86.62	4	2	1		57853	PREDICTED: alanine aminotransferase 2 [Vitis vinifera]
736	17747	gi|224145285	86.31	17	4	1		24030	GTP-binding family protein [Populus trichocarpa]
281	17618	gi|224102515	86.17	7	3	2		29608	porin family protein [Populus trichocarpa]
431	17252	gi|224075976	86.11	11	3	3	Carbamidomethylation	31893	hypothetical protein POPTR_0003s21070g [Populus trichocarpa]
713	314	gi|225432110	85.9	7	4	4	Carbamidomethylation	52920	PREDICTED: dihydrolipoyl dehydrogenase mitochondrial [Vitis vinifera]
1217	17674	gi|224104643	85.81	4	2	1		65194	thioredoxin family protein [Populus trichocarpa]
557	816	gi|526118026	85.52	9	3	2		55487	5-enolpyruvylshikimate-3-phosphate synthase [Vitis vinifera]
1003	1643	gi|225452186	85.09	12	4	4		30725	PREDICTED: prohibitin-3 mitochondrial [Vitis vinifera]
1039	814	gi|225433414	85.01	12	4	4	Carbamidomethylation	36594	PREDICTED: probable voltage-gated potassium channel subunit beta [Vitis vinifera]
343	1956	gi|225442434	84.96	17	2	1		14228	PREDICTED: profilin-1 [Vitis vinifera]
607	17430	gi|224055647	84.96	9	3	3		32125	succinate dehydrogenase iron-sulfur protein subunit [Populus trichocarpa]

637	17625	gi|566204315	84.96	6	4	3	Carbamidomethylation	68355	hypothetical protein POPTR_0014s14690g [Populus trichocarpa]
754	18129	gi|566148278	84.95	8	3	2	Carbamidomethylation	38178	UDP-glucose 4-epimerase family protein [Populus trichocarpa]
529	2344	gi|225443154	84.94	18	3	3	Carbamidomethylation; Oxidation (M)	18245	PREDICTED: lamin-like protein [Vitis vinifera]
696	171	gi|225441044	84.88	7	4	1		57520	PREDICTED: pyruvate kinase cytosolic isozyme-like [Vitis vinifera]
292	493	gi|225436843	84.86	33	3	1		14570	PREDICTED: histone H2AX [Vitis vinifera]
277	18717	gi|224059156	84.78	18	2	1		14119	Profilin 3 family protein [Populus trichocarpa]
670	17710	gi|566153375	84.75	8	3	1		36703	hypothetical protein POPTR_0001s37920g [Populus trichocarpa]
847	18399	gi|566174518	84.73	9	2	2	Carbamidomethylation	40168	hypothetical protein POPTR_0006s04880g [Populus trichocarpa]
690	2328	gi|225435249	84.7	13	2	2		27570	PREDICTED: proteasome subunit beta type-4 [Vitis vinifera]
1010	854	gi|731402959	84.66	8	3	1		35210	PREDICTED: probable quinone oxidoreductase [Vitis vinifera]
641	292	gi|225439231	84.59	6	3	3	Carbamidomethylation	55389	PREDICTED: 3-isopropylmalate dehydratase large subunit-like [Vitis vinifera]
661	17721	gi|566171771	84.49	7	3	1		53299	hypothetical protein POPTR_0005s16590g partial [Populus trichocarpa]
439	16776	gi|224095561	84.43	5	4	1		97704	hypothetical protein POPTR_0007s01200g [Populus trichocarpa]
763	17497	gi|566205259	84.36	18	4	2	Carbamidomethylation	29312	hypothetical protein POPTR_0015s00690g [Populus trichocarpa]

1141	18667	gi|566207232	84.35	8	2	2		29661	hypothetical protein POPTR_0015s12390g [Populus trichocarpa]
473	17771	gi|566148911	84.15	5	2	1		48782	hypothetical protein POPTR_0001s14280g [Populus trichocarpa]
423	17352	gi|566156897	83.83	6	3	2		46943	Delta-aminolevulinic acid dehydratase family protein [Populus trichocarpa]
1027	715	gi|731436956	83.68	7	3	3	Carbamidomethylation	39713	PREDICTED: porphobilinogen deaminase chloroplastic [Vitis vinifera]
639	601	gi|731390491	83.68	3	2	2		84964	PREDICTED: protein transport protein SEC23-like [Vitis vinifera]
1150	2321	gi|731416683	83.52	20	2	1	Carbamidomethylation	15282	PREDICTED: superoxide dismutase [Cu-Zn] isoform X2 [Vitis vinifera]
731	992	gi|225441912	83.39	9	3	1	Oxidation (M)	30951	PREDICTED: S-formylglutathione hydrolase isoform X4 [Vitis vinifera]
1125	17838	gi|224072208	83.34	11	3	2	Carbamidomethylation	38044	UDP-glucose 4-epimerase family protein [Populus trichocarpa]
622	17744	gi|566172645	83.23	4	2	1	Carbamidomethylation	70694	hypothetical protein POPTR_0005s22040g [Populus trichocarpa]
1096	19132	gi|224073194	83.22	4	3	3		67506	10-formyltetrahydrofolate synthetase family protein [Populus trichocarpa]
873	581	gi|731401982	83.04	7	3	1	Carbamidomethylation	60389	PREDICTED: dihydrolipoyllysine-residue acetyltransferase component 2 of pyruvate dehydrogenase complex mitochondrial [Vitis vinifera]
434	604	gi|731401756	83.03	6	3	1	Carbamidomethylation	45526	PREDICTED: oligouridylate-binding protein 1B isoform X2 [Vitis vinifera]

546	466	gi|225426743	83.03	16	4	1	Carbamidomethylation	33675	PREDICTED: bifunctional dTDP-4-dehydrorhamnose 3 5-epimerase/dTDP-4-dehydrorhamnose reductase [Vitis vinifera]
877	1149	gi|225424881	83.02	5	2	1		48673	PREDICTED: uncharacterized protein LOC100248339 [Vitis vinifera]
1068	1496	gi|731392621	82.84	5	2	1		64193	PREDICTED: protein disulfide isomerase-like 1-4 isoform X2 [Vitis vinifera]
850	895	gi|731406257	82.56	8	4	2	Carbamidomethylation	45773	PREDICTED: oligouridylate-binding protein 1 isoform X2 [Vitis vinifera]
724	239	gi|225458111	82.53	5	5	1		136997	PREDICTED: coatomer subunit alpha-1 [Vitis vinifera]
484	769	gi|359488553	82.45	2	2	1		115409	PREDICTED: alpha-mannosidase isoform X2 [Vitis vinifera]
719	18064	gi|566149112	82.42	24	5	3	Carbamidomethylation	22610	GTP-binding family protein [Populus trichocarpa]
540	18797	gi|566198890	82.33	30	3	3	Carbamidomethylation	9241	hypothetical protein POPTR_0013s01830g [Populus trichocarpa]
880	686	gi|225429234	82.31	9	3	3		34319	PREDICTED: mitochondrial import receptor subunit TOM40-1 [Vitis vinifera]
657	570	gi|225458567	82.29	23	5	5	Carbamidomethylation	34519	PREDICTED: 26S proteasome non-ATPase regulatory subunit 14 homolog [Vitis vinifera]
1296	709	gi|359484512	82.24	5	2	2		63994	PREDICTED: asparagine--tRNA ligase cytoplasmic 1 [Vitis vinifera]
957	18282	gi|566192369	82.02	10	3	3	Carbamidomethylation	57235	Serine carboxypeptidase precursor family protein [Populus trichocarpa]
693	16576	gi|224119200	81.79	4	5	1		136903	coatomer alpha subunit-like family protein [Populus trichocarpa]

533	2140	gi|225449677	81.76	10	2	2		11280	PREDICTED: 60S acidic ribosomal protein P2 [Vitis vinifera]
567	1384	gi|225437898	81.72	15	3	2		15168	PREDICTED: photosystem I reaction center subunit IV B chloroplastic-like [Vitis vinifera]
684	17548	gi|224139168	81.62	6	3	1	Carbamidomethylation; Oxidation (M)	65070	pyruvate decarboxylase family protein [Populus trichocarpa]
766	17374	gi|566147333	81.48	3	3	1		100058	hypothetical protein POPTR_0001s05320g [Populus trichocarpa]
794	276	gi|225432012	81.32	11	4	1	Carbamidomethylation	54243	PREDICTED: galactokinase [Vitis vinifera]
823	1233	gi|225448904	81.22	7	2	1		48063	PREDICTED: malate dehydrogenase [NADP] chloroplastic [Vitis vinifera]
961	2312	gi|225457389	81.17	8	2	2		27867	PREDICTED: chlorophyll a-b binding protein 4 chloroplastic [Vitis vinifera]
448	17984	gi|224078816	80.48	9	3	1		36460	Leucoanthocyanidin reductase family protein [Populus trichocarpa]
444	1421	gi|731386809	80.26	8	2	1		41704	PREDICTED: naringenin 2-oxoglutarate 3-dioxygenase-like [Vitis vinifera]
768	19424	gi|566211207	80.1	8	3	1	Carbamidomethylation; Oxidation (M)	38545	hypothetical protein POPTR_0017s03780g [Populus trichocarpa]
747	1507	gi|225470846	79.6	13	2	2		22027	PREDICTED: nascent polypeptide-associated complex subunit alpha-like protein 1 [Vitis vinifera]
941	17641	gi|566204499	79.58	7	2	1		47320	thioredoxin family protein [Populus trichocarpa]
381	17469	gi|224079472	79.53	5	2	2		47114	hypothetical protein POPTR_0004s08560g [Populus trichocarpa]

907	796	gi|225423755	79.46	7	3	3		44272	PREDICTED: photosystem II stability/assembly factor HCF136 chloroplastic [Vitis vinifera]
1143	2166	gi|359473071	79.45	4	2	2	Carbamidomethylation	65661	PREDICTED: bifunctional purine biosynthesis protein purH isoform X2 [Vitis vinifera]
432	17131	gi|224057882	79.28	5	4	3		89495	outer membrane family protein [Populus trichocarpa]
359	850	gi|225450384	79.23	4	3	2	Carbamidomethylation	57022	PREDICTED: beta-glucosidase 12-like [Vitis vinifera]
950	422	gi|731439184	79.11	6	3	1		109426	PREDICTED: 26S proteasome non-ATPase regulatory subunit 1 homolog A [Vitis vinifera]
544	18175	gi|566202597	79.1	22	3	1		16336	hypothetical protein POPTR_0014s04880g [Populus trichocarpa]
364	18170	gi|566157284	79.02	4	2	1	Carbamidomethylation	48713	chloroplast nucleoid DNA-binding family protein [Populus trichocarpa]
809	17351	gi|224108774	78.9	11	4	1	Carbamidomethylation	54256	Galactokinase family protein [Populus trichocarpa]
595	1304	gi|359475330	78.9	14	2	1		16329	PREDICTED: glycine-rich RNA-binding protein GRP2A [Vitis vinifera]
746	1323	gi|526117539	78.86	10	2	1	Carbamidomethylation	34687	class I extracellular chitinase precursor [Vitis vinifera]
878	61273	gi|225457387	78.47	8	1	1		26287	PREDICTED: expansin-A1 [Vitis vinifera]
1033	776	gi|731394453	78.42	7	3	3		46372	PREDICTED: dnaJ protein homolog isoform X2 [Vitis vinifera]
667	17535	gi|566180192	78.3	9	2	2		27792	hypothetical protein POPTR_0007s08020g [Populus trichocarpa]
288	719	gi|225424242	78.27	30	3	1		10611	PREDICTED: 10 kDa chaperonin [Vitis vinifera]
718	1500	gi|225455141	78.2	37	2	1		11333	PREDICTED: protein SPIRAL1-like 1 [Vitis vinifera]

344	19092	gi|224141613	78.1	18	2	1		14037	profilin 2 family protein [Populus trichocarpa]
459	2095	gi|225447745	78.06	8	1	1		27216	PREDICTED: chlorophyll a-b binding protein CP24 10A chloroplastic [Vitis vinifera]
844	1439	gi|731428465	77.86	6	2	2		37487	PREDICTED: thylakoid lumenal 29 kDa protein chloroplastic [Vitis vinifera]
679	651	gi|225434329	77.65	7	4	3		57685	PREDICTED: trans-cinnamate 4-monooxygenase [Vitis vinifera]
386	2534	gi|731417951	77.61	10	3	3		27594	PREDICTED: allene oxide cyclase [Vitis vinifera]
965	2325	gi|225443274	77.37	8	2	2		35506	PREDICTED: cell wall integrity protein scw1 [Vitis vinifera]
1441	787	gi|225450299	77.23	4	1	1		52389	PREDICTED: citrate synthase mitochondrial [Vitis vinifera]
916	18020	gi|224140807	77.22	3	3	1		109369	26S proteasome regulatory subunit family protein [Populus trichocarpa]
1017	1216	gi|731434246	77.05	4	2	2	Carbamidomethylation	56111	PREDICTED: NADH dehydrogenase [ubiquinone] flavoprotein 1 mitochondrial isoform X1 [Vitis vinifera]
620	16610	gi|566159486	77.03	3	3	1		113839	hypothetical protein POPTR_0002s22720g [Populus trichocarpa]
714	443	gi|731395934	76.87	7	3	1	Carbamidomethylation	58724	PREDICTED: putative proline--tRNA ligase C19C7.06 [Vitis vinifera]
662	824	gi|225451235	76.6	8	2	1		34370	PREDICTED: cysteine synthase [Vitis vinifera]
572	17746	gi|566215609	76.36	3	2	1		91834	hypothetical protein POPTR_0018s13110g [Populus trichocarpa]
1194	17864	gi|566153954	76.19	10	3	3		31003	phosphatase 2C family protein [Populus trichocarpa]
829	17743	gi|566175322	76.03	9	2	2		35273	aldo/keto reductase family protein [Populus trichocarpa]

1363	1362	gi|225440230	75.52	7	1	1		29238	PREDICTED: dihydrofolate reductase [Vitis vinifera]
611	1269	gi|225459906	75.45	8	3	2		34942	PREDICTED: probable fructokinase-5 [Vitis vinifera]
735	17382	gi|566183730	75.43	10	3	2	Carbamidomethylation	48131	hypothetical protein POPTR_0008s13180g [Populus trichocarpa]
1049	2128	gi|731439300	75.36	4	1	1		49685	PREDICTED: fumarylacetoacetase [Vitis vinifera]
853	71069	gi|566241274	75.36	4	2	1		58491	hypothetical protein POPTR_0019s118101g partial [Populus trichocarpa]
1216	2125	gi|731415654	75.07	3	2	2		73684	PREDICTED: acyl-CoA-binding domain-containing protein 4-like isoform X2 [Vitis vinifera]
613	970	gi|731413121	74.91	29	3	1	Carbamidomethylation	15960	PREDICTED: actin-depolymerizing factor 2-like isoform X2 [Vitis vinifera]
1142	1611	gi|731417625	74.84	12	2	2		19282	PREDICTED: uncharacterized protein LOC100241624 [Vitis vinifera]
894	1274	gi|225447549	74.73	4	2	1		66322	PREDICTED: glucose-6-phosphate 1-dehydrogenase chloroplastic-like [Vitis vinifera]
702	874	gi|359478691	74.73	4	3	3		92178	PREDICTED: beta-galactosidase 8 [Vitis vinifera]
770	1503	gi|225461001	74.72	7	2	2		46284	PREDICTED: polyadenylate-binding protein RBP45-like [Vitis vinifera]
362	1286	gi|731433731	74.65	8	3	1		30030	PREDICTED: glutathione S-transferase U21-like [Vitis vinifera]
483	17782	gi|224143559	74.59	9	3	1		44475	aspartate aminotransferase family protein [Populus trichocarpa]

1457	18343	gi|566161304	74.56	5	2	1		52669	hypothetical protein POPTR_0003s07020g [Populus trichocarpa]
936	17567	gi|224071575	74.27	6	4	1		65504	stress inducible family protein [Populus trichocarpa]
1029	951	gi|731371455	74.11	4	2	2		58282	PREDICTED: coatomer subunit delta [Vitis vinifera]
983	18541	gi|224103381	73.7	3	2	2		53021	diaminopimelate decarboxylase family protein [Populus trichocarpa]
275	1137	gi|526117471	73.48	11	3	2		27242	class IV endochitinase precursor [Vitis vinifera]
783	18001	gi|566202441	73.44	4	2	2		69477	DYNAMIN-LIKE protein 4 [Populus trichocarpa]
909	18514	gi|566165507	73.26	3	3	2	Carbamidomethylation	92789	hypothetical protein POPTR_0004s07940g [Populus trichocarpa]
139	19801	gi|224093204	73.23	5	2	1	Carbamidomethylation	35946	peroxidase precursor family protein [Populus trichocarpa]
987	16721	gi|566157090	73.14	1	3	3		252616	hypothetical protein POPTR_0002s09330g [Populus trichocarpa]
739	17366	gi|566149176	72.94	4	3	3		74291	acyl-CoA oxidase family protein [Populus trichocarpa]
1090	17325	gi|224086345	72.84	3	3	1		103853	ATPase 6 family protein [Populus trichocarpa]
1059	1301	gi|731426495	72.75	2	2	1		120041	PREDICTED: alpha-mannosidase [Vitis vinifera]
487	18733	gi|224143583	72.69	11	3	3		17416	peroxiredoxin family protein [Populus trichocarpa]
532	2407	gi|359495882	72.5	6	2	1	Carbamidomethylation	39482	PREDICTED: 2-alkenal reductase (NADP(+)-dependent) [Vitis vinifera]
549	784	gi|225462809	72.35	4	2	2		60994	PREDICTED: 2-hydroxyacyl-CoA lyase [Vitis vinifera]
1160	18395	gi|566182365	72.34	8	2	2		34377	hypothetical protein POPTR_0008s04660g [Populus trichocarpa]

414	18297	gi|566192144	72.22	6	2	2		36328	hypothetical protein POPTR_0010s22170g [Populus trichocarpa]
910	135	gi|731402494	72.12	2	2	2		102377	PREDICTED: coatomer subunit beta'-1 [Vitis vinifera]
1159	1849	gi|731397296	72.1	5	2	1		44232	PREDICTED: bifunctional aspartate aminotransferase and glutamate/aspartate-prephenate aminotransferase isoform X2 [Vitis vinifera]
1065	722	gi|225430200	71.99	5	2	1		40334	PREDICTED: uncharacterized protein LOC100252479 [Vitis vinifera]
760	2049	gi|731411015	71.91	5	2	2		46869	PREDICTED: 3-isopropylmalate dehydrogenase chloroplastic [Vitis vinifera]
1026	942	gi|225453935	71.79	10	3	3		40128	PREDICTED: 4-hydroxy-tetrahydrodipicolinate synthase chloroplastic [Vitis vinifera]
920	1228	gi|731439510	71.71	13	4	4	Carbamidomethylation	41887	PREDICTED: acetyl-CoA acetyltransferase cytosolic 1 isoform X2 [Vitis vinifera]
471	17044	gi|566194782	71.53	5	4	1	Carbamidomethylation	75679	NAD-dependent epimerase/dehydratase family protein [Populus trichocarpa]
954	519	gi|225450981	71.3	5	2	2	Carbamidomethylation	51125	PREDICTED: serine--tRNA ligase [Vitis vinifera]
774	950	gi|225440362	71.26	32	3	3	Carbamidomethylation; Oxidation (M)	10797	PREDICTED: mitochondrial import inner membrane translocase subunit Tim9 [Vitis vinifera]
1028	17403	gi|224119184	71.17	13	3	1	Carbamidomethylation	35019	type 2A protein phosphatase-2 [Populus trichocarpa]
1042	1569	gi|225457458	71.17	10	3	3		17173	PREDICTED: transcription factor BTF3 homolog 4 [Vitis vinifera]
615	18734	gi|566214355	71.13	7	2	1		25528	hypothetical protein POPTR_0018s05350g partial [Populus trichocarpa]

796	18859	gi|224107333	70.96	26	2	2		9716	hypothetical protein POPTR_0010s02580g [Populus trichocarpa]
1050	496	gi|225449659	70.95	5	3	1		57679	PREDICTED: pyruvate kinase cytosolic isozyme-like [Vitis vinifera]
493	669	gi|731386337	70.86	13	2	2		20640	PREDICTED: serine/arginine-rich splicing factor RSZ22 isoform X2 [Vitis vinifera]
874	18206	gi|566207525	70.78	4	3	1	Carbamidomethylation	83959	subtilase family protein [Populus trichocarpa]
946	17577	gi|566188043	70.78	6	2	2		43097	hypothetical protein POPTR_0009s14830g [Populus trichocarpa]
952	18215	gi|566147574	70.52	8	2	2		44838	hypothetical protein POPTR_0001s06560g [Populus trichocarpa]
793	18384	gi|224146080	70.48	14	2	2		17003	hypothetical protein POPTR_0019s05840g [Populus trichocarpa]
492	836	gi|225458231	70.43	7	2	2		30646	PREDICTED: proteasome subunit alpha type-1-B [Vitis vinifera]
1091	18379	gi|566182095	70.4	9	3	2		48288	hypothetical protein POPTR_0008s03160g [Populus trichocarpa]
887	17596	gi|566171781	70.25	3	3	2	Carbamidomethylation	84742	hypothetical protein POPTR_0005s16660g [Populus trichocarpa]
857	1380	gi|731390561	70.03	4	2	2	Carbamidomethylation	58745	PREDICTED: probable aldo-keto reductase 4 [Vitis vinifera]
1324	20107	gi|224138170	70.02	29	2	1		7523	hypothetical protein POPTR_0016s06300g [Populus trichocarpa]
699	17848	gi|224112931	69.86	3	2	2		70298	peptidase M1 family protein [Populus trichocarpa]
743	18092	gi|566156270	69.71	4	4	1	Carbamidomethylation	75868	hypothetical protein POPTR_0002s04800g [Populus trichocarpa]

716	18942	gi|566197584	69.64	5	2	1		42529	hypothetical protein POPTR_0012s09200g [Populus trichocarpa]
1158	19243	gi|224134737	69.5	7	1	1		23290	cytochrome b5 domain-containing family protein [Populus trichocarpa]
890	2338	gi|359496232	69.42	11	2	2		21717	PREDICTED: probable NAD(P)H dehydrogenase (quinone) FQR1-like 1 [Vitis vinifera]
911	17168	gi|224090159	69.33	2	2	2		151232	magnesium chelatase subunit family protein [Populus trichocarpa]
1024	1995	gi|731412542	69.22	5	3	3		58149	PREDICTED: uncharacterized protein LOC100244432 isoform X4 [Vitis vinifera]
695	18416	gi|224071567	69.18	4	2	1		65821	monocopper oxidase precursor family protein [Populus trichocarpa]
665	17587	gi|566184208	69.07	10	3	3	Carbamidomethylation	27210	hypothetical protein POPTR_0008s15820g [Populus trichocarpa]
776	17159	gi|224097404	68.79	9	2	2	Carbamidomethylation	34870	type X protein phosphatase-II [Populus trichocarpa]
851	1009	gi|359480225	68.58	19	4	1	Carbamidomethylation	27397	PREDICTED: peptidyl-prolyl cis-trans isomerase [Vitis vinifera]
1087	1842	gi|225447176	68.55	4	2	1		46608	PREDICTED: protein disulfide isomerase-like 2-3 [Vitis vinifera]
721	19786	gi|566207513	68.47	2	2	1		68058	hypothetical protein POPTR_0015s14020g [Populus trichocarpa]
623	18856	gi|224099491	68.43	24	2	1		10451	hypothetical protein POPTR_0008s12950g [Populus trichocarpa]
1020	357	gi|731399677	68.4	3	3	1	Carbamidomethylation	102437	PREDICTED: protein argonaute 4A-like [Vitis vinifera]

1093	2627	gi|731393556	68.28	8	2	2		29837	PREDICTED: rhodanese-like/PpiC domain-containing protein 12 isoform X2 [Vitis vinifera]
610	17842	gi|224057968	68.27	8	2	1		24349	photosystem I chain III family protein [Populus trichocarpa]
940	18534	gi|224063774	68.13	3	2	1		55604	3-phosphoshikimate 1-carboxyvinyltransferase family protein [Populus trichocarpa]
998	1092	gi|225456481	68.03	2	2	1		112435	PREDICTED: alpha-mannosidase [Vitis vinifera]
913	903	gi|225453158	68.03	5	2	1		41234	PREDICTED: probable fructokinase-6 chloroplastic [Vitis vinifera]
780	17094	gi|224065699	67.87	2	1	1		75559	Cell division protein ftsH [Populus trichocarpa]
967	2854	gi|731408416	67.86	4	2	1		53964	PREDICTED: serine carboxypeptidase-like 13 [Vitis vinifera]
1084	1487	gi|225460716	67.85	5	2	2	Carbamidomethylation	49588	PREDICTED: dihydrolipoyllysine-residue acetyltransferase component 5 of pyruvate dehydrogenase complex chloroplastic [Vitis vinifera]
1085	868	gi|225455106	67.81	7	2	1		33350	PREDICTED: protein BOBBER 1 [Vitis vinifera]
1161	1861	gi|731428311	67.56	4	2	1		51458	PREDICTED: bifunctional aspartate aminotransferase and glutamate/aspartate-prephenate aminotransferase-like [Vitis vinifera]
734	1851	gi|225439145	67.5	6	2	2		37191	PREDICTED: grpE protein homolog mitochondrial isoform X1 [Vitis vinifera]
771	1504	gi|731426021	67.41	30	2	1		12464	PREDICTED: protein SPIRAL1-like 1 isoform X2 [Vitis vinifera]

685	920	gi|225436438	67.27	19	2	2		10607	PREDICTED: cyclin-dependent kinases regulatory subunit 1 [Vitis vinifera]
573	1493	gi|225465605	67.11	3	2	1	Oxidation (M)	68015	PREDICTED: lysosomal beta glucosidase-like [Vitis vinifera]
498	1101	gi|225434062	67.1	16	4	2	Carbamidomethylation	28430	PREDICTED: chitinase 5-like [Vitis vinifera]
1126	794	gi|731374652	67.04	6	2	1		58335	PREDICTED: aldehyde dehydrogenase family 2 member B4 mitochondrial [Vitis vinifera]
978	693	gi|225437557	66.95	4	2	2		81022	PREDICTED: DNA replication licensing factor MCM7 [Vitis vinifera]
903	2381	gi|731384053	66.9	4	2	1		52608	PREDICTED: LL-diaminopimelate aminotransferase chloroplastic isoform X1 [Vitis vinifera]
1149	696	gi|225455513	66.9	16	2	2	Carbamidomethylation	13646	PREDICTED: 40S ribosomal protein S20-2 [Vitis vinifera]
1004	431	gi|731391105	66.78	7	3	1	Carbamidomethylation	49499	PREDICTED: calreticulin-3-like [Vitis vinifera]
915	17512	gi|566158761	66.57	17	4	1	Carbamidomethylation	29101	cyclophilin family protein [Populus trichocarpa]
709	1201	gi|225454278	66.54	6	2	2		40499	PREDICTED: bifunctional L-3-cyanoalanine synthase/cysteine synthase 1 mitochondrial [Vitis vinifera]
451	1075	gi|526117894	66.29	7	3	1		36735	anthocyanidin reductase [Vitis vinifera]
885	2329	gi|225435002	65.99	6	1	1	Carbamidomethylation	27396	PREDICTED: osmotin-like protein [Vitis vinifera]
1152	17671	gi|566183513	65.99	4	2	2		62808	hypothetical protein POPTR_0008s11770g [Populus trichocarpa]
789	17372	gi|224063299	65.98	4	2	2		67707	hypothetical protein POPTR_0002s10420g [Populus trichocarpa]

1031	19431	gi|566151403	65.96	28	2	2		8809	hypothetical protein POPTR_0001s28090g [Populus trichocarpa]
828	18273	gi|224106686	65.92	10	2	1		24434	elongation factor 1B alpha-subunit 2 family protein [Populus trichocarpa]
1099	19371	gi|566184421	65.8	7	2	2		45388	hypothetical protein POPTR_0008s17030g [Populus trichocarpa]
1295	18312	gi|224082494	65.69	5	2	2		38884	peroxidase precursor family protein [Populus trichocarpa]
645	2197	gi|526118093	65.64	13	2	2	Carbamidomethylation	22511	type II peroxiredoxin E [Vitis vinifera]
811	1526	gi|225440674	65.52	5	1	1		31431	PREDICTED: chlorophyll a-b binding protein CP29.1 chloroplastic [Vitis vinifera]
787	1838	gi|225440564	65.39	4	1	1		44667	PREDICTED: fructose-1 6-bisphosphatase chloroplastic-like [Vitis vinifera]
959	18943	gi|224140195	65.39	12	2	2		30031	29 kDa ribonucleoprotein [Populus trichocarpa]
1005	18013	gi|566174458	65.32	17	3	1	Carbamidomethylation; Oxidation (M)	23355	hypothetical protein POPTR_0006s04560g [Populus trichocarpa]
923	19722	gi|566148052	65.12	8	1	1		17358	hypothetical protein POPTR_0001s09270g [Populus trichocarpa]
1215	17557	gi|566168605	64.95	7	3	1		44259	hypothetical protein POPTR_0005s00670g [Populus trichocarpa]
918	18134	gi|566258065	64.82	9	1	1		16877	hypothetical protein POPTR_0133s00270g partial [Populus trichocarpa]
884	19687	gi|224089062	64.45	6	1	1		27753	hypothetical protein POPTR_0006s25870g [Populus trichocarpa]
560	19104	gi|224078057	64.45	5	1	1		28150	phosphorylase family protein [Populus trichocarpa]

958	17534	gi|224103431	64.39	10	2	1		23027	RAS-related GTP-binding family protein [Populus trichocarpa]
681	18430	gi|224064818	64.18	4	2	1		47984	hypothetical protein POPTR_0002s22410g [Populus trichocarpa]
845	1358	gi|731409226	64.05	4	2	2		43860	PREDICTED: 3-hydroxyisobutyryl-CoA hydrolase-like protein 5 [Vitis vinifera]
1466	71086	gi|225462878	64.04	15	1	1		21254	PREDICTED: polyadenylate-binding protein 3-like isoform X1 [Vitis vinifera]
951	19451	gi|566160279	64	5	1	1		36389	hypothetical protein POPTR_0003s01450g [Populus trichocarpa]
1092	1348	gi|225462545	63.9	15	3	3	Oxidation (M)	27262	PREDICTED: proteasome subunit alpha type-4 [Vitis vinifera]
933	744	gi|359497196	63.88	14	3	2		23133	PREDICTED: ras-related protein RABH1e [Vitis vinifera]
494	1812	gi|225430510	63.76	6	3	1		27121	PREDICTED: phospholipid hydroperoxide glutathione peroxidase 1 chloroplastic [Vitis vinifera]
517	1743	gi|225441809	63.67	29	3	3		8917	PREDICTED: uncharacterized protein LOC100245204 isoform X3 [Vitis vinifera]
583	521	gi|225458331	63.67	5	2	2		49132	PREDICTED: eukaryotic peptide chain release factor subunit 1-3 [Vitis vinifera]
1162	18938	gi|566170130	63.53	8	2	1		51590	hypothetical protein POPTR_0005s08100g [Populus trichocarpa]
1007	1666	gi|526117699	63.52	4	2	2		49406	3-dehydroquinate synthase-like [Vitis vinifera]
919	2600	gi|526117629	63.43	8	2	2	Carbamidomethylation	11686	lipid transfer protein precursor [Vitis vinifera]

1006	60998	gi|566171779	63.42	2	2	1	Carbamidomethylation	84881	hypothetical protein POPTR_0005s16650g partial [Populus trichocarpa]
1326	2392	gi|731382564	63.31	4	2	2	Carbamidomethylation	53798	PREDICTED: aminoacylase-1 isoform X2 [Vitis vinifera]
426	17604	gi|566194465	63.22	7	2	2		24921	SGS domain-containing family protein [Populus trichocarpa]
514	1264	gi|225442963	62.98	9	3	3		35176	PREDICTED: methyl-CpG-binding domain-containing protein 11 [Vitis vinifera]
1030	310	gi|225429772	62.85	3	3	1		104811	PREDICTED: plasma membrane ATPase 4 [Vitis vinifera]
922	18008	gi|566148837	62.37	5	2	2	Carbamidomethylation	30785	hypothetical protein POPTR_0001s13880g [Populus trichocarpa]
707	17276	gi|566170117	62.35	7	3	1		48844	hypothetical protein POPTR_0005s08050g [Populus trichocarpa]
1205	17498	gi|566160463	62.28	7	2	2		49456	hypothetical protein POPTR_0003s02420g [Populus trichocarpa]
1582	273	gi|731411266	62.01	1	1	1		134450	PREDICTED: cullin-associated NEDD8-dissociated protein 1 [Vitis vinifera]
988	17093	gi|224067984	61.78	3	2	2		68220	dynamin-like protein B [Populus trichocarpa]
1285	18022	gi|224112819	61.68	17	2	1	Carbamidomethylation	16047	actin-depolymerizing factor family protein [Populus trichocarpa]
973	1382	gi|359489656	61.68	6	2	2		52031	PREDICTED: probable polygalacturonase [Vitis vinifera]
966	18707	gi|224064081	61.48	9	2	2		28003	hypothetical protein POPTR_0002s16690g [Populus trichocarpa]
668	18137	gi|224087780	61.33	4	2	1		65398	pyruvate decarboxylase family protein [Populus trichocarpa]

1086	1211	gi|731391365	61.24	4	2	2	Carbamidomethylation	52738	PREDICTED: acetolactate synthase small subunit 2 chloroplastic isoform X2 [Vitis vinifera]
1664	71160	gi|225444938	61.08	3	1	1		53784	PREDICTED: ectonucleotide pyrophosphatase/phosphodiesterase family member 3 [Vitis vinifera]
792	126	gi|359486157	61.08	6	2	2		44506	PREDICTED: 60S ribosomal protein L3-2 isoform X1 [Vitis vinifera]
1307	1177	gi|225441573	60.67	3	3	3	Carbamidomethylation	107099	PREDICTED: eukaryotic translation initiation factor 3 subunit C [Vitis vinifera]
1011	1573	gi|731428130	60.63	5	3	3	Carbamidomethylation	49925	PREDICTED: kynurenine--oxoglutarate transaminase 1 [Vitis vinifera]
852	18117	gi|566167194	60.5	8	2	2	Carbamidomethylation	41861	glycerate dehydrogenase family protein [Populus trichocarpa]
859	1946	gi|225440849	60.41	19	1	1		8952	PREDICTED: probable small nuclear ribonucleoprotein G [Vitis vinifera]
1163	61208	gi|224109620	60.13	6	2	1		24252	chalcone isomerase family protein [Populus trichocarpa]
993	28950	gi|359483854	60.05	8	3	3	Carbamidomethylation	49342	PREDICTED: 3-oxoacyl-[acyl-carrier-protein] synthase I chloroplastic [Vitis vinifera]
1140	19475	gi|731439030	60.04	5	1	1		34963	PREDICTED: ATP-dependent Clp protease proteolytic subunit-related protein 4 chloroplastic-like [Vitis vinifera]
1066	17463	gi|224133304	59.73	2	1	1		60007	hypothetical protein POPTR_0015s07690g [Populus trichocarpa]
866	1287	gi|225460991	59.64	4	2	2		86153	PREDICTED: heat shock 70 kDa protein 16 [Vitis vinifera]
1460	18018	gi|224083504	59.6	2	2	2		105368	hypothetical protein POPTR_0005s07000g [Populus trichocarpa]

1208	6864	gi|359475352	59.6	3	1	1		49145	PREDICTED: uncharacterized protein At4g06744 [Vitis vinifera]
1044	19808	gi|566160273	59.48	6	1	1	Carbamidomethylation	17554	hypothetical protein POPTR_0003s01440g [Populus trichocarpa]
585	2306	gi|526117918	59.39	5	1	1		33457	basic endochitinase precursor [Vitis vinifera]
749	391	gi|225425280	59.3	5	2	1		48103	PREDICTED: uncharacterized protein LOC100247241 [Vitis vinifera]
1426	18884	gi|566207658	59.14	3	2	2		58317	clathrin adaptor complexes medium subunit-related family protein [Populus trichocarpa]
1503	20127	gi|225435931	59.13	11	2	1		13713	PREDICTED: cysteine proteinase inhibitor A [Vitis vinifera]
1101	2442	gi|225429295	58.98	8	2	1		25371	PREDICTED: uncharacterized protein LOC100254416 [Vitis vinifera]
848	19311	gi|566172679	58.98	2	1	1		63027	hypothetical protein POPTR_0005s22250g [Populus trichocarpa]
722	18082	gi|566176510	58.87	7	3	1		43124	nucleosome assembly family protein [Populus trichocarpa]
1138	18213	gi|224118508	58.85	7	2	2		39734	Chain A family protein [Populus trichocarpa]
858	29005	gi|224084784	58.7	3	2	1		61214	glycine hydroxymethyltransferase family protein [Populus trichocarpa]
1365	910	gi|225449380	58.64	4	1	1		39535	PREDICTED: mannose-1-phosphate guanylyltransferase 1 [Vitis vinifera]
1506	1120	gi|225465441	58.6	12	1	1		14675	PREDICTED: cytochrome b5 [Vitis vinifera]
843	1310	gi|731401385	58.57	5	2	2		36064	PREDICTED: uncharacterized protein At5g02240 [Vitis vinifera]

671	1615	gi|359497056	58.37	5	2	1		35941	PREDICTED: peroxidase 10-like [Vitis vinifera]
406	1422	gi|731439999	58.21	2	2	1	Carbamidomethylation	80675	PREDICTED: primary amine oxidase-like [Vitis vinifera]
1308	19771	gi|224106191	58.14	6	2	2	Carbamidomethylation	35572	cinnamyl-alcohol dehydrogenase family protein [Populus trichocarpa]
748	1292	gi|731378427	58.02	5	3	2		51975	PREDICTED: uncharacterized protein LOC100263310 [Vitis vinifera]
1070	18310	gi|224143674	58	5	2	2		38385	fructose-1 family protein [Populus trichocarpa]
818	1779	gi|225448795	57.93	11	3	3	Carbamidomethylation	19172	PREDICTED: cytochrome c oxidase subunit 6b-1 isoform X4 [Vitis vinifera]
1257	18812	gi|671741947	57.92	22	2	2		9397	photosystem II cytochrome b559 alpha subunit (chloroplast) [Camellia grandibracteata]
518	800	gi|225450811	57.87	12	2	1		14652	PREDICTED: histone H2A.1 [Vitis vinifera]
1593	61026	gi|731393292	57.83	2	1	1		72077	PREDICTED: glucosidase 2 subunit beta isoform X2 [Vitis vinifera]
1134	611	gi|225437455	57.66	2	1	1		53532	PREDICTED: fumarate hydratase 1 mitochondrial [Vitis vinifera]
1202	1309	gi|731398937	57.61	15	2	1	Carbamidomethylation	21183	PREDICTED: actin-depolymerizing factor 1-like [Vitis vinifera]
285	914	gi|225440109	57.45	13	2	1		10658	PREDICTED: 10 kDa chaperonin [Vitis vinifera]
881	2234	gi|225441977	57.38	2	2	1		66737	PREDICTED: pectinesterase [Vitis vinifera]
1035	5205	gi|359482448	57.28	2	2	1	Carbamidomethylation	83440	PREDICTED: subtilisin-like protease [Vitis vinifera]
1507	61055	gi|224081483	57.27	10	1	1		15916	hypothetical protein POPTR_0005s10440g [Populus trichocarpa]

819	19744	gi|224137260	57.25	12	1	1		11459	hypothetical protein POPTR_0016s01100g [Populus trichocarpa]
1058	17099	gi|566149188	57.24	7	1	1		24300	60S ribosomal protein L15 [Populus trichocarpa]
846	17730	gi|566189053	57.19	2	1	1		51981	hypothetical protein POPTR_0010s04610g [Populus trichocarpa]
558	2865	gi|359479995	57.13	2	2	1		61649	PREDICTED: pectinesterase-like [Vitis vinifera]
1436	2403	gi|225470816	57.09	23	1	1		5428	PREDICTED: wound-induced basic protein [Vitis vinifera]
833	423	gi|225434133	56.87	6	2	2		40613	PREDICTED: cysteine synthase chloroplastic/chromoplastic [Vitis vinifera]
757	306	gi|731438745	56.77	2	2	1		92847	PREDICTED: DNA replication licensing factor MCM6 [Vitis vinifera]
1462	18387	gi|566160574	56.64	2	1	1		57226	serine/threonine protein phosphatase 2A 55 kDa regulatory subunit B [Populus trichocarpa]
1034	1167	gi|225457034	56.59	4	2	2	Carbamidomethylation	51176	PREDICTED: 1-deoxy-D-xylulose 5-phosphate reductoisomerase chloroplastic [Vitis vinifera]
1041	71043	gi|224055553	56.53	13	3	3		19637	50S ribosomal protein L12-2 [Populus trichocarpa]
968	28940	gi|566162305	56.29	6	2	1	Carbamidomethylation	38904	UDP-glucose 4-epimerase family protein [Populus trichocarpa]
938	653	gi|225449060	56.2	5	3	2	Carbamidomethylation	62143	PREDICTED: protein disulfide isomerase-like 1-4 [Vitis vinifera]
1219	18326	gi|224109088	56.15	2	1	1		57503	SUCCINIC SEMIALDEHYDE DEHYDROGENASE family protein [Populus trichocarpa]
1509	2502	gi|225424908	56.14	4	2	2		29426	PREDICTED: mitochondrial outer membrane protein porin of 36 kDa [Vitis vinifera]

969	20226	gi|566161648	55.4	5	1	1		17801	glycine cleavage system protein H precursor [Populus trichocarpa]
1008	17393	gi|566151113	55.33	1	1	1		76209	hypothetical protein POPTR_0001s26420g [Populus trichocarpa]
1057	19441	gi|566166966	55.23	6	2	2		33173	hypothetical protein POPTR_0004s17010g [Populus trichocarpa]
738	29860	gi|566201923	54.9	3	2	2		43928	hypothetical protein POPTR_0014s01960g [Populus trichocarpa]
1036	1542	gi|225458237	54.89	5	1	1	Carbamidomethylation	29571	PREDICTED: gamma carbonic anhydrase 1 mitochondrial [Vitis vinifera]
1303	19816	gi|566197446	54.84	3	1	1		44436	hypothetical protein POPTR_0012s08440g [Populus trichocarpa]
1156	19582	gi|224062109	54.69	3	1	1		38699	dihydroflavonol reductase family protein [Populus trichocarpa]
1227	2552	gi|731393274	54.64	1	1	1		76795	PREDICTED: uncharacterized protein LOC100253534 isoform X2 [Vitis vinifera]
1103	1632	gi|225447707	54.61	6	1	1		27098	PREDICTED: uncharacterized protein LOC100263170 [Vitis vinifera]
1504	894	gi|225444609	54.52	7	1	1		26208	PREDICTED: eukaryotic translation initiation factor 3 subunit K [Vitis vinifera]
897	19869	gi|566170529	53.93	5	2	1		40018	hypothetical protein POPTR_0005s10260g [Populus trichocarpa]
1287	18403	gi|566171982	53.9	4	1	1		38050	hypothetical protein POPTR_0005s18180g [Populus trichocarpa]
435	1077	gi|225448675	53.88	10	2	2		23332	PREDICTED: probable chalcone--flavonone isomerase 3 [Vitis vinifera]

888	367	gi|225444472	53.72	8	3	3	Carbamidomethylation	44306	PREDICTED: aminomethyltransferase mitochondrial [Vitis vinifera]
864	2050	gi|731393849	53.52	3	1	1		56128	PREDICTED: UBP1-associated protein 2A [Vitis vinifera]
889	33319	gi|224062595	53.3	6	2	2		28191	photosystem II oxygen-evolving complex protein 2 precursor [Populus trichocarpa]
1366	18976	gi|566180962	52.8	3	1	1		60958	hypothetical protein POPTR_0007s12550g [Populus trichocarpa]
1128	243	gi|225429750	52.78	1	1	1	Carbamidomethylation	105919	PREDICTED: coatomer subunit beta-1 [Vitis vinifera]
1204	445	gi|225454087	52.73	3	2	1		66696	PREDICTED: NAD-dependent malic enzyme 59 kDa isoform mitochondrial [Vitis vinifera]
924	18794	gi|224066629	52.71	8	1	1		16281	thioredoxin family protein [Populus trichocarpa]
1720	19719	gi|566152742	52.46	10	1	1		14748	double-stranded DNA-binding family protein [Populus trichocarpa]
759	717	gi|225463033	52.45	2	2	2		110326	PREDICTED: 116 kDa U5 small nuclear ribonucleoprotein component [Vitis vinifera]
1203	17214	gi|224127578	52.36	3	2	1		67172	NAD-dependent malic enzyme family protein [Populus trichocarpa]
1859	1340	gi|225442595	52.36	2	1	1		60200	PREDICTED: protein disulfide isomerase-like 1-6 [Vitis vinifera]
745	19380	gi|731407226	52.27	4	1	1	Carbamidomethylation	37934	PREDICTED: 2-alkenal reductase (NADP(+)-dependent) [Vitis vinifera]
999	1435	gi|225464870	52.17	4	1	1	Carbamidomethylation	37627	PREDICTED: bifunctional nitrilase/nitrile hydratase NIT4B [Vitis vinifera]
799	1811	gi|225428031	51.96	4	2	2		45128	PREDICTED: WD-40 repeat-containing protein MSI3 [Vitis vinifera]

624	18132	gi|566151049	51.71	6	2	2		26982	hypothetical protein POPTR_0001s26020g [Populus trichocarpa]
1061	471	gi|225433575	51.55	1	1	1		105215	PREDICTED: probable glucan 1 3-alpha-glucosidase [Vitis vinifera]
955	949	gi|225457152	51.51	4	2	1		54621	PREDICTED: betaine aldehyde dehydrogenase 2 mitochondrial [Vitis vinifera]
855	61304	gi|566158623	51.49	3	1	1		31302	hypothetical protein POPTR_0002s17990g [Populus trichocarpa]
1302	18566	gi|359473657	51.18	2	1	1		65748	PREDICTED: delta(24)-sterol reductase [Vitis vinifera]
1325	61060	gi|566192679	51.1	27	2	1		6086	hypothetical protein POPTR_0010s25170g [Populus trichocarpa]
1220	19350	gi|224073782	51.04	9	1	1	Carbamidomethylation	21868	proline-rich family protein [Populus trichocarpa]
1218	18611	gi|224077508	50.85	6	2	2		39890	putative protein phosphatase [Populus trichocarpa]
825	1327	gi|731440653	50.68	6	1	1		23090	PREDICTED: ras-related protein RABB1c [Vitis vinifera]
914	18864	gi|731383454	50.63	4	1	1		43734	PREDICTED: probable N-acetyl-gamma-glutamyl-phosphate reductase chloroplastic [Vitis vinifera]
1468	61079	gi|224084676	50.19	3	1	1		31324	hypothetical protein POPTR_0005s17010g [Populus trichocarpa]
1300	19030	gi|566167266	50.05	4	1	1		37830	hypothetical protein POPTR_0004s18610g [Populus trichocarpa]
443	43809	gi|566195980	49.74	5	1	1		17497	Photosystem I reaction center subunit V family protein [Populus trichocarpa]
1195	17905	gi|224105715	49.72	4	2	1	Carbamidomethylation	69881	vacuolar sorting receptor protein BP-80 [Populus trichocarpa]

1508	17431	gi|566176019	49.63	4	1	1		39252	isocitrate dehydrogenase family protein [Populus trichocarpa]
1012	17447	gi|566185959	49.57	6	2	2		27367	Proteasome subunit alpha type 3 family protein [Populus trichocarpa]
1157	18775	gi|566181200	49.5	2	1	1		55243	serine carboxypeptidase S28 family protein [Populus trichocarpa]
1139	1090	gi|225465997	49.43	3	1	1		48382	PREDICTED: WD-40 repeat-containing protein MSI1 [Vitis vinifera]
905	17608	gi|224131618	49.23	7	2	2		26206	dienelactone hydrolase family protein [Populus trichocarpa]
1088	2359	gi|225460861	49.14	9	1	1		12136	PREDICTED: mitochondrial pyruvate carrier 2 isoform X2 [Vitis vinifera]
1409	17396	gi|224119992	49.12	2	2	2	Carbamidomethylation	143158	hypothetical protein POPTR_0012s13100g [Populus trichocarpa]
1377	18307	gi|224113315	48.77	2	1	1		66874	hypothetical protein POPTR_0010s25600g [Populus trichocarpa]
960	19955	gi|225445842	48.6	9	2	2		21531	PREDICTED: uncharacterized protein LOC100243229 [Vitis vinifera]
1221	19216	gi|566170063	48.56	4	1	1		32177	hypothetical protein POPTR_0005s07750g [Populus trichocarpa]
1465	61167	gi|566187811	48.5	5	1	1	Carbamidomethylation	16218	hypothetical protein POPTR_0009s13460g [Populus trichocarpa]
1383	1763	gi|731440118	48.5	1	1	1		144044	PREDICTED: kinesin-like protein KCA2 [Vitis vinifera]
723	19229	gi|566165862	48.26	2	1	1		41915	hypothetical protein POPTR_0004s10130g [Populus trichocarpa]
839	1695	gi|731408423	48.15	4	2	2	Carbamidomethylation	43098	PREDICTED: serine carboxypeptidase-like 18 isoform X4 [Vitis vinifera]

895	1486	gi|225465204	47.94	1	1	1	Carbamidomethylation	122695	PREDICTED: leucine--tRNA ligase cytoplasmic [Vitis vinifera]
947	5615	gi|225470242	47.77	6	1	1		16680	PREDICTED: uncharacterized protein At5g48480 [Vitis vinifera]
1190	17769	gi|566192876	47.53	5	2	1		47035	hypothetical protein POPTR_0010s26170g [Populus trichocarpa]
686	1660	gi|225452452	47.45	2	1	1		36545	PREDICTED: carbonic anhydrase 2 isoform X1 [Vitis vinifera]
1329	19947	gi|224084924	47.41	4	1	1	Carbamidomethylation	22828	Proteasome subunit beta type 3-2 family protein [Populus trichocarpa]
925	19953	gi|566205586	47.29	4	1	1		33515	hypothetical protein POPTR_0015s03360g [Populus trichocarpa]
307	19403	gi|566166342	47.27	1	1	1		95030	hypothetical protein POPTR_0004s13420g partial [Populus trichocarpa]
669	1373	gi|225465210	47.25	10	1	1		11974	PREDICTED: cytochrome c [Vitis vinifera]
970	901	gi|359493854	47.1	6	1	1		20058	PREDICTED: thioredoxin M4 chloroplastic [Vitis vinifera]
1144	18454	gi|224124728	47.03	7	2	1	Oxidation (M)	31993	peroxidase family protein [Populus trichocarpa]
1224	18074	gi|566161702	46.97	6	2	1		30568	Mitochondrial carnitine/acylcarnitine carrier-like family protein [Populus trichocarpa]
1077	2168	gi|359474991	46.7	3	1	1		37335	PREDICTED: heterodimeric geranylgeranyl pyrophosphate synthase small subunit chloroplastic [Vitis vinifera]
587	19810	gi|566163928	46.61	6	1	1		21587	disease resistance-responsive family protein [Populus trichocarpa]

962	2180	gi|225428049	46.51	3	1	1		42284	PREDICTED: glycerate dehydrogenase HPR peroxisomal [Vitis vinifera]
1097	18891	gi|566152365	46.44	4	2	2		43020	Vacuolar ATP synthase subunit C family protein [Populus trichocarpa]
1458	19298	gi|224122632	46.43	4	2	1		66196	Monocopper oxidase-like protein SKS1 precursor [Populus trichocarpa]
971	18088	gi|566179487	46.39	3	1	1		41063	hypothetical protein POPTR_0007s03900g [Populus trichocarpa]
1073	18054	gi|224068558	46.06	4	1	1		32725	NUCLEAR ENCODED CLP PROTEASE 1 family protein [Populus trichocarpa]
638	61222	gi|224059600	45.95	2	1	1		40957	hypothetical protein POPTR_0001s26960g [Populus trichocarpa]
1807	2203	gi|731387671	45.62	4	1	1	Carbamidomethylation	39674	PREDICTED: thioredoxin reductase NTRB-like [Vitis vinifera]
1100	18595	gi|566190935	45.55	3	2	1		61141	hypothetical protein POPTR_0010s15200g [Populus trichocarpa]
824	20173	gi|566181914	45.53	5	2	1		34297	hypothetical protein POPTR_0008s02330g [Populus trichocarpa]
1328	18988	gi|224072767	45.41	4	1	1		29070	hypothetical protein POPTR_0003s18420g [Populus trichocarpa]
1102	2038	gi|526117828	45.39	6	1	1		16599	serine/threonine kinase-like [Vitis vinifera]
1226	28969	gi|225468850	45.27	4	1	1		30830	PREDICTED: glyoxylate/succinic semialdehyde reductase 1 [Vitis vinifera]
441	43802	gi|224086681	45.06	2	1	1		50649	RNA recognition motif-containing family protein [Populus trichocarpa]

1075	17434	gi|566179312	44.87	2	2	2		92290	hypothetical protein POPTR_0007s02960g [Populus trichocarpa]
1297	17889	gi|566149128	44.79	7	2	2	Carbamidomethylation	47077	hypothetical protein POPTR_0001s15330g [Populus trichocarpa]
461	18903	gi|566146533	44.77	14	2	2		10147	acyl-CoA-binding family protein [Populus trichocarpa]
1510	2512	gi|359491781	44.71	6	1	1		18046	PREDICTED: miraculin-like partial [Vitis vinifera]
1198	1139	gi|359484892	44.58	3	2	2		86995	PREDICTED: eukaryotic translation initiation factor isoform 4G-1 [Vitis vinifera]
1330	29812	gi|224136252	44.42	19	1	1	Carbamidomethylation	8798	hypothetical protein POPTR_0015s11410g [Populus trichocarpa]
800	17900	gi|224104697	44.18	9	2	2		20848	lactoylglutathione lyase family protein [Populus trichocarpa]
1860	2400	gi|225434808	44.13	3	1	1		32777	PREDICTED: eukaryotic translation initiation factor 3 subunit G-B [Vitis vinifera]
376	2609	gi|359480551	44.09	3	1	1		29290	PREDICTED: mitochondrial outer membrane protein porin 2 [Vitis vinifera]
816	2775	gi|731436245	43.99	3	2	1		66120	PREDICTED: putative laccase-9 [Vitis vinifera]
1427	18658	gi|224117530	43.88	6	1	1		21810	endoribonuclease L-PSP family protein [Populus trichocarpa]
664	2476	gi|225440171	43.85	4	1	1		26618	PREDICTED: cysteine proteinase inhibitor 12-like [Vitis vinifera]
1078	19480	gi|224112341	43.84	3	1	1		46306	latex abundant family protein [Populus trichocarpa]
1016	18121	gi|566172301	43.63	5	2	1	Carbamidomethylation	54904	disulfide-isomerase family protein [Populus trichocarpa]
1013	2451	gi|225439775	43.58	4	1	1		27055	PREDICTED: glutathione S-transferase L3-like [Vitis vinifera]
1459	1965	gi|225459318	43.53	4	2	1	Carbamidomethylation	35188	PREDICTED: annexin D1 [Vitis vinifera]

777	19863	gi|566212628	43.46	7	1	1		17449	hypothetical protein POPTR_0017s10800g [Populus trichocarpa]
1811	412	gi|225443760	43.45	8	1	1		20875	PREDICTED: 60S ribosomal protein L11-1-like [Vitis vinifera]
1331	921	gi|731382079	43.3	1	1	1		110376	PREDICTED: neutral alpha-glucosidase C-like isoform X2 [Vitis vinifera]
1601	1597	gi|526118004	43.23	2	1	1		47748	probable E3 ubiquitin-protein ligase HERC1-like [Vitis vinifera]
1229	30041	gi|224066755	43.12	5	1	1		19335	thioredoxin m family protein [Populus trichocarpa]
1104	30070	gi|225443988	43.03	7	1	1		13228	PREDICTED: photosystem I reaction center subunit psaK chloroplastic [Vitis vinifera]
1151	17661	gi|566237896	43.02	3	2	2		58183	hypothetical protein POPTR_0019s09060g [Populus trichocarpa]
608	19106	gi|224078826	42.94	4	1	1		24905	Oxygen-evolving enhancer protein 3-1 [Populus trichocarpa]
1484	1093	gi|225438067	42.74	2	1	1		45402	PREDICTED: succinyl-CoA ligase [ADP-forming] subunit beta mitochondrial [Vitis vinifera]
1079	29179	gi|566195867	42.73	3	1	1		52643	hypothetical protein POPTR_0011s16630g [Populus trichocarpa]
706	2504	gi|731416298	42.67	4	1	1		25123	PREDICTED: eukaryotic translation initiation factor 3 subunit J [Vitis vinifera]
1106	19544	gi|566164408	42.57	2	1	1		45443	RNA-binding protein 45 [Populus trichocarpa]
1045	19921	gi|566210424	42.51	3	1	1		35704	hypothetical protein POPTR_0017s00350g [Populus trichocarpa]

1467	17767	gi|566157737	42.45	1	1	1		79950	hypothetical protein POPTR_0002s13010g [Populus trichocarpa]
772	30077	gi|566214618	42.18	7	1	1	Carbamidomethylation	13633	hypothetical protein POPTR_0018s06830g [Populus trichocarpa]
1804	17777	gi|566153407	42.1	3	1	1		42007	ferredoxin-NADP reductase family protein [Populus trichocarpa]
1105	2524	gi|225452110	42.09	10	1	1	Carbamidomethylation	9858	PREDICTED: non-specific lipid-transfer protein 2-like [Vitis vinifera]
1434	61182	gi|224085575	42.08	5	1	1		28237	NADH-ubiquinone oxidoreductase 24 kDa subunit family protein [Populus trichocarpa]
1146	17909	gi|224133898	42.02	2	1	1	Carbamidomethylation	53810	aspartyl aminopeptidase family protein [Populus trichocarpa]
1211	19737	gi|224070843	41.98	8	1	1		19159	glutaredoxin family protein [Populus trichocarpa]
1147	2026	gi|359492937	41.98	2	1	1		46528	PREDICTED: aspartic proteinase nepenthesin-1 [Vitis vinifera]
1123	1750	gi|225447488	41.93	2	1	1		63568	PREDICTED: uncharacterized protein LOC100266500 [Vitis vinifera]
1164	18678	gi|566210985	41.93	3	1	1		51941	glycosyl hydrolase family 17 family protein [Populus trichocarpa]
917	19566	gi|566199275	41.83	4	1	1		38655	hypothetical protein POPTR_0013s03740g [Populus trichocarpa]
1250	723	gi|731370529	41.56	2	1	1		66006	PREDICTED: serine/threonine-protein phosphatase 2A 65 kDa regulatory subunit A beta isoform-like [Vitis vinifera]
1512	2259	gi|225431922	41.42	13	1	1		13225	PREDICTED: DNA-directed RNA polymerases II IV and V subunit 9A [Vitis vinifera]

1511	2099	gi|225426826	41.29	4	1	1		36763	PREDICTED: replication factor C subunit 2 [Vitis vinifera]
1485	19087	gi|566258212	41.24	7	1	1		24279	hypothetical protein POPTR_0115s00200g partial [Populus trichocarpa]
416	1128	gi|731418015	41.09	2	1	1		53972	PREDICTED: tryptophan synthase beta chain 1 [Vitis vinifera]
682	1352	gi|225428908	41.04	1	1	1		53765	PREDICTED: limonoid UDP-glucosyltransferase [Vitis vinifera]
1212	341	gi|731391907	41.04	2	1	1		67493	PREDICTED: anthranilate synthase alpha subunit 2 chloroplastic isoform X2 [Vitis vinifera]
1808	61284	gi|224144643	40.83	4	1	1		40333	hypothetical protein POPTR_0019s04130g [Populus trichocarpa]
1463	758	gi|225430549	40.72	1	1	1		82761	PREDICTED: eukaryotic translation initiation factor 3 subunit B [Vitis vinifera]
568	43788	gi|552546256	40.65	21	1	1		4424	cytochrome b559 beta subunit (chloroplast) [Camellia pitardii]
1756	1933	gi|225455762	40.5	2	1	1		67078	PREDICTED: probable phenylalanine--tRNA ligase beta subunit [Vitis vinifera]
1397	1173	gi|731393107	40.37	1	1	1		108073	PREDICTED: DNA (cytosine-5)-methyltransferase CMT3 [Vitis vinifera]
994	19883	gi|566189043	40.24	4	1	1	Carbamidomethylation	36173	Peroxidase 6 precursor family protein [Populus trichocarpa]
1071	600	gi|359490767	40.08	5	1	1		23070	PREDICTED: nascent polypeptide-associated complex subunit alpha-like protein 2 [Vitis vinifera]
744	2032	gi|359474652	40.07	2	1	1		50670	PREDICTED: scopoletin glucosyltransferase-like [Vitis vinifera]

1327	17711	gi|224122948	40	2	2	2		79226	inorganic pyrophosphatase family protein [Populus trichocarpa]
1334	18960	gi|566255736	39.92	5	1	1		15873	hypothetical protein POPTR_0483s00200g partial [Populus trichocarpa]
1332	20744	gi|566189812	39.68	2	1	1	Carbamidomethylation	50971	N-carbamyl-L-amino acid hydrolase family protein [Populus trichocarpa]
1301	61101	gi|566151634	39.59	2	1	1	Carbamidomethylation	50974	hypothetical protein POPTR_0001s29250g [Populus trichocarpa]
1333	61311	gi|566256321	39.54	7	1	1		12448	glycine-rich RNA-binding family protein [Populus trichocarpa]
1805	1371	gi|225469850	38.85	2	1	1		39160	PREDICTED: uncharacterized protein DDB_G0288133 [Vitis vinifera]
1179	17054	gi|566183505	38.79	3	1	1	Carbamidomethylation	65372	phosphatase family protein [Populus trichocarpa]
1425	793	gi|225428898	38.59	3	2	2		62556	PREDICTED: D-3-phosphoglycerate dehydrogenase 1 chloroplastic [Vitis vinifera]
1410	362	gi|225464186	38.53	3	1	1	Carbamidomethylation	49585	PREDICTED: 26S proteasome regulatory subunit 4 homolog B [Vitis vinifera]
1309	2613	gi|731380876	38.36	6	2	1		30353	PREDICTED: mitochondrial carnitine/acylcarnitine carrier-like protein [Vitis vinifera]
1207	1616	gi|225426330	38.34	5	1	1		34913	PREDICTED: 26S proteasome non-ATPase regulatory subunit 7 homolog A [Vitis vinifera]
1311	29791	gi|224140057	38.31	3	1	1		29642	alcohol dehydroge family protein [Populus trichocarpa]
1290	18418	gi|566234529	38.21	12	1	1		13103	hypothetical protein POPTR_0019s07730g [Populus trichocarpa]

167	61202	gi|566152126	38.02	2	1	1		51807	hypothetical protein POPTR_0001s31980g [Populus trichocarpa]
1009	19977	gi|225456262	37.9	9	1	1		11952	PREDICTED: mitochondrial pyruvate carrier 1 [Vitis vinifera]
1861	19941	gi|566186111	37.74	8	1	1		12191	hypothetical protein POPTR_0009s03520g partial [Populus trichocarpa]
1757	261	gi|225458173	37.66	4	1	1		44535	PREDICTED: 26S protease regulatory subunit S10B homolog B [Vitis vinifera]
934	29335	gi|225466271	37.4	5	1	1		21853	PREDICTED: ras-related protein Rab5 [Vitis vinifera]
1176	21187	gi|225452785	37.39	4	1	1		29509	PREDICTED: NAP1-related protein 2 [Vitis vinifera]
963	2148	gi|225450543	37.38	5	1	1		18729	PREDICTED: uncharacterized protein LOC100245914 [Vitis vinifera]
1286	17395	gi|224074998	37.18	2	2	2		100185	hypothetical protein POPTR_0003s13040g [Populus trichocarpa]
1230	2245	gi|731381456	37.13	3	1	1		34629	PREDICTED: translocase of chloroplast 34 chloroplastic isoform X2 [Vitis vinifera]
1563	18220	gi|566152765	37.06	3	1	1		49287	hypothetical protein POPTR_0001s34620g [Populus trichocarpa]
1032	1444	gi|731435761	37.03	1	1	1		160776	PREDICTED: putative disease resistance RPP13-like protein 1 isoform X1 [Vitis vinifera]
505	2666	gi|225449032	37.01	2	1	1		52590	PREDICTED: dolichyl-diphosphooligosaccharide--protein glycosyltransferase subunit 1B [Vitis vinifera]
1206	20108	gi|566158274	36.98	3	1	1		34050	hypothetical protein POPTR_0002s15960g [Populus trichocarpa]

1083	44615	gi|566258388	36.3	1	2	1		194544	hypothetical protein POPTR_0101s00210g [Populus trichocarpa]
1809	17815	gi|566187112	36.26	2	1	1		38128	WD-40 repeat family protein [Populus trichocarpa]
1513	71220	gi|566172749	36.26	2	1	1		38756	GDSL-motif lipase/hydrolase family protein [Populus trichocarpa]
1486	61313	gi|566148380	36.18	4	1	1		23562	hypothetical protein POPTR_0001s11260g partial [Populus trichocarpa]
1095	18691	gi|671743260	36.1	4	1	1		24165	cytochrome b6 (chloroplast) [Camellia petelotii]
1056	622	gi|731385628	36.07	1	1	1		99551	PREDICTED: heat shock 70 kDa protein 17 [Vitis vinifera]
1288	18332	gi|224107655	35.91	3	1	1		33896	3-oxoacyl-[acyl-carrier-protein] reductase [Populus trichocarpa]
1231	19835	gi|224066547	35.88	6	1	1		19367	photosystem 2 family protein [Populus trichocarpa]
1759	29214	gi|224145927	35.84	2	1	1	Carbamidomethylation	61923	4-coumarate-CoA ligase family protein [Populus trichocarpa]
1862	18956	gi|566162026	35.71	6	1	1	Carbamidomethylation	27183	hypothetical protein POPTR_0003s10720g [Populus trichocarpa]
1688	2594	gi|731382912	35.67	1	1	1		81725	PREDICTED: subtilisin-like protease [Vitis vinifera]
1810	19721	gi|566206526	35.66	6	1	1		23501	hypothetical protein POPTR_0015s07940g [Populus trichocarpa]
1108	61448	gi|225453275	35.39	4	1	1	Carbamidomethylation	14633	PREDICTED: 20 kDa chaperonin chloroplastic [Vitis vinifera]
1000	737	gi|225424719	35.37	1	1	1		78467	PREDICTED: threonine--tRNA ligase mitochondrial [Vitis vinifera]
1335	61183	gi|731438465	35.1	3	1	1		26979	PREDICTED: 3-isopropylmalate dehydratase small subunit 3 [Vitis vinifera]

886	43827	gi|731410026	35.04	3	1	1		33957	PREDICTED: protein THYLAKOID FORMATION1 chloroplastic isoform X2 [Vitis vinifera]
1863	681	gi|731422501	34.74	1	1	1		125527	PREDICTED: protein TOPLESS isoform X1 [Vitis vinifera]
1638	1104	gi|731435142	34.52	1	1	1		84036	PREDICTED: protein ROOT HAIR DEFECTIVE 3 homolog 2 isoform X2 [Vitis vinifera]
1384	1214	gi|731426684	34.49	1	1	1		144086	PREDICTED: uncharacterized protein LOC100262718 isoform X4 [Vitis vinifera]
1266	71076	gi|224059096	34.37	2	2	1		84678	GLABRA 2 family protein [Populus trichocarpa]
592	1621	gi|225431409	34.32	2	1	1		39072	PREDICTED: probable mannitol dehydrogenase [Vitis vinifera]
1111	47263	gi|225453793	34.29	1	1	1		328472	PREDICTED: uncharacterized protein LOC100247154 [Vitis vinifera]
1222	2913	gi|359476206	34.17	2	1	1		50104	PREDICTED: protein ASPARTIC PROTEASE IN GUARD CELL 2-like isoform X2 [Vitis vinifera]
1336	1321	gi|225424750	33.97	7	1	1	Carbamidomethylation	11841	PREDICTED: gibberellin-regulated protein 11 [Vitis vinifera]
1432	1377	gi|225452877	33.92	3	1	1		57074	PREDICTED: cystathionine gamma-synthase chloroplastic [Vitis vinifera]
926	463	gi|731398611	33.9	2	1	1		43372	PREDICTED: proliferation-associated protein 2G4 [Vitis vinifera]
1312	61282	gi|359484939	33.62	1	1	1	Carbamidomethylation	109209	PREDICTED: L-arabinokinase-like isoform X1 [Vitis vinifera]
1145	2039	gi|225449198	33.59	4	1	1		31414	PREDICTED: eukaryotic translation initiation factor 3 subunit F [Vitis vinifera]

1442	25197	gi|566215197	33.42	2	1	1		96019	hypothetical protein POPTR_0018s10680g [Populus trichocarpa]
795	18582	gi|566156002	33.27	2	1	1		37272	hypothetical protein POPTR_0002s03260g [Populus trichocarpa]
1180	2753	gi|225429582	33.2	1	1	1		98913	PREDICTED: aminopeptidase M1 [Vitis vinifera]
581	6004	gi|731413856	33.17	1	2	2		155634	PREDICTED: TMV resistance protein N-like [Vitis vinifera]
1864	71225	gi|359487009	33.14	1	1	1		104506	PREDICTED: serine/threonine-protein phosphatase BSL3-like isoform X1 [Vitis vinifera]
1299	19552	gi|731395258	32.93	6	1	1		14682	PREDICTED: U6 snRNA-associated Sm-like protein LSm2 [Vitis vinifera]
1337	28945	gi|225445462	32.83	7	1	1		14555	PREDICTED: uncharacterized protein At2g34160 [Vitis vinifera]
1514	30061	gi|566197758	32.67	10	1	1		11735	hypothetical protein POPTR_0012s10210g partial [Populus trichocarpa]
1213	19793	gi|566181894	32.49	2	1	1		58001	hypothetical protein POPTR_0008s02250g [Populus trichocarpa]
1515	3083	gi|225443150	32.22	11	1	1		9286	PREDICTED: uncharacterized protein LOC100262209 [Vitis vinifera]
1866	1567	gi|225433215	32.05	6	1	1	Carbamidomethylation	26277	PREDICTED: DAG protein chloroplastic [Vitis vinifera]
1867	20184	gi|566187829	31.44	1	1	1		53103	hypothetical protein POPTR_0009s13560g [Populus trichocarpa]
944	18241	gi|566146307	31.35	3	2	1		66102	monocopper oxidase precursor family protein [Populus trichocarpa]
989	21244	gi|566177163	31.24	2	1	1		82625	UBIQUITIN-SPECIFIC PROTEASE 23 family protein [Populus trichocarpa]

1241	1860	gi|359474339	31.11	3	1	1		45436	PREDICTED: probable transaldolase isoform X1 [Vitis vinifera]
1001	2681	gi|225455153	30.66	4	1	1		26938	PREDICTED: mitochondrial import inner membrane translocase subunit TIM22-like [Vitis vinifera]
1315	18000	gi|526118245	30.54	3	1	1	Carbamidomethylation	23461	peroxiredoxin Q [Vitis vinifera]
1107	71241	gi|731386048	30.3	3	1	1		26122	PREDICTED: calcium uptake protein 3 mitochondrial-like [Vitis vinifera]
1080	71174	gi|566156540	29.96	1	1	1		114460	hypothetical protein POPTR_0002s06380g partial [Populus trichocarpa]
964	17552	gi|566205234	29.72	5	1	1		24533	hypothetical protein POPTR_0015s00570g [Populus trichocarpa]
1277	5139	gi|731404419	29.57	3	1	1	Carbamidomethylation	54013	PREDICTED: monothiol glutaredoxin-S17 isoform X1 [Vitis vinifera]
1072	1404	gi|225437172	29.53	3	1	1		38410	PREDICTED: 2-methyl-6-phytyl-1 4-hydroquinone methyltransferase chloroplastic [Vitis vinifera]
1251	17479	gi|224097148	28.95	2	1	1		41439	GDP-mannose 4 family protein [Populus trichocarpa]
1517	19012	gi|566155909	28.62	3	1	1		57213	hypothetical protein POPTR_0002s02740g [Populus trichocarpa]
1209	17486	gi|224067419	28.51	1	1	1		69742	malate oxidoreductase family protein [Populus trichocarpa]
1516	71248	gi|225456818	28.39	4	1	1		21329	PREDICTED: peptide methionine sulfoxide reductase [Vitis vinifera]
1165	20117	gi|224129342	28.25	4	1	1		22803	Photosystem I reaction center subunit XI family protein [Populus trichocarpa]
1443	24392	gi|526118271	28.2	7	1	1		12001	peptidyl-prolyl isomerase FKBP12-like [Vitis vinifera]

1037	19762	gi|566161718	28.18	3	1	1		43721	UBIQUITIN-SPECIFIC PROTEASE 20 family protein [Populus trichocarpa]
1868	48485	gi|224107257	27.91	1	1	1		93079	hypothetical protein POPTR_0010s03030g [Populus trichocarpa]
1662	18462	gi|566192083	27.6	1	1	1	Carbamidomethylation	111427	transport protein Sec24 [Populus trichocarpa]
1806	71161	gi|566149517	27.54	1	1	1		69963	hypothetical protein POPTR_0001s17110g [Populus trichocarpa]
1338	17506	gi|566243399	27.45	3	1	1		33846	hypothetical protein POPTR_0019s13040g [Populus trichocarpa]
820	71291	gi|566182405	27.44	3	1	1		23297	hypothetical protein POPTR_0008s04920g [Populus trichocarpa]
1518	71296	gi|566206075	27.39	1	1	1		59345	hypothetical protein POPTR_0015s05820g [Populus trichocarpa]
1869	71275	gi|566155466	27.27	2	1	1		44474	hypothetical protein POPTR_0002s00230g [Populus trichocarpa]
1047	20307	gi|566167359	27.27	4	1	1		15952	hypothetical protein POPTR_0004s19310g [Populus trichocarpa]
1046	5258	gi|359489366	26.76	2	1	1		42909	PREDICTED: fasciclin-like arabinogalactan protein 10 [Vitis vinifera]
1689	1929	gi|731421066	26.75	2	1	1		33932	PREDICTED: probable fructokinase-7 isoform X2 [Vitis vinifera]
927	18810	gi|566209344	26.74	3	1	1		27361	In2-1 family protein [Populus trichocarpa]
1270	30727	gi|359473558	26.69	3	1	1		39906	PREDICTED: uncharacterized protein LOC100853782 [Vitis vinifera]
1429	2813	gi|225427973	26.49	3	1	1		19853	PREDICTED: glycine-rich protein 2-like [Vitis vinifera]

1870	2631	gi|225450051	26.48	3	1	1		39579	PREDICTED: cycloartenol-C-24-methyltransferase [Vitis vinifera]
1121	46515	gi|566155205	26.19	1	1	1		59386	hypothetical protein POPTR_0001s46360g [Populus trichocarpa]
576	3274	gi|526117984	26.18	3	1	1		21537	uncharacterized protein LOC100265927 [Vitis vinifera]
1438	6691	gi|225437318	26.17	1	1	1		157286	PREDICTED: translocase of chloroplast 159 chloroplastic [Vitis vinifera]
1167	20075	gi|224092174	25.94	4	1	1		36141	hypothetical protein POPTR_0006s24360g [Populus trichocarpa]
1487	61392	gi|225464932	25.45	7	1	1		12133	PREDICTED: uncharacterized protein At2g27730 mitochondrial [Vitis vinifera]
1289	4188	gi|731375647	25.44	0	1	1		320504	PREDICTED: LOW QUALITY PROTEIN: uncharacterized protein LOC100265010 [Vitis vinifera]
1340	61530	gi|566256423	25.44	2	1	1		40507	hypothetical protein POPTR_0306s00210g [Populus trichocarpa]
1813	35612	gi|731418622	25.43	3	1	1	Carbamidomethylation	36326	PREDICTED: peroxidase 55-like [Vitis vinifera]
1814	71263	gi|225446625	25.38	1	1	1		57567	PREDICTED: uncharacterized protein LOC100267778 [Vitis vinifera]
1305	17749	gi|566170400	25.28	4	1	1		42882	hypothetical protein POPTR_0005s09680g [Populus trichocarpa]
1317	61434	gi|566168420	24.69	3	1	1		41147	hypothetical protein POPTR_0004s24220g [Populus trichocarpa]
1519	71338	gi|224136978	24.37	6	1	1		20524	ADP-ribosylation factor family protein [Populus trichocarpa]
1074	25166	gi|566207323	24.26	1	1	1		61858	hypothetical protein POPTR_0015s12920g [Populus trichocarpa]

334	71256	gi|224141723	24.22	1	1	1		107028	leucine-rich repeat transmembrane protein kinase [Populus trichocarpa]
1448	33635	gi|225441979	24.09	1	1	1		63296	PREDICTED: pectinesterase 3 [Vitis vinifera]
870	1307	gi|225463506	24.01	2	1	1		38294	PREDICTED: fructose-bisphosphate aldolase cytoplasmic isozyme 1 [Vitis vinifera]
1871	29683	gi|566197448	23.93	9	1	1	Carbamidomethylation	12293	hypothetical protein POPTR_0012s08450g [Populus trichocarpa]
1168	1670	gi|225436339	23.25	2	1	1		45109	PREDICTED: arogenate dehydratase 3 chloroplastic [Vitis vinifera]
1764	17863	gi|566211408	23.2	2	1	1		37992	hypothetical protein POPTR_0017s04750g [Populus trichocarpa]
1489	30408	gi|359474260	23.15	1	1	1		62968	PREDICTED: protein HOTHEAD [Vitis vinifera]
1019	71324	gi|731427792	23.11	1	1	1		64758	PREDICTED: serine/threonine-protein kinase TNNI3K-like isoform X2 [Vitis vinifera]
1002	71427	gi|225463990	22.61	3	1	1		29642	PREDICTED: transcription repressor MYB6 [Vitis vinifera]
1874	71387	gi|566257303	22.51	1	1	1		78394	hypothetical protein POPTR_0163s00240g [Populus trichocarpa]
1169	23587	gi|731388704	22.37	1	1	1		112307	PREDICTED: uncharacterized protein LOC104879229 isoform X1 [Vitis vinifera]
810	996	gi|526117499	22.32	2	1	1		44546	glutamate dehydrogenase [Vitis vinifera]
1292	71337	gi|566172843	22.28	0	1	1		168481	hypothetical protein POPTR_0005s23270g [Populus trichocarpa]
1567	71178	gi|552541020	22.16	0	1	1		269112	Ycf2 (chloroplast) [Camellia yunnanensis]

1185	44567	gi|731440108	21.99	1	1	1		128768	PREDICTED: uncharacterized protein At4g38062-like partial [Vitis vinifera]
1435	71177	gi|566177799	21.92	1	1	1		146475	hypothetical protein POPTR_0006s23660g [Populus trichocarpa]
1189	71348	gi|731437744	21.9	1	1	1		95541	PREDICTED: probable LRR receptor-like serine/threonine-protein kinase At1g12460 [Vitis vinifera]
953	3188	gi|225462357	21.73	3	1	1		25816	PREDICTED: probable glutathione S-transferase [Vitis vinifera]
1062	1772	gi|225451677	21.7	1	1	1		108901	PREDICTED: staphylococcal nuclease domain-containing protein 1 [Vitis vinifera]
1641	71199	gi|566165455	21.65	1	1	1		121979	hypothetical protein POPTR_0004s07590g [Populus trichocarpa]
822	20285	gi|566209825	21.58	1	1	1		60999	hypothetical protein POPTR_0016s11950g [Populus trichocarpa]
1109	17994	gi|566158382	21.56	1	1	1		77765	hypothetical protein POPTR_0002s16570g [Populus trichocarpa]
1724	48211	gi|224115854	21.49	1	1	1		110333	hypothetical protein POPTR_0011s01420g [Populus trichocarpa]
1602	1733	gi|225429462	21.4	1	1	1		71749	PREDICTED: far upstream element-binding protein 2 [Vitis vinifera]
1817	24599	gi|566202327	21.16	3	1	1		58845	hypothetical protein POPTR_0014s03820g [Populus trichocarpa]
1291	62031	gi|566168097	21.07	1	1	1		132788	hypothetical protein POPTR_0004s22740g [Populus trichocarpa]
1621	17724	gi|566176622	21.07	1	1	1		56966	asparaginyl-tRNA synthetase family protein [Populus trichocarpa]

1343	1898	gi|225449068	21.03	2	1	1		47175	PREDICTED: chorismate synthase 1 chloroplastic [Vitis vinifera]
1879	71556	gi|225427782	20.79	1	1	1		77493	PREDICTED: arginine decarboxylase [Vitis vinifera]
1877	71487	gi|225443827	20.68	2	1	1		37115	PREDICTED: senescence-specific cysteine protease SAG39-like [Vitis vinifera]
1386	1387	gi|225427288	20.67	2	1	1		68795	PREDICTED: dynamin-related protein 1E [Vitis vinifera]
1437	71208	gi|359494168	20.63	1	1	1		165261	PREDICTED: ABC transporter C family member 3-like [Vitis vinifera]
1124	71379	gi|225452124	20.47	5	1	1	Carbamidomethylation	28914	PREDICTED: zinc finger CCCH domain-containing protein 15 [Vitis vinifera]

Table S3 The identified proteins in HY1

Protein Group	Protein ID	Accession	-10lgP	Coverage (%)	#Peptides	#Unique	PTM	Avg. Mass	Description
3	6	gi|359495606	312.86	54	48	3	Carbamidomethylation; Oxidation (M)	80006	PREDICTED: heat shock cognate protein 80 [Vitis vinifera]
7	7	gi|225462013	308.16	50	45	3	Carbamidomethylation	80849	PREDICTED: heat shock cognate protein 80-like [Vitis vinifera]
6	1	gi|225426385	301.85	75	34	1	Carbamidomethylation; Oxidation (M)	50292	PREDICTED: tubulin beta-2 chain [Vitis vinifera]
1	16386	gi|669100005	300.66	76	41	41	Carbamidomethylation; Oxidation (M)	52635	ribulose bisphosphate carboxylase large chain (chloroplast) [Camellia crapnelliana]
10	16400	gi|224090883	296.85	71	32	1	Carbamidomethylation; Oxidation (M)	49907	hypothetical protein POPTR_0006s09610g [Populus trichocarpa]
2	16404	gi|224055984	294.41	72	25	3	Carbamidomethylation	41696	actin family protein [Populus trichocarpa]
12	8	gi|225426164	294.17	41	35	2	Carbamidomethylation	80852	PREDICTED: heat shock protein 83 [Vitis vinifera]
22	16395	gi|566195847	292.67	61	30	1	Carbamidomethylation; Oxidation (M)	50164	tubulin beta chain family protein [Populus trichocarpa]
18	15	gi|225464589	292.66	42	35	1	Carbamidomethylation	80868	PREDICTED: heat shock protein 83-like [Vitis vinifera]
24	5	gi|359486799	288.83	57	39	2	Carbamidomethylation; Oxidation (M)	71171	PREDICTED: heat shock cognate 70 kDa protein 2 [Vitis vinifera]
28	10	gi|225434984	288.39	60	40	1	Carbamidomethylation; Oxidation (M)	71235	PREDICTED: heat shock cognate 70 kDa protein 2 [Vitis vinifera]

17	16417	gi|224056837	283.71	41	38	1	Carbamidomethylation	80026	hypothetical protein POPTR_0001s47020g [Populus trichocarpa]
42	16411	gi|224100971	280.96	57	36	1	Carbamidomethylation; Oxidation (M)	71117	heat shock protein 70 cognate [Populus trichocarpa]
43	18	gi|225452767	279.2	66	27	3	Carbamidomethylation	49622	PREDICTED: tubulin alpha chain [Vitis vinifera]
32	16	gi|225440324	278.2	48	36	1	Carbamidomethylation; Oxidation (M)	71066	PREDICTED: heat shock cognate 70 kDa protein 2 [Vitis vinifera]
33	16410	gi|566191989	276.59	57	38	1	Carbamidomethylation; Oxidation (M)	71140	heat shock protein 70 cognate [Populus trichocarpa]
27	23	gi|225456143	269.2	60	28	1	Carbamidomethylation	49779	PREDICTED: tubulin beta chain [Vitis vinifera]
29	17	gi|225434722	267	61	30	2	Carbamidomethylation	50275	PREDICTED: tubulin beta-2 chain [Vitis vinifera]
8	16442	gi|224056705	265.77	55	21	1	Carbamidomethylation	41636	actin family protein [Populus trichocarpa]
76	36	gi|225426230	255.68	39	33	1	Carbamidomethylation	73581	PREDICTED: luminal-binding protein 5 [Vitis vinifera]
14	16499	gi|224117708	255.49	55	18	1	Carbamidomethylation	40618	actin family protein [Populus trichocarpa]
62	16494	gi|566146990	246.59	29	22	1	Carbamidomethylation; Oxidation (M)	71882	heat shock protein 70 cognate [Populus trichocarpa]
52	16430	gi|566232573	243.67	33	26	1	Carbamidomethylation	85012	hypothetical protein POPTR_0019s05430g [Populus trichocarpa]
73	46	gi|225445820	241.65	30	21	1	Carbamidomethylation	71402	PREDICTED: heat shock 70 kDa protein [Vitis vinifera]

40	88	gi|225426414	239.23	43	21	1	Carbamidomethylation	50055	PREDICTED: tubulin beta chain [Vitis vinifera]
49	33	gi|225437708	238.75	62	19	1	Carbamidomethylation	42793	PREDICTED: S-adenosylmethionine synthase 5 [Vitis vinifera]
39	34	gi|225432155	237.75	66	21	1	Carbamidomethylation	43114	PREDICTED: S-adenosylmethionine synthase 2 [Vitis vinifera]
108	20	gi|225440045	236.11	39	28	2	Carbamidomethylation; Oxidation (M)	89589	PREDICTED: cell division cycle protein 48 homolog [Vitis vinifera]
35	31	gi|225439223	236.01	30	23	4	Carbamidomethylation; Oxidation (M)	84991	PREDICTED: 5-methyltetrahydropteroyltriglutamate--homocysteine methyltransferase [Vitis vinifera]
61	16508	gi|224144808	234.11	51	18	1	Carbamidomethylation; Oxidation (M)	49595	Tubulin alpha-1 chain family protein [Populus trichocarpa]
58	16455	gi|566188155	232.1	28	20	2	Carbamidomethylation	89855	hypothetical protein POPTR_0009s15490g [Populus trichocarpa]
80	16510	gi|566146992	231.87	28	17	2	Carbamidomethylation; Oxidation (M)	71904	heat shock protein 70 cognate [Populus trichocarpa]
70	26	gi|225456079	230.54	42	18	17	Carbamidomethylation	59180	PREDICTED: ATP synthase subunit beta mitochondrial-like [Vitis vinifera]
75	44	gi|359473642	228.52	28	20	5	Carbamidomethylation	90465	PREDICTED: heat shock protein 90-1 [Vitis vinifera]
63	58	gi|225449120	228.42	41	22	1	Carbamidomethylation	49376	PREDICTED: elongation factor 1-alpha [Vitis vinifera]

94	16496	gi|224085900	228.3	24	23	6		94047	hypothetical protein POPTR_0005s26260g [Populus trichocarpa]
54	64	gi|731416007	228.03	57	19	1	Carbamidomethylation	42931	PREDICTED: S-adenosylmethionine synthase 4 [Vitis vinifera]
155	16503	gi|224073124	227.18	39	21	3	Carbamidomethylation	64544	chaperonin precursor family protein [Populus trichocarpa]
68	160	gi|359491599	220.4	39	15	2	Carbamidomethylation	36735	PREDICTED: glyceraldehyde-3-phosphate dehydrogenase cytosolic [Vitis vinifera]
129	16476	gi|566212215	219.39	43	18	2	Carbamidomethylation	53198	adenosylhomocysteinase family protein [Populus trichocarpa]
131	78	gi|359478916	218.49	36	14	3	Carbamidomethylation	48674	PREDICTED: ribulose bisphosphate carboxylase/oxygenase activase chloroplastic isoform X2 [Vitis vinifera]
109	16511	gi|566196410	218.28	29	23	3		73406	BiP isoform A family protein [Populus trichocarpa]
103	16444	gi|224094244	218.19	27	22	2	Carbamidomethylation	93953	elongation factor 2 family protein [Populus trichocarpa]
72	16464	gi|566199292	216.87	48	16	1	Carbamidomethylation	42361	hypothetical protein POPTR_0013s03790g [Populus trichocarpa]
111	42	gi|731393262	216.79	38	24	4	Carbamidomethylation	64608	PREDICTED: ruBisCO large subunit-binding protein subunit beta chloroplastic [Vitis vinifera]

119	16518	gi|566204581	216.45	20	14	3	Carbamidomethylation	82947	hypothetical protein POPTR_0014s16280g [Populus trichocarpa]
57	16553	gi|224133862	215.48	44	18	1	Carbamidomethylation	36770	glyceraldehyde-3-phosphate dehydrogenase family protein [Populus trichocarpa]
142	95	gi|731379116	214.53	21	14	14	Carbamidomethylation	100128	PREDICTED: puromycin-sensitive aminopeptidase isoform X2 [Vitis vinifera]
93	16500	gi|224136806	213.36	38	18	3	Carbamidomethylation	47928	hypothetical protein POPTR_0015s14380g [Populus trichocarpa]
120	39	gi|225462164	211.82	24	23	3	Carbamidomethylation	93979	PREDICTED: elongation factor 2 [Vitis vinifera]
118	104	gi|225456806	210.16	39	17	1	Carbamidomethylation	53079	PREDICTED: adenosylhomocysteinase-like [Vitis vinifera]
77	252	gi|526117553	209.91	33	10	1	Carbamidomethylation	39322	glutamine synthetase cytosolic isozyme 2 [Vitis vinifera]
181	16490	gi|566191652	209.77	19	26	1	Carbamidomethylation; Oxidation (M)	193360	clathrin heavy chain family protein [Populus trichocarpa]
101	100	gi|225457939	209.43	22	21	4	Carbamidomethylation	93220	PREDICTED: endoplasmin homolog [Vitis vinifera]
138	16468	gi|542688129	208.94	51	19	18		53694	ATP synthase CF1 beta subunit (chloroplast) [Camellia taliensis]
121	17009	gi|224065861	208.84	52	11	4	Carbamidomethylation	18243	Peptidyl-prolyl cis-trans isomerase family protein [Populus trichocarpa]

91	67	gi|224365668	208.69	44	21	17	Carbamidomethylation	55143	ATPase subunit 1 (mitochondrion) [Vitis vinifera]
123	16572	gi|566204141	207.64	38	16	10	Carbamidomethylation	42935	hypothetical protein POPTR_0014s13660g [Populus trichocarpa]
151	125	gi|731401345	206.12	31	12	1	Carbamidomethylation	47976	PREDICTED: ribulose bisphosphate carboxylase/oxygenase activase chloroplastic isoform X2 [Vitis vinifera]
140	41	gi|225456004	204.81	27	19	4	Carbamidomethylation	75635	PREDICTED: stromal 70 kDa heat shock-related protein chloroplastic [Vitis vinifera]
92	110	gi|359490179	204.68	22	15	4	Carbamidomethylation	78856	PREDICTED: transketolase chloroplastic [Vitis vinifera]
114	205	gi|526117591	204.07	31	13	1	Carbamidomethylation	49581	chalcone synthase [Vitis vinifera]
66	102	gi|731393868	202.81	18	15	1	Carbamidomethylation	89385	PREDICTED: 5-methyltetrahydropteroyltriglutamate--homocysteine methyltransferase 2 [Vitis vinifera]
125	16497	gi|224088798	201.23	38	16	1	Carbamidomethylation	46974	translation initiation factor eIF-4A family protein [Populus trichocarpa]
165	16612	gi|566182577	201.21	28	12	3	Carbamidomethylation	48316	hypothetical protein POPTR_0008s05870g [Populus trichocarpa]

130	87	gi|731407424	199.82	19	16	1	Carbamidomethylation	91983	PREDICTED: uncharacterized protein LOC100244028 [Vitis vinifera]
106	16526	gi|224063766	199.22	19	13	2	Carbamidomethylation	80716	transketolase family protein [Populus trichocarpa]
116	159	gi|225462777	199.09	40	15	3	Carbamidomethylation	42597	PREDICTED: chalcone synthase 2 [Vitis vinifera]
81	94	gi|225464999	198.52	36	14	4		42422	PREDICTED: phosphoglycerate kinase cytosolic [Vitis vinifera]
162	116	gi|731410136	197.92	16	13	2	Carbamidomethylation	95223	PREDICTED: heat shock protein 83 isoform X3 [Vitis vinifera]
97	16607	gi|224109060	197.75	35	16	1	Carbamidomethylation	50212	PHOSPHOGLYCERATE KINASE 1 family protein [Populus trichocarpa]
115	138	gi|731423140	196.72	31	16	1	Carbamidomethylation	55855	PREDICTED: enolase [Vitis vinifera]
169	92	gi|359479362	195.9	29	16	3		61984	PREDICTED: ruBisCO large subunit-binding protein subunit alpha [Vitis vinifera]
166	166	gi|225433510	195.13	29	12	4	Carbamidomethylation	51906	PREDICTED: serine hydroxymethyltransferase 4 [Vitis vinifera]
214	61	gi|731426977	194.79	27	20	4	Carbamidomethylation	102347	PREDICTED: ATP-dependent Clp protease ATP-binding subunit clpA homolog CD4B chloroplastic [Vitis vinifera]

99	16524	gi|566176113	194.17	30	17	1	Carbamidomethylation	49308	hypothetical protein POPTR_0006s13310g [Populus trichocarpa]
117	16660	gi|566163210	192.88	32	13	1	Carbamidomethylation	42709	naregenin-chalcone synthase family protein [Populus trichocarpa]
96	145	gi|225464995	192.53	33	15	2	Carbamidomethylation	50083	PREDICTED: phosphoglycerate kinase chloroplastic [Vitis vinifera]
288	136	gi|225456274	192.07	30	14	4	Carbamidomethylation	55180	PREDICTED: argininosuccinate synthase chloroplastic [Vitis vinifera]
176	16528	gi|224104681	191.18	28	15	1		61991	RUBISCO SUBUNIT BINDING-protein ALPHA SUBUNIT [Populus trichocarpa]
191	72	gi|225456880	191.01	30	13	3	Carbamidomethylation	52692	PREDICTED: elongation factor TuB chloroplastic [Vitis vinifera]
381	16668	gi|566187902	188.37	32	11	1	Carbamidomethylation	54181	vacuolar ATP synthase subunit B family protein [Populus trichocarpa]
259	200	gi|225427768	188.37	40	10	4	Carbamidomethylation	42948	PREDICTED: fructose-bisphosphate aldolase 1 chloroplastic [Vitis vinifera]
102	16541	gi|566192207	187.79	30	17	1	Carbamidomethylation	49434	hypothetical protein POPTR_0010s22560g [Populus trichocarpa]
200	146	gi|731418890	187.33	30	12	1	Carbamidomethylation	52944	PREDICTED: elongation factor Tu chloroplastic-like [Vitis vinifera]

195	85	gi|225460328	186.65	22	16	1	Carbamidomethylation	74178	PREDICTED: stromal 70 kDa heat shock-related protein chloroplastic-like [Vitis vinifera]
221	16575	gi|224063263	186.65	30	15	1	Carbamidomethylation	58692	2-dehydro-3-deoxyphosphoheptonate aldolase family protein [Populus trichocarpa]
271	128	gi|225430776	186.33	28	13	13	Carbamidomethylation	73659	PREDICTED: succinate dehydrogenase [ubiquinone] flavoprotein subunit mitochondrial [Vitis vinifera]
105	16546	gi|224098012	186.17	28	15	1	Carbamidomethylation	49521	ADR12-2 family protein [Populus trichocarpa]
394	345	gi|731427869	184.98	30	11	1	Carbamidomethylation	54279	PREDICTED: V-type proton ATPase subunit B 2 isoform X2 [Vitis vinifera]
98	16520	gi|224109716	184.86	29	17	1	Carbamidomethylation	49434	hypothetical protein POPTR_0010s22620g [Populus trichocarpa]
154	66	gi|225460961	184.62	17	15	2	Carbamidomethylation	110056	PREDICTED: aconitate hydratase cytoplasmic [Vitis vinifera]
207	16657	gi|566162272	184.01	27	11	1	Carbamidomethylation	52666	hypothetical protein POPTR_0003s12160g [Populus trichocarpa]
266	16506	gi|224065685	183.38	35	15	1	Carbamidomethylation	56639	catalase family protein [Populus trichocarpa]
218	16609	gi|224053971	181.39	24	10	1	Carbamidomethylation	52743	hypothetical protein POPTR_0001s08770g [Populus trichocarpa]

209	16775	gi|566198898	180.99	15	12	1		73464	heat shock protein 70 cognate [Populus trichocarpa]
193	130	gi|731402352	180.65	21	15	1	Carbamidomethylation	65010	PREDICTED: ruBisCO large subunit-binding protein subunit beta chloroplastic [Vitis vinifera]
210	16939	gi|224109788	180.47	28	10	1	Carbamidomethylation	37547	putative adenosine kinase family protein [Populus trichocarpa]
260	16525	gi|224109966	179.32	28	14	13	Carbamidomethylation; Oxidation (M)	68701	Vacuolar ATP synthase catalytic subunit A family protein [Populus trichocarpa]
95	143	gi|731415862	178.15	34	16	6	Carbamidomethylation	49362	PREDICTED: calreticulin [Vitis vinifera]
184	16693	gi|566209405	177.54	26	12	1		44445	hypothetical protein POPTR_0016s08780g [Populus trichocarpa]
227	71	gi|225456550	177.18	45	13	2	Carbamidomethylation	40797	PREDICTED: UDP-arabinopyranose mutase 1 [Vitis vinifera]
203	16583	gi|224079726	176.97	30	13	5	Carbamidomethylation	46428	isocitrate dehydrogenase family protein [Populus trichocarpa]
343	101	gi|225429228	176.27	17	11	1		72770	PREDICTED: heat shock 70 kDa protein mitochondrial [Vitis vinifera]
174	17040	gi|566184599	173.38	24	10	3		37085	glyceraldehyde 3-phosphate dehydrogenase family protein [Populus trichocarpa]

163	203	gi|526117485	173.31	29	7	1		40813	naringenin 2-oxoglutarate 3-dioxygenase [Vitis vinifera]
147	16723	gi|224141865	173.03	36	8	8	Carbamidomethylation	25015	GTP-binding family protein [Populus trichocarpa]
196	16586	gi|671743392	172.41	30	13	10		55440	ATP synthase CF1 alpha subunit (chloroplast) [Camellia reticulata]
237	16787	gi|224102193	172.17	33	10	2	Carbamidomethylation	35716	cytosolic malate dehydrogenase family protein [Populus trichocarpa]
88	16659	gi|224110772	170.75	27	11	1	Carbamidomethylation	37124	glyceraldehyde 3-phosphate dehydrogenase family protein [Populus trichocarpa]
133	494	gi|225449541	170.29	24	7	2	Carbamidomethylation	27128	PREDICTED: triosephosphate isomerase cytosolic [Vitis vinifera]
230	89	gi|731402464	170.09	17	11	3	Carbamidomethylation	93319	PREDICTED: heat shock 70 kDa protein 14-like [Vitis vinifera]
231	295	gi|359480275	170.02	30	8	1		35505	PREDICTED: malate dehydrogenase cytoplasmic [Vitis vinifera]
168	16986	gi|224079530	169.84	19	8	1		39256	glutamate-ammonia ligase family protein [Populus trichocarpa]
283	16605	gi|224141947	168.97	25	11	3	Carbamidomethylation	53416	hypothetical protein POPTR_0018s02400g [Populus trichocarpa]
296	308	gi|731392297	167.64	29	12	2		31832	PREDICTED: histone H2B-like [Vitis vinifera]

255	16808	gi|566149378	167.46	31	8	2	Carbamidomethylation	34116	hypothetical protein POPTR_0001s16430g [Populus trichocarpa]
261	16636	gi|566181869	166.9	26	12	2	Carbamidomethylation	54252	hypothetical protein POPTR_0008s02110g [Populus trichocarpa]
233	248	gi|225433375	166.88	24	11	1	Carbamidomethylation	61370	PREDICTED: chaperonin CPN60-2 mitochondrial [Vitis vinifera]
137	16871	gi|566205887	166.64	29	10	1	Carbamidomethylation; Oxidation (M)	38451	glutamine synthetase family protein [Populus trichocarpa]
223	237	gi|225446579	165.85	14	9	1	Carbamidomethylation	63486	PREDICTED: ketol-acid reductoisomerase chloroplastic [Vitis vinifera]
134	338	gi|225427917	164.34	27	10	2	Carbamidomethylation	34675	PREDICTED: triosephosphate isomerase chloroplastic [Vitis vinifera]
300	16811	gi|566182073	164.34	55	11	1		15828	hypothetical protein POPTR_0008s03050g [Populus trichocarpa]
408	16616	gi|224105855	164.09	14	10	1		73261	heat shock protein 70 [Populus trichocarpa]
201	258	gi|731404310	163.82	29	8	5	Carbamidomethylation	33129	PREDICTED: putative lactoylglutathione lyase [Vitis vinifera]
194	16571	gi|566204389	163.5	14	14	1	Carbamidomethylation	109035	hypothetical protein POPTR_0014s15170g [Populus trichocarpa]
213	253	gi|225451581	163.33	30	10	1	Carbamidomethylation	38690	PREDICTED: mitochondrial phosphate carrier protein 3 mitochondrial isoform X1 [Vitis vinifera]

246	16674	gi|542688125	162.91	18	6	6		56099	photosystem II p680 chlorophyll A apoprotein CP-47 (chloroplast) [Camellia taliensis]
320	269	gi|359476642	162.86	23	7	1	Carbamidomethylation	42804	PREDICTED: fructose-bisphosphate aldolase 1 chloroplastic-like [Vitis vinifera]
335	141	gi|731419756	162.43	23	9	2	Carbamidomethylation	52962	PREDICTED: UDP-glucose 6-dehydrogenase 1 [Vitis vinifera]
185	16585	gi|224099261	161.49	15	13	1	Carbamidomethylation	90156	heat shock family protein [Populus trichocarpa]
175	16857	gi|224122864	161.31	69	10	1	Carbamidomethylation	20653	hypothetical protein POPTR_0013s00700g [Populus trichocarpa]
444	206	gi|731385022	161.18	16	12	4	Carbamidomethylation	114759	PREDICTED: glycine dehydrogenase (decarboxylating) mitochondrial [Vitis vinifera]
197	139	gi|225468576	160.84	16	13	3	Carbamidomethylation	98187	PREDICTED: aconitate hydratase 1 [Vitis vinifera]
217	17140	gi|224075758	160.79	61	8	3	Carbamidomethylation	15268	hypothetical protein POPTR_0003s22120g [Populus trichocarpa]
224	17082	gi|224068456	160.63	21	8	3	Carbamidomethylation	39431	thioredoxin family protein [Populus trichocarpa]
391	16552	gi|224101561	160.34	18	10	2	Carbamidomethylation	65798	subunit B of the trimeric enzyme ATP Citrate lyase family protein [Populus trichocarpa]

143	198	gi|731406211	160.16	11	10	3	Carbamidomethylation	92443	PREDICTED: sucrose synthase [Vitis vinifera]
310	257	gi|225466253	160.06	21	9	1	Carbamidomethylation	46284	PREDICTED: isocitrate dehydrogenase [NADP] [Vitis vinifera]
242	16574	gi|224058852	159.08	15	11	2	Carbamidomethylation	94161	heat shock protein 70 [Populus trichocarpa]
500	500	gi|225440011	159.07	14	6	1		77480	PREDICTED: phenylalanine ammonia-lyase [Vitis vinifera]
333	16791	gi|566173570	158.89	19	8	2	Carbamidomethylation	44912	hypothetical protein POPTR_0005s27550g [Populus trichocarpa]
267	17042	gi|566164883	158.58	24	10	3	Carbamidomethylation	43246	ketol-acid reductoisomerase family protein partial [Populus trichocarpa]
326	17023	gi|566202051	158.02	14	7	1	Carbamidomethylation	71118	hypothetical protein POPTR_0014s02550g [Populus trichocarpa]
486	583	gi|225435357	157.51	11	6	1		77966	PREDICTED: phenylalanine ammonia-lyase [Vitis vinifera]
205	74	gi|731393844	157.41	14	15	3	Carbamidomethylation	101855	PREDICTED: protein argonaute 4 [Vitis vinifera]
150	217	gi|225437428	157.19	10	9	2	Carbamidomethylation	92483	PREDICTED: sucrose synthase 2 [Vitis vinifera]
145	16968	gi|566210965	156.95	24	10	1		39217	glutamate-ammonia ligase family protein [Populus trichocarpa]
370	16748	gi|224060514	155.2	17	9	5	Carbamidomethylation	70476	polyadenylate-binding family protein [Populus trichocarpa]

250	16953	gi|224105301	154.22	24	7	1	Carbamidomethylation	42832	latex plastidic aldolase-like family protein [Populus trichocarpa]
173	17182	gi|566152287	154.21	20	7	1	Carbamidomethylation	45060	hypothetical protein POPTR_0001s32770g [Populus trichocarpa]
171	16797	gi|224109062	153.09	26	10	1		42679	cytosolic phosphoglycerate kinase family protein [Populus trichocarpa]
346	194	gi|225442018	152.75	26	7	1	Carbamidomethylation	33909	PREDICTED: 40S ribosomal protein SA [Vitis vinifera]
228	132	gi|731428778	152.31	24	11	3	Carbamidomethylation	56891	PREDICTED: serine hydroxymethyltransferase mitochondrial [Vitis vinifera]
512	17246	gi|566190515	152.15	25	6	5	Carbamidomethylation	42536	hypothetical protein POPTR_0010s12790g [Populus trichocarpa]
513	286	gi|731412519	152.14	11	5	1		78831	PREDICTED: phenylalanine ammonia-lyase G1 [Vitis vinifera]
285	228	gi|225451995	151.87	35	9	2		29540	PREDICTED: 14-3-3-like protein A [Vitis vinifera]
139	16937	gi|224098421	151.87	30	8	2	Carbamidomethylation	27259	Triosephosphate isomerase family protein [Populus trichocarpa]
357	84	gi|225442993	151.84	28	11	10	Carbamidomethylation	48355	PREDICTED: DEAD-box ATP-dependent RNA helicase 56 [Vitis vinifera]
177	16855	gi|566203721	150.93	69	10	1	Carbamidomethylation	20680	hypothetical protein POPTR_0014s11180g [Populus trichocarpa]

734	17041	gi|224095604	150.47	30	7	1		31880	PROHIBITIN 2 family protein [Populus trichocarpa]
262	389	gi|225441754	150.36	16	8	3	Carbamidomethylation	53382	PREDICTED: adenylosuccinate synthetase 2 chloroplastic [Vitis vinifera]
420	742	gi|526117784	150.21	28	6	2	Carbamidomethylation	30262	2-Cys peroxiredoxin [Vitis vinifera]
400	16544	gi|566173508	150.11	22	10	1	Carbamidomethylation	56756	catalase family protein [Populus trichocarpa]
451	111	gi|225432252	149.89	26	10	8		47374	PREDICTED: 26S protease regulatory subunit 6A homolog [Vitis vinifera]
287	316	gi|359480881	149.06	11	7	7	Carbamidomethylation	94936	PREDICTED: beta-galactosidase 10 [Vitis vinifera]
292	374	gi|225426162	148.95	36	6	3	Carbamidomethylation	20013	PREDICTED: 40S ribosomal protein S10 [Vitis vinifera]
239	304	gi|225425053	148.88	18	8	1	Carbamidomethylation	53492	PREDICTED: 6-phosphogluconate dehydrogenase decarboxylating 3 [Vitis vinifera]
449	449	gi|731420830	148.79	34	8	2		31951	PREDICTED: prohibitin-1 mitochondrial [Vitis vinifera]
360	16781	gi|566165396	148.69	17	7	4	Carbamidomethylation	51757	hypothetical protein POPTR_0004s07280g [Populus trichocarpa]
428	16716	gi|566190996	148.46	16	9	1	Carbamidomethylation	66968	hypothetical protein POPTR_0010s15590g [Populus trichocarpa]
340	108	gi|526117643	148.34	12	8	4		101329	heat shock protein 101 [Vitis vinifera]

321	728	gi|359485890	148.26	7	6	2	Carbamidomethylation	83365	PREDICTED: beta-xylosidase/alpha-L-arabinofuranosidase 2-like [Vitis vinifera]
159	16920	gi|566167040	148.13	27	8	2	Carbamidomethylation	33502	hypothetical protein POPTR_0004s17530g [Populus trichocarpa]
190	315	gi|225457971	147.88	23	6	1	Carbamidomethylation	40415	PREDICTED: flavanone 3-dioxygenase [Vitis vinifera]
491	254	gi|225430619	147.71	36	8	8	Carbamidomethylation	22781	PREDICTED: 40S ribosomal protein S5 [Vitis vinifera]
311	16653	gi|224072811	147.7	24	9	1		41910	hypothetical protein POPTR_0003s20360g [Populus trichocarpa]
398	163	gi|225456339	147.25	24	8	1	Carbamidomethylation	41981	PREDICTED: UDP-arabinopyranose mutase 1 isoform X2 [Vitis vinifera]
308	17076	gi|224143955	146.97	50	6	1	Carbamidomethylation	17511	eukaryotic translation initiation factor 5A isoform V family protein [Populus trichocarpa]
158	16754	gi|566176273	146.53	8	9	1		92143	sucrose synthase family protein [Populus trichocarpa]
358	16705	gi|566210462	146.24	13	8	2	Carbamidomethylation	67453	Methylenetetrahydrofolate reductase family protein [Populus trichocarpa]
424	17030	gi|566150460	146.16	72	7	7	Carbamidomethylation	16862	calmodulin-like protein 6a [Populus trichocarpa]
334	17398	gi|224138586	145.97	18	5	2		27577	ascorbate peroxidase family protein [Populus trichocarpa]

445	16639	gi|224086120	145.7	19	9	1	Carbamidomethylation	67209	pyrophosphate-dependent phosphofructokinase alpha subunit family protein [Populus trichocarpa]
208	437	gi|225465198	144.91	14	7	1	Carbamidomethylation	48147	PREDICTED: elongation factor 1-gamma-like [Vitis vinifera]
361	17069	gi|224077927	144.44	17	6	1	Carbamidomethylation	42796	latex plastidic aldolase-like family protein [Populus trichocarpa]
375	448	gi|526117711	144.21	18	5	1	Carbamidomethylation	36875	malate dehydrogenase [Vitis vinifera]
432	563	gi|731376167	143.36	28	5	2	Carbamidomethylation	29267	PREDICTED: proteasome subunit beta type-5 [Vitis vinifera]
279	16633	gi|566186668	142.81	23	9	2		41811	ADP family protein [Populus trichocarpa]
481	17029	gi|566210440	142.43	25	10	10	Carbamidomethylation	49767	GDP dissociation inhibitor family protein [Populus trichocarpa]
245	16663	gi|566170698	141.5	13	11	1	Carbamidomethylation	98215	aconitate hydratase family protein [Populus trichocarpa]
366	227	gi|224365636	140.9	11	6	6		69587	PSII 32 kDa protein (mitochondrion) [Vitis vinifera]
315	16635	gi|566178983	140.74	23	9	1		41070	hypothetical protein POPTR_0007s01010g [Populus trichocarpa]
430	16872	gi|566170807	140.55	23	7	1	Carbamidomethylation	40534	Chain A family protein [Populus trichocarpa]
85	16867	gi|566147823	140.09	9	7	3	Oxidation (M)	83349	hypothetical protein POPTR_0001s07870g [Populus trichocarpa]

474	17251	gi|224080233	139.93	15	7	1	Carbamidomethylation	53053	UDP-glucose 6-dehydrogenase family protein [Populus trichocarpa]
425	16958	gi|224099363	139.51	8	7	5		101194	alpha-xylosidase family protein [Populus trichocarpa]
497	211	gi|225423507	138.93	16	7	1	Carbamidomethylation	52744	PREDICTED: UDP-glucose 6-dehydrogenase 3 [Vitis vinifera]
211	255	gi|526117940	137.56	6	5	2		91805	phospholipase D alpha [Vitis vinifera]
407	17442	gi|566152994	137.07	19	9	2	Carbamidomethylation	57135	hypothetical protein POPTR_0001s35790g [Populus trichocarpa]
254	1727	gi|225465837	136.93	6	4	2	Carbamidomethylation	46787	PREDICTED: protein ASPARTIC PROTEASE IN GUARD CELL 2 [Vitis vinifera]
616	17433	gi|566214954	136.77	18	6	1	Carbamidomethylation; Oxidation (M)	39277	hypothetical protein POPTR_0018s09170g [Populus trichocarpa]
519	525	gi|225426063	135.94	16	6	1	Carbamidomethylation	53896	PREDICTED: 6-phosphogluconate dehydrogenase decarboxylating 1 chloroplastic [Vitis vinifera]
324	692	gi|731388721	135.77	25	6	3		18965	PREDICTED: translationally-controlled tumor protein homolog [Vitis vinifera]
240	17045	gi|224084209	135.42	18	7	2	Carbamidomethylation	35142	O2 evolving complex 33kD family protein [Populus trichocarpa]

275	17021	gi|224083020	135.15	39	7	1	Carbamidomethylation; Oxidation (M)	18138	Peptidyl-prolyl cis-trans isomerase family protein [Populus trichocarpa]
578	337	gi|225423961	135.08	9	6	4	Carbamidomethylation	103148	PREDICTED: alpha-xylosidase 1 [Vitis vinifera]
332	122	gi|731430736	135.04	19	7	3	Carbamidomethylation	44590	PREDICTED: 60S ribosomal protein L3 [Vitis vinifera]
685	311	gi|225430390	134.73	15	5	5	Carbamidomethylation	57260	PREDICTED: threonine synthase chloroplastic [Vitis vinifera]
415	585	gi|225444377	134.27	16	4	1	Carbamidomethylation	38105	PREDICTED: thiamine thiazole synthase 2 chloroplastic [Vitis vinifera]
475	16889	gi|566170533	134.15	15	7	7		47176	hypothetical protein POPTR_0005s10290g [Populus trichocarpa]
469	271	gi|225439064	133.69	14	6	3		61092	PREDICTED: 2 3-bisphosphoglycerate-independent phosphoglycerate mutase [Vitis vinifera]
280	16922	gi|224058615	133.57	21	5	2	Carbamidomethylation	33817	hypothetical protein POPTR_0001s08570g [Populus trichocarpa]
429	16898	gi|224116086	133.39	16	4	1	Carbamidomethylation	37284	hypothetical protein POPTR_0011s00500g [Populus trichocarpa]
390	363	gi|225464852	133.35	62	7	2	Carbamidomethylation	16577	PREDICTED: ubiquitin-conjugating enzyme E2 variant 1D [Vitis vinifera]
557	16970	gi|568244554	133.28	18	4	4		39549	photosystem II protein D2 (plastid) [Camellia oleifera]

411	627	gi|225464720	132.88	14	5	2	Carbamidomethylation	44946	PREDICTED: oxygen-dependent coproporphyrinogen-III oxidase chloroplastic-like [Vitis vinifera]
630	16998	gi|566156890	132.45	14	7	1		44077	hypothetical protein POPTR_0002s08240g [Populus trichocarpa]
124	16804	gi|224053300	132.3	56	9	9	Carbamidomethylation	17702	ubiquitin/ribosomal protein 27a [Populus trichocarpa]
534	327	gi|731425536	131.6	14	6	1	Carbamidomethylation; Oxidation (M)	52880	PREDICTED: UDP-glucose 6-dehydrogenase 1 isoform X2 [Vitis vinifera]
467	16560	gi|566206267	131.48	9	6	2		100324	hypothetical protein POPTR_0015s06760g [Populus trichocarpa]
383	460	gi|359483716	131.37	26	7	1	Carbamidomethylation	29551	PREDICTED: 14-3-3-like protein D isoform X2 [Vitis vinifera]
297	301	gi|225452510	130.87	10	7	2		58057	PREDICTED: aldehyde dehydrogenase family 2 member B7 mitochondrial [Vitis vinifera]
183	859	gi|225429614	130.79	44	5	3	Carbamidomethylation	17293	PREDICTED: 17.3 kDa class II heat shock protein [Vitis vinifera]
416	187	gi|225457674	130.56	16	9	2	Carbamidomethylation	67344	PREDICTED: pyrophosphate--fructose 6-phosphate 1-phosphotransferase subunit alpha [Vitis vinifera]

797	697	gi|731423169	130.53	32	5	5	Carbamidomethylation	13646	PREDICTED: 40S ribosomal protein S20-2 [Vitis vinifera]
100	410	gi|359475042	130.51	45	8	2	Carbamidomethylation; Oxidation (M)	18313	PREDICTED: peptidyl-prolyl cis-trans isomerase [Vitis vinifera]
414	17232	gi|566169561	130.43	18	5	1	Carbamidomethylation	34208	hypothetical protein POPTR_0005s05040g [Populus trichocarpa]
515	785	gi|225459806	130.21	16	9	2	Carbamidomethylation	57322	PREDICTED: T-complex protein 1 subunit beta [Vitis vinifera]
421	17072	gi|224114557	130.1	17	4	1	Carbamidomethylation	35588	Malate dehydrogenase family protein [Populus trichocarpa]
647	16870	gi|224092216	130.04	11	5	5	Carbamidomethylation	57364	glycine hydroxymethyltransferase family protein [Populus trichocarpa]
241	16807	gi|224101329	129.96	13	6	1	Carbamidomethylation	45140	glyceraldehyde 3-phosphate dehydrogenase 1 family protein [Populus trichocarpa]
489	932	gi|225450626	129.91	16	6	1	Carbamidomethylation	39269	PREDICTED: probable protein disulfide-isomerase A6 [Vitis vinifera]
499	201	gi|225442156	129.71	31	7	1	Carbamidomethylation	26179	PREDICTED: 40S ribosomal protein S3-3 [Vitis vinifera]
586	18654	gi|224053535	129.45	16	4	1		38585	pyruvate dehydrogenase family protein [Populus trichocarpa]
462	818	gi|731396254	129.24	15	6	5	Carbamidomethylation	40707	PREDICTED: alcohol dehydrogenase class-3 [Vitis vinifera]

533	16729	gi|566160536	129.22	9	5	1		91733	phospholipase D family protein [Populus trichocarpa]
483	97	gi|225441955	128.69	23	9	9	Carbamidomethylation	47682	PREDICTED: 26S protease regulatory subunit 7 [Vitis vinifera]
305	251	gi|225461654	128.51	33	9	3	Carbamidomethylation	28864	PREDICTED: 14-3-3-like protein D [Vitis vinifera]
493	16923	gi|552540866	128.29	22	7	7		35220	cytochrome f (chloroplast) [Camellia danzaiensis]
573	17453	gi|224088276	128.25	13	5	1		50030	leucine aminopeptidase family protein [Populus trichocarpa]
265	16617	gi|568244555	127.68	18	6	6		51822	photosystem II CP43 chlorophyll apoprotein (plastid) [Camellia oleifera]
715	223	gi|225429530	127.48	14	6	2	Carbamidomethylation	54362	PREDICTED: UDP-sulfoquinovose synthase chloroplastic [Vitis vinifera]
1027	706	gi|225446693	127.39	10	5	1		74318	PREDICTED: ATP-dependent zinc metalloprotease FTSH 2 chloroplastic [Vitis vinifera]
303	481	gi|225442079	127.34	23	5	5	Carbamidomethylation	27325	PREDICTED: proteasome subunit alpha type-6 [Vitis vinifera]
378	238	gi|359481634	127.05	52	6	1	Carbamidomethylation	16594	PREDICTED: ubiquitin-conjugating enzyme E2 variant 1D [Vitis vinifera]
148	899	gi|225431269	126.98	12	4	2	Carbamidomethylation	36580	PREDICTED: peroxidase 72 [Vitis vinifera]

476	333	gi|225447725	126.93	15	7	4	Carbamidomethylation	61438	PREDICTED: calnexin homolog [Vitis vinifera]
423	282	gi|731423943	126.91	31	5	2	Carbamidomethylation	19961	PREDICTED: 40S ribosomal protein S10 [Vitis vinifera]
750	155	gi|225446449	126.88	9	6	3		97503	PREDICTED: 26S proteasome non-ATPase regulatory subunit 2 homolog A [Vitis vinifera]
418	17046	gi|224085984	126.87	11	6	1	Carbamidomethylation	61382	multi-copper oxidase type 1 family protein [Populus trichocarpa]
235	209	gi|731408286	126.82	16	6	6		53389	PREDICTED: xylose isomerase [Vitis vinifera]
257	364	gi|225451146	126.82	17	8	5		57701	PREDICTED: bifunctional 3-dehydroquinate dehydratase/shikimate dehydrogenase chloroplastic [Vitis vinifera]
277	222	gi|225430398	126.78	16	8	6		50823	PREDICTED: aspartate aminotransferase chloroplastic [Vitis vinifera]
438	218	gi|731395250	126.71	13	5	1	Carbamidomethylation	48931	PREDICTED: elongation factor Tu mitochondrial [Vitis vinifera]
484	229	gi|731427020	126.68	10	6	2	Carbamidomethylation; Oxidation (M)	76330	PREDICTED: glycine--tRNA ligase 1 mitochondrial [Vitis vinifera]

373	216	gi|526117840	126.5	15	9	2		58698	3-deoxy-D-arabino-heptulosonate-7-phosphate synthase [Vitis vinifera]
598	314	gi|225432110	126.49	17	9	9	Carbamidomethylation	52920	PREDICTED: dihydrolipoyl dehydrogenase mitochondrial [Vitis vinifera]
570	17258	gi|224140177	126.41	10	7	2	Carbamidomethylation	53698	adenylosuccinate synthetase family protein [Populus trichocarpa]
1025	18083	gi|566159452	126.29	9	4	1		75110	hypothetical protein POPTR_0002s22560g [Populus trichocarpa]
477	335	gi|225445867	126.21	14	6	2		60743	PREDICTED: leucine aminopeptidase 1 [Vitis vinifera]
591	664	gi|731403303	126.21	37	5	2	Carbamidomethylation	15026	PREDICTED: 40S ribosomal protein S12 [Vitis vinifera]
204	440	gi|225461156	126.12	61	6	2		13980	PREDICTED: probable histone H2A.2 [Vitis vinifera]
281	17277	gi|566170863	126.01	50	5	5		11409	histone H4 family protein [Populus trichocarpa]
215	16673	gi|224130834	125.98	12	5	3	Carbamidomethylation	58002	tRNA synthetase-related family protein [Populus trichocarpa]
468	417	gi|225429850	125.84	21	4	3		24915	PREDICTED: proteasome subunit beta type-6 [Vitis vinifera]
347	584	gi|225451279	125.6	20	6	2		29903	PREDICTED: 40S ribosomal protein S3a-2-like [Vitis vinifera]

330	17149	gi|224081102	125.49	54	8	3	Carbamidomethylation	15406	hypothetical protein POPTR_0005s07340g [Populus trichocarpa]
655	1175	gi|359483879	125.31	15	5	1	Carbamidomethylation	43617	PREDICTED: LOW QUALITY PROTEIN: pyruvate dehydrogenase E1 component subunit beta-3 chloroplastic-like [Vitis vinifera]
611	137	gi|526117707	125.14	18	6	2	Carbamidomethylation	53695	uncharacterized protein LOC100261274 [Vitis vinifera]
412	140	gi|359475304	125	8	7	7	Carbamidomethylation	98624	PREDICTED: coatomer subunit gamma-2 [Vitis vinifera]
236	313	gi|225458424	124.99	17	6	1	Carbamidomethylation	35120	PREDICTED: oxygen-evolving enhancer protein 1 chloroplastic [Vitis vinifera]
226	170	gi|225446944	124.95	21	8	1	Carbamidomethylation	43340	PREDICTED: protochlorophyllide reductase [Vitis vinifera]
824	1012	gi|225430650	124.93	16	5	1	Carbamidomethylation; Oxidation (M)	43927	PREDICTED: pyruvate dehydrogenase E1 component subunit beta-3 chloroplastic [Vitis vinifera]
523	16665	gi|224087339	124.81	12	6	1	Carbamidomethylation	57707	biotin carboxylase precursor family protein [Populus trichocarpa]
725	18948	gi|566197644	124.29	21	5	4		26318	hypothetical protein POPTR_0012s09540g [Populus trichocarpa]

389	17070	gi|224064707	124.17	14	6	3	Carbamidomethylation	58836	mitochondrial aldehyde dehydrogenase family protein [Populus trichocarpa]
238	165	gi|359493870	124.14	17	8	1	Carbamidomethylation	42815	PREDICTED: protochlorophyllide reductase chloroplastic [Vitis vinifera]
282	633	gi|225444649	124.05	16	4	4		26396	PREDICTED: 20 kDa chaperonin chloroplastic [Vitis vinifera]
918	17095	gi|224100953	123.73	21	5	5	Carbamidomethylation	39107	thymidine diphospho-glucose 4-6-dehydratase family protein [Populus trichocarpa]
529	17518	gi|224054542	123.35	21	5	1	Carbamidomethylation	22400	peptidyl-prolyl cis-trans isomerase family protein [Populus trichocarpa]
546	572	gi|731382516	122.9	14	4	4		37912	PREDICTED: ankyrin repeat domain-containing protein 2 isoform X2 [Vitis vinifera]
691	17257	gi|224108938	122.67	53	6	3	Carbamidomethylation	17381	hypothetical protein POPTR_0010s17020g [Populus trichocarpa]
363	413	gi|225457075	122.45	11	5	1	Carbamidomethylation	58613	PREDICTED: aldehyde dehydrogenase family 2 member B4 mitochondrial [Vitis vinifera]

615	1238	gi|225425166	122.29	16	4	1		39489	PREDICTED: pyruvate dehydrogenase E1 component subunit beta-1 mitochondrial [Vitis vinifera]
338	16703	gi|224063273	122.11	30	8	1		29380	14-3-3 protein 32kDa endonuclease [Populus trichocarpa]
631	17382	gi|566183730	122.05	21	6	5	Carbamidomethylation	48131	hypothetical protein POPTR_0008s13180g [Populus trichocarpa]
132	17575	gi|566170929	121.86	12	4	1	Carbamidomethylation	36394	hypothetical protein POPTR_0005s12070g [Populus trichocarpa]
525	750	gi|225457502	120.93	12	3	3	Carbamidomethylation	35357	PREDICTED: probable succinyl-CoA ligase [ADP-forming] subunit alpha mitochondrial [Vitis vinifera]
485	473	gi|225436253	120.86	11	6	1	Carbamidomethylation	66904	PREDICTED: probable methylenetetrahydrofolate reductase [Vitis vinifera]
907	699	gi|225435309	120.82	8	6	6	Carbamidomethylation	82345	PREDICTED: 4-hydroxy-3-methylbut-2-en-1-yl diphosphate synthase chloroplastic [Vitis vinifera]
470	312	gi|225428247	120.4	12	6	1	Carbamidomethylation	60225	PREDICTED: L-ascorbate oxidase homolog [Vitis vinifera]
349	81	gi|225461660	120.14	7	7	1		109823	PREDICTED: phosphoenolpyruvate carboxylase housekeeping isozyme [Vitis vinifera]

392	843	gi|225439878	119.89	19	4	1	Carbamidomethylation	24585	PREDICTED: soluble inorganic pyrophosphatase [Vitis vinifera]
875	17351	gi|224108774	119.87	14	5	1	Carbamidomethylation	54256	Galactokinase family protein [Populus trichocarpa]
626	156	gi|225441236	119.81	4	5	5	Carbamidomethylation	177678	PREDICTED: ferredoxin-dependent glutamate synthase chloroplastic [Vitis vinifera]
556	16779	gi|566175799	119.78	15	8	1	Carbamidomethylation	57465	pyruvate kinase family protein [Populus trichocarpa]
342	602	gi|359476682	119.75	20	4	1	Carbamidomethylation	24349	PREDICTED: soluble inorganic pyrophosphatase isoform X2 [Vitis vinifera]
399	685	gi|225459808	119.64	9	6	1		60137	PREDICTED: L-ascorbate oxidase homolog [Vitis vinifera]
478	1514	gi|225450791	119.38	15	2	1		22379	PREDICTED: translationally-controlled tumor protein homolog [Vitis vinifera]
83	925	gi|225465425	119.36	8	5	4	Carbamidomethylation	52683	PREDICTED: uncharacterized protein LOC100241465 [Vitis vinifera]
431	708	gi|731392629	119.32	37	6	1		17778	PREDICTED: 60S ribosomal protein L12 [Vitis vinifera]
376	17348	gi|566184073	119.12	24	5	5		22518	photosystem I 20kD family protein [Populus trichocarpa]

600	596	gi|225466690	118.98	20	6	6		42525	PREDICTED: sedoheptulose-1 7-bisphosphatase chloroplastic [Vitis vinifera]
815	276	gi|225432012	118.81	13	5	1	Carbamidomethylation	54243	PREDICTED: galactokinase [Vitis vinifera]
536	598	gi|225454009	118.62	11	5	1	Carbamidomethylation	80666	PREDICTED: transketolase chloroplastic [Vitis vinifera]
628	519	gi|225450981	118.46	11	6	5	Carbamidomethylation	51125	PREDICTED: serine--tRNA ligase [Vitis vinifera]
350	663	gi|225449052	118.15	42	6	1		17824	PREDICTED: 60S ribosomal protein L12 [Vitis vinifera]
222	18771	gi|566242213	117.92	9	4	2		29428	hypothetical protein POPTR_0019s12380g [Populus trichocarpa]
440	17320	gi|566189392	117.87	18	5	5		27258	hypothetical protein POPTR_0010s06320g [Populus trichocarpa]
564	293	gi|225455784	117.58	18	7	5	Carbamidomethylation	50558	PREDICTED: enolase 1 chloroplastic [Vitis vinifera]
516	433	gi|731438766	117.48	16	5	4		46349	PREDICTED: malate dehydrogenase glyoxysomal isoform X1 [Vitis vinifera]
464	16940	gi|566206623	117.47	8	5	1		54072	aldehyde dehydrogenase 1 precursor family protein [Populus trichocarpa]
319	17266	gi|224104631	117.3	23	6	1		27319	cytosolic ascorbate peroxidase family protein [Populus trichocarpa]

314	703	gi|731388929	117.15	14	7	3		56541	PREDICTED: bifunctional 3-dehydroquinate dehydratase/shikimate dehydrogenase chloroplastic isoform X2 [Vitis vinifera]
298	17234	gi|566207041	116.94	12	4	2	Carbamidomethylation	53652	glutamate 1-semialdehyde aminotransferase family protein [Populus trichocarpa]
352	527	gi|731368860	116.79	19	6	1		39024	PREDICTED: glutamine synthetase isoform X1 [Vitis vinifera]
380	16896	gi|224107259	116.55	17	7	1		47894	glutamine synthetase family protein [Populus trichocarpa]
249	16784	gi|566175737	116.53	16	7	7	Carbamidomethylation	47050	Monodehydroascorbate reductase family protein [Populus trichocarpa]
595	107	gi|225452950	116.51	5	4	2		109834	PREDICTED: chaperone protein ClpB3 chloroplastic [Vitis vinifera]
653	637	gi|225431563	116.26	8	4	1	Carbamidomethylation	51250	PREDICTED: UTP--glucose-1-phosphate uridylyltransferase isoform X1 [Vitis vinifera]
561	17197	gi|566153824	116.17	46	6	5	Carbamidomethylation	17248	ubiquitin-conjugating enzyme family protein [Populus trichocarpa]
395	17211	gi|224135485	116.08	10	5	1	Carbamidomethylation	58870	glycosyl hydrolase family 1 family protein [Populus trichocarpa]

723	171	gi|225441044	115.93	13	7	1		57520	PREDICTED: pyruvate kinase cytosolic isozyme-like [Vitis vinifera]
504	18346	gi|566184549	115.81	24	5	2	Carbamidomethylation	29191	20S proteasome beta subunit family protein [Populus trichocarpa]
594	1529	gi|225432466	115.75	22	3	3	Carbamidomethylation	18476	PREDICTED: peptide methionine sulfoxide reductase B5-like [Vitis vinifera]
526	710	gi|731382436	115.2	20	5	2		35672	PREDICTED: LOW QUALITY PROTEIN: isoflavone reductase-like protein [Vitis vinifera]
359	659	gi|225435177	115.06	20	6	1	Carbamidomethylation	27557	PREDICTED: L-ascorbate peroxidase 2 cytosolic [Vitis vinifera]
853	1208	gi|225424530	115.03	22	5	5		36021	PREDICTED: eukaryotic translation initiation factor 3 subunit I [Vitis vinifera]
633	606	gi|731438689	114.88	13	5	3		44313	PREDICTED: FAM10 family protein At4g22670 [Vitis vinifera]
461	17494	gi|224134414	114.86	13	3	2		25625	Proteasome subunit alpha type 2 family protein [Populus trichocarpa]
290	969	gi|225426753	114.84	9	6	2	Oxidation (M)	81719	PREDICTED: primary amine oxidase [Vitis vinifera]
674	18232	gi|566187006	114.83	10	4	2		50172	hypothetical protein POPTR_0009s08570g [Populus trichocarpa]

268	902	gi|225461808	114.78	13	7	1		75747	PREDICTED: trifunctional UDP-glucose 4 6-dehydratase/UDP-4-keto-6-deoxy-D-glucose 3 5-epimerase/UDP-4-keto-L-rhamnose-reductase RHM1 [Vitis vinifera]
974	520	gi|225431090	114.49	36	7	7	Carbamidomethylation	27195	PREDICTED: proteasome subunit alpha type-7 [Vitis vinifera]
402	541	gi|225436900	114.31	17	7	1		47680	PREDICTED: glutamine synthetase leaf isozyme chloroplastic [Vitis vinifera]
369	1025	gi|225456104	114.09	9	5	1	Carbamidomethylation	58411	PREDICTED: beta-glucosidase 44 [Vitis vinifera]
520	17820	gi|566175251	113.72	12	4	4		34569	hypothetical protein POPTR_0006s08710g [Populus trichocarpa]
509	832	gi|225461287	113.68	20	3	3	Carbamidomethylation	24076	PREDICTED: cytochrome b6-f complex iron-sulfur subunit 1 chloroplastic [Vitis vinifera]
458	123	gi|731391766	113.67	5	6	1	Carbamidomethylation	127035	PREDICTED: ubiquitin-activating enzyme E1 1 [Vitis vinifera]
528	16943	gi|566153872	113.61	13	5	4		40536	glycolate oxidase family protein [Populus trichocarpa]

688	17457	gi|224129210	113.46	16	7	7	Carbamidomethylation	41121	truncated acetyl Co-A acetyltransferase-like family protein [Populus trichocarpa]
712	231	gi|225450275	113.22	18	6	3	Carbamidomethylation	35771	PREDICTED: serine/threonine-protein phosphatase PP2A-2 catalytic subunit [Vitis vinifera]
559	247	gi|225452974	113.21	12	5	2		58482	PREDICTED: probable mitochondrial-processing peptidase subunit beta [Vitis vinifera]
863	17952	gi|224064888	113.17	32	3	1	Carbamidomethylation	11576	Ubiquitin-like family protein [Populus trichocarpa]
178	17181	gi|566259890	112.99	11	8	3		48155	elongation factor 1-gamma 1 family protein [Populus trichocarpa]
867	16706	gi|224113365	112.39	5	5	5		130750	NtN2 family protein [Populus trichocarpa]
842	16776	gi|224095561	112.31	8	5	2		97704	hypothetical protein POPTR_0007s01200g [Populus trichocarpa]
657	1650	gi|225439520	111.9	11	6	3		65247	PREDICTED: pyruvate decarboxylase 2 [Vitis vinifera]
636	1073	gi|225457407	111.31	12	5	5		43604	PREDICTED: malate dehydrogenase chloroplastic [Vitis vinifera]

872	593	gi|731412938	111.23	3	5	1	Carbamidomethylation	155024	PREDICTED: probable phosphoribosylformylglycinamidine synthase chloroplastic/mitochondrial [Vitis vinifera]
269	17194	gi|566181525	111.1	13	6	1		42160	hypothetical protein POPTR_0008s00350g [Populus trichocarpa]
527	17541	gi|566196811	111.01	8	4	1	Carbamidomethylation	62410	hypothetical protein POPTR_0012s04670g [Populus trichocarpa]
452	17771	gi|566148911	110.48	8	3	2		48782	hypothetical protein POPTR_0001s14280g [Populus trichocarpa]
758	16656	gi|224124848	110.42	30	7	7	Carbamidomethylation	24893	60S ribosomal protein L10 [Populus trichocarpa]
518	16921	gi|224123784	110.18	12	6	5	Carbamidomethylation	58663	hypothetical protein POPTR_0013s05500g [Populus trichocarpa]
960	17342	gi|224077760	109.96	26	4	1		15880	40S ribosomal protein S19 [Populus trichocarpa]
482	17536	gi|566178862	109.76	9	4	3		34943	hypothetical protein POPTR_0007s00300g [Populus trichocarpa]
877	1243	gi|225438781	109.46	7	3	1	Carbamidomethylation	71362	PREDICTED: polyadenylate-binding protein 2 [Vitis vinifera]
675	1205	gi|225443286	109.03	10	4	1		43933	PREDICTED: pyruvate dehydrogenase E1 component subunit alpha mitochondrial [Vitis vinifera]
708	17574	gi|566178300	108.97	8	3	2	Carbamidomethylation	45519	hypothetical protein POPTR_0006s26630g [Populus trichocarpa]

899	17949	gi|224132216	108.85	31	3	1	Carbamidomethylation	11688	hypothetical protein POPTR_0014s18990g [Populus trichocarpa]
558	17223	gi|224139104	107.93	15	5	5	Carbamidomethylation	33044	pyridoxin biosynthesis PDX1-like protein 3 [Populus trichocarpa]
410	369	gi|359492927	107.83	19	5	1		28650	PREDICTED: 14-3-3-like protein GF14 kappa [Vitis vinifera]
780	721	gi|225429209	107.82	7	4	3		53142	PREDICTED: serine carboxypeptidase-like 16 [Vitis vinifera]
538	17252	gi|224075976	107.46	21	5	5	Carbamidomethylation	31893	hypothetical protein POPTR_0003s21070g [Populus trichocarpa]
577	496	gi|225449659	107.3	10	6	1		57679	PREDICTED: pyruvate kinase cytosolic isozyme-like [Vitis vinifera]
703	549	gi|225430366	107.17	4	4	1		108100	PREDICTED: chaperone protein ClpB4 mitochondrial [Vitis vinifera]
1099	17612	gi|224104483	107.07	14	3	2		23714	hypothetical protein POPTR_0009s03300g [Populus trichocarpa]
592	17315	gi|224079274	107.04	14	5	2		41124	ubiquitin family protein [Populus trichocarpa]
677	17738	gi|224066507	106.91	11	5	1	Carbamidomethylation	43683	mRNA-binding family protein [Populus trichocarpa]
806	17298	gi|224063082	106.91	10	2	2		28992	Photosystem II 22 kDa family protein [Populus trichocarpa]
963	19656	gi|566169467	106.57	17	2	1		13987	hypothetical protein POPTR_0005s04590g [Populus trichocarpa]

293	807	gi|225461209	106.5	25	4	2		21740	PREDICTED: NAD(P)H dehydrogenase (quinone) FQR1 [Vitis vinifera]
465	2170	gi|225425388	106.45	30	2	2		13594	PREDICTED: nuclear transport factor 2 [Vitis vinifera]
709	319	gi|359478860	106.44	13	5	4	Carbamidomethylation	47055	PREDICTED: 26S protease regulatory subunit 6B homolog [Vitis vinifera]
724	979	gi|731416996	106.42	9	4	4		46827	PREDICTED: poly(rC)-binding protein 3 [Vitis vinifera]
539	17104	gi|671743459	106.41	46	4	4	Carbamidomethylation	9038	photosystem I subunit VII (chloroplast) [Camellia reticulata]
521	17558	gi|224127346	106.29	11	4	1		36420	arginase family protein [Populus trichocarpa]
442	412	gi|225443760	105.95	22	5	1	Carbamidomethylation	20875	PREDICTED: 60S ribosomal protein L11-1-like [Vitis vinifera]
738	17062	gi|224090212	105.87	14	4	2	Carbamidomethylation	29609	40S ribosomal protein S2 [Populus trichocarpa]
779	991	gi|359494079	105.29	10	4	4	Carbamidomethylation	65102	PREDICTED: LOW QUALITY PROTEIN: 65-kDa microtubule-associated protein 1-like [Vitis vinifera]
419	17014	gi|224140653	105.16	7	4	1		61182	phosphoglycerate mutase family protein [Populus trichocarpa]
571	547	gi|225456295	105.03	20	5	3		24961	PREDICTED: elongation factor 1-delta [Vitis vinifera]

849	17417	gi|671743328	104.99	8	3	3		56209	acetyl-CoA carboxylase carboxyltransferase beta subunit (chloroplast) [Camellia pubicosta]
328	466	gi|225426743	104.94	16	4	1	Carbamidomethylation	33675	PREDICTED: bifunctional dTDP-4-dehydrorhamnose 3 5-epimerase/dTDP-4-dehydrorhamnose reductase [Vitis vinifera]
524	16818	gi|224135747	104.9	30	7	1	Carbamidomethylation	26076	40S ribosomal protein S3 [Populus trichocarpa]
1071	19561	gi|224102811	104.7	11	3	3		40240	hypothetical protein POPTR_0009s16680g [Populus trichocarpa]
866	17297	gi|224141065	104.62	8	4	1		58604	mitochondrial processing peptidase beta subunit family protein [Populus trichocarpa]
789	239	gi|225458111	104.57	6	6	1		136997	PREDICTED: coatomer subunit alpha-1 [Vitis vinifera]
624	612	gi|225423953	104.4	17	5	2		41947	PREDICTED: ubiquitin receptor RAD23c isoform X2 [Vitis vinifera]
666	18119	gi|224063066	104.31	7	4	1		56188	disulfide-isomerase family protein [Populus trichocarpa]
717	17587	gi|566184208	104.3	16	4	2	Carbamidomethylation	27210	hypothetical protein POPTR_0008s15820g [Populus trichocarpa]

706	438	gi|225425318	104.29	5	5	5	Carbamidomethylation	108282	PREDICTED: DNA replication licensing factor MCM2 [Vitis vinifera]
434	372	gi|225446664	104.1	26	6	1	Carbamidomethylation	20845	PREDICTED: 60S ribosomal protein L11 [Vitis vinifera]
567	731	gi|225449971	104.03	4	4	3		121958	PREDICTED: protein transport protein SEC31 homolog B isoform X2 [Vitis vinifera]
368	18002	gi|566185685	104.02	14	3	3	Carbamidomethylation	22408	hypothetical protein POPTR_0009s01050g [Populus trichocarpa]
894	19692	gi|568244583	103.74	21	3	3		21881	clp protease proteolytic subunit (plastid) [Camellia oleifera]
244	226	gi|359497288	103.66	9	4	3	Carbamidomethylation	64041	PREDICTED: peptidyl-prolyl cis-trans isomerase FKBP62 isoform X1 [Vitis vinifera]
721	727	gi|225464809	103.61	10	5	1	Carbamidomethylation	56995	PREDICTED: gamma aminobutyrate transaminase 1 mitochondrial-like [Vitis vinifera]
480	17440	gi|566170829	103.53	16	4	1		40626	hypothetical protein POPTR_0005s11610g [Populus trichocarpa]
730	16959	gi|566202193	103.5	23	6	6		29275	hypothetical protein POPTR_0014s03240g [Populus trichocarpa]
871	605	gi|731399856	103.49	5	5	1		104341	PREDICTED: villin-2 [Vitis vinifera]
680	17789	gi|566172819	103.34	11	5	1	Carbamidomethylation	47443	hypothetical protein POPTR_0005s23110g [Populus trichocarpa]

443	16836	gi|566208295	103.08	7	8	1	Carbamidomethylation	102818	hypothetical protein POPTR_0016s02480g [Populus trichocarpa]
505	16874	gi|224082478	103.08	9	4	1	Carbamidomethylation	44381	ribosomal protein 1 [Populus trichocarpa]
501	642	gi|225432758	102.72	15	5	1	Carbamidomethylation	29774	PREDICTED: 40S ribosomal protein S3a-1 [Vitis vinifera]
761	29329	gi|224141875	102.33	18	3	3	Carbamidomethylation	16908	zinc finger family protein [Populus trichocarpa]
792	398	gi|731405921	102.32	11	4	3	Carbamidomethylation	34871	PREDICTED: phytochrome-associated serine/threonine-protein phosphatase [Vitis vinifera]
441	17083	gi|224091827	102.28	28	6	1	Carbamidomethylation	20659	60S ribosomal protein L11-2 [Populus trichocarpa]
612	17450	gi|566176147	102.16	8	3	3	Carbamidomethylation	41553	hypothetical protein POPTR_0006s13470g [Populus trichocarpa]
635	17873	gi|224058705	102.13	21	2	1	Carbamidomethylation	18641	cytochrome c oxidase-related family protein [Populus trichocarpa]
700	1029	gi|359473645	102.12	6	5	2	Carbamidomethylation	66079	PREDICTED: chaperonin 60 subunit beta 4 chloroplastic [Vitis vinifera]
649	18344	gi|224138966	101.99	9	3	3		44974	Chain D family protein [Populus trichocarpa]
632	192	gi|225434712	101.88	29	7	3		29576	PREDICTED: proliferating cell nuclear antigen [Vitis vinifera]
344	769	gi|359488553	101.84	4	4	2		115409	PREDICTED: alpha-mannosidase isoform X2 [Vitis vinifera]

704	321	gi|225457058	101.83	8	4	4	Carbamidomethylation	60477	PREDICTED: T-complex protein 1 subunit gamma [Vitis vinifera]
667	17053	gi|566184833	101.72	12	5	1	Carbamidomethylation	53373	alanine-2-oxoglutarate aminotransferase 1 family protein [Populus trichocarpa]
517	545	gi|526117441	101.59	5	5	2		101670	lipoxygenase [Vitis vinifera]
795	17473	gi|224086589	101.49	11	5	1	Carbamidomethylation	56517	POLLEN-PISTIL INTERACTION 2 family protein [Populus trichocarpa]
845	1222	gi|225457219	101.18	13	4	1	Carbamidomethylation	35220	PREDICTED: ATP synthase subunit gamma mitochondrial [Vitis vinifera]
574	18711	gi|224128708	100.64	12	3	1		33828	xyloglucan endo-1 family protein [Populus trichocarpa]
646	16664	gi|566190182	100.62	11	4	3	Carbamidomethylation	47998	26S proteasome subunit 8 family protein [Populus trichocarpa]
547	17489	gi|224071429	100.35	11	4	3	Carbamidomethylation	44978	Phosphoribulokinase family protein [Populus trichocarpa]
801	16895	gi|566191895	100.19	3	6	1		154710	phosphoribosylformylglycinamidine synthase family protein [Populus trichocarpa]
388	566	gi|731407251	100.12	3	3	1		92866	PREDICTED: phospholipase D alpha 1-like [Vitis vinifera]
463	746	gi|225447041	100.11	8	4	2	Carbamidomethylation	60008	PREDICTED: L-ascorbate oxidase homolog [Vitis vinifera]

364	811	gi|225456404	99.93	9	3	1	Carbamidomethylation	50437	PREDICTED: glutamate-1-semialdehyde 2 1-aminomutase chloroplastic [Vitis vinifera]
805	2319	gi|225452270	99.92	11	3	2		34598	PREDICTED: 28 kDa ribonucleoprotein chloroplastic-like [Vitis vinifera]
457	18405	gi|224095668	99.84	6	2	1		35550	hypothetical protein POPTR_0007s01850g [Populus trichocarpa]
751	17519	gi|224100211	99.52	10	4	1		43420	pyruvate dehydrogenase family protein [Populus trichocarpa]
492	953	gi|526118138	99.49	17	4	1		33840	uncharacterized protein LOC100264250 [Vitis vinifera]
494	729	gi|731421236	99.4	11	4	1		37446	PREDICTED: arginase 1 mitochondrial [Vitis vinifera]
1045	17402	gi|566197266	99.38	23	5	2	Carbamidomethylation	35019	hypothetical protein POPTR_0012s07510g [Populus trichocarpa]
692	325	gi|225456051	99.26	9	4	1	Carbamidomethylation	57589	PREDICTED: T-complex protein 1 subunit delta [Vitis vinifera]
606	17569	gi|566167161	99.17	9	3	1	Carbamidomethylation	40240	hypothetical protein POPTR_0004s18030g [Populus trichocarpa]
827	18399	gi|566174518	98.64	12	3	2	Carbamidomethylation	40168	hypothetical protein POPTR_0006s04880g [Populus trichocarpa]
645	17717	gi|224101117	98.19	10	3	3		34367	60S acidic ribosomal protein P0-A [Populus trichocarpa]

935	18215	gi|566147574	98.16	10	4	4		44838	hypothetical protein POPTR_0001s06560g [Populus trichocarpa]
923	1009	gi|359480225	97.7	17	4	1	Carbamidomethylation	27397	PREDICTED: peptidyl-prolyl cis-trans isomerase [Vitis vinifera]
762	601	gi|731390491	97.56	4	3	2		84964	PREDICTED: protein transport protein SEC23-like [Vitis vinifera]
619	18135	gi|566160281	97.5	12	3	1		25839	beta-hydroxyacyl-ACP dehydratase family protein [Populus trichocarpa]
84	736	gi|225455934	97.42	19	4	2	Carbamidomethylation; Oxidation (M)	20429	PREDICTED: ribulose bisphosphate carboxylase small chain chloroplastic [Vitis vinifera]
956	17512	gi|566158761	97.19	15	4	1	Carbamidomethylation	29101	cyclophilin family protein [Populus trichocarpa]
382	1653	gi|225429975	96.87	8	3	3		39098	PREDICTED: bark storage protein A [Vitis vinifera]
920	16906	gi|224118744	96.86	8	4	1	Carbamidomethylation	57369	chaperonin family protein [Populus trichocarpa]
627	2050	gi|731393849	96.84	6	3	2		56128	PREDICTED: UBP1-associated protein 2A [Vitis vinifera]
713	816	gi|526118026	96.79	18	5	4	Carbamidomethylation	55487	5-enolpyruvylshikimate-3-phosphate synthase [Vitis vinifera]
541	1587	gi|225453350	96.75	16	2	2		16310	PREDICTED: nucleoside diphosphate kinase B [Vitis vinifera]

854	18325	gi|566204282	96.73	12	4	3	Carbamidomethylation	37909	aldo/keto reductase family protein [Populus trichocarpa]
778	592	gi|225437537	96.71	26	4	4		22019	PREDICTED: GTP-binding protein SAR1A [Vitis vinifera]
580	17243	gi|224100839	96.71	21	4	2		19919	ATP synthase D chain-related family protein [Populus trichocarpa]
883	17917	gi|566206287	96.62	17	5	2		35464	ATP synthase gamma chain family protein [Populus trichocarpa]
270	17618	gi|224102515	96.61	10	4	2		29608	porin family protein [Populus trichocarpa]
1001	422	gi|731439184	96.56	7	4	4		109426	PREDICTED: 26S proteasome non-ATPase regulatory subunit 1 homolog A [Vitis vinifera]
341	17164	gi|566147610	96.33	24	5	2		20417	hypothetical protein POPTR_0001s06720g [Populus trichocarpa]
884	18553	gi|224066545	96.01	25	4	1	Carbamidomethylation	15325	40S ribosomal protein S12-1 [Populus trichocarpa]
610	987	gi|731411833	95.93	14	3	2	Carbamidomethylation	35859	PREDICTED: nitrilase-like protein 2 [Vitis vinifera]
329	17044	gi|566194782	95.46	7	5	1	Carbamidomethylation	75679	NAD-dependent epimerase/dehydratase family protein [Populus trichocarpa]
796	17548	gi|224139168	95.38	9	4	1	Carbamidomethylation; Oxidation (M)	65070	pyruvate decarboxylase family protein [Populus trichocarpa]
1016	1521	gi|225434319	95.27	42	5	4	Carbamidomethylation	16482	PREDICTED: ubiquitin-conjugating enzyme E2-17 kDa [Vitis vinifera]

488	17471	gi|566210997	95.18	11	4	4		47829	hypothetical protein POPTR_0017s02900g [Populus trichocarpa]
710	17490	gi|224137858	94.98	15	4	4		41555	hypothetical protein POPTR_0016s04600g [Populus trichocarpa]
979	852	gi|225423722	94.94	13	2	1		25584	PREDICTED: proteasome subunit alpha type-2-B [Vitis vinifera]
908	17238	gi|566164179	94.68	6	4	4	Carbamidomethylation	61299	hypothetical protein POPTR_0004s00550g [Populus trichocarpa]
542	1264	gi|225442963	94.44	8	3	2		35176	PREDICTED: methyl-CpG-binding domain-containing protein 11 [Vitis vinifera]
629	17782	gi|224143559	94.43	9	3	1		44475	aspartate aminotransferase family protein [Populus trichocarpa]
648	424	gi|225428772	94.33	5	5	2		121098	PREDICTED: presequence protease 2 chloroplastic/mitochondrial-like [Vitis vinifera]
551	887	gi|359479647	94.19	28	6	6	Carbamidomethylation	22491	PREDICTED: proteasome subunit beta type-2-A [Vitis vinifera]
560	809	gi|225445206	94.14	13	4	1		45116	PREDICTED: aspartate aminotransferase cytoplasmic [Vitis vinifera]
543	17370	gi|566192956	94.13	10	4	1	Carbamidomethylation	44225	hypothetical protein POPTR_0011s00650g [Populus trichocarpa]

773	17124	gi|566259876	93.61	19	4	1		23976	hypothetical protein POPTR_0022s00350g [Populus trichocarpa]
980	2321	gi|731416683	93.59	20	2	1	Carbamidomethylation	15282	PREDICTED: superoxide dismutase [Cu-Zn] isoform X2 [Vitis vinifera]
327	467	gi|225439749	93.37	46	4	3		15764	PREDICTED: histone H2A [Vitis vinifera]
1105	1389	gi|225450549	93.1	7	4	1	Carbamidomethylation	59190	PREDICTED: importin subunit alpha [Vitis vinifera]
426	444	gi|225437777	93.09	6	3	1	Carbamidomethylation	58724	PREDICTED: putative proline--tRNA ligase C19C7.06 [Vitis vinifera]
448	836	gi|225458231	92.85	16	4	4		30646	PREDICTED: proteasome subunit alpha type-1-B [Vitis vinifera]
940	2325	gi|225443274	92.78	12	3	3		35506	PREDICTED: cell wall integrity protein scw1 [Vitis vinifera]
553	16841	gi|669099987	92.61	7	6	5		83116	photosystem I P700 chlorophyll A apoprotein A1 (chloroplast) [Camellia crapnelliana]
295	1539	gi|225429604	92.6	21	3	1		17412	PREDICTED: 17.3 kDa class II heat shock protein [Vitis vinifera]
892	17535	gi|566180192	92.55	19	3	3		27792	hypothetical protein POPTR_0007s08020g [Populus trichocarpa]
1151	951	gi|731371455	92.45	8	4	3		58282	PREDICTED: coatomer subunit delta [Vitis vinifera]
775	17391	gi|566182904	92.28	10	5	5	Carbamidomethylation	59760	hypothetical protein POPTR_0008s07890g [Populus trichocarpa]

1035	3000	gi|225453620	92.23	7	2	2	Carbamidomethylation; Oxidation (M)	47761	PREDICTED: pyruvate dehydrogenase E1 component subunit alpha-3 chloroplastic [Vitis vinifera]
584	17961	gi|224107122	92.1	24	4	1	Oxidation (M)	19067	translationally controlled tumor-like family protein [Populus trichocarpa]
785	391	gi|225425280	92	10	4	1		48103	PREDICTED: uncharacterized protein LOC100247241 [Vitis vinifera]
1128	18313	gi|224053607	91.86	11	3	1		34050	EXGT1 family protein [Populus trichocarpa]
374	17196	gi|224121218	91.82	9	3	1	Carbamidomethylation	36046	GTP-binding protein beta chain [Populus trichocarpa]
548	18516	gi|224104953	91.76	5	3	1		68350	beta-D-glucan exohydrolase family protein [Populus trichocarpa]
460	18187	gi|566211618	91.65	10	3	2		35272	pfkB-type carbohydrate kinase family protein [Populus trichocarpa]
699	639	gi|731428049	91.5	9	3	2	Carbamidomethylation	47334	PREDICTED: delta-aminolevulinic acid dehydratase chloroplastic [Vitis vinifera]
562	804	gi|225460542	91.5	10	3	2		36331	PREDICTED: heterogeneous nuclear ribonucleoprotein 1 [Vitis vinifera]

1217	18282	gi|566192369	91.49	7	2	2		57235	Serine carboxypeptidase precursor family protein [Populus trichocarpa]
870	581	gi|731401982	91.28	5	3	3	Carbamidomethylation	60389	PREDICTED: dihydrolipoyllysine-residue acetyltransferase component 2 of pyruvate dehydrogenase complex mitochondrial [Vitis vinifera]
833	16576	gi|224119200	91.27	5	6	1		136903	coatomer alpha subunit-like family protein [Populus trichocarpa]
507	941	gi|225456914	91.11	25	4	2	Carbamidomethylation	16350	PREDICTED: 60S ribosomal protein L27a-3 [Vitis vinifera]
993	16891	gi|224109580	91.11	5	4	4	Oxidation (M)	115877	2-oxoglutarate dehydrogenase E1 component family protein [Populus trichocarpa]
1012	1064	gi|225462285	91.06	9	4	4		52600	PREDICTED: inosine-5'-monophosphate dehydrogenase [Vitis vinifera]
687	1304	gi|359475330	90.89	14	2	2		16329	PREDICTED: glycine-rich RNA-binding protein GRP2A [Vitis vinifera]
1029	1301	gi|731426495	90.88	3	3	2		120041	PREDICTED: alpha-mannosidase [Vitis vinifera]

843	1325	gi|731411781	90.74	14	3	3		25284	PREDICTED: manganese superoxide dismutase isoform X1 [Vitis vinifera]
618	17721	gi|566171771	90.53	9	4	2		53299	hypothetical protein POPTR_0005s16590g partial [Populus trichocarpa]
658	1011	gi|225435878	90.5	28	3	2		10567	PREDICTED: 10 kDa chaperonin [Vitis vinifera]
613	874	gi|359478691	90.47	4	3	1		92178	PREDICTED: beta-galactosidase 8 [Vitis vinifera]
1074	17671	gi|566183513	90.44	6	3	2		62808	hypothetical protein POPTR_0008s11770g [Populus trichocarpa]
695	743	gi|359482848	90.37	10	4	2		43630	PREDICTED: nucleosome assembly protein 1;4 [Vitis vinifera]
969	17098	gi|566200621	90.22	14	3	3		24286	60S ribosomal protein L15 [Populus trichocarpa]
1059	709	gi|359484512	90.12	5	2	2		63994	PREDICTED: asparagine--tRNA ligase cytoplasmic 1 [Vitis vinifera]
672	18559	gi|566206007	89.4	3	5	3	Carbamidomethylation	104242	hypothetical protein POPTR_0015s05540g [Populus trichocarpa]
1195	17004	gi|224143678	89.19	4	4	2		104783	plasma membrane H+ ATPase family protein [Populus trichocarpa]
807	17003	gi|224102919	89.18	5	3	2		60331	chaperonin family protein [Populus trichocarpa]
252	17233	gi|224065421	89.16	4	2	1		64078	hypothetical protein POPTR_0002s24970g [Populus trichocarpa]

588	30216	gi|566168757	88.98	7	2	1		42533	hypothetical protein POPTR_0005s01370g [Populus trichocarpa]
781	2084	gi|359474971	88.94	8	3	2	Oxidation (M)	59248	PREDICTED: ubiquitin domain-containing protein DSK2b-like isoform X2 [Vitis vinifera]
1041	204	gi|731401620	88.89	6	5	1		104406	PREDICTED: ATPase 10 plasma membrane-type [Vitis vinifera]
772	189	gi|731428324	88.85	2	4	4		252296	PREDICTED: acetyl-CoA carboxylase 1-like [Vitis vinifera]
957	19132	gi|224073194	88.85	4	3	3		67506	10-formyltetrahydrofolate synthetase family protein [Populus trichocarpa]
1030	1823	gi|225423947	88.79	7	3	2		50179	PREDICTED: dihydrolipoyllysine-residue acetyltransferase component 4 of pyruvate dehydrogenase complex chloroplastic [Vitis vinifera]
921	262	gi|225428386	88.75	9	3	2		44655	PREDICTED: 26S protease regulatory subunit S10B homolog B [Vitis vinifera]
767	18648	gi|566147678	88.61	13	2	2		22319	hypothetical protein POPTR_0001s07160g [Populus trichocarpa]
859	3226	gi|526118000	88.54	13	3	1		22732	germin-like protein 2 precursor [Vitis vinifera]

812	16798	gi|566149322	88.44	4	4	1		105097	putative plasma membrane H+ ATPase family protein [Populus trichocarpa]
753	17854	gi|224062834	88.4	4	2	1	Carbamidomethylation	70900	Polyadenylate-binding protein 5 [Populus trichocarpa]
735	367	gi|225444472	88.4	8	3	3	Carbamidomethylation	44306	PREDICTED: aminomethyltransferase mitochondrial [Vitis vinifera]
609	629	gi|359492254	88.34	11	7	2		40876	PREDICTED: ferredoxin--NADP reductase leaf-type isozyme chloroplastic [Vitis vinifera]
933	17557	gi|566168605	88.31	13	5	1		44259	hypothetical protein POPTR_0005s00670g [Populus trichocarpa]
566	17616	gi|566182000	88.25	9	5	1	Carbamidomethylation	57819	hypothetical protein POPTR_0008s02740g [Populus trichocarpa]
247	493	gi|225436843	88.14	41	4	1		14570	PREDICTED: histone H2AX [Vitis vinifera]
742	357	gi|731399677	88.08	5	5	1	Carbamidomethylation	102437	PREDICTED: protein argonaute 4A-like [Vitis vinifera]
502	1421	gi|731386809	87.97	8	2	1		41704	PREDICTED: naringenin 2-oxoglutarate 3-dioxygenase-like [Vitis vinifera]
786	16957	gi|224075445	87.89	10	4	1	Carbamidomethylation	47494	transaldolase family protein [Populus trichocarpa]
874	18542	gi|224124892	87.84	13	3	1		22641	rhicadhesin receptor family protein [Populus trichocarpa]

995	1430	gi|359481838	87.75	10	5	1	Carbamidomethylation	54048	PREDICTED: pyruvate kinase cytosolic isozyme [Vitis vinifera]
587	17503	gi|566165056	87.71	11	3	1	Carbamidomethylation	36365	hypothetical protein POPTR_0004s05340g [Populus trichocarpa]
955	17344	gi|224125422	87.71	7	4	2	Carbamidomethylation	56543	hypothetical protein POPTR_0013s03070g [Populus trichocarpa]
1338	2166	gi|359473071	87.04	4	2	2	Carbamidomethylation	65661	PREDICTED: bifunctional purine biosynthesis protein purH isoform X2 [Vitis vinifera]
550	667	gi|225470804	86.78	6	2	1		45224	PREDICTED: phosphoribulokinase chloroplastic [Vitis vinifera]
659	17430	gi|224055647	86.75	9	3	3		32125	succinate dehydrogenase iron-sulfur protein subunit [Populus trichocarpa]
1249	17804	gi|566168414	86.49	7	2	1		34495	quinone oxidoreductase-like family protein [Populus trichocarpa]
752	506	gi|731389917	86.32	6	3	3	Carbamidomethylation	54494	PREDICTED: 3-isopropylmalate dehydratase large subunit isoform X2 [Vitis vinifera]
994	17639	gi|566204313	86.28	5	3	3	Carbamidomethylation	64865	hypothetical protein POPTR_0014s14690g [Populus trichocarpa]
928	16830	gi|566205907	86.15	12	4	4	Carbamidomethylation	29789	40S ribosomal protein S4 [Populus trichocarpa]

1163	1632	gi|225447707	86.13	15	3	3	Carbamidomethylation	27098	PREDICTED: uncharacterized protein LOC100263170 [Vitis vinifera]
701	18416	gi|224071567	85.85	4	2	1		65821	monocopper oxidase precursor family protein [Populus trichocarpa]
1196	765	gi|225443272	85.82	7	4	1	Carbamidomethylation	66288	PREDICTED: D-3-phosphoglycerate dehydrogenase 3 chloroplastic-like [Vitis vinifera]
1062	17168	gi|224090159	85.74	3	3	1		151232	magnesium chelatase subunit family protein [Populus trichocarpa]
707	336	gi|225429870	85.7	8	5	5	Carbamidomethylation	60861	PREDICTED: aspartate--tRNA ligase cytoplasmic [Vitis vinifera]
623	540	gi|225449262	85.5	24	5	1		18173	PREDICTED: 18.1 kDa class I heat shock protein [Vitis vinifera]
579	962	gi|225447723	85.42	3	4	1		109436	PREDICTED: staphylococcal nuclease domain-containing protein 1 [Vitis vinifera]
453	1386	gi|225434203	85.32	8	3	1	Carbamidomethylation	38265	PREDICTED: 2-alkenal reductase (NADP(+)-dependent) [Vitis vinifera]
689	1622	gi|731406178	85.32	5	3	1		62855	PREDICTED: pectinesterase-like [Vitis vinifera]
811	17567	gi|224071575	85.26	6	4	2		65504	stress inducible family protein [Populus trichocarpa]

682	19424	gi|566211207	85.15	8	3	1	Carbamidomethylation; Oxidation (M)	38545	hypothetical protein POPTR_0017s03780g [Populus trichocarpa]
1004	1290	gi|225453909	85.1	13	2	2	Carbamidomethylation	24628	PREDICTED: proteasome subunit beta type-1 [Vitis vinifera]
639	1352	gi|225428908	85.06	6	3	3		53765	PREDICTED: limonoid UDP-glucosyltransferase [Vitis vinifera]
808	17608	gi|224131618	84.85	16	3	3		26206	dienelactone hydrolase family protein [Populus trichocarpa]
1145	654	gi|731415751	84.76	13	2	2	Carbamidomethylation	20904	PREDICTED: 60S ribosomal protein L18-2-like [Vitis vinifera]
953	1092	gi|225456481	84.75	2	2	1		112435	PREDICTED: alpha-mannosidase [Vitis vinifera]
967	135	gi|731402494	84.69	4	4	4		102377	PREDICTED: coatomer subunit beta'-1 [Vitis vinifera]
506	495	gi|731440833	84.64	17	3	3		24955	PREDICTED: 40S ribosomal protein S8 [Vitis vinifera]
880	17546	gi|566200975	84.41	19	4	1		24022	GTP-binding family protein [Populus trichocarpa]
1039	1348	gi|225462545	84.34	12	3	2	Oxidation (M)	27262	PREDICTED: proteasome subunit alpha type-4 [Vitis vinifera]
669	18129	gi|566148278	84.28	8	3	2	Carbamidomethylation	38178	UDP-glucose 4-epimerase family protein [Populus trichocarpa]
760	18078	gi|224073923	84.13	20	4	3		23116	hypothetical protein POPTR_0003s05230g [Populus trichocarpa]

302	851	gi|225450388	84.11	4	3	2	Carbamidomethylation	57174	PREDICTED: beta-glucosidase 12 [Vitis vinifera]
406	17746	gi|566215609	84.1	3	2	1		91834	hypothetical protein POPTR_0018s13110g [Populus trichocarpa]
822	18196	gi|566245692	84.07	11	3	1		25488	hypothetical protein POPTR_0019s15100g [Populus trichocarpa]
714	992	gi|225441912	83.8	8	3	1		30951	PREDICTED: S-formylglutathione hydrolase isoform X4 [Vitis vinifera]
916	551	gi|731440696	83.75	6	4	2		64402	PREDICTED: hsp70-Hsp90 organizing protein 3-like [Vitis vinifera]
787	18942	gi|566197584	83.42	5	2	1		42529	hypothetical protein POPTR_0012s09200g [Populus trichocarpa]
582	18216	gi|566259512	83.18	7	2	1		31098	hypothetical protein POPTR_0030s00360g [Populus trichocarpa]
643	2344	gi|225443154	83.18	18	3	3	Carbamidomethylation; Oxidation (M)	18245	PREDICTED: lamin-like protein [Vitis vinifera]
189	1530	gi|731430493	83.16	4	2	1	Carbamidomethylation	62956	PREDICTED: laccase-14-like [Vitis vinifera]
1152	776	gi|731394453	83.11	9	3	1		46372	PREDICTED: dnaJ protein homolog isoform X2 [Vitis vinifera]
565	17449	gi|224078584	83.05	10	3	1		33150	glyoxalase I homolog family protein [Populus trichocarpa]
540	17984	gi|224078816	83.05	9	3	1		36460	Leucoanthocyanidin reductase family protein [Populus trichocarpa]

1259	17713	gi|224100535	82.98	5	3	1		67584	hypothetical protein POPTR_0008s01000g [Populus trichocarpa]
1000	1629	gi|359490763	82.91	9	3	3	Carbamidomethylation	39503	PREDICTED: caffeic acid 3-O-methyltransferase [Vitis vinifera]
563	17374	gi|566147333	82.77	4	4	1		100058	hypothetical protein POPTR_0001s05320g [Populus trichocarpa]
1193	1496	gi|731392621	82.67	5	2	2		64193	PREDICTED: protein disulfide isomerase-like 1-4 isoform X2 [Vitis vinifera]
583	16888	gi|224131368	82.65	8	3	3		48921	hypothetical protein POPTR_0014s13720g [Populus trichocarpa]
855	1292	gi|731378427	82.47	6	3	3		51975	PREDICTED: uncharacterized protein LOC100263310 [Vitis vinifera]
1069	1487	gi|225460716	82.47	5	2	1	Carbamidomethylation	49588	PREDICTED: dihydrolipoyllysine-residue acetyltransferase component 5 of pyruvate dehydrogenase complex chloroplastic [Vitis vinifera]
798	19380	gi|731407226	82.44	7	2	2	Carbamidomethylation	37934	PREDICTED: 2-alkenal reductase (NADP(+)-dependent) [Vitis vinifera]
961	17127	gi|566149354	82.32	13	3	3		26031	Proteasome subunit alpha type 5-1 family protein [Populus trichocarpa]

530	17242	gi|566182348	82.19	5	3	3	Carbamidomethylation	70505	peptidase M1 family protein [Populus trichocarpa]
900	18013	gi|566174458	82.19	16	3	1	Carbamidomethylation	23355	hypothetical protein POPTR_0006s04560g [Populus trichocarpa]
1007	17769	gi|566192876	82.09	10	3	2	Carbamidomethylation	47035	hypothetical protein POPTR_0010s26170g [Populus trichocarpa]
696	17814	gi|566177370	82.07	7	4	3		60158	DEAD box RNA helicase family protein [Populus trichocarpa]
793	1381	gi|526117942	81.91	6	2	2		37456	beta 1-3 glucanase precursor [Vitis vinifera]
983	2328	gi|225435249	81.89	13	2	2		27570	PREDICTED: proteasome subunit beta type-4 [Vitis vinifera]
755	17545	gi|566169569	81.84	3	3	3	Carbamidomethylation	117048	transport Sec24 family protein [Populus trichocarpa]
447	1281	gi|359494275	81.58	10	3	1		25431	PREDICTED: probable glutathione S-transferase parC [Vitis vinifera]
387	719	gi|225424242	81.56	40	4	1		10611	PREDICTED: 10 kDa chaperonin [Vitis vinifera]
662	17008	gi|566186274	81.42	14	5	1		29153	proliferating cell nuclear antigen family protein [Populus trichocarpa]
968	28895	gi|224111100	81.03	11	3	1	Carbamidomethylation	27203	hypothetical protein POPTR_0010s09180g [Populus trichocarpa]
508	17844	gi|566198902	80.95	26	2	1		15349	hypothetical protein POPTR_0013s01890g [Populus trichocarpa]

1075	17628	gi|566213642	80.91	4	2	1		43635	hypothetical protein POPTR_0018s01390g [Populus trichocarpa]
472	17352	gi|566156897	80.64	6	3	2		46943	Delta-aminolevulinic acid dehydratase family protein [Populus trichocarpa]
944	1323	gi|526117539	80.59	10	2	1	Carbamidomethylation	34687	class I extracellular chitinase precursor [Vitis vinifera]
731	17730	gi|566189053	80.43	6	3	2		51981	hypothetical protein POPTR_0010s04610g [Populus trichocarpa]
1140	17395	gi|224074998	80.31	5	4	4	Carbamidomethylation	100185	hypothetical protein POPTR_0003s13040g [Populus trichocarpa]
575	2506	gi|225465704	80.18	11	2	2		11389	PREDICTED: 60S acidic ribosomal protein P2B [Vitis vinifera]
331	16640	gi|671743230	80.09	6	4	4	Carbamidomethylation	82370	photosystem I P700 apoprotein A2 (chloroplast) [Camellia petelotii]
810	18861	gi|566159684	80.07	4	2	1	Oxidation (M)	72140	hypothetical protein POPTR_0002s23950g [Populus trichocarpa]
1261	18395	gi|566182365	80.06	8	2	2		34377	hypothetical protein POPTR_0008s04660g [Populus trichocarpa]
1178	1844	gi|731439310	79.91	2	1	1		71338	PREDICTED: fumarylacetoacetase isoform X2 [Vitis vinifera]
403	18170	gi|566157284	79.84	4	3	1	Carbamidomethylation	48713	chloroplast nucleoid DNA-binding family protein [Populus trichocarpa]

882	18339	gi|225462348	79.84	8	3	3	Carbamidomethylation	57728	PREDICTED: zinc finger CCCH domain-containing protein 37 isoform X2 [Vitis vinifera]
272	18717	gi|224059156	79.77	18	2	1		14119	Profilin 3 family protein [Populus trichocarpa]
803	18121	gi|566172301	79.68	6	4	2	Carbamidomethylation	54904	disulfide-isomerase family protein [Populus trichocarpa]
1172	854	gi|731402959	79.56	5	2	1		35210	PREDICTED: probable quinone oxidoreductase [Vitis vinifera]
1390	782	gi|225458719	79.47	7	3	2	Carbamidomethylation	63799	PREDICTED: D-3-phosphoglycerate dehydrogenase 3 chloroplastic-like [Vitis vinifera]
589	17620	gi|224058427	79.05	9	3	3		28418	reticulon family protein [Populus trichocarpa]
746	1211	gi|731391365	78.92	4	2	1	Carbamidomethylation	52738	PREDICTED: acetolactate synthase small subunit 2 chloroplastic isoform X2 [Vitis vinifera]
637	866	gi|225425304	78.91	37	3	3	Carbamidomethylation	9611	PREDICTED: 40S ribosomal protein S27-2 [Vitis vinifera]
924	16961	gi|566166593	78.84	4	4	1		122467	hypothetical protein POPTR_0004s14960g [Populus trichocarpa]
999	669	gi|731386337	78.8	19	4	2	Carbamidomethylation	20640	PREDICTED: serine/arginine-rich splicing factor RSZ22 isoform X2 [Vitis vinifera]

552	1269	gi|225459906	78.76	5	2	1		34942	PREDICTED: probable fructokinase-5 [Vitis vinifera]
590	784	gi|225462809	78.69	4	2	2		60994	PREDICTED: 2-hydroxyacyl-CoA lyase [Vitis vinifera]
1037	341	gi|731391907	78.62	6	3	3		67493	PREDICTED: anthranilate synthase alpha subunit 2 chloroplastic isoform X2 [Vitis vinifera]
769	61055	gi|224081483	78.38	15	3	3		15916	hypothetical protein POPTR_0005s10440g [Populus trichocarpa]
998	306	gi|731438745	78.33	5	4	3		92847	PREDICTED: DNA replication licensing factor MCM6 [Vitis vinifera]
356	17645	gi|566146919	78.19	4	3	1		68964	hypothetical protein POPTR_0001s03040g [Populus trichocarpa]
878	1353	gi|225428047	78.05	23	3	2	Carbamidomethylation	17518	PREDICTED: SKP1-like protein 1A isoform X5 [Vitis vinifera]
986	17900	gi|224104697	77.81	16	3	1		20848	lactoylglutathione lyase family protein [Populus trichocarpa]
490	17131	gi|224057882	77.72	3	3	2		89495	outer membrane family protein [Populus trichocarpa]
768	1503	gi|225461001	77.71	7	2	2		46284	PREDICTED: polyadenylate-binding protein RBP45-like [Vitis vinifera]
651	18418	gi|566234529	77.26	29	4	2	Carbamidomethylation	13103	hypothetical protein POPTR_0019s07730g [Populus trichocarpa]
992	18184	gi|224122176	77.17	3	3	2		113456	hypothetical protein POPTR_0012s10830g [Populus trichocarpa]

1157	600	gi|359490767	77.17	11	2	1		23070	PREDICTED: nascent polypeptide-associated complex subunit alpha-like protein 2 [Vitis vinifera]
581	18015	gi|224116326	77.13	9	3	1		36463	Leucoanthocyanidin reductase family protein [Populus trichocarpa]
868	18469	gi|566187699	76.92	8	3	3		36049	ubiquitin fusion degradation UFD1 family protein [Populus trichocarpa]
736	1667	gi|225432306	76.69	30	3	3	Carbamidomethylation	9210	PREDICTED: 40S ribosomal protein S21 [Vitis vinifera]
1026	844	gi|526117645	76.69	2	3	1		153182	magnesium chelatase H subunit [Vitis vinifera]
898	1573	gi|731428130	76.69	12	4	1		49925	PREDICTED: kynurenine--oxoglutarate transaminase 1 [Vitis vinifera]
732	18175	gi|566202597	76.54	17	2	1		16336	hypothetical protein POPTR_0014s04880g [Populus trichocarpa]
141	19801	gi|224093204	76.53	5	2	2	Carbamidomethylation	35946	peroxidase precursor family protein [Populus trichocarpa]
748	17779	gi|224136089	76.2	9	2	2		26956	hypothetical protein POPTR_0015s10490g [Populus trichocarpa]
1255	19537	gi|566201057	75.88	9	3	3		42430	hypothetical protein POPTR_0013s13100g [Populus trichocarpa]
1264	1135	gi|225458305	75.78	8	3	2		62648	PREDICTED: glucose-6-phosphate isomerase cytosolic [Vitis vinifera]

1086	1861	gi|731428311	75.61	4	2	1		51458	PREDICTED: bifunctional aspartate aminotransferase and glutamate/aspartate-prephenate aminotransferase-like [Vitis vinifera]
1317	17838	gi|224072208	75.55	9	2	1	Carbamidomethylation	38044	UDP-glucose 4-epimerase family protein [Populus trichocarpa]
673	1500	gi|225455141	75.43	37	2	1		11333	PREDICTED: protein SPIRAL1-like 1 [Vitis vinifera]
602	17249	gi|566161432	75.37	4	3	1		74324	acyl-CoA oxidase family protein [Populus trichocarpa]
1141	17260	gi|224102365	75.34	7	3	3	Carbamidomethylation	59185	T-complex protein 1 [Populus trichocarpa]
620	604	gi|731401756	75.32	5	2	2		45526	PREDICTED: oligouridylate-binding protein 1B isoform X2 [Vitis vinifera]
909	1267	gi|359494718	75.3	9	3	1		34301	PREDICTED: LOW QUALITY PROTEIN: basic endochitinase [Vitis vinifera]
1104	1995	gi|731412542	75.22	5	3	3		58149	PREDICTED: uncharacterized protein LOC100244432 isoform X4 [Vitis vinifera]
1282	17680	gi|566162135	75.21	10	3	1		27086	hypothetical protein POPTR_0003s11350g [Populus trichocarpa]

621	794	gi|731374652	75.11	6	2	1		58335	PREDICTED: aldehyde dehydrogenase family 2 member B4 mitochondrial [Vitis vinifera]
1465	715	gi|731436956	75.03	7	3	3	Carbamidomethylation	39713	PREDICTED: porphobilinogen deaminase chloroplastic [Vitis vinifera]
1199	17602	gi|566191545	74.79	8	2	1	Carbamidomethylation	40561	hypothetical protein POPTR_0010s18770g [Populus trichocarpa]
840	17497	gi|566205259	74.77	18	4	2	Carbamidomethylation	29312	hypothetical protein POPTR_0015s00690g [Populus trichocarpa]
1204	1643	gi|225452186	74.67	10	3	3		30725	PREDICTED: prohibitin-3 mitochondrial [Vitis vinifera]
1019	2338	gi|359496232	74.59	11	2	2		21717	PREDICTED: probable NAD(P)H dehydrogenase (quinone) FQR1-like 1 [Vitis vinifera]
1051	1439	gi|731428465	74.57	6	2	2		37487	PREDICTED: thylakoid lumenal 29 kDa protein chloroplastic [Vitis vinifera]
384	18371	gi|224116936	74.42	4	2	1		63722	pyruvate decarboxylase family protein [Populus trichocarpa]
690	2306	gi|526117918	74.36	5	1	1		33457	basic endochitinase precursor [Vitis vinifera]

1248	60998	gi|566171779	74.05	3	3	2	Carbamidomethylation	84881	hypothetical protein POPTR_0005s16650g partial [Populus trichocarpa]
456	19091	gi|566214265	73.76	18	2	1		14037	hypothetical protein POPTR_0018s04840g [Populus trichocarpa]
496	18733	gi|224143583	73.6	11	3	3		17416	peroxiredoxin family protein [Populus trichocarpa]
301	753	gi|731391225	73.47	10	3	1		38311	PREDICTED: endochitinase A-like isoform X1 [Vitis vinifera]
818	18849	gi|224065507	73.43	14	1	1		15080	Photosystem I reaction center subunit IV A family protein [Populus trichocarpa]
856	18092	gi|566156270	73.37	3	3	1	Carbamidomethylation	75868	hypothetical protein POPTR_0002s04800g [Populus trichocarpa]
1364	18666	gi|566197993	73.21	8	2	1		29095	hypothetical protein POPTR_0012s11630g [Populus trichocarpa]
1018	18943	gi|224140195	73.13	12	2	2		30031	29 kDa ribonucleoprotein [Populus trichocarpa]
607	2407	gi|359495882	73.06	8	3	1	Carbamidomethylation	39482	PREDICTED: 2-alkenal reductase (NADP(+)-dependent) [Vitis vinifera]
605	903	gi|225453158	72.91	9	3	2	Carbamidomethylation	41234	PREDICTED: probable fructokinase-6 chloroplastic [Vitis vinifera]
813	423	gi|225434133	72.85	6	2	2		40613	PREDICTED: cysteine synthase chloroplastic/chromoplastic [Vitis vinifera]

1366	814	gi|225433414	72.8	8	2	2		36594	PREDICTED: probable voltage-gated potassium channel subunit beta [Vitis vinifera]
829	1201	gi|225454278	72.76	6	2	2		40499	PREDICTED: bifunctional L-3-cyanoalanine synthase/cysteine synthase 1 mitochondrial [Vitis vinifera]
1168	942	gi|225453935	72.68	10	3	3		40128	PREDICTED: 4-hydroxy-tetrahydrodipicolinate synthase chloroplastic [Vitis vinifera]
413	18297	gi|566192144	72.63	6	2	2		36328	hypothetical protein POPTR_0010s22170g [Populus trichocarpa]
522	17341	gi|566238007	72.59	9	2	2	Carbamidomethylation	30925	Chlorophyll a-b binding protein CP26 [Populus trichocarpa]
1349	2125	gi|731415654	72.53	3	2	2		73684	PREDICTED: acyl-CoA-binding domain-containing protein 4-like isoform X2 [Vitis vinifera]
737	18199	gi|566185810	72.16	4	3	1		94037	beta-galactosidase family protein [Populus trichocarpa]
930	18534	gi|224063774	72.11	6	2	1	Carbamidomethylation	55604	3-phosphoshikimate 1-carboxyvinyltransferase family protein [Populus trichocarpa]
1033	17511	gi|566203118	72.03	7	2	2	Carbamidomethylation	27871	phosphomannomutase family protein [Populus trichocarpa]
454	1956	gi|225442434	72	17	2	1		14228	PREDICTED: profilin-1 [Vitis vinifera]

984	18859	gi|224107333	71.99	26	2	2		9716	hypothetical protein POPTR_0010s02580g [Populus trichocarpa]
1038	2373	gi|731384063	71.91	7	3	1		43516	PREDICTED: LL-diaminopimelate aminotransferase chloroplastic isoform X7 [Vitis vinifera]
857	18017	gi|566196102	71.7	10	2	1		24822	hypothetical protein POPTR_0012s00690g [Populus trichocarpa]
850	17506	gi|566243399	71.7	8	2	2		33846	hypothetical protein POPTR_0019s13040g [Populus trichocarpa]
661	18856	gi|224099491	71.5	24	2	1		10451	hypothetical protein POPTR_0008s12950g [Populus trichocarpa]
711	1505	gi|731426023	71.47	30	2	1		12464	PREDICTED: protein SPIRAL1-like 1 isoform X2 [Vitis vinifera]
1134	17479	gi|224097148	71.39	9	4	3		41439	GDP-mannose 4 family protein [Populus trichocarpa]
864	18273	gi|224106686	71.36	10	2	1		24434	elongation factor 1B alpha-subunit 2 family protein [Populus trichocarpa]
1144	18280	gi|566202445	71.36	3	2	2		69760	hypothetical protein POPTR_0014s04320g [Populus trichocarpa]
1046	1933	gi|225455762	71.32	5	3	3	Carbamidomethylation	67078	PREDICTED: probable phenylalanine--tRNA ligase beta subunit [Vitis vinifera]
1092	868	gi|225455106	71.12	7	2	2		33350	PREDICTED: protein BOBBER 1 [Vitis vinifera]

1156	1849	gi|731397296	71.11	5	2	1		44232	PREDICTED: bifunctional aspartate aminotransferase and glutamate/aspartate-prephenate aminotransferase isoform X2 [Vitis vinifera]
1244	18188	gi|566147227	70.85	4	3	3	Carbamidomethylation	110034	hypothetical protein POPTR_0001s04700g [Populus trichocarpa]
1053	1184	gi|731387210	70.82	7	2	1		42062	PREDICTED: splicing factor U2af large subunit B isoform X4 [Vitis vinifera]
1042	28950	gi|359483854	70.8	11	4	4	Carbamidomethylation	49342	PREDICTED: 3-oxoacyl-[acyl-carrier-protein] synthase I chloroplastic [Vitis vinifera]
1643	18052	gi|566163395	70.64	9	2	2		36315	hypothetical protein POPTR_0003s18620g [Populus trichocarpa]
1124	947	gi|225463556	70.49	3	3	1		125024	PREDICTED: protein TOPLESS [Vitis vinifera]
1091	915	gi|225435203	70.39	15	2	2		15652	PREDICTED: 40S ribosomal protein S23-like [Vitis vinifera]
1622	787	gi|225450299	70.31	4	1	1		52389	PREDICTED: citrate synthase mitochondrial [Vitis vinifera]
1017	17675	gi|566198526	70.26	4	3	2		67271	hypothetical protein POPTR_0012s14780g [Populus trichocarpa]
943	28901	gi|566156965	70.22	11	4	1		51266	hypothetical protein POPTR_0002s08650g [Populus trichocarpa]

1303	243	gi|225429750	70.22	2	2	2	Carbamidomethylation	105919	PREDICTED: coatomer subunit beta-1 [Vitis vinifera]
1441	1091	gi|225428916	70.16	8	2	2		39009	PREDICTED: probable cinnamyl alcohol dehydrogenase 1 [Vitis vinifera]
828	744	gi|359497196	70.15	10	2	1		23133	PREDICTED: ras-related protein RABH1e [Vitis vinifera]
756	1216	gi|731434246	70.1	5	3	3	Carbamidomethylation	56111	PREDICTED: NADH dehydrogenase [ubiquinone] flavoprotein 1 mitochondrial isoform X1 [Vitis vinifera]
641	2452	gi|225439785	70.1	8	2	1		26785	PREDICTED: glutathione S-transferase L3 [Vitis vinifera]
985	2329	gi|225435002	70.09	6	1	1	Carbamidomethylation	27396	PREDICTED: osmotin-like protein [Vitis vinifera]
1203	1726	gi|225440169	69.84	5	3	2		57401	PREDICTED: cytochrome P450 98A2 [Vitis vinifera]
1197	1177	gi|225441573	69.7	3	3	3	Carbamidomethylation	107099	PREDICTED: eukaryotic translation initiation factor 3 subunit C [Vitis vinifera]
819	18439	gi|224081475	69.65	5	2	1		58488	hypothetical protein POPTR_0005s10320g [Populus trichocarpa]
1155	2513	gi|731440658	69.64	33	3	1		13203	PREDICTED: ras-related protein RIC1-like partial [Vitis vinifera]

537	19104	gi|224078057	69.29	8	2	2		28150	phosphorylase family protein [Populus trichocarpa]
1132	19116	gi|224078882	69.26	8	2	2	Carbamidomethylation	34470	oxidoreductase family protein [Populus trichocarpa]
1154	17864	gi|566153954	68.71	10	3	3		31003	phosphatase 2C family protein [Populus trichocarpa]
1068	17463	gi|224133304	68.69	3	2	1		60007	hypothetical protein POPTR_0015s07690g [Populus trichocarpa]
816	18329	gi|224120174	68.65	2	2	1		68576	Phosphoglucomutase family protein [Populus trichocarpa]
1090	18064	gi|566149112	68.59	17	3	1		22610	GTP-binding family protein [Populus trichocarpa]
1328	18888	gi|225423849	68.56	4	2	2		65257	PREDICTED: protein decapping 5 [Vitis vinifera]
1003	648	gi|731424733	68.47	1	2	2		241380	PREDICTED: glutamate synthase 1 [NADH] chloroplastic isoform X2 [Vitis vinifera]
936	812	gi|731423236	68.22	5	3	1		65721	PREDICTED: monocopper oxidase-like protein SKU5 [Vitis vinifera]
555	1075	gi|526117894	68.22	7	3	1		36735	anthocyanidin reductase [Vitis vinifera]
1171	2854	gi|731408416	68.14	4	2	1		53964	PREDICTED: serine carboxypeptidase-like 13 [Vitis vinifera]
716	17372	gi|224063299	68.07	5	3	1		67707	hypothetical protein POPTR_0002s10420g [Populus trichocarpa]

1127	683	gi|225456085	68.06	5	3	3		57173	PREDICTED: eukaryotic peptide chain release factor GTP-binding subunit ERF3A [Vitis vinifera]
847	29074	gi|225461628	68.03	4	2	1		45420	PREDICTED: UBP1-associated protein 2C [Vitis vinifera]
774	2011	gi|731437310	67.96	5	2	1	Carbamidomethylation	52411	PREDICTED: acetolactate synthase small subunit 2 chloroplastic [Vitis vinifera]
1101	1570	gi|731427302	67.91	10	3	3		17173	PREDICTED: transcription factor BTF3 homolog 4 [Vitis vinifera]
1143	18312	gi|224082494	67.84	5	2	1		38884	peroxidase precursor family protein [Populus trichocarpa]
1436	597	gi|225441924	67.46	4	1	1	Carbamidomethylation	51126	PREDICTED: eukaryotic translation initiation factor 3 subunit E [Vitis vinifera]
962	2234	gi|225441977	67.35	4	3	1		66737	PREDICTED: pectinesterase [Vitis vinifera]
1169	19371	gi|566184421	67.33	8	3	3		45388	hypothetical protein POPTR_0008s17030g [Populus trichocarpa]
763	1752	gi|225469443	67.27	13	3	1		23539	PREDICTED: uncharacterized protein LOC100266972 [Vitis vinifera]

1008	1358	gi|731409226	66.92	6	3	2		43860	PREDICTED: 3-hydroxyisobutyryl-CoA hydrolase-like protein 5 [Vitis vinifera]
941	18678	gi|566210985	66.8	6	2	2	Carbamidomethylation	51941	glycosyl hydrolase family 17 family protein [Populus trichocarpa]
1350	1372	gi|225462440	66.71	3	2	2		107317	PREDICTED: serine/threonine-protein phosphatase BSL3 [Vitis vinifera]
1158	949	gi|225457152	66.67	4	2	1		54621	PREDICTED: betaine aldehyde dehydrogenase 2 mitochondrial [Vitis vinifera]
1022	2307	gi|225441664	66.62	5	1	1		36307	PREDICTED: uncharacterized protein LOC100255512 [Vitis vinifera]
1241	17094	gi|224065699	66.53	2	1	1		75559	Cell division protein ftsH [Populus trichocarpa]
887	876	gi|225429341	66.39	6	3	3	Carbamidomethylation	44078	PREDICTED: 3-oxo-Delta(4 5)-steroid 5-beta-reductase-like [Vitis vinifera]
1023	18144	gi|224073967	66.16	8	1	1		15188	photosystem I 11K family protein [Populus trichocarpa]
386	914	gi|225440109	66.03	24	3	1		10658	PREDICTED: 10 kDa chaperonin [Vitis vinifera]
572	19089	gi|566149765	65.78	3	1	1		43680	3-isopropylmalate dehydrogenase 2 family protein [Populus trichocarpa]

622	18430	gi|224064818	65.76	4	2	1		47984	hypothetical protein POPTR_0002s22410g [Populus trichocarpa]
1283	19228	gi|224086367	65.69	10	2	1		27400	Proteasome subunit alpha type 4 family protein [Populus trichocarpa]
982	17498	gi|566160463	65.65	7	2	2		49456	hypothetical protein POPTR_0003s02420g [Populus trichocarpa]
1052	2095	gi|225447745	65.59	8	1	1		27216	PREDICTED: chlorophyll a-b binding protein CP24 10A chloroplastic [Vitis vinifera]
1271	18310	gi|224143674	65.5	11	3	3		38385	fructose-1 family protein [Populus trichocarpa]
885	653	gi|225449060	65.3	3	2	2		62143	PREDICTED: protein disulfide isomerase-like 1-4 [Vitis vinifera]
927	16960	gi|566211039	65.2	5	4	4		124467	DegP protease family protein [Populus trichocarpa]
1280	18523	gi|566146801	65.15	8	3	1		30343	Mitochondrial carnitine/acylcarnitine carrier-like family protein [Populus trichocarpa]
1100	2594	gi|731382912	65.1	3	2	2		81725	PREDICTED: subtilisin-like protease [Vitis vinifera]
971	2314	gi|225452570	65.1	12	1	1	Carbamidomethylation	16225	PREDICTED: ferredoxin-thioredoxin reductase catalytic chain chloroplastic [Vitis vinifera]
1916	1362	gi|225440230	64.97	7	1	1		29238	PREDICTED: dihydrofolate reductase [Vitis vinifera]

728	1063	gi|359491801	64.88	4	3	3		83367	PREDICTED: dolichyl-diphosphooligosaccharide--protein glycosyltransferase subunit STT3B [Vitis vinifera]
890	577	gi|731438110	64.86	5	2	2	Carbamidomethylation	88481	PREDICTED: probable cytosolic oligopeptidase A [Vitis vinifera]
1009	18299	gi|731436460	64.85	3	4	4	Carbamidomethylation	123310	PREDICTED: leucine--tRNA ligase cytoplasmic-like [Vitis vinifera]
1486	2627	gi|731393556	64.84	8	2	2		29837	PREDICTED: rhodanese-like/PpiC domain-containing protein 12 isoform X2 [Vitis vinifera]
970	1354	gi|731430037	64.61	4	1	1	Carbamidomethylation	43016	PREDICTED: probable cinnamyl alcohol dehydrogenase 6 [Vitis vinifera]
1256	2753	gi|225429582	64.56	3	2	2		98913	PREDICTED: aminopeptidase M1 [Vitis vinifera]
893	19431	gi|566151403	64.55	28	2	2		8809	hypothetical protein POPTR_0001s28090g [Populus trichocarpa]
1308	864	gi|731439997	64.52	14	3	2	Carbamidomethylation	33522	PREDICTED: phosphoglycolate phosphatase 1B chloroplastic [Vitis vinifera]
951	17240	gi|566202791	64.41	4	2	1		59276	t-complex polypeptide 1 family protein [Populus trichocarpa]

1273	2502	gi|225424908	64.31	8	3	2		29426	PREDICTED: mitochondrial outer membrane protein porin of 36 kDa [Vitis vinifera]
1013	920	gi|225436438	64.17	19	2	2		10607	PREDICTED: cyclin-dependent kinases regulatory subunit 1 [Vitis vinifera]
1170	2442	gi|225429295	64.16	8	2	2		25371	PREDICTED: uncharacterized protein LOC100254416 [Vitis vinifera]
1064	1010	gi|225451999	63.95	5	2	2		59112	PREDICTED: T-complex protein 1 subunit zeta [Vitis vinifera]
1186	792	gi|225465764	63.81	8	3	3		43403	PREDICTED: UDP-D-apiose/UDP-D-xylose synthase 2 [Vitis vinifera]
1218	18384	gi|224146080	63.67	14	2	2		17003	hypothetical protein POPTR_0019s05840g [Populus trichocarpa]
1056	61273	gi|225457387	63.62	8	1	1		26287	PREDICTED: expansin-A1 [Vitis vinifera]
660	18810	gi|566209344	63.57	7	2	1		27361	In2-1 family protein [Populus trichocarpa]
1257	1167	gi|225457034	63.5	4	2	2	Carbamidomethylation	51176	PREDICTED: 1-deoxy-D-xylulose 5-phosphate reductoisomerase chloroplastic [Vitis vinifera]
902	17304	gi|224108085	63.32	15	2	2		16429	40S ribosomal protein S16 [Populus trichocarpa]

799	1233	gi|225448904	63.2	5	1	1		48063	PREDICTED: malate dehydrogenase [NADP] chloroplastic [Vitis vinifera]
1047	17005	gi|566146555	63.08	12	3	3		23812	60S ribosomal protein L13 [Populus trichocarpa]
1129	1837	gi|225428043	63.02	5	2	1	Carbamidomethylation	49886	PREDICTED: uncharacterized protein LOC100257179 isoform X1 [Vitis vinifera]
1284	18539	gi|224092348	62.95	18	2	2	Carbamidomethylation	10699	hypothetical protein POPTR_0006s25990g [Populus trichocarpa]
1297	1237	gi|225446509	62.87	3	2	2		77096	PREDICTED: acyl-coenzyme A oxidase 3 peroxisomal [Vitis vinifera]
1467	19771	gi|224106191	62.83	6	2	2	Carbamidomethylation	35572	cinnamyl-alcohol dehydrogenase family protein [Populus trichocarpa]
576	61001	gi|731437331	62.8	17	3	1		17820	PREDICTED: 60S ribosomal protein L27a-3 [Vitis vinifera]
954	17743	gi|566175322	62.7	9	2	2		35273	aldo/keto reductase family protein [Populus trichocarpa]
765	17207	gi|566151109	62.29	6	3	1	Carbamidomethylation	37403	hypothetical protein POPTR_0001s26400g [Populus trichocarpa]
1066	737	gi|225424719	62.06	3	2	2	Carbamidomethylation	78467	PREDICTED: threonine--tRNA ligase mitochondrial [Vitis vinifera]

739	1743	gi|225441809	62.06	29	3	3		8917	PREDICTED: uncharacterized protein LOC100245204 isoform X3 [Vitis vinifera]
1418	18109	gi|566168240	61.86	2	1	1	Carbamidomethylation	55680	hypothetical protein POPTR_0004s23390g [Populus trichocarpa]
888	18288	gi|224099811	61.74	8	2	1	Oxidation (M)	34198	O-acetylserine (thiol)lyase family protein [Populus trichocarpa]
1265	19527	gi|566204649	61.64	6	2	2		42787	hypothetical protein POPTR_0014s16700g [Populus trichocarpa]
503	1639	gi|526118028	61.4	7	3	2		30741	aquaporin-like [Vitis vinifera]
820	774	gi|225444063	61.31	6	2	2	Carbamidomethylation	44423	PREDICTED: obg-like ATPase 1 [Vitis vinifera]
906	1750	gi|225447488	61.3	4	2	1		63568	PREDICTED: uncharacterized protein LOC100266500 [Vitis vinifera]
1471	1547	gi|225454994	61.18	9	2	1		28211	PREDICTED: peptide methionine sulfoxide reductase A1-like [Vitis vinifera]
1028	1287	gi|225460991	61.02	2	1	1		86153	PREDICTED: heat shock 70 kDa protein 16 [Vitis vinifera]
1040	17909	gi|224133898	60.67	8	2	2	Carbamidomethylation	53810	aspartyl aminopeptidase family protein [Populus trichocarpa]

1247	609	gi|731392506	60.66	3	3	3	Carbamidomethylation	129324	PREDICTED: carbamoyl-phosphate synthase large chain chloroplastic [Vitis vinifera]
931	29005	gi|224084784	60.63	3	2	1		61214	glycine hydroxymethyltransferase family protein [Populus trichocarpa]
1365	20409	gi|566215019	60.53	10	2	2	Oxidation (M)	36357	hypothetical protein POPTR_0018s09500g [Populus trichocarpa]
1676	18611	gi|224077508	60.45	6	2	2		39890	putative protein phosphatase [Populus trichocarpa]
353	18536	gi|566165881	60.39	6	2	2		27272	mangrin family protein [Populus trichocarpa]
783	19856	gi|566210416	60.36	6	2	1	Carbamidomethylation	38764	hypothetical protein POPTR_0017s00310g [Populus trichocarpa]
1276	17918	gi|224063643	60.36	14	2	2	Carbamidomethylation	17863	40S ribosomal protein S11 [Populus trichocarpa]
1021	19686	gi|224141787	60.29	6	1	1		27379	20S proteasome beta subunit G1 family protein [Populus trichocarpa]
1118	1382	gi|359489656	60.28	6	2	2		52031	PREDICTED: probable polygalacturonase [Vitis vinifera]
1867	18737	gi|566200149	60.26	4	1	1		47169	eIF4-gamma/eIF5/eIF2-epsilon domain-containing family protein [Populus trichocarpa]
1044	19744	gi|224137260	59.75	12	1	1		11459	hypothetical protein POPTR_0016s01100g [Populus trichocarpa]

719	1615	gi|359497056	59.7	5	2	1		35941	PREDICTED: peroxidase 10-like [Vitis vinifera]
1329	1196	gi|731405060	59.68	4	2	2		73829	PREDICTED: phospho-N-acetylmuramoyl-pentapeptide-transferase homolog isoform X2 [Vitis vinifera]
1469	1845	gi|225434277	59.49	8	2	2		22623	PREDICTED: CBS domain-containing protein CBSX3 mitochondrial [Vitis vinifera]
1159	17641	gi|566204499	59.29	7	2	1		47320	thioredoxin family protein [Populus trichocarpa]
1675	18515	gi|566245088	59.26	6	2	1		33717	Xyloglucan endotransglucosylase/hydrolase protein 9 precursor [Populus trichocarpa]
634	1144	gi|731392975	59.13	3	3	1		103818	PREDICTED: linoleate 13S-lipoxygenase 2-1 chloroplastic-like [Vitis vinifera]
1167	20226	gi|566161648	59.09	5	1	1		17801	glycine cleavage system protein H precursor [Populus trichocarpa]
757	19311	gi|566172679	59.02	2	1	1		63027	hypothetical protein POPTR_0005s22250g [Populus trichocarpa]
1088	720	gi|359476926	58.91	8	1	1	Carbamidomethylation	18454	PREDICTED: photosystem I reaction center subunit N chloroplastic [Vitis vinifera]

644	17927	gi|566171321	58.53	9	2	1		33667	pfkB-type carbohydrate kinase family protein [Populus trichocarpa]
1220	19808	gi|566160273	58.39	6	1	1	Carbamidomethylation	17554	hypothetical protein POPTR_0003s01440g [Populus trichocarpa]
1135	17596	gi|566171781	58.38	2	2	1		84742	hypothetical protein POPTR_0005s16660g [Populus trichocarpa]
996	1894	gi|359480888	58.19	4	1	1		39762	PREDICTED: RNA-binding protein Musashi homolog 2 [Vitis vinifera]
987	1542	gi|225458237	58.18	5	1	1	Carbamidomethylation	29571	PREDICTED: gamma carbonic anhydrase 1 mitochondrial [Vitis vinifera]
1085	61000	gi|566173396	58.02	7	3	2	Carbamidomethylation	33622	hypothetical protein POPTR_0005s26700g [Populus trichocarpa]
1054	19960	gi|566150184	57.97	8	2	2		23580	hypothetical protein POPTR_0001s21460g [Populus trichocarpa]
1130	1156	gi|225436289	57.96	4	1	1		43082	PREDICTED: probable aldo-keto reductase 2 isoform X1 [Vitis vinifera]
1288	61290	gi|224125776	57.78	7	2	1		23594	hypothetical protein POPTR_0013s04850g [Populus trichocarpa]
1267	17452	gi|566147835	57.69	6	1	1		26392	hypothetical protein POPTR_0001s07920g partial [Populus trichocarpa]
1277	18363	gi|566206536	57.37	4	1	1	Carbamidomethylation	32775	hypothetical protein POPTR_0015s080101g [Populus trichocarpa]

1351	17777	gi|566153407	57.27	6	2	2		42007	ferredoxin-NADP reductase family protein [Populus trichocarpa]
851	2197	gi|526118093	57.09	8	1	1	Carbamidomethylation	22511	type II peroxiredoxin E [Vitis vinifera]
1081	837	gi|731425891	57.08	8	2	2		31554	PREDICTED: hypersensitive-induced response protein 1 isoform X2 [Vitis vinifera]
914	17902	gi|566176831	57.05	4	3	3		85828	hypothetical protein POPTR_0006s17890g [Populus trichocarpa]
837	2406	gi|225434195	56.76	6	2	1	Carbamidomethylation; Oxidation (M)	37907	PREDICTED: 2-alkenal reductase (NADP(+)-dependent) [Vitis vinifera]
976	60999	gi|731403637	56.75	4	3	2		88717	PREDICTED: nudix hydrolase 3 [Vitis vinifera]
1449	2180	gi|225428049	56.74	7	3	3		42284	PREDICTED: glycerate dehydrogenase HPR peroxisomal [Vitis vinifera]
593	2865	gi|359479995	56.72	2	2	1		61649	PREDICTED: pectinesterase-like [Vitis vinifera]
1063	21187	gi|225452785	56.72	8	2	2		29509	PREDICTED: NAP1-related protein 2 [Vitis vinifera]
1309	18805	gi|566162337	56.51	5	1	1		26306	glutathione peroxidase family protein [Populus trichocarpa]
617	1101	gi|225434062	56.44	6	2	1		28430	PREDICTED: chitinase 5-like [Vitis vinifera]

817	19480	gi|224112341	56.22	3	1	1		46306	latex abundant family protein [Populus trichocarpa]
604	970	gi|731413121	56.15	9	1	1		15960	PREDICTED: actin-depolymerizing factor 2-like isoform X2 [Vitis vinifera]
1077	28951	gi|566215969	56.14	4	2	1	Carbamidomethylation	46998	hypothetical protein POPTR_0018s14920g [Populus trichocarpa]
959	1851	gi|225439145	56.1	5	2	2		37191	PREDICTED: grpE protein homolog mitochondrial isoform X1 [Vitis vinifera]
989	17293	gi|566206565	55.74	7	2	2	Carbamidomethylation	39138	hypothetical protein POPTR_0015s08180g [Populus trichocarpa]
977	1688	gi|359479353	55.56	18	3	1		21048	PREDICTED: lactoylglutathione lyase isoform X2 [Vitis vinifera]
1198	17093	gi|224067984	55.31	3	2	2		68220	dynamin-like protein B [Populus trichocarpa]
1361	6864	gi|359475352	55.28	3	1	1		49145	PREDICTED: uncharacterized protein At4g06744 [Vitis vinifera]
1032	686	gi|225429234	55.27	12	3	3		34319	PREDICTED: mitochondrial import receptor subunit TOM40-1 [Vitis vinifera]
1270	19350	gi|224073782	55.24	9	1	1	Carbamidomethylation	21868	proline-rich family protein [Populus trichocarpa]
876	487	gi|225433247	55.22	26	3	3	Carbamidomethylation	15031	PREDICTED: 60S ribosomal protein L23 [Vitis vinifera]

1656	18326	gi|224109088	55.22	2	1	1		57503	SUCCINIC SEMIALDEHYDE DEHYDROGENASE family protein [Populus trichocarpa]
1488	19481	gi|224122412	55.08	7	1	1		23172	cytochrome b5 domain-containing family protein [Populus trichocarpa]
1353	2552	gi|731393274	55.02	2	2	2		76795	PREDICTED: uncharacterized protein LOC100253534 isoform X2 [Vitis vinifera]
650	1307	gi|225463506	54.89	7	2	2		38294	PREDICTED: fructose-bisphosphate aldolase cytoplasmic isozyme 1 [Vitis vinifera]
858	29364	gi|359478417	54.68	3	2	1		58240	PREDICTED: beta-glucosidase 12-like [Vitis vinifera]
1813	61026	gi|731393292	54.53	2	1	1		72077	PREDICTED: glucosidase 2 subunit beta isoform X2 [Vitis vinifera]
1360	19030	gi|566167266	54.5	4	1	1		37830	hypothetical protein POPTR_0004s18610g [Populus trichocarpa]
860	2356	gi|359497418	54.13	18	2	2		11555	PREDICTED: mitochondrial pyruvate carrier 2-like partial [Vitis vinifera]
846	17778	gi|566199576	54.06	3	3	3	Carbamidomethylation	123531	hypothetical protein POPTR_0013s05320g [Populus trichocarpa]

1306	29225	gi|731410214	53.73	2	1	1		66761	PREDICTED: glucose-6-phosphate 1-dehydrogenase chloroplastic-like [Vitis vinifera]
973	427	gi|731399148	53.68	11	1	1		14802	PREDICTED: 40S ribosomal protein S15a [Vitis vinifera]
1094	18241	gi|566146307	53.67	4	2	1		66102	monocopper oxidase precursor family protein [Populus trichocarpa]
978	463	gi|731398611	53.52	6	3	3		43372	PREDICTED: proliferation-associated protein 2G4 [Vitis vinifera]
1098	18265	gi|566189013	53.46	5	1	1		30317	glycine-rich family protein [Populus trichocarpa]
1188	1772	gi|225451677	53.45	1	1	1		108901	PREDICTED: staphylococcal nuclease domain-containing protein 1 [Vitis vinifera]
1487	5205	gi|359482448	53.4	2	2	2	Carbamidomethylation	83440	PREDICTED: subtilisin-like protease [Vitis vinifera]
861	18691	gi|671743260	53.36	9	2	2		24165	cytochrome b6 (chloroplast) [Camellia petelotii]
1647	17947	gi|224063787	53.32	6	1	1		17073	ribosomal protein S13 [Populus trichocarpa]
1354	19087	gi|566258212	53.22	7	1	1		24279	hypothetical protein POPTR_0115s00200g partial [Populus trichocarpa]
839	19582	gi|224062109	53.13	3	1	1		38699	dihydroflavonol reductase family protein [Populus trichocarpa]

1682	2403	gi|225470816	52.88	23	1	1		5428	PREDICTED: wound-induced basic protein [Vitis vinifera]
1606	61058	gi|225459324	52.75	3	1	1		31418	PREDICTED: uncharacterized protein LOC100260886 isoform X2 [Vitis vinifera]
1111	2871	gi|225455386	52.7	3	2	1		65593	PREDICTED: probable pectinesterase/pectinesterase inhibitor 34 [Vitis vinifera]
1174	2895	gi|359490940	52.4	4	2	1	Carbamidomethylation	48807	PREDICTED: actin-related protein 4 [Vitis vinifera]
1678	362	gi|225464186	52.29	4	2	2		49585	PREDICTED: 26S proteasome regulatory subunit 4 homolog B [Vitis vinifera]
1183	18756	gi|566146785	52.2	3	1	1		43851	UBIQUITIN-SPECIFIC PROTEASE 20 family protein [Populus trichocarpa]
1034	16800	gi|224129102	52.19	5	2	2	Carbamidomethylation	42388	hypothetical protein POPTR_0014s15960g [Populus trichocarpa]
1651	29056	gi|224069802	52.19	5	1	1	Carbamidomethylation	30843	Ni-binding urease accessory protein UreG [Populus trichocarpa]
1136	16899	gi|566190691	52.14	3	1	1	Carbamidomethylation	65588	Ser/Thr specific protein phosphatase 2A A regulatory subunit alpha isoform [Populus trichocarpa]

679	379	gi|225442192	52.13	8	2	2		21908	PREDICTED: 60S ribosomal protein L9 [Vitis vinifera]
1175	1946	gi|225440849	52.07	19	1	1		8952	PREDICTED: probable small nuclear ribonucleoprotein G [Vitis vinifera]
1593	18446	gi|224064850	52	8	2	1		28407	chlorophyll a/b-binding family protein [Populus trichocarpa]
1173	2259	gi|225431922	51.88	19	2	2	Carbamidomethylation	13225	PREDICTED: DNA-directed RNA polymerases II IV and V subunit 9A [Vitis vinifera]
958	19547	gi|566190820	51.87	4	4	1		63045	hypothetical protein POPTR_0010s14520g [Populus trichocarpa]
1913	2173	gi|731434735	51.84	14	1	1	Carbamidomethylation	10306	PREDICTED: LOW QUALITY PROTEIN: costars family protein [Vitis vinifera]
603	1422	gi|731439999	51.54	2	2	1	Carbamidomethylation	80675	PREDICTED: primary amine oxidase-like [Vitis vinifera]
1252	17523	gi|224107106	51.53	2	1	1		79571	MULTIFUNCTIONAL family protein [Populus trichocarpa]
1281	18074	gi|566161702	51.41	8	3	1		30568	Mitochondrial carnitine/acylcarnitine carrier-like family protein [Populus trichocarpa]
1368	1903	gi|225440898	51.37	5	2	2		51575	PREDICTED: cell division protein FtsZ homolog 2-1 chloroplastic [Vitis vinifera]

678	18132	gi|566151049	51.27	6	2	2		26982	hypothetical protein POPTR_0001s26020g [Populus trichocarpa]
2072	61174	gi|731412260	51.15	4	1	1	Carbamidomethylation	31064	PREDICTED: hydroxyacylglutathione hydrolase cytoplasmic [Vitis vinifera]
1371	1617	gi|225429460	51.13	15	2	2		14040	PREDICTED: uncharacterized protein LOC100267911 [Vitis vinifera]
1272	419	gi|731396853	51.12	12	3	3	Carbamidomethylation	28310	PREDICTED: 40S ribosomal protein S6 isoform X1 [Vitis vinifera]
1142	18105	gi|224145917	50.92	3	2	2		44521	Uroporphyrinogen decarboxylase family protein [Populus trichocarpa]
1078	18775	gi|566181200	50.9	2	1	1		55243	serine carboxypeptidase S28 family protein [Populus trichocarpa]
1683	1309	gi|731398937	50.63	11	1	1	Carbamidomethylation	21183	PREDICTED: actin-depolymerizing factor 1-like [Vitis vinifera]
1274	17648	gi|566184227	49.94	4	2	2	Carbamidomethylation	64966	pyruvate phosphotransferase family protein [Populus trichocarpa]
1453	18565	gi|731375306	49.83	2	1	1		65748	PREDICTED: delta(24)-sterol reductase [Vitis vinifera]
1219	928	gi|225445670	49.73	6	2	1		24997	PREDICTED: proteasome subunit beta type-6 [Vitis vinifera]

1485	18748	gi|566195800	49.72	5	3	1		48359	hypothetical protein POPTR_0011s163102g partial [Populus trichocarpa]
794	18394	gi|566200465	49.66	6	1	1		19556	hypothetical protein POPTR_0013s10300g partial [Populus trichocarpa]
2028	2444	gi|731438661	49.66	4	1	1		32525	PREDICTED: uncharacterized protein LOC104878720 [Vitis vinifera]
1232	623	gi|731385630	49.61	3	2	2		99551	PREDICTED: heat shock 70 kDa protein 17 [Vitis vinifera]
1684	2348	gi|225463408	49.59	10	1	1		14802	PREDICTED: DNA-binding protein DDB_G0278111 [Vitis vinifera]
1367	18956	gi|566162026	49.54	9	2	2	Carbamidomethylation	27183	hypothetical protein POPTR_0003s10720g [Populus trichocarpa]
862	18055	gi|566207899	49.52	6	1	1		23136	hypothetical protein POPTR_0016s00400g [Populus trichocarpa]
1285	61101	gi|566151634	49.46	2	1	1	Carbamidomethylation	50974	hypothetical protein POPTR_0001s29250g [Populus trichocarpa]
401	43802	gi|224086681	49.41	2	1	1		50649	RNA recognition motif-containing family protein [Populus trichocarpa]
1176	2477	gi|731383208	49.38	4	1	1		29098	PREDICTED: heterodimeric geranylgeranyl pyrophosphate synthase small subunit chloroplastic-like [Vitis vinifera]

1392	61182	gi|224085575	49.26	5	1	1		28237	NADH-ubiquinone oxidoreductase 24 kDa subunit family protein [Populus trichocarpa]
1014	18054	gi|224068558	49.22	4	1	1		32725	NUCLEAR ENCODED CLP PROTEASE 1 family protein [Populus trichocarpa]
1057	18864	gi|731383454	49.04	4	1	1		43734	PREDICTED: probable N-acetyl-gamma-glutamyl-phosphate reductase chloroplastic [Vitis vinifera]
1466	964	gi|225438529	48.92	11	2	2		27683	PREDICTED: probable ATP synthase 24 kDa subunit mitochondrial [Vitis vinifera]
1160	2400	gi|225434808	48.9	3	1	1		32777	PREDICTED: eukaryotic translation initiation factor 3 subunit G-B [Vitis vinifera]
1024	901	gi|359493854	48.84	6	1	1		20058	PREDICTED: thioredoxin M4 chloroplastic [Vitis vinifera]
1112	18794	gi|224066629	48.7	8	1	1		16281	thioredoxin family protein [Populus trichocarpa]
1076	17434	gi|566179312	48.64	3	2	1		92290	hypothetical protein POPTR_0007s02960g [Populus trichocarpa]
642	2533	gi|359494249	48.39	4	1	1		21360	PREDICTED: photosystem I reaction center subunit V chloroplastic [Vitis vinifera]

889	1613	gi|731375703	48.35	7	2	1	Oxidation (M)	35679	PREDICTED: lignin-forming anionic peroxidase-like [Vitis vinifera]
1109	946	gi|225429638	48.33	10	2	2	Carbamidomethylation	39454	PREDICTED: omega-amidase chloroplastic [Vitis vinifera]
1574	471	gi|225433575	48.24	2	2	1		105215	PREDICTED: probable glucan 1 3-alpha-glucosidase [Vitis vinifera]
1456	61208	gi|224109620	47.84	4	1	1		24252	chalcone isomerase family protein [Populus trichocarpa]
1489	1120	gi|225465441	47.83	12	1	1		14675	PREDICTED: cytochrome b5 [Vitis vinifera]
1072	1842	gi|225447176	47.75	6	3	2		46608	PREDICTED: protein disulfide isomerase-like 2-3 [Vitis vinifera]
1650	1703	gi|731376062	47.73	2	1	1	Carbamidomethylation	83343	PREDICTED: urease isoform X2 [Vitis vinifera]
946	30077	gi|566214618	47.68	7	1	1	Carbamidomethylation	13633	hypothetical protein POPTR_0018s06830g [Populus trichocarpa]
1868	1616	gi|225426330	47.61	5	1	1		34913	PREDICTED: 26S proteasome non-ATPase regulatory subunit 7 homolog A [Vitis vinifera]
1332	18228	gi|224065629	47.55	9	1	1		16418	CP12 domain-containing family protein [Populus trichocarpa]
1313	2104	gi|225456602	47.53	6	1	1		23813	PREDICTED: germin-like protein subfamily T member 2 [Vitis vinifera]

1679	18377	gi|566172753	47.41	4	2	2		55215	pleckstrin homology domain-containing family protein [Populus trichocarpa]
2128	61276	gi|225461673	47.37	2	1	1		68443	PREDICTED: peptide-N4-(N-acetyl-beta-glucosaminyl)asparagine amidase A-like [Vitis vinifera]
1491	1626	gi|359482440	47.36	2	1	1		57853	PREDICTED: alanine aminotransferase 2 [Vitis vinifera]
1108	1268	gi|225440238	47.31	6	2	2		27191	PREDICTED: proteasome subunit alpha type-3 [Vitis vinifera]
1435	28924	gi|566179175	47.26	1	1	1		134532	isoleucyl-tRNA synthetase family protein [Populus trichocarpa]
929	1104	gi|731435142	47.24	2	2	1		84036	PREDICTED: protein ROOT HAIR DEFECTIVE 3 homolog 2 isoform X2 [Vitis vinifera]
1240	763	gi|225433440	47.12	5	2	2		53869	PREDICTED: glutathione reductase cytosolic [Vitis vinifera]
1624	2000	gi|731418793	47.1	2	1	1		59155	PREDICTED: glucose-6-phosphate 1-dehydrogenase cytoplasmic isoform [Vitis vinifera]
1286	20021	gi|566184796	47.03	2	1	1		58710	hypothetical protein POPTR_0008s18930g [Populus trichocarpa]

1424	1387	gi|225427288	46.95	4	3	2		68795	PREDICTED: dynamin-related protein 1E [Vitis vinifera]
1250	17393	gi|566151113	46.8	1	1	1		76209	hypothetical protein POPTR_0001s26420g [Populus trichocarpa]
1165	18872	gi|224103201	46.68	3	2	1		59749	C2 domain-containing family protein [Populus trichocarpa]
1089	5615	gi|225470242	46.66	6	1	1		16680	PREDICTED: uncharacterized protein At5g48480 [Vitis vinifera]
1960	2091	gi|225450155	46.62	4	1	1		26703	PREDICTED: thylakoid lumenal 19 kDa protein chloroplastic [Vitis vinifera]
1645	19363	gi|225460394	46.61	2	2	2		67755	PREDICTED: patellin-3 [Vitis vinifera]
852	19883	gi|566189043	46.56	4	1	1	Carbamidomethylation	36173	Peroxidase 6 precursor family protein [Populus trichocarpa]
1474	2099	gi|225426826	46.5	4	1	1		36763	PREDICTED: replication factor C subunit 2 [Vitis vinifera]
741	351	gi|225457231	46.49	3	2	1		56145	PREDICTED: flavonoid 3'-monooxygenase [Vitis vinifera]
638	17884	gi|224066487	46.37	5	1	1		17636	hypothetical protein POPTR_0002s05220g [Populus trichocarpa]
1331	19816	gi|566197446	46.36	3	1	1		44436	hypothetical protein POPTR_0012s08440g [Populus trichocarpa]

1653	18022	gi|224112819	46.35	12	1	1	Carbamidomethylation	16047	actin-depolymerizing factor family protein [Populus trichocarpa]
1110	33319	gi|224062595	46.32	6	2	2		28191	photosystem II oxygen-evolving complex protein 2 precursor [Populus trichocarpa]
1147	18882	gi|224075096	46.25	1	1	1		73889	acyl-coA synthetase family protein [Populus trichocarpa]
1269	1090	gi|225465997	46.24	3	1	1		48382	PREDICTED: WD-40 repeat-containing protein MSI1 [Vitis vinifera]
1322	18255	gi|566174566	46.06	5	2	2		68630	hypothetical protein POPTR_0006s05130g [Populus trichocarpa]
1685	20127	gi|225435931	45.59	7	1	1		13713	PREDICTED: cysteine proteinase inhibitor A [Vitis vinifera]
1605	3107	gi|225450219	45.47	7	2	2		26900	PREDICTED: 30S ribosomal protein 2 chloroplastic [Vitis vinifera]
1490	29317	gi|225426084	45.35	8	1	1		20541	PREDICTED: disease resistance response protein 206 [Vitis vinifera]
937	19812	gi|566163926	45.33	6	1	1		21587	hypothetical protein POPTR_0003s21460g [Populus trichocarpa]
1148	1371	gi|225469850	45.32	4	1	1		39160	PREDICTED: uncharacterized protein DDB_G0288133 [Vitis vinifera]

1221	2524	gi|225452110	45.16	10	1	1	Carbamidomethylation	9858	PREDICTED: non-specific lipid-transfer protein 2-like [Vitis vinifera]
764	19565	gi|566199277	45.15	4	1	1		38735	hypothetical protein POPTR_0013s03750g [Populus trichocarpa]
925	28907	gi|224097915	45.15	6	2	1		30342	plasma membrane intrinsic protein 1-1 [Populus trichocarpa]
1492	61278	gi|566214298	45.1	13	1	1		8219	hypothetical protein POPTR_0018s05030g partial [Populus trichocarpa]
1428	1093	gi|225438067	45	4	1	1		45402	PREDICTED: succinyl-CoA ligase [ADP-forming] subunit beta mitochondrial [Vitis vinifera]
1251	1139	gi|359484892	44.97	3	2	1		86995	PREDICTED: eukaryotic translation initiation factor isoform 4G-1 [Vitis vinifera]
1355	17276	gi|566170117	44.91	3	1	1		48844	hypothetical protein POPTR_0005s08050g [Populus trichocarpa]
1369	17911	gi|566203837	44.88	5	2	1		39770	DNAJ heat shock family protein [Populus trichocarpa]
1594	2785	gi|731408402	44.88	3	1	1		29415	PREDICTED: (DL)-glycerol-3-phosphatase 2 [Vitis vinifera]
2030	2203	gi|731387671	44.81	4	1	1	Carbamidomethylation	39674	PREDICTED: thioredoxin reductase NTRB-like [Vitis vinifera]

2031	61167	gi|566187811	44.1	5	1	1	Carbamidomethylation	16218	hypothetical protein POPTR_0009s13460g [Populus trichocarpa]
1096	2775	gi|731436245	44.04	1	1	1		66120	PREDICTED: putative laccase-9 [Vitis vinifera]
749	768	gi|359483250	43.99	6	2	1		28691	PREDICTED: LOW QUALITY PROTEIN: 60S ribosomal protein L7-2 [Vitis vinifera]
1048	18261	gi|224109256	43.96	6	2	2		29460	ferritin 2 precursor family protein [Populus trichocarpa]
1213	19953	gi|566205586	43.92	4	1	1		33515	hypothetical protein POPTR_0015s03360g [Populus trichocarpa]
1212	1811	gi|225428031	43.9	4	2	2		45128	PREDICTED: WD-40 repeat-containing protein MSI3 [Vitis vinifera]
1620	61007	gi|225452430	43.88	8	2	2		19663	PREDICTED: 50S ribosomal protein L12 chloroplastic [Vitis vinifera]
317	19403	gi|566166342	43.87	1	1	1		95030	hypothetical protein POPTR_0004s13420g partial [Populus trichocarpa]
1372	20605	gi|225467859	43.86	3	1	1	Carbamidomethylation	33928	PREDICTED: chlorophyllase-1 [Vitis vinifera]
1352	17693	gi|224091819	43.68	3	2	1		88349	MIF4G domain-containing family protein [Populus trichocarpa]
1215	17528	gi|566162704	43.62	8	1	1		18629	hypothetical protein POPTR_0003s14870g [Populus trichocarpa]

1316	17396	gi|224119992	43.61	2	2	2	Carbamidomethylation	143158	hypothetical protein POPTR_0012s13100g [Populus trichocarpa]
1648	18006	gi|224141351	43.6	2	1	1		68914	Structure-specific recognition protein 1 [Populus trichocarpa]
1222	17990	gi|224072588	43.58	3	1	1	Carbamidomethylation	30737	hypothetical protein POPTR_0003s17100g partial [Populus trichocarpa]
1403	6489	gi|225432798	43.47	3	1	1		38396	PREDICTED: perakine reductase [Vitis vinifera]
1102	2189	gi|526118301	43.37	4	1	1		41463	cysteine protease precursor [Vitis vinifera]
1680	18830	gi|566214901	43.31	5	1	1		35399	hypothetical protein POPTR_0018s08850g [Populus trichocarpa]
1677	61060	gi|566192679	43.29	27	2	2		6086	hypothetical protein POPTR_0010s25170g [Populus trichocarpa]
1686	1130	gi|225464489	43.14	9	1	1		12497	PREDICTED: 60S ribosomal protein L36-3 [Vitis vinifera]
1627	29810	gi|566156983	43.12	18	1	1		8764	hypothetical protein POPTR_0002s08770g partial [Populus trichocarpa]
1493	2040	gi|731428304	43.11	2	1	1		81014	PREDICTED: apoptotic chromatin condensation inducer in the nucleus-like isoform X2 [Vitis vinifera]
1082	1594	gi|731439095	43.05	4	1	1	Carbamidomethylation	31959	PREDICTED: GLABRA2 expression modulator [Vitis vinifera]

972	19793	gi|566181894	43.04	2	1	1		58001	hypothetical protein POPTR_0008s02250g [Populus trichocarpa]
1287	30041	gi|224066755	42.92	5	1	1		19335	thioredoxin m family protein [Populus trichocarpa]
1494	61284	gi|224144643	42.91	4	1	1		40333	hypothetical protein POPTR_0019s04130g [Populus trichocarpa]
1468	46566	gi|224144781	42.86	9	2	2	Carbamidomethylation	27942	hypothetical protein POPTR_0019s04910g [Populus trichocarpa]
2073	18556	gi|224067088	42.78	1	1	1		73926	hypothetical protein POPTR_0002s10850g [Populus trichocarpa]
1050	2148	gi|225450543	42.77	5	1	1		18729	PREDICTED: uncharacterized protein LOC100245914 [Vitis vinifera]
1087	18780	gi|224141359	42.66	5	2	2		40664	hypothetical protein POPTR_0017s11530g [Populus trichocarpa]
1095	2154	gi|225458519	42.65	2	1	1	Carbamidomethylation	75018	PREDICTED: uncharacterized protein LOC100267853 [Vitis vinifera]
1450	29091	gi|224120122	42.65	3	1	1	Carbamidomethylation	39228	Sorbitol Dehydrogenase family protein [Populus trichocarpa]
1339	17903	gi|224128320	42.36	2	1	1		80920	PROLIFERA family protein [Populus trichocarpa]
1010	2039	gi|225449198	42.25	4	1	1		31414	PREDICTED: eukaryotic translation initiation factor 3 subunit F [Vitis vinifera]

1325	18093	gi|566214132	42.23	1	1	1	Carbamidomethylation	108955	hypothetical protein POPTR_0018s03980g [Populus trichocarpa]
585	2150	gi|225464047	42.17	5	1	1		22575	PREDICTED: cysteine proteinase inhibitor 12 [Vitis vinifera]
1426	29551	gi|225458850	42.04	7	1	1	Carbamidomethylation	21998	PREDICTED: LIM domain-containing protein WLIM1 [Vitis vinifera]
1278	2026	gi|359492937	41.91	2	1	1		46528	PREDICTED: aspartic proteinase nepenthesin-1 [Vitis vinifera]
1103	17842	gi|224057968	41.9	4	1	1		24349	photosystem I chain III family protein [Populus trichocarpa]
1495	2245	gi|731381456	41.87	3	1	1		34629	PREDICTED: translocase of chloroplast 34 chloroplastic isoform X2 [Vitis vinifera]
1113	856	gi|225454422	41.85	8	1	1		16418	PREDICTED: eukaryotic translation initiation factor 1A [Vitis vinifera]
2129	36609	gi|224102065	41.79	7	1	1	Carbamidomethylation	15668	hypothetical protein POPTR_0008s15310g [Populus trichocarpa]
1357	1435	gi|225464870	41.77	4	1	1	Carbamidomethylation	37627	PREDICTED: bifunctional nitrilase/nitrile hydratase NIT4B [Vitis vinifera]
1243	1965	gi|225459318	41.69	3	1	1		35188	PREDICTED: annexin D1 [Vitis vinifera]
1253	18018	gi|224083504	41.67	2	2	1		105368	hypothetical protein POPTR_0005s07000g [Populus trichocarpa]

1630	18575	gi|566153712	41.59	3	1	1		52249	transport protein sec61 [Populus trichocarpa]
1575	1607	gi|225445166	41.55	1	1	1		122877	PREDICTED: uncharacterized protein LOC100262932 [Vitis vinifera]
1223	28940	gi|566162305	41.53	2	1	1	Carbamidomethylation	38904	UDP-glucose 4-epimerase family protein [Populus trichocarpa]
2070	17520	gi|224104913	41.44	14	1	1	Carbamidomethylation	12354	60S ribosomal protein L30 [Populus trichocarpa]
351	18903	gi|566146533	41.43	14	2	2		10147	acyl-CoA-binding family protein [Populus trichocarpa]
1524	833	gi|359483831	41.42	4	2	1		37846	PREDICTED: dnaJ homolog subfamily B member 13 [Vitis vinifera]
1689	1078	gi|359495228	41.39	1	1	1		81899	PREDICTED: far upstream element-binding protein 1 isoform X2 [Vitis vinifera]
2130	813	gi|225425356	41.36	5	1	1		32651	PREDICTED: U2 small nuclear ribonucleoprotein A' [Vitis vinifera]
1443	19496	gi|224078630	41.29	2	1	1		62205	ATP-binding-cassette transporter family protein [Populus trichocarpa]
1208	2512	gi|359491781	41.11	6	1	1		18046	PREDICTED: miraculin-like partial [Vitis vinifera]

1370	1096	gi|225445041	41.09	4	2	1		54472	PREDICTED: mitochondrial-processing peptidase subunit alpha [Vitis vinifera]
1496	1412	gi|225427234	40.96	8	1	1		17554	PREDICTED: glycine cleavage system H protein mitochondrial [Vitis vinifera]
1289	2600	gi|526117629	40.9	8	1	1	Carbamidomethylation	11686	lipid transfer protein precursor [Vitis vinifera]
568	61045	gi|566152205	40.72	0	1	1		298421	hypothetical protein POPTR_0001s32460g [Populus trichocarpa]
2071	18658	gi|224117530	40.69	6	1	1		21810	endoribonuclease L-PSP family protein [Populus trichocarpa]
2131	3535	gi|225428388	40.61	3	1	1		40284	PREDICTED: COP9 signalosome complex subunit 5b-like [Vitis vinifera]
1206	18993	gi|566235447	40.59	3	1	1	Carbamidomethylation	30476	hypothetical protein POPTR_0019s08170g partial [Populus trichocarpa]
718	18947	gi|566178547	40.49	3	1	1		25381	hypothetical protein POPTR_0006s28120g [Populus trichocarpa]
1275	17994	gi|566158382	40.46	3	2	2		77765	hypothetical protein POPTR_0002s16570g [Populus trichocarpa]
1358	19982	gi|566197668	40.31	6	1	1		18634	hypothetical protein POPTR_0012s09690g partial [Populus trichocarpa]
1550	1377	gi|225452877	40.03	3	1	1		57074	PREDICTED: cystathionine gamma-synthase chloroplastic [Vitis vinifera]

1291	61448	gi|225453275	39.98	4	1	1	Carbamidomethylation	14633	PREDICTED: 20 kDa chaperonin chloroplastic [Vitis vinifera]
1070	1200	gi|225452053	39.96	8	2	1		21963	PREDICTED: 40S ribosomal protein S7 [Vitis vinifera]
1330	18541	gi|224103381	39.85	2	1	1		53021	diaminopimelate decarboxylase family protein [Populus trichocarpa]
1687	29854	gi|224130644	39.73	6	1	1		21941	GRF1-INTERACTING FACTOR 2 family protein [Populus trichocarpa]
2132	1340	gi|225442595	39.72	2	1	1		60200	PREDICTED: protein disulfide isomerase-like 1-6 [Vitis vinifera]
1373	19921	gi|566210424	39.71	3	1	1		35704	hypothetical protein POPTR_0017s00350g [Populus trichocarpa]
1470	61034	gi|566183937	39.55	3	1	1		36660	hypothetical protein POPTR_0008s14270g [Populus trichocarpa]
1020	18464	gi|224116582	39.51	1	1	1		50722	2-oxoacid dehydrogenase family protein [Populus trichocarpa]
676	2504	gi|731416298	39.49	4	1	1		25123	PREDICTED: eukaryotic translation initiation factor 3 subunit J [Vitis vinifera]
1320	1981	gi|731400731	39.47	2	1	1		35100	PREDICTED: histone deacetylase HDT1 [Vitis vinifera]
1389	17767	gi|566157737	39.45	2	1	1		79950	hypothetical protein POPTR_0002s13010g [Populus trichocarpa]

1476	19947	gi|224084924	39.4	4	1	1	Carbamidomethylation	22828	Proteasome subunit beta type 3-2 family protein [Populus trichocarpa]
1497	18555	gi|566167707	39.36	4	1	1		29259	alcohol dehydroge family protein [Populus trichocarpa]
1374	19544	gi|566164408	39.08	2	1	1		45443	RNA-binding protein 45 [Populus trichocarpa]
2032	29683	gi|566197448	39.01	9	1	1	Carbamidomethylation	12293	hypothetical protein POPTR_0012s08450g [Populus trichocarpa]
1652	22114	gi|566162647	38.9	2	1	1		37095	hypothetical protein POPTR_0003s14560g [Populus trichocarpa]
2135	19820	gi|566210973	38.89	6	1	1		15927	hypothetical protein POPTR_0017s02810g [Populus trichocarpa]
167	61202	gi|566152126	38.65	2	1	1		51807	hypothetical protein POPTR_0001s31980g [Populus trichocarpa]
2133	19336	gi|225444159	38.47	5	1	1		32002	PREDICTED: coatomer subunit epsilon-1 [Vitis vinifera]
2134	2169	gi|731371107	38.47	1	1	1		89995	PREDICTED: probable splicing factor 3A subunit 1 [Vitis vinifera]
1473	61161	gi|566152274	38.15	6	1	1		14728	hypothetical protein POPTR_0001s32720g [Populus trichocarpa]
1406	61018	gi|359496591	38.02	5	2	2		35516	PREDICTED: bifunctional epoxide hydrolase 2 isoform X1 [Vitis vinifera]

1149	17737	gi|224096586	37.86	4	1	1		45989	eukaryotic translation initiation factor 4A family protein [Populus trichocarpa]
1006	2032	gi|359474652	37.61	2	1	1		50670	PREDICTED: scopoletin glucosyltransferase-like [Vitis vinifera]
1499	1321	gi|225424750	37.51	7	1	1	Carbamidomethylation	11841	PREDICTED: gibberellin-regulated protein 11 [Vitis vinifera]
865	43789	gi|671743335	37.41	21	1	1		4424	photosystem II cytochrome b559 beta subunit (chloroplast) [Camellia pubicosta]
1413	19146	gi|566160209	37.3	2	1	1	Carbamidomethylation	54758	hypothetical protein POPTR_0003s011001g partial [Populus trichocarpa]
1688	18988	gi|224072767	37.23	4	1	1		29070	hypothetical protein POPTR_0003s18420g [Populus trichocarpa]
1498	20007	gi|225454373	37.08	4	1	1		29156	PREDICTED: 50S ribosomal protein L5 chloroplastic [Vitis vinifera]
1753	17013	gi|566164286	37.03	2	1	1		44313	T-protein of the glycine decarboxylase complex [Populus trichocarpa]
1869	1134	gi|225423779	37.01	1	1	1		109803	PREDICTED: alanine--tRNA ligase [Vitis vinifera]
1722	3862	gi|731416275	36.68	2	1	1		71591	PREDICTED: mitochondrial Rho GTPase 1 [Vitis vinifera]

1341	18358	gi|566203933	36.49	10	1	1		13893	60S ribosomal protein L22-1 [Populus trichocarpa]
1451	20125	gi|566183496	36.48	2	1	1	Carbamidomethylation	36732	hypothetical protein POPTR_0008s11690g partial [Populus trichocarpa]
1623	19559	gi|566213202	36.38	8	1	1		12683	hypothetical protein POPTR_0017s13710g [Populus trichocarpa]
1228	2027	gi|225428747	36.26	3	1	1		33152	PREDICTED: 2-hydroxyisoflavanone dehydratase [Vitis vinifera]
1209	43827	gi|731410026	36.07	3	1	1		33957	PREDICTED: protein THYLAKOID FORMATION1 chloroplastic isoform X2 [Vitis vinifera]
1911	29347	gi|566150318	35.68	3	1	1		44042	strictosidine synthase family protein [Populus trichocarpa]
947	61324	gi|731386196	35.65	3	1	1		28590	PREDICTED: protein ASPARTIC PROTEASE IN GUARD CELL 1-like [Vitis vinifera]
1646	30104	gi|731427528	35.6	2	1	1		35075	PREDICTED: putative DNA-binding protein ESCAROLA [Vitis vinifera]
1262	2695	gi|731421487	35.57	3	1	1		35236	PREDICTED: uncharacterized protein LOC100265498 [Vitis vinifera]
1356	19834	gi|224082594	35.4	6	1	1		19313	photosystem 2 family protein [Populus trichocarpa]

1565	797	gi|225433894	35.08	1	1	1		246915	PREDICTED: U5 small nuclear ribonucleoprotein 200 kDa helicase [Vitis vinifera]
1455	29791	gi|224140057	35.07	3	1	1		29642	alcohol dehydroge family protein [Populus trichocarpa]
1224	30061	gi|566197758	35	10	1	1		11735	hypothetical protein POPTR_0012s10210g partial [Populus trichocarpa]
2074	2670	gi|225439378	34.85	4	1	1	Carbamidomethylation	24311	PREDICTED: ran-binding protein 1 homolog a [Vitis vinifera]
942	61222	gi|224059600	34.77	2	1	1		40957	hypothetical protein POPTR_0001s26960g [Populus trichocarpa]
1407	445	gi|225454087	34.68	1	1	1		66696	PREDICTED: NAD-dependent malic enzyme 59 kDa isoform mitochondrial [Vitis vinifera]
1214	1666	gi|526117699	34.64	2	1	1		49406	3-dehydroquinate synthase-like [Vitis vinifera]
1681	19370	gi|566181287	34.58	5	1	1	Carbamidomethylation	58258	hypothetical protein POPTR_0007s14380g [Populus trichocarpa]
804	18014	gi|566177270	34.52	1	1	1		108292	elongation factor Tu family protein [Populus trichocarpa]
838	1097	gi|731384037	34.38	2	1	1		37513	PREDICTED: proline iminopeptidase isoform X2 [Vitis vinifera]

1655	28969	gi|225468850	34.35	4	1	1		30830	PREDICTED: glyoxylate/succinic semialdehyde reductase 1 [Vitis vinifera]
844	18582	gi|566156002	34.25	2	1	1		37272	hypothetical protein POPTR_0002s03260g [Populus trichocarpa]
903	19106	gi|224078826	34.09	4	1	1		24905	Oxygen-evolving enhancer protein 3-1 [Populus trichocarpa]
1452	23632	gi|566210204	34.07	2	1	1		34838	peroxidase family protein [Populus trichocarpa]
663	2666	gi|225449032	34.03	2	1	1		52590	PREDICTED: dolichyl-diphosphooligosaccharide--protein glycosyltransferase subunit 1B [Vitis vinifera]
1340	723	gi|731370529	34.02	2	1	1		66006	PREDICTED: serine/threonine-protein phosphatase 2A 65 kDa regulatory subunit A beta isoform-like [Vitis vinifera]
652	1077	gi|225448675	33.97	3	1	1		23332	PREDICTED: probable chalcone--flavonone isomerase 3 [Vitis vinifera]
1657	2473	gi|225465847	33.9	8	1	1		21605	PREDICTED: NADPH:quinone oxidoreductase [Vitis vinifera]
1201	1733	gi|225429462	33.65	1	1	1		71749	PREDICTED: far upstream element-binding protein 2 [Vitis vinifera]

1454	17669	gi|566185352	33.63	1	1	1		79054	hypothetical protein POPTR_0008s22410g [Populus trichocarpa]
1475	61183	gi|731438465	33.58	3	1	1		26979	PREDICTED: 3-isopropylmalate dehydratase small subunit 3 [Vitis vinifera]
1658	28945	gi|225445462	33.56	7	1	1		14555	PREDICTED: uncharacterized protein At2g34160 [Vitis vinifera]
2029	7372	gi|225441521	33.36	4	1	1		18805	PREDICTED: 60S ribosomal protein L21-1 [Vitis vinifera]
1649	1809	gi|225445005	33.32	7	1	1		13980	PREDICTED: 60S ribosomal protein L31 [Vitis vinifera]
1437	17582	gi|566179618	33	2	1	1		48055	hypothetical protein POPTR_0007s04700g [Populus trichocarpa]
1629	1384	gi|225437898	32.79	6	1	1		15168	PREDICTED: photosystem I reaction center subunit IV B chloroplastic-like [Vitis vinifera]
1500	19090	gi|566204168	32.3	3	1	1		56223	Citrate synthase family protein [Populus trichocarpa]
1654	29587	gi|731417548	32.22	2	1	1		37774	PREDICTED: pyridoxal kinase isoform X8 [Vitis vinifera]
1723	5132	gi|225442452	32.17	3	1	1		54242	PREDICTED: hexokinase-2 [Vitis vinifera]

1268	1444	gi|731435761	32.16	1	1	1		160776	PREDICTED: putative disease resistance RPP13-like protein 1 isoform X1 [Vitis vinifera]
625	20939	gi|566209297	32.09	1	1	1		101212	hypothetical protein POPTR_0016s08100g [Populus trichocarpa]
1362	1964	gi|731433023	32.08	5	1	1	Carbamidomethylation	39210	PREDICTED: GDSL esterase/lipase At4g26790 [Vitis vinifera]
2136	2310	gi|225427364	32.03	3	1	1	Carbamidomethylation	51132	PREDICTED: poly(U)-specific endoribonuclease-B [Vitis vinifera]
1097	20307	gi|566167359	32.03	4	1	1		15952	hypothetical protein POPTR_0004s19310g [Populus trichocarpa]
1375	950	gi|225440362	32.01	12	1	1	Oxidation (M)	10797	PREDICTED: mitochondrial import inner membrane translocase subunit Tim9 [Vitis vinifera]
1501	20744	gi|566189812	31.8	2	1	1	Carbamidomethylation	50971	N-carbamyl-L-amino acid hydrolase family protein [Populus trichocarpa]
1326	1541	gi|225453754	31.43	15	2	2		23373	PREDICTED: probable xyloglucan endotransglucosylase/hydrolase protein 19 [Vitis vinifera]
1290	20079	gi|566163874	31.4	2	1	1		47484	hypothetical protein POPTR_0003s21170g [Populus trichocarpa]
1139	20266	gi|224130034	30.83	2	1	1		43250	Fasciclin-like arabinogalactan protein 8 precursor [Populus trichocarpa]

1690	61365	gi|566201869	30.79	3	1	1		29322	hypothetical protein POPTR_0014s01660g [Populus trichocarpa]
826	1128	gi|731418015	30.71	2	1	1		53972	PREDICTED: tryptophan synthase beta chain 1 [Vitis vinifera]
1608	6691	gi|225437318	30.51	1	1	1		157286	PREDICTED: translocase of chloroplast 159 chloroplastic [Vitis vinifera]
1503	61391	gi|731394556	30.05	7	1	1		12133	PREDICTED: uncharacterized protein At2g27730 mitochondrial [Vitis vinifera]
1376	61371	gi|566197290	30.02	11	1	1	Carbamidomethylation	9151	hypothetical protein POPTR_0012s07660g [Populus trichocarpa]
1502	3083	gi|225443150	29.89	11	1	1		9286	PREDICTED: uncharacterized protein LOC100262209 [Vitis vinifera]
1691	18205	gi|224054120	29.87	2	1	1		73852	lecithin:cholesterol acyltransferase family protein [Populus trichocarpa]
1083	29783	gi|566151677	29.86	2	1	1		44246	hypothetical protein POPTR_0001s29460g [Populus trichocarpa]
1504	1908	gi|225424859	28.98	4	1	1		25630	PREDICTED: glutathione S-transferase U17 [Vitis vinifera]
2138	17647	gi|566195150	28.97	4	1	1		25143	hypothetical protein POPTR_0011s12680g [Populus trichocarpa]

1626	819	gi|731423558	28.94	1	1	1		120839	PREDICTED: valine--tRNA ligase isoform X2 [Vitis vinifera]
1505	1351	gi|225464146	28.63	4	1	1		32622	PREDICTED: protein transport protein SEC13 homolog B [Vitis vinifera]
2075	17815	gi|566187112	28.39	2	1	1		38128	WD-40 repeat family protein [Populus trichocarpa]
1506	61403	gi|224107797	28.33	3	1	1		48955	clathrin adaptor complexes medium subunit family protein [Populus trichocarpa]
1457	5138	gi|731404421	28.15	3	1	1	Carbamidomethylation	51194	PREDICTED: monothiol glutaredoxin-S17 isoform X2 [Vitis vinifera]
1607	938	gi|225450669	28.07	1	1	1		113734	PREDICTED: eukaryotic translation initiation factor 3 subunit A [Vitis vinifera]
2139	2547	gi|731422864	27.82	15	1	1	Carbamidomethylation	8948	PREDICTED: probable isoaspartyl peptidase/L-asparaginase 3 [Vitis vinifera]
825	997	gi|731422162	27.72	2	1	1		44588	PREDICTED: glutamate dehydrogenase isoform X1 [Vitis vinifera]
1659	61411	gi|566162940	27.52	3	1	1		30690	Ubiquitin thiolesterase otubain-like family protein [Populus trichocarpa]

1917	2158	gi|731427572	27.44	3	1	1	Carbamidomethylation	31645	PREDICTED: splicing factor U2af small subunit B [Vitis vinifera]
1114	1310	gi|731401385	27.43	3	1	1		36064	PREDICTED: uncharacterized protein At5g02240 [Vitis vinifera]
1625	61109	gi|731411017	27.37	1	1	1		146120	PREDICTED: putative disease resistance RPP13-like protein 1 [Vitis vinifera]
2141	1567	gi|225433215	27.27	3	1	1	Carbamidomethylation	26277	PREDICTED: DAG protein chloroplastic [Vitis vinifera]
1692	18812	gi|671741947	27.26	11	1	1		9397	photosystem II cytochrome b559 alpha subunit (chloroplast) [Camellia grandibracteata]
1693	61430	gi|224059134	27	5	1	1		35532	mitotic phosphoprotein N' end [Populus trichocarpa]
1695	758	gi|225430549	27	1	1	1		82761	PREDICTED: eukaryotic translation initiation factor 3 subunit B [Vitis vinifera]
1694	2500	gi|526117641	26.55	4	1	1		21825	type II peroxiredoxin F [Vitis vinifera]
1211	3386	gi|731395454	26.46	3	1	1		19689	PREDICTED: uncharacterized protein OsI_027940 isoform X2 [Vitis vinifera]
1661	61433	gi|566168430	26.38	3	1	1		35849	hypothetical protein POPTR_0004s24220g [Populus trichocarpa]

1216	28967	gi|566209280	26.35	2	1	1		52718	hypothetical protein POPTR_0016s08010g [Populus trichocarpa]
895	20365	gi|566172986	25.94	2	1	1		29701	Histone H1 family protein [Populus trichocarpa]
1816	29461	gi|566170004	25.92	2	1	1	Carbamidomethylation	38544	hypothetical protein POPTR_0005s07370g [Populus trichocarpa]
1814	43899	gi|731420144	25.54	1	1	1		71058	PREDICTED: putative disease resistance RPP13-like protein 3 [Vitis vinifera]
1915	745	gi|731406154	25.18	1	1	1		199529	PREDICTED: brefeldin A-inhibited guanine nucleotide-exchange protein 5 [Vitis vinifera]
1507	32124	gi|566179420	25.06	9	1	1		10192	hypothetical protein POPTR_0007s03510g [Populus trichocarpa]
1417	1320	gi|731440862	24.88	8	1	1		15642	PREDICTED: 60S ribosomal protein L32-1 [Vitis vinifera]
1399	2681	gi|225455153	24.88	4	1	1		26938	PREDICTED: mitochondrial import inner membrane translocase subunit TIM22-like [Vitis vinifera]
1478	61490	gi|225443482	24.75	2	1	1		51597	PREDICTED: shikimate O-hydroxycinnamoyltransferase [Vitis vinifera]
1660	2120	gi|731389062	24.63	3	1	1		25568	PREDICTED: endo-1 3;1 4-beta-D-glucanase-like [Vitis vinifera]

1743	17036	gi|224063997	24.33	2	1	1		67268	NAD-dependent malic enzyme family protein [Populus trichocarpa]
1150	3189	gi|359494267	24.22	3	1	1		25766	PREDICTED: probable glutathione S-transferase [Vitis vinifera]
1662	61514	gi|225453289	23.91	4	1	1		21798	PREDICTED: ATP synthase subunit delta' mitochondrial [Vitis vinifera]
2145	3223	gi|359493124	23.8	4	1	1		23188	PREDICTED: abscisic stress-ripening protein 2 [Vitis vinifera]
1225	1695	gi|731408423	23.11	2	1	1		43098	PREDICTED: serine carboxypeptidase-like 18 isoform X4 [Vitis vinifera]
1792	18448	gi|566208224	23.08	2	1	1		98331	hypothetical protein POPTR_0016s02100g [Populus trichocarpa]
2147	19918	gi|731420480	23.07	2	1	1		38718	PREDICTED: plasminogen activator inhibitor 1 RNA-binding protein [Vitis vinifera]
1402	2034	gi|731399524	22.5	2	1	1		58565	PREDICTED: 70 kDa peptidyl-prolyl isomerase-like [Vitis vinifera]
1696	20644	gi|566185390	22.48	5	1	1		32450	hypothetical protein POPTR_0008s22600g [Populus trichocarpa]
1379	1670	gi|225436339	22.4	2	1	1		45109	PREDICTED: arogenate dehydratase 3 chloroplastic [Vitis vinifera]

2151	6509	gi|731426219	22.02	2	1	1		39322	PREDICTED: peptidyl-prolyl cis-trans isomerase CYP26-2 chloroplastic [Vitis vinifera]
1334	3166	gi|225464653	22	1	1	1		51852	PREDICTED: UDP-glycosyltransferase 88A1-like [Vitis vinifera]
2149	20529	gi|566149485	22	4	1	1		21742	germin-like family protein [Populus trichocarpa]
1697	33635	gi|225441979	21.86	1	1	1		63296	PREDICTED: pectinesterase 3 [Vitis vinifera]
1635	61530	gi|566256423	21.84	2	1	1		40507	hypothetical protein POPTR_0306s00210g [Populus trichocarpa]
1429	46181	gi|566175847	21.68	1	1	1		165976	hypothetical protein POPTR_0006s12120g partial [Populus trichocarpa]
2082	607	gi|225431169	21.55	3	1	1		28116	PREDICTED: 60S ribosomal protein L8 [Vitis vinifera]
1202	3347	gi|526117481	21.49	4	1	1		25140	chalcone--flavonone isomerase 2 [Vitis vinifera]
2084	61649	gi|566167089	21.47	2	1	1	Carbamidomethylation	54241	amidase family protein [Populus trichocarpa]
2079	17389	gi|566168468	21.44	3	1	1	Carbamidomethylation	68078	RNase L inhibitor family protein [Populus trichocarpa]
2035	61367	gi|566163422	21.42	3	1	1		34848	hypothetical protein POPTR_0003s18750g [Populus trichocarpa]
1363	20075	gi|224092174	21.36	4	1	1		36141	hypothetical protein POPTR_0006s24360g [Populus trichocarpa]

1387	25433	gi|566207901	21.16	1	1	1		90345	hypothetical protein POPTR_0016s00410g [Populus trichocarpa]
1415	29159	gi|566185166	21.15	2	1	1		63357	hypothetical protein POPTR_0008s21080g [Populus trichocarpa]
1870	19364	gi|566164258	21.06	3	1	1		59785	hypothetical protein POPTR_0004s00900g [Populus trichocarpa]
2039	20707	gi|566184883	20.88	1	1	1		68439	hypothetical protein POPTR_0008s19430g [Populus trichocarpa]
1571	61748	gi|566256176	20.8	4	1	1	Oxidation (M)	64121	AMP-dependent synthetase and ligase family protein [Populus trichocarpa]
2086	21289	gi|731386964	20.67	1	1	1		94119	PREDICTED: beta-galactosidase 3 [Vitis vinifera]
1699	61836	gi|731390764	20.62	1	1	1		112504	PREDICTED: pentatricopeptide repeat-containing protein At1g06710 mitochondrial isoform X2 [Vitis vinifera]
2153	2086	gi|225462297	20.52	5	1	1		56591	PREDICTED: succinate-semialdehyde dehydrogenase mitochondrial-like [Vitis vinifera]
1616	35039	gi|224055551	20.39	3	1	1		52190	monodehydroascorbate reductase family protein [Populus trichocarpa]
1638	25166	gi|566207323	20.35	1	1	1		61858	hypothetical protein POPTR_0015s12920g [Populus trichocarpa]

1872	3118	gi|359484223	20.26	1	1	1		85770	PREDICTED: probable galactinol--sucrose galactosyltransferase 6 [Vitis vinifera]
2155	30805	gi|566188564	20.2	3	1	1		29723	hypothetical protein POPTR_0010s00930g [Populus trichocarpa]
1700	19305	gi|566197081	20.2	1	1	1		125576	hypothetical protein POPTR_0012s06420g [Populus trichocarpa]
1177	22431	gi|566197112	20.06	5	1	1	Carbamidomethylation	14392	hypothetical protein POPTR_0012s06720g [Populus trichocarpa]

Table S4 The identified proteins in CK2

Protein Group	Protein ID	Accession	-10lgP	Coverage (%)	#Peptides	#Unique	PTM	Avg. Mass	Description
2	6	gi|359495606	290.99	53	48	3	Carbamidomethylation; Oxidation (M)	80006	PREDICTED: heat shock cognate protein 80 [Vitis vinifera]
3	7	gi|225462013	288.01	51	47	3	Carbamidomethylation	80849	PREDICTED: heat shock cognate protein 80-like [Vitis vinifera]
1	16386	gi|669100005	278.08	70	43	43	Carbamidomethylation; Oxidation (M)	52635	ribulose bisphosphate carboxylase large chain (chloroplast) [Camellia crapnelliana]
4	8	gi|225426164	273	41	39	2	Carbamidomethylation	80852	PREDICTED: heat shock protein 83 [Vitis vinifera]
5	15	gi|225464589	269.85	43	40	1	Carbamidomethylation	80868	PREDICTED: heat shock protein 83-like [Vitis vinifera]
9	16417	gi|224056837	269.1	40	39	2	Carbamidomethylation; Oxidation (M)	80026	hypothetical protein POPTR_0001s47020g [Populus trichocarpa]
17	1	gi|225426385	266.36	74	32	1	Carbamidomethylation	50292	PREDICTED: tubulin beta-2 chain [Vitis vinifera]
26	16395	gi|566195847	265.92	65	30	1	Carbamidomethylation	50164	tubulin beta chain family protein [Populus trichocarpa]
13	5	gi|359486799	261.06	53	39	2	Carbamidomethylation; Oxidation (M)	71171	PREDICTED: heat shock cognate 70 kDa protein 2 [Vitis vinifera]
14	10	gi|225434984	258.38	56	40	1	Carbamidomethylation; Oxidation (M)	71235	PREDICTED: heat shock cognate 70 kDa protein 2 [Vitis vinifera]
19	16410	gi|566191989	256.37	53	37	1	Carbamidomethylation; Oxidation (M)	71140	heat shock protein 70 cognate [Populus trichocarpa]
10	16404	gi|224055984	254.67	71	25	3	Carbamidomethylation; Oxidation (M)	41696	actin family protein [Populus trichocarpa]
12	12	gi|731410014	253.55	69	24	1	Carbamidomethylation	41593	PREDICTED: actin-101 [Vitis vinifera]
53	18	gi|225452767	249.82	66	30	3	Carbamidomethylation	49622	PREDICTED: tubulin alpha chain [Vitis vinifera]
40	17	gi|225434722	247.75	63	28	2	Carbamidomethylation	50275	PREDICTED: tubulin beta-2 chain [Vitis vinifera]
22	16	gi|225440324	244.78	43	34	1	Carbamidomethylation; Oxidation (M)	71066	PREDICTED: heat shock cognate 70 kDa protein 2 [Vitis vinifera]
77	20	gi|225440045	244.24	52	40	3	Carbamidomethylation; Oxidation (M)	89589	PREDICTED: cell division cycle protein 48 homolog [Vitis vinifera]

15	16442	gi|224056705	244	58	22	1	Carbamidomethylation	41636	actin family protein [Populus trichocarpa]
58	19	gi|731439963	240.57	62	28	1	Carbamidomethylation	49600	PREDICTED: tubulin alpha chain-like [Vitis vinifera]
81	16444	gi|224094244	232.25	29	28	4	Carbamidomethylation	93953	elongation factor 2 family protein [Populus trichocarpa]
54	16430	gi|566232573	232.15	35	27	1	Carbamidomethylation	85012	hypothetical protein POPTR_0019s05430g [Populus trichocarpa]
74	36	gi|225426230	231.89	35	32	1	Carbamidomethylation	73581	PREDICTED: luminal-binding protein 5 [Vitis vinifera]
52	58	gi|225449120	226.7	46	25	1	Carbamidomethylation	49376	PREDICTED: elongation factor 1-alpha [Vitis vinifera]
28	34	gi|225432155	222.55	64	19	1	Carbamidomethylation	43114	PREDICTED: S-adenosylmethionine synthase 2 [Vitis vinifera]
36	33	gi|225437708	222.42	65	18	1	Carbamidomethylation	42793	PREDICTED: S-adenosylmethionine synthase 5 [Vitis vinifera]
169	38	gi|225448483	221.48	21	31	1	Carbamidomethylation	192967	PREDICTED: clathrin heavy chain 1 [Vitis vinifera]
72	46	gi|225445820	219.22	33	23	2	Carbamidomethylation; Oxidation (M)	71402	PREDICTED: heat shock 70 kDa protein [Vitis vinifera]
150	16490	gi|566191652	218.34	21	35	2	Carbamidomethylation	193360	clathrin heavy chain family protein [Populus trichocarpa]
124	39	gi|225462164	216.01	29	28	4	Carbamidomethylation	93979	PREDICTED: elongation factor 2 [Vitis vinifera]
41	31	gi|225439223	215	35	27	4	Carbamidomethylation; Oxidation (M)	84991	PREDICTED: 5-methyltetrahydropteroyltriglutamate--homocysteine methyltransferase [Vitis vinifera]
61	16455	gi|566188155	213.72	32	26	3	Carbamidomethylation	89855	hypothetical protein POPTR_0009s15490g [Populus trichocarpa]
96	26	gi|225456079	213.48	44	19	5	Carbamidomethylation	59180	PREDICTED: ATP synthase subunit beta mitochondrial-like [Vitis vinifera]
98	70	gi|359490716	213.37	30	28	1	Carbamidomethylation	73489	PREDICTED: luminal-binding protein 5-like isoform X2 [Vitis vinifera]

100	16510	gi|566146992	212.31	28	21	2	Carbamidomethylation; Oxidation (M)	71904	heat shock protein 70 cognate [Populus trichocarpa]
66	16494	gi|566146990	211.47	28	22	1	Carbamidomethylation; Oxidation (M)	71882	heat shock protein 70 cognate [Populus trichocarpa]
86	44	gi|359473642	207.04	26	20	3	Carbamidomethylation	90465	PREDICTED: heat shock protein 90-1 [Vitis vinifera]
64	16461	gi|566169643	206.61	56	21	1	Carbamidomethylation	42375	NAD-dependent epimerase/dehydratase family protein [Populus trichocarpa]
67	86	gi|225439902	206.38	43	22	1	Carbamidomethylation; Oxidation (M)	49336	PREDICTED: elongation factor 1-alpha [Vitis vinifera]
139	41	gi|225456004	205.68	31	22	1	Carbamidomethylation	75635	PREDICTED: stromal 70 kDa heat shock-related protein chloroplastic [Vitis vinifera]
76	16464	gi|566199292	205	52	19	1	Carbamidomethylation	42361	hypothetical protein POPTR_0013s03790g [Populus trichocarpa]
110	16500	gi|224136806	204.78	43	20	5	Carbamidomethylation	47928	hypothetical protein POPTR_0015s14380g [Populus trichocarpa]
130	16475	gi|224099437	203.43	38	15	1	Carbamidomethylation	59970	H+-transporting two-sector ATPase family protein [Populus trichocarpa]
129	16508	gi|224144808	202.73	43	17	2	Carbamidomethylation; Oxidation (M)	49595	Tubulin alpha-1 chain family protein [Populus trichocarpa]
99	16496	gi|224085900	202.68	24	25	7		94047	hypothetical protein POPTR_0005s26260g [Populus trichocarpa]
126	16476	gi|566212215	201.33	47	21	2	Carbamidomethylation; Oxidation (M)	53198	adenosylhomocysteinase family protein [Populus trichocarpa]
138	42	gi|731393262	199.78	40	27	5	Carbamidomethylation	64608	PREDICTED: ruBisCO large subunit-binding protein subunit beta chloroplastic [Vitis vinifera]
174	16468	gi|542688129	199.44	46	16	14		53694	ATP synthase CF1 beta subunit (chloroplast) [Camellia taliensis]
105	16511	gi|566196410	197.58	28	23	3		73406	BiP isoform A family protein [Populus trichocarpa]

160	78	gi|359478916	196.34	40	14	4	Carbamidomethylation	48674	PREDICTED: ribulose bisphosphate carboxylase/oxygenase activase chloroplastic isoform X2 [Vitis vinifera]
151	16497	gi|224088798	195.56	43	19	1	Carbamidomethylation	46974	translation initiation factor eIF-4A family protein [Populus trichocarpa]
149	60	gi|225429488	195.5	42	19	1	Carbamidomethylation	46873	PREDICTED: eukaryotic initiation factor 4A-11 [Vitis vinifera]
221	17009	gi|224065861	194.11	52	14	4	Carbamidomethylation	18243	Peptidyl-prolyl cis-trans isomerase family protein [Populus trichocarpa]
152	16518	gi|566204581	193.63	22	16	3	Carbamidomethylation	82947	hypothetical protein POPTR_0014s16280g [Populus trichocarpa]
123	294	gi|225437549	191.9	22	13	1	Carbamidomethylation	48111	PREDICTED: calreticulin [Vitis vinifera]
50	112	gi|731394274	191.36	44	17	2	Carbamidomethylation	42738	PREDICTED: S-adenosylmethionine synthase 1 isoform X2 [Vitis vinifera]
207	16503	gi|224073124	191.03	32	20	2	Carbamidomethylation	64544	chaperonin precursor family protein [Populus trichocarpa]
187	72	gi|225456880	190.7	34	17	4	Carbamidomethylation	52692	PREDICTED: elongation factor TuB chloroplastic [Vitis vinifera]
158	67	gi|224365668	190.32	35	20	17	Carbamidomethylation	55143	ATPase subunit 1 (mitochondrion) [Vitis vinifera]
56	16553	gi|224133862	190.3	44	18	1	Carbamidomethylation	36770	glyceraldehyde-3-phosphate dehydrogenase family protein [Populus trichocarpa]
135	94	gi|225464999	189.66	36	15	5		42422	PREDICTED: phosphoglycerate kinase cytosolic [Vitis vinifera]
172	166	gi|225433510	189.3	33	12	4	Carbamidomethylation	51906	PREDICTED: serine hydroxymethyltransferase 4 [Vitis vinifera]
266	89	gi|731402464	188.37	21	15	4	Carbamidomethylation	93319	PREDICTED: heat shock 70 kDa protein 14-like [Vitis vinifera]

60	16520	gi|224109716	188.01	34	19	1	Carbamidomethylation	49434	hypothetical protein POPTR_0010s22620g [Populus trichocarpa]
208	116	gi|731410136	187.36	17	15	3	Carbamidomethylation	95223	PREDICTED: heat shock protein 83 isoform X3 [Vitis vinifera]
91	16524	gi|566176113	187.32	35	19	1	Carbamidomethylation	49308	hypothetical protein POPTR_0006s13310g [Populus trichocarpa]
142	104	gi|225456806	186.23	36	18	1	Carbamidomethylation	53079	PREDICTED: adenosylhomocysteinase-like [Vitis vinifera]
157	119	gi|731415672	185.96	21	19	1		72363	PREDICTED: mediator of RNA polymerase II transcription subunit 37a [Vitis vinifera]
212	66	gi|225460961	184.96	19	17	2	Carbamidomethylation; Oxidation (M)	110056	PREDICTED: aconitate hydratase cytoplasmic [Vitis vinifera]
101	16541	gi|566192207	184.76	40	20	1	Carbamidomethylation	49434	hypothetical protein POPTR_0010s22560g [Populus trichocarpa]
180	74	gi|731393844	184.52	21	21	4	Carbamidomethylation	101855	PREDICTED: protein argonaute 4 [Vitis vinifera]
125	100	gi|225457939	184.37	23	22	3	Carbamidomethylation	93220	PREDICTED: endoplasmin homolog [Vitis vinifera]
249	61	gi|731426977	182.6	26	23	17	Carbamidomethylation	102347	PREDICTED: ATP-dependent Clp protease ATP-binding subunit clpA homolog CD4B chloroplastic [Vitis vinifera]
141	138	gi|731423140	182.21	27	16	1	Carbamidomethylation	55855	PREDICTED: enolase [Vitis vinifera]
214	16544	gi|566173508	182.19	32	13	2	Carbamidomethylation	56756	catalase family protein [Populus trichocarpa]
155	16674	gi|542688125	182.06	26	12	12		56099	photosystem II p680 chlorophyll A apoprotein CP-47 (chloroplast) [Camellia taliensis]
70	252	gi|526117553	181.97	33	10	1	Carbamidomethylation	39322	glutamine synthetase cytosolic isozyme 2 [Vitis vinifera]
95	160	gi|359491599	181.03	40	15	2	Carbamidomethylation	36735	PREDICTED: glyceraldehyde-3-phosphate dehydrogenase cytosolic [Vitis vinifera]

83	102	gi|731393868	180.78	21	17	1	Carbamidomethylation	89385	PREDICTED: 5-methyltetrahydropteroyltriglutamate--homocysteine methyltransferase 2 [Vitis vinifera]
177	81	gi|225461660	180.59	20	21	2	Carbamidomethylation	109823	PREDICTED: phosphoenolpyruvate carboxylase housekeeping isozyme [Vitis vinifera]
245	92	gi|359479362	180.02	27	15	2		61984	PREDICTED: ruBisCO large subunit-binding protein subunit alpha [Vitis vinifera]
292	82	gi|225443399	179.9	30	18	1	Carbamidomethylation	68723	PREDICTED: V-type proton ATPase catalytic subunit A [Vitis vinifera]
159	16526	gi|224063766	179.18	21	15	4	Carbamidomethylation	80716	transketolase family protein [Populus trichocarpa]
217	128	gi|225430776	178.74	23	12	5	Carbamidomethylation	73659	PREDICTED: succinate dehydrogenase [ubiquinone] flavoprotein subunit mitochondrial [Vitis vinifera]
259	108	gi|526117643	178.39	20	17	5	Carbamidomethylation	101329	heat shock protein 101 [Vitis vinifera]
163	145	gi|225464995	178.14	32	15	2	Carbamidomethylation	50083	PREDICTED: phosphoglycerate kinase chloroplastic [Vitis vinifera]
113	16693	gi|566209405	178.1	33	14	1	Carbamidomethylation	44445	hypothetical protein POPTR_0016s08780g [Populus trichocarpa]
179	16538	gi|566153806	177.81	18	17	1		110763	phosphoenolpyruvate carboxylase family protein [Populus trichocarpa]
213	85	gi|225460328	176.71	21	16	1	Carbamidomethylation	74178	PREDICTED: stromal 70 kDa heat shock-related protein chloroplastic-like [Vitis vinifera]
211	16609	gi|224053971	176.59	30	15	2	Carbamidomethylation	52743	hypothetical protein POPTR_0001s08770g [Populus trichocarpa]
230	16528	gi|224104681	176.52	27	17	2		61991	RUBISCO SUBUNIT BINDING-protein ALPHA SUBUNIT [Populus trichocarpa]

396	97	gi|225441955	176.3	48	17	17	Carbamidomethylation	47682	PREDICTED: 26S protease regulatory subunit 7 [Vitis vinifera]
82	353	gi|359483839	176.16	52	11	1	Carbamidomethylation	27890	PREDICTED: chlorophyll a-b binding protein of LHCII type 1 [Vitis vinifera]
117	203	gi|526117485	175.68	31	9	2	Carbamidomethylation	40813	naringenin 2-oxoglutarate 3-dioxygenase [Vitis vinifera]
186	84	gi|225442993	175.63	36	17	15	Carbamidomethylation	48355	PREDICTED: DEAD-box ATP-dependent RNA helicase 56 [Vitis vinifera]
457	111	gi|225432252	175.16	38	13	1	Carbamidomethylation	47374	PREDICTED: 26S protease regulatory subunit 6A homolog [Vitis vinifera]
268	161	gi|731388175	175.13	26	14	3	Carbamidomethylation	65990	PREDICTED: ATP-citrate synthase beta chain protein 2 [Vitis vinifera]
79	534	gi|225461832	175.04	33	12	1	Carbamidomethylation; Oxidation (M)	28275	PREDICTED: chlorophyll a-b binding protein of LHCII type 1 [Vitis vinifera]
199	122	gi|731430736	175.04	33	14	1	Carbamidomethylation	44590	PREDICTED: 60S ribosomal protein L3 [Vitis vinifera]
161	110	gi|359490179	174.78	21	14	3	Carbamidomethylation	78856	PREDICTED: transketolase chloroplastic [Vitis vinifera]
231	16583	gi|224079726	174.75	31	14	4	Carbamidomethylation	46428	isocitrate dehydrogenase family protein [Populus trichocarpa]
235	16672	gi|566203657	174.73	26	11	6	Carbamidomethylation	56866	hypothetical protein POPTR_0014s10800g [Populus trichocarpa]
282	16560	gi|566206267	174.04	17	16	4	Carbamidomethylation	100324	hypothetical protein POPTR_0015s06760g [Populus trichocarpa]
290	136	gi|225456274	173.59	33	17	5	Carbamidomethylation	55180	PREDICTED: argininosuccinate synthase chloroplastic [Vitis vinifera]
197	127	gi|731411443	173.25	29	13	1	Carbamidomethylation	44506	PREDICTED: 60S ribosomal protein L3-2 isoform X1 [Vitis vinifera]

188	16607	gi|224109060	172.54	28	13	1	Carbamidomethylation	50212	PHOSPHOGLYCERATE KINASE 1 family protein [Populus trichocarpa]
225	16612	gi|566182577	172.09	34	11	3	Carbamidomethylation	48316	hypothetical protein POPTR_0008s05870g [Populus trichocarpa]
227	16545	gi|566168009	171.73	28	17	2	Oxidation (M)	62041	RUBISCO SUBUNIT BINDING-protein ALPHA SUBUNIT [Populus trichocarpa]
270	130	gi|731402352	171.08	23	18	2	Carbamidomethylation	65010	PREDICTED: ruBisCO large subunit-binding protein subunit beta chloroplastic [Vitis vinifera]
361	16605	gi|224141947	170.88	27	12	2	Carbamidomethylation	53416	hypothetical protein POPTR_0018s02400g [Populus trichocarpa]
107	16804	gi|224053300	170.62	68	14	3	Carbamidomethylation	17702	ubiquitin/ribosomal protein 27a [Populus trichocarpa]
183	16572	gi|566204141	170.57	34	13	4	Carbamidomethylation	42935	hypothetical protein POPTR_0014s13660g [Populus trichocarpa]
243	16574	gi|224058852	170.11	17	13	3	Carbamidomethylation	94161	heat shock protein 70 [Populus trichocarpa]
90	159	gi|225462777	169.63	36	15	2	Carbamidomethylation	42597	PREDICTED: chalcone synthase 2 [Vitis vinifera]
108	143	gi|731415862	169.45	35	15	5	Carbamidomethylation	49362	PREDICTED: calreticulin [Vitis vinifera]
223	146	gi|731418890	169.34	28	13	1	Carbamidomethylation	52944	PREDICTED: elongation factor Tu chloroplastic-like [Vitis vinifera]
206	16770	gi|566212213	168.72	26	10	2	Carbamidomethylation	51876	SERINE HYDROXYMETHYLTRANSFERASE 4 family protein [Populus trichocarpa]
281	95	gi|731379116	168.54	19	15	5	Carbamidomethylation	100128	PREDICTED: puromycin-sensitive aminopeptidase isoform X2 [Vitis vinifera]
484	155	gi|225446449	168.39	16	11	2	Carbamidomethylation	97503	PREDICTED: 26S proteasome non-ATPase regulatory subunit 2 homolog A [Vitis vinifera]

455	154	gi|225430151	167.68	47	17	16	Carbamidomethylation	47221	PREDICTED: 26S protease regulatory subunit 8 homolog A [Vitis vinifera]
237	16604	gi|566150406	167.41	17	17	1	Carbamidomethylation	101827	Argonaute 4 family protein [Populus trichocarpa]
321	16552	gi|224101561	166.78	23	14	3	Carbamidomethylation	65798	subunit B of the trimeric enzyme ATP Citrate lyase family protein [Populus trichocarpa]
144	16585	gi|224099261	166.2	17	16	2	Carbamidomethylation	90156	heat shock family protein [Populus trichocarpa]
399	16713	gi|566213382	166.11	22	10	1	Carbamidomethylation	53743	phosphogluconate dehydrogenase family protein [Populus trichocarpa]
247	101	gi|225429228	165.65	25	14	2		72770	PREDICTED: heat shock 70 kDa protein mitochondrial [Vitis vinifera]
330	141	gi|731419756	165.59	26	11	3	Carbamidomethylation	52962	PREDICTED: UDP-glucose 6-dehydrogenase 1 [Vitis vinifera]
366	16639	gi|224086120	165.2	21	10	1	Carbamidomethylation	67209	pyrophosphate-dependent phosphofructokinase alpha subunit family protein [Populus trichocarpa]
233	16575	gi|224063263	164.86	25	12	1	Carbamidomethylation	58692	2-dehydro-3-deoxyphosphoheptonate aldolase family protein [Populus trichocarpa]
148	217	gi|225437428	164.63	13	12	3	Carbamidomethylation	92483	PREDICTED: sucrose synthase 2 [Vitis vinifera]
255	139	gi|225468576	164.63	17	14	2	Carbamidomethylation; Oxidation (M)	98187	PREDICTED: aconitate hydratase 1 [Vitis vinifera]
425	16576	gi|224119200	164.4	15	17	2	Carbamidomethylation	136903	coatomer alpha subunit-like family protein [Populus trichocarpa]
140	16640	gi|671743230	164.27	19	11	10	Carbamidomethylation	82370	photosystem I P700 apoprotein A2 (chloroplast) [Camellia petelotii]
191	16617	gi|568244555	164.17	31	12	12		51822	photosystem II CP43 chlorophyll apoprotein (plastid) [Camellia oleifera]
109	121	gi|225425884	163.97	44	17	1	Carbamidomethylation	36759	PREDICTED: glyceraldehyde-3-phosphate dehydrogenase cytosolic [Vitis vinifera]

404	238	gi|359481634	163.91	72	10	5	Carbamidomethylation	16594	PREDICTED: ubiquitin-conjugating enzyme E2 variant 1D [Vitis vinifera]
258	93	gi|359496771	163.79	23	14	1		73271	PREDICTED: heat shock 70 kDa protein mitochondrial-like [Vitis vinifera]
287	16787	gi|224102193	162.99	33	11	4	Carbamidomethylation; Oxidation (M)	35716	cytosolic malate dehydrogenase family protein [Populus trichocarpa]
189	16723	gi|224141865	162.97	46	10	10	Carbamidomethylation	25015	GTP-binding family protein [Populus trichocarpa]
440	16615	gi|224078391	161.97	31	11	11	Carbamidomethylation	54253	vacuolar ATP synthase subunit B family protein [Populus trichocarpa]
236	198	gi|731406211	161.31	11	11	3	Carbamidomethylation	92443	PREDICTED: sucrose synthase [Vitis vinifera]
453	107	gi|225452950	161.18	17	13	9	Carbamidomethylation	109834	PREDICTED: chaperone protein ClpB3 chloroplastic [Vitis vinifera]
364	189	gi|731428324	160.6	7	13	4	Carbamidomethylation	252296	PREDICTED: acetyl-CoA carboxylase 1-like [Vitis vinifera]
319	123	gi|731391766	159.72	18	17	3	Carbamidomethylation	127035	PREDICTED: ubiquitin-activating enzyme E1 1 [Vitis vinifera]
313	16705	gi|566210462	159.46	19	11	2	Carbamidomethylation	67453	Methylenetetrahydrofolate reductase family protein [Populus trichocarpa]
406	132	gi|731428778	159.34	27	13	2	Carbamidomethylation	56891	PREDICTED: serine hydroxymethyltransferase mitochondrial [Vitis vinifera]
370	16601	gi|224069951	159.16	24	13	2	Carbamidomethylation	61124	Chaperonin CPN60-2 family protein [Populus trichocarpa]
336	16665	gi|224087339	158.81	23	10	2		57707	biotin carboxylase precursor family protein [Populus trichocarpa]
275	16573	gi|566151627	158.72	24	15	3		73210	heat shock protein 70 [Populus trichocarpa]
525	137	gi|526117707	158.24	33	11	6	Carbamidomethylation	53695	uncharacterized protein LOC100261274 [Vitis vinifera]
360	16970	gi|568244554	158.12	27	7	7		39549	photosystem II protein D2 (plastid) [Camellia oleifera]

80	186	gi|526117870	158.04	33	12	1	Carbamidomethylation	42744	chalcone synthase [Vitis vinifera]
649	16755	gi|566211390	157.76	13	9	1	Carbamidomethylation	97445	hypothetical protein POPTR_0017s04660g [Populus trichocarpa]
428	158	gi|731388277	157.61	28	10	3	Carbamidomethylation	58138	PREDICTED: importin subunit alpha-1 isoform X1 [Vitis vinifera]
277	295	gi|359480275	156.95	35	9	2	Oxidation (M)	35505	PREDICTED: malate dehydrogenase cytoplasmic [Vitis vinifera]
475	17030	gi|566150460	156.87	55	10	9	Carbamidomethylation	16862	calmodulin-like protein 6a [Populus trichocarpa]
295	194	gi|225442018	156.61	29	10	1	Carbamidomethylation	33909	PREDICTED: 40S ribosomal protein SA [Vitis vinifera]
548	140	gi|359475304	156.56	16	12	5	Carbamidomethylation	98624	PREDICTED: coatomer subunit gamma-2 [Vitis vinifera]
567	204	gi|731401620	155.8	18	15	4	Carbamidomethylation	104406	PREDICTED: ATPase 10 plasma membrane-type [Vitis vinifera]
528	500	gi|225440011	155.66	16	9	2		77480	PREDICTED: phenylalanine ammonia-lyase [Vitis vinifera]
69	168	gi|359482256	155.32	33	12	1	Carbamidomethylation; Oxidation (M)	42618	PREDICTED: S-adenosylmethionine synthase 3 [Vitis vinifera]
599	206	gi|731385022	155.32	16	11	6	Carbamidomethylation	114759	PREDICTED: glycine dehydrogenase (decarboxylating) mitochondrial [Vitis vinifera]
541	16799	gi|224097588	154.85	24	10	1	Carbamidomethylation	46583	DnaJ family protein [Populus trichocarpa]
506	286	gi|731412519	154.52	16	9	1	Carbamidomethylation	78831	PREDICTED: phenylalanine ammonia-lyase G1 [Vitis vinifera]
118	281	gi|225465030	154.48	60	13	2		17196	PREDICTED: ubiquitin-NEDD8-like protein RUB2 [Vitis vinifera]
210	16754	gi|566176273	154.44	10	12	4		92143	sucrose synthase family protein [Populus trichocarpa]
359	349	gi|359491680	154.42	47	8	3	Carbamidomethylation	17414	PREDICTED: eukaryotic translation initiation factor 5A-2 [Vitis vinifera]
607	156	gi|225441236	154.42	9	11	4	Carbamidomethylation	177678	PREDICTED: ferredoxin-dependent glutamate synthase chloroplastic [Vitis vinifera]

378	16586	gi|671743392	154.25	27	15	3		55440	ATP synthase CF1 alpha subunit (chloroplast) [Camellia reticulata]
391	187	gi|225457674	154.02	17	9	1	Carbamidomethylation	67344	PREDICTED: pyrophosphate--fructose 6-phosphate 1-phosphotransferase subunit alpha [Vitis vinifera]
234	16610	gi|566159486	153.93	13	15	2	Carbamidomethylation	113839	hypothetical protein POPTR_0002s22720g [Populus trichocarpa]
346	16776	gi|224095561	153.88	12	9	1	Carbamidomethylation	97704	hypothetical protein POPTR_0007s01200g [Populus trichocarpa]
84	16660	gi|566163210	153.82	30	13	1	Carbamidomethylation	42709	naregenin-chalcone synthase family protein [Populus trichocarpa]
280	228	gi|225451995	153.66	40	11	4		29540	PREDICTED: 14-3-3-like protein A [Vitis vinifera]
344	16703	gi|224063273	153.46	37	10	1		29380	14-3-3 protein 32kDa endonuclease [Populus trichocarpa]
116	16659	gi|224110772	153.35	35	14	1	Carbamidomethylation	37124	glyceraldehyde 3-phosphate dehydrogenase family protein [Populus trichocarpa]
392	304	gi|225425053	153	21	10	1	Carbamidomethylation	53492	PREDICTED: 6-phosphogluconate dehydrogenase decarboxylating 3 [Vitis vinifera]
468	16898	gi|224116086	152.95	25	7	2	Carbamidomethylation	37284	hypothetical protein POPTR_0011s00500g [Populus trichocarpa]
216	16920	gi|566167040	152.82	27	9	1	Carbamidomethylation	33502	hypothetical protein POPTR_0004s17530g [Populus trichocarpa]
394	16826	gi|224094759	152.81	18	7	1	Carbamidomethylation	69860	Succinate dehydrogenase flavoprotein subunit [Populus trichocarpa]
417	200	gi|225427768	152.78	26	9	3	Carbamidomethylation	42948	PREDICTED: fructose-bisphosphate aldolase 1 chloroplastic [Vitis vinifera]

203	338	gi|225427917	152.73	28	11	2	Carbamidomethylation	34675	PREDICTED: triosephosphate isomerase chloroplastic [Vitis vinifera]
380	163	gi|225456339	152.44	30	11	1	Carbamidomethylation	41981	PREDICTED: UDP-arabinopyranose mutase 1 isoform X2 [Vitis vinifera]
416	16670	gi|224073851	151.92	14	10	1	Carbamidomethylation	94625	heat shock protein 70 [Populus trichocarpa]
379	221	gi|225445664	150.1	19	9	1		57338	PREDICTED: biotin carboxylase 1 chloroplastic [Vitis vinifera]
446	444	gi|225437777	149.87	16	7	2	Carbamidomethylation	58724	PREDICTED: putative proline--tRNA ligase C19C7.06 [Vitis vinifera]
375	16712	gi|224100783	149.7	16	9	2	Carbamidomethylation	77598	phenylalanine ammonia-lyase family protein [Populus trichocarpa]
552	223	gi|225429530	149.43	19	8	3	Carbamidomethylation	54362	PREDICTED: UDP-sulfoquinovose synthase chloroplastic [Vitis vinifera]
418	192	gi|225434712	149.36	37	9	4	Carbamidomethylation	29576	PREDICTED: proliferating cell nuclear antigen [Vitis vinifera]
388	17072	gi|224114557	149.36	23	7	3	Carbamidomethylation	35588	Malate dehydrogenase family protein [Populus trichocarpa]
402	16781	gi|566165396	149.15	20	9	1	Carbamidomethylation	51757	hypothetical protein POPTR_0004s07280g [Populus trichocarpa]
184	16939	gi|224109788	149.12	28	8	1	Carbamidomethylation	37547	putative adenosine kinase family protein [Populus trichocarpa]
250	214	gi|526117762	149.09	39	11	1	Carbamidomethylation	29376	14-3-3 protein [Vitis vinifera]
352	254	gi|225430619	148.99	42	10	10	Carbamidomethylation	22781	PREDICTED: 40S ribosomal protein S5 [Vitis vinifera]
565	239	gi|225458111	148.47	8	9	1		136997	PREDICTED: coatomer subunit alpha-1 [Vitis vinifera]
626	241	gi|731413130	148.41	15	11	5	Oxidation (M)	116111	PREDICTED: 2-oxoglutarate dehydrogenase mitochondrial [Vitis vinifera]
334	17104	gi|671743459	148.36	81	6	6	Carbamidomethylation	9038	photosystem I subunit VII (chloroplast) [Camellia reticulata]

373	237	gi|225446579	148.26	17	10	4	Carbamidomethylation; Oxidation (M)	63486	PREDICTED: ketol-acid reductoisomerase chloroplastic [Vitis vinifera]
181	494	gi|225449541	147.78	29	8	2	Carbamidomethylation	27128	PREDICTED: triosephosphate isomerase cytosolic [Vitis vinifera]
520	16872	gi|566170807	147.62	26	10	1	Carbamidomethylation	40534	Chain A family protein [Populus trichocarpa]
232	16732	gi|435856353	147.22	29	10	10	Carbamidomethylation	38951	photosystem II protein D1 (chloroplast) [Camellia sinensis]
964	362	gi|225464186	147.13	25	8	8	Carbamidomethylation	49585	PREDICTED: 26S proteasome regulatory subunit 4 homolog B [Vitis vinifera]
608	16748	gi|224060514	146.71	15	9	4	Carbamidomethylation	70476	polyadenylate-binding family protein [Populus trichocarpa]
551	135	gi|731402494	146.67	17	13	5	Carbamidomethylation	102377	PREDICTED: coatomer subunit beta'-1 [Vitis vinifera]
492	585	gi|225444377	146.48	23	6	1	Carbamidomethylation	38105	PREDICTED: thiamine thiazole synthase 2 chloroplastic [Vitis vinifera]
300	16706	gi|224113365	146.01	10	11	1	Carbamidomethylation	130750	NtN2 family protein [Populus trichocarpa]
173	165	gi|359493870	145.68	24	12	4	Carbamidomethylation	42815	PREDICTED: protochlorophyllide reductase chloroplastic [Vitis vinifera]
479	282	gi|731423943	145.06	45	8	2	Carbamidomethylation	19961	PREDICTED: 40S ribosomal protein S10 [Vitis vinifera]
424	317	gi|225441799	145.01	31	10	3	Carbamidomethylation	29974	PREDICTED: 40S ribosomal protein S2-3 [Vitis vinifera]
441	374	gi|225426162	144.86	43	8	2	Carbamidomethylation	20013	PREDICTED: 40S ribosomal protein S10 [Vitis vinifera]
655	393	gi|359473789	144.66	32	9	1	Carbamidomethylation; Oxidation (M)	28023	PREDICTED: peptidyl-prolyl cis-trans isomerase CYP20-2 chloroplastic [Vitis vinifera]
170	170	gi|225446944	144.66	35	11	3	Carbamidomethylation	43340	PREDICTED: protochlorophyllide reductase [Vitis vinifera]
472	16661	gi|134093182	144.63	22	13	1		55322	ATP synthase CF1 alpha subunit (chloroplast) [Populus trichocarpa]
288	176	gi|225448546	144.33	34	10	1	Carbamidomethylation	29880	PREDICTED: 40S ribosomal protein S4-1 [Vitis vinifera]

502	16729	gi|566160536	144.06	14	9	1	Carbamidomethylation	91733	phospholipase D family protein [Populus trichocarpa]
289	16635	gi|566178983	144.03	33	13	1	Oxidation (M)	41070	hypothetical protein POPTR_0007s01010g [Populus trichocarpa]
482	17010	gi|566171046	143.74	11	6	1	Carbamidomethylation	70165	hypothetical protein POPTR_0005s12530g [Populus trichocarpa]
448	218	gi|731395250	143.41	23	9	2	Carbamidomethylation	48931	PREDICTED: elongation factor Tu mitochondrial [Vitis vinifera]
447	371	gi|225457957	143.38	44	9	1	Carbamidomethylation	17938	PREDICTED: peptidyl-prolyl cis-trans isomerase [Vitis vinifera]
269	16633	gi|566186668	143.3	29	12	2		41811	ADP family protein [Populus trichocarpa]
353	177	gi|731398850	143.22	13	10	2	Carbamidomethylation	113970	PREDICTED: ubiquitin-activating enzyme E1 1-like isoform X2 [Vitis vinifera]
720	16801	gi|224105405	143.16	10	7	1	Carbamidomethylation	98780	hypothetical protein POPTR_0009s11800g [Populus trichocarpa]
316	307	gi|225468761	143.1	26	7	1	Carbamidomethylation	35289	PREDICTED: oxygen-evolving enhancer protein 1 chloroplastic [Vitis vinifera]
405	16636	gi|566181869	142.74	28	15	3	Carbamidomethylation	54252	hypothetical protein POPTR_0008s02110g [Populus trichocarpa]
430	16889	gi|566170533	142.68	20	8	8	Carbamidomethylation	47176	hypothetical protein POPTR_0005s10290g [Populus trichocarpa]
467	229	gi|731427020	142.52	18	11	4	Carbamidomethylation; Oxidation (M)	76330	PREDICTED: glycine--tRNA ligase 1 mitochondrial [Vitis vinifera]
311	16634	gi|224059642	142.51	29	12	1		42100	adenine nucleotide translocator family protein [Populus trichocarpa]
540	222	gi|225430398	142.44	21	10	8		50823	PREDICTED: aspartate aminotransferase chloroplastic [Vitis vinifera]
577	209	gi|731408286	142.31	27	11	11	Carbamidomethylation	53389	PREDICTED: xylose isomerase [Vitis vinifera]

456	363	gi|225464852	142.26	57	6	1	Carbamidomethylation	16577	PREDICTED: ubiquitin-conjugating enzyme E2 variant 1D [Vitis vinifera]
487	367	gi|225444472	142.18	20	8	3	Carbamidomethylation	44306	PREDICTED: aminomethyltransferase mitochondrial [Vitis vinifera]
491	171	gi|225441044	141.72	19	9	2		57520	PREDICTED: pyruvate kinase cytosolic isozyme-like [Vitis vinifera]
279	175	gi|225434482	141.59	34	10	1	Carbamidomethylation	29851	PREDICTED: 40S ribosomal protein S4 [Vitis vinifera]
542	583	gi|225435357	141.47	13	8	2	Carbamidomethylation	77966	PREDICTED: phenylalanine ammonia-lyase [Vitis vinifera]
521	563	gi|731376167	141.3	28	5	5	Carbamidomethylation	29267	PREDICTED: proteasome subunit beta type-5 [Vitis vinifera]
342	258	gi|731404310	141.07	25	7	4	Carbamidomethylation	33129	PREDICTED: putative lactoylglutathione lyase [Vitis vinifera]
228	17398	gi|224138586	140.95	18	5	2		27577	ascorbate peroxidase family protein [Populus trichocarpa]
489	17097	gi|566161423	140.94	28	7	7	Carbamidomethylation	24286	60S ribosomal protein L15 [Populus trichocarpa]
594	173	gi|225451806	140.81	28	10	1	Carbamidomethylation	40505	PREDICTED: UDP-arabinopyranose mutase 3 [Vitis vinifera]
584	17023	gi|566202051	140.72	11	6	1	Carbamidomethylation	71118	hypothetical protein POPTR_0014s02550g [Populus trichocarpa]
309	149	gi|225435480	140.53	26	11	1		42838	PREDICTED: ADP ATP carrier protein mitochondrial [Vitis vinifera]
613	16728	gi|566174183	140.48	28	9	8	Carbamidomethylation	43533	26S proteasome subunit 7 family protein [Populus trichocarpa]
298	313	gi|225458424	140.37	22	7	1	Carbamidomethylation	35120	PREDICTED: oxygen-evolving enhancer protein 1 chloroplastic [Vitis vinifera]
198	440	gi|225461156	140.33	61	7	3		13980	PREDICTED: probable histone H2A.2 [Vitis vinifera]

733	231	gi|225450275	140.2	33	7	1	Carbamidomethylation	35771	PREDICTED: serine/threonine-protein phosphatase PP2A-2 catalytic subunit [Vitis vinifera]
443	253	gi|225451581	140.16	23	8	3	Carbamidomethylation	38690	PREDICTED: mitochondrial phosphate carrier protein 3 mitochondrial isoform X1 [Vitis vinifera]
604	525	gi|225426063	140.06	18	8	2	Carbamidomethylation	53896	PREDICTED: 6-phosphogluconate dehydrogenase decarboxylating 1 chloroplastic [Vitis vinifera]
555	625	gi|225431122	139.99	26	10	1	Carbamidomethylation	40534	PREDICTED: ferredoxin--NADP reductase leaf-type isozyme chloroplastic [Vitis vinifera]
595	411	gi|359497202	139.96	16	8	8	Carbamidomethylation	60614	PREDICTED: T-complex protein 1 subunit eta [Vitis vinifera]
314	17045	gi|224084209	139.96	21	7	1	Carbamidomethylation	35142	O2 evolving complex 33kD family protein [Populus trichocarpa]
432	16791	gi|566173570	139.7	24	9	1	Carbamidomethylation	44912	hypothetical protein POPTR_0005s27550g [Populus trichocarpa]
638	17264	gi|224121954	139.25	28	6	1	Carbamidomethylation	26774	hypothetical protein POPTR_0012s09720g [Populus trichocarpa]
355	16655	gi|224146592	138.91	47	11	11	Carbamidomethylation	24975	60S ribosomal protein L10 [Populus trichocarpa]
331	188	gi|731408817	138.4	29	11	1	Oxidation (M)	41099	PREDICTED: ADP ATP carrier protein 3 mitochondrial [Vitis vinifera]
678	16687	gi|224062085	138.35	27	9	8	Carbamidomethylation	44915	26S proteasome AAA-ATPase subunit family protein [Populus trichocarpa]
285	255	gi|526117940	138.23	9	7	2	Carbamidomethylation	91805	phospholipase D alpha [Vitis vinifera]
127	16968	gi|566210965	138.21	29	10	1	Carbamidomethylation	39217	glutamate-ammonia ligase family protein [Populus trichocarpa]
194	16815	gi|224117218	138.16	22	9	1	Carbamidomethylation	43107	NADPH-protochlorophyllide oxidoreductase family protein [Populus trichocarpa]

656	17350	gi|566209174	138.03	24	6	1	Carbamidomethylation	29522	peroxiredoxin family protein [Populus trichocarpa]
601	292	gi|225439231	137.83	15	7	2	Carbamidomethylation	55389	PREDICTED: 3-isopropylmalate dehydratase large subunit-like [Vitis vinifera]
515	447	gi|359494836	137.8	11	7	3	Carbamidomethylation	61508	PREDICTED: pyrophosphate--fructose 6-phosphate 1-phosphotransferase subunit beta [Vitis vinifera]
702	247	gi|225452974	137.75	20	12	8		58482	PREDICTED: probable mitochondrial-processing peptidase subunit beta [Vitis vinifera]
347	365	gi|225459918	137.65	34	8	3	Carbamidomethylation	30824	PREDICTED: chlorophyll a-b binding protein CP26 chloroplastic [Vitis vinifera]
426	448	gi|526117711	137.54	21	6	1	Carbamidomethylation	36875	malate dehydrogenase [Vitis vinifera]
471	314	gi|225432110	137.27	19	10	10	Carbamidomethylation	52920	PREDICTED: dihydrolipoyl dehydrogenase mitochondrial [Vitis vinifera]
554	16962	gi|224128376	137.18	19	8	4	Carbamidomethylation	39401	thioredoxin family protein [Populus trichocarpa]
708	16798	gi|566149322	136.87	11	10	1	Carbamidomethylation	105097	putative plasma membrane H+ ATPase family protein [Populus trichocarpa]
415	16874	gi|224082478	136.7	21	9	1	Carbamidomethylation	44381	ribosomal protein 1 [Populus trichocarpa]
384	16823	gi|224104269	136.26	37	9	1	Carbamidomethylation	22228	peptidyl-prolyl cis-trans isomerase family protein [Populus trichocarpa]
531	16824	gi|566159427	135.75	21	9	1	Carbamidomethylation	49034	Elongation factor Tu family protein [Populus trichocarpa]
646	321	gi|225457058	135.65	16	9	1	Carbamidomethylation	60477	PREDICTED: T-complex protein 1 subunit gamma [Vitis vinifera]
306	17071	gi|224101413	135.45	45	6	1	Carbamidomethylation	17417	eukaryotic translation initiation factor 5A isoform VI family protein [Populus trichocarpa]

519	610	gi|225464928	135.43	15	9	2		46452	PREDICTED: eukaryotic initiation factor 4A-3 [Vitis vinifera]
133	16986	gi|224079530	135.3	26	10	1	Carbamidomethylation	39256	glutamate-ammonia ligase family protein [Populus trichocarpa]
576	337	gi|225423961	135.11	11	8	4	Carbamidomethylation	103148	PREDICTED: alpha-xylosidase 1 [Vitis vinifera]
516	17017	gi|566210666	135.06	14	7	1	Carbamidomethylation	51605	hypothetical protein POPTR_0017s01390g [Populus trichocarpa]
193	16937	gi|224098421	134.88	34	10	2	Carbamidomethylation	27259	Triosephosphate isomerase family protein [Populus trichocarpa]
403	16833	gi|566186876	134.87	12	11	2	Carbamidomethylation	122028	hypothetical protein POPTR_0009s07950g partial [Populus trichocarpa]
296	16829	gi|224087813	134.77	29	8	1	Carbamidomethylation	29763	hypothetical protein POPTR_0006s10460g [Populus trichocarpa]
579	16969	gi|224097488	134.76	7	8	1	Carbamidomethylation	103480	hypothetical protein POPTR_0008s01100g [Populus trichocarpa]
593	16923	gi|552540866	134.63	27	8	8		35220	cytochrome f (chloroplast) [Camellia danzaiensis]
333	16825	gi|566210060	134.31	29	8	1	Carbamidomethylation	29720	hypothetical protein POPTR_0016s13210g [Populus trichocarpa]
559	404	gi|225435782	134.17	18	8	4	Carbamidomethylation	44564	PREDICTED: 60S ribosomal protein L4 [Vitis vinifera]
848	17053	gi|566184833	134.1	17	7	2	Carbamidomethylation	53373	alanine-2-oxoglutarate aminotransferase 1 family protein [Populus trichocarpa]
327	17257	gi|224108938	133.74	38	5	1	Carbamidomethylation	17381	hypothetical protein POPTR_0010s17020g [Populus trichocarpa]
751	16897	gi|224135653	133.48	10	8	1	Carbamidomethylation	106660	putative plasma membrane H+ ATPase family protein [Populus trichocarpa]
254	16941	gi|224059598	133.44	36	7	7		24937	40S ribosomal protein S8 [Populus trichocarpa]

459	16756	gi|566157403	133.14	20	9	1	Carbamidomethylation	57254	hypothetical protein POPTR_0002s10990g [Populus trichocarpa]
765	335	gi|225445867	133.04	14	6	3	Carbamidomethylation	60743	PREDICTED: leucine aminopeptidase 1 [Vitis vinifera]
611	414	gi|225429452	132.74	17	8	7	Carbamidomethylation	57141	PREDICTED: serine hydroxymethyltransferase 3 chloroplastic [Vitis vinifera]
783	17061	gi|224092184	132.62	15	7	2	Carbamidomethylation	53653	hypothetical protein POPTR_0006s24440g [Populus trichocarpa]
813	416	gi|225456641	132.26	10	8	1	Carbamidomethylation	105309	PREDICTED: plasma membrane ATPase 1 isoform X1 [Vitis vinifera]
524	417	gi|225429850	132.23	31	6	4		24915	PREDICTED: proteasome subunit beta type-6 [Vitis vinifera]
512	351	gi|225457231	132.22	14	6	2		56145	PREDICTED: flavonoid 3'-monooxygenase [Vitis vinifera]
634	17820	gi|566175251	131.87	12	5	5		34569	hypothetical protein POPTR_0006s08710g [Populus trichocarpa]
168	17277	gi|566170863	131.82	53	8	8		11409	histone H4 family protein [Populus trichocarpa]
556	271	gi|225439064	131.81	15	8	4		61092	PREDICTED: 2 3-bisphosphoglycerate-independent phosphoglycerate mutase [Vitis vinifera]
966	852	gi|225423722	131.72	30	5	2		25584	PREDICTED: proteasome subunit alpha type-2-B [Vitis vinifera]
566	16785	gi|224075451	131.61	12	9	2	Carbamidomethylation	77174	Glycyl-tRNA synthetase family protein [Populus trichocarpa]
690	455	gi|225434259	131.58	18	8	1	Carbamidomethylation	49810	PREDICTED: guanosine nucleotide diphosphate dissociation inhibitor 2 [Vitis vinifera]
337	333	gi|225447725	131.56	14	6	3	Carbamidomethylation	61438	PREDICTED: calnexin homolog [Vitis vinifera]
703	449	gi|731420830	131.16	26	6	1		31951	PREDICTED: prohibitin-1 mitochondrial [Vitis vinifera]

444	245	gi|225462254	131.15	25	11	2	Carbamidomethylation	45573	PREDICTED: glyceraldehyde-3-phosphate dehydrogenase GAPCP1 chloroplastic [Vitis vinifera]
682	243	gi|225429750	131.03	13	11	4	Carbamidomethylation	105919	PREDICTED: coatomer subunit beta-1 [Vitis vinifera]
782	16998	gi|566156890	130.93	19	8	2		44077	hypothetical protein POPTR_0002s08240g [Populus trichocarpa]
693	17028	gi|224095286	130.8	20	8	1	Carbamidomethylation	49785	GDP dissociation inhibitor family protein [Populus trichocarpa]
648	16707	gi|224126969	130.77	20	10	2		58085	hypothetical protein POPTR_0013s15380g [Populus trichocarpa]
166	467	gi|225439749	130.73	50	5	3		15764	PREDICTED: histone H2A [Vitis vinifera]
793	17494	gi|224134414	130.65	30	5	2		25625	Proteasome subunit alpha type 2 family protein [Populus trichocarpa]
374	584	gi|225451279	130.56	20	6	1		29903	PREDICTED: 40S ribosomal protein S3a-2-like [Vitis vinifera]
462	16945	gi|224090986	130.46	29	8	7	Carbamidomethylation	31176	chlorophyll a/b binding protein CP29 [Populus trichocarpa]
883	16899	gi|566190691	130.41	16	7	1	Carbamidomethylation	65588	Ser/Thr specific protein phosphatase 2A A regulatory subunit alpha isoform [Populus trichocarpa]
335	16800	gi|224129102	130.31	26	9	9	Carbamidomethylation	42388	hypothetical protein POPTR_0014s15960g [Populus trichocarpa]
294	595	gi|225447408	130.15	35	8	3	Carbamidomethylation	28115	PREDICTED: 60S ribosomal protein L8 [Vitis vinifera]
185	16812	gi|566168262	130.09	20	11	4		47873	elongation factor 1-gamma 1 family protein [Populus trichocarpa]
809	306	gi|731438745	129.96	11	8	3	Carbamidomethylation	92847	PREDICTED: DNA replication licensing factor MCM6 [Vitis vinifera]

759	16820	gi|566256635	129.89	17	8	1	Carbamidomethylation	52351	hypothetical protein POPTR_0261s00200g [Populus trichocarpa]
585	16940	gi|566206623	129.7	15	8	2	Carbamidomethylation	54072	aldehyde dehydrogenase 1 precursor family protein [Populus trichocarpa]
982	16786	gi|566208504	129.65	11	9	2	Carbamidomethylation; Oxidation (M)	128539	hypothetical protein POPTR_0016s03630g [Populus trichocarpa]
781	273	gi|731411266	129.6	10	10	1	Carbamidomethylation	134450	PREDICTED: cullin-associated NEDD8-dissociated protein 1 [Vitis vinifera]
817	16828	gi|224120474	129.4	13	12	2	Carbamidomethylation	117667	hypothetical protein POPTR_0012s03410g [Populus trichocarpa]
509	460	gi|359483716	129.38	23	6	2		29551	PREDICTED: 14-3-3-like protein D isoform X2 [Vitis vinifera]
305	16840	gi|566158870	129.34	59	9	9		17678	hypothetical protein POPTR_0002s19210g [Populus trichocarpa]
122	453	gi|225447576	129.31	31	6	1	Carbamidomethylation	28468	PREDICTED: chlorophyll a-b binding protein 151 chloroplastic [Vitis vinifera]
497	602	gi|359476682	128.91	24	7	1	Carbamidomethylation	24349	PREDICTED: soluble inorganic pyrophosphatase isoform X2 [Vitis vinifera]
242	17038	gi|566174988	128.82	28	7	2		28378	hypothetical protein POPTR_0006s07290g [Populus trichocarpa]
771	400	gi|731401656	128.7	35	7	7	Carbamidomethylation	25978	PREDICTED: proteasome subunit alpha type-5 [Vitis vinifera]
381	379	gi|225442192	128.67	32	7	3		21908	PREDICTED: 60S ribosomal protein L9 [Vitis vinifera]
705	17520	gi|224104913	128.43	53	5	2	Carbamidomethylation	12354	60S ribosomal protein L30 [Populus trichocarpa]
239	17145	gi|566159084	128.38	6	7	1		92938	hypothetical protein POPTR_0002s20340g [Populus trichocarpa]

291	216	gi|526117840	128.29	15	9	1	Carbamidomethylation	58698	3-deoxy-D-arabino-heptulosonate-7-phosphate synthase [Vitis vinifera]
948	754	gi|225428865	128.26	12	6	1		70683	PREDICTED: polyadenylate-binding protein 2 [Vitis vinifera]
621	341	gi|731391907	128.03	14	7	5	Carbamidomethylation	67493	PREDICTED: anthranilate synthase alpha subunit 2 chloroplastic isoform X2 [Vitis vinifera]
711	276	gi|225432012	127.93	18	7	3	Carbamidomethylation	54243	PREDICTED: galactokinase [Vitis vinifera]
940	16946	gi|224075980	127.89	8	7	1	Carbamidomethylation	123548	hypothetical protein POPTR_0003s21100g [Populus trichocarpa]
511	364	gi|225451146	127.86	16	10	6		57701	PREDICTED: bifunctional 3-dehydroquinate dehydratase/shikimate dehydrogenase chloroplastic [Vitis vinifera]
407	301	gi|225452510	127.79	13	8	2	Carbamidomethylation	58057	PREDICTED: aldehyde dehydrogenase family 2 member B7 mitochondrial [Vitis vinifera]
537	16958	gi|224099363	127.7	9	7	2		101194	alpha-xylosidase family protein [Populus trichocarpa]
673	390	gi|225425838	127.4	31	7	2	Carbamidomethylation	26900	PREDICTED: adenylate kinase 4-like [Vitis vinifera]
442	17021	gi|224083020	127.29	47	8	2	Carbamidomethylation; Oxidation (M)	18138	Peptidyl-prolyl cis-trans isomerase family protein [Populus trichocarpa]
195	361	gi|225431804	127.11	27	6	1		24165	PREDICTED: 60S ribosomal protein L19-2 [Vitis vinifera]
589	16788	gi|566171760	127.1	5	9	1		252377	acetyl-CoA carboxylase family protein [Populus trichocarpa]
1018	741	gi|731399769	126.97	26	5	2	Carbamidomethylation	30174	PREDICTED: 2-Cys peroxiredoxin isoform X1 [Vitis vinifera]
989	17036	gi|224063997	126.86	12	6	2		67268	NAD-dependent malic enzyme family protein [Populus trichocarpa]

365	16784	gi|566175737	126.85	24	9	2	Carbamidomethylation	47050	Monodehydroascorbate reductase family protein [Populus trichocarpa]
494	17234	gi|566207041	126.66	15	5	3	Carbamidomethylation	53652	glutamate 1-semialdehyde aminotransferase family protein [Populus trichocarpa]
653	428	gi|225464279	126.57	12	11	1	Carbamidomethylation	119705	PREDICTED: protein argonaute 1 [Vitis vinifera]
1016	17054	gi|566183505	126.52	15	6	1	Carbamidomethylation	65372	phosphatase family protein [Populus trichocarpa]
398	427	gi|731399148	126.37	55	8	8	Carbamidomethylation	14802	PREDICTED: 40S ribosomal protein S15a [Vitis vinifera]
937	311	gi|225430390	126.3	14	7	3	Carbamidomethylation	57260	PREDICTED: threonine synthase chloroplastic [Vitis vinifera]
721	441	gi|225462272	126.25	28	6	1		31679	PREDICTED: prohibitin-1 mitochondrial isoform X1 [Vitis vinifera]
328	226	gi|359497288	126.24	17	9	4	Carbamidomethylation; Oxidation (M)	64041	PREDICTED: peptidyl-prolyl cis-trans isomerase FKBP62 isoform X1 [Vitis vinifera]
882	325	gi|225456051	125.69	20	10	3	Carbamidomethylation	57589	PREDICTED: T-complex protein 1 subunit delta [Vitis vinifera]
780	16750	gi|566160146	125.56	10	10	1	Carbamidomethylation	134331	TIP120 family protein [Populus trichocarpa]
368	17311	gi|566254838	125.52	27	7	2	Carbamidomethylation	28211	hypothetical protein POPTR_0789s00200g [Populus trichocarpa]
383	181	gi|359476269	125.38	46	10	1	Carbamidomethylation	26279	PREDICTED: 40S ribosomal protein S3-3 [Vitis vinifera]
913	457	gi|225433588	125.38	10	7	4	Carbamidomethylation	90882	PREDICTED: probable methionine--tRNA ligase [Vitis vinifera]
816	17246	gi|566190515	125.03	17	5	3	Carbamidomethylation	42536	hypothetical protein POPTR_0010s12790g [Populus trichocarpa]
175	16867	gi|566147823	124.83	14	9	4	Carbamidomethylation; Oxidation (M)	83349	hypothetical protein POPTR_0001s07870g [Populus trichocarpa]

324	16841	gi|669099987	124.72	12	11	10	Carbamidomethylation	83116	photosystem I P700 chlorophyll A apoprotein A1 (chloroplast) [Camellia crapnelliana]
616	16955	gi|566202195	124.5	27	7	7		34821	hypothetical protein POPTR_0014s03240g [Populus trichocarpa]
500	627	gi|225464720	124.35	17	6	2	Carbamidomethylation	44946	PREDICTED: oxygen-dependent coproporphyrinogen-III oxidase chloroplastic-like [Vitis vinifera]
658	293	gi|225455784	124.32	22	8	6	Carbamidomethylation	50558	PREDICTED: enolase 1 chloroplastic [Vitis vinifera]
1010	16991	gi|566196314	124.06	4	6	1	Carbamidomethylation	244200	NADH-dependent glutamate synthase family protein [Populus trichocarpa]
631	17258	gi|224140177	123.98	12	7	3	Carbamidomethylation	53698	adenylosuccinate synthetase family protein [Populus trichocarpa]
664	413	gi|225457075	123.67	16	7	1	Carbamidomethylation	58613	PREDICTED: aldehyde dehydrogenase family 2 member B4 mitochondrial [Vitis vinifera]
785	572	gi|731382516	123.65	17	5	5	Carbamidomethylation	37912	PREDICTED: ankyrin repeat domain-containing protein 2 isoform X2 [Vitis vinifera]
397	618	gi|225464412	123.6	35	7	7		16188	PREDICTED: 40S ribosomal protein S17 [Vitis vinifera]
911	17183	gi|224098004	123.58	14	6	1	Carbamidomethylation	47073	19S proteosome subunit 9 family protein [Populus trichocarpa]
514	316	gi|359480881	123.51	10	6	6	Carbamidomethylation	94936	PREDICTED: beta-galactosidase 10 [Vitis vinifera]
498	269	gi|359476642	123.44	19	7	1	Carbamidomethylation	42804	PREDICTED: fructose-bisphosphate aldolase 1 chloroplastic-like [Vitis vinifera]
1158	17457	gi|224129210	123.31	14	4	2	Carbamidomethylation	41121	truncated acetyl Co-A acetyltransferase-like family protein [Populus trichocarpa]
535	481	gi|225442079	123.15	33	8	8	Carbamidomethylation	27325	PREDICTED: proteasome subunit alpha type-6 [Vitis vinifera]
312	17348	gi|566184073	123.13	25	6	6	Carbamidomethylation	22518	photosystem I 20kD family protein [Populus trichocarpa]

660	538	gi|225439982	123.12	53	7	7	Carbamidomethylation	17793	PREDICTED: 60S ribosomal protein L12 [Vitis vinifera]
176	17140	gi|224075758	123.08	51	7	1	Carbamidomethylation	15268	hypothetical protein POPTR_0003s22120g [Populus trichocarpa]
811	326	gi|359492510	123.08	19	8	1	Carbamidomethylation	41451	PREDICTED: probable L-ascorbate peroxidase 6 chloroplastic isoform X2 [Vitis vinifera]
832	16895	gi|566191895	123.01	8	7	3	Carbamidomethylation	154710	phosphoribosylformylglycinamidine synthase family protein [Populus trichocarpa]
549	16779	gi|566175799	123.01	20	10	2	Carbamidomethylation	57465	pyruvate kinase family protein [Populus trichocarpa]
777	16892	gi|224143423	122.99	10	9	2	Carbamidomethylation	105985	putative coatmer beta subunit family protein [Populus trichocarpa]
586	728	gi|359485890	122.96	7	6	2	Carbamidomethylation	83365	PREDICTED: beta-xylosidase/alpha-L-arabinofuranosidase 2-like [Vitis vinifera]
715	454	gi|225449132	122.87	32	7	1		19768	PREDICTED: ATP synthase subunit d mitochondrial [Vitis vinifera]
1028	17343	gi|224068388	122.85	13	4	1		53197	Fumarate hydratase 1 family protein [Populus trichocarpa]
434	17013	gi|566164286	122.83	15	7	2	Carbamidomethylation	44313	T-protein of the glycine decarboxylase complex [Populus trichocarpa]
680	431	gi|731391105	122.79	15	8	4	Carbamidomethylation	49499	PREDICTED: calreticulin-3-like [Vitis vinifera]
464	547	gi|225456295	122.06	18	5	4		24961	PREDICTED: elongation factor 1-delta [Vitis vinifera]
838	445	gi|225454087	121.97	11	6	2		66696	PREDICTED: NAD-dependent malic enzyme 59 kDa isoform mitochondrial [Vitis vinifera]
677	473	gi|225436253	121.96	13	7	1	Carbamidomethylation	66904	PREDICTED: probable methylenetetrahydrofolate reductase [Vitis vinifera]

789	17259	gi|566170121	121.83	13	5	2	Carbamidomethylation	56464	1L-myo-inositol 1-phosphate synthase family protein [Populus trichocarpa]
438	251	gi|225461654	121.71	26	7	2		28864	PREDICTED: 14-3-3-like protein D [Vitis vinifera]
317	17341	gi|566238007	121.68	20	6	1	Carbamidomethylation	30925	Chlorophyll a-b binding protein CP26 [Populus trichocarpa]
698	558	gi|225437493	121.53	12	5	1	Carbamidomethylation	58594	PREDICTED: importin subunit alpha [Vitis vinifera]
323	17040	gi|566184599	121.43	17	8	2		37085	glyceraldehyde 3-phosphate dehydrogenase family protein [Populus trichocarpa]
583	626	gi|225449078	121.28	25	6	1	Carbamidomethylation; Oxidation (M)	26327	PREDICTED: chlorophyll a-b binding protein 6 chloroplastic [Vitis vinifera]
410	487	gi|225433247	121.26	42	5	5	Carbamidomethylation	15031	PREDICTED: 60S ribosomal protein L23 [Vitis vinifera]
802	697	gi|731423169	121.02	35	6	6	Carbamidomethylation	13646	PREDICTED: 40S ribosomal protein S20-2 [Vitis vinifera]
1020	16907	gi|566174304	120.78	6	8	1	Carbamidomethylation	177274	ferredoxin-dependent glutamate synthase family protein [Populus trichocarpa]
1208	17327	gi|224086948	120.44	16	5	2	Carbamidomethylation	51291	eukaryotic translation initiation factor 3E family protein [Populus trichocarpa]
667	16922	gi|224058615	120.43	27	7	3	Carbamidomethylation	33817	hypothetical protein POPTR_0001s08570g [Populus trichocarpa]
670	17027	gi|566195881	120.27	20	7	1	Carbamidomethylation	44659	hypothetical protein POPTR_0011s16690g [Populus trichocarpa]
676	639	gi|731428049	120.18	11	4	2	Carbamidomethylation	47334	PREDICTED: delta-aminolevulinic acid dehydratase chloroplastic [Vitis vinifera]
578	17069	gi|224077927	120	16	6	1	Carbamidomethylation	42796	latex plastidic aldolase-like family protein [Populus trichocarpa]
815	391	gi|225425280	119.8	16	7	2		48103	PREDICTED: uncharacterized protein LOC100247241 [Vitis vinifera]

633	927	gi|731440185	119.51	13	5	2		30189	PREDICTED: 60S ribosomal protein L5-like [Vitis vinifera]
897	1124	gi|731392283	119.46	18	6	1	Carbamidomethylation	38986	PREDICTED: UDP-glucuronic acid decarboxylase 6 [Vitis vinifera]
993	818	gi|731396254	119.37	13	5	4	Carbamidomethylation	40707	PREDICTED: alcohol dehydrogenase class-3 [Vitis vinifera]
615	17819	gi|224131818	119.26	24	4	1		28513	chlorophyll a/b-binding family protein [Populus trichocarpa]
806	648	gi|731424733	119.16	4	6	1	Carbamidomethylation	241380	PREDICTED: glutamate synthase 1 [NADH] chloroplastic isoform X2 [Vitis vinifera]
1030	520	gi|225431090	119.12	34	6	6		27195	PREDICTED: proteasome subunit alpha type-7 [Vitis vinifera]
357	16803	gi|224089867	118.98	9	10	1	Carbamidomethylation	103746	hypothetical protein POPTR_0006s02680g [Populus trichocarpa]
843	16957	gi|224075445	118.95	15	7	2	Carbamidomethylation	47494	transaldolase family protein [Populus trichocarpa]
341	16818	gi|224135747	118.77	36	9	1	Carbamidomethylation	26076	40S ribosomal protein S3 [Populus trichocarpa]
1039	16938	gi|224145979	118.63	16	7	1	Carbamidomethylation	58735	hypothetical protein POPTR_0019s04980g [Populus trichocarpa]
729	336	gi|225429870	118.62	14	8	8	Carbamidomethylation	60861	PREDICTED: aspartate--tRNA ligase cytoplasmic [Vitis vinifera]
885	571	gi|225441589	118.38	6	6	1	Carbamidomethylation	123527	PREDICTED: importin-5 [Vitis vinifera]
763	17190	gi|224114826	118.32	39	5	1	Carbamidomethylation	17193	ubiquitin-conjugating enzyme family protein [Populus trichocarpa]
460	235	gi|225444782	118.32	34	8	1	Carbamidomethylation	26314	PREDICTED: 40S ribosomal protein S3-3 [Vitis vinifera]
833	16827	gi|566182445	118.18	11	8	2	Carbamidomethylation; Oxidation (M)	115769	2-oxoglutarate dehydrogenase E1 component family protein [Populus trichocarpa]
463	17207	gi|566151109	118.1	18	7	1		37403	hypothetical protein POPTR_0001s26400g [Populus trichocarpa]

818	16835	gi|224082111	117.99	35	9	8	Carbamidomethylation	28421	hypothetical protein POPTR_0005s17280g [Populus trichocarpa]
385	412	gi|225443760	117.95	29	7	1	Carbamidomethylation	20875	PREDICTED: 60S ribosomal protein L11-1-like [Vitis vinifera]
740	831	gi|225456392	117.8	16	5	1	Carbamidomethylation	29387	PREDICTED: 60S ribosomal protein L7a [Vitis vinifera]
893	320	gi|225441629	117.7	15	7	1	Carbamidomethylation	58891	PREDICTED: T-complex protein 1 subunit theta [Vitis vinifera]
476	389	gi|225441754	117.56	12	7	3		53382	PREDICTED: adenylosuccinate synthetase 2 chloroplastic [Vitis vinifera]
1014	611	gi|225437455	117.38	11	4	2		53532	PREDICTED: fumarate hydratase 1 mitochondrial [Vitis vinifera]
522	17062	gi|224090212	117.14	23	7	2	Carbamidomethylation	29609	40S ribosomal protein S2 [Populus trichocarpa]
696	519	gi|225450981	117.13	17	7	7	Carbamidomethylation	51125	PREDICTED: serine--tRNA ligase [Vitis vinifera]
539	17042	gi|566164883	117.09	20	8	2	Carbamidomethylation	43246	ketol-acid reductoisomerase family protein partial [Populus trichocarpa]
1109	1010	gi|225451999	117.06	13	6	6		59112	PREDICTED: T-complex protein 1 subunit zeta [Vitis vinifera]
753	17243	gi|224100839	117.03	30	7	1		19919	ATP synthase D chain-related family protein [Populus trichocarpa]
632	692	gi|731388721	116.88	25	5	3		18965	PREDICTED: translationally-controlled tumor protein homolog [Vitis vinifera]
976	17094	gi|224065699	116.83	12	6	6	Carbamidomethylation	75559	Cell division protein ftsH [Populus trichocarpa]
452	369	gi|359492927	116.54	27	6	2		28650	PREDICTED: 14-3-3-like protein GF14 kappa [Vitis vinifera]
1034	17453	gi|224088276	116.41	11	4	1	Carbamidomethylation	50030	leucine aminopeptidase family protein [Populus trichocarpa]
618	17126	gi|224141487	116.19	13	7	1	Carbamidomethylation	52967	UDP-glucose 6-dehydrogenase family protein [Populus trichocarpa]

965	17353	gi|224142207	116.03	9	4	2	Carbamidomethylation	65147	hypothetical protein POPTR_0018s09380g [Populus trichocarpa]
695	619	gi|225453250	115.98	8	4	4		69341	PREDICTED: NAD-dependent malic enzyme 62 kDa isoform mitochondrial isoform X2 [Vitis vinifera]
857	637	gi|225431563	115.9	12	6	1	Carbamidomethylation	51250	PREDICTED: UTP--glucose-1-phosphate uridylyltransferase isoform X1 [Vitis vinifera]
784	17223	gi|224139104	115.77	16	6	6	Carbamidomethylation	33044	pyridoxin biosynthesis PDX1-like protein 3 [Populus trichocarpa]
1049	398	gi|731405921	115.77	21	6	6	Carbamidomethylation	34871	PREDICTED: phytochrome-associated serine/threonine-protein phosphatase [Vitis vinifera]
910	17018	gi|566148676	115.74	11	7	1	Carbamidomethylation	77001	Glycyl-tRNA synthetase family protein [Populus trichocarpa]
1059	699	gi|225435309	115.66	9	6	2	Carbamidomethylation	82345	PREDICTED: 4-hydroxy-3-methylbut-2-en-1-yl diphosphate synthase chloroplastic [Vitis vinifera]
823	964	gi|225438529	115.52	27	7	6		27683	PREDICTED: probable ATP synthase 24 kDa subunit mitochondrial [Vitis vinifera]
119	859	gi|225429614	115.48	26	4	4	Oxidation (M)	17293	PREDICTED: 17.3 kDa class II heat shock protein [Vitis vinifera]
768	17070	gi|224064707	115.42	11	6	3	Carbamidomethylation	58836	mitochondrial aldehyde dehydrogenase family protein [Populus trichocarpa]
534	17014	gi|224140653	115.39	14	7	3	Carbamidomethylation; Oxidation (M)	61182	phosphoglycerate mutase family protein [Populus trichocarpa]
345	17005	gi|566146555	115.29	35	8	3		23812	60S ribosomal protein L13 [Populus trichocarpa]
1012	471	gi|225433575	115.24	7	5	4	Carbamidomethylation	105215	PREDICTED: probable glucan 1 3-alpha-glucosidase [Vitis vinifera]

755	466	gi|225426743	115.15	23	6	1	Carbamidomethylation	33675	PREDICTED: bifunctional dTDP-4-dehydrorhamnose 3 5-epimerase/dTDP-4-dehydrorhamnose reductase [Vitis vinifera]
1170	597	gi|225441924	115.1	13	4	1	Carbamidomethylation	51126	PREDICTED: eukaryotic translation initiation factor 3 subunit E [Vitis vinifera]
218	18507	gi|566242200	115.02	14	5	3		19093	hypothetical protein POPTR_0019s12370g partial [Populus trichocarpa]
790	17298	gi|224063082	114.94	22	5	5		28992	Photosystem II 22 kDa family protein [Populus trichocarpa]
748	727	gi|225464809	114.82	13	6	3	Carbamidomethylation	56995	PREDICTED: gamma aminobutyrate transaminase 1 mitochondrial-like [Vitis vinifera]
734	16882	gi|224053613	114.71	19	8	8	Carbamidomethylation	49072	Eukaryotic peptide chain release factor subunit 1-2 family protein [Populus trichocarpa]
1121	17015	gi|224129596	114.69	19	7	1	Carbamidomethylation	45020	Succinyl-CoA ligase beta-chain family protein [Populus trichocarpa]
473	17412	gi|566189935	114.69	14	5	1	Carbamidomethylation	29602	ribosomal protein S3a [Populus trichocarpa]
950	508	gi|731432625	114.68	5	5	1	Carbamidomethylation	121307	PREDICTED: protein transport protein Sec24-like At4g32640 [Vitis vinifera]
412	774	gi|225444063	114.67	16	7	2	Carbamidomethylation	44423	PREDICTED: obg-like ATPase 1 [Vitis vinifera]
738	651	gi|225434329	114.62	16	8	1		57685	PREDICTED: trans-cinnamate 4-monooxygenase [Vitis vinifera]
376	17083	gi|224091827	114.61	29	7	1	Carbamidomethylation	20659	60S ribosomal protein L11-2 [Populus trichocarpa]
439	642	gi|225432758	114.56	18	6	2	Carbamidomethylation	29774	PREDICTED: 40S ribosomal protein S3a-1 [Vitis vinifera]
887	17046	gi|224085984	114.53	11	6	1	Carbamidomethylation	61382	multi-copper oxidase type 1 family protein [Populus trichocarpa]
828	17528	gi|566162704	114.3	32	4	2	Carbamidomethylation	18629	hypothetical protein POPTR_0003s14870g [Populus trichocarpa]

697	17210	gi|224136368	114.28	6	6	1		107885	110 kDa 4SNc-Tudor domain family protein [Populus trichocarpa]
628	357	gi|731399677	114.2	9	9	1	Carbamidomethylation	102437	PREDICTED: protein argonaute 4A-like [Vitis vinifera]
308	669	gi|731386337	114.05	23	5	4	Carbamidomethylation	20640	PREDICTED: serine/arginine-rich splicing factor RSZ22 isoform X2 [Vitis vinifera]
962	600	gi|359490767	113.85	24	4	1		23070	PREDICTED: nascent polypeptide-associated complex subunit alpha-like protein 2 [Vitis vinifera]
746	17008	gi|566186274	113.77	25	7	2	Carbamidomethylation	29153	proliferating cell nuclear antigen family protein [Populus trichocarpa]
523	380	gi|225459292	113.74	26	7	3		28739	PREDICTED: 14-3-3 protein 7 [Vitis vinifera]
692	592	gi|225437537	113.69	34	6	6		22019	PREDICTED: GTP-binding protein SAR1A [Vitis vinifera]
1156	17405	gi|566183896	113.61	15	4	1	Carbamidomethylation	35738	TGF-beta receptor-interacting protein 1 [Populus trichocarpa]
1027	691	gi|731397286	113.5	10	5	1	Carbamidomethylation	56371	PREDICTED: inositol-3-phosphate synthase [Vitis vinifera]
1070	17433	gi|566214954	113.5	13	4	3	Carbamidomethylation	39277	hypothetical protein POPTR_0018s09170g [Populus trichocarpa]
961	1208	gi|225424530	113.17	15	4	1		36021	PREDICTED: eukaryotic translation initiation factor 3 subunit I [Vitis vinifera]
947	16960	gi|566211039	113.14	6	6	6		124467	DegP protease family protein [Populus trichocarpa]
766	17148	gi|224054150	113.1	22	6	5	Carbamidomethylation	38004	transducin family protein [Populus trichocarpa]
912	628	gi|225435754	112.87	8	7	2	Carbamidomethylation	123396	PREDICTED: importin-5 [Vitis vinifera]
687	424	gi|225428772	112.8	7	7	2		121098	PREDICTED: presequence protease 2 chloroplastic/mitochondrial-like [Vitis vinifera]

510	17173	gi|566149424	112.78	16	6	6	Oxidation (M)	46427	oligouridylate-binding family protein [Populus trichocarpa]
435	17211	gi|224135485	112.71	11	6	2	Carbamidomethylation	58870	glycosyl hydrolase family 1 family protein [Populus trichocarpa]
779	17297	gi|224141065	112.65	10	5	1		58604	mitochondrial processing peptidase beta subunit family protein [Populus trichocarpa]
1088	17535	gi|566180192	112.63	23	4	4		27792	hypothetical protein POPTR_0007s08020g [Populus trichocarpa]
1108	438	gi|225425318	112.47	7	6	1		108282	PREDICTED: DNA replication licensing factor MCM2 [Vitis vinifera]
1072	935	gi|225462922	112.34	14	5	1	Carbamidomethylation	46982	PREDICTED: 26S proteasome non-ATPase regulatory subunit 11 homolog [Vitis vinifera]
582	1727	gi|225465837	112.31	6	5	3	Carbamidomethylation	46787	PREDICTED: protein ASPARTIC PROTEASE IN GUARD CELL 2 [Vitis vinifera]
968	17512	gi|566158761	112.3	22	6	2	Carbamidomethylation; Oxidation (M)	29101	cyclophilin family protein [Populus trichocarpa]
939	529	gi|731399749	112.2	18	5	5	Carbamidomethylation	44581	PREDICTED: GDHB glutamate dehydrogenase isoform X1 [Vitis vinifera]
853	773	gi|225440478	112.12	15	3	2	Carbamidomethylation	35390	PREDICTED: aldo-keto reductase family 4 member C9-like [Vitis vinifera]
469	17131	gi|224057882	111.83	10	7	1		89495	outer membrane family protein [Populus trichocarpa]
760	458	gi|359475502	111.59	18	5	1	Carbamidomethylation	37652	PREDICTED: malate dehydrogenase glyoxysomal [Vitis vinifera]
797	664	gi|731403303	111.55	32	4	2	Carbamidomethylation	15026	PREDICTED: 40S ribosomal protein S12 [Vitis vinifera]
367	17233	gi|224065421	111.46	9	5	1		64078	hypothetical protein POPTR_0002s24970g [Populus trichocarpa]

1033	17416	gi|671741939	111.45	14	5	5		56223	acetyl-CoA carboxylase carboxyltransferase beta subunit (chloroplast) [Camellia grandibracteata]
1199	17016	gi|566148627	111.4	7	6	1	Carbamidomethylation	107277	hypothetical protein POPTR_0001s12700g [Populus trichocarpa]
967	17498	gi|566160463	111.33	14	4	4		49456	hypothetical protein POPTR_0003s02420g [Populus trichocarpa]
505	882	gi|225449292	111.19	23	6	1	Oxidation (M)	17090	PREDICTED: 18.2 kDa class I heat shock protein [Vitis vinifera]
747	1030	gi|225453246	111.1	9	4	2		69276	PREDICTED: dynamin-related protein 1E [Vitis vinifera]
350	17618	gi|224102515	110.95	16	5	4		29608	porin family protein [Populus trichocarpa]
867	17635	gi|224092266	110.92	8	4	1	Carbamidomethylation	63096	hypothetical protein POPTR_0006s25280g [Populus trichocarpa]
241	446	gi|225465463	110.88	32	7	1	Carbamidomethylation	23516	PREDICTED: 60S ribosomal protein L13a-4 [Vitis vinifera]
1040	1597	gi|526118004	110.86	13	5	5		47748	probable E3 ubiquitin-protein ligase HERC1-like [Vitis vinifera]
730	16961	gi|566166593	110.79	6	7	2		122467	hypothetical protein POPTR_0004s14960g [Populus trichocarpa]
936	731	gi|225449971	110.69	5	5	3	Carbamidomethylation	121958	PREDICTED: protein transport protein SEC31 homolog B isoform X2 [Vitis vinifera]
1110	17066	gi|566167939	110.68	9	6	2		84308	hypothetical protein POPTR_0004s22010g [Populus trichocarpa]
920	499	gi|731416612	110.64	13	6	6		63619	PREDICTED: probable nucleolar protein 5-2 [Vitis vinifera]
532	17587	gi|566184208	110.56	16	4	4	Carbamidomethylation	27210	hypothetical protein POPTR_0008s15820g [Populus trichocarpa]
1061	17255	gi|566259528	110.47	5	5	1	Carbamidomethylation	119647	transport Sec24 family protein [Populus trichocarpa]

971	17256	gi|224125002	110.44	9	4	3	Carbamidomethylation	59410	importin alpha-1 subunit family protein [Populus trichocarpa]
389	376	gi|526117723	110.38	14	6	1	Carbamidomethylation	56980	catalase [Vitis vinifera]
820	17516	gi|224061539	110.25	23	5	1		21920	hypothetical protein POPTR_0001s45810g [Populus trichocarpa]
742	743	gi|359482848	110.24	10	4	1		43630	PREDICTED: nucleosome assembly protein 1;4 [Vitis vinifera]
1371	17442	gi|566152994	110.09	11	5	2	Carbamidomethylation	57135	hypothetical protein POPTR_0001s35790g [Populus trichocarpa]
990	17452	gi|566147835	109.9	19	4	3		26392	hypothetical protein POPTR_0001s07920g partial [Populus trichocarpa]
972	758	gi|225430549	109.72	8	4	2	Carbamidomethylation	82761	PREDICTED: eukaryotic translation initiation factor 3 subunit B [Vitis vinifera]
942	17188	gi|224101523	109.58	11	5	1	Carbamidomethylation	54092	Galactokinase family protein [Populus trichocarpa]
597	633	gi|225444649	109.57	16	4	4		26396	PREDICTED: 20 kDa chaperonin chloroplastic [Vitis vinifera]
862	490	gi|225446595	109.53	33	5	1	Carbamidomethylation	17220	PREDICTED: ubiquitin-conjugating enzyme E2 36 [Vitis vinifera]
97	410	gi|359475042	109.45	25	6	1	Carbamidomethylation; Oxidation (M)	18313	PREDICTED: peptidyl-prolyl cis-trans isomerase [Vitis vinifera]
153	17575	gi|566170929	109.14	12	4	1	Carbamidomethylation	36394	hypothetical protein POPTR_0005s12070g [Populus trichocarpa]
558	17266	gi|224104631	109	18	5	1		27319	cytosolic ascorbate peroxidase family protein [Populus trichocarpa]
1026	590	gi|225424316	108.89	12	5	2	Carbamidomethylation	63641	PREDICTED: phosphoglucomutase cytoplasmic [Vitis vinifera]
1204	17396	gi|224119992	108.89	4	4	2	Carbamidomethylation	143158	hypothetical protein POPTR_0012s13100g [Populus trichocarpa]

1022	720	gi|359476926	108.83	18	3	1	Carbamidomethylation	18454	PREDICTED: photosystem I reaction center subunit N chloroplastic [Vitis vinifera]
1405	735	gi|225455902	108.8	13	5	1	Carbamidomethylation	65334	PREDICTED: serine/threonine-protein phosphatase 2A 65 kDa regulatory subunit A beta isoform [Vitis vinifera]
248	17149	gi|224081102	108.68	51	7	3	Carbamidomethylation	15406	hypothetical protein POPTR_0005s07340g [Populus trichocarpa]
627	545	gi|526117441	108.62	5	6	1		101670	lipoxygenase [Vitis vinifera]
1165	957	gi|731389741	108.59	12	7	6	Carbamidomethylation	69804	PREDICTED: probable methyltransferase PMT3 [Vitis vinifera]
945	591	gi|225456270	108.43	10	6	3	Carbamidomethylation	62432	PREDICTED: nucleolar protein 56-like [Vitis vinifera]
873	17254	gi|224075058	108.42	11	5	1	Carbamidomethylation	53712	6-phosphogluconate dehydrogenase family protein [Populus trichocarpa]
914	593	gi|731412938	108.29	4	5	1	Carbamidomethylation	155024	PREDICTED: probable phosphoribosylformylglycinamidine synthase chloroplastic/mitochondrial [Vitis vinifera]
723	655	gi|225437420	108.22	26	4	4	Carbamidomethylation	21061	PREDICTED: 60S ribosomal protein L18-2 [Vitis vinifera]
620	16954	gi|224076908	108.02	18	7	1		40750	hypothetical protein POPTR_0004s06400g [Populus trichocarpa]
727	612	gi|225423953	107.9	17	5	2		41947	PREDICTED: ubiquitin receptor RAD23c isoform X2 [Vitis vinifera]
988	462	gi|225463163	107.87	15	7	1	Carbamidomethylation	60505	PREDICTED: T-complex protein 1 subunit gamma [Vitis vinifera]
1267	1093	gi|225438067	107.83	14	5	1	Carbamidomethylation	45402	PREDICTED: succinyl-CoA ligase [ADP-forming] subunit beta mitochondrial [Vitis vinifera]
1002	17552	gi|566205234	107.81	23	4	1		24533	hypothetical protein POPTR_0015s00570g [Populus trichocarpa]

724	646	gi|225456268	107.77	20	5	2	Carbamidomethylation	26974	PREDICTED: adenylate kinase 4 [Vitis vinifera]
483	17506	gi|566243399	107.34	13	5	1		33846	hypothetical protein POPTR_0019s13040g [Populus trichocarpa]
449	17658	gi|224092113	107.22	10	5	2		40471	carbon-nitrogen hydrolase family protein [Populus trichocarpa]
1060	577	gi|731438110	107.09	9	5	5	Carbamidomethylation	88481	PREDICTED: probable cytosolic oligopeptidase A [Vitis vinifera]
772	832	gi|225461287	107.07	28	4	4	Carbamidomethylation	24076	PREDICTED: cytochrome b6-f complex iron-sulfur subunit 1 chloroplastic [Vitis vinifera]
1032	686	gi|225429234	107.01	21	5	5		34319	PREDICTED: mitochondrial import receptor subunit TOM40-1 [Vitis vinifera]
1008	932	gi|225450626	106.91	12	5	1	Carbamidomethylation	39269	PREDICTED: probable protein disulfide-isomerase A6 [Vitis vinifera]
796	17473	gi|224086589	106.82	11	4	1	Carbamidomethylation	56517	POLLEN-PISTIL INTERACTION 2 family protein [Populus trichocarpa]
938	552	gi|731438607	106.77	12	5	4		46196	PREDICTED: LOW QUALITY PROTEIN: hsp70-Hsp90 organizing protein 3-like [Vitis vinifera]
821	17044	gi|566194782	106.57	10	7	1	Carbamidomethylation	75679	NAD-dependent epimerase/dehydratase family protein [Populus trichocarpa]
904	601	gi|731390491	106.54	7	4	2		84964	PREDICTED: protein transport protein SEC23-like [Vitis vinifera]
996	690	gi|359473310	106.46	25	5	1	Carbamidomethylation	35042	PREDICTED: serine/threonine-protein phosphatase PP2A-2 catalytic subunit [Vitis vinifera]
986	17093	gi|224067984	106.36	12	6	2		68220	dynamin-like protein B [Populus trichocarpa]
602	17620	gi|224058427	106.35	15	5	2		28418	reticulon family protein [Populus trichocarpa]
977	17342	gi|224077760	106.23	33	5	1	Carbamidomethylation	15880	40S ribosomal protein S19 [Populus trichocarpa]

570	17370	gi|566192956	106.2	13	5	1	Carbamidomethylation	44225	hypothetical protein POPTR_0011s00650g [Populus trichocarpa]
754	463	gi|731398611	106.15	18	7	1	Carbamidomethylation	43372	PREDICTED: proliferation-associated protein 2G4 [Vitis vinifera]
834	17225	gi|566211906	106.06	7	5	2		90312	dynamin-like family protein [Populus trichocarpa]
624	675	gi|225442801	105.97	51	5	1		14493	PREDICTED: probable histone H2A variant 3 [Vitis vinifera]
252	493	gi|225436843	105.93	46	5	1		14570	PREDICTED: histone H2AX [Vitis vinifera]
749	17737	gi|224096586	105.83	9	5	1		45989	eukaryotic translation initiation factor 4A family protein [Populus trichocarpa]
689	1063	gi|359491801	105.76	6	5	5		83367	PREDICTED: dolichyl-diphosphooligosaccharide--protein glycosyltransferase subunit STT3B [Vitis vinifera]
1420	1009	gi|359480225	105.68	19	5	1	Carbamidomethylation	27397	PREDICTED: peptidyl-prolyl cis-trans isomerase [Vitis vinifera]
691	422	gi|731439184	105.56	10	7	7		109426	PREDICTED: 26S proteasome non-ATPase regulatory subunit 1 homolog A [Vitis vinifera]
925	17124	gi|566259876	105.53	28	6	1	Carbamidomethylation	23976	hypothetical protein POPTR_0022s00350g [Populus trichocarpa]
985	609	gi|731392506	105.41	4	5	2	Carbamidomethylation	129324	PREDICTED: carbamoyl-phosphate synthase large chain chloroplastic [Vitis vinifera]
1001	17374	gi|566147333	105.4	5	6	3		100058	hypothetical protein POPTR_0001s05320g [Populus trichocarpa]
1195	844	gi|526117645	105.33	4	5	2		153182	magnesium chelatase H subunit [Vitis vinifera]
603	1264	gi|225442963	105.33	9	4	4		35176	PREDICTED: methyl-CpG-binding domain-containing protein 11 [Vitis vinifera]
1004	17608	gi|224131618	105.31	20	4	4		26206	dienelactone hydrolase family protein [Populus trichocarpa]

956	17344	gi|224125422	105.29	10	6	4	Carbamidomethylation	56543	hypothetical protein POPTR_0013s03070g [Populus trichocarpa]
605	934	gi|225430153	105.24	51	5	1		14274	PREDICTED: probable histone H2A variant 3 [Vitis vinifera]
1044	17789	gi|566172819	105.15	8	4	2	Carbamidomethylation	47443	hypothetical protein POPTR_0005s23110g [Populus trichocarpa]
731	17469	gi|224079472	105.11	11	4	4		47114	hypothetical protein POPTR_0004s08560g [Populus trichocarpa]
154	899	gi|225431269	105.03	10	3	1	Carbamidomethylation	36580	PREDICTED: peroxidase 72 [Vitis vinifera]
844	629	gi|359492254	104.96	19	7	1	Carbamidomethylation	40876	PREDICTED: ferredoxin--NADP reductase leaf-type isozyme chloroplastic [Vitis vinifera]
1099	1376	gi|225438619	104.87	36	4	1	Carbamidomethylation	12309	PREDICTED: 60S ribosomal protein L30 [Vitis vinifera]
1013	693	gi|225437557	104.75	9	6	6		81022	PREDICTED: DNA replication licensing factor MCM7 [Vitis vinifera]
1346	596	gi|225466690	104.72	12	4	4		42525	PREDICTED: sedoheptulose-1 7-bisphosphatase chloroplastic [Vitis vinifera]
739	405	gi|225443308	104.66	9	7	1	Carbamidomethylation	106276	PREDICTED: coatomer subunit beta-1 [Vitis vinifera]
756	17242	gi|566182348	104.62	12	5	1	Carbamidomethylation	70505	peptidase M1 family protein [Populus trichocarpa]
941	17168	gi|224090159	104.6	4	5	2		151232	magnesium chelatase subunit family protein [Populus trichocarpa]
851	667	gi|225470804	104.58	10	4	1		45224	PREDICTED: phosphoribulokinase chloroplastic [Vitis vinifera]
1085	17222	gi|224057898	104.54	8	7	1	Carbamidomethylation	93907	hypothetical protein POPTR_0001s12380g [Populus trichocarpa]
701	700	gi|731391332	104.28	13	5	1		34474	PREDICTED: 60S ribosomal protein L5 [Vitis vinifera]

1255	17459	gi|566206568	104.17	9	4	2	Carbamidomethylation	54924	Betaine-aldehyde dehydrogenase family protein [Populus trichocarpa]
1009	18849	gi|224065507	104.17	19	3	2		15080	Photosystem I reaction center subunit IV A family protein [Populus trichocarpa]
413	784	gi|225462809	104.12	8	4	4		60994	PREDICTED: 2-hydroxyacyl-CoA lyase [Vitis vinifera]
886	17464	gi|566146625	104.12	9	4	1	Carbamidomethylation	45025	Phosphoribulokinase family protein [Populus trichocarpa]
1050	17196	gi|224121218	103.9	22	6	2	Carbamidomethylation	36046	GTP-binding protein beta chain [Populus trichocarpa]
699	746	gi|225447041	103.84	6	3	1	Carbamidomethylation	60008	PREDICTED: L-ascorbate oxidase homolog [Vitis vinifera]
1115	17000	gi|224135449	103.77	16	8	1		57629	chaperonin family protein [Populus trichocarpa]
650	17352	gi|566156897	103.73	12	4	2	Carbamidomethylation	46943	Delta-aminolevulinic acid dehydratase family protein [Populus trichocarpa]
1339	17135	gi|566177234	103.71	9	6	5	Carbamidomethylation	80627	minichromosome maintenance family protein [Populus trichocarpa]
596	17487	gi|566148625	103.55	10	4	1	Carbamidomethylation	54744	hypothetical protein POPTR_0001s12690g [Populus trichocarpa]
896	17503	gi|566165056	103.52	14	4	1	Carbamidomethylation	36365	hypothetical protein POPTR_0004s05340g [Populus trichocarpa]
877	662	gi|225447135	103.47	55	5	2	Carbamidomethylation	11288	PREDICTED: small ubiquitin-related modifier 1 [Vitis vinifera]
795	17268	gi|566211562	103.42	12	7	2	Carbamidomethylation	49984	hypothetical protein POPTR_0017s05490g [Populus trichocarpa]
684	668	gi|225451148	103.33	16	8	1		57291	PREDICTED: bifunctional 3-dehydroquinate dehydratase/shikimate dehydrogenase chloroplastic-like [Vitis vinifera]
501	769	gi|359488553	103.26	5	5	2		115409	PREDICTED: alpha-mannosidase isoform X2 [Vitis vinifera]

1015	772	gi|225464906	103.19	30	3	3		16377	PREDICTED: 40S ribosomal protein S14 [Vitis vinifera]
1065	875	gi|225452831	103.16	15	6	5		43750	PREDICTED: malate dehydrogenase chloroplastic-like [Vitis vinifera]
686	711	gi|731416002	103.11	12	5	2	Carbamidomethylation	46432	PREDICTED: ATP-citrate synthase alpha chain protein 2 [Vitis vinifera]
1077	17382	gi|566183730	103.08	12	4	1	Carbamidomethylation	48131	hypothetical protein POPTR_0008s13180g [Populus trichocarpa]
550	569	gi|225453215	103.02	27	7	2		23975	PREDICTED: 60S ribosomal protein L13-1 [Vitis vinifera]
1023	17641	gi|566204499	103.02	10	3	2		47320	thioredoxin family protein [Populus trichocarpa]
767	526	gi|225448739	102.86	12	5	1		69044	PREDICTED: leukotriene A-4 hydrolase homolog [Vitis vinifera]
668	17320	gi|566189392	102.81	18	6	6		27258	hypothetical protein POPTR_0010s06320g [Populus trichocarpa]
875	17869	gi|566168995	102.74	22	3	1		19031	callus protein P23 [Populus trichocarpa]
812	947	gi|225463556	102.71	4	4	1	Carbamidomethylation	125024	PREDICTED: protein TOPLESS [Vitis vinifera]
903	17449	gi|224078584	102.61	15	4	2		33150	glyoxalase I homolog family protein [Populus trichocarpa]
156	925	gi|225465425	102.6	8	4	3	Carbamidomethylation	52683	PREDICTED: uncharacterized protein LOC100241465 [Vitis vinifera]
560	17536	gi|566178862	102.56	11	5	4		34943	hypothetical protein POPTR_0007s00300g [Populus trichocarpa]
263	559	gi|225433548	102.49	29	7	1	Carbamidomethylation	23525	PREDICTED: 60S ribosomal protein L13a-4 [Vitis vinifera]
1443	854	gi|731402959	102.48	15	4	4		35210	PREDICTED: probable quinone oxidoreductase [Vitis vinifera]
1047	606	gi|731438689	102.31	11	4	1		44313	PREDICTED: FAM10 family protein At4g22670 [Vitis vinifera]

662	17164	gi|566147610	102.1	28	7	3	Carbamidomethylation	20417	hypothetical protein POPTR_0001s06720g [Populus trichocarpa]
1134	676	gi|731421194	101.88	26	4	2	Carbamidomethylation	20876	PREDICTED: 40S ribosomal protein S13 [Vitis vinifera]
1064	1127	gi|731397334	101.76	7	4	3		73259	PREDICTED: apoptotic chromatin condensation inducer in the nucleus isoform X2 [Vitis vinifera]
916	703	gi|731388929	101.72	14	7	2	Carbamidomethylation	56541	PREDICTED: bifunctional 3-dehydroquinate dehydratase/shikimate dehydrogenase chloroplastic isoform X2 [Vitis vinifera]
898	17332	gi|566165023	101.68	8	5	1	Carbamidomethylation	60040	hypothetical protein POPTR_0004s05160g [Populus trichocarpa]
979	17589	gi|224055291	101.53	8	4	1		56959	hypothetical protein POPTR_0001s28140g [Populus trichocarpa]
810	1238	gi|225425166	101.5	12	3	3		39489	PREDICTED: pyruvate dehydrogenase E1 component subunit beta-1 mitochondrial [Vitis vinifera]
1373	785	gi|225459806	101.42	7	4	1		57322	PREDICTED: T-complex protein 1 subunit beta [Vitis vinifera]
1212	685	gi|225459808	101.41	8	5	1		60137	PREDICTED: L-ascorbate oxidase homolog [Vitis vinifera]
1200	723	gi|731370529	101.35	9	4	1	Carbamidomethylation	66006	PREDICTED: serine/threonine-protein phosphatase 2A 65 kDa regulatory subunit A beta isoform-like [Vitis vinifera]
704	17569	gi|566167161	101.1	11	4	1	Carbamidomethylation	40240	hypothetical protein POPTR_0004s18030g [Populus trichocarpa]
741	17252	gi|224075976	101.09	18	5	5	Carbamidomethylation	31893	hypothetical protein POPTR_0003s21070g [Populus trichocarpa]
917	549	gi|225430366	101.06	7	6	2	Carbamidomethylation	108100	PREDICTED: chaperone protein ClpB4 mitochondrial [Vitis vinifera]

1149	17458	gi|224105455	101.06	10	4	1	Carbamidomethylation	51957	chloroplast biogenesis family protein [Populus trichocarpa]
1403	18383	gi|224104629	100.95	7	4	1		46345	seed maturation protein PM37 [Populus trichocarpa]
744	17558	gi|224127346	100.68	11	4	1		36420	arginase family protein [Populus trichocarpa]
826	683	gi|225456085	100.67	9	6	6		57173	PREDICTED: eukaryotic peptide chain release factor GTP-binding subunit ERF3A [Vitis vinifera]
1252	17293	gi|566206565	100.63	19	5	5	Carbamidomethylation	39138	hypothetical protein POPTR_0015s08180g [Populus trichocarpa]
1146	599	gi|225459126	100.46	9	5	1		68420	PREDICTED: glucose-6-phosphate isomerase 1 chloroplastic [Vitis vinifera]
1272	423	gi|225434133	100.4	15	5	2	Carbamidomethylation	40613	PREDICTED: cysteine synthase chloroplastic/chromoplastic [Vitis vinifera]
1283	1226	gi|731439514	100.35	15	4	1	Carbamidomethylation	41530	PREDICTED: acetyl-CoA acetyltransferase cytosolic 1 isoform X4 [Vitis vinifera]
672	816	gi|526118026	100.32	13	4	4	Carbamidomethylation	55487	5-enolpyruvylshikimate-3-phosphate synthase [Vitis vinifera]
339	1257	gi|225430939	100.28	22	6	5	Carbamidomethylation	33557	PREDICTED: isopentenyl-diphosphate Delta-isomerase I [Vitis vinifera]
1141	814	gi|225433414	100.21	14	4	4		36594	PREDICTED: probable voltage-gated potassium channel subunit beta [Vitis vinifera]
750	678	gi|731422506	100.12	4	4	1	Carbamidomethylation	125112	PREDICTED: protein TOPLESS isoform X4 [Vitis vinifera]
224	17157	gi|566173207	100.12	51	7	1	Carbamidomethylation	15284	hypothetical protein POPTR_0005s25530g [Populus trichocarpa]
787	17625	gi|566204315	100.05	6	5	3		68355	hypothetical protein POPTR_0014s14690g [Populus trichocarpa]
978	17574	gi|566178300	99.9	10	4	1	Carbamidomethylation	45519	hypothetical protein POPTR_0006s26630g [Populus trichocarpa]

1076	1012	gi|225430650	99.88	20	6	6	Carbamidomethylation; Oxidation (M)	43927	PREDICTED: pyruvate dehydrogenase E1 component subunit beta-3 chloroplastic [Vitis vinifera]
902	18232	gi|566187006	99.72	7	2	2		50172	hypothetical protein POPTR_0009s08570g [Populus trichocarpa]
943	17401	gi|566160244	99.63	3	4	3	Carbamidomethylation	181683	hypothetical protein POPTR_0003s01280g [Populus trichocarpa]
640	829	gi|225436257	99.53	12	4	4		29530	PREDICTED: chlorophyll a-b binding protein 8 chloroplastic [Vitis vinifera]
1304	1117	gi|225455336	99.31	6	3	2	Carbamidomethylation	96439	PREDICTED: importin subunit beta-1 [Vitis vinifera]
1094	1322	gi|225424805	99.21	11	4	2	Carbamidomethylation	43702	PREDICTED: pyruvate dehydrogenase E1 component subunit alpha mitochondrial [Vitis vinifera]
1261	17790	gi|566149831	99.02	6	3	1	Carbamidomethylation	70825	malate oxidoreductase family protein [Populus trichocarpa]
1078	17430	gi|224055647	98.99	16	5	3		32125	succinate dehydrogenase iron-sulfur protein subunit [Populus trichocarpa]
922	17240	gi|566202791	98.96	11	5	4	Carbamidomethylation	59276	t-complex polypeptide 1 family protein [Populus trichocarpa]
856	537	gi|225465200	98.94	29	6	1	Carbamidomethylation	23863	PREDICTED: ras-related protein RABA2a [Vitis vinifera]
1340	17514	gi|566170365	98.93	12	4	2	Carbamidomethylation	39182	hypothetical protein POPTR_0005s09400g partial [Populus trichocarpa]
801	17315	gi|224079274	98.9	14	5	2		41124	ubiquitin family protein [Populus trichocarpa]
1193	1310	gi|731401385	98.89	7	3	3		36064	PREDICTED: uncharacterized protein At5g02240 [Vitis vinifera]
1118	605	gi|731399856	98.88	5	4	2		104341	PREDICTED: villin-2 [Vitis vinifera]
1298	17873	gi|224058705	98.84	29	3	3	Carbamidomethylation	18641	cytochrome c oxidase-related family protein [Populus trichocarpa]

1086	908	gi|225426312	98.77	11	5	1		68263	PREDICTED: dynamin-related protein 5A [Vitis vinifera]
707	866	gi|225425304	98.76	44	3	3	Carbamidomethylation	9611	PREDICTED: 40S ribosomal protein S27-2 [Vitis vinifera]
1218	748	gi|225441549	98.66	8	4	3		67018	PREDICTED: DEAD-box ATP-dependent RNA helicase 37-like [Vitis vinifera]
273	17510	gi|224078822	98.58	40	4	1		13770	hypothetical protein POPTR_0004s03100g [Populus trichocarpa]
1815	2169	gi|731371107	98.35	6	4	4		89995	PREDICTED: probable splicing factor 3A subunit 1 [Vitis vinifera]
1301	17622	gi|224060455	98.13	5	3	1	Carbamidomethylation	85898	transport family protein [Populus trichocarpa]
946	766	gi|225436215	98	7	3	2	Carbamidomethylation	70115	PREDICTED: lysine--tRNA ligase-like [Vitis vinifera]
546	969	gi|225426753	97.97	11	7	2	Carbamidomethylation; Oxidation (M)	81719	PREDICTED: primary amine oxidase [Vitis vinifera]
1097	17414	gi|566206247	97.91	26	5	1		19365	hypothetical protein POPTR_0015s06660g [Populus trichocarpa]
737	17890	gi|566181315	97.86	15	3	1		27367	malate dehydrogenase family protein partial [Populus trichocarpa]
1169	17395	gi|224074998	97.79	5	4	4		100185	hypothetical protein POPTR_0003s13040g [Populus trichocarpa]
1019	759	gi|731412750	97.75	8	4	4		62116	PREDICTED: adenylosuccinate lyase [Vitis vinifera]
1392	1329	gi|731405046	97.74	9	5	1	Carbamidomethylation	86013	PREDICTED: DNA replication licensing factor MCM3 homolog 2 [Vitis vinifera]
706	729	gi|731421236	97.69	13	5	2		37446	PREDICTED: arginase 1 mitochondrial [Vitis vinifera]
901	1006	gi|225439858	97.5	21	3	1		17634	PREDICTED: 60S ribosomal protein L23a-like [Vitis vinifera]
1282	792	gi|225465764	97.35	15	5	5	Carbamidomethylation	43403	PREDICTED: UDP-D-apiose/UDP-D-xylose synthase 2 [Vitis vinifera]

716	750	gi|225457502	97.28	14	3	1	Carbamidomethylation	35357	PREDICTED: probable succinyl-CoA ligase [ADP-forming] subunit alpha mitochondrial [Vitis vinifera]
718	811	gi|225456404	97.13	9	3	1	Carbamidomethylation	50437	PREDICTED: glutamate-1-semialdehyde 2 1-aminomutase chloroplastic [Vitis vinifera]
997	17771	gi|566148911	97.08	7	3	1	Carbamidomethylation	48782	hypothetical protein POPTR_0001s14280g [Populus trichocarpa]
1219	17681	gi|224140109	96.97	22	4	2		17267	hypothetical protein POPTR_0016s08070g [Populus trichocarpa]
639	17842	gi|224057968	96.95	17	3	1	Carbamidomethylation	24349	photosystem I chain III family protein [Populus trichocarpa]
1369	17917	gi|566206287	96.91	13	4	2		35464	ATP synthase gamma chain family protein [Populus trichocarpa]
1087	497	gi|225443506	96.82	13	4	4	Carbamidomethylation	46436	PREDICTED: magnesium-chelatase subunit ChlI chloroplastic-like [Vitis vinifera]
571	17844	gi|566198902	96.67	31	3	1		15349	hypothetical protein POPTR_0013s01890g [Populus trichocarpa]
1175	848	gi|225427017	96.65	9	3	3		37604	PREDICTED: L-lactate dehydrogenase A isoform X2 [Vitis vinifera]
1494	1243	gi|225438781	96.58	7	4	1	Carbamidomethylation	71362	PREDICTED: polyadenylate-binding protein 2 [Vitis vinifera]
831	623	gi|731385630	96.57	5	4	4		99551	PREDICTED: heat shock 70 kDa protein 17 [Vitis vinifera]
563	18002	gi|566185685	96.49	14	3	1	Carbamidomethylation	22408	hypothetical protein POPTR_0009s01050g [Populus trichocarpa]
974	17944	gi|224093408	96.39	17	3	1	Carbamidomethylation	18464	hypothetical protein POPTR_0007s04160g [Populus trichocarpa]
1467	17468	gi|566177699	96.23	10	4	2	Carbamidomethylation	48113	hypothetical protein POPTR_0006s23050g [Populus trichocarpa]

625	548	gi|225434863	96.06	8	6	1		88320	PREDICTED: protein TOC75-3 chloroplastic [Vitis vinifera]
283	1401	gi|731386455	95.35	2	4	2		95638	PREDICTED: sucrose synthase 7 isoform X3 [Vitis vinifera]
1329	2050	gi|731393849	95.15	7	4	4	Carbamidomethylation	56128	PREDICTED: UBP1-associated protein 2A [Vitis vinifera]
1184	721	gi|225429209	95.15	7	4	3		53142	PREDICTED: serine carboxypeptidase-like 16 [Vitis vinifera]
1269	17511	gi|566203118	94.98	20	5	5	Carbamidomethylation	27871	phosphomannomutase family protein [Populus trichocarpa]
1139	919	gi|225461703	94.93	23	5	2		24079	PREDICTED: ras-related protein RIC2 [Vitis vinifera]
1238	17916	gi|566182153	94.78	9	3	2	Carbamidomethylation	57417	Serine carboxypeptidase precursor family protein [Populus trichocarpa]
619	659	gi|225435177	94.67	12	4	1	Carbamidomethylation	27557	PREDICTED: L-ascorbate peroxidase 2 cytosolic [Vitis vinifera]
544	586	gi|731439234	94.4	8	5	2	Carbamidomethylation	74297	PREDICTED: peroxisomal acyl-coenzyme A oxidase 1 [Vitis vinifera]
830	887	gi|359479647	94.39	24	5	5	Carbamidomethylation	22491	PREDICTED: proteasome subunit beta type-2-A [Vitis vinifera]
1537	17712	gi|224120862	94.36	5	3	1	Carbamidomethylation	96570	importin beta-2 family protein [Populus trichocarpa]
728	18912	gi|671743347	94.27	37	3	3		7800	photosystem II phosphoprotein (chloroplast) [Camellia pubicosta]
1370	18119	gi|224063066	94.25	4	2	1		56188	disulfide-isomerase family protein [Populus trichocarpa]
1325	17391	gi|566182904	94.12	9	4	4	Carbamidomethylation	59760	hypothetical protein POPTR_0008s07890g [Populus trichocarpa]
1287	17504	gi|224062301	94.09	16	4	1	Carbamidomethylation	38139	hypothetical protein POPTR_0002s04790g [Populus trichocarpa]
1201	1301	gi|731426495	94.06	4	4	2		120041	PREDICTED: alpha-mannosidase [Vitis vinifera]

1309	991	gi|359494079	94.04	8	4	4	Carbamidomethylation	65102	PREDICTED: LOW QUALITY PROTEIN: 65-kDa microtubule-associated protein 1-like [Vitis vinifera]
1157	809	gi|225445206	94.02	13	4	1		45116	PREDICTED: aspartate aminotransferase cytoplasmic [Vitis vinifera]
900	17567	gi|224071575	94	9	4	2	Carbamidomethylation	65504	stress inducible family protein [Populus trichocarpa]
1229	17393	gi|566151113	94	8	4	4		76209	hypothetical protein POPTR_0001s26420g [Populus trichocarpa]
890	804	gi|225460542	93.95	10	3	1		36331	PREDICTED: heterogeneous nuclear ribonucleoprotein 1 [Vitis vinifera]
1262	716	gi|225448823	93.93	3	4	2		134756	PREDICTED: splicing factor 3B subunit 3-like [Vitis vinifera]
1248	17743	gi|566175322	93.91	11	3	2		35273	aldo/keto reductase family protein [Populus trichocarpa]
1323	17463	gi|224133304	93.9	9	4	4		60007	hypothetical protein POPTR_0015s07690g [Populus trichocarpa]
712	566	gi|731407251	93.72	4	4	1		92866	PREDICTED: phospholipase D alpha 1-like [Vitis vinifera]
495	878	gi|225452827	93.64	28	4	4		13746	PREDICTED: 60S ribosomal protein L34 [Vitis vinifera]
358	17645	gi|566146919	93.58	6	4	1		68964	hypothetical protein POPTR_0001s03040g [Populus trichocarpa]
1463	1021	gi|731389102	93.53	11	3	3	Carbamidomethylation	39883	PREDICTED: ATP-dependent (S)-NAD(P)H-hydrate dehydratase [Vitis vinifera]
852	1189	gi|731387811	93.29	8	4	1	Carbamidomethylation	41241	PREDICTED: alcohol dehydrogenase 1 [Vitis vinifera]
659	17918	gi|224063643	93.11	22	3	1	Carbamidomethylation	17863	40S ribosomal protein S11 [Populus trichocarpa]
1246	652	gi|225444828	93.1	10	5	1		57127	PREDICTED: threonine synthase chloroplastic [Vitis vinifera]

725	17566	gi|566254203	93.1	25	4	3		17971	hypothetical protein POPTR_1064s00200g [Populus trichocarpa]
1167	17260	gi|224102365	93.05	12	6	6	Carbamidomethylation	59185	T-complex protein 1 [Populus trichocarpa]
1284	17563	gi|566182449	93.02	19	4	2	Carbamidomethylation	23916	hypothetical protein POPTR_0008s05160g [Populus trichocarpa]
1079	805	gi|225432846	92.94	18	4	1	Carbamidomethylation	38533	PREDICTED: 2-methyl-6-phytyl-1 4-hydroquinone methyltransferase chloroplastic [Vitis vinifera]
1408	1732	gi|225461632	92.64	8	3	3		44771	PREDICTED: uncharacterized protein At3g15000 mitochondrial [Vitis vinifera]
1582	17911	gi|566203837	92.58	10	4	3		39770	DNAJ heat shock family protein [Populus trichocarpa]
1421	790	gi|731404581	92.53	6	3	1	Carbamidomethylation	61348	PREDICTED: apoptosis inhibitor 5 isoform X2 [Vitis vinifera]
1385	1222	gi|225457219	92.46	11	3	1	Carbamidomethylation	35220	PREDICTED: ATP synthase subunit gamma mitochondrial [Vitis vinifera]
1457	763	gi|225433440	92.41	11	4	4		53869	PREDICTED: glutathione reductase cytosolic [Vitis vinifera]
1481	1855	gi|225447510	92.36	14	4	1	Carbamidomethylation	41132	PREDICTED: acetyl-CoA acetyltransferase cytosolic 1 [Vitis vinifera]
1145	17671	gi|566183513	92.27	6	3	2		62808	hypothetical protein POPTR_0008s11770g [Populus trichocarpa]
761	913	gi|225425595	92.06	12	4	1	Carbamidomethylation	46628	PREDICTED: ATP-citrate synthase alpha chain protein 1 [Vitis vinifera]
987	653	gi|225449060	91.94	6	3	3		62143	PREDICTED: protein disulfide isomerase-like 1-4 [Vitis vinifera]
1071	17475	gi|224107361	91.89	4	5	2		128813	ADP-forming family protein [Populus trichocarpa]
504	540	gi|225449262	91.8	28	7	1		18173	PREDICTED: 18.1 kDa class I heat shock protein [Vitis vinifera]

1066	17465	gi|566170876	91.71	11	4	4	Carbamidomethylation	41487	GDP-mannose 4 family protein [Populus trichocarpa]
1113	18456	gi|566258110	91.7	14	3	1		21715	hypothetical protein POPTR_0128s00210g [Populus trichocarpa]
1235	17990	gi|224072588	91.58	12	4	4	Carbamidomethylation	30737	hypothetical protein POPTR_0003s17100g partial [Populus trichocarpa]
859	762	gi|225448367	91.29	10	3	3		34281	PREDICTED: 60S acidic ribosomal protein P0 [Vitis vinifera]
1461	1177	gi|225441573	91.28	4	4	2	Carbamidomethylation	107099	PREDICTED: eukaryotic translation initiation factor 3 subunit C [Vitis vinifera]
1333	1354	gi|731430037	91.27	9	3	3	Carbamidomethylation	43016	PREDICTED: probable cinnamyl alcohol dehydrogenase 6 [Vitis vinifera]
1211	1290	gi|225453909	90.81	17	3	3	Carbamidomethylation	24628	PREDICTED: proteasome subunit beta type-1 [Vitis vinifera]
1675	1233	gi|225448904	90.71	7	2	1		48063	PREDICTED: malate dehydrogenase [NADP] chloroplastic [Vitis vinifera]
1111	17448	gi|566149991	90.71	11	4	1	Carbamidomethylation	47475	hypothetical protein POPTR_0001s20510g [Populus trichocarpa]
1615	907	gi|731395413	90.53	18	4	3	Carbamidomethylation	31798	PREDICTED: bifunctional protein FolD 2 [Vitis vinifera]
1127	1517	gi|225445883	90.53	26	3	1	Carbamidomethylation	14777	PREDICTED: 40S ribosomal protein S12 [Vitis vinifera]
1190	17346	gi|224138722	90.51	18	5	1		39574	isocitrate dehydrogenase family protein [Populus trichocarpa]
1397	857	gi|225424598	90.5	16	3	3	Carbamidomethylation	29498	PREDICTED: proteasome subunit beta type-7-A [Vitis vinifera]
545	807	gi|225461209	90.48	17	3	1		21740	PREDICTED: NAD(P)H dehydrogenase (quinone) FQR1 [Vitis vinifera]
1281	2193	gi|225435604	90.15	15	5	5		41693	PREDICTED: enoyl-[acyl-carrier-protein] reductase [NADH] chloroplastic-like [Vitis vinifera]

934	17949	gi|224132216	90.14	27	4	1	Carbamidomethylation	11688	hypothetical protein POPTR_0014s18990g [Populus trichocarpa]
1379	17500	gi|224107863	90.07	11	4	2		43257	pyruvate dehydrogenase family protein [Populus trichocarpa]
1520	17549	gi|566187890	89.96	9	4	4	Carbamidomethylation	55453	26S proteasome regulatory subunit S3 family protein [Populus trichocarpa]
382	17604	gi|566194465	89.92	15	4	3		24921	SGS domain-containing family protein [Populus trichocarpa]
1468	715	gi|731436956	89.88	10	4	2	Carbamidomethylation	39713	PREDICTED: porphobilinogen deaminase chloroplastic [Vitis vinifera]
1407	17943	gi|566153313	89.88	4	3	1		82969	hypothetical protein POPTR_0001s37610g [Populus trichocarpa]
1181	1650	gi|225439520	89.85	9	5	2		65247	PREDICTED: pyruvate decarboxylase 2 [Vitis vinifera]
1633	1742	gi|731436287	89.68	3	2	2	Carbamidomethylation	96261	PREDICTED: histidine--tRNA ligase [Vitis vinifera]
891	710	gi|731382436	89.53	9	3	1		35672	PREDICTED: LOW QUALITY PROTEIN: isoflavone reductase-like protein [Vitis vinifera]
657	682	gi|225423491	89.53	19	5	1		29672	PREDICTED: 14-3-3-like protein GF14 iota [Vitis vinifera]
1564	1271	gi|225459587	89.51	6	3	2		55671	PREDICTED: protein disulfide-isomerase [Vitis vinifera]
371	1639	gi|526118028	89.5	10	5	5		30741	aquaporin-like [Vitis vinifera]
253	987	gi|731411833	89.39	9	2	2	Carbamidomethylation	35859	PREDICTED: nitrilase-like protein 2 [Vitis vinifera]
860	18055	gi|566207899	89.27	20	3	3		23136	hypothetical protein POPTR_0016s00400g [Populus trichocarpa]
1098	861	gi|731383682	89.16	15	3	2		23042	PREDICTED: ras-related protein Rab7 [Vitis vinifera]
1295	986	gi|225440013	89.16	17	5	1		40309	PREDICTED: isocitrate dehydrogenase [NAD] catalytic subunit 5 mitochondrial [Vitis vinifera]

923	17249	gi|566161432	89.02	6	5	2		74324	acyl-CoA oxidase family protein [Populus trichocarpa]
854	18108	gi|566183164	89.01	19	2	1		19589	hypothetical protein POPTR_0008s09660g [Populus trichocarpa]
1285	1608	gi|225452228	88.99	12	3	1	Carbamidomethylation	30877	PREDICTED: succinate dehydrogenase [ubiquinone] iron-sulfur subunit 1 mitochondrial [Vitis vinifera]
1194	17624	gi|224104329	88.81	5	3	1		96949	alpha-glucan phosphorylase family protein [Populus trichocarpa]
1209	709	gi|359484512	88.74	7	4	1		63994	PREDICTED: asparagine--tRNA ligase cytoplasmic 1 [Vitis vinifera]
1166	706	gi|225446693	88.7	10	6	4		74318	PREDICTED: ATP-dependent zinc metalloprotease FTSH 2 chloroplastic [Vitis vinifera]
1436	18027	gi|566197725	88.58	8	3	3		56765	hypothetical protein POPTR_0012s10030g [Populus trichocarpa]
1102	2044	gi|225443859	88.54	6	2	1		24656	PREDICTED: calcyclin-binding protein [Vitis vinifera]
929	17773	gi|566213322	88.54	8	3	2	Carbamidomethylation	55497	hypothetical protein POPTR_0017s14220g [Populus trichocarpa]
846	813	gi|225425356	88.53	13	3	3		32651	PREDICTED: U2 small nuclear ribonucleoprotein A' [Vitis vinifera]
1095	17619	gi|566174513	88.41	5	5	2		104323	Villin 2 family protein [Populus trichocarpa]
1418	17577	gi|566188043	88.38	17	4	1	Oxidation (M)	43097	hypothetical protein POPTR_0009s14830g [Populus trichocarpa]
814	819	gi|731423558	88.27	5	5	4	Carbamidomethylation; Oxidation (M)	120839	PREDICTED: valine--tRNA ligase isoform X2 [Vitis vinifera]
1290	796	gi|225423755	88.18	9	3	3		44272	PREDICTED: photosystem II stability/assembly factor HCF136 chloroplastic [Vitis vinifera]
1358	977	gi|225451247	88.12	27	3	3		15707	PREDICTED: 40S ribosomal protein S24-1 [Vitis vinifera]

1140	874	gi|359478691	88.09	8	4	2		92178	PREDICTED: beta-galactosidase 8 [Vitis vinifera]
845	705	gi|225428009	88.05	10	3	1	Carbamidomethylation	31769	PREDICTED: L-ascorbate peroxidase 3 peroxisomal [Vitis vinifera]
1319	18399	gi|566174518	87.9	9	2	2	Carbamidomethylation	40168	hypothetical protein POPTR_0006s04880g [Populus trichocarpa]
1231	17548	gi|224139168	87.9	9	4	1	Carbamidomethylation; Oxidation (M)	65070	pyruvate decarboxylase family protein [Populus trichocarpa]
1745	17822	gi|224074207	87.86	10	3	3	Carbamidomethylation	46983	carbamoyl phosphate synthetase a family protein [Populus trichocarpa]
636	836	gi|225458231	87.78	15	4	4	Carbamidomethylation	30646	PREDICTED: proteasome subunit alpha type-1-B [Vitis vinifera]
1395	1156	gi|225436289	87.77	18	4	1	Oxidation (M)	43082	PREDICTED: probable aldo-keto reductase 2 isoform X1 [Vitis vinifera]
804	1029	gi|359473645	87.74	3	3	1	Carbamidomethylation	66079	PREDICTED: chaperonin 60 subunit beta 4 chloroplastic [Vitis vinifera]
1159	2139	gi|225431755	87.74	5	4	1		78694	PREDICTED: glyoxysomal fatty acid beta-oxidation multifunctional protein MFP-a [Vitis vinifera]
610	17523	gi|224107106	87.71	6	5	1		79571	MULTIFUNCTIONAL family protein [Populus trichocarpa]
1133	870	gi|225443554	87.62	18	4	1		24263	PREDICTED: ras-related protein RABA1f [Vitis vinifera]
564	18110	gi|566168071	87.59	15	3	1		22042	Superoxide dismutase family protein [Populus trichocarpa]
1313	765	gi|225443272	87.56	7	4	1	Oxidation (M)	66288	PREDICTED: D-3-phosphoglycerate dehydrogenase 3 chloroplastic-like [Vitis vinifera]
1521	17602	gi|566191545	87.51	13	4	2	Carbamidomethylation	40561	hypothetical protein POPTR_0010s18770g [Populus trichocarpa]
1187	1529	gi|225432466	87.5	17	2	2	Carbamidomethylation	18476	PREDICTED: peptide methionine sulfoxide reductase B5-like [Vitis vinifera]

1035	17702	gi|566185160	87.45	4	4	4		123192	hypothetical protein POPTR_0008s21030g [Populus trichocarpa]
1483	17738	gi|224066507	87.43	7	3	1	Carbamidomethylation	43683	mRNA-binding family protein [Populus trichocarpa]
1198	695	gi|359487652	87.41	8	4	2	Carbamidomethylation	54144	PREDICTED: fumarate hydratase 1 mitochondrial [Vitis vinifera]
1069	17377	gi|224121958	87.36	8	5	2		60274	nucleolar family protein [Populus trichocarpa]
1422	17937	gi|224101127	87.27	15	3	2	Carbamidomethylation	30103	Chlorophyll a-b binding protein CP29.3 [Populus trichocarpa]
1315	17480	gi|566164885	87.13	7	4	1		81287	Vesicle-fusing ATPase family protein [Populus trichocarpa]
1278	1605	gi|225460977	87.03	13	3	1	Carbamidomethylation	20368	PREDICTED: reactive Intermediate Deaminase A chloroplastic [Vitis vinifera]
1410	18208	gi|566210556	87.02	8	3	2		40209	putative protein phosphatase [Populus trichocarpa]
1419	725	gi|225434692	86.98	4	3	1		95490	PREDICTED: alpha-glucan phosphorylase H isozyme [Vitis vinifera]
340	17987	gi|224119894	86.62	6	3	1		61014	Calnexin precursor family protein [Populus trichocarpa]
1330	1632	gi|225447707	86.58	18	4	1	Carbamidomethylation	27098	PREDICTED: uncharacterized protein LOC100263170 [Vitis vinifera]
1260	18418	gi|566234529	86.51	22	3	2	Carbamidomethylation	13103	hypothetical protein POPTR_0019s07730g [Populus trichocarpa]
1469	1178	gi|225461906	86.5	5	3	1	Carbamidomethylation	67756	PREDICTED: DNA ligase 1 [Vitis vinifera]
915	17665	gi|566230552	86.21	16	4	3		26405	vacuolar ATPase subunit E family protein [Populus trichocarpa]
918	941	gi|225456914	86.13	21	4	3	Carbamidomethylation	16350	PREDICTED: 60S ribosomal protein L27a-3 [Vitis vinifera]
1120	18196	gi|566245692	86.01	11	3	1		25488	hypothetical protein POPTR_0019s15100g [Populus trichocarpa]
1573	1120	gi|225465441	85.95	17	2	1		14675	PREDICTED: cytochrome b5 [Vitis vinifera]

674	1286	gi|731433731	85.9	8	4	2		30030	PREDICTED: glutathione S-transferase U21-like [Vitis vinifera]
1125	730	gi|731433246	85.9	5	3	3	Carbamidomethylation	78819	PREDICTED: threonine--tRNA ligase mitochondrial-like [Vitis vinifera]
791	1498	gi|225459760	85.78	12	4	4		33454	PREDICTED: thioredoxin-like protein CDSP32 chloroplastic [Vitis vinifera]
1289	17585	gi|224133188	85.77	15	4	1	Carbamidomethylation	35890	GTP-binding protein beta chain [Populus trichocarpa]
136	736	gi|225455934	85.76	19	4	2	Carbamidomethylation; Oxidation (M)	20429	PREDICTED: ribulose bisphosphate carboxylase small chain chloroplastic [Vitis vinifera]
1503	17777	gi|566153407	85.69	11	5	4		42007	ferredoxin-NADP reductase family protein [Populus trichocarpa]
1388	17389	gi|566168468	85.67	8	4	4		68078	RNase L inhibitor family protein [Populus trichocarpa]
855	18134	gi|566258065	85.59	14	2	2		16877	hypothetical protein POPTR_0133s00270g partial [Populus trichocarpa]
803	17596	gi|566171781	85.47	5	4	2		84742	hypothetical protein POPTR_0005s16660g [Populus trichocarpa]
1383	18212	gi|224057272	85.45	8	2	2		45612	aspartyl protease family protein [Populus trichocarpa]
865	17513	gi|225428380	85.35	9	5	4	Carbamidomethylation	70298	PREDICTED: SUMO-activating enzyme subunit 2 isoform X1 [Vitis vinifera]
1793	900	gi|225446797	85.29	9	4	4	Carbamidomethylation	55221	PREDICTED: switch-associated protein 70 [Vitis vinifera]
318	1530	gi|731430493	85.27	4	2	2	Carbamidomethylation	62956	PREDICTED: laccase-14-like [Vitis vinifera]
1196	717	gi|225463033	85.25	5	4	4	Carbamidomethylation	110326	PREDICTED: 116 kDa U5 small nuclear ribonucleoprotein component [Vitis vinifera]
1210	794	gi|731374652	85.19	8	3	1		58335	PREDICTED: aldehyde dehydrogenase family 2 member B4 mitochondrial [Vitis vinifera]

1599	17965	gi|224062213	85.08	31	3	3		17225	hypothetical protein POPTR_0002s04420g [Populus trichocarpa]
1795	17815	gi|566187112	85.08	12	3	3	Carbamidomethylation	38128	WD-40 repeat family protein [Populus trichocarpa]
1256	17497	gi|566205259	85.03	17	4	3		29312	hypothetical protein POPTR_0015s00690g [Populus trichocarpa]
1207	845	gi|225428011	84.93	8	5	3		61287	PREDICTED: synaptotagmin-2 [Vitis vinifera]
1233	1091	gi|225428916	84.9	11	3	2		39009	PREDICTED: probable cinnamyl alcohol dehydrogenase 1 [Vitis vinifera]
1765	17753	gi|566209835	84.85	8	3	3	Carbamidomethylation	54998	hypothetical protein POPTR_0016s12030g [Populus trichocarpa]
958	1421	gi|731386809	84.79	8	2	1		41704	PREDICTED: naringenin 2-oxoglutarate 3-dioxygenase-like [Vitis vinifera]
1546	1772	gi|225451677	84.74	4	4	1		108901	PREDICTED: staphylococcal nuclease domain-containing protein 1 [Vitis vinifera]
1116	787	gi|225450299	84.73	7	3	3		52389	PREDICTED: citrate synthase mitochondrial [Vitis vinifera]
868	17931	gi|224079093	84.71	29	3	3		12417	60S ribosomal protein L36-3 [Populus trichocarpa]
1495	17782	gi|224143559	84.68	9	3	1		44475	aspartate aminotransferase family protein [Populus trichocarpa]
614	17746	gi|566215609	84.62	4	3	2		91834	hypothetical protein POPTR_0018s13110g [Populus trichocarpa]
1396	17720	gi|566197177	84.57	6	4	3		52151	geranylgeranyl reductase family protein [Populus trichocarpa]
1682	17909	gi|224133898	84.45	7	3	3	Carbamidomethylation	53810	aspartyl aminopeptidase family protein [Populus trichocarpa]
182	17829	gi|566171611	84.42	13	3	1	Oxidation (M)	20390	Ribulose bisphosphate carboxylase small chain 1A family protein [Populus trichocarpa]

1225	1102	gi|225452700	84.39	10	4	2		29376	PREDICTED: mitochondrial outer membrane protein porin of 34 kDa [Vitis vinifera]
1213	17408	gi|566192263	84.29	14	5	5		47039	chorismate synthase family protein [Populus trichocarpa]
1681	17796	gi|566194692	84.25	11	3	1	Carbamidomethylation	32070	hypothetical protein POPTR_0011s10100g [Populus trichocarpa]
906	888	gi|225448819	84.21	24	3	3		14272	PREDICTED: 60S ribosomal protein L35 [Vitis vinifera]
1089	868	gi|225455106	84.12	9	3	3		33350	PREDICTED: protein BOBBER 1 [Vitis vinifera]
839	1022	gi|225456800	84.06	9	5	2	Carbamidomethylation	77878	PREDICTED: programmed cell death protein 4 [Vitis vinifera]
1522	856	gi|225454422	84.03	23	3	3		16418	PREDICTED: eukaryotic translation initiation factor 1A [Vitis vinifera]
1073	17694	gi|224059670	84	16	3	2		25980	60S ribosomal protein L6 [Populus trichocarpa]
1460	18068	gi|359474588	83.83	6	3	1	Carbamidomethylation	69281	PREDICTED: probable methyltransferase PMT14 [Vitis vinifera]
1051	946	gi|225429638	83.76	10	4	1		39454	PREDICTED: omega-amidase chloroplastic [Vitis vinifera]
1100	17927	gi|566171321	83.62	14	3	1		33667	pfkB-type carbohydrate kinase family protein [Populus trichocarpa]
1644	17821	gi|566182683	83.55	12	3	3		34990	hypothetical protein POPTR_0008s06540g [Populus trichocarpa]
1250	797	gi|225433894	83.43	2	4	4	Carbamidomethylation	246915	PREDICTED: U5 small nuclear ribonucleoprotein 200 kDa helicase [Vitis vinifera]
871	18472	gi|566162504	83.43	27	3	2	Carbamidomethylation	11964	hypothetical protein POPTR_0003s13600g [Populus trichocarpa]
1386	1270	gi|225437762	83.43	8	3	3	Carbamidomethylation	59105	PREDICTED: protoporphyrinogen oxidase 1 chloroplastic [Vitis vinifera]

757	17507	gi|224066959	83.41	13	4	1		29005	14-3-3-like protein GF14 omicron [Populus trichocarpa]
1274	1320	gi|731440862	83.39	25	4	4	Carbamidomethylation	15642	PREDICTED: 60S ribosomal protein L32-1 [Vitis vinifera]
829	2095	gi|225447745	83.38	11	2	2		27216	PREDICTED: chlorophyll a-b binding protein CP24 10A chloroplastic [Vitis vinifera]
1639	17708	gi|566160633	83.36	8	3	1		51164	WD-40 repeat protein MSI4 [Populus trichocarpa]
1052	903	gi|225453158	83.1	9	3	1		41234	PREDICTED: probable fructokinase-6 chloroplastic [Vitis vinifera]
1220	1380	gi|731390561	83.1	6	3	3	Carbamidomethylation	58745	PREDICTED: probable aldo-keto reductase 4 [Vitis vinifera]
1646	18314	gi|224088282	83.06	4	2	1		73935	hypothetical protein POPTR_0006s19780g [Populus trichocarpa]
1192	1503	gi|225461001	83.01	7	2	2		46284	PREDICTED: polyadenylate-binding protein RBP45-like [Vitis vinifera]
1152	1353	gi|225428047	82.98	18	3	3	Carbamidomethylation	17518	PREDICTED: SKP1-like protein 1A isoform X5 [Vitis vinifera]
1502	1087	gi|731394579	82.92	14	3	3	Carbamidomethylation	24605	PREDICTED: 60S ribosomal protein L10a-1-like [Vitis vinifera]
951	979	gi|731416996	82.84	7	3	2		46827	PREDICTED: poly(rC)-binding protein 3 [Vitis vinifera]
1894	2166	gi|359473071	82.76	4	2	2		65661	PREDICTED: bifunctional purine biosynthesis protein purH isoform X2 [Vitis vinifera]
1374	1194	gi|225464297	82.65	4	2	1	Carbamidomethylation	84117	PREDICTED: protein transport protein SEC23 [Vitis vinifera]
1424	2203	gi|731387671	82.52	7	2	2	Carbamidomethylation	39674	PREDICTED: thioredoxin reductase NTRB-like [Vitis vinifera]
1123	17626	gi|224072049	82.44	13	4	1		28211	reticulon family protein [Populus trichocarpa]
637	1507	gi|225470846	82.44	13	2	1		22027	PREDICTED: nascent polypeptide-associated complex subunit alpha-like protein 1 [Vitis vinifera]

1690	1404	gi|225437172	82.35	12	3	1		38410	PREDICTED: 2-methyl-6-phytyl-1 4-hydroquinone methyltransferase chloroplastic [Vitis vinifera]
1126	901	gi|359493854	82.35	17	3	2		20058	PREDICTED: thioredoxin M4 chloroplastic [Vitis vinifera]
1742	17697	gi|566213932	82.35	5	3	1		82500	Eukaryotic translation initiation factor 3 subunit 9 family protein [Populus trichocarpa]
629	18170	gi|566157284	82.24	4	3	1	Carbamidomethylation	48713	chloroplast nucleoid DNA-binding family protein [Populus trichocarpa]
973	17578	gi|566198207	82.24	11	4	1		32557	hypothetical protein POPTR_0012s12860g [Populus trichocarpa]
1043	1707	gi|225434191	82.15	9	3	1	Carbamidomethylation	37891	PREDICTED: 2-alkenal reductase (NADP(+)-dependent) [Vitis vinifera]
1132	17447	gi|566185959	82.05	15	4	4	Carbamidomethylation; Oxidation (M)	27367	Proteasome subunit alpha type 3 family protein [Populus trichocarpa]
969	687	gi|359490482	82.03	12	4	1		33398	PREDICTED: probable mediator of RNA polymerase II transcription subunit 36b [Vitis vinifera]
870	1352	gi|225428908	81.89	5	2	2		53765	PREDICTED: limonoid UDP-glucosyltransferase [Vitis vinifera]
1223	812	gi|731423236	81.87	7	3	2		65721	PREDICTED: monocopper oxidase-like protein SKU5 [Vitis vinifera]
1778	864	gi|731439997	81.82	17	4	2	Carbamidomethylation	33522	PREDICTED: phosphoglycolate phosphatase 1B chloroplastic [Vitis vinifera]
1191	1587	gi|225453350	81.8	16	2	2		16310	PREDICTED: nucleoside diphosphate kinase B [Vitis vinifera]
836	1099	gi|731418950	81.8	1	2	1		248350	PREDICTED: nuclear pore complex protein GP210 [Vitis vinifera]

1006	915	gi|225435203	81.76	20	3	3		15652	PREDICTED: 40S ribosomal protein S23-like [Vitis vinifera]
1683	17964	gi|566197760	81.72	26	3	3		14976	hypothetical protein POPTR_0012s10220g [Populus trichocarpa]
1574	1135	gi|225458305	81.63	6	2	1		62648	PREDICTED: glucose-6-phosphate isomerase cytosolic [Vitis vinifera]
1172	544	gi|731392981	81.46	4	5	1	Carbamidomethylation	102178	PREDICTED: linoleate 13S-lipoxygenase 2-1 chloroplastic-like isoform X2 [Vitis vinifera]
1670	2521	gi|225453076	81.45	5	3	3		80919	PREDICTED: NADH dehydrogenase [ubiquinone] iron-sulfur protein 1 mitochondrial [Vitis vinifera]
921	745	gi|731406154	81.31	2	4	4		199529	PREDICTED: brefeldin A-inhibited guanine nucleotide-exchange protein 5 [Vitis vinifera]
842	18405	gi|224095668	81.25	6	2	1		35550	hypothetical protein POPTR_0007s01850g [Populus trichocarpa]
874	17667	gi|224086942	81.13	12	4	2	Carbamidomethylation; Oxidation (M)	31883	putative esterase D family protein [Populus trichocarpa]
1291	17946	gi|224136782	81.11	23	3	1		17114	ribosomal protein S13 [Populus trichocarpa]
1751	17674	gi|224104643	81.1	5	3	1		65194	thioredoxin family protein [Populus trichocarpa]
1703	1541	gi|225453754	81.04	15	2	2	Carbamidomethylation	23373	PREDICTED: probable xyloglucan endotransglucosylase/hydrolase protein 19 [Vitis vinifera]
1479	1176	gi|225431737	81.01	26	3	3	Carbamidomethylation	14068	PREDICTED: 60S ribosomal protein L22-2 [Vitis vinifera]
1024	18339	gi|225462348	80.88	6	2	2		57728	PREDICTED: zinc finger CCCH domain-containing protein 37 isoform X2 [Vitis vinifera]
1296	17636	gi|224063625	80.87	20	4	1	Carbamidomethylation	22553	RAS 5 family protein [Populus trichocarpa]

1389	17732	gi|566198611	80.83	4	3	3		82290	hypothetical protein POPTR_0013s00230g [Populus trichocarpa]
1489	18006	gi|224141351	80.54	5	3	2		68914	Structure-specific recognition protein 1 [Populus trichocarpa]
1581	17691	gi|566181655	80.49	13	3	3		38024	hypothetical protein POPTR_0008s01070g [Populus trichocarpa]
1240	1505	gi|731426023	80.47	30	2	1		12464	PREDICTED: protein SPIRAL1-like 1 isoform X2 [Vitis vinifera]
1036	1011	gi|225435878	80.39	28	3	2		10567	PREDICTED: 10 kDa chaperonin [Vitis vinifera]
1616	17905	gi|224105715	80.29	7	3	3	Carbamidomethylation	69881	vacuolar sorting receptor protein BP-80 [Populus trichocarpa]
1215	1086	gi|731399561	80.25	2	3	2		187436	PREDICTED: transcription elongation factor SPT6 [Vitis vinifera]
1045	2109	gi|225451233	80.09	9	2	1	Carbamidomethylation	34208	PREDICTED: cysteine synthase [Vitis vinifera]
726	17935	gi|566203775	79.91	14	4	4		33272	hypothetical protein POPTR_0014s11480g [Populus trichocarpa]
745	18137	gi|224087780	79.83	4	2	1		65398	pyruvate decarboxylase family protein [Populus trichocarpa]
1354	2328	gi|225435249	79.58	13	2	2		27570	PREDICTED: proteasome subunit beta type-4 [Vitis vinifera]
1202	942	gi|225453935	79.48	12	4	4		40128	PREDICTED: 4-hydroxy-tetrahydrodipicolinate synthase chloroplastic [Vitis vinifera]
1817	17861	gi|566214720	79.42	14	3	3		27275	Eukaryotic translation initiation factor 3 subunit 11 family protein [Populus trichocarpa]
1341	17638	gi|224146696	79.4	14	4	1	Carbamidomethylation	27517	hypothetical protein POPTR_0019s14190g [Populus trichocarpa]
1351	1323	gi|526117539	79.27	10	2	1	Carbamidomethylation	34687	class I extracellular chitinase precursor [Vitis vinifera]
1553	17838	gi|224072208	79.26	11	3	2	Carbamidomethylation	38044	UDP-glucose 4-epimerase family protein [Populus trichocarpa]

1606	1512	gi|225454123	79.24	14	2	1		22997	PREDICTED: pre-mRNA cleavage factor Im 25 kDa subunit 2 [Vitis vinifera]
675	1508	gi|359476741	79.05	36	2	2	Carbamidomethylation	8039	PREDICTED: 60S ribosomal protein L38 [Vitis vinifera]
1292	18022	gi|224112819	78.92	21	3	1	Carbamidomethylation	16047	actin-depolymerizing factor family protein [Populus trichocarpa]
1297	17680	gi|566162135	78.81	15	4	1	Oxidation (M)	27086	hypothetical protein POPTR_0003s11350g [Populus trichocarpa]
2080	1365	gi|731401209	78.8	3	3	2		93515	PREDICTED: acetyl-coenzyme A carboxylase carboxyl transferase subunit alpha chloroplastic [Vitis vinifera]
1380	17616	gi|566182000	78.77	7	4	1	Carbamidomethylation	57819	hypothetical protein POPTR_0008s02740g [Populus trichocarpa]
1714	17835	gi|566173634	78.69	3	3	3		121829	hypothetical protein POPTR_0005s27880g [Populus trichocarpa]
1406	17847	gi|566193929	78.68	10	4	1		37642	hypothetical protein POPTR_0011s05650g [Populus trichocarpa]
1177	18144	gi|224073967	78.61	12	2	2		15188	photosystem I 11K family protein [Populus trichocarpa]
1343	1325	gi|731411781	78.59	10	2	2		25284	PREDICTED: manganese superoxide dismutase isoform X1 [Vitis vinifera]
1003	722	gi|225430200	78.51	10	4	1		40334	PREDICTED: uncharacterized protein LOC100252479 [Vitis vinifera]
1327	1108	gi|731389140	78.46	7	3	3	Carbamidomethylation	58551	PREDICTED: RAN GTPase-activating protein 2 [Vitis vinifera]
1168	1064	gi|225462285	78.41	13	7	3		52600	PREDICTED: inosine-5'-monophosphate dehydrogenase [Vitis vinifera]
858	992	gi|225441912	78.28	12	4	2	Oxidation (M)	30951	PREDICTED: S-formylglutathione hydrolase isoform X4 [Vitis vinifera]

1372	1027	gi|359482368	78.27	2	3	3		274480	PREDICTED: pre-mRNA-processing-splicing factor 8 [Vitis vinifera]
1042	938	gi|225450669	78.26	5	5	2		113734	PREDICTED: eukaryotic translation initiation factor 3 subunit A [Vitis vinifera]
530	1381	gi|526117942	78.14	6	2	1		37456	beta 1-3 glucanase precursor [Vitis vinifera]
1173	17721	gi|566171771	77.91	9	4	2		53299	hypothetical protein POPTR_0005s16590g partial [Populus trichocarpa]
1275	1348	gi|225462545	77.87	18	3	3	Carbamidomethylation	27262	PREDICTED: proteasome subunit alpha type-4 [Vitis vinifera]
1258	18265	gi|566189013	77.64	10	2	1	Oxidation (M)	30317	glycine-rich family protein [Populus trichocarpa]
1390	18276	gi|566208308	77.57	6	2	2	Carbamidomethylation	39253	hypothetical protein POPTR_0016s02570g [Populus trichocarpa]
1836	881	gi|225433707	77.53	4	3	2		118913	PREDICTED: probable importin-7 homolog [Vitis vinifera]
1288	3000	gi|225453620	77.5	14	4	1	Carbamidomethylation; Oxidation (M)	47761	PREDICTED: pyruvate dehydrogenase E1 component subunit alpha-3 chloroplastic [Vitis vinifera]
1227	1526	gi|225440674	77.49	9	2	1	Carbamidomethylation	31431	PREDICTED: chlorophyll a-b binding protein CP29.1 chloroplastic [Vitis vinifera]
588	783	gi|225452120	77.49	18	4	4		17069	PREDICTED: 60S ribosomal protein L26-1 [Vitis vinifera]
1417	910	gi|225449380	77.44	17	5	2	Carbamidomethylation	39535	PREDICTED: mannose-1-phosphate guanylyltransferase 1 [Vitis vinifera]
1464	1769	gi|225461445	77.41	7	4	1		81247	PREDICTED: vesicle-fusing ATPase [Vitis vinifera]
1433	17950	gi|224105287	77.35	13	3	3	Carbamidomethylation	34749	NADP dependent sorbitol 6-phosphate dehydrogenase family protein [Populus trichocarpa]
1224	876	gi|225429341	77.33	8	3	3	Carbamidomethylation	44078	PREDICTED: 3-oxo-Delta(4 5)-steroid 5-beta-reductase-like [Vitis vinifera]

1188	18542	gi|224124892	77.32	13	2	1		22641	rhicadhesin receptor family protein [Populus trichocarpa]
1253	17785	gi|566149444	77.26	8	3	3		54813	aldehyde dehydrogenase family 7 member a1 turgor responsive family protein [Populus trichocarpa]
1570	17769	gi|566192876	77.21	10	3	2		47035	hypothetical protein POPTR_0010s26170g [Populus trichocarpa]
1554	17860	gi|224083163	77.21	4	3	1	Carbamidomethylation	101703	putative translation initiation family protein [Populus trichocarpa]
1645	18242	gi|566214941	77.18	2	2	1		107829	elongation factor Ts family protein [Populus trichocarpa]
1926	1145	gi|359475871	77.17	12	2	2	Carbamidomethylation	32719	PREDICTED: protein MEMO1 [Vitis vinifera]
1150	18051	gi|566186535	77.17	4	4	2	Carbamidomethylation	122558	transducin family protein [Populus trichocarpa]
980	17648	gi|566184227	77.11	8	4	3		64966	pyruvate phosphotransferase family protein [Populus trichocarpa]
1897	793	gi|225428898	77.05	6	3	1		62556	PREDICTED: D-3-phosphoglycerate dehydrogenase 1 chloroplastic [Vitis vinifera]
1205	18018	gi|224083504	76.99	3	3	2		105368	hypothetical protein POPTR_0005s07000g [Populus trichocarpa]
1286	1338	gi|225441385	76.97	11	4	4		25906	PREDICTED: 60S ribosomal protein L6-1 [Vitis vinifera]
992	1515	gi|225439868	76.81	8	2	1	Carbamidomethylation	39872	PREDICTED: diaminopimelate epimerase chloroplastic [Vitis vinifera]
2100	17988	gi|566191104	76.77	4	3	2		81785	hypothetical protein POPTR_0010s16210g [Populus trichocarpa]
1101	18112	gi|224065405	76.75	28	2	2		8817	wound-responsive family protein [Populus trichocarpa]
1082	973	gi|225450215	76.67	12	3	2		26049	PREDICTED: 60S ribosomal protein L6-3 [Vitis vinifera]

1505	1879	gi|359492060	76.44	7	3	3	Carbamidomethylation	46276	PREDICTED: polyadenylate-binding protein RBP47-like [Vitis vinifera]
1794	890	gi|225442223	76.39	7	3	3		49618	PREDICTED: glycylpeptide N-tetradecanoyltransferase 1 [Vitis vinifera]
1679	1643	gi|225452186	76.28	13	4	4		30725	PREDICTED: prohibitin-3 mitochondrial [Vitis vinifera]
1760	18326	gi|224109088	76.28	4	2	1	Carbamidomethylation	57503	SUCCINIC SEMIALDEHYDE DEHYDROGENASE family protein [Populus trichocarpa]
1270	1838	gi|225440564	76.23	6	2	2		44667	PREDICTED: fructose-1 6-bisphosphatase chloroplastic-like [Vitis vinifera]
825	2509	gi|731394072	76.07	8	2	2		14980	PREDICTED: 60S acidic ribosomal protein P2 isoform X1 [Vitis vinifera]
1254	1237	gi|225446509	76.04	3	2	2		77096	PREDICTED: acyl-coenzyme A oxidase 3 peroxisomal [Vitis vinifera]
1555	18117	gi|566167194	76.02	7	2	1		41861	glycerate dehydrogenase family protein [Populus trichocarpa]
1174	858	gi|359475902	75.97	7	4	3	Carbamidomethylation	61615	PREDICTED: protein TIC 55 chloroplastic [Vitis vinifera]
1478	951	gi|731371455	75.97	8	4	2		58282	PREDICTED: coatomer subunit delta [Vitis vinifera]
1631	1192	gi|731382587	75.96	2	2	1		119503	PREDICTED: epidermal growth factor receptor substrate 15-like 1 [Vitis vinifera]
2033	17767	gi|566157737	75.95	4	3	3		79950	hypothetical protein POPTR_0002s13010g [Populus trichocarpa]
1331	17774	gi|224124820	75.82	5	3	1		60626	DEAD box protein P68 [Populus trichocarpa]
587	1077	gi|225448675	75.8	10	2	2		23332	PREDICTED: probable chalcone--flavonone isomerase 3 [Vitis vinifera]
1432	1622	gi|731406178	75.78	5	3	2		62855	PREDICTED: pectinesterase-like [Vitis vinifera]

927	18185	gi|566211620	75.77	7	2	1		29136	hypothetical protein POPTR_0017s05770g [Populus trichocarpa]
1585	18206	gi|566207525	75.71	3	2	2		83959	subtilase family protein [Populus trichocarpa]
1981	17920	gi|224087657	75.65	11	3	3		32555	UNCOUPLING family protein [Populus trichocarpa]
1214	744	gi|359497196	75.61	15	3	2		23133	PREDICTED: ras-related protein RABH1e [Vitis vinifera]
1888	1251	gi|731432083	75.6	3	2	1		115512	PREDICTED: protein transport protein Sec24-like At3g07100 [Vitis vinifera]
1350	18388	gi|224130478	75.59	5	2	2		53414	Tubulin gamma-1 chain family protein [Populus trichocarpa]
1402	1362	gi|225440230	75.54	10	2	2	Carbamidomethylation	29238	PREDICTED: dihydrofolate reductase [Vitis vinifera]
1276	3842	gi|225433424	75.48	6	2	1	Carbamidomethylation	48624	PREDICTED: 3-ketoacyl-CoA thiolase 2 peroxisomal [Vitis vinifera]
622	850	gi|225450384	75.45	4	3	2	Carbamidomethylation	57022	PREDICTED: beta-glucosidase 12-like [Vitis vinifera]
1781	18050	gi|566187644	75.4	3	2	1	Carbamidomethylation	91239	hypothetical protein POPTR_0009s12440g [Populus trichocarpa]
1523	17962	gi|359476900	75.36	11	3	3		40711	PREDICTED: isocitrate dehydrogenase [NAD] regulatory subunit 1 mitochondrial-like [Vitis vinifera]
1222	18534	gi|224063774	75.35	4	3	3		55604	3-phosphoshikimate 1-carboxyvinyltransferase family protein [Populus trichocarpa]
1249	1287	gi|225460991	75.33	4	2	1	Carbamidomethylation	86153	PREDICTED: heat shock 70 kDa protein 16 [Vitis vinifera]
1800	18096	gi|566198674	75.01	2	2	1		120951	hypothetical protein POPTR_0013s00590g [Populus trichocarpa]
1239	18329	gi|224120174	74.9	2	2	1		68576	Phosphoglucomutase family protein [Populus trichocarpa]

1135	931	gi|225434323	74.84	14	4	1	Carbamidomethylation	30353	PREDICTED: eukaryotic translation initiation factor 2 subunit beta [Vitis vinifera]
1142	1384	gi|225437898	74.71	15	3	2		15168	PREDICTED: photosystem I reaction center subunit IV B chloroplastic-like [Vitis vinifera]
1151	837	gi|731425891	74.65	12	3	2		31554	PREDICTED: hypersensitive-induced response protein 1 isoform X2 [Vitis vinifera]
1747	18040	gi|566174827	74.57	4	3	1	Carbamidomethylation	86014	hypothetical protein POPTR_0006s06460g [Populus trichocarpa]
1234	17900	gi|224104697	74.44	16	3	1		20848	lactoylglutathione lyase family protein [Populus trichocarpa]
1400	1201	gi|225454278	74.4	6	2	2		40499	PREDICTED: bifunctional L-3-cyanoalanine synthase/cysteine synthase 1 mitochondrial [Vitis vinifera]
1409	976	gi|731405314	74.36	4	3	2		97279	PREDICTED: serine/threonine-protein phosphatase 6 regulatory subunit 2 [Vitis vinifera]
907	18175	gi|566202597	74.32	17	2	1		16336	hypothetical protein POPTR_0014s04880g [Populus trichocarpa]
1119	1386	gi|225434203	74.3	8	3	1	Carbamidomethylation	38265	PREDICTED: 2-alkenal reductase (NADP(+)-dependent) [Vitis vinifera]
876	920	gi|225436438	74.25	34	3	3		10607	PREDICTED: cyclin-dependent kinases regulatory subunit 1 [Vitis vinifera]
1780	1344	gi|225454138	74.23	7	3	3	Carbamidomethylation	55947	PREDICTED: DEAD-box ATP-dependent RNA helicase 8 [Vitis vinifera]
1237	17901	gi|566170058	74.15	7	3	3	Carbamidomethylation	65727	hypothetical protein POPTR_0005s07720g [Populus trichocarpa]
1221	1211	gi|731391365	74.01	6	3	1	Carbamidomethylation	52738	PREDICTED: acetolactate synthase small subunit 2 chloroplastic isoform X2 [Vitis vinifera]

1080	17581	gi|566187534	73.87	7	4	4		50027	cyclase associated protein 1 [Populus trichocarpa]
1458	17863	gi|566211408	73.8	9	4	1		37992	hypothetical protein POPTR_0017s04750g [Populus trichocarpa]
1359	3226	gi|526118000	73.79	13	2	1		22732	germin-like protein 2 precursor [Vitis vinifera]
1498	2197	gi|526118093	73.68	13	2	2	Carbamidomethylation	22511	type II peroxiredoxin E [Vitis vinifera]
1948	1628	gi|359482834	73.65	8	3	1		49834	PREDICTED: WD-40 repeat-containing protein MSI4 [Vitis vinifera]
1180	1542	gi|225458237	73.61	9	2	1	Carbamidomethylation	29571	PREDICTED: gamma carbonic anhydrase 1 mitochondrial [Vitis vinifera]
1578	1304	gi|359475330	73.59	14	2	2		16329	PREDICTED: glycine-rich RNA-binding protein GRP2A [Vitis vinifera]
1357	18063	gi|566168268	73.46	16	3	1	Carbamidomethylation	22487	GTP-binding family protein [Populus trichocarpa]
1864	18286	gi|566188208	73.35	2	2	2		139500	TITAN7 family protein [Populus trichocarpa]
1445	18061	gi|566211774	73.23	9	3	2		40195	hypothetical protein POPTR_0017s06410g [Populus trichocarpa]
1556	18123	gi|566212877	73.22	4	2	1	Carbamidomethylation	57107	hypothetical protein POPTR_0017s12170g [Populus trichocarpa]
503	914	gi|225440109	73.21	25	4	2		10658	PREDICTED: 10 kDa chaperonin [Vitis vinifera]
1767	1372	gi|225462440	73.07	2	2	2		107317	PREDICTED: serine/threonine-protein phosphatase BSL3 [Vitis vinifera]
1736	1297	gi|359489218	72.91	6	3	3	Carbamidomethylation	90315	PREDICTED: nucleolin [Vitis vinifera]
1423	970	gi|731413121	72.86	18	3	1		15960	PREDICTED: actin-depolymerizing factor 2-like isoform X2 [Vitis vinifera]

1739	17713	gi|224100535	72.82	5	3	1		67584	hypothetical protein POPTR_0008s01000g [Populus trichocarpa]
2151	17930	gi|566208589	72.81	6	3	1		56137	dyskerin family protein [Populus trichocarpa]
1128	17984	gi|224078816	72.77	9	3	1		36460	Leucoanthocyanidin reductase family protein [Populus trichocarpa]
1312	17866	gi|566215051	72.72	4	3	2		106250	hypothetical protein POPTR_0018s09690g [Populus trichocarpa]
1544	18331	gi|566174691	72.67	5	2	2		63982	tubulin-specific chaperone C-related family protein [Populus trichocarpa]
1719	18675	gi|566175530	72.66	8	2	1	Carbamidomethylation	39660	hypothetical protein POPTR_0006s10360g [Populus trichocarpa]
1605	18439	gi|224081475	72.63	5	2	2		58488	hypothetical protein POPTR_0005s10320g [Populus trichocarpa]
1439	1602	gi|225449234	72.54	22	2	2		12856	PREDICTED: 60S ribosomal protein L35a-1 [Vitis vinifera]
1925	18205	gi|224054120	72.35	4	2	2		73852	lecithin:cholesterol acyltransferase family protein [Populus trichocarpa]
1673	18188	gi|566147227	72.17	3	2	2		110034	hypothetical protein POPTR_0001s04700g [Populus trichocarpa]
1277	18416	gi|224071567	72.16	4	2	1		65821	monocopper oxidase precursor family protein [Populus trichocarpa]
645	719	gi|225424242	72.15	40	4	1		10611	PREDICTED: 10 kDa chaperonin [Vitis vinifera]
1575	18273	gi|224106686	72.06	10	2	1		24434	elongation factor 1B alpha-subunit 2 family protein [Populus trichocarpa]
1348	17644	gi|566245781	71.92	13	4	1		30429	Eukaryotic translation initiation factor 2 beta subunit family protein [Populus trichocarpa]

2086	18372	gi|566167797	71.92	12	2	2		29714	hypothetical protein POPTR_0004s21400g [Populus trichocarpa]
1839	18136	gi|224109362	71.89	10	2	2		31536	Eukaryotic translation initiation factor 3 subunit 5 family protein [Populus trichocarpa]
1597	782	gi|225458719	71.85	6	3	1	Oxidation (M)	63799	PREDICTED: D-3-phosphoglycerate dehydrogenase 3 chloroplastic-like [Vitis vinifera]
1759	18295	gi|224107711	71.82	8	2	2		35785	b-keto acyl reductase family protein [Populus trichocarpa]
401	1367	gi|526118089	71.79	11	3	3		17263	type II peroxiredoxin C [Vitis vinifera]
1984	18662	gi|566201753	71.78	10	2	2	Carbamidomethylation	34979	hypothetical protein POPTR_0014s01090g [Populus trichocarpa]
623	1653	gi|225429975	71.71	6	2	1		39098	PREDICTED: bark storage protein A [Vitis vinifera]
1932	18659	gi|224055093	71.56	13	2	2		22171	hypothetical protein POPTR_0001s26920g [Populus trichocarpa]
1821	18494	gi|224066943	71.53	6	3	3		35968	annexin 1 family protein [Populus trichocarpa]
1435	1626	gi|359482440	71.43	5	3	3	Carbamidomethylation	57853	PREDICTED: alanine aminotransferase 2 [Vitis vinifera]
1805	18395	gi|566182365	71.41	8	2	2		34377	hypothetical protein POPTR_0008s04660g [Populus trichocarpa]
1755	18049	gi|566147740	71.24	11	3	2		36450	hypothetical protein POPTR_0001s07480g [Populus trichocarpa]
1658	18304	gi|566184588	71.05	9	2	1		33826	hypothetical protein POPTR_0008s17880g [Populus trichocarpa]
1425	1946	gi|225440849	71.05	28	2	2		8952	PREDICTED: probable small nuclear ribonucleoprotein G [Vitis vinifera]
1473	1181	gi|731387206	70.99	5	2	2		62778	PREDICTED: splicing factor U2af large subunit B isoform X2 [Vitis vinifera]

1228	1439	gi|731428465	70.85	6	3	3		37487	PREDICTED: thylakoid lumenal 29 kDa protein chloroplastic [Vitis vinifera]
998	17994	gi|566158382	70.81	4	3	3		77765	hypothetical protein POPTR_0002s16570g [Populus trichocarpa]
1933	18666	gi|566197993	70.79	8	2	1		29095	hypothetical protein POPTR_0012s11630g [Populus trichocarpa]
1185	1405	gi|225433434	70.76	6	3	3		65411	PREDICTED: dihydroxy-acid dehydratase chloroplastic [Vitis vinifera]
1451	18861	gi|566159684	70.74	4	2	1	Oxidation (M)	72140	hypothetical protein POPTR_0002s23950g [Populus trichocarpa]
1320	1842	gi|225447176	70.63	4	2	1		46608	PREDICTED: protein disulfide isomerase-like 2-3 [Vitis vinifera]
1263	18113	gi|224140305	70.57	7	2	1		34792	hypothetical protein POPTR_0016s10400g [Populus trichocarpa]
1531	2325	gi|225443274	70.52	8	2	2		35506	PREDICTED: cell wall integrity protein scw1 [Vitis vinifera]
1124	17675	gi|566198526	70.49	5	4	2		67271	hypothetical protein POPTR_0012s14780g [Populus trichocarpa]
2154	2552	gi|731393274	70.39	3	2	1		76795	PREDICTED: uncharacterized protein LOC100253534 isoform X2 [Vitis vinifera]
1766	17966	gi|566191728	70.39	6	3	1	Carbamidomethylation	64682	hypothetical protein POPTR_0010s19790g [Populus trichocarpa]
1909	1844	gi|731439310	70.32	2	1	1		71338	PREDICTED: fumarylacetoacetase isoform X2 [Vitis vinifera]
1959	2587	gi|359480790	70.29	4	2	1		64379	PREDICTED: patellin-3-like [Vitis vinifera]
485	18717	gi|224059156	70.18	18	2	1		14119	Profilin 3 family protein [Populus trichocarpa]

1671	1478	gi|225460855	70.05	5	3	2	Carbamidomethylation	61200	PREDICTED: trigger factor-like protein TIG Chloroplastic [Vitis vinifera]
1399	18130	gi|566160621	69.95	5	3	1		55240	DEAD box RNA helicase family protein [Populus trichocarpa]
1709	19451	gi|566160279	69.81	5	1	1		36389	hypothetical protein POPTR_0003s01450g [Populus trichocarpa]
1529	1623	gi|225437378	69.77	17	2	2	Carbamidomethylation	11879	PREDICTED: small nuclear ribonucleoprotein Sm D2-like [Vitis vinifera]
1901	18333	gi|224111390	69.6	7	2	1	Carbamidomethylation	32619	transport protein SEC13 [Populus trichocarpa]
1717	18181	gi|566177775	69.59	6	2	2		35282	hypothetical protein POPTR_0006s23520g [Populus trichocarpa]
2214	5417	gi|731391337	69.49	7	2	1	Carbamidomethylation	45764	PREDICTED: mitogen-activated protein kinase homolog MMK1 [Vitis vinifera]
1700	18572	gi|566204149	69.47	4	2	1	Carbamidomethylation	82263	hypothetical protein POPTR_0014s13710g [Populus trichocarpa]
1485	2088	gi|731379285	69.43	9	2	2		27000	PREDICTED: probable phospholipid hydroperoxide glutathione peroxidase [Vitis vinifera]
1586	18209	gi|566170408	69.35	2	2	2		106236	alanyl-tRNA synthetase family protein [Populus trichocarpa]
1900	18153	gi|566188415	69.3	4	2	2		64285	hypothetical protein POPTR_0009s17070g [Populus trichocarpa]
1106	18715	gi|224107783	69.27	12	2	1	Carbamidomethylation	16476	hypothetical protein POPTR_0010s05590g [Populus trichocarpa]
1525	2467	gi|225450774	69.27	9	2	2		35393	PREDICTED: ATP-dependent Clp protease proteolytic subunit-related protein 3 chloroplastic [Vitis vinifera]

2153	18189	gi|224085886	69.21	4	2	2	Carbamidomethylation	63489	hypothetical protein POPTR_0005s26090g [Populus trichocarpa]
1695	18292	gi|566158822	69.17	8	2	2		39133	serine/threonine protein kinase [Populus trichocarpa]
774	1101	gi|225434062	69.12	13	3	2	Carbamidomethylation	28430	PREDICTED: chitinase 5-like [Vitis vinifera]
1716	1094	gi|225461050	68.99	6	2	2		49698	PREDICTED: tryptophan synthase beta chain 1 [Vitis vinifera]
994	17669	gi|566185352	68.94	5	4	1		79054	hypothetical protein POPTR_0008s22410g [Populus trichocarpa]
1674	18207	gi|224077052	68.91	2	2	2		121102	guanylate-binding family protein [Populus trichocarpa]
1803	3543	gi|225430488	68.91	3	2	1		73594	PREDICTED: transmembrane 9 superfamily member 9 [Vitis vinifera]
1652	2038	gi|526117828	68.88	12	2	1		16599	serine/threonine kinase-like [Vitis vinifera]
1569	17693	gi|224091819	68.87	3	3	1		88349	MIF4G domain-containing family protein [Populus trichocarpa]
1091	18417	gi|566195824	68.84	3	3	1		60887	hypothetical protein POPTR_0011s16410g [Populus trichocarpa]
1669	2043	gi|225428141	68.77	7	3	3		68945	PREDICTED: SEC1 family transport protein SLY1 [Vitis vinifera]
1105	18711	gi|224128708	68.72	7	2	1		33828	xyloglucan endo-1 family protein [Populus trichocarpa]
1757	18204	gi|566175538	68.69	2	1	1		96436	hypothetical protein POPTR_0006s10420g [Populus trichocarpa]
1512	1309	gi|731398937	68.68	15	2	1	Carbamidomethylation	21183	PREDICTED: actin-depolymerizing factor 1-like [Vitis vinifera]
1592	1863	gi|359487567	68.64	4	2	2	Carbamidomethylation	41694	PREDICTED: GDSL esterase/lipase At1g09390 [Vitis vinifera]
1804	18228	gi|224065629	68.59	15	2	2	Carbamidomethylation	16418	CP12 domain-containing family protein [Populus trichocarpa]

1772	1218	gi|731413519	68.52	5	2	2		48243	PREDICTED: vacuolar protein sorting-associated protein 4 [Vitis vinifera]
1588	1567	gi|225433215	68.5	9	2	2	Carbamidomethylation	26277	PREDICTED: DAG protein chloroplastic [Vitis vinifera]
1816	1116	gi|225463618	68.41	6	3	1	Carbamidomethylation	80442	PREDICTED: pyrophosphate-energized vacuolar membrane proton pump [Vitis vinifera]
533	1060	gi|225432662	68.34	14	3	3		17620	PREDICTED: 40S ribosomal protein S18 [Vitis vinifera]
1799	2232	gi|225459360	68.18	6	3	1		56704	PREDICTED: probable glucan 1 3-beta-glucosidase A [Vitis vinifera]
1226	18015	gi|224116326	67.83	9	3	1		36463	Leucoanthocyanidin reductase family protein [Populus trichocarpa]
1509	18384	gi|224146080	67.76	14	2	2		17003	hypothetical protein POPTR_0019s05840g [Populus trichocarpa]
1431	17864	gi|566153954	67.75	10	3	1		31003	phosphatase 2C family protein [Populus trichocarpa]
1743	18308	gi|731406703	67.66	4	2	2		66565	PREDICTED: arginine--tRNA ligase cytoplasmic-like isoform X2 [Vitis vinifera]
1835	17772	gi|566168111	67.65	10	3	1		43492	hypothetical protein POPTR_0004s22790g [Populus trichocarpa]
1490	2011	gi|731437310	67.64	7	3	1	Carbamidomethylation	52411	PREDICTED: acetolactate synthase small subunit 2 chloroplastic [Vitis vinifera]
1904	1412	gi|225427234	67.6	14	2	2		17554	PREDICTED: glycine cleavage system H protein mitochondrial [Vitis vinifera]
1360	1588	gi|225429995	67.53	13	2	1	Carbamidomethylation	17724	PREDICTED: protein PEROXIN-4 [Vitis vinifera]
1680	17711	gi|224122948	67.51	4	3	1		79226	inorganic pyrophosphatase family protein [Populus trichocarpa]
1587	18220	gi|566152765	67.48	5	2	2		49287	hypothetical protein POPTR_0001s34620g [Populus trichocarpa]

1511	18493	gi|566164895	67.48	10	2	1		24783	hypothetical protein POPTR_0004s044401g partial [Populus trichocarpa]
2393	2099	gi|225426826	67.32	7	2	2		36763	PREDICTED: replication factor C subunit 2 [Vitis vinifera]
568	19286	gi|566208913	67.2	3	2	1		37900	hypothetical protein POPTR_0016s05800g [Populus trichocarpa]
1347	17633	gi|224109084	67.14	9	5	1		53455	inosine monophosphate dehydrogenase family protein [Populus trichocarpa]
2087	18379	gi|566182095	67.13	6	2	1		48288	hypothetical protein POPTR_0008s03160g [Populus trichocarpa]
1568	1358	gi|731409226	67.11	6	2	1		43860	PREDICTED: 3-hydroxyisobutyryl-CoA hydrolase-like protein 5 [Vitis vinifera]
1005	18393	gi|224100515	67.1	9	2	2		45833	hypothetical protein POPTR_0008s00700g [Populus trichocarpa]
1647	18387	gi|566160574	67.03	5	2	1		57226	serine/threonine protein phosphatase 2A 55 kDa regulatory subunit B [Populus trichocarpa]
1985	1545	gi|225451299	67.01	10	2	2		30245	PREDICTED: probable ribose-5-phosphate isomerase 3 chloroplastic [Vitis vinifera]
1557	1481	gi|731415988	66.99	2	2	1		119890	PREDICTED: FACT complex subunit SPT16-like [Vitis vinifera]
2210	1979	gi|731436817	66.83	5	2	2		49774	PREDICTED: cysteine desulfurase 1 mitochondrial-like [Vitis vinifera]
1641	842	gi|731424103	66.8	3	3	1	Carbamidomethylation	151229	PREDICTED: tripeptidyl-peptidase 2 isoform X1 [Vitis vinifera]
1686	6864	gi|359475352	66.79	3	1	1		49145	PREDICTED: uncharacterized protein At4g06744 [Vitis vinifera]
1796	17856	gi|731401507	66.68	3	3	3		138211	PREDICTED: 5-oxoprolinase [Vitis vinifera]
792	1269	gi|225459906	66.67	5	2	1		34942	PREDICTED: probable fructokinase-5 [Vitis vinifera]

1178	18154	gi|224130362	66.64	6	2	1		42752	MAP kinase family protein [Populus trichocarpa]
1784	1831	gi|225426811	66.57	6	2	1		39029	PREDICTED: bifunctional UDP-glucose 4-epimerase and UDP-xylose 4-epimerase 1 [Vitis vinifera]
1164	18545	gi|566170295	66.51	3	2	1	Oxidation (M)	119716	Valyl-tRNA synthetase family protein [Populus trichocarpa]
2058	18336	gi|566207035	66.48	2	1	1		62799	hypothetical protein POPTR_0015s11280g [Populus trichocarpa]
1691	921	gi|731382079	66.47	3	3	3		110376	PREDICTED: neutral alpha-glucosidase C-like isoform X2 [Vitis vinifera]
1660	18355	gi|224084344	66.46	7	2	2		40622	RNA recognition motif-containing family protein [Populus trichocarpa]
770	18599	gi|224135091	66.46	6	2	2		24193	hypothetical protein POPTR_0015s01130g [Populus trichocarpa]
1299	1671	gi|731424196	66.41	13	2	1		18730	PREDICTED: pre-mRNA cleavage factor Im 25 kDa subunit 2 isoform X2 [Vitis vinifera]
805	2307	gi|225441664	66.37	5	1	1		36307	PREDICTED: uncharacterized protein LOC100255512 [Vitis vinifera]
1437	1933	gi|225455762	66.23	3	2	2		67078	PREDICTED: probable phenylalanine--tRNA ligase beta subunit [Vitis vinifera]
1872	3224	gi|225435122	66.22	3	3	1	Carbamidomethylation	116639	PREDICTED: calcium-transporting ATPase 4 endoplasmic reticulum-type-like [Vitis vinifera]
1966	18830	gi|566214901	66.21	5	1	1		35399	hypothetical protein POPTR_0018s08850g [Populus trichocarpa]
1893	17849	gi|566211354	66.12	11	3	1		34355	phosphatase 2C family protein [Populus trichocarpa]

1353	1611	gi|731417625	66.07	12	2	2		19282	PREDICTED: uncharacterized protein LOC100241624 [Vitis vinifera]
1107	1812	gi|225430510	66.03	6	3	3		27121	PREDICTED: phospholipid hydroperoxide glutathione peroxidase 1 chloroplastic [Vitis vinifera]
1930	18447	gi|359474555	65.98	6	2	2		43514	PREDICTED: probable ADP-ribosylation factor GTPase-activating protein AGD8 [Vitis vinifera]
1725	2319	gi|225452270	65.98	6	2	1		34598	PREDICTED: 28 kDa ribonucleoprotein chloroplastic-like [Vitis vinifera]
592	1307	gi|225463506	65.97	7	2	2		38294	PREDICTED: fructose-bisphosphate aldolase cytoplasmic isozyme 1 [Vitis vinifera]
1818	3118	gi|359484223	65.78	3	3	1	Carbamidomethylation	85770	PREDICTED: probable galactinol--sucrose galactosyltransferase 6 [Vitis vinifera]
1619	18678	gi|566210985	65.66	6	2	1	Carbamidomethylation	51941	glycosyl hydrolase family 17 family protein [Populus trichocarpa]
1620	1371	gi|225469850	65.63	6	2	2		39160	PREDICTED: uncharacterized protein DDB_G0288133 [Vitis vinifera]
1889	1982	gi|731402641	65.58	5	1	1		35414	PREDICTED: cytochrome c1-2 heme protein mitochondrial-like isoform X3 [Vitis vinifera]
1025	1485	gi|731439529	65.57	2	1	1		66300	PREDICTED: splicing factor 3B subunit 2 [Vitis vinifera]
1232	1619	gi|225461983	65.5	7	3	1		36179	PREDICTED: heterogeneous nuclear ribonucleoprotein 1 [Vitis vinifera]
2158	2125	gi|731415654	65.48	3	2	2		73684	PREDICTED: acyl-CoA-binding domain-containing protein 4-like isoform X2 [Vitis vinifera]
1962	18679	gi|225443109	65.39	10	2	2	Carbamidomethylation	38454	PREDICTED: protein RAE1 [Vitis vinifera]

1398	1079	gi|225445949	65.34	5	3	2	Carbamidomethylation	49372	PREDICTED: KH domain-containing protein At4g18375 [Vitis vinifera]
1508	18213	gi|224118508	65.28	7	2	2		39734	Chain A family protein [Populus trichocarpa]
1496	1995	gi|731412542	65.27	3	2	2		58149	PREDICTED: uncharacterized protein LOC100244432 isoform X4 [Vitis vinifera]
1819	18252	gi|224081300	65.24	5	2	1		50528	26S proteasome regulatory subunit family protein [Populus trichocarpa]
1722	1667	gi|225432306	65.22	22	2	2	Carbamidomethylation	9210	PREDICTED: 40S ribosomal protein S21 [Vitis vinifera]
1318	18109	gi|566168240	65.19	3	2	1	Carbamidomethylation	55680	hypothetical protein POPTR_0004s23390g [Populus trichocarpa]
1484	18065	gi|224100909	65.19	2	3	1		134447	splicing factor family protein [Populus trichocarpa]
1058	1665	gi|225459083	65.18	6	2	2	Carbamidomethylation; Oxidation (M)	58401	PREDICTED: argininosuccinate lyase chloroplastic [Vitis vinifera]
1411	18221	gi|566206893	65.18	4	2	2		52011	hypothetical protein POPTR_0015s10280g [Populus trichocarpa]
2349	18762	gi|566160631	65.11	2	1	1		73609	endomembrane protein 70 [Populus trichocarpa]
1720	18739	gi|566186085	65.09	16	2	1		14506	hypothetical protein POPTR_0009s03370g partial [Populus trichocarpa]
1442	19692	gi|568244583	65.02	7	2	2		21881	clp protease proteolytic subunit (plastid) [Camellia oleifera]
2123	18303	gi|566160558	65	5	2	2	Carbamidomethylation	54211	UBIQUITIN-SPECIFIC PROTEASE 6 family protein [Populus trichocarpa]
1259	1593	gi|359497369	64.9	4	2	1	Carbamidomethylation	64520	PREDICTED: eukaryotic translation initiation factor 3 subunit D-like [Vitis vinifera]
2025	18244	gi|566198992	64.85	4	2	2	Carbamidomethylation	78312	hypothetical protein POPTR_0013s02400g [Populus trichocarpa]

1954	18467	gi|566253285	64.8	9	2	1		36419	hypothetical protein POPTR_1567s00200g partial [Populus trichocarpa]
1608	998	gi|225435024	64.79	9	2	1		25842	PREDICTED: THO complex subunit 4A [Vitis vinifera]
1471	17798	gi|224146178	64.64	5	3	1	Carbamidomethylation	49470	hypothetical protein POPTR_0019s08290g [Populus trichocarpa]
1982	17970	gi|552539635	64.56	29	3	2		14625	ATP synthase CF1 epsilon subunit (chloroplast) [Camellia cuspidata]
1048	1090	gi|225465997	64.43	6	3	2		48382	PREDICTED: WD-40 repeat-containing protein MSI1 [Vitis vinifera]
1447	18691	gi|671743260	64.42	10	2	2		24165	cytochrome b6 (chloroplast) [Camellia petelotii]
1029	1128	gi|731418015	64.38	7	3	2		53972	PREDICTED: tryptophan synthase beta chain 1 [Vitis vinifera]
1656	18081	gi|566159456	64.36	7	3	1		50614	hypothetical protein POPTR_0002s22580g [Populus trichocarpa]
1560	18330	gi|566234718	64.16	2	2	1		124541	exportin1 family protein [Populus trichocarpa]
1650	1474	gi|225453042	64.15	2	2	2	Carbamidomethylation	113229	PREDICTED: cell division cycle 5-like protein [Vitis vinifera]
1715	17999	gi|566176289	64.11	15	3	3	Carbamidomethylation	23433	peroxiredoxin Q family protein [Populus trichocarpa]
1441	2329	gi|225435002	64.06	6	1	1	Carbamidomethylation	27396	PREDICTED: osmotin-like protein [Vitis vinifera]
2478	1547	gi|225454994	63.89	9	2	1		28211	PREDICTED: peptide methionine sulfoxide reductase A1-like [Vitis vinifera]
1801	2035	gi|731402548	63.86	2	2	2		91875	PREDICTED: N-alpha-acetyltransferase 15 NatA auxiliary subunit isoform X3 [Vitis vinifera]
1558	18246	gi|566215059	63.82	6	2	1		67827	hypothetical protein POPTR_0018s09760g [Populus trichocarpa]

1958	2389	gi|731369373	63.75	5	2	1	Carbamidomethylation	51603	PREDICTED: glucan endo-1 3-beta-glucosidase 3 isoform X2 [Vitis vinifera]
1721	1195	gi|225444525	63.73	7	2	1	Carbamidomethylation	41018	PREDICTED: ATP synthase gamma chain chloroplastic [Vitis vinifera]
1986	18690	gi|566167629	63.72	8	2	2	Carbamidomethylation	40491	homoserine dehydrogenase family protein [Populus trichocarpa]
1136	1292	gi|731378427	63.71	5	2	2		51975	PREDICTED: uncharacterized protein LOC100263310 [Vitis vinifera]
1454	19656	gi|566169467	63.69	9	1	1		13987	hypothetical protein POPTR_0005s04590g [Populus trichocarpa]
1696	18529	gi|566151474	63.66	20	2	2	Carbamidomethylation	12414	hypothetical protein POPTR_0001s28430g [Populus trichocarpa]
1038	2304	gi|225435608	63.46	8	1	1	Carbamidomethylation	31609	PREDICTED: zinc finger CCCH domain-containing protein 14-like [Vitis vinifera]
1179	1373	gi|225465210	63.27	17	2	2		11974	PREDICTED: cytochrome c [Vitis vinifera]
2213	18710	gi|224065729	63.23	15	2	2		24619	hypothetical protein POPTR_0002s01660g [Populus trichocarpa]
1450	18856	gi|224099491	63.15	24	2	1		10451	hypothetical protein POPTR_0008s12950g [Populus trichocarpa]
2368	1736	gi|731410731	63.14	2	2	1		178423	PREDICTED: UDP-glucose:glycoprotein glucosyltransferase isoform X2 [Vitis vinifera]
892	18516	gi|224104953	63.09	4	2	1		68350	beta-D-glucan exohydrolase family protein [Populus trichocarpa]
1607	1262	gi|731436828	62.96	6	2	1	Carbamidomethylation	57638	PREDICTED: ATP-dependent 6-phosphofructokinase 5 chloroplastic-like [Vitis vinifera]

1530	2484	gi|225465947	62.7	7	2	2		52209	PREDICTED: uridine 5'-monophosphate synthase [Vitis vinifera]
1413	18306	gi|566152880	62.61	4	2	1		86547	hypothetical protein POPTR_0001s35150g [Populus trichocarpa]
598	18297	gi|566192144	62.59	6	2	2		36328	hypothetical protein POPTR_0010s22170g [Populus trichocarpa]
1927	1415	gi|225437227	62.56	4	2	2		60053	PREDICTED: glutathione reductase chloroplastic [Vitis vinifera]
1771	18320	gi|566166505	62.45	4	2	1		65003	hypothetical protein POPTR_0004s14530g [Populus trichocarpa]
1951	1375	gi|359487733	62.37	6	2	1		46705	PREDICTED: amidase 1 [Vitis vinifera]
1414	18312	gi|224082494	62.23	4	2	2		38884	peroxidase precursor family protein [Populus trichocarpa]
1612	2305	gi|526117321	62.16	8	1	1		23675	dehydroascorbate reductase [Vitis vinifera]
1176	1075	gi|526117894	62.12	7	3	1		36735	anthocyanidin reductase [Vitis vinifera]
1446	1659	gi|731419120	61.79	6	2	2		29507	PREDICTED: beta carbonic anhydrase 1 chloroplastic isoform X2 [Vitis vinifera]
776	2170	gi|225425388	61.73	11	1	1		13594	PREDICTED: nuclear transport factor 2 [Vitis vinifera]
1684	1728	gi|225463807	61.73	5	2	1		45416	PREDICTED: alpha-galactosidase [Vitis vinifera]
1600	18004	gi|224086130	61.6	10	3	1	Carbamidomethylation	32243	U2 snRNP auxiliary factor small subunit family protein [Populus trichocarpa]
1666	19042	gi|566210832	61.57	7	1	1		25245	hypothetical protein POPTR_0017s02160g [Populus trichocarpa]
1820	2451	gi|225439775	61.48	8	2	1		27055	PREDICTED: glutathione S-transferase L3-like [Vitis vinifera]
959	1642	gi|225462170	61.48	12	2	2		15615	PREDICTED: 60S ribosomal protein L27 [Vitis vinifera]

1688	19243	gi|224134737	61.41	7	1	1		23290	cytochrome b5 domain-containing family protein [Populus trichocarpa]
2126	18462	gi|566192083	61.39	3	2	1	Carbamidomethylation	111427	transport protein Sec24 [Populus trichocarpa]
2265	1203	gi|731391355	61.21	4	2	2		85892	PREDICTED: LETM1 and EF-hand domain-containing protein 1 mitochondrial [Vitis vinifera]
1404	1609	gi|225437963	61.19	6	2	2		43931	PREDICTED: NADH dehydrogenase [ubiquinone] 1 alpha subcomplex subunit 9 mitochondrial [Vitis vinifera]
1840	18775	gi|566181200	61.16	4	2	2	Carbamidomethylation	55243	serine carboxypeptidase S28 family protein [Populus trichocarpa]
2161	18550	gi|225456944	61.13	6	2	2		57954	PREDICTED: suppressor of mec-8 and unc-52 protein homolog 1 [Vitis vinifera]
1328	18099	gi|224104247	61.08	4	2	2		44001	aminotransferase 2 family protein [Populus trichocarpa]
2084	18328	gi|224063293	61.04	7	2	2	Carbamidomethylation	31523	FH INTERACTING protein 1 [Populus trichocarpa]
1448	2442	gi|225429295	61	8	2	2		25371	PREDICTED: uncharacterized protein LOC100254416 [Vitis vinifera]
1634	18533	gi|566204671	60.98	2	1	1		65660	hypothetical protein POPTR_0014s16810g [Populus trichocarpa]
2159	3507	gi|731400520	60.72	4	2	2		70745	PREDICTED: pentatricopeptide repeat-containing protein At1g80270 mitochondrial-like [Vitis vinifera]
1923	2081	gi|225448215	60.57	10	3	1		40979	PREDICTED: serine/threonine-protein kinase SRK2A [Vitis vinifera]

1186	1864	gi|731404286	60.54	3	2	2		51076	PREDICTED: dihydrolipoyllysine-residue succinyltransferase component of 2-oxoglutarate dehydrogenase complex 1 mitochondrial [Vitis vinifera]
2043	1161	gi|526117964	60.53	3	2	1		84928	alpha-galactosidase [Vitis vinifera]
1724	18859	gi|224107333	60.52	26	2	2		9716	hypothetical protein POPTR_0010s02580g [Populus trichocarpa]
2124	1990	gi|225441489	60.48	5	2	2		38483	PREDICTED: activator of 90 kDa heat shock protein ATPase homolog [Vitis vinifera]
1924	1026	gi|225443231	60.48	6	3	1		50032	PREDICTED: methanol O-anthraniloyltransferase [Vitis vinifera]
1375	1486	gi|225465204	60.45	2	3	2	Carbamidomethylation	122695	PREDICTED: leucine--tRNA ligase cytoplasmic [Vitis vinifera]
1365	1382	gi|359489656	60.44	6	2	2		52031	PREDICTED: probable polygalacturonase [Vitis vinifera]
1785	2331	gi|731432527	60.22	7	1	1		26117	PREDICTED: DAG protein chloroplastic [Vitis vinifera]
1823	1581	gi|731408703	60.12	6	2	2	Carbamidomethylation	54961	PREDICTED: serine/threonine-protein phosphatase 5 isoform X2 [Vitis vinifera]
1882	1763	gi|731440118	60.05	2	2	2	Carbamidomethylation	144044	PREDICTED: kinesin-like protein KCA2 [Vitis vinifera]
2056	18140	gi|566168611	59.73	2	2	1	Carbamidomethylation	120029	hypothetical protein POPTR_0005s00700g [Populus trichocarpa]
1542	1387	gi|225427288	59.7	4	2	1		68795	PREDICTED: dynamin-related protein 1E [Vitis vinifera]
1952	1340	gi|225442595	59.66	4	2	2		60200	PREDICTED: protein disulfide isomerase-like 1-6 [Vitis vinifera]
1773	18879	gi|566193798	59.59	3	1	1		49306	thioredoxin family protein [Populus trichocarpa]
1243	2306	gi|526117918	59.58	5	1	1		33457	basic endochitinase precursor [Vitis vinifera]

824	1104	gi|731435142	59.36	3	2	1		84036	PREDICTED: protein ROOT HAIR DEFECTIVE 3 homolog 2 isoform X2 [Vitis vinifera]
1770	18310	gi|224143674	59.35	5	2	2		38385	fructose-1 family protein [Populus trichocarpa]
1367	1213	gi|731426682	59.2	3	3	3	Oxidation (M)	143427	PREDICTED: uncharacterized protein LOC100262718 isoform X3 [Vitis vinifera]
1401	18354	gi|566203998	59.14	2	2	1		101817	AAA-type ATPase family protein [Populus trichocarpa]
2071	1738	gi|526117810	59.12	5	1	1		38980	farnesyl diphosphate synthase [Vitis vinifera]
2083	1167	gi|225457034	58.99	4	2	2	Carbamidomethylation	51176	PREDICTED: 1-deoxy-D-xylulose 5-phosphate reductoisomerase chloroplastic [Vitis vinifera]
1837	1625	gi|731441218	58.99	9	3	3		36022	PREDICTED: 1-aminocyclopropane-1-carboxylate oxidase [Vitis vinifera]
1571	1158	gi|225462270	58.91	12	3	3	Carbamidomethylation	31886	PREDICTED: 2-dehydro-3-deoxyphosphooctonate aldolase [Vitis vinifera]
1825	18724	gi|566179116	58.9	9	2	2		29952	integral membrane HRF1 family protein [Populus trichocarpa]
1434	928	gi|225445670	58.84	9	3	1		24997	PREDICTED: proteasome subunit beta type-6 [Vitis vinifera]
1782	2000	gi|731418793	58.73	4	2	2		59155	PREDICTED: glucose-6-phosphate 1-dehydrogenase cytoplasmic isoform [Vitis vinifera]
1903	18400	gi|566150061	58.72	3	2	1		89791	hypothetical protein POPTR_0001s20820g [Populus trichocarpa]
1842	1668	gi|225448536	58.53	7	2	2		38803	PREDICTED: eukaryotic translation initiation factor 2 subunit alpha [Vitis vinifera]
1576	1576	gi|225427798	58.5	4	2	1		62899	PREDICTED: amidophosphoribosyltransferase chloroplastic [Vitis vinifera]
547	1227	gi|225443962	58.36	2	2	2		81691	PREDICTED: CSC1-like protein ERD4 [Vitis vinifera]

2152	1689	gi|359491530	58.36	7	3	3		73129	PREDICTED: probable inactive receptor kinase At1g48480 [Vitis vinifera]
2428	18469	gi|566187699	58.36	6	2	2		36049	ubiquitin fusion degradation UFD1 family protein [Populus trichocarpa]
2068	18268	gi|566195934	58.34	4	2	1		83449	alkaline alpha galactosidase I family protein [Populus trichocarpa]
2215	18735	gi|224062667	58.33	5	2	1		43605	enoyl-CoA hydratase/isomerase family protein [Populus trichocarpa]
2085	18334	gi|566172110	58.24	8	2	2	Carbamidomethylation	39318	casein kinase II alpha chain family protein [Populus trichocarpa]
1577	18391	gi|224066191	58.23	8	2	1	Carbamidomethylation	29403	transcription factor APFI family protein [Populus trichocarpa]
1987	2668	gi|731391510	58.23	11	2	2		25576	PREDICTED: uncharacterized protein LOC104879510 isoform X2 [Vitis vinifera]
1846	19104	gi|224078057	58.13	5	1	1		28150	phosphorylase family protein [Populus trichocarpa]
1623	2312	gi|225457389	58.08	5	1	1		27867	PREDICTED: chlorophyll a-b binding protein 4 chloroplastic [Vitis vinifera]
1241	1562	gi|224365629	58.06	6	2	2		43947	apocytochrome b (mitochondrion) [Vitis vinifera]
1449	18810	gi|566209344	57.82	7	2	1		27361	In2-1 family protein [Populus trichocarpa]
1983	18074	gi|566161702	57.72	8	3	1		30568	Mitochondrial carnitine/acylcarnitine carrier-like family protein [Populus trichocarpa]
414	18536	gi|566165881	57.58	8	2	2		27272	mangrin family protein [Populus trichocarpa]
1603	18271	gi|224105935	57.56	8	2	2	Carbamidomethylation	24127	RAN BINDING protein 1 [Populus trichocarpa]
1659	18348	gi|566173129	57.51	5	2	2		41052	hypothetical protein POPTR_0005s25100g [Populus trichocarpa]

1559	2180	gi|225428049	57.35	4	2	1		42284	PREDICTED: glycerate dehydrogenase HPR peroxisomal [Vitis vinifera]
1758	4071	gi|225445080	57.3	3	1	1		53828	PREDICTED: hexokinase-1-like [Vitis vinifera]
1148	1152	gi|225453336	57.23	2	2	2		90759	PREDICTED: glutamine--tRNA ligase [Vitis vinifera]
1412	18241	gi|566146307	57.18	4	2	1		66102	monocopper oxidase precursor family protein [Populus trichocarpa]
2432	1565	gi|731437645	57.07	8	2	2	Carbamidomethylation	32643	PREDICTED: LOW QUALITY PROTEIN: ruvB-like 2 [Vitis vinifera]
1928	1696	gi|225428556	56.96	4	2	2		48755	PREDICTED: dolichyl-diphosphooligosaccharide--protein glycosyltransferase 48 kDa subunit isoform X2 [Vitis vinifera]
1964	18794	gi|224066629	56.9	16	2	2		16281	thioredoxin family protein [Populus trichocarpa]
1672	18089	gi|566160305	56.89	7	3	3		45443	hypothetical protein POPTR_0003s01630g [Populus trichocarpa]
1992	18833	gi|224145402	56.79	26	2	2		8337	ubiquinol-cytochrome C reductase complex ubiquinone-binding family protein [Populus trichocarpa]
2479	1596	gi|731405420	56.75	4	2	1		60227	PREDICTED: reticuline oxidase-like protein [Vitis vinifera]
1584	1779	gi|225448795	56.71	11	3	3	Carbamidomethylation	19172	PREDICTED: cytochrome c oxidase subunit 6b-1 isoform X4 [Vitis vinifera]
1566	18219	gi|566163043	56.66	3	2	2		67870	glucose-6-phosphate dehydrogenase family protein [Populus trichocarpa]
1936	19160	gi|225435731	56.48	7	1	1		28535	PREDICTED: serine/arginine-rich SC35-like splicing factor SCL30A [Vitis vinifera]
1856	1495	gi|731437632	56.44	6	2	1	Carbamidomethylation	44812	PREDICTED: branched-chain-amino-acid aminotransferase 5 chloroplastic [Vitis vinifera]

2186	19551	gi|224073260	56.4	14	1	1		10707	small nuclear ribonucleoprotein D [Populus trichocarpa]
1334	1908	gi|225424859	56.2	8	2	2		25630	PREDICTED: glutathione S-transferase U17 [Vitis vinifera]
2110	2310	gi|225427364	56.14	3	1	1	Carbamidomethylation	51132	PREDICTED: poly(U)-specific endoribonuclease-B [Vitis vinifera]
1955	18515	gi|566245088	56.01	8	2	1		33717	Xyloglucan endotransglucosylase/hydrolase protein 9 precursor [Populus trichocarpa]
2002	19680	gi|566152777	55.92	15	1	1	Carbamidomethylation	12696	hypothetical protein POPTR_0001s34690g [Populus trichocarpa]
2216	18746	gi|566153195	55.9	8	2	2		42888	cell division family protein [Populus trichocarpa]
1929	18351	gi|566216001	55.88	5	2	2	Carbamidomethylation	74542	hypothetical protein POPTR_0019s00300g [Populus trichocarpa]
574	18787	gi|224146534	55.77	6	2	1	Oxidation (M)	29228	hypothetical protein POPTR_0019s12390g [Populus trichocarpa]
1345	2315	gi|731376404	55.76	7	1	1	Carbamidomethylation	28963	PREDICTED: chlorophyll a-b binding protein chloroplastic [Vitis vinifera]
1798	17963	gi|224125250	55.72	3	3	3		110348	glycoside hydrolase family 2 family protein [Populus trichocarpa]
1487	1096	gi|225445041	55.68	4	2	1		54472	PREDICTED: mitochondrial-processing peptidase subunit alpha [Vitis vinifera]
1657	18299	gi|731436460	55.68	2	3	1	Carbamidomethylation	123310	PREDICTED: leucine--tRNA ligase cytoplasmic-like [Vitis vinifera]
1761	1651	gi|225438962	55.66	5	1	1		35957	PREDICTED: peroxidase 73 [Vitis vinifera]
2088	1901	gi|225430912	55.64	6	1	1		35298	PREDICTED: membrane-associated protein VIPP1 chloroplastic [Vitis vinifera]

2112	2040	gi|731428304	55.54	2	1	1		81014	PREDICTED: apoptotic chromatin condensation inducer in the nucleus-like isoform X2 [Vitis vinifera]
954	19311	gi|566172679	55.53	2	1	1		63027	hypothetical protein POPTR_0005s22250g [Populus trichocarpa]
2274	18911	gi|566184573	55.52	2	1	1		90021	urease family protein [Populus trichocarpa]
1526	1621	gi|225431409	55.49	4	2	1		39072	PREDICTED: probable mannitol dehydrogenase [Vitis vinifera]
1533	2595	gi|359473679	55.48	4	1	1	Carbamidomethylation	35252	PREDICTED: uncharacterized protein LOC100852583 [Vitis vinifera]
1598	1688	gi|359479353	55.37	18	3	1		21048	PREDICTED: lactoylglutathione lyase isoform X2 [Vitis vinifera]
1883	18756	gi|566146785	55.36	3	1	1		43851	UBIQUITIN-SPECIFIC PROTEASE 20 family protein [Populus trichocarpa]
2166	18758	gi|566207939	55.34	6	2	1	Carbamidomethylation	33480	hypothetical protein POPTR_0016s00580g [Populus trichocarpa]
2160	18544	gi|225456299	55.25	9	2	2		20927	PREDICTED: 60S ribosomal protein L17-2-like isoform X2 [Vitis vinifera]
1697	18539	gi|224092348	55.2	17	2	2	Carbamidomethylation	10699	hypothetical protein POPTR_0006s25990g [Populus trichocarpa]
2255	18535	gi|224064627	55.16	4	2	1		82942	pre-mRNA splicing factor ATP-dependent RNA helicase family protein [Populus trichocarpa]
2156	18214	gi|566175269	55.09	4	2	2		42962	hypothetical protein POPTR_0006s08830g [Populus trichocarpa]
1881	3282	gi|359486347	55.06	7	3	3	Carbamidomethylation	43181	PREDICTED: uroporphyrinogen decarboxylase [Vitis vinifera]
1604	18313	gi|224053607	54.96	6	2	1		34050	EXGT1 family protein [Populus trichocarpa]

1990	18806	gi|224116660	54.84	4	2	1	Carbamidomethylation	69893	hypothetical protein POPTR_0011s09560g [Populus trichocarpa]
1663	1666	gi|526117699	54.8	4	2	2		49406	3-dehydroquinate synthase-like [Vitis vinifera]
1510	18430	gi|224064818	54.77	4	2	1		47984	hypothetical protein POPTR_0002s22410g [Populus trichocarpa]
1941	2116	gi|731436778	54.75	8	1	1		21619	PREDICTED: probable NAD(P)H dehydrogenase (quinone) FQR1-like 1 [Vitis vinifera]
1692	3335	gi|731400729	54.69	7	3	1	Carbamidomethylation	40133	PREDICTED: splicing factor U2af small subunit B-like [Vitis vinifera]
1685	18453	gi|224122636	54.66	4	2	2		46434	putative calmodulin-binding family protein [Populus trichocarpa]
1355	1617	gi|225429460	54.64	15	2	2		14040	PREDICTED: uncharacterized protein LOC100267911 [Vitis vinifera]
1931	2586	gi|225431100	54.45	4	2	2		42836	PREDICTED: 26S proteasome non-ATPase regulatory subunit 4 homolog [Vitis vinifera]
2219	2388	gi|731424368	54.3	2	1	1		72886	PREDICTED: pentatricopeptide repeat-containing protein At3g49240 [Vitis vinifera]
2792	19031	gi|224077834	54.21	3	1	1	Carbamidomethylation	56616	phenylalanyl-tRNA synthetase alpha chain family protein [Populus trichocarpa]
1698	1424	gi|225432062	54.2	5	2	2		44703	PREDICTED: 30S ribosomal protein S1 chloroplastic [Vitis vinifera]
2122	18269	gi|566168843	54.18	3	2	2		104889	Lon protease 2 family protein [Populus trichocarpa]
1849	2320	gi|731416920	54.09	15	1	1	Carbamidomethylation	15217	PREDICTED: superoxide dismutase [Cu-Zn] 2-like [Vitis vinifera]
2189	2330	gi|225438277	54.09	3	1	1	Carbamidomethylation	45078	PREDICTED: alcohol dehydrogenase 1 [Vitis vinifera]

2871	19315	gi|224067318	54.07	7	1	1		22986	30S ribosomal protein S9 [Populus trichocarpa]
1699	18555	gi|566167707	54.06	8	2	2		29259	alcohol dehydroge family protein [Populus trichocarpa]
1506	18122	gi|224085976	54.05	3	3	2		82530	prolyl oligopeptidase family protein [Populus trichocarpa]
1293	18423	gi|224056200	54.01	4	2	1		48114	5-methylthioribose kinase family protein [Populus trichocarpa]
1827	2871	gi|225455386	54.01	3	2	1		65593	PREDICTED: probable pectinesterase/pectinesterase inhibitor 34 [Vitis vinifera]
1949	18176	gi|566175819	53.94	4	3	2		67279	hypothetical protein POPTR_0006s11980g [Populus trichocarpa]
1527	1615	gi|359497056	53.86	5	2	1		35941	PREDICTED: peroxidase 10-like [Vitis vinifera]
1705	18847	gi|224141239	53.79	16	2	1		15020	hypothetical protein POPTR_0017s08140g [Populus trichocarpa]
1905	18595	gi|566190935	53.74	2	1	1		61141	hypothetical protein POPTR_0010s15200g [Populus trichocarpa]
1768	18086	gi|224071469	53.58	8	3	3		50726	eukaryotic translation initiation factor 2 subunit 3 family protein [Populus trichocarpa]
1831	19686	gi|224141787	53.51	6	1	1		27379	20S proteasome beta subunit G1 family protein [Populus trichocarpa]
1843	1435	gi|225464870	53.48	9	2	2	Carbamidomethylation	37627	PREDICTED: bifunctional nitrilase/nitrile hydratase NIT4B [Vitis vinifera]
714	1200	gi|225452053	53.45	9	2	2		21963	PREDICTED: 40S ribosomal protein S7 [Vitis vinifera]
1762	1798	gi|225442268	53.44	2	1	1		73167	PREDICTED: transmembrane 9 superfamily member 7 [Vitis vinifera]
1541	1095	gi|731423779	53.41	3	3	1		63328	PREDICTED: plastidial pyruvate kinase 2 [Vitis vinifera]
1499	19350	gi|224073782	53.3	9	1	1	Carbamidomethylation	21868	proline-rich family protein [Populus trichocarpa]

1415	1991	gi|225431173	53.28	13	1	1		12904	PREDICTED: peptidyl-prolyl cis-trans isomerase Pin1 [Vitis vinifera]
1643	18537	gi|566159086	53.27	3	2	1		61936	hypothetical protein POPTR_0002s20370g [Populus trichocarpa]
1143	18948	gi|566197644	53.11	12	2	1		26318	hypothetical protein POPTR_0012s09540g [Populus trichocarpa]
2453	19707	gi|225435790	53.02	5	1	1		36898	PREDICTED: uncharacterized protein LOC100241562 [Vitis vinifera]
1621	18776	gi|566201937	53.01	19	2	2	Carbamidomethylation	9653	hypothetical protein POPTR_0014s02030g [Populus trichocarpa]
1491	18525	gi|566206477	52.89	11	2	1		24609	hypothetical protein POPTR_0015s07620g partial [Populus trichocarpa]
1988	18784	gi|566181342	52.85	4	2	1		49846	hypothetical protein POPTR_0007s14620g [Populus trichocarpa]
1783	1377	gi|225452877	52.82	4	2	2		57074	PREDICTED: cystathionine gamma-synthase chloroplastic [Vitis vinifera]
1271	1683	gi|225467712	52.79	4	2	2		62107	PREDICTED: UPF0051 protein ABCI8 chloroplastic [Vitis vinifera]
1452	1795	gi|225465530	52.78	5	2	2	Carbamidomethylation	37226	PREDICTED: cinnamoyl-CoA reductase 1 [Vitis vinifera]
2485	19210	gi|566157463	52.69	1	1	1		101527	hypothetical protein POPTR_0002s11380g [Populus trichocarpa]
1938	19253	gi|225457361	52.66	6	1	1		29994	PREDICTED: ribulose-phosphate 3-epimerase chloroplastic [Vitis vinifera]
1624	2344	gi|225443154	52.65	9	1	1	Carbamidomethylation; Oxidation (M)	18245	PREDICTED: lamin-like protein [Vitis vinifera]
2223	19709	gi|566149450	52.61	13	1	1	Carbamidomethylation	12595	ring box family protein [Populus trichocarpa]

935	18766	gi|566202435	52.57	13	2	1		22118	uridylate kinase family protein [Populus trichocarpa]
2222	19298	gi|224122632	52.56	3	1	1		66196	Monocopper oxidase-like protein SKS1 precursor [Populus trichocarpa]
1613	2336	gi|225441619	52.53	7	1	1		19639	PREDICTED: putative H/ACA ribonucleoprotein complex subunit 1-like protein 1 [Vitis vinifera]
2162	18556	gi|224067088	52.42	3	2	2	Carbamidomethylation	73926	hypothetical protein POPTR_0002s10850g [Populus trichocarpa]
1342	1215	gi|225462161	52.38	5	2	2		53484	PREDICTED: NADH dehydrogenase [ubiquinone] flavoprotein 1 mitochondrial isoform X2 [Vitis vinifera]
2483	19033	gi|526117729	52.38	2	1	1		61635	9 10[9' 10']carotenoid cleavage dioxygenase [Vitis vinifera]
1092	1422	gi|731439999	52.38	2	2	1	Carbamidomethylation	80675	PREDICTED: primary amine oxidase-like [Vitis vinifera]
2102	3619	gi|731435290	52.23	2	1	1	Carbamidomethylation	55485	PREDICTED: phosphoribosylamine--glycine ligase [Vitis vinifera]
1848	2475	gi|91984022	52.18	6	1	1		17459	cytochrome b6/f complex subunit IV (chloroplast) [Vitis vinifera]
1994	18850	gi|224141859	52.17	15	2	1		14175	putative ubiquinol-cytochrome c reductase family protein [Populus trichocarpa]
2960	19421	gi|566155879	52.08	3	1	1		53408	hypothetical protein POPTR_0002s02580g [Populus trichocarpa]
1718	18195	gi|731385121	52.03	1	3	2		173607	PREDICTED: myosin-15 [Vitis vinifera]
1957	2854	gi|731408416	51.98	4	2	1		53964	PREDICTED: serine carboxypeptidase-like 13 [Vitis vinifera]
1841	1592	gi|225459957	51.92	12	2	2		21003	PREDICTED: uncharacterized protein LOC100261996 [Vitis vinifera]

137	19143	gi|566199750	51.89	3	1	1	Carbamidomethylation	38299	hypothetical protein POPTR_0013s06300g [Populus trichocarpa]
1950	1658	gi|225435836	51.78	6	2	2		31222	PREDICTED: transcription factor Pur-alpha 1 [Vitis vinifera]
1676	18451	gi|224089557	51.78	6	2	1	Carbamidomethylation	42428	truncated acetyl Co-A acetyltransferase-like family protein [Populus trichocarpa]
1561	1762	gi|731419532	51.76	5	2	1		58564	PREDICTED: ATP-dependent 6-phosphofructokinase 5 chloroplastic [Vitis vinifera]
2107	19002	gi|224103819	51.74	4	1	1		34955	L-galactose dehydrogenase family protein [Populus trichocarpa]
1934	18854	gi|224077654	51.71	1	1	1		172709	DNA (cytosine-5)-methyltransferase AthI family protein [Populus trichocarpa]
1991	2674	gi|225430941	51.69	14	2	1	Carbamidomethylation	17767	PREDICTED: putative 4-hydroxy-4-methyl-2-oxoglutarate aldolase 2 [Vitis vinifera]
2506	19993	gi|566196533	51.58	8	2	2	Carbamidomethylation	14778	cytochrome b5 isoform Cb5-C family protein [Populus trichocarpa]
1873	18432	gi|224116788	51.57	2	1	1		96196	hypothetical protein POPTR_0011s06910g [Populus trichocarpa]
2268	1780	gi|731433292	51.28	0	1	1		414522	PREDICTED: E3 ubiquitin-protein ligase UPL1 isoform X2 [Vitis vinifera]
2430	18575	gi|566153712	51.21	5	2	2		52249	transport protein sec61 [Populus trichocarpa]
1726	19048	gi|566151438	51.13	3	1	1		43604	C2 domain-containing family protein [Populus trichocarpa]
1806	5205	gi|359482448	51.06	2	1	1		83440	PREDICTED: subtilisin-like protease [Vitis vinifera]
1117	18261	gi|224109256	51.06	6	2	2		29460	ferritin 2 precursor family protein [Populus trichocarpa]
999	2049	gi|731411015	51.03	3	1	1		46869	PREDICTED: 3-isopropylmalate dehydrogenase chloroplastic [Vitis vinifera]

2431	18607	gi|566152849	51.02	6	2	2		45355	hypothetical protein POPTR_0001s34960g [Populus trichocarpa]
2155	18200	gi|225447584	51.01	1	2	1		148746	PREDICTED: nuclear pore complex protein NUP133 [Vitis vinifera]
1514	1106	gi|225435632	50.97	3	2	2		67979	PREDICTED: formate--tetrahydrofolate ligase [Vitis vinifera]
2164	1820	gi|359478076	50.77	3	1	1	Carbamidomethylation	68073	PREDICTED: 2-isopropylmalate synthase A-like [Vitis vinifera]
2434	18933	gi|224084864	50.72	3	2	1		62868	hypothetical protein POPTR_0005s15790g [Populus trichocarpa]
1989	18802	gi|224126895	50.66	9	2	1		33437	Xyloglucan endotransglucosylase/hydrolase protein 9 precursor [Populus trichocarpa]
1677	18489	gi|224080381	50.65	4	2	2		53896	diaminopimelate decarboxylase family protein [Populus trichocarpa]
1583	18046	gi|566168917	50.64	3	3	1		113232	hypothetical protein POPTR_0005s02130g [Populus trichocarpa]
2003	19728	gi|224070184	50.34	11	1	1		18056	deoxyuridine 5'-triphosphate nucleotidohydrolase family protein [Populus trichocarpa]
2437	19080	gi|225425274	50.27	4	1	1		29736	PREDICTED: cytochrome b-c1 complex subunit Rieske-4 mitochondrial [Vitis vinifera]
1216	1098	gi|359475506	50.27	6	3	3		44050	PREDICTED: proline iminopeptidase isoform X1 [Vitis vinifera]
1859	1261	gi|225452863	50.23	3	2	1	Carbamidomethylation	58071	PREDICTED: KH domain-containing protein At4g18375 [Vitis vinifera]
1995	18957	gi|225430198	50.22	14	2	1		14707	PREDICTED: cytochrome b-c1 complex subunit 7-2 [Vitis vinifera]

1493	18942	gi|566197584	49.98	3	1	1		42529	hypothetical protein POPTR_0012s09200g [Populus trichocarpa]
2711	19046	gi|566160505	49.94	2	1	1		75650	hypothetical protein POPTR_0003s02610g [Populus trichocarpa]
1727	2348	gi|225463408	49.89	10	1	1		14802	PREDICTED: DNA-binding protein DDB_G0278111 [Vitis vinifera]
1902	1971	gi|359492139	49.88	2	2	1		94211	PREDICTED: probable methyltransferase PMT24 [Vitis vinifera]
2138	19431	gi|566151403	49.82	20	1	1		8809	hypothetical protein POPTR_0001s28090g [Populus trichocarpa]
2074	19090	gi|566204168	49.74	3	1	1		56223	Citrate synthase family protein [Populus trichocarpa]
1965	18812	gi|671741947	49.72	23	2	2		9397	photosystem II cytochrome b559 alpha subunit (chloroplast) [Camellia grandibracteata]
2177	1889	gi|359477208	49.67	2	1	1	Carbamidomethylation	84391	PREDICTED: flowering time control protein FCA isoform X2 [Vitis vinifera]
2004	2173	gi|731434735	49.64	14	1	1	Carbamidomethylation	10306	PREDICTED: LOW QUALITY PROTEIN: costars family protein [Vitis vinifera]
2090	1892	gi|359478198	49.61	3	1	1		45369	PREDICTED: acyl-[acyl-carrier-protein] desaturase chloroplastic [Vitis vinifera]
1723	18835	gi|224089058	49.59	5	2	1		49180	aspartate transaminase family protein [Populus trichocarpa]
2169	18926	gi|225452167	49.56	4	2	2		56983	PREDICTED: histone-lysine N-methyltransferase setd3 isoform X2 [Vitis vinifera]
2224	1847	gi|731389447	49.53	2	1	1		84064	PREDICTED: acylamino-acid-releasing enzyme isoform X2 [Vitis vinifera]

1999	3539	gi|731378986	49.52	13	1	1		13972	PREDICTED: LOW QUALITY PROTEIN: mitochondrial import inner membrane translocase subunit Tim13 [Vitis vinifera]
1826	1989	gi|731417792	49.47	2	1	1		72111	PREDICTED: KH domain-containing protein At4g18375 [Vitis vinifera]
1653	2026	gi|359492937	49.4	2	1	1		46528	PREDICTED: aspartic proteinase nepenthesin-1 [Vitis vinifera]
1625	19721	gi|566206526	49.38	6	1	1		23501	hypothetical protein POPTR_0015s07940g [Populus trichocarpa]
1822	1981	gi|731400731	49.31	7	2	2		35100	PREDICTED: histone deacetylase HDT1 [Vitis vinifera]
1589	18589	gi|566146861	49.23	5	3	1		61877	Pyruvate kinase isozyme G family protein [Populus trichocarpa]
1426	18636	gi|224126029	49.2	3	1	1		37880	co-chaperone grpE family protein [Populus trichocarpa]
2103	18565	gi|731375306	49.19	4	2	2		65748	PREDICTED: delta(24)-sterol reductase [Vitis vinifera]
1704	1935	gi|731400451	49.18	2	1	1		47908	PREDICTED: cystathionine beta-lyase chloroplastic isoform X2 [Vitis vinifera]
1866	19066	gi|566189139	49.12	10	1	1		18857	hypothetical protein POPTR_0010s05030g [Populus trichocarpa]
1336	19126	gi|566206069	49.12	1	2	1		96052	hypothetical protein POPTR_0015s05790g [Populus trichocarpa]
2225	19727	gi|224125772	49.11	7	1	1		23609	hypothetical protein POPTR_0013s04840g [Populus trichocarpa]
2316	1725	gi|225430834	49.02	2	1	1		71703	PREDICTED: probable Xaa-Pro aminopeptidase P [Vitis vinifera]
2001	19276	gi|566198430	49.02	3	1	1	Carbamidomethylation	54941	hypothetical protein POPTR_0012s14150g [Populus trichocarpa]
1847	1931	gi|225453329	48.78	11	1	1		12870	PREDICTED: tubulin-folding cofactor A [Vitis vinifera]

3063	1669	gi|225456459	48.68	5	1	1		42395	PREDICTED: V-type proton ATPase subunit C isoform X1 [Vitis vinifera]
2427	18425	gi|566186422	48.67	3	2	2		83467	hypothetical protein POPTR_0009s05400g [Populus trichocarpa]
2713	19211	gi|566211720	48.62	2	1	1		131864	hypothetical protein POPTR_0017s06170g [Populus trichocarpa]
1845	18906	gi|566208409	48.6	3	2	1		57917	hypothetical protein POPTR_0016s03090g [Populus trichocarpa]
1710	19456	gi|566208324	48.56	2	1	1	Carbamidomethylation	59394	hypothetical protein POPTR_0016s02620g [Populus trichocarpa]
2312	19120	gi|566149278	48.55	3	1	1	Carbamidomethylation	42201	hypothetical protein POPTR_0001s15980g [Populus trichocarpa]
2005	19737	gi|224070843	48.44	8	1	1		19159	glutaredoxin family protein [Populus trichocarpa]
1865	1860	gi|359474339	48.43	3	1	1		45436	PREDICTED: probable transaldolase isoform X1 [Vitis vinifera]
1790	19420	gi|566153420	48.3	7	1	1		26572	hypothetical protein POPTR_0001s38130g [Populus trichocarpa]
1518	19744	gi|224137260	48.13	12	1	1		11459	hypothetical protein POPTR_0016s01100g [Populus trichocarpa]
1898	18093	gi|566214132	48.08	3	3	3		108955	hypothetical protein POPTR_0018s03980g [Populus trichocarpa]
1829	1868	gi|731403923	48.04	2	2	1		86106	PREDICTED: cycloartenol Synthase isoform X2 [Vitis vinifera]
2348	1891	gi|731386596	47.97	1	1	1	Carbamidomethylation	287778	PREDICTED: translational activator GCN1 [Vitis vinifera]
2091	19131	gi|224073524	47.92	8	1	1		16505	hypothetical protein POPTR_0003s03980g [Populus trichocarpa]

800	2055	gi|731433025	47.89	9	1	1		12656	PREDICTED: mitochondrial pyruvate carrier 2-like isoform X1 [Vitis vinifera]
2081	18245	gi|224123934	47.87	9	2	2	Oxidation (M)	31085	hypothetical protein POPTR_0013s06360g [Populus trichocarpa]
1802	1067	gi|225449362	47.78	6	2	2	Carbamidomethylation	35638	PREDICTED: serine/threonine-protein phosphatase PP1 isozyme 4 [Vitis vinifera]
2059	18860	gi|566192855	47.63	3	1	1		34711	hypothetical protein POPTR_0010s26140g [Populus trichocarpa]
1810	2400	gi|225434808	47.61	3	1	1		32777	PREDICTED: eukaryotic translation initiation factor 3 subunit G-B [Vitis vinifera]
3064	19746	gi|224085103	47.59	22	1	1	Carbamidomethylation	7996	ubiquinol-cytochrome C reductase complex 7.8 kDa family protein [Populus trichocarpa]
2353	1913	gi|731398376	47.56	3	1	1		49041	PREDICTED: D-lactate dehydrogenase [cytochrome] mitochondrial isoform X2 [Vitis vinifera]
2188	19570	gi|566214582	47.54	7	1	1		23541	SNF7 family protein [Populus trichocarpa]
2069	1758	gi|359495836	47.51	3	2	1		64385	PREDICTED: galacturonosyltransferase 8 [Vitis vinifera]
2677	2143	gi|225444537	47.44	3	1	1	Carbamidomethylation	42935	PREDICTED: uncharacterized protein LOC100253669 [Vitis vinifera]
2226	2473	gi|225465847	47.4	8	1	1		21605	PREDICTED: NADPH:quinone oxidoreductase [Vitis vinifera]
1579	3312	gi|225439821	47.29	1	2	1	Carbamidomethylation	116087	PREDICTED: calcium-transporting ATPase 4 endoplasmic reticulum-type-like [Vitis vinifera]
1808	19159	gi|224101399	47.26	2	1	1		49569	kinase family protein [Populus trichocarpa]
2218	19041	gi|566177717	47.25	7	1	1	Carbamidomethylation	20905	zinc knuckle family protein [Populus trichocarpa]

2955	19336	gi|225444159	47.25	5	1	1		32002	PREDICTED: coatomer subunit epsilon-1 [Vitis vinifera]
1746	1735	gi|225451318	47.24	4	1	1		37997	PREDICTED: probable aldo-keto reductase 1 [Vitis vinifera]
2135	2189	gi|526118301	47.03	4	1	1		41463	cysteine protease precursor [Vitis vinifera]
2167	18765	gi|566188404	47.01	3	1	1		40604	pantothenate kinase-related family protein [Populus trichocarpa]
1963	1383	gi|359476759	46.93	3	1	1		47072	PREDICTED: basic leucine zipper and W2 domain-containing protein 2 [Vitis vinifera]
2082	18287	gi|224120380	46.79	4	2	1		61938	hypothetical protein POPTR_0012s03790g [Populus trichocarpa]
2227	1854	gi|225470285	46.75	5	1	1		32382	PREDICTED: ATP-dependent Clp protease proteolytic subunit-related protein 2 chloroplastic [Vitis vinifera]
1702	1695	gi|731408423	46.68	4	2	2	Carbamidomethylation	43098	PREDICTED: serine carboxypeptidase-like 18 isoform X4 [Vitis vinifera]
1651	18227	gi|566196001	46.66	13	2	1	Carbamidomethylation	17516	dimethylmenaquinone methyltransferase family protein [Populus trichocarpa]
1993	3173	gi|731382683	46.63	5	2	1	Carbamidomethylation	56461	PREDICTED: probable serine/threonine-protein kinase At4g35230 [Vitis vinifera]
2221	19296	gi|566205313	46.58	5	1	1		28442	glucosamine/galactosamine-6-phosphate isomerase family protein [Populus trichocarpa]
2009	19793	gi|566181894	46.57	2	1	1		58001	hypothetical protein POPTR_0008s02250g [Populus trichocarpa]
2396	18831	gi|224126789	46.54	2	1	1		65325	pyridine nucleotide-disulfide oxidoreductase family protein [Populus trichocarpa]

1638	1861	gi|731428311	46.48	2	1	1		51458	PREDICTED: bifunctional aspartate aminotransferase and glutamate/aspartate-prephenate aminotransferase-like [Vitis vinifera]
2125	18407	gi|566158256	46.35	6	2	1	Carbamidomethylation	39552	hypothetical protein POPTR_0002s15850g [Populus trichocarpa]
2394	18538	gi|224090627	46.3	2	2	2	Carbamidomethylation	157531	WD-40 repeat family protein [Populus trichocarpa]
2008	19792	gi|566210696	46.12	3	1	1		42582	hypothetical protein POPTR_0017s01530g [Populus trichocarpa]
908	18901	gi|566198700	46.09	4	2	1		42593	hypothetical protein POPTR_0013s00760g [Populus trichocarpa]
2163	1686	gi|225456369	46.08	9	2	2		23558	PREDICTED: nuclear transcription factor Y subunit C-1 isoform X1 [Vitis vinifera]
2195	19773	gi|566160873	46.01	9	1	1	Carbamidomethylation	25118	hypothetical protein POPTR_0003s04700g [Populus trichocarpa]
2961	2398	gi|225435925	46	3	1	1	Carbamidomethylation	50361	PREDICTED: cysteine desulfurase 2 chloroplastic [Vitis vinifera]
1244	2470	gi|225442639	45.9	9	1	1		15488	PREDICTED: uncharacterized protein LOC100260053 [Vitis vinifera]
2044	1904	gi|225431685	45.86	2	2	1		92333	PREDICTED: probable methyltransferase PMT26 [Vitis vinifera]
2801	2365	gi|225441864	45.85	3	1	1	Carbamidomethylation	57917	PREDICTED: thioredoxin reductase NTRC [Vitis vinifera]
1916	2154	gi|225458519	45.77	2	1	1	Carbamidomethylation	75018	PREDICTED: uncharacterized protein LOC100267853 [Vitis vinifera]
1279	18829	gi|566179807	45.75	2	1	1		51892	hypothetical protein POPTR_0007s05880g [Populus trichocarpa]

2491	19769	gi|566175218	45.72	10	1	1	Carbamidomethylation	12754	hypothetical protein POPTR_0006s08530g [Populus trichocarpa]
2006	2585	gi|225435395	45.71	3	1	1	Carbamidomethylation	35206	PREDICTED: cinnamoyl-CoA reductase 1 [Vitis vinifera]
2105	1794	gi|225452562	45.69	3	1	1		35739	PREDICTED: peroxidase 31-like [Vitis vinifera]
1728	2412	gi|225453100	45.68	4	1	1		34790	PREDICTED: ATP-dependent Clp protease proteolytic subunit 3 chloroplastic [Vitis vinifera]
2007	2373	gi|731384063	45.64	3	1	1		43516	PREDICTED: LL-diaminopimelate aminotransferase chloroplastic isoform X7 [Vitis vinifera]
2172	19167	gi|224143157	45.59	1	1	1		73679	clathrin assembly family protein [Populus trichocarpa]
2180	2415	gi|225450075	45.55	3	1	1		41952	PREDICTED: peptidyl-prolyl cis-trans isomerase FKBP42 [Vitis vinifera]
2037	19354	gi|224062258	45.51	4	1	1		28277	NADH-ubiquinone oxidoreductase 24 kDa subunit family protein [Populus trichocarpa]
1084	1395	gi|731435511	45.49	1	2	1		152684	PREDICTED: putative disease resistance RPP13-like protein 1 isoform X2 [Vitis vinifera]
2395	18626	gi|225454791	45.39	1	1	1		70066	PREDICTED: ATPase family AAA domain-containing protein 3-B [Vitis vinifera]
2228	19806	gi|224113431	45.32	11	1	1		9789	hypothetical protein POPTR_0010s24670g [Populus trichocarpa]
2793	19082	gi|731405196	45.27	1	1	1	Carbamidomethylation	102309	PREDICTED: uncharacterized protein LOC100254898 isoform X2 [Vitis vinifera]
2101	18450	gi|566210049	45.19	1	1	1		96663	hypothetical protein POPTR_0016s13160g [Populus trichocarpa]
794	2504	gi|731416298	45.14	4	1	1		25123	PREDICTED: eukaryotic translation initiation factor 3 subunit J [Vitis vinifera]

2136	19405	gi|566259912	45.13	3	1	1		52371	hypothetical protein POPTR_0022s00470g [Populus trichocarpa]
1997	1711	gi|225466119	45.04	3	1	1		42250	PREDICTED: luc7-like protein 3 [Vitis vinifera]
2408	19489	gi|224095005	45.03	4	1	1	Carbamidomethylation	33972	xyloglucan endotransglycosylase hydrolase 2 family protein [Populus trichocarpa]
2176	1726	gi|225440169	45.01	2	1	1		57401	PREDICTED: cytochrome P450 98A2 [Vitis vinifera]
3068	2417	gi|731400181	45	1	1	1		80319	PREDICTED: uncharacterized protein C18orf8 isoform X2 [Vitis vinifera]
2753	19149	gi|566215907	44.95	1	1	1		131624	hypothetical protein POPTR_0018s14630g [Populus trichocarpa]
1914	19018	gi|224055587	44.84	4	1	1		40024	GHMP kinase-related family protein [Populus trichocarpa]
1895	18935	gi|566214668	44.82	3	1	1		53306	casein kinase family protein [Populus trichocarpa]
2372	19110	gi|566206676	44.8	2	1	1		79072	hypothetical protein POPTR_0015s08830g [Populus trichocarpa]
3065	19783	gi|731396358	44.75	3	1	1		46292	PREDICTED: 3-ketoacyl-CoA thiolase 2 peroxisomal-like [Vitis vinifera]
2190	19580	gi|566155691	44.68	1	1	1		99018	ubiquitin family protein [Populus trichocarpa]
2481	18891	gi|566152365	44.66	5	2	2		43020	Vacuolar ATP synthase subunit C family protein [Populus trichocarpa]
1953	3245	gi|225460680	44.63	7	2	2		37122	PREDICTED: fructose-1 6-bisphosphatase cytosolic isoform X2 [Vitis vinifera]
2541	1408	gi|731408196	44.58	2	1	1		87158	PREDICTED: subtilisin-like protease isoform X4 [Vitis vinifera]
2879	19784	gi|566180489	44.57	4	1	1		35792	Peroxidase 50 precursor family protein [Populus trichocarpa]

1729	19820	gi|566210973	44.55	6	1	1		15927	hypothetical protein POPTR_0017s02810g [Populus trichocarpa]
2010	19808	gi|566160273	44.54	6	1	1	Carbamidomethylation	17554	hypothetical protein POPTR_0003s01440g [Populus trichocarpa]
944	1764	gi|359478415	44.49	3	2	1		58697	PREDICTED: beta-glucosidase 12 [Vitis vinifera]
1939	19259	gi|566215941	44.44	2	1	1		50246	hypothetical protein POPTR_0018s14790g [Populus trichocarpa]
1321	19117	gi|566173131	44.4	3	1	1		39060	dihydroflavonol reductase family protein [Populus trichocarpa]
2178	19380	gi|731407226	44.33	4	1	1	Carbamidomethylation	37934	PREDICTED: 2-alkenal reductase (NADP(+)-dependent) [Vitis vinifera]
2480	18864	gi|731383454	44.29	6	2	2		43734	PREDICTED: probable N-acetyl-gamma-glutamyl-phosphate reductase chloroplastic [Vitis vinifera]
2963	19511	gi|566151722	44.16	2	1	1		68698	hypothetical protein POPTR_0001s29700g [Populus trichocarpa]
1622	1778	gi|731427762	44.15	2	2	1		78808	PREDICTED: prolyl endopeptidase isoform X2 [Vitis vinifera]
1850	19810	gi|566163928	44.15	6	1	1		21587	disease resistance-responsive family protein [Populus trichocarpa]
1956	1351	gi|225464146	44.11	6	2	1		32622	PREDICTED: protein transport protein SEC13 homolog B [Vitis vinifera]
1844	1733	gi|225429462	44.1	2	2	2		71749	PREDICTED: far upstream element-binding protein 2 [Vitis vinifera]
2954	2469	gi|731394805	44.07	5	1	1		30948	PREDICTED: ribosome-recycling factor chloroplastic [Vitis vinifera]
909	18903	gi|566146533	44.05	14	2	2		10147	acyl-CoA-binding family protein [Populus trichocarpa]

2137	2392	gi|731382564	44.04	3	1	1		53798	PREDICTED: aminoacylase-1 isoform X2 [Vitis vinifera]
2400	2395	gi|225435776	44.03	3	1	1		66471	PREDICTED: probable lysine--tRNA ligase cytoplasmic [Vitis vinifera]
1968	18997	gi|566236086	44	2	1	1		48154	hypothetical protein POPTR_0019s08450g [Populus trichocarpa]
2435	2151	gi|225432664	43.99	2	1	1		64004	PREDICTED: uncharacterized protein LOC100259596 [Vitis vinifera]
3066	19794	gi|224073306	43.97	2	1	1		50609	hypothetical protein POPTR_0003s01420g [Populus trichocarpa]
3069	19817	gi|566215175	43.9	19	1	1		7135	hypothetical protein POPTR_0018s10540g [Populus trichocarpa]
1973	2457	gi|526117557	43.88	4	1	1		32485	alpha-soluble NSF attachment protein [Vitis vinifera]
1786	19216	gi|566170063	43.84	4	1	1		32177	hypothetical protein POPTR_0005s07750g [Populus trichocarpa]
2301	1805	gi|731402392	43.82	3	1	1		65873	PREDICTED: malonate--CoA ligase isoform X3 [Vitis vinifera]
1701	18582	gi|566156002	43.75	6	2	1	Carbamidomethylation	37272	hypothetical protein POPTR_0002s03260g [Populus trichocarpa]
2075	1877	gi|731392751	43.72	3	1	1		45321	PREDICTED: ATPase GET3-like [Vitis vinifera]
2182	2477	gi|731383208	43.33	4	1	1		29098	PREDICTED: heterodimeric geranylgeranyl pyrophosphate synthase small subunit chloroplastic-like [Vitis vinifera]
2953	2434	gi|225446899	43.26	2	1	1		70904	PREDICTED: DEAD-box ATP-dependent RNA helicase 53-like [Vitis vinifera]
1912	18960	gi|566255736	43.22	5	1	1		15873	hypothetical protein POPTR_0483s00200g partial [Populus trichocarpa]

2073	2410	gi|731412185	43.17	2	1	1		67073	PREDICTED: phosphoinositide phospholipase C 6 [Vitis vinifera]
2076	19116	gi|224078882	43.15	5	1	1	Carbamidomethylation	34470	oxidoreductase family protein [Populus trichocarpa]
1908	18882	gi|224075096	43.1	1	1	1		73889	acyl-coA synthetase family protein [Populus trichocarpa]
2443	19371	gi|566184421	43.09	3	1	1		45388	hypothetical protein POPTR_0008s17030g [Populus trichocarpa]
2230	2420	gi|225447221	42.77	6	1	1		18627	PREDICTED: photosystem II repair protein PSB27-H1 chloroplastic [Vitis vinifera]
2229	19825	gi|224096296	42.74	3	1	1		40670	hypothetical protein POPTR_0007s06500g [Populus trichocarpa]
2290	1975	gi|731432424	42.67	1	1	1		90087	PREDICTED: ubiquitin carboxyl-terminal hydrolase 14 [Vitis vinifera]
1189	18932	gi|731370779	42.6	2	2	1		114446	PREDICTED: phosphoenolpyruvate carboxylase 4 isoform X2 [Vitis vinifera]
3067	19816	gi|566197446	42.56	3	1	1		44436	hypothetical protein POPTR_0012s08440g [Populus trichocarpa]
1534	19185	gi|566157031	42.54	2	2	1		61576	glycine hydroxymethyltransferase family protein [Populus trichocarpa]
2050	1703	gi|731376062	42.52	2	1	1		83343	PREDICTED: urease isoform X2 [Vitis vinifera]
3070	2554	gi|731387738	42.48	3	1	1		36411	PREDICTED: SAL1 phosphatase isoform X4 [Vitis vinifera]
1280	19106	gi|224078826	42.44	4	1	1		24905	Oxygen-evolving enhancer protein 3-1 [Populus trichocarpa]
2796	19136	gi|566185250	42.24	2	1	1		55234	hypothetical protein POPTR_0008s21950g [Populus trichocarpa]
2802	19475	gi|731439030	42.22	5	1	1		34963	PREDICTED: ATP-dependent Clp protease proteolytic subunit-related protein 4 chloroplastic-like [Vitis vinifera]

2183	19483	gi|225450514	42.2	8	1	1		15973	PREDICTED: trafficking protein particle complex subunit 4 [Vitis vinifera]
2233	19884	gi|225423730	42.08	6	1	1		16692	PREDICTED: glycine cleavage system H protein 2 mitochondrial [Vitis vinifera]
2488	2625	gi|359494696	42.03	4	1	1		43512	PREDICTED: fructose-1 6-bisphosphatase chloroplastic [Vitis vinifera]
1967	2206	gi|225455268	42.02	3	1	1		34609	PREDICTED: probable 6-phosphogluconolactonase 4 chloroplastic [Vitis vinifera]
2012	2478	gi|225430602	42	5	1	1		21039	PREDICTED: protein OPI10 homolog [Vitis vinifera]
612	19403	gi|566166342	41.98	1	1	1		95030	hypothetical protein POPTR_0004s13420g partial [Populus trichocarpa]
2350	1816	gi|225433688	41.97	3	1	1		38722	PREDICTED: eukaryotic translation initiation factor 3 subunit H [Vitis vinifera]
1465	3036	gi|731428733	41.88	0	1	1		407701	PREDICTED: E3 ubiquitin-protein ligase UPL2-like [Vitis vinifera]
1519	19858	gi|566194536	41.84	4	1	1		28624	hypothetical protein POPTR_0011s09130g [Populus trichocarpa]
2712	19140	gi|225437598	41.83	1	1	1		130089	PREDICTED: nodal modulator 1 [Vitis vinifera]
1500	19865	gi|566174225	41.79	5	1	1		22376	hypothetical protein POPTR_0006s03170g [Populus trichocarpa]
2011	19863	gi|566212628	41.72	7	1	1		17449	hypothetical protein POPTR_0017s10800g [Populus trichocarpa]
1427	18641	gi|566169633	41.69	5	1	1		30715	hypothetical protein POPTR_0005s05400g [Populus trichocarpa]
3071	19870	gi|566170203	41.5	2	1	1		70724	hypothetical protein POPTR_0005s08490g [Populus trichocarpa]

2310	4083	gi|225425706	41.34	2	1	1		69593	PREDICTED: probable methyltransferase PMT2 [Vitis vinifera]
2000	19229	gi|566165862	41.22	2	1	1		41915	hypothetical protein POPTR_0004s10130g [Populus trichocarpa]
2493	2091	gi|225450155	41.2	4	1	1		26703	PREDICTED: thylakoid lumenal 19 kDa protein chloroplastic [Vitis vinifera]
1517	1743	gi|225441809	41.19	11	1	1		8917	PREDICTED: uncharacterized protein LOC100245204 isoform X3 [Vitis vinifera]
2231	19838	gi|731395888	41.18	3	1	1		44982	PREDICTED: protein notum homolog [Vitis vinifera]
2492	19881	gi|224128482	41.13	13	1	1	Carbamidomethylation	9705	NADH-ubiquinone oxidoreductase-related family protein [Populus trichocarpa]
1626	19883	gi|566189043	41.08	4	1	1	Carbamidomethylation	36173	Peroxidase 6 precursor family protein [Populus trichocarpa]
2289	4368	gi|225440262	41.06	1	1	1		142059	PREDICTED: splicing factor 3B subunit 1 [Vitis vinifera]
2482	18956	gi|566162026	40.97	9	2	2	Carbamidomethylation	27183	hypothetical protein POPTR_0003s10720g [Populus trichocarpa]
1906	3919	gi|225461593	40.95	1	1	1		62767	PREDICTED: apoptosis inhibitor 5 [Vitis vinifera]
2714	19480	gi|224112341	40.94	3	1	1		46306	latex abundant family protein [Populus trichocarpa]
2232	2958	gi|731419100	40.93	6	1	1	Carbamidomethylation	19892	PREDICTED: universal stress protein A-like protein [Vitis vinifera]
2113	1793	gi|225438019	40.9	4	1	1		44983	PREDICTED: heterogeneous nuclear ribonucleoprotein 1-like [Vitis vinifera]
2181	19463	gi|566214148	40.88	3	1	1		45872	hypothetical protein POPTR_0018s04060g [Populus trichocarpa]
2406	19378	gi|566229616	40.84	4	1	1		39992	hypothetical protein POPTR_0019s04140g [Populus trichocarpa]

1970	19264	gi|225423909	40.83	1	1	1		88054	PREDICTED: mediator of RNA polymerase II transcription subunit 25 [Vitis vinifera]
1730	2500	gi|526117641	40.81	4	1	1		21825	type II peroxiredoxin F [Vitis vinifera]
2184	2596	gi|225453513	40.75	12	1	1		8552	PREDICTED: protein transport protein Sec61 subunit beta-like [Vitis vinifera]
2494	1810	gi|225464882	40.73	2	1	1		62406	PREDICTED: nicalin-1 [Vitis vinifera]
2375	1992	gi|225453494	40.71	1	1	1		82431	PREDICTED: elongation factor G-2 mitochondrial [Vitis vinifera]
2484	19061	gi|224094809	40.69	2	1	1		62357	hypothetical protein POPTR_0007s13050g [Populus trichocarpa]
2131	19169	gi|225464210	40.69	4	1	1	Carbamidomethylation	49642	PREDICTED: probable pectate lyase 8 [Vitis vinifera]
2800	2087	gi|731440022	40.67	3	1	1		73346	PREDICTED: phosphoenolpyruvate carboxykinase [ATP]-like [Vitis vinifera]
2870	19184	gi|731399255	40.52	3	1	1		52783	PREDICTED: dnaJ protein P58IPK homolog [Vitis vinifera]
2234	19917	gi|566157925	40.31	3	1	1		38251	transcription factor family protein [Populus trichocarpa]
2436	18967	gi|224143772	40.28	2	1	1		72623	calmodulin-binding family protein [Populus trichocarpa]
2441	19360	gi|224106974	40.26	4	1	1		29365	SNARE 12 family protein [Populus trichocarpa]
2869	19142	gi|566146661	40.21	3	1	1		54980	GHMP kinase family protein [Populus trichocarpa]
1475	1676	gi|225428560	40.19	2	2	1		83942	PREDICTED: phospholipase A-2-activating protein isoform X3 [Vitis vinifera]
2454	2413	gi|359475077	40.11	2	1	1	Carbamidomethylation	75336	PREDICTED: tRNA-dihydrouridine(47) synthase [NAD(P)(+)]-like [Vitis vinifera]
1532	5075	gi|225424713	40.09	2	2	1		96484	PREDICTED: AP-1 complex subunit gamma-2 [Vitis vinifera]

1851	2533	gi|359494249	40.09	4	1	1		21360	PREDICTED: photosystem I reaction center subunit V chloroplastic [Vitis vinifera]
2496	2458	gi|731433370	40.06	6	1	1		26707	PREDICTED: protein CWC15 homolog isoform X2 [Vitis vinifera]
2399	19150	gi|566182120	40.03	4	1	1		30970	hypothetical protein POPTR_0008s03290g [Populus trichocarpa]
3072	19879	gi|566209743	39.91	5	1	1		26607	hypothetical protein POPTR_0016s11470g [Populus trichocarpa]
1832	2187	gi|359496589	39.9	3	1	1		41483	PREDICTED: clathrin light chain 1 [Vitis vinifera]
1731	19921	gi|566210424	39.76	3	1	1		35704	hypothetical protein POPTR_0017s00350g [Populus trichocarpa]
1154	19118	gi|224124994	39.7	5	1	1		17963	hypothetical protein POPTR_0013s00850g [Populus trichocarpa]
1678	19364	gi|566164258	39.7	2	1	1		59785	hypothetical protein POPTR_0004s00900g [Populus trichocarpa]
1890	18988	gi|224072767	39.68	4	1	1		29070	hypothetical protein POPTR_0003s18420g [Populus trichocarpa]
2165	18638	gi|224093896	39.58	7	2	1		27844	hypothetical protein POPTR_0007s06770g [Populus trichocarpa]
2455	19908	gi|731409599	39.54	3	1	1		39025	PREDICTED: GDSL esterase/lipase At4g26790-like [Vitis vinifera]
2089	18993	gi|566235447	39.48	3	1	1	Carbamidomethylation	30476	hypothetical protein POPTR_0019s08170g partial [Populus trichocarpa]
2956	19343	gi|224115158	39.3	8	1	1		16558	RNA-binding family protein [Populus trichocarpa]
2495	2528	gi|225433606	39.24	3	1	1		46608	PREDICTED: UBX domain-containing protein 4 [Vitis vinifera]

2499	19925	gi|224079549	39.22	3	1	1	Carbamidomethylation	44567	METHIONINE AMINOPEPTIDASE 2A family protein [Populus trichocarpa]
2634	19152	gi|225455232	39.16	4	1	1		39694	PREDICTED: polyadenylate-binding protein-interacting protein 12 [Vitis vinifera]
2611	18970	gi|566167505	39.11	2	1	1		112715	adaptin family protein [Populus trichocarpa]
2191	19591	gi|224117732	39.08	1	1	1		104104	nucleoporin family protein [Populus trichocarpa]
2497	2502	gi|225424908	38.94	4	1	1		29426	PREDICTED: mitochondrial outer membrane protein porin of 36 kDa [Vitis vinifera]
1706	3386	gi|731395454	38.9	7	2	2		19689	PREDICTED: uncharacterized protein OsI_027940 isoform X2 [Vitis vinifera]
2072	2136	gi|731388161	38.88	2	1	1		80692	PREDICTED: uncharacterized protein LOC100258488 isoform X2 [Vitis vinifera]
1915	19022	gi|566168505	38.8	2	1	1		79078	hypothetical protein POPTR_0005s00240g [Populus trichocarpa]
2502	19943	gi|566210850	38.76	3	1	1		41273	hypothetical protein POPTR_0017s02230g [Populus trichocarpa]
2498	19923	gi|224074373	38.68	3	1	1		44452	26S proteasome regulatory subunit family protein [Populus trichocarpa]
2873	5119	gi|731392421	38.64	3	1	1		32565	PREDICTED: glutathione S-transferase L3-like isoform X2 [Vitis vinifera]
2500	2524	gi|225452110	38.44	10	1	1	Carbamidomethylation	9858	PREDICTED: non-specific lipid-transfer protein 2-like [Vitis vinifera]
2708	18968	gi|224103873	38.34	4	1	1	Carbamidomethylation	35544	putative cinnamoyl-CoA reductase family protein [Populus trichocarpa]
1590	18890	gi|566159528	38.31	2	1	1		59138	acyl-CoA dehydrogenase-related family protein [Populus trichocarpa]

2179	2100	gi|731427257	38.25	4	1	1		22951	PREDICTED: miraculin-like [Vitis vinifera]
1455	19941	gi|566186111	38.24	8	1	1		12191	hypothetical protein POPTR_0009s03520g partial [Populus trichocarpa]
2501	19934	gi|731379459	38.22	3	1	1		51544	PREDICTED: methyl-CpG-binding domain-containing protein 13 isoform X4 [Vitis vinifera]
1961	18619	gi|225468302	38.21	7	2	1	Carbamidomethylation	35578	PREDICTED: peroxidase 66 [Vitis vinifera]
2875	19423	gi|566213671	38.17	3	1	1		41079	hypothetical protein POPTR_0018s01560g [Populus trichocarpa]
2452	2544	gi|731382451	38.15	2	1	1		59004	PREDICTED: bifunctional dihydrofolate reductase-thymidylate synthase-like [Vitis vinifera]
2235	19947	gi|224084924	38.12	4	1	1	Carbamidomethylation	22828	Proteasome subunit beta type 3-2 family protein [Populus trichocarpa]
2140	19946	gi|566206307	38.09	3	1	1		42484	nuclear transport factor 2 family protein [Populus trichocarpa]
1943	2560	gi|225428263	37.89	7	1	1		14294	PREDICTED: small nuclear ribonucleoprotein Sm D3 [Vitis vinifera]
1614	20214	gi|566156678	37.87	6	1	1		23173	hypothetical protein POPTR_0002s07090g [Populus trichocarpa]
2403	19309	gi|224097184	37.71	2	1	1		73308	Phosphoenolpyruvate carboxykinase family protein [Populus trichocarpa]
2192	1899	gi|225444143	37.65	3	1	1		56177	PREDICTED: patellin-4 [Vitis vinifera]
2369	18780	gi|224141359	37.61	3	1	1		40664	hypothetical protein POPTR_0017s11530g [Populus trichocarpa]
1886	19065	gi|359476173	37.59	2	1	1		64986	PREDICTED: cysteine--tRNA ligase cytoplasmic isoform X1 [Vitis vinifera]

2130	19030	gi|566167266	37.58	4	1	1		37830	hypothetical protein POPTR_0004s18610g [Populus trichocarpa]
2958	19372	gi|731438600	37.49	3	1	1		59387	PREDICTED: serine--tRNA ligase mitochondrial [Vitis vinifera]
2456	1881	gi|731426493	37.47	5	1	1	Carbamidomethylation	34081	PREDICTED: inositol polyphosphate multikinase beta [Vitis vinifera]
2451	19586	gi|566205032	37.46	3	1	1	Carbamidomethylation	37930	hypothetical protein POPTR_0014s18850g [Populus trichocarpa]
1627	2600	gi|526117629	37.33	8	1	1	Carbamidomethylation	11686	lipid transfer protein precursor [Vitis vinifera]
2171	19115	gi|566156766	37.31	2	2	1	Carbamidomethylation	91787	hypothetical protein POPTR_0002s07640g [Populus trichocarpa]
3075	2014	gi|225446046	37.28	3	1	1		42983	PREDICTED: afadin- and alpha-actinin-binding protein isoform X2 [Vitis vinifera]
2449	5615	gi|225470242	37.24	6	1	1		16680	PREDICTED: uncharacterized protein At5g48480 [Vitis vinifera]
2013	19960	gi|566150184	37.15	5	1	1		23580	hypothetical protein POPTR_0001s21460g [Populus trichocarpa]
1907	18840	gi|731433439	37.03	1	1	1		203049	PREDICTED: proteasome activator subunit 4 [Vitis vinifera]
2429	3034	gi|225463799	37	15	2	2	Carbamidomethylation	13178	PREDICTED: transcription elongation factor SPT4 homolog 2 [Vitis vinifera]
2185	19506	gi|566167219	36.89	1	1	1		69580	hypothetical protein POPTR_0004s18320g [Populus trichocarpa]
3073	19953	gi|566205586	36.86	4	1	1		33515	hypothetical protein POPTR_0015s03360g [Populus trichocarpa]
2503	19961	gi|566204052	36.85	2	1	1		46261	hypothetical protein POPTR_0014s13120g [Populus trichocarpa]
1245	19970	gi|224135953	36.79	16	1	1		6931	40S ribosomal protein S30 [Populus trichocarpa]

2174	2259	gi|225431922	36.77	13	1	1		13225	PREDICTED: DNA-directed RNA polymerases II IV and V subunit 9A [Vitis vinifera]
2442	19369	gi|224067716	36.77	11	1	1	Carbamidomethylation	13535	hypothetical protein POPTR_0002s14760g [Populus trichocarpa]
2966	19600	gi|566153916	36.74	2	1	1		57345	hypothetical protein POPTR_0001s40690g [Populus trichocarpa]
1910	18888	gi|225423849	36.73	2	1	1		65257	PREDICTED: protein decapping 5 [Vitis vinifera]
2134	19232	gi|224077516	36.71	2	1	1		59212	4-coumarate--CoA ligase family protein [Populus trichocarpa]
3077	19979	gi|566188099	36.65	12	1	1		8182	hypothetical protein POPTR_0009s15150g [Populus trichocarpa]
1833	2135	gi|731379048	36.54	1	1	1		106833	PREDICTED: nuclear pore complex protein NUP98A [Vitis vinifera]
2965	19598	gi|224126151	36.54	3	1	1		50523	transferase family protein [Populus trichocarpa]
3074	19955	gi|225445842	36.48	6	1	1		21531	PREDICTED: uncharacterized protein LOC100243229 [Vitis vinifera]
2109	2594	gi|731382912	36.41	2	1	1		81725	PREDICTED: subtilisin-like protease [Vitis vinifera]
2795	19146	gi|566160209	36.27	2	1	1	Carbamidomethylation	54758	hypothetical protein POPTR_0003s011001g partial [Populus trichocarpa]
2504	2289	gi|731384376	36.26	4	1	1		35786	PREDICTED: cinnamoyl-CoA reductase 1 [Vitis vinifera]
2440	19328	gi|225444641	36.25	2	1	1		59678	PREDICTED: 3-oxoacyl-[acyl-carrier-protein] synthase II chloroplastic [Vitis vinifera]
2957	19353	gi|566155202	36.24	5	1	1		20741	hypothetical protein POPTR_0001s46330g partial [Populus trichocarpa]
2446	19439	gi|224117556	36.24	4	1	1		25438	glutathione transferase family protein [Populus trichocarpa]

2505	19986	gi|224117028	36.22	8	1	1		15358	hypothetical protein POPTR_0011s11080g [Populus trichocarpa]
2236	19969	gi|225469438	36.2	6	1	1		17534	PREDICTED: pollen-specific protein C13 [Vitis vinifera]
3078	2602	gi|225424272	36.17	7	1	1		17200	PREDICTED: MLP-like protein 34 [Vitis vinifera]
2868	19095	gi|225463253	36.16	2	1	1		61124	PREDICTED: DEAD-box ATP-dependent RNA helicase 5 [Vitis vinifera]
1830	19376	gi|566208632	36.1	4	1	1		30390	hypothetical protein POPTR_0016s04290g [Populus trichocarpa]
2409	19512	gi|225444639	36.04	6	1	1		20529	PREDICTED: 21 kDa protein [Vitis vinifera]
1937	19195	gi|566188857	35.98	7	1	1		14979	hypothetical protein POPTR_0010s02790g [Populus trichocarpa]
2486	19221	gi|225443738	35.95	1	1	1		66499	PREDICTED: uncharacterized protein LOC100241189 [Vitis vinifera]
1300	19995	gi|566208495	35.93	1	1	1		80383	DNA topoisomerase 2 family protein [Populus trichocarpa]
1362	2032	gi|359474652	35.91	2	1	1		50670	PREDICTED: scopoletin glucosyltransferase-like [Vitis vinifera]
2750	19063	gi|566149094	35.9	4	1	1	Carbamidomethylation	26501	hypothetical protein POPTR_0001s15200g [Populus trichocarpa]
2329	2241	gi|225446767	35.89	2	1	1		40475	PREDICTED: uncharacterized oxidoreductase At1g06690 chloroplastic [Vitis vinifera]
1809	2858	gi|731411254	35.84	5	1	1		21305	PREDICTED: 22.0 kDa class IV heat shock protein-like [Vitis vinifera]
3076	19977	gi|225456262	35.79	9	1	1		11952	PREDICTED: mitochondrial pyruvate carrier 1 [Vitis vinifera]
2507	20005	gi|224111560	35.77	2	1	1		96995	hydroxyproline-rich glycoprotein [Populus trichocarpa]

2412	19595	gi|566192632	35.72	3	1	1		35033	nodulin 35 family protein [Populus trichocarpa]
3080	2671	gi|359497879	35.56	13	1	1		12600	PREDICTED: probable aquaporin TIP-type partial [Vitis vinifera]
2872	19334	gi|224067846	35.42	4	1	1		28816	hypothetical protein POPTR_0002s15600g [Populus trichocarpa]
808	3590	gi|731418129	35.23	0	1	1		565777	PREDICTED: auxin transport protein BIG [Vitis vinifera]
3081	2631	gi|225450051	35.21	3	1	1		39579	PREDICTED: cycloartenol-C-24-methyltransferase [Vitis vinifera]
2139	19544	gi|566164408	35.15	2	1	1		45443	RNA-binding protein 45 [Populus trichocarpa]
3079	20009	gi|566201939	35.12	2	1	1		70875	hypothetical protein POPTR_0014s02040g [Populus trichocarpa]
1828	1915	gi|225443496	35.03	1	1	1		81071	PREDICTED: DEAD-box ATP-dependent RNA helicase 21-like [Vitis vinifera]
2014	2527	gi|526117353	34.95	3	1	1		36687	mitochondrial alternative oxidase 2 [Vitis vinifera]
2751	1874	gi|225452328	34.94	5	1	1		44293	PREDICTED: altered inheritance of mitochondria protein 32 [Vitis vinifera]
1913	19001	gi|566150858	34.86	1	1	1		114715	pumilio/Puf RNA-binding domain-containing family protein [Populus trichocarpa]
2962	19435	gi|359486976	34.84	3	1	1		30800	PREDICTED: uncharacterized protein DS12 from 2D-PAGE of leaf chloroplastic isoform X2 [Vitis vinifera]
2448	19527	gi|566204649	34.82	3	1	1		42787	hypothetical protein POPTR_0014s16700g [Populus trichocarpa]
2398	18910	gi|566214115	34.8	2	1	1		50293	DNA repair ATPase-related family protein [Populus trichocarpa]
2447	19442	gi|224094580	34.79	4	1	1		38015	hypothetical protein POPTR_0007s12020g [Populus trichocarpa]

2509	20027	gi|225456703	34.79	11	1	1		17349	PREDICTED: ubiquitin-conjugating enzyme E2 2 [Vitis vinifera]
2508	2793	gi|731369035	34.69	4	1	1		29422	PREDICTED: anthranilate synthase beta subunit 1 chloroplastic-like isoform X2 [Vitis vinifera]
2193	19607	gi|224093148	34.62	5	1	1		29641	Tetrapyrrole-binding family protein [Populus trichocarpa]
2300	19011	gi|566195960	34.51	1	1	1		205867	hypothetical protein POPTR_0011s17070g [Populus trichocarpa]
2247	2210	gi|225435307	34.5	2	1	1		49888	PREDICTED: ruvB-like protein 1 [Vitis vinifera]
2133	19202	gi|224106822	34.5	2	1	1		66805	ABC transporter family protein [Populus trichocarpa]
3085	2659	gi|225442357	34.44	5	1	1		22473	PREDICTED: charged multivesicular body protein 1b [Vitis vinifera]
3083	20020	gi|224107749	34.25	2	1	1		58085	eukaryotic translation initiation factor 4B family protein [Populus trichocarpa]
3082	20017	gi|225449424	34.24	5	1	1		23431	PREDICTED: thylakoid lumenal 15 kDa protein 1 chloroplastic [Vitis vinifera]
2035	19059	gi|566164187	34.22	4	1	1		32911	hypothetical protein POPTR_0004s00590g [Populus trichocarpa]
743	19546	gi|566147155	34.21	2	1	1		62520	hypothetical protein POPTR_0001s04270g [Populus trichocarpa]
2330	6691	gi|225437318	34.12	1	1	1		157286	PREDICTED: translocase of chloroplast 159 chloroplastic [Vitis vinifera]
2445	2120	gi|731389062	34.1	5	1	1		25568	PREDICTED: endo-1 3;1 4-beta-D-glucanase-like [Vitis vinifera]
3084	20043	gi|731393417	34.03	3	1	1		43134	PREDICTED: probable linoleate 9S-lipoxygenase 5 [Vitis vinifera]

2973	20082	gi|566259648	33.84	7	1	1		16066	hypothetical protein POPTR_0026s00220g [Populus trichocarpa]
641	20075	gi|224092174	33.82	4	1	1		36141	hypothetical protein POPTR_0006s24360g [Populus trichocarpa]
2237	2663	gi|359490173	33.75	4	1	1		25366	PREDICTED: clp protease-related protein At4g12060 chloroplastic [Vitis vinifera]
2959	19389	gi|566188774	33.71	2	1	1		72607	hypothetical protein POPTR_0010s02290g [Populus trichocarpa]
2411	7014	gi|225433463	33.69	1	1	1		98952	PREDICTED: beta-galactosidase 9 isoform X1 [Vitis vinifera]
2709	19009	gi|224138022	33.6	2	1	1		84828	alkaline alpha galactosidase family protein [Populus trichocarpa]
1787	2753	gi|225429582	33.54	1	1	1		98913	PREDICTED: aminopeptidase M1 [Vitis vinifera]
2874	19399	gi|224130486	33.54	13	1	1		9171	hypothetical protein POPTR_0014s09080g [Populus trichocarpa]
2613	19194	gi|224102881	33.53	3	1	1	Carbamidomethylation	45777	tRNA synthetase class 1 family protein [Populus trichocarpa]
1535	2681	gi|225455153	33.49	4	1	1		26938	PREDICTED: mitochondrial import inner membrane translocase subunit TIM22-like [Vitis vinifera]
1811	2627	gi|731393556	33.32	4	1	1		29837	PREDICTED: rhodanese-like/PpiC domain-containing protein 12 isoform X2 [Vitis vinifera]
1732	2790	gi|225455278	33.15	18	1	1	Oxidation (M)	15102	PREDICTED: cytochrome b5 isoform E [Vitis vinifera]
2964	19550	gi|731393208	33.06	2	1	1		64874	PREDICTED: uncharacterized protein LOC100253639 [Vitis vinifera]
2675	1731	gi|359489456	33.01	4	1	1		26638	PREDICTED: vesicle-associated protein 1-2 [Vitis vinifera]
1974	20096	gi|224123444	32.99	10	1	1		14627	prefoldin family protein [Populus trichocarpa]

1733	2718	gi|731395801	32.99	5	1	1		21364	PREDICTED: nitrogen regulatory protein P-II homolog isoform X2 [Vitis vinifera]
2376	2149	gi|731382802	32.97	1	1	1		99070	PREDICTED: transportin-1 isoform X1 [Vitis vinifera]
2967	19614	gi|225438035	32.95	4	1	1		26519	PREDICTED: rho GDP-dissociation inhibitor 1 [Vitis vinifera]
2968	19616	gi|566177300	32.87	3	1	1		34068	hypothetical protein POPTR_0006s20660g [Populus trichocarpa]
2132	19200	gi|566163352	32.86	2	1	1		85586	hypothetical protein POPTR_0003s18380g [Populus trichocarpa]
3086	20107	gi|224138170	32.81	11	1	1		7523	hypothetical protein POPTR_0016s06300g [Populus trichocarpa]
2511	20101	gi|566177606	32.76	16	1	1		9314	hypothetical protein POPTR_0006s22550g [Populus trichocarpa]
1940	19267	gi|225458434	32.69	2	1	1		58096	PREDICTED: allene oxide synthase [Vitis vinifera]
2404	2219	gi|731406552	32.67	3	1	1	Carbamidomethylation	50140	PREDICTED: angio-associated migratory cell protein-like isoform X2 [Vitis vinifera]
2015	20117	gi|224129342	32.65	4	1	1		22803	Photosystem I reaction center subunit XI family protein [Populus trichocarpa]
2876	2765	gi|731388932	32.54	1	1	1		117730	PREDICTED: uncharacterized protein LOC100244334 [Vitis vinifera]
2510	20092	gi|731380117	32.53	6	1	1		27501	PREDICTED: thylakoid lumenal protein At1g12250 chloroplastic isoform X3 [Vitis vinifera]
2490	19612	gi|566148044	32.48	3	1	1	Carbamidomethylation	28219	glutathione S-transferase family protein [Populus trichocarpa]
1857	2027	gi|225428747	32.44	3	1	1		33152	PREDICTED: 2-hydroxyisoflavanone dehydratase [Vitis vinifera]

1774	2720	gi|225470916	32.42	2	1	1	Carbamidomethylation	52293	PREDICTED: protein ASPARTIC PROTEASE IN GUARD CELL 2 [Vitis vinifera]
3087	20110	gi|566207660	32.4	7	1	1		21645	cytochrome b5 domain-containing family protein [Populus trichocarpa]
2187	2797	gi|225428420	32.38	9	1	1		12203	PREDICTED: 40S ribosomal protein S25-2 [Vitis vinifera]
3088	2651	gi|225465259	32.36	6	1	1		27590	PREDICTED: NADPH-dependent pterin aldehyde reductase [Vitis vinifera]
2141	20108	gi|566158274	32.3	3	1	1		34050	hypothetical protein POPTR_0002s15960g [Populus trichocarpa]
2378	19525	gi|566170977	32.28	3	1	1		29462	hypothetical protein POPTR_0005s12280g [Populus trichocarpa]
2256	1179	gi|225437618	32.19	4	2	1	Carbamidomethylation	82698	PREDICTED: probable pre-mRNA-splicing factor ATP-dependent RNA helicase [Vitis vinifera]
3089	20121	gi|566199681	32.11	7	1	1		14910	hypothetical protein POPTR_0013s05920g [Populus trichocarpa]
1852	3038	gi|225446032	31.93	4	1	1		19273	PREDICTED: ribulose bisphosphate carboxylase small chain clone 512 [Vitis vinifera]
2402	19256	gi|224072659	31.9	2	1	1		53805	glutathione-disulfide reductase family protein [Populus trichocarpa]
2111	2046	gi|731401073	31.87	2	1	1		75858	PREDICTED: phosphoinositide phospholipase C 2-like [Vitis vinifera]
2239	2782	gi|225459768	31.72	5	1	1		17006	PREDICTED: plastocyanin [Vitis vinifera]
2512	2196	gi|225457367	31.72	7	1	1		15124	PREDICTED: peptidyl-prolyl cis-trans isomerase NIMA-interacting 4 [Vitis vinifera]
2238	20118	gi|224105291	31.69	3	1	1		38331	GIANT CHLOROPLAST 1 family protein [Populus trichocarpa]

2410	19530	gi|224073041	31.66	3	1	1		25942	cysteine proteinase inhibitor [Populus trichocarpa]
2803	19529	gi|566173751	31.65	3	1	1		39313	farnesyl-pyrophosphate synthetase fps2 family protein [Populus trichocarpa]
2460	3297	gi|731381391	31.51	1	1	1		60014	PREDICTED: replication factor C subunit 3-like [Vitis vinifera]
1972	2785	gi|731408402	31.48	3	1	1		29415	PREDICTED: (DL)-glycerol-3-phosphatase 2 [Vitis vinifera]
3091	20137	gi|224089182	31.28	5	1	1	Carbamidomethylation	17094	zinc finger family protein [Populus trichocarpa]
3090	20133	gi|566153514	31.22	11	1	1		13590	hypothetical protein POPTR_0001s38670g partial [Populus trichocarpa]
2970	19629	gi|566157027	31.19	3	1	1		34854	hypothetical protein POPTR_0002s09060g [Populus trichocarpa]
3092	20145	gi|671743286	31.09	7	1	1		19547	NADH-plastoquinone oxidoreductase subunit I (chloroplast) [Camellia petelotii]
2016	20158	gi|566158427	31.03	7	1	1		17387	calmodulin 2 family protein [Populus trichocarpa]
2241	2692	gi|359479028	31.03	3	1	1		40721	PREDICTED: probable trans-2-enoyl-CoA reductase mitochondrial [Vitis vinifera]
2674	1843	gi|359474650	31	3	1	1	Carbamidomethylation	55226	PREDICTED: scopoletin glucosyltransferase [Vitis vinifera]
2401	19171	gi|225445124	30.75	3	1	1		67861	PREDICTED: SNW/SKI-interacting protein [Vitis vinifera]
2106	18943	gi|224140195	30.45	5	1	1		30031	29 kDa ribonucleoprotein [Populus trichocarpa]
2969	2218	gi|731422566	30.42	4	1	1		34464	PREDICTED: translocase of chloroplast 34 chloroplastic [Vitis vinifera]
1734	2879	gi|359495735	30.39	4	1	1		27933	PREDICTED: uncharacterized protein LOC100855146 [Vitis vinifera]
2513	20159	gi|731382351	30.14	2	1	1		43783	PREDICTED: uncharacterized protein LOC100245856 [Vitis vinifera]

2199	20282	gi|224109066	29.87	1	1	1		61143	hypothetical protein POPTR_0010s17900g [Populus trichocarpa]
2458	2721	gi|225443266	29.84	3	1	1		39304	PREDICTED: mitogen-activated protein kinase kinase 5 [Vitis vinifera]
3095	20200	gi|224106319	29.79	3	1	1	Carbamidomethylation	59309	hypothetical protein POPTR_0009s04630g [Populus trichocarpa]
2194	3087	gi|731391278	29.67	2	1	1	Carbamidomethylation	56898	PREDICTED: eukaryotic translation initiation factor 2A [Vitis vinifera]
2878	2780	gi|731389634	29.65	1	1	1		77547	PREDICTED: LOW QUALITY PROTEIN: 1-aminocyclopropane-1-carboxylate oxidase homolog 1 [Vitis vinifera]
2444	4252	gi|359488906	29.6	1	1	1		135812	PREDICTED: ABC transporter B family member 19 [Vitis vinifera]
3093	20179	gi|225445170	29.57	5	1	1		24265	PREDICTED: DNA-directed RNA polymerases II and IV subunit 5A [Vitis vinifera]
3096	2808	gi|731384926	29.55	6	1	1		15933	PREDICTED: 60S ribosomal protein L28-1 isoform X3 [Vitis vinifera]
3097	2748	gi|526117331	29.46	7	1	1		14247	thioredoxin h [Vitis vinifera]
2977	2730	gi|225439546	29.39	1	1	1		91491	PREDICTED: probably inactive leucine-rich repeat receptor-like protein kinase IMK2 [Vitis vinifera]
2108	19006	gi|731424546	29.38	1	1	1		95956	PREDICTED: uncharacterized protein LOC100252015 isoform X4 [Vitis vinifera]
3094	20198	gi|225465945	29.29	1	1	1		100867	PREDICTED: golgin candidate 6 isoform X1 [Vitis vinifera]
2972	19644	gi|225442791	29.2	1	1	1	Carbamidomethylation	68124	PREDICTED: plastidic ATP/ADP-transporter [Vitis vinifera]
2242	20217	gi|225445840	29.2	5	1	1		38575	PREDICTED: homoserine kinase [Vitis vinifera]

2805	19632	gi|731380670	29.15	3	1	1		31325	PREDICTED: uncharacterized protein At1g32220 chloroplastic isoform X2 [Vitis vinifera]
3098	20213	gi|224114830	29.14	2	1	1		47731	putative 4-methyl-5(b-hydroxyethyl)-thiazole monophosphate family protein [Populus trichocarpa]
2794	19138	gi|566161892	28.64	2	1	1	Carbamidomethylation	62833	hypothetical protein POPTR_0003s09970g [Populus trichocarpa]
2676	19213	gi|566185272	28.62	2	1	1		58606	hypothetical protein POPTR_0008s22050g partial [Populus trichocarpa]
1891	19028	gi|566198361	28.61	1	1	1		90791	hypothetical protein POPTR_0012s13720g [Populus trichocarpa]
3100	2792	gi|225429450	28.5	6	1	1		22528	PREDICTED: PITH domain-containing protein 1 [Vitis vinifera]
2877	2261	gi|359487708	28.48	3	1	1		43172	PREDICTED: flap endonuclease 1 isoform X2 [Vitis vinifera]
2198	5258	gi|359489366	28.34	2	1	1		42909	PREDICTED: fasciclin-like arabinogalactan protein 10 [Vitis vinifera]
2196	2813	gi|225427973	28.26	3	1	1		19853	PREDICTED: glycine-rich protein 2-like [Vitis vinifera]
2980	2752	gi|359476953	28.22	3	1	1		34854	PREDICTED: pre-mRNA-splicing factor ISY1 homolog [Vitis vinifera]
1735	2815	gi|225445984	28.18	7	1	1		15131	PREDICTED: glutaredoxin-C4 [Vitis vinifera]
2373	2682	gi|225429325	28.17	5	1	1		24120	PREDICTED: thioredoxin domain-containing protein 9 homolog [Vitis vinifera]
2514	2759	gi|225464240	28.02	5	1	1		20829	PREDICTED: uncharacterized protein At4g13200 chloroplastic [Vitis vinifera]
3099	20226	gi|566161648	27.96	5	1	1		17801	glycine cleavage system protein H precursor [Populus trichocarpa]

2405	1929	gi|731421066	27.94	2	1	1		33932	PREDICTED: probable fructokinase-7 isoform X2 [Vitis vinifera]
2612	19034	gi|566188113	27.94	1	1	1		113294	adaptin family protein [Populus trichocarpa]
3101	20248	gi|224121470	27.88	5	1	1		32058	3-PHOSPHOSERINE PHOSPHATASE family protein [Populus trichocarpa]
2979	4942	gi|731401567	27.88	4	1	1		45798	PREDICTED: 4-coumarate--CoA ligase-like 7 [Vitis vinifera]
2885	20256	gi|359497044	27.73	2	1	1		58077	PREDICTED: lysosomal Pro-X carboxypeptidase [Vitis vinifera]
2974	20086	gi|224096333	27.65	2	1	1		57960	hypothetical protein POPTR_0007s06670g [Populus trichocarpa]
1853	3107	gi|225450219	27.59	3	1	1		26900	PREDICTED: 30S ribosomal protein 2 chloroplastic [Vitis vinifera]
1854	20280	gi|566186893	27.18	2	1	1		44046	hypothetical protein POPTR_0009s08040g [Populus trichocarpa]
1945	2713	gi|359481856	27.09	6	1	1		21178	PREDICTED: dirigent protein 22-like [Vitis vinifera]
3102	20265	gi|731387184	27.08	2	1	1		61791	PREDICTED: U1 small nuclear ribonucleoprotein 70 kDa [Vitis vinifera]
2457	20178	gi|224135493	26.98	1	1	1		66080	transducin family protein [Populus trichocarpa]
3103	20302	gi|566170882	26.68	4	1	1		21960	HISTONE H1-3 family protein [Populus trichocarpa]
2806	7631	gi|731402564	26.65	1	1	1		100977	PREDICTED: katanin p60 ATPase-containing subunit A1 [Vitis vinifera]
2332	2147	gi|225451754	26.45	3	1	1	Carbamidomethylation	46712	PREDICTED: eukaryotic translation initiation factor 3 subunit M [Vitis vinifera]
2978	2993	gi|731425464	26.29	1	1	1		71773	PREDICTED: RNA polymerase-associated protein LEO1 isoform X2 [Vitis vinifera]

2804	19536	gi|566188433	25.94	2	1	1		73876	hypothetical protein POPTR_0009s171801g partial [Populus trichocarpa]
2243	3506	gi|225451561	25.79	5	1	1		20516	PREDICTED: translocon-associated protein subunit beta [Vitis vinifera]
2976	20188	gi|566204034	25.63	2	1	1		54966	hypothetical protein POPTR_0014s13020g [Populus trichocarpa]
1480	19130	gi|566211364	25.61	2	1	1		45881	hypothetical protein POPTR_0017s04550g [Populus trichocarpa]
2459	20364	gi|224132674	25.61	2	1	1		40747	hypothetical protein POPTR_0015s01030g [Populus trichocarpa]
2094	19363	gi|225460394	25.47	1	1	1		67755	PREDICTED: patellin-3 [Vitis vinifera]
3104	20346	gi|225435189	24.92	7	1	1		14801	PREDICTED: nuclear transcription factor Y subunit B-10-like [Vitis vinifera]
2981	20309	gi|731422386	24.68	1	1	1		94993	PREDICTED: WEB family protein At5g16730 chloroplastic-like [Vitis vinifera]
2200	2293	gi|731422266	24.54	2	1	1		60025	PREDICTED: cytochrome P450 89A2-like [Vitis vinifera]
2143	20252	gi|566215395	24.5	6	1	1		21500	hypothetical protein POPTR_0018s11910g [Populus trichocarpa]
3108	20416	gi|566200837	24.44	8	1	1		13900	ribosomal protein L7Ae/L30e/S12e/Gadd45 [Populus trichocarpa]
3106	20379	gi|566195508	24.44	2	1	1		46302	arginine/serine-rich family protein [Populus trichocarpa]
3107	20401	gi|731370548	24.19	2	1	1		50905	PREDICTED: transcription factor TGA2-like [Vitis vinifera]
2520	20467	gi|566166948	24.07	1	1	1		116430	hypothetical protein POPTR_0004s16940g [Populus trichocarpa]

2519	20458	gi|566153056	23.93	2	1	1		58525	glucose-6-phosphate 1-dehydrogenase family protein [Populus trichocarpa]
1543	2034	gi|731399524	23.77	2	1	1		58565	PREDICTED: 70 kDa peptidyl-prolyl isomerase-like [Vitis vinifera]
2521	2819	gi|225438426	23.69	2	1	1		80118	PREDICTED: monosaccharide-sensing protein 2 [Vitis vinifera]
2678	5138	gi|731404421	23.53	3	1	1	Carbamidomethylation	51194	PREDICTED: monothiol glutaredoxin-S17 isoform X2 [Vitis vinifera]
2983	5864	gi|731395998	23.5	1	1	1		112155	PREDICTED: poly [ADP-ribose] polymerase 1 [Vitis vinifera]
2880	4772	gi|225427419	23.42	2	1	1		46142	PREDICTED: COBW domain-containing protein 1 [Vitis vinifera]
1593	3306	gi|359490455	23.09	1	1	1		52229	PREDICTED: UDP-glycosyltransferase 88A1-like [Vitis vinifera]
2522	20493	gi|566191071	23.03	3	1	1		44571	hypothetical protein POPTR_0010s16010g [Populus trichocarpa]
2886	20278	gi|731393636	22.98	1	1	1		102554	PREDICTED: replication factor C subunit 1 [Vitis vinifera]
2120	21249	gi|731414681	22.71	0	1	1	Carbamidomethylation	153010	PREDICTED: putative disease resistance RPP13-like protein 1 isoform X2 [Vitis vinifera]
2982	20423	gi|224080325	22.4	3	1	1		41179	ERECTA-LIKE 4 family protein [Populus trichocarpa]
2758	20465	gi|566161176	22.31	4	1	1		21945	germin-like family protein [Populus trichocarpa]
2462	20509	gi|225464926	22.28	2	1	1		34781	PREDICTED: N-carbamoylputrescine amidase [Vitis vinifera]
2523	3109	gi|225467027	22.18	6	1	1		16011	PREDICTED: uncharacterized protein LOC100262366 [Vitis vinifera]
2889	20422	gi|224082496	22.16	3	1	1		38736	peroxidase precursor family protein [Populus trichocarpa]

1712	20694	gi|224079413	22.08	2	1	1		36896	hypothetical protein POPTR_0004s09110g [Populus trichocarpa]
3114	20632	gi|566174372	21.93	8	1	1	Carbamidomethylation	12415	hypothetical protein POPTR_0006s04020g [Populus trichocarpa]
2989	20605	gi|225467859	21.87	3	1	1	Carbamidomethylation	33928	PREDICTED: chlorophyllase-1 [Vitis vinifera]
3111	20551	gi|731427546	21.86	6	1	1		33441	PREDICTED: cinnamoyl-CoA reductase 2 isoform X2 [Vitis vinifera]
1834	20644	gi|566185390	21.66	5	1	1		32450	hypothetical protein POPTR_0008s22600g [Populus trichocarpa]

Table S5 The identified proteins in HY2

Protein Group	Protein ID	Accession	-10lgP	Coverage (%)	#Peptides	#Unique	PTM	Avg. Mass	Description
8	6	gi|359495606	305.96	52	48	3	Carbamidomethylation; Oxidation (M)	80006	PREDICTED: heat shock cognate protein 80 [Vitis vinifera]
9	7	gi|225462013	303.99	51	47	3	Carbamidomethylation	80849	PREDICTED: heat shock cognate protein 80-like [Vitis vinifera]
22	4	gi|225470745	294.51	74	38	1	Carbamidomethylation; Oxidation (M)	50553	PREDICTED: tubulin beta-1 chain [Vitis vinifera]
30	16395	gi|566195847	292.87	65	37	1	Carbamidomethylation; Oxidation (M)	50164	tubulin beta chain family protein [Populus trichocarpa]
13	8	gi|225426164	284.87	41	37	2	Carbamidomethylation	80852	PREDICTED: heat shock protein 83 [Vitis vinifera]
12	16417	gi|224056837	284.19	40	38	2	Carbamidomethylation; Oxidation (M)	80026	hypothetical protein POPTR_0001s47020g [Populus trichocarpa]
18	5	gi|359486799	283.62	54	37	2	Carbamidomethylation; Oxidation (M)	71171	PREDICTED: heat shock cognate 70 kDa protein 2 [Vitis vinifera]
40	17	gi|225434722	280.01	65	35	2	Carbamidomethylation	50275	PREDICTED: tubulin beta-2 chain [Vitis vinifera]
21	16410	gi|566191989	278.18	54	36	1	Carbamidomethylation; Oxidation (M)	71140	heat shock protein 70 cognate [Populus trichocarpa]
1	16404	gi|224055984	276.61	72	27	3	Carbamidomethylation	41696	actin family protein [Populus trichocarpa]
2	12	gi|731410014	274.7	70	26	1	Carbamidomethylation	41593	PREDICTED: actin-101 [Vitis vinifera]
68	20	gi|225440045	274.53	54	43	2	Carbamidomethylation; Oxidation (M)	89589	PREDICTED: cell division cycle protein 48 homolog [Vitis vinifera]
10	16386	gi|669100005	272.97	68	36	36	Carbamidomethylation	52635	ribulose bisphosphate carboxylase large chain (chloroplast) [Camellia crapnelliana]
56	18	gi|225452767	265.15	66	32	3	Carbamidomethylation; Oxidation (M)	49622	PREDICTED: tubulin alpha chain [Vitis vinifera]
3	16442	gi|224056705	261.86	58	24	1	Carbamidomethylation	41636	actin family protein [Populus trichocarpa]
35	16	gi|225440324	261.08	46	33	1	Carbamidomethylation; Oxidation (M)	71066	PREDICTED: heat shock cognate 70 kDa protein 2 [Vitis vinifera]
6	16451	gi|224112777	261.01	63	23	1	Carbamidomethylation	41717	actin family protein [Populus trichocarpa]

70	36	gi|225426230	256.33	35	31	1	Carbamidomethylation	73581	PREDICTED: luminal-binding protein 5 [Vitis vinifera]
58	19	gi|731439963	255.45	66	30	1	Carbamidomethylation; Oxidation (M)	49600	PREDICTED: tubulin alpha chain-like [Vitis vinifera]
78	16444	gi|224094244	254.79	34	33	4	Carbamidomethylation	93953	elongation factor 2 family protein [Populus trichocarpa]
158	38	gi|225448483	251.53	24	40	1	Carbamidomethylation	192967	PREDICTED: clathrin heavy chain 1 [Vitis vinifera]
105	16454	gi|224133614	249.57	42	32	1	Carbamidomethylation; Oxidation (M)	90417	hypothetical protein POPTR_0015s09220g [Populus trichocarpa]
135	16490	gi|566191652	248.39	25	45	2	Carbamidomethylation	193360	clathrin heavy chain family protein [Populus trichocarpa]
67	16430	gi|566232573	244.12	33	26	1	Carbamidomethylation	85012	hypothetical protein POPTR_0019s05430g [Populus trichocarpa]
7	16499	gi|224117708	243.92	55	20	1	Carbamidomethylation	40618	actin family protein [Populus trichocarpa]
25	34	gi|225432155	240.08	62	22	1	Carbamidomethylation; Oxidation (M)	43114	PREDICTED: S-adenosylmethionine synthase 2 [Vitis vinifera]
62	16455	gi|566188155	238.91	31	26	3	Carbamidomethylation	89855	hypothetical protein POPTR_0009s15490g [Populus trichocarpa]
71	46	gi|225445820	238.25	30	22	1	Carbamidomethylation; Oxidation (M)	71402	PREDICTED: heat shock 70 kDa protein [Vitis vinifera]
28	33	gi|225437708	238.19	63	20	1	Carbamidomethylation; Oxidation (M)	42793	PREDICTED: S-adenosylmethionine synthase 5 [Vitis vinifera]
92	39	gi|225462164	236.74	35	34	6	Carbamidomethylation	93979	PREDICTED: elongation factor 2 [Vitis vinifera]
50	58	gi|225449120	235.88	41	24	2	Carbamidomethylation; Oxidation (M)	49376	PREDICTED: elongation factor 1-alpha [Vitis vinifera]
86	70	gi|359490716	235.08	30	27	1	Carbamidomethylation	73489	PREDICTED: luminal-binding protein 5-like isoform X2 [Vitis vinifera]
64	26	gi|225456079	233.52	48	22	7	Carbamidomethylation	59180	PREDICTED: ATP synthase subunit beta mitochondrial-like [Vitis vinifera]

144	17009	gi|224065861	232.61	60	20	7	Carbamidomethylation	18243	Peptidyl-prolyl cis-trans isomerase family protein [Populus trichocarpa]
54	31	gi|225439223	231.05	35	28	4	Carbamidomethylation; Oxidation (M)	84991	PREDICTED: 5-methyltetrahydropteroyltriglutamate--homocysteine methyltransferase [Vitis vinifera]
127	41	gi|225456004	230.19	36	24	1	Carbamidomethylation	75635	PREDICTED: stromal 70 kDa heat shock-related protein chloroplastic [Vitis vinifera]
118	16476	gi|566212215	230.07	47	21	2	Carbamidomethylation; Oxidation (M)	53198	adenosylhomocysteinase family protein [Populus trichocarpa]
82	44	gi|359473642	229.07	30	25	4	Carbamidomethylation	90465	PREDICTED: heat shock protein 90-1 [Vitis vinifera]
41	64	gi|731416007	227.93	54	20	1	Carbamidomethylation; Oxidation (M)	42931	PREDICTED: S-adenosylmethionine synthase 4 [Vitis vinifera]
111	16510	gi|566146992	227.82	28	19	2	Carbamidomethylation	71904	heat shock protein 70 cognate [Populus trichocarpa]
104	16500	gi|224136806	227.25	43	20	5	Carbamidomethylation	47928	hypothetical protein POPTR_0015s14380g [Populus trichocarpa]
66	16494	gi|566146990	223.09	28	21	1	Carbamidomethylation; Oxidation (M)	71882	heat shock protein 70 cognate [Populus trichocarpa]
166	16503	gi|224073124	221.5	37	21	3	Carbamidomethylation	64544	chaperonin precursor family protein [Populus trichocarpa]
59	88	gi|225426414	221.07	42	24	1	Carbamidomethylation	50055	PREDICTED: tubulin beta chain [Vitis vinifera]
113	42	gi|731393262	220.81	38	26	5	Carbamidomethylation	64608	PREDICTED: ruBisCO large subunit-binding protein subunit beta chloroplastic [Vitis vinifera]
134	63	gi|225433506	220.76	44	21	2	Carbamidomethylation	53181	PREDICTED: adenosylhomocysteinase [Vitis vinifera]
114	16475	gi|224099437	220.54	38	16	1	Carbamidomethylation	59970	H+-transporting two-sector ATPase family protein [Populus trichocarpa]
189	16517	gi|224097420	219.47	42	22	3	Carbamidomethylation	68619	Vacuolar ATP synthase catalytic subunit A family protein [Populus trichocarpa]

155	78	gi|359478916	218.87	40	15	4	Carbamidomethylation; Oxidation (M)	48674	PREDICTED: ribulose bisphosphate carboxylase/oxygenase activase chloroplastic isoform X2 [Vitis vinifera]
125	16508	gi|224144808	216.7	51	19	2	Carbamidomethylation; Oxidation (M)	49595	Tubulin alpha-1 chain family protein [Populus trichocarpa]
128	16602	gi|224052904	216.61	44	17	1	Carbamidomethylation; Oxidation (M)	49819	alpha-tubulin 2 family protein [Populus trichocarpa]
168	66	gi|225460961	215.02	23	19	2	Carbamidomethylation; Oxidation (M)	110056	PREDICTED: aconitate hydratase cytoplasmic [Vitis vinifera]
115	16464	gi|566199292	214.18	53	18	1	Carbamidomethylation	42361	hypothetical protein POPTR_0013s03790g [Populus trichocarpa]
136	138	gi|731423140	213.98	27	16	1	Carbamidomethylation	55855	PREDICTED: enolase [Vitis vinifera]
100	16511	gi|566196410	213.78	28	22	3		73406	BiP isoform A family protein [Populus trichocarpa]
76	86	gi|225439902	213.66	42	20	1	Carbamidomethylation; Oxidation (M)	49336	PREDICTED: elongation factor 1-alpha [Vitis vinifera]
123	67	gi|224365668	212.74	40	23	20	Carbamidomethylation	55143	ATPase subunit 1 (mitochondrion) [Vitis vinifera]
206	95	gi|731379116	211.81	24	19	7	Carbamidomethylation	100128	PREDICTED: puromycin-sensitive aminopeptidase isoform X2 [Vitis vinifera]
216	68	gi|225446609	211.5	22	23	2	Carbamidomethylation	110322	PREDICTED: phosphoenolpyruvate carboxylase housekeeping isozyme [Vitis vinifera]
264	97	gi|225441955	211.38	53	20	3	Carbamidomethylation	47682	PREDICTED: 26S protease regulatory subunit 7 [Vitis vinifera]
209	16519	gi|224105937	211	17	31	1	Carbamidomethylation	191356	hypothetical protein POPTR_0009s07740g [Populus trichocarpa]
152	294	gi|225437549	210.63	24	14	1	Carbamidomethylation	48111	PREDICTED: calreticulin [Vitis vinifera]
244	82	gi|225443399	210.33	38	20	1	Carbamidomethylation	68723	PREDICTED: V-type proton ATPase catalytic subunit A [Vitis vinifera]
141	16518	gi|566204581	210.19	24	18	4	Carbamidomethylation	82947	hypothetical protein POPTR_0014s16280g [Populus trichocarpa]

52	112	gi|731394274	209.72	52	18	1	Carbamidomethylation	42738	PREDICTED: S-adenosylmethionine synthase 1 isoform X2 [Vitis vinifera]
112	16496	gi|224085900	208.86	23	23	6		94047	hypothetical protein POPTR_0005s26260g [Populus trichocarpa]
218	81	gi|225461660	208.7	23	25	3	Carbamidomethylation	109823	PREDICTED: phosphoenolpyruvate carboxylase housekeeping isozyme [Vitis vinifera]
131	16498	gi|224142804	208.4	43	19	2	Carbamidomethylation	46871	translation initiation factor eIF-4A family protein [Populus trichocarpa]
238	61	gi|731426977	207.44	27	24	6	Carbamidomethylation	102347	PREDICTED: ATP-dependent Clp protease ATP-binding subunit clpA homolog CD4B chloroplastic [Vitis vinifera]
284	16513	gi|566214281	206.89	52	18	1	Carbamidomethylation	47960	hypothetical protein POPTR_0018s04900g [Populus trichocarpa]
84	252	gi|526117553	205.74	35	11	1	Carbamidomethylation	39322	glutamine synthetase cytosolic isozyme 2 [Vitis vinifera]
160	166	gi|225433510	204.61	32	14	4	Carbamidomethylation	51906	PREDICTED: serine hydroxymethyltransferase 4 [Vitis vinifera]
192	92	gi|359479362	204.53	34	19	4	Carbamidomethylation	61984	PREDICTED: ruBisCO large subunit-binding protein subunit alpha [Vitis vinifera]
243	16468	gi|542688129	204.23	53	18	17	Carbamidomethylation	53694	ATP synthase CF1 beta subunit (chloroplast) [Camellia taliensis]
175	116	gi|731410136	204.17	20	18	4	Carbamidomethylation	95223	PREDICTED: heat shock protein 83 isoform X3 [Vitis vinifera]
298	141	gi|731419756	204.11	40	17	4	Carbamidomethylation	52962	PREDICTED: UDP-glucose 6-dehydrogenase 1 [Vitis vinifera]
109	94	gi|225464999	203.96	41	16	4		42422	PREDICTED: phosphoglycerate kinase cytosolic [Vitis vinifera]
174	128	gi|225430776	203.18	31	15	7	Carbamidomethylation	73659	PREDICTED: succinate dehydrogenase [ubiquinone] flavoprotein subunit mitochondrial [Vitis vinifera]
198	72	gi|225456880	202.41	34	17	5	Carbamidomethylation	52692	PREDICTED: elongation factor TuB chloroplastic [Vitis vinifera]

173	85	gi|225460328	201.12	26	19	1	Carbamidomethylation	74178	PREDICTED: stromal 70 kDa heat shock-related protein chloroplastic-like [Vitis vinifera]
162	74	gi|731393844	200.76	22	21	4	Carbamidomethylation	101855	PREDICTED: protein argonaute 4 [Vitis vinifera]
251	158	gi|731388277	200.56	34	15	3	Carbamidomethylation	58138	PREDICTED: importin subunit alpha-1 isoform X1 [Vitis vinifera]
272	16615	gi|224078391	200.39	38	14	2	Carbamidomethylation	54253	vacuolar ATP synthase subunit B family protein [Populus trichocarpa]
233	89	gi|731402464	200.28	22	16	5	Carbamidomethylation	93319	PREDICTED: heat shock 70 kDa protein 14-like [Vitis vinifera]
241	16563	gi|566206349	199.41	46	17	1	Carbamidomethylation	40931	REVERSIBLY GLYCOSYLATED POLYPEPTIDE 3 family protein [Populus trichocarpa]
368	111	gi|225432252	199.34	46	15	1	Carbamidomethylation	47374	PREDICTED: 26S protease regulatory subunit 6A homolog [Vitis vinifera]
65	16520	gi|224109716	199.06	33	18	1	Carbamidomethylation	49434	hypothetical protein POPTR_0010s22620g [Populus trichocarpa]
225	139	gi|225468576	199.05	22	17	3	Carbamidomethylation; Oxidation (M)	98187	PREDICTED: aconitate hydratase 1 [Vitis vinifera]
53	16553	gi|224133862	198.68	41	15	1	Carbamidomethylation	36770	glyceraldehyde-3-phosphate dehydrogenase family protein [Populus trichocarpa]
473	16658	gi|224125162	198.54	48	14	1	Carbamidomethylation	47544	26S proteasome AAA-ATPase subunit family protein [Populus trichocarpa]
148	87	gi|731407424	198.47	20	17	1	Carbamidomethylation	91983	PREDICTED: uncharacterized protein LOC100244028 [Vitis vinifera]
234	16672	gi|566203657	197.88	22	11	6	Carbamidomethylation	56866	hypothetical protein POPTR_0014s10800g [Populus trichocarpa]
121	145	gi|225464995	197.54	33	15	2		50083	PREDICTED: phosphoglycerate kinase chloroplastic [Vitis vinifera]
194	130	gi|731402352	197.38	29	19	4	Carbamidomethylation	65010	PREDICTED: ruBisCO large subunit-binding protein subunit beta chloroplastic [Vitis vinifera]

215	115	gi|225447278	197.36	19	16	1	Carbamidomethylation	107431	PREDICTED: aconitate hydratase cytoplasmic [Vitis vinifera]
129	16607	gi|224109060	196.42	37	15	2		50212	PHOSPHOGLYCERATE KINASE 1 family protein [Populus trichocarpa]
149	83	gi|225430261	195.59	45	18	2	Carbamidomethylation	48395	PREDICTED: DEAD-box ATP-dependent RNA helicase 56 [Vitis vinifera]
119	16541	gi|566192207	195.14	38	19	1	Carbamidomethylation	49434	hypothetical protein POPTR_0010s22560g [Populus trichocarpa]
193	16612	gi|566182577	195.11	32	12	4	Carbamidomethylation; Oxidation (M)	48316	hypothetical protein POPTR_0008s05870g [Populus trichocarpa]
85	160	gi|359491599	194.8	47	16	2	Carbamidomethylation	36735	PREDICTED: glyceraldehyde-3-phosphate dehydrogenase cytosolic [Vitis vinifera]
307	345	gi|731427869	193.68	35	13	1	Carbamidomethylation	54279	PREDICTED: V-type proton ATPase subunit B 2 isoform X2 [Vitis vinifera]
207	16528	gi|224104681	193.14	30	20	3		61991	RUBISCO SUBUNIT BINDING-protein ALPHA SUBUNIT [Populus trichocarpa]
101	102	gi|731393868	192.96	21	17	2	Carbamidomethylation	89385	PREDICTED: 5-methyltetrahydropteroyltriglutamate--homocysteine methyltransferase 2 [Vitis vinifera]
342	16799	gi|224097588	192.94	29	13	1	Carbamidomethylation	46583	DnaJ family protein [Populus trichocarpa]
147	203	gi|526117485	192.6	34	11	9	Carbamidomethylation	40813	naringenin 2-oxoglutarate 3-dioxygenase [Vitis vinifera]
416	16685	gi|224074966	192.46	17	13	1	Carbamidomethylation	106321	peptidase M1 family protein [Populus trichocarpa]
288	16584	gi|224136316	191.8	21	17	1	Carbamidomethylation	103518	ATP-dependent clp protease ATP-binding subunit clpA family protein [Populus trichocarpa]
182	16575	gi|224063263	190.95	36	19	1	Carbamidomethylation	58692	2-dehydro-3-deoxyphosphoheptonate aldolase family protein [Populus trichocarpa]

387	154	gi|225430151	190.84	50	16	3	Carbamidomethylation	47221	PREDICTED: 26S protease regulatory subunit 8 homolog A [Vitis vinifera]
235	16531	gi|566170554	190.79	28	12	1	Carbamidomethylation	56700	hypothetical protein POPTR_0005s10340g [Populus trichocarpa]
183	16787	gi|224102193	190.73	36	13	3	Carbamidomethylation; Oxidation (M)	35716	cytosolic malate dehydrogenase family protein [Populus trichocarpa]
191	16609	gi|224053971	190.31	33	15	2	Carbamidomethylation	52743	hypothetical protein POPTR_0001s08770g [Populus trichocarpa]
133	100	gi|225457939	190.3	22	21	4	Carbamidomethylation	93220	PREDICTED: endoplasmin homolog [Vitis vinifera]
176	122	gi|731430736	189.94	34	15	1	Carbamidomethylation	44590	PREDICTED: 60S ribosomal protein L3 [Vitis vinifera]
475	107	gi|225452950	189.87	22	17	12		109834	PREDICTED: chaperone protein ClpB3 chloroplastic [Vitis vinifera]
201	16506	gi|224065685	188.63	32	15	1	Carbamidomethylation	56639	catalase family protein [Populus trichocarpa]
385	206	gi|731385022	188.57	17	13	7	Carbamidomethylation	114759	PREDICTED: glycine dehydrogenase (decarboxylating) mitochondrial [Vitis vinifera]
210	16678	gi|566192863	188.41	33	13	3	Carbamidomethylation	57068	hypothetical protein POPTR_0010s26150g [Populus trichocarpa]
159	16526	gi|224063766	188.37	23	15	3	Carbamidomethylation	80716	transketolase family protein [Populus trichocarpa]
380	137	gi|526117707	188.23	43	15	8	Carbamidomethylation	53695	uncharacterized protein LOC100261274 [Vitis vinifera]
186	16572	gi|566204141	188.07	39	16	4	Carbamidomethylation	42935	hypothetical protein POPTR_0014s13660g [Populus trichocarpa]
442	16579	gi|224133400	187.95	13	14	1	Carbamidomethylation	136521	coatomer alpha subunit-like family protein [Populus trichocarpa]
187	16604	gi|566150406	187.94	18	17	1	Carbamidomethylation	101827	Argonaute 4 family protein [Populus trichocarpa]
130	16585	gi|224099261	187.73	21	21	3	Carbamidomethylation	90156	heat shock family protein [Populus trichocarpa]

278	93	gi|359496771	187.48	28	15	3		73271	PREDICTED: heat shock 70 kDa protein mitochondrial-like [Vitis vinifera]
293	123	gi|731391766	187.47	22	21	3	Carbamidomethylation	127035	PREDICTED: ubiquitin-activating enzyme E1 1 [Vitis vinifera]
200	146	gi|731418890	187.36	32	13	1	Carbamidomethylation; Oxidation (M)	52944	PREDICTED: elongation factor Tu chloroplastic-like [Vitis vinifera]
277	16537	gi|566148864	187.16	31	15	1		60991	Chaperonin CPN60-2 family protein [Populus trichocarpa]
161	110	gi|359490179	186.94	23	15	3	Carbamidomethylation	78856	PREDICTED: transketolase chloroplastic [Vitis vinifera]
418	136	gi|225456274	186.93	31	15	5	Carbamidomethylation	55180	PREDICTED: argininosuccinate synthase chloroplastic [Vitis vinifera]
181	127	gi|731411443	186.61	31	14	1	Carbamidomethylation	44506	PREDICTED: 60S ribosomal protein L3-2 isoform X1 [Vitis vinifera]
103	16693	gi|566209405	186.43	33	13	1	Carbamidomethylation	44445	hypothetical protein POPTR_0016s08780g [Populus trichocarpa]
376	248	gi|225433375	185.65	27	13	2	Carbamidomethylation	61370	PREDICTED: chaperonin CPN60-2 mitochondrial [Vitis vinifera]
252	132	gi|731428778	185.56	31	15	2	Carbamidomethylation	56891	PREDICTED: serine hydroxymethyltransferase mitochondrial [Vitis vinifera]
421	444	gi|225437777	185.52	17	9	4	Carbamidomethylation	58724	PREDICTED: putative proline--tRNA ligase C19C7.06 [Vitis vinifera]
463	156	gi|225441236	185.5	11	13	4	Carbamidomethylation	177678	PREDICTED: ferredoxin-dependent glutamate synthase chloroplastic [Vitis vinifera]
126	143	gi|731415862	185.48	38	16	6	Carbamidomethylation	49362	PREDICTED: calreticulin [Vitis vinifera]
248	101	gi|225429228	185.04	23	13	2		72770	PREDICTED: heat shock 70 kDa protein mitochondrial [Vitis vinifera]
212	16545	gi|566168009	184.99	25	17	1		62041	RUBISCO SUBUNIT BINDING-protein ALPHA SUBUNIT [Populus trichocarpa]

265	187	gi|225457674	184.94	24	13	4	Carbamidomethylation	67344	PREDICTED: pyrophosphate--fructose 6-phosphate 1-phosphotransferase subunit alpha [Vitis vinifera]
464	172	gi|225442805	184.76	44	14	1	Carbamidomethylation	47100	PREDICTED: 26S protease regulatory subunit 8 homolog A [Vitis vinifera]
321	109	gi|731424802	184.3	17	13	4	Carbamidomethylation	101244	PREDICTED: heat shock protein 101 isoform X1 [Vitis vinifera]
405	16771	gi|566148111	184	17	13	1	Carbamidomethylation	103436	peptidase M1 family protein [Populus trichocarpa]
319	16826	gi|224094759	183.95	22	9	1	Carbamidomethylation	69860	Succinate dehydrogenase flavoprotein subunit [Populus trichocarpa]
333	16639	gi|224086120	183.11	22	11	1	Carbamidomethylation	67209	pyrophosphate-dependent phosphofructokinase alpha subunit family protein [Populus trichocarpa]
394	200	gi|225427768	183.03	35	12	4	Carbamidomethylation	42948	PREDICTED: fructose-bisphosphate aldolase 1 chloroplastic [Vitis vinifera]
282	17072	gi|224114557	182.62	20	8	2	Carbamidomethylation	35588	Malate dehydrogenase family protein [Populus trichocarpa]
228	16574	gi|224058852	182.58	18	15	4	Carbamidomethylation	94161	heat shock protein 70 [Populus trichocarpa]
357	161	gi|731388175	182.2	29	14	3	Carbamidomethylation	65990	PREDICTED: ATP-citrate synthase beta chain protein 2 [Vitis vinifera]
258	16544	gi|566173508	181.83	32	13	3	Carbamidomethylation; Oxidation (M)	56756	catalase family protein [Populus trichocarpa]
410	16576	gi|224119200	181.75	14	15	3	Carbamidomethylation	136903	coatomer alpha subunit-like family protein [Populus trichocarpa]
297	16663	gi|566170698	181.16	17	12	1	Carbamidomethylation	98215	aconitate hydratase family protein [Populus trichocarpa]
137	16871	gi|566205887	181.09	34	12	1	Carbamidomethylation; Oxidation (M)	38451	glutamine synthetase family protein [Populus trichocarpa]
312	16665	gi|224087339	181.07	32	13	3		57707	biotin carboxylase precursor family protein [Populus trichocarpa]
199	16705	gi|566210462	180.75	19	11	2	Carbamidomethylation	67453	Methylenetetrahydrofolate reductase family protein [Populus trichocarpa]
89	16804	gi|224053300	180.53	69	13	4	Carbamidomethylation	17702	ubiquitin/ribosomal protein 27a [Populus trichocarpa]

73	168	gi|359482256	179.66	47	15	1	Carbamidomethylation; Oxidation (M)	42618	PREDICTED: S-adenosylmethionine synthase 3 [Vitis vinifera]
302	163	gi|225456339	179.32	32	12	1	Carbamidomethylation	41981	PREDICTED: UDP-arabinopyranose mutase 1 isoform X2 [Vitis vinifera]
462	189	gi|731428324	179.26	8	15	3	Carbamidomethylation	252296	PREDICTED: acetyl-CoA carboxylase 1-like [Vitis vinifera]
308	241	gi|731413130	179.23	17	13	4	Oxidation (M)	116111	PREDICTED: 2-oxoglutarate dehydrogenase mitochondrial [Vitis vinifera]
96	159	gi|225462777	179.21	34	13	2	Carbamidomethylation	42597	PREDICTED: chalcone synthase 2 [Vitis vinifera]
327	16560	gi|566206267	178.47	15	13	4	Carbamidomethylation	100324	hypothetical protein POPTR_0015s06760g [Populus trichocarpa]
454	239	gi|225458111	178.35	11	13	4	Carbamidomethylation	136997	PREDICTED: coatomer subunit alpha-1 [Vitis vinifera]
88	186	gi|526117870	178.32	35	13	1	Carbamidomethylation	42744	chalcone synthase [Vitis vinifera]
372	16674	gi|542688125	178.25	21	11	11		56099	photosystem II p680 chlorophyll A apoprotein CP-47 (chloroplast) [Camellia taliensis]
570	231	gi|225450275	178.24	45	10	8	Carbamidomethylation	35771	PREDICTED: serine/threonine-protein phosphatase PP2A-2 catalytic subunit [Vitis vinifera]
324	317	gi|225441799	178.09	35	12	3	Carbamidomethylation	29974	PREDICTED: 40S ribosomal protein S2-3 [Vitis vinifera]
581	135	gi|731402494	177.6	17	13	2	Carbamidomethylation	102377	PREDICTED: coatomer subunit beta'-1 [Vitis vinifera]
381	16712	gi|224100783	177.48	18	12	3	Carbamidomethylation	77598	phenylalanine ammonia-lyase family protein [Populus trichocarpa]
379	16552	gi|224101561	177.22	27	15	4	Carbamidomethylation	65798	subunit B of the trimeric enzyme ATP Citrate lyase family protein [Populus trichocarpa]
745	17442	gi|566152994	177.18	24	11	2	Carbamidomethylation	57135	hypothetical protein POPTR_0001s35790g [Populus trichocarpa]
306	16573	gi|566151627	176.7	22	13	2		73210	heat shock protein 70 [Populus trichocarpa]

205	16583	gi|224079726	176.43	32	14	4	Carbamidomethylation	46428	isocitrate dehydrogenase family protein [Populus trichocarpa]
420	16601	gi|224069951	176.26	23	11	1	Carbamidomethylation	61124	Chaperonin CPN60-2 family protein [Populus trichocarpa]
337	371	gi|225457957	176.14	44	11	1	Carbamidomethylation	17938	PREDICTED: peptidyl-prolyl cis-trans isomerase [Vitis vinifera]
260	16775	gi|566198898	175.74	15	12	1		73464	heat shock protein 70 cognate [Populus trichocarpa]
203	353	gi|359483839	175.68	49	9	1	Carbamidomethylation	27890	PREDICTED: chlorophyll a-b binding protein of LHCII type 1 [Vitis vinifera]
280	16605	gi|224141947	175.53	29	12	1	Carbamidomethylation	53416	hypothetical protein POPTR_0018s02400g [Populus trichocarpa]
296	238	gi|359481634	175.49	72	11	5	Carbamidomethylation	16594	PREDICTED: ubiquitin-conjugating enzyme E2 variant 1D [Vitis vinifera]
599	362	gi|225464186	175.49	32	10	10	Carbamidomethylation	49585	PREDICTED: 26S proteasome regulatory subunit 4 homolog B [Vitis vinifera]
564	16895	gi|566191895	175.23	12	13	4	Carbamidomethylation	154710	phosphoribosylformylglycinamidine synthase family protein [Populus trichocarpa]
408	16953	gi|224105301	175.23	32	10	1	Carbamidomethylation	42832	latex plastidic aldolase-like family protein [Populus trichocarpa]
481	16713	gi|566213382	175.13	26	11	1	Carbamidomethylation	53743	phosphogluconate dehydrogenase family protein [Populus trichocarpa]
211	16939	gi|224109788	175.08	28	9	2	Carbamidomethylation	37547	putative adenosine kinase family protein [Populus trichocarpa]
349	16779	gi|566175799	175.06	34	15	3	Carbamidomethylation	57465	pyruvate kinase family protein [Populus trichocarpa]
317	16610	gi|566159486	175.02	15	18	2	Carbamidomethylation	113839	hypothetical protein POPTR_0002s22720g [Populus trichocarpa]
415	155	gi|225446449	174.66	15	10	2	Carbamidomethylation	97503	PREDICTED: 26S proteasome non-ATPase regulatory subunit 2 homolog A [Vitis vinifera]
341	221	gi|225445664	174.41	27	12	2		57338	PREDICTED: biotin carboxylase 1 chloroplastic [Vitis vinifera]

208	295	gi|359480275	174.39	33	11	1	Oxidation (M)	35505	PREDICTED: malate dehydrogenase cytoplasmic [Vitis vinifera]
270	16808	gi|566149378	174.23	28	10	3	Carbamidomethylation	34116	hypothetical protein POPTR_0001s16430g [Populus trichocarpa]
227	338	gi|225427917	173.92	31	13	3	Carbamidomethylation	34675	PREDICTED: triosephosphate isomerase chloroplastic [Vitis vinifera]
440	16898	gi|224116086	173.8	31	8	2	Carbamidomethylation	37284	hypothetical protein POPTR_0011s00500g [Populus trichocarpa]
188	16723	gi|224141865	173.33	46	10	10	Carbamidomethylation	25015	GTP-binding family protein [Populus trichocarpa]
423	16872	gi|566170807	173.28	31	13	1	Carbamidomethylation	40534	Chain A family protein [Populus trichocarpa]
184	494	gi|225449541	172.88	39	9	2	Carbamidomethylation	27128	PREDICTED: triosephosphate isomerase cytosolic [Vitis vinifera]
633	16786	gi|566208504	172.48	14	12	2	Carbamidomethylation; Oxidation (M)	128539	hypothetical protein POPTR_0016s03630g [Populus trichocarpa]
543	17029	gi|566210440	172.15	29	12	1	Carbamidomethylation	49767	GDP dissociation inhibitor family protein [Populus trichocarpa]
301	211	gi|225423507	172.05	32	15	1	Carbamidomethylation	52744	PREDICTED: UDP-glucose 6-dehydrogenase 3 [Vitis vinifera]
593	500	gi|225440011	171.92	15	9	1		77480	PREDICTED: phenylalanine ammonia-lyase [Vitis vinifera]
143	217	gi|225437428	171.91	16	14	2	Carbamidomethylation	92483	PREDICTED: sucrose synthase 2 [Vitis vinifera]
530	563	gi|731376167	171.78	38	8	3	Carbamidomethylation	29267	PREDICTED: proteasome subunit beta type-5 [Vitis vinifera]
444	625	gi|225431122	171.59	31	14	1	Carbamidomethylation	40534	PREDICTED: ferredoxin--NADP reductase leaf-type isozyme chloroplastic [Vitis vinifera]
377	177	gi|731398850	170.98	13	13	2	Carbamidomethylation	113970	PREDICTED: ubiquitin-activating enzyme E1 1-like isoform X2 [Vitis vinifera]
329	171	gi|225441044	170.97	31	14	2	Carbamidomethylation	57520	PREDICTED: pyruvate kinase cytosolic isozyme-like [Vitis vinifera]

223	16920	gi|566167040	170.75	27	10	1	Carbamidomethylation	33502	hypothetical protein POPTR_0004s17530g [Populus trichocarpa]
335	218	gi|731395250	170.2	26	9	2	Carbamidomethylation	48931	PREDICTED: elongation factor Tu mitochondrial [Vitis vinifera]
434	17244	gi|224113389	170.1	25	11	1		46762	DnaJ family protein [Populus trichocarpa]
446	17030	gi|566150460	170.08	54	9	9	Carbamidomethylation	16862	calmodulin-like protein 6a [Populus trichocarpa]
110	467	gi|225439749	170.01	50	9	4		15764	PREDICTED: histone H2A [Vitis vinifera]
865	17701	gi|566176059	169.88	22	9	1	Carbamidomethylation	57207	hypothetical protein POPTR_0006s13050g [Populus trichocarpa]
585	455	gi|225434259	169.55	25	11	1	Carbamidomethylation	49810	PREDICTED: guanosine nucleotide diphosphate dissociation inhibitor 2 [Vitis vinifera]
399	16671	gi|224077754	169.33	38	14	1	Carbamidomethylation	41253	type IIIa membrane protein cp-wap13 [Populus trichocarpa]
518	286	gi|731412519	169.03	16	9	1	Carbamidomethylation	78831	PREDICTED: phenylalanine ammonia-lyase G1 [Vitis vinifera]
224	440	gi|225461156	168.41	61	9	3		13980	PREDICTED: probable histone H2A.2 [Vitis vinifera]
339	257	gi|225466253	168.21	31	12	2	Carbamidomethylation	46284	PREDICTED: isocitrate dehydrogenase [NADP] [Vitis vinifera]
432	16781	gi|566165396	168.15	21	9	2	Carbamidomethylation	51757	hypothetical protein POPTR_0004s07280g [Populus trichocarpa]
489	17168	gi|224090159	167.78	15	16	2		151232	magnesium chelatase subunit family protein [Populus trichocarpa]
553	16721	gi|566157090	167.26	7	13	1	Carbamidomethylation	252616	hypothetical protein POPTR_0002s09330g [Populus trichocarpa]
747	16730	gi|566149642	167.16	14	11	1	Carbamidomethylation	103496	hypothetical protein POPTR_0001s17940g [Populus trichocarpa]
469	17186	gi|224122082	167.13	25	10	2	Carbamidomethylation	40896	type IIIa membrane protein cp-wap13 [Populus trichocarpa]

291	228	gi|225451995	166.51	47	12	2		29540	PREDICTED: 14-3-3-like protein A [Vitis vinifera]
436	585	gi|225444377	166.25	29	7	1	Carbamidomethylation	38105	PREDICTED: thiamine thiazole synthase 2 chloroplastic [Vitis vinifera]
447	844	gi|526117645	166.25	14	16	2		153182	magnesium chelatase H subunit [Vitis vinifera]
396	374	gi|225426162	165.68	43	9	2	Carbamidomethylation	20013	PREDICTED: 40S ribosomal protein S10 [Vitis vinifera]
97	16660	gi|566163210	165.47	28	12	1	Carbamidomethylation	42709	naregenin-chalcone synthase family protein [Populus trichocarpa]
285	560	gi|225468027	165.42	44	7	1	Carbamidomethylation	17468	PREDICTED: eukaryotic translation initiation factor 5A [Vitis vinifera]
448	16833	gi|566186876	164.84	13	12	1	Carbamidomethylation	122028	hypothetical protein POPTR_0009s07950g partial [Populus trichocarpa]
422	173	gi|225451806	164.71	36	13	1	Carbamidomethylation	40505	PREDICTED: UDP-arabinopyranose mutase 3 [Vitis vinifera]
505	209	gi|731408286	164.66	25	11	11	Carbamidomethylation	53389	PREDICTED: xylose isomerase [Vitis vinifera]
429	16962	gi|224128376	164.52	22	9	1	Carbamidomethylation	39401	thioredoxin family protein [Populus trichocarpa]
859	431	gi|731391105	164.23	25	8	4	Carbamidomethylation	49499	PREDICTED: calreticulin-3-like [Vitis vinifera]
388	367	gi|225444472	164.21	20	9	4	Carbamidomethylation	44306	PREDICTED: aminomethyltransferase mitochondrial [Vitis vinifera]
647	17023	gi|566202051	164.14	15	7	1	Carbamidomethylation	71118	hypothetical protein POPTR_0014s02550g [Populus trichocarpa]
449	16824	gi|566159427	163.51	25	11	1	Carbamidomethylation	49034	Elongation factor Tu family protein [Populus trichocarpa]
220	198	gi|731406211	163.38	14	12	4	Carbamidomethylation	92443	PREDICTED: sucrose synthase [Vitis vinifera]
441	17021	gi|224083020	163.22	48	10	2	Carbamidomethylation; Oxidation (M)	18138	Peptidyl-prolyl cis-trans isomerase family protein [Populus trichocarpa]
998	17690	gi|224053869	163.02	16	10	2	Carbamidomethylation	100242	hypothetical protein POPTR_0001s09660g [Populus trichocarpa]

604	583	gi|225435357	162.83	15	10	2		77966	PREDICTED: phenylalanine ammonia-lyase [Vitis vinifera]
700	411	gi|359497202	162.78	23	10	3	Carbamidomethylation	60614	PREDICTED: T-complex protein 1 subunit eta [Vitis vinifera]
625	292	gi|225439231	162.3	27	11	11	Carbamidomethylation	55389	PREDICTED: 3-isopropylmalate dehydratase large subunit-like [Vitis vinifera]
171	308	gi|731392297	162.27	28	14	13	Oxidation (M)	31832	PREDICTED: histone H2B-like [Vitis vinifera]
443	319	gi|359478860	162.22	45	14	13	Carbamidomethylation	47055	PREDICTED: 26S protease regulatory subunit 6B homolog [Vitis vinifera]
348	16670	gi|224073851	161.89	14	11	1	Carbamidomethylation	94625	heat shock protein 70 [Populus trichocarpa]
555	16755	gi|566211390	161.56	13	8	1	Carbamidomethylation	97445	hypothetical protein POPTR_0017s04660g [Populus trichocarpa]
662	16907	gi|566174304	161.43	9	11	1	Carbamidomethylation	177274	ferredoxin-dependent glutamate synthase family protein [Populus trichocarpa]
397	192	gi|225434712	161.16	37	9	4	Carbamidomethylation	29576	PREDICTED: proliferating cell nuclear antigen [Vitis vinifera]
435	16703	gi|224063273	161.11	45	12	2		29380	14-3-3 protein 32kDa endonuclease [Populus trichocarpa]
720	16798	gi|566149322	160.94	13	10	4	Carbamidomethylation	105097	putative plasma membrane H+ ATPase family protein [Populus trichocarpa]
362	254	gi|225430619	160.84	37	10	10	Carbamidomethylation	22781	PREDICTED: 40S ribosomal protein S5 [Vitis vinifera]
165	16968	gi|566210965	160.79	33	11	2	Carbamidomethylation	39217	glutamate-ammonia ligase family protein [Populus trichocarpa]
197	16754	gi|566176273	160.74	12	13	5	Carbamidomethylation	92143	sucrose synthase family protein [Populus trichocarpa]
102	281	gi|225465030	160.63	60	11	2		17196	PREDICTED: ubiquitin-NEDD8-like protein RUB2 [Vitis vinifera]
650	17197	gi|566153824	160.2	52	8	1	Carbamidomethylation	17248	ubiquitin-conjugating enzyme family protein [Populus trichocarpa]
364	194	gi|225442018	160.16	22	8	1	Carbamidomethylation	33909	PREDICTED: 40S ribosomal protein SA [Vitis vinifera]

743	17001	gi|566167613	160.11	23	9	2	Carbamidomethylation; Oxidation (M)	60440	chaperonin family protein [Populus trichocarpa]
356	16776	gi|224095561	159.82	11	8	2	Carbamidomethylation	97704	hypothetical protein POPTR_0007s01200g [Populus trichocarpa]
465	229	gi|731427020	159.81	20	12	3	Carbamidomethylation; Oxidation (M)	76330	PREDICTED: glycine--tRNA ligase 1 mitochondrial [Vitis vinifera]
246	176	gi|225448546	159.55	39	12	1	Carbamidomethylation	29880	PREDICTED: 40S ribosomal protein S4-1 [Vitis vinifera]
554	204	gi|731401620	159.32	14	11	3	Carbamidomethylation	104406	PREDICTED: ATPase 10 plasma membrane-type [Vitis vinifera]
366	237	gi|225446579	158.32	17	11	2	Carbamidomethylation; Oxidation (M)	63486	PREDICTED: ketol-acid reductoisomerase chloroplastic [Vitis vinifera]
779	17453	gi|224088276	158.24	21	8	2	Carbamidomethylation	50030	leucine aminopeptidase family protein [Populus trichocarpa]
279	170	gi|225446944	158.08	30	9	2	Carbamidomethylation	43340	PREDICTED: protochlorophyllide reductase [Vitis vinifera]
266	175	gi|225434482	157.97	39	12	1	Carbamidomethylation	29851	PREDICTED: 40S ribosomal protein S4 [Vitis vinifera]
545	16729	gi|566160536	157.95	14	9	1	Carbamidomethylation	91733	phospholipase D family protein [Populus trichocarpa]
468	282	gi|731423943	157.78	46	9	2	Carbamidomethylation	19961	PREDICTED: 40S ribosomal protein S10 [Vitis vinifera]
1037	785	gi|225459806	157.5	19	9	1	Carbamidomethylation	57322	PREDICTED: T-complex protein 1 subunit beta [Vitis vinifera]
417	16756	gi|566157403	157.2	20	10	1	Carbamidomethylation	57254	hypothetical protein POPTR_0002s10990g [Populus trichocarpa]
290	258	gi|731404310	157.06	27	8	5	Carbamidomethylation	33129	PREDICTED: putative lactoylglutathione lyase [Vitis vinifera]
325	214	gi|526117762	157.04	50	13	1	Carbamidomethylation	29376	14-3-3 protein [Vitis vinifera]
677	335	gi|225445867	156.54	18	8	2	Carbamidomethylation	60743	PREDICTED: leucine aminopeptidase 1 [Vitis vinifera]
787	16921	gi|224123784	156.4	23	10	2	Carbamidomethylation	58663	hypothetical protein POPTR_0013s05500g [Populus trichocarpa]

94	16659	gi|224110772	156.27	35	14	1	Carbamidomethylation	37124	glyceraldehyde 3-phosphate dehydrogenase family protein [Populus trichocarpa]
638	17101	gi|566165991	156.19	21	8	1	Carbamidomethylation	51717	hypothetical protein POPTR_0004s10870g [Populus trichocarpa]
736	17378	gi|566157671	156.06	16	6	1	Carbamidomethylation	71798	hypothetical protein POPTR_0002s12570g [Populus trichocarpa]
788	16938	gi|224145979	155.84	18	9	2	Carbamidomethylation	58735	hypothetical protein POPTR_0019s04980g [Populus trichocarpa]
556	428	gi|225464279	155.7	13	11	2	Carbamidomethylation	119705	PREDICTED: protein argonaute 1 [Vitis vinifera]
580	247	gi|225452974	155.57	28	16	11		58482	PREDICTED: probable mitochondrial-processing peptidase subunit beta [Vitis vinifera]
623	16897	gi|224135653	155.55	11	8	1	Carbamidomethylation	106660	putative plasma membrane H+ ATPase family protein [Populus trichocarpa]
172	16867	gi|566147823	155.5	15	10	3	Carbamidomethylation; Oxidation (M)	83349	hypothetical protein POPTR_0001s07870g [Populus trichocarpa]
425	417	gi|225429850	155.41	31	7	5		24915	PREDICTED: proteasome subunit beta type-6 [Vitis vinifera]
1155	1142	gi|225426194	155.33	15	9	1	Carbamidomethylation	100047	PREDICTED: beta-adaptin-like protein B [Vitis vinifera]
540	223	gi|225429530	155.3	20	8	3	Carbamidomethylation	54362	PREDICTED: UDP-sulfoquinovose synthase chloroplastic [Vitis vinifera]
759	16828	gi|224120474	155.25	12	10	1	Carbamidomethylation	117667	hypothetical protein POPTR_0012s03410g [Populus trichocarpa]
350	16815	gi|224117218	155.08	23	8	1	Carbamidomethylation	43107	NADPH-protochlorophyllide oxidoreductase family protein [Populus trichocarpa]
499	16706	gi|224113365	154.94	10	11	1	Carbamidomethylation	130750	NtN2 family protein [Populus trichocarpa]

492	16993	gi|566199099	154.9	43	9	1	Carbamidomethylation	19933	hypothetical protein POPTR_0013s02900g [Populus trichocarpa]
739	16970	gi|568244554	154.6	25	6	6		39549	photosystem II protein D2 (plastid) [Camellia oleifera]
346	16640	gi|671743230	154.38	14	9	8	Carbamidomethylation	82370	photosystem I P700 apoprotein A2 (chloroplast) [Camellia petelotii]
477	16791	gi|566173570	154.26	27	11	1	Carbamidomethylation	44912	hypothetical protein POPTR_0005s27550g [Populus trichocarpa]
651	337	gi|225423961	154.14	11	8	4	Carbamidomethylation	103148	PREDICTED: alpha-xylosidase 1 [Vitis vinifera]
512	17017	gi|566210666	153.8	15	6	1	Carbamidomethylation	51605	hypothetical protein POPTR_0017s01390g [Populus trichocarpa]
568	558	gi|225437493	153.72	18	8	1	Carbamidomethylation	58594	PREDICTED: importin subunit alpha [Vitis vinifera]
672	852	gi|225423722	153.43	36	6	3		25584	PREDICTED: proteasome subunit alpha type-2-B [Vitis vinifera]
844	596	gi|225466690	153.42	23	8	6		42525	PREDICTED: sedoheptulose-1 7-bisphosphatase chloroplastic [Vitis vinifera]
178	16985	gi|224100781	153.19	21	8	1	Carbamidomethylation	37413	putative adenosine kinase family protein [Populus trichocarpa]
351	16891	gi|224109580	153.17	15	12	1	Carbamidomethylation; Oxidation (M)	115877	2-oxoglutarate dehydrogenase E1 component family protein [Populus trichocarpa]
456	222	gi|225430398	153.11	22	10	2		50823	PREDICTED: aspartate aminotransferase chloroplastic [Vitis vinifera]
108	28868	gi|224120992	153.05	63	10	1		14687	UBIQUITIN EXTENSION protein 1 [Populus trichocarpa]
594	449	gi|731420830	153.03	30	7	1		31951	PREDICTED: prohibitin-1 mitochondrial [Vitis vinifera]
375	17019	gi|566193834	153.02	17	11	1	Carbamidomethylation	63609	hypothetical protein POPTR_0011s05250g [Populus trichocarpa]
610	17053	gi|566184833	152.9	22	9	2	Carbamidomethylation	53373	alanine-2-oxoglutarate aminotransferase 1 family protein [Populus trichocarpa]

467	452	gi|225434588	152.89	49	11	1	Carbamidomethylation	21882	PREDICTED: peptidyl-prolyl cis-trans isomerase CYP20-1 [Vitis vinifera]
500	16940	gi|566206623	152.65	17	10	3	Carbamidomethylation	54072	aldehyde dehydrogenase 1 precursor family protein [Populus trichocarpa]
529	16969	gi|224097488	152.44	8	9	1	Carbamidomethylation	103480	hypothetical protein POPTR_0008s01100g [Populus trichocarpa]
502	314	gi|225432110	152.19	18	9	4	Carbamidomethylation	52920	PREDICTED: dihydrolipoyl dehydrogenase mitochondrial [Vitis vinifera]
292	16655	gi|224146592	152.16	50	13	13	Carbamidomethylation	24975	60S ribosomal protein L10 [Populus trichocarpa]
1071	16949	gi|224056978	152.1	10	8	1	Carbamidomethylation	123482	hypothetical protein POPTR_0001s04200g [Populus trichocarpa]
609	16960	gi|566211039	151.93	10	9	3		124467	DegP protease family protein [Populus trichocarpa]
528	17207	gi|566151109	151.75	25	10	1	Carbamidomethylation	37403	hypothetical protein POPTR_0001s26400g [Populus trichocarpa]
696	416	gi|225456641	151.6	11	8	1	Carbamidomethylation	105309	PREDICTED: plasma membrane ATPase 1 isoform X1 [Vitis vinifera]
867	273	gi|731411266	151.42	11	10	2	Carbamidomethylation	134450	PREDICTED: cullin-associated NEDD8-dissociated protein 1 [Vitis vinifera]
771	593	gi|731412938	151.37	8	10	1	Carbamidomethylation	155024	PREDICTED: probable phosphoribosylformylglycinamidine synthase chloroplastic/mitochondrial [Vitis vinifera]
635	311	gi|225430390	151.33	22	10	4	Carbamidomethylation	57260	PREDICTED: threonine synthase chloroplastic [Vitis vinifera]
315	16797	gi|224109062	151.23	31	11	1		42679	cytosolic phosphoglycerate kinase family protein [Populus trichocarpa]
519	16951	gi|224097508	151.04	10	12	1	Carbamidomethylation	132402	NtN2 family protein [Populus trichocarpa]

912	17418	gi|568244568	150.75	21	7	7		56272	acetyl-CoA carboxylase carboxyltransferase beta subunit (plastid) [Camellia oleifera]
318	16634	gi|224059642	150.34	26	11	1		42100	adenine nucleotide translocator family protein [Populus trichocarpa]
480	269	gi|359476642	150.32	25	9	1	Carbamidomethylation	42804	PREDICTED: fructose-bisphosphate aldolase 1 chloroplastic-like [Vitis vinifera]
656	441	gi|225462272	150.21	32	7	1		31679	PREDICTED: prohibitin-1 mitochondrial isoform X1 [Vitis vinifera]
289	165	gi|359493870	149.91	22	10	4	Carbamidomethylation	42815	PREDICTED: protochlorophyllide reductase chloroplastic [Vitis vinifera]
498	140	gi|359475304	149.84	16	13	4	Carbamidomethylation	98624	PREDICTED: coatomer subunit gamma-2 [Vitis vinifera]
682	293	gi|225455784	149.56	22	8	6	Carbamidomethylation	50558	PREDICTED: enolase 1 chloroplastic [Vitis vinifera]
393	363	gi|225464852	149.41	57	7	1	Carbamidomethylation	16577	PREDICTED: ubiquitin-conjugating enzyme E2 variant 1D [Vitis vinifera]
320	17257	gi|224108938	149.35	38	5	1	Carbamidomethylation	17381	hypothetical protein POPTR_0010s17020g [Populus trichocarpa]
354	487	gi|225433247	149.19	49	6	6	Carbamidomethylation	15031	PREDICTED: 60S ribosomal protein L23 [Vitis vinifera]
371	16912	gi|566213236	149.1	41	10	1		29568	14-3-3 family protein [Populus trichocarpa]
841	276	gi|225432012	148.93	22	9	4	Carbamidomethylation	54243	PREDICTED: galactokinase [Vitis vinifera]
732	525	gi|225426063	148.88	24	9	2	Carbamidomethylation	53896	PREDICTED: 6-phosphogluconate dehydrogenase decarboxylating 1 chloroplastic [Vitis vinifera]
774	310	gi|225429772	148.87	12	9	2	Carbamidomethylation	104811	PREDICTED: plasma membrane ATPase 4 [Vitis vinifera]
267	16829	gi|224087813	148.8	31	9	1	Carbamidomethylation	29763	hypothetical protein POPTR_0006s10460g [Populus trichocarpa]
478	16958	gi|224099363	148.12	11	9	2		101194	alpha-xylosidase family protein [Populus trichocarpa]

345	301	gi|225452510	147.58	16	11	3	Carbamidomethylation	58057	PREDICTED: aldehyde dehydrogenase family 2 member B7 mitochondrial [Vitis vinifera]
313	16825	gi|566210060	147.58	31	9	1	Carbamidomethylation	29720	hypothetical protein POPTR_0016s13210g [Populus trichocarpa]
157	17277	gi|566170863	147.57	53	8	8		11409	histone H4 family protein [Populus trichocarpa]
276	17071	gi|224101413	147.51	45	6	1	Carbamidomethylation	17417	eukaryotic translation initiation factor 5A isoform VI family protein [Populus trichocarpa]
587	17069	gi|224077927	147.3	22	8	1	Carbamidomethylation	42796	latex plastidic aldolase-like family protein [Populus trichocarpa]
509	17097	gi|566161423	147.25	32	8	8	Carbamidomethylation	24286	60S ribosomal protein L15 [Populus trichocarpa]
566	316	gi|359480881	147.25	13	8	7	Carbamidomethylation	94936	PREDICTED: beta-galactosidase 10 [Vitis vinifera]
837	17036	gi|224063997	147.24	12	6	2		67268	NAD-dependent malic enzyme family protein [Populus trichocarpa]
843	320	gi|225441629	147.17	17	8	2	Carbamidomethylation	58891	PREDICTED: T-complex protein 1 subunit theta [Vitis vinifera]
295	16633	gi|566186668	147.13	26	11	2		41811	ADP family protein [Populus trichocarpa]
871	306	gi|731438745	147.12	12	8	4	Carbamidomethylation	92847	PREDICTED: DNA replication licensing factor MCM6 [Vitis vinifera]
309	216	gi|526117840	147.12	20	14	2	Carbamidomethylation	58698	3-deoxy-D-arabino-heptulosonate-7-phosphate synthase [Vitis vinifera]
513	378	gi|225439761	146.88	18	9	5	Carbamidomethylation	44771	PREDICTED: 60S ribosomal protein L4 [Vitis vinifera]
644	17494	gi|224134414	146.88	30	5	2		25625	Proteasome subunit alpha type 2 family protein [Populus trichocarpa]
783	17094	gi|224065699	146.69	22	10	10	Carbamidomethylation	75559	Cell division protein ftsH [Populus trichocarpa]
586	590	gi|225424316	146.66	19	8	3	Carbamidomethylation	63641	PREDICTED: phosphoglucomutase cytoplasmic [Vitis vinifera]
583	16749	gi|566209033	146.58	8	10	1	Carbamidomethylation	130968	UBIQUITIN-SPECIFIC PROTEASE 12 family protein [Populus trichocarpa]

982	16991	gi|566196314	146.58	5	8	2	Carbamidomethylation	244200	NADH-dependent glutamate synthase family protein [Populus trichocarpa]
1042	458	gi|359475502	146.49	28	7	1	Carbamidomethylation	37652	PREDICTED: malate dehydrogenase glyoxysomal [Vitis vinifera]
740	16788	gi|566171760	146.35	7	11	1	Carbamidomethylation	252377	acetyl-CoA carboxylase family protein [Populus trichocarpa]
572	447	gi|359494836	146.19	12	9	3	Carbamidomethylation	61508	PREDICTED: pyrophosphate--fructose 6-phosphate 1-phosphotransferase subunit beta [Vitis vinifera]
652	16827	gi|566182445	146.01	14	11	1	Carbamidomethylation; Oxidation (M)	115769	2-oxoglutarate dehydrogenase E1 component family protein [Populus trichocarpa]
607	16870	gi|224092216	145.98	18	8	7	Carbamidomethylation	57364	glycine hydroxymethyltransferase family protein [Populus trichocarpa]
654	16748	gi|224060514	145.95	16	8	4	Carbamidomethylation	70476	polyadenylate-binding family protein [Populus trichocarpa]
514	1030	gi|225453246	145.74	21	10	5		69276	PREDICTED: dynamin-related protein 1E [Vitis vinifera]
455	16783	gi|224084622	145.6	48	12	1		29301	14-3-3 protein 32kDa endonuclease [Populus trichocarpa]
395	255	gi|526117940	145.59	9	7	2	Carbamidomethylation	91805	phospholipase D alpha [Vitis vinifera]
989	17343	gi|224068388	145.47	18	6	1	Carbamidomethylation	53197	Fumarate hydratase 1 family protein [Populus trichocarpa]
653	550	gi|359488197	145.24	9	9	3		123095	PREDICTED: protease Do-like 7 isoform X1 [Vitis vinifera]
344	149	gi|225435480	144.63	24	10	1		42838	PREDICTED: ADP ATP carrier protein mitochondrial [Vitis vinifera]
665	16955	gi|566202195	144.39	29	7	7		34821	hypothetical protein POPTR_0014s03240g [Populus trichocarpa]
360	17194	gi|566181525	144.39	23	9	1		42160	hypothetical protein POPTR_0008s00350g [Populus trichocarpa]

674	424	gi|225428772	144.37	11	10	4	Carbamidomethylation; Oxidation (M)	121098	PREDICTED: presequence protease 2 chloroplastic/mitochondrial-like [Vitis vinifera]
262	16965	gi|224104103	144.2	36	7	7		24868	40S ribosomal protein S8 [Populus trichocarpa]
851	17457	gi|224129210	144.15	14	5	2	Carbamidomethylation	41121	truncated acetyl Co-A acetyltransferase-like family protein [Populus trichocarpa]
527	17126	gi|224141487	143.93	24	12	2	Carbamidomethylation	52967	UDP-glucose 6-dehydrogenase family protein [Populus trichocarpa]
624	1727	gi|225465837	143.93	6	5	4	Carbamidomethylation	46787	PREDICTED: protein ASPARTIC PROTEASE IN GUARD CELL 2 [Vitis vinifera]
668	400	gi|731401656	143.47	41	8	8	Carbamidomethylation	25978	PREDICTED: proteasome subunit alpha type-5 [Vitis vinifera]
697	750	gi|225457502	143.47	28	7	7	Carbamidomethylation	35357	PREDICTED: probable succinyl-CoA ligase [ADP-forming] subunit alpha mitochondrial [Vitis vinifera]
772	601	gi|731390491	143.45	12	7	4	Carbamidomethylation	84964	PREDICTED: protein transport protein SEC23-like [Vitis vinifera]
941	16998	gi|566156890	143.44	18	8	3		44077	hypothetical protein POPTR_0002s08240g [Populus trichocarpa]
230	17398	gi|224138586	143.12	24	7	3		27577	ascorbate peroxidase family protein [Populus trichocarpa]
620	243	gi|225429750	143.09	13	10	3	Carbamidomethylation	105919	PREDICTED: coatomer subunit beta-1 [Vitis vinifera]
369	17040	gi|566184599	143.06	28	11	3		37085	glyceraldehyde 3-phosphate dehydrogenase family protein [Populus trichocarpa]
578	16637	gi|566192573	143	20	12	2	Carbamidomethylation	56288	hypothetical protein POPTR_0010s24550g [Populus trichocarpa]
1170	690	gi|359473310	142.88	36	7	1	Carbamidomethylation	35042	PREDICTED: serine/threonine-protein phosphatase PP2A-2 catalytic subunit [Vitis vinifera]
701	16821	gi|566215982	142.85	16	9	1	Carbamidomethylation	60519	hypothetical protein POPTR_0019s00210g [Populus trichocarpa]

629	16785	gi|224075451	142.8	14	11	2	Carbamidomethylation	77174	Glycyl-tRNA synthetase family protein [Populus trichocarpa]
796	17264	gi|224121954	142.77	24	5	1	Carbamidomethylation	26774	hypothetical protein POPTR_0012s09720g [Populus trichocarpa]
457	333	gi|225447725	142.66	25	7	4	Carbamidomethylation	61438	PREDICTED: calnexin homolog [Vitis vinifera]
494	16889	gi|566170533	142.61	20	8	8	Carbamidomethylation	47176	hypothetical protein POPTR_0005s10290g [Populus trichocarpa]
877	17061	gi|224092184	142.37	17	6	1	Carbamidomethylation	53653	hypothetical protein POPTR_0006s24440g [Populus trichocarpa]
805	17066	gi|566167939	142.2	18	12	4	Carbamidomethylation	84308	hypothetical protein POPTR_0004s22010g [Populus trichocarpa]
741	1010	gi|225451999	142.16	21	10	1		59112	PREDICTED: T-complex protein 1 subunit zeta [Vitis vinifera]
591	541	gi|225436900	142.1	27	9	1	Carbamidomethylation	47680	PREDICTED: glutamine synthetase leaf isozyme chloroplastic [Vitis vinifera]
937	648	gi|731424733	142.05	4	8	2	Carbamidomethylation	241380	PREDICTED: glutamate synthase 1 [NADH] chloroplastic isoform X2 [Vitis vinifera]
510	245	gi|225462254	141.87	23	10	2	Carbamidomethylation	45573	PREDICTED: glyceraldehyde-3-phosphate dehydrogenase GAPCP1 chloroplastic [Vitis vinifera]
766	490	gi|225446595	141.84	46	8	1	Carbamidomethylation	17220	PREDICTED: ubiquitin-conjugating enzyme E2 36 [Vitis vinifera]
486	17820	gi|566175251	141.82	12	5	5		34569	hypothetical protein POPTR_0006s08710g [Populus trichocarpa]
549	969	gi|225426753	141.41	16	10	3	Carbamidomethylation; Oxidation (M)	81719	PREDICTED: primary amine oxidase [Vitis vinifera]
303	181	gi|359476269	141.22	50	12	2	Carbamidomethylation	26279	PREDICTED: 40S ribosomal protein S3-3 [Vitis vinifera]
558	413	gi|225457075	141.1	16	7	1	Carbamidomethylation	58613	PREDICTED: aldehyde dehydrogenase family 2 member B4 mitochondrial [Vitis vinifera]

523	16732	gi|435856353	141.1	24	8	8	Carbamidomethylation	38951	photosystem II protein D1 (chloroplast) [Camellia sinensis]
693	17446	gi|224122988	141.09	19	9	1	Carbamidomethylation	58241	Importin alpha-1 subunit family protein [Populus trichocarpa]
874	1222	gi|225457219	141.09	29	8	4	Carbamidomethylation	35220	PREDICTED: ATP synthase subunit gamma mitochondrial [Vitis vinifera]
322	16800	gi|224129102	141.06	23	8	8	Carbamidomethylation	42388	hypothetical protein POPTR_0014s15960g [Populus trichocarpa]
437	16635	gi|566178983	140.89	26	10	1		41070	hypothetical protein POPTR_0007s01010g [Populus trichocarpa]
896	17351	gi|224108774	140.68	22	8	1	Carbamidomethylation	54256	Galactokinase family protein [Populus trichocarpa]
430	379	gi|225442192	140.56	32	7	3		21908	PREDICTED: 60S ribosomal protein L9 [Vitis vinifera]
715	390	gi|225425838	140.5	33	7	3	Carbamidomethylation	26900	PREDICTED: adenylate kinase 4-like [Vitis vinifera]
330	16840	gi|566158870	140.28	63	9	9		17678	hypothetical protein POPTR_0002s19210g [Populus trichocarpa]
232	16937	gi|224098421	140.17	32	9	1	Carbamidomethylation	27259	Triosephosphate isomerase family protein [Populus trichocarpa]
582	261	gi|225458173	140.11	26	8	1	Carbamidomethylation	44535	PREDICTED: 26S protease regulatory subunit S10B homolog B [Vitis vinifera]
807	745	gi|731406154	139.79	5	8	3	Carbamidomethylation	199529	PREDICTED: brefeldin A-inhibited guanine nucleotide-exchange protein 5 [Vitis vinifera]
643	16896	gi|224107259	139.69	23	8	1	Carbamidomethylation	47894	glutamine synthetase family protein [Populus trichocarpa]
770	16892	gi|224143423	139.69	12	9	2	Carbamidomethylation	105985	putative coatmer beta subunit family protein [Populus trichocarpa]
858	699	gi|225435309	139.69	16	10	2	Carbamidomethylation	82345	PREDICTED: 4-hydroxy-3-methylbut-2-en-1-yl diphosphate synthase chloroplastic [Vitis vinifera]

1043	814	gi|225433414	139.62	22	6	6		36594	PREDICTED: probable voltage-gated potassium channel subunit beta [Vitis vinifera]
1158	17498	gi|566160463	139.61	16	5	5		49456	hypothetical protein POPTR_0003s02420g [Populus trichocarpa]
642	17296	gi|566213188	139.53	28	8	2	Carbamidomethylation	43893	nodule-enhanced malate dehydrogenase family protein [Populus trichocarpa]
544	16617	gi|568244555	139.43	18	8	8		51822	photosystem II CP43 chlorophyll apoprotein (plastid) [Camellia oleifera]
939	359	gi|225465253	139.32	11	8	1		100128	PREDICTED: dynamin-2A [Vitis vinifera]
522	251	gi|225461654	139.26	31	8	2		28864	PREDICTED: 14-3-3-like protein D [Vitis vinifera]
812	17358	gi|224114720	139.11	25	8	8	Carbamidomethylation	45639	Magnesium-chelatase subunit chlI family protein [Populus trichocarpa]
742	772	gi|225464906	139.09	48	6	6		16377	PREDICTED: 40S ribosomal protein S14 [Vitis vinifera]
300	235	gi|225444782	138.97	50	12	2	Carbamidomethylation	26314	PREDICTED: 40S ribosomal protein S3-3 [Vitis vinifera]
1151	609	gi|731392506	138.92	7	8	3	Carbamidomethylation	129324	PREDICTED: carbamoyl-phosphate synthase large chain chloroplastic [Vitis vinifera]
551	17104	gi|671743459	138.84	73	5	5	Carbamidomethylation	9038	photosystem I subunit VII (chloroplast) [Camellia reticulata]
666	17844	gi|566198902	138.77	41	6	1		15349	hypothetical protein POPTR_0013s01890g [Populus trichocarpa]
508	602	gi|359476682	138.76	25	6	1	Carbamidomethylation	24349	PREDICTED: soluble inorganic pyrophosphatase isoform X2 [Vitis vinifera]
658	598	gi|225454009	138.73	13	6	1	Carbamidomethylation	80666	PREDICTED: transketolase chloroplastic [Vitis vinifera]
501	17258	gi|224140177	138.41	15	9	3	Carbamidomethylation	53698	adenylosuccinate synthetase family protein [Populus trichocarpa]
250	226	gi|359497288	138.3	19	11	4	Carbamidomethylation; Oxidation (M)	64041	PREDICTED: peptidyl-prolyl cis-trans isomerase FKBP62 isoform X1 [Vitis vinifera]

695	17238	gi|566164179	138.3	17	8	2	Carbamidomethylation	61299	hypothetical protein POPTR_0004s00550g [Populus trichocarpa]
818	17259	gi|566170121	138.22	16	7	3	Carbamidomethylation	56464	1L-myo-inositol 1-phosphate synthase family protein [Populus trichocarpa]
390	16874	gi|224082478	138.13	24	10	2	Carbamidomethylation	44381	ribosomal protein 1 [Populus trichocarpa]
535	307	gi|225468761	138.11	24	6	1	Carbamidomethylation	35289	PREDICTED: oxygen-evolving enhancer protein 1 chloroplastic [Vitis vinifera]
576	843	gi|225439878	138.09	25	6	1	Carbamidomethylation	24585	PREDICTED: soluble inorganic pyrophosphatase [Vitis vinifera]
1123	17291	gi|224100197	138.03	32	7	1	Carbamidomethylation	35036	type 2A protein phosphtase-II [Populus trichocarpa]
304	16784	gi|566175737	138.02	24	9	2	Carbamidomethylation	47050	Monodehydroascorbate reductase family protein [Populus trichocarpa]
202	595	gi|225447408	138.01	34	7	3	Carbamidomethylation	28115	PREDICTED: 60S ribosomal protein L8 [Vitis vinifera]
336	188	gi|731408817	137.95	22	9	1		41099	PREDICTED: ADP ATP carrier protein 3 mitochondrial [Vitis vinifera]
484	16593	gi|552541036	137.92	21	12	8		55539	ATP synthase CF1 alpha subunit (chloroplast) [Camellia yunnanensis]
524	364	gi|225451146	137.77	16	10	7		57701	PREDICTED: bifunctional 3-dehydroquinate dehydratase/shikimate dehydrogenase chloroplastic [Vitis vinifera]
749	1570	gi|731427302	137.61	31	6	6		17173	PREDICTED: transcription factor BTF3 homolog 4 [Vitis vinifera]
559	369	gi|359492927	137.45	27	6	2		28650	PREDICTED: 14-3-3-like protein GF14 kappa [Vitis vinifera]
617	17520	gi|224104913	137.28	53	5	2	Carbamidomethylation	12354	60S ribosomal protein L30 [Populus trichocarpa]
257	16818	gi|224135747	136.99	49	13	2	Carbamidomethylation	26076	40S ribosomal protein S3 [Populus trichocarpa]
746	592	gi|225437537	136.77	42	8	8		22019	PREDICTED: GTP-binding protein SAR1A [Vitis vinifera]

391	621	gi|225442061	136.76	17	7	1		45655	PREDICTED: oligouridylate-binding protein 1B isoform X1 [Vitis vinifera]
826	1012	gi|225430650	136.7	28	8	1	Carbamidomethylation; Oxidation (M)	43927	PREDICTED: pyruvate dehydrogenase E1 component subunit beta-3 chloroplastic [Vitis vinifera]
782	581	gi|731401982	136.6	11	7	2	Carbamidomethylation	60389	PREDICTED: dihydrolipoyllysine-residue acetyltransferase component 2 of pyruvate dehydrogenase complex mitochondrial [Vitis vinifera]
754	17771	gi|566148911	136.55	17	5	3	Carbamidomethylation	48782	hypothetical protein POPTR_0001s14280g [Populus trichocarpa]
606	17234	gi|566207041	136.5	15	5	3	Carbamidomethylation	53652	glutamate 1-semialdehyde aminotransferase family protein [Populus trichocarpa]
885	17148	gi|224054150	136.48	29	8	2	Carbamidomethylation	38004	transducin family protein [Populus trichocarpa]
675	765	gi|225443272	136.37	18	10	2	Carbamidomethylation; Oxidation (M)	66288	PREDICTED: D-3-phosphoglycerate dehydrogenase 3 chloroplastic-like [Vitis vinifera]
806	17401	gi|566160244	136.29	5	6	4	Carbamidomethylation	181683	hypothetical protein POPTR_0003s01280g [Populus trichocarpa]
1095	303	gi|731406982	136.15	9	7	1	Carbamidomethylation	102765	PREDICTED: coatomer subunit beta'-1-like [Vitis vinifera]
314	618	gi|225464412	136.04	35	8	7		16188	PREDICTED: 40S ribosomal protein S17 [Vitis vinifera]
875	963	gi|225424564	136.02	16	8	8	Carbamidomethylation	59200	PREDICTED: T-complex protein 1 subunit epsilon [Vitis vinifera]
824	462	gi|225463163	135.96	18	9	1	Carbamidomethylation	60505	PREDICTED: T-complex protein 1 subunit gamma [Vitis vinifera]
936	16961	gi|566166593	135.95	8	8	1	Carbamidomethylation	122467	hypothetical protein POPTR_0004s14960g [Populus trichocarpa]
802	932	gi|225450626	135.94	15	6	1	Carbamidomethylation	39269	PREDICTED: probable protein disulfide-isomerase A6 [Vitis vinifera]

1006	445	gi|225454087	135.83	12	6	2		66696	PREDICTED: NAD-dependent malic enzyme 59 kDa isoform mitochondrial [Vitis vinifera]
631	17070	gi|224064707	135.82	13	7	3	Carbamidomethylation	58836	mitochondrial aldehyde dehydrogenase family protein [Populus trichocarpa]
538	253	gi|225451581	135.67	29	10	4	Carbamidomethylation	38690	PREDICTED: mitochondrial phosphate carrier protein 3 mitochondrial isoform X1 [Vitis vinifera]
1259	17015	gi|224129596	135.67	16	6	1	Carbamidomethylation	45020	Succinyl-CoA ligase beta-chain family protein [Populus trichocarpa]
588	538	gi|225439982	135.36	49	6	1	Carbamidomethylation	17793	PREDICTED: 60S ribosomal protein L12 [Vitis vinifera]
569	461	gi|731404671	135.33	27	7	2		29622	PREDICTED: 14-3-3-like protein D isoform X1 [Vitis vinifera]
81	410	gi|359475042	135.26	37	9	1	Carbamidomethylation	18313	PREDICTED: peptidyl-prolyl cis-trans isomerase [Vitis vinifera]
984	17228	gi|224103569	135.26	8	7	1	Carbamidomethylation	112946	hypothetical protein POPTR_0009s10650g [Populus trichocarpa]
1028	938	gi|225450669	135.25	7	7	4		113734	PREDICTED: eukaryotic translation initiation factor 3 subunit A [Vitis vinifera]
577	473	gi|225436253	135.12	13	7	1	Carbamidomethylation	66904	PREDICTED: probable methylenetetrahydrofolate reductase [Vitis vinifera]
691	664	gi|731403303	135.1	33	5	1	Carbamidomethylation	15026	PREDICTED: 40S ribosomal protein S12 [Vitis vinifera]
526	271	gi|225439064	134.93	16	7	4		61092	PREDICTED: 2 3-bisphosphoglycerate-independent phosphoglycerate mutase [Vitis vinifera]
352	17173	gi|566149424	134.82	18	7	1	Oxidation (M)	46427	oligouridylate-binding family protein [Populus trichocarpa]
704	519	gi|225450981	134.75	26	8	8	Carbamidomethylation	51125	PREDICTED: serine--tRNA ligase [Vitis vinifera]
414	437	gi|225465198	134.72	13	6	1		48147	PREDICTED: elongation factor 1-gamma-like [Vitis vinifera]

828	599	gi|225459126	134.68	14	8	2	Carbamidomethylation	68420	PREDICTED: glucose-6-phosphate isomerase 1 chloroplastic [Vitis vinifera]
517	17045	gi|224084209	134.66	18	6	1	Carbamidomethylation	35142	O2 evolving complex 33kD family protein [Populus trichocarpa]
612	708	gi|731392629	134.62	49	6	1	Carbamidomethylation	17778	PREDICTED: 60S ribosomal protein L12 [Vitis vinifera]
563	962	gi|225447723	134.51	8	9	3		109436	PREDICTED: staphylococcal nuclease domain-containing protein 1 [Vitis vinifera]
401	361	gi|225431804	134.46	31	7	7		24165	PREDICTED: 60S ribosomal protein L19-2 [Vitis vinifera]
602	16801	gi|224105405	134.46	11	9	1	Carbamidomethylation	98780	hypothetical protein POPTR_0009s11800g [Populus trichocarpa]
671	17062	gi|224090212	134.21	28	9	2	Carbamidomethylation	29609	40S ribosomal protein S2 [Populus trichocarpa]
949	637	gi|225431563	133.82	14	6	1	Carbamidomethylation	51250	PREDICTED: UTP--glucose-1-phosphate uridylyltransferase isoform X1 [Vitis vinifera]
637	667	gi|225470804	133.74	23	7	1	Carbamidomethylation	45224	PREDICTED: phosphoribulokinase chloroplastic [Vitis vinifera]
835	17480	gi|566164885	133.73	12	8	1		81287	Vesicle-fusing ATPase family protein [Populus trichocarpa]
905	17229	gi|566176021	133.48	12	8	1		77716	phenylalanine ammonia-lyase family protein [Populus trichocarpa]
836	17095	gi|224100953	133.07	21	6	1	Carbamidomethylation	39107	thymidine diphospho-glucose 4-6-dehydratase family protein [Populus trichocarpa]
1051	691	gi|731397286	133.01	17	7	2	Carbamidomethylation; Oxidation (M)	56371	PREDICTED: inositol-3-phosphate synthase [Vitis vinifera]
622	17131	gi|224057882	133.01	11	8	2		89495	outer membrane family protein [Populus trichocarpa]
1221	706	gi|225446693	132.99	14	7	4		74318	PREDICTED: ATP-dependent zinc metalloprotease FTSH 2 chloroplastic [Vitis vinifera]
180	17005	gi|566146555	132.91	36	10	3		23812	60S ribosomal protein L13 [Populus trichocarpa]

969	17353	gi|224142207	132.74	11	5	2	Carbamidomethylation	65147	hypothetical protein POPTR_0018s09380g [Populus trichocarpa]
920	1769	gi|225461445	132.58	14	9	2		81247	PREDICTED: vesicle-fusing ATPase [Vitis vinifera]
820	422	gi|731439184	132.58	12	9	9		109426	PREDICTED: 26S proteasome non-ATPase regulatory subunit 1 homolog A [Vitis vinifera]
856	17252	gi|224075976	132.51	26	6	6	Carbamidomethylation	31893	hypothetical protein POPTR_0003s21070g [Populus trichocarpa]
777	947	gi|225463556	132.42	7	7	1	Carbamidomethylation	125024	PREDICTED: protein TOPLESS [Vitis vinifera]
1005	17515	gi|224141339	132.28	25	7	1	Carbamidomethylation	44746	PYRUVATE DEHYDROGENASE E1 BETA family protein [Populus trichocarpa]
608	16922	gi|224058615	132.11	24	6	3	Carbamidomethylation	33817	hypothetical protein POPTR_0001s08570g [Populus trichocarpa]
340	17042	gi|566164883	132.1	25	9	2	Carbamidomethylation	43246	ketol-acid reductoisomerase family protein partial [Populus trichocarpa]
1070	628	gi|225435754	132.09	7	7	1	Carbamidomethylation	123396	PREDICTED: importin-5 [Vitis vinifera]
626	482	gi|526118008	131.99	12	5	1		56117	flavonoid 3' hydroxylase [Vitis vinifera]
597	572	gi|731382516	131.96	17	5	5	Carbamidomethylation	37912	PREDICTED: ankyrin repeat domain-containing protein 2 isoform X2 [Vitis vinifera]
958	17018	gi|566148676	131.94	13	8	1	Carbamidomethylation	77001	Glycyl-tRNA synthetase family protein [Populus trichocarpa]
854	17027	gi|566195881	131.85	17	5	2	Carbamidomethylation	44659	hypothetical protein POPTR_0011s16690g [Populus trichocarpa]
1252	398	gi|731405921	131.78	26	5	5	Carbamidomethylation	34871	PREDICTED: phytochrome-associated serine/threonine-protein phosphatase [Vitis vinifera]
539	313	gi|225458424	131.76	19	6	1	Carbamidomethylation	35120	PREDICTED: oxygen-evolving enhancer protein 1 chloroplastic [Vitis vinifera]

728	17433	gi|566214954	131.57	18	6	5	Carbamidomethylation	39277	hypothetical protein POPTR_0018s09170g [Populus trichocarpa]
311	17311	gi|566254838	131.52	23	6	2		28211	hypothetical protein POPTR_0789s00200g [Populus trichocarpa]
496	389	gi|225441754	131.46	16	9	3	Carbamidomethylation	53382	PREDICTED: adenylosuccinate synthetase 2 chloroplastic [Vitis vinifera]
613	610	gi|225464928	131.46	15	7	2		46452	PREDICTED: eukaryotic initiation factor 4A-3 [Vitis vinifera]
627	16893	gi|566170007	131.45	29	8	8	Carbamidomethylation	28435	hypothetical protein POPTR_0005s07380g [Populus trichocarpa]
991	611	gi|225437455	131.41	18	7	2	Carbamidomethylation	53532	PREDICTED: fumarate hydratase 1 mitochondrial [Vitis vinifera]
762	741	gi|731399769	131.41	36	7	2	Carbamidomethylation	30174	PREDICTED: 2-Cys peroxiredoxin isoform X1 [Vitis vinifera]
952	468	gi|731369971	131.23	19	6	4	Carbamidomethylation	42894	PREDICTED: probable fructose-bisphosphate aldolase 3 chloroplastic [Vitis vinifera]
663	17656	gi|566190366	131.21	13	7	2		63088	cytosolic phosphoglucomutase family protein [Populus trichocarpa]
964	17635	gi|224092266	131.15	14	7	1	Carbamidomethylation	63096	hypothetical protein POPTR_0006s25280g [Populus trichocarpa]
1329	693	gi|225437557	131.15	10	6	2	Carbamidomethylation	81022	PREDICTED: DNA replication licensing factor MCM7 [Vitis vinifera]
533	17013	gi|566164286	130.92	15	7	2	Carbamidomethylation	44313	T-protein of the glycine decarboxylase complex [Populus trichocarpa]
868	754	gi|225428865	130.83	10	5	1		70683	PREDICTED: polyadenylate-binding protein 2 [Vitis vinifera]
723	562	gi|225425272	130.83	9	7	1	Carbamidomethylation	74889	PREDICTED: glycine--tRNA ligase 1 mitochondrial [Vitis vinifera]
891	341	gi|731391907	130.35	13	6	3	Carbamidomethylation	67493	PREDICTED: anthranilate synthase alpha subunit 2 chloroplastic isoform X2 [Vitis vinifera]

487	412	gi|225443760	130.27	36	8	1	Carbamidomethylation	20875	PREDICTED: 60S ribosomal protein L11-1-like [Vitis vinifera]
892	991	gi|359494079	130.24	12	6	6	Carbamidomethylation	65102	PREDICTED: LOW QUALITY PROTEIN: 65-kDa microtubule-associated protein 1-like [Vitis vinifera]
659	16707	gi|224126969	129.83	18	10	1		58085	hypothetical protein POPTR_0013s15380g [Populus trichocarpa]
986	717	gi|225463033	129.7	14	9	9	Carbamidomethylation	110326	PREDICTED: 116 kDa U5 small nuclear ribonucleoprotein component [Vitis vinifera]
1089	1348	gi|225462545	129.42	38	8	4	Carbamidomethylation; Oxidation (M)	27262	PREDICTED: proteasome subunit alpha type-4 [Vitis vinifera]
1030	17713	gi|224100535	129.36	12	7	1		67584	hypothetical protein POPTR_0008s01000g [Populus trichocarpa]
687	481	gi|225442079	129.21	25	6	6		27325	PREDICTED: proteasome subunit alpha type-6 [Vitis vinifera]
532	627	gi|225464720	129.18	27	8	3	Carbamidomethylation	44946	PREDICTED: oxygen-dependent coproporphyrinogen-III oxidase chloroplastic-like [Vitis vinifera]
702	17198	gi|566259520	129.08	22	6	1	Carbamidomethylation	34228	hypothetical protein POPTR_0030s00390g [Populus trichocarpa]
247	18507	gi|566242200	129.02	14	8	5		19093	hypothetical protein POPTR_0019s12370g partial [Populus trichocarpa]
781	644	gi|731431241	128.99	13	5	1		47331	PREDICTED: protein TIC 40 chloroplastic isoform X2 [Vitis vinifera]
676	17464	gi|566146625	128.97	23	7	1	Carbamidomethylation	45025	Phosphoribulokinase family protein [Populus trichocarpa]
727	1063	gi|359491801	128.94	11	8	8		83367	PREDICTED: dolichyl-diphosphooligosaccharide--protein glycosyltransferase subunit STT3B [Vitis vinifera]
947	818	gi|731396254	128.91	16	5	4	Carbamidomethylation	40707	PREDICTED: alcohol dehydrogenase class-3 [Vitis vinifera]

846	17503	gi|566165056	128.77	15	6	1	Carbamidomethylation	36365	hypothetical protein POPTR_0004s05340g [Populus trichocarpa]
902	17297	gi|224141065	128.67	13	6	1		58604	mitochondrial processing peptidase beta subunit family protein [Populus trichocarpa]
1085	17473	gi|224086589	128.49	11	5	1	Carbamidomethylation	56517	POLLEN-PISTIL INTERACTION 2 family protein [Populus trichocarpa]
993	18380	gi|224136183	128.26	11	4	2		70641	acetolactate synthase family protein [Populus trichocarpa]
1275	17796	gi|566194692	128.08	19	4	2	Carbamidomethylation	32070	hypothetical protein POPTR_0011s10100g [Populus trichocarpa]
255	16812	gi|566168262	128.08	16	10	3		47873	elongation factor 1-gamma 1 family protein [Populus trichocarpa]
229	446	gi|225465463	128.07	32	7	7	Carbamidomethylation	23516	PREDICTED: 60S ribosomal protein L13a-4 [Vitis vinifera]
1137	773	gi|225440478	128.04	15	3	2	Carbamidomethylation	35390	PREDICTED: aldo-keto reductase family 4 member C9-like [Vitis vinifera]
1054	17789	gi|566172819	128.03	14	7	2	Carbamidomethylation	47443	hypothetical protein POPTR_0005s23110g [Populus trichocarpa]
237	17140	gi|224075758	127.88	56	8	3	Carbamidomethylation	15268	hypothetical protein POPTR_0003s22120g [Populus trichocarpa]
660	17452	gi|566147835	127.51	25	6	5		26392	hypothetical protein POPTR_0001s07920g partial [Populus trichocarpa]
1027	732	gi|731414624	127.51	7	7	5		122431	PREDICTED: protein transport protein SEC31 homolog B isoform X1 [Vitis vinifera]
855	17794	gi|566152502	127.4	19	6	1	Carbamidomethylation	29370	hypothetical protein POPTR_0001s33400g [Populus trichocarpa]
458	17083	gi|224091827	127.34	41	9	2	Carbamidomethylation	20659	60S ribosomal protein L11-2 [Populus trichocarpa]

688	1226	gi|731439514	127.22	17	6	1	Carbamidomethylation	41530	PREDICTED: acetyl-CoA acetyltransferase cytosolic 1 isoform X4 [Vitis vinifera]
1183	1116	gi|225463618	127.04	13	9	1	Carbamidomethylation	80442	PREDICTED: pyrophosphate-energized vacuolar membrane proton pump [Vitis vinifera]
760	17008	gi|566186274	126.96	25	7	2	Carbamidomethylation	29153	proliferating cell nuclear antigen family protein [Populus trichocarpa]
983	1095	gi|731423779	126.88	16	6	4		63328	PREDICTED: plastidial pyruvate kinase 2 [Vitis vinifera]
678	902	gi|225461808	126.86	16	10	1	Carbamidomethylation	75747	PREDICTED: trifunctional UDP-glucose 4 6-dehydratase/UDP-4-keto-6-deoxy-D-glucose 3 5-epimerase/UDP-4-keto-L-rhamnose-reductase RHM1 [Vitis vinifera]
640	545	gi|526117441	126.79	7	8	3		101670	lipoxygenase [Vitis vinifera]
1141	828	gi|225427605	126.75	16	7	7	Carbamidomethylation	68375	PREDICTED: ABC transporter E family member 2 [Vitis vinifera]
840	728	gi|359485890	126.72	6	5	2	Carbamidomethylation	83365	PREDICTED: beta-xylosidase/alpha-L-arabinofuranosidase 2-like [Vitis vinifera]
281	17233	gi|224065421	126.64	10	7	1		64078	hypothetical protein POPTR_0002s24970g [Populus trichocarpa]
1069	3590	gi|731418129	126.57	2	7	3	Carbamidomethylation	565777	PREDICTED: auxin transport protein BIG [Vitis vinifera]
765	612	gi|225423953	126.41	25	6	3	Oxidation (M)	41947	PREDICTED: ubiquitin receptor RAD23c isoform X2 [Vitis vinifera]
904	1634	gi|731413091	126.4	24	6	1		38789	PREDICTED: UDP-glucuronic acid decarboxylase 5 [Vitis vinifera]
684	676	gi|731421194	126.39	31	6	3	Carbamidomethylation	20876	PREDICTED: 40S ribosomal protein S13 [Vitis vinifera]
259	17038	gi|566174988	126.31	28	7	2		28378	hypothetical protein POPTR_0006s07290g [Populus trichocarpa]
698	365	gi|225459918	126.3	26	5	3	Carbamidomethylation	30824	PREDICTED: chlorophyll a-b binding protein CP26 chloroplastic [Vitis vinifera]

1148	1177	gi|225441573	125.96	8	7	4	Carbamidomethylation	107099	PREDICTED: eukaryotic translation initiation factor 3 subunit C [Vitis vinifera]
485	584	gi|225451279	125.69	17	5	1		29903	PREDICTED: 40S ribosomal protein S3a-2-like [Vitis vinifera]
1075	17184	gi|224125232	125.63	14	6	1	Carbamidomethylation	47047	19S proteosome subunit 9 family protein [Populus trichocarpa]
734	520	gi|225431090	125.54	30	5	5		27195	PREDICTED: proteasome subunit alpha type-7 [Vitis vinifera]
832	727	gi|225464809	125.52	12	6	2	Carbamidomethylation	56995	PREDICTED: gamma aminobutyrate transaminase 1 mitochondrial-like [Vitis vinifera]
1348	17054	gi|566183505	125.52	12	5	2	Carbamidomethylation	65372	phosphatase family protein [Populus trichocarpa]
1038	17917	gi|566206287	125.49	18	6	2		35464	ATP synthase gamma chain family protein [Populus trichocarpa]
959	1027	gi|359482368	125.47	5	9	9		274480	PREDICTED: pre-mRNA-processing-splicing factor 8 [Vitis vinifera]
511	547	gi|225456295	125.39	18	5	4		24961	PREDICTED: elongation factor 1-delta [Vitis vinifera]
521	17129	gi|566149060	125.13	17	9	1	Carbamidomethylation	57242	hypothetical protein POPTR_0001s15050g [Populus trichocarpa]
1163	2169	gi|731371107	125.1	8	5	5		89995	PREDICTED: probable splicing factor 3A subunit 1 [Vitis vinifera]
116	859	gi|225429614	124.94	45	5	5	Carbamidomethylation; Oxidation (M)	17293	PREDICTED: 17.3 kDa class II heat shock protein [Vitis vinifera]
630	17572	gi|566179689	124.92	19	9	1	Carbamidomethylation	50228	hypothetical protein POPTR_0007s05160g [Populus trichocarpa]
1076	748	gi|225441549	124.91	18	8	7	Carbamidomethylation	67018	PREDICTED: DEAD-box ATP-dependent RNA helicase 37-like [Vitis vinifera]
1593	28871	gi|225460917	124.83	16	6	6	Carbamidomethylation	64249	PREDICTED: imidazole glycerol phosphate synthase hisHF chloroplastic [Vitis vinifera]
848	17363	gi|224109746	124.78	14	5	5	Carbamidomethylation	26377	putative chlorophyll a/b-binding family protein [Populus trichocarpa]

931	17535	gi|566180192	124.72	23	5	5		27792	hypothetical protein POPTR_0007s08020g [Populus trichocarpa]
942	17237	gi|566165848	124.6	20	9	1		59085	hypothetical protein POPTR_0004s10100g [Populus trichocarpa]
1425	17327	gi|224086948	124.45	19	6	1	Carbamidomethylation	51291	eukaryotic translation initiation factor 3E family protein [Populus trichocarpa]
888	17298	gi|224063082	124.42	22	5	5		28992	Photosystem II 22 kDa family protein [Populus trichocarpa]
1111	17653	gi|566190217	123.95	12	3	1	Carbamidomethylation	35803	TGF-beta receptor-interacting protein 1 [Populus trichocarpa]
1258	17778	gi|566199576	123.81	6	6	1	Carbamidomethylation	123531	hypothetical protein POPTR_0013s05320g [Populus trichocarpa]
1126	17256	gi|224125002	123.21	10	5	1	Carbamidomethylation	59410	importin alpha-1 subunit family protein [Populus trichocarpa]
413	16803	gi|224089867	123.16	8	9	1	Carbamidomethylation	103746	hypothetical protein POPTR_0006s02680g [Populus trichocarpa]
1437	17909	gi|224133898	123.09	15	6	3	Carbamidomethylation	53810	aspartyl aminopeptidase family protein [Populus trichocarpa]
226	17618	gi|224102515	123.03	20	6	4		29608	porin family protein [Populus trichocarpa]
811	548	gi|225434863	122.97	11	7	2		88320	PREDICTED: protein TOC75-3 chloroplastic [Vitis vinifera]
1165	600	gi|359490767	122.81	32	5	1		23070	PREDICTED: nascent polypeptide-associated complex subunit alpha-like protein 2 [Vitis vinifera]
894	686	gi|225429234	122.79	24	5	5		34319	PREDICTED: mitochondrial import receptor subunit TOM40-1 [Vitis vinifera]
967	1154	gi|225464635	122.66	19	7	1		40639	PREDICTED: protein SGT1 homolog isoform X2 [Vitis vinifera]
985	529	gi|731399749	122.49	18	5	5	Carbamidomethylation	44581	PREDICTED: GDHB glutamate dehydrogenase isoform X1 [Vitis vinifera]
1099	606	gi|731438689	122.49	16	6	2	Oxidation (M)	44313	PREDICTED: FAM10 family protein At4g22670 [Vitis vinifera]

1303	17135	gi|566177234	122.48	12	8	7	Carbamidomethylation	80627	minichromosome maintenance family protein [Populus trichocarpa]
1120	17016	gi|566148627	122.33	9	7	2	Carbamidomethylation	107277	hypothetical protein POPTR_0001s12700g [Populus trichocarpa]
1196	17396	gi|224119992	122.3	5	5	1	Carbamidomethylation	143158	hypothetical protein POPTR_0012s13100g [Populus trichocarpa]
845	466	gi|225426743	122.26	19	5	2	Carbamidomethylation	33675	PREDICTED: bifunctional dTDP-4-dehydrorhamnose 3 5-epimerase/dTDP-4-dehydrorhamnose reductase [Vitis vinifera]
450	17591	gi|224111204	122.14	25	6	6	Carbamidomethylation	22208	photosystem I 20kD family protein [Populus trichocarpa]
775	405	gi|225443308	122.06	7	6	1	Carbamidomethylation	106276	PREDICTED: coatomer subunit beta-1 [Vitis vinifera]
1398	725	gi|225434692	121.99	9	6	2		95490	PREDICTED: alpha-glucan phosphorylase H isozyme [Vitis vinifera]
1210	17616	gi|566182000	121.94	14	7	1	Carbamidomethylation	57819	hypothetical protein POPTR_0008s02740g [Populus trichocarpa]
1093	17512	gi|566158761	121.93	15	4	1	Carbamidomethylation	29101	cyclophilin family protein [Populus trichocarpa]
1060	874	gi|359478691	121.88	9	5	2		92178	PREDICTED: beta-galactosidase 8 [Vitis vinifera]
1014	1233	gi|225448904	121.88	13	5	1	Carbamidomethylation	48063	PREDICTED: malate dehydrogenase [NADP] chloroplastic [Vitis vinifera]
332	453	gi|225447576	121.72	31	6	1	Carbamidomethylation	28468	PREDICTED: chlorophyll a-b binding protein 151 chloroplastic [Vitis vinifera]
542	630	gi|526117351	121.62	13	7	1	Carbamidomethylation	56123	uncharacterized protein LOC100251487 [Vitis vinifera]
1171	791	gi|359483518	121.54	14	7	4	Carbamidomethylation	61477	PREDICTED: apoptosis inhibitor 5 isoform X1 [Vitis vinifera]
795	639	gi|731428049	121.52	11	4	2	Carbamidomethylation	47334	PREDICTED: delta-aminolevulinic acid dehydratase chloroplastic [Vitis vinifera]

861	678	gi|731422506	121.44	6	6	1	Carbamidomethylation	125112	PREDICTED: protein TOPLESS isoform X4 [Vitis vinifera]
945	652	gi|225444828	121.25	16	7	1	Carbamidomethylation	57127	PREDICTED: threonine synthase chloroplastic [Vitis vinifera]
1174	1666	gi|526117699	121.2	18	6	4	Carbamidomethylation	49406	3-dehydroquinate synthase-like [Vitis vinifera]
690	692	gi|731388721	121.04	25	5	3		18965	PREDICTED: translationally-controlled tumor protein homolog [Vitis vinifera]
1121	935	gi|225462922	120.85	14	5	1	Carbamidomethylation	46982	PREDICTED: 26S proteasome non-ATPase regulatory subunit 11 homolog [Vitis vinifera]
872	16957	gi|224075445	120.79	17	8	2	Carbamidomethylation	47494	transaldolase family protein [Populus trichocarpa]
833	17372	gi|224063299	120.77	12	7	1		67707	hypothetical protein POPTR_0002s10420g [Populus trichocarpa]
996	17268	gi|566211562	120.71	12	7	2	Carbamidomethylation	49984	hypothetical protein POPTR_0017s05490g [Populus trichocarpa]
1053	697	gi|731423169	120.67	35	5	5	Carbamidomethylation	13646	PREDICTED: 40S ribosomal protein S20-2 [Vitis vinifera]
706	17210	gi|224136368	120.63	6	7	2		107885	110 kDa 4SNc-Tudor domain family protein [Populus trichocarpa]
1350	17641	gi|566204499	120.49	14	4	4	Carbamidomethylation	47320	thioredoxin family protein [Populus trichocarpa]
1133	968	gi|731379157	120.33	10	6	2	Carbamidomethylation	83594	PREDICTED: LOW QUALITY PROTEIN: GMP synthase [glutamine-hydrolyzing] [Vitis vinifera]
721	17795	gi|566151913	120.21	14	8	2	Carbamidomethylation; Oxidation (M)	62800	D-3-phosphoglycerate dehydrogenase family protein [Populus trichocarpa]
817	875	gi|225452831	120.2	27	8	1	Carbamidomethylation	43750	PREDICTED: malate dehydrogenase chloroplastic-like [Vitis vinifera]
1062	17293	gi|566206565	120.15	18	5	5	Carbamidomethylation	39138	hypothetical protein POPTR_0015s08180g [Populus trichocarpa]

1584	17822	gi|224074207	120.06	13	3	3	Carbamidomethylation	46983	carbamoyl phosphate synthetase a family protein [Populus trichocarpa]
886	651	gi|225434329	120	16	9	1		57685	PREDICTED: trans-cinnamate 4-monooxygenase [Vitis vinifera]
784	16888	gi|224131368	119.94	17	7	4	Carbamidomethylation	48921	hypothetical protein POPTR_0014s13720g [Populus trichocarpa]
592	17014	gi|224140653	119.79	12	6	3	Carbamidomethylation; Oxidation (M)	61182	phosphoglycerate mutase family protein [Populus trichocarpa]
1234	17623	gi|224098483	119.56	22	5	1	Carbamidomethylation	39656	cytokinesis defective 1 family protein [Populus trichocarpa]
461	569	gi|225453215	119.49	28	9	2		23975	PREDICTED: 60S ribosomal protein L13-1 [Vitis vinifera]
525	17587	gi|566184208	119.48	16	4	1	Carbamidomethylation	27210	hypothetical protein POPTR_0008s15820g [Populus trichocarpa]
1016	832	gi|225461287	119.32	31	4	4	Carbamidomethylation	24076	PREDICTED: cytochrome b6-f complex iron-sulfur subunit 1 chloroplastic [Vitis vinifera]
374	493	gi|225436843	119.3	46	6	1		14570	PREDICTED: histone H2AX [Vitis vinifera]
1058	17711	gi|224122948	119.26	11	9	1		79226	inorganic pyrophosphatase family protein [Populus trichocarpa]
860	964	gi|225438529	119.11	22	6	6		27683	PREDICTED: probable ATP synthase 24 kDa subunit mitochondrial [Vitis vinifera]
1116	457	gi|225433588	119.08	9	6	3	Carbamidomethylation	90882	PREDICTED: probable methionine--tRNA ligase [Vitis vinifera]
1197	438	gi|225425318	119.05	8	6	2	Carbamidomethylation	108282	PREDICTED: DNA replication licensing factor MCM2 [Vitis vinifera]
1074	549	gi|225430366	119.02	7	7	2		108100	PREDICTED: chaperone protein ClpB4 mitochondrial [Vitis vinifera]
973	17225	gi|566211906	118.95	10	7	3		90312	dynamin-like family protein [Populus trichocarpa]
1154	758	gi|225430549	118.93	11	6	3	Carbamidomethylation	82761	PREDICTED: eukaryotic translation initiation factor 3 subunit B [Vitis vinifera]

598	1085	gi|225430422	118.54	19	5	1	Carbamidomethylation	17796	PREDICTED: 40S ribosomal protein S11 [Vitis vinifera]
1363	17552	gi|566205234	118.51	30	5	1		24533	hypothetical protein POPTR_0015s00570g [Populus trichocarpa]
483	17658	gi|224092113	118.36	10	5	2		40471	carbon-nitrogen hydrolase family protein [Populus trichocarpa]
864	454	gi|225449132	118.29	32	6	6		19768	PREDICTED: ATP synthase subunit d mitochondrial [Vitis vinifera]
895	1208	gi|225424530	118.24	13	3	1		36021	PREDICTED: eukaryotic translation initiation factor 3 subunit I [Vitis vinifera]
816	17625	gi|566204315	118.23	9	8	4	Carbamidomethylation	68355	hypothetical protein POPTR_0014s14690g [Populus trichocarpa]
689	654	gi|731415751	118.21	29	5	2	Carbamidomethylation	20904	PREDICTED: 60S ribosomal protein L18-2-like [Vitis vinifera]
1008	18208	gi|566210556	118.12	16	7	1		40209	putative protein phosphatase [Populus trichocarpa]
611	357	gi|731399677	117.99	8	8	1	Carbamidomethylation	102437	PREDICTED: protein argonaute 4A-like [Vitis vinifera]
946	18119	gi|224063066	117.9	11	6	1		56188	disulfide-isomerase family protein [Populus trichocarpa]
428	17510	gi|224078822	117.76	40	5	1		13770	hypothetical protein POPTR_0004s03100g [Populus trichocarpa]
531	642	gi|225432758	117.75	15	5	2	Carbamidomethylation	29774	PREDICTED: 40S ribosomal protein S3a-1 [Vitis vinifera]
712	629	gi|359492254	117.65	22	10	2	Carbamidomethylation	40876	PREDICTED: ferredoxin--NADP reductase leaf-type isozyme chloroplastic [Vitis vinifera]
953	1329	gi|731405046	117.52	11	6	6	Carbamidomethylation	86013	PREDICTED: DNA replication licensing factor MCM3 homolog 2 [Vitis vinifera]
548	17536	gi|566178862	117.47	17	6	5		34943	hypothetical protein POPTR_0007s00300g [Populus trichocarpa]
382	669	gi|731386337	117.45	23	5	4	Carbamidomethylation	20640	PREDICTED: serine/arginine-rich splicing factor RSZ22 isoform X2 [Vitis vinifera]

957	1264	gi|225442963	117.45	9	4	4		35176	PREDICTED: methyl-CpG-binding domain-containing protein 11 [Vitis vinifera]
1211	1022	gi|225456800	117.42	11	7	1		77878	PREDICTED: programmed cell death protein 4 [Vitis vinifera]
1199	17450	gi|566176147	117.41	14	7	3	Carbamidomethylation	41553	hypothetical protein POPTR_0006s13470g [Populus trichocarpa]
470	899	gi|225431269	117.34	12	4	2	Carbamidomethylation	36580	PREDICTED: peroxidase 72 [Vitis vinifera]
713	1005	gi|225435084	117.33	34	6	1		17444	PREDICTED: 60S ribosomal protein L23a [Vitis vinifera]
1399	17093	gi|224067984	117.11	14	6	3	Carbamidomethylation	68220	dynamin-like protein B [Populus trichocarpa]
1676	17459	gi|566206568	116.99	9	4	2	Carbamidomethylation	54924	Betaine-aldehyde dehydrogenase family protein [Populus trichocarpa]
1594	17583	gi|224137814	116.96	17	6	1	Carbamidomethylation	51194	eukaryotic translation initiation factor 3E family protein [Populus trichocarpa]
803	17342	gi|224077760	116.94	34	6	6	Carbamidomethylation	15880	40S ribosomal protein S19 [Populus trichocarpa]
1096	17698	gi|224058900	116.91	23	6	1	Carbamidomethylation	27383	Proteasome subunit alpha type 3 family protein [Populus trichocarpa]
751	619	gi|225453250	116.87	8	4	4		69341	PREDICTED: NAD-dependent malic enzyme 62 kDa isoform mitochondrial isoform X2 [Vitis vinifera]
831	551	gi|731440696	116.78	10	6	4		64402	PREDICTED: hsp70-Hsp90 organizing protein 3-like [Vitis vinifera]
1380	1009	gi|359480225	116.65	17	4	1	Carbamidomethylation	27397	PREDICTED: peptidyl-prolyl cis-trans isomerase [Vitis vinifera]
1207	1093	gi|225438067	116.6	11	4	1	Carbamidomethylation	45402	PREDICTED: succinyl-CoA ligase [ADP-forming] subunit beta mitochondrial [Vitis vinifera]
1055	1087	gi|731394579	116.6	23	5	5	Carbamidomethylation	24605	PREDICTED: 60S ribosomal protein L10a-1-like [Vitis vinifera]
735	17345	gi|224075876	116.59	26	6	1	Carbamidomethylation	20527	peptidyl-prolyl cis-trans isomerase family protein [Populus trichocarpa]

927	17254	gi|224075058	116.56	18	6	1	Carbamidomethylation	53712	6-phosphogluconate dehydrogenase family protein [Populus trichocarpa]
398	1257	gi|225430939	116.5	25	7	6	Carbamidomethylation	33557	PREDICTED: isopentenyl-diphosphate Delta-isomerase I [Vitis vinifera]
1173	17769	gi|566192876	116.43	17	6	2	Carbamidomethylation	47035	hypothetical protein POPTR_0010s26170g [Populus trichocarpa]
929	17681	gi|224140109	116.38	34	7	2		17267	hypothetical protein POPTR_0016s08070g [Populus trichocarpa]
1052	903	gi|225453158	116.37	16	5	2	Carbamidomethylation	41234	PREDICTED: probable fructokinase-6 chloroplastic [Vitis vinifera]
883	17487	gi|566148625	116.33	10	4	1	Carbamidomethylation	54744	hypothetical protein POPTR_0001s12690g [Populus trichocarpa]
1571	1117	gi|225455336	116.3	10	5	2	Carbamidomethylation	96439	PREDICTED: importin subunit beta-1 [Vitis vinifera]
1304	17697	gi|566213932	116.25	10	7	3		82500	Eukaryotic translation initiation factor 3 subunit 9 family protein [Populus trichocarpa]
1339	597	gi|225441924	116.18	15	5	1	Carbamidomethylation	51126	PREDICTED: eukaryotic translation initiation factor 3 subunit E [Vitis vinifera]
681	17370	gi|566192956	116.13	20	6	1	Carbamidomethylation	44225	hypothetical protein POPTR_0011s00650g [Populus trichocarpa]
1009	17548	gi|224139168	116.02	9	4	3	Carbamidomethylation; Oxidation (M)	65070	pyruvate decarboxylase family protein [Populus trichocarpa]
879	743	gi|359482848	116	10	4	2		43630	PREDICTED: nucleosome assembly protein 1;4 [Vitis vinifera]
1098	17046	gi|224085984	115.99	10	5	3	Carbamidomethylation	61382	multi-copper oxidase type 1 family protein [Populus trichocarpa]
1291	17602	gi|566191545	115.99	16	6	2	Carbamidomethylation	40561	hypothetical protein POPTR_0010s18770g [Populus trichocarpa]
1531	17393	gi|566151113	115.74	9	5	1		76209	hypothetical protein POPTR_0001s26420g [Populus trichocarpa]

710	766	gi|225436215	115.66	12	6	3	Carbamidomethylation	70115	PREDICTED: lysine--tRNA ligase-like [Vitis vinifera]
1201	910	gi|225449380	115.58	17	5	1	Carbamidomethylation	39535	PREDICTED: mannose-1-phosphate guanylyltransferase 1 [Vitis vinifera]
628	17918	gi|224063643	115.52	25	5	1	Carbamidomethylation	17863	40S ribosomal protein S11 [Populus trichocarpa]
1021	1517	gi|225445883	115.32	27	4	1	Carbamidomethylation	14777	PREDICTED: 40S ribosomal protein S12 [Vitis vinifera]
614	17566	gi|566254203	115.26	36	7	1		17971	hypothetical protein POPTR_1064s00200g [Populus trichocarpa]
1287	17346	gi|224138722	115.18	20	5	1		39574	isocitrate dehydrogenase family protein [Populus trichocarpa]
1205	2521	gi|225453076	115.18	9	5	5	Carbamidomethylation	80919	PREDICTED: NADH dehydrogenase [ubiquinone] iron-sulfur protein 1 mitochondrial [Vitis vinifera]
1424	823	gi|225445396	115.1	23	6	1	Carbamidomethylation	37783	PREDICTED: serine-threonine kinase receptor-associated protein [Vitis vinifera]
1566	17913	gi|224065745	115.09	6	4	1	Carbamidomethylation	102963	putative translation initiation family protein [Populus trichocarpa]
616	17211	gi|224135485	114.94	10	5	1	Carbamidomethylation	58870	glycosyl hydrolase family 1 family protein [Populus trichocarpa]
1209	591	gi|225456270	114.92	11	6	2	Carbamidomethylation	62432	PREDICTED: nucleolar protein 56-like [Vitis vinifera]
615	17300	gi|566213629	114.82	51	5	1		14301	histone H2A family protein [Populus trichocarpa]
752	17352	gi|566156897	114.8	14	5	3	Carbamidomethylation	46943	Delta-aminolevulinic acid dehydratase family protein [Populus trichocarpa]
708	380	gi|225459292	114.56	27	6	2		28739	PREDICTED: 14-3-3 protein 7 [Vitis vinifera]
764	463	gi|731398611	114.52	20	9	1	Carbamidomethylation	43372	PREDICTED: proliferation-associated protein 2G4 [Vitis vinifera]
943	1115	gi|731378460	114.45	18	5	2		38028	PREDICTED: UDP-glucose 4-epimerase GEPI48 [Vitis vinifera]

1040	576	gi|91984005	114.42	18	4	4		35318	cytochrome f (chloroplast) [Vitis vinifera]
1079	17344	gi|224125422	114.33	8	5	3	Carbamidomethylation	56543	hypothetical protein POPTR_0013s03070g [Populus trichocarpa]
1500	1094	gi|225461050	114.31	15	5	5	Carbamidomethylation	49698	PREDICTED: tryptophan synthase beta chain 1 [Vitis vinifera]
776	17757	gi|566179485	114.28	12	5	1	Carbamidomethylation	41133	alcohol-dehydrogenase family protein [Populus trichocarpa]
914	816	gi|526118026	114.1	18	6	4	Carbamidomethylation	55487	5-enolpyruvylshikimate-3-phosphate synthase [Vitis vinifera]
1020	646	gi|225456268	114.05	20	5	2	Carbamidomethylation	26974	PREDICTED: adenylate kinase 4 [Vitis vinifera]
977	812	gi|731423236	114.03	10	5	1		65721	PREDICTED: monocopper oxidase-like protein SKU5 [Vitis vinifera]
1175	747	gi|731409481	113.99	27	4	4		26284	PREDICTED: carboxymethylenebutenolidase homolog [Vitis vinifera]
1568	17817	gi|566160765	113.8	12	5	1		58378	hypothetical protein POPTR_0003s04140g [Populus trichocarpa]
780	17513	gi|225428380	113.74	10	6	5	Carbamidomethylation	70298	PREDICTED: SUMO-activating enzyme subunit 2 isoform X1 [Vitis vinifera]
431	427	gi|731399148	113.68	50	6	6		14802	PREDICTED: 40S ribosomal protein S15a [Vitis vinifera]
933	17873	gi|224058705	113.64	29	3	3	Carbamidomethylation	18641	cytochrome c oxidase-related family protein [Populus trichocarpa]
1331	716	gi|225448823	113.61	4	5	2		134756	PREDICTED: splicing factor 3B subunit 3-like [Vitis vinifera]
853	2170	gi|225425388	113.58	54	4	4	Carbamidomethylation	13594	PREDICTED: nuclear transport factor 2 [Vitis vinifera]
880	17749	gi|566170400	113.51	10	4	1		42882	hypothetical protein POPTR_0005s09680g [Populus trichocarpa]
881	391	gi|225425280	113.43	19	8	2		48103	PREDICTED: uncharacterized protein LOC100247241 [Vitis vinifera]

1644	17649	gi|566186484	113.42	11	5	1	Carbamidomethylation; Oxidation (M)	77490	hypothetical protein POPTR_0009s05730g [Populus trichocarpa]
648	17449	gi|224078584	113.19	15	4	2		33150	glyoxalase I homolog family protein [Populus trichocarpa]
1663	17624	gi|224104329	113.1	8	4	1		96949	alpha-glucan phosphorylase family protein [Populus trichocarpa]
1473	759	gi|731412750	113.1	10	4	4		62116	PREDICTED: adenylosuccinate lyase [Vitis vinifera]
785	526	gi|225448739	113.1	13	6	2		69044	PREDICTED: leukotriene A-4 hydrolase homolog [Vitis vinifera]
1162	19584	gi|566204607	113.02	13	6	3		52568	hypothetical protein POPTR_0014s16480g [Populus trichocarpa]
1344	16899	gi|566190691	112.99	9	4	1	Carbamidomethylation	65588	Ser/Thr specific protein phosphatase 2A A regulatory subunit alpha isoform [Populus trichocarpa]
1443	18265	gi|566189013	112.98	22	4	4	Carbamidomethylation; Oxidation (M)	30317	glycine-rich family protein [Populus trichocarpa]
1198	17963	gi|224125250	112.97	7	5	5	Carbamidomethylation	110348	glycoside hydrolase family 2 family protein [Populus trichocarpa]
685	17341	gi|566238007	112.96	12	3	1	Carbamidomethylation	30925	Chlorophyll a-b binding protein CP26 [Populus trichocarpa]
921	687	gi|359490482	112.74	18	5	2	Carbamidomethylation	33398	PREDICTED: probable mediator of RNA polymerase II transcription subunit 36b [Vitis vinifera]
897	1376	gi|225438619	112.64	36	4	1	Carbamidomethylation	12309	PREDICTED: 60S ribosomal protein L30 [Vitis vinifera]
1292	17738	gi|224066507	112.64	13	6	1	Carbamidomethylation	43683	mRNA-binding family protein [Populus trichocarpa]
618	927	gi|731440185	112.63	13	4	2		30189	PREDICTED: 60S ribosomal protein L5-like [Vitis vinifera]
1495	957	gi|731389741	112.53	9	5	1	Carbamidomethylation	69804	PREDICTED: probable methyltransferase PMT3 [Vitis vinifera]
1779	17786	gi|566209837	112.49	14	5	2	Carbamidomethylation	54725	Signal recognition particle 54 kDa protein 1 [Populus trichocarpa]
1516	28886	gi|224113185	112.48	13	5	1	Carbamidomethylation	57926	hypothetical protein POPTR_0010s23940g [Populus trichocarpa]

930	17374	gi|566147333	112.48	5	5	3		100058	hypothetical protein POPTR_0001s05320g [Populus trichocarpa]
1041	1487	gi|225460716	112.37	10	4	2		49588	PREDICTED: dihydrolipoyllysine-residue acetyltransferase component 5 of pyruvate dehydrogenase complex chloroplastic [Vitis vinifera]
761	774	gi|225444063	112.34	13	5	2	Carbamidomethylation	44423	PREDICTED: obg-like ATPase 1 [Vitis vinifera]
1288	499	gi|731416612	112.33	12	5	5		63619	PREDICTED: probable nucleolar protein 5-2 [Vitis vinifera]
1342	1205	gi|225443286	112.23	11	4	2	Carbamidomethylation	43933	PREDICTED: pyruvate dehydrogenase E1 component subunit alpha mitochondrial [Vitis vinifera]
1222	325	gi|225456051	112.12	9	4	2	Carbamidomethylation	57589	PREDICTED: T-complex protein 1 subunit delta [Vitis vinifera]
1365	907	gi|731395413	112.06	18	4	3	Carbamidomethylation	31798	PREDICTED: bifunctional protein FolD 2 [Vitis vinifera]
842	729	gi|731421236	112.04	17	6	2	Carbamidomethylation	37446	PREDICTED: arginase 1 mitochondrial [Vitis vinifera]
955	18534	gi|224063774	112.02	13	6	4	Carbamidomethylation	55604	3-phosphoshikimate 1-carboxyvinyltransferase family protein [Populus trichocarpa]
1115	17785	gi|566149444	111.86	13	6	6		54813	aldehyde dehydrogenase family 7 member a1 turgor responsive family protein [Populus trichocarpa]
1312	1156	gi|225436289	111.68	22	5	1	Oxidation (M)	43082	PREDICTED: probable aldo-keto reductase 2 isoform X1 [Vitis vinifera]
1064	17196	gi|224121218	111.61	18	5	1	Carbamidomethylation	36046	GTP-binding protein beta chain [Populus trichocarpa]
1226	1310	gi|731401385	111.61	7	3	3		36064	PREDICTED: uncharacterized protein At5g02240 [Vitis vinifera]
1278	18379	gi|566182095	111.48	15	6	2	Carbamidomethylation	48288	hypothetical protein POPTR_0008s03160g [Populus trichocarpa]

1768	735	gi|225455902	111.39	8	3	2	Carbamidomethylation	65334	PREDICTED: serine/threonine-protein phosphatase 2A 65 kDa regulatory subunit A beta isoform [Vitis vinifera]
965	1215	gi|225462161	111.38	12	4	4	Carbamidomethylation	53484	PREDICTED: NADH dehydrogenase [ubiquinone] flavoprotein 1 mitochondrial isoform X2 [Vitis vinifera]
1609	19577	gi|224089368	111.35	7	3	1		73326	endomembrane protein 70 [Populus trichocarpa]
1104	17882	gi|566163268	111.23	35	5	1	Carbamidomethylation	14720	hypothetical protein POPTR_0003s17960g [Populus trichocarpa]
755	710	gi|731382436	111.14	18	4	1		35672	PREDICTED: LOW QUALITY PROTEIN: isoflavone reductase-like protein [Vitis vinifera]
1414	18242	gi|566214941	111.12	8	7	2	Carbamidomethylation	107829	elongation factor Ts family protein [Populus trichocarpa]
827	17620	gi|224058427	111.04	15	5	2		28418	reticulon family protein [Populus trichocarpa]
992	809	gi|225445206	110.95	18	6	1	Carbamidomethylation	45116	PREDICTED: aspartate aminotransferase cytoplasmic [Vitis vinifera]
1604	17821	gi|566182683	110.94	17	4	4		34990	hypothetical protein POPTR_0008s06540g [Populus trichocarpa]
1289	17577	gi|566188043	110.87	21	5	1	Oxidation (M)	43097	hypothetical protein POPTR_0009s14830g [Populus trichocarpa]
1159	17715	gi|566158632	110.83	15	5	1	Carbamidomethylation; Oxidation (M)	48694	hypothetical protein POPTR_0002s18020g [Populus trichocarpa]
850	787	gi|225450299	110.81	11	5	5		52389	PREDICTED: citrate synthase mitochondrial [Vitis vinifera]
768	1297	gi|359489218	110.77	6	3	3	Carbamidomethylation	90315	PREDICTED: nucleolin [Vitis vinifera]
924	17954	gi|224115436	110.77	26	5	1		21917	GA family protein [Populus trichocarpa]

1426	17767	gi|566157737	110.73	7	5	3	Carbamidomethylation	79950	hypothetical protein POPTR_0002s13010g [Populus trichocarpa]
1235	17431	gi|566176019	110.43	19	5	1		39252	isocitrate dehydrogenase family protein [Populus trichocarpa]
1293	17458	gi|224105455	110.43	10	4	3	Carbamidomethylation	51957	chloroplast biogenesis family protein [Populus trichocarpa]
1118	605	gi|731399856	110.34	4	3	1		104341	PREDICTED: villin-2 [Vitis vinifera]
1063	662	gi|225447135	110.34	55	5	2	Carbamidomethylation	11288	PREDICTED: small ubiquitin-related modifier 1 [Vitis vinifera]
1307	1325	gi|731411781	110.29	14	4	4		25284	PREDICTED: manganese superoxide dismutase isoform X1 [Vitis vinifera]
1091	17240	gi|566202791	110.12	11	5	4	Carbamidomethylation	59276	t-complex polypeptide 1 family protein [Populus trichocarpa]
1505	28906	gi|731410313	110	9	5	2		75856	PREDICTED: transmembrane 9 superfamily member 12 [Vitis vinifera]
590	17412	gi|566189935	109.78	11	4	1	Carbamidomethylation	29602	ribosomal protein S3a [Populus trichocarpa]
1101	17901	gi|566170058	109.77	10	6	6	Carbamidomethylation	65727	hypothetical protein POPTR_0005s07720g [Populus trichocarpa]
793	718	gi|225465809	109.62	24	6	2	Carbamidomethylation	20492	PREDICTED: peptidyl-prolyl cis-trans isomerase CYP22 [Vitis vinifera]
862	769	gi|359488553	109.57	5	5	1		115409	PREDICTED: alpha-mannosidase isoform X2 [Vitis vinifera]
968	17557	gi|566168605	109.53	13	5	1		44259	hypothetical protein POPTR_0005s00670g [Populus trichocarpa]
459	17575	gi|566170929	109.48	10	3	1	Carbamidomethylation	36394	hypothetical protein POPTR_0005s12070g [Populus trichocarpa]
961	975	gi|359494701	109.35	9	6	1	Oxidation (M)	91552	PREDICTED: dynamin-related protein 3B isoform X1 [Vitis vinifera]

1544	18314	gi|224088282	109.19	7	3	1		73935	hypothetical protein POPTR_0006s19780g [Populus trichocarpa]
1353	1838	gi|225440564	109.12	11	3	3		44667	PREDICTED: fructose-1 6-bisphosphatase chloroplastic-like [Vitis vinifera]
1131	1086	gi|731399561	108.93	3	5	4		187436	PREDICTED: transcription elongation factor SPT6 [Vitis vinifera]
138	955	gi|225465423	108.9	8	5	4	Carbamidomethylation	53142	PREDICTED: uncharacterized protein LOC100251726 [Vitis vinifera]
789	336	gi|225429870	108.88	15	8	8	Carbamidomethylation	60861	PREDICTED: aspartate--tRNA ligase cytoplasmic [Vitis vinifera]
737	17164	gi|566147610	108.83	24	6	1		20417	hypothetical protein POPTR_0001s06720g [Populus trichocarpa]
887	17242	gi|566182348	108.82	13	6	2	Carbamidomethylation	70505	peptidase M1 family protein [Populus trichocarpa]
1286	1064	gi|225462285	108.81	15	7	3		52600	PREDICTED: inosine-5'-monophosphate dehydrogenase [Vitis vinifera]
453	878	gi|225452827	108.78	34	5	5		13746	PREDICTED: 60S ribosomal protein L34 [Vitis vinifera]
1156	17408	gi|566192263	108.72	17	6	6		47039	chorismate synthase family protein [Populus trichocarpa]
719	633	gi|225444649	108.61	13	3	3		26396	PREDICTED: 20 kDa chaperonin chloroplastic [Vitis vinifera]
971	1305	gi|225451833	108.6	19	5	1		29200	PREDICTED: 60S ribosomal protein L7a [Vitis vinifera]
997	17124	gi|566259876	108.58	24	5	1		23976	hypothetical protein POPTR_0022s00350g [Populus trichocarpa]
1108	16945	gi|224090986	108.58	20	5	4	Carbamidomethylation	31176	chlorophyll a/b binding protein CP29 [Populus trichocarpa]
1127	17927	gi|566171321	108.57	17	4	1		33667	pfkB-type carbohydrate kinase family protein [Populus trichocarpa]
948	794	gi|731374652	108.57	12	5	2		58335	PREDICTED: aldehyde dehydrogenase family 2 member B4 mitochondrial [Vitis vinifera]

1178	18655	gi|566163024	108.37	12	3	1		39970	hypothetical protein POPTR_0003s16480g [Populus trichocarpa]
1406	17463	gi|224133304	108.25	12	6	6	Carbamidomethylation	60007	hypothetical protein POPTR_0015s07690g [Populus trichocarpa]
791	16841	gi|669099987	108.21	9	8	7	Carbamidomethylation	83116	photosystem I P700 chlorophyll A apoprotein A1 (chloroplast) [Camellia crapnelliana]
1683	1982	gi|731402641	108.04	21	4	2		35414	PREDICTED: cytochrome c1-2 heme protein mitochondrial-like isoform X3 [Vitis vinifera]
1034	17567	gi|224071575	108.02	10	5	2	Carbamidomethylation	65504	stress inducible family protein [Populus trichocarpa]
1215	18056	gi|224089509	108	26	4	4		23148	hypothetical protein POPTR_0006s00360g [Populus trichocarpa]
1279	3000	gi|225453620	107.95	16	5	1	Carbamidomethylation; Oxidation (M)	47761	PREDICTED: pyruvate dehydrogenase E1 component subunit alpha-3 chloroplastic [Vitis vinifera]
935	18315	gi|566200085	107.93	5	5	1	Carbamidomethylation	125013	hypothetical protein POPTR_0013s08030g [Populus trichocarpa]
703	1103	gi|731398322	107.88	10	6	1	Carbamidomethylation	68750	PREDICTED: lysosomal beta glucosidase-like isoform X2 [Vitis vinifera]
1674	18050	gi|566187644	107.8	8	5	1	Carbamidomethylation	91239	hypothetical protein POPTR_0009s12440g [Populus trichocarpa]
1346	17381	gi|566198861	107.74	24	6	5	Carbamidomethylation	29461	hypothetical protein POPTR_0013s01700g [Populus trichocarpa]
1427	986	gi|225440013	107.7	18	5	2		40309	PREDICTED: isocitrate dehydrogenase [NAD] catalytic subunit 5 mitochondrial [Vitis vinifera]
503	784	gi|225462809	107.58	8	4	4		60994	PREDICTED: 2-hydroxyacyl-CoA lyase [Vitis vinifera]

1444	1903	gi|225440898	107.51	13	4	1		51575	PREDICTED: cell division protein FtsZ homolog 2-1 chloroplastic [Vitis vinifera]
1621	17504	gi|224062301	107.34	16	4	1	Carbamidomethylation	38139	hypothetical protein POPTR_0002s04790g [Populus trichocarpa]
1743	952	gi|225438095	107.17	9	5	1	Carbamidomethylation	70388	PREDICTED: probable methyltransferase PMT8 [Vitis vinifera]
1003	18658	gi|224117530	107.1	14	3	1		21810	endoribonuclease L-PSP family protein [Populus trichocarpa]
1039	17574	gi|566178300	106.94	13	6	2	Carbamidomethylation	45519	hypothetical protein POPTR_0006s26630g [Populus trichocarpa]
979	17819	gi|224131818	106.91	24	4	1		28513	chlorophyll a/b-binding family protein [Populus trichocarpa]
916	913	gi|225425595	106.89	12	4	1	Carbamidomethylation	46628	PREDICTED: ATP-citrate synthase alpha chain protein 1 [Vitis vinifera]
1359	1127	gi|731397334	106.84	8	4	3		73259	PREDICTED: apoptotic chromatin condensation inducer in the nucleus isoform X2 [Vitis vinifera]
1214	17231	gi|224087752	106.81	13	4	4	Carbamidomethylation	33055	pyridoxin biosynthesis PDX1-like protein 3 [Populus trichocarpa]
1117	1159	gi|225432366	106.72	5	5	5		125073	PREDICTED: putative chromatin-remodeling complex ATPase chain [Vitis vinifera]
1200	17475	gi|224107361	106.63	5	6	1		128813	ADP-forming family protein [Populus trichocarpa]
1433	2484	gi|225465947	106.53	7	3	3		52209	PREDICTED: uridine 5'-monophosphate synthase [Vitis vinifera]
1369	2050	gi|731393849	106.44	6	4	3	Carbamidomethylation	56128	PREDICTED: UBP1-associated protein 2A [Vitis vinifera]
1184	18199	gi|566185810	106.4	6	5	1		94037	beta-galactosidase family protein [Populus trichocarpa]
1264	1237	gi|225446509	106.4	7	4	3	Carbamidomethylation	77096	PREDICTED: acyl-coenzyme A oxidase 3 peroxisomal [Vitis vinifera]

1152	17996	gi|566203263	106.39	6	5	5		91348	hypothetical protein POPTR_0014s08590g [Populus trichocarpa]
1373	17911	gi|566203837	106.18	8	3	3		39770	DNAJ heat shock family protein [Populus trichocarpa]
966	1238	gi|225425166	106.12	12	3	1		39489	PREDICTED: pyruvate dehydrogenase E1 component subunit beta-1 mitochondrial [Vitis vinifera]
797	17869	gi|566168995	106.1	22	3	1		19031	callus protein P23 [Populus trichocarpa]
903	430	gi|731386341	106.06	9	6	1	Carbamidomethylation	74377	PREDICTED: trifunctional UDP-glucose 4 6-dehydratase/UDP-4-keto-6-deoxy-D-glucose 3 5-epimerase/UDP-4-keto-L-rhamnose-reductase RHM1 [Vitis vinifera]
1429	1174	gi|225426166	106.05	16	5	2	Carbamidomethylation	42682	PREDICTED: mitogen-activated protein kinase homolog MMK2 [Vitis vinifera]
1757	1742	gi|731436287	106.03	4	3	3	Carbamidomethylation	96261	PREDICTED: histidine--tRNA ligase [Vitis vinifera]
801	17266	gi|224104631	106.02	21	6	1		27319	cytosolic ascorbate peroxidase family protein [Populus trichocarpa]
1186	1106	gi|225435632	105.95	7	5	1	Carbamidomethylation	67979	PREDICTED: formate--tetrahydrofolate ligase [Vitis vinifera]
1724	1234	gi|731390743	105.95	15	4	1		40016	PREDICTED: PTI1-like tyrosine-protein kinase 3 isoform X2 [Vitis vinifera]
1001	1605	gi|225460977	105.93	17	4	2	Carbamidomethylation	20368	PREDICTED: reactive Intermediate Deaminase A chloroplastic [Vitis vinifera]
1135	18650	gi|566159091	105.8	24	4	1	Carbamidomethylation	20543	hypothetical protein POPTR_0002s20400g [Populus trichocarpa]
1306	813	gi|225425356	105.73	15	4	4		32651	PREDICTED: U2 small nuclear ribonucleoprotein A' [Vitis vinifera]
1243	17916	gi|566182153	105.66	9	3	3	Carbamidomethylation	57417	Serine carboxypeptidase precursor family protein [Populus trichocarpa]

1157	17332	gi|566165023	105.57	11	6	3	Carbamidomethylation	60040	hypothetical protein POPTR_0004s05160g [Populus trichocarpa]
995	18399	gi|566174518	105.5	9	2	1	Carbamidomethylation	40168	hypothetical protein POPTR_0006s04880g [Populus trichocarpa]
1217	1290	gi|225453909	105.38	17	3	3	Carbamidomethylation	24628	PREDICTED: proteasome subunit beta type-1 [Vitis vinifera]
1341	17514	gi|566170365	105.28	12	4	2	Carbamidomethylation	39182	hypothetical protein POPTR_0005s09400g partial [Populus trichocarpa]
1645	856	gi|225454422	105.18	28	4	4		16418	PREDICTED: eukaryotic translation initiation factor 1A [Vitis vinifera]
1212	1387	gi|225427288	105.14	10	5	1		68795	PREDICTED: dynamin-related protein 1E [Vitis vinifera]
1474	18307	gi|224113315	105.08	9	6	1		66874	hypothetical protein POPTR_0010s25600g [Populus trichocarpa]
667	807	gi|225461209	104.89	23	4	2		21740	PREDICTED: NAD(P)H dehydrogenase (quinone) FQR1 [Vitis vinifera]
1582	577	gi|731438110	104.83	6	4	4		88481	PREDICTED: probable cytosolic oligopeptidase A [Vitis vinifera]
1833	1979	gi|731436817	104.82	12	5	5		49774	PREDICTED: cysteine desulfurase 1 mitochondrial-like [Vitis vinifera]
956	17598	gi|566158065	104.75	12	5	1		37108	arginase family protein [Populus trichocarpa]
1415	17782	gi|224143559	104.7	14	5	1	Carbamidomethylation	44475	aspartate aminotransferase family protein [Populus trichocarpa]
1176	19132	gi|224073194	104.58	7	5	1	Carbamidomethylation	67506	10-formyltetrahydrofolate synthetase family protein [Populus trichocarpa]
1782	18332	gi|224107655	104.53	16	3	1		33896	3-oxoacyl-[acyl-carrier-protein] reductase [Populus trichocarpa]
1452	17990	gi|224072588	104.52	12	4	4	Carbamidomethylation	30737	hypothetical protein POPTR_0003s17100g partial [Populus trichocarpa]
1416	17448	gi|566149991	104.46	11	5	1	Carbamidomethylation	47475	hypothetical protein POPTR_0001s20510g [Populus trichocarpa]

1596	17500	gi|224107863	104.45	11	4	2		43257	pyruvate dehydrogenase family protein [Populus trichocarpa]
1501	1158	gi|225462270	104.37	20	5	5	Carbamidomethylation	31886	PREDICTED: 2-dehydro-3-deoxyphosphooctonate aldolase [Vitis vinifera]
1385	1541	gi|225453754	103.91	15	2	2	Carbamidomethylation	23373	PREDICTED: probable xyloglucan endotransglucosylase/hydrolase protein 19 [Vitis vinifera]
1086	781	gi|225436672	103.83	8	4	1	Carbamidomethylation	56836	PREDICTED: flavonoid 3' 5'-hydroxylase 2 [Vitis vinifera]
683	675	gi|225442801	103.81	51	5	1		14493	PREDICTED: probable histone H2A variant 3 [Vitis vinifera]
1011	811	gi|225456404	103.71	9	3	1	Carbamidomethylation	50437	PREDICTED: glutamate-1-semialdehyde 2 1-aminomutase chloroplastic [Vitis vinifera]
1407	28873	gi|566186314	103.68	10	6	5	Carbamidomethylation	70914	hypothetical protein POPTR_0009s04780g [Populus trichocarpa]
1576	2154	gi|225458519	103.56	5	3	1	Carbamidomethylation	75018	PREDICTED: uncharacterized protein LOC100267853 [Vitis vinifera]
1730	854	gi|731402959	103.52	11	3	1		35210	PREDICTED: probable quinone oxidoreductase [Vitis vinifera]
699	17320	gi|566189392	103.24	18	6	6		27258	hypothetical protein POPTR_0010s06320g [Populus trichocarpa]
1230	18027	gi|566197725	103.14	8	3	1		56765	hypothetical protein POPTR_0012s10030g [Populus trichocarpa]
1673	17804	gi|566168414	103.13	13	3	1		34495	quinone oxidoreductase-like family protein [Populus trichocarpa]
1090	17447	gi|566185959	103.08	23	6	1	Carbamidomethylation; Oxidation (M)	27367	Proteasome subunit alpha type 3 family protein [Populus trichocarpa]
1729	2586	gi|225431100	102.97	10	3	3	Carbamidomethylation	42836	PREDICTED: 26S proteasome non-ATPase regulatory subunit 4 homolog [Vitis vinifera]
686	867	gi|225451645	102.94	44	3	3	Carbamidomethylation	9595	PREDICTED: 40S ribosomal protein S27-2-like [Vitis vinifera]
1567	17508	gi|566212181	102.82	6	4	1	Carbamidomethylation	90000	methionyl-tRNA synthetase family protein [Populus trichocarpa]

1496	848	gi|225427017	102.77	14	5	5		37604	PREDICTED: L-lactate dehydrogenase A isoform X2 [Vitis vinifera]
1007	16954	gi|224076908	102.63	16	6	1		40750	hypothetical protein POPTR_0004s06400g [Populus trichocarpa]
283	987	gi|731411833	102.44	14	3	2	Carbamidomethylation	35859	PREDICTED: nitrilase-like protein 2 [Vitis vinifera]
1237	746	gi|225447041	102.36	6	3	1	Carbamidomethylation	60008	PREDICTED: L-ascorbate oxidase homolog [Vitis vinifera]
972	17585	gi|224133188	102.32	19	5	2	Carbamidomethylation	35890	GTP-binding protein beta chain [Populus trichocarpa]
910	1369	gi|526117665	102.21	12	4	1	Carbamidomethylation	41194	alcohol dehydrogenase 2 [Vitis vinifera]
1598	1135	gi|225458305	102.07	9	4	3		62648	PREDICTED: glucose-6-phosphate isomerase cytosolic [Vitis vinifera]
804	1281	gi|359494275	102.03	13	5	1	Carbamidomethylation	25431	PREDICTED: probable glutathione S-transferase parC [Vitis vinifera]
1167	18339	gi|225462348	102.01	8	3	3	Carbamidomethylation	57728	PREDICTED: zinc finger CCCH domain-containing protein 37 isoform X2 [Vitis vinifera]
1195	1179	gi|225437618	102	5	2	2	Carbamidomethylation	82698	PREDICTED: probable pre-mRNA-splicing factor ATP-dependent RNA helicase [Vitis vinifera]
1242	18064	gi|566149112	102	20	4	2	Carbamidomethylation	22610	GTP-binding family protein [Populus trichocarpa]
1586	1415	gi|225437227	101.96	10	5	5		60053	PREDICTED: glutathione reductase chloroplastic [Vitis vinifera]
1575	805	gi|225432846	101.95	18	4	1	Carbamidomethylation	38533	PREDICTED: 2-methyl-6-phytyl-1 4-hydroquinone methyltransferase chloroplastic [Vitis vinifera]
1402	2892	gi|359474491	101.93	4	4	4	Carbamidomethylation	138987	PREDICTED: regulator of nonsense transcripts 1 homolog isoform X2 [Vitis vinifera]
1946	2190	gi|225442910	101.92	11	4	1	Carbamidomethylation	55294	PREDICTED: signal recognition particle 54 kDa protein 2 [Vitis vinifera]
750	1639	gi|526118028	101.91	16	6	3		30741	aquaporin-like [Vitis vinifera]
1270	18130	gi|566160621	101.89	13	5	4		55240	DEAD box RNA helicase family protein [Populus trichocarpa]

1102	537	gi|225465200	101.81	25	5	1		23863	PREDICTED: ras-related protein RABA2a [Vitis vinifera]
1502	28880	gi|225462066	101.78	12	4	1		45076	PREDICTED: DNA damage-inducible protein 1 [Vitis vinifera]
560	17506	gi|566243399	101.65	13	4	1		33846	hypothetical protein POPTR_0019s13040g [Populus trichocarpa]
962	17523	gi|224107106	101.64	9	6	1	Carbamidomethylation	79571	MULTIFUNCTIONAL family protein [Populus trichocarpa]
975	17759	gi|224107999	101.61	31	6	1		23112	Proteasome subunit beta type 2-2 family protein [Populus trichocarpa]
976	887	gi|359479647	101.5	27	6	1		22491	PREDICTED: proteasome subunit beta type-2-A [Vitis vinifera]
1535	1573	gi|731428130	101.34	10	3	1		49925	PREDICTED: kynurenine--oxoglutarate transaminase 1 [Vitis vinifera]
1216	1864	gi|731404286	101.31	10	4	4		51076	PREDICTED: dihydrolipoyllysine-residue succinyltransferase component of 2-oxoglutarate dehydrogenase complex 1 mitochondrial [Vitis vinifera]
1506	1989	gi|731417792	101.29	6	3	3	Carbamidomethylation	72111	PREDICTED: KH domain-containing protein At4g18375 [Vitis vinifera]
1132	17511	gi|566203118	101.2	25	7	7	Carbamidomethylation	27871	phosphomannomutase family protein [Populus trichocarpa]
1508	1593	gi|359497369	101.17	7	3	3	Carbamidomethylation	64520	PREDICTED: eukaryotic translation initiation factor 3 subunit D-like [Vitis vinifera]
1142	17694	gi|224059670	101.06	19	4	2		25980	60S ribosomal protein L6 [Populus trichocarpa]
411	17645	gi|566146919	101.05	7	5	1		68964	hypothetical protein POPTR_0001s03040g [Populus trichocarpa]
1503	18083	gi|566159452	101.02	9	4	1		75110	hypothetical protein POPTR_0002s22560g [Populus trichocarpa]
1314	17589	gi|224055291	101.01	8	4	1		56959	hypothetical protein POPTR_0001s28140g [Populus trichocarpa]

1442	17614	gi|224054837	100.97	23	5	3	Carbamidomethylation	23819	hypothetical protein POPTR_0001s24290g [Populus trichocarpa]
1056	423	gi|225434133	100.94	15	5	2	Carbamidomethylation	40613	PREDICTED: cysteine synthase chloroplastic/chromoplastic [Vitis vinifera]
1534	864	gi|731439997	100.5	15	5	1	Carbamidomethylation	33522	PREDICTED: phosphoglycolate phosphatase 1B chloroplastic [Vitis vinifera]
926	1505	gi|731426023	100.27	30	2	1		12464	PREDICTED: protein SPIRAL1-like 1 isoform X2 [Vitis vinifera]
1081	885	gi|225449290	100.2	19	4	1		17051	PREDICTED: 18.2 kDa class I heat shock protein [Vitis vinifera]
925	18472	gi|566162504	100.16	52	4	3	Carbamidomethylation	11964	hypothetical protein POPTR_0003s13600g [Populus trichocarpa]
1390	1632	gi|225447707	100.14	13	3	3	Carbamidomethylation	27098	PREDICTED: uncharacterized protein LOC100263170 [Vitis vinifera]
1122	18129	gi|566148278	100.13	11	4	1	Carbamidomethylation	38178	UDP-glucose 4-epimerase family protein [Populus trichocarpa]
1417	1344	gi|225454138	100.07	12	4	4	Carbamidomethylation	55947	PREDICTED: DEAD-box ATP-dependent RNA helicase 8 [Vitis vinifera]
1189	1587	gi|225453350	100.01	16	3	3		16310	PREDICTED: nucleoside diphosphate kinase B [Vitis vinifera]
1362	1405	gi|225433434	99.97	6	4	2		65411	PREDICTED: dihydroxy-acid dehydratase chloroplastic [Vitis vinifera]
1351	1772	gi|225451677	99.96	4	5	1		108901	PREDICTED: staphylococcal nuclease domain-containing protein 1 [Vitis vinifera]
1498	17838	gi|224072208	99.87	12	3	1	Carbamidomethylation	38044	UDP-glucose 4-epimerase family protein [Populus trichocarpa]
619	18002	gi|566185685	99.79	14	3	1	Carbamidomethylation	22408	hypothetical protein POPTR_0009s01050g [Populus trichocarpa]
1261	797	gi|225433894	99.75	3	7	2	Carbamidomethylation	246915	PREDICTED: U5 small nuclear ribonucleoprotein 200 kDa helicase [Vitis vinifera]

773	18108	gi|566183164	99.68	19	2	1		19589	hypothetical protein POPTR_0008s09660g [Populus trichocarpa]
830	18405	gi|224095668	99.58	9	3	1		35550	hypothetical protein POPTR_0007s01850g [Populus trichocarpa]
1441	18849	gi|224065507	99.51	19	3	2		15080	Photosystem I reaction center subunit IV A family protein [Populus trichocarpa]
1097	942	gi|225453935	99.37	17	6	5		40128	PREDICTED: 4-hydroxy-tetrahydrodipicolinate synthase chloroplastic [Vitis vinifera]
1114	623	gi|731385630	99.34	5	4	4		99551	PREDICTED: heat shock 70 kDa protein 17 [Vitis vinifera]
1109	915	gi|225435203	99.18	39	4	4	Carbamidomethylation	15652	PREDICTED: 40S ribosomal protein S23-like [Vitis vinifera]
974	709	gi|359484512	99.16	12	6	6		63994	PREDICTED: asparagine--tRNA ligase cytoplasmic 1 [Vitis vinifera]
1666	1092	gi|225456481	99.07	5	6	3		112435	PREDICTED: alpha-mannosidase [Vitis vinifera]
2227	1301	gi|731426495	99.06	4	4	2		120041	PREDICTED: alpha-mannosidase [Vitis vinifera]
907	17507	gi|224066959	99.06	17	5	2		29005	14-3-3-like protein GF14 omicron [Populus trichocarpa]
1903	1187	gi|731395849	99.02	14	4	1		39121	PREDICTED: pto-interacting protein 1 [Vitis vinifera]
1240	17549	gi|566187890	98.92	14	6	6	Carbamidomethylation	55453	26S proteasome regulatory subunit S3 family protein [Populus trichocarpa]
1916	18040	gi|566174827	98.79	5	4	1	Carbamidomethylation	86014	hypothetical protein POPTR_0006s06460g [Populus trichocarpa]
1710	28876	gi|566161682	98.78	13	5	5	Carbamidomethylation	49311	AP47/50p mRNA family protein [Populus trichocarpa]
794	659	gi|225435177	98.74	15	5	1	Carbamidomethylation	27557	PREDICTED: L-ascorbate peroxidase 2 cytosolic [Vitis vinifera]
1061	973	gi|225450215	98.74	21	5	2		26049	PREDICTED: 60S ribosomal protein L6-3 [Vitis vinifera]

1330	17692	gi|566192732	98.6	15	5	1	Carbamidomethylation	44037	hypothetical protein POPTR_0010s25520g [Populus trichocarpa]
1084	703	gi|731388929	98.56	10	5	2	Carbamidomethylation	56541	PREDICTED: bifunctional 3-dehydroquinate dehydratase/shikimate dehydrogenase chloroplastic isoform X2 [Vitis vinifera]
963	698	gi|731416963	98.36	24	5	1	Carbamidomethylation	18774	PREDICTED: peptidyl-prolyl cis-trans isomerase CYP19-3 [Vitis vinifera]
1316	17430	gi|224055647	98.34	14	4	2		32125	succinate dehydrogenase iron-sulfur protein subunit [Populus trichocarpa]
825	1498	gi|225459760	98.32	12	5	4		33454	PREDICTED: thioredoxin-like protein CDSP32 chloroplastic [Vitis vinifera]
839	17365	gi|224098962	98.23	15	5	1		29630	14-3-3-like protein GF14 iota [Populus trichocarpa]
1606	2232	gi|225459360	98.22	8	4	2		56704	PREDICTED: probable glucan 1 3-beta-glucosidase A [Vitis vinifera]
347	17149	gi|224081102	98.13	49	7	2	Carbamidomethylation	15406	hypothetical protein POPTR_0005s07340g [Populus trichocarpa]
1518	18223	gi|566197639	98.12	12	4	4		44386	putative proteasome regulatory subunit family protein [Populus trichocarpa]
1668	1607	gi|225445166	97.96	5	6	1	Carbamidomethylation	122877	PREDICTED: uncharacterized protein LOC100262932 [Vitis vinifera]
1395	18046	gi|566168917	97.84	4	4	1		113232	hypothetical protein POPTR_0005s02130g [Populus trichocarpa]
1520	28901	gi|566156965	97.79	10	3	1		51266	hypothetical protein POPTR_0002s08650g [Populus trichocarpa]
1065	17773	gi|566213322	97.78	8	3	2	Carbamidomethylation	55497	hypothetical protein POPTR_0017s14220g [Populus trichocarpa]

1047	653	gi|225449060	97.73	6	3	3		62143	PREDICTED: protein disulfide isomerase-like 1-4 [Vitis vinifera]
367	953	gi|526118138	97.7	17	4	1		33840	uncharacterized protein LOC100264250 [Vitis vinifera]
1188	1608	gi|225452228	97.66	12	3	1	Carbamidomethylation	30877	PREDICTED: succinate dehydrogenase [ubiquinone] iron-sulfur subunit 1 mitochondrial [Vitis vinifera]
901	1099	gi|731418950	97.53	2	5	3		248350	PREDICTED: nuclear pore complex protein GP210 [Vitis vinifera]
1445	951	gi|731371455	97.39	8	4	2		58282	PREDICTED: coatomer subunit delta [Vitis vinifera]
1481	976	gi|731405314	97.23	5	4	3		97279	PREDICTED: serine/threonine-protein phosphatase 6 regulatory subunit 2 [Vitis vinifera]
1682	3862	gi|731416275	97.02	9	5	3	Carbamidomethylation	71591	PREDICTED: mitochondrial Rho GTPase 1 [Vitis vinifera]
1430	17391	gi|566182904	97.01	8	4	4	Carbamidomethylation	59760	hypothetical protein POPTR_0008s07890g [Populus trichocarpa]
940	782	gi|225458719	96.98	9	5	1	Carbamidomethylation; Oxidation (M)	63799	PREDICTED: D-3-phosphoglycerate dehydrogenase 3 chloroplastic-like [Vitis vinifera]
1266	819	gi|731423558	96.97	6	7	6	Carbamidomethylation; Oxidation (M)	120839	PREDICTED: valine--tRNA ligase isoform X2 [Vitis vinifera]
716	17947	gi|224063787	96.97	28	4	1		17073	ribosomal protein S13 [Populus trichocarpa]
1775	7752	gi|731393817	96.88	7	3	3		83302	PREDICTED: serrate RNA effector molecule [Vitis vinifera]
1488	3543	gi|225430488	96.81	6	3	1		73594	PREDICTED: transmembrane 9 superfamily member 9 [Vitis vinifera]
1125	683	gi|225456085	96.81	9	5	5		57173	PREDICTED: eukaryotic peptide chain release factor GTP-binding subunit ERF3A [Vitis vinifera]
1282	17905	gi|224105715	96.68	7	3	3	Carbamidomethylation	69881	vacuolar sorting receptor protein BP-80 [Populus trichocarpa]
1166	1338	gi|225441385	96.66	19	5	3		25906	PREDICTED: 60S ribosomal protein L6-1 [Vitis vinifera]

1835	19089	gi|566149765	96.64	10	3	2		43680	3-isopropylmalate dehydrogenase 2 family protein [Populus trichocarpa]
2076	1243	gi|225438781	96.58	7	3	1	Carbamidomethylation	71362	PREDICTED: polyadenylate-binding protein 2 [Vitis vinifera]
808	18121	gi|566172301	96.42	10	5	2	Carbamidomethylation	54904	disulfide-isomerase family protein [Populus trichocarpa]
1146	1529	gi|225432466	96.36	17	2	2	Carbamidomethylation	18476	PREDICTED: peptide methionine sulfoxide reductase B5-like [Vitis vinifera]
1305	17633	gi|224109084	96.32	10	5	1		53455	inosine monophosphate dehydrogenase family protein [Populus trichocarpa]
1379	1728	gi|225463807	96.32	9	4	1	Carbamidomethylation	45416	PREDICTED: alpha-galactosidase [Vitis vinifera]
1783	1377	gi|225452877	96.31	8	3	2		57074	PREDICTED: cystathionine gamma-synthase chloroplastic [Vitis vinifera]
1411	1354	gi|731430037	96.2	9	3	3	Carbamidomethylation	43016	PREDICTED: probable cinnamyl alcohol dehydrogenase 6 [Vitis vinifera]
2096	1669	gi|225456459	96.18	8	3	2		42395	PREDICTED: V-type proton ATPase subunit C isoform X1 [Vitis vinifera]
1136	17721	gi|566171771	96.17	9	4	2		53299	hypothetical protein POPTR_0005s16590g partial [Populus trichocarpa]
1010	17569	gi|566167161	96.15	9	3	1	Carbamidomethylation	40240	hypothetical protein POPTR_0004s18030g [Populus trichocarpa]
1855	18328	gi|224063293	95.99	16	4	4	Carbamidomethylation	31523	FH INTERACTING protein 1 [Populus trichocarpa]
1517	695	gi|359487652	95.94	9	5	2	Carbamidomethylation	54144	PREDICTED: fumarate hydratase 1 mitochondrial [Vitis vinifera]
1311	28872	gi|224118920	95.88	16	5	2	Carbamidomethylation	48600	Actin-related family protein [Populus trichocarpa]
546	1024	gi|225434935	95.74	17	4	1	Carbamidomethylation	27348	PREDICTED: triosephosphate isomerase cytosolic isoform X1 [Vitis vinifera]
1153	18325	gi|566204282	95.67	13	4	2	Carbamidomethylation	37909	aldo/keto reductase family protein [Populus trichocarpa]

1711	17930	gi|566208589	95.63	10	4	4		56137	dyskerin family protein [Populus trichocarpa]
1276	17671	gi|566183513	95.56	6	3	2		62808	hypothetical protein POPTR_0008s11770g [Populus trichocarpa]
1219	18232	gi|566187006	95.45	7	2	2		50172	hypothetical protein POPTR_0009s08570g [Populus trichocarpa]
1179	18135	gi|566160281	95.41	14	3	3		25839	beta-hydroxyacyl-ACP dehydratase family protein [Populus trichocarpa]
1419	18268	gi|566195934	95.4	5	4	2	Carbamidomethylation	83449	alkaline alpha galactosidase I family protein [Populus trichocarpa]
731	17541	gi|566196811	95.2	6	4	1	Carbamidomethylation	62410	hypothetical protein POPTR_0012s04670g [Populus trichocarpa]
1504	715	gi|731436956	95.17	10	4	2	Carbamidomethylation	39713	PREDICTED: porphobilinogen deaminase chloroplastic [Vitis vinifera]
1332	2139	gi|225431755	95.1	8	5	1	Carbamidomethylation	78694	PREDICTED: glyoxysomal fatty acid beta-oxidation multifunctional protein MFP-a [Vitis vinifera]
632	1508	gi|359476741	95.06	54	3	3	Carbamidomethylation	8039	PREDICTED: 60S ribosomal protein L38 [Vitis vinifera]
1082	804	gi|225460542	94.98	10	3	1		36331	PREDICTED: heterogeneous nuclear ribonucleoprotein 1 [Vitis vinifera]
1273	3842	gi|225433424	94.85	10	3	1	Carbamidomethylation	48624	PREDICTED: 3-ketoacyl-CoA thiolase 2 peroxisomal [Vitis vinifera]
1472	2222	gi|225450401	94.8	5	4	3		83484	PREDICTED: DEAD-box ATP-dependent RNA helicase 3 chloroplastic [Vitis vinifera]
1585	17732	gi|566198611	94.68	8	6	6		82290	hypothetical protein POPTR_0013s00230g [Populus trichocarpa]
1806	723	gi|731370529	94.67	6	3	1	Carbamidomethylation	66006	PREDICTED: serine/threonine-protein phosphatase 2A 65 kDa regulatory subunit A beta isoform-like [Vitis vinifera]

584	1421	gi|731386809	94.57	11	3	1		41704	PREDICTED: naringenin 2-oxoglutarate 3-dioxygenase-like [Vitis vinifera]
1583	908	gi|225426312	94.56	9	4	1		68263	PREDICTED: dynamin-related protein 5A [Vitis vinifera]
1577	2138	gi|731422323	94.54	5	3	1	Carbamidomethylation	104895	PREDICTED: alanine--tRNA ligase-like [Vitis vinifera]
1228	901	gi|359493854	94.53	22	4	4		20058	PREDICTED: thioredoxin M4 chloroplastic [Vitis vinifera]
1194	18912	gi|671743347	94.46	23	2	2		7800	photosystem II phosphoprotein (chloroplast) [Camellia pubicosta]
1044	762	gi|225448367	94.45	10	3	3		34281	PREDICTED: 60S acidic ribosomal protein P0 [Vitis vinifera]
869	700	gi|731391332	94.44	13	4	1		34474	PREDICTED: 60S ribosomal protein L5 [Vitis vinifera]
1333	17866	gi|566215051	94.41	5	4	2		106250	hypothetical protein POPTR_0018s09690g [Populus trichocarpa]
1105	17949	gi|224132216	94.4	28	5	2	Carbamidomethylation	11688	hypothetical protein POPTR_0014s18990g [Populus trichocarpa]
1519	861	gi|731383682	94.12	20	4	3		23042	PREDICTED: ras-related protein Rab7 [Vitis vinifera]
1790	2166	gi|359473071	94.04	5	3	3	Carbamidomethylation	65661	PREDICTED: bifunctional purine biosynthesis protein purH isoform X2 [Vitis vinifera]
2295	28921	gi|566184237	93.96	6	3	2		90135	hypothetical protein POPTR_0008s16030g [Populus trichocarpa]
2114	17805	gi|566180142	93.95	17	4	4		36527	hypothetical protein POPTR_0007s07780g [Populus trichocarpa]
1499	1178	gi|225461906	93.86	5	3	1	Carbamidomethylation	67756	PREDICTED: DNA ligase 1 [Vitis vinifera]
1995	1606	gi|225452646	93.83	11	3	1	Carbamidomethylation	32721	PREDICTED: protein transport protein SEC13 homolog B [Vitis vinifera]
1431	28907	gi|224097915	93.7	19	5	1		30342	plasma membrane intrinsic protein 1-1 [Populus trichocarpa]

1497	18416	gi|224071567	93.62	7	4	1		65821	monocopper oxidase precursor family protein [Populus trichocarpa]
1017	544	gi|731392981	93.61	4	4	1	Carbamidomethylation	102178	PREDICTED: linoleate 13S-lipoxygenase 2-1 chloroplastic-like isoform X2 [Vitis vinifera]
1280	19211	gi|566211720	93.57	5	4	2	Carbamidomethylation	131864	hypothetical protein POPTR_0017s06170g [Populus trichocarpa]
1471	19116	gi|224078882	93.55	10	3	3	Carbamidomethylation	34470	oxidoreductase family protein [Populus trichocarpa]
1475	18255	gi|566174566	93.47	9	5	2		68630	hypothetical protein POPTR_0006s05130g [Populus trichocarpa]
1032	566	gi|731407251	93.27	5	5	2		92866	PREDICTED: phospholipase D alpha 1-like [Vitis vinifera]
1507	792	gi|225465764	93.24	10	3	2	Carbamidomethylation	43403	PREDICTED: UDP-D-apiose/UDP-D-xylose synthase 2 [Vitis vinifera]
1397	18065	gi|224100909	93.23	4	5	2		134447	splicing factor family protein [Populus trichocarpa]
2092	1249	gi|731421815	92.98	12	4	4	Carbamidomethylation	53279	PREDICTED: tubulin gamma-1 chain [Vitis vinifera]
1244	946	gi|225429638	92.84	10	4	1		39454	PREDICTED: omega-amidase chloroplastic [Vitis vinifera]
1665	28919	gi|224088822	92.79	6	3	2		78516	ftsH-like protease family protein [Populus trichocarpa]
1772	18244	gi|566198992	92.75	7	4	2	Carbamidomethylation	78312	hypothetical protein POPTR_0013s02400g [Populus trichocarpa]
1394	1429	gi|731408438	92.74	4	3	1	Carbamidomethylation	107998	PREDICTED: villin-4 [Vitis vinifera]
1549	18759	gi|566182594	92.64	28	3	3		12883	60S ribosomal protein L35a [Populus trichocarpa]
1092	586	gi|731439234	92.6	6	4	3	Carbamidomethylation	74297	PREDICTED: peroxisomal acyl-coenzyme A oxidase 1 [Vitis vinifera]
1545	1496	gi|731392621	92.59	6	3	1		64193	PREDICTED: protein disulfide isomerase-like 1-4 isoform X2 [Vitis vinifera]
857	977	gi|225451247	92.57	23	3	3		15707	PREDICTED: 40S ribosomal protein S24-1 [Vitis vinifera]

1525	18215	gi|566147574	92.55	10	3	3		44838	hypothetical protein POPTR_0001s06560g [Populus trichocarpa]
1677	17966	gi|566191728	92.45	8	4	1	Carbamidomethylation	64682	hypothetical protein POPTR_0010s19790g [Populus trichocarpa]
1793	18204	gi|566175538	92.25	4	2	2		96436	hypothetical protein POPTR_0006s10420g [Populus trichocarpa]
1477	18117	gi|566167194	92.24	12	3	3	Carbamidomethylation	41861	glycerate dehydrogenase family protein [Populus trichocarpa]
2244	18220	gi|566152765	92.19	12	4	4		49287	hypothetical protein POPTR_0001s34620g [Populus trichocarpa]
909	2123	gi|526117649	92.15	11	5	3	Carbamidomethylation	41054	alcohol dehydrogenase 1 [Vitis vinifera]
1605	1649	gi|225469310	92.02	16	4	4	Carbamidomethylation	39287	PREDICTED: sorbitol dehydrogenase [Vitis vinifera]
1035	28878	gi|225433841	92.02	5	6	4	Carbamidomethylation	135558	PREDICTED: probable isoleucine--tRNA ligase cytoplasmic isoform X2 [Vitis vinifera]
1239	17965	gi|224062213	91.88	24	2	2		17225	hypothetical protein POPTR_0002s04420g [Populus trichocarpa]
1446	18246	gi|566215059	91.84	9	4	1		67827	hypothetical protein POPTR_0018s09760g [Populus trichocarpa]
1744	2325	gi|225443274	91.74	12	3	3		35506	PREDICTED: cell wall integrity protein scw1 [Vitis vinifera]
1391	1503	gi|225461001	91.68	7	2	2		46284	PREDICTED: polyadenylate-binding protein RBP45-like [Vitis vinifera]
1295	17578	gi|566198207	91.44	11	4	1		32557	hypothetical protein POPTR_0012s12860g [Populus trichocarpa]
1528	2319	gi|225452270	91.39	8	2	2		34598	PREDICTED: 28 kDa ribonucleoprotein chloroplastic-like [Vitis vinifera]
1515	2321	gi|731416683	91.38	16	2	2	Carbamidomethylation	15282	PREDICTED: superoxide dismutase [Cu-Zn] isoform X2 [Vitis vinifera]

1233	1651	gi|225438962	91.31	6	2	2		35957	PREDICTED: peroxidase 73 [Vitis vinifera]
573	1077	gi|225448675	91.2	16	3	3		23332	PREDICTED: probable chalcone--flavonone isomerase 3 [Vitis vinifera]
1267	845	gi|225428011	91.17	8	5	2		61287	PREDICTED: synaptotagmin-2 [Vitis vinifera]
1285	18981	gi|224146411	90.98	8	3	1		49063	dihydrolipoamide S-acetyltransferase family protein [Populus trichocarpa]
718	17746	gi|566215609	90.96	4	3	2		91834	hypothetical protein POPTR_0018s13110g [Populus trichocarpa]
1364	17497	gi|566205259	90.93	21	5	4	Carbamidomethylation	29312	hypothetical protein POPTR_0015s00690g [Populus trichocarpa]
1881	842	gi|731424103	90.77	2	2	1	Carbamidomethylation	151229	PREDICTED: tripeptidyl-peptidase 2 isoform X1 [Vitis vinifera]
1453	2304	gi|225435608	90.73	11	2	2	Carbamidomethylation	31609	PREDICTED: zinc finger CCCH domain-containing protein 14-like [Vitis vinifera]
1262	18807	gi|566190669	90.67	7	3	1		48407	hypothetical protein POPTR_0010s13650g [Populus trichocarpa]
898	888	gi|225448819	90.65	24	3	3		14272	PREDICTED: 60S ribosomal protein L35 [Vitis vinifera]
1532	28891	gi|224087393	90.61	12	4	1		50473	hypothetical protein POPTR_0006s08310g [Populus trichocarpa]
849	1653	gi|225429975	90.61	8	3	2		39098	PREDICTED: bark storage protein A [Vitis vinifera]
999	18187	gi|566211618	90.6	9	3	1		35272	pfkB-type carbohydrate kinase family protein [Populus trichocarpa]
1948	1854	gi|225470285	90.58	14	3	3		32382	PREDICTED: ATP-dependent Clp protease proteolytic subunit-related protein 2 chloroplastic [Vitis vinifera]
1202	730	gi|731433246	90.41	7	5	5	Carbamidomethylation	78819	PREDICTED: threonine--tRNA ligase mitochondrial-like [Vitis vinifera]

1478	18611	gi|224077508	90.35	12	4	1		39890	putative protein phosphatase [Populus trichocarpa]
1522	1588	gi|225429995	90.29	29	3	2	Carbamidomethylation	17724	PREDICTED: protein PEROXIN-4 [Vitis vinifera]
786	18054	gi|224068558	90.28	12	3	2		32725	NUCLEAR ENCODED CLP PROTEASE 1 family protein [Populus trichocarpa]
1324	1091	gi|225428916	90.21	14	4	3		39009	PREDICTED: probable cinnamyl alcohol dehydrogenase 1 [Vitis vinifera]
1049	1119	gi|225450677	90.14	4	4	1	Carbamidomethylation	124814	PREDICTED: topless-related protein 3 [Vitis vinifera]
1533	1552	gi|359495476	90.09	23	4	4		29035	PREDICTED: chlorophyll a-b binding protein 13 chloroplastic [Vitis vinifera]
1334	1145	gi|359475871	90.08	20	4	4	Carbamidomethylation	32719	PREDICTED: protein MEMO1 [Vitis vinifera]
1451	2318	gi|731430027	90.03	26	3	1	Carbamidomethylation	17695	PREDICTED: SKP1-like protein 1B [Vitis vinifera]
1013	17931	gi|224079093	89.97	29	4	4		12417	60S ribosomal protein L36-3 [Populus trichocarpa]
1720	2044	gi|225443859	89.91	6	2	1		24656	PREDICTED: calcyclin-binding protein [Vitis vinifera]
2351	19380	gi|731407226	89.88	9	2	2	Carbamidomethylation	37934	PREDICTED: 2-alkenal reductase (NADP(+)-dependent) [Vitis vinifera]
1476	1271	gi|225459587	89.82	6	4	1		55671	PREDICTED: protein disulfide-isomerase [Vitis vinifera]
1591	18337	gi|566204836	89.77	4	2	1	Carbamidomethylation	84382	hypothetical protein POPTR_0014s17660g [Populus trichocarpa]
1947	2175	gi|225437374	89.76	8	4	2		49415	PREDICTED: UDP-glucuronic acid decarboxylase 2-like [Vitis vinifera]
1910	18049	gi|566147740	89.72	9	3	1		36450	hypothetical protein POPTR_0001s07480g [Populus trichocarpa]
1066	18175	gi|566202597	89.71	17	3	1		16336	hypothetical protein POPTR_0014s04880g [Populus trichocarpa]

1124	829	gi|225436257	89.63	12	3	3		29530	PREDICTED: chlorophyll a-b binding protein 8 chloroplastic [Vitis vinifera]
460	1401	gi|731386455	89.59	1	3	2		95638	PREDICTED: sucrose synthase 7 isoform X3 [Vitis vinifera]
1542	28895	gi|224111100	89.51	14	4	1	Carbamidomethylation	27203	hypothetical protein POPTR_0010s09180g [Populus trichocarpa]
384	753	gi|731391225	89.43	10	4	1		38311	PREDICTED: endochitinase A-like isoform X1 [Vitis vinifera]
600	1507	gi|225470846	89.35	13	2	1		22027	PREDICTED: nascent polypeptide-associated complex subunit alpha-like protein 1 [Vitis vinifera]
918	1352	gi|225428908	89.3	6	3	3		53765	PREDICTED: limonoid UDP-glucosyltransferase [Vitis vinifera]
1484	1481	gi|731415988	89.29	4	4	1		119890	PREDICTED: FACT complex subunit SPT16-like [Vitis vinifera]
1704	18105	gi|224145917	89.21	10	3	3		44521	Uroporphyrinogen decarboxylase family protein [Populus trichocarpa]
1723	17680	gi|566162135	89.18	18	5	1	Oxidation (M)	27086	hypothetical protein POPTR_0003s11350g [Populus trichocarpa]
1050	1029	gi|359473645	89.13	4	4	2	Carbamidomethylation	66079	PREDICTED: chaperonin 60 subunit beta 4 chloroplastic [Vitis vinifera]
2202	19149	gi|566215907	89.13	3	3	3		131624	hypothetical protein POPTR_0018s14630g [Populus trichocarpa]
932	17315	gi|224079274	89.07	9	4	1		41124	ubiquitin family protein [Populus trichocarpa]
1002	17604	gi|566194465	89.04	12	3	2		24921	SGS domain-containing family protein [Populus trichocarpa]
1143	876	gi|225429341	88.98	9	3	2	Carbamidomethylation	44078	PREDICTED: 3-oxo-Delta(4 5)-steroid 5-beta-reductase-like [Vitis vinifera]
1460	17626	gi|224072049	88.84	13	4	1		28211	reticulon family protein [Populus trichocarpa]
1857	2404	gi|225456349	88.73	14	3	1		26827	PREDICTED: gamma carbonic anhydrase-like 2 mitochondrial [Vitis vinifera]

1169	2109	gi|225451233	88.71	9	2	1	Carbamidomethylation	34208	PREDICTED: cysteine synthase [Vitis vinifera]
1648	17900	gi|224104697	88.63	16	3	1		20848	lactoylglutathione lyase family protein [Populus trichocarpa]
1713	20422	gi|224082496	88.62	18	3	2	Carbamidomethylation	38736	peroxidase precursor family protein [Populus trichocarpa]
1569	763	gi|225433440	88.62	11	4	4		53869	PREDICTED: glutathione reductase cytosolic [Vitis vinifera]
1372	18219	gi|566163043	88.62	6	4	1		67870	glucose-6-phosphate dehydrogenase family protein [Populus trichocarpa]
1526	20416	gi|566200837	88.58	47	4	2	Carbamidomethylation	13900	ribosomal protein L7Ae/L30e/S12e/Gadd45 [Populus trichocarpa]
1602	18196	gi|566245692	88.56	11	3	1		25488	hypothetical protein POPTR_0019s15100g [Populus trichocarpa]
1150	18146	gi|566178654	88.56	6	4	1	Carbamidomethylation	74689	hypothetical protein POPTR_0006s28750g [Populus trichocarpa]
1294	722	gi|225430200	88.53	14	7	3		40334	PREDICTED: uncharacterized protein LOC100252479 [Vitis vinifera]
1692	17468	gi|566177699	88.49	8	3	1		48113	hypothetical protein POPTR_0006s23050g [Populus trichocarpa]
908	17329	gi|566191085	88.4	11	6	1	Carbamidomethylation	51784	hypothetical protein POPTR_0010s16120g [Populus trichocarpa]
1087	18542	gi|224124892	88.3	13	2	1		22641	rhicadhesin receptor family protein [Populus trichocarpa]
823	18516	gi|224104953	88.25	6	4	2		68350	beta-D-glucan exohydrolase family protein [Populus trichocarpa]
980	18015	gi|224116326	88.15	10	4	1		36463	Leucoanthocyanidin reductase family protein [Populus trichocarpa]
1817	19367	gi|566170761	88.13	9	3	1		48329	hypothetical protein POPTR_0005s11300g [Populus trichocarpa]

1894	1736	gi|731410731	88.08	2	3	1		178423	PREDICTED: UDP-glucose:glycoprotein glucosyltransferase isoform X2 [Vitis vinifera]
1647	17528	gi|566162704	88.02	28	3	2	Carbamidomethylation	18629	hypothetical protein POPTR_0003s14870g [Populus trichocarpa]
2029	18286	gi|566188208	88.02	3	3	3		139500	TITAN7 family protein [Populus trichocarpa]
1993	3535	gi|225428388	88	14	4	4	Carbamidomethylation	40284	PREDICTED: COP9 signalosome complex subunit 5b-like [Vitis vinifera]
1392	2305	gi|526117321	87.93	12	2	2		23675	dehydroascorbate reductase [Vitis vinifera]
1366	19068	gi|224075417	87.87	13	3	1	Carbamidomethylation	34152	EXGT1 family protein [Populus trichocarpa]
2024	2210	gi|225435307	87.82	6	2	1	Carbamidomethylation	49888	PREDICTED: ruvB-like protein 1 [Vitis vinifera]
1272	17479	gi|224097148	87.82	11	4	4	Carbamidomethylation	41439	GDP-mannose 4 family protein [Populus trichocarpa]
495	1532	gi|225460502	87.73	4	2	2	Carbamidomethylation	64083	PREDICTED: laccase-14 [Vitis vinifera]
1521	778	gi|359483949	87.58	11	4	1	Carbamidomethylation	42343	PREDICTED: ferredoxin--NADP reductase root isozyme chloroplastic [Vitis vinifera]
1409	18329	gi|224120174	87.55	8	4	3	Carbamidomethylation	68576	Phosphoglucomutase family protein [Populus trichocarpa]
1354	1683	gi|225467712	87.53	7	3	2	Carbamidomethylation	62107	PREDICTED: UPF0051 protein ABCI8 chloroplastic [Vitis vinifera]
1412	17743	gi|566175322	87.48	9	2	1		35273	aldo/keto reductase family protein [Populus trichocarpa]
1180	1353	gi|225428047	87.47	22	3	1	Carbamidomethylation	17518	PREDICTED: SKP1-like protein 1A isoform X5 [Vitis vinifera]
1524	17674	gi|224104643	87.41	6	3	1		65194	thioredoxin family protein [Populus trichocarpa]
1926	17828	gi|224082834	87.4	7	4	1	Carbamidomethylation	69308	dehydration-responsive family protein [Populus trichocarpa]
1902	1995	gi|731412542	87.37	11	4	4		58149	PREDICTED: uncharacterized protein LOC100244432 isoform X4 [Vitis vinifera]

1187	931	gi|225434323	87.34	14	4	4	Carbamidomethylation	30353	PREDICTED: eukaryotic translation initiation factor 2 subunit beta [Vitis vinifera]
1389	1090	gi|225465997	87.34	9	4	3	Carbamidomethylation	48382	PREDICTED: WD-40 repeat-containing protein MSI1 [Vitis vinifera]
1268	868	gi|225455106	87.25	9	3	3		33350	PREDICTED: protein BOBBER 1 [Vitis vinifera]
1550	1108	gi|731389140	87.23	8	4	2	Carbamidomethylation	58551	PREDICTED: RAN GTPase-activating protein 2 [Vitis vinifera]
1890	1901	gi|225430912	87.16	16	3	3		35298	PREDICTED: membrane-associated protein VIPP1 chloroplastic [Vitis vinifera]
1821	18326	gi|224109088	87.09	6	3	3	Carbamidomethylation	57503	SUCCINIC SEMIALDEHYDE DEHYDROGENASE family protein [Populus trichocarpa]
1396	28899	gi|224101049	87.05	14	4	3	Carbamidomethylation	35917	type 1 protein phosphatase-1 [Populus trichocarpa]
1345	1381	gi|526117942	86.97	6	2	1		37456	beta 1-3 glucanase precursor [Vitis vinifera]
1511	17840	gi|224054773	86.87	20	4	1		17854	17.4 kDa class I heat shock family protein [Populus trichocarpa]
1078	28882	gi|566208767	86.87	8	6	2		89399	hypothetical protein POPTR_0016s04960g [Populus trichocarpa]
1726	890	gi|225442223	86.81	7	3	3		49618	PREDICTED: glycylpeptide N-tetradecanoyltransferase 1 [Vitis vinifera]
1537	18393	gi|224100515	86.74	13	3	3	Carbamidomethylation	45833	hypothetical protein POPTR_0008s00700g [Populus trichocarpa]
870	941	gi|225456914	86.74	21	4	3	Carbamidomethylation	16350	PREDICTED: 60S ribosomal protein L27a-3 [Vitis vinifera]
1048	1128	gi|731418015	86.72	14	5	4		53972	PREDICTED: tryptophan synthase beta chain 1 [Vitis vinifera]
1803	17920	gi|224087657	86.7	12	3	2		32555	UNCOUPLING family protein [Populus trichocarpa]
1762	18184	gi|224122176	86.61	4	3	1		113456	hypothetical protein POPTR_0012s10830g [Populus trichocarpa]

1298	18261	gi|224109256	86.28	12	3	2		29460	ferritin 2 precursor family protein [Populus trichocarpa]
1714	28968	gi|224099715	86.19	10	3	3	Carbamidomethylation	36854	spermidine synthase family protein [Populus trichocarpa]
1891	508	gi|731432625	86.17	2	2	1	Carbamidomethylation	121307	PREDICTED: protein transport protein Sec24-like At4g32640 [Vitis vinifera]
1245	1184	gi|731387210	86.13	8	3	3		42062	PREDICTED: splicing factor U2af large subunit B isoform X4 [Vitis vinifera]
1458	28904	gi|566214978	86.04	6	4	4	Carbamidomethylation	87044	hypothetical protein POPTR_0018s09300g [Populus trichocarpa]
1374	17736	gi|224080971	86.03	13	2	1	Carbamidomethylation	18386	hypothetical protein POPTR_0005s06420g [Populus trichocarpa]
1387	1863	gi|359487567	85.95	4	2	2	Carbamidomethylation	41694	PREDICTED: GDSL esterase/lipase At1g09390 [Vitis vinifera]
1646	1276	gi|225457405	85.88	10	3	1		51267	PREDICTED: geranylgeranyl diphosphate reductase chloroplastic [Vitis vinifera]
1630	1120	gi|225465441	85.82	17	2	1		14675	PREDICTED: cytochrome b5 [Vitis vinifera]
978	17596	gi|566171781	85.59	5	4	4		84742	hypothetical protein POPTR_0005s16660g [Populus trichocarpa]
1763	29006	gi|566209510	85.58	2	2	2		144287	P glycoprotein1 [Populus trichocarpa]
1418	1211	gi|731391365	85.47	10	4	2	Carbamidomethylation	52738	PREDICTED: acetolactate synthase small subunit 2 chloroplastic isoform X2 [Vitis vinifera]
1816	2587	gi|359480790	85.43	7	4	1		64379	PREDICTED: patellin-3-like [Vitis vinifera]
1784	18355	gi|224084344	85.41	10	3	2	Carbamidomethylation	40622	RNA recognition motif-containing family protein [Populus trichocarpa]
1308	1323	gi|526117539	85.39	10	2	1	Carbamidomethylation	34687	class I extracellular chitinase precursor [Vitis vinifera]
1547	1688	gi|359479353	85.24	18	3	1		21048	PREDICTED: lactoylglutathione lyase isoform X2 [Vitis vinifera]

1625	18005	gi|224074217	85.19	16	3	1	Carbamidomethylation	22693	GTP-binding family protein [Populus trichocarpa]
2085	29041	gi|224057608	85.03	5	2	1	Carbamidomethylation	65798	dehydratase family protein [Populus trichocarpa]
1257	17955	gi|224097434	84.89	10	3	1	Carbamidomethylation	40856	cytokinesis defective 1 family protein [Populus trichocarpa]
1480	17856	gi|731401507	84.88	3	3	3	Carbamidomethylation	138211	PREDICTED: 5-oxoprolinase [Vitis vinifera]
1747	1351	gi|225464146	84.86	10	3	1		32622	PREDICTED: protein transport protein SEC13 homolog B [Vitis vinifera]
1025	1214	gi|731426684	84.68	6	5	3	Oxidation (M)	144086	PREDICTED: uncharacterized protein LOC100262718 isoform X4 [Vitis vinifera]
1672	18606	gi|566198676	84.67	5	4	1		124815	hypothetical protein POPTR_0013s00600g [Populus trichocarpa]
1781	17712	gi|224120862	84.66	7	4	1	Carbamidomethylation	96570	importin beta-2 family protein [Populus trichocarpa]
1447	17864	gi|566153954	84.65	15	4	2		31003	phosphatase 2C family protein [Populus trichocarpa]
1349	3233	gi|731379296	84.62	5	5	2	Carbamidomethylation	114179	PREDICTED: uncharacterized protein LOC100264973 [Vitis vinifera]
1966	2392	gi|731382564	84.4	9	3	3	Carbamidomethylation	53798	PREDICTED: aminoacylase-1 isoform X2 [Vitis vinifera]
1696	17854	gi|224062834	84.32	6	3	2	Carbamidomethylation	70900	Polyadenylate-binding protein 5 [Populus trichocarpa]
1632	1512	gi|225454123	84.25	14	2	1		22997	PREDICTED: pre-mRNA cleavage factor Im 25 kDa subunit 2 [Vitis vinifera]
1680	28969	gi|225468850	84.19	12	3	3		30830	PREDICTED: glyoxylate/succinic semialdehyde reductase 1 [Vitis vinifera]
1886	18911	gi|566184573	84.17	4	2	1		90021	urease family protein [Populus trichocarpa]
1227	540	gi|225449262	84.16	24	5	1		18173	PREDICTED: 18.1 kDa class I heat shock protein [Vitis vinifera]

1482	18304	gi|566184588	84.1	14	3	2		33826	hypothetical protein POPTR_0008s17880g [Populus trichocarpa]
2252	19669	gi|359487440	84.09	12	2	2		26076	PREDICTED: serine/arginine-rich SC35-like splicing factor SCL33 isoform X2 [Vitis vinifera]
1588	721	gi|225429209	83.97	7	4	2		53142	PREDICTED: serine carboxypeptidase-like 16 [Vitis vinifera]
1815	19228	gi|224086367	83.95	24	5	1	Carbamidomethylation	27400	Proteasome subunit alpha type 4 family protein [Populus trichocarpa]
1455	3226	gi|526118000	83.92	13	2	1		22732	germin-like protein 2 precursor [Vitis vinifera]
2042	471	gi|225433575	83.74	4	2	2	Carbamidomethylation	105215	PREDICTED: probable glucan 1 3-alpha-glucosidase [Vitis vinifera]
1712	17815	gi|566187112	83.7	12	3	3	Carbamidomethylation	38128	WD-40 repeat family protein [Populus trichocarpa]
1487	18132	gi|566151049	83.7	13	3	3	Carbamidomethylation	26982	hypothetical protein POPTR_0001s26020g [Populus trichocarpa]
661	18371	gi|224116936	83.57	4	2	1		63722	pyruvate decarboxylase family protein [Populus trichocarpa]
1685	1173	gi|731393107	83.52	5	3	1		108073	PREDICTED: DNA (cytosine-5)-methyltransferase CMT3 [Vitis vinifera]
1707	18000	gi|526118245	83.48	12	2	1	Carbamidomethylation	23461	peroxiredoxin Q [Vitis vinifera]
1669	3156	gi|225454813	83.33	4	3	2	Carbamidomethylation	123938	PREDICTED: exportin-1 [Vitis vinifera]
1607	1550	gi|225427294	83.23	23	4	1		23133	PREDICTED: ras-related protein RABH1b [Vitis vinifera]
757	914	gi|225440109	83.17	25	4	2		10658	PREDICTED: 10 kDa chaperonin [Vitis vinifera]
1106	18512	gi|566209992	83.13	9	2	1	Carbamidomethylation	39168	hypothetical protein POPTR_0016s12850g [Populus trichocarpa]
1231	17665	gi|566230552	83.1	13	3	3		26405	vacuolar ATPase subunit E family protein [Populus trichocarpa]
1191	18766	gi|566202435	83.07	24	4	3		22118	uridylate kinase family protein [Populus trichocarpa]

1428	18241	gi|566146307	83.05	10	5	2	Carbamidomethylation	66102	monocopper oxidase precursor family protein [Populus trichocarpa]
1470	1495	gi|731437632	82.97	12	4	4	Carbamidomethylation	44812	PREDICTED: branched-chain-amino-acid aminotransferase 5 chloroplastic [Vitis vinifera]
1168	1292	gi|731378427	82.81	6	3	1		51975	PREDICTED: uncharacterized protein LOC100263310 [Vitis vinifera]
1336	1201	gi|225454278	82.79	6	2	2		40499	PREDICTED: bifunctional L-3-cyanoalanine synthase/cysteine synthase 1 mitochondrial [Vitis vinifera]
847	836	gi|225458231	82.77	12	4	4	Carbamidomethylation	30646	PREDICTED: proteasome subunit alpha type-1-B [Vitis vinifera]
1798	29073	gi|225429981	82.75	4	2	2		110340	PREDICTED: ER membrane protein complex subunit 1 [Vitis vinifera]
1094	1011	gi|225435878	82.61	28	3	2		10567	PREDICTED: 10 kDa chaperonin [Vitis vinifera]
1246	17984	gi|224078816	82.58	11	4	1		36460	Leucoanthocyanidin reductase family protein [Populus trichocarpa]
1933	1251	gi|731432083	82.54	2	2	1		115512	PREDICTED: protein transport protein Sec24-like At3g07100 [Vitis vinifera]
2245	18096	gi|566198674	82.53	3	3	1		120951	hypothetical protein POPTR_0013s00590g [Populus trichocarpa]
1930	19298	gi|224122632	82.5	7	3	1		66196	Monocopper oxidase-like protein SKS1 precursor [Populus trichocarpa]
1335	1287	gi|225460991	82.47	5	3	1	Carbamidomethylation	86153	PREDICTED: heat shock 70 kDa protein 16 [Vitis vinifera]
1780	28905	gi|566161636	82.47	8	3	1	Carbamidomethylation	63019	Pyruvate kinase isozyme G family protein [Populus trichocarpa]
1996	2199	gi|225435169	82.4	9	3	1	Carbamidomethylation	42533	PREDICTED: mitogen-activated protein kinase homolog NTF6 [Vitis vinifera]
1297	18773	gi|566148271	82.35	12	3	1		25715	nucleoside diphosphate kinase family protein [Populus trichocarpa]

1368	18320	gi|566166505	82.34	7	4	1		65003	hypothetical protein POPTR_0004s14530g [Populus trichocarpa]
670	850	gi|225450384	82.34	4	3	2	Carbamidomethylation	57022	PREDICTED: beta-glucosidase 12-like [Vitis vinifera]
1973	2310	gi|225427364	82.33	6	2	2	Carbamidomethylation	51132	PREDICTED: poly(U)-specific endoribonuclease-B [Vitis vinifera]
1818	17777	gi|566153407	82.29	11	4	1		42007	ferredoxin-NADP reductase family protein [Populus trichocarpa]
919	19219	gi|566167417	82.29	11	2	2		11431	60s acidic ribosomal family protein [Populus trichocarpa]
1836	2035	gi|731402548	82.22	5	3	2		91875	PREDICTED: N-alpha-acetyltransferase 15 NatA auxiliary subunit isoform X3 [Vitis vinifera]
1929	1167	gi|225457034	82.16	9	3	3	Carbamidomethylation	51176	PREDICTED: 1-deoxy-D-xylulose 5-phosphate reductoisomerase chloroplastic [Vitis vinifera]
1355	17249	gi|566161432	82.04	4	3	2		74324	acyl-CoA oxidase family protein [Populus trichocarpa]
1626	18023	gi|566255780	81.88	18	3	1		16020	actin-depolymerizing factor family protein [Populus trichocarpa]
1699	18494	gi|224066943	81.85	6	3	3		35968	annexin 1 family protein [Populus trichocarpa]
1463	18725	gi|566211608	81.79	15	3	3		30060	hypothetical protein POPTR_0017s05720g [Populus trichocarpa]
361	736	gi|225455934	81.77	12	3	3		20429	PREDICTED: ribulose bisphosphate carboxylase small chain chloroplastic [Vitis vinifera]
1296	18139	gi|224107417	81.76	6	4	2		57907	dehydroquinate dehydratase family protein [Populus trichocarpa]
1440	1542	gi|225458237	81.71	9	2	1	Carbamidomethylation	29571	PREDICTED: gamma carbonic anhydrase 1 mitochondrial [Vitis vinifera]
1523	18334	gi|566172110	81.69	12	3	3	Carbamidomethylation	39318	casein kinase II alpha chain family protein [Populus trichocarpa]
1553	19910	gi|566152841	81.65	7	2	1	Carbamidomethylation	26914	hypothetical protein POPTR_0001s34950g [Populus trichocarpa]

990	705	gi|225428009	81.65	10	3	1	Carbamidomethylation	31769	PREDICTED: L-ascorbate peroxidase 3 peroxisomal [Vitis vinifera]
2457	28931	gi|566162125	81.58	6	3	3		86023	hypothetical protein POPTR_0003s11300g [Populus trichocarpa]
1650	18022	gi|224112819	81.51	21	3	1	Carbamidomethylation	16047	actin-depolymerizing factor family protein [Populus trichocarpa]
2210	19110	gi|566206676	81.49	3	2	2		79072	hypothetical protein POPTR_0015s08830g [Populus trichocarpa]
1546	17720	gi|566197177	81.44	6	3	1		52151	geranylgeranyl reductase family protein [Populus trichocarpa]
1624	1629	gi|359490763	81.43	9	3	3	Carbamidomethylation	39503	PREDICTED: caffeic acid 3-O-methyltransferase [Vitis vinifera]
2053	18762	gi|566160631	81.41	4	2	1		73609	endomembrane protein 70 [Populus trichocarpa]
1834	18886	gi|566175785	81.41	13	4	3	Carbamidomethylation	36260	hypothetical protein POPTR_0006s11790g [Populus trichocarpa]
1718	1628	gi|359482834	81.22	6	2	1		49834	PREDICTED: WD-40 repeat-containing protein MSI4 [Vitis vinifera]
1229	1386	gi|225434203	81.18	11	4	1	Carbamidomethylation	38265	PREDICTED: 2-alkenal reductase (NADP(+)-dependent) [Vitis vinifera]
2001	18395	gi|566182365	81.16	8	2	2		34377	hypothetical protein POPTR_0008s04660g [Populus trichocarpa]
1856	18691	gi|671743260	81.11	20	3	3		24165	cytochrome b6 (chloroplast) [Camellia petelotii]
1789	19451	gi|566160279	81.04	5	1	1		36389	hypothetical protein POPTR_0003s01450g [Populus trichocarpa]
1461	17675	gi|566198526	81	5	4	2		67271	hypothetical protein POPTR_0012s14780g [Populus trichocarpa]
1981	18830	gi|566214901	81	10	2	2	Oxidation (M)	35399	hypothetical protein POPTR_0018s08850g [Populus trichocarpa]

2119	18878	gi|566200709	81	5	2	1		63530	putative beta-fructofuranosidase family protein [Populus trichocarpa]
1824	2099	gi|225426826	80.97	7	2	1		36763	PREDICTED: replication factor C subunit 2 [Vitis vinifera]
1358	17669	gi|566185352	80.85	7	5	1	Carbamidomethylation	79054	hypothetical protein POPTR_0008s22410g [Populus trichocarpa]
2102	18659	gi|224055093	80.66	13	2	2		22171	hypothetical protein POPTR_0001s26920g [Populus trichocarpa]
1808	1820	gi|359478076	80.51	7	3	2	Carbamidomethylation	68073	PREDICTED: 2-isopropylmalate synthase A-like [Vitis vinifera]
1843	2016	gi|731410536	80.49	10	2	2	Carbamidomethylation	38859	PREDICTED: protein RAE1 [Vitis vinifera]
1791	18556	gi|224067088	80.48	8	3	3	Carbamidomethylation	73926	hypothetical protein POPTR_0002s10850g [Populus trichocarpa]
1896	18808	gi|224141215	80.42	2	3	2	Carbamidomethylation	141876	hypothetical protein POPTR_0017s07760g [Populus trichocarpa]
1716	1435	gi|225464870	80.39	9	2	2	Carbamidomethylation	37627	PREDICTED: bifunctional nitrilase/nitrile hydratase NIT4B [Vitis vinifera]
2288	19080	gi|225425274	80.38	7	2	2		29736	PREDICTED: cytochrome b-c1 complex subunit Rieske-4 mitochondrial [Vitis vinifera]
2039	1858	gi|359496003	80.26	7	3	3		43409	PREDICTED: probable transaldolase [Vitis vinifera]
1797	2328	gi|225435249	80.22	13	2	2		27570	PREDICTED: proteasome subunit beta type-4 [Vitis vinifera]
1145	981	gi|225445442	80.19	9	3	1		35854	PREDICTED: NADH-cytochrome b5 reductase-like protein [Vitis vinifera]
1693	1664	gi|225462058	80.05	7	4	1	Carbamidomethylation	83482	PREDICTED: probable galactinol--sucrose galactosyltransferase 1 [Vitis vinifera]
1232	17648	gi|566184227	80.01	5	2	1		64966	pyruvate phosphotransferase family protein [Populus trichocarpa]

2326	29454	gi|225438605	79.98	4	1	1	Carbamidomethylation	51322	PREDICTED: alanine--glyoxylate aminotransferase 2 homolog 1 mitochondrial [Vitis vinifera]
1057	719	gi|225424242	79.97	40	4	1		10611	PREDICTED: 10 kDa chaperonin [Vitis vinifera]
1777	18705	gi|224058237	79.64	6	2	1		68244	Dynamin-like family protein [Populus trichocarpa]
1361	2560	gi|225428263	79.58	26	3	3		14294	PREDICTED: small nuclear ribonucleoprotein Sm D3 [Vitis vinifera]
1456	1732	gi|225461632	79.55	6	2	2		44771	PREDICTED: uncharacterized protein At3g15000 mitochondrial [Vitis vinifera]
2000	18794	gi|224066629	79.48	16	2	2		16281	thioredoxin family protein [Populus trichocarpa]
1952	1546	gi|225460837	79.44	7	2	2		43944	PREDICTED: cell division protein FtsZ homolog 1 chloroplastic [Vitis vinifera]
1012	1320	gi|731440862	79.43	25	3	3	Carbamidomethylation	15642	PREDICTED: 60S ribosomal protein L32-1 [Vitis vinifera]
2084	18882	gi|224075096	79.36	4	2	2	Carbamidomethylation	73889	acyl-coA synthetase family protein [Populus trichocarpa]
1449	28940	gi|566162305	79.14	8	3	1	Carbamidomethylation	38904	UDP-glucose 4-epimerase family protein [Populus trichocarpa]
1822	1102	gi|225452700	79.14	10	4	2		29376	PREDICTED: mitochondrial outer membrane protein porin of 34 kDa [Vitis vinifera]
2064	17582	gi|566179618	79.08	5	2	2		48055	hypothetical protein POPTR_0007s04700g [Populus trichocarpa]
1337	18971	gi|566214594	78.93	10	4	1		54543	phosphofructokinase family protein [Populus trichocarpa]
1804	18317	gi|566147070	78.87	9	3	3		32560	hypothetical protein POPTR_0001s03780g [Populus trichocarpa]
2030	18372	gi|566167797	78.83	12	3	2		29714	hypothetical protein POPTR_0004s21400g [Populus trichocarpa]

1907	28956	gi|359482292	78.8	6	3	1		66341	PREDICTED: threonine dehydratase biosynthetic chloroplastic [Vitis vinifera]
1838	2997	gi|225455529	78.77	19	3	1	Carbamidomethylation	17124	PREDICTED: 40S ribosomal protein S13 [Vitis vinifera]
1883	2117	gi|225465870	78.71	1	2	1		205009	PREDICTED: DNA-directed RNA polymerase V subunit 1 [Vitis vinifera]
1628	2095	gi|225447745	78.7	11	2	2		27216	PREDICTED: chlorophyll a-b binding protein CP24 10A chloroplastic [Vitis vinifera]
2094	1372	gi|225462440	78.58	5	4	3	Carbamidomethylation	107317	PREDICTED: serine/threonine-protein phosphatase BSL3 [Vitis vinifera]
1381	1304	gi|359475330	78.53	14	2	2		16329	PREDICTED: glycine-rich RNA-binding protein GRP2A [Vitis vinifera]
2479	29080	gi|566191056	78.49	6	2	2	Carbamidomethylation	53716	serine carboxypeptidase S10 family protein [Populus trichocarpa]
1686	2088	gi|731379285	78.45	15	3	3		27000	PREDICTED: probable phospholipid hydroperoxide glutathione peroxidase [Vitis vinifera]
756	992	gi|225441912	78.41	12	4	2		30951	PREDICTED: S-formylglutathione hydrolase isoform X4 [Vitis vinifera]
1622	18313	gi|224053607	78.4	11	3	1		34050	EXGT1 family protein [Populus trichocarpa]
1512	857	gi|225424598	78.13	16	3	3	Carbamidomethylation	29498	PREDICTED: proteasome subunit beta type-7-A [Vitis vinifera]
1610	3914	gi|225428111	78.12	9	3	1		39867	PREDICTED: 24-methylenesterol C-methyltransferase 2 [Vitis vinifera]
1367	2725	gi|359475312	77.9	6	4	2		50616	PREDICTED: cyclase-associated protein 1 [Vitis vinifera]
1687	29004	gi|566163578	77.89	1	3	1		212580	hypothetical protein POPTR_0003s19630g [Populus trichocarpa]

2350	29029	gi|224111756	77.85	4	2	2		67310	hypothetical protein POPTR_0010s14130g [Populus trichocarpa]
1684	1904	gi|225431685	77.82	5	4	1		92333	PREDICTED: probable methyltransferase PMT26 [Vitis vinifera]
2163	23457	gi|731425791	77.82	8	3	3		30802	PREDICTED: NAP1-related protein 2 [Vitis vinifera]
1864	18439	gi|224081475	77.68	5	2	2		58488	hypothetical protein POPTR_0005s10320g [Populus trichocarpa]
1651	1526	gi|225440674	77.68	9	2	1	Carbamidomethylation	31431	PREDICTED: chlorophyll a-b binding protein CP29.1 chloroplastic [Vitis vinifera]
1922	1847	gi|731389447	77.64	4	2	1		84064	PREDICTED: acylamino-acid-releasing enzyme isoform X2 [Vitis vinifera]
2082	29002	gi|566239233	77.61	4	2	2		94826	aspartate kinase family protein [Populus trichocarpa]
1888	17748	gi|566214622	77.59	5	2	1		51313	WD-40 repeat protein MSI4 [Populus trichocarpa]
1840	18330	gi|566234718	77.45	2	2	1		124541	exportin1 family protein [Populus trichocarpa]
1536	837	gi|731425891	77.45	12	3	2		31554	PREDICTED: hypersensitive-induced response protein 1 isoform X2 [Vitis vinifera]
1770	17545	gi|566169569	77.43	2	2	1	Carbamidomethylation	117048	transport Sec24 family protein [Populus trichocarpa]
1820	18205	gi|224054120	77.31	5	3	1		73852	lecithin:cholesterol acyltransferase family protein [Populus trichocarpa]
2010	2331	gi|731432527	77.31	10	2	2		26117	PREDICTED: DAG protein chloroplastic [Vitis vinifera]
1938	17861	gi|566214720	77.15	10	2	1		27275	Eukaryotic translation initiation factor 3 subunit 11 family protein [Populus trichocarpa]
1805	1796	gi|225434189	77.1	9	3	1	Carbamidomethylation	38648	PREDICTED: 2-alkenal reductase (NADP(+)-dependent) [Vitis vinifera]
1432	18489	gi|224080381	77.08	8	3	3		53896	diaminopimelate decarboxylase family protein [Populus trichocarpa]

1343	18959	gi|566176600	77.04	8	4	1		60686	hypothetical protein POPTR_0006s15730g [Populus trichocarpa]
1776	1975	gi|731432424	77.02	4	4	4		90087	PREDICTED: ubiquitin carboxyl-terminal hydrolase 14 [Vitis vinifera]
1633	18136	gi|224109362	76.98	10	2	1		31536	Eukaryotic translation initiation factor 3 subunit 5 family protein [Populus trichocarpa]
1994	2000	gi|731418793	76.93	10	5	1		59155	PREDICTED: glucose-6-phosphate 1-dehydrogenase cytoplasmic isoform [Vitis vinifera]
1965	28917	gi|225454787	76.88	8	4	3		61899	PREDICTED: 4-coumarate--CoA ligase 2 [Vitis vinifera]
1844	19010	gi|566191106	76.85	3	2	1	Carbamidomethylation	83138	hypothetical protein POPTR_0010s16220g [Populus trichocarpa]
2345	1879	gi|359492060	76.75	7	3	3	Carbamidomethylation	46276	PREDICTED: polyadenylate-binding protein RBP47-like [Vitis vinifera]
1320	17944	gi|224093408	76.74	13	2	1	Carbamidomethylation	18464	hypothetical protein POPTR_0007s04160g [Populus trichocarpa]
1485	18451	gi|224089557	76.66	8	3	1	Carbamidomethylation	42428	truncated acetyl Co-A acetyltransferase-like family protein [Populus trichocarpa]
2067	1203	gi|731391355	76.62	5	3	3		85892	PREDICTED: LETM1 and EF-hand domain-containing protein 1 mitochondrial [Vitis vinifera]
1727	28910	gi|566206175	76.44	12	4	1	Carbamidomethylation	41940	Actin-related family protein [Populus trichocarpa]
2150	28945	gi|225445462	76.34	24	3	1		14555	PREDICTED: uncharacterized protein At2g34160 [Vitis vinifera]
1951	18690	gi|566167629	76.29	8	2	2	Carbamidomethylation	40491	homoserine dehydrogenase family protein [Populus trichocarpa]
1253	1075	gi|526117894	76.2	8	4	1		36735	anthocyanidin reductase [Vitis vinifera]
1649	28950	gi|359483854	76.02	6	3	1	Carbamidomethylation	49342	PREDICTED: 3-oxoacyl-[acyl-carrier-protein] synthase I chloroplastic [Vitis vinifera]

1284	28933	gi|566151363	76	4	4	1		120035	C2 domain-containing family protein [Populus trichocarpa]
1695	18180	gi|566186687	75.9	9	3	1		39152	hypothetical protein POPTR_0009s06780g [Populus trichocarpa]
1697	18176	gi|566175819	75.85	6	3	1		67279	hypothetical protein POPTR_0006s11980g [Populus trichocarpa]
2272	19555	gi|566215053	75.84	12	3	1		30007	hypothetical protein POPTR_0018s09700g [Populus trichocarpa]
738	17884	gi|224066487	75.78	14	3	3		17636	hypothetical protein POPTR_0002s05220g [Populus trichocarpa]
1698	19081	gi|566197479	75.7	6	2	1		60216	ethylene-responsive DEAD box RNA helicase family protein [Populus trichocarpa]
1022	1812	gi|225430510	75.68	6	3	1		27121	PREDICTED: phospholipid hydroperoxide glutathione peroxidase 1 chloroplastic [Vitis vinifera]
1769	29034	gi|566203845	75.65	9	2	2		41752	hypothetical protein POPTR_0014s11810g [Populus trichocarpa]
1679	1439	gi|731428465	75.63	8	4	4		37487	PREDICTED: thylakoid lumenal 29 kDa protein chloroplastic [Vitis vinifera]
1923	2049	gi|731411015	75.61	8	2	1		46869	PREDICTED: 3-isopropylmalate dehydrogenase chloroplastic [Vitis vinifera]
2134	29290	gi|224141019	75.6	6	2	1		57002	cystathionine gamma synthase family protein [Populus trichocarpa]
1529	18144	gi|224073967	75.6	12	2	2		15188	photosystem I 11K family protein [Populus trichocarpa]
1578	1299	gi|731419061	75.56	3	2	1		94432	PREDICTED: ATP-dependent RNA helicase-like protein DB10 isoform X2 [Vitis vinifera]
1734	1567	gi|225433215	75.43	9	2	2	Carbamidomethylation	26277	PREDICTED: DAG protein chloroplastic [Vitis vinifera]

2077	1581	gi|731408703	75.36	7	3	3	Carbamidomethylation	54961	PREDICTED: serine/threonine-protein phosphatase 5 isoform X2 [Vitis vinifera]
2156	18462	gi|566192083	75.3	3	2	1	Carbamidomethylation	111427	transport protein Sec24 [Populus trichocarpa]
1563	1382	gi|359489656	75.3	6	2	2		52031	PREDICTED: probable polygalacturonase [Vitis vinifera]
1936	2203	gi|731387671	75.28	7	2	2	Carbamidomethylation	39674	PREDICTED: thioredoxin reductase NTRB-like [Vitis vinifera]
1908	20223	gi|225456266	75.26	6	4	1		53511	PREDICTED: probable aspartyl aminopeptidase [Vitis vinifera]
2250	1362	gi|225440230	75.24	10	2	2	Carbamidomethylation	29238	PREDICTED: dihydrofolate reductase [Vitis vinifera]
2149	28941	gi|225469630	75.13	8	3	2		61225	PREDICTED: EH domain-containing protein 3-like [Vitis vinifera]
1144	920	gi|225436438	75.1	34	3	3		10607	PREDICTED: cyclin-dependent kinases regulatory subunit 1 [Vitis vinifera]
1138	18449	gi|566155267	75.08	5	3	2		41202	hypothetical protein POPTR_0001s46720g [Populus trichocarpa]
1616	18677	gi|566167809	74.96	9	2	1		40197	ISOCITRATE DEHYDROGENASE SUBUNIT 2 family protein [Populus trichocarpa]
2584	2872	gi|359483619	74.84	17	2	2		15087	PREDICTED: prefoldin subunit 6 [Vitis vinifera]
951	18170	gi|566157284	74.7	4	2	1	Carbamidomethylation	48713	chloroplast nucleoid DNA-binding family protein [Populus trichocarpa]
1678	18410	gi|566146519	74.69	3	3	1		118989	glycosyl hydrolase family 31 family protein [Populus trichocarpa]
1309	18273	gi|224106686	74.69	10	2	1		24434	elongation factor 1B alpha-subunit 2 family protein [Populus trichocarpa]
2541	17937	gi|224101127	74.46	10	2	1	Carbamidomethylation	30103	Chlorophyll a-b binding protein CP29.3 [Populus trichocarpa]
2586	18467	gi|566253285	74.44	9	2	1		36419	hypothetical protein POPTR_1567s00200g partial [Populus trichocarpa]

1590	1079	gi|225445949	74.3	8	4	3	Carbamidomethylation	49372	PREDICTED: KH domain-containing protein At4g18375 [Vitis vinifera]
981	17667	gi|224086942	74.19	12	4	2	Carbamidomethylation	31883	putative esterase D family protein [Populus trichocarpa]
1690	18861	gi|566159684	74.19	4	2	1	Oxidation (M)	72140	hypothetical protein POPTR_0002s23950g [Populus trichocarpa]
2093	28935	gi|225459762	74.17	9	3	1		40307	PREDICTED: 24-methylenesterol C-methyltransferase 2 [Vitis vinifera]
1667	979	gi|731416996	74.11	7	3	2		46827	PREDICTED: poly(rC)-binding protein 3 [Vitis vinifera]
2251	1725	gi|225430834	74.11	4	2	1		71703	PREDICTED: probable Xaa-Pro aminopeptidase P [Vitis vinifera]
1639	1412	gi|225427234	74.08	14	2	1		17554	PREDICTED: glycine cleavage system H protein mitochondrial [Vitis vinifera]
1557	1384	gi|225437898	74.04	15	3	2		15168	PREDICTED: photosystem I reaction center subunit IV B chloroplastic-like [Vitis vinifera]
1652	18675	gi|566175530	74.02	8	2	1	Carbamidomethylation	39660	hypothetical protein POPTR_0006s10360g [Populus trichocarpa]
2097	5417	gi|731391337	73.95	9	3	1	Carbamidomethylation	45764	PREDICTED: mitogen-activated protein kinase homolog MMK1 [Vitis vinifera]
1898	19056	gi|731418338	73.76	3	2	2		84933	PREDICTED: acetyl-coenzyme A synthetase chloroplastic/glyoxysomal [Vitis vinifera]
1750	1309	gi|731398937	73.54	15	2	1	Carbamidomethylation	21183	PREDICTED: actin-depolymerizing factor 1-like [Vitis vinifera]
2087	1375	gi|359487733	73.48	6	2	1		46705	PREDICTED: amidase 1 [Vitis vinifera]
1867	1545	gi|225451299	73.42	10	2	2		30245	PREDICTED: probable ribose-5-phosphate isomerase 3 chloroplastic [Vitis vinifera]

1848	29179	gi|566195867	73.33	6	2	1		52643	hypothetical protein POPTR_0011s16630g [Populus trichocarpa]
2268	19500	gi|224074887	73.32	8	2	1		36489	replication factor C 40 kDa family protein [Populus trichocarpa]
1949	17722	gi|566203642	73.3	8	3	3	Carbamidomethylation	61529	hypothetical protein POPTR_0014s10720g [Populus trichocarpa]
2219	5132	gi|225442452	73.29	4	2	1		54242	PREDICTED: hexokinase-2 [Vitis vinifera]
1715	18678	gi|566210985	73.26	6	2	1	Carbamidomethylation	51941	glycosyl hydrolase family 17 family protein [Populus trichocarpa]
2459	900	gi|225446797	73.17	5	2	2		55221	PREDICTED: switch-associated protein 70 [Vitis vinifera]
1438	17581	gi|566187534	73.13	5	3	1		50027	cyclase associated protein 1 [Populus trichocarpa]
1599	998	gi|225435024	73	13	3	2		25842	PREDICTED: THO complex subunit 4A [Vitis vinifera]
2816	29115	gi|566149039	72.99	12	2	1	Carbamidomethylation	22002	hypothetical protein POPTR_0001s14940g partial [Populus trichocarpa]
1983	29203	gi|225440590	72.94	8	2	1	Carbamidomethylation	32488	PREDICTED: mitochondrial uncoupling protein 1 [Vitis vinifera]
2184	18308	gi|731406703	72.84	7	3	3	Carbamidomethylation; Oxidation (M)	66565	PREDICTED: arginine--tRNA ligase cytoplasmic-like isoform X2 [Vitis vinifera]
1893	1576	gi|225427798	72.82	6	3	1	Carbamidomethylation	62899	PREDICTED: amidophosphoribosyltransferase chloroplastic [Vitis vinifera]
2478	2043	gi|225428141	72.57	5	2	2		68945	PREDICTED: SEC1 family transport protein SLY1 [Vitis vinifera]
1934	17898	gi|566255384	72.54	8	2	1		37527	hypothetical protein POPTR_0584s00210g [Populus trichocarpa]
1788	18006	gi|224141351	72.48	3	2	1		68914	Structure-specific recognition protein 1 [Populus trichocarpa]
2197	1499	gi|225456248	72.47	9	2	1		34358	PREDICTED: 3-oxoacyl-[acyl-carrier-protein] reductase 4 [Vitis vinifera]

2352	29220	gi|224084852	72.42	44	2	2		10061	hypothetical protein POPTR_0005s16040g [Populus trichocarpa]
1613	28977	gi|224092186	72.41	17	3	3	Carbamidomethylation	21905	eukaryotic translation initiation factor SUI1 family protein [Populus trichocarpa]
2123	18710	gi|224065729	72.37	15	2	2		24619	hypothetical protein POPTR_0002s01660g [Populus trichocarpa]
1597	28918	gi|566164041	72.35	5	4	1		88406	hypothetical protein POPTR_0003s22060g [Populus trichocarpa]
2088	19366	gi|566184879	72.33	19	2	1		19164	hypothetical protein POPTR_0008s19410g [Populus trichocarpa]
2154	18206	gi|566207525	72.3	4	3	1	Carbamidomethylation	83959	subtilase family protein [Populus trichocarpa]
2098	19938	gi|566166867	71.98	5	3	3		59346	dihydrofolate reductase-thymidylate synthase family protein [Populus trichocarpa]
1659	19350	gi|224073782	71.96	9	1	1	Carbamidomethylation	21868	proline-rich family protein [Populus trichocarpa]
1956	956	gi|225434949	71.91	26	2	1		10898	PREDICTED: mitochondrial import inner membrane translocase subunit Tim9 [Vitis vinifera]
2002	1623	gi|225437378	71.84	17	2	2	Carbamidomethylation	11879	PREDICTED: small nuclear ribonucleoprotein Sm D2-like [Vitis vinifera]
1809	2410	gi|731412185	71.8	6	2	1	Carbamidomethylation	67073	PREDICTED: phosphoinositide phospholipase C 6 [Vitis vinifera]
1953	2576	gi|731430343	71.67	7	2	1	Carbamidomethylation; Oxidation (M)	47441	PREDICTED: histidinol dehydrogenase chloroplastic isoform X7 [Vitis vinifera]
1866	1021	gi|731389102	71.63	7	2	2	Carbamidomethylation	39883	PREDICTED: ATP-dependent (S)-NAD(P)H-hydrate dehydratase [Vitis vinifera]
2273	29082	gi|526117529	71.54	10	2	2	Carbamidomethylation	27234	caffeoyl-CoA O-methyltransferase [Vitis vinifera]
2130	29186	gi|225430017	71.46	5	2	1		52717	PREDICTED: la protein 1 [Vitis vinifera]

1464	1262	gi|731436828	71.45	10	3	2	Carbamidomethylation	57638	PREDICTED: ATP-dependent 6-phosphofructokinase 5 chloroplastic-like [Vitis vinifera]
1931	1686	gi|225456369	71.41	15	3	3		23558	PREDICTED: nuclear transcription factor Y subunit C-1 isoform X1 [Vitis vinifera]
601	1367	gi|526118089	71.36	11	3	3		17263	type II peroxiredoxin C [Vitis vinifera]
1928	28916	gi|359474156	71.19	10	4	1		54166	PREDICTED: monodehydroascorbate reductase chloroplastic isoform X2 [Vitis vinifera]
1975	2401	gi|225449382	71.18	11	3	3		33427	PREDICTED: eukaryotic translation initiation factor 3 subunit G [Vitis vinifera]
1976	19002	gi|224103819	71.14	7	2	2		34955	L-galactose dehydrogenase family protein [Populus trichocarpa]
2206	18458	gi|566160637	71.11	3	2	1		89989	hypothetical protein POPTR_0003s03120g [Populus trichocarpa]
1728	1845	gi|225434277	71.06	13	3	3		22623	PREDICTED: CBS domain-containing protein CBSX3 mitochondrial [Vitis vinifera]
1700	1798	gi|225442268	71	3	2	1		73167	PREDICTED: transmembrane 9 superfamily member 7 [Vitis vinifera]
2027	18628	gi|566194620	70.95	1	2	2	Carbamidomethylation	411805	hypothetical protein POPTR_0011s09640g [Populus trichocarpa]
1467	2306	gi|526117918	70.9	5	1	1		33457	basic endochitinase precursor [Vitis vinifera]
1601	18207	gi|224077052	70.88	2	2	2		121102	guanylate-binding family protein [Populus trichocarpa]
655	1307	gi|225463506	70.8	7	2	2		38294	PREDICTED: fructose-bisphosphate aldolase cytoplasmic isozyme 1 [Vitis vinifera]
1382	18711	gi|224128708	70.78	7	2	1		33828	xyloglucan endo-1 family protein [Populus trichocarpa]
792	1269	gi|225459906	70.77	5	2	1		34942	PREDICTED: probable fructokinase-5 [Vitis vinifera]

2157	19551	gi|224073260	70.77	23	2	2		10707	small nuclear ribonucleoprotein D [Populus trichocarpa]
758	18717	gi|224059156	70.74	18	2	1		14119	Profilin 3 family protein [Populus trichocarpa]
2346	20591	gi|566167733	70.74	12	3	3		33452	NITRILASE-LIKE protein 1 [Populus trichocarpa]
1130	28982	gi|225432556	70.7	9	4	3	Carbamidomethylation	49609	PREDICTED: NEDD8-activating enzyme E1 catalytic subunit [Vitis vinifera]
2004	1611	gi|731417625	70.61	12	2	2		19282	PREDICTED: uncharacterized protein LOC100241624 [Vitis vinifera]
2115	1643	gi|225452186	70.61	9	3	3		30725	PREDICTED: prohibitin-3 mitochondrial [Vitis vinifera]
427	2534	gi|731417951	70.58	8	2	2		27594	PREDICTED: allene oxide cyclase [Vitis vinifera]
1356	20587	gi|566186995	70.49	11	3	3		33415	hypothetical protein POPTR_0009s08480g [Populus trichocarpa]
1689	1218	gi|731413519	70.44	5	2	2		48243	PREDICTED: vacuolar protein sorting-associated protein 4 [Vitis vinifera]
1220	18715	gi|224107783	70.44	12	2	1	Carbamidomethylation	16476	hypothetical protein POPTR_0010s05590g [Populus trichocarpa]
471	18787	gi|224146534	70.39	9	3	1	Oxidation (M)	29228	hypothetical protein POPTR_0019s12390g [Populus trichocarpa]
2275	1165	gi|225428566	70.3	5	2	1	Carbamidomethylation	52523	PREDICTED: 4-hydroxy-3-methylbut-2-enyl diphosphate reductase chloroplastic [Vitis vinifera]
2055	18879	gi|566193798	70.11	3	2	2		49306	thioredoxin family protein [Populus trichocarpa]
2036	1192	gi|731382587	70.07	2	2	1		119503	PREDICTED: epidermal growth factor receptor substrate 15-like 1 [Vitis vinifera]
1927	1971	gi|359492139	70.05	5	4	1		94211	PREDICTED: probable methyltransferase PMT24 [Vitis vinifera]

1837	2961	gi|225429736	70	6	3	3	Carbamidomethylation	60112	PREDICTED: 3-ketoacyl-CoA synthase 10 [Vitis vinifera]
2539	29030	gi|224110036	69.96	9	2	1		30282	uridylyltransferase-related family protein [Populus trichocarpa]
2266	18407	gi|566158256	69.96	8	3	2	Carbamidomethylation	39552	hypothetical protein POPTR_0002s15850g [Populus trichocarpa]
2120	18682	gi|224146371	69.96	3	2	1		63347	putative beta-fructofuranosidase family protein [Populus trichocarpa]
2511	29064	gi|225426649	69.94	8	2	2		35134	PREDICTED: uncharacterized protein LOC100244375 [Vitis vinifera]
2296	18765	gi|566188404	69.81	9	3	3	Carbamidomethylation	40604	pantothenate kinase-related family protein [Populus trichocarpa]
1849	1609	gi|225437963	69.76	6	2	2		43931	PREDICTED: NADH dehydrogenase [ubiquinone] 1 alpha subcomplex subunit 9 mitochondrial [Vitis vinifera]
2213	19437	gi|566192561	69.66	7	2	1		32755	hypothetical protein POPTR_0010s24480g [Populus trichocarpa]
2046	1861	gi|731428311	69.55	9	3	2		51458	PREDICTED: bifunctional aspartate aminotransferase and glutamate/aspartate-prephenate aminotransferase-like [Vitis vinifera]
1904	921	gi|731382079	69.49	3	3	1		110376	PREDICTED: neutral alpha-glucosidase C-like isoform X2 [Vitis vinifera]
2109	19699	gi|134093226	69.4	7	1	1		22041	ATP-dependent Clp protease proteolytic subunit (chloroplast) [Populus trichocarpa]
1370	858	gi|359475902	69.33	7	4	3	Carbamidomethylation	61615	PREDICTED: protein TIC 55 chloroplastic [Vitis vinifera]
1977	18384	gi|224146080	69.33	14	2	2		17003	hypothetical protein POPTR_0019s05840g [Populus trichocarpa]
1912	2137	gi|359477212	69.32	3	2	2		91584	PREDICTED: uncharacterized protein LOC100258488 isoform X1 [Vitis vinifera]

1819	18341	gi|224066611	69.28	14	3	2	Carbamidomethylation	38531	peroxidase precursor family protein [Populus trichocarpa]
1731	19841	gi|224136125	69.22	13	3	2		20526	60S ribosomal protein L17-1 [Populus trichocarpa]
2477	19200	gi|566163352	69.21	4	2	2		85586	hypothetical protein POPTR_0003s18380g [Populus trichocarpa]
2936	29208	gi|225465292	69.17	8	2	2		33830	PREDICTED: 2-C-methyl-D-erythritol 4-phosphate cytidylyltransferase chloroplastic [Vitis vinifera]
1932	18581	gi|566256880	69.08	4	3	2		62963	hypothetical protein POPTR_0214s00200g [Populus trichocarpa]
1810	17950	gi|224105287	69.04	8	2	2		34749	NADP dependent sorbitol 6-phosphate dehydrogenase family protein [Populus trichocarpa]
2317	29275	gi|225450096	69.02	9	2	2	Carbamidomethylation	39782	PREDICTED: actin-related protein 7 [Vitis vinifera]
1909	1486	gi|225465204	69	3	3	2	Carbamidomethylation	122695	PREDICTED: leucine--tRNA ligase cytoplasmic [Vitis vinifera]
1841	29013	gi|224099725	68.9	5	2	1		69331	hypothetical protein POPTR_0008s14720g [Populus trichocarpa]
1937	2020	gi|731374917	68.89	3	1	1		45060	PREDICTED: serine/threonine-protein kinase TNNI3K isoform X2 [Vitis vinifera]
2228	1763	gi|731440118	68.84	3	3	2	Carbamidomethylation	144044	PREDICTED: kinesin-like protein KCA2 [Vitis vinifera]
1554	29204	gi|225458782	68.8	7	2	1		44534	PREDICTED: 3-oxo-Delta(4 5)-steroid 5-beta-reductase-like [Vitis vinifera]
1847	2197	gi|526118093	68.8	8	1	1	Carbamidomethylation	22511	type II peroxiredoxin E [Vitis vinifera]
1623	18835	gi|224089058	68.66	8	3	1		49180	aspartate transaminase family protein [Populus trichocarpa]
2538	881	gi|225433707	68.64	2	2	2		118913	PREDICTED: probable importin-7 homolog [Vitis vinifera]

2305	2091	gi|225450155	68.63	9	2	2		26703	PREDICTED: thylakoid lumenal 19 kDa protein chloroplastic [Vitis vinifera]
1905	18310	gi|224143674	68.61	11	3	3		38385	fructose-1 family protein [Populus trichocarpa]
2501	28948	gi|566151358	68.6	3	3	3		154446	hypothetical protein POPTR_0001s27800g [Populus trichocarpa]
1913	18538	gi|224090627	68.6	2	2	1	Carbamidomethylation	157531	WD-40 repeat family protein [Populus trichocarpa]
2126	2753	gi|225429582	68.58	2	2	2		98913	PREDICTED: aminopeptidase M1 [Vitis vinifera]
2418	19026	gi|566229479	68.45	2	3	3	Carbamidomethylation	283021	hypothetical protein POPTR_0019s04090g [Populus trichocarpa]
2101	29028	gi|225452161	68.41	2	2	2	Carbamidomethylation	130963	PREDICTED: protein TPLATE [Vitis vinifera]
1858	28957	gi|224085670	68.22	21	4	4		15447	60S ribosomal protein L14 [Populus trichocarpa]
2298	19932	gi|731430611	68.18	8	3	1		48137	PREDICTED: protease Do-like 1 chloroplastic [Vitis vinifera]
2270	1696	gi|225428556	68	4	2	1		48755	PREDICTED: dolichyl-diphosphooligosaccharide--protein glycosyltransferase 48 kDa subunit isoform X2 [Vitis vinifera]
1845	1562	gi|224365629	67.99	6	2	2		43947	apocytochrome b (mitochondrion) [Vitis vinifera]
2267	18214	gi|566175269	67.78	11	3	3		42962	hypothetical protein POPTR_0006s08830g [Populus trichocarpa]
1778	19496	gi|224078630	67.73	5	2	1		62205	ATP-binding-cassette transporter family protein [Populus trichocarpa]
2587	29200	gi|225447109	67.65	10	2	2	Carbamidomethylation	24636	PREDICTED: protein polybromo-1 [Vitis vinifera]
2231	29055	gi|224069814	67.6	9	2	2		28786	Ni-binding urease accessory protein UreG [Populus trichocarpa]
1067	2307	gi|225441664	67.51	5	1	1		36307	PREDICTED: uncharacterized protein LOC100255512 [Vitis vinifera]

2265	1670	gi|225436339	67.46	10	3	3		45109	PREDICTED: arogenate dehydratase 3 chloroplastic [Vitis vinifera]
2303	894	gi|225444609	67.45	9	2	1		26208	PREDICTED: eukaryotic translation initiation factor 3 subunit K [Vitis vinifera]
2447	19489	gi|224095005	67.38	7	2	2	Carbamidomethylation	33972	xyloglucan endotransglycosylase hydrolase 2 family protein [Populus trichocarpa]
1812	18418	gi|566234529	67.37	22	2	1	Carbamidomethylation	13103	hypothetical protein POPTR_0019s07730g [Populus trichocarpa]
1968	28967	gi|566209280	67.35	6	3	1		52718	hypothetical protein POPTR_0016s08010g [Populus trichocarpa]
2304	22198	gi|224096918	67.33	4	2	1		48882	dolichyl-diphosphooligosaccharide-protein glycosyltransferase 48kDa subunit [Populus trichocarpa]
2696	22799	gi|224077880	67.29	4	2	1		82133	beta-galactosidase family protein [Populus trichocarpa]
1603	19042	gi|566210832	67.28	7	1	1		25245	hypothetical protein POPTR_0017s02160g [Populus trichocarpa]
2320	29340	gi|225436922	67.14	5	2	1	Carbamidomethylation	59280	PREDICTED: RAN GTPase-activating protein 1 [Vitis vinifera]
1753	1946	gi|225440849	67.09	28	2	2		8952	PREDICTED: probable small nuclear ribonucleoprotein G [Vitis vinifera]
2153	28984	gi|566256791	67.09	5	3	1	Carbamidomethylation; Oxidation (M)	67694	hypothetical protein POPTR_0231s00220g [Populus trichocarpa]
1706	1358	gi|731409226	66.89	4	2	1		43860	PREDICTED: 3-hydroxyisobutyryl-CoA hydrolase-like protein 5 [Vitis vinifera]
1465	18856	gi|224099491	66.81	24	2	1		10451	hypothetical protein POPTR_0008s12950g [Populus trichocarpa]
1036	1101	gi|225434062	66.78	15	4	2	Carbamidomethylation	28430	PREDICTED: chitinase 5-like [Vitis vinifera]

1961	19481	gi|224122412	66.76	7	1	1		23172	cytochrome b5 domain-containing family protein [Populus trichocarpa]
1620	19286	gi|566208913	66.75	3	2	1		37900	hypothetical protein POPTR_0016s05800g [Populus trichocarpa]
1592	1371	gi|225469850	66.73	6	2	2		39160	PREDICTED: uncharacterized protein DDB_G0288133 [Vitis vinifera]
1751	18847	gi|224141239	66.73	16	2	1		15020	hypothetical protein POPTR_0017s08140g [Populus trichocarpa]
1764	18970	gi|566167505	66.68	2	1	1		112715	adaptin family protein [Populus trichocarpa]
1920	18595	gi|566190935	66.63	5	2	1	Carbamidomethylation	61141	hypothetical protein POPTR_0010s15200g [Populus trichocarpa]
1897	18854	gi|224077654	66.55	3	3	1		172709	DNA (cytosine-5)-methyltransferase AthI family protein [Populus trichocarpa]
2542	18575	gi|566153712	66.53	5	2	2		52249	transport protein sec61 [Populus trichocarpa]
2062	1272	gi|225440480	66.49	10	3	1		35139	PREDICTED: aldo-keto reductase family 4 member C9-like [Vitis vinifera]
2204	29022	gi|225454115	66.42	2	2	2		131061	PREDICTED: K(+) efflux antiporter 2 chloroplastic [Vitis vinifera]
2013	19680	gi|566152777	66.28	15	1	1	Carbamidomethylation	12696	hypothetical protein POPTR_0001s34690g [Populus trichocarpa]
2297	28946	gi|224077626	66.02	7	3	1	Carbamidomethylation	50063	3-oxoacyl-[acyl-carrier-protein] synthase I [Populus trichocarpa]
2585	20382	gi|225439033	65.98	2	2	1	Carbamidomethylation	139714	PREDICTED: uncharacterized protein LOC100248418 [Vitis vinifera]
1807	18287	gi|224120380	65.95	6	3	3		61938	hypothetical protein POPTR_0012s03790g [Populus trichocarpa]
2509	18295	gi|224107711	65.84	8	2	2		35785	b-keto acyl reductase family protein [Populus trichocarpa]

1829	2131	gi|731388570	65.82	2	2	1		125817	PREDICTED: DEAD-box ATP-dependent RNA helicase 46 isoform X2 [Vitis vinifera]
2131	29187	gi|225464130	65.81	11	2	2	Carbamidomethylation	30020	PREDICTED: nifU-like protein 4 mitochondrial [Vitis vinifera]
2125	29101	gi|731379074	65.66	4	2	1		72114	PREDICTED: probable methyltransferase PMT18 isoform X2 [Vitis vinifera]
1068	1967	gi|731424493	65.55	7	2	2		21410	PREDICTED: protein MATERNALLY EXPRESSED GENE 5 isoform X3 [Vitis vinifera]
2063	18114	gi|566209593	65.55	10	3	1		36623	hypothetical protein POPTR_0016s10390g [Populus trichocarpa]
1635	17842	gi|224057968	65.48	13	2	1	Carbamidomethylation	24349	photosystem I chain III family protein [Populus trichocarpa]
1760	29039	gi|731391349	65.45	3	2	2		73345	PREDICTED: UDP-glycosyltransferase 74E2 [Vitis vinifera]
2583	18891	gi|566152365	65.37	7	3	2		43020	Vacuolar ATP synthase subunit C family protein [Populus trichocarpa]
2194	1805	gi|731402392	65.35	3	1	1		65873	PREDICTED: malonate--CoA ligase isoform X3 [Vitis vinifera]
1842	29021	gi|224103461	65.32	3	2	1		84015	hypothetical protein POPTR_0009s11520g [Populus trichocarpa]
1921	2019	gi|225431914	65.18	4	2	1		60422	PREDICTED: dihydrolipoyl dehydrogenase 2 chloroplastic [Vitis vinifera]
2461	18123	gi|566212877	65.01	3	1	1	Carbamidomethylation	57107	hypothetical protein POPTR_0017s12170g [Populus trichocarpa]
1758	17798	gi|224146178	64.92	4	2	1	Carbamidomethylation	49470	hypothetical protein POPTR_0019s08290g [Populus trichocarpa]
1457	19656	gi|566169467	64.9	9	1	1		13987	hypothetical protein POPTR_0005s04590g [Populus trichocarpa]

1871	29301	gi|224080283	64.78	3	2	1	Carbamidomethylation	84128	hypothetical protein POPTR_0004s15790g [Populus trichocarpa]
1917	28914	gi|566148153	64.74	9	4	1		54002	Monodehydroascorbate reductase family protein [Populus trichocarpa]
2106	1951	gi|731402186	64.73	7	1	1		24523	PREDICTED: vacuolar protein sorting-associated protein 32 homolog 2 [Vitis vinifera]
2079	28966	gi|566177670	64.69	6	3	1		52363	RNA recognition motif-containing family protein [Populus trichocarpa]
1147	1956	gi|225442434	64.69	18	2	1		14228	PREDICTED: profilin-1 [Vitis vinifera]
1088	1642	gi|225462170	64.61	12	2	2		15615	PREDICTED: 60S ribosomal protein L27 [Vitis vinifera]
1413	29053	gi|225448156	64.61	4	2	1		42820	PREDICTED: ATP phosphoribosyltransferase 2 chloroplastic [Vitis vinifera]
1900	1894	gi|359480888	64.6	10	2	1		39762	PREDICTED: RNA-binding protein Musashi homolog 2 [Vitis vinifera]
1538	29005	gi|224084784	64.52	3	2	1		61214	glycine hydroxymethyltransferase family protein [Populus trichocarpa]
2127	29111	gi|731435721	64.51	3	2	2		70515	PREDICTED: uncharacterized protein LOC100257572 isoform X2 [Vitis vinifera]
1249	18948	gi|566197644	64.46	12	2	1		26318	hypothetical protein POPTR_0012s09540g [Populus trichocarpa]
1942	19687	gi|224089062	64.43	6	1	1		27753	hypothetical protein POPTR_0006s25870g [Populus trichocarpa]
2356	2259	gi|225431922	64.39	19	2	2	Carbamidomethylation	13225	PREDICTED: DNA-directed RNA polymerases II IV and V subunit 9A [Vitis vinifera]
1735	18662	gi|566201753	64.27	9	2	2	Carbamidomethylation	34979	hypothetical protein POPTR_0014s01090g [Populus trichocarpa]
1862	18423	gi|224056200	64.12	6	3	2	Carbamidomethylation	48114	5-methylthioribose kinase family protein [Populus trichocarpa]
2052	29003	gi|225436363	64.1	2	2	2		113112	PREDICTED: AP-2 complex subunit alpha-1 [Vitis vinifera]

714	1227	gi|225443962	64.1	3	3	3		81691	PREDICTED: CSC1-like protein ERD4 [Vitis vinifera]
1839	28965	gi|359487628	64.02	24	3	1		14010	PREDICTED: 60S ribosomal protein L22-2 [Vitis vinifera]
852	18297	gi|566192144	63.76	6	2	2		36328	hypothetical protein POPTR_0010s22170g [Populus trichocarpa]
2368	29822	gi|225449062	63.75	10	2	1		12458	PREDICTED: V-type proton ATPase subunit G1 [Vitis vinifera]
2069	19309	gi|224097184	63.58	4	2	1		73308	Phosphoenolpyruvate carboxykinase family protein [Populus trichocarpa]
1906	17849	gi|566211354	63.58	11	3	1		34355	phosphatase 2C family protein [Populus trichocarpa]
2310	18228	gi|224065629	63.52	15	2	2	Carbamidomethylation	16418	CP12 domain-containing family protein [Populus trichocarpa]
1830	2040	gi|731428304	63.47	2	1	1		81014	PREDICTED: apoptotic chromatin condensation inducer in the nucleus-like isoform X2 [Vitis vinifera]
1954	29099	gi|225432812	63.44	4	2	2		52461	PREDICTED: ATP sulfurylase 1 chloroplastic [Vitis vinifera]
2714	18397	gi|566177989	63.43	5	2	1		50804	hypothetical protein POPTR_0006s24710g [Populus trichocarpa]
1492	2852	gi|731410160	63.34	4	2	2	Carbamidomethylation	44936	PREDICTED: transcription initiation factor TFIID subunit 15b isoform X2 [Vitis vinifera]
2070	2338	gi|359496232	63.32	8	1	1		21717	PREDICTED: probable NAD(P)H dehydrogenase (quinone) FQR1-like 1 [Vitis vinifera]
2223	2415	gi|225450075	63.24	6	2	1		41952	PREDICTED: peptidyl-prolyl cis-trans isomerase FKBP42 [Vitis vinifera]
2349	2595	gi|359473679	63.12	6	2	2	Carbamidomethylation	35252	PREDICTED: uncharacterized protein LOC100852583 [Vitis vinifera]
2508	1566	gi|731438444	63.11	5	2	2	Carbamidomethylation	51427	PREDICTED: ruvB-like 2 [Vitis vinifera]
2547	29147	gi|731421096	63.07	6	2	2	Carbamidomethylation	40246	PREDICTED: ATPase ASNA1 homolog isoform X2 [Vitis vinifera]

1969	3565	gi|359488798	63.01	7	3	3		50050	PREDICTED: uridine kinase-like protein 5 isoform X1 [Vitis vinifera]
2549	18336	gi|566207035	62.96	4	2	1		62799	hypothetical protein POPTR_0015s11280g [Populus trichocarpa]
2220	1726	gi|225440169	62.96	5	2	2		57401	PREDICTED: cytochrome P450 98A2 [Vitis vinifera]
2099	28970	gi|566200435	62.96	11	3	3		40267	hypothetical protein POPTR_0013s10150g [Populus trichocarpa]
2299	1892	gi|359478198	62.92	6	2	1		45369	PREDICTED: acyl-[acyl-carrier-protein] desaturase chloroplastic [Vitis vinifera]
2377	19993	gi|566196533	62.91	8	2	2	Carbamidomethylation	14778	cytochrome b5 isoform Cb5-C family protein [Populus trichocarpa]
2406	18283	gi|566175427	62.9	2	1	1		83585	acetyl co-enzyme A carboxylase carboxyltransferase alpha subunit family protein [Populus trichocarpa]
1939	29074	gi|225461628	62.83	4	2	1		45420	PREDICTED: UBP1-associated protein 2C [Vitis vinifera]
2271	2760	gi|225437515	62.8	6	1	1	Carbamidomethylation	38873	PREDICTED: dnaJ protein ERDJ3B [Vitis vinifera]
1986	29326	gi|224073746	62.69	11	2	2	Carbamidomethylation	16415	zinc finger family protein [Populus trichocarpa]
2247	1474	gi|225453042	62.68	2	2	2		113229	PREDICTED: cell division cycle 5-like protein [Vitis vinifera]
2269	29025	gi|566161931	62.66	6	2	2	Carbamidomethylation	67485	dehydration-responsive family protein [Populus trichocarpa]
2344	28964	gi|224131934	62.55	7	3	3		57186	hypothetical protein POPTR_0014s16990g [Populus trichocarpa]
1959	1881	gi|731426493	62.34	5	1	1	Carbamidomethylation	34081	PREDICTED: inositol polyphosphate multikinase beta [Vitis vinifera]
2171	19746	gi|224085103	62.31	22	1	1	Carbamidomethylation	7996	ubiquinol-cytochrome C reductase complex 7.8 kDa family protein [Populus trichocarpa]
1552	28971	gi|566173499	62.3	6	4	4		96991	hypothetical protein POPTR_0005s27240g [Populus trichocarpa]

1745	18862	gi|224066707	62.28	10	3	3		36793	fructose-1 family protein [Populus trichocarpa]
2260	18447	gi|359474555	62.27	4	1	1		43514	PREDICTED: probable ADP-ribosylation factor GTPase-activating protein AGD8 [Vitis vinifera]
2307	29092	gi|566200745	62.23	4	2	1		53188	hypothetical protein POPTR_0013s11690g [Populus trichocarpa]
1972	1152	gi|225453336	62.14	3	3	2		90759	PREDICTED: glutamine--tRNA ligase [Vitis vinifera]
2276	29117	gi|566259557	62.07	5	2	2	Carbamidomethylation	54920	hypothetical protein POPTR_0028s00200g partial [Populus trichocarpa]
2124	18739	gi|566186085	62.06	16	2	1		14506	hypothetical protein POPTR_0009s03370g partial [Populus trichocarpa]
1979	18181	gi|566177775	62.04	6	2	1		35282	hypothetical protein POPTR_0006s23520g [Populus trichocarpa]
2428	2087	gi|731440022	62.01	4	2	1		73346	PREDICTED: phosphoenolpyruvate carboxykinase [ATP]-like [Vitis vinifera]
2550	29230	gi|224130030	61.92	17	2	2		17369	peptidyl-prolyl cis-trans isomerase cyclophilin-type family protein [Populus trichocarpa]
2555	2398	gi|225435925	61.81	3	1	1	Carbamidomethylation	50361	PREDICTED: cysteine desulfurase 2 chloroplastic [Vitis vinifera]
2489	6864	gi|359475352	61.75	3	1	1		49145	PREDICTED: uncharacterized protein At4g06744 [Vitis vinifera]
1851	1667	gi|225432306	61.7	22	2	2	Carbamidomethylation	9210	PREDICTED: 40S ribosomal protein S21 [Vitis vinifera]
1967	28962	gi|225444798	61.55	5	3	1		74572	PREDICTED: phospholipid:diacylglycerol acyltransferase 1 [Vitis vinifera]
1705	2906	gi|731398176	61.5	2	3	2	Carbamidomethylation	177869	PREDICTED: intron-binding protein aquarius-like [Vitis vinifera]
1319	2451	gi|225439775	61.49	8	2	1		27055	PREDICTED: glutathione S-transferase L3-like [Vitis vinifera]

2347	28979	gi|225464234	61.45	16	3	3	Carbamidomethylation	24603	PREDICTED: vesicle-associated membrane protein 722 [Vitis vinifera]
2958	7371	gi|225441539	61.44	20	1	1		18749	PREDICTED: 60S ribosomal protein L21-1 [Vitis vinifera]
2766	29066	gi|731389932	61.42	5	2	2		60485	PREDICTED: glutamyl-tRNA(Gln) amidotransferase subunit B chloroplastic/mitochondrial [Vitis vinifera]
1631	2936	gi|731422016	61.38	2	2	2		109785	PREDICTED: exportin-2 [Vitis vinifera]
2937	19098	gi|224109936	61.35	32	2	2	Carbamidomethylation	13215	hypothetical protein POPTR_0010s26160g [Populus trichocarpa]
2415	1738	gi|526117810	61.3	5	1	1		38980	farnesyl diphosphate synthase [Vitis vinifera]
2869	29098	gi|225431247	61.24	18	2	2		12750	PREDICTED: thioredoxin H-type 2 [Vitis vinifera]
2179	1408	gi|731408196	61.21	2	1	1		87158	PREDICTED: subtilisin-like protease isoform X4 [Vitis vinifera]
2308	1899	gi|225444143	61.14	6	2	2		56177	PREDICTED: patellin-4 [Vitis vinifera]
2203	3257	gi|225446080	61.13	3	2	1		60376	PREDICTED: 4-coumarate--CoA ligase 2 [Vitis vinifera]
2695	29048	gi|224139722	61.06	5	2	2		67639	hypothetical protein POPTR_0016s03570g [Populus trichocarpa]
2301	20922	gi|566205979	61.03	4	2	1		46529	hypothetical protein POPTR_0015s05410g [Populus trichocarpa]
1539	18942	gi|566197584	60.93	5	2	2		42529	hypothetical protein POPTR_0012s09200g [Populus trichocarpa]
1997	19198	gi|566191500	60.9	7	3	1	Carbamidomethylation	38352	allyl alcohol dehydrogenase family protein [Populus trichocarpa]
2348	21187	gi|225452785	60.9	11	3	3		29509	PREDICTED: NAP1-related protein 2 [Vitis vinifera]
1579	1991	gi|225431173	60.85	13	1	1		12904	PREDICTED: peptidyl-prolyl cis-trans isomerase Pin1 [Vitis vinifera]

2309	2125	gi|731415654	60.84	3	2	2		73684	PREDICTED: acyl-CoA-binding domain-containing protein 4-like isoform X2 [Vitis vinifera]
1826	2038	gi|526117828	60.84	12	2	1		16599	serine/threonine kinase-like [Vitis vinifera]
1877	2329	gi|225435002	60.79	6	1	1	Carbamidomethylation	27396	PREDICTED: osmotin-like protein [Vitis vinifera]
2086	4639	gi|225442172	60.73	4	2	1		75488	PREDICTED: phospholipid:diacylglycerol acyltransferase 1 [Vitis vinifera]
1657	19104	gi|224078057	60.56	5	1	1		28150	phosphorylase family protein [Populus trichocarpa]
2940	20107	gi|224138170	60.45	29	2	2		7523	hypothetical protein POPTR_0016s06300g [Populus trichocarpa]
2480	2389	gi|731369373	60.45	5	2	1	Carbamidomethylation	51603	PREDICTED: glucan endo-1 3-beta-glucosidase 3 isoform X2 [Vitis vinifera]
2019	20158	gi|566158427	60.44	7	2	2		17387	calmodulin 2 family protein [Populus trichocarpa]
2959	2330	gi|225438277	60.44	3	1	1	Carbamidomethylation	45078	PREDICTED: alcohol dehydrogenase 1 [Vitis vinifera]
2083	17693	gi|224091819	60.41	3	2	1		88349	MIF4G domain-containing family protein [Populus trichocarpa]
2162	19817	gi|566215175	60.4	34	2	2		7135	hypothetical protein POPTR_0018s10540g [Populus trichocarpa]
2543	19629	gi|566157027	60.38	9	2	2		34854	hypothetical protein POPTR_0002s09060g [Populus trichocarpa]
1454	2032	gi|359474652	60.29	7	2	2	Carbamidomethylation	50670	PREDICTED: scopoletin glucosyltransferase-like [Vitis vinifera]
1383	1373	gi|225465210	60.26	17	2	2		11974	PREDICTED: cytochrome c [Vitis vinifera]
2765	29050	gi|566181279	60.25	2	2	1		140709	hypothetical protein POPTR_0007s14320g [Populus trichocarpa]

1736	18086	gi|224071469	60.21	4	1	1		50726	eukaryotic translation initiation factor 2 subunit 3 family protein [Populus trichocarpa]
1565	18535	gi|224064627	60.19	3	1	1		82942	pre-mRNA splicing factor ATP-dependent RNA helicase family protein [Populus trichocarpa]
2065	18227	gi|566196001	60.08	13	2	2	Carbamidomethylation	17516	dimethylmenaquinone methyltransferase family protein [Populus trichocarpa]
2540	29049	gi|224141563	60.05	7	2	1		23553	peroxiredoxin Q family protein [Populus trichocarpa]
2246	5138	gi|731404421	60	9	3	2	Carbamidomethylation	51194	PREDICTED: monothiol glutaredoxin-S17 isoform X2 [Vitis vinifera]
1873	2434	gi|225446899	59.94	3	2	1		70904	PREDICTED: DEAD-box ATP-dependent RNA helicase 53-like [Vitis vinifera]
2222	1703	gi|731376062	59.77	3	2	1		83343	PREDICTED: urease isoform X2 [Vitis vinifera]
2137	29372	gi|566170569	59.73	9	2	1		29811	hypothetical protein POPTR_0005s10420g [Populus trichocarpa]
2893	29710	gi|359495737	59.73	5	1	1		32269	PREDICTED: ATP-dependent Clp protease proteolytic subunit-related protein 4 chloroplastic-like [Vitis vinifera]
2311	2271	gi|731410110	59.7	2	2	1	Carbamidomethylation	108803	PREDICTED: vacuolar protein-sorting-associated protein 11 homolog isoform X1 [Vitis vinifera]
2484	19418	gi|731390081	59.69	6	2	2	Carbamidomethylation	50947	PREDICTED: probable pectate lyase 8 [Vitis vinifera]
2129	18859	gi|224107333	59.69	26	2	2		9716	hypothetical protein POPTR_0010s02580g [Populus trichocarpa]
1694	928	gi|225445670	59.66	9	3	1		24997	PREDICTED: proteasome subunit beta type-6 [Vitis vinifera]
1799	19396	gi|731382729	59.66	9	2	1	Carbamidomethylation	39538	PREDICTED: isocitrate dehydrogenase [NAD] regulatory subunit 1 mitochondrial isoform X2 [Vitis vinifera]

769	1200	gi|225452053	59.63	10	3	3		21963	PREDICTED: 40S ribosomal protein S7 [Vitis vinifera]
1827	18555	gi|566167707	59.63	8	2	2		29259	alcohol dehydroge family protein [Populus trichocarpa]
2960	29805	gi|566161524	59.61	16	1	1		12520	hypothetical protein POPTR_0003s081902g partial [Populus trichocarpa]
1654	18775	gi|566181200	59.58	4	2	1	Carbamidomethylation	55243	serine carboxypeptidase S28 family protein [Populus trichocarpa]
1656	29353	gi|225431940	59.5	16	2	2	Carbamidomethylation	18056	PREDICTED: universal stress protein A-like protein [Vitis vinifera]
1924	2559	gi|225433676	59.47	7	2	1		37475	PREDICTED: probable aldo-keto reductase 2 [Vitis vinifera]
2133	1668	gi|225448536	59.45	7	2	2		38803	PREDICTED: eukaryotic translation initiation factor 2 subunit alpha [Vitis vinifera]
2068	29026	gi|731395931	59.33	2	2	2		114613	PREDICTED: protein translocase subunit SecA chloroplastic [Vitis vinifera]
1265	1489	gi|731400676	59.12	12	2	2		18669	PREDICTED: 60S ribosomal protein L24 [Vitis vinifera]
2820	2245	gi|731381456	59.08	7	2	2		34629	PREDICTED: translocase of chloroplast 34 chloroplastic isoform X2 [Vitis vinifera]
1247	18903	gi|566146533	59.08	27	3	3		10147	acyl-CoA-binding family protein [Populus trichocarpa]
2189	20645	gi|224146640	59.04	2	2	2		111716	chloroplast inner envelope family protein [Populus trichocarpa]
1107	2502	gi|225424908	58.9	8	2	1		29426	PREDICTED: mitochondrial outer membrane protein porin of 36 kDa [Vitis vinifera]
1556	2442	gi|225429295	58.68	8	2	2		25371	PREDICTED: uncharacterized protein LOC100254416 [Vitis vinifera]
2458	1931	gi|225453329	58.65	18	2	2		12870	PREDICTED: tubulin-folding cofactor A [Vitis vinifera]
2005	18545	gi|566170295	58.54	3	2	1	Oxidation (M)	119716	Valyl-tRNA synthetase family protein [Populus trichocarpa]

2172	19514	gi|566254378	58.31	15	1	1	Carbamidomethylation	9714	hypothetical protein POPTR_0997s00210g partial [Populus trichocarpa]
2715	29152	gi|566212923	58.28	6	2	2	Carbamidomethylation	56707	hypothetical protein POPTR_0017s12380g [Populus trichocarpa]
1990	19431	gi|566151403	58.27	20	1	1		8809	hypothetical protein POPTR_0001s28090g [Populus trichocarpa]
2274	18997	gi|566236086	58.26	4	2	1	Carbamidomethylation	48154	hypothetical protein POPTR_0019s08450g [Populus trichocarpa]
1386	20214	gi|566156678	58.11	6	2	2		23173	hypothetical protein POPTR_0002s07090g [Populus trichocarpa]
1708	2798	gi|225455286	58.04	8	2	2		34977	PREDICTED: vacuolar protein sorting-associated protein 26A [Vitis vinifera]
1828	18641	gi|566169633	58.03	8	2	2		30715	hypothetical protein POPTR_0005s05400g [Populus trichocarpa]
1741	29811	gi|224144252	58.01	14	1	1		14788	hypothetical protein POPTR_0018s13330g [Populus trichocarpa]
2590	29257	gi|225433191	57.99	8	2	2		31525	PREDICTED: probable plastid-lipid-associated protein 6 chloroplastic [Vitis vinifera]
1614	1104	gi|731435142	57.97	3	2	1		84036	PREDICTED: protein ROOT HAIR DEFECTIVE 3 homolog 2 isoform X2 [Vitis vinifera]
2363	2312	gi|225457389	57.81	5	1	1		27867	PREDICTED: chlorophyll a-b binding protein 4 chloroplastic [Vitis vinifera]
1558	19793	gi|566181894	57.71	3	2	1		58001	hypothetical protein POPTR_0008s02250g [Populus trichocarpa]
2680	29068	gi|731430671	57.63	2	2	2	Carbamidomethylation	122832	PREDICTED: exocyst complex component SEC5A [Vitis vinifera]
2255	3802	gi|225463809	57.62	6	2	1	Carbamidomethylation	44723	PREDICTED: alpha-galactosidase [Vitis vinifera]

2831	19296	gi|566205313	57.57	6	2	2		28442	glucosamine/galactosamine-6-phosphate isomerase family protein [Populus trichocarpa]
1876	2344	gi|225443154	57.56	9	1	1	Carbamidomethylation; Oxidation (M)	18245	PREDICTED: lamin-like protein [Vitis vinifera]
2221	19275	gi|566185320	57.55	3	2	2		87131	hypothetical protein POPTR_0008s22260g [Populus trichocarpa]
2941	29258	gi|731430003	57.52	18	2	2		21619	PREDICTED: rac-like GTP-binding protein 5 [Vitis vinifera]
1302	19311	gi|566172679	57.52	2	1	1		63027	hypothetical protein POPTR_0005s22250g [Populus trichocarpa]
2512	29093	gi|566177795	57.47	7	2	2	Carbamidomethylation	39471	eukaryotic translation initiation factor 2B family protein [Populus trichocarpa]
2116	28975	gi|225445059	57.42	8	3	3		53815	PREDICTED: glucan endo-1 3-beta-glucosidase 13 [Vitis vinifera]
2151	1379	gi|731402065	57.4	4	3	1		60930	PREDICTED: chaperonin CPN60-like 2 mitochondrial [Vitis vinifera]
1811	18744	gi|224111090	57.38	5	2	1	Oxidation (M)	64765	pyruvate phosphotransferase family protein [Populus trichocarpa]
2132	18351	gi|566216001	57.36	5	2	2	Carbamidomethylation	74542	hypothetical protein POPTR_0019s00300g [Populus trichocarpa]
1868	950	gi|225440362	57.21	20	2	1	Oxidation (M)	10797	PREDICTED: mitochondrial import inner membrane translocase subunit Tim9 [Vitis vinifera]
1825	18938	gi|566170130	57.2	4	2	1		51590	hypothetical protein POPTR_0005s08100g [Populus trichocarpa]
1634	20008	gi|224118114	57.19	3	3	2		96198	hypothetical protein POPTR_0012s01060g [Populus trichocarpa]
1869	2120	gi|731389062	57.13	8	2	2		25568	PREDICTED: endo-1 3;1 4-beta-D-glucanase-like [Vitis vinifera]
2121	18453	gi|224122636	57.12	4	2	2		46434	putative calmodulin-binding family protein [Populus trichocarpa]

2158	2668	gi|731391510	57.05	11	2	2		25576	PREDICTED: uncharacterized protein LOC104879510 isoform X2 [Vitis vinifera]
2232	29061	gi|225438479	57.02	4	2	2		91235	PREDICTED: ribonucleoside-diphosphate reductase large subunit [Vitis vinifera]
1636	18525	gi|566206477	56.97	11	2	1		24609	hypothetical protein POPTR_0015s07620g partial [Populus trichocarpa]
1950	29037	gi|224087605	56.95	5	2	2	Carbamidomethylation	55105	kinase family protein [Populus trichocarpa]
2870	18074	gi|566161702	56.95	6	2	2		30568	Mitochondrial carnitine/acylcarnitine carrier-like family protein [Populus trichocarpa]
1737	19762	gi|566161718	56.95	3	1	1		43721	UBIQUITIN-SPECIFIC PROTEASE 20 family protein [Populus trichocarpa]
2514	19334	gi|224067846	56.82	9	2	2		28816	hypothetical protein POPTR_0002s15600g [Populus trichocarpa]
1985	19730	gi|224093270	56.78	14	1	1	Carbamidomethylation	10603	hypothetical protein POPTR_0007s03140g [Populus trichocarpa]
2354	18901	gi|566198700	56.71	4	2	2		42593	hypothetical protein POPTR_0013s00760g [Populus trichocarpa]
2558	29377	gi|225442853	56.64	4	1	1	Carbamidomethylation	47945	PREDICTED: uncharacterized protein LOC100253140 [Vitis vinifera]
2476	28980	gi|566209963	56.6	5	3	3		90934	AMP deaminase family protein [Populus trichocarpa]
2118	28983	gi|224108087	56.49	4	3	1	Carbamidomethylation	59278	RAN GTPASE ACTIVATING protein 2 [Populus trichocarpa]
2588	2854	gi|731408416	56.4	4	2	1		53964	PREDICTED: serine carboxypeptidase-like 13 [Vitis vinifera]
2259	1346	gi|225447779	56.18	4	2	2		49572	PREDICTED: pinin [Vitis vinifera]
2502	18469	gi|566187699	56.18	6	2	2		36049	ubiquitin fusion degradation UFD1 family protein [Populus trichocarpa]

2411	29356	gi|225465060	56.14	3	1	1		50015	PREDICTED: acetylornithine aminotransferase mitochondrial [Vitis vinifera]
2283	1793	gi|225438019	56.07	4	1	1		44983	PREDICTED: heterogeneous nuclear ribonucleoprotein 1-like [Vitis vinifera]
1863	28990	gi|731393582	56.04	4	3	3		58065	PREDICTED: dihydropyrimidinase [Vitis vinifera]
2277	3624	gi|359491024	56.03	7	2	2	Carbamidomethylation	25764	PREDICTED: thaumatin-like protein [Vitis vinifera]
1325	18810	gi|566209344	55.96	7	2	1		27361	In2-1 family protein [Populus trichocarpa]
2938	1596	gi|731405420	55.89	4	2	1		60227	PREDICTED: reticuline oxidase-like protein [Vitis vinifera]
2725	2336	gi|225441619	55.88	7	1	1		19639	PREDICTED: putative H/ACA ribonucleoprotein complex subunit 1-like protein 1 [Vitis vinifera]
2358	2411	gi|225448433	55.85	6	2	1	Carbamidomethylation	67768	PREDICTED: phosphoinositide phospholipase C 2 [Vitis vinifera]
2254	18271	gi|224105935	55.85	11	2	2	Carbamidomethylation	24127	RAN BINDING protein 1 [Populus trichocarpa]
2160	29248	gi|566178170	55.83	5	2	1		51300	hypothetical protein POPTR_0006s25850g [Populus trichocarpa]
2492	19736	gi|224086707	55.81	2	1	1	Carbamidomethylation	73852	glycosyl hydrolase family protein [Populus trichocarpa]
1732	1758	gi|359495836	55.66	3	2	2		64385	PREDICTED: galacturonosyltransferase 8 [Vitis vinifera]
2562	29618	gi|225432296	55.65	10	1	1		16839	PREDICTED: uncharacterized protein LOC100249327 [Vitis vinifera]
2365	18984	gi|566208306	55.57	5	1	1	Carbamidomethylation	28285	hypothetical protein POPTR_0016s02570g [Populus trichocarpa]
2412	29388	gi|224101457	55.55	10	1	1		19688	RNA recognition motif-containing family protein [Populus trichocarpa]
1859	1026	gi|225443231	55.51	6	3	2		50032	PREDICTED: methanol O-anthraniloyltransferase [Vitis vinifera]

2565	19710	gi|225444714	55.48	12	1	1	Carbamidomethylation	13434	PREDICTED: RING-box protein 1a [Vitis vinifera]
2003	1794	gi|225452562	55.43	7	2	2		35739	PREDICTED: peroxidase 31-like [Vitis vinifera]
2557	29352	gi|225465543	55.43	8	2	1		22596	PREDICTED: uncharacterized protein LOC100263614 [Vitis vinifera]
2233	18943	gi|224140195	55.41	12	2	2		30031	29 kDa ribonucleoprotein [Populus trichocarpa]
2886	29669	gi|359493330	55.39	5	1	1	Carbamidomethylation	33897	PREDICTED: carboxymethylenebutenolidase homolog [Vitis vinifera]
1717	1592	gi|225459957	55.32	12	2	2		21003	PREDICTED: uncharacterized protein LOC100261996 [Vitis vinifera]
2942	29268	gi|731378520	55.24	9	2	2	Carbamidomethylation	23444	PREDICTED: RNA polymerase II transcriptional coactivator KELP [Vitis vinifera]
2548	2871	gi|225455386	55.2	2	2	1		65593	PREDICTED: probable pectinesterase/pectinesterase inhibitor 34 [Vitis vinifera]
1787	2039	gi|225449198	55.13	6	2	1		31414	PREDICTED: eukaryotic translation initiation factor 3 subunit F [Vitis vinifera]
2332	19031	gi|224077834	55.1	3	1	1	Carbamidomethylation	56616	phenylalanyl-tRNA synthetase alpha chain family protein [Populus trichocarpa]
3174	19090	gi|566204168	55.07	3	1	1		56223	Citrate synthase family protein [Populus trichocarpa]
2338	29960	gi|566149116	55.04	5	2	2		39813	nuclear RNA-binding family protein [Populus trichocarpa]
2306	18004	gi|224086130	54.74	8	2	1	Carbamidomethylation	32243	U2 snRNP auxiliary factor small subunit family protein [Populus trichocarpa]
2236	29135	gi|224105121	54.74	2	2	2		98894	hypothetical protein POPTR_0009s14030g [Populus trichocarpa]
2061	23574	gi|731397903	54.71	2	2	1		174618	PREDICTED: DNA (cytosine-5)-methyltransferase 1-like [Vitis vinifera]

2364	29812	gi|224136252	54.7	19	1	1	Carbamidomethylation	8798	hypothetical protein POPTR_0015s11410g [Populus trichocarpa]
3688	29813	gi|731383113	54.69	8	1	1	Carbamidomethylation	27145	PREDICTED: zinc finger protein GIS2-like [Vitis vinifera]
2165	1981	gi|731400731	54.64	7	3	3		35100	PREDICTED: histone deacetylase HDT1 [Vitis vinifera]
1813	1078	gi|359495228	54.61	2	1	1		81899	PREDICTED: far upstream element-binding protein 1 isoform X2 [Vitis vinifera]
2771	29342	gi|731438722	54.6	2	1	1		104093	PREDICTED: uncharacterized protein LOC100263199 partial [Vitis vinifera]
2724	29431	gi|359475310	54.58	4	1	1		52362	PREDICTED: glucan endo-1 3-beta-D-glucosidase [Vitis vinifera]
3330	29469	gi|224075888	54.52	4	1	1	Carbamidomethylation	33209	transcription factor family protein [Populus trichocarpa]
1853	29932	gi|225449374	54.49	7	2	2		28605	PREDICTED: uncharacterized protein At2g39795 mitochondrial [Vitis vinifera]
1914	19728	gi|224070184	54.49	11	1	1		18056	deoxyuridine 5'-triphosphate nucleotidohydrolase family protein [Populus trichocarpa]
2598	3507	gi|731400520	54.44	3	2	1		70745	PREDICTED: pentatricopeptide repeat-containing protein At1g80270 mitochondrial-like [Vitis vinifera]
2768	2426	gi|225437596	54.41	2	2	1		94375	PREDICTED: receptor-like protein kinase ANXUR1 [Vitis vinifera]
2366	2473	gi|225465847	54.4	8	1	1		21605	PREDICTED: NADPH:quinone oxidoreductase [Vitis vinifera]
2446	20005	gi|224111560	54.39	3	2	1	Carbamidomethylation	96995	hydroxyproline-rich glycoprotein [Populus trichocarpa]
2947	29304	gi|566197864	54.37	11	2	2		33444	hypothetical protein POPTR_0012s10940g [Populus trichocarpa]
1889	18109	gi|566168240	54.35	3	2	1	Carbamidomethylation	55680	hypothetical protein POPTR_0004s23390g [Populus trichocarpa]

1984	1795	gi|225465530	54.34	5	2	1	Carbamidomethylation	37226	PREDICTED: cinnamoyl-CoA reductase 1 [Vitis vinifera]
2100	18348	gi|566173129	54.28	3	1	1		41052	hypothetical protein POPTR_0005s25100g [Populus trichocarpa]
2302	1731	gi|359489456	54.22	4	1	1		26638	PREDICTED: vesicle-associated protein 1-2 [Vitis vinifera]
2683	4884	gi|359476827	54.2	0	1	1		440796	PREDICTED: transcription-associated protein 1 [Vitis vinifera]
2552	3013	gi|731391200	54.18	15	2	2	Carbamidomethylation	18111	PREDICTED: peptidyl-prolyl cis-trans isomerase FKBP16-3 chloroplastic isoform X2 [Vitis vinifera]
2321	29360	gi|225448412	54.14	7	2	1	Carbamidomethylation	28575	PREDICTED: GDSL esterase/lipase CPRD49 [Vitis vinifera]
2047	29009	gi|731402039	54.04	8	2	2		31328	PREDICTED: protein FLX-like 3 isoform X2 [Vitis vinifera]
1935	18906	gi|566208409	54.04	3	2	1		57917	hypothetical protein POPTR_0016s03090g [Populus trichocarpa]
2718	29295	gi|731399352	53.93	5	1	1	Carbamidomethylation	35023	PREDICTED: translin isoform X2 [Vitis vinifera]
2880	29580	gi|359486553	53.92	5	1	1		32528	PREDICTED: zinc finger CCCH domain-containing protein 11 [Vitis vinifera]
2778	2127	gi|731439308	53.92	4	1	1		45537	PREDICTED: fumarylacetoacetase isoform X1 [Vitis vinifera]
2825	19580	gi|566155691	53.86	1	1	1		99018	ubiquitin family protein [Populus trichocarpa]
2961	4942	gi|731401567	53.84	4	1	1		45798	PREDICTED: 4-coumarate--CoA ligase-like 7 [Vitis vinifera]
2593	18537	gi|566159086	53.81	3	2	1		61936	hypothetical protein POPTR_0002s20370g [Populus trichocarpa]
2962	29819	gi|225440520	53.7	5	1	1		31574	PREDICTED: probable proteasome inhibitor [Vitis vinifera]
2944	29289	gi|566153022	53.64	8	2	1		40170	hypothetical protein POPTR_0001s35950g partial [Populus trichocarpa]

2335	2376	gi|731384061	53.54	3	1	1		47636	PREDICTED: LL-diaminopimelate aminotransferase chloroplastic isoform X6 [Vitis vinifera]
2104	2207	gi|225435594	53.47	1	1	1		72574	PREDICTED: selenoprotein O [Vitis vinifera]
2516	29321	gi|224081334	53.41	4	1	1		48708	hypothetical protein POPTR_0005s09200g [Populus trichocarpa]
1739	2470	gi|225442639	53.35	9	1	1		15488	PREDICTED: uncharacterized protein LOC100260053 [Vitis vinifera]
1860	1779	gi|225448795	53.29	11	3	3	Carbamidomethylation	19172	PREDICTED: cytochrome c oxidase subunit 6b-1 isoform X4 [Vitis vinifera]
2253	19154	gi|566260103	53.25	9	2	1		27424	hypothetical protein POPTR_0021s00560g [Populus trichocarpa]
2769	29302	gi|224064484	53.21	3	1	1		66420	arginyl-tRNA synthetase family protein [Populus trichocarpa]
1980	18783	gi|224101527	53.18	5	2	1		35400	peroxidase 40 family protein [Populus trichocarpa]
2141	18112	gi|224065405	53.09	14	1	1		8817	wound-responsive family protein [Populus trichocarpa]
2367	18343	gi|566161304	53.02	4	1	1		52669	hypothetical protein POPTR_0003s07020g [Populus trichocarpa]
2943	5131	gi|526117689	53.02	5	2	2		52463	hexokinase [Vitis vinifera]
2974	19328	gi|225444641	53.01	3	2	2	Oxidation (M)	59678	PREDICTED: 3-oxoacyl-[acyl-carrier-protein] synthase II chloroplastic [Vitis vinifera]
3689	3539	gi|731378986	52.99	13	1	1		13972	PREDICTED: LOW QUALITY PROTEIN: mitochondrial import inner membrane translocase subunit Tim13 [Vitis vinifera]
2818	20759	gi|225463758	52.99	6	2	2		43390	PREDICTED: reticulocalbin-2 [Vitis vinifera]
2554	1851	gi|225439145	52.96	3	1	1		37191	PREDICTED: grpE protein homolog mitochondrial isoform X1 [Vitis vinifera]

2744	29286	gi|566160921	52.94	1	1	1		109190	hypothetical protein POPTR_0003s05060g [Populus trichocarpa]
3536	29636	gi|731397329	52.86	2	1	1		72522	PREDICTED: uncharacterized protein LOC100242349 isoform X2 [Vitis vinifera]
2953	29478	gi|224117526	52.82	10	1	1	Carbamidomethylation	23539	OTU-like cysteine protease family protein [Populus trichocarpa]
2315	2180	gi|225428049	52.81	3	1	1		42284	PREDICTED: glycerate dehydrogenase HPR peroxisomal [Vitis vinifera]
2145	19744	gi|224137260	52.73	12	1	1		11459	hypothetical protein POPTR_0016s01100g [Populus trichocarpa]
2117	19229	gi|566165862	52.72	7	3	3		41915	hypothetical protein POPTR_0004s10130g [Populus trichocarpa]
2207	19950	gi|224060461	52.6	6	2	1		39296	mannitol dehydrogenase 1 family protein [Populus trichocarpa]
2015	2958	gi|731419100	52.56	6	1	1	Carbamidomethylation	19892	PREDICTED: universal stress protein A-like protein [Vitis vinifera]
2128	29125	gi|225457807	52.55	10	2	2		20135	PREDICTED: thioredoxin [Vitis vinifera]
2357	29345	gi|566204667	52.54	3	2	2	Carbamidomethylation	93158	hypothetical protein POPTR_0014s16780g [Populus trichocarpa]
2949	29348	gi|731402497	52.54	5	2	2		46720	PREDICTED: nuclear pore complex protein NUP50A [Vitis vinifera]
2607	19253	gi|225457361	52.52	6	1	1		29994	PREDICTED: ribulose-phosphate 3-epimerase chloroplastic [Vitis vinifera]
2836	30023	gi|359487013	52.51	3	2	2		55071	PREDICTED: uncharacterized protein LOC100259997 [Vitis vinifera]
2006	1619	gi|225461983	52.43	5	2	1		36179	PREDICTED: heterogeneous nuclear ribonucleoprotein 1 [Vitis vinifera]

2939	29250	gi|731400636	52.43	7	2	2	Carbamidomethylation	44292	PREDICTED: DEAD-box ATP-dependent RNA helicase 38 isoform X2 [Vitis vinifera]
2466	2215	gi|731411822	52.41	5	1	1	Carbamidomethylation	29958	PREDICTED: serine/arginine-rich splicing factor RS2Z33-like isoform X2 [Vitis vinifera]
2313	2102	gi|225434197	52.4	6	2	1	Carbamidomethylation	38070	PREDICTED: 2-alkenal reductase (NADP(+)-dependent) [Vitis vinifera]
2826	29467	gi|224142233	52.36	6	2	2		36105	Peroxidase 16 precursor family protein [Populus trichocarpa]
1748	1743	gi|225441809	52.28	29	3	3		8917	PREDICTED: uncharacterized protein LOC100245204 isoform X3 [Vitis vinifera]
2822	4692	gi|731403895	52.16	2	1	1		86125	PREDICTED: cycloartenol synthase-like [Vitis vinifera]
3432	29501	gi|566168456	52.06	2	1	1		107171	hypothetical protein POPTR_0004s24340g [Populus trichocarpa]
2948	29317	gi|225426084	52.05	13	2	2		20541	PREDICTED: disease resistance response protein 206 [Vitis vinifera]
2353	1321	gi|225424750	52.01	17	2	2	Carbamidomethylation	11841	PREDICTED: gibberellin-regulated protein 11 [Vitis vinifera]
2281	29312	gi|566171425	52	2	1	1		50173	hypothetical protein POPTR_0005s14880g [Populus trichocarpa]
2449	29169	gi|225434156	51.95	1	1	1	Carbamidomethylation	102086	PREDICTED: formin-like protein 1 [Vitis vinifera]
1637	29149	gi|566192982	51.85	3	2	1		59112	calcium-binding EF hand family protein [Populus trichocarpa]
1874	1617	gi|225429460	51.85	9	1	1		14040	PREDICTED: uncharacterized protein LOC100267911 [Vitis vinifera]
2876	29316	gi|566211927	51.8	5	2	2		43994	hypothetical protein POPTR_0017s07040g [Populus trichocarpa]
2956	29522	gi|359488959	51.73	1	1	1		117470	PREDICTED: probable ubiquitin conjugation factor E4 [Vitis vinifera]

2421	2394	gi|225423637	51.68	3	1	1	Carbamidomethylation	53687	PREDICTED: UDP-N-acetylglucosamine diphosphorylase 1 [Vitis vinifera]
2014	19737	gi|224070843	51.51	8	1	1		19159	glutaredoxin family protein [Populus trichocarpa]
2515	29307	gi|566167103	51.43	1	1	1		145388	hypothetical protein POPTR_0004s17740g [Populus trichocarpa]
2369	2315	gi|731376404	51.35	7	1	1	Carbamidomethylation	28963	PREDICTED: chlorophyll a-b binding protein chloroplastic [Vitis vinifera]
2950	19375	gi|566186176	51.32	3	2	2		108942	hypothetical protein POPTR_0009s03980g [Populus trichocarpa]
3324	20043	gi|731393417	51.29	3	1	1		43134	PREDICTED: probable linoleate 9S-lipoxygenase 5 [Vitis vinifera]
2521	19708	gi|224073126	51.25	4	1	1		38413	hypothetical protein POPTR_0003s20890g [Populus trichocarpa]
2767	29109	gi|224104085	51.25	5	2	1		42423	ULTRACURVATA 2 family protein [Populus trichocarpa]
2328	2026	gi|359492937	51.23	2	1	1		46528	PREDICTED: aspartic proteinase nepenthesin-1 [Vitis vinifera]
2596	20092	gi|731380117	51.22	6	1	1		27501	PREDICTED: thylakoid lumenal protein At1g12250 chloroplastic isoform X3 [Vitis vinifera]
3436	29578	gi|566206225	51.22	5	1	1	Carbamidomethylation	38607	hypothetical protein POPTR_0015s06550g [Populus trichocarpa]
2007	18582	gi|566156002	51.21	4	2	1		37272	hypothetical protein POPTR_0002s03260g [Populus trichocarpa]
2166	5205	gi|359482448	51.17	2	2	1	Carbamidomethylation	83440	PREDICTED: subtilisin-like protease [Vitis vinifera]
2464	19140	gi|225437598	51.17	1	1	1		130089	PREDICTED: nodal modulator 1 [Vitis vinifera]
2571	2549	gi|359490081	51.15	3	1	1	Carbamidomethylation	37910	PREDICTED: probable isoaspartyl peptidase/L-asparaginase 3 isoform X1 [Vitis vinifera]

1982	18866	gi|224108744	51.15	7	2	1		29000	enoyl-CoA hydratase/isomerase family protein [Populus trichocarpa]
2591	7334	gi|225437581	51.13	7	2	1		31520	PREDICTED: ATP-dependent Clp protease proteolytic subunit 5 chloroplastic [Vitis vinifera]
2717	29247	gi|224062053	51.11	2	1	1		89364	hypothetical protein POPTR_0002s02940g [Populus trichocarpa]
3141	29420	gi|566188679	51.02	3	1	1	Carbamidomethylation	60358	hypothetical protein POPTR_0010s01850g [Populus trichocarpa]
2355	29283	gi|225440492	51	8	2	2		30483	PREDICTED: uracil phosphoribosyltransferase [Vitis vinifera]
2257	18270	gi|224122936	50.96	2	2	2		107004	Lon protease 2 family protein [Populus trichocarpa]
2649	29299	gi|224069969	50.94	1	1	1		157594	CLIP-associating family protein [Populus trichocarpa]
2814	29043	gi|225464033	50.9	7	2	2		35049	PREDICTED: ABC transporter I family member 6 chloroplastic [Vitis vinifera]
2878	2693	gi|225430537	50.86	9	1	1		13625	PREDICTED: DNA-directed RNA polymerases II IV and V subunit 11 [Vitis vinifera]
2604	2417	gi|731400181	50.77	1	1	1		80319	PREDICTED: uncharacterized protein C18orf8 isoform X2 [Vitis vinifera]
2139	19167	gi|224143157	50.7	1	1	1		73679	clathrin assembly family protein [Populus trichocarpa]
1971	3012	gi|225445470	50.68	35	3	1		13986	PREDICTED: NHP2-like protein 1 [Vitis vinifera]
2589	29244	gi|224117704	50.62	4	2	1		70910	dehydration-responsive family protein [Populus trichocarpa]
1987	29335	gi|225466271	50.6	10	2	1		21853	PREDICTED: ras-related protein Rab5 [Vitis vinifera]
1752	2723	gi|359485189	50.56	3	2	1	Carbamidomethylation	59681	PREDICTED: aspartic proteinase nepenthesin-2 [Vitis vinifera]
1513	1828	gi|225434253	50.49	5	2	1		34679	PREDICTED: uncharacterized protein LOC100266227 isoform X2 [Vitis vinifera]

2527	20110	gi|566207660	50.48	7	1	1		21645	cytochrome b5 domain-containing family protein [Populus trichocarpa]
2823	2395	gi|225435776	50.43	3	1	1		66471	PREDICTED: probable lysine--tRNA ligase cytoplasmic [Vitis vinifera]
1434	1422	gi|731439999	50.38	2	2	1	Carbamidomethylation	80675	PREDICTED: primary amine oxidase-like [Vitis vinifera]
2600	18828	gi|566208534	50.32	2	1	1		50151	chloroplast cystathionine beta lyase family protein [Populus trichocarpa]
2161	2627	gi|731393556	50.31	7	2	2		29837	PREDICTED: rhodanese-like/PpiC domain-containing protein 12 isoform X2 [Vitis vinifera]
2049	3312	gi|225439821	50.27	1	2	1	Carbamidomethylation	116087	PREDICTED: calcium-transporting ATPase 4 endoplasmic reticulum-type-like [Vitis vinifera]
2238	19332	gi|731424956	50.18	2	1	1		58108	PREDICTED: cytochrome P450 71A3-like [Vitis vinifera]
1435	1440	gi|731435737	50.1	1	2	1		160776	PREDICTED: putative disease resistance RPP13-like protein 1 isoform X1 [Vitis vinifera]
2546	29133	gi|225442194	50.04	6	2	2		45670	PREDICTED: stomatin-like protein 2 mitochondrial [Vitis vinifera]
1182	19117	gi|566173131	50.01	3	1	1		39060	dihydroflavonol reductase family protein [Populus trichocarpa]
2488	29384	gi|359481218	49.93	2	1	1	Carbamidomethylation	69404	PREDICTED: vacuolar-sorting receptor 1 [Vitis vinifera]
3690	29828	gi|224118638	49.92	5	1	1		36589	hypothetical protein POPTR_0012s04410g [Populus trichocarpa]
1749	28998	gi|566189517	49.91	6	3	2		53512	hypothetical protein POPTR_0010s07010g [Populus trichocarpa]
2465	1735	gi|225451318	49.87	4	1	1		37997	PREDICTED: probable aldo-keto reductase 1 [Vitis vinifera]
2089	1096	gi|225445041	49.8	4	2	1		54472	PREDICTED: mitochondrial-processing peptidase subunit alpha [Vitis vinifera]
2235	29130	gi|731385930	49.66	2	3	3		98079	PREDICTED: ABC transporter F family member 4 [Vitis vinifera]

2720	3537	gi|225436003	49.66	4	1	1	Carbamidomethylation	31003	PREDICTED: NADH--cytochrome b5 reductase 1 [Vitis vinifera]
2834	20027	gi|225456703	49.62	11	1	1		17349	PREDICTED: ubiquitin-conjugating enzyme E2 2 [Vitis vinifera]
3269	2365	gi|225441864	49.53	3	1	1	Carbamidomethylation	57917	PREDICTED: thioredoxin reductase NTRC [Vitis vinifera]
2504	28999	gi|566201913	49.44	5	3	2	Carbamidomethylation	82038	hypothetical protein POPTR_0014s01910g [Populus trichocarpa]
2378	19946	gi|566206307	49.41	3	1	1		42484	nuclear transport factor 2 family protein [Populus trichocarpa]
1960	1908	gi|225424859	49.39	4	1	1		25630	PREDICTED: glutathione S-transferase U17 [Vitis vinifera]
3692	29842	gi|224099925	49.37	15	1	1		10144	hypothetical protein POPTR_0008s16780g [Populus trichocarpa]
2881	29597	gi|566259532	49.35	9	1	1	Carbamidomethylation	20415	cytidine/deoxycytidylate deaminase family protein [Populus trichocarpa]
2951	2469	gi|731394805	49.34	5	1	1		30948	PREDICTED: ribosome-recycling factor chloroplastic [Vitis vinifera]
2436	18860	gi|566192855	49.32	3	1	1		34711	hypothetical protein POPTR_0010s26140g [Populus trichocarpa]
2595	2891	gi|731417628	49.28	9	2	2		37526	PREDICTED: methyltransferase-like protein 10 [Vitis vinifera]
2608	19825	gi|224096296	49.27	3	1	1		40670	hypothetical protein POPTR_0007s06500g [Populus trichocarpa]
2140	19792	gi|566210696	49.22	3	1	1		42582	hypothetical protein POPTR_0017s01530g [Populus trichocarpa]
2964	29854	gi|224130644	49.09	6	1	1		21941	GRF1-INTERACTING FACTOR 2 family protein [Populus trichocarpa]
3441	29655	gi|566151879	49.04	2	1	1		90753	hypothetical protein POPTR_0001s30620g [Populus trichocarpa]
2603	29529	gi|566260143	48.93	2	1	1		59928	hypothetical protein POPTR_0021s00750g [Populus trichocarpa]

2723	29421	gi|225427100	48.9	3	1	1		50818	PREDICTED: glycerol-3-phosphate acyltransferase chloroplastic [Vitis vinifera]
2682	29347	gi|566150318	48.85	3	1	1		44042	strictosidine synthase family protein [Populus trichocarpa]
2963	29831	gi|225451011	48.84	10	1	1		19134	PREDICTED: signal peptidase complex subunit 3B [Vitis vinifera]
3425	29448	gi|731392112	48.78	4	1	1		52085	PREDICTED: protein TIC 62 chloroplastic isoform X3 [Vitis vinifera]
1468	19865	gi|566174225	48.74	5	1	1		22376	hypothetical protein POPTR_0006s03170g [Populus trichocarpa]
2545	29132	gi|566175887	48.74	12	2	2		36595	hypothetical protein POPTR_0006s12310g [Populus trichocarpa]
1962	2412	gi|225453100	48.7	4	1	1		34790	PREDICTED: ATP-dependent Clp protease proteolytic subunit 3 chloroplastic [Vitis vinifera]
2592	2808	gi|731384926	48.6	11	2	2		15933	PREDICTED: 60S ribosomal protein L28-1 isoform X3 [Vitis vinifera]
2334	20190	gi|566181645	48.57	3	1	1		39280	hypothetical protein POPTR_0008s01030g [Populus trichocarpa]
2016	2467	gi|225450774	48.52	4	1	1		35393	PREDICTED: ATP-dependent Clp protease proteolytic subunit-related protein 3 chloroplastic [Vitis vinifera]
2318	2734	gi|359476844	48.48	4	2	1	Carbamidomethylation	53239	PREDICTED: probable serine protease EDA2 isoform X2 [Vitis vinifera]
2256	18018	gi|224083504	48.42	2	2	2		105368	hypothetical protein POPTR_0005s07000g [Populus trichocarpa]
3693	19018	gi|224055587	48.39	4	1	1		40024	GHMP kinase-related family protein [Populus trichocarpa]
2887	19171	gi|225445124	48.33	3	1	1		67861	PREDICTED: SNW/SKI-interacting protein [Vitis vinifera]

2902	2348	gi|225463408	48.24	10	1	1		14802	PREDICTED: DNA-binding protein DDB_G0278111 [Vitis vinifera]
2830	19831	gi|224093434	48.23	3	1	1		37278	hypothetical protein POPTR_0007s04240g [Populus trichocarpa]
2467	2147	gi|225451754	48.23	3	1	1	Carbamidomethylation	46712	PREDICTED: eukaryotic translation initiation factor 3 subunit M [Vitis vinifera]
2773	1874	gi|225452328	48.14	5	1	1		44293	PREDICTED: altered inheritance of mitochondria protein 32 [Vitis vinifera]
2138	29428	gi|566192701	48.13	2	1	1		104522	DNA gyrase subunit A family protein [Populus trichocarpa]
2331	19773	gi|566160873	48.11	9	1	1	Carbamidomethylation	25118	hypothetical protein POPTR_0003s04700g [Populus trichocarpa]
2510	29076	gi|566182607	48.09	3	2	1		74847	hypothetical protein POPTR_0008s06060g [Populus trichocarpa]
1653	29254	gi|225448661	48.04	16	2	2		14325	PREDICTED: 40S ribosomal protein S26-3 [Vitis vinifera]
2564	2806	gi|731411414	47.95	3	1	1		57917	PREDICTED: prolycopene isomerase chloroplastic isoform X2 [Vitis vinifera]
2291	30025	gi|225434658	47.94	15	2	2		9074	PREDICTED: uncharacterized protein LOC100265554 [Vitis vinifera]
3328	19112	gi|566150079	47.94	1	1	1		116365	hypothetical protein POPTR_0001s20900g [Populus trichocarpa]
2743	3398	gi|225424148	47.92	2	2	1	Carbamidomethylation	98262	PREDICTED: ALG-2 interacting protein X [Vitis vinifera]
2697	1261	gi|225452863	47.89	2	1	1	Carbamidomethylation	58071	PREDICTED: KH domain-containing protein At4g18375 [Vitis vinifera]
2833	29600	gi|224128744	47.86	7	1	1		19295	hypothetical protein POPTR_0014s13900g [Populus trichocarpa]

2371	29858	gi|731388004	47.86	8	1	1		18213	PREDICTED: heterogeneous nuclear ribonucleoprotein F isoform X2 [Vitis vinifera]
2284	19030	gi|566167266	47.79	4	1	1		37830	hypothetical protein POPTR_0004s18610g [Populus trichocarpa]
1251	2504	gi|731416298	47.79	4	1	1		25123	PREDICTED: eukaryotic translation initiation factor 3 subunit J [Vitis vinifera]
2894	29711	gi|224140059	47.73	6	1	1	Carbamidomethylation	29293	alcohol dehydroge family protein [Populus trichocarpa]
2945	29292	gi|225462452	47.72	5	2	2		40230	PREDICTED: GDSL esterase/lipase At5g45670 [Vitis vinifera]
3691	29834	gi|566155559	47.68	5	1	1		39393	hypothetical protein POPTR_0002s00720g [Populus trichocarpa]
2486	29349	gi|224085427	47.67	3	1	1		54899	hypothetical protein POPTR_0005s22910g [Populus trichocarpa]
2493	2414	gi|225437209	47.66	4	1	1		28420	PREDICTED: NADH dehydrogenase [ubiquinone] flavoprotein 2 mitochondrial [Vitis vinifera]
2599	18515	gi|566245088	47.63	3	1	1		33717	Xyloglucan endotransglucosylase/hydrolase protein 9 precursor [Populus trichocarpa]
2372	2189	gi|526118301	47.62	4	1	1		41463	cysteine protease precursor [Vitis vinifera]
2135	18776	gi|566201937	47.61	19	2	2	Carbamidomethylation	9653	hypothetical protein POPTR_0014s02030g [Populus trichocarpa]
3555	29714	gi|224077096	47.6	5	1	1		33993	hypothetical protein POPTR_0004s04550g [Populus trichocarpa]
1875	29429	gi|224146385	47.6	9	1	1		15015	hypothetical protein POPTR_0019s11300g [Populus trichocarpa]

2370	19371	gi|566184421	47.52	3	1	1		45388	hypothetical protein POPTR_0008s17030g [Populus trichocarpa]
2144	2104	gi|225456602	47.43	6	1	1		23813	PREDICTED: germin-like protein subfamily T member 2 [Vitis vinifera]
1254	2356	gi|359497418	47.43	10	1	1		11555	PREDICTED: mitochondrial pyruvate carrier 2-like partial [Vitis vinifera]
2278	5018	gi|225430908	47.34	2	1	1		49271	PREDICTED: putative RNA-binding protein Luc7-like 2 [Vitis vinifera]
1846	29167	gi|566192270	47.24	14	2	1		12361	Vacuolar ATP synthase subunit G 2 family protein [Populus trichocarpa]
2230	3260	gi|731422518	47.19	2	2	1		100348	PREDICTED: DNA (cytosine-5)-methyltransferase CMT2-like isoform X1 [Vitis vinifera]
2526	19499	gi|566209050	47.08	2	1	1		62817	hypothetical protein POPTR_0016s06480g [Populus trichocarpa]
2483	29159	gi|566185166	47.05	4	2	2		63357	hypothetical protein POPTR_0008s21080g [Populus trichocarpa]
2967	29873	gi|225454793	46.94	13	1	1		11346	PREDICTED: NADH dehydrogenase [ubiquinone] iron-sulfur protein 6 mitochondrial [Vitis vinifera]
2903	19878	gi|224086791	46.81	3	1	1		51097	hypothetical protein POPTR_0006s03490g [Populus trichocarpa]
3694	19442	gi|224094580	46.75	4	1	1		38015	hypothetical protein POPTR_0007s12020g [Populus trichocarpa]
2609	18539	gi|224092348	46.61	11	1	1	Carbamidomethylation	10699	hypothetical protein POPTR_0006s25990g [Populus trichocarpa]
1740	2187	gi|359496589	46.61	3	1	1		41483	PREDICTED: clathrin light chain 1 [Vitis vinifera]

2815	5621	gi|225430474	46.59	2	2	2		135946	PREDICTED: DNA-directed RNA polymerases IV and V subunit 2-like [Vitis vinifera]
2884	19210	gi|566157463	46.52	1	1	1		101527	hypothetical protein POPTR_0002s11380g [Populus trichocarpa]
1767	29015	gi|566234018	46.47	3	2	2		96141	hypothetical protein POPTR_0019s07200g [Populus trichocarpa]
2506	3405	gi|225458227	46.47	2	1	1		41470	PREDICTED: ornithine carbamoyltransferase chloroplastic [Vitis vinifera]
2017	19259	gi|566215941	46.44	2	1	1		50246	hypothetical protein POPTR_0018s14790g [Populus trichocarpa]
2290	29878	gi|225428753	46.41	6	1	1	Carbamidomethylation	33195	PREDICTED: probable carboxylesterase 12 [Vitis vinifera]
1112	1764	gi|359478415	46.41	3	2	1		58697	PREDICTED: beta-glucosidase 12 [Vitis vinifera]
2570	1816	gi|225433688	46.39	3	1	1		38722	PREDICTED: eukaryotic translation initiation factor 3 subunit H [Vitis vinifera]
2211	29489	gi|566206658	46.38	3	1	1		42473	hypothetical protein POPTR_0015s08730g [Populus trichocarpa]
2333	19771	gi|224106191	46.38	3	1	1	Carbamidomethylation	35572	cinnamyl-alcohol dehydrogenase family protein [Populus trichocarpa]
2965	29863	gi|731432620	46.11	3	1	1		72654	PREDICTED: calcium homeostasis endoplasmic reticulum protein-like isoform X2 [Vitis vinifera]
2487	19052	gi|224056427	46.1	3	1	1		46693	C2 domain-containing family protein [Populus trichocarpa]
2612	1626	gi|359482440	46.04	2	1	1		57853	PREDICTED: alanine aminotransferase 2 [Vitis vinifera]
1958	19216	gi|566170063	46.01	4	1	1		32177	hypothetical protein POPTR_0005s07750g [Populus trichocarpa]
2362	19316	gi|731386000	45.98	1	1	1		130746	PREDICTED: trafficking protein particle complex subunit 9 isoform X2 [Vitis vinifera]

3535	29622	gi|224098848	45.94	4	1	1		30293	hypothetical protein POPTR_0008s08330g [Populus trichocarpa]
1978	1733	gi|225429462	45.9	2	2	2		71749	PREDICTED: far upstream element-binding protein 2 [Vitis vinifera]
3437	29609	gi|566210253	45.84	6	1	1	Carbamidomethylation	25571	hypothetical protein POPTR_0016s14360g [Populus trichocarpa]
3335	19398	gi|566193348	45.81	2	1	1	Carbamidomethylation	84956	hypothetical protein POPTR_0011s02570g [Populus trichocarpa]
1139	29374	gi|225437358	45.81	2	2	1		59869	PREDICTED: beta-glucosidase 11-like isoform X2 [Vitis vinifera]
2875	20322	gi|224118700	45.8	2	1	1		66027	transducin family protein [Populus trichocarpa]
2957	19925	gi|224079549	45.74	3	1	1	Carbamidomethylation	44567	METHIONINE AMINOPEPTIDASE 2A family protein [Populus trichocarpa]
2966	29869	gi|224113857	45.68	9	1	1	Carbamidomethylation	27332	hypothetical protein POPTR_0011s00810g [Populus trichocarpa]
1870	1097	gi|731384037	45.68	5	2	2		37513	PREDICTED: proline iminopeptidase isoform X2 [Vitis vinifera]
2008	29294	gi|224140325	45.61	4	2	1		95901	GAMMA-ADAPTIN 1 family protein [Populus trichocarpa]
2872	29158	gi|566170688	45.28	1	2	2	Carbamidomethylation	204912	DNA-directed RNA polymerase II largest subunit family protein [Populus trichocarpa]
2610	19881	gi|224128482	45.26	13	1	1	Carbamidomethylation	9705	NADH-ubiquinone oxidoreductase-related family protein [Populus trichocarpa]
2879	5240	gi|225456794	45.22	4	1	1	Carbamidomethylation	35026	PREDICTED: haloacid dehalogenase-like hydrolase domain-containing protein At3g48420 [Vitis vinifera]
2090	2487	gi|225438081	45.19	5	2	2		51926	PREDICTED: casein kinase I-like [Vitis vinifera]

2824	29451	gi|566161694	45.09	3	1	1		48027	hypothetical protein POPTR_0003s08980g [Populus trichocarpa]
2373	19960	gi|566150184	45.03	5	1	1		23580	hypothetical protein POPTR_0001s21460g [Populus trichocarpa]
2594	3547	gi|731380326	45.03	5	2	2		36873	PREDICTED: palmitoyl-protein thioesterase 1-like [Vitis vinifera]
2567	2457	gi|526117557	45.02	4	1	1		32485	alpha-soluble NSF attachment protein [Vitis vinifera]
3422	2143	gi|225444537	45.01	3	1	1	Carbamidomethylation	42935	PREDICTED: uncharacterized protein LOC100253669 [Vitis vinifera]
2437	19146	gi|566160209	44.98	2	1	1	Carbamidomethylation	54758	hypothetical protein POPTR_0003s011001g partial [Populus trichocarpa]
3699	29924	gi|224064766	44.89	2	1	1	Carbamidomethylation	95047	transport family protein [Populus trichocarpa]
3338	2231	gi|225456155	44.81	2	1	1		64675	PREDICTED: translocon at the outer membrane of chloroplasts 64 [Vitis vinifera]
3695	29875	gi|566179360	44.8	4	1	1		46766	hypothetical protein POPTR_0007s03190g [Populus trichocarpa]
2057	19886	gi|224098816	44.71	3	1	1		45803	latex abundant family protein [Populus trichocarpa]
3545	29660	gi|566189493	44.56	4	1	1		42450	hypothetical protein POPTR_0010s06900g [Populus trichocarpa]
2613	29927	gi|225454190	44.51	5	1	1		23301	PREDICTED: uncharacterized protein LOC100244723 [Vitis vinifera]
2968	29876	gi|225456211	44.5	4	1	1		40298	PREDICTED: deoxyhypusine synthase [Vitis vinifera]
3696	29886	gi|359490488	44.5	8	1	1		33858	PREDICTED: short-chain dehydrogenase TIC 32 chloroplastic isoform X1 [Vitis vinifera]
3442	24309	gi|566183391	44.49	5	1	1		24918	hypothetical protein POPTR_0008s11090g [Populus trichocarpa]

3546	29662	gi|225429824	44.42	2	1	1		51346	PREDICTED: uncharacterized protein LOC100254151 [Vitis vinifera]
2969	29887	gi|224118830	44.35	10	1	1		12613	NADH2 dehydrogenase family protein [Populus trichocarpa]
3421	29415	gi|359487063	44.33	1	1	1		109568	PREDICTED: valine--tRNA ligase mitochondrial-like [Vitis vinifera]
3537	18968	gi|224103873	44.31	4	1	1	Carbamidomethylation	35544	putative cinnamoyl-CoA reductase family protein [Populus trichocarpa]
3439	29650	gi|225443254	44.3	7	1	1	Carbamidomethylation	28438	PREDICTED: RNA-binding protein 42 [Vitis vinifera]
2568	19607	gi|224093148	44.24	5	1	1		29641	Tetrapyrrole-binding family protein [Populus trichocarpa]
2234	18735	gi|224062667	44.2	3	1	1		43605	enoyl-CoA hydratase/isomerase family protein [Populus trichocarpa]
2258	1695	gi|731408423	44.17	4	2	2	Carbamidomethylation	43098	PREDICTED: serine carboxypeptidase-like 18 isoform X4 [Vitis vinifera]
2614	19816	gi|566197446	44.12	3	1	1		44436	hypothetical protein POPTR_0012s08440g [Populus trichocarpa]
2375	19820	gi|566210973	44.07	6	1	1		15927	hypothetical protein POPTR_0017s02810g [Populus trichocarpa]
2828	29474	gi|566187252	43.95	2	1	1		66494	hypothetical protein POPTR_0009s10080g [Populus trichocarpa]
2018	29895	gi|224080484	43.94	11	1	1		16572	Vacuolar ATP synthase 16 kDa proteolipid subunit 1/3/5 family protein [Populus trichocarpa]
1250	2898	gi|225431633	43.91	2	1	1		38428	PREDICTED: ubiquitin carboxyl-terminal hydrolase isozyme L5 [Vitis vinifera]
2904	20082	gi|566259648	43.9	7	1	1		16066	hypothetical protein POPTR_0026s00220g [Populus trichocarpa]
1514	29330	gi|566210077	43.88	6	2	1		28007	ferritin precursor family protein [Populus trichocarpa]

3698	7381	gi|731386977	43.85	2	1	1		63982	PREDICTED: D-2-hydroxyglutarate dehydrogenase mitochondrial isoform X2 [Vitis vinifera]
3697	29905	gi|731389767	43.78	1	1	1		148262	PREDICTED: uncharacterized protein LOC100251059 isoform X2 [Vitis vinifera]
2970	6196	gi|225428342	43.78	2	1	1		63050	PREDICTED: glutamate--tRNA ligase chloroplastic/mitochondrial [Vitis vinifera]
2191	29584	gi|566211007	43.66	7	1	1		20326	HMGI/Y family protein [Populus trichocarpa]
2520	20552	gi|731427542	43.66	5	1	1		40531	PREDICTED: cinnamoyl-CoA reductase 2 isoform X1 [Vitis vinifera]
2611	29892	gi|731419051	43.65	3	1	1		43398	PREDICTED: anthranilate phosphoribosyltransferase chloroplastic isoform X2 [Vitis vinifera]
3326	19138	gi|566161892	43.61	4	1	1	Carbamidomethylation	62833	hypothetical protein POPTR_0003s09970g [Populus trichocarpa]
2330	19131	gi|224073524	43.5	8	1	1		16505	hypothetical protein POPTR_0003s03980g [Populus trichocarpa]
2817	17835	gi|566173634	43.49	1	1	1		121829	hypothetical protein POPTR_0005s27880g [Populus trichocarpa]
3420	29413	gi|566213840	43.48	1	1	1	Carbamidomethylation	100766	hypothetical protein POPTR_0018s02410g [Populus trichocarpa]
2376	19810	gi|566163928	43.48	6	1	1		21587	disease resistance-responsive family protein [Populus trichocarpa]
2525	29670	gi|731425425	43.48	5	1	1		32384	PREDICTED: phosphoserine phosphatase chloroplastic [Vitis vinifera]
2560	19884	gi|225423730	43.47	6	1	1		16692	PREDICTED: glycine cleavage system H protein 2 mitochondrial [Vitis vinifera]
2874	18122	gi|224085976	43.42	2	1	1		82530	prolyl oligopeptidase family protein [Populus trichocarpa]

2819	29249	gi|224100879	43.42	2	1	1	Carbamidomethylation	41126	hypothetical protein POPTR_0008s04540g [Populus trichocarpa]
1660	19118	gi|224124994	43.37	5	1	1		17963	hypothetical protein POPTR_0013s00850g [Populus trichocarpa]
488	29953	gi|566172133	43.23	4	1	1		26305	hypothetical protein POPTR_0005s19050g [Populus trichocarpa]
3268	19509	gi|566157213	43.18	3	1	1		58089	seryl-tRNA synthetase family protein [Populus trichocarpa]
2891	19484	gi|566208951	43.15	3	1	1		47348	hypothetical protein POPTR_0016s06010g [Populus trichocarpa]
2374	29923	gi|225459577	43.15	12	1	1		12290	PREDICTED: acetyltransferase At1g77540 [Vitis vinifera]
3272	29668	gi|566207628	43.12	3	1	1		71422	hypothetical protein POPTR_0015s14790g [Populus trichocarpa]
3567	29925	gi|566154461	43.01	4	1	1		46632	DNA repair protein recA [Populus trichocarpa]
2954	18613	gi|224109026	42.89	2	1	1		65580	diminuto family protein [Populus trichocarpa]
3700	29926	gi|566152935	42.88	3	1	1	Carbamidomethylation	44131	hypothetical protein POPTR_0001s35410g [Populus trichocarpa]
1469	19970	gi|224135953	42.79	16	1	1		6931	40S ribosomal protein S30 [Populus trichocarpa]
2885	19961	gi|566204052	42.75	2	1	1		46261	hypothetical protein POPTR_0014s13120g [Populus trichocarpa]
2448	20025	gi|566211368	42.73	6	2	2		45725	hypothetical protein POPTR_0017s04570g [Populus trichocarpa]
2882	29625	gi|566172500	42.72	2	1	1		66332	hypothetical protein POPTR_0005s21350g [Populus trichocarpa]
2971	29951	gi|566180089	42.69	4	1	1		32542	hypothetical protein POPTR_0007s07510g [Populus trichocarpa]

3701	29935	gi|225458607	42.59	3	1	1		70485	PREDICTED: protein CYPRO4 [Vitis vinifera]
2616	5615	gi|225470242	42.59	6	1	1		16680	PREDICTED: uncharacterized protein At5g48480 [Vitis vinifera]
3551	19079	gi|224078604	42.47	3	1	1		41098	ATP synthase gamma chain 1 family protein [Populus trichocarpa]
2883	29627	gi|566215393	42.45	7	1	1		22604	putative SNARE family protein [Populus trichocarpa]
3531	29585	gi|566147174	42.36	2	1	1		80454	hypothetical protein POPTR_0001s04370g [Populus trichocarpa]
3702	18833	gi|224145402	42.36	17	1	1		8337	ubiquinol-cytochrome C reductase complex ubiquinone-binding family protein [Populus trichocarpa]
3703	29940	gi|225459538	42.28	5	1	1	Carbamidomethylation	43711	PREDICTED: lysM domain-containing GPI-anchored protein 1 [Vitis vinifera]
1796	29059	gi|224062521	42.19	2	2	1	Carbamidomethylation	59330	RAN GTPASE ACTIVATING protein 1 [Populus trichocarpa]
3704	19184	gi|731399255	42.15	3	1	1		52783	PREDICTED: dnaJ protein P58IPK homolog [Vitis vinifera]
2615	19033	gi|526117729	42.04	3	1	1		61635	9 10[9' 10']carotenoid cleavage dioxygenase [Vitis vinifera]
3550	29687	gi|731433331	42.01	2	1	1	Carbamidomethylation	69344	PREDICTED: probable 28S rRNA (cytosine(4447)-C(5))-methyltransferase isoform X2 [Vitis vinifera]
2103	2206	gi|225455268	41.97	5	2	2		34609	PREDICTED: probable 6-phosphogluconolactonase 4 chloroplastic [Vitis vinifera]
2776	29450	gi|224099567	41.91	6	1	1	Carbamidomethylation	40920	semialdehyde dehydrogenase family protein [Populus trichocarpa]
2561	1933	gi|225455762	41.89	2	1	1		67078	PREDICTED: probable phenylalanine--tRNA ligase beta subunit [Vitis vinifera]
1193	29787	gi|224102583	41.67	2	3	1		93021	hypothetical protein POPTR_0008s20490g [Populus trichocarpa]

3529	29573	gi|731417029	41.64	3	1	1		55196	PREDICTED: phosphoribosylaminoimidazole carboxylase chloroplastic isoform X3 [Vitis vinifera]
2888	19891	gi|566202809	41.6	12	1	1		8349	Protein transport protein SEC61 beta subunit [Populus trichocarpa]
1004	19403	gi|566166342	41.51	1	1	1		95030	hypothetical protein POPTR_0004s13420g partial [Populus trichocarpa]
3427	29456	gi|224120424	41.49	2	1	1		147785	translation initiation factor family protein [Populus trichocarpa]
2566	2662	gi|225427778	41.48	3	1	1		39091	PREDICTED: epimerase family protein SDR39U1 homolog chloroplastic [Vitis vinifera]
3433	29503	gi|731420110	41.48	2	1	1		82139	PREDICTED: translation initiation factor eIF-2B subunit epsilon [Vitis vinifera]
2173	19883	gi|566189043	41.42	4	1	1	Carbamidomethylation	36173	Peroxidase 6 precursor family protein [Populus trichocarpa]
2360	29422	gi|225451641	41.41	5	2	2		32577	PREDICTED: histidine biosynthesis bifunctional protein hisIE chloroplastic [Vitis vinifera]
3428	2631	gi|225450051	41.29	3	1	1		39579	PREDICTED: cycloartenol-C-24-methyltransferase [Vitis vinifera]
2617	19806	gi|224113431	41.23	11	1	1		9789	hypothetical protein POPTR_0010s24670g [Populus trichocarpa]
2972	20226	gi|566161648	41.11	5	1	1		17801	glycine cleavage system protein H precursor [Populus trichocarpa]
2975	29978	gi|224065328	41.02	5	1	1		21889	hypothetical protein POPTR_0002s24210g [Populus trichocarpa]
2745	29462	gi|225435323	41.01	2	1	1	Carbamidomethylation	68176	PREDICTED: protein SAND [Vitis vinifera]
3707	19595	gi|566192632	40.9	3	1	1		35033	nodulin 35 family protein [Populus trichocarpa]
2518	1478	gi|225460855	40.88	2	1	1	Carbamidomethylation	61200	PREDICTED: trigger factor-like protein TIG Chloroplastic [Vitis vinifera]

2952	1778	gi|731427762	40.83	1	1	1		78808	PREDICTED: prolyl endopeptidase isoform X2 [Vitis vinifera]
3538	19343	gi|224115158	40.75	8	1	1		16558	RNA-binding family protein [Populus trichocarpa]
3705	19463	gi|566214148	40.72	3	1	1		45872	hypothetical protein POPTR_0018s04060g [Populus trichocarpa]
2973	18956	gi|566162026	40.7	6	1	1	Carbamidomethylation	27183	hypothetical protein POPTR_0003s10720g [Populus trichocarpa]
2460	18638	gi|224093896	40.66	3	1	1		27844	hypothetical protein POPTR_0007s06770g [Populus trichocarpa]
2827	2014	gi|225446046	40.64	3	1	1		42983	PREDICTED: afadin- and alpha-actinin-binding protein isoform X2 [Vitis vinifera]
3534	29621	gi|225436041	40.63	4	1	1		36603	PREDICTED: uncharacterized protein LOC100241087 [Vitis vinifera]
2020	2500	gi|526117641	40.6	4	1	1		21825	type II peroxiredoxin F [Vitis vinifera]
3548	29674	gi|731425417	40.5	3	1	1		27200	PREDICTED: glycine-rich RNA-binding protein blt801 [Vitis vinifera]
1140	20079	gi|566163874	40.47	2	1	1		47484	hypothetical protein POPTR_0003s21170g [Populus trichocarpa]
2110	19364	gi|566164258	40.42	2	1	1		59785	hypothetical protein POPTR_0004s00900g [Populus trichocarpa]
2774	5994	gi|731375872	40.41	2	1	1		73729	PREDICTED: signal recognition particle subunit SRP72 [Vitis vinifera]
3271	2239	gi|731386510	40.36	3	1	1		41384	PREDICTED: protein DJ-1 homolog D [Vitis vinifera]
2168	29470	gi|225456991	40.33	10	1	1		13364	PREDICTED: glutaredoxin [Vitis vinifera]
1872	20302	gi|566170882	40.32	8	2	2		21960	HISTONE H1-3 family protein [Populus trichocarpa]

2698	29178	gi|224076226	40.26	9	2	2		37465	Peroxidase 42 precursor family protein [Populus trichocarpa]
2871	25302	gi|224093876	40.25	7	2	2		41205	GTP-binding family protein [Populus trichocarpa]
2892	29705	gi|225430808	40.18	4	1	1		40179	PREDICTED: splicing factor 3B subunit 4 [Vitis vinifera]
2821	20252	gi|566215395	40.17	5	1	1		21500	hypothetical protein POPTR_0018s11910g [Populus trichocarpa]
3530	29576	gi|566155359	40.16	1	1	1		149105	VARICOSE family protein [Populus trichocarpa]
3549	29685	gi|731428902	40.11	1	1	1		116462	PREDICTED: pre-mRNA-processing protein 40A [Vitis vinifera]
2285	29551	gi|225458850	40.01	4	1	1		21998	PREDICTED: LIM domain-containing protein WLIM1 [Vitis vinifera]
3708	29982	gi|225456479	40	2	1	1		67613	PREDICTED: signal recognition particle subunit SRP68 [Vitis vinifera]
2618	29975	gi|225432652	39.86	4	1	1	Carbamidomethylation	33077	PREDICTED: uncharacterized protein LOC100242666 [Vitis vinifera]
3533	29611	gi|731438931	39.84	3	1	1		51437	PREDICTED: alpha-1 3-mannosyl-glycoprotein 2-beta-N-acetylglucosaminyltransferase isoform X2 [Vitis vinifera]
2174	19858	gi|566194536	39.73	4	1	1		28624	hypothetical protein POPTR_0011s09130g [Populus trichocarpa]
3542	29649	gi|225457574	39.63	5	1	1		31304	PREDICTED: WUSCHEL-related homeobox 8 [Vitis vinifera]
2601	29484	gi|225446724	39.61	3	1	1	Carbamidomethylation	58127	PREDICTED: glutamyl-tRNA(Gln) amidotransferase subunit A chloroplastic/mitochondrial [Vitis vinifera]
2890	19399	gi|224130486	39.6	13	1	1		9171	hypothetical protein POPTR_0014s09080g [Populus trichocarpa]

2784	29715	gi|224107511	39.54	4	1	1		30527	RNA-binding protein RNP1 precursor [Populus trichocarpa]
3706	29968	gi|566202901	39.51	3	1	1	Carbamidomethylation	44101	methionine aminopeptidase 1 family protein [Populus trichocarpa]
2976	29983	gi|566183679	39.49	2	1	1		49185	serine carboxypeptidase S10 family protein [Populus trichocarpa]
2977	18864	gi|731383454	39.27	4	1	1		43734	PREDICTED: probable N-acetyl-gamma-glutamyl-phosphate reductase chloroplastic [Vitis vinifera]
2379	2858	gi|731411254	39.26	5	1	1		21305	PREDICTED: 22.0 kDa class IV heat shock protein-like [Vitis vinifera]
3547	2876	gi|731430482	39.15	3	1	1	Carbamidomethylation	41987	PREDICTED: angio-associated migratory cell protein [Vitis vinifera]
3175	29460	gi|224137970	39.11	4	1	1	Carbamidomethylation	33648	DNAJ heat shock N-terminal domain-containing family protein [Populus trichocarpa]
899	20075	gi|224092174	39.11	4	1	1		36141	hypothetical protein POPTR_0006s24360g [Populus trichocarpa]
2195	1665	gi|225459083	39.06	2	1	1		58401	PREDICTED: argininosuccinate lyase chloroplastic [Vitis vinifera]
2889	29680	gi|566184371	38.93	2	1	1		45333	hypothetical protein POPTR_0008s16710g [Populus trichocarpa]
3709	5408	gi|225426544	38.93	13	1	1		11408	PREDICTED: 60S acidic ribosomal protein P1 [Vitis vinifera]
1991	2790	gi|225455278	38.8	18	1	1	Oxidation (M)	15102	PREDICTED: cytochrome b5 isoform E [Vitis vinifera]
2111	2574	gi|225434245	38.79	3	1	1		38123	PREDICTED: 4-hydroxy-tetrahydrodipicolinate reductase 1 chloroplastic [Vitis vinifera]
2978	19943	gi|566210850	38.77	3	1	1		41273	hypothetical protein POPTR_0017s02230g [Populus trichocarpa]
2450	29305	gi|566167023	38.71	2	1	1		50772	hypothetical protein POPTR_0004s17420g [Populus trichocarpa]

2606	29567	gi|225448511	38.55	2	1	1		59202	PREDICTED: sorting and assembly machinery component 50 homolog B [Vitis vinifera]
2619	30004	gi|566173253	38.51	2	1	1		57186	hypothetical protein POPTR_0005s25730g [Populus trichocarpa]
3544	29659	gi|224066789	38.44	2	1	1	Carbamidomethylation	57660	hypothetical protein POPTR_0002s07790g [Populus trichocarpa]
2573	30006	gi|225436773	38.42	4	1	1		36439	PREDICTED: OTU domain-containing protein 6B [Vitis vinifera]
3434	29504	gi|566174766	38.4	3	1	1	Carbamidomethylation	52170	hypothetical protein POPTR_0006s06160g [Populus trichocarpa]
3443	18831	gi|224126789	38.4	2	1	1		65325	pyridine nucleotide-disulfide oxidoreductase family protein [Populus trichocarpa]
2107	1676	gi|225428560	38.35	2	2	1		83942	PREDICTED: phospholipase A-2-activating protein isoform X3 [Vitis vinifera]
763	3445	gi|731406152	38.33	1	1	1		209109	PREDICTED: dedicator of cytokinesis protein 8 isoform X3 [Vitis vinifera]
2777	2388	gi|731424368	38.32	2	1	1		72886	PREDICTED: pentatricopeptide repeat-containing protein At3g49240 [Vitis vinifera]
3712	19906	gi|225459530	38.21	2	1	1		55804	PREDICTED: glutamic acid-rich protein [Vitis vinifera]
2021	19995	gi|566208495	38.17	1	1	1		80383	DNA topoisomerase 2 family protein [Populus trichocarpa]
2979	19908	gi|731409599	38.14	3	1	1		39025	PREDICTED: GDSL esterase/lipase At4g26790-like [Vitis vinifera]
3713	2602	gi|225424272	38.1	7	1	1		17200	PREDICTED: MLP-like protein 34 [Vitis vinifera]
3710	19794	gi|224073306	38.03	2	1	1		50609	hypothetical protein POPTR_0003s01420g [Populus trichocarpa]
2176	2512	gi|359491781	37.95	6	1	1		18046	PREDICTED: miraculin-like partial [Vitis vinifera]

3714	30011	gi|225435429	37.94	4	1	1		42775	PREDICTED: uncharacterized protein LOC100267343 [Vitis vinifera]
3711	2413	gi|359475077	37.92	2	1	1	Carbamidomethylation	75336	PREDICTED: tRNA-dihydrouridine(47) synthase [NAD(P)(+)]-like [Vitis vinifera]
2175	2796	gi|225450236	37.8	9	1	1		12047	PREDICTED: 40S ribosomal protein S25 [Vitis vinifera]
2336	29701	gi|225464531	37.79	2	1	1		49751	PREDICTED: BAHD acyltransferase DCR [Vitis vinifera]
3423	20023	gi|566205509	37.78	3	1	1		39789	hypothetical protein POPTR_0015s02150g [Populus trichocarpa]
3265	29414	gi|225436347	37.77	2	1	1		99445	PREDICTED: uncharacterized protein LOC100261914 [Vitis vinifera]
2523	20467	gi|566166948	37.74	1	1	1		116430	hypothetical protein POPTR_0004s16940g [Populus trichocarpa]
3543	29657	gi|566176657	37.74	3	1	1	Carbamidomethylation	47264	hypothetical protein POPTR_0006s16050g [Populus trichocarpa]
2022	2600	gi|526117629	37.74	8	1	1	Carbamidomethylation	11686	lipid transfer protein precursor [Vitis vinifera]
3539	29642	gi|566211614	37.64	2	1	1		64182	asparaginyl-tRNA synthetase family protein [Populus trichocarpa]
2556	2718	gi|731395801	37.61	10	2	2		21364	PREDICTED: nitrogen regulatory protein P-II homolog isoform X2 [Vitis vinifera]
3715	30021	gi|566168047	37.59	2	1	1		56955	serine carboxypeptidase family protein [Populus trichocarpa]
1989	20639	gi|224073090	37.47	1	1	1		72063	Histone-lysine N-methyltransferase family protein [Populus trichocarpa]
2896	19915	gi|566166166	37.41	4	1	1	Carbamidomethylation	26598	hypothetical protein POPTR_0004s11800g [Populus trichocarpa]
2361	19077	gi|566200735	37.29	5	2	1		47505	hypothetical protein POPTR_0013s11650g [Populus trichocarpa]

3331	2192	gi|731436255	37.26	3	1	1		72082	PREDICTED: LOW QUALITY PROTEIN: polyadenylate-binding protein 3 partial [Vitis vinifera]
3429	1750	gi|225447488	37.26	2	1	1		63568	PREDICTED: uncharacterized protein LOC100266500 [Vitis vinifera]
2563	29664	gi|566187835	37.25	3	1	1		45220	hypothetical protein POPTR_0009s13600g [Populus trichocarpa]
3559	29733	gi|224142329	37.25	4	1	1		30751	tetratricopeptide repeat-containing family protein [Populus trichocarpa]
2832	29594	gi|224082148	37.12	4	1	1		43446	hypothetical protein POPTR_0005s16700g [Populus trichocarpa]
2620	2524	gi|225452110	37.11	10	1	1	Carbamidomethylation	9858	PREDICTED: non-specific lipid-transfer protein 2-like [Vitis vinifera]
3556	2466	gi|225429195	37.1	2	1	1		63400	PREDICTED: probable zinc metallopeptidase EGY3 chloroplastic [Vitis vinifera]
2837	25594	gi|224086851	37.07	6	1	1		27383	tubulin folding cofactor B family protein [Populus trichocarpa]
2772	29357	gi|225431489	37.03	2	1	1		95225	PREDICTED: ATP-dependent zinc metalloprotease FtsH [Vitis vinifera]
2835	1711	gi|225466119	37.01	3	1	1		42250	PREDICTED: luc7-like protein 3 [Vitis vinifera]
3553	29707	gi|731393379	36.96	4	1	1		28946	PREDICTED: factor of DNA methylation 4-like [Vitis vinifera]
2574	30033	gi|224122560	36.94	12	1	1	Carbamidomethylation	11604	hypothetical protein POPTR_0012s14230g [Populus trichocarpa]
2462	19525	gi|566170977	36.89	3	1	1		29462	hypothetical protein POPTR_0005s12280g [Populus trichocarpa]
3552	29702	gi|224090669	36.8	2	1	1		50892	PDE1 SUPPRESSOR 1 family protein [Populus trichocarpa]
1754	1964	gi|731433023	36.73	6	2	1	Carbamidomethylation	39210	PREDICTED: GDSL esterase/lipase At4g26790 [Vitis vinifera]
2981	30031	gi|731396740	36.69	4	1	1		35076	PREDICTED: nuclear pore complex protein NUP58-like [Vitis vinifera]

2517	29392	gi|566182169	36.63	1	1	1	Carbamidomethylation	117003	exocyst complex component Sec8 family protein [Populus trichocarpa]
2775	29443	gi|224144994	36.62	2	1	1		46883	hypothetical protein POPTR_0019s08680g [Populus trichocarpa]
3557	20213	gi|224114830	36.58	2	1	1		47731	putative 4-methyl-5(b-hydroxyethyl)-thiazole monophosphate family protein [Populus trichocarpa]
2380	1383	gi|359476759	36.47	3	1	1		47072	PREDICTED: basic leucine zipper and W2 domain-containing protein 2 [Vitis vinifera]
3719	30058	gi|566156450	36.3	3	1	1		48749	4-HYDROXYPHENYLPYRUVATE DIOXYGENASE family protein [Populus trichocarpa]
3716	19969	gi|225469438	36.03	6	1	1		17534	PREDICTED: pollen-specific protein C13 [Vitis vinifera]
2327	3386	gi|731395454	36.01	7	2	1		19689	PREDICTED: uncharacterized protein OsI_027940 isoform X2 [Vitis vinifera]
2143	20265	gi|731387184	36	2	1	1		61791	PREDICTED: U1 small nuclear ribonucleoprotein 70 kDa [Vitis vinifera]
2108	19106	gi|224078826	35.92	4	1	1		24905	Oxygen-evolving enhancer protein 3-1 [Populus trichocarpa]
2621	20096	gi|224123444	35.92	10	1	1		14627	prefoldin family protein [Populus trichocarpa]
2982	30038	gi|566161658	35.92	6	1	1		21323	hypothetical protein POPTR_0003s08810g partial [Populus trichocarpa]
2980	30029	gi|224061849	35.89	2	1	1		79175	hypothetical protein POPTR_0002s00760g [Populus trichocarpa]
3718	4681	gi|731397495	35.88	2	1	1		70457	PREDICTED: RNA-binding protein 39 [Vitis vinifera]
3541	29647	gi|225442841	35.85	5	1	1		18913	PREDICTED: monothiol glutaredoxin-S15 mitochondrial [Vitis vinifera]

3426	19838	gi|731395888	35.82	3	1	1		44982	PREDICTED: protein notum homolog [Vitis vinifera]
2722	2135	gi|731379048	35.7	1	1	1		106833	PREDICTED: nuclear pore complex protein NUP98A [Vitis vinifera]
2905	24288	gi|224092582	35.65	9	1	1		24079	Stromal cell-derived factor 2-like protein precursor [Populus trichocarpa]
3717	19808	gi|566160273	35.6	6	1	1	Carbamidomethylation	17554	hypothetical protein POPTR_0003s01440g [Populus trichocarpa]
3720	30060	gi|225470348	35.46	3	1	1		49168	PREDICTED: trafficking protein particle complex subunit 13 [Vitis vinifera]
2907	30269	gi|225430161	35.38	6	1	1		18409	PREDICTED: N-alpha-acetyltransferase 50 [Vitis vinifera]
3721	30065	gi|731383801	35.34	3	1	1		35361	PREDICTED: DNA-directed RNA polymerases II IV and V subunit 3 [Vitis vinifera]
2329	18960	gi|566255736	35.32	5	1	1		15873	hypothetical protein POPTR_0483s00200g partial [Populus trichocarpa]
2984	19460	gi|566146659	35.31	3	1	1		54423	hypothetical protein POPTR_0001s01790g [Populus trichocarpa]
3532	2477	gi|731383208	35.26	4	1	1		29098	PREDICTED: heterodimeric geranylgeranyl pyrophosphate synthase small subunit chloroplastic-like [Vitis vinifera]
2624	30094	gi|224132038	35.25	2	1	1		55654	serine carboxypeptidase S10 family protein [Populus trichocarpa]
2985	30069	gi|225446523	35.17	8	1	1	Carbamidomethylation	40179	PREDICTED: zingipain-2 [Vitis vinifera]
3327	6818	gi|359475014	35.17	1	1	1		69107	PREDICTED: RNA-binding protein 39 [Vitis vinifera]
3724	30077	gi|566214618	35	7	1	1	Carbamidomethylation	13633	hypothetical protein POPTR_0018s06830g [Populus trichocarpa]
1328	2027	gi|225428747	34.95	3	1	1		33152	PREDICTED: 2-hydroxyisoflavanone dehydratase [Vitis vinifera]

2983	30043	gi|566206612	34.92	6	1	1		18239	hypothetical protein POPTR_0015s08470g [Populus trichocarpa]
2147	30450	gi|566257178	34.9	2	1	1	Oxidation (M)	58755	hypothetical protein POPTR_0177s00210g [Populus trichocarpa]
3329	29468	gi|225462062	34.88	1	1	1		151429	PREDICTED: enhancer of mRNA-decapping protein 4 [Vitis vinifera]
2829	20188	gi|566204034	34.88	2	1	1		54966	hypothetical protein POPTR_0014s13020g [Populus trichocarpa]
3431	29500	gi|224062736	34.84	4	1	1		36694	ribose-phosphate pyrophosphokinase 4 family protein [Populus trichocarpa]
3727	19336	gi|225444159	34.83	5	1	1		32002	PREDICTED: coatomer subunit epsilon-1 [Vitis vinifera]
3729	2420	gi|225447221	34.82	6	1	1		18627	PREDICTED: photosystem II repair protein PSB27-H1 chloroplastic [Vitis vinifera]
2986	2692	gi|359479028	34.81	3	1	1		40721	PREDICTED: probable trans-2-enoyl-CoA reductase mitochondrial [Vitis vinifera]
2622	19941	gi|566186111	34.72	8	1	1		12191	hypothetical protein POPTR_0009s03520g partial [Populus trichocarpa]
3725	20605	gi|225467859	34.66	3	1	1	Carbamidomethylation	33928	PREDICTED: chlorophyllase-1 [Vitis vinifera]
3726	30081	gi|225451663	34.63	4	1	1		29408	PREDICTED: syntaxin-22 [Vitis vinifera]
2169	20531	gi|225442133	34.61	7	2	1		22029	PREDICTED: auxin-binding protein ABP19a [Vitis vinifera]
3728	19955	gi|225445842	34.59	6	1	1		21531	PREDICTED: uncharacterized protein LOC100243229 [Vitis vinifera]
2987	30087	gi|566169425	34.55	2	1	1		65228	hypothetical protein POPTR_0005s04420g [Populus trichocarpa]
3558	19573	gi|566187444	34.53	2	1	1		54252	allene oxide synthase family protein [Populus trichocarpa]

3528	29561	gi|566209115	34.52	4	1	1	Carbamidomethylation	35581	tatD-related deoxyribonuclease family protein [Populus trichocarpa]
3730	20379	gi|566195508	34.48	2	1	1		46302	arginine/serine-rich family protein [Populus trichocarpa]
2623	30083	gi|566255151	34.39	6	1	1		20944	hypothetical protein POPTR_0661s00200g [Populus trichocarpa]
3325	29441	gi|566177781	34.31	3	1	1		47632	hypothetical protein POPTR_0006s23550g [Populus trichocarpa]
2491	18988	gi|224072767	34.28	4	1	1		29070	hypothetical protein POPTR_0003s18420g [Populus trichocarpa]
3731	19369	gi|224067716	34.1	11	1	1	Carbamidomethylation	13535	hypothetical protein POPTR_0002s14760g [Populus trichocarpa]
3560	29743	gi|566155545	34.09	4	1	1	Carbamidomethylation	38236	hypothetical protein POPTR_0002s00650g [Populus trichocarpa]
2989	30099	gi|731386598	34.07	2	1	1		40869	PREDICTED: GDSL esterase/lipase At5g33370 [Vitis vinifera]
3218	29644	gi|566209274	34.02	1	1	1		66062	hypothetical protein POPTR_0016s07950g [Populus trichocarpa]
2237	4252	gi|359488906	34.01	2	2	2	Oxidation (M)	135812	PREDICTED: ABC transporter B family member 19 [Vitis vinifera]
3723	30070	gi|225443988	34.01	7	1	1		13228	PREDICTED: photosystem I reaction center subunit psaK chloroplastic [Vitis vinifera]
3722	19986	gi|224117028	33.9	8	1	1		15358	hypothetical protein POPTR_0011s11080g [Populus trichocarpa]
2895	29723	gi|224114964	33.87	5	1	1		34813	hypothetical protein POPTR_0011s12160g [Populus trichocarpa]
2988	19544	gi|566164408	33.83	2	1	1		45443	RNA-binding protein 45 [Populus trichocarpa]
3732	30120	gi|731428892	33.76	10	1	1		15960	PREDICTED: uncharacterized protein LOC100259164 isoform X2 [Vitis vinifera]

2451	6489	gi|225432798	33.7	3	1	1		38396	PREDICTED: perakine reductase [Vitis vinifera]
2994	30176	gi|731396112	33.7	3	1	1		55464	PREDICTED: glutathione gamma-glutamylcysteinyltransferase 1 [Vitis vinifera]
2900	2793	gi|731369035	33.63	4	1	1		29422	PREDICTED: anthranilate synthase beta subunit 1 chloroplastic-like isoform X2 [Vitis vinifera]
3561	21049	gi|566164165	33.53	3	1	1		36059	chromoplast-specific carotenoid-associated protein CHRC [Populus trichocarpa]
2990	30127	gi|359479048	33.4	7	1	1		17061	PREDICTED: protein disulfide isomerase-like 5-1 [Vitis vinifera]
2559	29541	gi|224053282	33.22	2	1	1		51942	Uridine 5'-monophosphate synthase family protein [Populus trichocarpa]
2142	29648	gi|359473178	33.07	4	1	1		40072	PREDICTED: 2-methylene-furan-3-one reductase [Vitis vinifera]
1560	2681	gi|225455153	33.05	4	1	1		26938	PREDICTED: mitochondrial import inner membrane translocase subunit TIM22-like [Vitis vinifera]
3540	29643	gi|225434205	32.86	5	1	1		20681	PREDICTED: 60S ribosome subunit biogenesis protein NIP7 homolog [Vitis vinifera]
2572	20159	gi|731382351	32.83	2	1	1		43783	PREDICTED: uncharacterized protein LOC100245856 [Vitis vinifera]
3435	29510	gi|566209622	32.82	1	1	1		89923	hypothetical protein POPTR_0016s10620g [Populus trichocarpa]
3438	29640	gi|566202105	32.79	6	1	1		23731	hypothetical protein POPTR_0014s02820g [Populus trichocarpa]
2991	2748	gi|526117331	32.54	7	1	1		14247	thioredoxin h [Vitis vinifera]
2522	19256	gi|224072659	32.52	2	1	1		53805	glutathione-disulfide reductase family protein [Populus trichocarpa]
2785	29749	gi|566199841	32.43	2	1	1		73415	hypothetical protein POPTR_0013s06690g [Populus trichocarpa]
3036	19194	gi|224102881	32.37	3	1	1	Carbamidomethylation	45777	tRNA synthetase class 1 family protein [Populus trichocarpa]

2992	30147	gi|225434417	32.22	6	1	1		26437	PREDICTED: U2 small nuclear ribonucleoprotein B" 2 [Vitis vinifera]
3563	6909	gi|225438579	32.02	3	1	1		39381	PREDICTED: uncharacterized protein LOC100247396 [Vitis vinifera]
3176	2594	gi|731382912	31.97	2	1	1		81725	PREDICTED: subtilisin-like protease [Vitis vinifera]
2898	29748	gi|566162341	31.93	3	1	1		68078	hypothetical protein POPTR_0003s12640g [Populus trichocarpa]
2337	29778	gi|224135649	31.87	3	1	1		28394	hypothetical protein POPTR_0015s07660g [Populus trichocarpa]
2747	29683	gi|566197448	31.85	9	1	1	Carbamidomethylation	12293	hypothetical protein POPTR_0012s08450g [Populus trichocarpa]
3267	29437	gi|224095855	31.85	2	1	1		76011	hypothetical protein POPTR_0007s03550g [Populus trichocarpa]
3562	29759	gi|225439352	31.78	2	1	1		50930	PREDICTED: fasciclin-like arabinogalactan protein 17 [Vitis vinifera]
497	20523	gi|224123460	31.58	4	1	1		20643	hypothetical protein POPTR_0013s04000g [Populus trichocarpa]
3735	19979	gi|566188099	31.39	12	1	1		8182	hypothetical protein POPTR_0009s15150g [Populus trichocarpa]
3424	29432	gi|731417542	31.34	4	1	1		40609	PREDICTED: pyridoxal kinase isoform X5 [Vitis vinifera]
2569	29717	gi|225465619	31.24	7	1	1		26018	PREDICTED: thioredoxin domain-containing protein PLP3A isoform X2 [Vitis vinifera]
3733	19232	gi|224077516	31.22	2	1	1		59212	4-coumarate--CoA ligase family protein [Populus trichocarpa]
3736	2759	gi|225464240	31.02	5	1	1		20829	PREDICTED: uncharacterized protein At4g13200 chloroplastic [Vitis vinifera]

3734	30164	gi|225448041	30.94	2	1	1		57832	PREDICTED: uncharacterized protein LOC100246257 [Vitis vinifera]
3217	29368	gi|224100723	30.93	1	1	1		197122	KOW domain-containing transcription factor family protein [Populus trichocarpa]
2899	20086	gi|224096333	30.73	2	1	1		57960	hypothetical protein POPTR_0007s06670g [Populus trichocarpa]
3566	29795	gi|731423901	30.48	8	1	1		16577	PREDICTED: trafficking protein particle complex subunit 1 isoform X2 [Vitis vinifera]
2626	2815	gi|225445984	30.44	7	1	1		15131	PREDICTED: glutaredoxin-C4 [Vitis vinifera]
2993	2663	gi|359490173	30.28	4	1	1		25366	PREDICTED: clp protease-related protein At4g12060 chloroplastic [Vitis vinifera]
3564	2261	gi|359487708	30.24	3	1	1		43172	PREDICTED: flap endonuclease 1 isoform X2 [Vitis vinifera]
3266	18995	gi|566146741	30.09	2	1	1		97119	hypothetical protein POPTR_0001s02190g [Populus trichocarpa]
2995	30224	gi|566202657	30	4	1	1		20954	H+-transporting two-sector ATPase family protein [Populus trichocarpa]
2496	2682	gi|225429325	29.94	5	1	1		24120	PREDICTED: thioredoxin domain-containing protein 9 homolog [Vitis vinifera]
3737	30174	gi|224087965	29.88	4	1	1	Carbamidomethylation	35532	hypothetical protein POPTR_0006s14820g [Populus trichocarpa]
2897	2782	gi|225459768	29.85	5	1	1		17006	PREDICTED: plastocyanin [Vitis vinifera]
3738	19616	gi|566177300	29.75	3	1	1		34068	hypothetical protein POPTR_0006s20660g [Populus trichocarpa]
2382	19376	gi|566208632	29.73	4	1	1		30390	hypothetical protein POPTR_0016s04290g [Populus trichocarpa]
3739	30189	gi|225458599	29.51	3	1	1	Carbamidomethylation	30834	PREDICTED: protein TIC 22 chloroplastic [Vitis vinifera]

3568	30067	gi|359478392	29.18	2	1	1		73213	PREDICTED: outer envelope protein 80 chloroplastic [Vitis vinifera]
3740	30230	gi|225423716	29.17	3	1	1		40852	PREDICTED: glutathione S-transferase omega-like 2 [Vitis vinifera]
3270	23597	gi|731420542	29.15	1	1	1		210037	PREDICTED: eukaryotic translation initiation factor 4G isoform X1 [Vitis vinifera]
3565	29780	gi|225454892	29.09	12	1	1	Carbamidomethylation	25916	PREDICTED: polyadenylate-binding protein 2 [Vitis vinifera]
2748	29770	gi|225438787	29.08	4	1	1		36637	PREDICTED: U1 small nuclear ribonucleoprotein A isoform X2 [Vitis vinifera]
3430	18626	gi|225454791	28.64	2	1	1		70066	PREDICTED: ATPase family AAA domain-containing protein 3-B [Vitis vinifera]
3743	30257	gi|225433514	28.51	3	1	1		52339	PREDICTED: selenium-binding protein 1 [Vitis vinifera]
3744	30266	gi|731440611	28.36	6	1	1	Carbamidomethylation	32449	PREDICTED: uncharacterized protein LOC100854177 [Vitis vinifera]
3554	29712	gi|359482034	28.35	3	1	1		29126	PREDICTED: secoisolariciresinol dehydrogenase-like [Vitis vinifera]
2901	29794	gi|731406441	28.32	3	1	1		39154	PREDICTED: mitogen-activated protein kinase kinase 2 [Vitis vinifera]
3569	30116	gi|566207206	28.09	3	1	1		25482	hypothetical protein POPTR_0015s12230g [Populus trichocarpa]
3742	30245	gi|225452543	27.83	10	1	1		13965	PREDICTED: multifunctional methyltransferase subunit TRM112-like protein At1g22270 [Vitis vinifera]
3741	2785	gi|731408402	27.8	3	1	1		29415	PREDICTED: (DL)-glycerol-3-phosphatase 2 [Vitis vinifera]
2339	30263	gi|224129620	27.44	5	1	1		24277	hypothetical protein POPTR_0014s18330g [Populus trichocarpa]

2575	20266	gi|224130034	27.39	2	1	1		43250	Fasciclin-like arabinogalactan protein 8 precursor [Populus trichocarpa]
3031	29531	gi|225433902	27.28	6	1	1		19543	PREDICTED: peptidyl-prolyl cis-trans isomerase E [Vitis vinifera]
2261	29986	gi|566171231	27.06	2	1	1		50025	hypothetical protein POPTR_0005s13910g [Populus trichocarpa]
2452	29548	gi|224099505	27.06	1	1	1		62404	hypothetical protein POPTR_0008s13110g [Populus trichocarpa]
2996	30254	gi|731392009	27.04	11	1	1		9735	PREDICTED: mitochondrial import inner membrane translocase subunit TIM10 [Vitis vinifera]
3333	29557	gi|566152952	26.97	2	1	1		101224	vesicle tethering family protein [Populus trichocarpa]
3745	20346	gi|225435189	26.87	7	1	1		14801	PREDICTED: nuclear transcription factor Y subunit B-10-like [Vitis vinifera]
3570	30146	gi|566197842	26.78	1	1	1		59140	hypothetical protein POPTR_0012s10800g [Populus trichocarpa]
3572	18620	gi|225468608	26.61	3	1	1	Carbamidomethylation	35710	PREDICTED: peroxidase 66 [Vitis vinifera]
3749	30342	gi|225457225	26.53	14	1	1	Carbamidomethylation	21042	PREDICTED: PLASMODESMATA CALLOSE-BINDING PROTEIN 3 [Vitis vinifera]
3750	30343	gi|566190046	26.51	2	1	1		71866	hypothetical protein POPTR_0010s10050g [Populus trichocarpa]
2782	1915	gi|225443496	26.34	1	1	1		81071	PREDICTED: DEAD-box ATP-dependent RNA helicase 21-like [Vitis vinifera]
3334	29562	gi|731393820	26.29	1	1	1		99418	PREDICTED: protein FAR1-RELATED SEQUENCE 3 isoform X2 [Vitis vinifera]
3751	19028	gi|566198361	26.27	1	1	1		90791	hypothetical protein POPTR_0012s13720g [Populus trichocarpa]

3752	19612	gi|566148044	26.16	3	1	1	Carbamidomethylation	28219	glutathione S-transferase family protein [Populus trichocarpa]
2781	29572	gi|566178826	26.02	2	1	1		55940	hypothetical protein POPTR_0006s29500g [Populus trichocarpa]
3746	30309	gi|224088794	25.94	2	1	1		64185	conserved oligomeric Golgi complex component-related family protein [Populus trichocarpa]
3573	30301	gi|566153219	25.74	1	1	1		82405	hypothetical protein POPTR_0001s37160g [Populus trichocarpa]
2998	30399	gi|225423539	25.68	5	1	1		14982	PREDICTED: uncharacterized protein LOC100262861 [Vitis vinifera]
3342	30186	gi|731389219	25.4	6	1	1		16425	PREDICTED: probable prefoldin subunit 2 [Vitis vinifera]
1878	30440	gi|731404500	25.37	1	1	1		113106	PREDICTED: glycogen phosphorylase 1-like [Vitis vinifera]
3143	29570	gi|566187799	25.32	1	1	1		123734	hypothetical protein POPTR_0009s13380g [Populus trichocarpa]
3747	30336	gi|225456165	25.26	3	1	1		38868	PREDICTED: prostaglandin E synthase 2 [Vitis vinifera]
3336	2730	gi|225439546	25.2	1	1	1		91491	PREDICTED: probably inactive leucine-rich repeat receptor-like protein kinase IMK2 [Vitis vinifera]
3222	25258	gi|224101571	24.77	1	1	1		65688	mRNA for putative aldehyde dehydrogenase family protein [Populus trichocarpa]
2627	30394	gi|731405738	24.69	1	1	1		160488	PREDICTED: probable disease resistance protein At4g27220 isoform X1 [Vitis vinifera]
2999	30567	gi|731412337	24.63	3	1	1		33241	PREDICTED: RNA-binding protein Nova-2 isoform X3 [Vitis vinifera]
3575	30466	gi|225427112	24.62	2	1	1		40773	PREDICTED: plastoglobulin-1 chloroplastic [Vitis vinifera]
2909	30480	gi|566208493	24.48	2	1	1	Carbamidomethylation	69814	hypothetical protein POPTR_0016s03540g [Populus trichocarpa]

2453	2813	gi|225427973	24.43	3	1	1		19853	PREDICTED: glycine-rich protein 2-like [Vitis vinifera]
3754	20129	gi|224057220	24.38	4	1	1		22345	cysteine protease inhibitor family protein [Populus trichocarpa]
3755	7618	gi|731413580	24.26	3	1	1	Carbamidomethylation	41817	PREDICTED: uncharacterized protein LOC100256822 [Vitis vinifera]
3574	19644	gi|225442791	24.26	2	1	1		68124	PREDICTED: plastidic ATP/ADP-transporter [Vitis vinifera]
3343	6691	gi|225437318	24	1	1	1		157286	PREDICTED: translocase of chloroplast 159 chloroplastic [Vitis vinifera]
3756	25467	gi|225440807	23.98	8	1	1		18280	PREDICTED: peptidyl-prolyl cis-trans isomerase CYP18-2 [Vitis vinifera]
3577	30505	gi|225447498	23.89	1	1	1	Carbamidomethylation	78851	PREDICTED: long chain acyl-CoA synthetase 8 [Vitis vinifera]
3758	2988	gi|731425525	23.87	1	1	1		70478	PREDICTED: uncharacterized protein LOC100260418 isoform X3 [Vitis vinifera]
2794	23148	gi|566200455	23.59	6	1	1		23053	hypothetical protein POPTR_0013s10250g [Populus trichocarpa]
3278	30335	gi|566185108	23.59	4	1	1		20300	hypothetical protein POPTR_0008s20770g [Populus trichocarpa]
3759	30499	gi|731423435	23.59	10	1	1		17889	PREDICTED: protein phosphatase inhibitor 2 isoform X3 [Vitis vinifera]
1722	30489	gi|225458119	23.52	2	1	1		41106	PREDICTED: cysteine proteinase RD19a [Vitis vinifera]
3762	30629	gi|225451245	23.51	8	1	1		20212	PREDICTED: tRNA(adenine(34)) deaminase chloroplastic [Vitis vinifera]
2471	3251	gi|359475474	23.47	2	1	1		53125	PREDICTED: serine carboxypeptidase-like 18 [Vitis vinifera]

2387	30601	gi|731411898	23.4	3	1	1		42756	PREDICTED: aminoacyl tRNA synthase complex-interacting multifunctional protein 1 isoform X2 [Vitis vinifera]
3580	30526	gi|225458143	23.4	2	1	1	Carbamidomethylation	47431	PREDICTED: zingipain-2 [Vitis vinifera]
3225	30407	gi|224053991	23.38	1	1	1		62720	glucose-methanol-choline oxidoreductase family protein [Populus trichocarpa]
2630	30528	gi|731368974	23.04	2	1	1		52059	PREDICTED: IAA-alanine resistance protein 1 [Vitis vinifera]
3452	30647	gi|566191598	22.98	4	1	1		22740	hypothetical protein POPTR_0010s19060g [Populus trichocarpa]
2629	20108	gi|566158274	22.89	3	1	1		34050	hypothetical protein POPTR_0002s15960g [Populus trichocarpa]
3764	30652	gi|225440692	22.72	2	1	1		56466	PREDICTED: LIMR family protein At5g01460 [Vitis vinifera]
3582	21171	gi|359475270	22.49	4	1	1		20882	PREDICTED: NEDD8-conjugating enzyme Ubc12 [Vitis vinifera]
2341	5144	gi|225431120	22.48	11	1	1		23415	PREDICTED: glycine-rich protein 2-like [Vitis vinifera]
3090	23162	gi|566166782	22.47	0	1	1		223354	phosphatidylinositol 4-kinase family protein [Populus trichocarpa]
2844	18812	gi|671741947	22.39	11	1	1		9397	photosystem II cytochrome b559 alpha subunit (chloroplast) [Camellia grandibracteata]
3767	21085	gi|566187962	22.36	2	1	1		33780	Chain A family protein [Populus trichocarpa]
3768	30805	gi|566188564	22.36	3	1	1		29723	hypothetical protein POPTR_0010s00930g [Populus trichocarpa]
2912	30580	gi|225451445	22.32	5	1	1		22489	PREDICTED: mitochondrial import receptor subunit TOM20 [Vitis vinifera]
3341	19011	gi|566195960	22.2	1	1	1		205867	hypothetical protein POPTR_0011s17070g [Populus trichocarpa]

3761	2289	gi|731384376	22.04	4	1	1		35786	PREDICTED: cinnamoyl-CoA reductase 1 [Vitis vinifera]
2242	30717	gi|359491817	22.01	3	1	1	Carbamidomethylation	41965	PREDICTED: uncharacterized protein At4g15970-like [Vitis vinifera]
2749	30295	gi|566205166	21.97	2	1	1		139960	hypothetical protein POPTR_0015s00250g [Populus trichocarpa]
3578	21900	gi|566180229	21.89	2	1	1		71241	hypothetical protein POPTR_0007s08250g [Populus trichocarpa]
3590	3123	gi|359473301	21.77	2	1	1		55596	PREDICTED: KH domain-containing protein At4g18375 isoform X2 [Vitis vinifera]
2529	30330	gi|566167973	21.77	1	1	1		105310	hypothetical protein POPTR_0004s22170g [Populus trichocarpa]
3000	30644	gi|225453022	21.6	13	1	1	Carbamidomethylation	15113	PREDICTED: pathogenesis-related protein PR-4 [Vitis vinifera]
2855	30779	gi|566212673	21.58	1	1	1	Carbamidomethylation	134320	hypothetical protein POPTR_0017s11020g [Populus trichocarpa]
3769	30838	gi|225430352	21.52	19	1	1		23983	PREDICTED: stromal cell-derived factor 2-like protein [Vitis vinifera]
3593	20721	gi|225449250	21.52	9	1	1		18193	PREDICTED: 18.1 kDa class I heat shock protein [Vitis vinifera]
3005	30857	gi|566187626	21.06	3	1	1		37674	hypothetical protein POPTR_0009s12340g [Populus trichocarpa]
3460	30959	gi|225465290	21	17	1	1		7858	PREDICTED: NADH dehydrogenase [ubiquinone] 1 beta subcomplex subunit 3-B [Vitis vinifera]

Table S6 The idetified differentially expressed proteins between CK1 and HY1

Protein Group	Protein ID	Accession	Significance (-10lgP)	Coverage (%)	#Peptides	#Unique	PTM	Sample 1 Area	Sample 2 Area	Sample 3 Area	Sample 4 Area	Sample 5 Area	Sample 6 Area	Sample Profile (Ratio)	CK1 Area	H1 Area	Group Profile (Ratio)	Avg. Mass	Description
688	1634	gi|225455513	102.19	25	3	3	Carbamidomethylation; Carbamidomethylation	4.56E+06	6.72E+06	8.54E+06	4.47E+07	3.72E+07	3.84E+07	0.12:0.18:0.23:1.20:1.00:1.03	6.61E+06	4.01E+07	0.16:1.00	13646	PREDICTED: 40S ribosomal protein S20-2 [Vitis vinifera]
221	501	gi|731406213	99.59	6	5	4	Carbamidomethylation	4.75E+07	4.88E+07	4.74E+07	1.20E+08	1.10E+08	1.19E+08	0.43:0.44:0.43:1.09:1.00:1.08	4.79E+07	1.16E+08	0.41:1.00	92443	PREDICTED: sucrose synthase [Vitis vinifera]
1057	1226	gi|225436257	95.02	12	3	3		8.27E+07	6.94E+07	1.03E+08	0	0	0	1.00:0.84:1.24:0:0:0	8.50E+07	0	1.00:0	29530	PREDICTED: chlorophyll a-b binding protein 8 chloroplastic [Vitis vinifera]
1049	22099	gi|566146555	88.9	9	2	2		0	0	0	1.76E+07	1.91E+07	1.79E+07	1.00:0.93	0	1.82E+07	00:01.0	23812	protein L13
180	284	gi|225433510	64.87	4	2	1		4.53E+07	4.71E+07	4.13E+07	7.94E+07	7.53E+07	7.32E+07	0.60:0.63:0.55:1.05:1.00:0.97	4.46E+07	7.60E+07	0.59:1.00	51906	PREDICTED: serine hydroxymethyltransferase 4 [Vitis vinifera]
672	800	gi|225447576	64.52	15	4	1	Carbamidomethylation; Carbamidomethylation	7.92E+06	1.39E+08	1.54E+08	1.40E+07	1.44E+07	6.56E+06	0.55:9.63:10.69:0.97:1.00:0.45	1.12E+08	1.17E+07	9.59:1.00	28468	PREDICTED: chlorophyll a-b binding protein 151 chloroplastic [Vitis vinifera]
1306	3095	gi|526117629	63.14	8	1	1	Carbamidomethylation	5.90E+08	6.86E+08	5.86E+08	1.99E+08	1.87E+08	1.87E+08	3.15:3.66:3.13:1.06:1.00:1.00	6.21E+08	1.91E+08	3.25:1.00	11686	lipid transfer protein precursor [Vitis vinifera]
1280	1980	gi|225457389	60	5	1	1		2.99E+07	2.65E+07	2.47E+07	0	0	0	1.00:0.89:0.83:0:0:0	2.70E+07	0	1.00:0	27867	PREDICTED: chlorophyll a-b binding protein 4 chloroplastic [Vitis vinifera]
1481	3143	gi|225443988	60	7	1	1		1.72E+08	1.87E+08	1.79E+08	6.14E+07	7.10E+07	-	2.43:2.64:2.52:0.87:1.00:-	1.79E+08	6.62E+07	2.71:1.00	13228	PREDICTED: photosystem I reaction center subunit psaK chloroplastic [Vitis vinifera]
311	903	gi|566176273	60	3	3	1		1.31E+08	1.34E+08	1.37E+08	2.41E+08	2.33E+08	2.84E+08	0.56:0.58:0.59:1.03:1.00:1.22	1.34E+08	2.53E+08	0.53:1.00	92143	sucrose synthase family protein [Populus trichocarpa]
1096	22084	gi|731437331	60	6	1	1		7.73E+06	1.16E+07	5.24E+06	7.66E+07	6.32E+07	6.55E+07	0.12:0.18:0.08:1.21:1.00:1.04	8.20E+06	6.84E+07	0.12:1.00	17820	PREDICTED: 60S ribosomal protein L27a-3 [Vitis vinifera]
1163	22123	gi|225435203	60	8	1	1		2.14E+06	0	7.50E+05	2.66E+07	2.32E+07	2.36E+07	0.09:0:0.03:1.14:1.00:1.02	9.65E+05	2.45E+07	0.04:1.00	15652	PREDICTED: 40S ribosomal protein S23-like [Vitis vinifera]
1276	22379	gi|225432586	60	7	1	1		2.32E+06	0	0	1.74E+07	1.43E+07	1.37E+07	0.16:0:0:1.21:1.00:0.96	7.72E+05	1.51E+07	0.05:1.00	16449	PREDICTED: 40S ribosomal protein S16 [Vitis vinifera]

1491	3536	gi|225463990	60	3	1	1		6.38E+08	5.92E+08	5.77E+08	3.64E+07	2.64E+08	3.96E+08	2.42:2.24:2.19:0.14:1.00:1.50	6.02E+08	2.32E+08	2.59:1.00	29642	PREDICTED: transcription repressor MYB6 [Vitis vinifera]
1313	3435	gi|731408416	60	4	2	1		6.00E+07	6.48E+07	6.08E+07	2.37E+07	4.00E+07	4.16E+07	1.50:1.62:1.52:0.59:1.00:1.04	6.19E+07	3.51E+07	1.76:1.00	53964	PREDICTED: serine carboxypeptidase-like 13 [Vitis vinifera]
977	1803	gi|225429772	60	1	1	1		3.87E+07	4.20E+07	3.90E+07	1.58E+07	1.93E+07	1.98E+07	2.01:2.18:2.02:0.82:1.00:1.03	3.99E+07	1.83E+07	2.18:1.00	104811	PREDICTED: plasma membrane ATPase 4 [Vitis vinifera]
1422	2651	gi|566256321	59.67	7	1	1		1.27E+09	1.38E+09	1.35E+09	4.98E+08	4.85E+08	4.41E+08	2.61:2.84:2.77:1.03:1.00:0.91	1.33E+09	4.75E+08	2.80:1.00	12448	glycine-rich RNA-binding family protein [Populus trichocarpa]
574	22062	gi|225432758	58.61	6	2	1	Carbamidomethylation	0	0	-	1.71E+07	1.54E+07	1.49E+07	0:0:-:1.11:1.00:0.97	0	1.58E+07	00:01.0	29774	PREDICTED: 40S ribosomal protein S3a-1 [Vitis vinifera]
1492	3734	gi|359494267	58.55	3	1	1		5.90E+07	6.05E+07	5.66E+07	3.47E+07	2.64E+07	2.04E+07	2.23:2.29:2.14:1.31:1.00:0.77	5.87E+07	2.72E+07	2.16:1.00	25766	PREDICTED: probable glutathione S-transferase [Vitis vinifera]
466	774	gi|671743459	58.4	70	5	5	Carbamidomethylation; Carbamidomethylation	1.84E+08	1.42E+08	1.62E+08	5.08E+07	2.93E+07	2.97E+07	6.29:4.83:5.53:1.74:1.00:1.01	1.63E+08	3.48E+07	4.67:1.00	9038	photosystem I subunit VII (chloroplast) [Camellia reticulata]
1426	2944	gi|566161648	56.77	5	1	1		3.17E+07	2.09E+07	2.49E+07	1.96E+07	9.14E+06	1.74E+07	3.47:2.28:2.73:2.15:1.00:1.90	2.58E+07	1.54E+07	1.68:1.00	17801	glycine cleavage system protein H precursor [Populus trichocarpa]
764	1359	gi|225429209	56.01	5	3	2		1.13E+08	1.14E+08	1.16E+08	7.85E+07	7.22E+07	7.64E+07	1.56:1.58:1.60:1.09:1.00:1.06	1.14E+08	7.57E+07	1.51:1.00	53142	PREDICTED: serine carboxypeptidase-like 16 [Vitis vinifera]
1042	3388	gi|731398611	55.13	2	1	1		1.56E+07	9.32E+06	1.25E+07	3.16E+07	2.14E+07	2.07E+07	0.73:0.44:0.58:1.48:1.00:0.97	1.25E+07	2.46E+07	0.51:1.00	43372	PREDICTED: proliferation-associated protein 2G4 [Vitis vinifera]
759	1066	gi|566158065	53.07	3	1	1		5.29E+07	5.84E+07	3.50E+07	3.51E+07	1.41E+07	2.20E+07	3.74:4.13:2.48:2.48:1.00:1.56	4.88E+07	2.50E+07	1.95:1.00	37108	arginase family protein [Populus trichocarpa]
1416	2440	gi|566187811	51.49	5	1	1	Carbamidomethylation	8.08E+06	1.01E+07	8.47E+06	3.15E+06	4.81E+06	4.76E+06	1.68:2.10:1.76:0.65:1.00:0.99	8.88E+06	4.24E+06	2.10:1.00	16218	hypothetical protein POPTR_0009s13460g [Populus trichocarpa]

6	28	gi|359486799	50.79	8	4	1		1.47E+07	9.54E+06	1.12E+07	3.25E+07	1.45E+07	1.99E+07	1.01:0.66:0.77:2.25:1.00:1.37	1.18E+07	2.23E+07	0.53:1.00	71171	PREDICTED: heat shock cognate 70 kDa protein 2 [Vitis vinifera]
358	589	gi|224084209	50.66	2	1	1		1.66E+08	1.47E+08	1.72E+08	5.30E+07	5.64E+07	5.31E+07	2.95:2.61:3.05:0.94:1.00:0.94	1.62E+08	5.42E+07	2.99:1.00	35142	O2 evolving complex 33kD family protein [Populus trichocarpa]
1483	3258	gi|566255736	50.05	5	1	1		1.02E+08	1.11E+08	1.03E+08	8.17E+07	2.85E+07	7.41E+07	3.57:3.91:3.63:2.87:1.00:2.60	1.05E+08	6.14E+07	1.72:1.00	15873	hypothetical protein POPTR_0483s00200g partial [Populus trichocarpa]
458	653	gi|224104269	48.28	17	3	1		3.28E+07	3.56E+07	3.36E+07	7.58E+07	6.44E+07	6.64E+07	0.51:0.55:0.52:1.18:1.00:1.03	3.40E+07	6.89E+07	0.49:1.00	22228	peptidyl-prolyl cis-trans isomerase family protein [Populus trichocarpa]
1509	7727	gi|225453275	47.49	4	1	1	Carbamidomethylation	5.63E+07	7.51E+07	6.58E+07	2.96E+07	3.65E+07	3.88E+07	1.54:2.06:1.81:0.81:1.00:1.06	6.57E+07	3.49E+07	1.88:1.00	14633	PREDICTED: 20 kDa chaperonin chloroplastic [Vitis vinifera]
465	22032	gi|225451279	46.72	8	2	1		1.86E+06	5.91E+05	1.32E+06	1.60E+07	1.25E+07	1.38E+07	0.15:0.05:0.11:1.28:1.00:1.10	1.26E+06	1.41E+07	0.09:1.00	29903	PREDICTED: 40S ribosomal protein S3a-2-like [Vitis vinifera]
960	14659	gi|566202763	46.53	7	1	1		6.03E+06	6.25E+06	6.56E+06	2.78E+07	2.31E+07	2.57E+07	0.26:0.27:0.28:1.20:1.00:1.11	6.28E+06	2.55E+07	0.25:1.00	24245	60S ribosomal protein L15 [Populus trichocarpa]
857	22042	gi|225456914	44.51	7	1	1		4.84E+06	3.83E+06	4.32E+06	6.21E+07	4.94E+07	3.85E+07	0.10:0.08:0.09:1.26:1.00:0.78	4.33E+06	5.00E+07	0.09:1.00	16350	PREDICTED: 60S ribosomal protein L27a-3 [Vitis vinifera]
557	1567	gi|731395934	44.5	2	1	1		2.06E+07	2.03E+07	1.73E+07	5.11E+07	4.49E+07	4.79E+07	0.46:0.45:0.39:1.14:1.00:1.07	1.94E+07	4.80E+07	0.40:1.00	58724	PREDICTED: putative proline--tRNA ligase C19C7.06 [Vitis vinifera]
456	577	gi|224077927	43.56	3	1	1		1.22E+08	1.46E+08	1.51E+08	7.66E+07	8.54E+07	1.02E+08	1.43:1.71:1.77:0.90:1.00:1.20	1.40E+08	8.80E+07	1.59:1.00	42796	latex plastidic aldolase-like family protein [Populus trichocarpa]
134	485	gi|731393844	43.35	3	3	2		1.83E+07	1.53E+07	1.46E+07	3.13E+07	2.81E+07	2.50E+07	0.65:0.54:0.52:1.11:1.00:0.89	1.61E+07	2.81E+07	0.57:1.00	101855	PREDICTED: protein argonaute 4 [Vitis vinifera]
1245	2676	gi|566175322	43.3	3	1	1		1.46E+07	2.05E+07	1.68E+07	1.09E+07	8.66E+06	7.42E+06	1.69:2.36:1.94:1.25:1.00:0.86	1.73E+07	8.74E+06	1.98:1.00	35273	aldo/keto reductase family protein [Populus trichocarpa]
1461	22329	gi|566203933	39.99	10	1	1		0	0	0	1.20E+07	9.57E+06	8.84E+06	0:0:0:1.26:1.00:0.92	0	1.01E+07	00:01.0	13893	60S ribosomal protein L22-1 [Populus trichocarpa]

858	22074	gi|731410160	38.1	2	1	1		1.01E+07	9.03E+06	9.46E+06	6.19E+06	6.02E+06	6.09E+06	1.69:1.50:1.57:1.03:1.00:1.01	9.54E+06	6.10E+06	1.56:1.00	44936	PREDICTED: transcription initiation factor TFIID subunit 15b isoform X2 [Vitis vinifera]
1160	3488	gi|225459318	38.08	3	1	1		8.47E+06	9.94E+06	9.26E+06	1.72E+07	1.66E+07	1.54E+07	0.51:0.60:0.56:1.03:1.00:0.93	9.22E+06	1.64E+07	0.56:1.00	35188	PREDICTED: annexin D1 [Vitis vinifera]
304	559	gi|552540866	36.63	4	1	1		2.97E+06	3.45E+06	2.81E+06	7.40E+06	5.20E+06	6.11E+06	0.57:0.66:0.54:1.42:1.00:1.17	3.08E+06	6.24E+06	0.49:1.00	35220	cytochrome f (chloroplast) [Camellia danzaiensis]
285	437	gi|224053300	36.6	5	1	1		8.00E+06	6.48E+06	6.37E+06	5.25E+07	4.02E+07	4.42E+07	0.20:0.16:0.16:1.30:1.00:1.10	6.95E+06	4.56E+07	0.15:1.00	17702	ubiquitin/ribosomal protein 27a [Populus trichocarpa]
1201	3338	gi|731436245	36.44	1	1	1		1.86E+08	1.54E+08	1.73E+08	3.73E+07	7.38E+07	5.91E+07	2.52:2.08:2.35:0.50:1.00:0.80	1.71E+08	5.67E+07	3.02:1.00	66120	PREDICTED: putative laccase-9 [Vitis vinifera]
1470	2753	gi|225447745	35.89	8	1	1		4.40E+07	4.65E+07	4.19E+07	1.50E+06	3.08E+06	2.97E+06	14.28:15.09:13.62:0.49:1.00:0.96	4.41E+07	2.46E+06	17.95:1.00	27216	PREDICTED: chlorophyll a-b binding protein CP24 10A chloroplastic [Vitis vinifera]
1382	6409	gi|225427973	35.24	3	1	1		7.44E+07	9.05E+07	1.03E+08	7.54E+07	5.96E+07	2.53E+07	1.25:1.52:1.73:1.26:1.00:0.42	8.93E+07	5.34E+07	1.67:1.00	19853	PREDICTED: glycine-rich protein 2-like [Vitis vinifera]
341	812	gi|224064707	34.96	2	2	2		4.85E+07	3.51E+07	3.64E+07	2.44E+07	2.56E+07	2.26E+07	1.90:1.37:1.42:0.95:1.00:0.89	4.00E+07	2.42E+07	1.65:1.00	58836	mitochondrial aldehyde dehydrogenase family protein [Populus trichocarpa]
278	455	gi|359483839	34.48	33	6	1	Carbamidomethylation	1.99E+07	1.11E+07	1.08E+07	0	0	0	1.00:0.56:0.55:0:0:0	1.39E+07	0	1.00:0	27890	PREDICTED: chlorophyll a-b binding protein of LHCII type 1 [Vitis vinifera]
1220	1694	gi|731416683	34.43	14	1	1	Carbamidomethylation	2.04E+06	1.24E+06	1.36E+06	8.17E+06	5.28E+06	5.00E+06	0.39:0.24:0.26:1.55:1.00:0.95	1.55E+06	6.15E+06	0.25:1.00	15282	PREDICTED: superoxide dismutase [Cu-Zn] isoform X2 [Vitis vinifera]
1447	4160	gi|566212112	34.04	1	1	1	Carbamidomethylation	1.31E+07	1.24E+07	1.52E+07	6.21E+06	5.52E+06	5.14E+06	2.37:2.24:2.76:1.13:1.00:0.93	1.36E+07	5.62E+06	2.41:1.00	67792	hypothetical protein POPTR_0017s08080g [Populus trichocarpa]
1284	2062	gi|359497056	33.61	3	1	1		3.22E+07	3.86E+07	3.90E+07	6.04E+07	5.20E+07	5.54E+07	0.62:0.74:0.75:1.16:1.00:1.06	3.66E+07	5.59E+07	0.65:1.00	35941	PREDICTED: peroxidase 10-like [Vitis vinifera]

673	869	gi|225449262	32.62	11	2	1		4.23E+07	4.77E+07	4.31E+07	2.08E+07	1.84E+07	2.22E+07	2.30:2.59:2.34:1.13:1.00:1.21	4.44E+07	2.05E+07	2.17:1.00	18173	PREDICTED: 18.1 kDa class I heat shock protein [Vitis vinifera]
1270	22200	gi|224092348	32.22	11	1	1	Carbamidomethylation	0	0	0	1.45E+07	9.69E+06	9.17E+06	0:0:0:1.49:1.00:0.95	0	1.11E+07	00:01.0	10699	hypothetical protein POPTR_0006s25990g [Populus trichocarpa]
165	293	gi|225468576	32.1	2	2	1		2.08E+07	-	1.81E+07	4.73E+07	4.24E+07	3.88E+07	0.49:-:0.43:1.12:1.00:0.92	1.95E+07	4.28E+07	0.45:1.00	98187	PREDICTED: aconitate hydratase 1 [Vitis vinifera]
1393	2697	gi|224098816	31.19	3	1	1		1.78E+06	1.14E+06	3.44E+05	5.39E+06	4.81E+06	3.23E+06	0.37:0.24:0.07:1.12:1.00:0.67	1.09E+06	4.48E+06	0.24:1.00	45803	latex abundant family protein [Populus trichocarpa]
310	532	gi|671743315	30.38	5	3	3		7.83E+07	9.53E+07	9.86E+07	4.83E+07	3.32E+07	4.63E+07	2.36:2.87:2.97:1.45:1.00:1.39	9.08E+07	4.26E+07	2.13:1.00	51822	photosystem II CP43 chlorophyll apoprotein (chloroplast) [Camellia pubicosta]
704	1197	gi|566170863	30.37	10	1	1		1.76E+07	1.77E+07	2.00E+07	1.58E+08	1.31E+08	1.50E+08	0.13:0.13:0.15:1.20:1.00:1.15	1.84E+07	1.47E+08	0.13:1.00	11409	histone H4 family protein [Populus trichocarpa]
419	3070	gi|731430736	30.18	11	3	2	Carbamidomethylation	2.64E+06	3.70E+06	1.35E+06	1.76E+07	1.42E+07	1.29E+07	0.19:0.26:0.09:1.23:1.00:0.91	2.42E+06	1.49E+07	0.16:1.00	44590	PREDICTED: 60S ribosomal protein L3 [Vitis vinifera]
1212	14747	gi|225469850	30	4	1	1		0	0	2.15E+06	1.57E+07	8.50E+06	1.56E+06	0:0:0.25:1.84:1.00:0.18	7.17E+05	8.57E+06	0.08:1.00	39160	PREDICTED: uncharacterized protein DDB_G0288133 [Vitis vinifera]
300	866	gi|225437428	29.6	3	2	1		4.40E+07	1.29E+07	4.01E+07	1.10E+08	1.13E+08	8.88E+07	0.39:0.11:0.35:0.97:1.00:0.78	3.79E+07	1.04E+08	0.36:1.00	92483	PREDICTED: sucrose synthase 2 [Vitis vinifera]
794	1140	gi|731421236	29.45	2	1	1		3.89E+07	3.84E+07	3.92E+07	3.06E+07	1.79E+07	2.17E+07	2.17:2.14:2.18:1.70:1.00:1.21	3.89E+07	2.34E+07	1.66:1.00	37446	PREDICTED: arginase 1 mitochondrial [Vitis vinifera]
1048	22044	gi|224104103	29.3	7	1	1		0	0	0	5.64E+06	4.14E+06	6.24E+06	0:0:0:1.36:1.00:1.51	0	5.34E+06	00:01.0	24868	40S ribosomal protein S8 [Populus trichocarpa]
788	22030	gi|225462285	29.08	2	1	1		6.64E+06	4.24E+06	4.88E+06	1.28E+07	1.13E+07	6.27E+06	0.59:0.38:0.43:1.13:1.00:0.56	5.25E+06	1.01E+07	0.52:1.00	52600	PREDICTED: inosine-5'-monophosphate dehydrogenase [Vitis vinifera]
254	1246	gi|359492254	28.99	2	1	1		2.31E+07	2.09E+07	2.75E+07	1.64E+07	1.27E+07	9.80E+06	1.82:1.64:2.16:1.29:1.00:0.77	2.38E+07	1.29E+07	1.84:1.00	40876	PREDICTED: ferredoxin--NADP reductase leaf-type isozyme chloroplastic [Vitis vinifera]

808	1973	gi|224066545	28.91	11	2	1		1.03E+07	1.04E+07	1.03E+07	6.15E+06	4.46E+06	0	2.31:2.34:2.31:1.38:1.00:0	1.03E+07	3.54E+06	2.92:1.00	15325	40S ribosomal protein S12-1 [Populus trichocarpa]
1021	1861	gi|566162704	28.08	8	1	1		1.65E+07	1.13E+07	1.28E+07	3.70E+05	2.50E+06	2.24E+06	6.61:4.50:5.13:0.15:1.00:0.89	1.35E+07	1.87E+06	7.24:1.00	18629	hypothetical protein POPTR_0003s14870g [Populus trichocarpa]
1074	2016	gi|225429975	28.07	2	1	1		4.72E+07	4.67E+07	3.86E+07	6.83E+07	7.64E+07	7.59E+07	0.62:0.61:0.51:0.89:1.00:0.99	4.42E+07	7.35E+07	0.60:1.00	39098	PREDICTED: bark storage protein A [Vitis vinifera]
1004	22157	gi|224124892	27.4	6	1	1		2.61E+07	2.53E+07	2.54E+07	4.29E+07	3.45E+07	4.78E+07	0.76:0.73:0.74:1.24:1.00:1.38	2.56E+07	4.18E+07	0.61:1.00	22641	rhicadhesin receptor family protein [Populus trichocarpa]
1003	22131	gi|225428011	26.92	2	1	1		2.05E+06	0	0	3.98E+06	2.62E+06	2.33E+06	0.78:0:0:1.52:1.00:0.89	6.82E+05	2.98E+06	0.23:1.00	61287	PREDICTED: synaptotagmin-2 [Vitis vinifera]
1486	3357	gi|225424750	26.88	7	1	1	Carbamidomethylation	1.46E+07	1.88E+07	1.80E+07	6.99E+06	7.13E+06	7.86E+06	2.05:2.64:2.52:0.98:1.00:1.10	1.71E+07	7.33E+06	2.34:1.00	11841	PREDICTED: gibberellin-regulated protein 11 [Vitis vinifera]
802	1500	gi|566165056	26.48	3	1	1		2.28E+07	1.44E+07	1.40E+07	4.83E+07	3.86E+07	3.11E+07	0.59:0.37:0.36:1.25:1.00:0.81	1.71E+07	3.93E+07	0.43:1.00	36365	hypothetical protein POPTR_0004s05340g [Populus trichocarpa]
260	381	gi|225457971	26.01	4	2	1	Carbamidomethylation	8.40E+07	1.37E+08	1.33E+08	5.02E+07	6.07E+07	7.25E+07	1.38:2.26:2.20:0.83:1.00:1.19	1.18E+08	6.11E+07	1.93:1.00	40415	PREDICTED: flavanone 3-dioxygenase [Vitis vinifera]
547	1249	gi|566197644	25.98	3	1	1		1.51E+07	1.11E+07	1.15E+07	8.50E+06	7.51E+06	7.20E+06	2.01:1.47:1.54:1.13:1.00:0.96	1.22E+07	7.64E+06	1.60:1.00	26318	hypothetical protein POPTR_0012s09540g [Populus trichocarpa]
1538	30003	gi|224064488	25.35	3	1	1		0	0	0	6.05E+06	5.22E+06	5.92E+06	0:0:0:1.16:1.00:1.13	0	5.73E+06	00:01.0	38979	DNAJ heat shock family protein [Populus trichocarpa]
1456	7857	gi|566197051	25.11	1	1	1		1.21E+08	1.50E+08	1.60E+08	6.52E+07	7.88E+07	7.94E+07	1.54:1.90:2.03:0.83:1.00:1.01	1.44E+08	7.44E+07	1.93:1.00	92823	hypothetical protein POPTR_0012s06300g [Populus trichocarpa]
683	1434	gi|359480225	25.01	8	3	1	Carbamidomethylation	2.33E+07	2.61E+07	2.29E+07	1.20E+07	1.58E+07	1.42E+07	1.47:1.65:1.45:0.76:1.00:0.89	2.41E+07	1.40E+07	1.72:1.00	27397	PREDICTED: peptidyl-prolyl cis-trans isomerase [Vitis vinifera]
432	1514	gi|359475502	24.51	2	1	1	Carbamidomethylation	1.79E+07	1.65E+07	1.92E+07	6.38E+06	1.34E+07	8.13E+06	1.33:1.23:1.43:0.48:1.00:0.61	1.78E+07	9.30E+06	1.92:1.00	37652	PREDICTED: malate dehydrogenase glyoxysomal [Vitis vinifera]

1285	2067	gi|225425513	24.26	4	1	1		2.08E+07	1.23E+07	2.19E+07	1.14E+07	1.21E+07	9.12E+06	1.71:1.01:1.80:0.94:1.00:0.75	1.83E+07	1.09E+07	1.68:1.00	15254	PREDICTED: uncharacterized protein At4g28440 [Vitis vinifera]
1343	22406	gi|225464489	23.8	9	1	1		0	0	0	2.90E+06	1.89E+06	2.06E+06	0:0:0:1.53:1.00:1.09	0	2.29E+06	00:01.0	12497	PREDICTED: 60S ribosomal protein L36-3 [Vitis vinifera]
397	665	gi|225423961	23.45	1	1	1		1.01E+07	5.70E+06	4.75E+06	1.29E+07	1.08E+07	1.15E+07	0.94:0.53:0.44:1.20:1.00:1.07	6.86E+06	1.17E+07	0.58:1.00	103148	PREDICTED: alpha-xylosidase 1 [Vitis vinifera]
805	1643	gi|566171771	23.29	2	1	1		6.22E+06	4.40E+06	2.73E+06	1.42E+07	1.28E+07	1.13E+07	0.49:0.35:0.21:1.11:1.00:0.88	4.45E+06	1.28E+07	0.35:1.00	53299	hypothetical protein POPTR_0005s16590g partial [Populus trichocarpa]
669	22071	gi|224090212	22.83	6	2	1		2.23E+07	0	0	7.80E+07	8.56E+07	8.87E+07	0.26:0:0:0.91:1.00:1.04	7.43E+06	8.41E+07	0.09:1.00	29609	40S ribosomal protein S2 [Populus trichocarpa]
892	1552	gi|224095668	22.67	5	1	1		3.71E+07	2.60E+07	2.20E+07	5.32E+07	4.52E+07	3.60E+07	0.82:0.58:0.49:1.18:1.00:0.80	2.84E+07	4.48E+07	0.63:1.00	35550	hypothetical protein POPTR_0007s01850g [Populus trichocarpa]
948	2264	gi|225434133	22.66	3	1	1		1.03E+07	9.88E+06	1.08E+07	1.93E+07	1.54E+07	1.41E+07	0.67:0.64:0.70:1.26:1.00:0.92	1.04E+07	1.63E+07	0.64:1.00	40613	PREDICTED: cysteine synthase chloroplastic/chromoplastic [Vitis vinifera]
760	1097	gi|225459564	22.44	7	1	1		7.79E+06	7.65E+07	9.24E+07	7.10E+06	2.84E+06	2.15E+06	2.74:26.88:32.48:2.50:1.00:0.76	5.89E+07	4.03E+06	14.60:1.00	28575	PREDICTED: photosystem II 22 kDa protein chloroplastic [Vitis vinifera]
1462	22346	gi|224066487	21.92	5	1	1		3.25E+06	4.18E+06	3.25E+06	5.87E+07	4.98E+07	5.48E+07	0.07:0.08:0.07:1.18:1.00:1.10	3.56E+06	5.44E+07	0.07:1.00	17636	hypothetical protein POPTR_0002s05220g [Populus trichocarpa]
357	522	gi|566184073	21.61	8	2	1	Carbamidomethylation	4.12E+07	4.57E+07	0	7.63E+06	1.37E+07	2.05E+07	3.00:3.33:0:0.56:1.00:1.49	3.47E+07	1.39E+07	2.49:1.00	22518	photosystem I 20kD family protein [Populus trichocarpa]
390	7356	gi|359497883	21.45	26	5	1		3.16E+06	2.53E+06	5.20E+05	2.33E+07	3.28E+07	1.63E+07	0.10:0.08:0.02:0.71:1.00:0.50	2.07E+06	2.41E+07	0.09:1.00	15406	PREDICTED: histone H3.3 [Vitis vinifera]
1431	3106	gi|224109620	21.3	4	1	1		3.48E+07	4.14E+07	3.76E+07	1.70E+07	2.34E+07	3.08E+07	1.49:1.77:1.61:0.73:1.00:1.32	3.79E+07	2.37E+07	1.60:1.00	24252	chalcone isomerase family protein [Populus trichocarpa]
694	3014	gi|225443760	20.76	8	1	1		0	-	0	8.05E+06	7.28E+06	6.81E+06	0:-:0:1.11:1.00:0.94	0	7.38E+06	00:01.0	20875	PREDICTED: 60S ribosomal protein L11-1-like [Vitis vinifera]

819	22248	gi|566151109	20.65	4	2	1		0	0	0	1.66E+07	1.40E+07	1.11E+07	0:0:0:1.19:1.00:0.79	0	1.39E+07	00:01.0	37403	hypothetical protein POPTR_0001s26400g [Populus trichocarpa]
1487	3368	gi|731438465	20.56	3	1	1		1.28E+07	1.35E+07	1.02E+07	6.17E+06	8.94E+06	8.41E+06	1.43:1.51:1.14:0.69:1.00:0.94	1.22E+07	7.84E+06	1.55:1.00	26979	PREDICTED: 3-isopropylmalate dehydratase small subunit 3 [Vitis vinifera]
308	404	gi|671743230	20.34	3	2	2	Carbamidomethylation	7.00E+07	4.26E+07	7.26E+07	5.57E+05	1.01E+06	1.35E+06	69.47:42.25:72.08:0.55:1.00:1.34	6.07E+07	9.71E+05	62.49:1.00	82370	photosystem I P700 apoprotein A2 (chloroplast) [Camellia petelotii]
463	1862	gi|731388721	20.26	10	1	1		3.00E+07	3.23E+07	2.85E+07	5.04E+07	4.46E+07	4.22E+07	0.67:0.72:0.64:1.13:1.00:0.95	3.03E+07	4.57E+07	0.66:1.00	18965	PREDICTED: translationally-controlled tumor protein homolog [Vitis vinifera]

Table S7 The identified differentially expressed proteins between CK2 and HY2

Protein Gr	Protein ID	Accession	Significance (-10lgP)	Coverage (%)	#Peptides	#Unique	Sample 7 Area	Sample 8 Area	Sample 9 Area	Sample 10 Area	Sample 11 Area	Sample 12 Area	Sample Profile (Ratio)	CK2 Area	H2 Area	Group Profile (Ratio)	Avg. Mass	Description
1304	1895	gi|731428049	101.58	10	3	3	2.26E+07	1.68E+07	1.70E+07	6.75E+07	5.05E+07	3.58E+07	0.63:0.47:0.47:1.88:1.41:1.00	1.88E+07	5.13E+07	0.37:1.00	47334	PREDICTED: delta-aminolevulinic acid dehydratase chloroplastic [Vitis vinifera]
383	292	gi|542688125	100.7	11	6	6	3.67E+08	3.52E+08	3.28E+08	1.56E+08	1.36E+08	1.29E+08	2.86:2.74:2.55:1.21:1.06:1.00	3.49E+08	1.40E+08	2.49:1.00	56099	photosystem II p680 chlorophyll A apoprotein CP-47 (chloroplast) [Camellia taliensis]
1021	900	gi|566207041	90.61	9	3	2	3.54E+07	3.09E+07	2.52E+07	1.01E+08	6.78E+07	5.60E+07	0.63:0.55:0.45:1.80:1.21:1.00	3.05E+07	7.48E+07	0.41:1.00	53652	glutamate 1-semialdehyde aminotransferase family protein [Populus trichocarpa]
1398	970	gi|731403303	86.64	19	2	1	7.28E+07	5.43E+07	4.60E+07	1.80E+08	1.49E+08	1.01E+08	0.72:0.54:0.46:1.79:1.47:1.00	5.77E+07	1.43E+08	0.40:1.00	15026	PREDICTED: 40S ribosomal protein S12 [Vitis vinifera]
265	218	gi|359478916	80.62	25	6	2	1.03E+08	1.14E+08	1.13E+08	2.51E+08	1.98E+08	1.64E+08	0.63:0.70:0.69:1.53:1.21:1.00	1.10E+08	2.04E+08	0.54:1.00	48674	PREDICTED: ribulose bisphosphate carboxylase/oxygenase activase chloroplastic isoform X2 [Vitis vinifera]
526	600	gi|552541026	77.25	3	3	3	2.29E+08	2.06E+08	1.50E+08	3.69E+07	5.53E+07	8.25E+07	2.78:2.50:1.81:0.45:0.67:1.00	1.95E+08	6.09E+07	3.21:1.00	83146	photosystem I P700 chlorophyll A apoprotein A1 (chloroplast) [Camellia yunnanensis]
472	404	gi|671743230	76.9	5	3	3	1.28E+08	1.12E+08	9.70E+07	5.84E+07	5.80E+07	4.96E+07	2.59:2.26:1.95:1.18:1.17:1.00	1.13E+08	5.54E+07	2.03:1.00	82370	photosystem I P700 apoprotein A2 (chloroplast) [Camellia petelotii]
10	2	gi|669100005	75.46	33	14	14	3.41E+09	2.97E+09	2.74E+09	1.63E+09	1.61E+09	1.50E+09	2.28:1.98:1.83:1.09:1.07:1.00	3.04E+09	1.58E+09	1.93:1.00	52635	ribulose bisphosphate carboxylase large chain (chloroplast) [Camellia crapnelliana]
174	527	gi|731424802	75.2	2	1	1	3.10E+07	2.33E+07	1.61E+07	8.84E+07	5.12E+07	3.30E+07	0.94:0.71:0.49:2.68:1.55:1.00	2.35E+07	5.75E+07	0.41:1.00	101244	PREDICTED: heat shock protein 101 isoform X1 [Vitis vinifera]
1143	45542	gi|731394579	74.86	7	1	1	1.92E+07	1.67E+07	1.49E+07	5.12E+07	4.75E+07	3.71E+07	0.52:0.45:0.40:1.38:1.28:1.00	1.69E+07	4.53E+07	0.37:1.00	24605	PREDICTED: 60S ribosomal protein L10a-1-like [Vitis vinifera]
215	482	gi|731419756	72.51	9	3	2	3.22E+07	2.59E+07	3.92E+07	9.27E+07	6.24E+07	4.20E+07	0.77:0.62:0.93:2.21:1.49:1.00	3.24E+07	6.57E+07	0.49:1.00	52962	PREDICTED: UDP-glucose 6-dehydrogenase 1 [Vitis vinifera]
725	836	gi|731401656	70.96	14	2	2	4.27E+07	3.21E+07	2.85E+07	8.71E+07	6.76E+07	5.43E+07	0.79:0.59:0.52:1.60:1.24:1.00	3.45E+07	6.97E+07	0.49:1.00	25978	PREDICTED: proteasome subunit alpha type-5 [Vitis vinifera]

254	223	gi|224109060	67.4	11	3	1	1.97E+07	1.36E+07	1.01E+07	8.56E+07	3.98E+07	2.70E+07	0.73:0.50:<br>0.37:3.16:<br>1.47:1.00	1.45E+07	5.08E+07	0.28:1.00	50212	PHOSPHOGLYCERAT E KINASE 1 family protein [Populus trichocarpa]
805	1639	gi|566242200	66.35	9	1	1	7.46E+08	6.43E+08	5.79E+08	2.09E+09	1.25E+09	1.04E+09	0.71:0.62:<br>0.55:2.01:<br>1.20:1.00	6.56E+08	1.38E+09	0.47:1.00	19093	hypothetical protein POPTR_0019s12370g partial [Populus trichocarpa]
124	477	gi|224065861	65.3	44	6	2	2.05E+08	1.36E+07	1.42E+07	5.93E+08	4.12E+08	3.52E+08	0.58:0.04:<br>0.04:1.68:<br>1.17:1.00	8.57E+07	4.52E+08	0.19:1.00	18243	Peptidyl-prolyl cis-trans isomerase family protein [Populus trichocarpa]
940	1517	gi|359494079	64.86	2	1	1	1.63E+07	1.38E+07	1.22E+07	3.51E+07	3.07E+07	1.89E+07	0.86:0.73:<br>0.65:1.86:<br>1.63:1.00	1.41E+07	2.82E+07	0.50:1.00	65102	PREDICTED: LOW QUALITY PROTEIN: 65-kDa microtubule-associated protein 1-like [Vitis vinifera]
2406	2285	gi|731426021	63.53	30	2	1	1.54E+07	1.10E+07	7.96E+06	5.79E+07	3.61E+07	2.46E+07	0.63:0.45:<br>0.32:2.35:<br>1.46:1.00	1.14E+07	3.95E+07	0.29:1.00	12464	PREDICTED: protein SPIRAL1-like 1 isoform X2 [Vitis vinifera]
1827	2316	gi|526118093	63.02	8	1	1	2.33E+07	2.43E+07	2.01E+07	6.33E+07	4.27E+07	3.25E+07	0.72:0.75:<br>0.62:1.95:<br>1.31:1.00	2.25E+07	4.62E+07	0.49:1.00	22511	type II peroxiredoxin E [Vitis vinifera]
2407	2582	gi|731407226	62.96	5	1	1	8.98E+06	8.39E+06	5.45E+06	4.03E+07	2.11E+07	1.79E+07	0.50:0.47:<br>0.30:2.25:<br>1.18:1.00	7.61E+06	2.65E+07	0.29:1.00	37934	PREDICTED: 2-alkenal reductase (NADP(+)-dependent) [Vitis vinifera]
605	697	gi|225426753	62.88	4	2	1	2.74E+07	1.79E+07	1.53E+07	7.33E+07	4.65E+07	4.18E+07	0.65:0.43:<br>0.37:1.75:<br>1.11:1.00	2.02E+07	5.39E+07	0.37:1.00	81719	PREDICTED: primary amine oxidase [Vitis vinifera]
2600	54896	gi|224070184	62.45	11	1	1	7.12E+06	5.65E+06	4.38E+06	3.04E+07	1.60E+07	1.20E+07	0.59:0.47:<br>0.37:2.54:<br>1.34:1.00	5.72E+06	1.95E+07	0.29:1.00	18056	deoxyuridine 5'-triphosphate nucleotidohydrolase family protein [Populus trichocarpa]
714	2170	gi|225455784	62.44	3	1	1	4.33E+07	4.73E+07	4.32E+07	1.73E+08	1.02E+08	7.53E+07	0.57:0.63:<br>0.57:2.30:<br>1.35:1.00	4.46E+07	1.17E+08	0.38:1.00	50558	PREDICTED: enolase 1 chloroplastic [Vitis vinifera]
1743	37684	gi|225451247	62.16	11	1	1	2.50E+07	1.75E+07	1.69E+07	1.13E+08	5.15E+07	6.64E+07	0.38:0.26:<br>0.25:1.70:<br>0.78:1.00	1.98E+07	7.70E+07	0.26:1.00	15707	PREDICTED: 40S ribosomal protein S24-1 [Vitis vinifera]
1406	1863	gi|225448367	62.08	4	1	1	8.04E+07	6.96E+07	6.21E+07	1.99E+08	1.27E+08	1.06E+08	0.76:0.66:<br>0.58:1.88:<br>1.20:1.00	7.07E+07	1.44E+08	0.49:1.00	34281	PREDICTED: 60S acidic ribosomal protein P0 [Vitis vinifera]
2462	45857	gi|225448536	60.22	4	1	1	1.35E+07	1.07E+07	8.09E+06	4.02E+07	2.45E+07	1.76E+07	0.77:0.61:<br>0.46:2.29:<br>1.39:1.00	1.08E+07	2.74E+07	0.39:1.00	38803	PREDICTED: eukaryotic translation initiation factor 2 subunit alpha [Vitis vinifera]
2010	45804	gi|225449234	60.09	16	1	1	1.11E+07	1.06E+07	7.11E+06	2.70E+07	2.48E+07	2.12E+07	0.52:0.50:<br>0.34:1.27:<br>1.17:1.00	9.60E+06	2.44E+07	0.39:1.00	12856	PREDICTED: 60S ribosomal protein L35a-1 [Vitis vinifera]
939	1418	gi|359485890	60	1	1	1	1.28E+07	1.66E+07	1.71E+07	2.35E+06	6.30E+06	7.71E+06	1.66:2.15:<br>2.22:0.31:<br>0.82:1.00	1.55E+07	5.46E+06	2.84:1.00	83365	PREDICTED: beta-xylosidase/alpha-L-arabinofuranosidase 2-like [Vitis vinifera]
1732	7561	gi|225447707	60	6	1	1	1.75E+07	1.53E+07	1.21E+07	6.80E+07	3.82E+07	2.71E+07	0.64:0.57:<br>0.45:2.51:<br>1.41:1.00	1.50E+07	4.45E+07	0.34:1.00	27098	PREDICTED: uncharacterized protein LOC100263170 [Vitis vinifera]

1851	63948	gi|566205838	60	2	1	1	7.21E+06	5.55E+06	4.07E+06	2.62E+07	1.41E+07	1.12E+07	0.64:0.49:0.36:2.34:1.26:1.00	5.61E+06	1.72E+07	0.33:1.00	130678	hypothetical protein POPTR_0015s04720g [Populus trichocarpa]
2725	3095	gi|526117629	60	8	1	1	1.74E+08	2.29E+08	3.02E+08	3.87E+07	9.72E+07	1.39E+08	1.25:1.64:2.17:0.28:0.70:1.00	2.35E+08	9.17E+07	2.56:1.00	11686	lipid transfer protein precursor [Vitis vinifera]
2742	7736	gi|566167359	60	4	1	1	2.01E+07	1.95E+07	2.06E+07	4.72E+06	8.23E+06	9.77E+06	2.06:1.99:2.11:0.48:0.84:1.00	2.01E+07	7.57E+06	2.65:1.00	15952	hypothetical protein POPTR_0004s19310g [Populus trichocarpa]
596	798	gi|566167040	60	15	3	1	1.02E+08	7.22E+07	6.05E+07	2.60E+08	1.82E+08	1.37E+08	0.74:0.53:0.44:1.90:1.34:1.00	7.81E+07	1.93E+08	0.40:1.00	33502	hypothetical protein POPTR_0004s17530g [Populus trichocarpa]
2664	46648	gi|225428420	60	9	1	1	2.15E+07	1.32E+07	1.30E+07	4.24E+07	4.45E+07	3.93E+07	0.55:0.33:0.33:1.08:1.13:1.00	1.59E+07	4.21E+07	0.38:1.00	12203	PREDICTED: 40S ribosomal protein S25-2 [Vitis vinifera]
689	582	gi|225439064	59.65	8	3	3	4.52E+07	3.94E+07	2.47E+07	9.78E+07	8.24E+07	6.29E+07	0.72:0.63:0.39:1.55:1.31:1.00	3.64E+07	8.09E+07	0.45:1.00	61092	PREDICTED: 2 3-bisphosphoglycerate-independent phosphoglycerate mutase [Vitis vinifera]
1454	22432	gi|224104913	59.54	14	1	1	2.56E+07	2.98E+07	2.61E+07	8.61E+07	6.23E+07	4.41E+07	0.58:0.68:0.59:1.95:1.41:1.00	2.76E+07	6.42E+07	0.43:1.00	12354	60S ribosomal protein L30 [Populus trichocarpa]
2727	3143	gi|225443988	59.1	7	1	1	3.82E+07	4.94E+07	4.59E+07	0	1.71E+07	2.07E+07	1.85:2.39:2.22:0:0.83:1.00	4.45E+07	1.26E+07	3.53:1.00	13228	PREDICTED: photosystem I reaction center subunit psaK chloroplastic [Vitis vinifera]
299	532	gi|671743315	58	8	4	4	2.41E+08	2.18E+08	2.03E+08	8.16E+07	7.98E+07	7.44E+07	3.24:2.93:2.73:1.10:1.07:1.00	2.20E+08	7.86E+07	2.81:1.00	51822	photosystem II CP43 chlorophyll apoprotein (chloroplast) [Camellia pubicosta]
48	96	gi|225426230	57.98	9	5	1	1.10E+08	8.94E+07	8.37E+07	2.16E+08	1.69E+08	1.13E+08	0.97:0.79:0.74:1.91:1.49:1.00	9.42E+07	1.66E+08	0.57:1.00	73581	PREDICTED: luminal-binding protein 5 [Vitis vinifera]
767	1058	gi|526117441	57.27	2	1	1	1.24E+07	1.10E+07	9.89E+06	2.40E+07	1.85E+07	1.55E+07	0.80:0.71:0.64:1.55:1.19:1.00	1.11E+07	1.93E+07	0.57:1.00	101670	lipoxygenase [Vitis vinifera]
2726	3102	gi|566186111	57.18	8	1	1	1.08E+08	1.40E+08	1.34E+08	5.15E+06	1.51E+07	5.78E+07	1.87:2.41:2.31:0.09:0.26:1.00	1.27E+08	2.60E+07	4.88:1.00	12191	hypothetical protein POPTR_0009s03520g partial [Populus trichocarpa]
1569	1877	gi|224078584	57.12	5	1	1	6.90E+07	5.56E+07	4.80E+07	1.78E+08	9.05E+07	7.36E+07	0.94:0.76:0.65:2.42:1.23:1.00	5.75E+07	1.14E+08	0.50:1.00	33150	glyoxalase I homolog family protein [Populus trichocarpa]
693	1064	gi|566192956	56.77	3	1	1	2.31E+07	2.03E+07	1.75E+07	7.34E+07	4.39E+07	3.12E+07	0.74:0.65:0.56:2.35:1.41:1.00	2.03E+07	4.95E+07	0.41:1.00	44225	hypothetical protein POPTR_0011s00650g [Populus trichocarpa]
1722	2034	gi|225452270	56.05	4	1	1	3.32E+07	2.68E+07	2.41E+07	6.84E+07	5.65E+07	4.74E+07	0.70:0.57:0.51:1.45:1.19:1.00	2.80E+07	5.74E+07	0.49:1.00	34598	PREDICTED: 28 kDa ribonucleoprotein chloroplastic-like [Vitis vinifera]
90	172	gi|566196410	55.93	12	6	2	4.76E+07	3.89E+07	3.05E+07	1.50E+08	7.21E+07	5.38E+07	0.88:0.72:0.57:2.79:1.34:1.00	3.90E+07	8.46E+07	0.46:1.00	73406	BiP isoform A family protein [Populus trichocarpa]
1614	1236	gi|225460542	55.57	5	1	1	5.03E+07	4.00E+07	3.12E+07	1.22E+08	7.68E+07	5.95E+07	0.85:0.67:0.53:2.05:1.29:1.00	4.05E+07	8.61E+07	0.47:1.00	36331	PREDICTED: heterogeneous nuclear ribonucleoprotein 1 [Vitis vinifera]

764	808	gi|731376167	55.13	19	3	2	4.03E+07	3.53E+07	3.10E+07	7.25E+07	6.42E+07	4.75E+07	0.85:0.74:0.65:1.53:1.35:1.00	3.55E+07	6.14E+07	0.58:1.00	29267	PREDICTED: proteasome subunit beta type-5 [Vitis vinifera]
720	990	gi|225461156	55.07	39	2	1	1.05E+08	6.57E+07	1.30E+07	3.30E+08	1.72E+08	1.10E+08	0.96:0.60:0.12:3.01:1.57:1.00	5.58E+07	2.04E+08	0.27:1.00	13980	PREDICTED: probable histone H2A.2 [Vitis vinifera]
1689	14744	gi|225434277	54.76	4	1	1	1.83E+07	7.08E+06	7.89E+06	2.26E+07	2.74E+07	2.68E+07	0.68:0.26:0.29:0.84:1.02:1.00	1.11E+07	2.56E+07	0.43:1.00	22623	PREDICTED: CBS domain-containing protein CBSX3 mitochondrial [Vitis vinifera]
847	747	gi|224114826	53.62	12	1	1	1.79E+07	1.54E+07	1.42E+07	6.53E+07	4.18E+07	2.81E+07	0.64:0.55:0.51:2.32:1.49:1.00	1.59E+07	4.51E+07	0.35:1.00	17193	ubiquitin-conjugating enzyme family protein [Populus trichocarpa]
762	23720	gi|566254838	53.5	5	1	1	3.29E+07	2.32E+07	2.02E+07	5.88E+07	5.98E+07	5.09E+07	0.65:0.45:0.40:1.16:1.18:1.00	2.54E+07	5.65E+07	0.45:1.00	28211	hypothetical protein POPTR_0789s00200g [Populus trichocarpa]
67	33	gi|566225321	53.26	40	9	1	3.52E+08	3.40E+08	3.47E+08	6.68E+08	5.03E+08	4.27E+08	0.82:0.80:0.81:1.56:1.18:1.00	3.46E+08	5.32E+08	0.65:1.00	41712	actin family protein [Populus trichocarpa]
2440	22430	gi|224084852	53.06	15	1	1	2.45E+07	1.86E+07	1.38E+07	6.93E+07	4.39E+07	3.04E+07	0.81:0.61:0.46:2.28:1.45:1.00	1.90E+07	4.79E+07	0.40:1.00	10061	hypothetical protein POPTR_0005s16040g [Populus trichocarpa]
987	2038	gi|225457458	53.06	18	3	3	1.47E+07	8.26E+06	1.60E+07	5.16E+07	2.66E+07	1.76E+07	0.83:0.47:0.91:2.93:1.51:1.00	1.36E+07	3.20E+07	0.42:1.00	17173	PREDICTED: transcription factor BTF3 homolog 4 [Vitis vinifera]
397	3846	gi|224143423	52.43	1	1	1	1.40E+07	1.02E+07	7.58E+06	6.60E+07	1.95E+07	1.63E+07	0.85:0.63:0.46:4.04:1.20:1.00	1.06E+07	2.75E+07	0.38:1.00	105985	putative coatmer beta subunit family protein [Populus trichocarpa]
2436	22188	gi|526118000	52.15	6	1	1	8.10E+06	6.09E+06	5.15E+06	2.30E+07	1.59E+07	9.63E+06	0.84:0.63:0.53:2.39:1.65:1.00	6.45E+06	1.62E+07	0.40:1.00	22732	germin-like protein 2 precursor [Vitis vinifera]
2317	3104	gi|225439775	51.91	4	1	1	1.31E+08	1.15E+08	1.02E+08	2.65E+08	2.10E+08	1.61E+08	0.82:0.72:0.64:1.65:1.31:1.00	1.16E+08	2.12E+08	0.55:1.00	27055	PREDICTED: glutathione S-transferase L3-like [Vitis vinifera]
2542	3188	gi|566189043	50.93	4	1	1	8.66E+07	6.87E+07	4.86E+07	1.93E+08	1.54E+08	1.04E+08	0.83:0.66:0.47:1.85:1.49:1.00	6.79E+07	1.50E+08	0.45:1.00	36173	Peroxidase 6 precursor family protein [Populus trichocarpa]
38	106	gi|224094244	50.84	12	7	2	3.76E+07	3.14E+07	2.68E+07	2.43E+08	2.15E+08	4.36E+07	0.86:0.72:0.61:5.58:4.93:1.00	3.19E+07	1.67E+08	0.19:1.00	93953	elongation factor 2 family protein [Populus trichocarpa]
2644	46034	gi|731411254	50.13	5	1	1	1.72E+07	2.08E+07	1.96E+07	5.25E+06	7.07E+06	1.10E+07	1.57:1.90:1.79:0.48:0.65:1.00	1.92E+07	7.76E+06	2.47:1.00	21305	PREDICTED: 22.0 kDa class IV heat shock protein-like [Vitis vinifera]
520	629	gi|224083020	49.72	24	3	1	3.71E+07	3.08E+07	2.34E+07	1.20E+08	9.50E+07	5.54E+07	0.67:0.56:0.42:2.16:1.71:1.00	3.04E+07	9.01E+07	0.34:1.00	18138	Peptidyl-prolyl cis-trans isomerase family protein [Populus trichocarpa]
543	965	gi|566210666	49.71	5	1	1	1.10E+07	1.05E+07	7.68E+06	3.90E+07	2.21E+07	1.57E+07	0.70:0.67:0.49:2.49:1.41:1.00	9.73E+06	2.56E+07	0.38:1.00	51605	hypothetical protein POPTR_0017s01390g [Populus trichocarpa]
1318	14637	gi|566183513	49.27	6	3	2	4.53E+07	3.65E+07	2.98E+07	1.04E+08	7.87E+07	5.51E+07	0.82:0.66:0.54:1.89:1.43:1.00	3.72E+07	7.93E+07	0.47:1.00	62808	hypothetical protein POPTR_0008s11770g [Populus trichocarpa]
2179	3088	gi|566150510	49.02	6	1	1	2.00E+07	2.21E+07	2.14E+07	5.32E+07	5.29E+07	4.28E+07	0.47:0.52:0.50:1.24:1.24:1.00	2.12E+07	4.96E+07	0.43:1.00	24081	elongation factor 1B alpha-subunit 2 family protein [Populus trichocarpa]

110	111	gi|225456079	48.99	20	8	2	8.92E+07	7.13E+07	7.41E+07	1.53E+08	1.26E+08	9.39E+07	0.95:0.76:0.79:1.63:1.34:1.00	7.87E+07	1.24E+08	0.63:1.00	59180	PREDICTED: ATP synthase subunit beta mitochondrial-like [Vitis vinifera]
1683	3310	gi|671743260	48.29	4	1	1	3.01E+07	2.75E+07	2.95E+07	9.30E+06	6.70E+06	7.19E+06	4.19:3.83:4.10:1.29:0.93:1.00	2.90E+07	7.53E+06	3.85:1.00	24165	cytochrome b6 (chloroplast) [Camellia petelotii]
332	734	gi|225430619	48.07	19	3	3	1.76E+08	1.65E+08	8.91E+07	3.41E+08	2.22E+08	2.19E+08	0.80:0.75:0.41:1.56:1.01:1.00	1.43E+08	2.61E+08	0.55:1.00	22781	PREDICTED: 40S ribosomal protein S5 [Vitis vinifera]
2533	2967	gi|224112341	47.56	3	1	1	2.59E+07	2.27E+07	1.82E+07	6.22E+07	4.32E+07	2.87E+07	0.90:0.79:0.63:2.16:1.50:1.00	2.23E+07	4.47E+07	0.50:1.00	46306	latex abundant family protein [Populus trichocarpa]
1720	1958	gi|224073967	47	12	2	2	1.49E+08	1.30E+08	1.16E+08	8.36E+07	7.09E+07	7.05E+07	2.11:1.85:1.65:1.18:1.01:1.00	1.32E+08	7.50E+07	1.76:1.00	15188	photosystem I 11K family protein [Populus trichocarpa]
1262	1674	gi|225460716	46	6	2	1	3.22E+06	3.33E+06	1.76E+06	1.04E+07	5.68E+06	4.27E+06	0.75:0.78:0.41:2.44:1.33:1.00	2.77E+06	6.80E+06	0.41:1.00	49588	PREDICTED: dihydrolipoyllysine-residue acetyltransferase component 5 of pyruvate dehydrogenase complex chloroplastic [Vitis vinifera]
253	1055	gi|225431871	45.83	3	1	1	7.74E+06	3.79E+06	3.22E+06	3.83E+07	2.08E+07	1.23E+07	0.63:0.31:0.26:3.11:1.69:1.00	4.92E+06	2.38E+07	0.21:1.00	58138	PREDICTED: importin subunit alpha-1 isoform X1 [Vitis vinifera]
2077	45601	gi|566183164	45.77	19	2	1	5.54E+07	4.40E+07	3.77E+07	1.32E+08	1.01E+08	7.93E+07	0.70:0.55:0.48:1.67:1.27:1.00	4.57E+07	1.04E+08	0.44:1.00	19589	hypothetical protein POPTR_0008s09660g [Populus trichocarpa]
1800	1939	gi|566174518	45.72	7	1	1	2.94E+07	1.88E+07	8.02E+06	8.18E+07	4.91E+07	3.82E+07	0.77:0.49:0.21:2.14:1.28:1.00	1.88E+07	5.64E+07	0.33:1.00	40168	hypothetical protein POPTR_0006s04880g [Populus trichocarpa]
562	939	gi|566206623	45.61	8	3	1	1.07E+07	8.31E+06	8.45E+06	3.90E+07	2.43E+07	1.63E+07	0.66:0.51:0.52:2.39:1.49:1.00	9.16E+06	2.66E+07	0.35:1.00	54072	aldehyde dehydrogenase 1 precursor family protein [Populus trichocarpa]
2592	46154	gi|359496589	45.05	3	1	1	2.22E+07	2.22E+07	1.90E+07	3.26E+07	4.15E+07	3.64E+07	0.61:0.61:0.52:0.89:1.14:1.00	2.11E+07	3.68E+07	0.57:1.00	41483	PREDICTED: clathrin light chain 1 [Vitis vinifera]
674	764	gi|225441754	45.03	8	3	1	4.13E+07	8.97E+06	9.40E+06	9.00E+07	6.23E+07	5.20E+07	0.79:0.17:0.18:1.73:1.20:1.00	1.99E+07	6.81E+07	0.29:1.00	53382	PREDICTED: adenylosuccinate synthetase 2 chloroplastic [Vitis vinifera]
167	29851	gi|526117645	44.91	1	1	1	6.69E+06	3.50E+06	2.81E+06	1.78E+07	1.41E+07	9.09E+06	0.74:0.39:0.31:1.95:1.55:1.00	4.33E+06	1.37E+07	0.32:1.00	153182	magnesium chelatase H subunit [Vitis vinifera]
483	559	gi|552540866	44.07	4	1	1	3.87E+07	3.35E+07	2.95E+07	2.26E+07	1.68E+07	1.22E+07	3.18:2.76:2.43:1.86:1.38:1.00	3.39E+07	1.72E+07	1.98:1.00	35220	cytochrome f (chloroplast) [Camellia danzaiensis]
108	117	gi|566146990	43.81	9	7	1	8.28E+07	6.63E+07	5.69E+07	1.80E+08	1.14E+08	9.25E+07	0.90:0.72:0.62:1.95:1.23:1.00	6.87E+07	1.29E+08	0.53:1.00	71882	heat shock protein 70 cognate [Populus trichocarpa]
846	637	gi|568244554	43.76	10	3	3	2.43E+08	2.04E+08	1.80E+08	1.25E+08	1.04E+08	8.15E+07	2.98:2.50:2.21:1.53:1.27:1.00	2.09E+08	1.03E+08	2.02:1.00	39549	photosystem II protein D2 (plastid) [Camellia oleifera]
528	504	gi|552540956	43.72	8	3	3	1.91E+08	2.94E+08	1.66E+08	8.63E+07	6.80E+07	7.11E+07	2.69:4.14:2.34:1.21:0.96:1.00	2.17E+08	7.51E+07	2.89:1.00	38951	photosystem II Qb protein D1 (chloroplast) [Camellia impressinervis]

431	1825	gi|225439231	43.39	5	2	1	3.77E+07	4.53E+07	2.45E+07	8.69E+07	6.23E+07	4.68E+07	0.81:0.97:0.52:1.86:1.33:1.00	3.58E+07	6.53E+07	0.55:1.00	55389	PREDICTED: 3-isopropylmalate dehydratase large subunit-like [Vitis vinifera]
2174	1659	gi|359475330	43.38	7	1	1	1.30E+08	1.16E+08	1.11E+08	1.85E+08	1.94E+08	1.78E+08	0.73:0.65:0.62:1.04:1.09:1.00	1.19E+08	1.86E+08	0.64:1.00	16329	PREDICTED: glycine-rich RNA-binding protein GRP2A [Vitis vinifera]
807	1995	gi|566149424	43.37	4	1	1	1.38E+07	1.06E+07	9.48E+06	4.08E+07	2.49E+07	1.81E+07	0.76:0.59:0.52:2.25:1.38:1.00	1.13E+07	2.80E+07	0.40:1.00	46427	oligouridylate-binding family protein [Populus trichocarpa]
2617	2185	gi|566151403	42.94	20	1	1	3.00E+07	2.53E+07	2.63E+07	7.68E+07	6.42E+07	4.01E+07	0.75:0.63:0.66:1.92:1.60:1.00	2.72E+07	6.04E+07	0.45:1.00	8809	hypothetical protein POPTR_0001s28090g [Populus trichocarpa]
680	1614	gi|225466690	42.91	8	2	1	1.51E+07	5.03E+06	4.51E+06	4.19E+07	3.06E+07	2.35E+07	0.64:0.21:0.19:1.78:1.30:1.00	8.21E+06	3.20E+07	0.26:1.00	42525	PREDICTED: sedoheptulose-1 7-bisphosphatase chloroplastic [Vitis vinifera]
1172	45437	gi|566149444	42.74	6	2	2	9.91E+06	7.24E+06	1.44E+07	3.95E+07	2.72E+07	2.20E+07	0.45:0.33:0.65:1.79:1.23:1.00	1.05E+07	2.96E+07	0.36:1.00	54813	aldehyde dehydrogenase family 7 member a1 turgor responsive family protein [Populus trichocarpa]
730	1587	gi|566213188	42.66	5	1	1	5.59E+06	4.32E+06	3.00E+06	3.51E+07	1.39E+07	8.64E+06	0.65:0.50:0.35:4.06:1.61:1.00	4.30E+06	1.92E+07	0.22:1.00	43893	nodule-enhanced malate dehydrogenase family protein [Populus trichocarpa]
682	2754	gi|225465837	42.52	6	1	1	5.26E+07	4.14E+07	3.27E+07	1.15E+08	8.32E+07	6.82E+07	0.77:0.61:0.48:1.68:1.22:1.00	4.22E+07	8.86E+07	0.48:1.00	46787	PREDICTED: protein ASPARTIC PROTEASE IN GUARD CELL 2 [Vitis vinifera]
2599	54893	gi|225457361	41.92	6	1	1	0	1.09E+07	8.79E+06	5.61E+07	3.05E+07	1.95E+07	0:0.56:0.45:2.88:1.56:1.00	6.55E+06	3.54E+07	0.19:1.00	29994	PREDICTED: ribulose-phosphate 3-epimerase chloroplastic [Vitis vinifera]
1231	3913	gi|731426684	41.8	1	1	1	3.91E+07	3.19E+07	2.90E+07	1.06E+08	7.32E+07	4.97E+07	0.79:0.64:0.58:2.14:1.47:1.00	3.34E+07	7.64E+07	0.44:1.00	144086	PREDICTED: uncharacterized protein LOC100262718 isoform X4 [Vitis vinifera]
810	22054	gi|566176147	41.02	4	2	1	2.34E+07	2.53E+07	2.15E+07	9.11E+06	1.67E+07	1.75E+07	1.34:1.45:1.23:0.52:0.96:1.00	2.34E+07	1.44E+07	1.62:1.00	41553	hypothetical protein POPTR_0006s13470g [Populus trichocarpa]
683	3965	gi|224114720	40.89	7	2	2	1.44E+07	1.03E+07	7.63E+06	6.42E+07	4.27E+07	1.73E+07	0.84:0.60:0.44:3.72:2.48:1.00	1.08E+07	4.14E+07	0.26:1.00	45639	Magnesium-chelatase subunit chlI family protein [Populus trichocarpa]
105	123	gi|224365668	40.75	7	3	3	1.70E+08	1.62E+08	1.45E+08	2.74E+08	2.48E+08	2.26E+08	0.75:0.72:0.64:1.21:1.10:1.00	1.59E+08	2.49E+08	0.64:1.00	55143	ATPase subunit 1 (mitochondrion) [Vitis vinifera]
644	2151	gi|225444472	39.97	8	2	2	4.75E+07	3.48E+07	3.44E+07	7.10E+07	6.18E+07	6.48E+07	0.73:0.54:0.53:1.10:0.95:1.00	3.89E+07	6.59E+07	0.59:1.00	44306	PREDICTED: aminomethyltransferase mitochondrial [Vitis vinifera]
785	2419	gi|224137032	39.74	1	1	1	2.55E+07	2.17E+07	1.70E+07	5.75E+07	4.42E+07	2.76E+07	0.92:0.78:0.61:2.08:1.60:1.00	2.14E+07	4.31E+07	0.50:1.00	147951	subtilase family protein [Populus trichocarpa]

2720	2669	gi|224130034	39.66	2	1	1	1.75E+07	2.50E+07	2.26E+07	7.72E+06	1.53E+07	1.75E+07	1.00:1.43:1.29:0.44:0.88:1.00	2.17E+07	1.35E+07	1.61:1.00	43250	Fasciclin-like arabinogalactan protein 8 precursor [Populus trichocarpa]
372	470	gi|224109788	39.39	11	2	1	3.41E+07	3.38E+07	3.09E+07	1.13E+08	7.50E+07	5.13E+07	0.66:0.66:0.60:2.21:1.46:1.00	3.29E+07	7.99E+07	0.41:1.00	37547	putative adenosine kinase family protein [Populus trichocarpa]
1167	22126	gi|731439997	39.19	4	1	1	2.33E+07	1.27E+07	1.54E+07	4.92E+07	3.87E+07	1.94E+07	1.20:0.65:0.79:2.54:2.00:1.00	1.71E+07	3.58E+07	0.48:1.00	33522	PREDICTED: phosphoglycolate phosphatase 1B chloroplastic [Vitis vinifera]
2310	2791	gi|225440849	39.04	19	1	1	2.81E+07	1.94E+07	1.60E+07	6.00E+07	4.60E+07	3.48E+07	0.81:0.56:0.46:1.72:1.32:1.00	2.12E+07	4.69E+07	0.45:1.00	8952	PREDICTED: probable small nuclear ribonucleoprotein G [Vitis vinifera]
2377	72648	gi|566239233	38.98	2	1	1	2.06E+07	9.95E+06	1.88E+07	7.86E+07	3.38E+07	2.19E+07	0.94:0.46:0.86:3.59:1.55:1.00	1.65E+07	4.48E+07	0.37:1.00	94826	aspartate kinase family protein [Populus trichocarpa]
2467	46193	gi|225435608	38.7	8	1	1	4.03E+07	0	0	4.46E+07	4.37E+07	6.05E+07	0.66:0:0:0.74:0.72:1.00	1.34E+07	4.96E+07	0.27:1.00	31609	PREDICTED: zinc finger CCCH domain-containing protein 14-like [Vitis vinifera]
374	825	gi|225457957	38.3	34	4	1	1.11E+07	8.72E+06	8.55E+06	2.75E+07	2.12E+07	1.66E+07	0.67:0.53:0.52:1.66:1.28:1.00	9.46E+06	2.17E+07	0.43:1.00	17938	PREDICTED: peptidyl-prolyl cis-trans isomerase [Vitis vinifera]
1791	7321	gi|566160631	38.26	2	1	1	1.25E+07	9.78E+06	6.93E+06	4.01E+07	2.07E+07	1.56E+07	0.80:0.63:0.45:2.58:1.33:1.00	9.73E+06	2.55E+07	0.38:1.00	73609	endomembrane protein 70 [Populus trichocarpa]
1294	927	gi|225444649	37.02	10	2	2	2.22E+08	2.19E+08	2.09E+08	4.01E+08	3.19E+08	2.66E+08	0.83:0.82:0.78:1.51:1.20:1.00	2.16E+08	3.28E+08	0.66:1.00	26396	PREDICTED: 20 kDa chaperonin chloroplastic [Vitis vinifera]
1181	1032	gi|225455934	36.92	12	3	1	2.19E+09	2.10E+09	1.91E+09	1.29E+09	1.23E+09	1.08E+09	2.03:1.95:1.77:1.20:1.15:1.00	2.06E+09	1.20E+09	1.72:1.00	20429	PREDICTED: ribulose bisphosphate carboxylase small chain chloroplastic [Vitis vinifera]
2247	22157	gi|224124892	36.9	6	1	1	5.73E+07	5.11E+07	4.47E+07	1.29E+08	1.13E+08	9.43E+07	0.61:0.54:0.47:1.37:1.20:1.00	5.11E+07	1.12E+08	0.46:1.00	22641	rhicadhesin receptor family protein [Populus trichocarpa]
2137	7457	gi|224109088	36.57	2	1	1	1.79E+07	1.81E+07	1.38E+07	3.94E+07	3.84E+07	2.60E+07	0.69:0.70:0.53:1.52:1.48:1.00	1.66E+07	3.46E+07	0.48:1.00	57503	SUCCINIC SEMIALDEHYDE DEHYDROGENASE family protein [Populus trichocarpa]
549	1143	gi|225430366	36.57	1	1	1	1.15E+07	8.72E+06	7.51E+06	2.59E+07	1.78E+07	1.28E+07	0.90:0.68:0.59:2.02:1.39:1.00	9.26E+06	1.89E+07	0.49:1.00	108100	PREDICTED: chaperone protein ClpB4 mitochondrial [Vitis vinifera]
114	128	gi|224073124	36.52	11	4	1	2.17E+07	1.97E+07	1.58E+07	7.60E+07	2.89E+07	2.06E+07	1.05:0.96:0.77:3.69:1.40:1.00	1.91E+07	3.76E+07	0.51:1.00	64544	chaperonin precursor family protein [Populus trichocarpa]
1251	1523	gi|566175251	36.42	5	2	2	5.67E+07	1.26E+07	9.28E+06	1.24E+08	2.20E+07	7.28E+07	0.78:0.17:0.13:1.71:0.30:1.00	2.62E+07	7.30E+07	0.36:1.00	34569	hypothetical protein POPTR_0006s08710g [Populus trichocarpa]

347	468	gi|225427917	36.33	9	2	1	9.86E+07	8.49E+07	7.89E+07	1.69E+08	1.40E+08	1.09E+08	0.90:0.78:0.72:1.55:1.28:1.00	8.75E+07	1.39E+08	0.63:1.00	34675	PREDICTED: triosephosphate isomerase chloroplastic [Vitis vinifera]
1557	1173	gi|224131818	36.02	5	2	1	3.13E+08	3.11E+08	3.11E+08	1.52E+08	1.62E+08	1.46E+08	2.15:2.13:2.13:1.04:1.11:1.00	3.12E+08	1.54E+08	2.03:1.00	28513	chlorophyll a/b-binding family protein [Populus trichocarpa]
2733	3734	gi|359494267	35.73	3	1	1	3.67E+07	3.88E+07	3.86E+07	1.05E+07	2.15E+07	2.96E+07	1.24:1.31:1.31:0.35:0.73:1.00	3.81E+07	2.05E+07	1.86:1.00	25766	PREDICTED: probable glutathione S-transferase [Vitis vinifera]
2618	2762	gi|225435002	35.69	6	1	1	8.38E+07	7.98E+07	7.38E+07	1.44E+08	1.31E+08	1.17E+08	0.72:0.68:0.63:1.23:1.12:1.00	7.91E+07	1.31E+08	0.61:1.00	27396	PREDICTED: osmotin-like protein [Vitis vinifera]
2187	7497	gi|566167266	34.75	4	1	1	2.03E+07	1.57E+07	1.26E+07	4.96E+07	2.97E+07	2.27E+07	0.89:0.69:0.55:2.18:1.31:1.00	1.62E+07	3.40E+07	0.48:1.00	37830	hypothetical protein POPTR_0004s18610g [Populus trichocarpa]
2233	3166	gi|566210985	34.74	3	1	1	9.57E+06	5.87E+06	5.20E+06	1.74E+07	1.46E+07	1.13E+07	0.85:0.52:0.46:1.54:1.30:1.00	6.88E+06	1.44E+07	0.48:1.00	51941	glycosyl hydrolase family 17 family protein [Populus trichocarpa]
170	353	gi|359496771	34.29	8	4	1	1.01E+08	8.03E+07	4.21E+07	2.32E+08	9.80E+07	8.44E+07	1.19:0.95:0.50:2.74:1.16:1.00	7.44E+07	1.38E+08	0.54:1.00	73271	PREDICTED: heat shock 70 kDa protein mitochondrial-like [Vitis vinifera]
1343	15240	gi|566170761	34.18	2	1	1	1.34E+07	1.71E+07	1.32E+07	3.52E+07	3.25E+07	2.67E+07	0.50:0.64:0.49:1.32:1.22:1.00	1.46E+07	3.15E+07	0.46:1.00	48329	hypothetical protein POPTR_0005s11300g [Populus trichocarpa]
1957	1621	gi|566168995	33.08	13	1	1	4.88E+07	2.56E+07	2.77E+07	1.07E+08	7.45E+07	5.28E+07	0.93:0.48:0.53:2.04:1.41:1.00	3.40E+07	7.82E+07	0.44:1.00	19031	callus protein P23 [Populus trichocarpa]
1290	17624	gi|566195934	32.83	1	1	1	2.72E+07	2.35E+07	2.03E+07	5.03E+07	4.71E+07	3.32E+07	0.82:0.71:0.61:1.51:1.42:1.00	2.36E+07	4.35E+07	0.54:1.00	83449	alkaline alpha galactosidase I family protein [Populus trichocarpa]
157	168	gi|359490179	32.76	2	1	1	1.38E+08	1.09E+08	1.09E+08	2.10E+08	2.08E+08	1.45E+08	0.96:0.76:0.76:1.45:1.44:1.00	1.19E+08	1.88E+08	0.63:1.00	78856	PREDICTED: transketolase chloroplastic [Vitis vinifera]
829	855	gi|225430650	32.36	3	1	1	1.26E+07	1.20E+07	1.10E+07	3.06E+07	2.43E+07	1.90E+07	0.66:0.64:0.58:1.61:1.28:1.00	1.19E+07	2.46E+07	0.48:1.00	43927	PREDICTED: pyruvate dehydrogenase E1 component subunit beta-3 chloroplastic [Vitis vinifera]
1296	1359	gi|225429209	31.77	1	1	1	3.70E+07	4.20E+07	4.38E+07	3.35E+06	5.70E+06	2.60E+07	1.42:1.61:1.68:0.13:0.22:1.00	4.09E+07	1.17E+07	3.50:1.00	53142	PREDICTED: serine carboxypeptidase-like 16 [Vitis vinifera]
415	585	gi|566210462	31.55	2	1	1	2.91E+07	2.45E+07	2.08E+07	8.63E+07	3.93E+07	6.35E+07	0.46:0.39:0.33:1.36:0.62:1.00	2.48E+07	6.30E+07	0.39:1.00	67453	Methylenetetrahydrofolate reductase family protein [Populus trichocarpa]
942	1557	gi|566178300	31.24	4	1	1	2.20E+07	2.31E+07	2.31E+07	5.73E+07	4.07E+07	3.30E+07	0.67:0.70:0.70:1.74:1.23:1.00	2.28E+07	4.36E+07	0.52:1.00	45519	hypothetical protein POPTR_0006s26630g [Populus trichocarpa]
2562	2875	gi|566184421	31.15	3	1	1	2.77E+07	1.77E+07	1.23E+07	4.51E+07	3.72E+07	2.15E+07	1.29:0.82:0.57:2.10:1.73:1.00	1.92E+07	3.46E+07	0.56:1.00	45388	hypothetical protein POPTR_0008s17030g [Populus trichocarpa]
1491	2202	gi|225447176	30.95	1	1	1	8.97E+06	1.15E+07	1.04E+07	1.93E+06	6.08E+06	5.43E+06	1.65:2.12:1.91:0.36:1.12:1.00	1.03E+07	4.48E+06	2.30:1.00	46608	PREDICTED: protein disulfide isomerase-like 2-3 [Vitis vinifera]

2197	45734	gi|225454123	30.79	7	1	1	1.08E+07	9.56E+06	6.95E+06	2.06E+07	1.70E+07	1.35E+07	0.80:0.71:0.51:1.53:1.26:1.00	9.10E+06	1.71E+07	0.53:1.00	22997	PREDICTED: pre-mRNA cleavage factor Im 25 kDa subunit 2 [Vitis vinifera]
590	1041	gi|225447725	30.77	5	2	1	4.19E+07	3.40E+07	3.17E+07	6.73E+07	5.92E+07	5.31E+07	0.79:0.64:0.60:1.27:1.11:1.00	3.58E+07	5.99E+07	0.60:1.00	61438	PREDICTED: calnexin homolog [Vitis vinifera]
1130	1545	gi|224104631	30.49	4	1	1	2.84E+08	3.04E+08	3.01E+08	1.12E+08	1.86E+08	2.18E+08	1.30:1.39:1.38:0.51:0.86:1.00	2.96E+08	1.72E+08	1.72:1.00	27319	cytosolic ascorbate peroxidase family protein [Populus trichocarpa]
1567	1773	gi|731436956	30.39	4	1	1	2.03E+07	1.39E+07	1.71E+07	2.79E+07	2.88E+07	2.21E+07	0.92:0.63:0.78:1.26:1.31:1.00	1.71E+07	2.63E+07	0.65:1.00	39713	PREDICTED: porphobilinogen deaminase chloroplastic [Vitis vinifera]
2235	3340	gi|224109362	30.36	10	2	1	1.14E+07	7.62E+06	5.46E+06	4.05E+07	2.04E+07	1.85E+07	0.62:0.41:0.29:2.18:1.10:1.00	8.17E+06	2.65E+07	0.31:1.00	31536	Eukaryotic translation initiation factor 3 subunit 5 family protein [Populus trichocarpa]
615	3014	gi|225443760	30.28	8	1	1	3.08E+07	2.48E+07	2.39E+07	5.87E+07	5.11E+07	4.00E+07	0.77:0.62:0.60:1.47:1.28:1.00	2.65E+07	4.99E+07	0.53:1.00	20875	PREDICTED: 60S ribosomal protein L11-1-like [Vitis vinifera]
609	1158	gi|552540940	30.1	3	1	1	4.86E+07	4.42E+07	3.91E+07	7.58E+07	6.46E+07	5.77E+07	0.84:0.77:0.68:1.31:1.12:1.00	4.40E+07	6.60E+07	0.67:1.00	58361	acetyl-CoA carboxylase carboxyl transferase beta (chloroplast) [Camellia impressinervis]
747	1691	gi|224063997	29.97	6	3	1	3.58E+06	2.40E+06	9.78E+05	7.58E+06	7.53E+06	5.05E+06	0.71:0.47:0.19:1.50:1.49:1.00	2.32E+06	6.72E+06	0.34:1.00	67268	NAD-dependent malic enzyme family protein [Populus trichocarpa]
888	2264	gi|225434133	29.75	7	2	1	5.61E+07	4.41E+07	3.70E+07	1.26E+08	1.00E+08	7.36E+07	0.76:0.60:0.50:1.71:1.36:1.00	4.57E+07	1.00E+08	0.46:1.00	40613	PREDICTED: cysteine synthase chloroplastic/chromoplastic [Vitis vinifera]
2301	2433	gi|225458237	29.7	5	1	1	2.30E+07	2.46E+07	2.22E+07	4.21E+07	4.05E+07	3.66E+07	0.63:0.67:0.61:1.15:1.11:1.00	2.33E+07	3.98E+07	0.59:1.00	29571	PREDICTED: gamma carbonic anhydrase 1 mitochondrial [Vitis vinifera]
34	122	gi|225462164	29.66	7	4	1	1.52E+08	1.44E+08	8.83E+07	2.79E+08	2.11E+08	1.70E+08	0.89:0.85:0.52:1.64:1.24:1.00	1.33E+08	2.20E+08	0.60:1.00	93979	PREDICTED: elongation factor 2 [Vitis vinifera]
193	257	gi|225433375	29.28	2	1	1	4.72E+07	3.45E+07	3.57E+07	1.08E+08	6.43E+07	5.10E+07	0.93:0.68:0.70:2.12:1.26:1.00	3.91E+07	7.45E+07	0.53:1.00	61370	PREDICTED: chaperonin CPN60-2 mitochondrial [Vitis vinifera]
1320	22312	gi|731430037	28.72	4	1	1	1.26E+07	9.46E+06	8.01E+06	3.02E+07	1.74E+07	1.41E+07	0.89:0.67:0.57:2.14:1.23:1.00	1.00E+07	2.05E+07	0.49:1.00	43016	PREDICTED: probable cinnamyl alcohol dehydrogenase 6 [Vitis vinifera]
2491	55198	gi|566185913	28.35	3	1	1	6.00E+07	4.65E+07	4.00E+07	1.18E+08	8.34E+07	6.76E+07	0.89:0.69:0.59:1.74:1.23:1.00	4.88E+07	8.96E+07	0.54:1.00	55777	hypothetical protein POPTR_0009s02300g partial [Populus trichocarpa]
1342	14827	gi|731380214	28.11	4	1	1	3.32E+06	5.74E+06	5.97E+06	2.00E+07	1.58E+07	2.63E+06	1.26:2.19:2.27:7.63:6.02:1.00	5.01E+06	1.28E+07	0.39:1.00	37604	PREDICTED: L-lactate dehydrogenase A isoform X1 [Vitis vinifera]
2682	72842	gi|225432296	27.88	10	1	1	4.52E+06	2.74E+06	2.51E+06	1.73E+07	1.10E+07	6.62E+06	0.68:0.41:0.38:2.62:1.66:1.00	3.26E+06	1.17E+07	0.28:1.00	16839	PREDICTED: uncharacterized protein LOC100249327 [Vitis vinifera]

1426	30756	gi|731440862	27.88	11	1	1	2.00E+07	1.27E+07	1.42E+07	3.83E+07	3.72E+07	2.18E+07	0.91:0.58:0.65:1.75:1.70:1.00	1.58E+07	3.24E+07	0.49:1.00	15642	PREDICTED: 60S ribosomal protein L32-1 [Vitis vinifera]
88	280	gi|225443399	27.87	10	4	1	7.02E+07	4.91E+07	4.38E+07	9.74E+07	8.83E+07	8.79E+07	0.80:0.56:0.50:1.11:1.00:1.00	5.44E+07	9.12E+07	0.60:1.00	68723	PREDICTED: V-type proton ATPase catalytic subunit A [Vitis vinifera]
135	121	gi|542688129	27.83	4	2	2	8.24E+07	6.85E+07	5.96E+07	4.90E+07	4.48E+07	3.72E+07	2.22:1.84:1.60:1.32:1.20:1.00	7.02E+07	4.37E+07	1.61:1.00	53694	ATP synthase CF1 beta subunit (chloroplast) [Camellia taliensis]
678	1383	gi|526118026	27.6	2	1	1	2.91E+07	3.11E+06	3.15E+06	2.24E+07	3.90E+07	9.12E+06	3.19:0.34:0.35:2.46:4.28:1.00	1.18E+07	2.35E+07	0.50:1.00	55487	5-enolpyruvylshikimate-3-phosphate synthase [Vitis vinifera]
131	7344	gi|224119200	27.59	2	3	1	1.87E+07	1.48E+07	9.13E+06	3.21E+07	2.26E+07	1.82E+07	1.02:0.81:0.50:1.76:1.24:1.00	1.42E+07	2.43E+07	0.58:1.00	136903	coatomer alpha subunit-like family protein [Populus trichocarpa]
1104	22120	gi|224129210	27.55	4	1	1	1.85E+07	1.44E+07	1.47E+07	4.26E+07	3.09E+07	1.83E+07	1.01:0.79:0.80:2.32:1.68:1.00	1.59E+07	3.06E+07	0.52:1.00	41121	truncated acetyl Co-A acetyltransferase-like family protein [Populus trichocarpa]
1717	1481	gi|566202597	27.31	11	1	1	2.69E+07	2.19E+07	1.97E+07	4.66E+07	4.14E+07	3.38E+07	0.80:0.65:0.58:1.38:1.23:1.00	2.28E+07	4.06E+07	0.56:1.00	16336	hypothetical protein POPTR_0014s04880g [Populus trichocarpa]
953	522	gi|566184073	27.25	7	2	2	2.31E+08	2.18E+08	1.96E+08	1.40E+08	1.19E+08	1.29E+08	1.80:1.70:1.52:1.09:0.92:1.00	2.15E+08	1.29E+08	1.66:1.00	22518	photosystem I 20kD family protein [Populus trichocarpa]
672	729	gi|731404671	27	10	2	1	1.41E+07	1.32E+07	9.28E+06	3.81E+07	2.23E+07	1.54E+07	0.91:0.85:0.60:2.47:1.44:1.00	1.22E+07	2.53E+07	0.48:1.00	29622	PREDICTED: 14-3-3-like protein D isoform X1 [Vitis vinifera]
154	1094	gi|566150406	26.97	3	2	1	1.91E+07	0	0	8.42E+07	4.89E+07	2.08E+07	0.92:0:0:4.05:2.35:1.00	6.37E+06	5.13E+07	0.12:1.00	101827	Argonaute 4 family protein [Populus trichocarpa]
1664	1489	gi|225453350	26.75	11	1	1	5.77E+07	5.17E+07	4.66E+07	9.50E+07	8.24E+07	6.67E+07	0.87:0.77:0.70:1.43:1.24:1.00	5.20E+07	8.14E+07	0.64:1.00	16310	PREDICTED: nucleoside diphosphate kinase B [Vitis vinifera]
159	237	gi|224063263	26.67	9	4	1	6.31E+07	6.75E+07	8.02E+07	1.13E+08	1.19E+08	1.05E+08	0.60:0.64:0.76:1.07:1.13:1.00	6.82E+07	1.12E+08	0.61:1.00	58692	2-dehydro-3-deoxyphosphoheptonate aldolase family protein [Populus trichocarpa]
1893	1754	gi|224084784	26.48	1	1	1	2.91E+07	3.65E+07	3.51E+07	9.68E+06	1.79E+07	2.75E+07	1.06:1.33:1.28:0.35:0.65:1.00	3.36E+07	1.84E+07	1.83:1.00	61214	glycine hydroxymethyltransferase family protein [Populus trichocarpa]
751	1422	gi|731387895	26.19	3	1	1	8.81E+07	6.14E+07	8.52E+07	1.93E+08	1.49E+08	8.79E+07	1.00:0.70:0.97:2.20:1.70:1.00	7.82E+07	1.43E+08	0.55:1.00	51263	PREDICTED: UTP--glucose-1-phosphate uridylyltransferase isoform X2 [Vitis vinifera]
1644	22905	gi|225434323	26.01	4	1	1	3.50E+07	2.51E+07	2.32E+07	6.00E+07	4.88E+07	4.02E+07	0.87:0.62:0.58:1.49:1.21:1.00	2.78E+07	4.97E+07	0.56:1.00	30353	PREDICTED: eukaryotic translation initiation factor 2 subunit beta [Vitis vinifera]
1979	7488	gi|224107655	25.99	7	1	1	0	0	0	2.22E+07	6.63E+06	3.26E+06	0:0:0:6.81:2.03:1.00	0	1.07E+07	00:01.0	33896	3-oxoacyl-[acyl-carrier-protein] reductase [Populus trichocarpa]
2136	2831	gi|224089062	25.18	6	1	1	2.33E+07	1.77E+07	1.36E+07	6.72E+07	4.85E+07	3.04E+07	0.77:0.58:0.45:2.21:1.60:1.00	1.82E+07	4.87E+07	0.37:1.00	27753	hypothetical protein POPTR_0006s25870g [Populus trichocarpa]

2525	4507	gi|225451318	25.08	4	1	1	3.10E+07	2.71E+07	2.37E+07	7.01E+07	4.43E+07	3.70E+07	0.84:0.73:0.64:1.89:1.20:1.00	2.73E+07	5.05E+07	0.54:1.00	37997	PREDICTED: probable aldo-keto reductase 1 [Vitis vinifera]
1849	46057	gi|731389447	25.08	2	1	1	1.32E+07	6.34E+06	6.03E+06	5.00E+07	2.57E+07	2.14E+07	0.62:0.30:0.28:2.34:1.20:1.00	8.54E+06	3.24E+07	0.26:1.00	84064	PREDICTED: acylamino-acid-releasing enzyme isoform X2 [Vitis vinifera]
2722	2806	gi|224137260	24.89	12	1	1	1.23E+08	9.12E+07	8.35E+07	2.23E+08	1.67E+08	1.43E+08	0.86:0.64:0.58:1.56:1.17:1.00	9.93E+07	1.78E+08	0.56:1.00	11459	hypothetical protein POPTR_0016s01100g [Populus trichocarpa]
1136	2707	gi|224120174	24.82	2	1	1	7.04E+06	5.03E+06	3.59E+06	2.04E+07	1.07E+07	1.07E+07	0.66:0.47:0.34:1.91:1.00:1.00	5.22E+06	1.39E+07	0.37:1.00	68576	Phosphoglucomutase family protein [Populus trichocarpa]
227	231	gi|566204141	24.35	9	3	1	1.70E+08	1.37E+08	1.26E+08	3.99E+08	2.23E+08	1.90E+08	0.89:0.72:0.66:2.10:1.17:1.00	1.44E+08	2.55E+08	0.57:1.00	42935	hypothetical protein POPTR_0014s13660g [Populus trichocarpa]
1170	37574	gi|225464906	24.12	9	1	1	5.25E+07	5.01E+07	3.54E+07	7.18E+07	8.32E+07	7.16E+07	0.73:0.70:0.49:1.00:1.16:1.00	4.73E+07	7.60E+07	0.62:1.00	16377	PREDICTED: 40S ribosomal protein S14 [Vitis vinifera]
1120	979	gi|224108938	23.93	8	1	1	2.93E+07	2.23E+07	2.06E+07	6.99E+07	4.06E+07	2.84E+07	1.03:0.78:0.73:2.46:1.43:1.00	2.41E+07	4.63E+07	0.52:1.00	17381	hypothetical protein POPTR_0010s17020g [Populus trichocarpa]
1505	1179	gi|566170929	23.83	5	1	1	8.90E+07	7.20E+07	5.85E+07	1.94E+08	1.35E+08	1.11E+08	0.80:0.65:0.53:1.75:1.22:1.00	7.32E+07	1.47E+08	0.50:1.00	36394	hypothetical protein POPTR_0005s12070g [Populus trichocarpa]
572	1069	gi|225457219	23.77	3	1	1	8.90E+06	1.35E+07	1.14E+07	3.12E+07	2.38E+07	2.10E+07	0.42:0.64:0.54:1.49:1.13:1.00	1.16E+07	2.53E+07	0.46:1.00	35220	PREDICTED: ATP synthase subunit gamma mitochondrial [Vitis vinifera]
591	1171	gi|224135449	23.63	2	1	1	4.92E+07	4.28E+07	4.51E+07	7.95E+07	6.95E+07	6.17E+07	0.80:0.69:0.73:1.29:1.13:1.00	4.57E+07	7.02E+07	0.65:1.00	57629	chaperonin family protein [Populus trichocarpa]
454	1592	gi|225453246	23.5	2	1	1	9.80E+06	1.40E+07	1.07E+07	3.52E+07	2.17E+07	1.95E+07	0.50:0.72:0.55:1.81:1.11:1.00	1.15E+07	2.55E+07	0.45:1.00	69276	PREDICTED: dynamin-related protein 1E [Vitis vinifera]
1735	22123	gi|225435203	23.26	8	1	1	5.52E+07	5.19E+07	3.84E+07	8.55E+07	7.43E+07	6.26E+07	0.88:0.83:0.61:1.36:1.19:1.00	4.85E+07	7.41E+07	0.65:1.00	15652	PREDICTED: 40S ribosomal protein S23-like [Vitis vinifera]
1293	45435	gi|224059670	23.19	5	1	1	9.77E+06	7.58E+06	6.65E+06	3.90E+07	2.83E+07	1.98E+07	0.49:0.38:0.34:1.97:1.43:1.00	8.00E+06	2.90E+07	0.28:1.00	25980	60S ribosomal protein L6 [Populus trichocarpa]
602	22099	gi|566146555	23.09	8	1	1	4.24E+07	2.84E+07	2.41E+07	6.73E+07	1.25E+08	3.93E+07	1.08:0.72:0.61:1.71:3.18:1.00	3.16E+07	7.72E+07	0.41:1.00	23812	60S ribosomal protein L13 [Populus trichocarpa]
2147	1881	gi|526117942	23.02	4	1	1	3.35E+07	2.25E+07	1.46E+07	5.28E+07	4.32E+07	4.11E+07	0.81:0.55:0.36:1.28:1.05:1.00	2.35E+07	4.57E+07	0.51:1.00	37456	beta 1-3 glucanase precursor [Vitis vinifera]
1366	1649	gi|731371455	22.88	2	1	1	2.47E+07	1.78E+07	1.33E+07	4.44E+07	3.02E+07	2.65E+07	0.93:0.67:0.50:1.68:1.14:1.00	1.86E+07	3.37E+07	0.55:1.00	58282	PREDICTED: coatomer subunit delta [Vitis vinifera]
1991	14731	gi|225465441	22.73	12	1	1	1.75E+07	1.46E+07	1.29E+07	2.96E+07	2.36E+07	1.89E+07	0.93:0.77:0.68:1.57:1.25:1.00	1.50E+07	2.41E+07	0.62:1.00	14675	PREDICTED: cytochrome b5 [Vitis vinifera]
1585	3017	gi|225455106	22.6	4	1	1	3.36E+07	3.30E+07	3.45E+07	5.95E+07	5.15E+07	4.64E+07	0.72:0.71:0.74:1.28:1.11:1.00	3.37E+07	5.25E+07	0.64:1.00	33350	PREDICTED: protein BOBBER 1 [Vitis vinifera]

2220	2201	gi|731408423	22.49	2	1	1	1.33E+07	1.60E+07	1.43E+07	1.22E+06	8.02E+06	8.97E+06	1.49:1.79:1.60:0.14:0.89:1.00	1.46E+07	6.07E+06	2.40:1.00	43098	PREDICTED: serine carboxypeptidase-like 18 isoform X4 [Vitis vinifera]
1961	1934	gi|225437898	22.42	6	2	1	6.38E+07	6.51E+07	6.77E+07	3.20E+07	3.10E+07	3.16E+07	2.02:2.06:2.14:1.01:0.98:1.00	6.56E+07	3.15E+07	2.08:1.00	15168	PREDICTED: photosystem I reaction center subunit IV B chloroplastic-like [Vitis vinifera]
1853	1401	gi|225435878	22.36	10	1	1	5.23E+07	5.19E+07	4.97E+07	1.24E+08	7.74E+07	7.24E+07	0.72:0.72:0.69:1.71:1.07:1.00	5.13E+07	8.71E+07	0.59:1.00	10567	PREDICTED: 10 kDa chaperonin [Vitis vinifera]
652	1407	gi|225432012	22.27	2	1	1	4.61E+07	7.72E+06	4.71E+06	1.57E+08	9.48E+06	1.22E+07	3.77:0.63:0.38:12.83:0.77:1.00	1.95E+07	4.74E+07	0.41:1.00	54243	PREDICTED: galactokinase [Vitis vinifera]
1969	3021	gi|224078826	22.15	4	1	1	5.84E+08	6.09E+08	5.89E+08	4.01E+08	3.98E+08	3.88E+08	1.51:1.57:1.52:1.03:1.03:1.00	5.94E+08	3.95E+08	1.50:1.00	24905	Oxygen-evolving enhancer protein 3-1 [Populus trichocarpa]
1049	1672	gi|566204499	22.13	4	1	1	6.78E+07	1.93E+07	1.55E+07	5.26E+07	3.82E+07	9.68E+07	0.70:0.20:0.16:0.54:0.39:1.00	3.42E+07	6.25E+07	0.55:1.00	47320	thioredoxin family protein [Populus trichocarpa]
819	2031	gi|225425838	22	10	2	1	8.51E+06	9.09E+06	7.44E+06	1.35E+07	1.50E+07	1.70E+07	0.50:0.54:0.44:0.79:0.88:1.00	8.25E+06	1.52E+07	0.54:1.00	26900	PREDICTED: adenylate kinase 4-like [Vitis vinifera]
2753	22542	gi|566210973	21.81	6	1	1	5.72E+07	5.80E+07	4.96E+07	2.30E+07	3.03E+07	3.36E+07	1.70:1.73:1.48:0.68:0.90:1.00	5.49E+07	2.90E+07	1.90:1.00	15927	hypothetical protein POPTR_0017s02810g [Populus trichocarpa]
277	445	gi|225427768	21.71	17	4	1	1.07E+08	9.33E+07	8.22E+07	2.60E+08	1.53E+08	1.35E+08	0.80:0.69:0.61:1.93:1.13:1.00	9.43E+07	1.73E+08	0.54:1.00	42948	PREDICTED: fructose-bisphosphate aldolase 1 chloroplastic [Vitis vinifera]
2219	1982	gi|224107333	21.66	12	1	1	4.15E+07	3.94E+07	3.53E+07	8.25E+07	5.00E+07	6.90E+07	0.60:0.57:0.51:1.20:0.72:1.00	3.87E+07	7.06E+07	0.55:1.00	9716	hypothetical protein POPTR_0010s02580g [Populus trichocarpa]
834	1045	gi|225459292	21.6	6	2	1	5.36E+06	3.50E+06	3.35E+06	1.27E+07	8.17E+06	6.55E+06	0.82:0.53:0.51:1.94:1.25:1.00	4.07E+06	9.27E+06	0.44:1.00	28739	PREDICTED: 14-3-3 protein 7 [Vitis vinifera]
2629	7527	gi|566152777	21.53	15	1	1	9.12E+06	9.57E+06	8.60E+06	3.28E+07	2.37E+07	1.44E+07	0.63:0.67:0.60:2.28:1.64:1.00	9.10E+06	2.36E+07	0.39:1.00	12696	hypothetical protein POPTR_0001s34690g [Populus trichocarpa]
919	1679	gi|526117723	21.5	3	1	1	5.98E+07	4.64E+07	5.00E+07	9.72E+07	8.50E+07	6.04E+07	0.99:0.77:0.83:1.61:1.41:1.00	5.21E+07	8.09E+07	0.64:1.00	56980	catalase [Vitis vinifera]
2551	54869	gi|566187444	21.45	2	1	1	1.69E+07	1.31E+07	1.24E+07	0	2.10E+06	3.84E+06	4.39:3.40:3.24:0:0.55:1.00	1.41E+07	1.98E+06	7.14:1.00	54252	allene oxide synthase family protein [Populus trichocarpa]
983	22324	gi|731440185	21.32	8	2	1	4.32E+07	3.50E+07	3.42E+07	7.16E+07	8.14E+07	4.85E+07	0.89:0.72:0.70:1.48:1.68:1.00	3.75E+07	6.54E+07	0.57:1.00	30189	PREDICTED: 60S ribosomal protein L5-like [Vitis vinifera]
775	45654	gi|224129596	21.32	6	1	1	0	0	0	7.51E+07	4.16E+07	0	0:0:0:1.00:0.55:0	0	3.89E+07	00:01.0	45020	Succinyl-CoA ligase beta-chain family protein [Populus trichocarpa]
908	1133	gi|225438781	21.24	2	1	1	1.98E+07	1.53E+07	1.56E+07	3.44E+07	3.04E+07	2.38E+07	0.83:0.64:0.65:1.44:1.28:1.00	1.69E+07	2.95E+07	0.57:1.00	71362	PREDICTED: polyadenylate-binding protein 2 [Vitis vinifera]

2541	1666	gi|731384053	21.14	2	1	1	7.90E+07	7.59E+07	6.08E+07	1.61E+08	1.08E+08	9.50E+07	0.83:0.80:0.64:1.69:1.13:1.00	7.19E+07	1.21E+08	0.59:1.00	52608	PREDICTED: LL-diaminopimelate aminotransferase chloroplastic isoform X1 [Vitis vinifera]
502	1083	gi|359481634	21.13	23	2	1	3.66E+07	3.18E+07	2.32E+07	6.45E+07	5.02E+07	4.27E+07	0.86:0.74:0.54:1.51:1.17:1.00	3.05E+07	5.25E+07	0.58:1.00	16594	PREDICTED: ubiquitin-conjugating enzyme E2 variant 1D [Vitis vinifera]
1507	1711	gi|224057272	21.03	5	1	1	6.95E+07	1.60E+07	6.20E+07	1.59E+08	4.00E+07	9.06E+07	0.77:0.18:0.68:1.76:0.44:1.00	4.92E+07	9.66E+07	0.51:1.00	45612	aspartyl protease family protein [Populus trichocarpa]
1836	7461	gi|731428733	20.89	0	1	1	5.54E+07	5.60E+07	5.26E+07	8.28E+07	9.37E+07	8.94E+07	0.62:0.63:0.59:0.93:1.05:1.00	5.47E+07	8.86E+07	0.62:1.00	407701	PREDICTED: E3 ubiquitin-protein ligase UPL2-like [Vitis vinifera]
2747	14746	gi|224070843	20.74	8	1	1	1.77E+07	1.25E+07	1.10E+07	2.55E+07	2.31E+07	2.01E+07	0.88:0.62:0.55:1.27:1.15:1.00	1.37E+07	2.29E+07	0.60:1.00	19159	glutaredoxin family protein [Populus trichocarpa]
2621	3035	gi|359491781	20.62	6	1	1	4.78E+07	4.03E+07	3.36E+07	6.00E+07	6.77E+07	5.64E+07	0.85:0.72:0.60:1.06:1.20:1.00	4.06E+07	6.14E+07	0.66:1.00	18046	PREDICTED: miraculin-like partial [Vitis vinifera]
1368	1841	gi|566185810	20.18	2	1	1	0	0	3.26E+06	3.83E+07	2.10E+07	1.13E+07	0:0:0.29:3.38:1.86:1.00	1.09E+06	2.36E+07	0.05:1.00	94037	beta-galactosidase family protein [Populus trichocarpa]

Table S8 The *Vitis vinifera* orthologs of differentially expreesed proteins between HY1 and CK1

GI of identified differentially expressed proteins	Vitis vinifera orthologs
gi|359486799|ref|XP_002283532.2|	tr|F6HNX5|F6HNX5_VITVI
gi|731393844|ref|XP_010651616.1|	tr|D7T1K2|D7T1K2_VITVI
gi|225468576|ref|XP_002263337.1|	tr|D7T1R6|D7T1R6_VITVI
gi|225433510|ref|XP_002266276.1|	tr|F6GWF3|F6GWF3_VITVI
gi|731406213|ref|XP_010656084.1|	tr|F6HGZ9|F6HGZ9_VITVI
gi|359492254|ref|XP_002274366.2|	tr|E0CQ75|E0CQ75_VITVI
gi|225457971|ref|XP_002275563.1|	tr|A5ANR7|A5ANR7_VITVI
gi|359483839|ref|XP_003633024.1|	tr|A5BAI4|A5BAI4_VITVI
gi|224053300|ref|XP_002297752.1|	tr|A5C8S5|A5C8S5_VITVI
gi|225437428|ref|XP_002271896.1|	tr|A5C6H7|A5C6H7_VITVI
gi|552540866|ref|YP_008592859.1|	sp|Q0ZJ07|CYF_VITVI
gi|671743230|ref|YP_009050761.1|	sp|Q0ZJ21|PSAB_VITVI
gi|671743315|ref|YP_009050845.1|	sp|Q0ZJ24|PSBC_VITVI
gi|566176273|ref|XP_006381564.1|	tr|F6HGZ9|F6HGZ9_VITVI
gi|224064707|ref|XP_002301540.1|	tr|D7SHU4|D7SHU4_VITVI
gi|566184073|ref|XP_002311618.2|	tr|D7TDX6|D7TDX6_VITVI
gi|224084209|ref|XP_002307234.1|	tr|F6I229|F6I229_VITVI
gi|359497883|ref|XP_003635681.1|	tr|F6HRP4|F6HRP4_VITVI
gi|225423961|ref|XP_002282429.1|	tr|F6HEX2|F6HEX2_VITVI
gi|731430736|ref|XP_010665150.1|	tr|F6GY49|F6GY49_VITVI
gi|359475502|ref|XP_003631692.1|	tr|A5BEJ8|A5BEJ8_VITVI
gi|224077927|ref|XP_002305462.1|	tr|F6GWQ0|F6GWQ0_VITVI
gi|224104269|ref|XP_002313378.1|	tr|D7SJX8|D7SJX8_VITVI
gi|731388721|ref|XP_010649714.1|	tr|A5BM68|A5BM68_VITVI
gi|225451279|ref|XP_002277791.1|	tr|F6HTT2|F6HTT2_VITVI
gi|671743459|ref|YP_009050989.1|	tr|B6VJW4|B6VJW4_VITVI
gi|566197644|ref|XP_002318702.2|	tr|D7SHA2|D7SHA2_VITVI
gi|731395934|ref|XP_010652337.1|	tr|D7U2U2|D7U2U2_VITVI
gi|225432758|ref|XP_002283142.1|	tr|D7T724|D7T724_VITVI
gi|224090212|ref|XP_002308954.1|	tr|A5BIQ8|A5BIQ8_VITVI
gi|225447576|ref|XP_002271687.1|	tr|A5ASG6|A5ASG6_VITVI
gi|225449262|ref|XP_002280581.1|	tr|F6HNN4|F6HNN4_VITVI
gi|359480225|ref|XP_002273421.2|	tr|D7U2S0|D7U2S0_VITVI
gi|225455513|ref|XP_002265347.1|	tr|A5AJ83|A5AJ83_VITVI
gi|225443760|ref|XP_002270266.1|	tr|F6HMA9|F6HMA9_VITVI
gi|566170863|ref|XP_006383122.1|	tr|A5B4J3|A5B4J3_VITVI

gi|566158065|ref|XP_002301277.2|	tr|D7U7W7|D7U7W7_VITVI
gi|225459564|ref|XP_002285857.1|	tr|E0CR63|E0CR63_VITVI
gi|225429209|ref|XP_002272116.1|	tr|D7T3Q1|D7T3Q1_VITVI
gi|225462285|ref|XP_002265046.1|	tr|F6H9U0|F6H9U0_VITVI
gi|731421236|ref|XP_010661662.1|	tr|D7U7W7|D7U7W7_VITVI
gi|566165056|ref|XP_002305754.2|	tr|F6HM78|F6HM78_VITVI
gi|566171771|ref|XP_002306586.2|	tr|E0CRH7|E0CRH7_VITVI
gi|224066545|ref|XP_002302129.1|	tr|D7U865|D7U865_VITVI
gi|566151109|ref|XP_002298397.2|	tr|A5BIQ8|A5BIQ8_VITVI
gi|225456914|ref|XP_002281019.1|	tr|A5BE36|A5BE36_VITVI
gi|731410160|ref|XP_010657457.1|	tr|F6HHN8|F6HHN8_VITVI
gi|224095668|ref|XP_002310430.1|	tr|A5B8T3|A5B8T3_VITVI
gi|225434133|ref|XP_002277485.1|	tr|D7T2G6|D7T2G6_VITVI
gi|566202763|ref|XP_006375250.1|	tr|F6HD52|F6HD52_VITVI
gi|225429772|ref|XP_002282619.1|	tr|F6H3A8|F6H3A8_VITVI
gi|225428011|ref|XP_002278341.1|	tr|D7U561|D7U561_VITVI
gi|224124892|ref|XP_002319448.1|	tr|Q0MYQ7|Q0MYQ7_VITVI
gi|566162704|ref|XP_006385815.1|	tr|F6H2R3|F6H2R3_VITVI
gi|731398611|ref|XP_010653323.1|	tr|D7SRG7|D7SRG7_VITVI
gi|224104103|ref|XP_002313320.1|	tr|A5AUZ0|A5AUZ0_VITVI
gi|566146555|ref|XP_006368290.1|	tr|D7TF52|D7TF52_VITVI
gi|225436257|ref|XP_002273201.1|	tr|F6I5I9|F6I5I9_VITVI
gi|225429975|ref|XP_002283985.1|	tr|F6H3E6|F6H3E6_VITVI
gi|731437331|ref|XP_002272813.2|	tr|A5BKX2|A5BKX2_VITVI
gi|225459318|ref|XP_002285795.1|	tr|A5BTZ8|A5BTZ8_VITVI
gi|225435203|ref|XP_002284863.1|	tr|F6HNK2|F6HNK2_VITVI
gi|731436245|ref|XP_010645817.1|	tr|F6HJD7|F6HJD7_VITVI
gi|225469850|ref|XP_002266051.1|	tr|D7TGB6|D7TGB6_VITVI
gi|731416683|ref|XP_010659986.1|	tr|F6HTY5|F6HTY5_VITVI
gi|566175322|ref|XP_002308181.2|	tr|F6HL41|F6HL41_VITVI
gi|224092348|ref|XP_002309570.1|	tr|F6HYE7|F6HYE7_VITVI
gi|225432586|ref|XP_002281350.1|	tr|A5ASC2|A5ASC2_VITVI
gi|225457389|ref|XP_002284856.1|	tr|A5AEI0|A5AEI0_VITVI
gi|359497056|ref|XP_002263033.2|	tr|F6HD61|F6HD61_VITVI
gi|225425513|ref|XP_002266183.1|	tr|D7TDE2|D7TDE2_VITVI
gi|526117629|ref|NP_001268074.1|	tr|F6GXX3|F6GXX3_VITVI
gi|731408416|ref|XP_010656845.1|	tr|F6GXH4|F6GXH4_VITVI
gi|225464489|ref|XP_002268754.1|	tr|E0CV06|E0CV06_VITVI

gi|225427973|ref|XP_002277628.1|	tr|F6I111|F6I111_VITVI
gi|224098816|ref|XP_002311278.1|	tr|F6H9S5|F6H9S5_VITVI
gi|566187811|ref|XP_002313723.2|	tr|F6I111|F6I111_VITVI
gi|566256321|ref|XP_006388123.1|	tr|A5C101|A5C101_VITVI
gi|566161648|ref|XP_006385625.1|	tr|D7TTY4|D7TTY4_VITVI
gi|224109620|ref|XP_002315258.1|	tr|D7T475|D7T475_VITVI
gi|566212112|ref|XP_006373055.1|	tr|C4P7W1|C4P7W1_VITVI
gi|566197051|ref|XP_006376775.1|	tr|D7TE76|D7TE76_VITVI
gi|566203933|ref|XP_002321184.2|	tr|A5CBL0|A5CBL0_VITVI
gi|224066487|ref|XP_002302108.1|	tr|F6HI46|F6HI46_VITVI
gi|225447745|ref|XP_002263201.1|	tr|A5ASW8|A5ASW8_VITVI
gi|225443988|ref|XP_002280687.1|	tr|F6HLW0|F6HLW0_VITVI
gi|566255736|ref|XP_006387910.1|	tr|F6HZ27|F6HZ27_VITVI
gi|225424750|ref|XP_002267612.1|	tr|D7TNM7|D7TNM7_VITVI
gi|731438465|ref|XP_010647190.1|	tr|A5B9M3|A5B9M3_VITVI
gi|225463990|ref|XP_002263795.1|	tr|D7TX69|D7TX69_VITVI
gi|359494267|ref|XP_002266007.2|	tr|F6HR77|F6HR77_VITVI
gi|225453275|ref|XP_002267346.1|	tr|F6GVE3|F6GVE3_VITVI
gi|224064488|ref|XP_002301501.1|	tr|A5BIW0|A5BIW0_VITVI

Table S9 The *Vitis vinifera* orthologs of differentially expreesed proteins between HY2 and CK2

GI of identified differentially expressed proteins	Vitis vinifera orthologs
gi|669100005|ref|YP_009047940.1|	sp|P56648|RBL_VITVI
gi|669100005|ref|YP_009047940.1|	tr|A5BTM9|A5BTM9_VITVI
gi|669100005|ref|YP_009047940.1|	tr|D7U748|D7U748_VITVI
gi|225462164|ref|XP_002266780.1|	tr|F6H4T7|F6H4T7_VITVI
gi|224094244|ref|XP_002310106.1|	tr|F6H4T7|F6H4T7_VITVI
gi|225426230|ref|XP_002263323.1|	tr|F6HU55|F6HU55_VITVI
gi|566225321|ref|XP_006371013.1|	tr|F6I0I5|F6I0I5_VITVI
gi|225443399|ref|XP_002267279.1|	tr|D7SS06|D7SS06_VITVI
gi|566196410|ref|XP_002317789.2|	tr|F6HU55|F6HU55_VITVI
gi|224365668|ref|YP_002608395.1|	tr|B6VJY3|B6VJY3_VITVI
gi|566146990|ref|XP_006368489.1|	tr|F6HYK6|F6HYK6_VITVI
gi|225456079|ref|XP_002280824.1|	tr|F6GTT2|F6GTT2_VITVI
gi|224073124|ref|XP_002303983.1|	tr|D7SLM9|D7SLM9_VITVI
gi|224065861|ref|XP_002301975.1|	tr|A5AKD8|A5AKD8_VITVI
gi|224119200|ref|XP_002318013.1|	tr|F6GZQ1|F6GZQ1_VITVI
gi|542688129|ref|YP_008520146.1|	sp|Q0ZJ13|ATPB_VITVI
gi|566150406|ref|XP_006369390.1|	tr|D7T1K2|D7T1K2_VITVI
gi|359490179|ref|XP_002266494.2|	tr|F6HAM6|F6HAM6_VITVI
gi|224063263|ref|XP_002301067.1|	tr|F6H0X2|F6H0X2_VITVI
gi|526117645|ref|NP_001268078.1|	tr|F6HKY8|F6HKY8_VITVI
gi|359496771|ref|XP_002272173.2|	tr|F6HCT7|F6HCT7_VITVI
gi|731424802|ref|XP_010663020.1|	tr|F6GT15|F6GT15_VITVI
gi|225433375|ref|XP_002285608.1|	tr|D7TS57|D7TS57_VITVI
gi|731419756|ref|XP_010661131.1|	tr|F6HF82|F6HF82_VITVI
gi|566204141|ref|XP_002321065.2|	tr|F6H409|F6H409_VITVI
gi|225431871|ref|XP_002275593.1|	tr|D7SY66|D7SY66_VITVI
gi|224109060|ref|XP_002315066.1|	tr|A5CAF8|A5CAF8_VITVI
gi|359478916|ref|XP_003632187.1|	tr|D7SKB2|D7SKB2_VITVI
gi|225427768|ref|XP_002267726.1|	tr|A5BDH7|A5BDH7_VITVI
gi|671743315|ref|YP_009050845.1|	sp|Q0ZJ24|PSBC_VITVI

gi|225430619|ref|XP_002266736.1|	tr|D7TDS1|D7TDS1_VITVI
gi|225427917|ref|XP_002274871.1|	tr|F6I134|F6I134_VITVI
gi|224109788|ref|XP_002315310.1|	tr|F6HNF4|F6HNF4_VITVI
gi|225457957|ref|XP_002274869.1|	tr|A5AKD8|A5AKD8_VITVI
gi|542688125|ref|YP_008520165.1|	sp|Q0ZIZ3|PSBB_VITVI
gi|224143423|ref|XP_002324951.1|	tr|A5AHP0|A5AHP0_VITVI
gi|566210462|ref|XP_006372317.1|	tr|F6I5I7|F6I5I7_VITVI
gi|225439231|ref|XP_002271046.1|	tr|F6HMP3|F6HMP3_VITVI
gi|225453246|ref|XP_002265511.1|	tr|D7SM53|D7SM53_VITVI
gi|671743230|ref|YP_009050761.1|	sp|Q0ZJ21|PSAB_VITVI
gi|552540866|ref|YP_008592859.1|	sp|Q0ZJ07|CYF_VITVI
gi|359481634|ref|XP_003632650.1|	tr|D7TH48|D7TH48_VITVI
gi|224083020|ref|XP_002306932.1|	tr|A5AKD8|A5AKD8_VITVI
gi|552541026|ref|YP_008593111.1|	sp|Q0ZJ20|PSAA_VITVI
gi|552540956|ref|YP_008592913.1|	sp|Q0ZJ40|PSBA_VITVI
gi|566210666|ref|XP_006372413.1|	tr|F6I0H8|F6I0H8_VITVI
gi|225430366|ref|XP_002282880.1|	tr|D7SUY2|D7SUY2_VITVI
gi|566206623|ref|XP_002321583.2|	tr|D7SHU4|D7SHU4_VITVI
gi|225457219|ref|XP_002284053.1|	tr|D7SI12|D7SI12_VITVI
gi|225447725|ref|XP_002277666.1|	tr|F6HWL2|F6HWL2_VITVI
gi|224135449|ref|XP_002322076.1|	tr|F6GTS3|F6GTS3_VITVI
gi|566167040|ref|XP_002306169.2|	tr|F6I134|F6I134_VITVI
gi|566146555|ref|XP_006368290.1|	tr|D7TF52|D7TF52_VITVI
gi|225426753|ref|XP_002275872.1|	tr|F6HUI4|F6HUI4_VITVI
gi|552540940|ref|YP_008592942.1|	sp|Q0ZJ11|ACCD_VITVI
gi|225443760|ref|XP_002270266.1|	tr|F6HMA9|F6HMA9_VITVI
gi|225444472|ref|XP_002272701.1|	tr|F6H7I9|F6H7I9_VITVI
gi|225432012|ref|XP_002279647.1|	tr|F6H710|F6H710_VITVI
gi|731404671|ref|XP_010655514.1|	tr|D7TJL0|D7TJL0_VITVI
gi|225441754|ref|XP_002283367.1|	tr|D7TI00|D7TI00_VITVI
gi|526118026|ref|NP_001268176.1|	tr|D0VBC7|D0VBC7_VITVI
gi|225466690|ref|XP_002263049.1|	tr|D7TMH3|D7TMH3_VITVI

gi|225465837|ref|XP_002264626.1|	tr|F6HVD5|F6HVD5_VITVI
gi|224114720|ref|XP_002316838.1|	tr|F6HM73|F6HM73_VITVI
gi|225439064|ref|XP_002266205.1|	tr|C5DB50|C5DB50_VITVI
gi|566192956|ref|XP_002317199.2|	tr|F6HTQ1|F6HTQ1_VITVI
gi|225455784|ref|XP_002274334.1|	tr|F6GTG3|F6GTG3_VITVI
gi|225461156|ref|XP_002282795.1|	tr|E0CT43|E0CT43_VITVI
gi|731401656|ref|XP_010654370.1|	tr|F6HXL1|F6HXL1_VITVI
gi|566213188|ref|XP_006373422.1|	tr|F6GT74|F6GT74_VITVI
gi|224063997|ref|XP_002301340.1|	tr|D7UC26|D7UC26_VITVI
gi|731387895|ref|XP_010649415.1|	tr|F6I0H8|F6I0H8_VITVI
gi|566254838|ref|XP_006387604.1|	tr|A5AW13|A5AW13_VITVI
gi|731376167|ref|XP_010655500.1|	tr|D7TAR8|D7TAR8_VITVI
gi|526117441|ref|NP_001268023.1|	tr|D7SLA9|D7SLA9_VITVI
gi|224129596|ref|XP_002320625.1|	tr|A5BF93|A5BF93_VITVI
gi|224137032|ref|XP_002322477.1|	tr|F6H6M8|F6H6M8_VITVI
gi|566242200|ref|XP_006371499.1|	tr|F6HAV9|F6HAV9_VITVI
gi|566149424|ref|XP_002298224.2|	tr|F6HXU4|F6HXU4_VITVI
gi|566176147|ref|XP_006381503.1|	tr|F6GUN5|F6GUN5_VITVI
gi|225425838|ref|XP_002266762.1|	tr|F6HG55|F6HG55_VITVI
gi|225430650|ref|XP_002269441.1|	tr|F6HI27|F6HI27_VITVI
gi|225459292|ref|XP_002285787.1|	tr|E0CRL1|E0CRL1_VITVI
gi|568244554|ref|YP_008963301.1|	sp|Q0ZJ25|PSBD_VITVI
gi|224114826|ref|XP_002316867.1|	tr|F6H4Z9|F6H4Z9_VITVI
gi|225434133|ref|XP_002277485.1|	tr|D7T2G6|D7T2G6_VITVI
gi|225438781|ref|XP_002283105.1|	tr|D7SVT7|D7SVT7_VITVI
gi|526117723|ref|NP_001268098.1|	tr|F6I0K4|F6I0K4_VITVI
gi|359485890|ref|XP_002264183.2|	tr|F6I6R4|F6I6R4_VITVI
gi|359494079|ref|XP_002277391.2|	tr|F6HET4|F6HET4_VITVI
gi|566178300|ref|XP_002308647.2|	tr|F6H3T7|F6H3T7_VITVI
gi|566184073|ref|XP_002311618.2|	tr|D7TDX6|D7TDX6_VITVI
gi|731440185|ref|XP_010646096.1|	tr|F6I1X5|F6I1X5_VITVI
gi|225457458|ref|XP_002265041.1|	tr|D7TXR6|D7TXR6_VITVI

gi|566207041|ref|XP_002321728.2|	tr|A5BEM6|A5BEM6_VITVI
gi|566204499|ref|XP_002320494.2|	tr|D7TEF8|D7TEF8_VITVI
gi|224129210|ref|XP_002320528.1|	tr|F6HHQ7|F6HHQ7_VITVI
gi|224108938|ref|XP_002315023.1|	tr|A5BZP4|A5BZP4_VITVI
gi|224104631|ref|XP_002313506.1|	tr|D7TQA5|D7TQA5_VITVI
gi|224120174|ref|XP_002318262.1|	tr|D7T1T9|D7T1T9_VITVI
gi|731394579|ref|XP_010651886.1|	tr|F6HHG2|F6HHG2_VITVI
gi|731439997|ref|XP_002268808.3|	tr|F6HCV4|F6HCV4_VITVI
gi|225464906|ref|XP_002274381.1|	tr|D7TK38|D7TK38_VITVI
gi|566149444|ref|XP_002299720.2|	tr|D7TCD6|D7TCD6_VITVI
gi|225455934|ref|XP_002276967.1|	tr|A5C718|A5C718_VITVI
gi|731426684|ref|XP_010663697.1|	tr|F6GSN7|F6GSN7_VITVI
gi|566175251|ref|XP_002309064.2|	tr|F6HL96|F6HL96_VITVI
gi|225460716|ref|XP_002271286.1|	tr|F6GY10|F6GY10_VITVI
gi|566195934|ref|XP_006377983.1|	tr|D7UAQ2|D7UAQ2_VITVI
gi|224059670|ref|XP_002299962.1|	tr|F6HB97|F6HB97_VITVI
gi|225444649|ref|XP_002276749.1|	tr|Q6B4V4|Q6B4V4_VITVI
gi|225429209|ref|XP_002272116.1|	tr|D7T3Q1|D7T3Q1_VITVI
gi|731428049|ref|XP_010664203.1|	tr|A5B0X9|A5B0X9_VITVI
gi|566183513|ref|XP_002312387.2|	tr|E0CQ39|E0CQ39_VITVI
gi|731430037|ref|XP_002269356.2|	tr|E0CQB7|E0CQB7_VITVI
gi|731380214|ref|XP_010662388.1|	tr|D7TN36|D7TN36_VITVI
gi|566170761|ref|XP_006383073.1|	tr|D7SNV6|D7SNV6_VITVI
gi|731371455|ref|XP_010648953.1|	tr|D7T9L8|D7T9L8_VITVI
gi|566185810|ref|XP_002314274.2|	tr|D7SKW9|D7SKW9_VITVI
gi|731403303|ref|XP_010655009.1|	tr|D7U865|D7U865_VITVI
gi|225448367|ref|XP_002268645.1|	tr|F6HC03|F6HC03_VITVI
gi|731440862|ref|XP_010646637.1|	tr|A5BD13|A5BD13_VITVI
gi|224104913|ref|XP_002313617.1|	tr|D7SW25|D7SW25_VITVI
gi|225447176|ref|XP_002276520.1|	tr|D7TEF8|D7TEF8_VITVI
gi|566170929|ref|XP_006383152.1|	tr|F6GWS4|F6GWS4_VITVI
gi|224057272|ref|XP_002299201.1|	tr|F6HVD5|F6HVD5_VITVI

gi|224131818|ref|XP_002321186.1|	tr|A5ASG6|A5ASG6_VITVI
gi|731436956|ref|XP_010647324.1|	tr|D7ST07|D7ST07_VITVI
gi|224078584|ref|XP_002305564.1|	tr|F6H7L5|F6H7L5_VITVI
gi|225455106|ref|XP_002265766.1|	tr|E0CVH6|E0CVH6_VITVI
gi|225460542|ref|XP_002277226.1|	tr|F6GYT6|F6GYT6_VITVI
gi|225434323|ref|XP_002265847.1|	tr|F6GU78|F6GU78_VITVI
gi|225453350|ref|XP_002271352.1|	tr|A5B878|A5B878_VITVI
gi|671743260|ref|YP_009050792.1|	sp|Q0ZIY9|CYB6_VITVI
gi|225434277|ref|XP_002262902.1|	tr|D7SJF5|D7SJF5_VITVI
gi|566202597|ref|XP_002320883.2|	tr|A5B878|A5B878_VITVI
gi|224073967|ref|XP_002304206.1|	tr|A5BHE6|A5BHE6_VITVI
gi|225452270|ref|XP_002270233.1|	tr|F6HUS8|F6HUS8_VITVI
gi|225447707|ref|XP_002277051.1|	tr|F6GXR1|F6GXR1_VITVI
gi|225435203|ref|XP_002284863.1|	tr|F6HNK2|F6HNK2_VITVI
gi|225451247|ref|XP_002276521.1|	tr|A5BH65|A5BH65_VITVI
gi|566160631|ref|XP_006385363.1|	tr|A5BV91|A5BV91_VITVI
gi|566174518|ref|XP_002308027.2|	tr|F6HKZ2|F6HKZ2_VITVI
gi|526118093|ref|NP_001268192.1|	tr|F6HKH2|F6HKH2_VITVI
gi|731428733|ref|XP_010664436.1|	tr|F6H151|F6H151_VITVI
gi|731389447|ref|XP_010649982.1|	tr|D7T777|D7T777_VITVI
gi|566205838|ref|XP_002322112.2|	tr|F6GSN7|F6GSN7_VITVI
gi|225435878|ref|XP_002264840.1|	tr|D7T1A7|D7T1A7_VITVI
gi|224084784|ref|XP_002307405.1|	tr|F6H0N8|F6H0N8_VITVI
gi|566168995|ref|XP_006382473.1|	tr|D7U9U6|D7U9U6_VITVI
gi|225437898|ref|XP_002266424.1|	tr|F6HZU4|F6HZU4_VITVI
gi|224078826|ref|XP_002305643.1|	tr|F6H8B4|F6H8B4_VITVI
gi|224107655|ref|XP_002314551.1|	tr|D7TAP7|D7TAP7_VITVI
gi|225465441|ref|XP_002265677.1|	tr|A5ATD8|A5ATD8_VITVI
gi|225449234|ref|XP_002280039.1|	tr|A5C3V1|A5C3V1_VITVI
gi|566183164|ref|XP_002311347.2|	tr|D7SMC3|D7SMC3_VITVI
gi|224089062|ref|XP_002308619.1|	tr|D7SKV3|D7SKV3_VITVI
gi|224109088|ref|XP_002315079.1|	tr|F6H9T6|F6H9T6_VITVI

gi|526117942|ref|NP_001268153.1|	tr|F6HLL8|F6HLL8_VITVI
gi|359475330|ref|XP_003631658.1|	tr|F6HQM5|F6HQM5_VITVI
gi|566150510|ref|XP_002299649.2|	tr|F6HAD6|F6HAD6_VITVI
gi|566167266|ref|XP_002306207.2|	tr|F6I0W9|F6I0W9_VITVI
gi|225454123|ref|XP_002269748.1|	tr|F6I6I8|F6I6I8_VITVI
gi|224107333|ref|XP_002314449.1|	tr|F6HZ31|F6HZ31_VITVI
gi|731408423|ref|XP_010656847.1|	tr|F6GXH6|F6GXH6_VITVI
gi|566210985|ref|XP_006372566.1|	tr|D7TSK7|D7TSK7_VITVI
gi|224109362|ref|XP_002315172.1|	tr|D7TMG2|D7TMG2_VITVI
gi|224124892|ref|XP_002319448.1|	tr|Q0MYQ7|Q0MYQ7_VITVI
gi|225458237|ref|XP_002282021.1|	tr|A5C3G7|A5C3G7_VITVI
gi|225440849|ref|XP_002282193.1|	tr|D7TJ87|D7TJ87_VITVI
gi|225439775|ref|XP_002275882.1|	tr|D7TQB0|D7TQB0_VITVI
gi|566239233|ref|XP_002325506.2|	tr|D7TL78|D7TL78_VITVI
gi|731426021|ref|XP_010663458.1|	tr|D7SMC3|D7SMC3_VITVI
gi|731407226|ref|XP_002285167.2|	tr|D7TC06|D7TC06_VITVI
gi|526118000|ref|NP_001267914.1|	tr|Q0MYQ7|Q0MYQ7_VITVI
gi|224084852|ref|XP_002307422.1|	tr|E0CRB0|E0CRB0_VITVI
gi|225448536|ref|XP_002276751.1|	tr|F6HC61|F6HC61_VITVI
gi|225435608|ref|XP_002285629.1|	tr|F6GUE5|F6GUE5_VITVI
gi|566185913|ref|XP_002314250.2|	tr|D7T1P6|D7T1P6_VITVI
gi|225451318|ref|XP_002273776.1|	tr|D7TUJ9|D7TUJ9_VITVI
gi|224112341|ref|XP_002316158.1|	tr|F6H9S5|F6H9S5_VITVI
gi|731384053|ref|XP_010647988.1|	tr|F6HBK3|F6HBK3_VITVI
gi|566189043|ref|XP_006378181.1|	tr|F6HPJ9|F6HPJ9_VITVI
gi|566187444|ref|XP_002313825.2|	tr|F6HQI3|F6HQI3_VITVI
gi|566184421|ref|XP_002312594.2|	tr|F6H8S2|F6H8S2_VITVI
gi|359496589|ref|XP_003635272.1|	tr|F6HWS4|F6HWS4_VITVI
gi|225457361|ref|XP_002284772.1|	tr|F6GT65|F6GT65_VITVI
gi|224070184|ref|XP_002303134.1|	tr|E0CQ38|E0CQ38_VITVI
gi|566151403|ref|XP_006369651.1|	tr|D7T1A7|D7T1A7_VITVI
gi|225435002|ref|XP_002281193.1|	tr|F6GV28|F6GV28_VITVI

gi|359491781|ref|XP_002266302.2|	tr|F6HW68|F6HW68_VITVI
gi|566152777|ref|XP_002300023.2|	tr|D7TWV6|D7TWV6_VITVI
gi|731411254|ref|XP_010657906.1|	tr|F6HJZ4|F6HJZ4_VITVI
gi|225428420|ref|XP_002283830.1|	tr|A5BRH6|A5BRH6_VITVI
gi|225432296|ref|XP_002273224.1|	tr|D7T6F0|D7T6F0_VITVI
gi|224130034|ref|XP_002320736.1|	tr|F6I327|F6I327_VITVI
gi|224137260|ref|XP_002322513.1|	tr|D7T881|D7T881_VITVI
gi|526117629|ref|NP_001268074.1|	tr|F6GXX3|F6GXX3_VITVI
gi|566186111|ref|XP_006379014.1|	tr|F6HNP2|F6HNP2_VITVI
gi|225443988|ref|XP_002280687.1|	tr|F6HLW0|F6HLW0_VITVI
gi|359494267|ref|XP_002266007.2|	tr|F6HR77|F6HR77_VITVI
gi|566167359|ref|XP_006384619.1|	tr|F6GZC5|F6GZC5_VITVI
gi|224070843|ref|XP_002303260.1|	tr|D7U0W6|D7U0W6_VITVI
gi|566210973|ref|XP_006372560.1|	tr|A5BIM9|A5BIM9_VITVI

Table S10 KEGG pathways mapped by differentially expressed proteins between CK1 and HY1

Term	Input number	Background number	Corrected P value	Pathway ID
Ribosome	16	256	0.001225464	vvi03010
Photosynthesis	9	84	0.001225464	vvi00195
Photosynthesis - antenna proteins	5	19	0.001225464	vvi00196
Glyoxylate and dicarboxylate metabolism	3	49	0.752846803	vvi00630
Limonene and pinene degradation	1	5	0.752846803	vvi00903
C5-Branched dibasic acid metabolism	1	6	0.752846803	vvi00660
Histidine metabolism	1	8	0.752846803	vvi00340
Fructose and mannose metabolism	2	38	0.752846803	vvi00051
Lysine degradation	1	11	0.752846803	vvi00310
Valine, leucine and isoleucine biosynthesis	1	11	0.752846803	vvi00290
Glycine, serine and threonine metabolism	2	45	0.752846803	vvi00260
One carbon pool by folate	1	14	0.752846803	vvi00670
Carbon fixation in photosynthetic organism	2	52	0.752846803	vvi00710
Pyruvate metabolism	2	52	0.752846803	vvi00620
Tryptophan metabolism	1	16	0.752846803	vvi00380
Cysteine and methionine metabolism	2	55	0.752846803	vvi00270
Biosynthesis of amino acids	4	146	0.752846803	vvi01230
Mismatch repair	1	20	0.752846803	vvi03430
Homologous recombination	1	21	0.752846803	vvi03440
Valine, leucine and isoleucine degradation	1	22	0.752846803	vvi00280
Carbon metabolism	4	156	0.752846803	vvi01200
Fatty acid degradation	1	24	0.752846803	vvi00071
Ascorbate and aldarate metabolism	1	24	0.752846803	vvi00053
Sulfur metabolism	1	25	0.752846803	vvi00920
DNA replication	1	26	0.752846803	vvi03030
Glycolysis / Gluconeogenesis	2	74	0.752846803	vvi00010
beta-Alanine metabolism	1	29	0.752846803	vvi00410
Aminoacyl-tRNA biosynthesis	1	31	0.752846803	vvi00970
2-Oxocarboxylic acid metabolism	1	34	0.752846803	vvi01210
Pentose phosphate pathway	1	34	0.752846803	vvi00030

Cyanoamino acid metabolism	1	34	0.752846803	vvi00460
Glycerolipid metabolism	1	36	0.752846803	vvi00561
Citrate cycle (TCA cycle)	1	36	0.752846803	vvi00020
Nucleotide excision repair	1	37	0.752846803	vvi03420
Protein processing in endoplasmic reticulu	3	143	0.752846803	vvi04141
Metabolic pathways	23	1197	0.752846803	vvi01100
Starch and sucrose metabolism	2	97	0.752846803	vvi00500
Pentose and glucuronate interconversions	1	43	0.752846803	vvi00040
Arginine and proline metabolism	1	48	0.784173544	vvi00330
Pyrimidine metabolism	1	62	0.858056868	vvi00240
Flavonoid biosynthesis	1	65	0.858056868	vvi00941
Endocytosis	1	65	0.858056868	vvi04144
Phenylalanine metabolism	1	81	0.877642959	vvi00360
Glutathione metabolism	1	82	0.877642959	vvi00480
Purine metabolism	1	83	0.877642959	vvi00230
Amino sugar and nucleotide sugar metabol	1	87	0.877642959	vvi00520
Spliceosome	1	89	0.877642959	vvi03040
Phenylpropanoid biosynthesis	1	107	0.904528066	vvi00940
Biosynthesis of secondary metabolites	9	644	0.904528066	vvi01110
Oxidative phosphorylation	1	120	0.904528066	vvi00190

Table S11 KEGG pathways mapped by differentially expressed proteins between CK2 and HY2

Term	Input number	Background number	Corrected P value	ID
Carbon metabolism	22	156	0.001632539	vvi01200
Carbon fixation in photosynthetic organisms	11	52	0.004375366	vvi00710
Photosynthesis	13	84	0.009634559	vvi00195
Glycolysis / Gluconeogenesis	12	74	0.009634559	vvi00010
Pyruvate metabolism	8	52	0.08171715	vvi00620
Propanoate metabolism	3	11	0.265191727	vvi00640
Glyoxylate and dicarboxylate metabolism	6	49	0.335115825	vvi00630
One carbon pool by folate	3	14	0.335115825	vvi00670
Tryptophan metabolism	3	16	0.39643069	vvi00380
Lysine biosynthesis	2	7	0.39928269	vvi00300
Histidine metabolism	2	8	0.437328944	vvi00340
Glycine, serine and threonine metabolism	5	45	0.437328944	vvi00260
Pentose phosphate pathway	4	34	0.450517474	vvi00030
Citrate cycle (TCA cycle)	4	36	0.450517474	vvi00020
Biosynthesis of amino acids	11	146	0.450517474	vvi01230
RNA degradation	5	51	0.450517474	vvi03018
Lysine degradation	2	11	0.450517474	vvi00310
Ascorbate and aldarate metabolism	3	24	0.456905882	vvi00053
beta-Alanine metabolism	3	29	0.622148487	vvi00410
Limonene and pinene degradation	1	5	0.788503056	vvi00903
Porphyrin and chlorophyll metabolism	3	36	0.826401313	vvi00860
Valine, leucine and isoleucine degradation	2	22	0.882598879	vvi00280
Fatty acid biosynthesis	2	24	0.882598879	vvi00061
Fatty acid degradation	2	24	0.882598879	vvi00071
Metabolic pathways	62	1197	0.882598879	vvi01100
Purine metabolism	5	83	0.882598879	vvi00230
Amino sugar and nucleotide sugar metabolism	5	87	0.882598879	vvi00520
Biotin metabolism	1	12	0.882598879	vvi00780
Alanine, aspartate and glutamate metabolism	2	33	0.882598879	vvi00250

Cysteine and methionine metabolism	3	55	0.882598879	vvi00270
Butanoate metabolism	1	15	0.882598879	vvi00650
Glycerolipid metabolism	2	36	0.882598879	vvi00561
Isoquinoline alkaloid biosynthesis	1	16	0.882598879	vvi00950
Protein processing in endoplasmic reticulum	7	143	0.882598879	vvi04141
Fructose and mannose metabolism	2	38	0.882598879	vvi00051
Galactose metabolism	2	38	0.882598879	vvi00052
Biosynthesis of secondary metabolites	31	644	0.882598879	vvi01110
Tropane, piperidine and pyridine alkaloid biosynthesis	1	17	0.882598879	vvi00960
Fatty acid metabolism	2	41	0.882598879	vvi01212
Proteasome	2	41	0.882598879	vvi03050
Ribosome	12	256	0.882598879	vvi03010
Biosynthesis of unsaturated fatty acids	1	19	0.882598879	vvi01040
Photosynthesis - antenna proteins	1	19	0.882598879	vvi00196
Inositol phosphate metabolism	1	19	0.882598879	vvi00562
Pentose and glucuronate interconversions	2	43	0.882598879	vvi00040
RNA transport	4	91	0.89913987	vvi03013
Arginine and proline metabolism	2	48	0.89913987	vvi00330
Starch and sucrose metabolism	4	97	0.89913987	vvi00500
Oxidative phosphorylation	5	120	0.89913987	vvi00190
Sulfur metabolism	1	25	0.89913987	vvi00920
mRNA surveillance pathway	2	54	0.90203373	vvi03015
Tyrosine metabolism	1	28	0.90203373	vvi00350
Phenylalanine metabolism	3	81	0.90203373	vvi00360
Phenylalanine, tyrosine and tryptophan biosynthesis	1	30	0.90203373	vvi00400
Protein export	1	32	0.90203373	vvi03060
Cyanoamino acid metabolism	1	34	0.90203373	vvi00460
Phagosome	2	63	0.90203373	vvi04145
alpha-Linolenic acid metabolism	1	37	0.911817697	vvi00592
Phenylpropanoid biosynthesis	3	107	0.950922744	vvi00940

Peroxisome	1	48	0.950922744	vvi04146
Spliceosome	2	89	0.958572493	vvi03040
Pyrimidine metabolism	1	62	0.958572493	vvi00240
Endocytosis	1	65	0.958572493	vvi04144

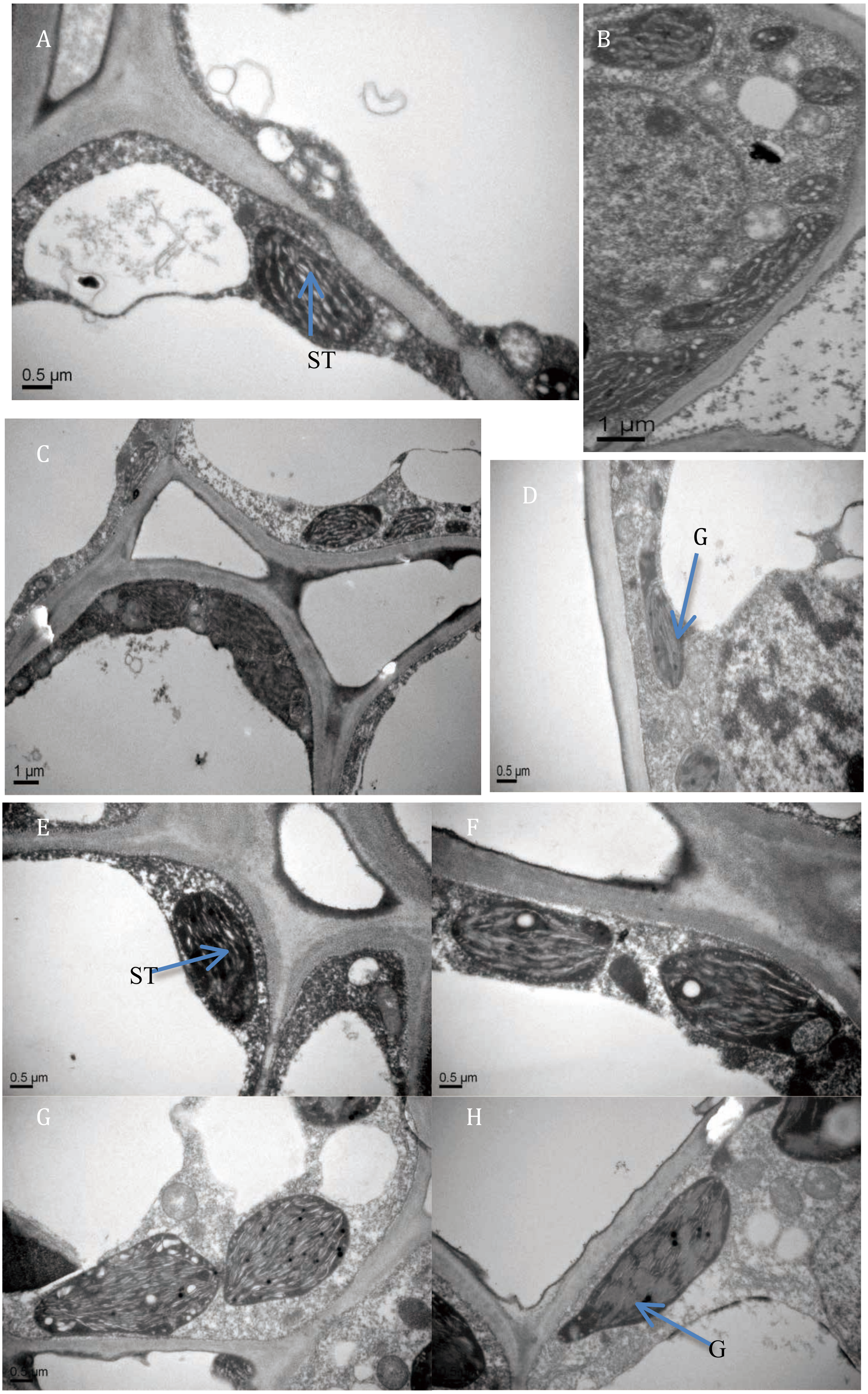

A
ST
0.5 µm
B
1 µm
C
1 µm
D
G
0.5 µm
E
ST
0.5 µm
F
0.5 µm
G
0.5 µm
H
G

Fig. S1 Chloroplast ultrastructure of two kinds of mutants with different maturity. A-D, chloroplast ultrastructure of HY1 from first leaf to forth leaf; E-H, chloroplast ultrastructure of HY2 from first leaf to forth leaf. ST refers to swelling thylakoid; G refers to grana.

gi|224064818

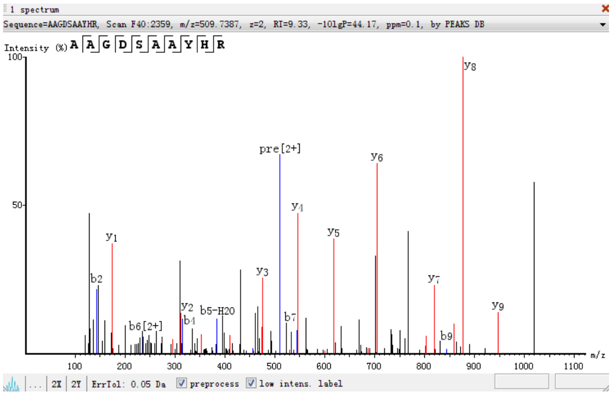

gi|224071575

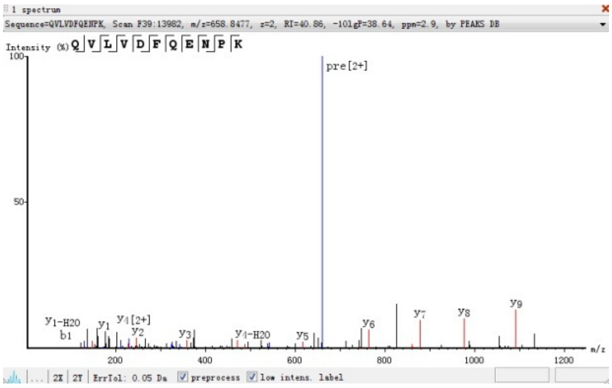

gi|224086345

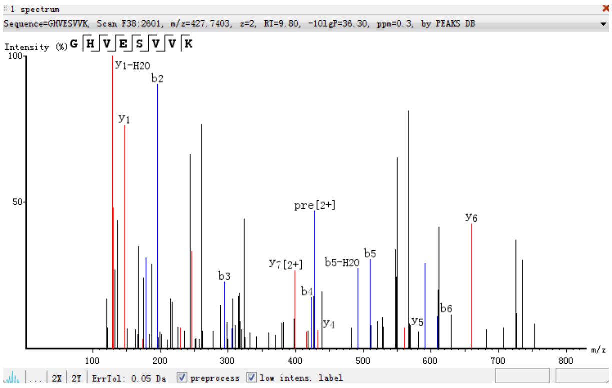

gi|224093204

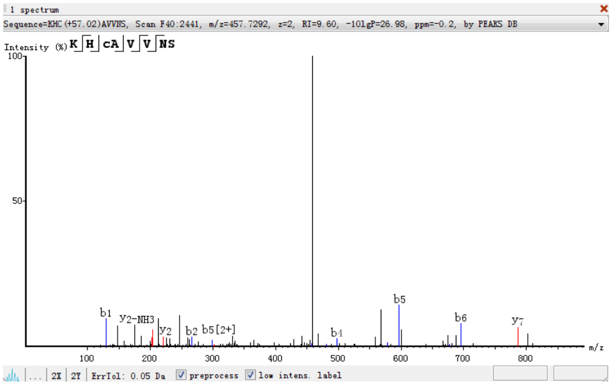

gi|224104643

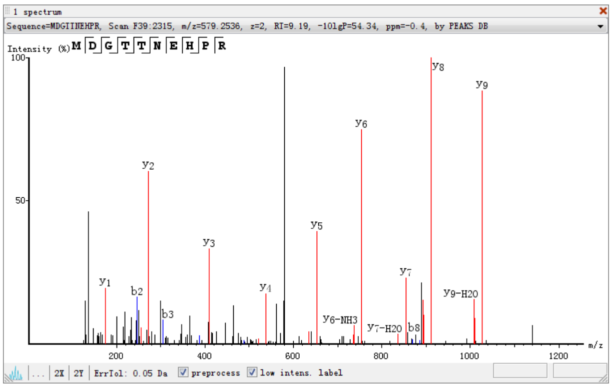

gi|224113315

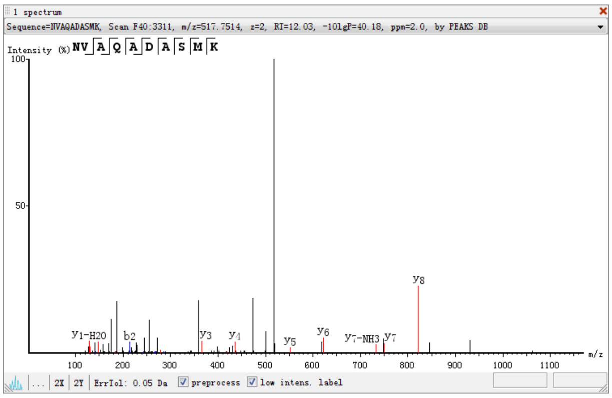

gi|224122632

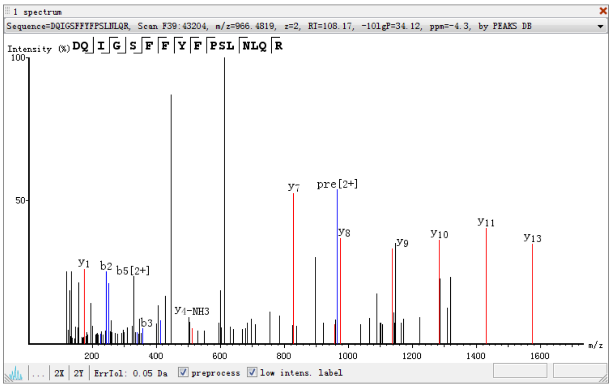

gi|224138170

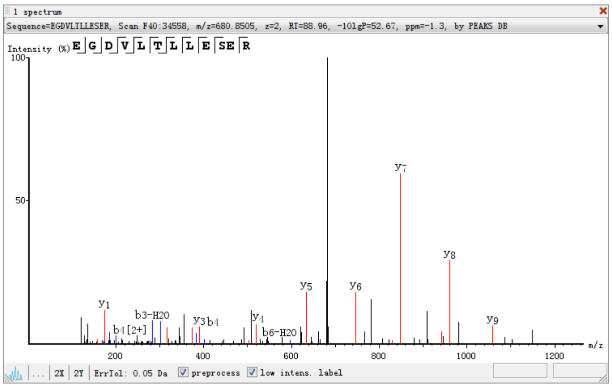

gi|224143559

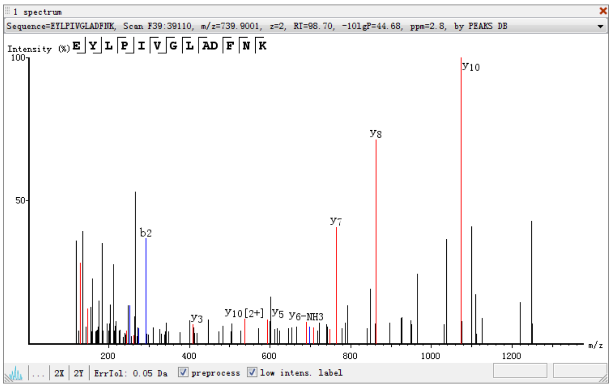

gi|224144643

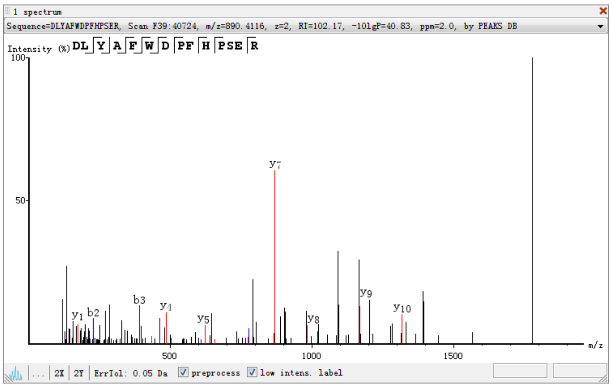

gi|224145285

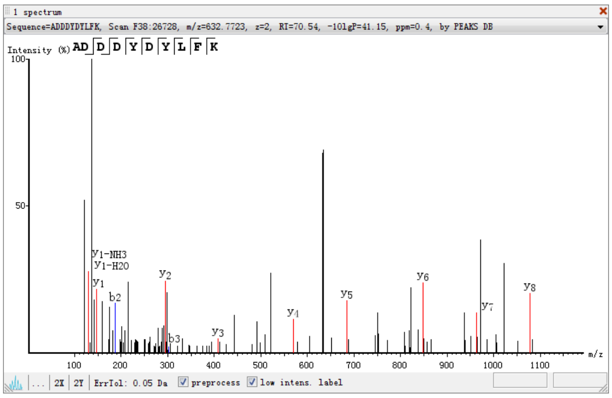

gi|224145927

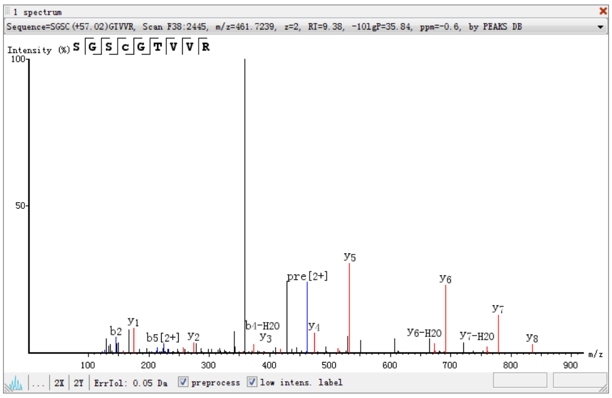

gi|225424881

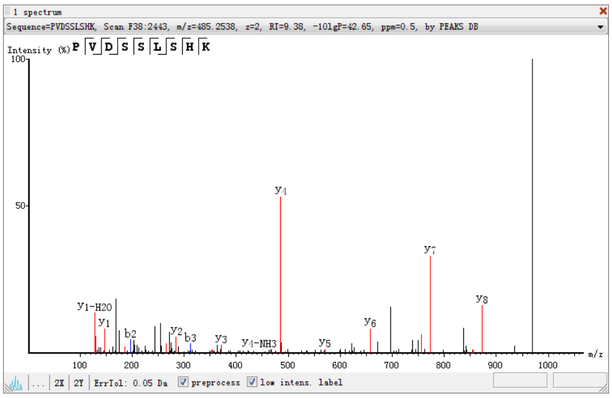

gi|225427782

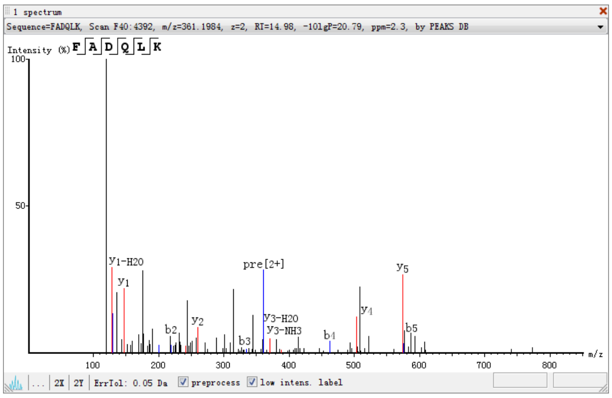

gi|225429850

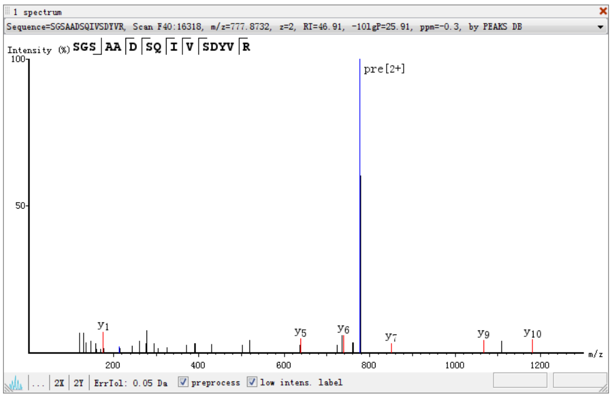

gi|224107257

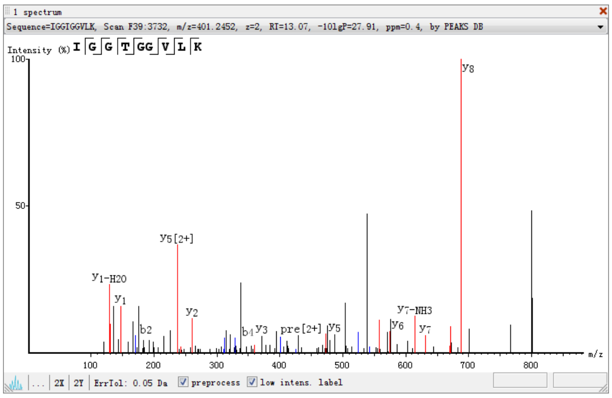

gi|225430510

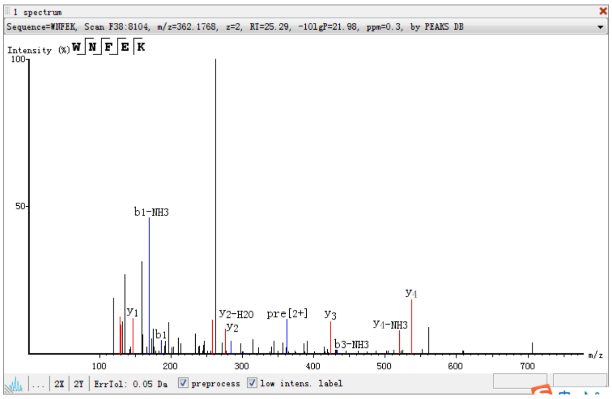

gi|225430549

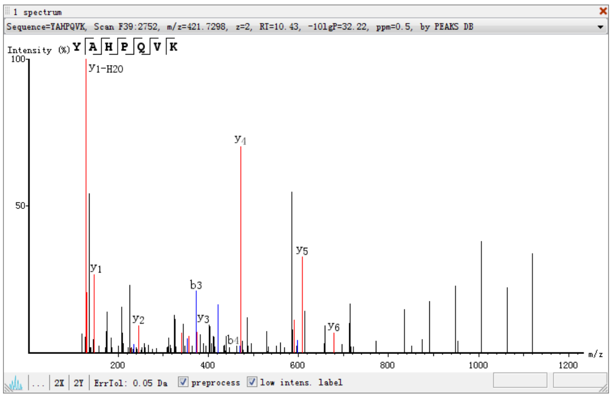

gi|225433215

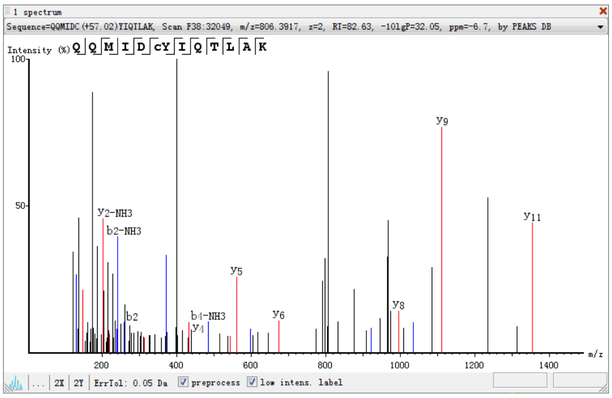

gi|225434808

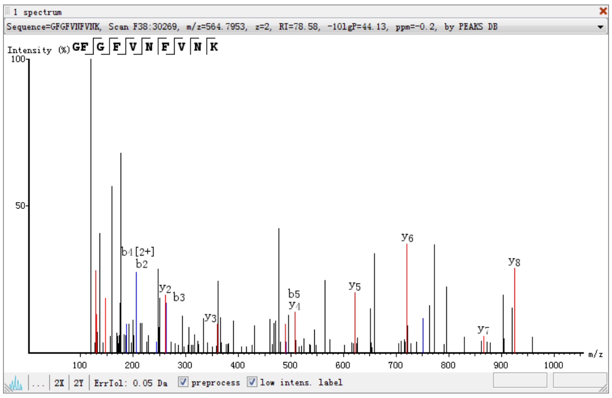

gi|225435480

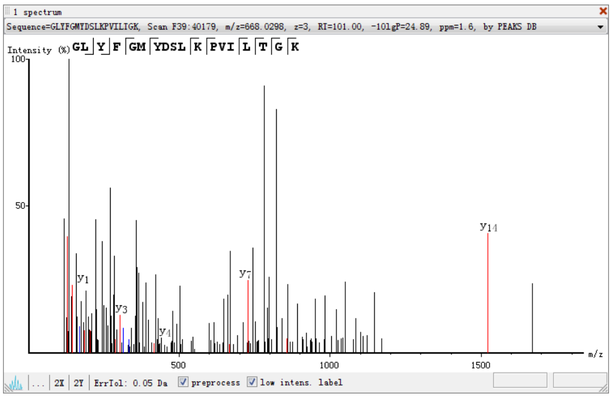

gi|225435931

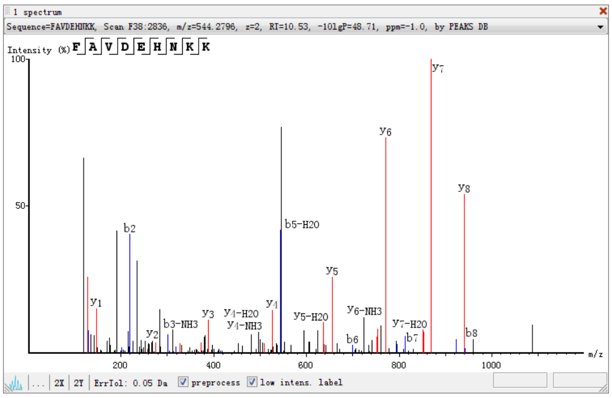

gi|225436843

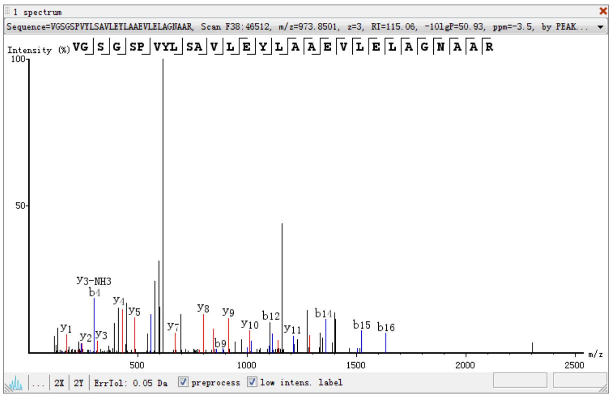

gi|225437028

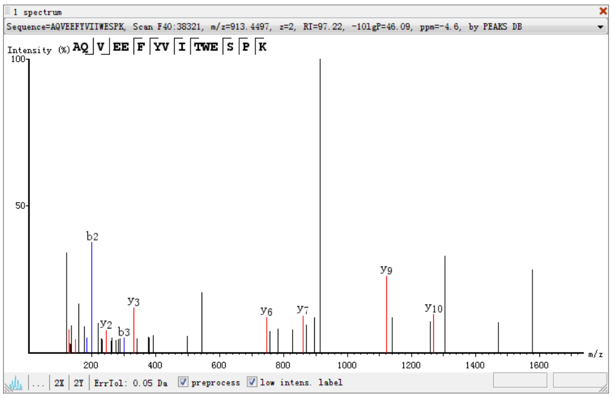

gi|225438067

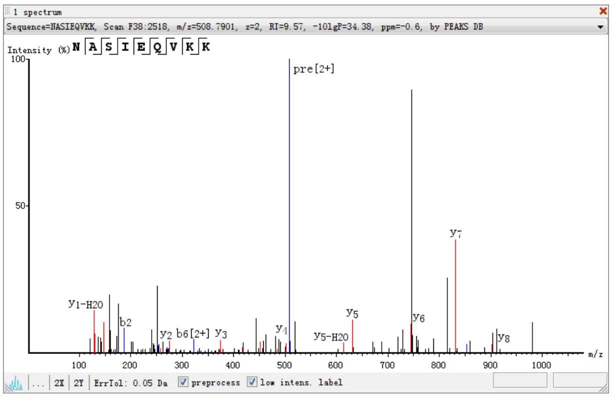

gi|225441044

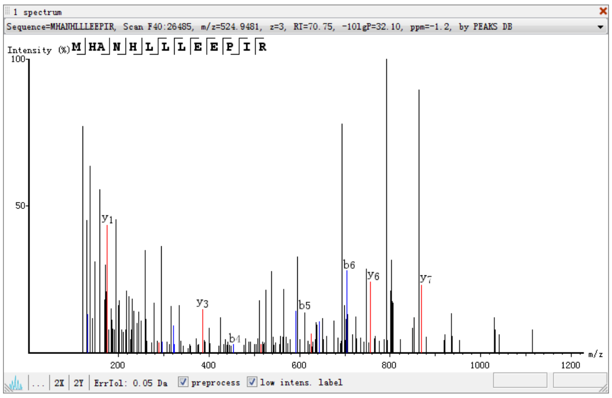

gi|225442434

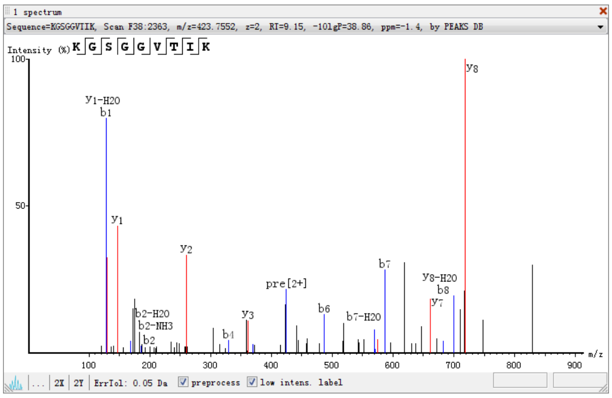

gi|225442595

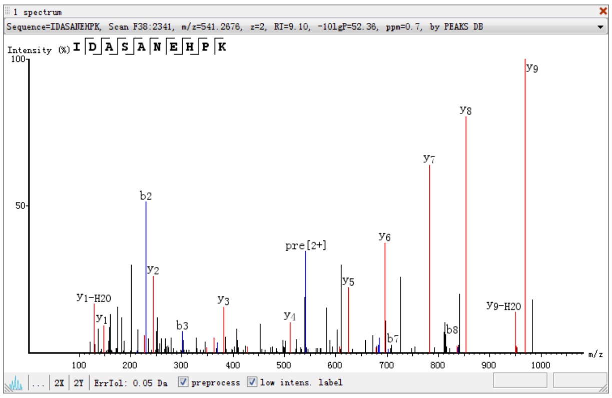

gi|225443827

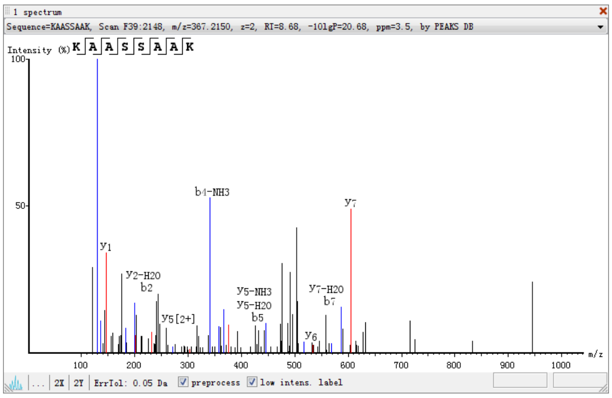

gi|225444609

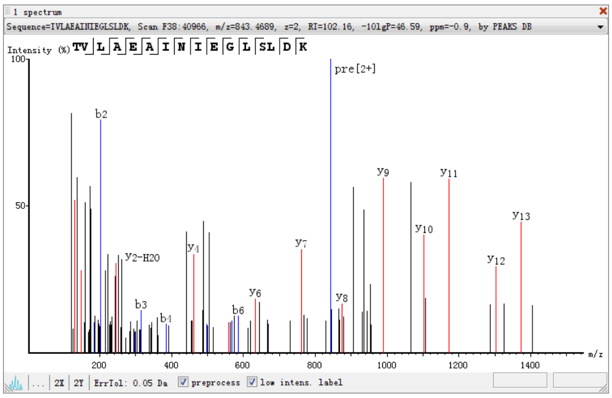

gi|225444938

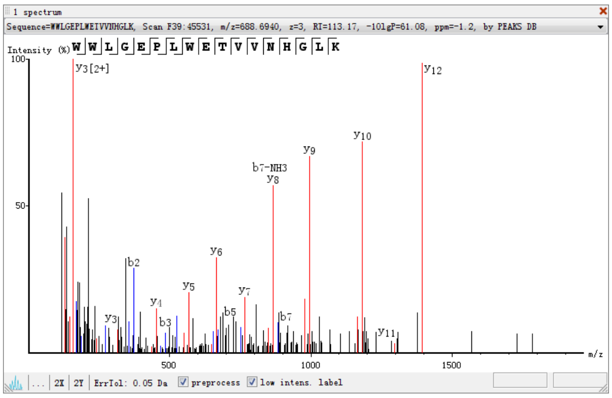

gi|225445664

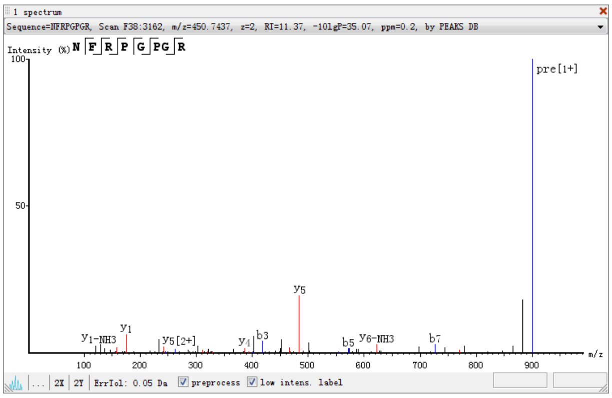

gi|225446625

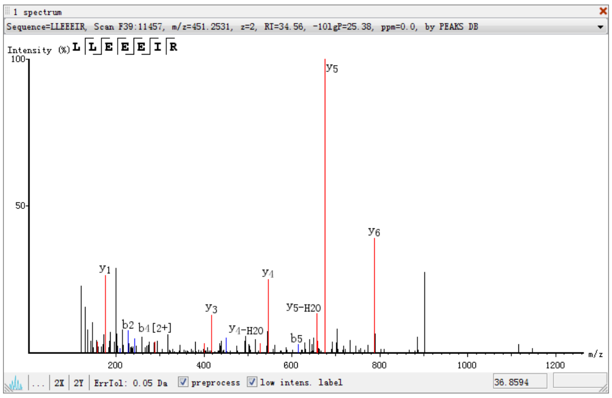

gi|225449380

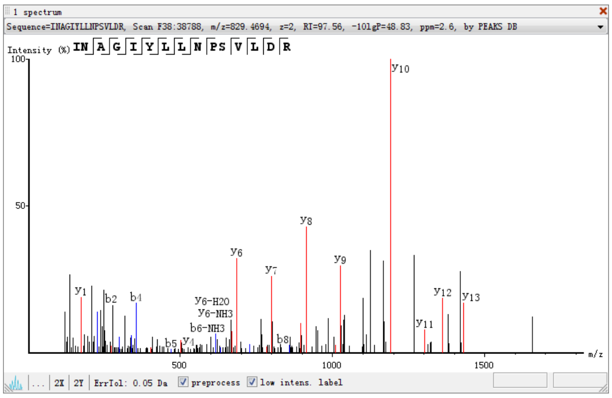

gi|225450051

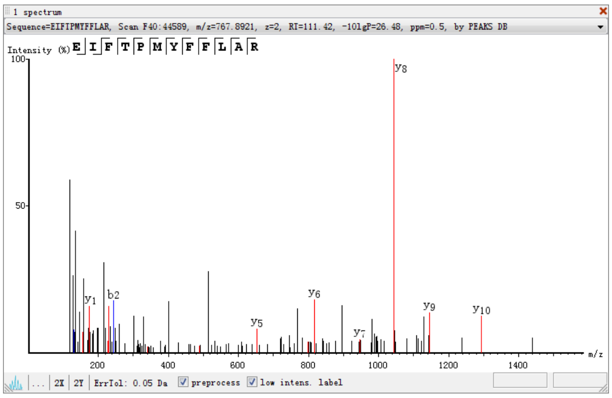

gi|225450626

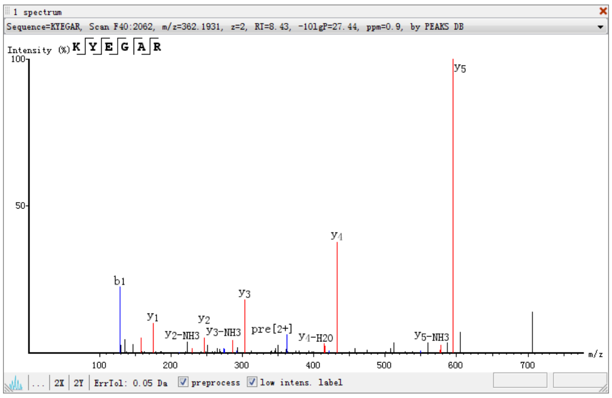

gi|225454009

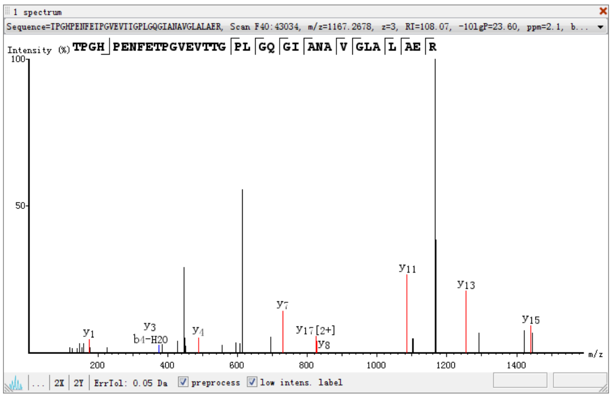

gi|225455762

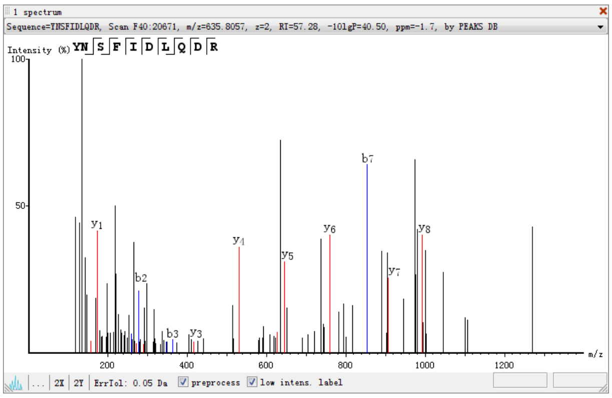

gi|225456481

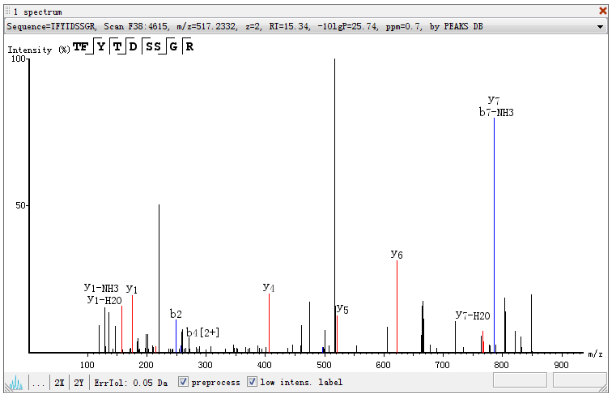

gi|225457152

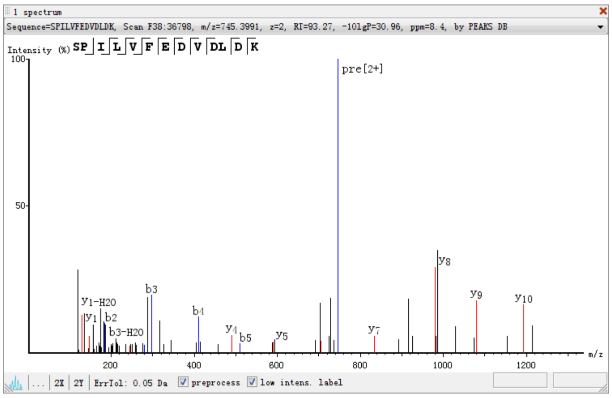

gi|225458111

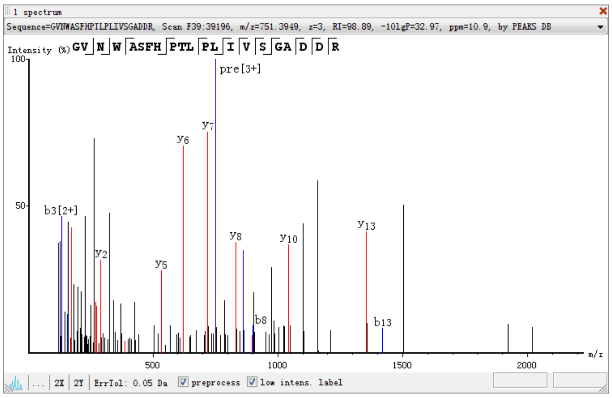

gi|225458173

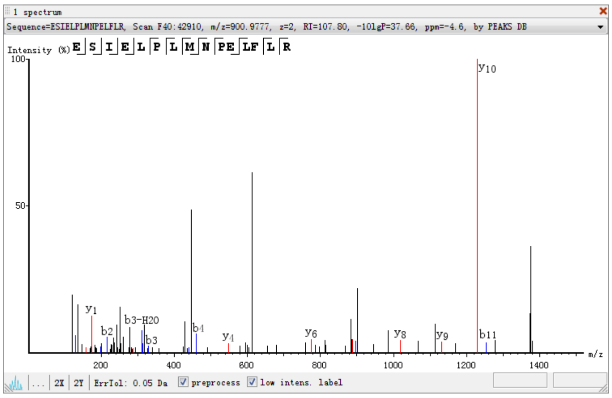

gi|225459318

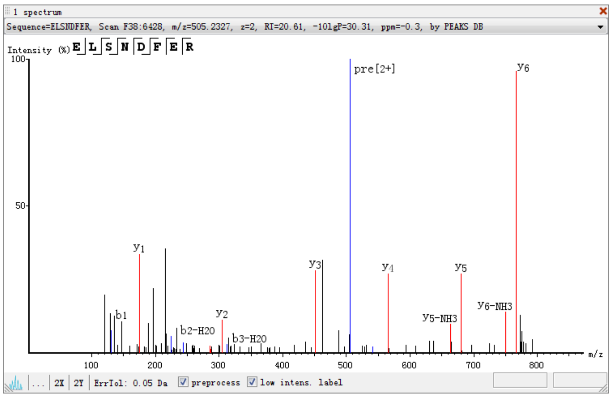

gi|225459806

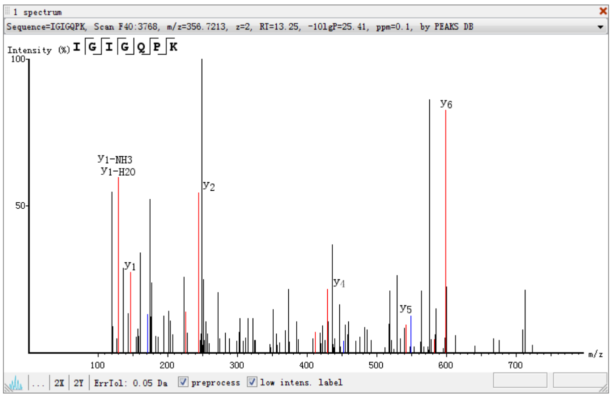

gi|225460328

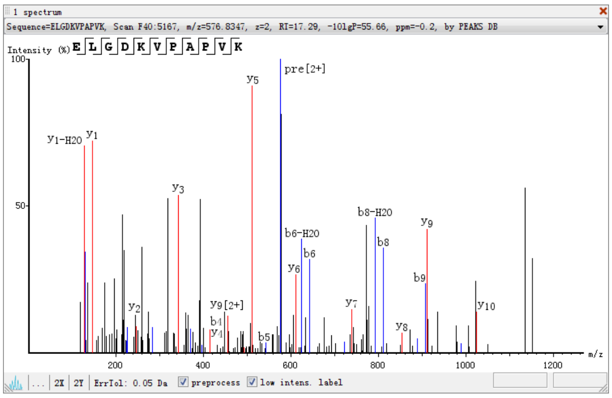

gi|225465030

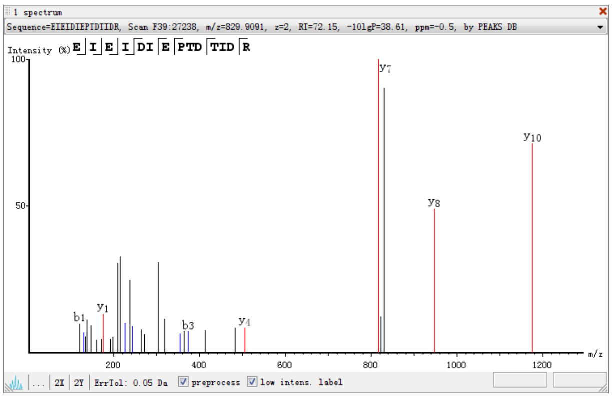

gi|225465605

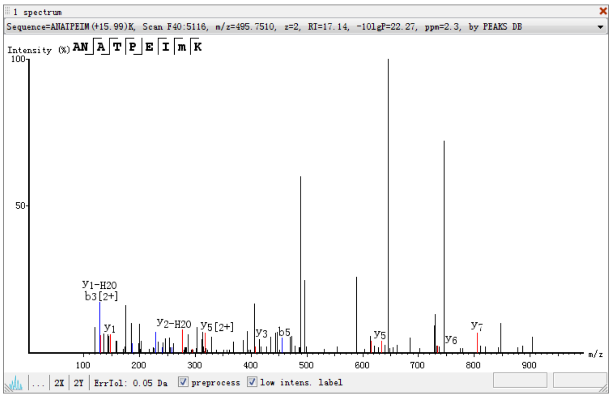

gi|225469850

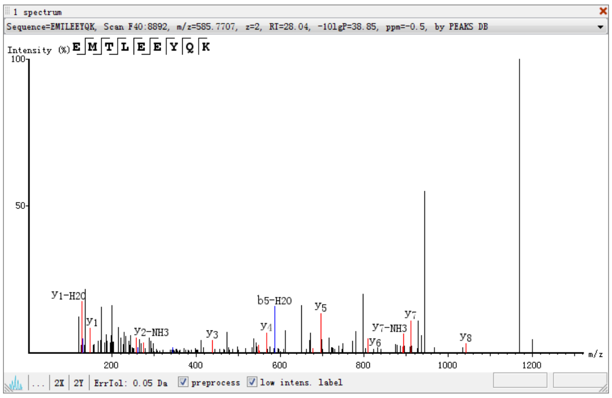

gi|359487009

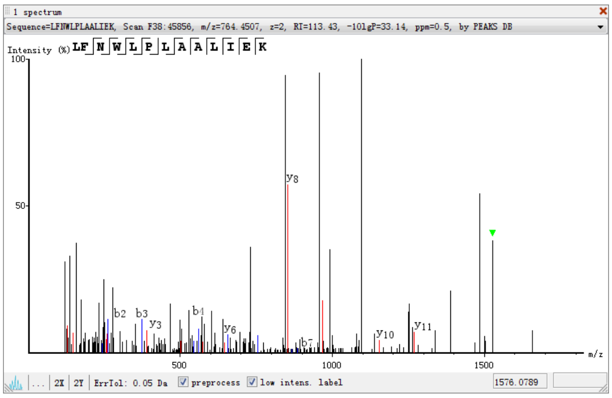

gi|359495476

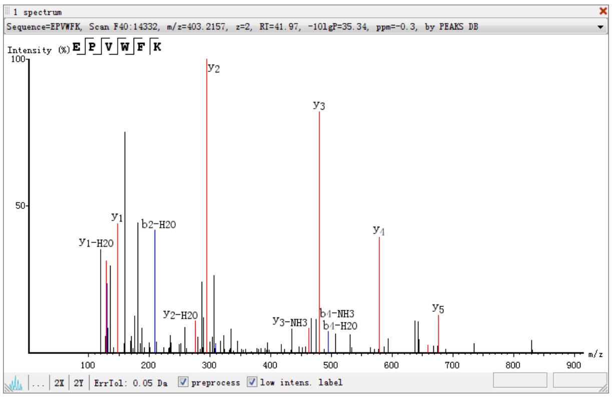

gi|526118004

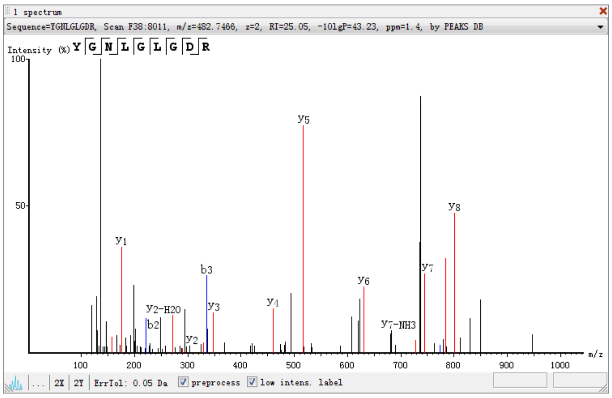

gi|552541020

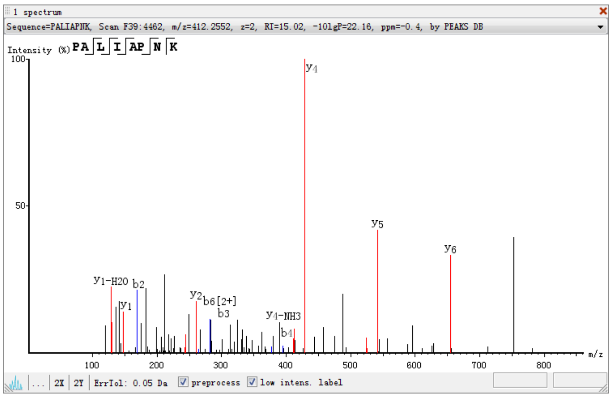

gi|566147298

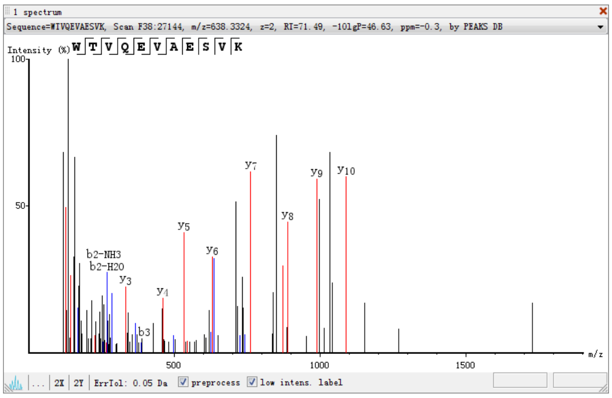

gi|566149517

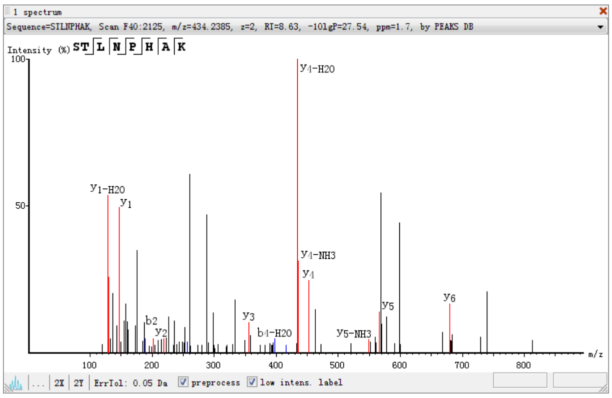

gi|566152742

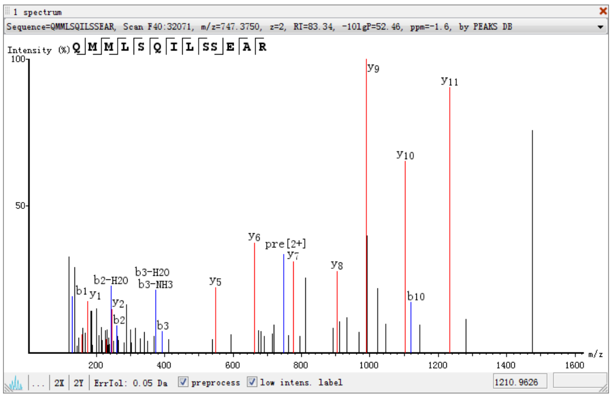

gi|566152765

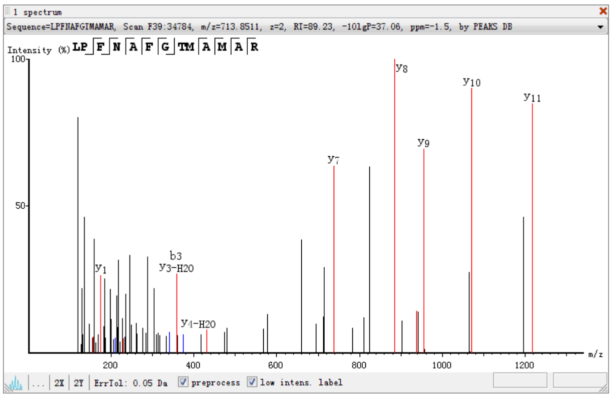

gi|566153407

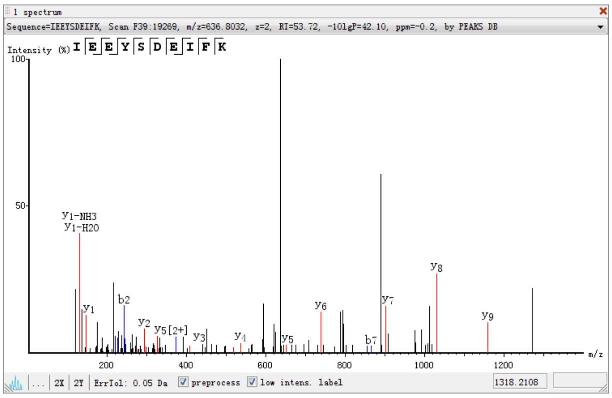

gi|566155466

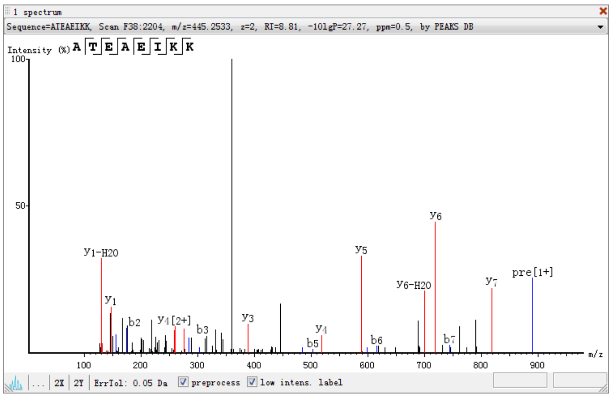

gi|566158761

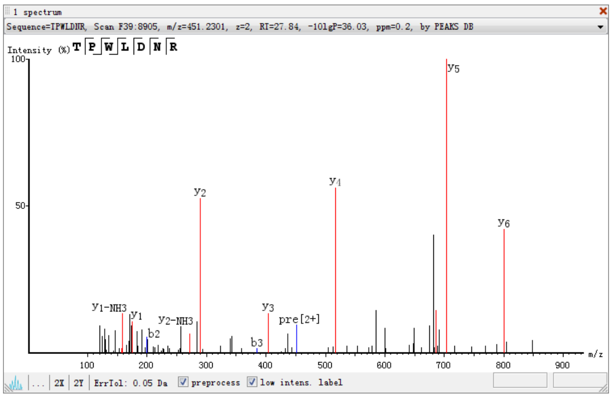

gi|566160574

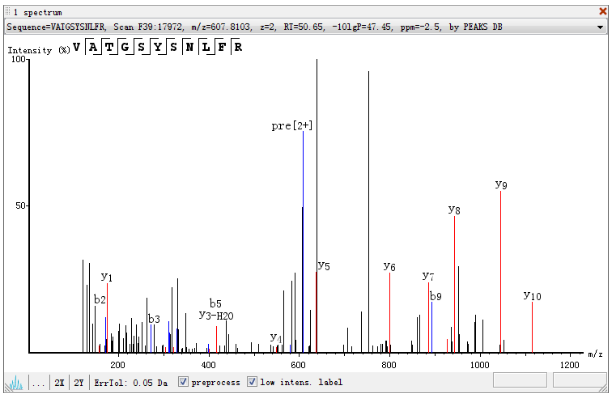

gi|566161272

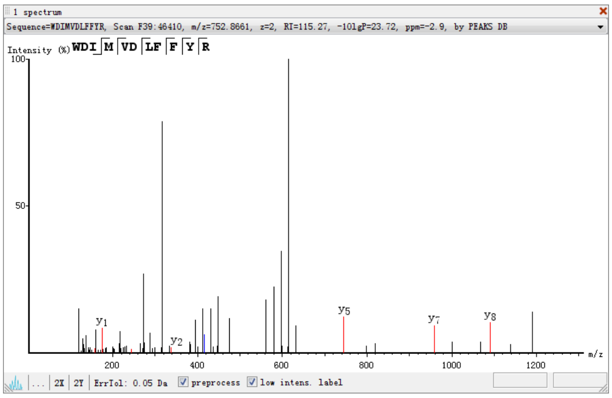

gi|566162026

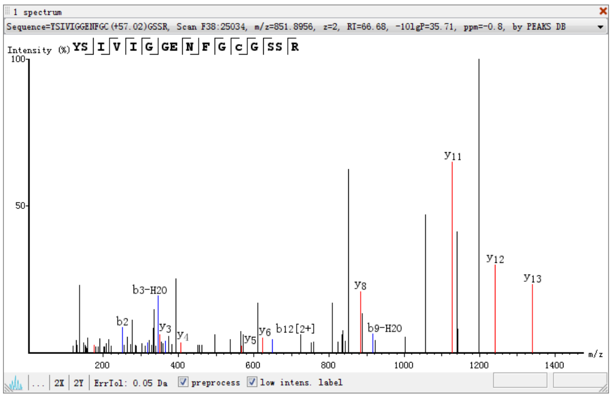

gi|566166593

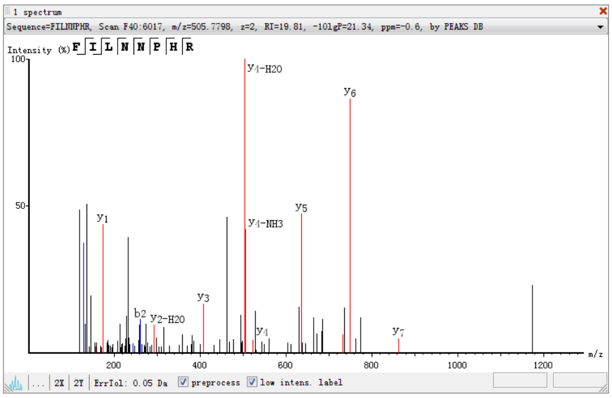

gi|566168009

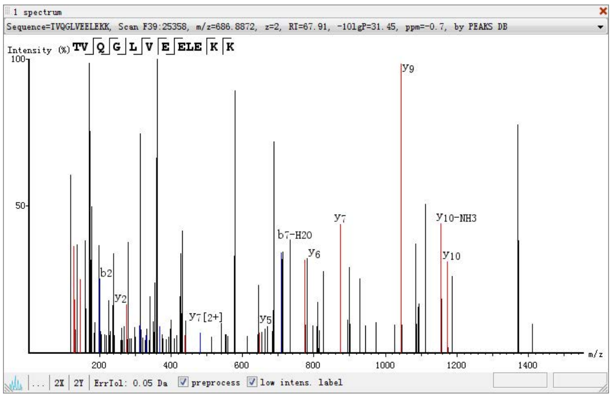

gi|566172301

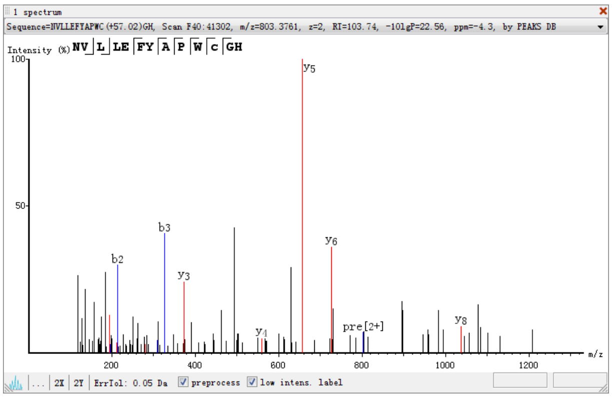

gi|566174458

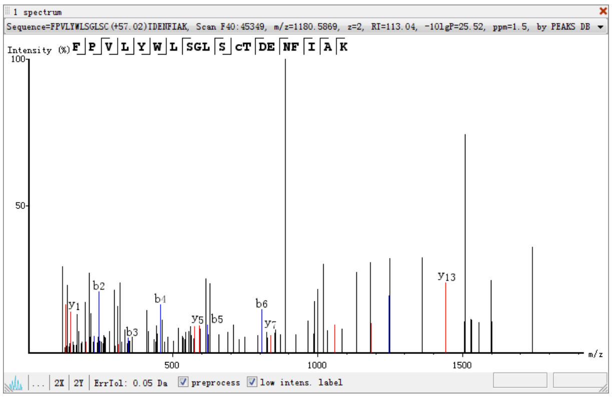

gi|566176622

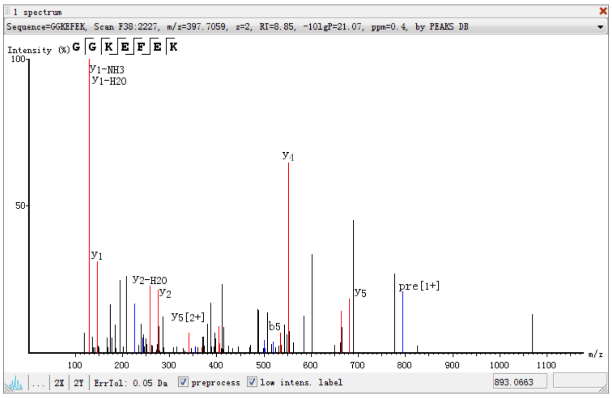

gi|566177370

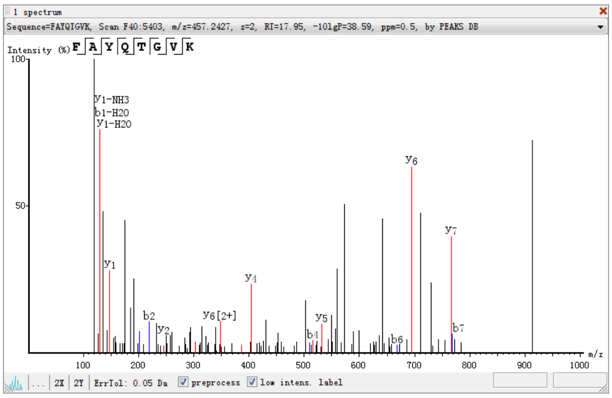

gi|566186111

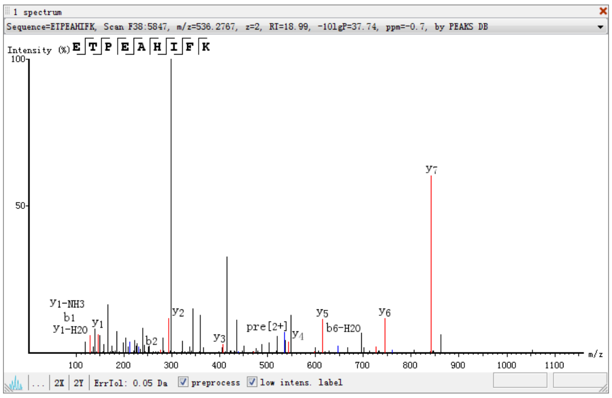

gi|566186274

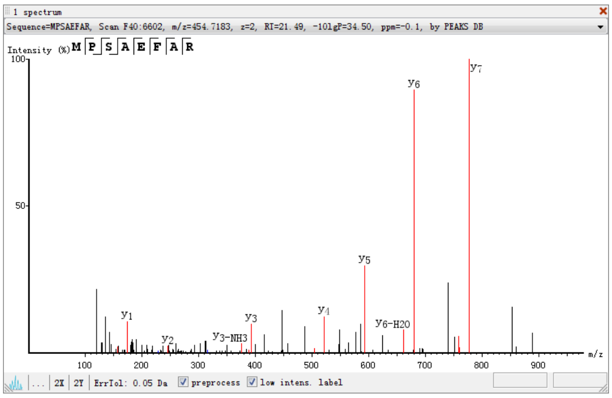

gi|566187112

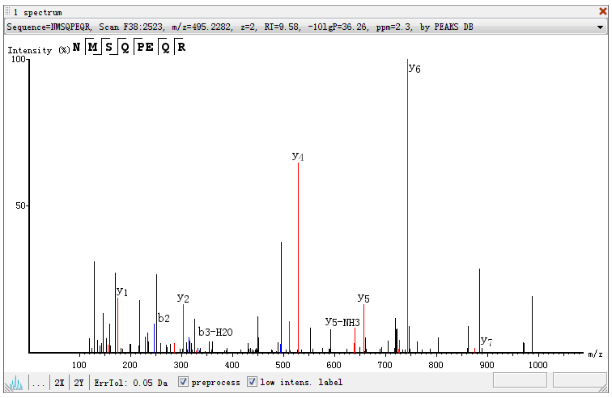

gi|566187811

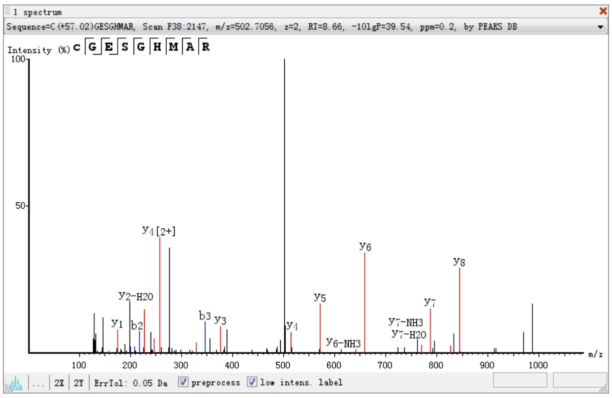

gi|566187829

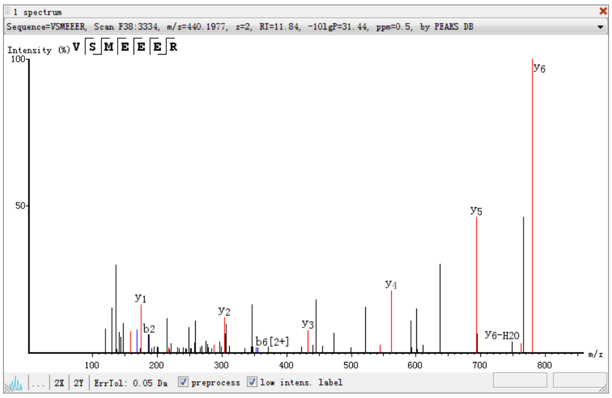

gi|566192083

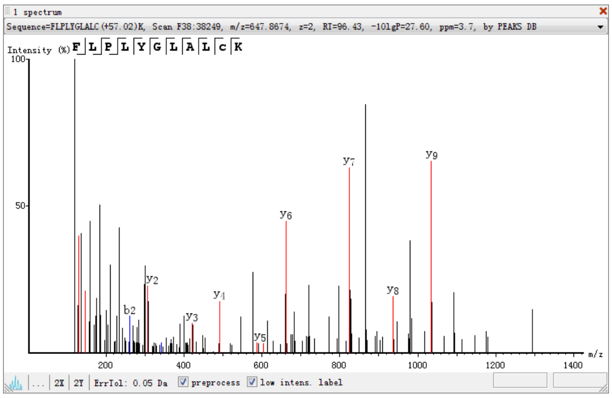

gi|566192679

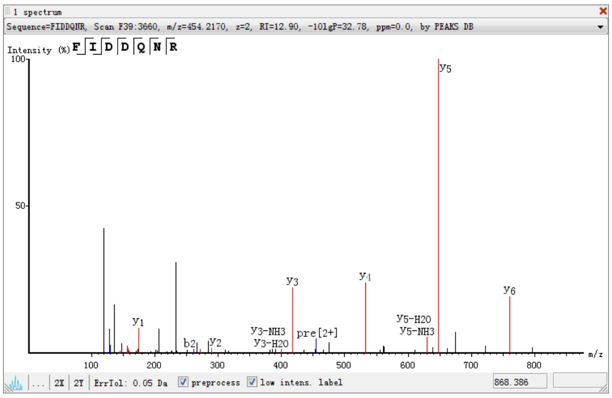

gi|566197448

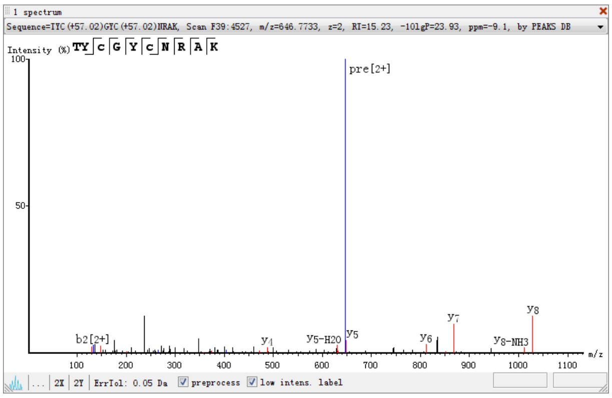

gi|566202327

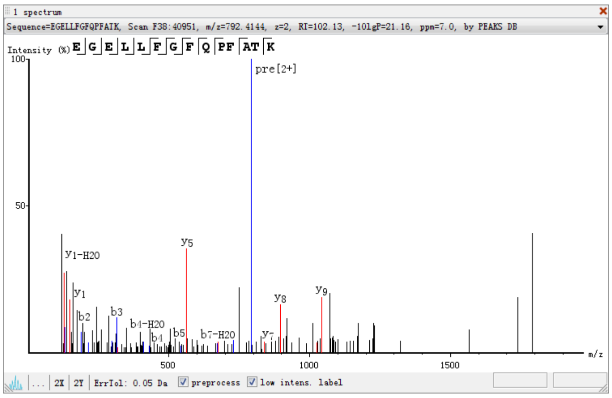

gi|566206526

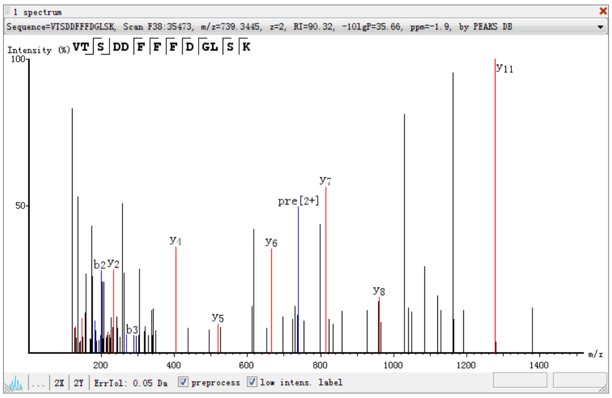

gi|566211408

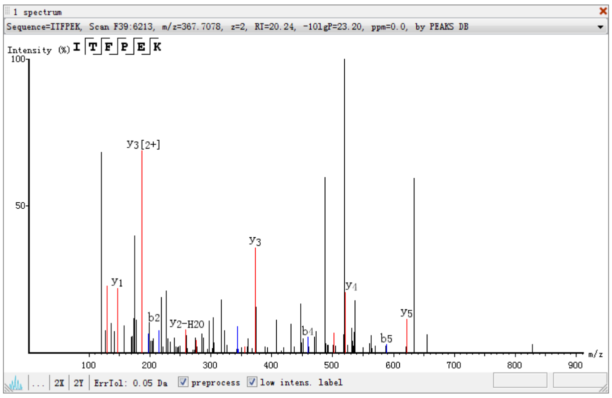

gi|566215197

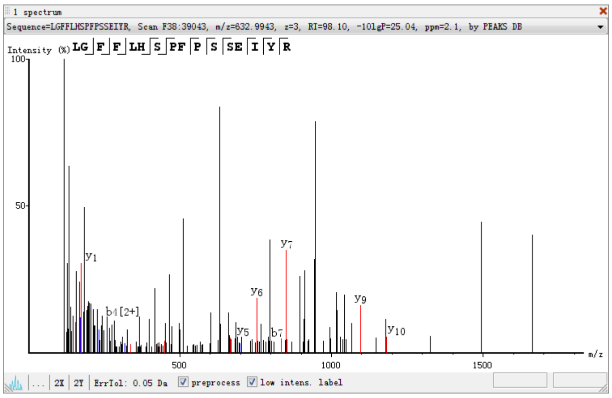

gi|566215982

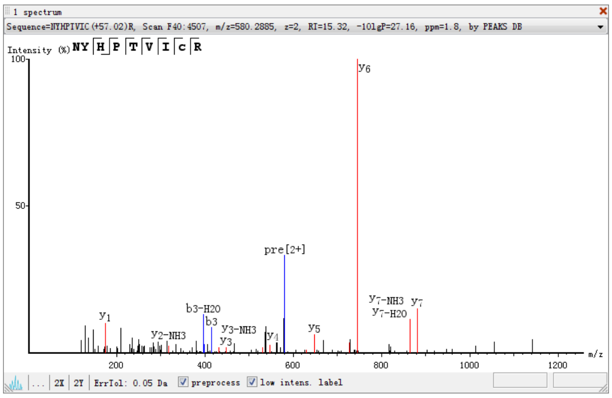

gi|566234529

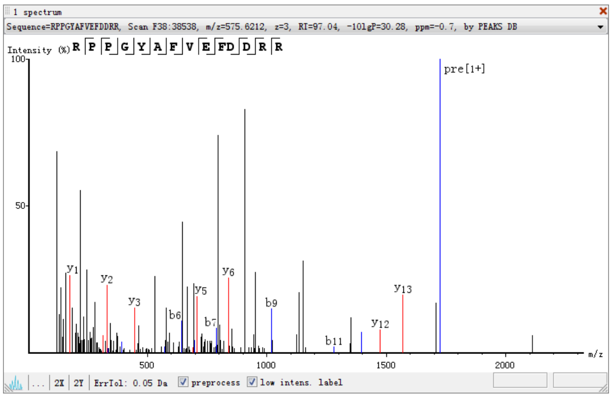

gi|566257303

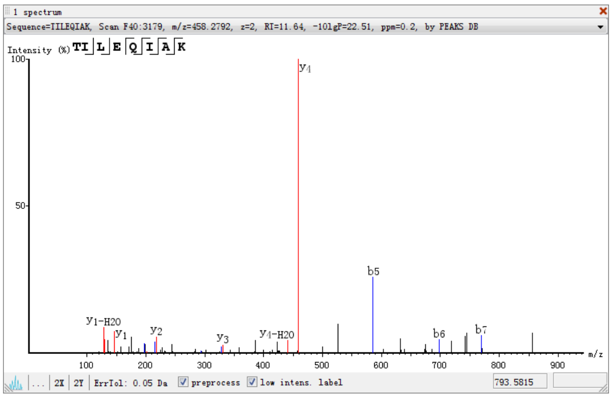

gi|566258212

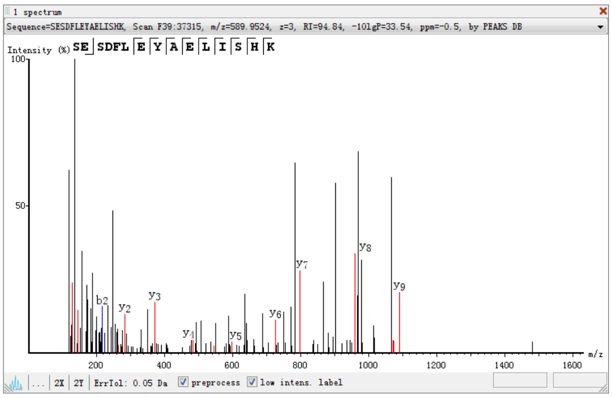

gi|731380876

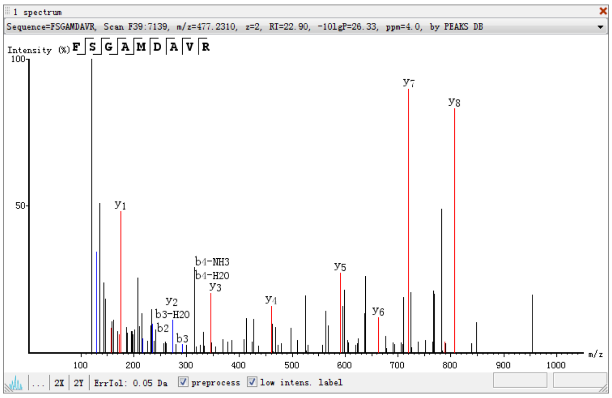

gi|731382912

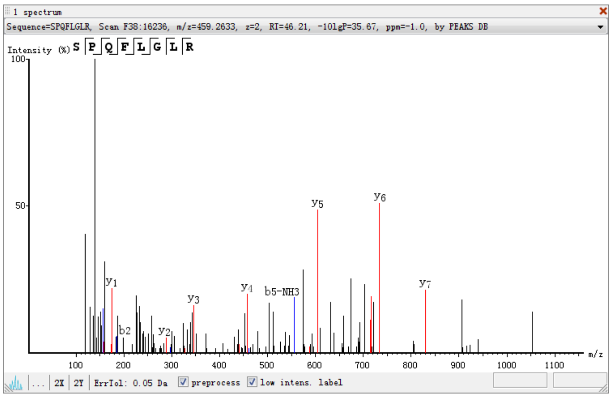

gi|731387671

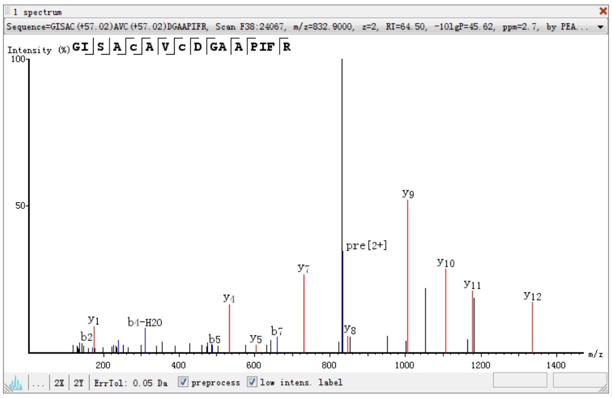

gi|731391105

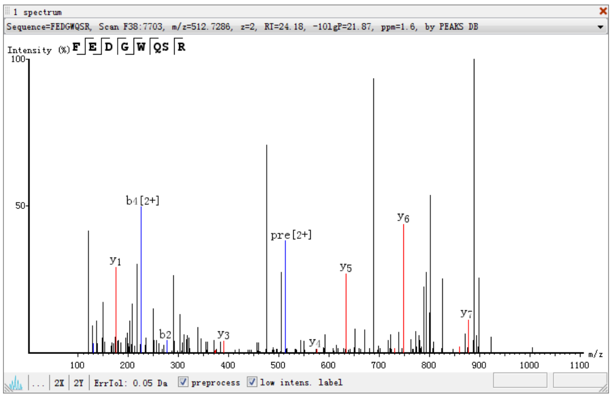

gi|731393107

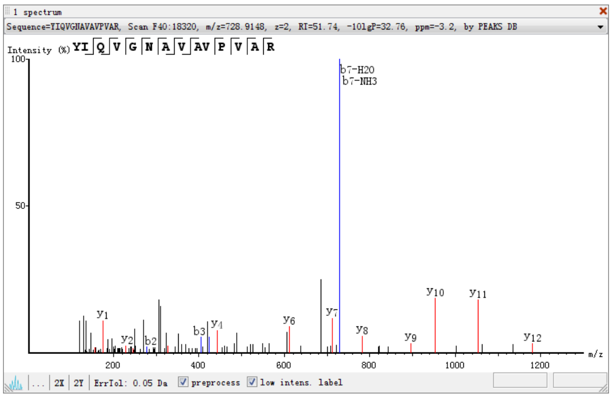

gi|731393292

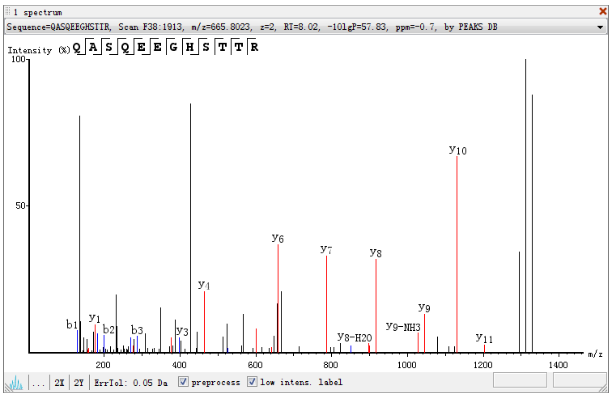

gi|731395934

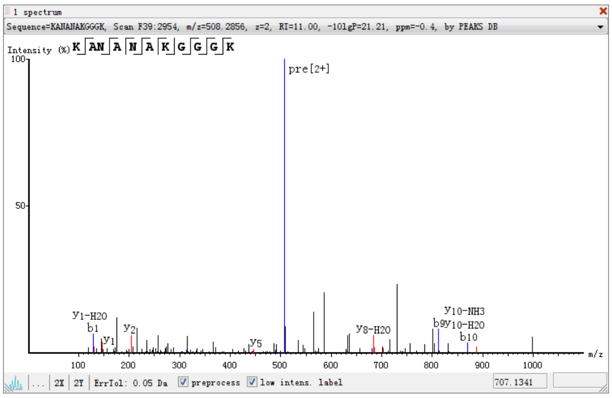

gi|731412938

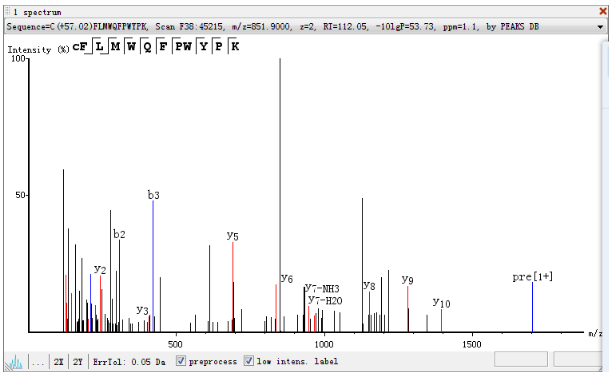

gi|731418622

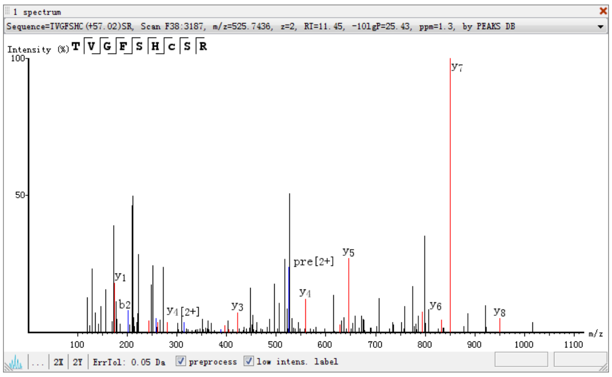

gi|731421066

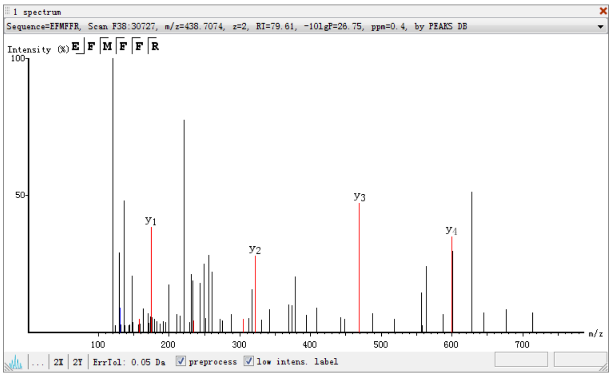

gi|731422501

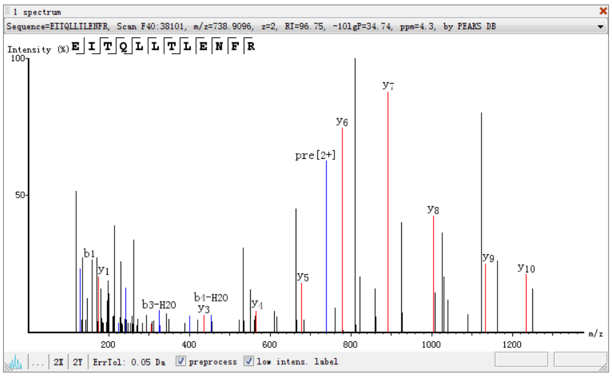

gi|731435142

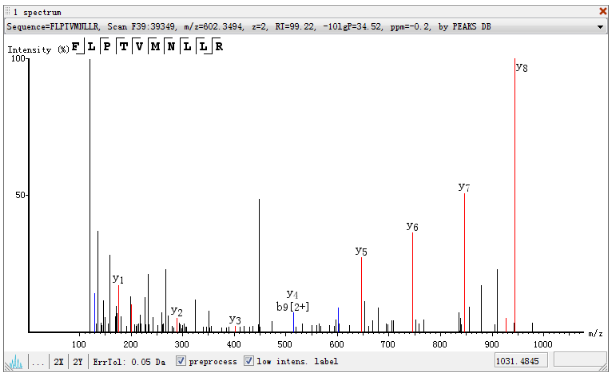

gi|733214771

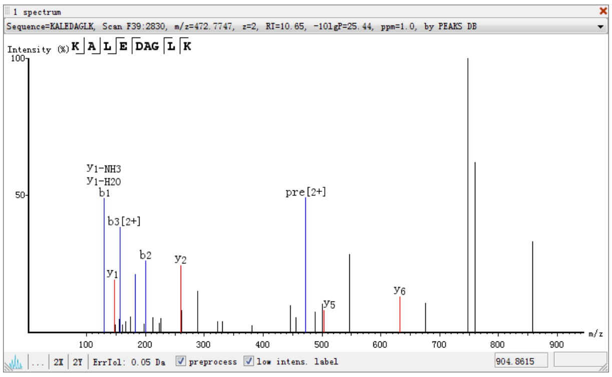

Fig.S2 The spectra of the proteins identified by one unique peptide with only one spectrum in CK1. Upper is the accession of protein, and under is its corresponding spectrum.

gi|224060455

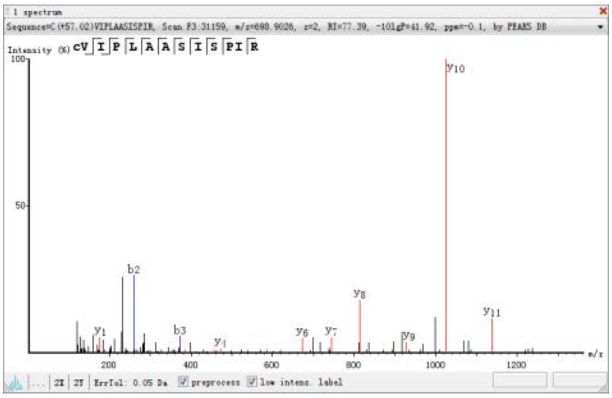

gi|224066959

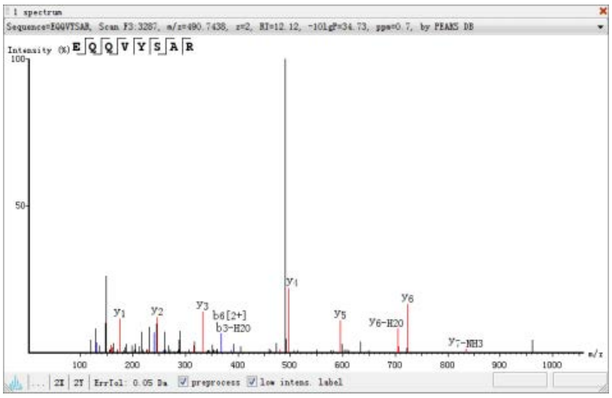

gi|224067318

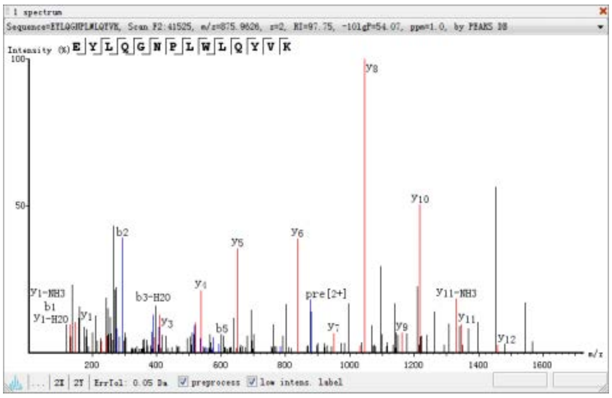

gi|224067846

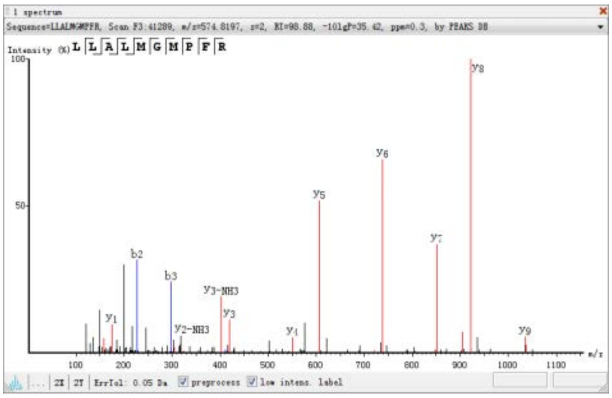

gi|224073306

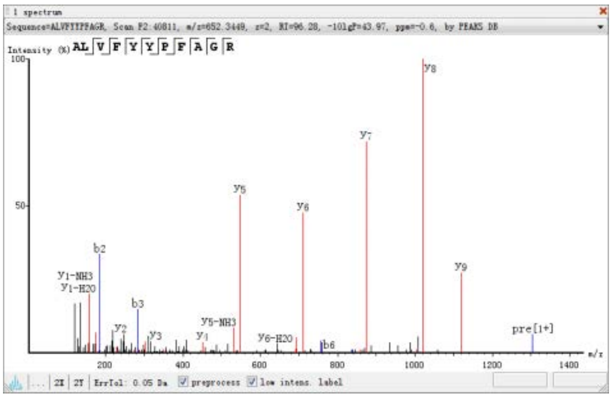

gi|224073851

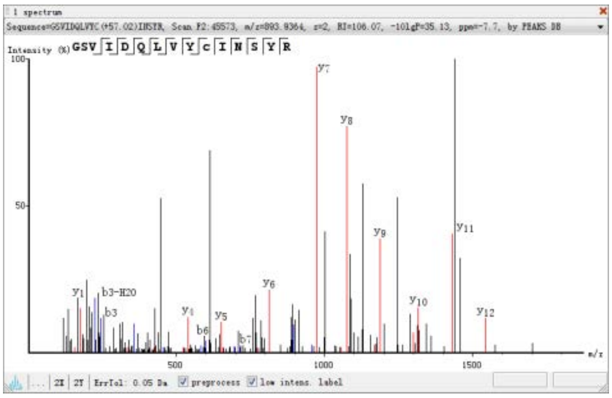

gi|224079530

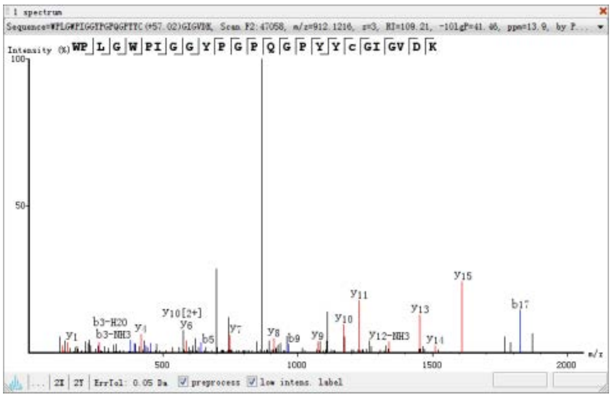

gi|224080325

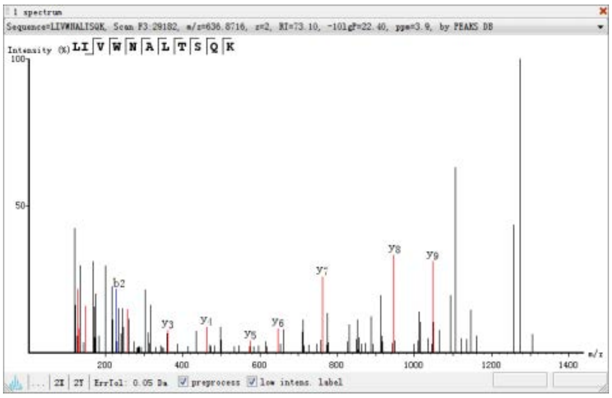

gi|224083163

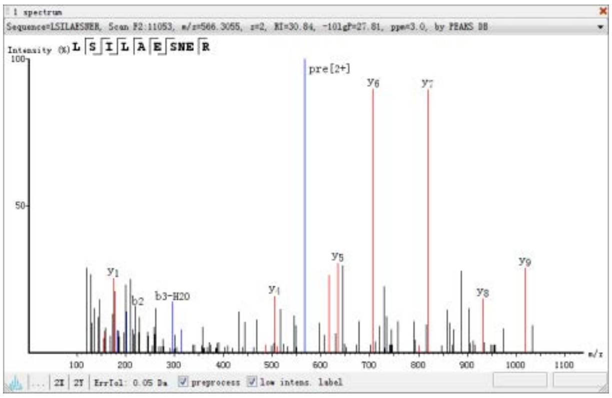

gi|224084864

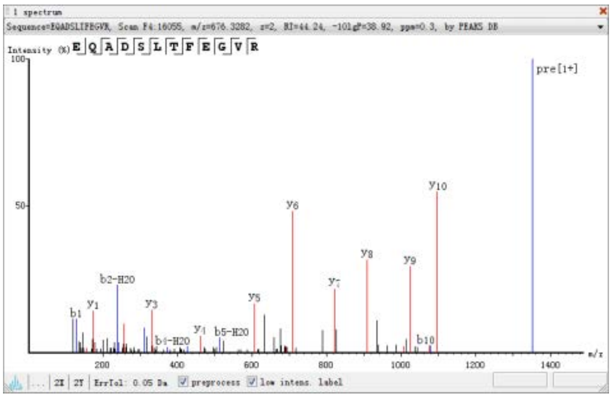

gi|224085103

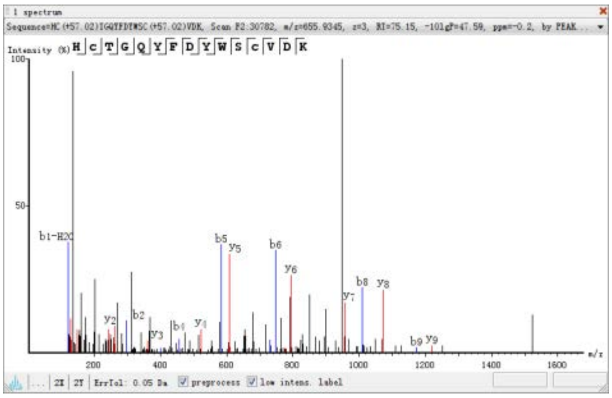

gi|224086130

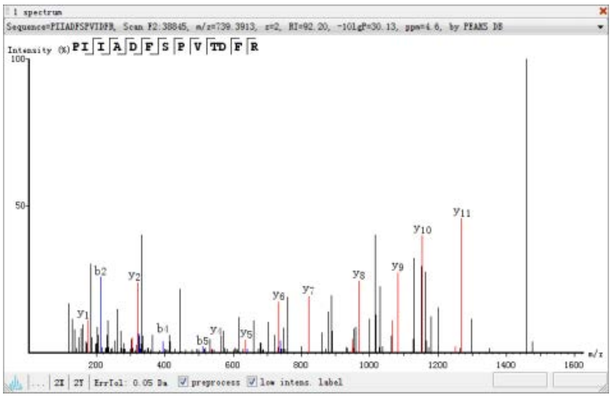

gi|224089058

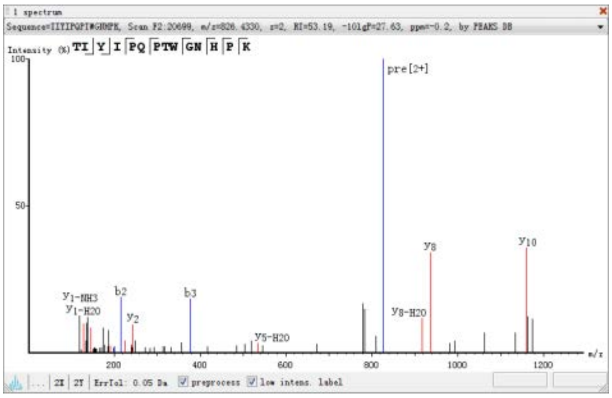

gi|224089182

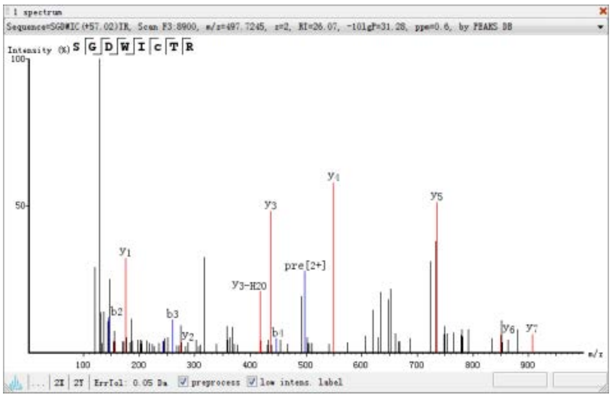

gi|224092266

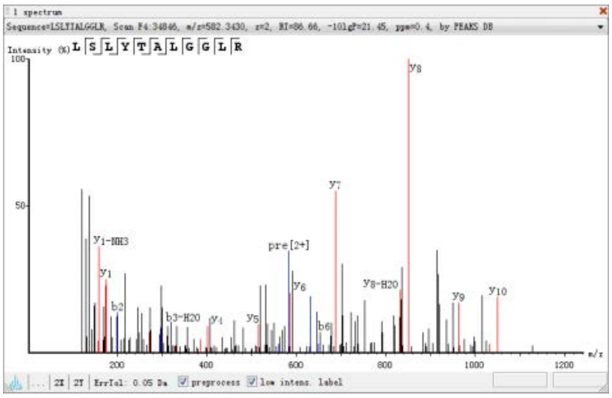

gi|224093896

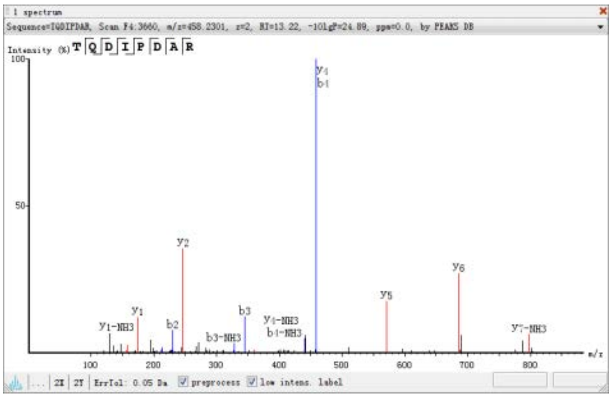

gi|224094759

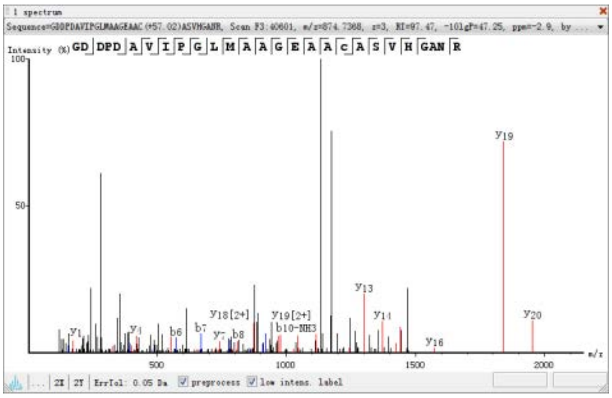

gi|224094809

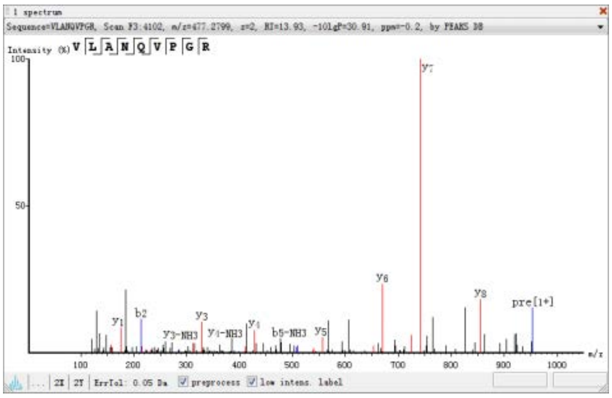

gi|224096333

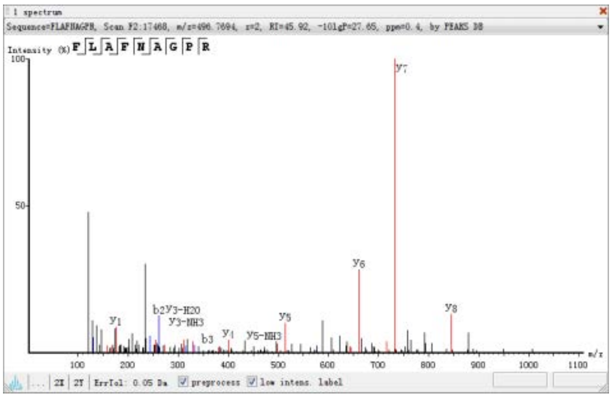

gi|224100839

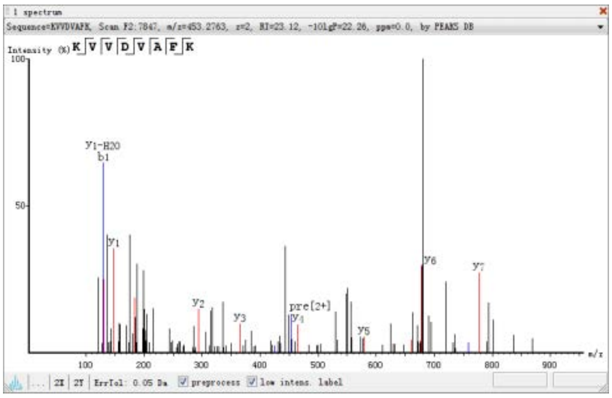

gi|224100909

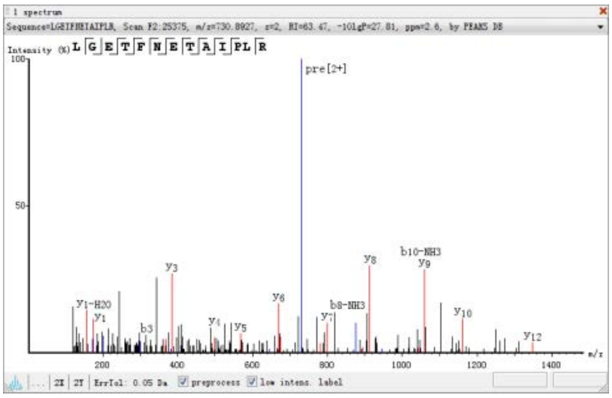

gi|224102881

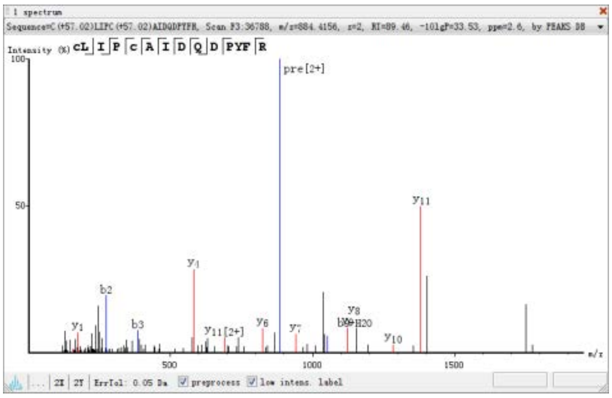

gi|224103873

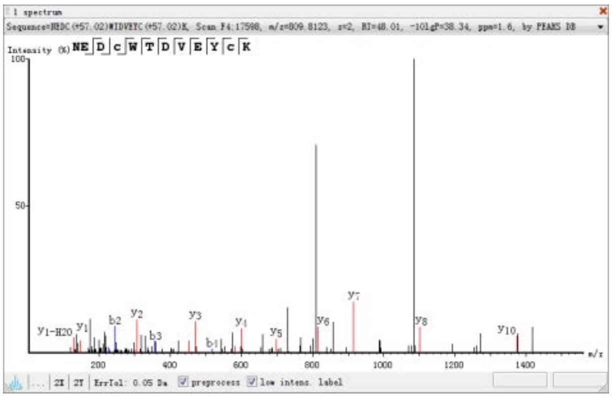

gi|224104643

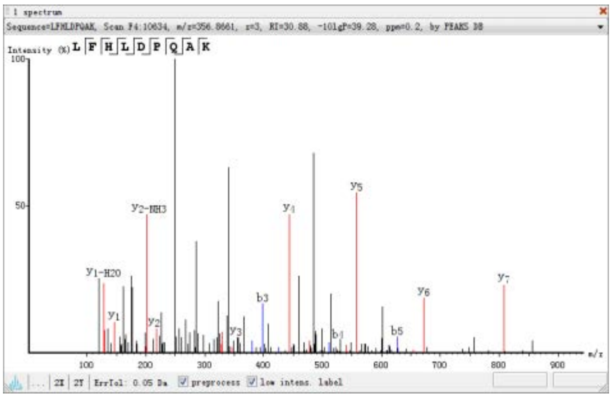

gi|224105405

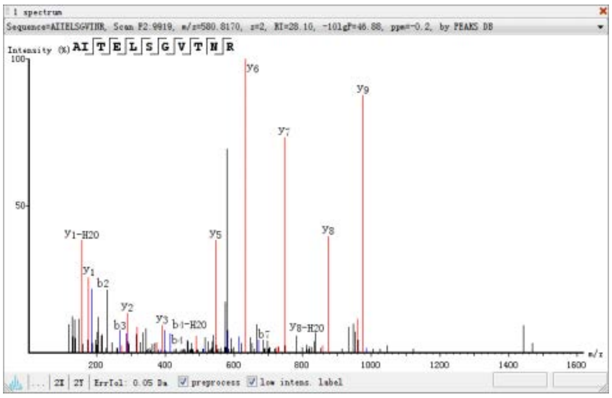

gi|224105455

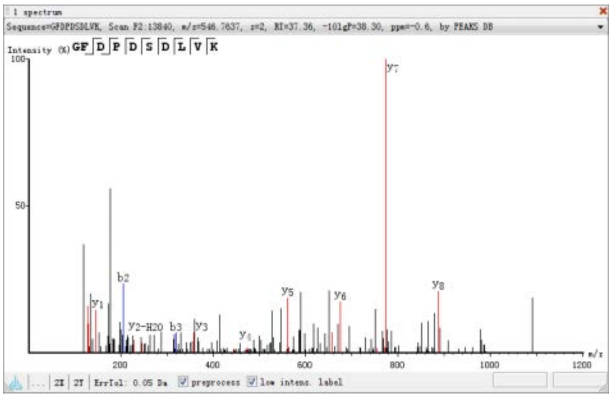

gi|224106319

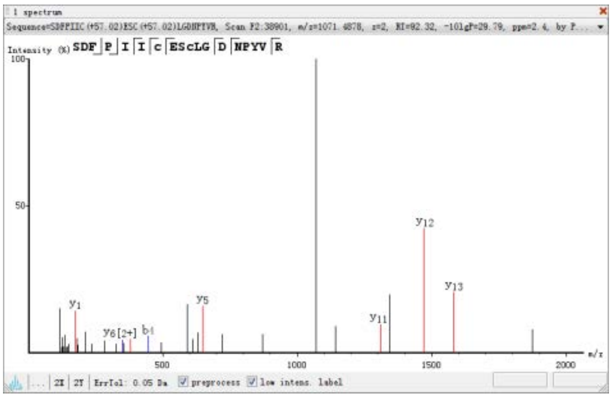

gi|224107749

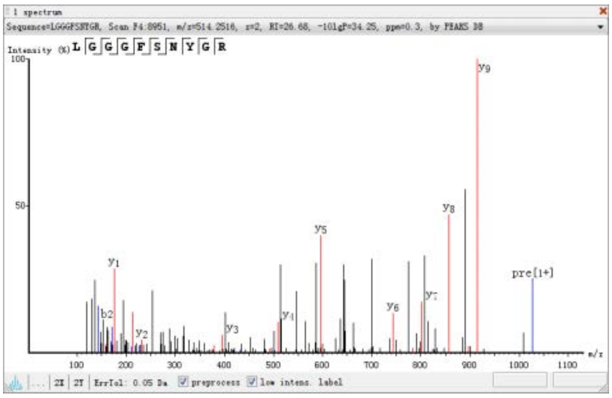

gi|224112341

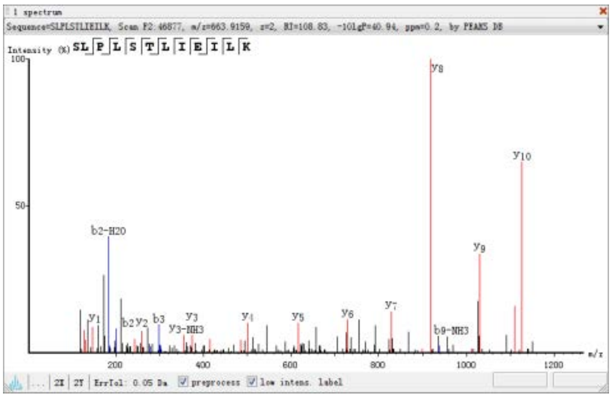

gi|224114830

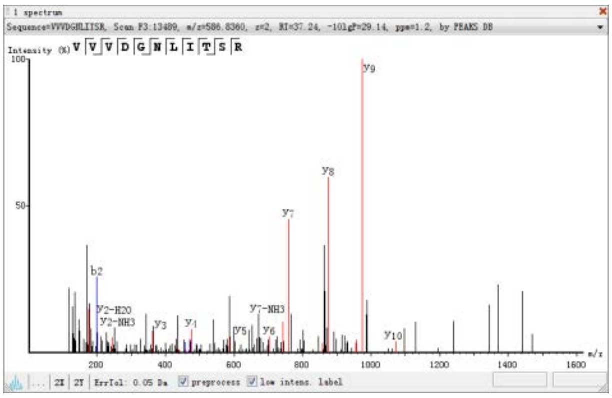

gi|224115158

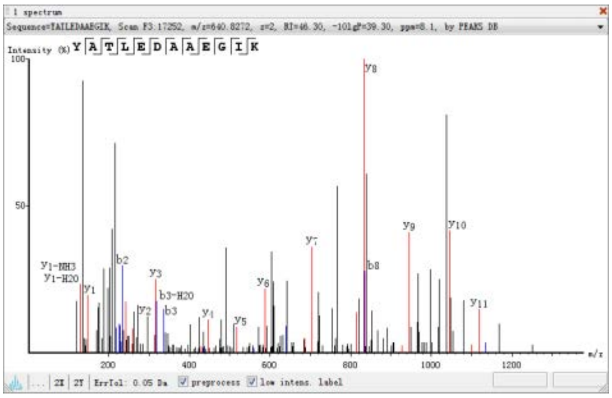

gi|224116788

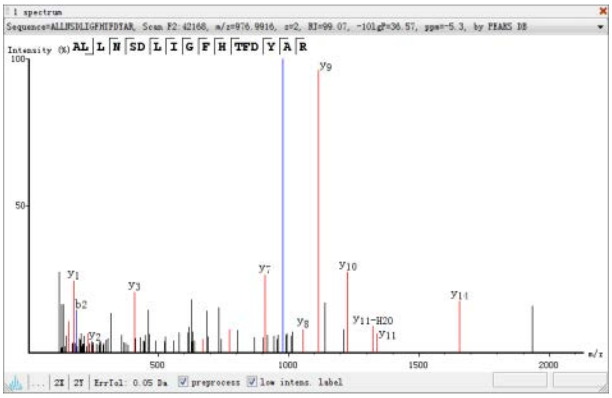

gi|224121470

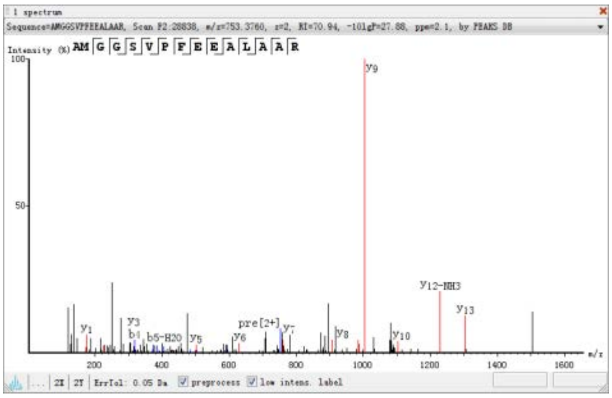

gi|224124820

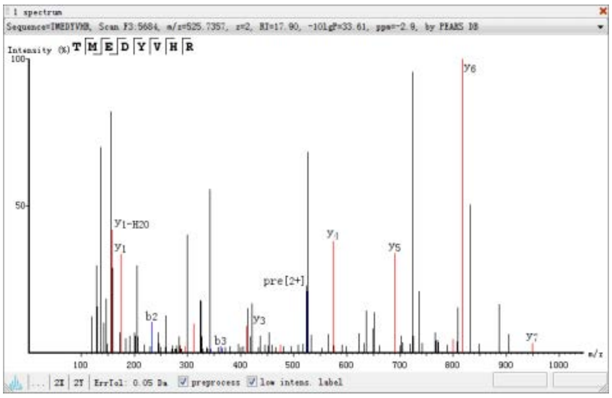

gi|224126151

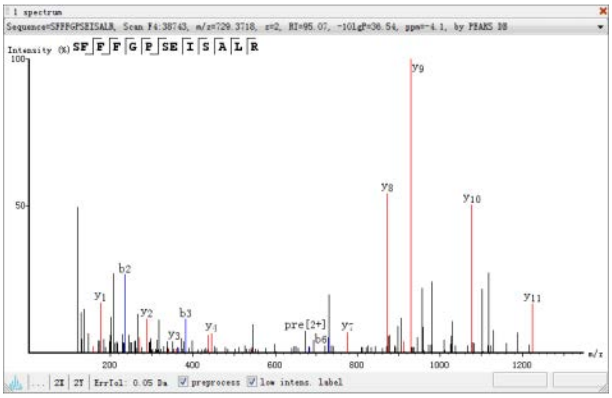

gi|224126789

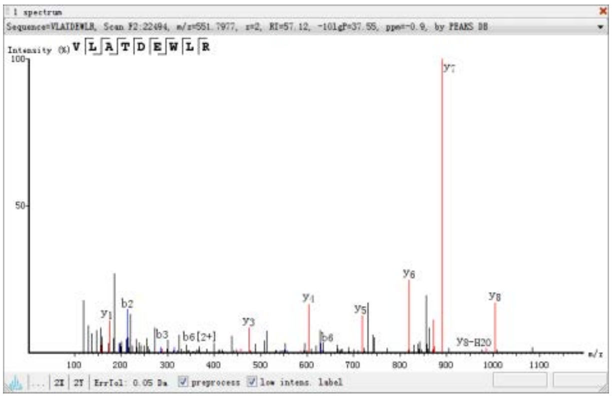

gi|224129596

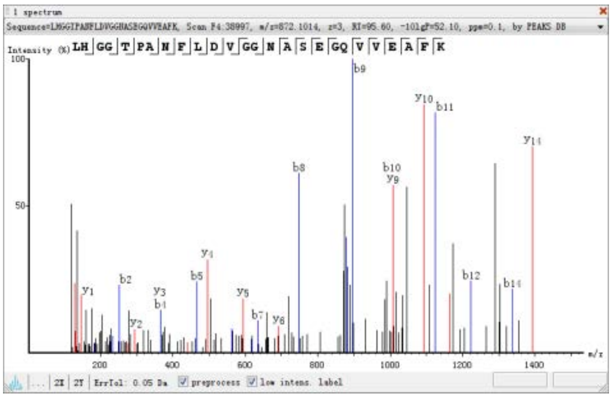

gi|224130362

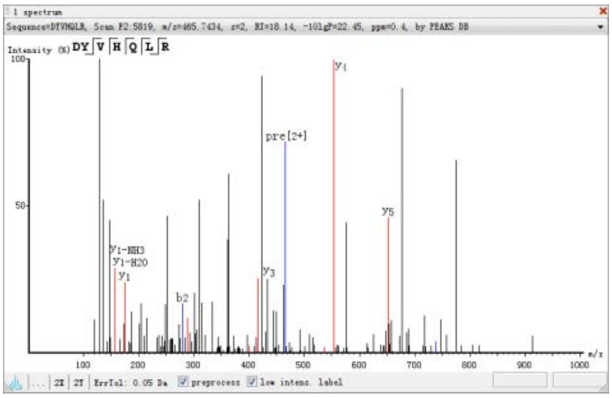

gi|224130486

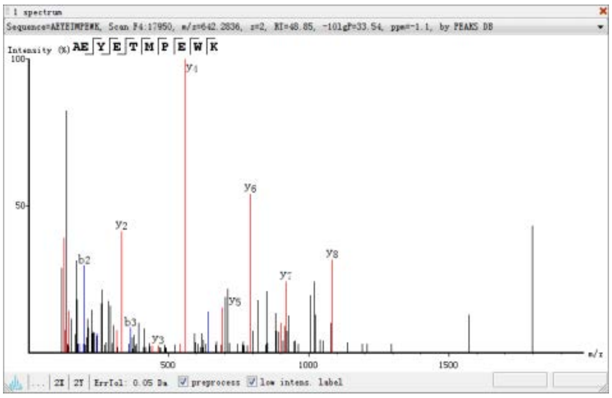

gi|224138022

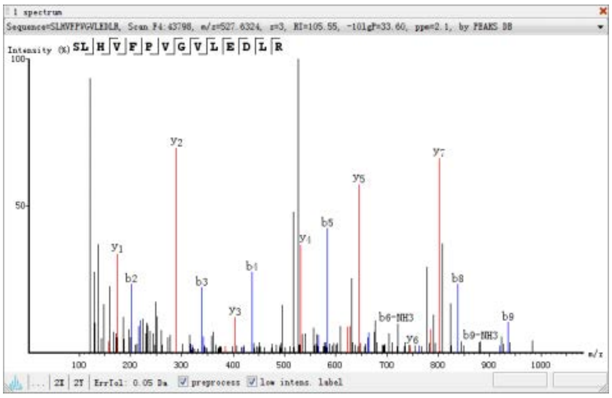

gi|224138170

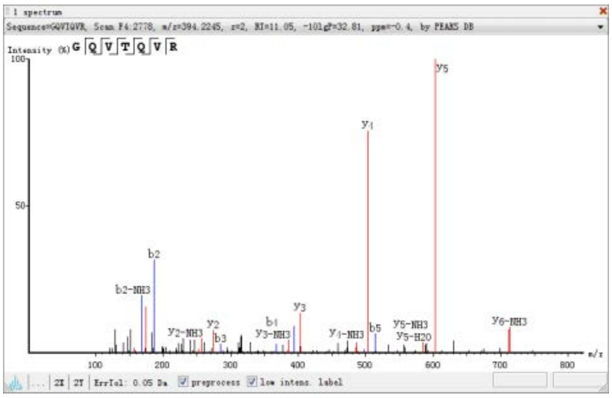

gi|224141359

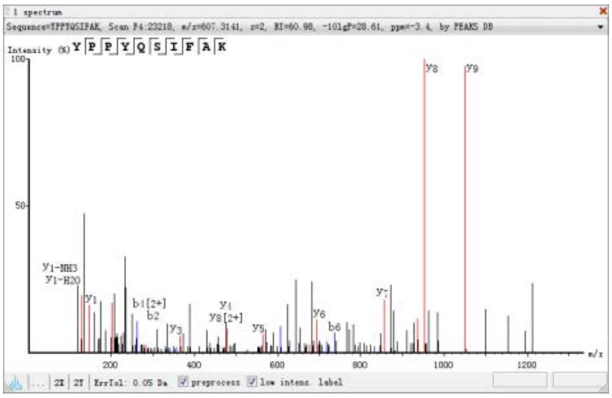

gi|224143559

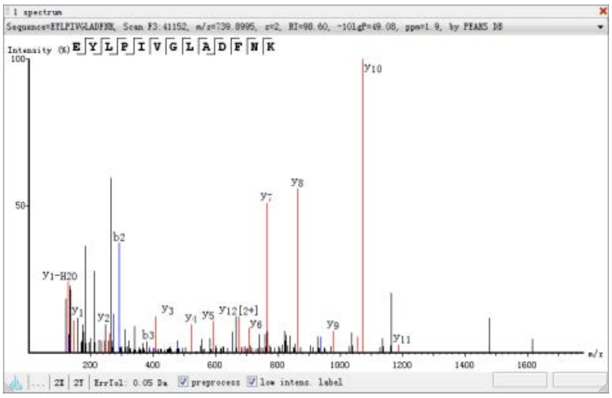

gi|224143772

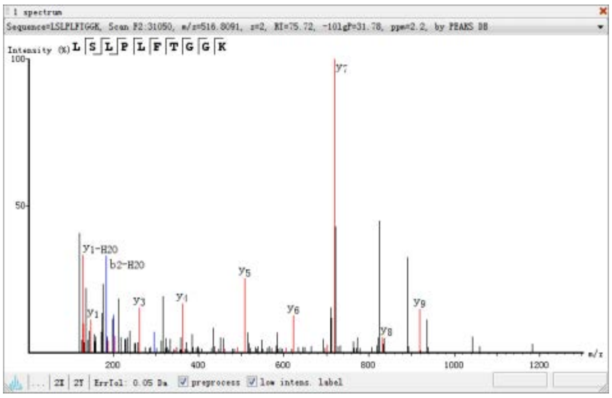

gi|224146178

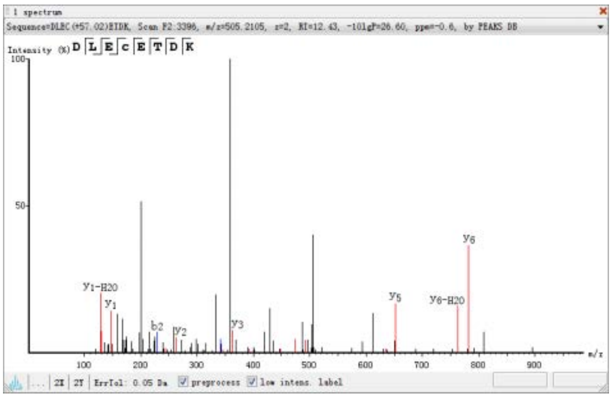

gi|224146696

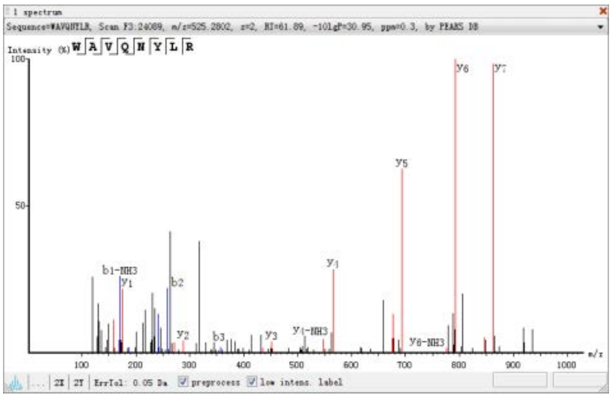

gi|225423491

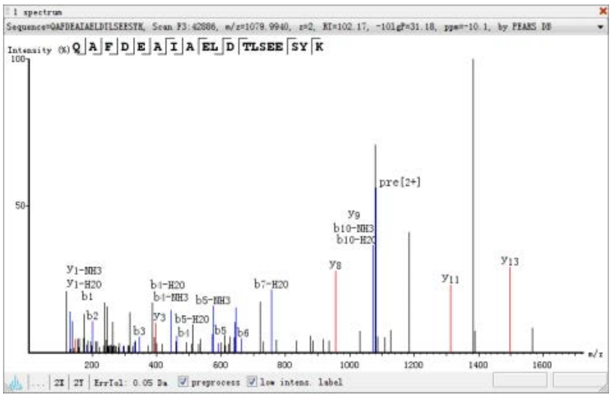

gi|225424272

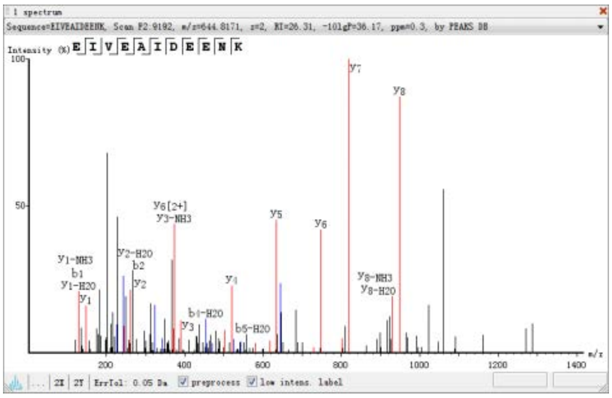

gi|225424713

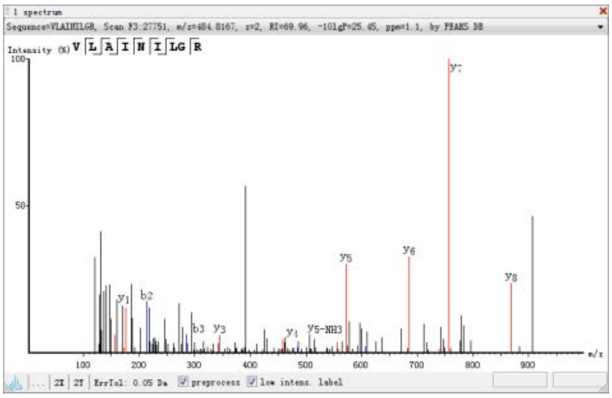

gi|225425274

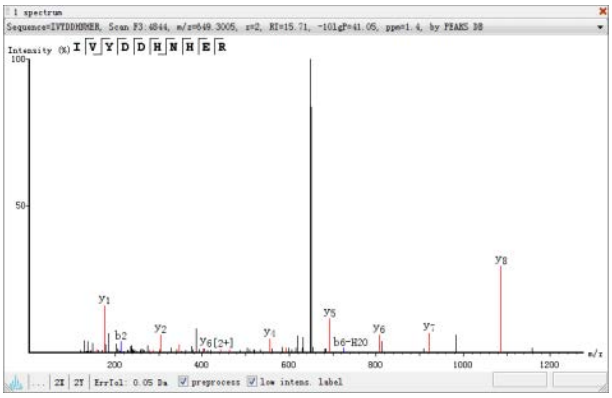

gi|225425595

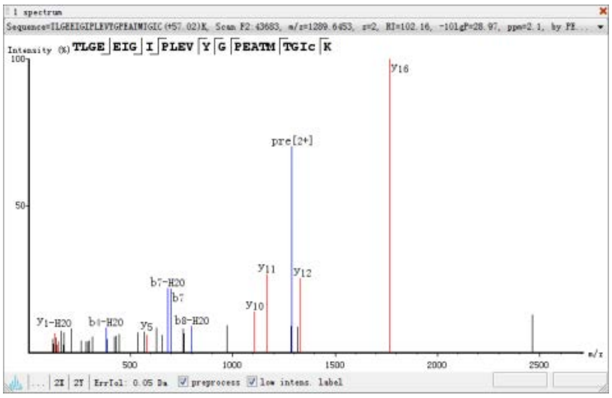

gi|225425706

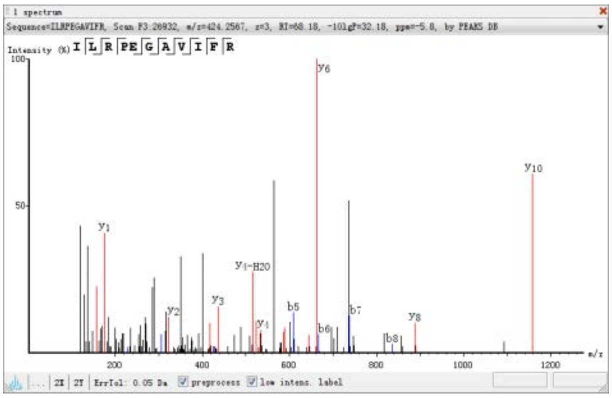

gi|225426312

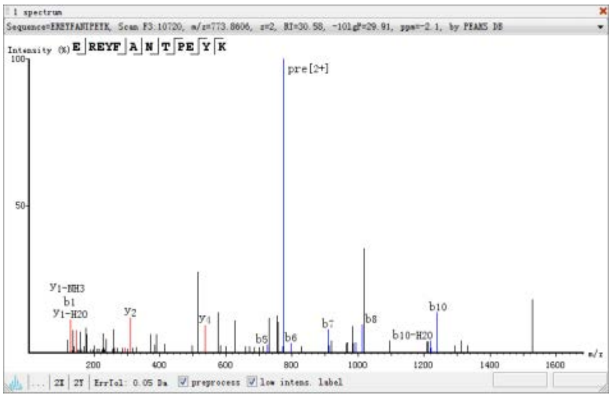

gi|225427419

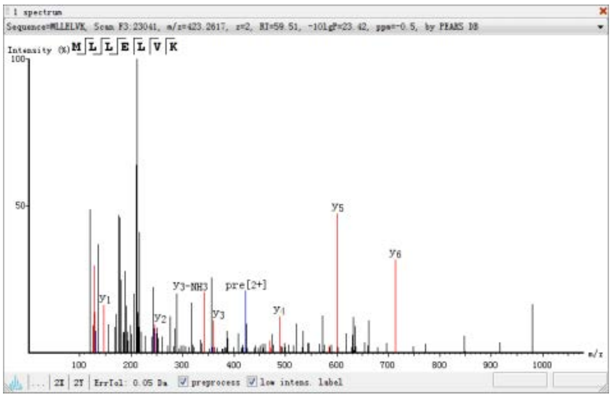

gi|225428009

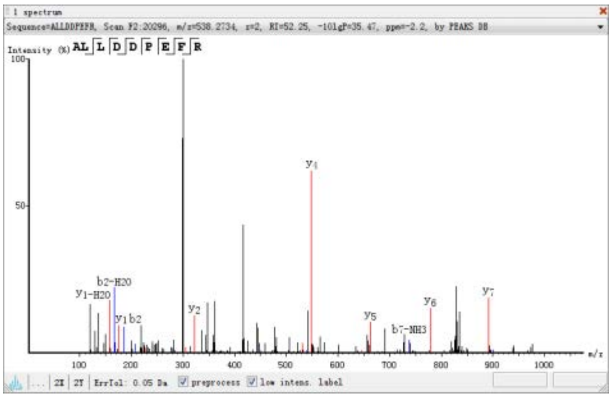

gi|225428865

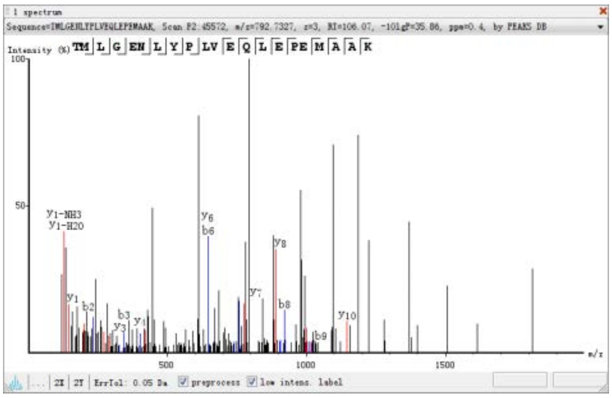

gi|225428898

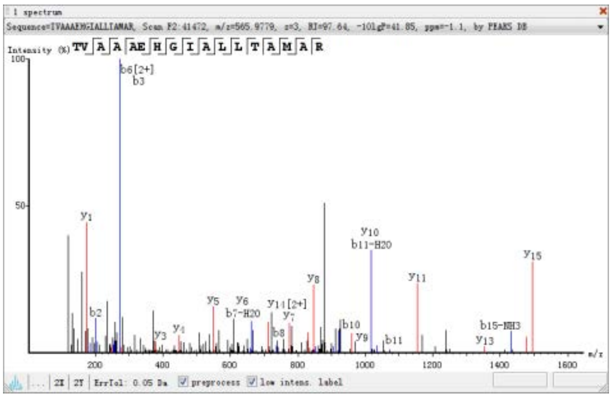

gi|225429450

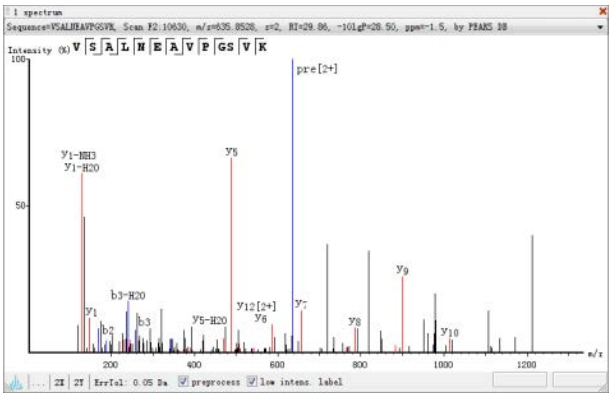

gi|225429995

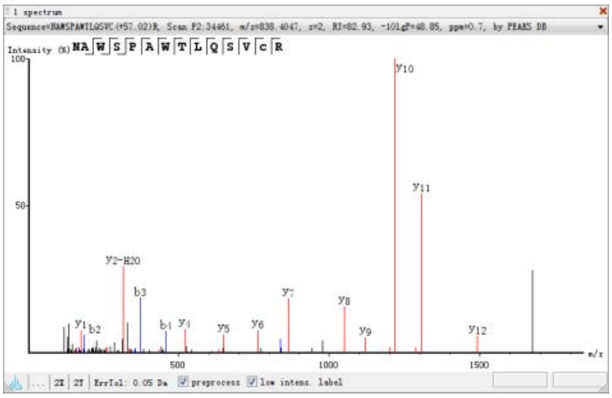

gi|225430488

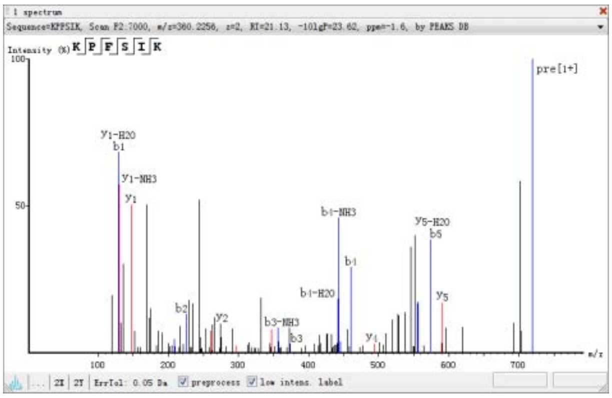

gi|225431755

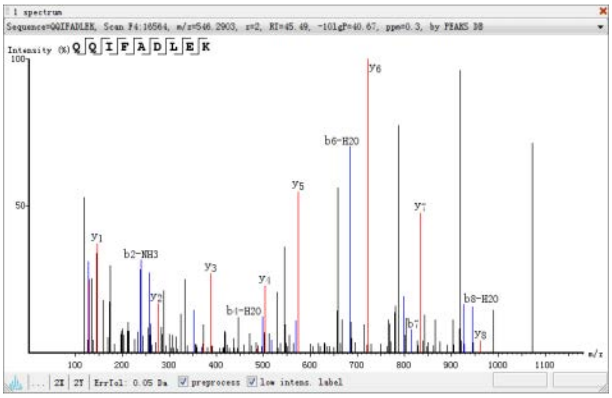

gi|225431922

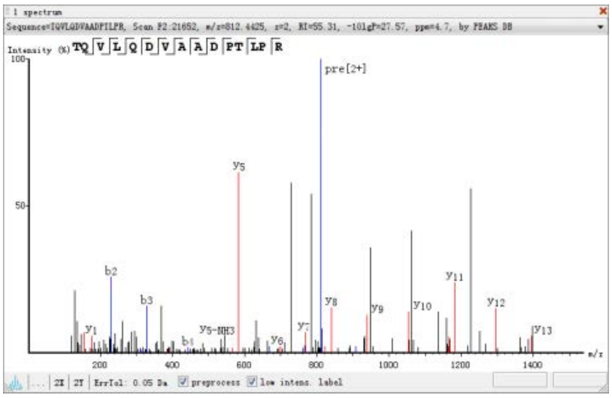

gi|225432664

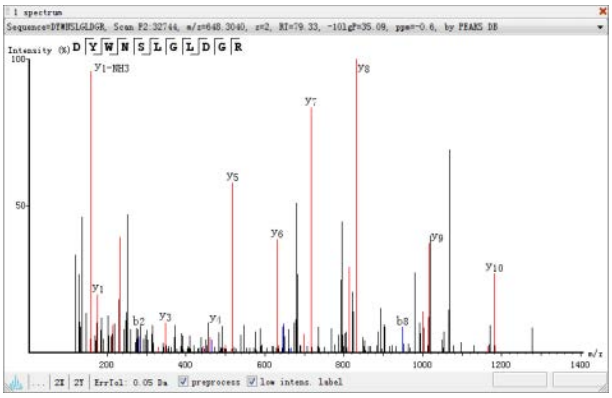

gi|225434191

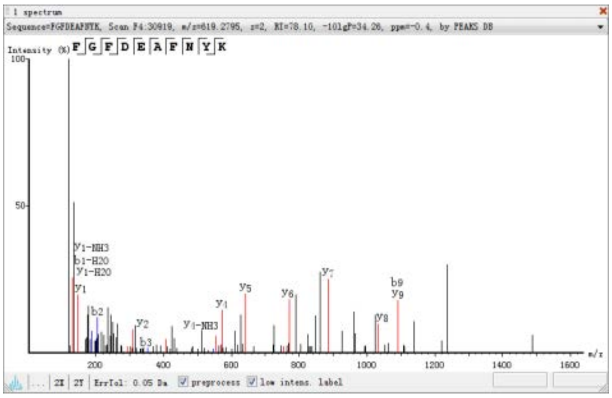

gi|225434259

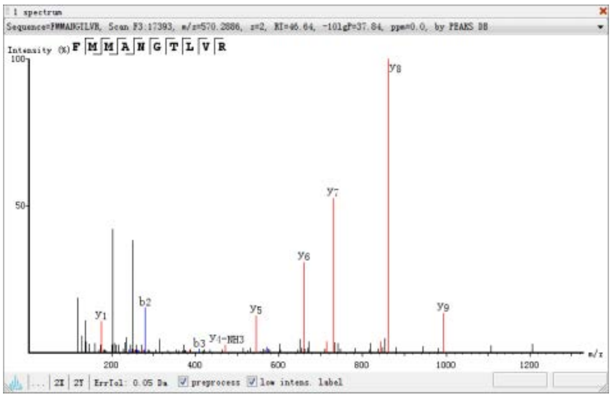

gi|225434692

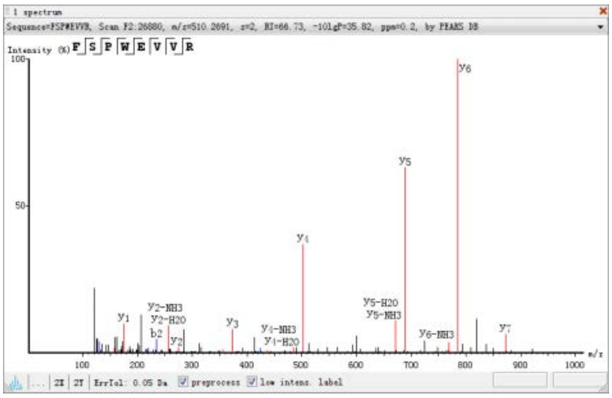

gi|225434984

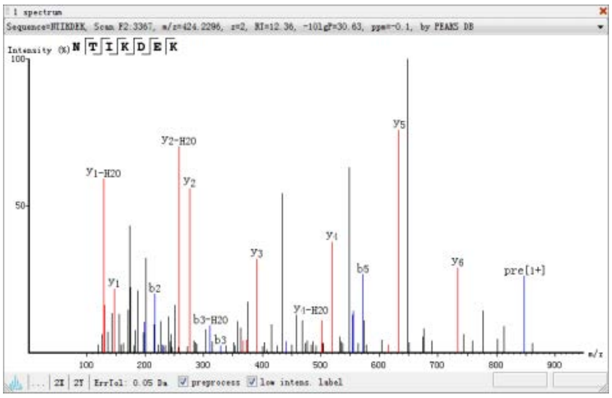

gi|225435122

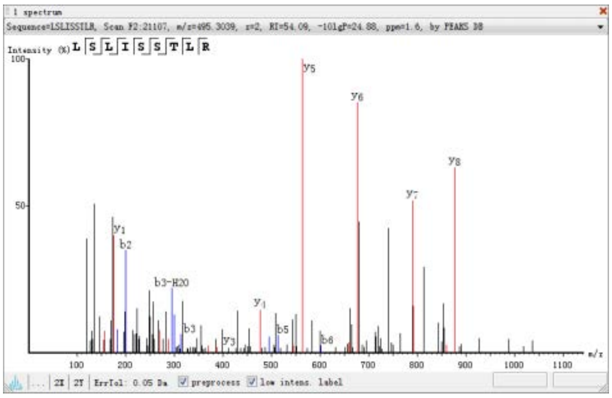

gi|225435189

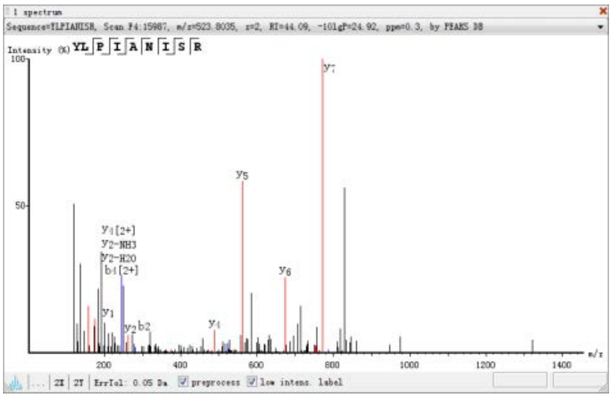

gi|225435925

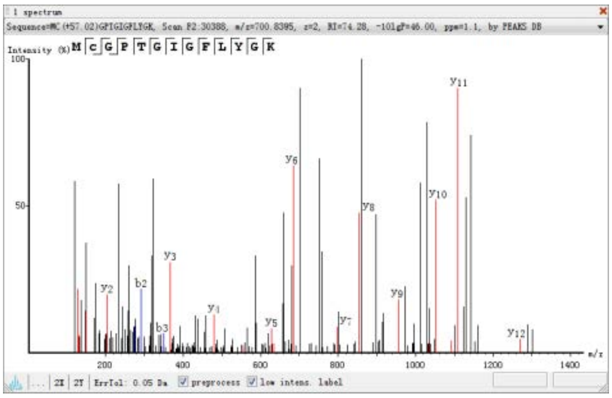

gi|225437493

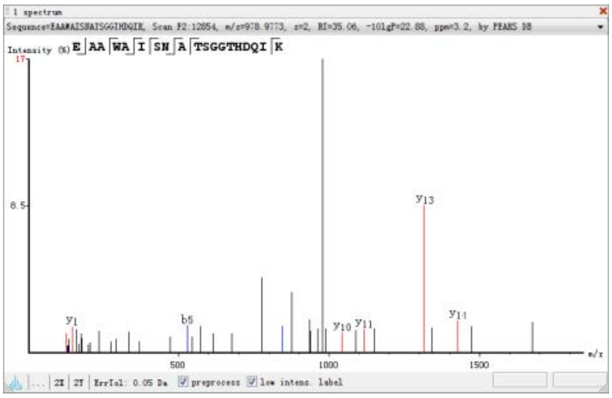

gi|225437598

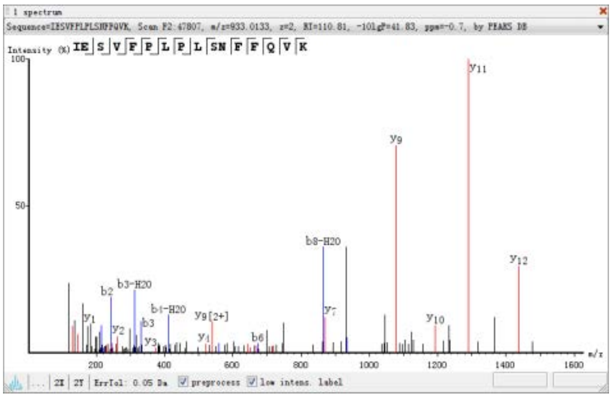

gi|225437618

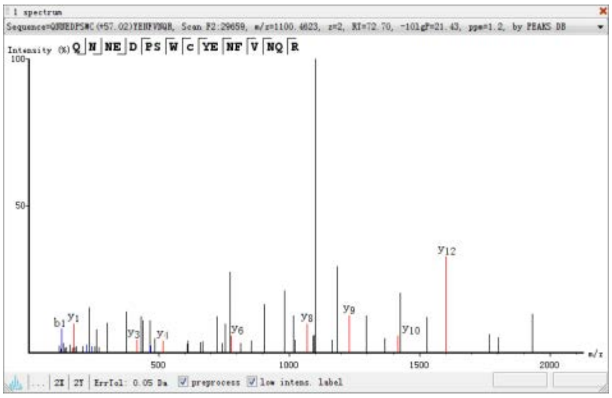

gi|225438035

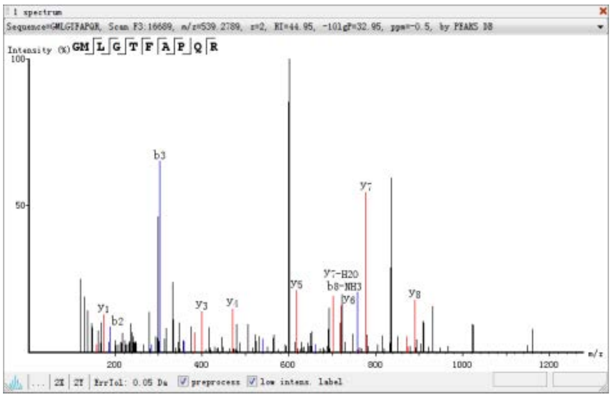

gi|225438067

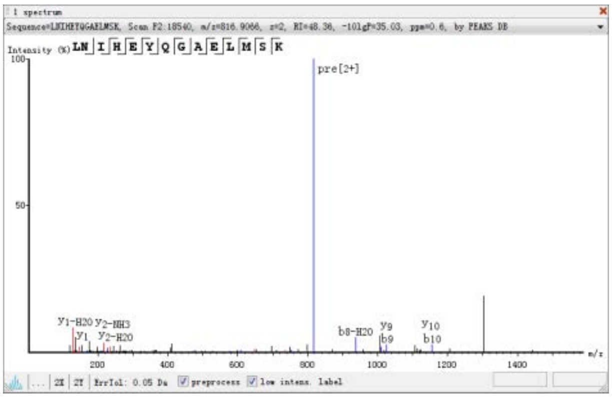

gi|225439775

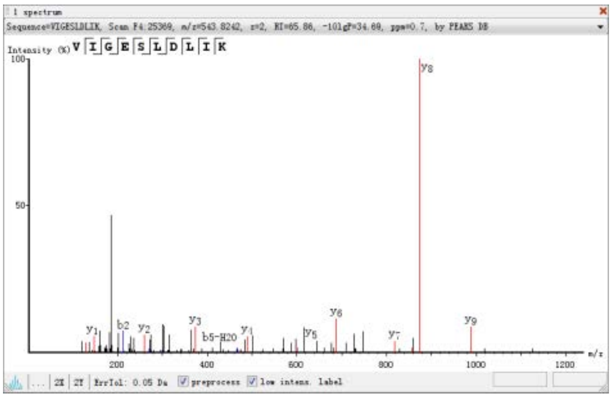

gi|225442357

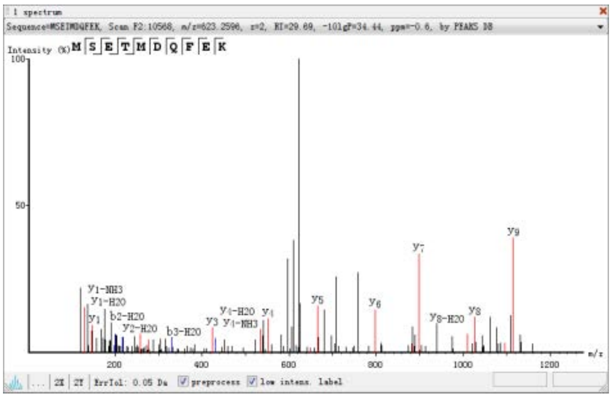

gi|225442791

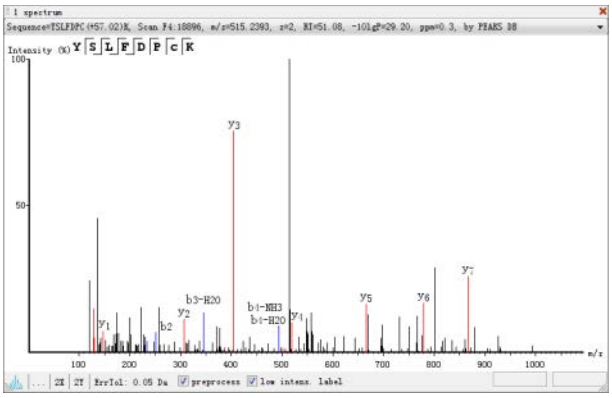

gi|225443109

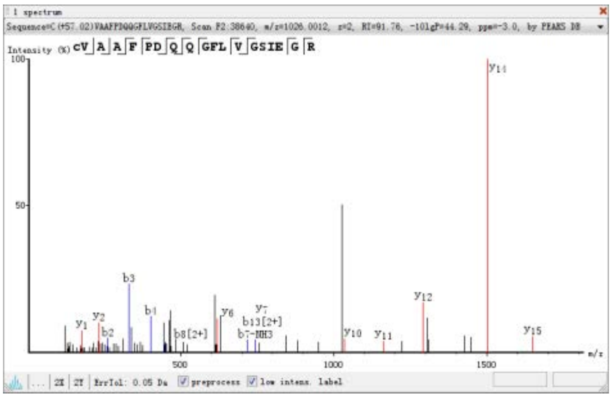

gi|225443231

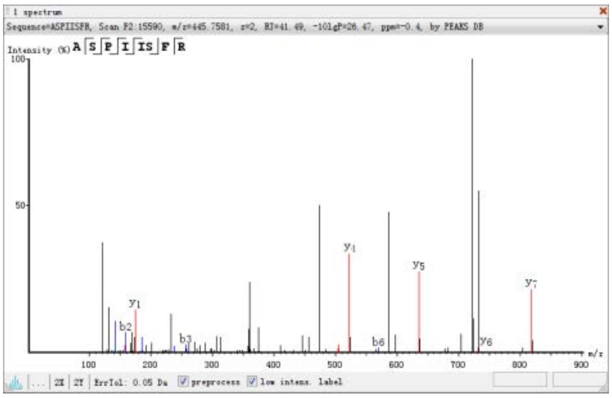

gi|225443738

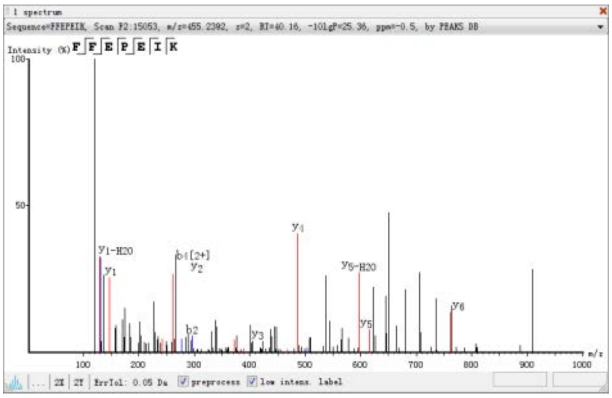

gi|225444159

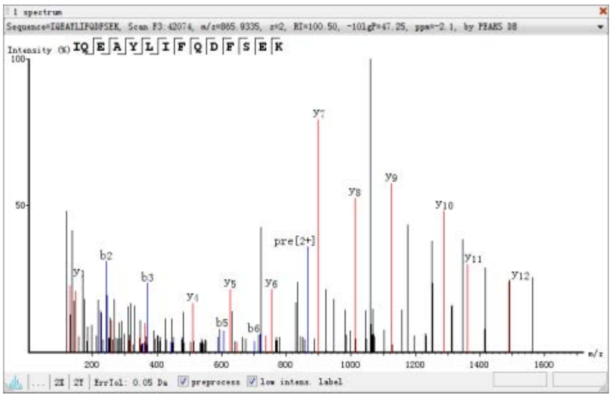

gi|225444525

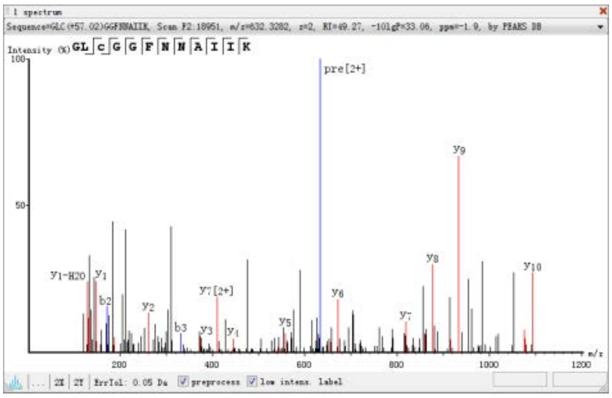

gi|225444537

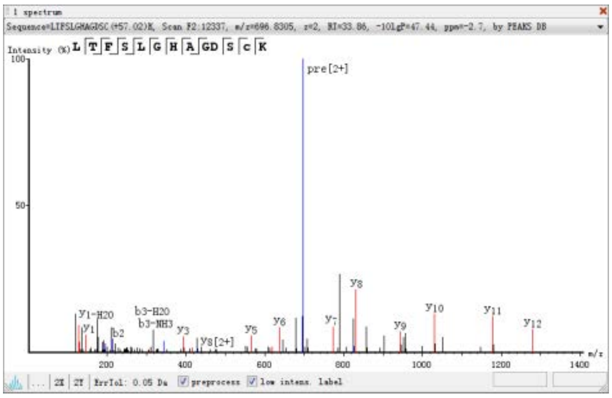

gi|225445080

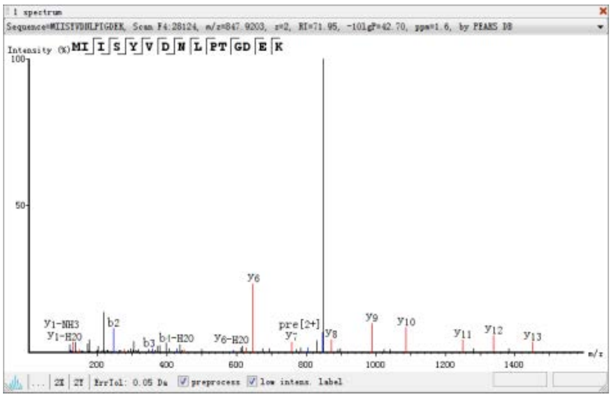

gi|225445170

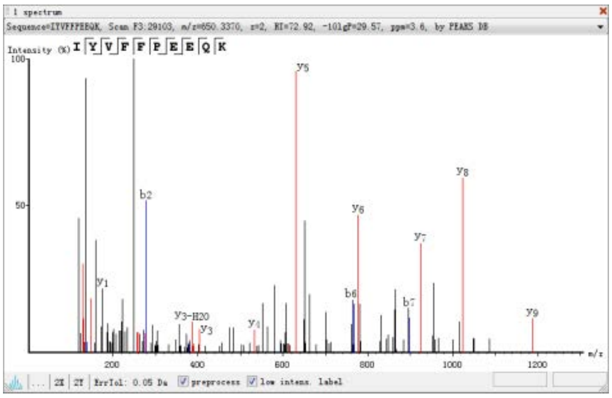

gi|225445664

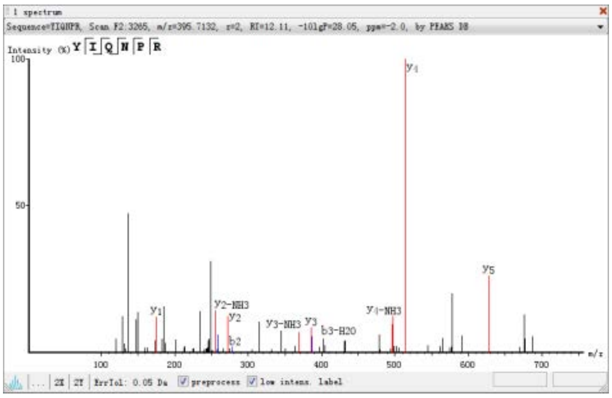

gi|225445842

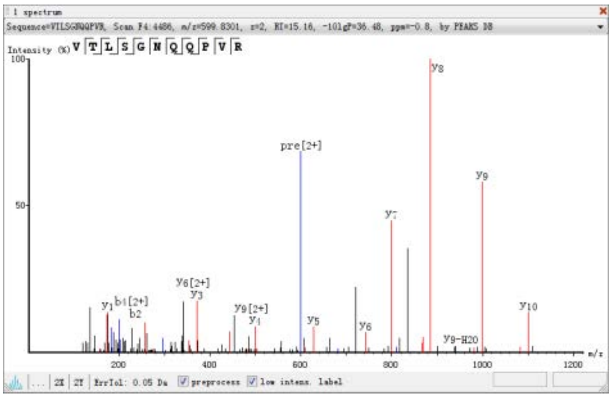

gi|225445883

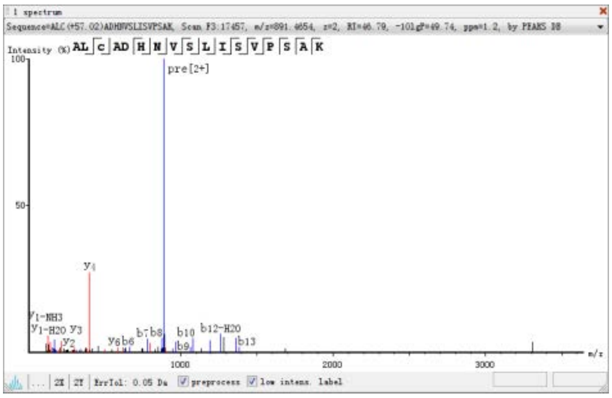

gi|225446046

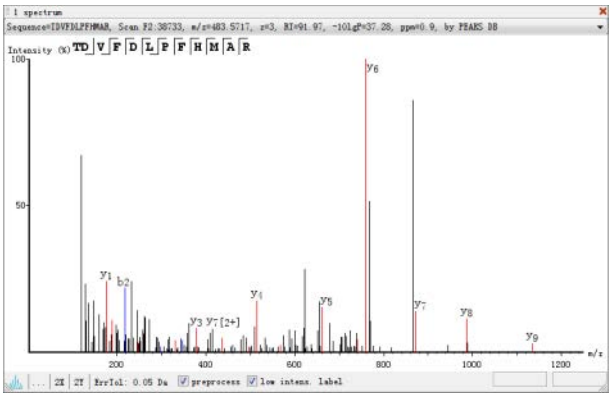

gi|225446899

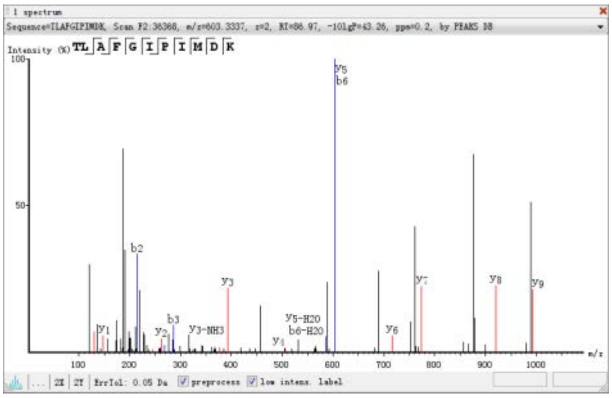

gi|225447510

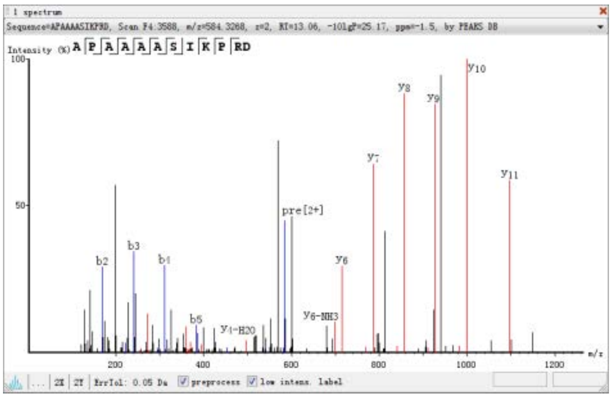

gi|225447584

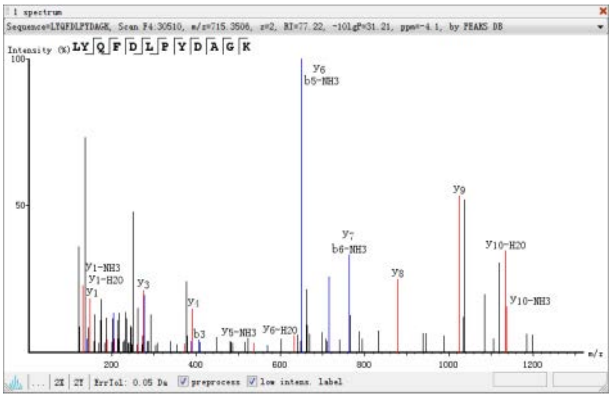

gi|225447707

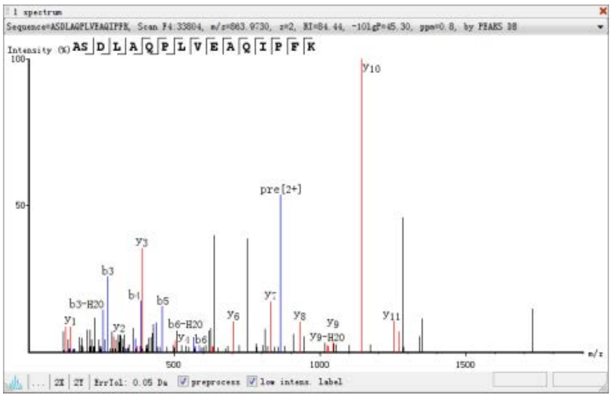

gi|225449078

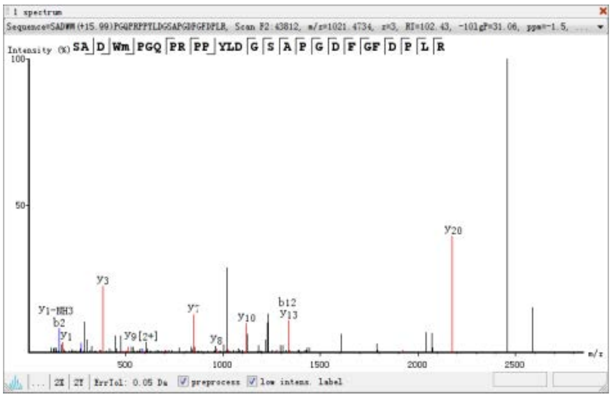

gi|225449424

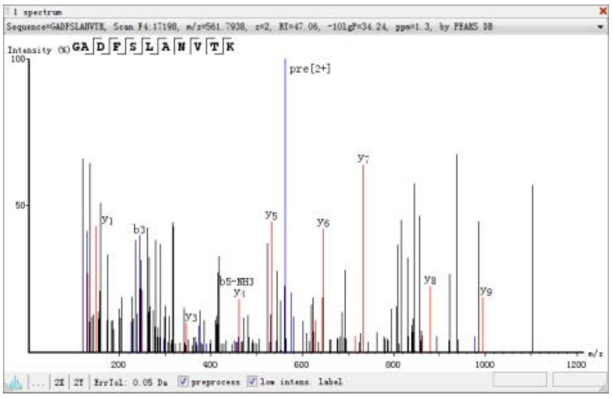

gi|225450051

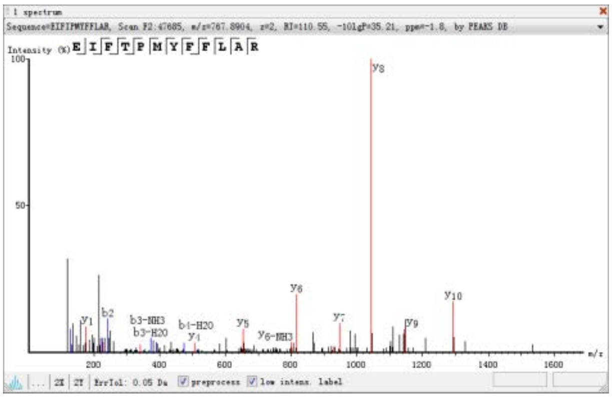

gi|225451233

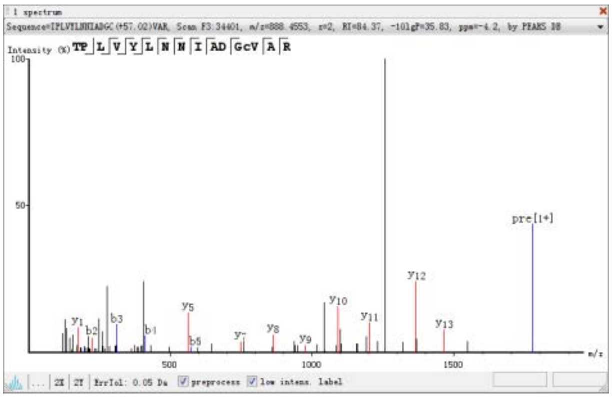

gi|225452228

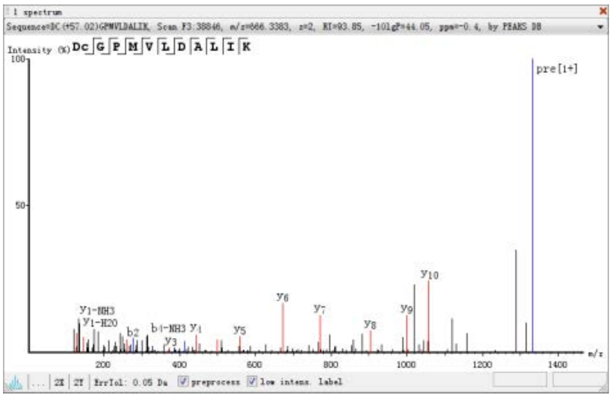

gi|225452328

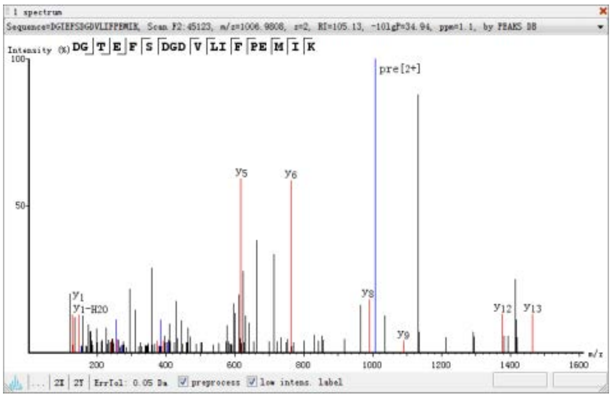

gi|225452562

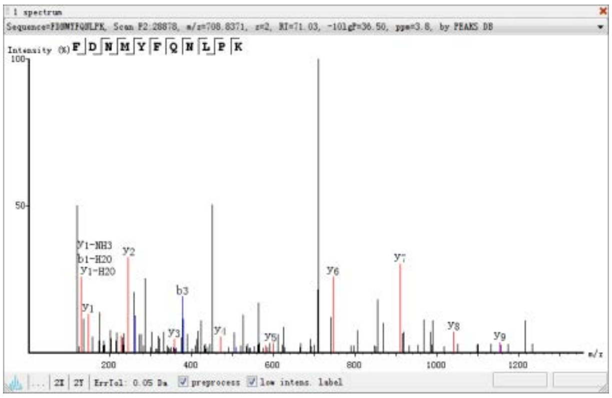

gi|225454791

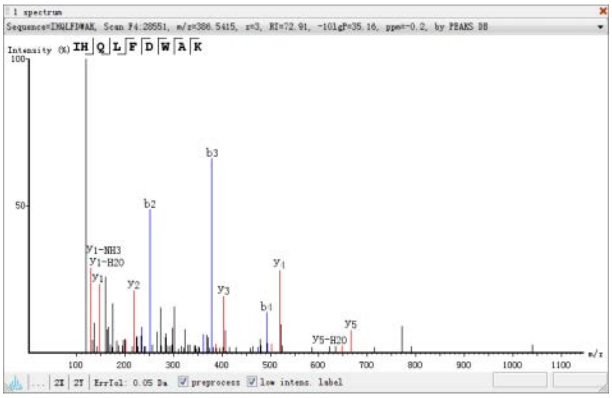

gi|225454994

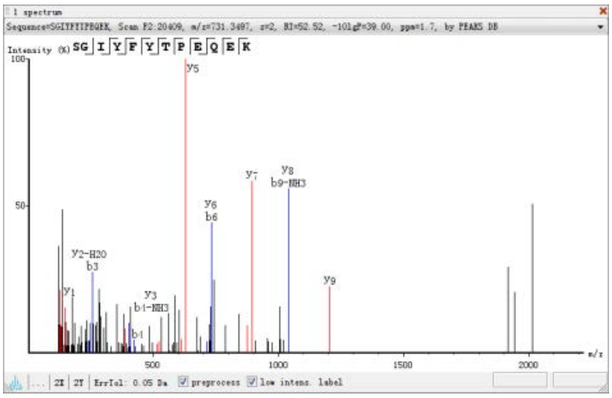

gi|225455232

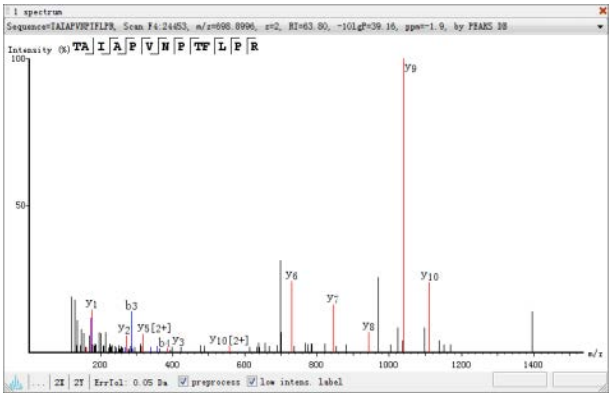

gi|225455386

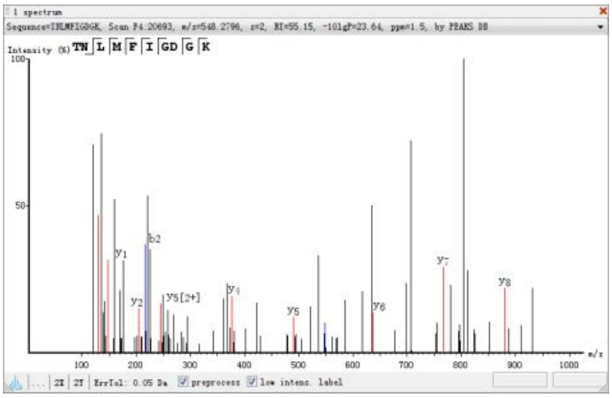

gi|225455902

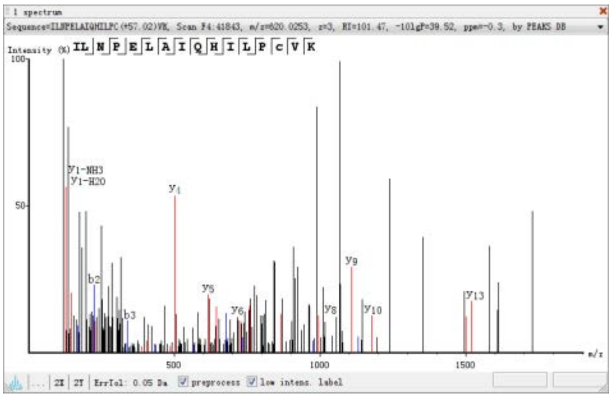

gi|225456262

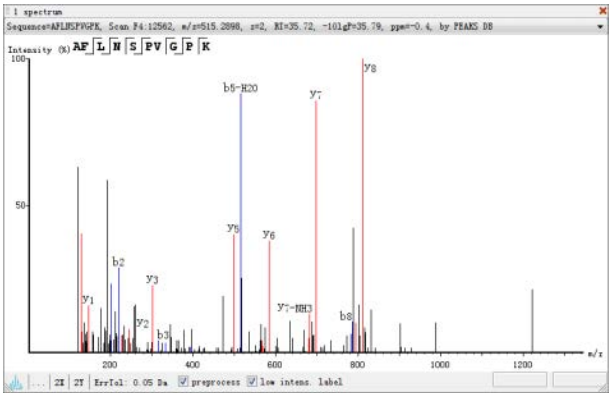

gi|225456459

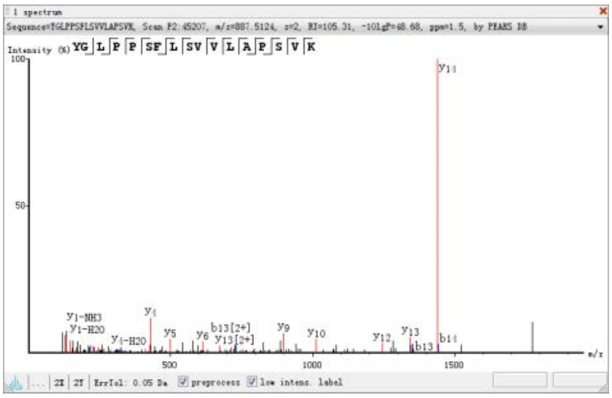

gi|225456641

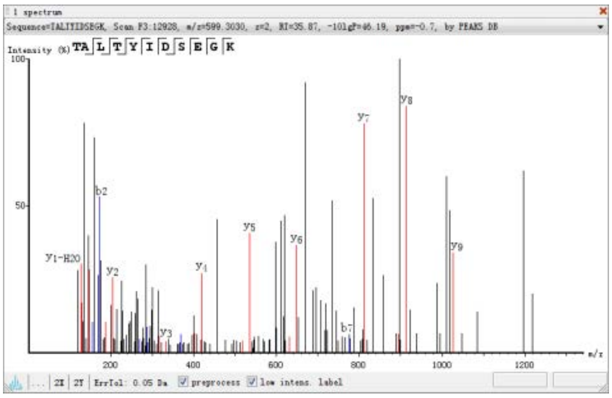

gi|225457502

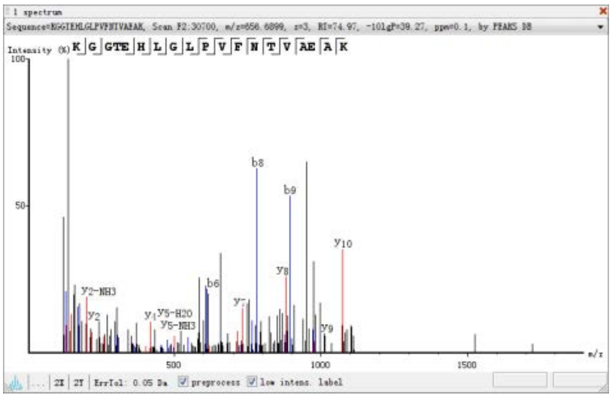

gi|225458719

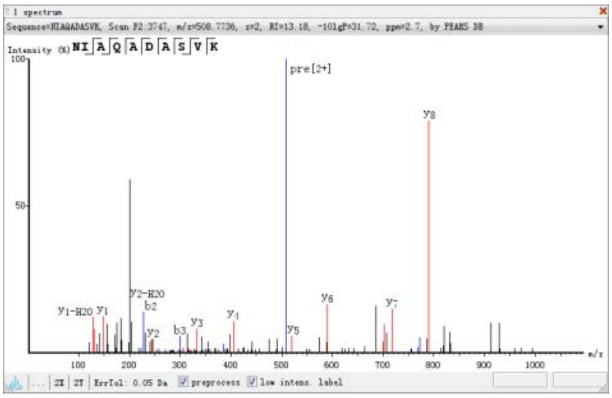

gi|225459126

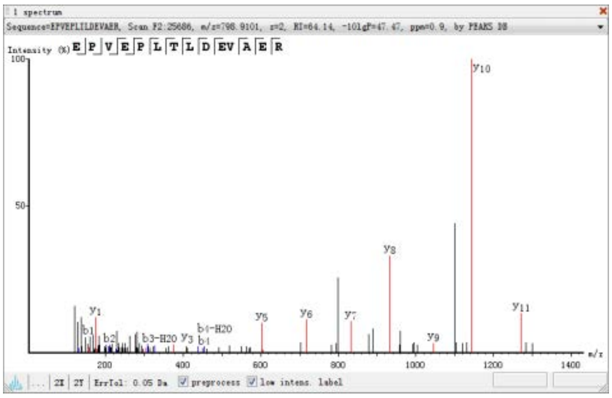

gi|225459360

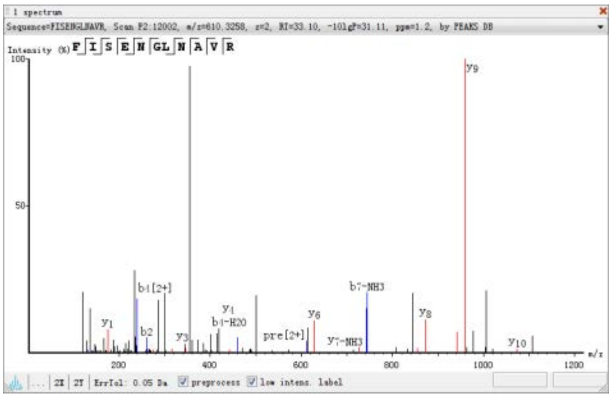

gi|225459808

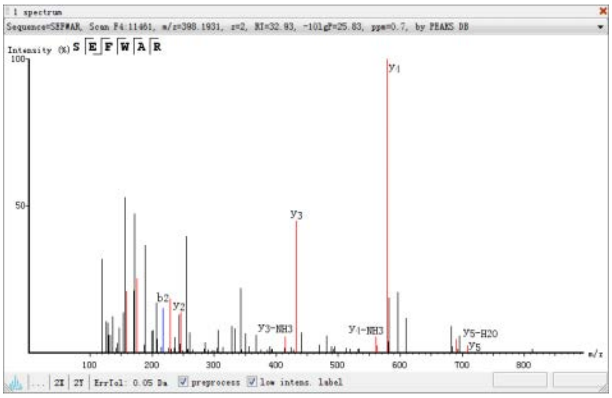

gi|225461906

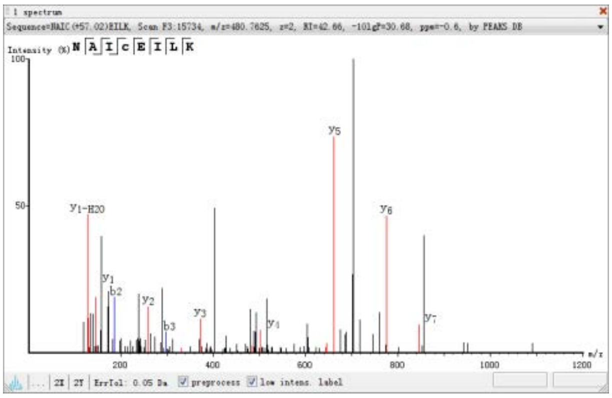

gi|225462922

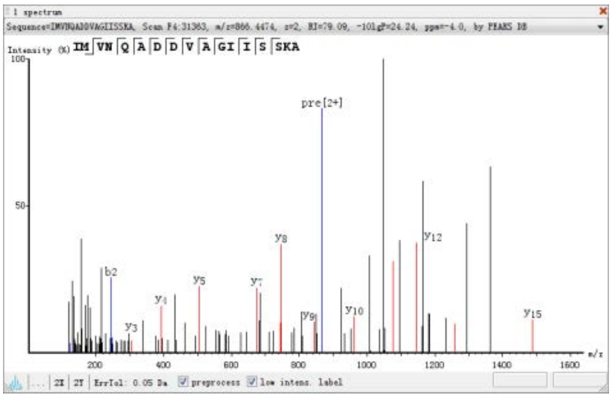

gi|225463163

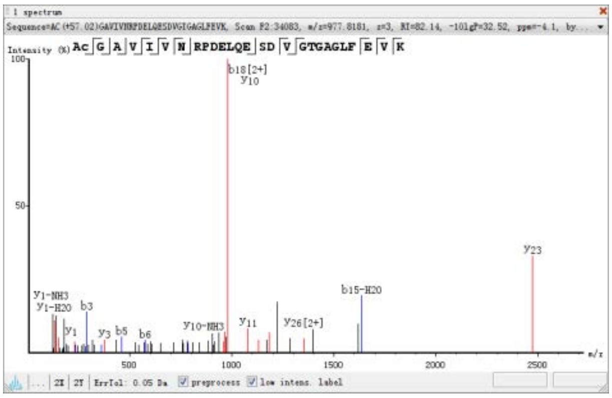

gi|225463253

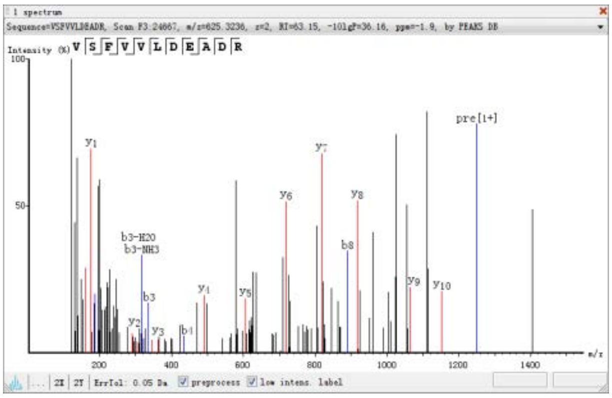

gi|225463556

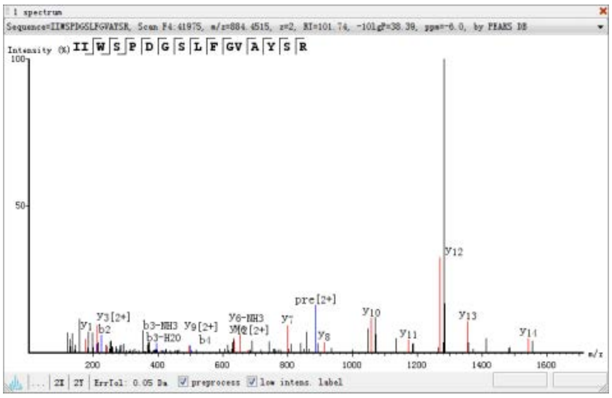

gi|225465259

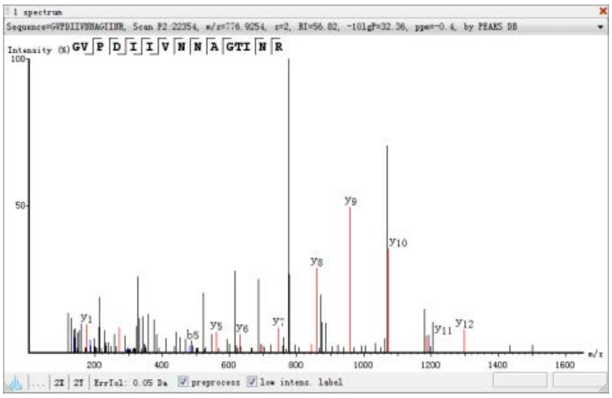

gi|225465945

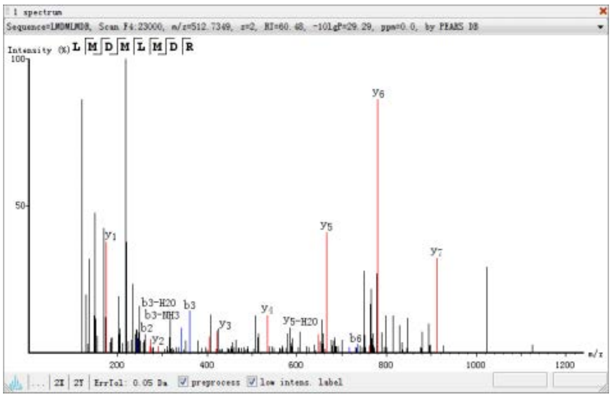

gi|359473789

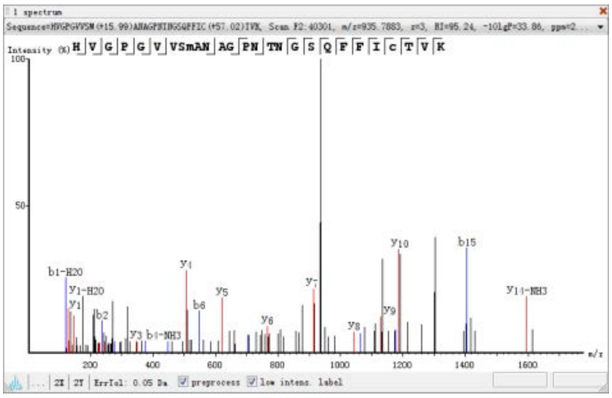

gi|359476269

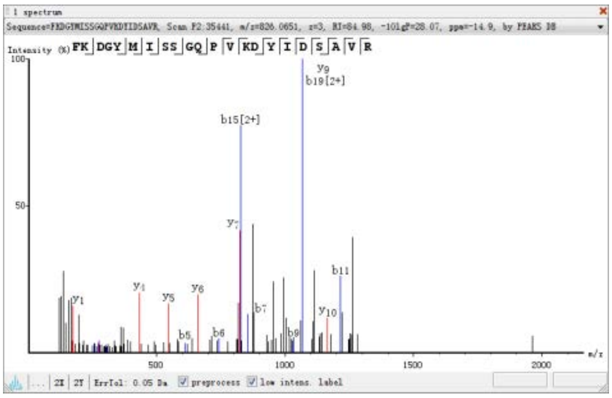

gi|359476926

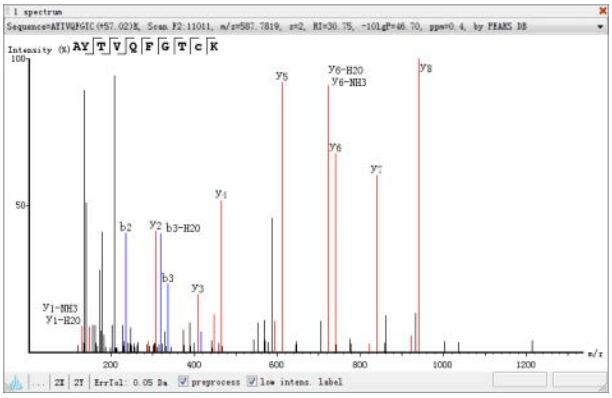

gi|359478076

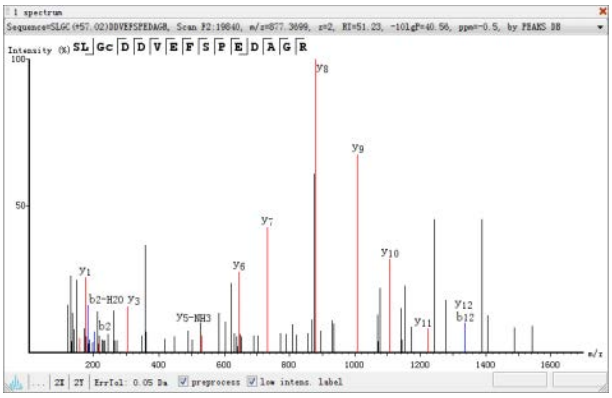

gi|359478415

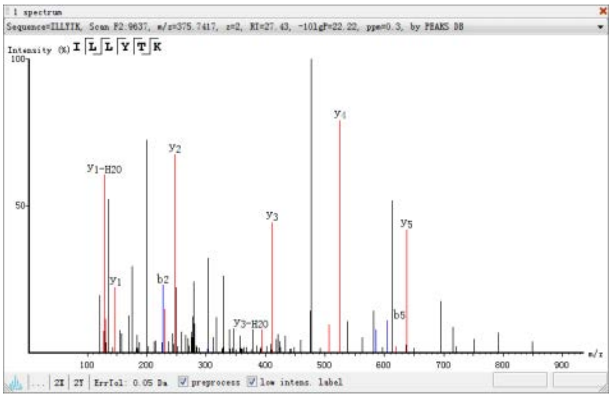

gi|359480225

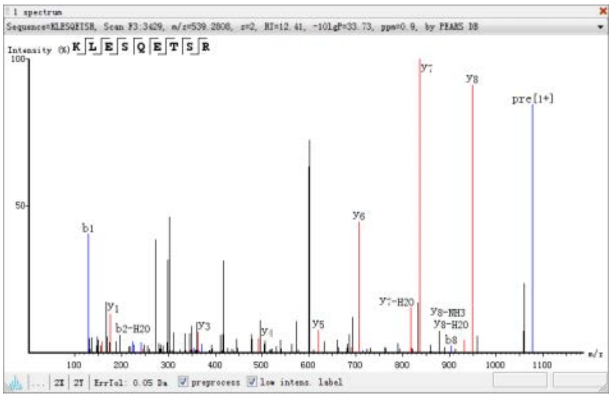

gi|359482256

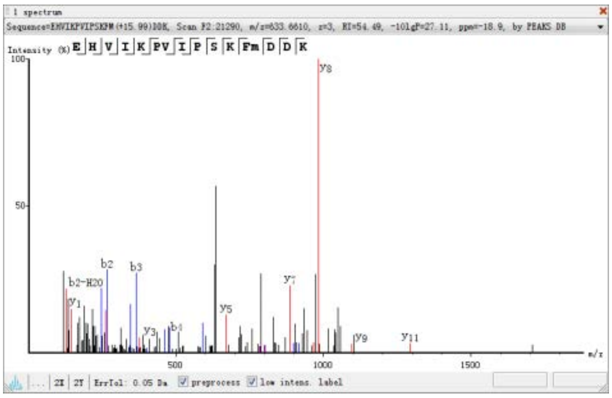

gi|359482834

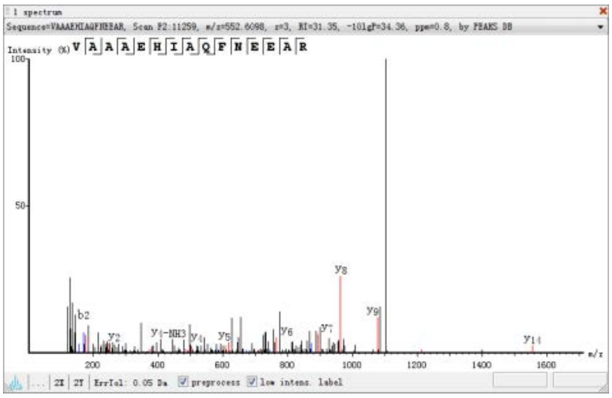

gi|359486976

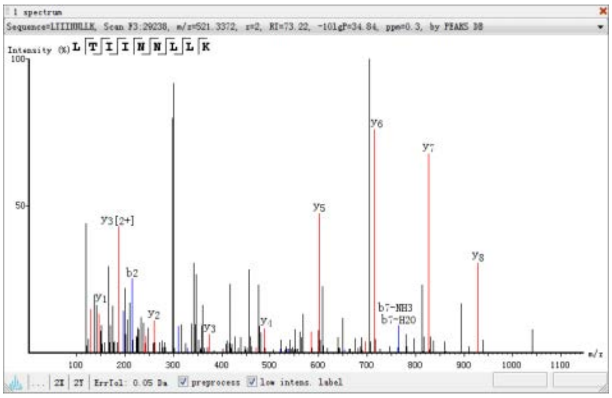

gi|359487708

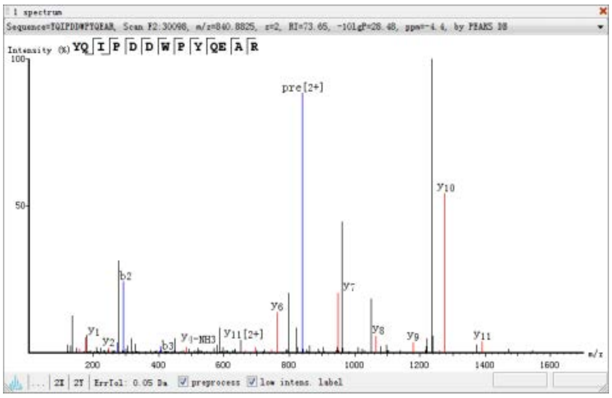

gi|359487733

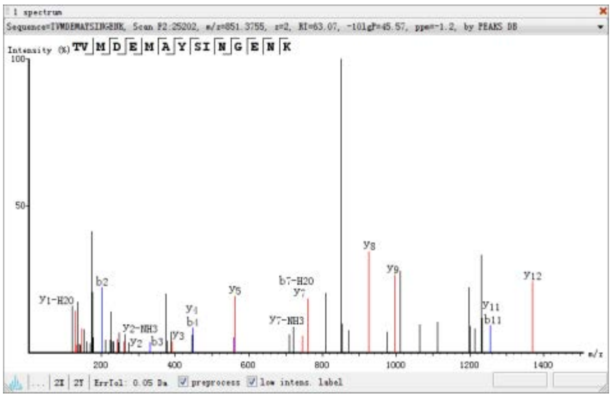

gi|359489456

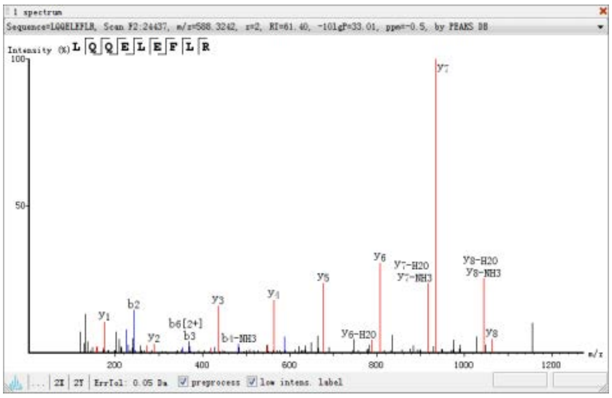

gi|359492510

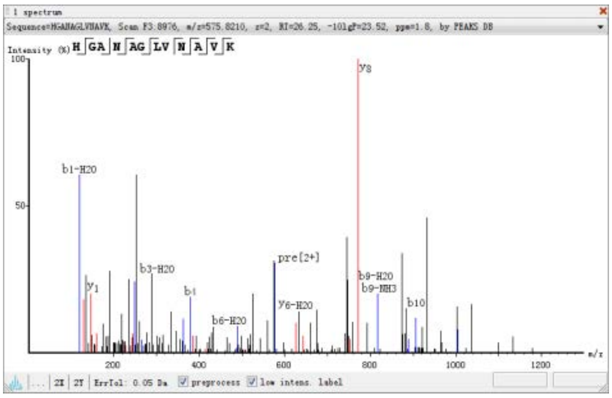

gi|359494696

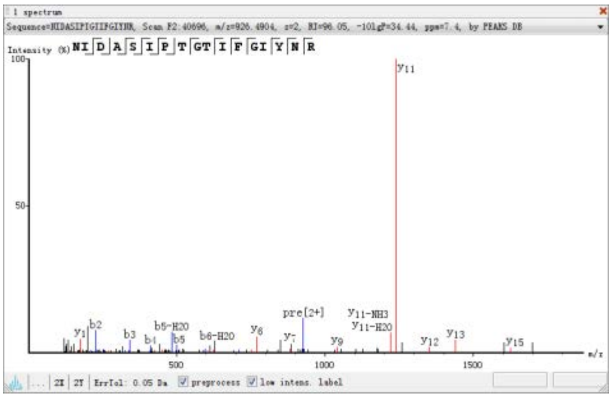

gi|359495836

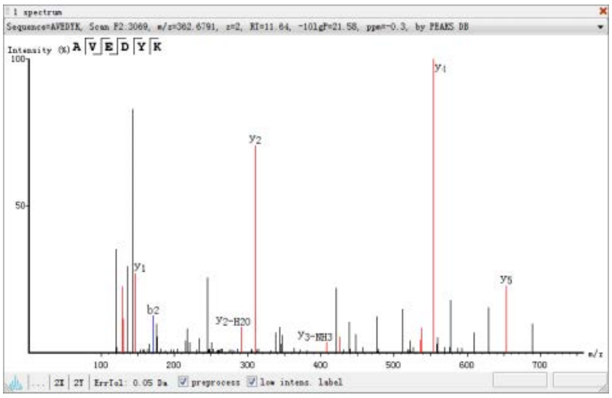

gi|359497879

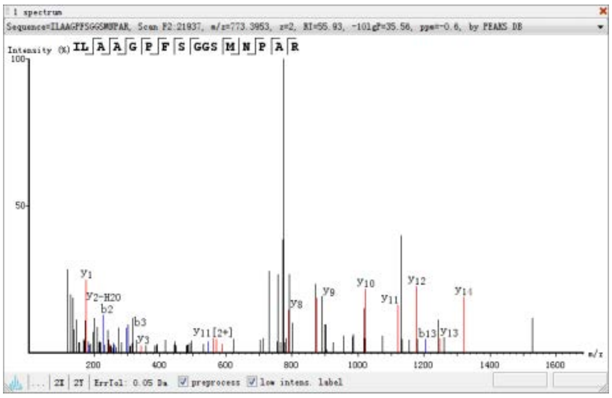

gi|526117331

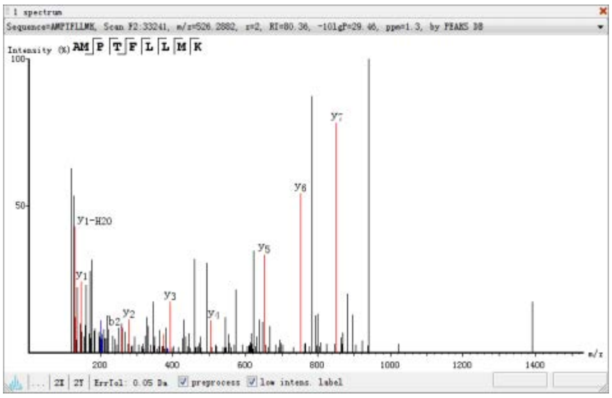

gi|526117729

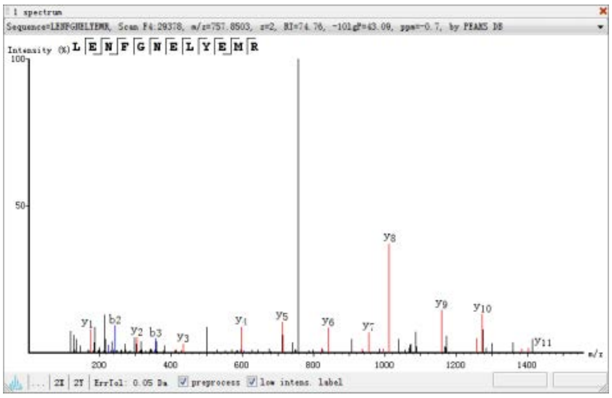

gi|526117964

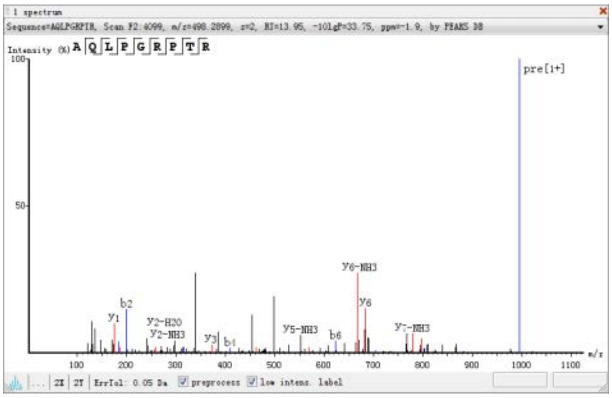

gi|566146661

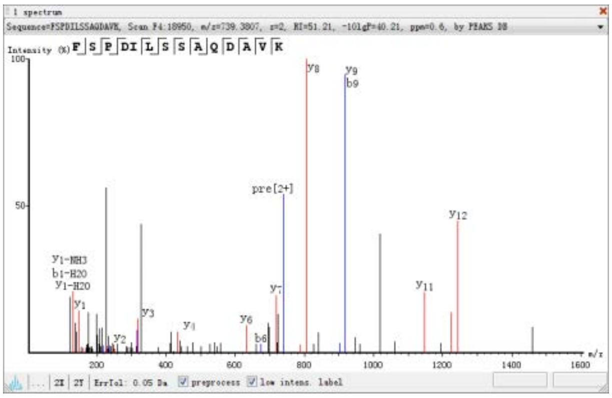

gi|566148044

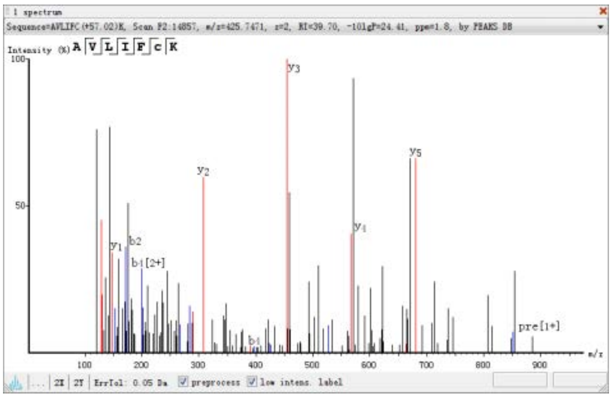

gi|566148676

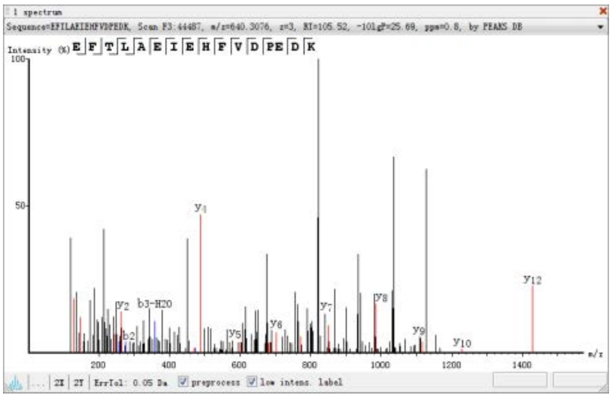

gi|566149094

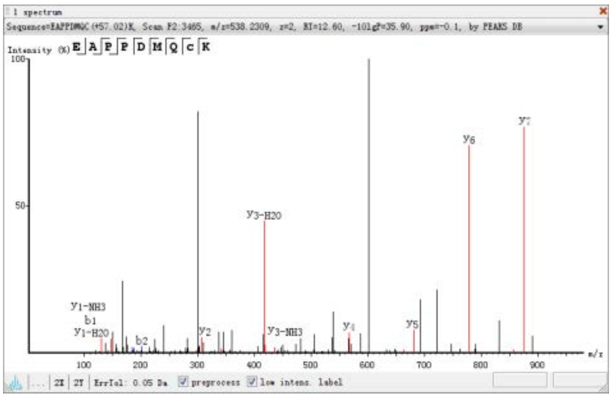

gi|566149831

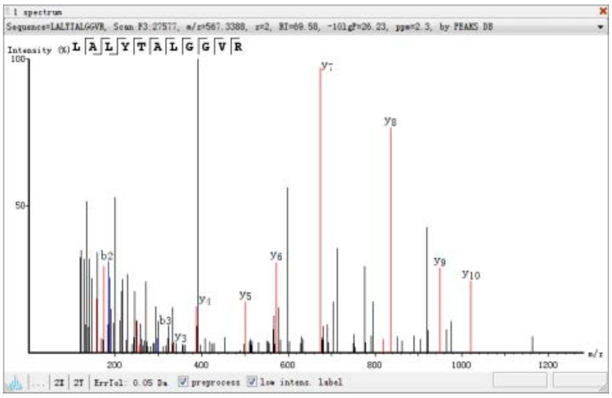

gi|566150858

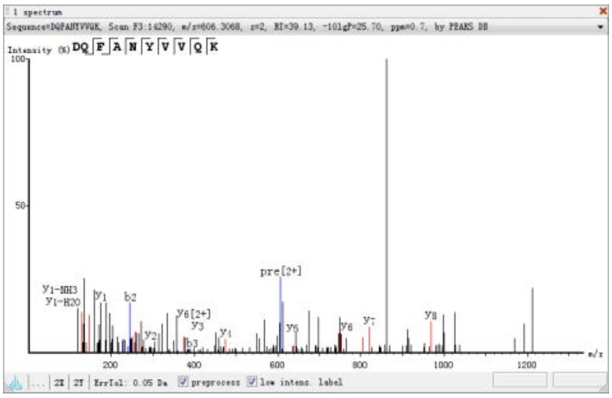

gi|566153514

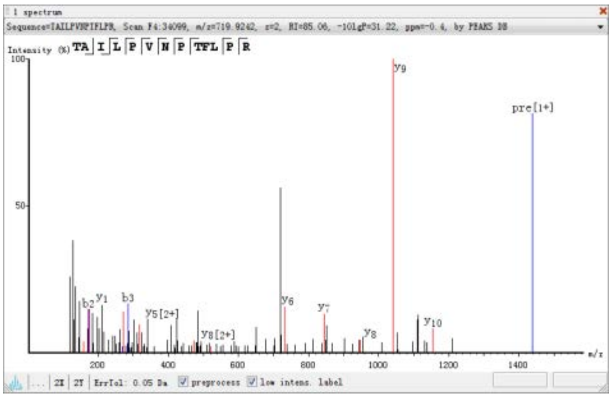

gi|566156766

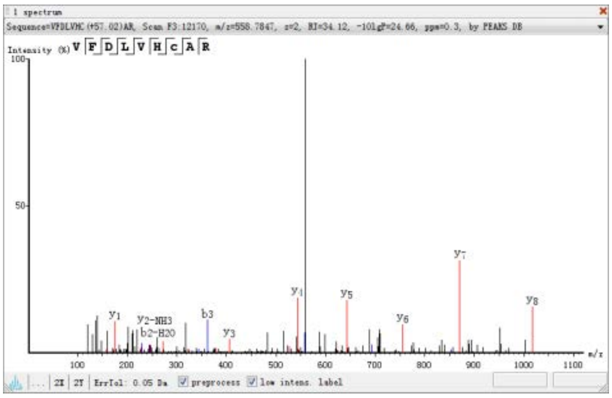

gi|566157027

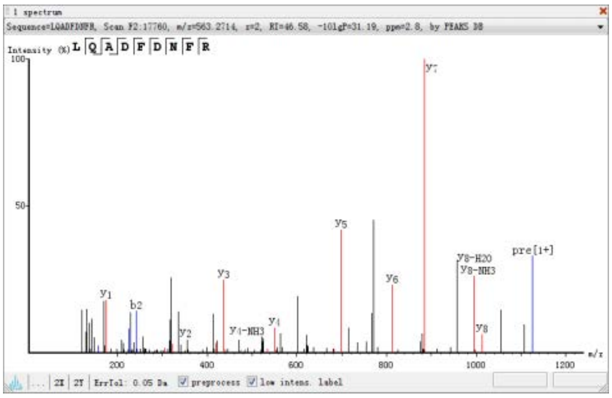

gi|566157463

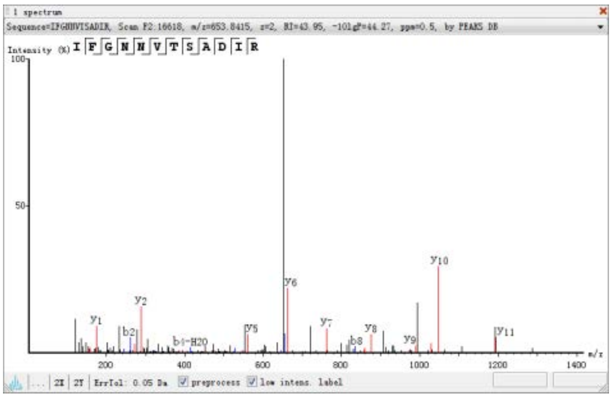

gi|566159456

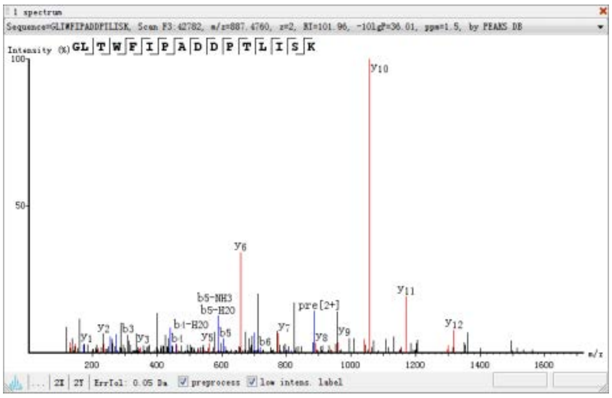

gi|566160209

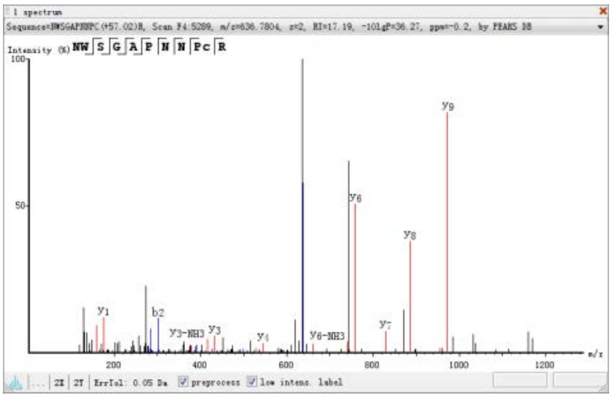

gi|566160505

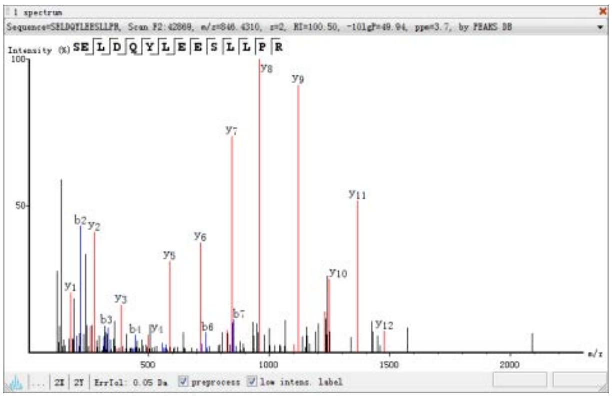

gi|566160621

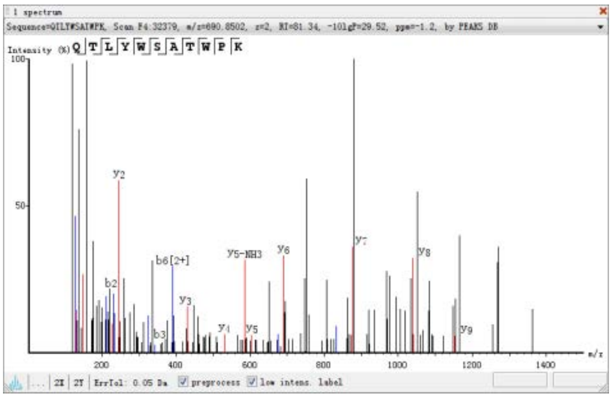

gi|566160631

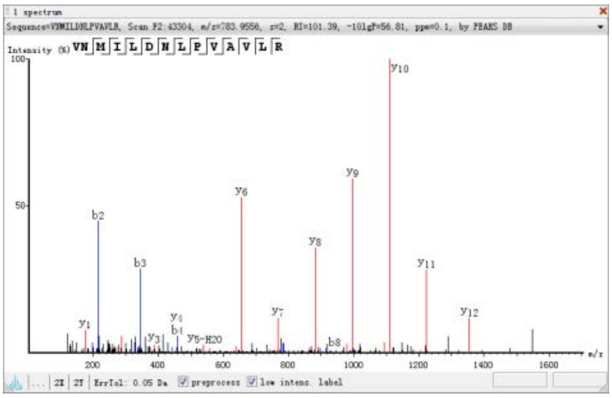

gi|566161176

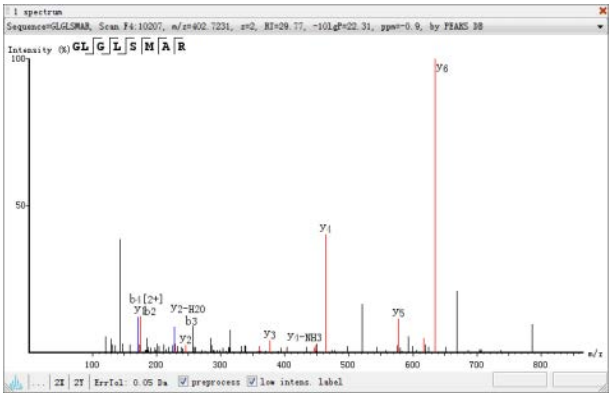

gi|566161648

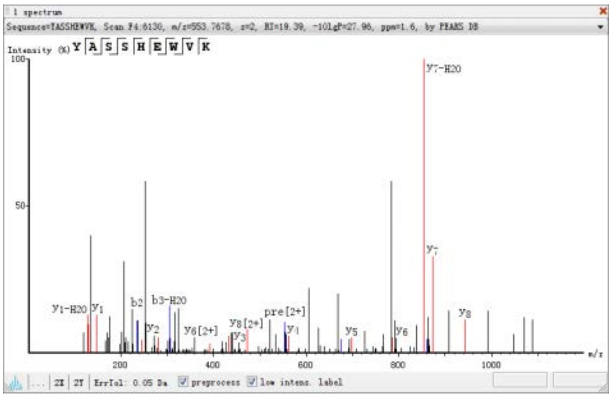

gi|566161892

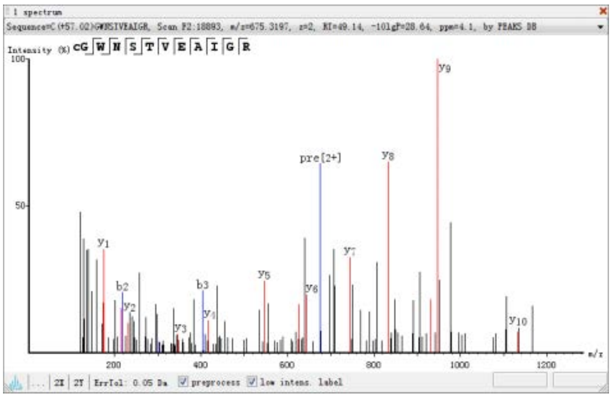

gi|566167505

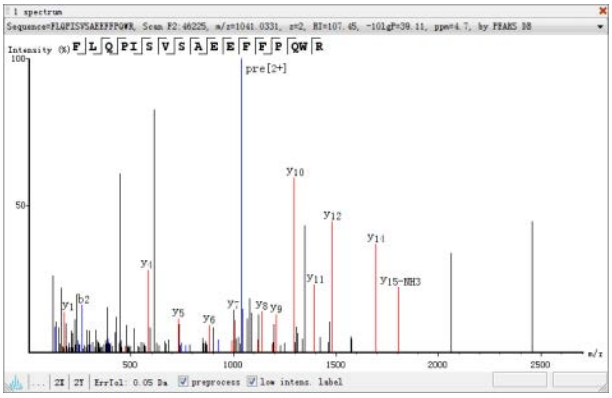

gi|566168071

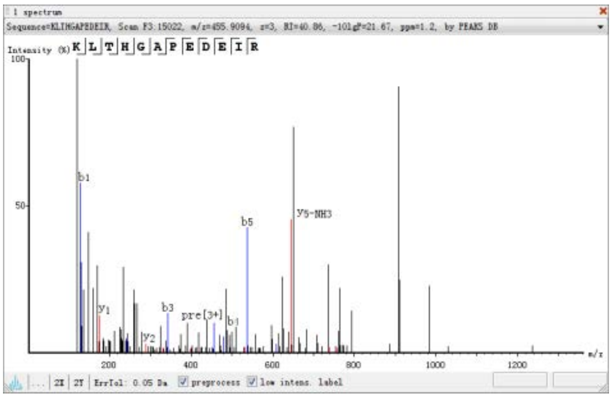

gi|566168111

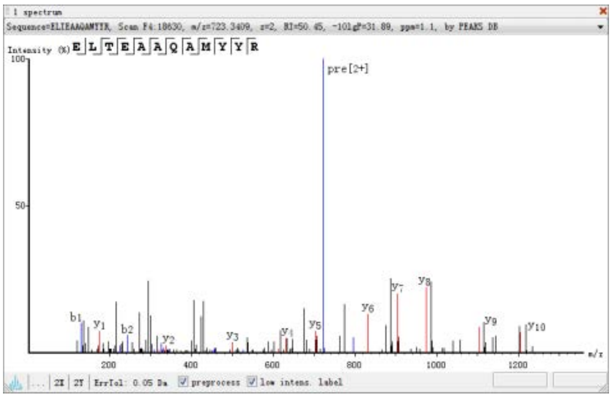

gi|566168611

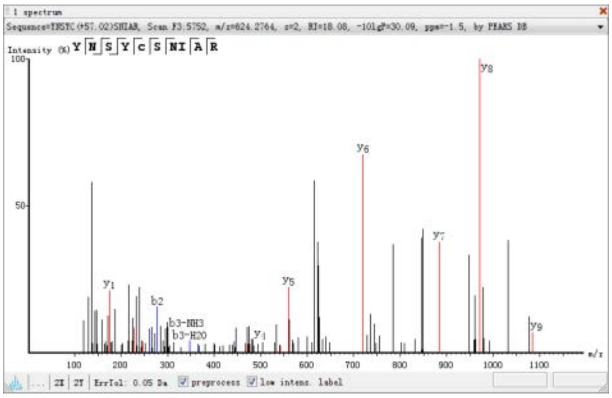

gi|566170203

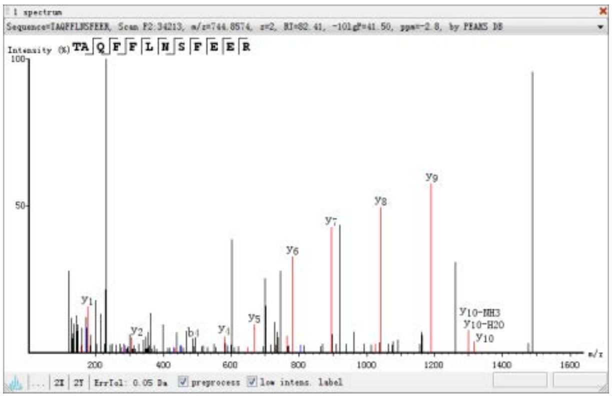

gi|566170807

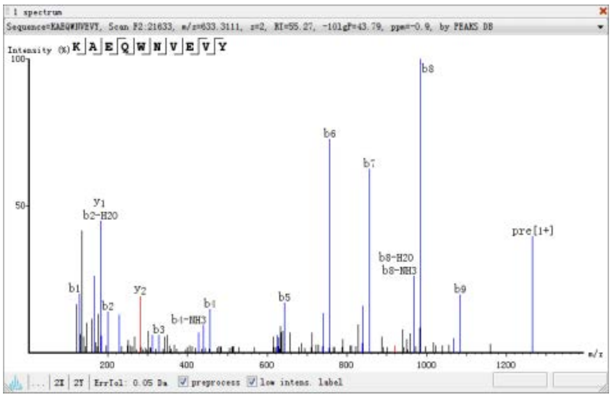

gi|566170882

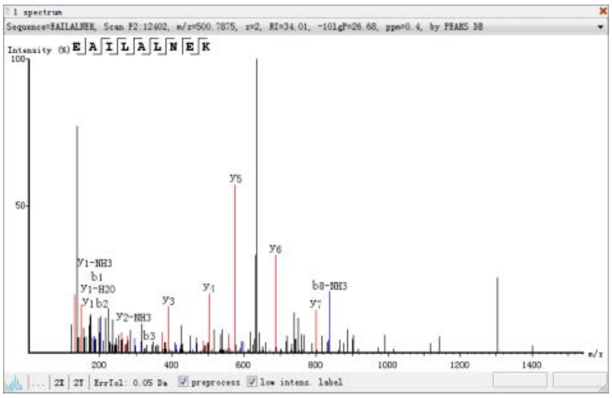

gi|566171760

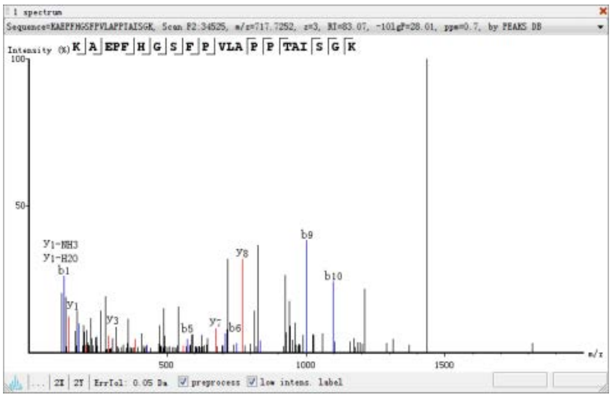

gi|566173207

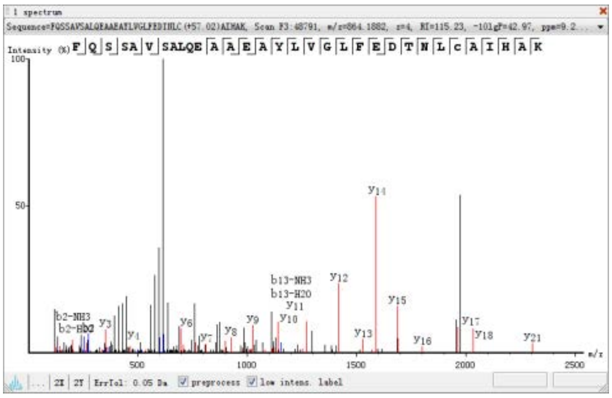

gi|566173751

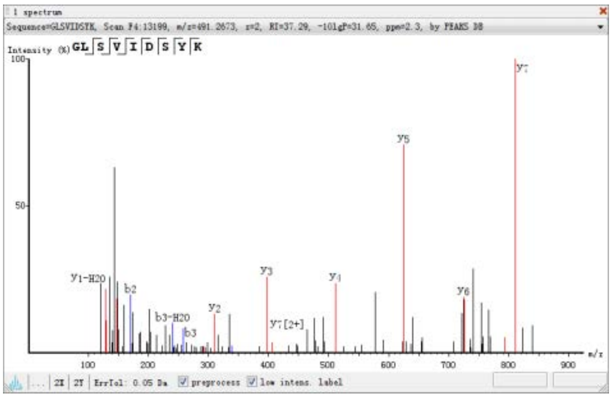

gi|566174372

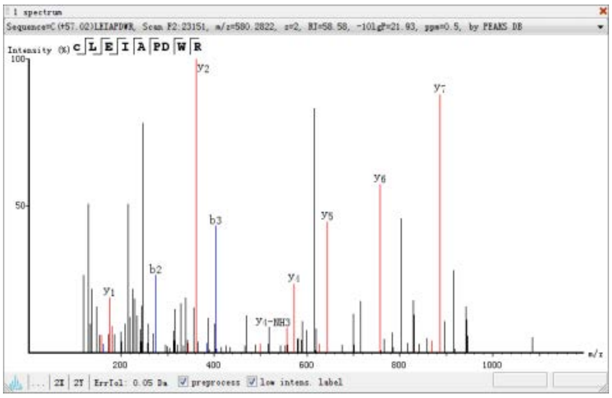

gi|566175530

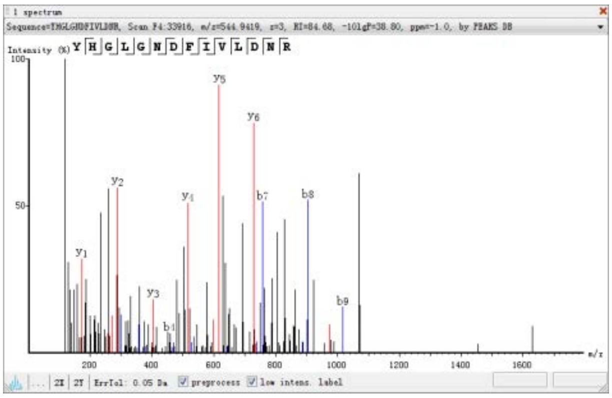

gi|566176113

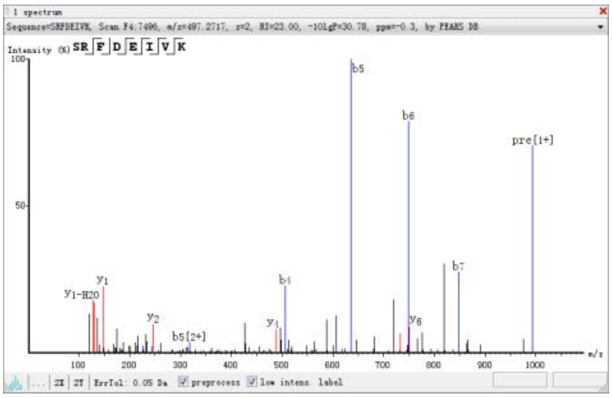

gi|566177300

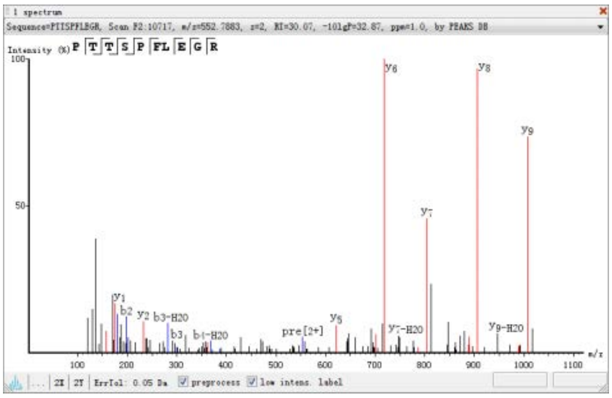

gi|566181315

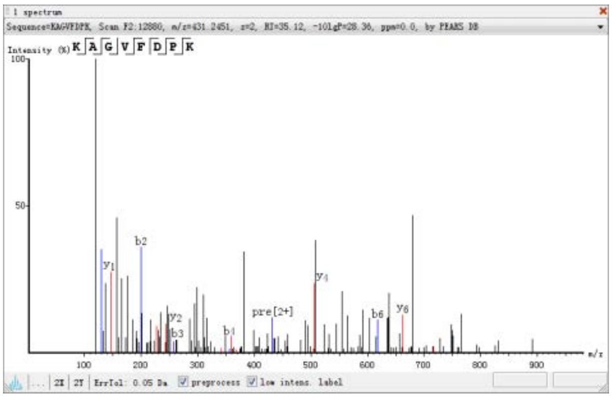

gi|566181342

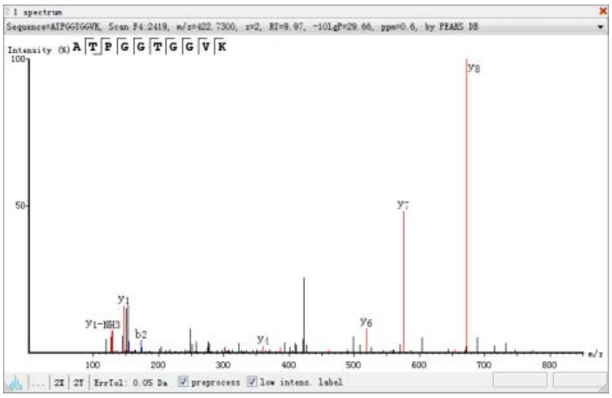

gi|566185250

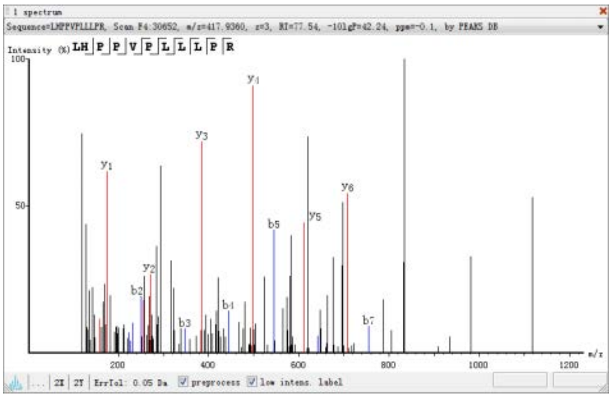

gi|566185272

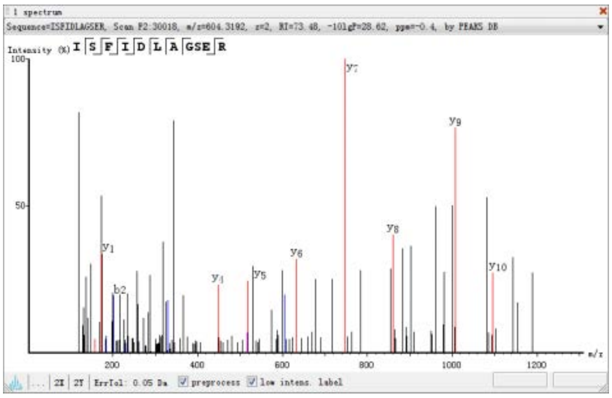

gi|566185685

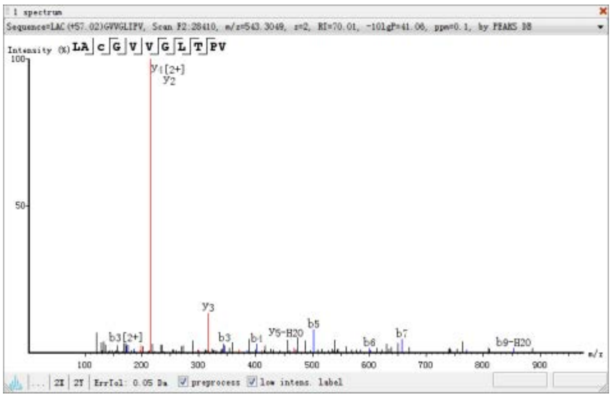

gi|566188099

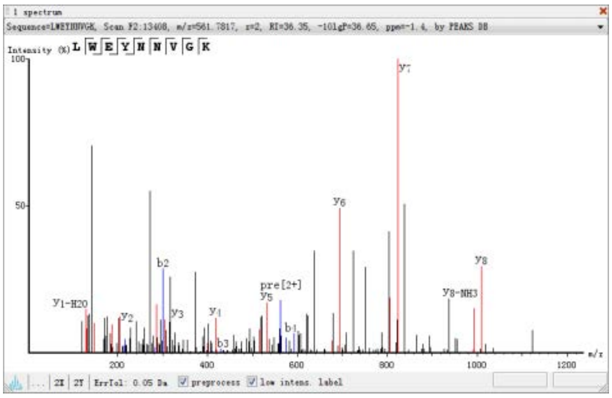

gi|566188113

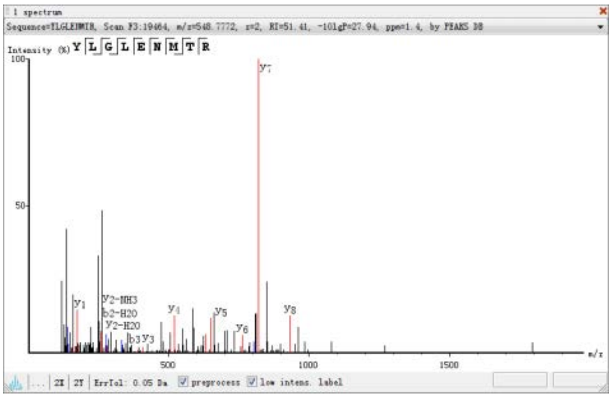

gi|566188433

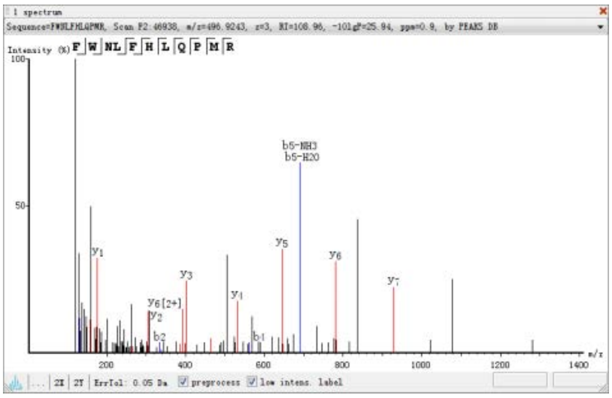

gi|566188774

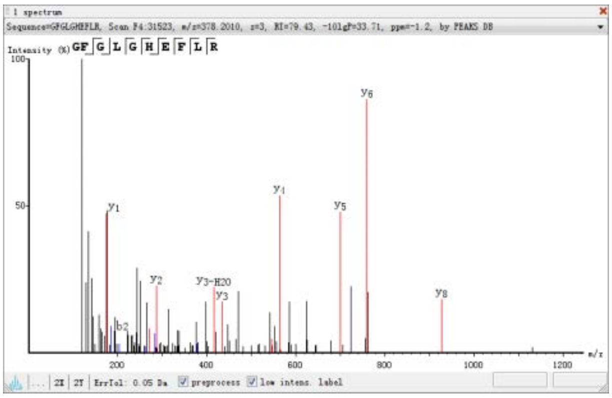

gi|566191728

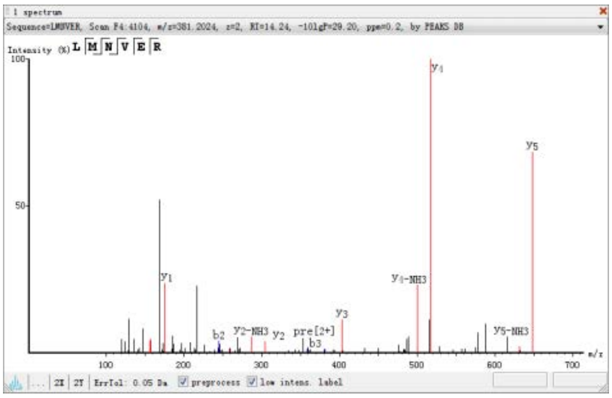

gi|566195508

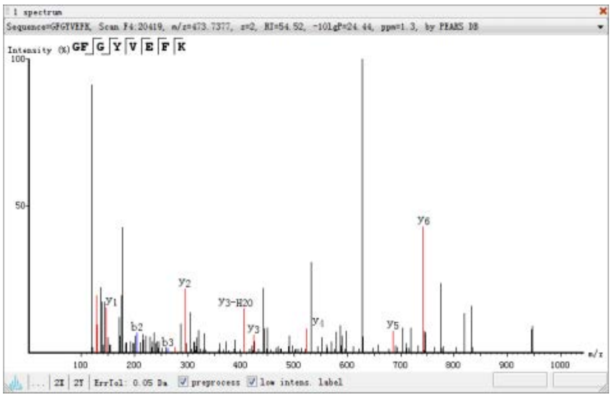

gi|566195934

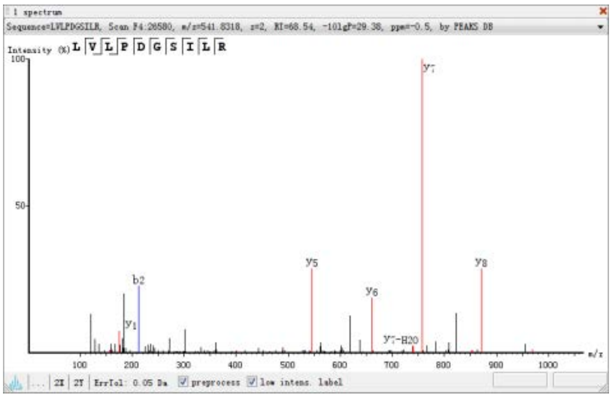

gi|566197446

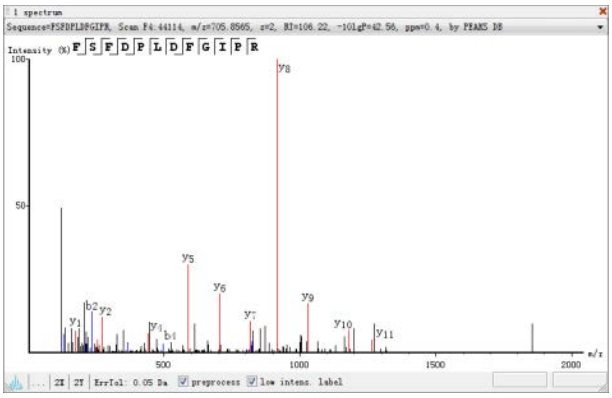

gi|566197644

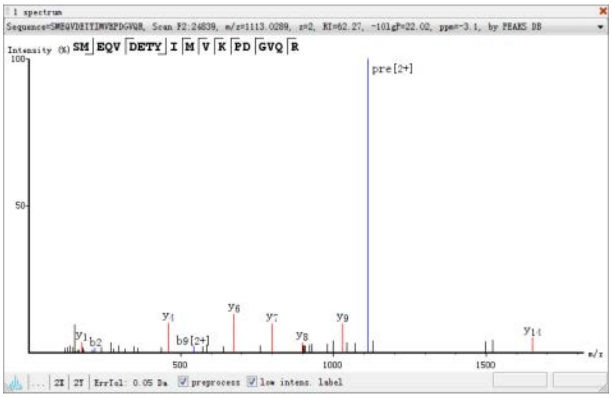

gi|566199681

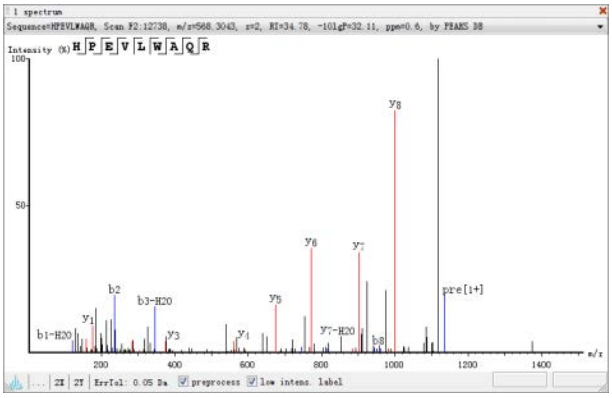

gi|566200837

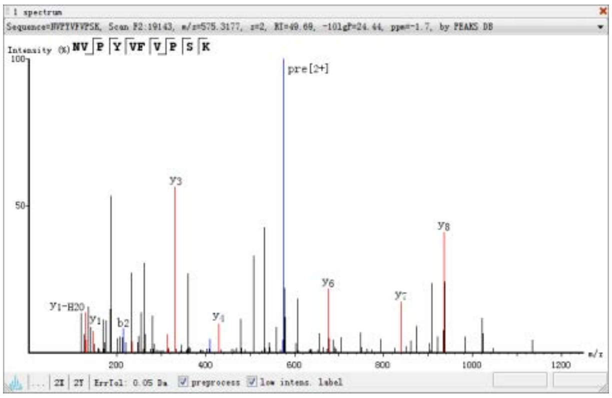

gi|566201939

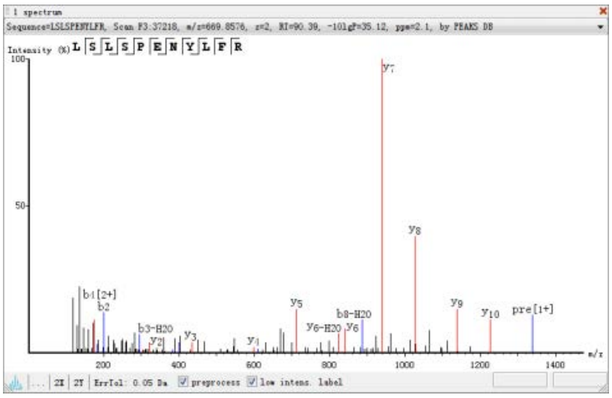

gi|566204034

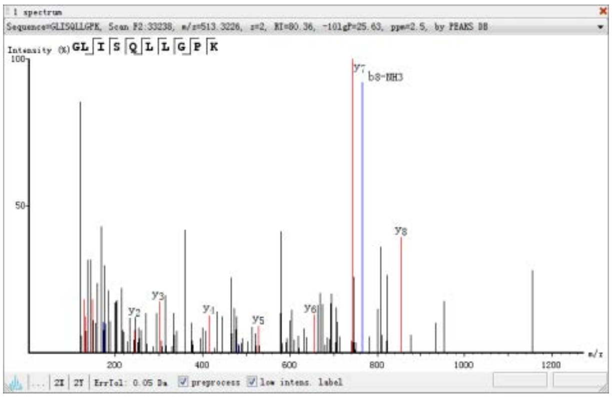

gi|566204149

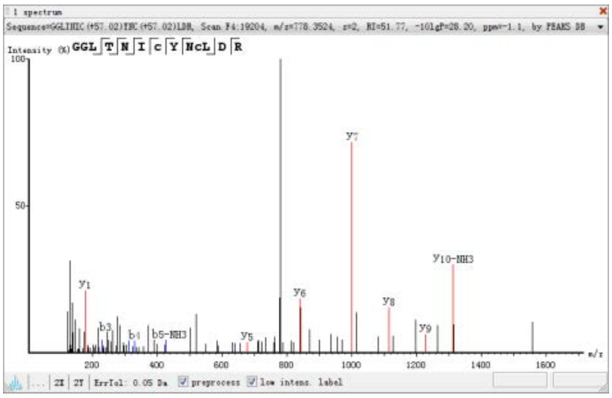

gi|566205586

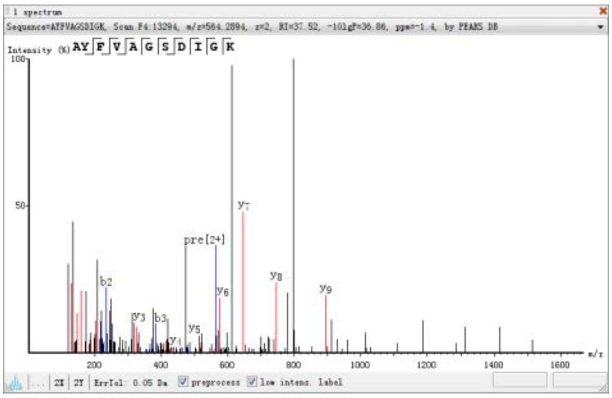

gi|566206247

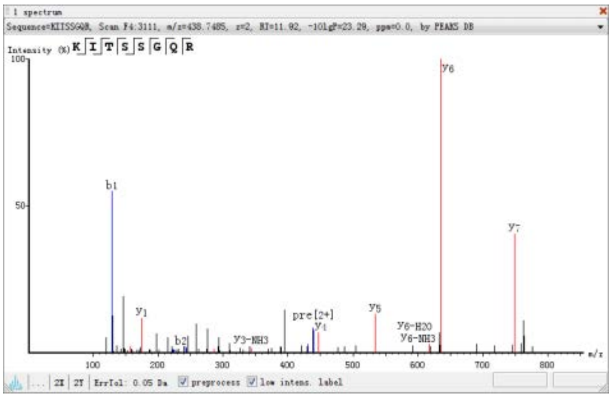

gi|566207660

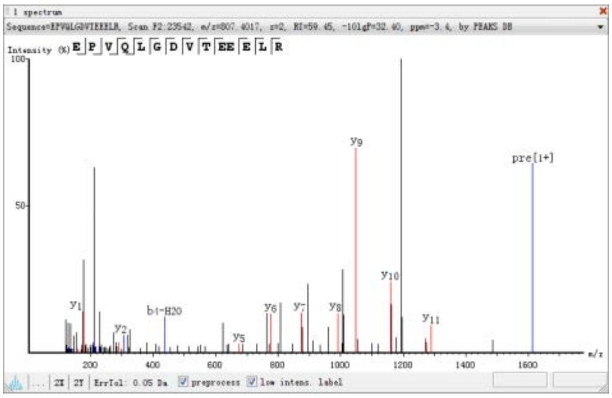

gi|566207939

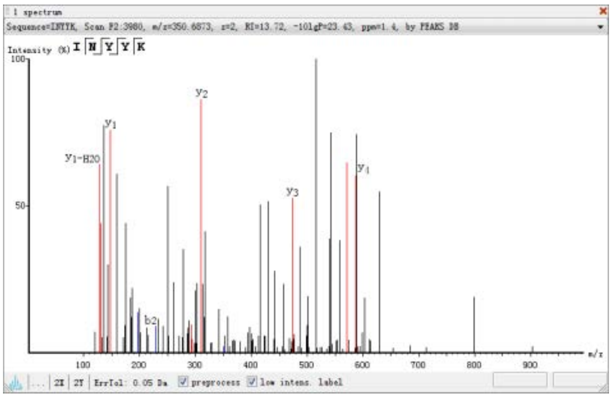

gi|566208589

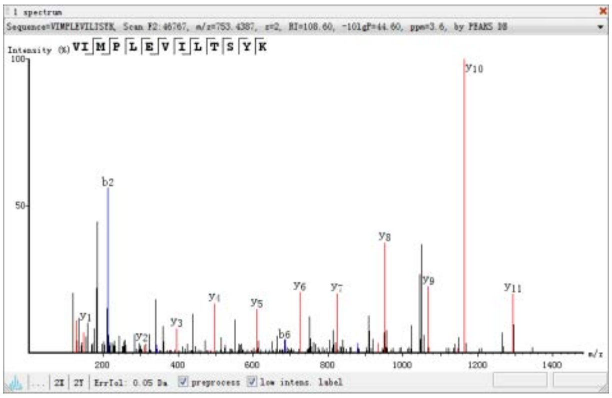

gi|566208913

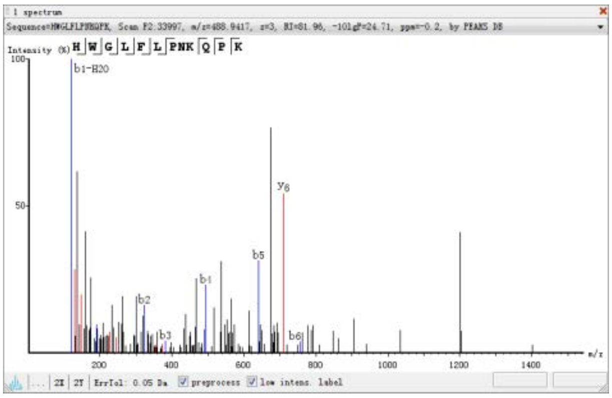

gi|566209743

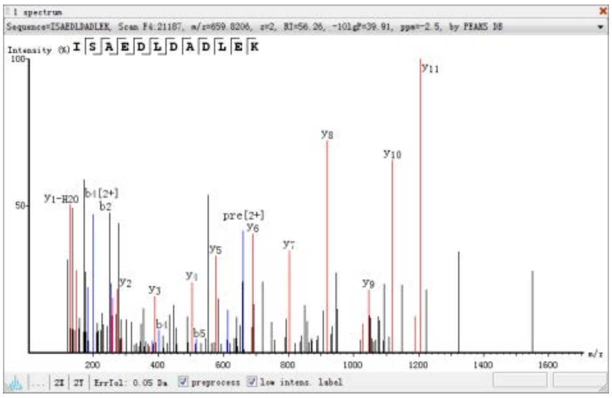

gi|566210049

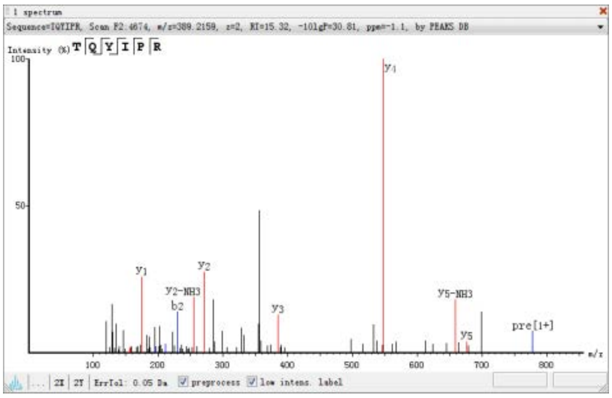

gi|566210060

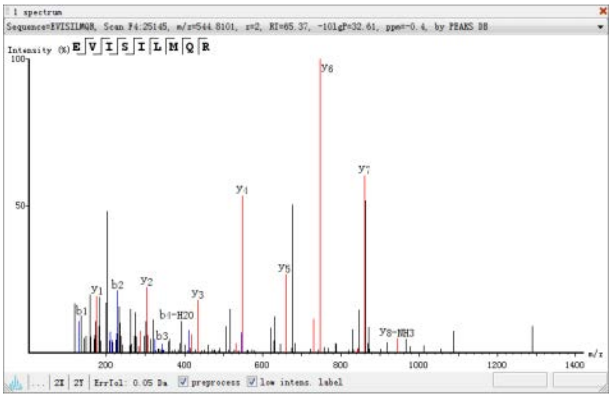

gi|566210666

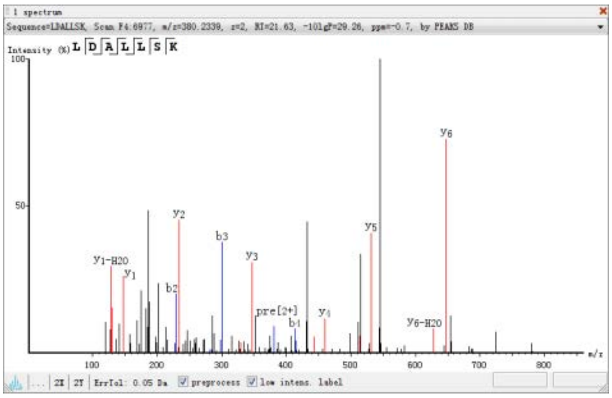

gi|566211354

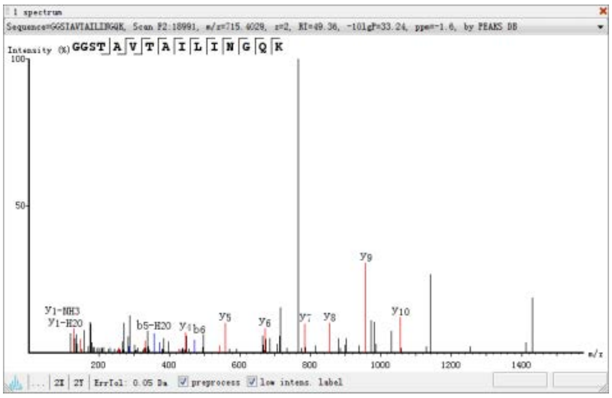

gi|566212877

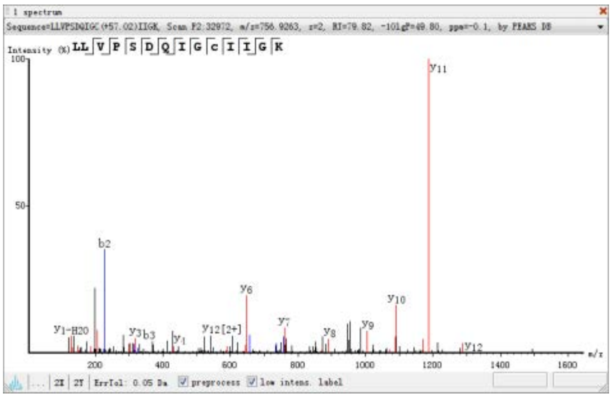

gi|566213932

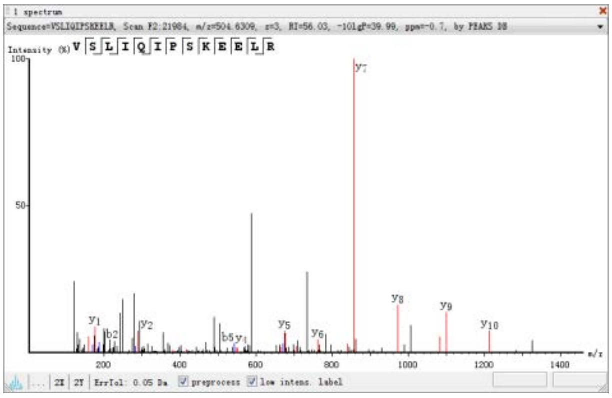

gi|566214115

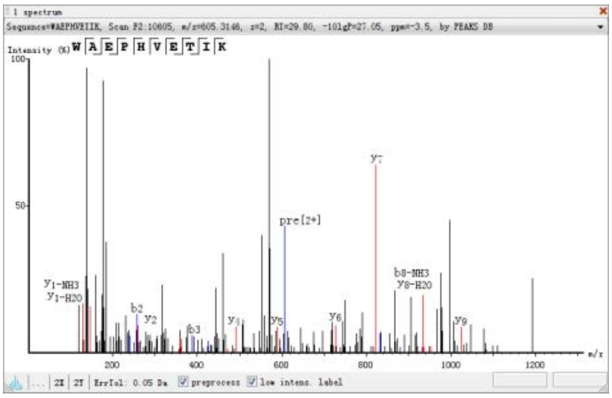

gi|566215175

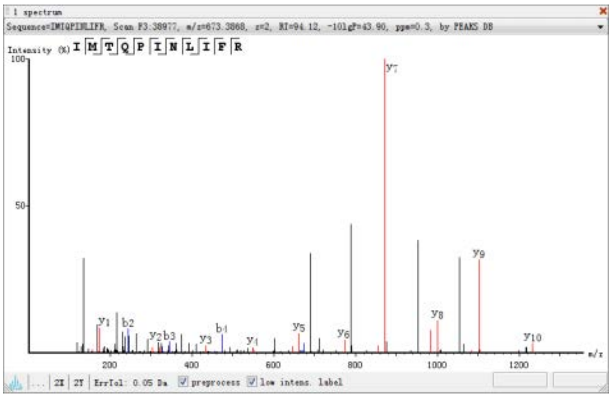

gi|566215907

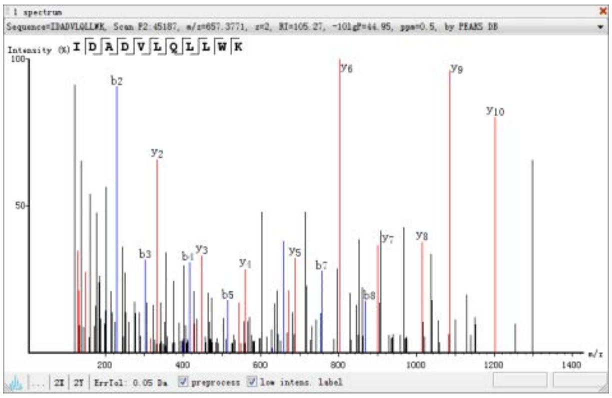

gi|566245781

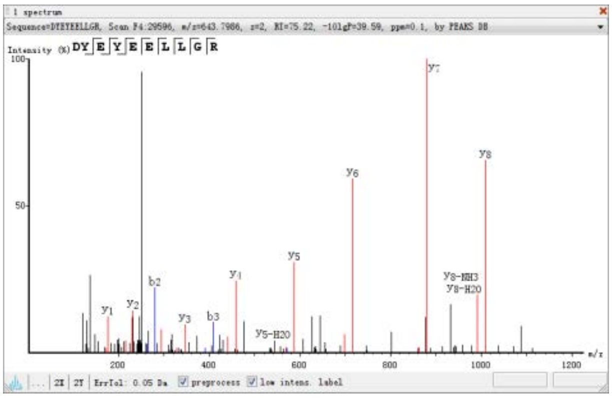

gi|566253285

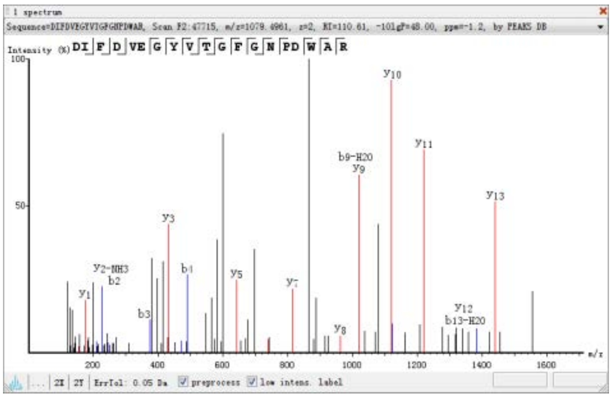

gi|566256635

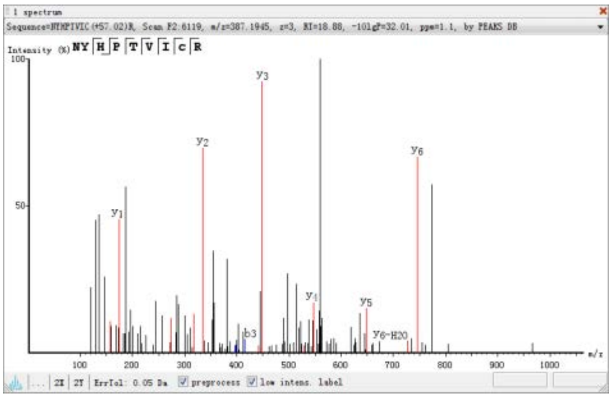

gi|566259876

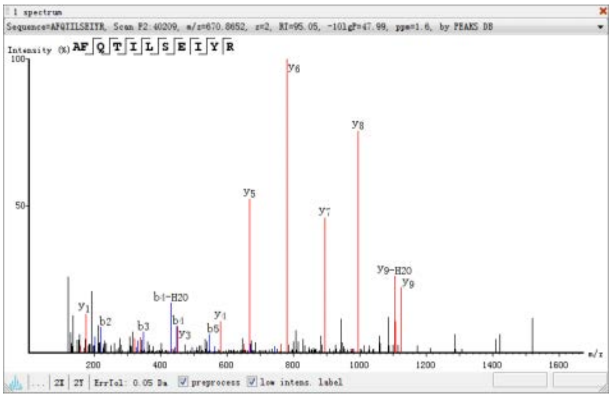

gi|671743286

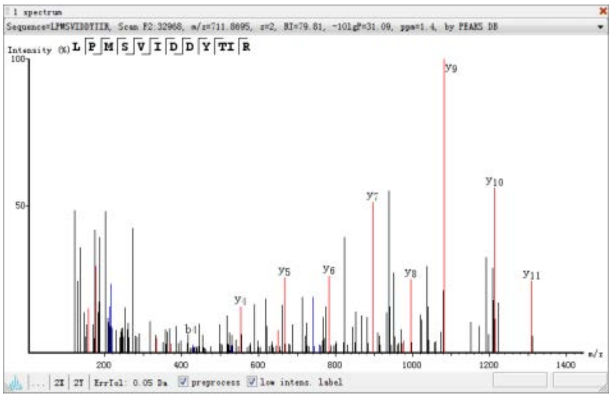

gi|731370548

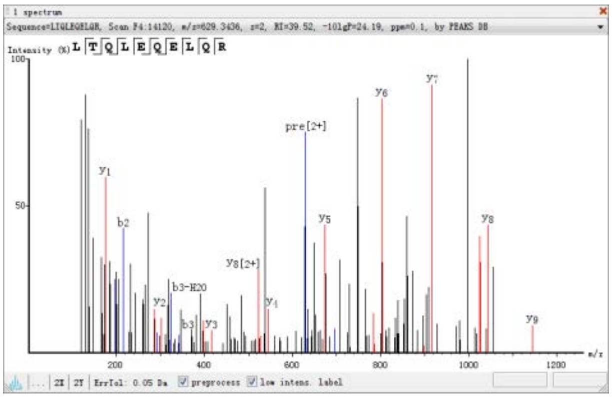

gi|731370779

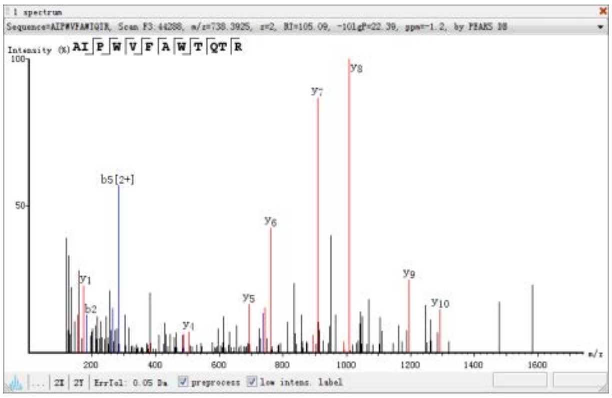

gi|731380670

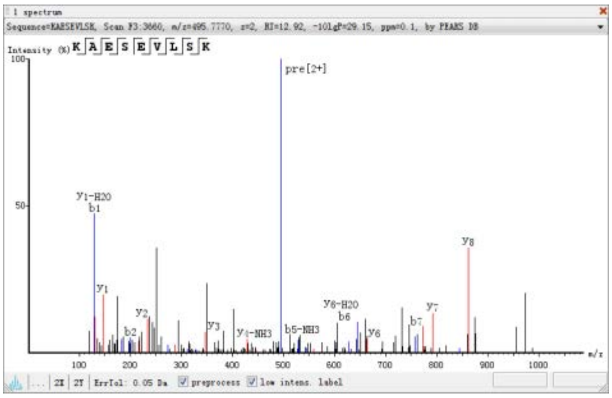

gi|731381391

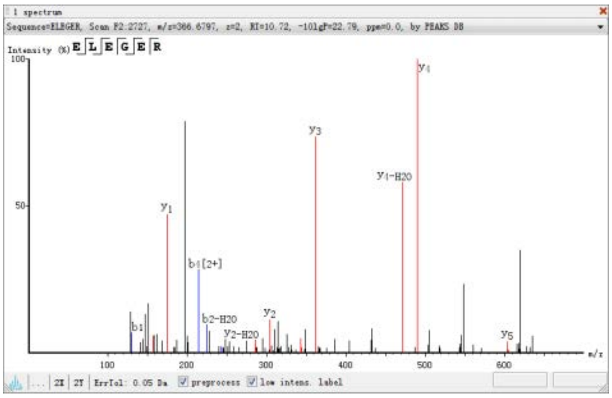

gi|731382683

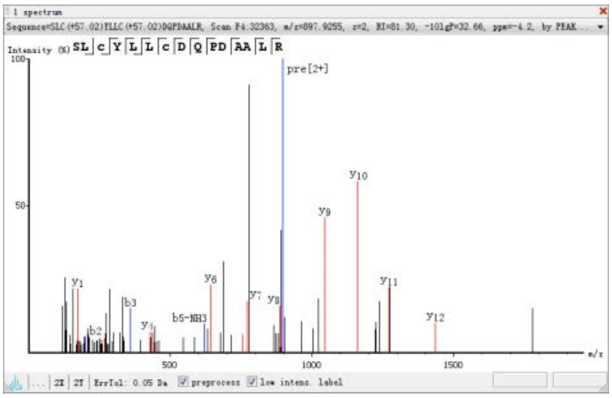

gi|731384926

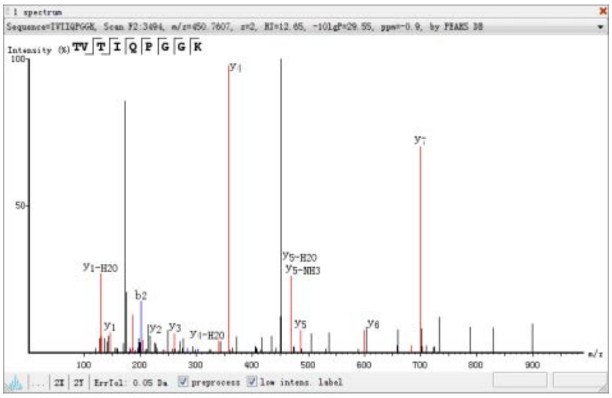

gi|731386596

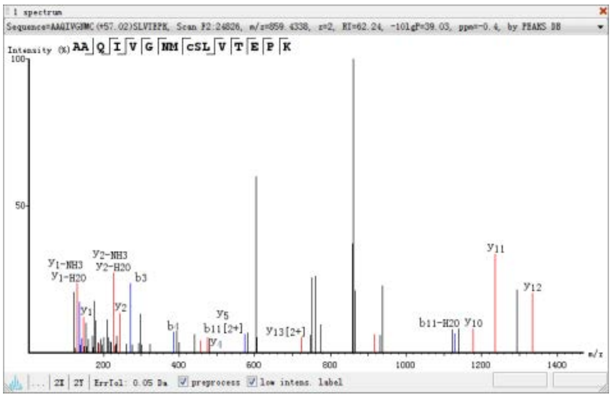

gi|731387184

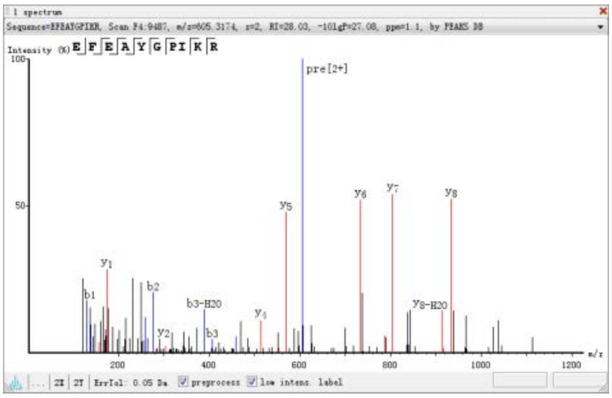

gi|731387738

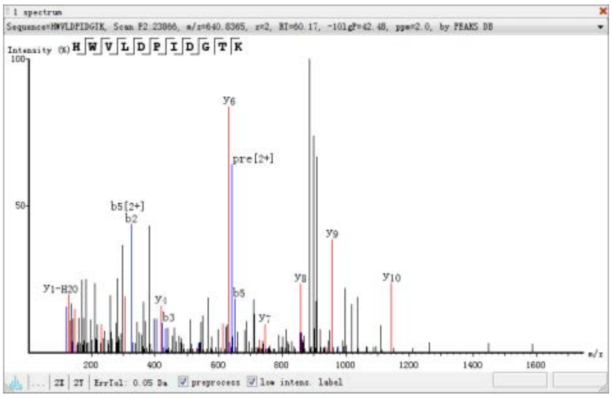

gi|731393208

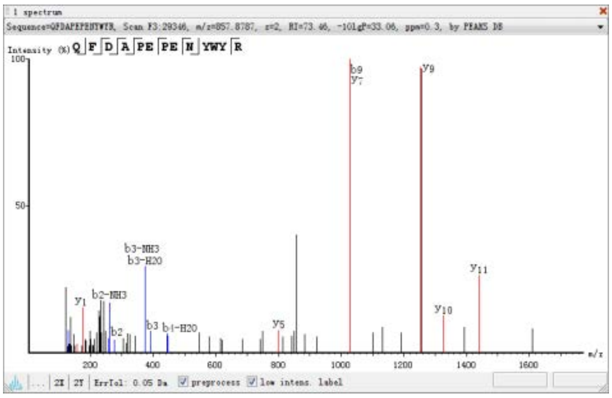

gi|731393274

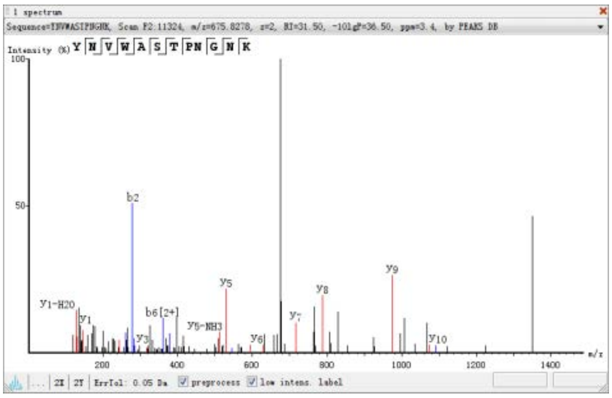

gi|731393417

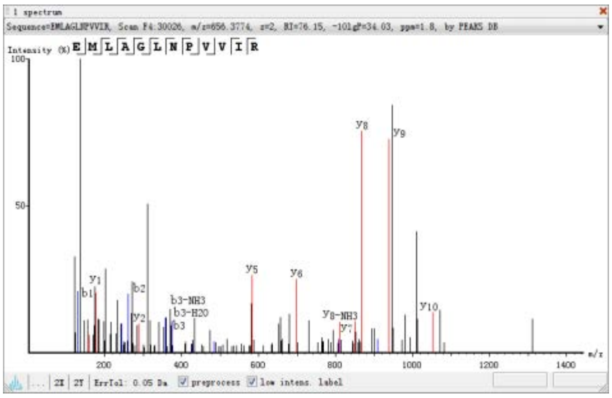

gi|731393636

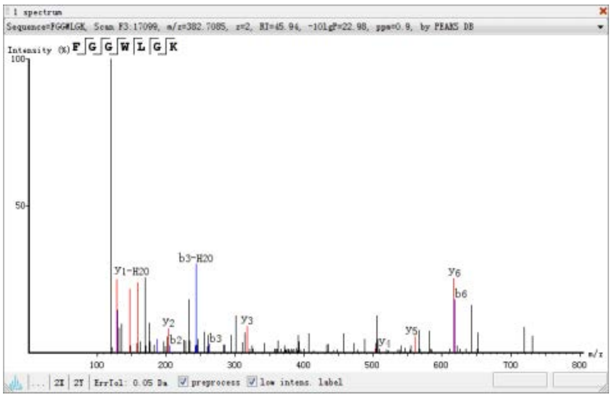

gi|731394805

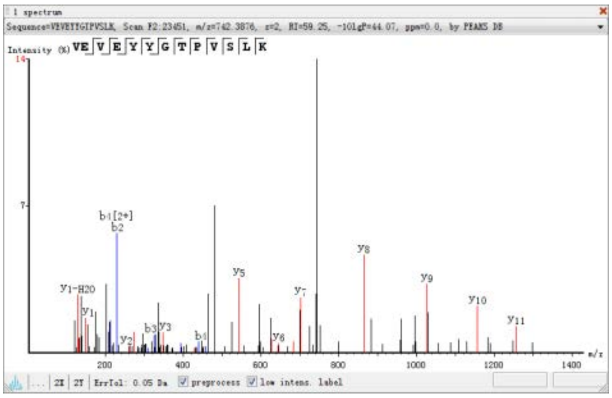

gi|731395998

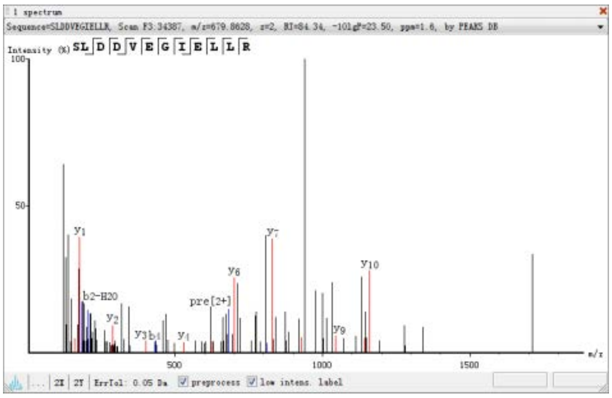

gi|731396358

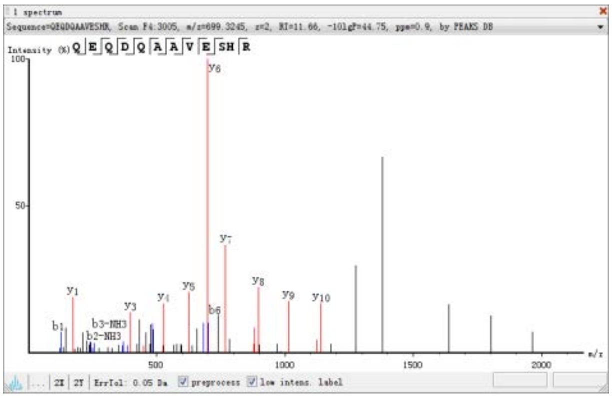

gi|731398376

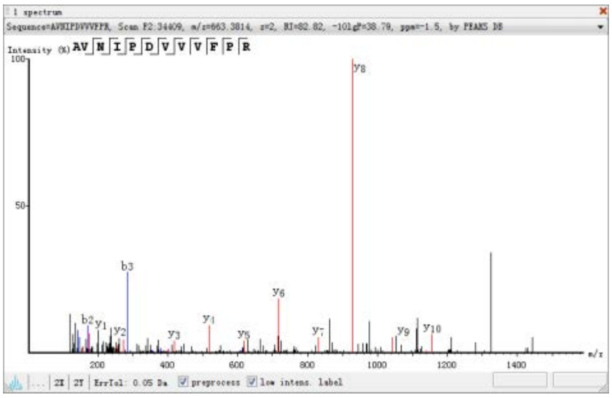

gi|731399255

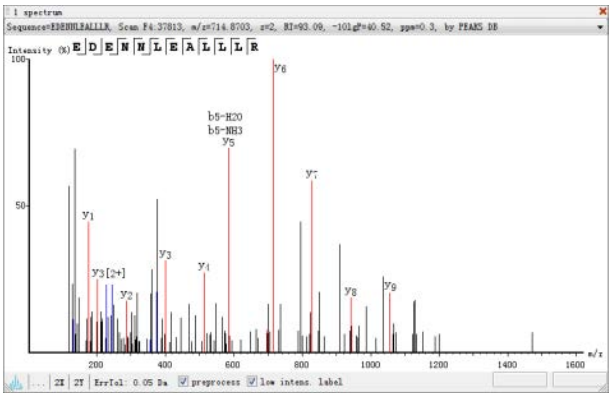

gi|731400181

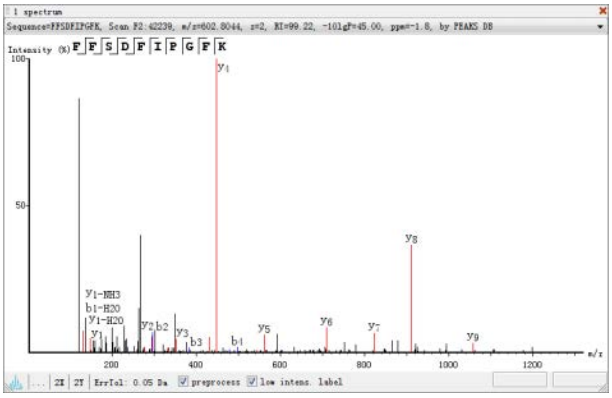

gi|731400729

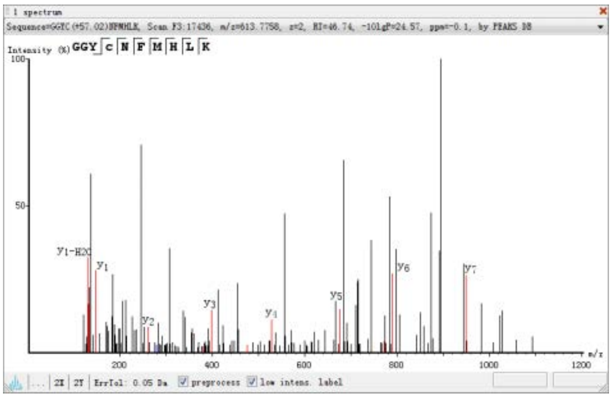

gi|731402564

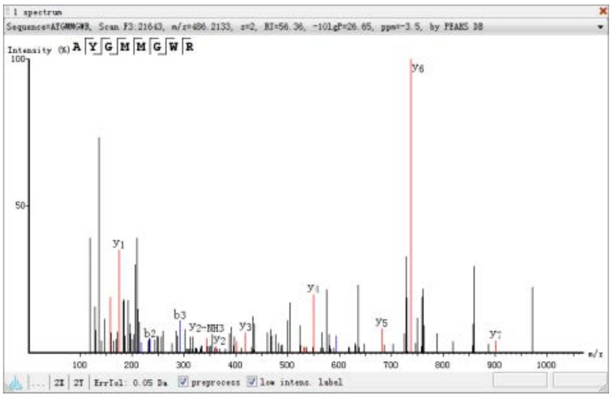

gi|731403923

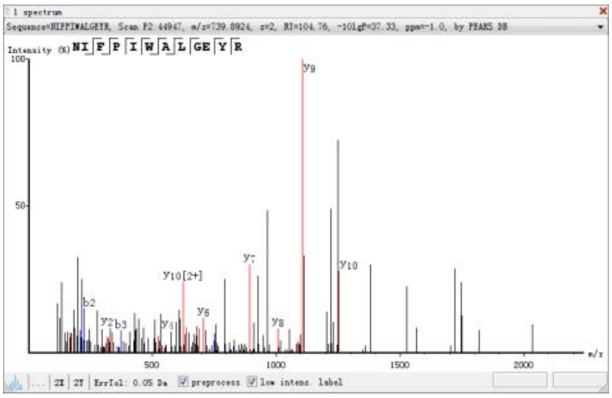

gi|731405196

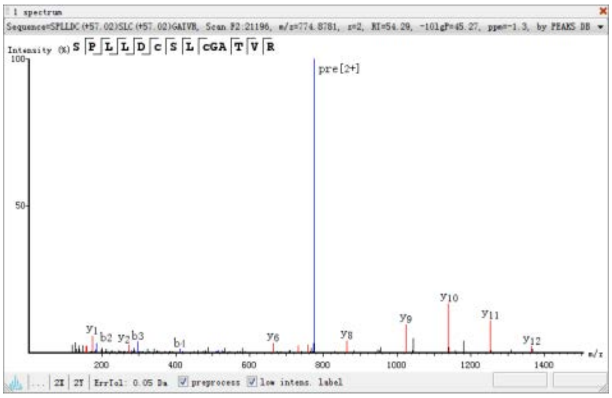

gi|731405420

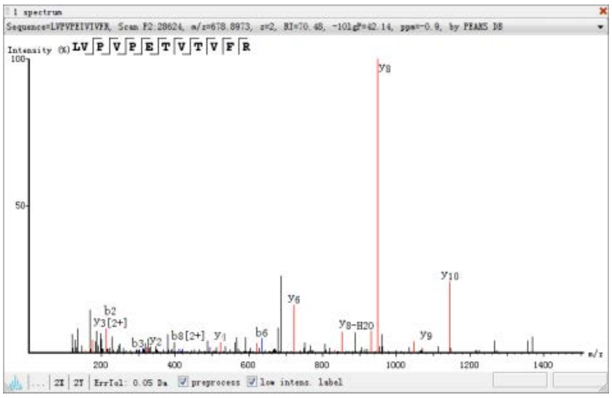

gi|731408196

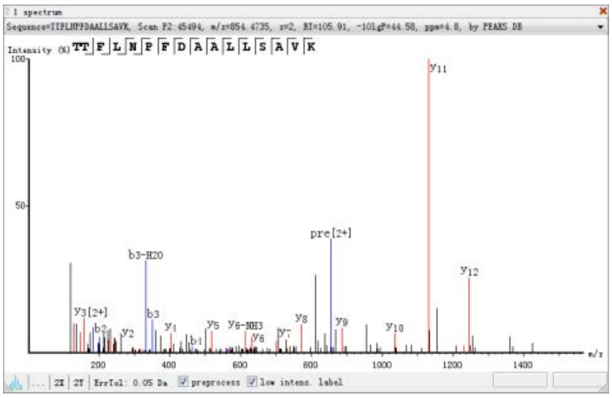

gi|731410731

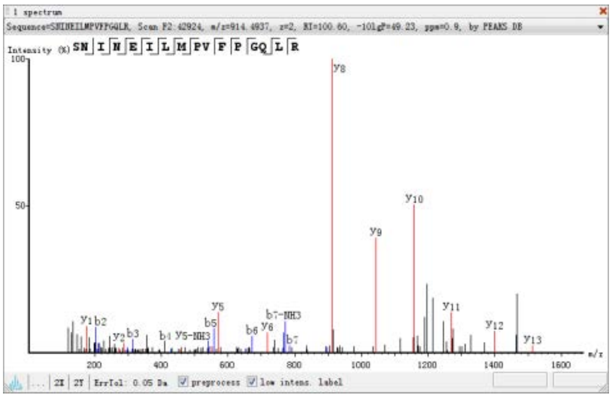

gi|731412938

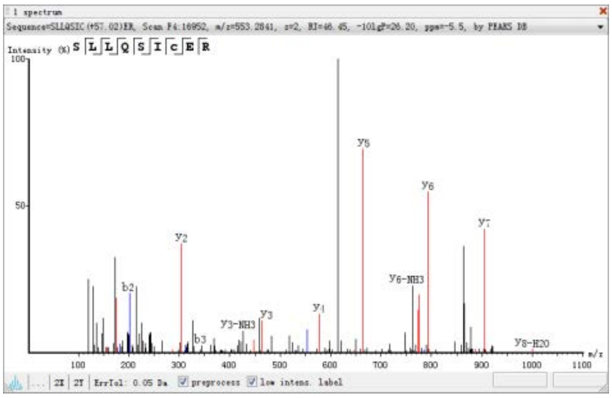

gi|731420830

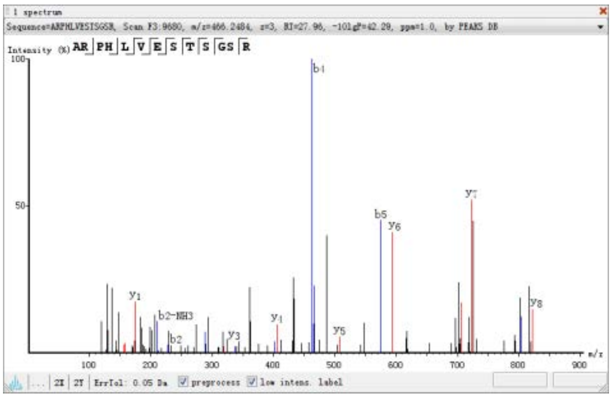

gi|731422386

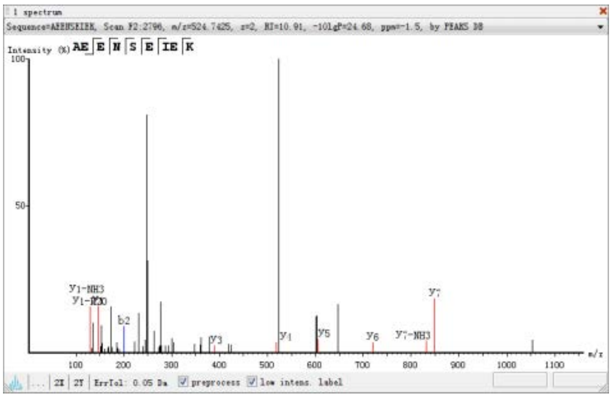

gi|731422566

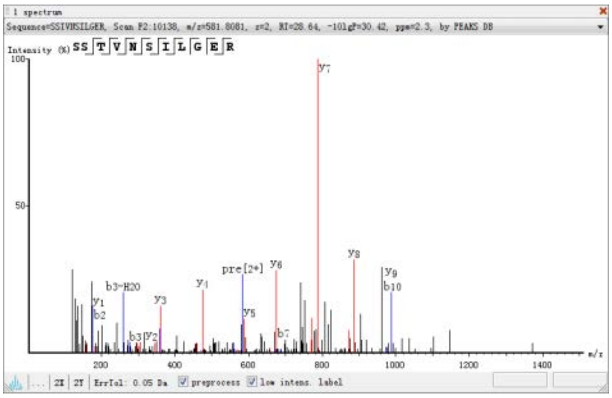

gi|731424103

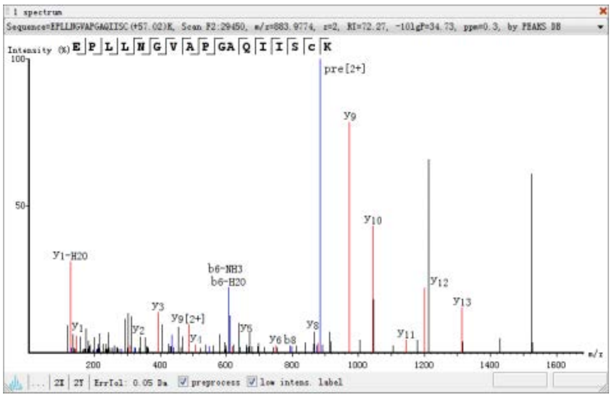

gi|731425464

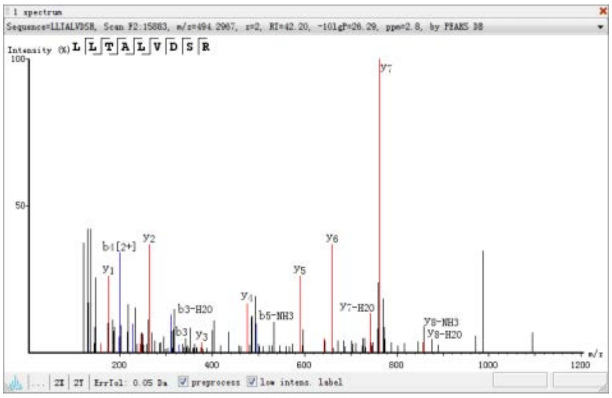

gi|731427546

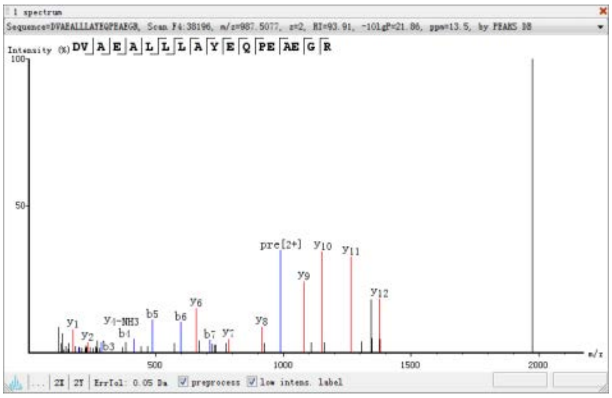

gi|731428733

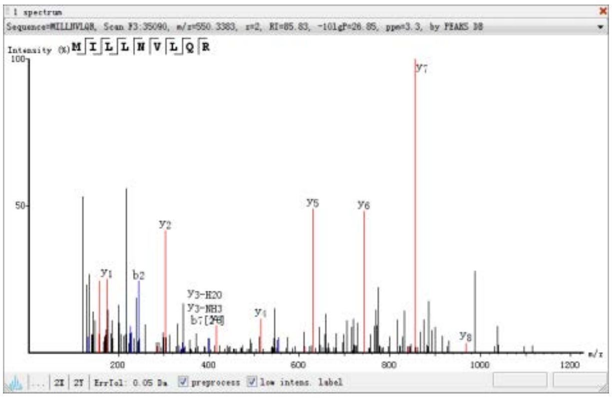

gi|731433292

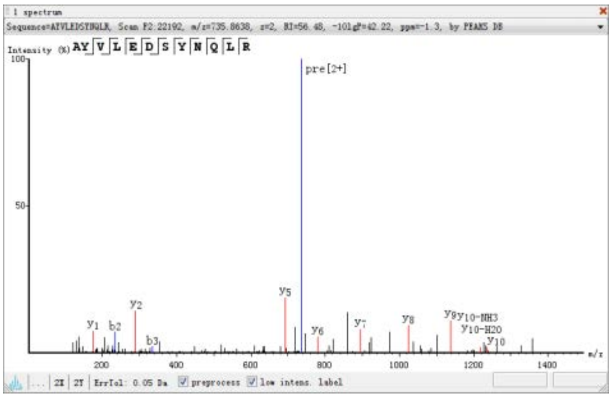

gi|731435290

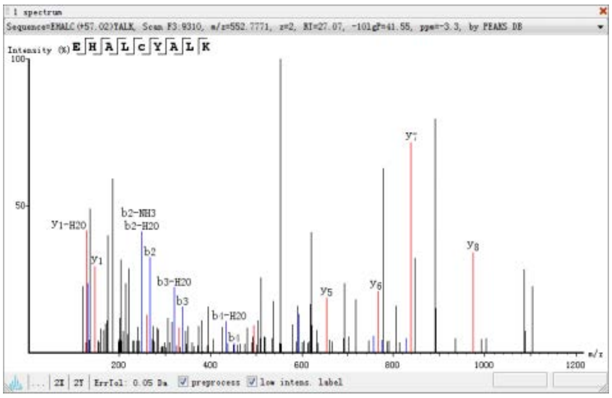

gi|731436460

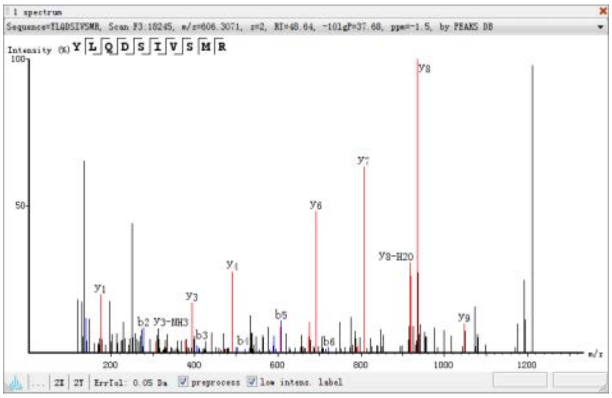

gi|731436828

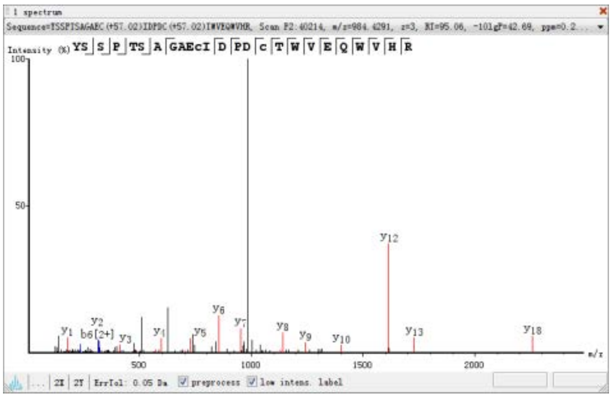

gi|731437632

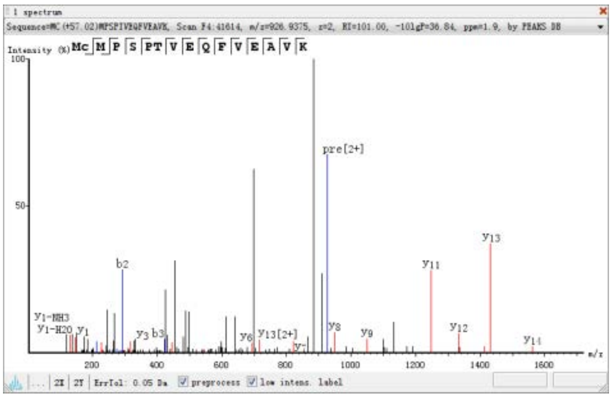

gi|731438600

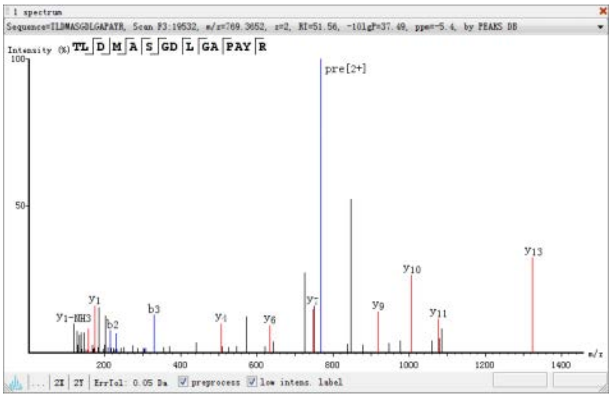

gi|731439030

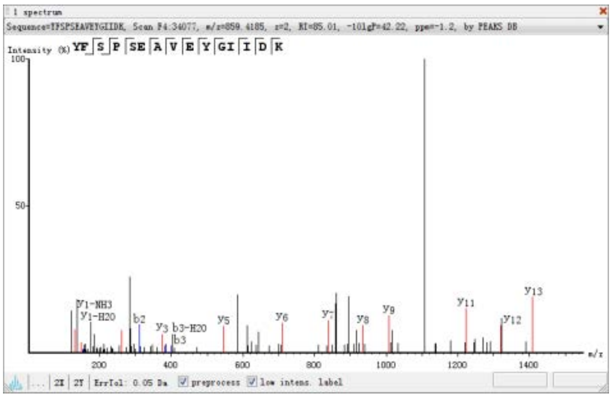

gi|731439963

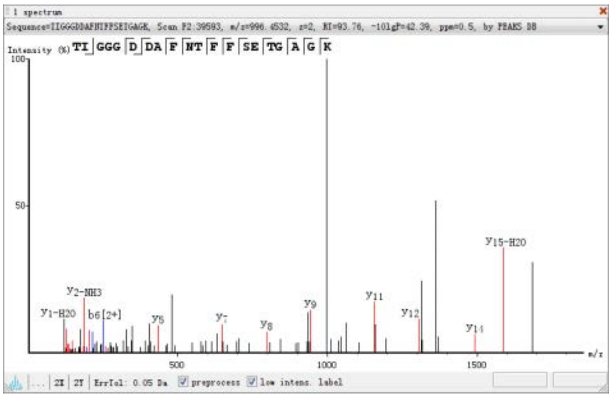

gi|731440022

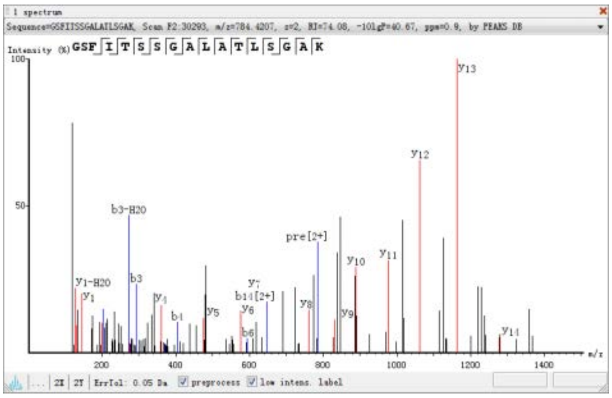

Fig.S3 The spectra of the proteins identified by one unique peptide with only one spectrum in CK2. Upper is the accession of protein, and under is its corresponding spectrum.

gi|225464589

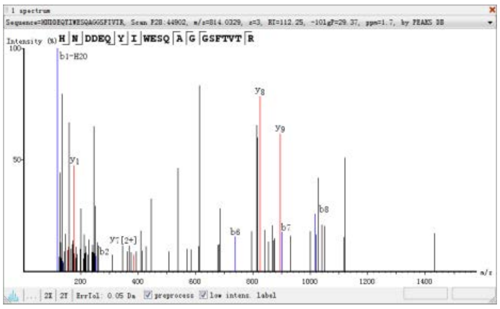

gi|225434984

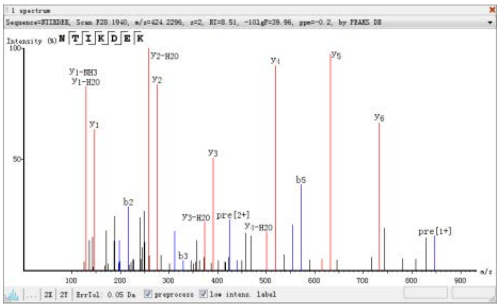

gi|225456143

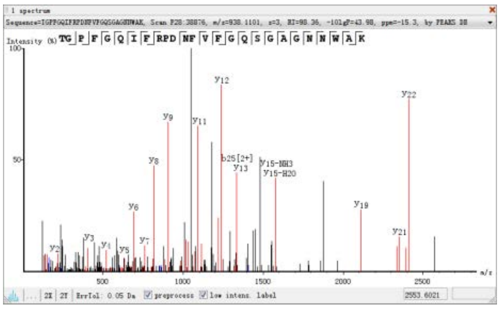

gi|225426414

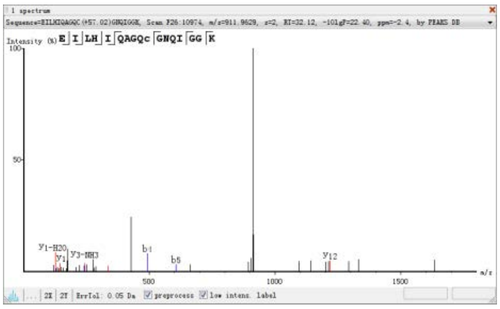

gi|224144808

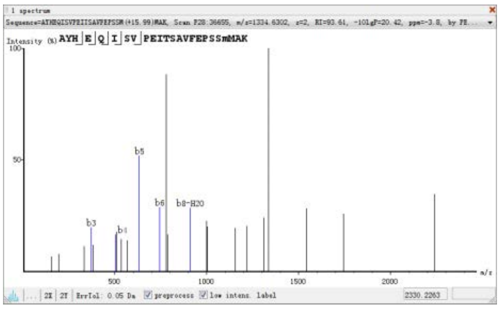

gi|731416007

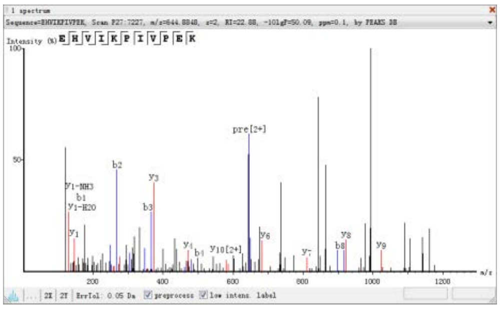

gi|731407424

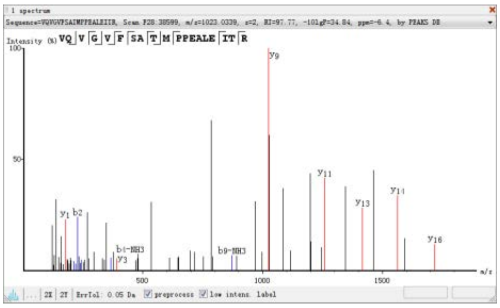

gi|566187902

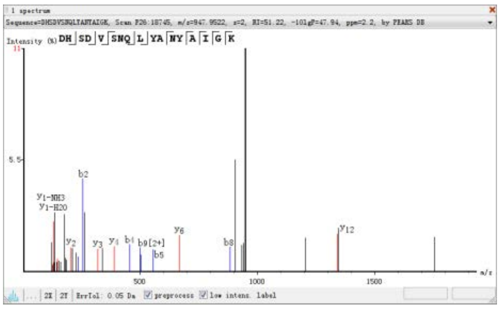

gi|566162272

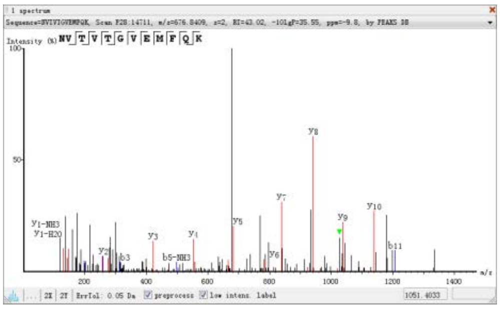

gi|566198898

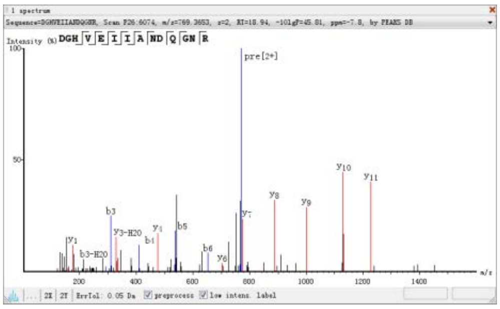

gi|731402352

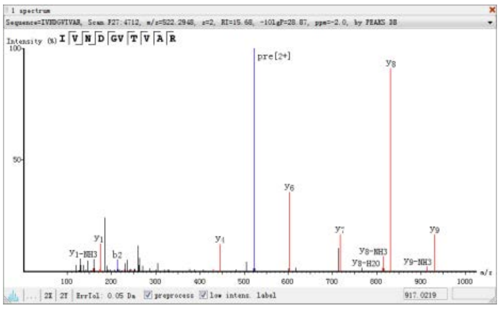

gi|225440011

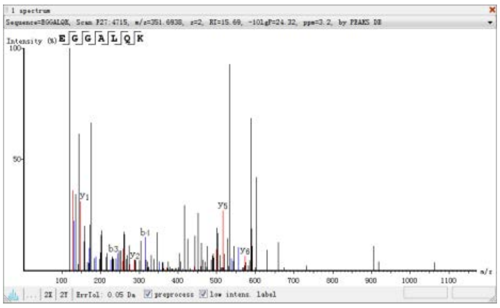

gi|566173508

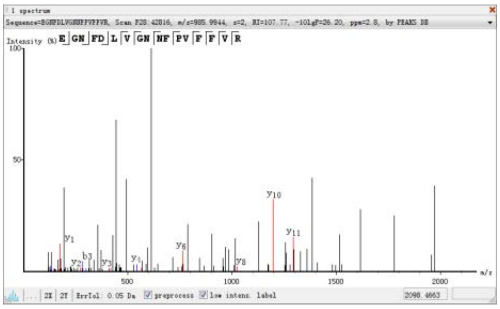

gi|566190996

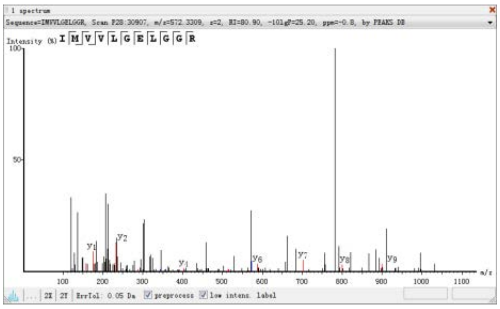

gi|526117711

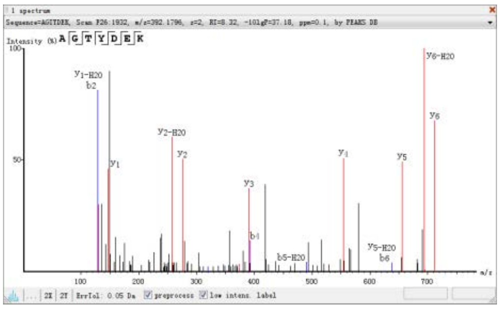

gi|566170698

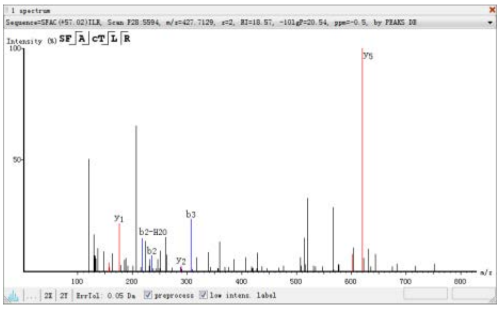

gi|224365636

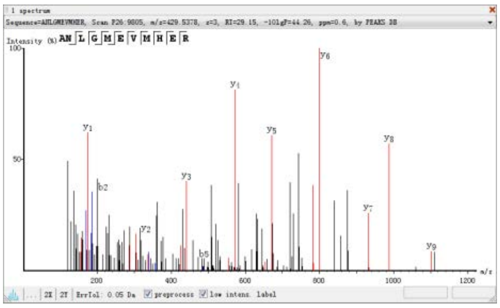

gi|566214954

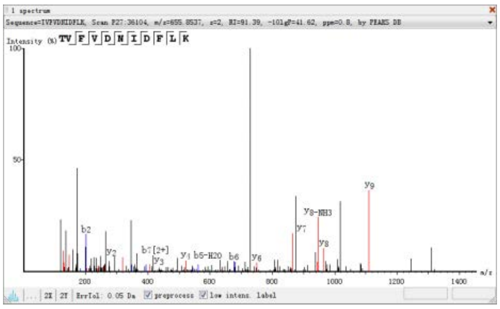

gi|225426063

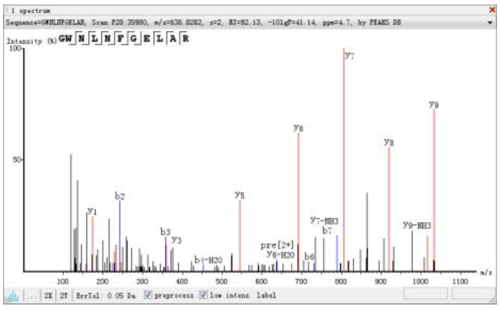

gi|566160536

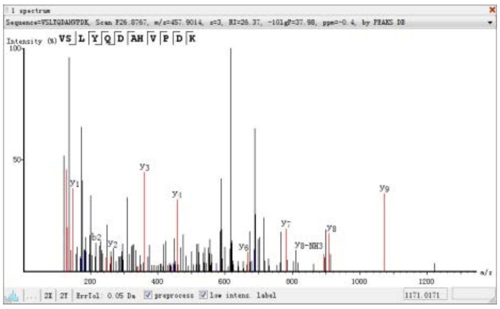

gi|225430650

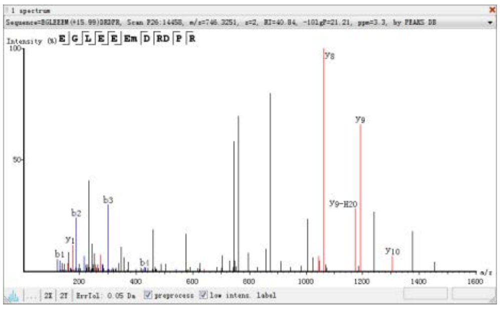

gi|224054542

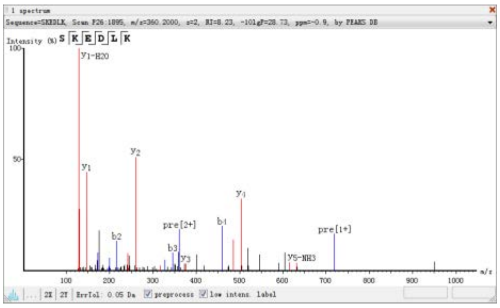

gi|225425166

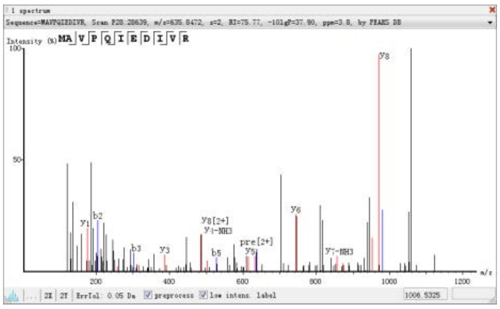

gi|224063273

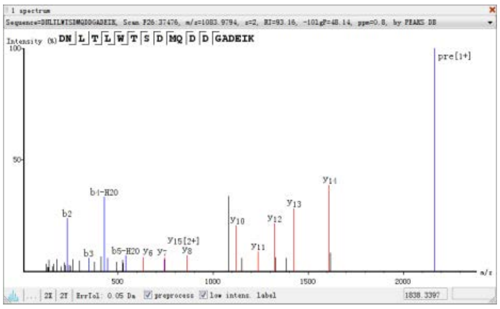

gi|225428247

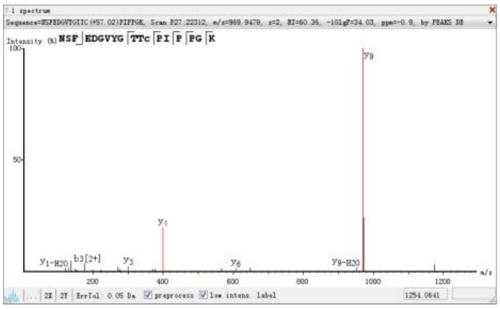

gi|224108774

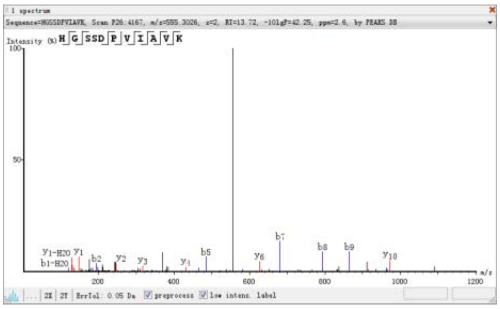

gi|731392629

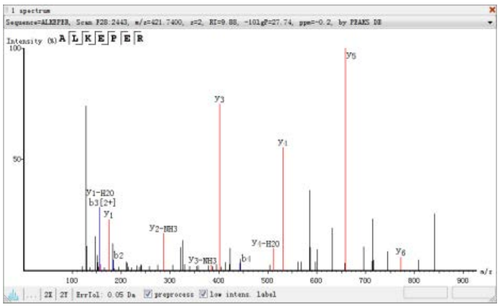

gi|225454009

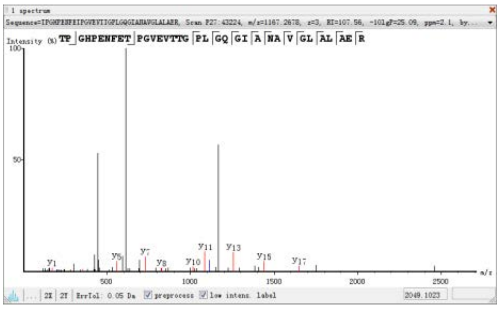

gi|225441044

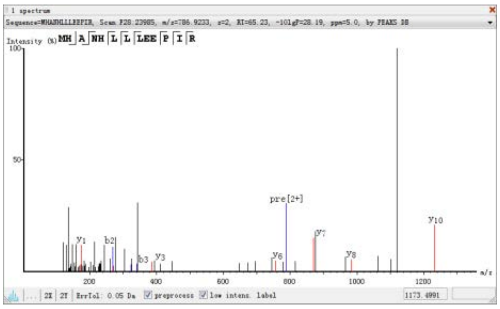

gi|225436900

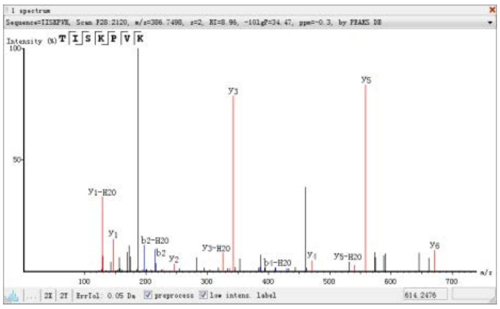

gi|731412938

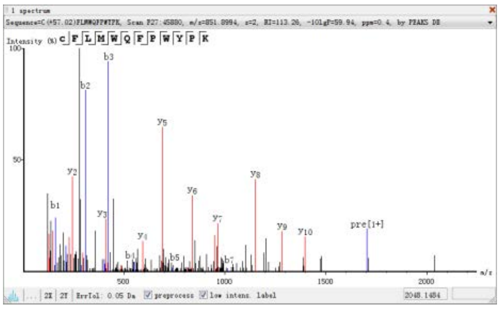

gi|225430366

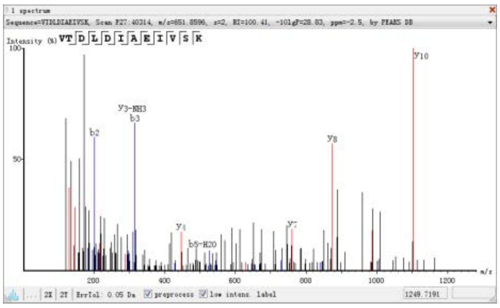

gi|224127346

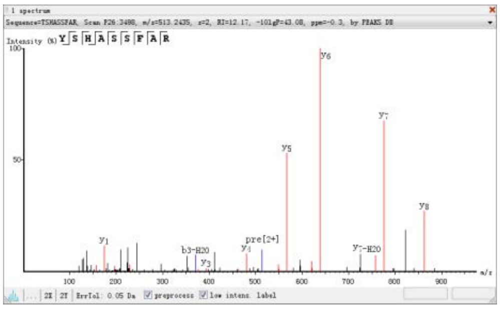

gi|731399856

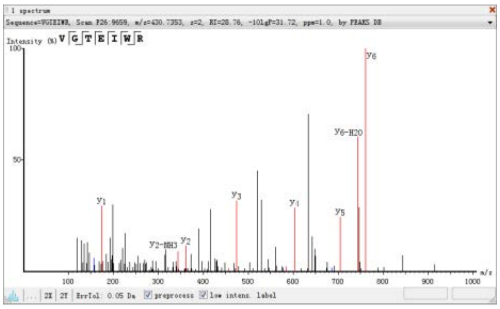

gi|225432758

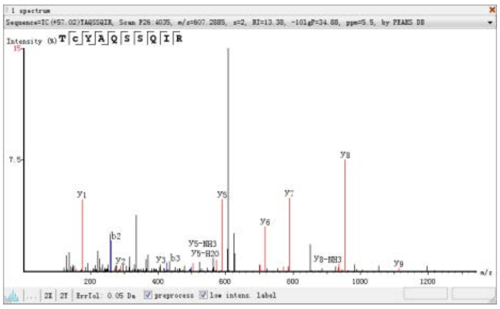

gi|224091827

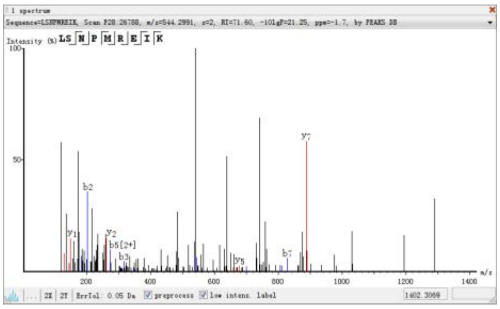

gi|566191895

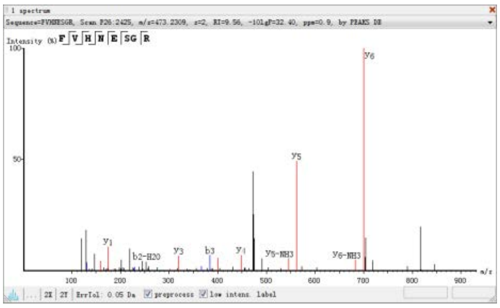

gi|566167161

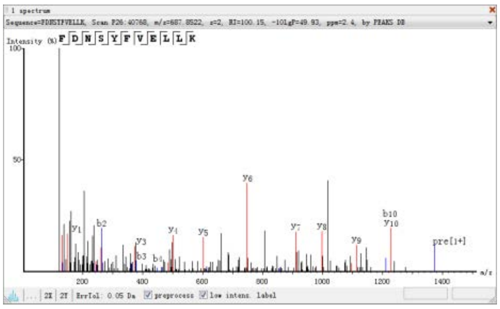

gi|224066545

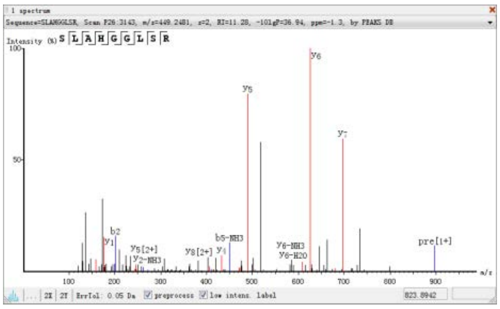

gi|225423722

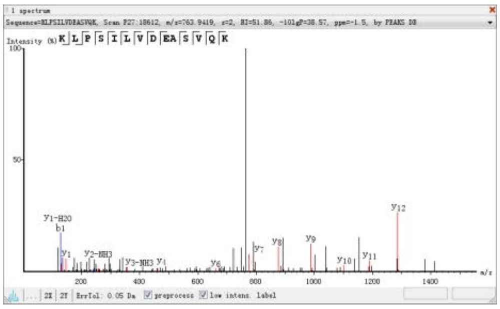

gi|225445206

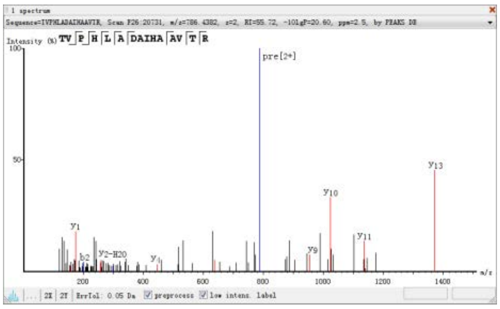

gi|225450549

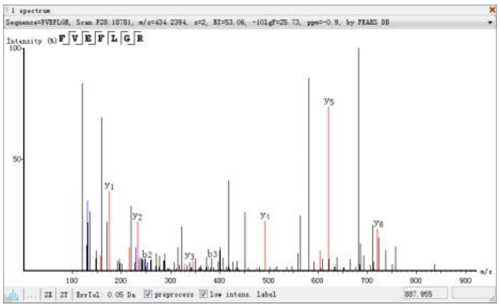

gi|224119200

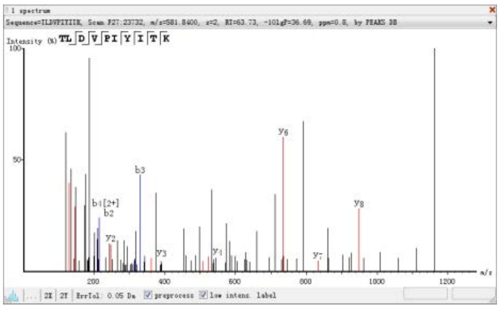

gi|731401620

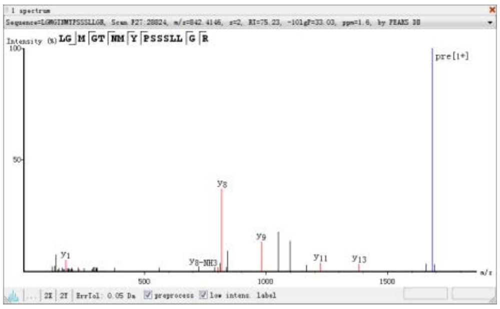

gi|224075445

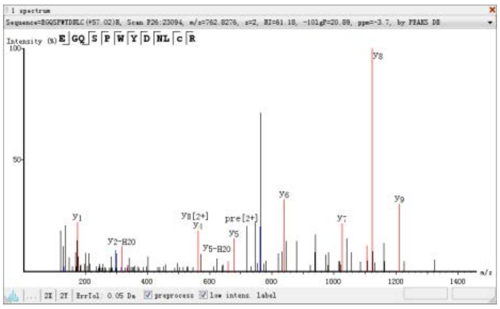

gi|566168414

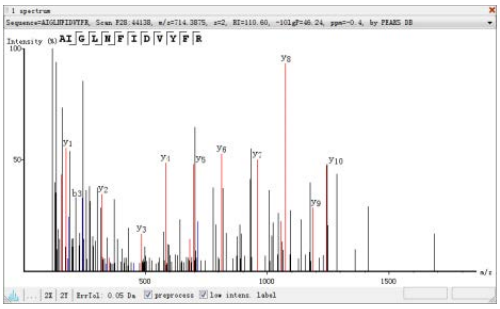

gi|225443272

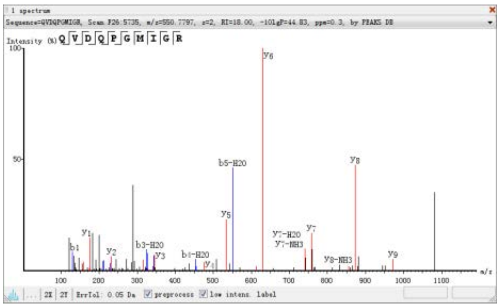

gi|225447723

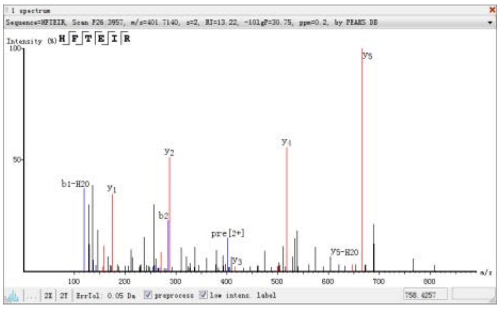

gi|566200975

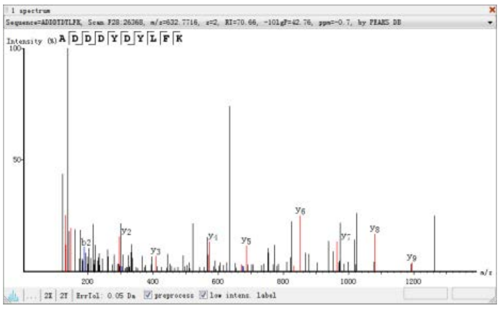

gi|566259512

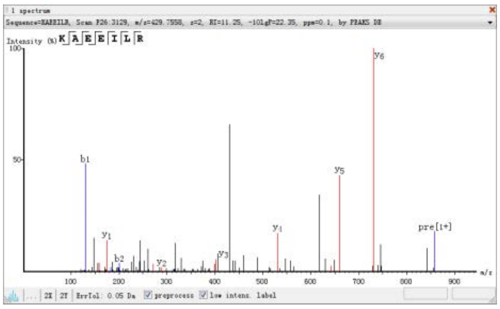

gi|224078584

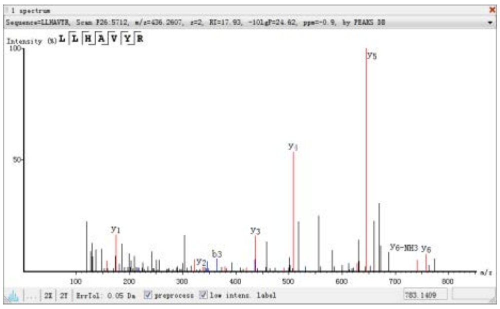

gi|224100535

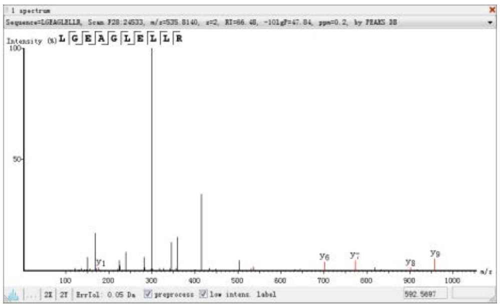

gi|731392621

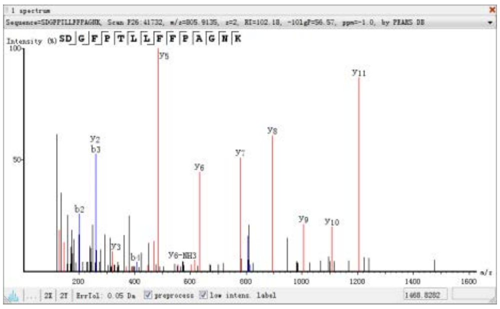

gi|566213642

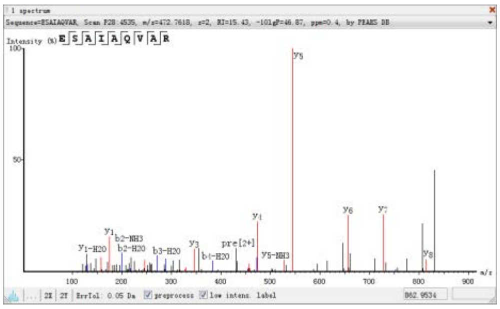

gi|566166593

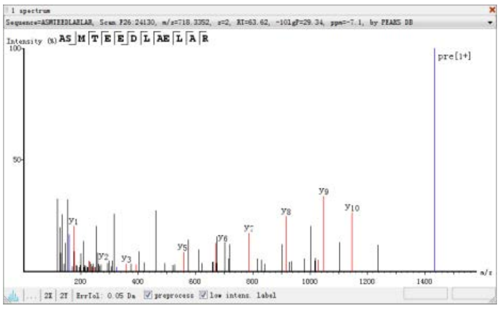

gi|224104697

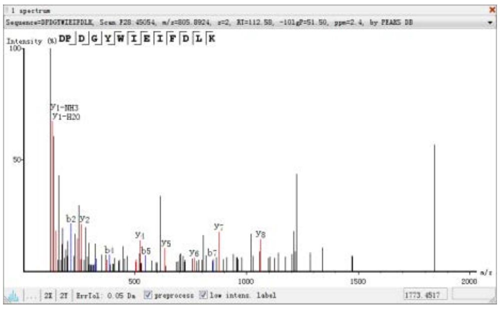

gi|224072208

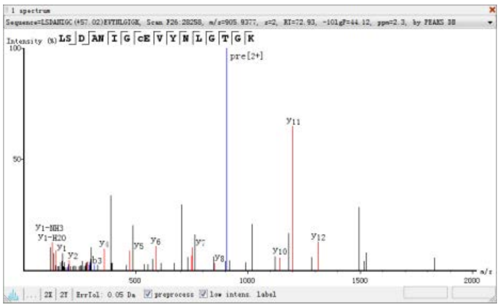

gi|566162135

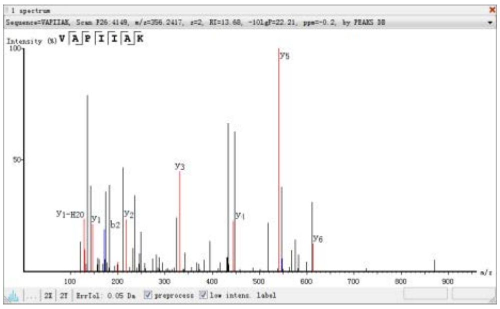

gi|566214265

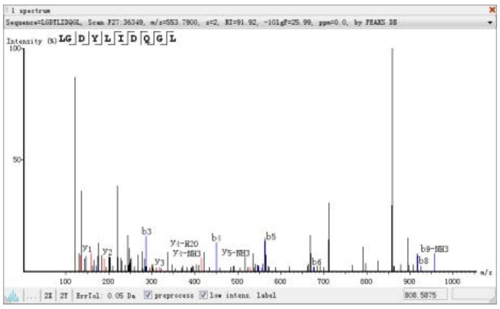

gi|225442434

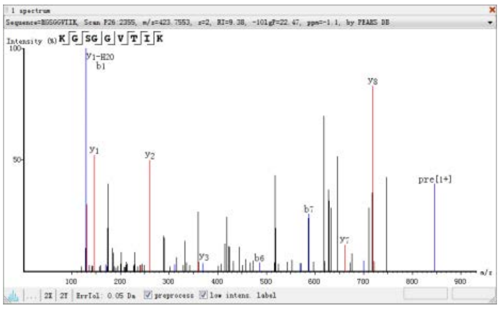

gi|566156965

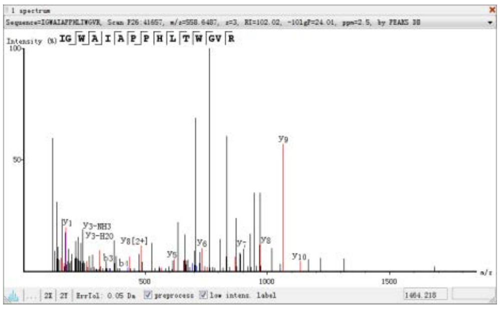

gi|731423236

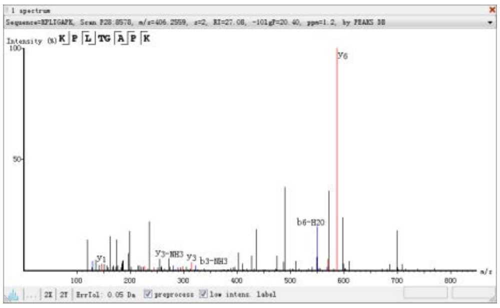

gi|731408416

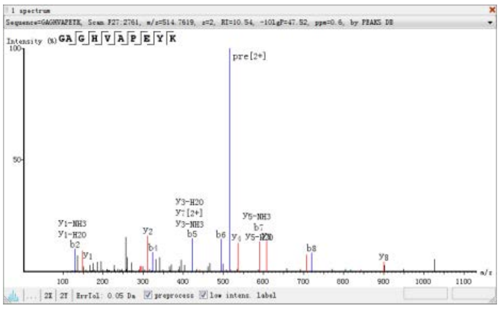

gi|224086367

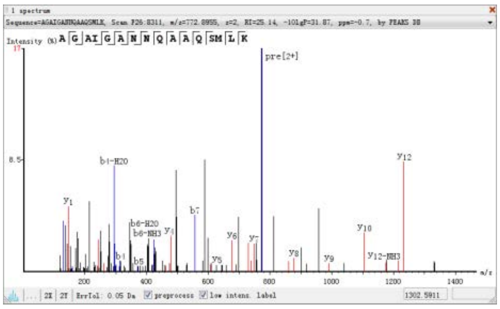

gi|566146801

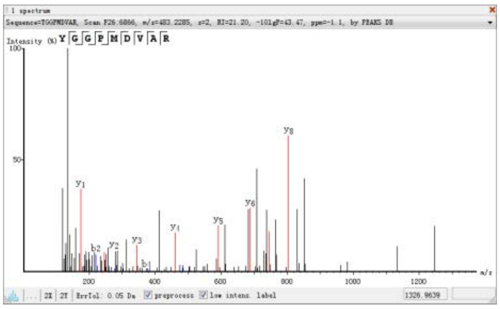

gi|225440230

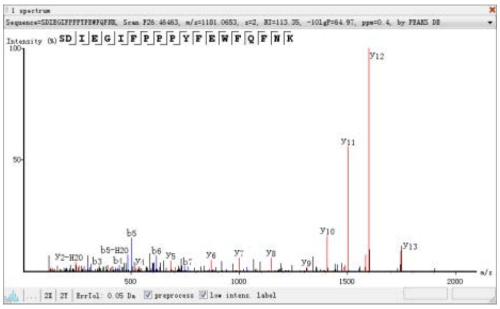

gi|566202791

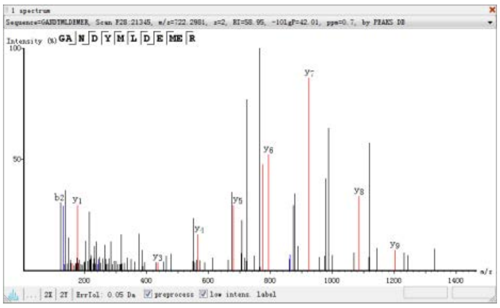

gi|225428043

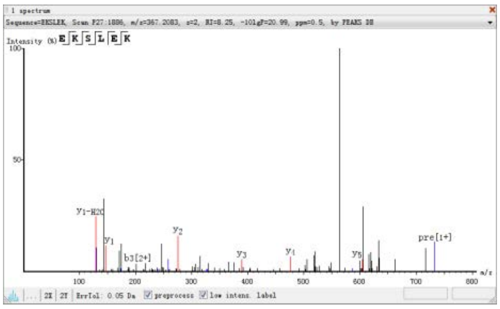

gi|224099811

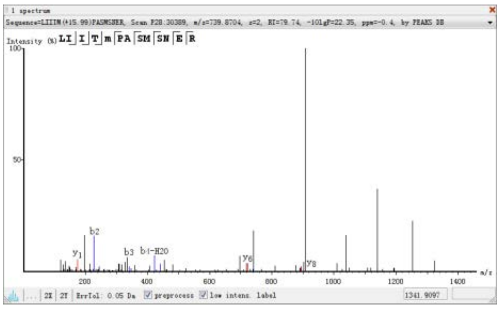

gi|566200149

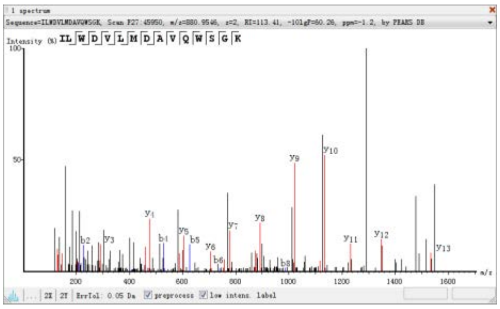

gi|566245088

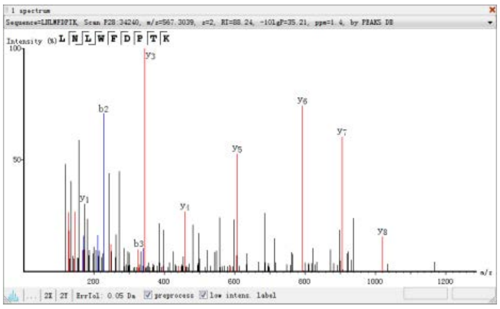

gi|566171321

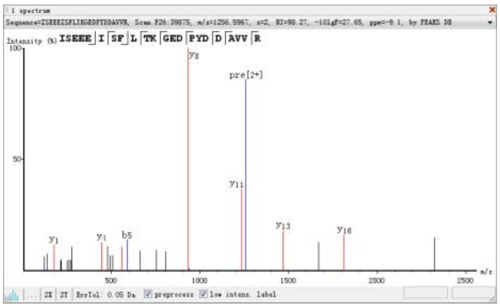

gi|225434195

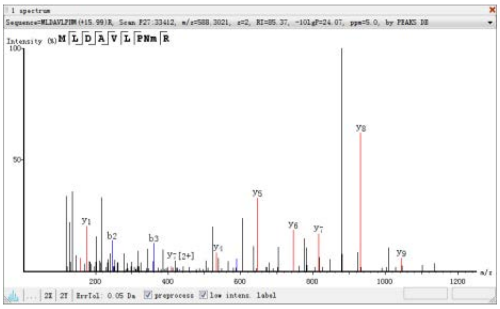

gi|359478417

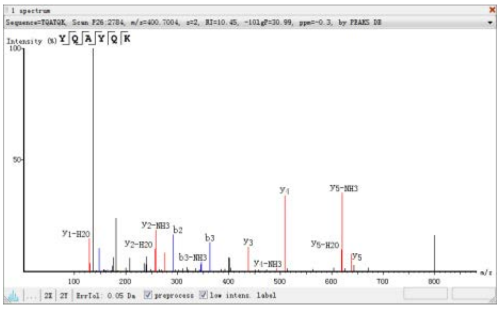

gi|731393292

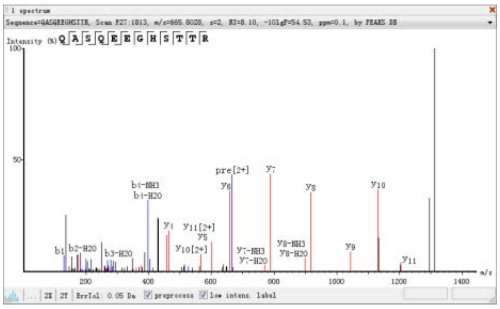

gi|225455386

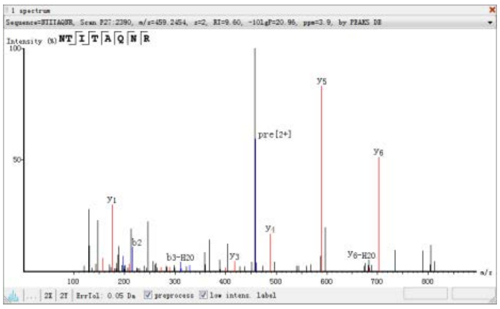

gi|359490940

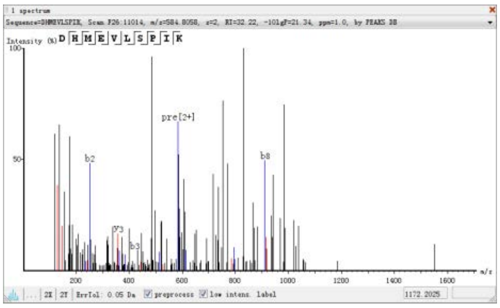

gi|224064850

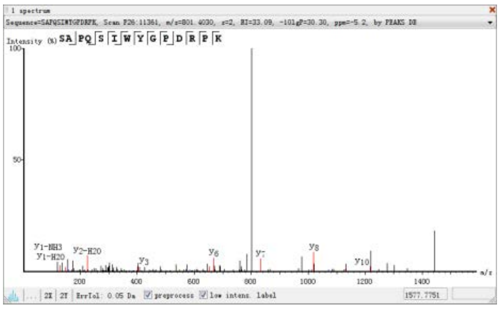

gi|566190820

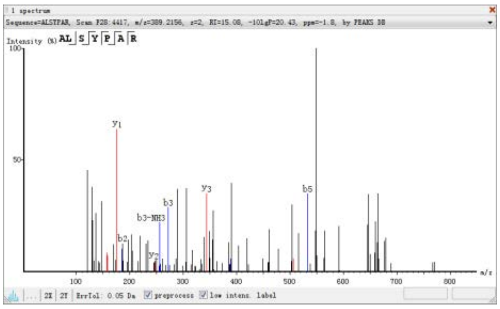

gi|731434735

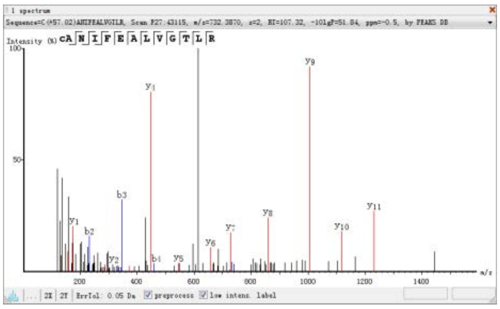

gi|566161702

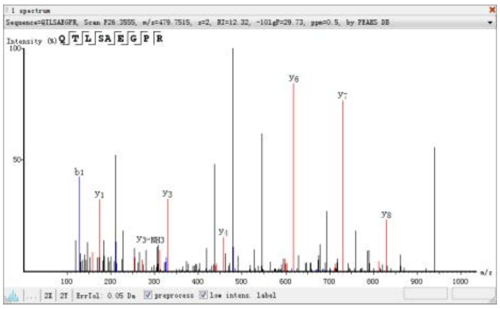

gi|731412260

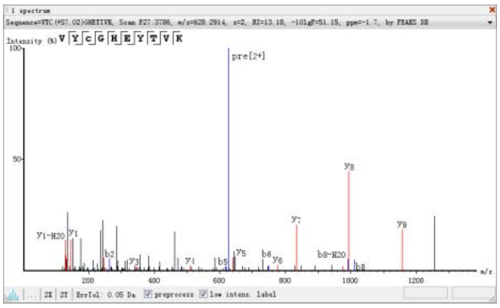

gi|566195800

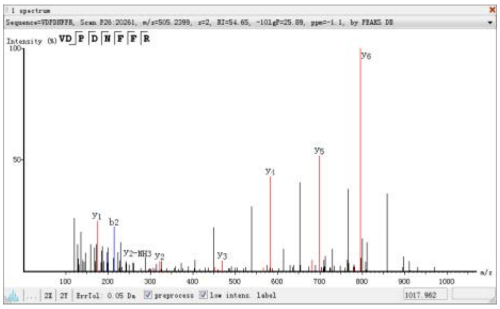

gi|731438661

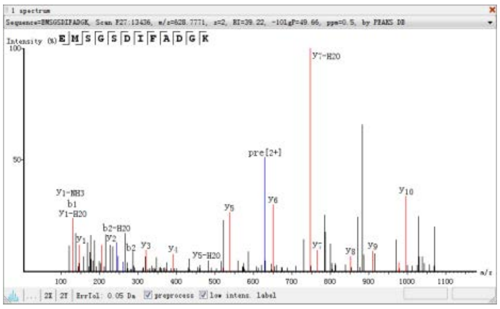

gi|566179312

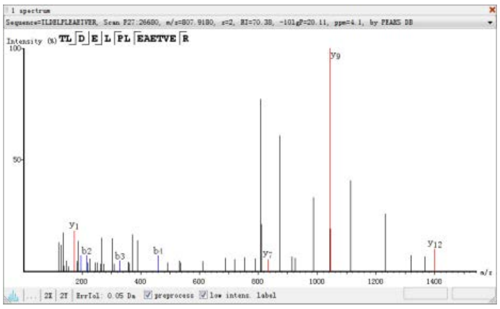

gi|225426330

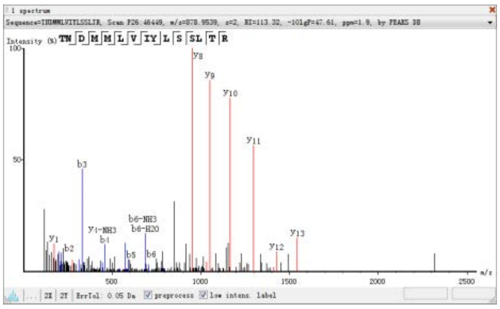

gi|225461673

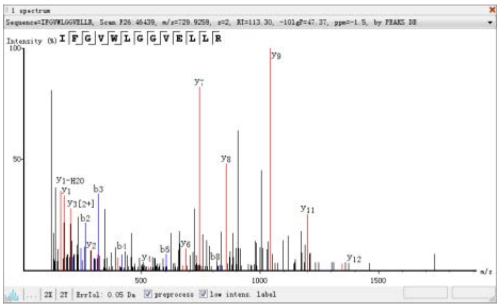

gi|224103201

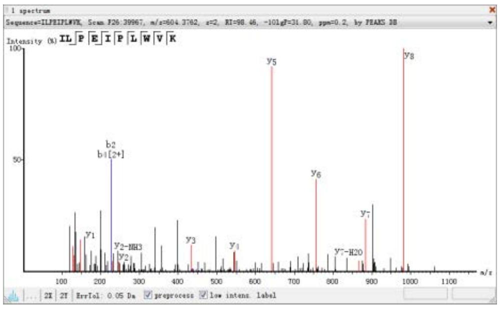

gi|225450155

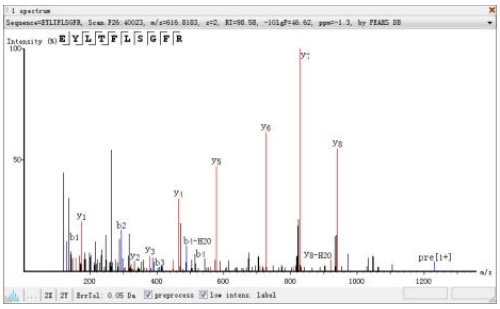

gi|225457231

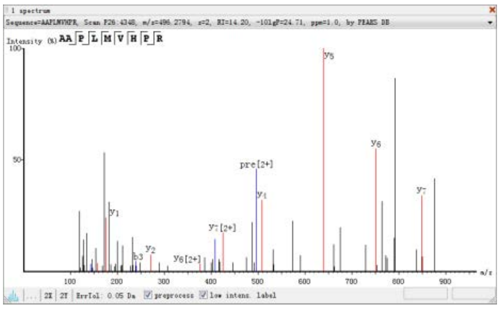

gi|224097915

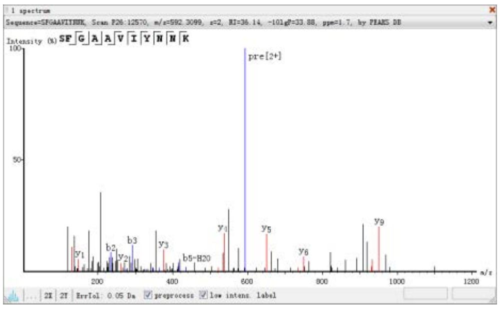

gi|731408402

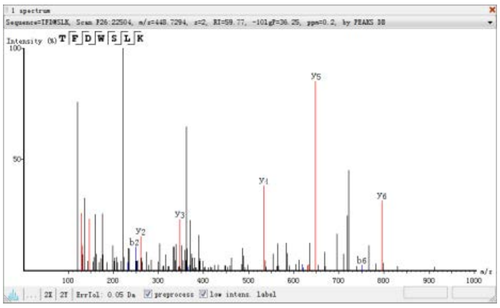

gi|731387671

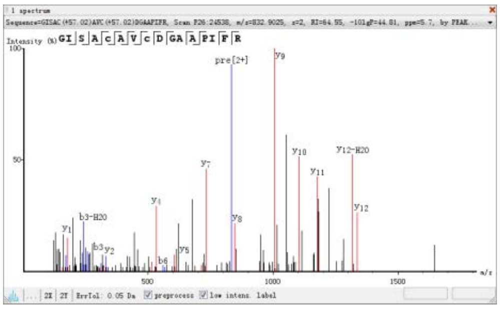

gi|566187811

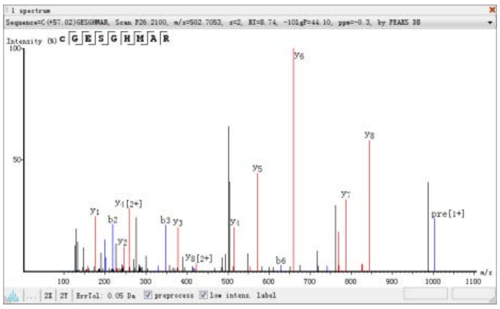

gi|224091819

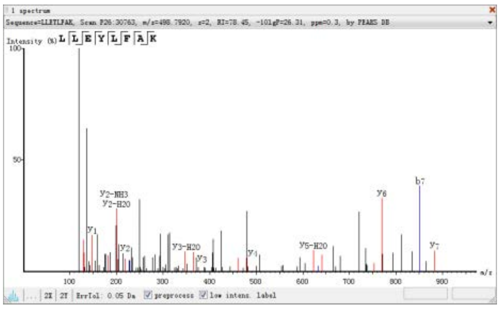

gi|566214901

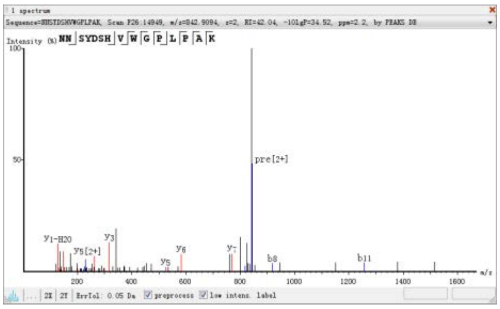

gi|224067088

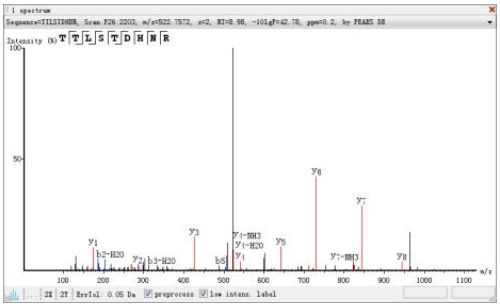

gi|224102065

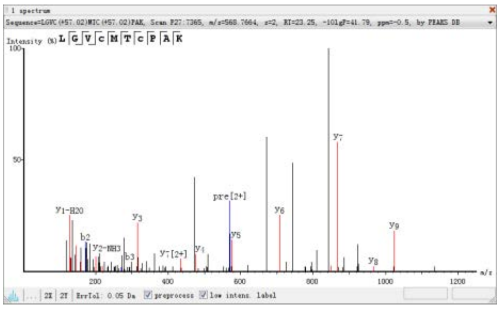

gi|224104913

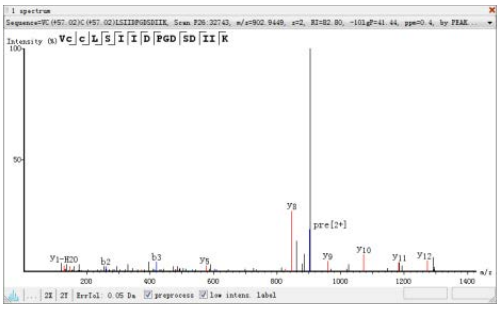

gi|359483831

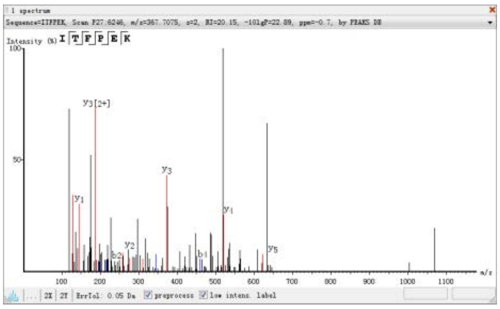

gi|225425356

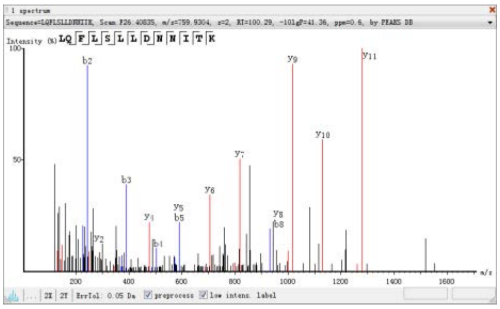

gi|224117530

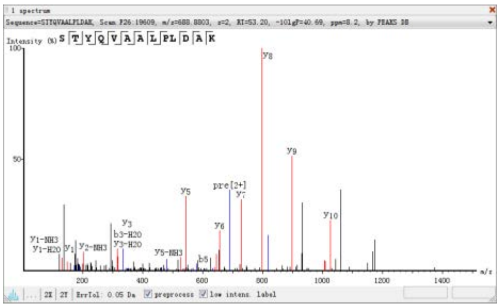

gi|225428388

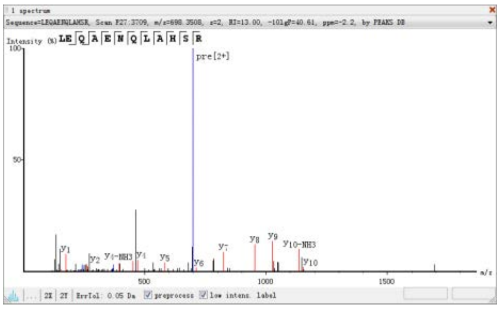

gi|225452877

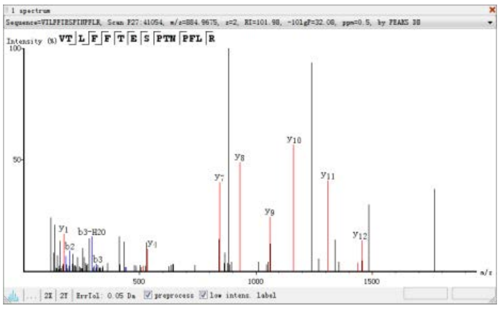

gi|225452053

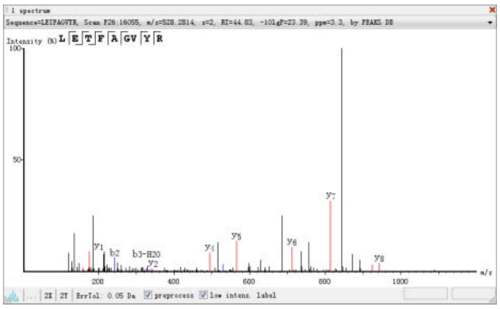

gi|225442595

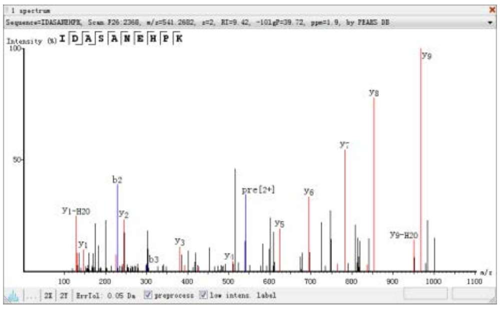

gi|566197448

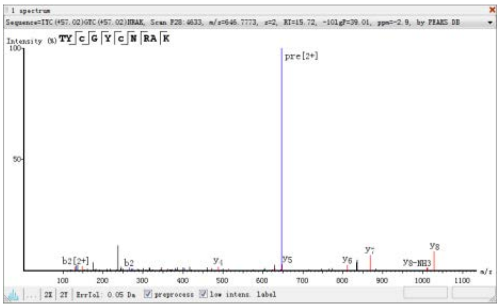

gi|566210973

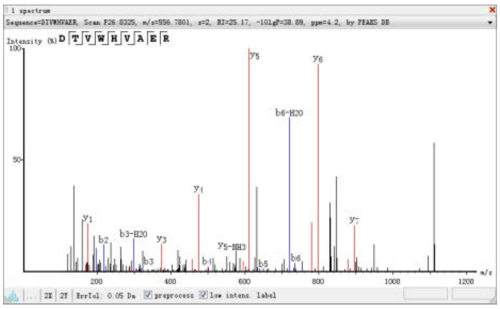

gi|225444159

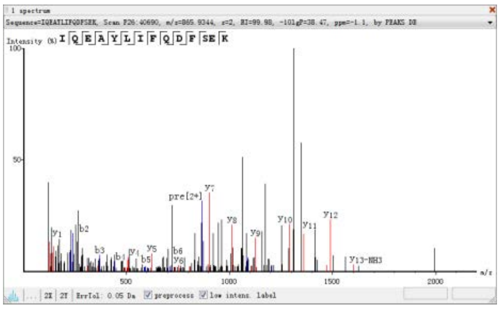

gi|731371107

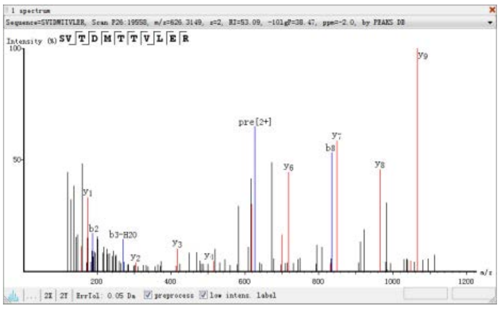

gi|566164286

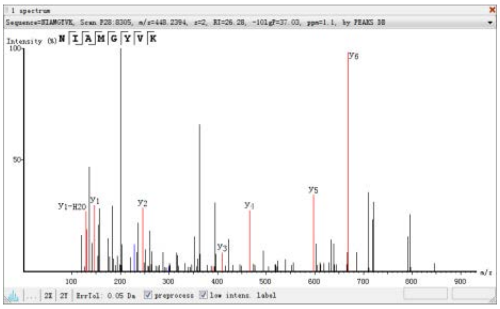

gi|225423779

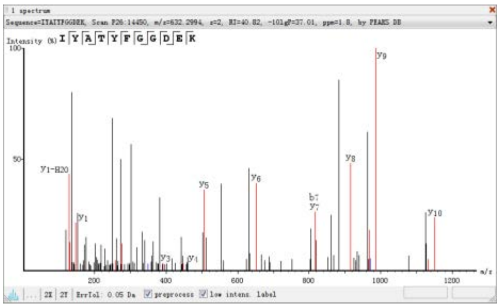

gi|731416275

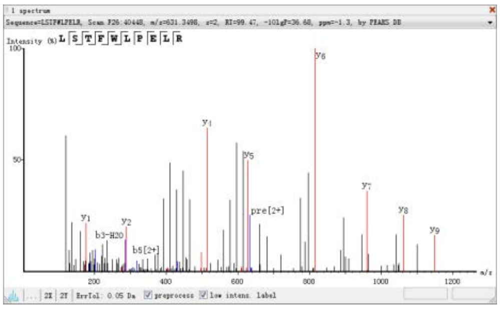

gi|566150318

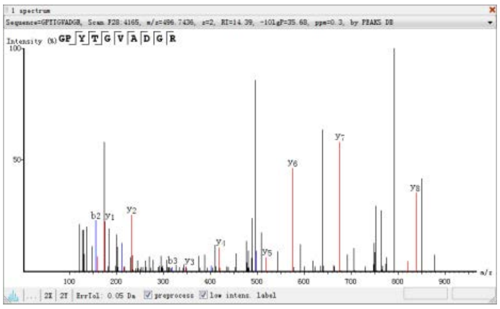

gi|731427528

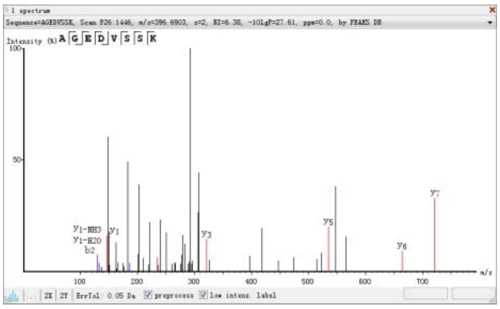

gi|225439378

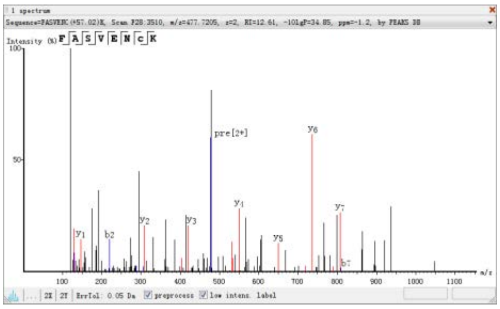

gi|566181287

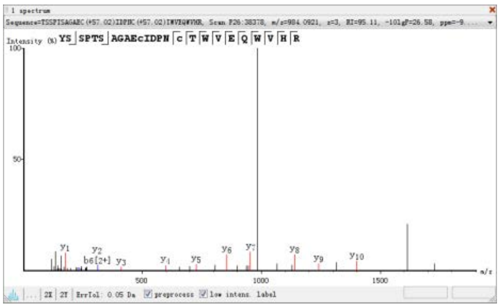

gi|225441521

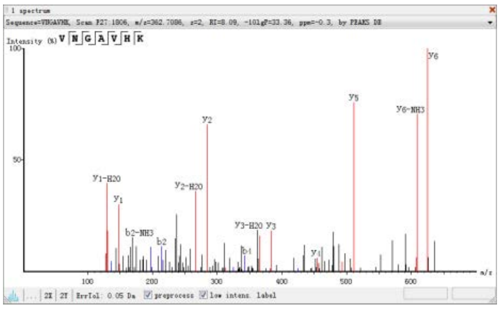

gi|225442452

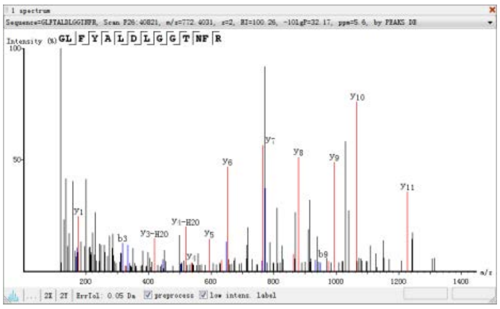

gi|225427364

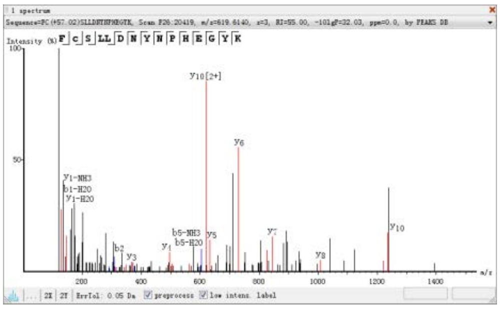

gi|566195150

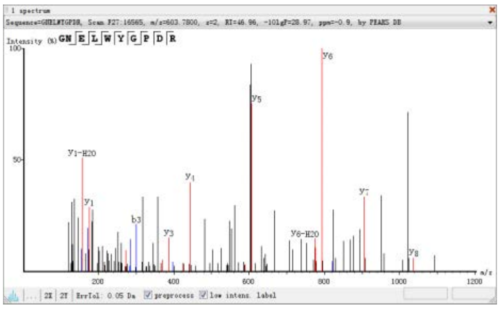

gi|566187112

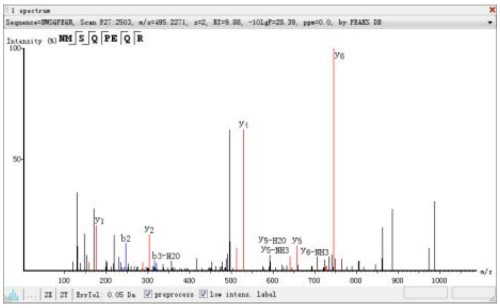

gi|731422864

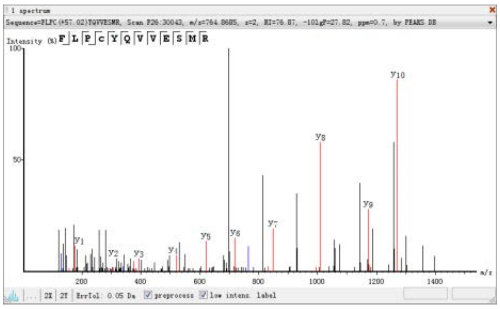

gi|731427572

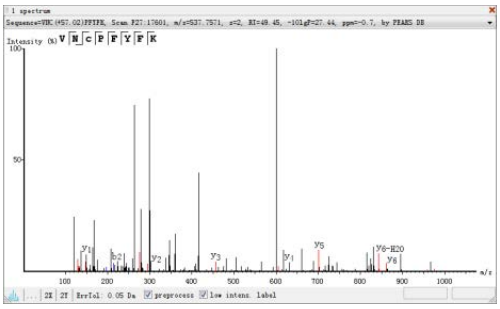

gi|225433215

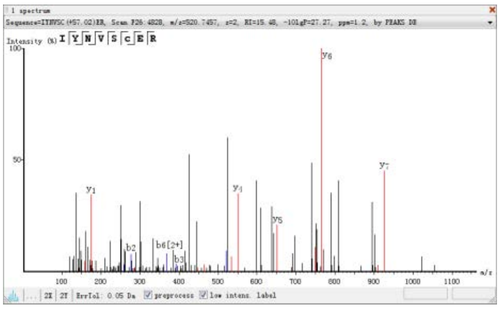

gi|566170004

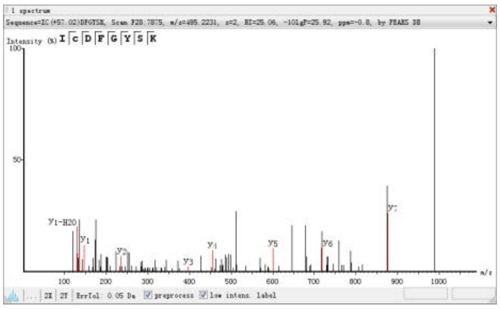

gi|731420144

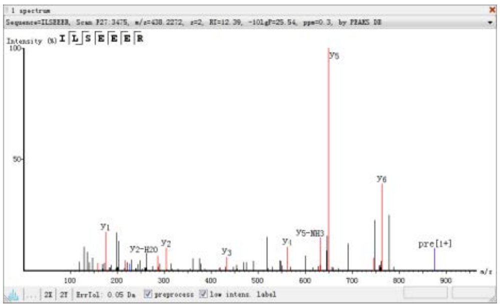

gi|731406154

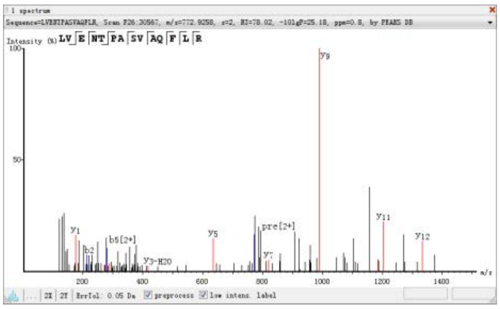

gi|224063997

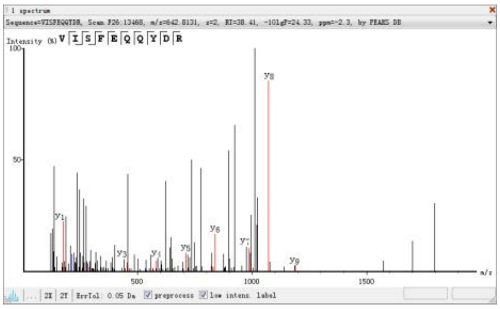

gi|359493124

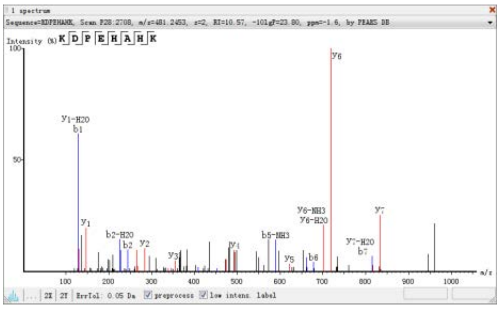

gi|731420480

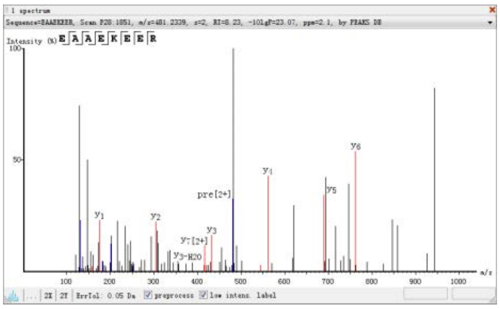

gi|731426219

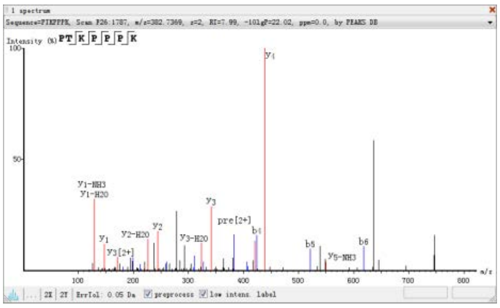

gi|566149485

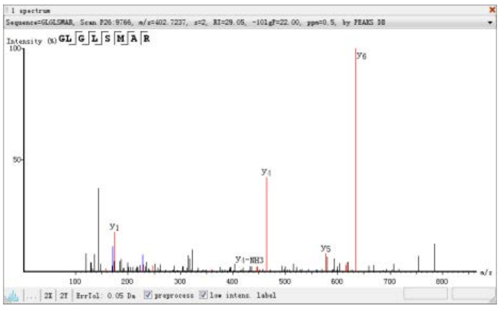

gi|225431169

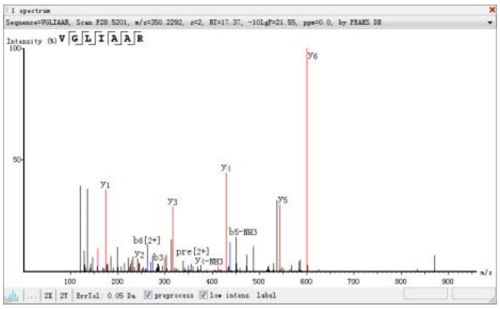

gi|566167089

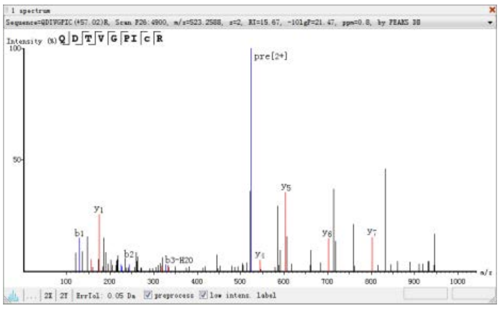

gi|566168468

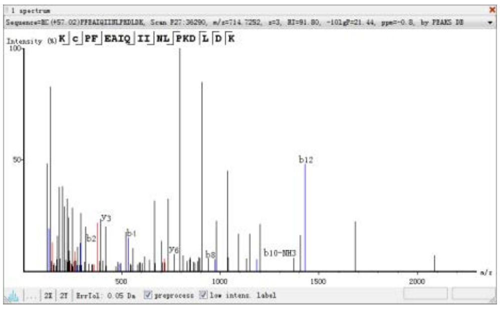

gi|566163422

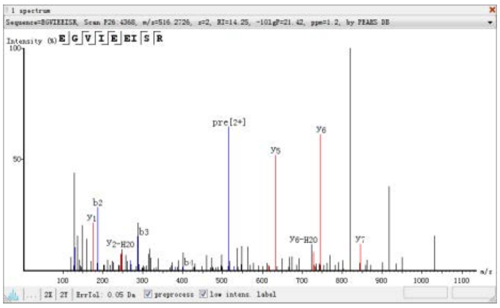

gi|566184883

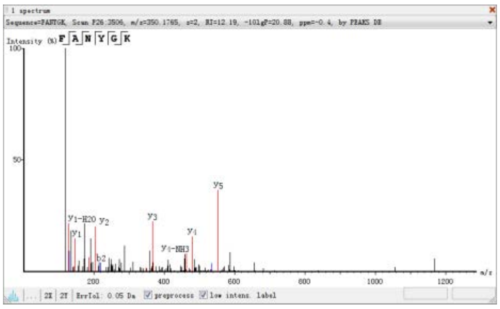

gi|731386964

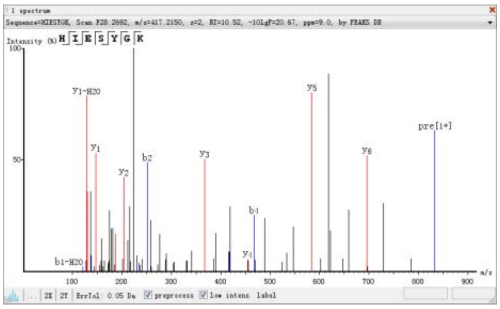

gi|225462297

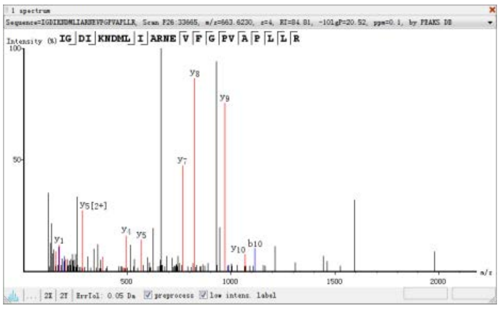

gi|359484223

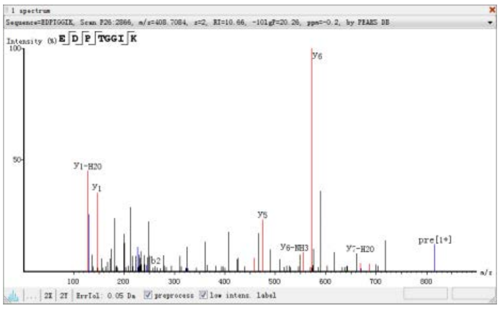

gi|566188564

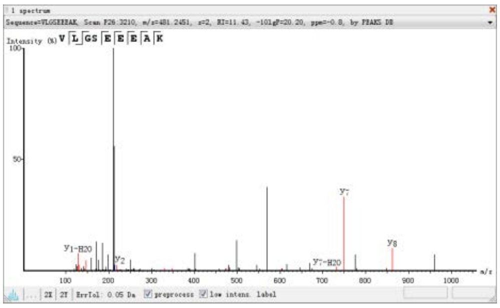

Fig.S4 The spectra of the proteins identified by one unique peptide with only one spectrum in HY1. Upper is the accession of protein, and under is its corresponding spectrum.

gi|224112777

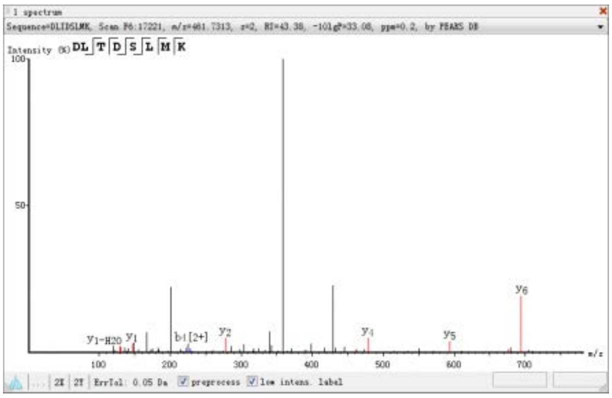

gi|731416007

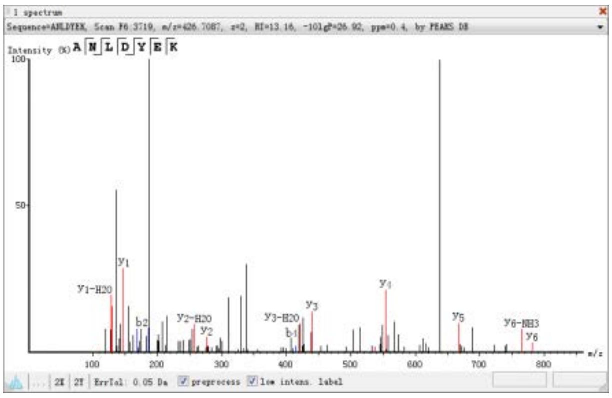

gi|225426414

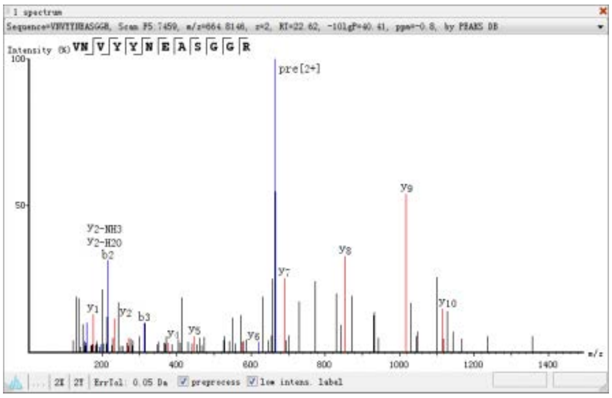

gi|224052904

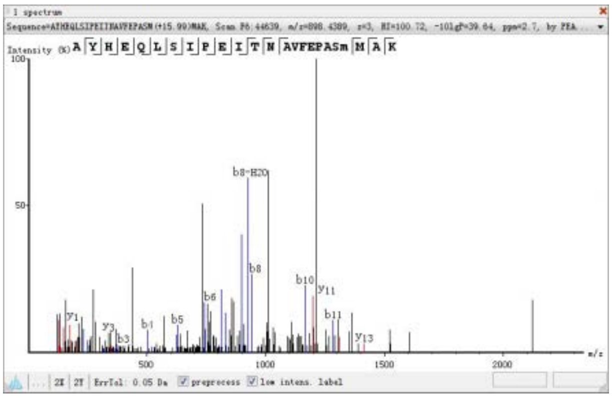

gi|225437549

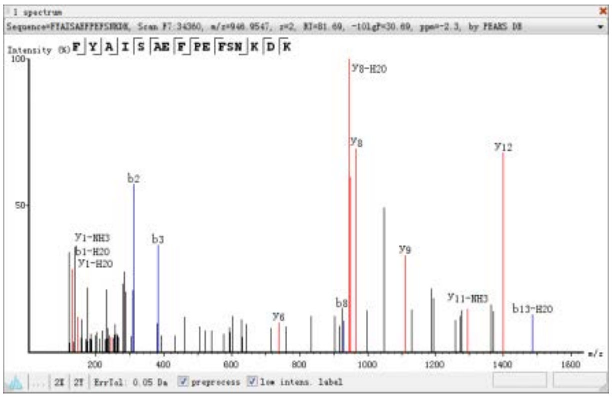

gi|566206349

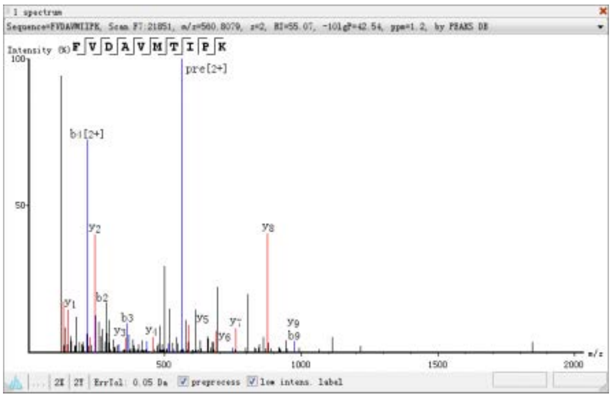

gi|731407424

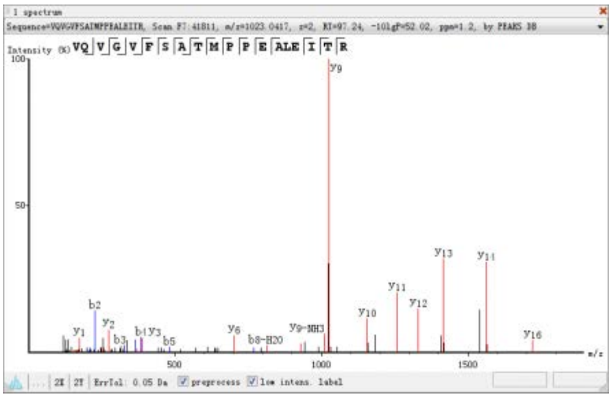

gi|731427869

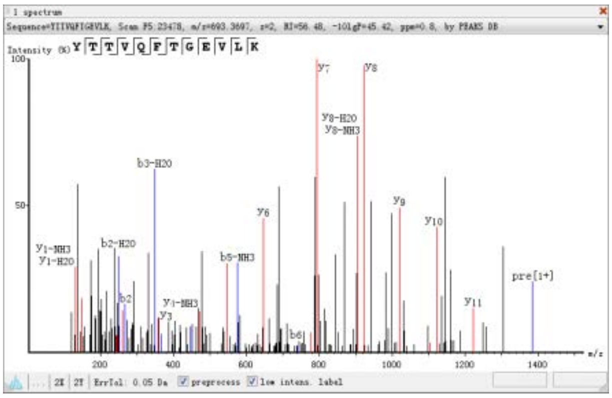

gi|224097588

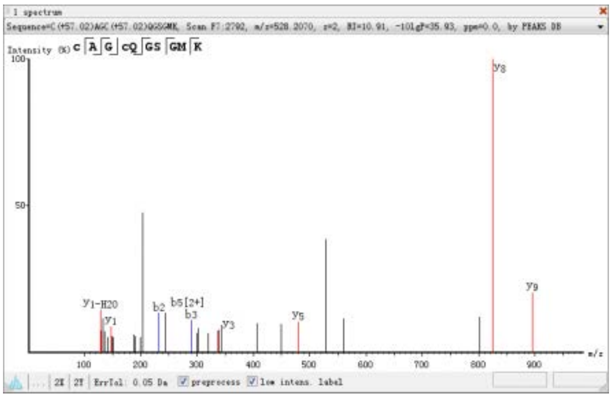

gi|224136316

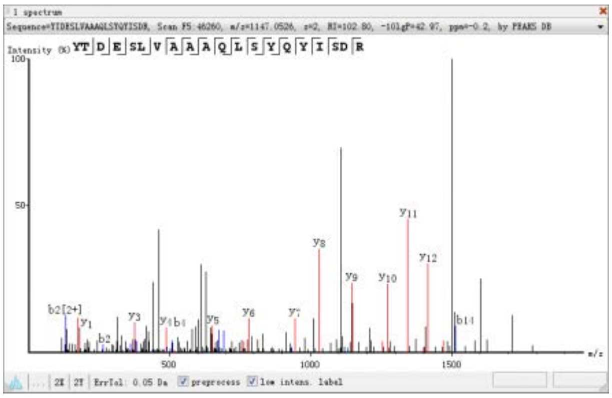

gi|566170554

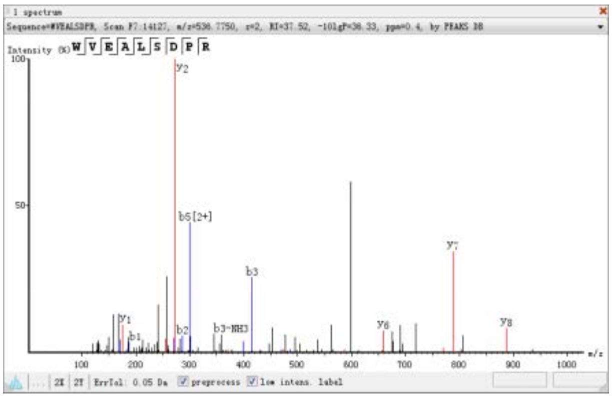

gi|731418890

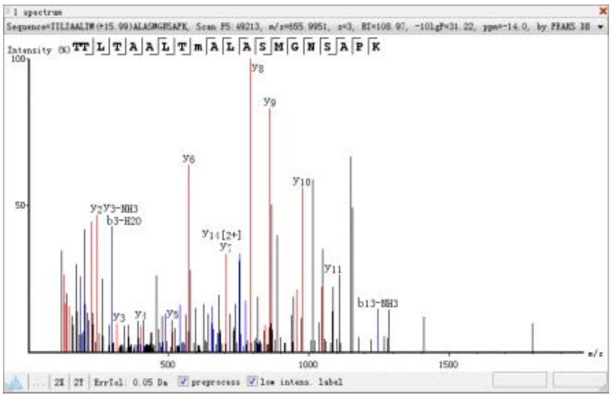

gi|225442805

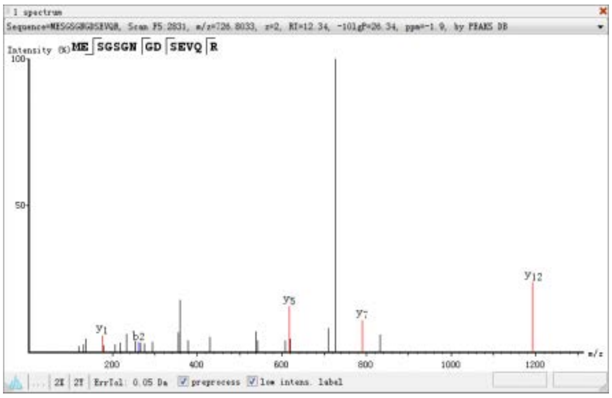

gi|566148111

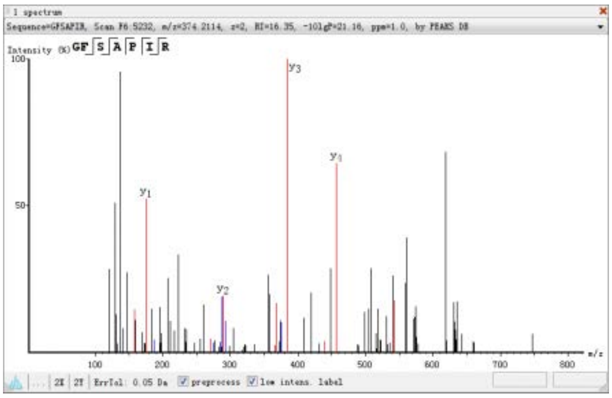

gi|566205887

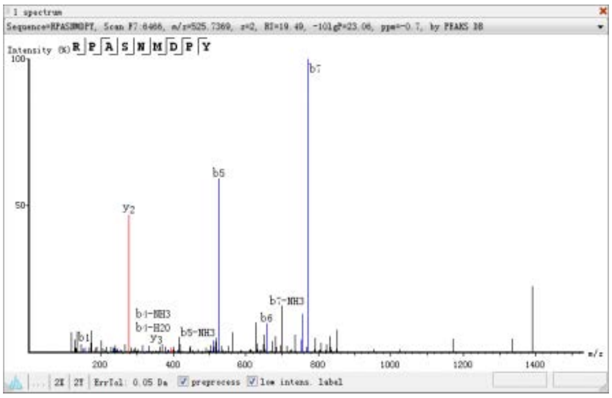

gi|224069951

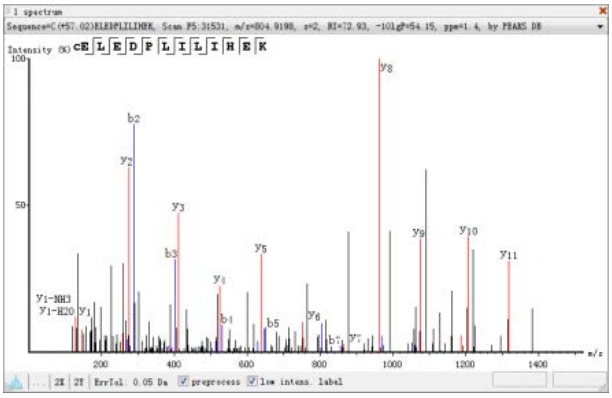

gi|225434259

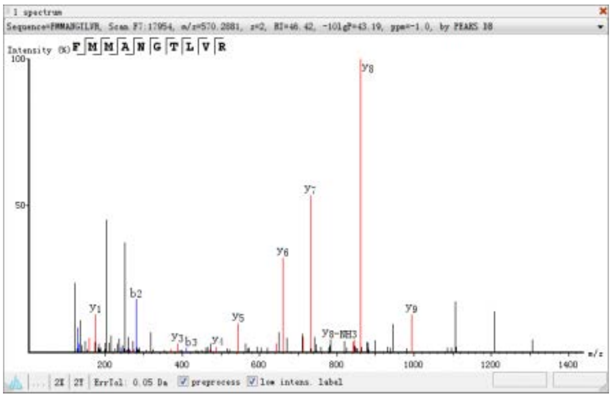

gi|225451806

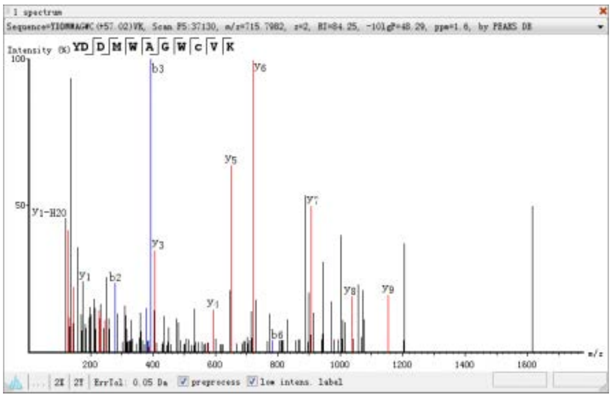

gi|566157403

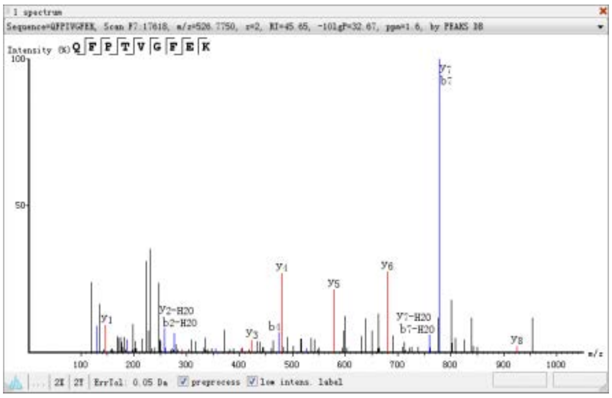

gi|566157671

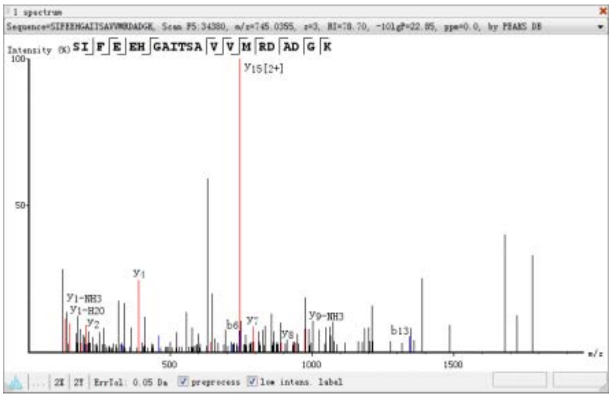

gi|224135653

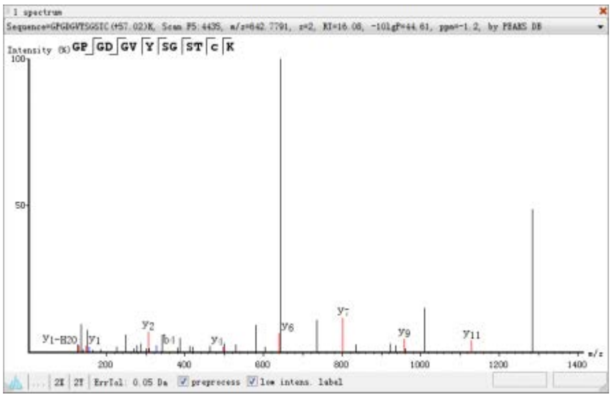

gi|225426194

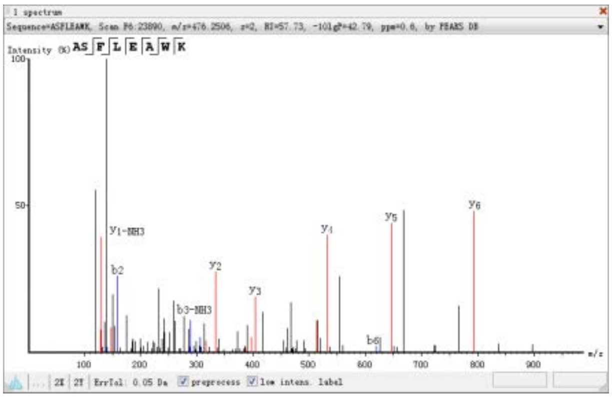

gi|566199099

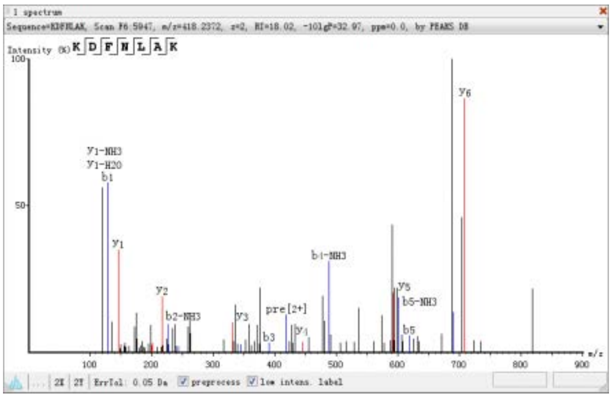

gi|225437493

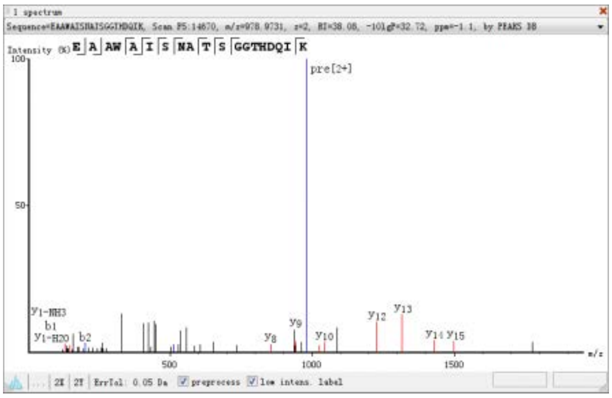

gi|224109580

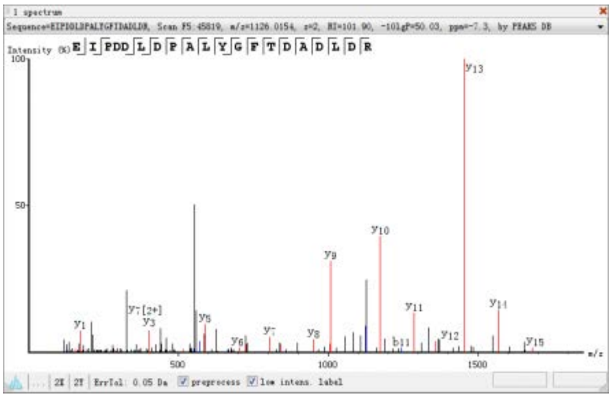

gi|566193834

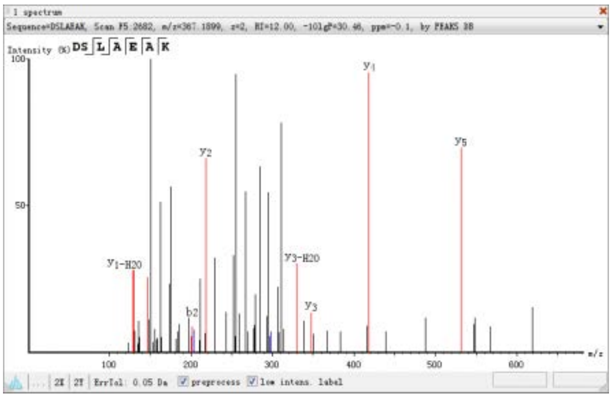

gi|225434588

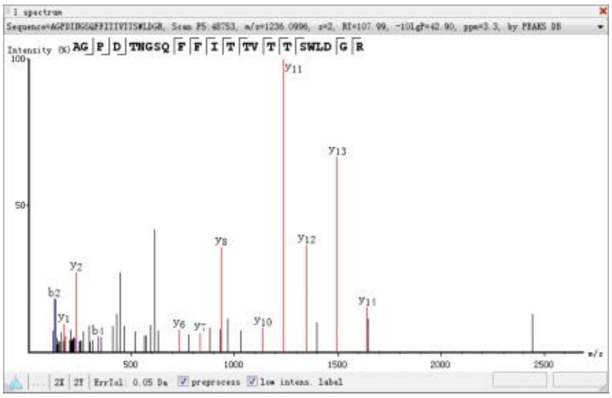

gi|731412938

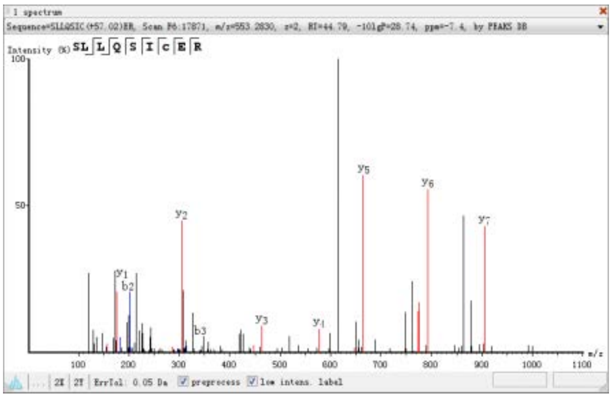

gi|224109062

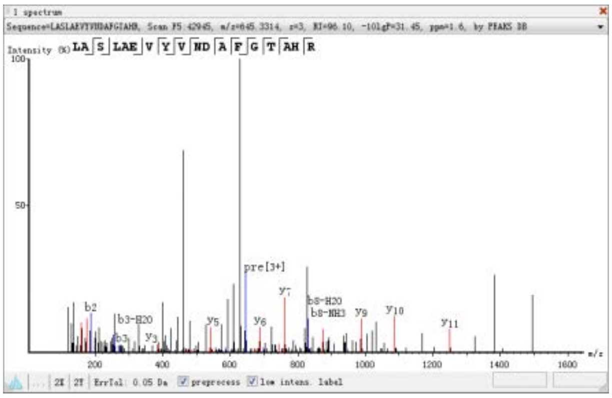

gi|224097508

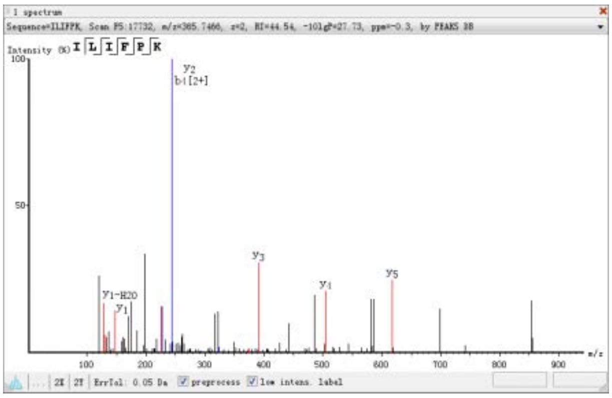

gi|224059642

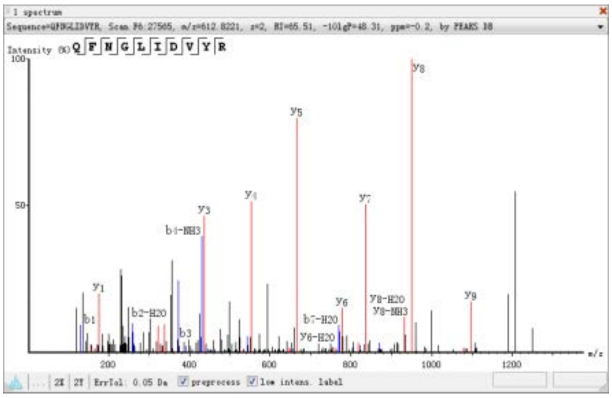

gi|225462272

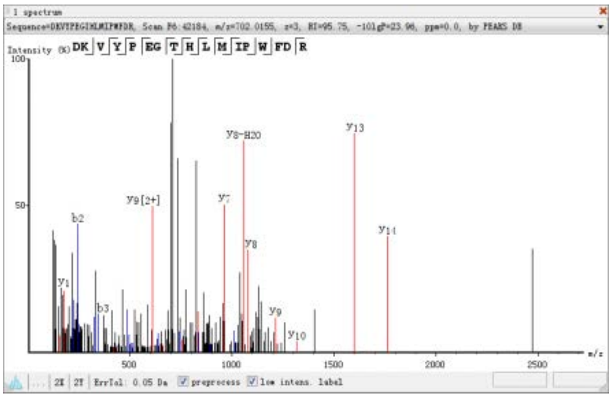

gi|566213236

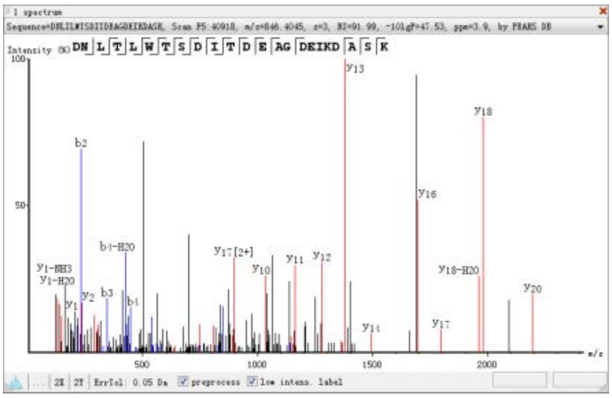

gi|224077927

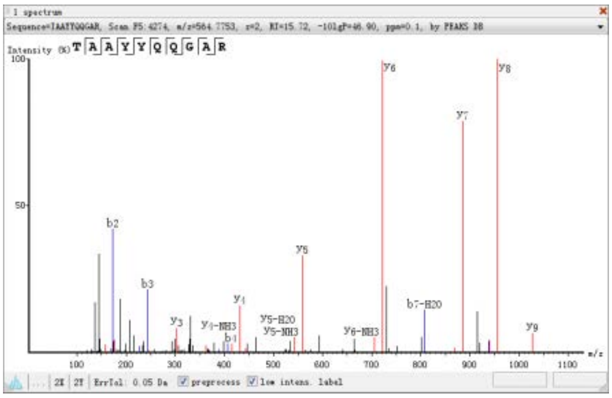

gi|359475502

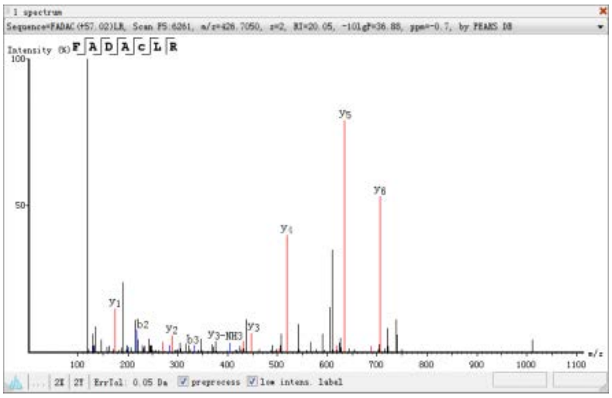

gi|224084622

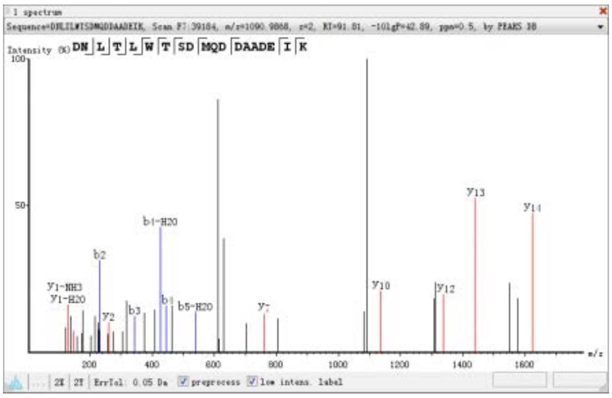

gi|359473310

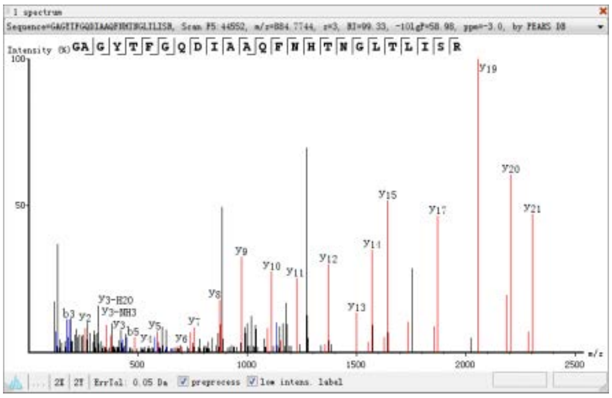

gi|224092184

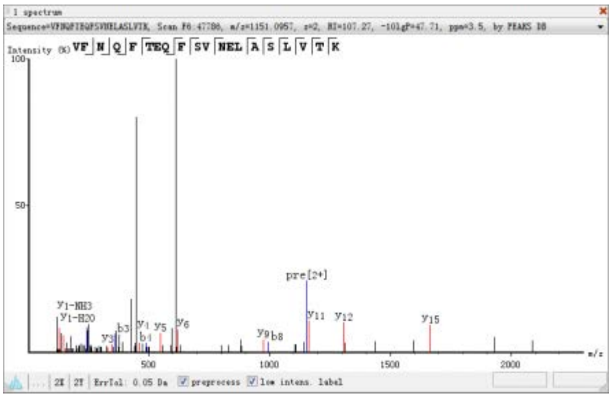

gi|225446595

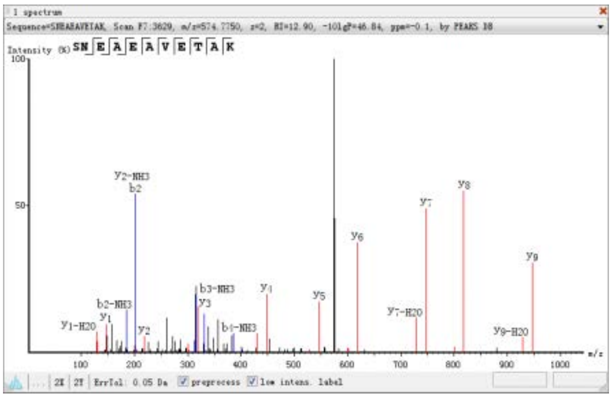

gi|224122988

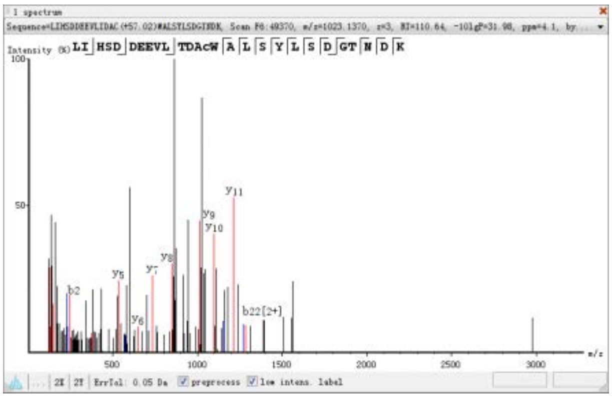

gi|224108774

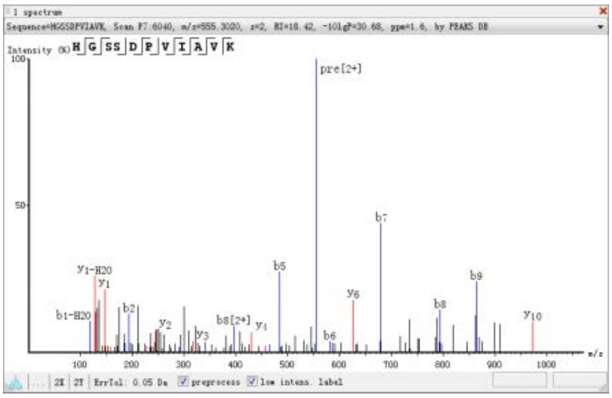

gi|224107259

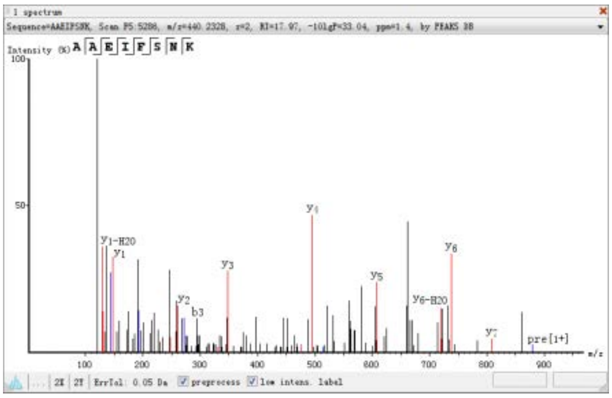

gi|225442061

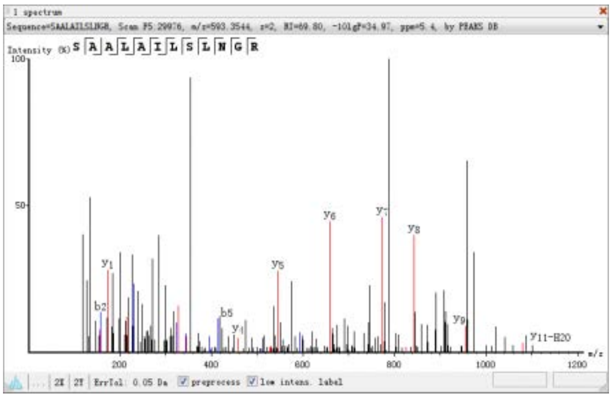

gi|566166593

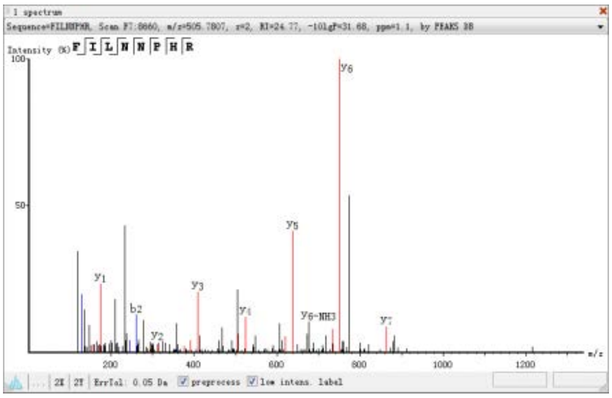

gi|731392629

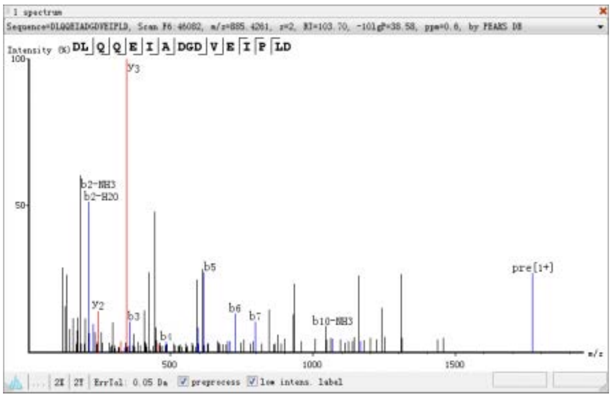

gi|566176021

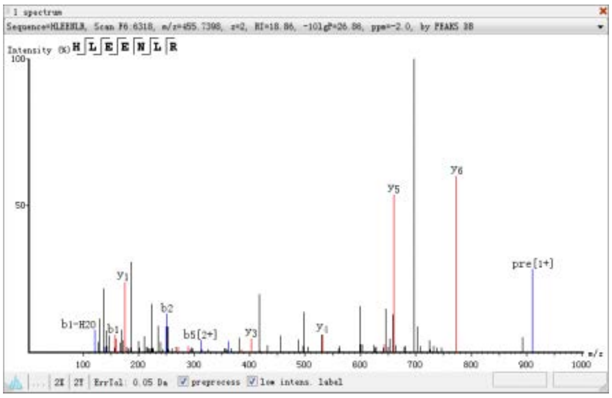

gi|224126969

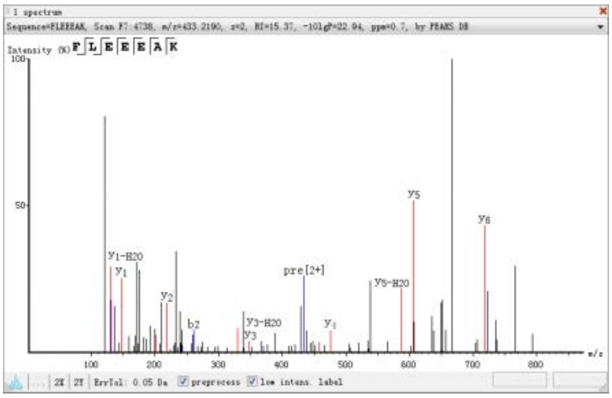

gi|731431241

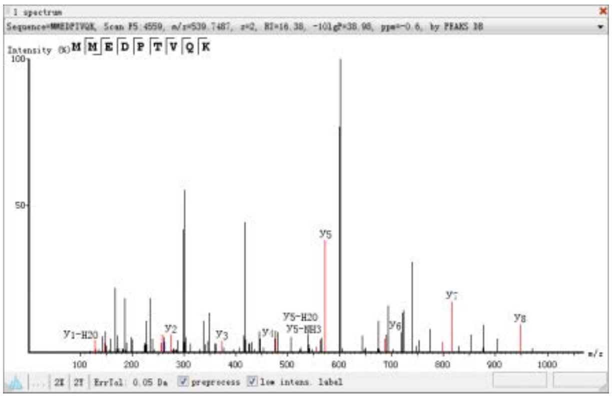

gi|225461808

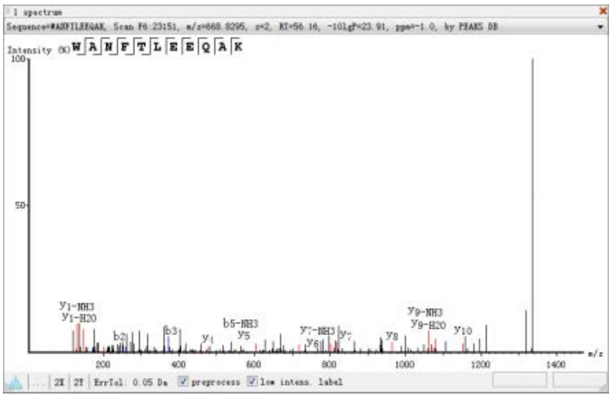

gi|566149060

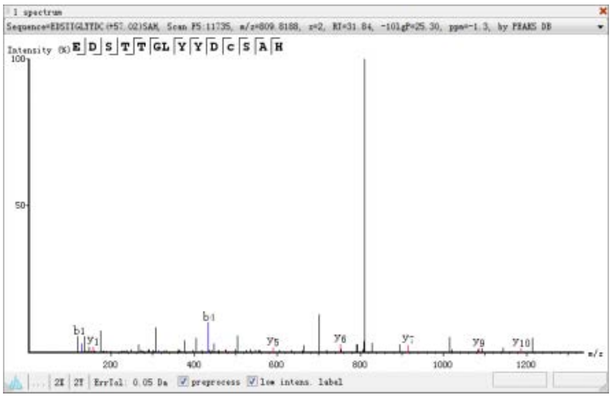

gi|566179689

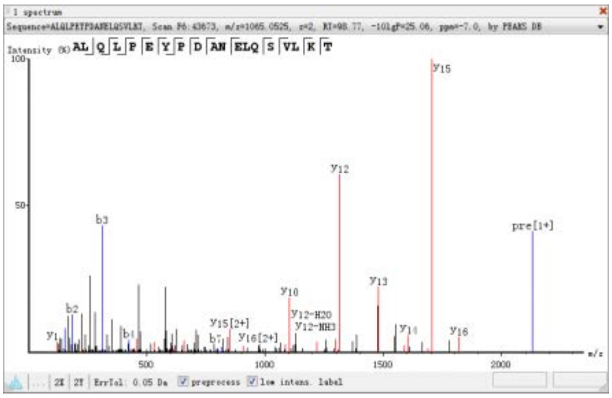

gi|225464635

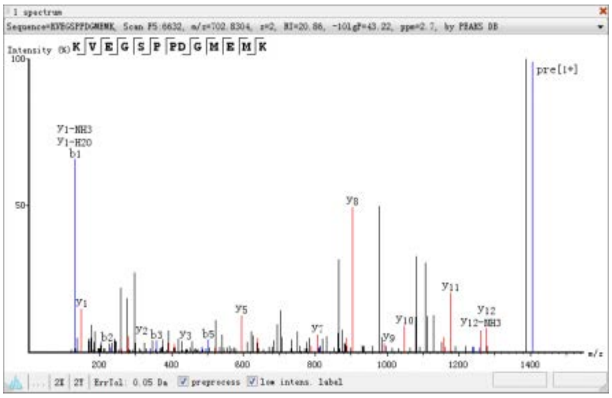

gi|526117351

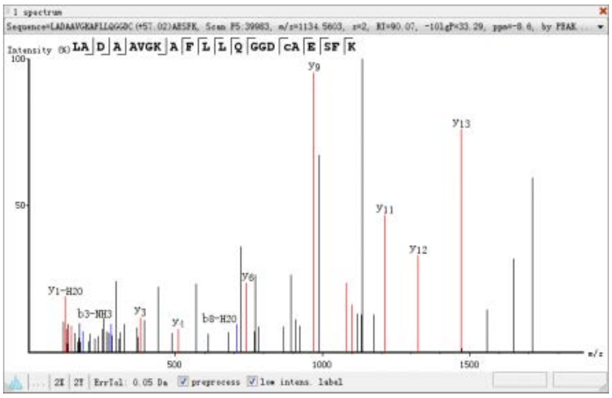

gi|225452831

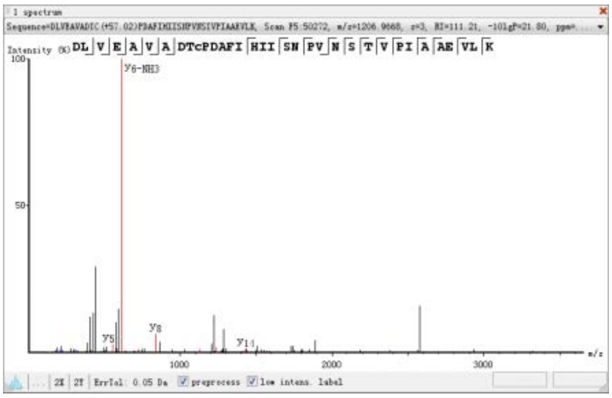

gi|566210556

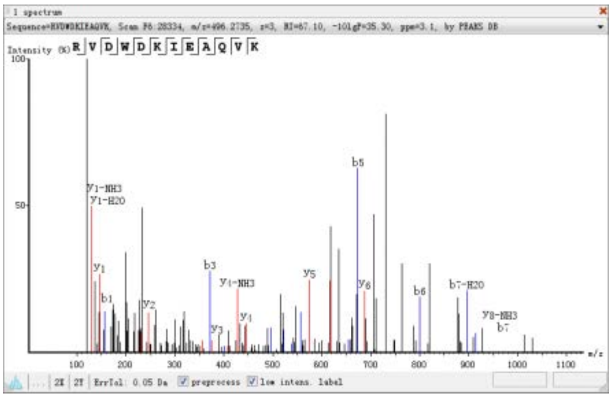

gi|359480225

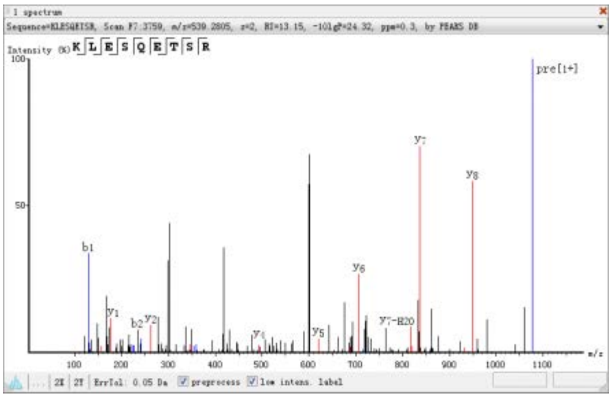

gi|224075876

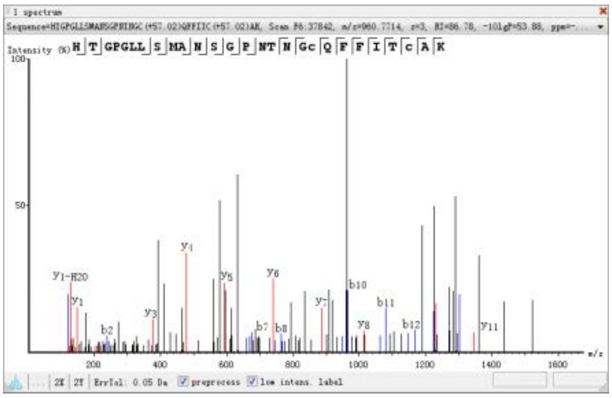

gi|225449380

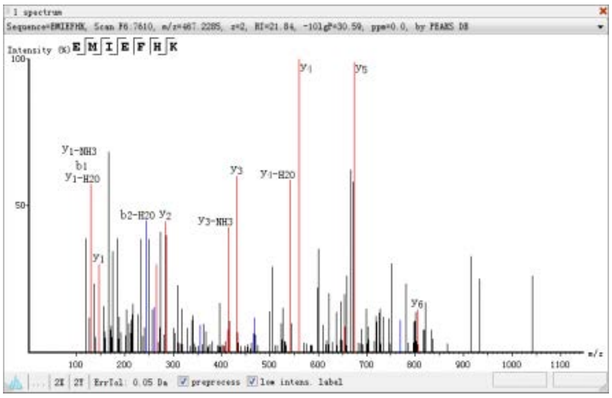

gi|224063643

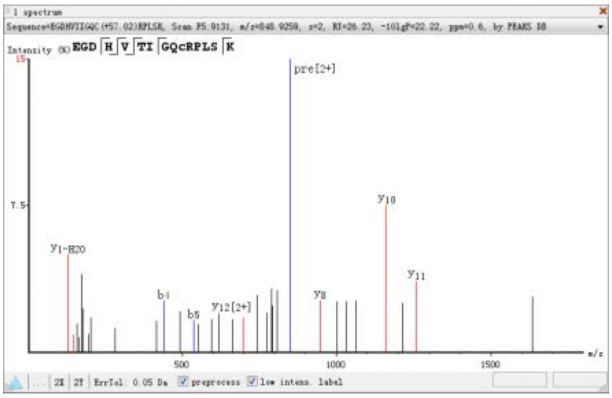

gi|225445396

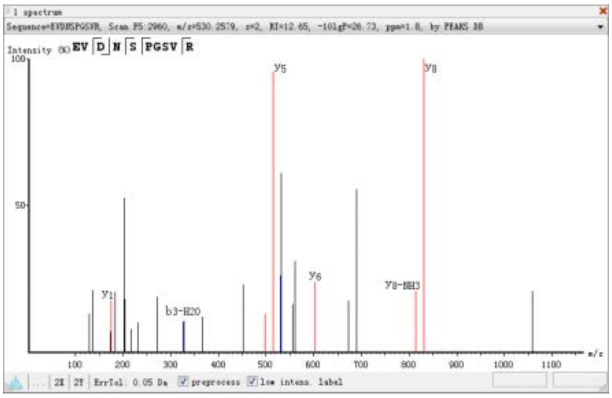

gi|566179485

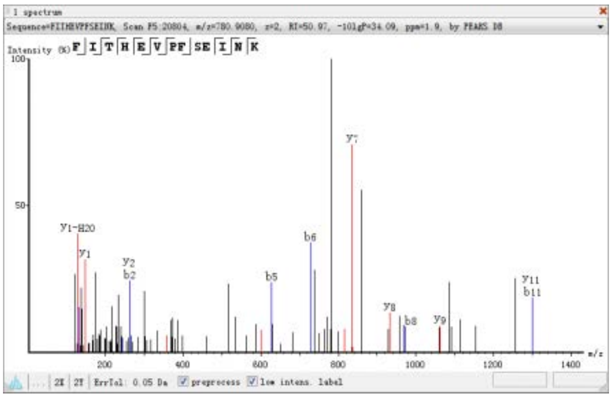

gi|566186484

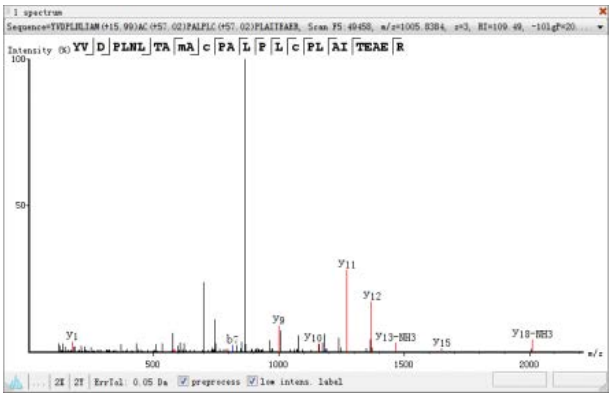

gi|566168605

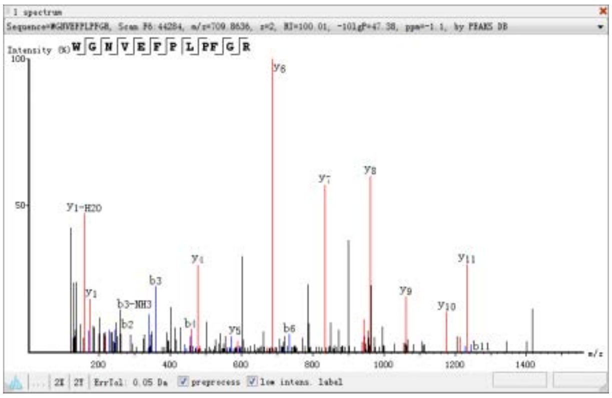

gi|359494701

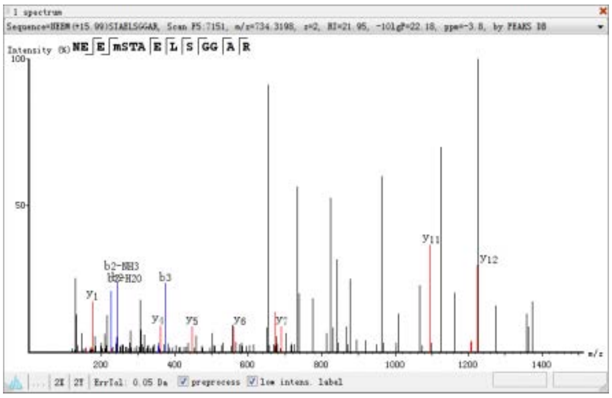

gi|224088282

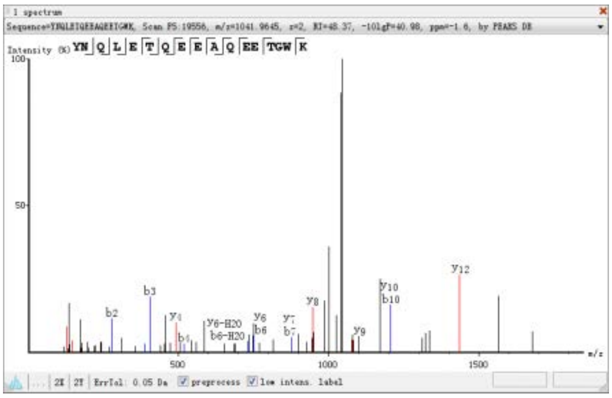

gi|566147610

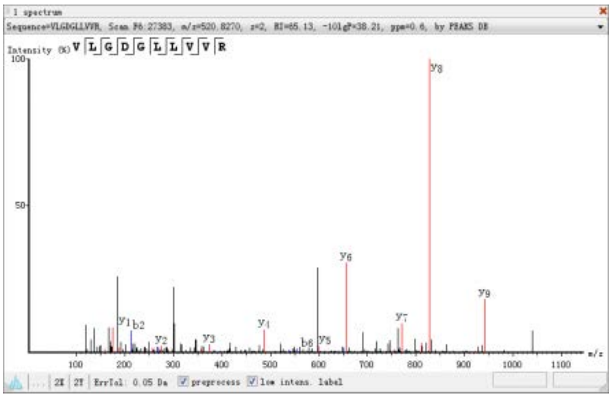

gi|566163024

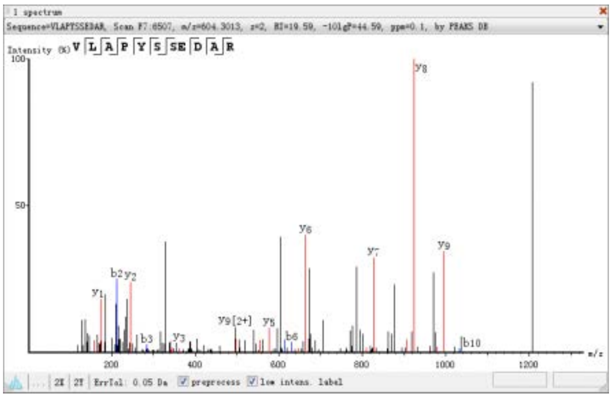

gi|225453620

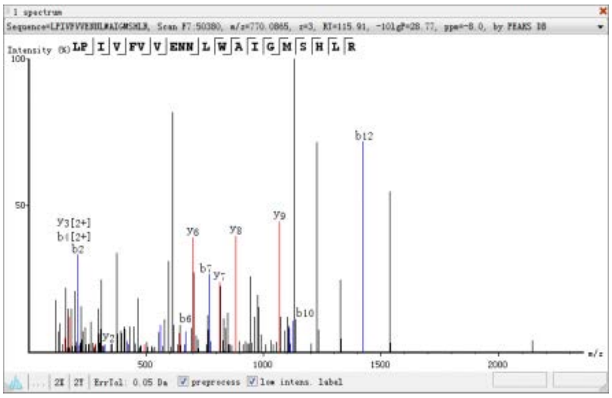

gi|566200085

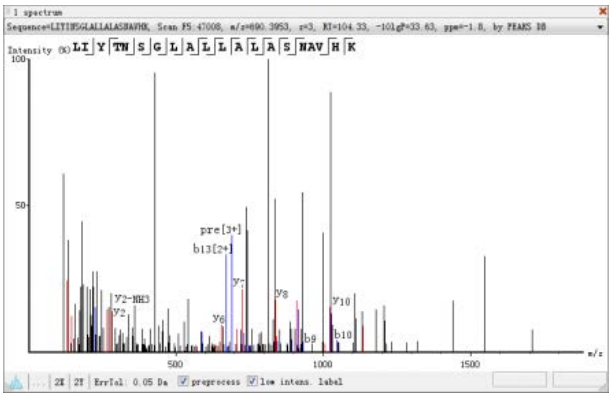

gi|225440898

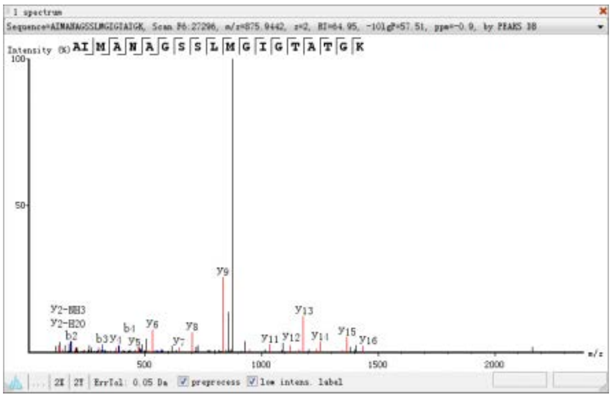

gi|225438095

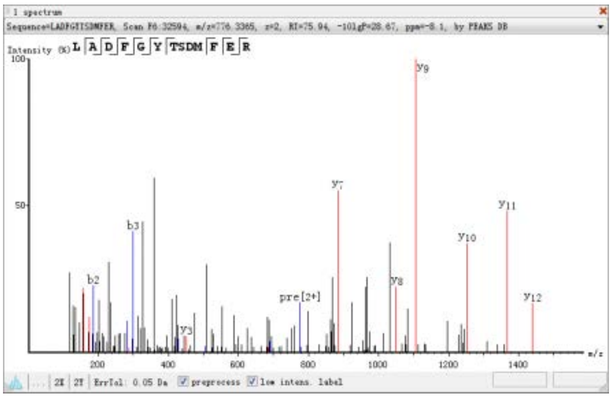

gi|731386341

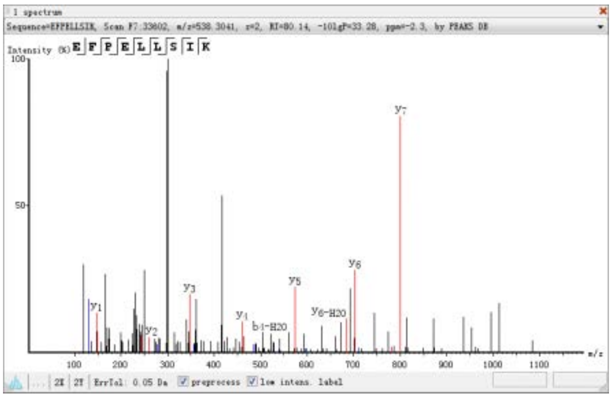

gi|225435632

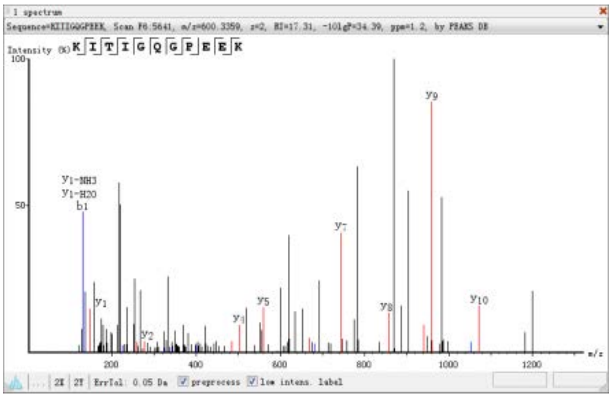

gi|731402959

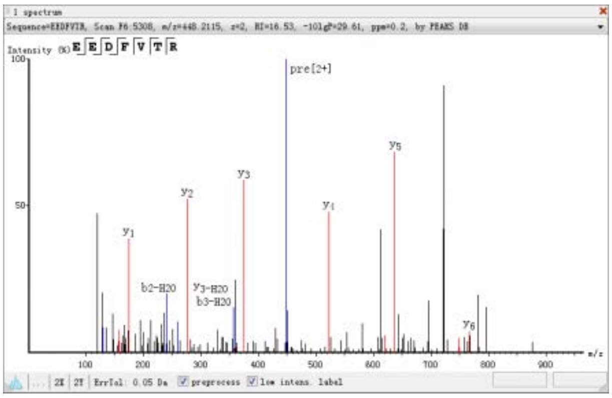

gi|566197725

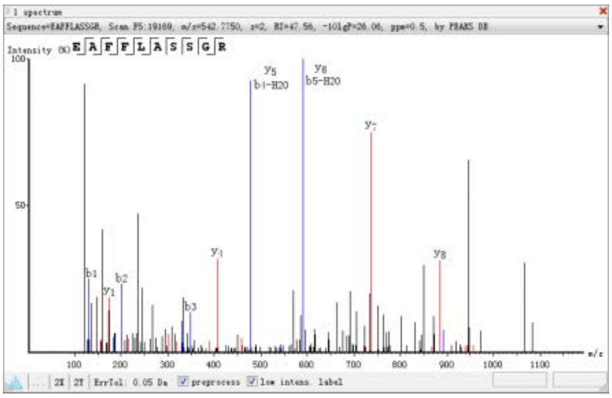

gi|566168414

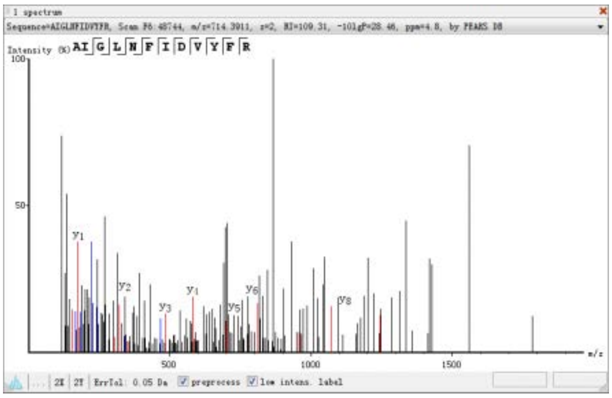

gi|566212181

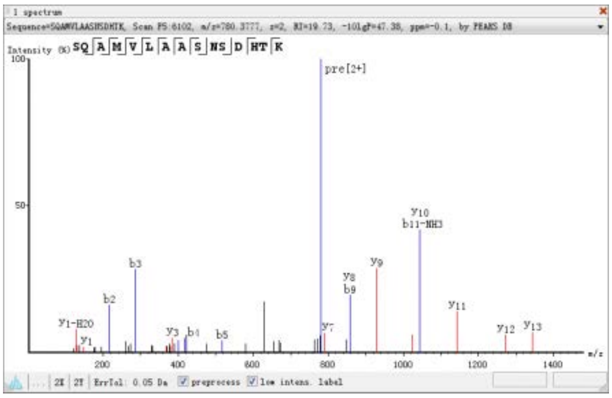

gi|526117665

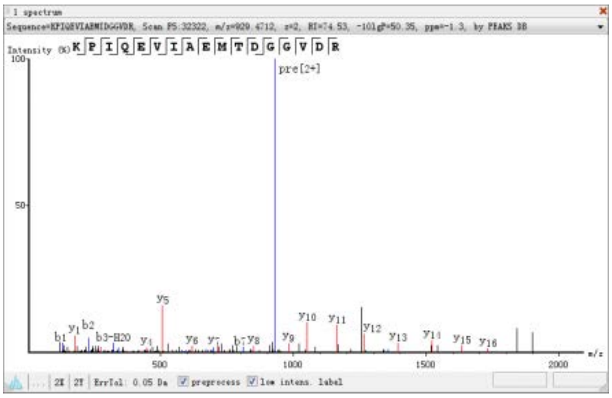

gi|359494275

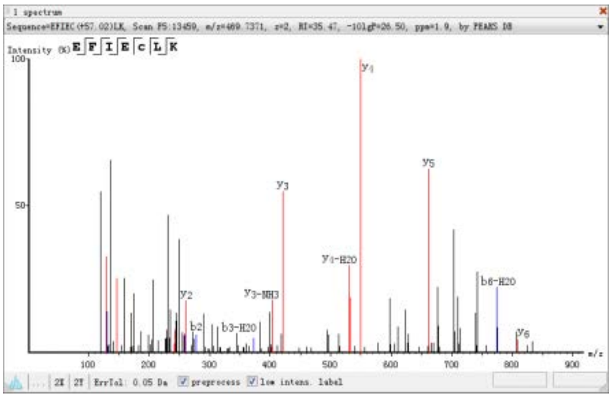

gi|225442910

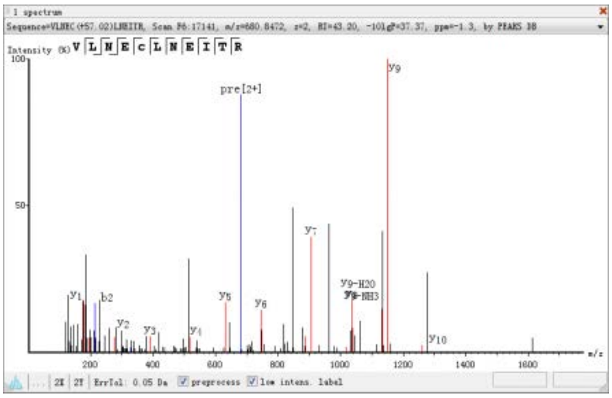

gi|225462066

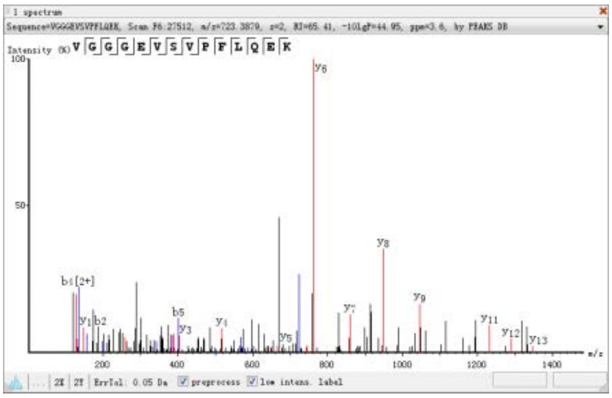

gi|224072208

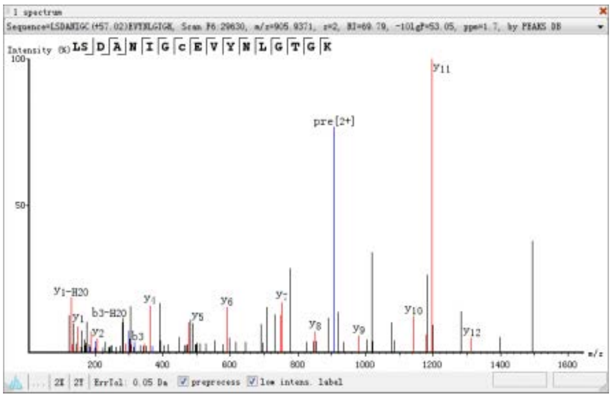

gi|566174827

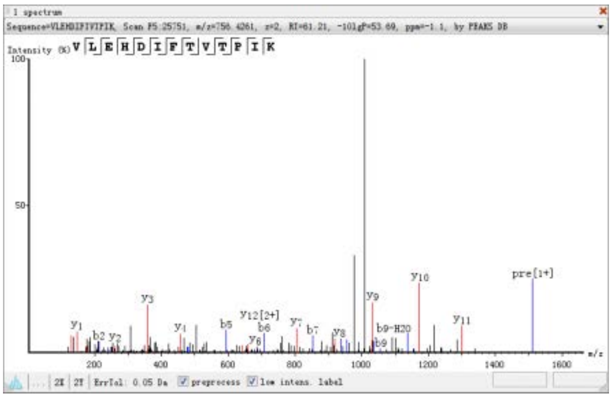

gi|225445166

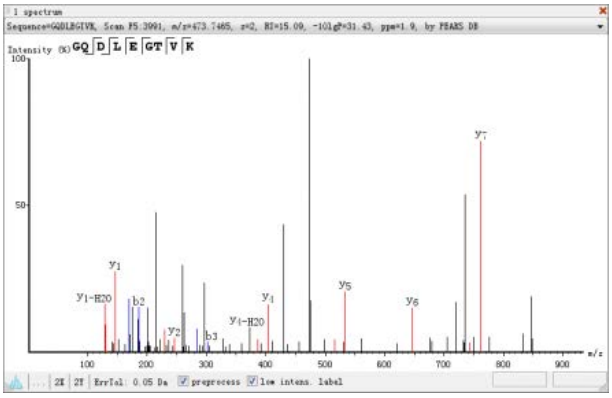

gi|225458719

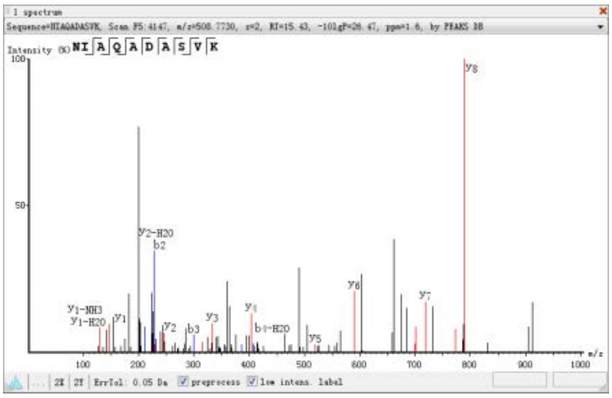

gi|566196811

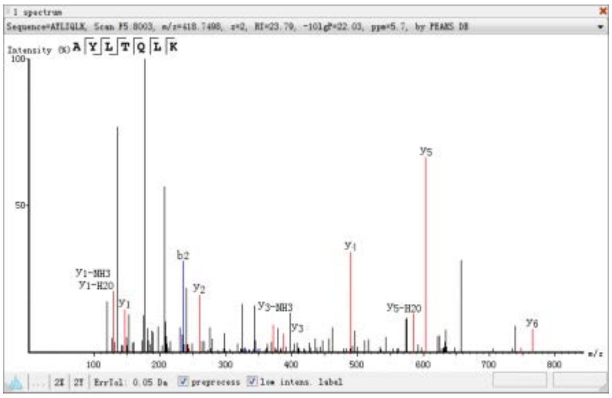

gi|225433424

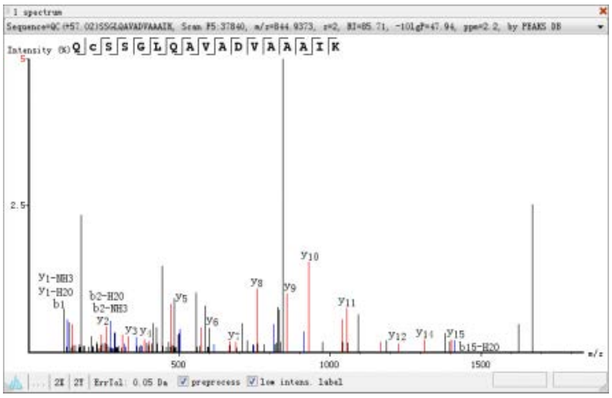

gi|731422323

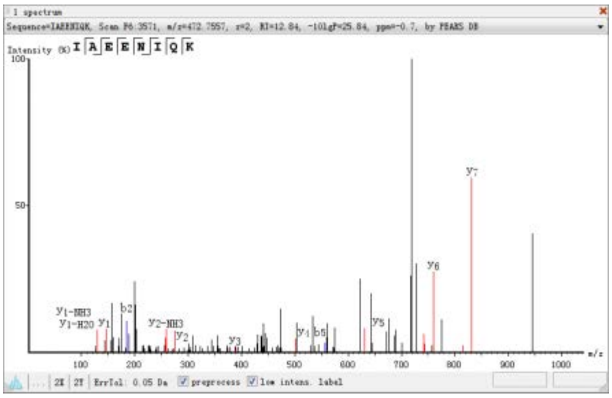

gi|225461906

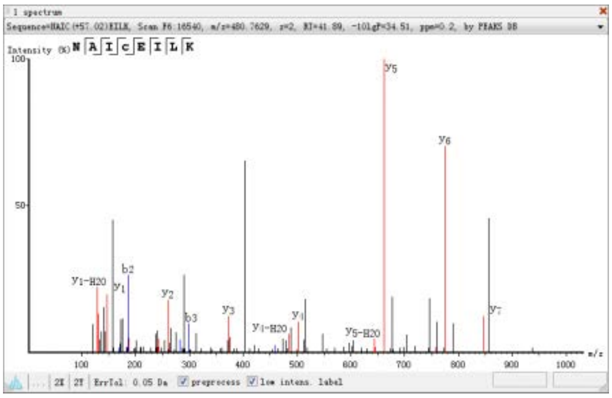

gi|225452646

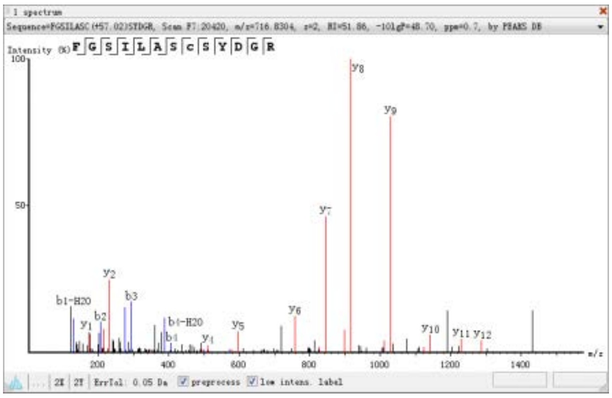

gi|224097915

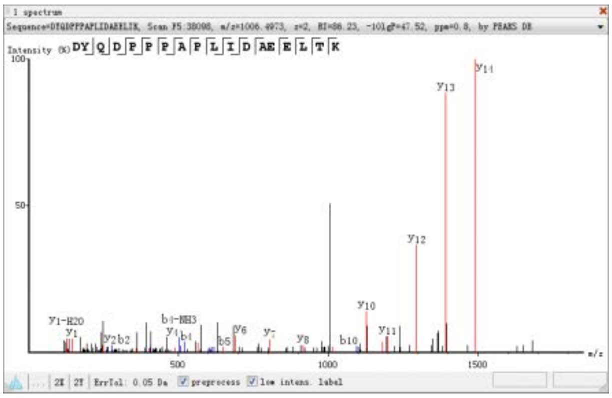

gi|731392621

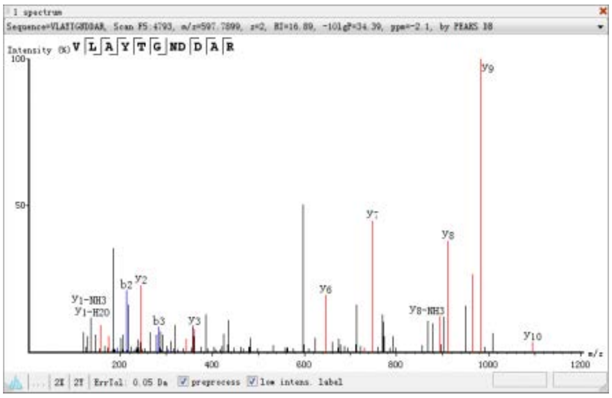

gi|566215059

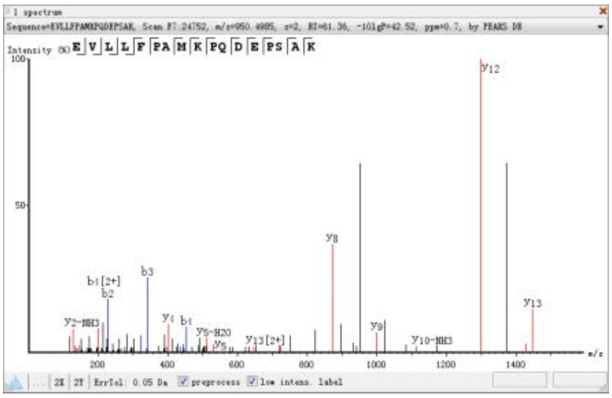

gi|224087393

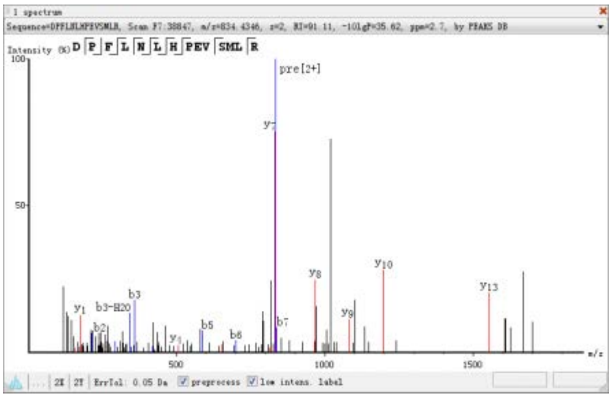

gi|225450677

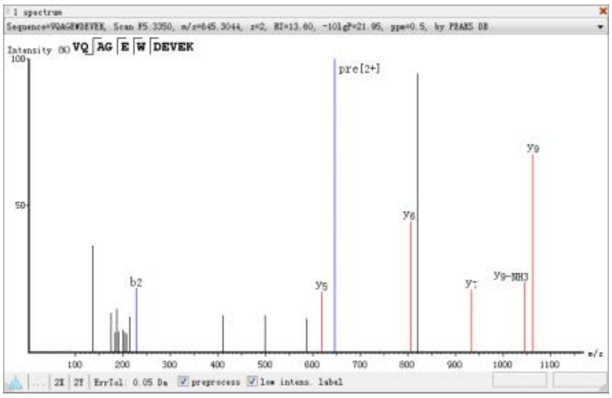

gi|225451233

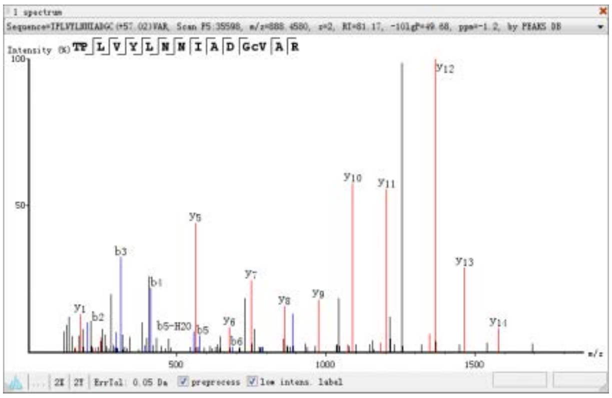

gi|224054773

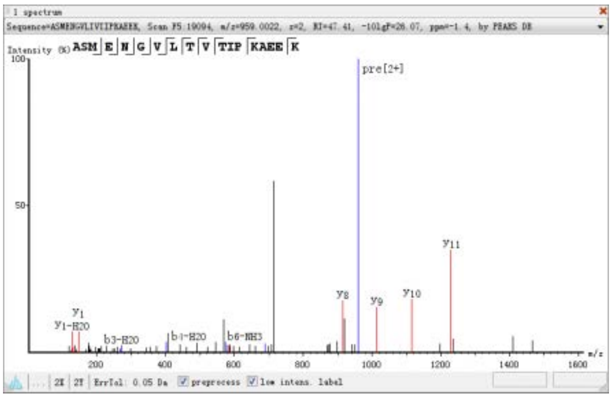

gi|224074217

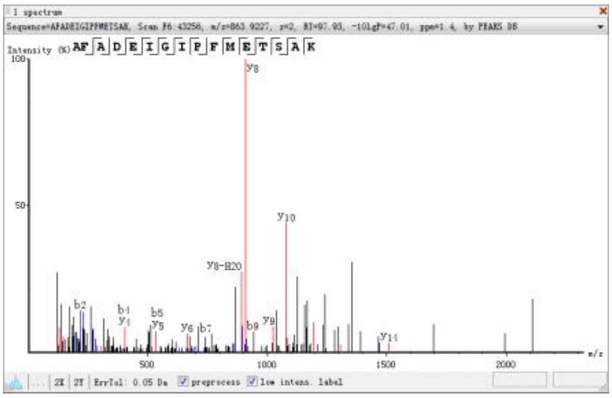

gi|731393107

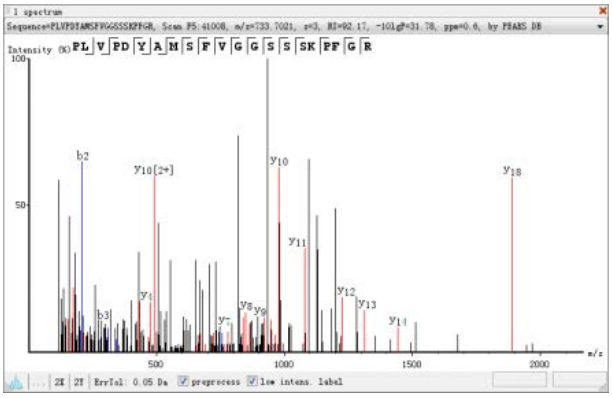

gi|566153407

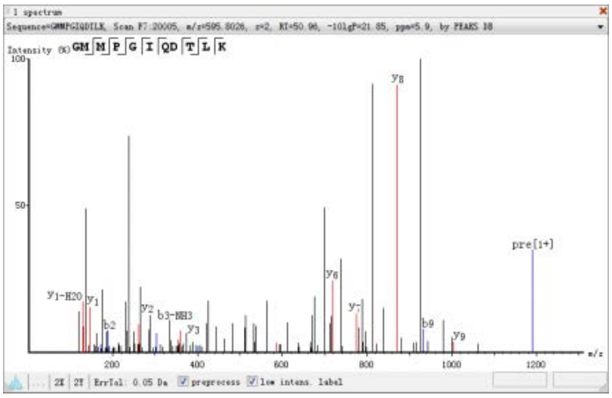

gi|566200709

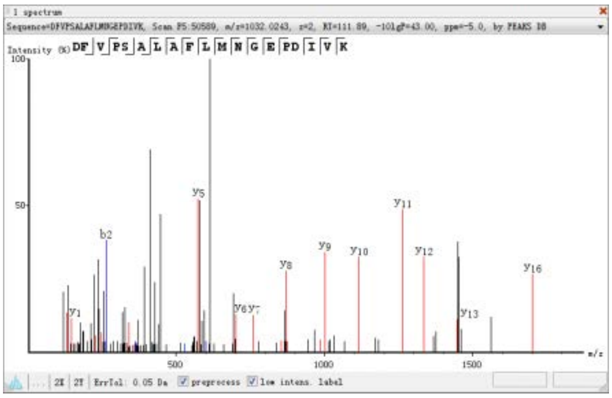

gi|566214594

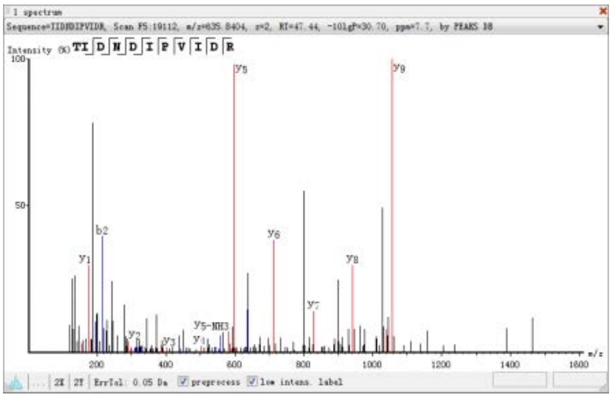

gi|225465870

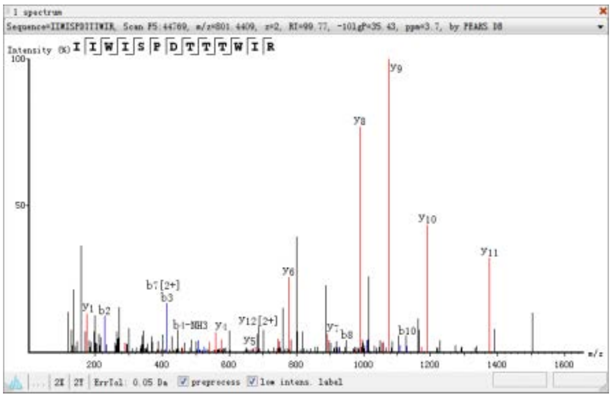

gi|566176600

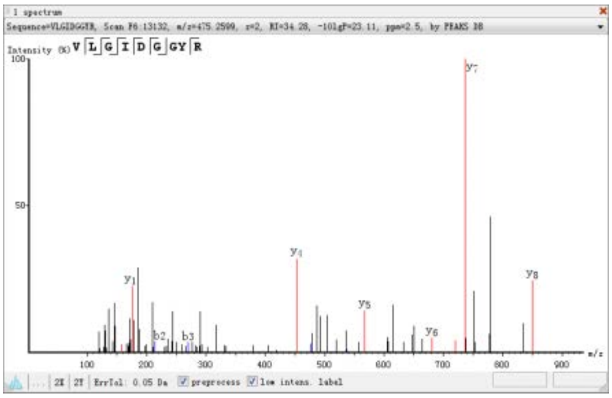

gi|566197479

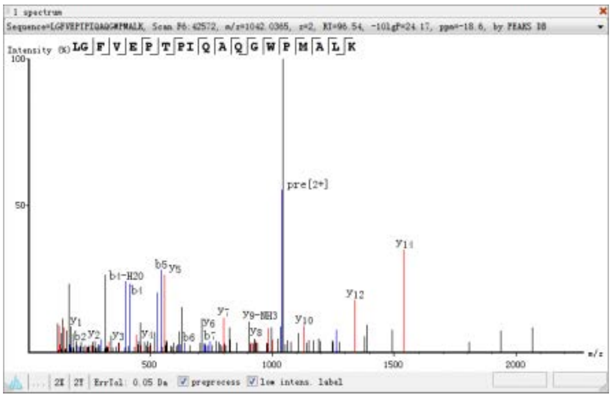

gi|225430510

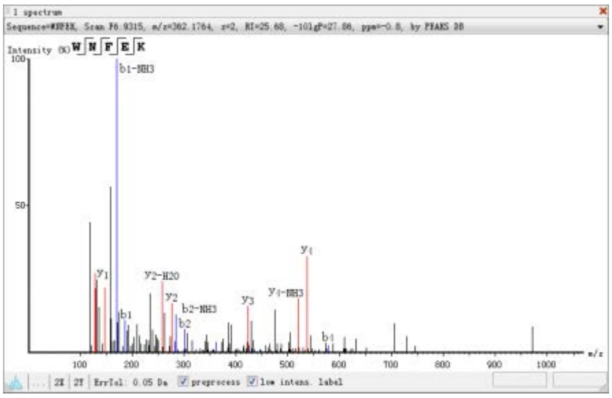

gi|566253285

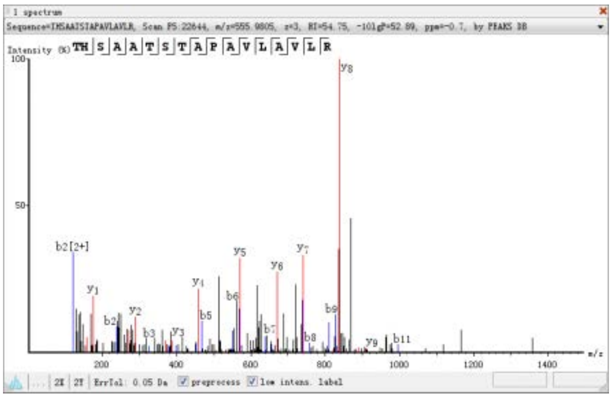

gi|225427234

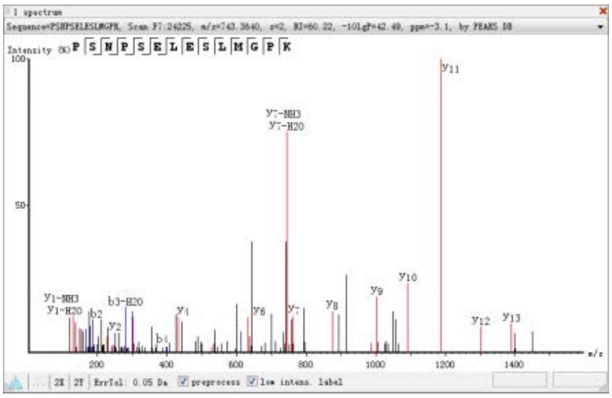

gi|731391337

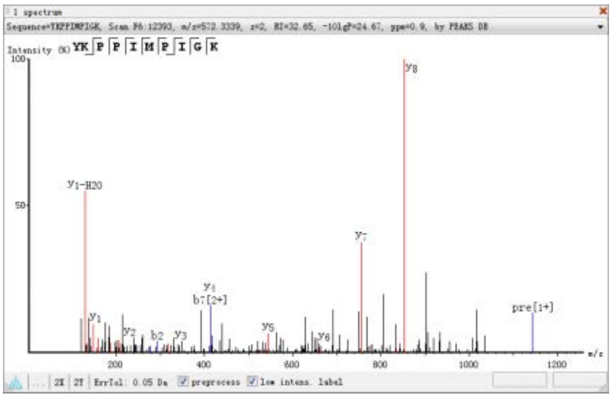

gi|731430343

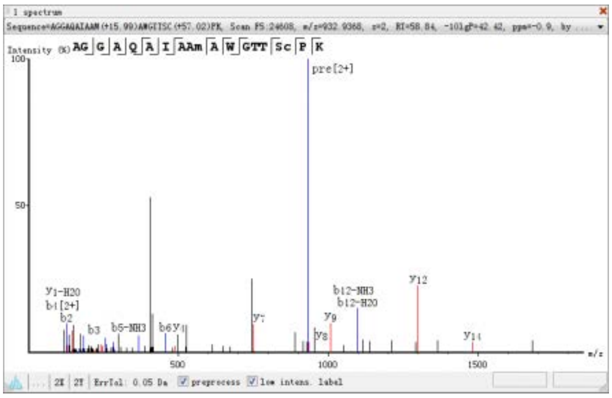

gi|359474156

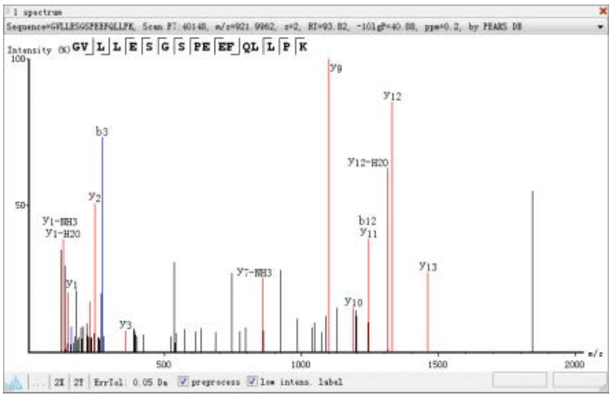

gi|225428566

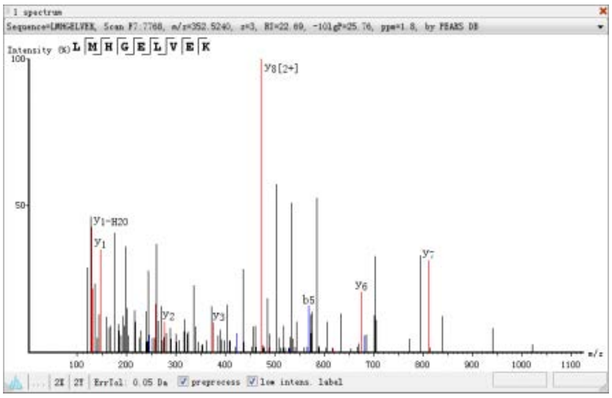

gi|225444609

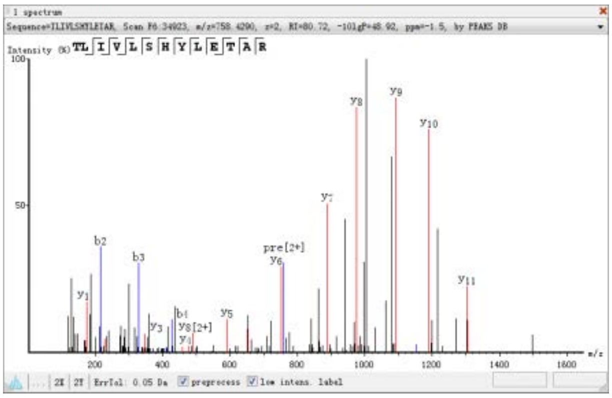

gi|224096918

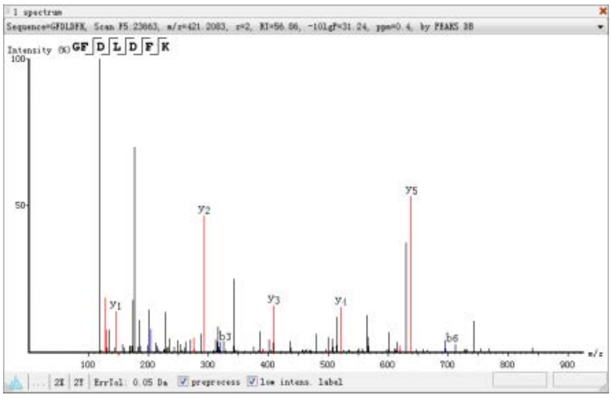

gi|566256791

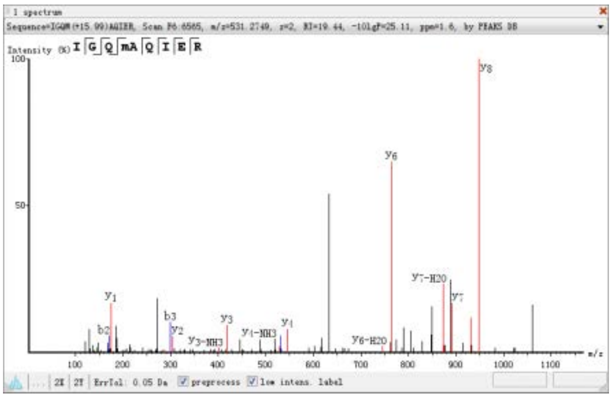

gi|566208913

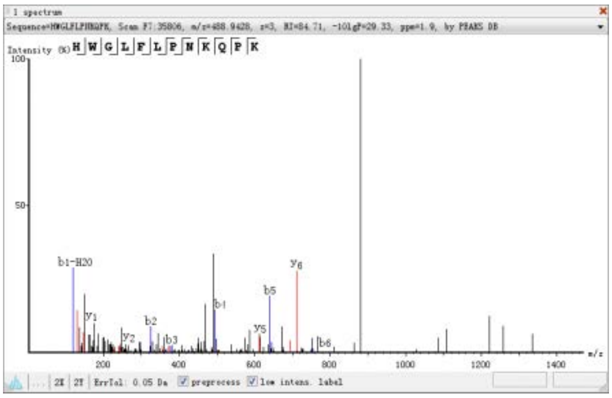

gi|225440480

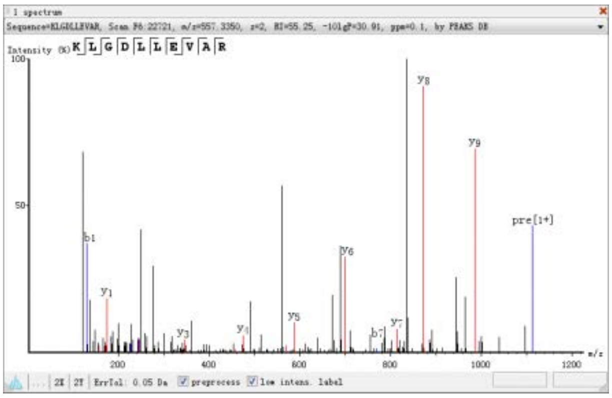

gi|566209593

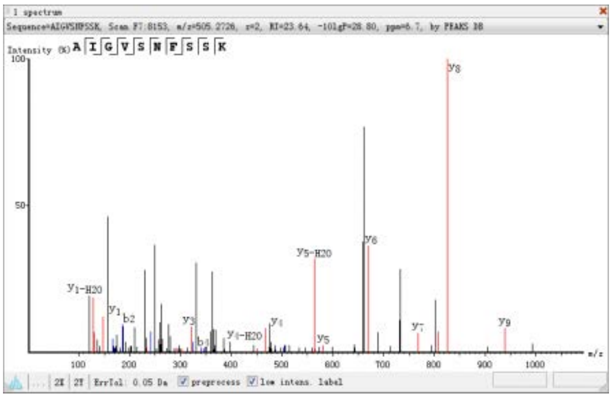

gi|224103461

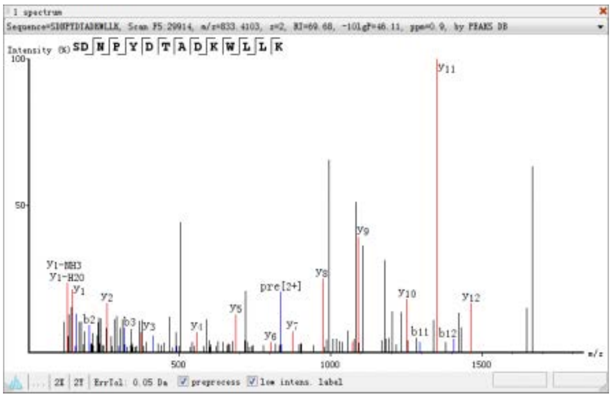

gi|224146178

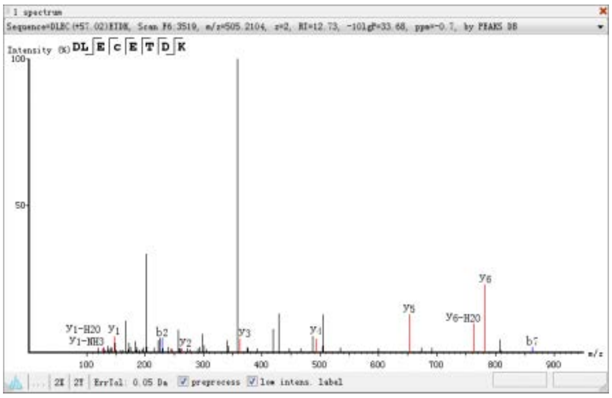

gi|566148153

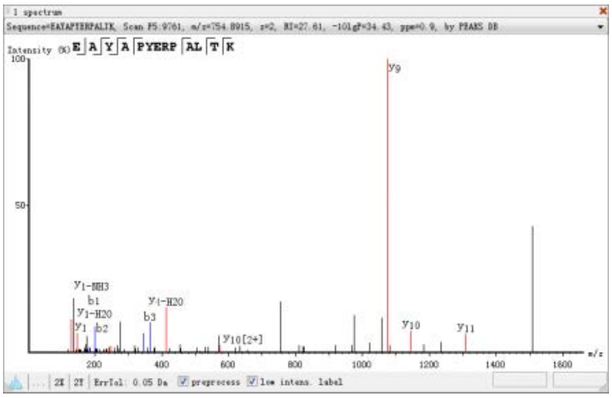

gi|566177670

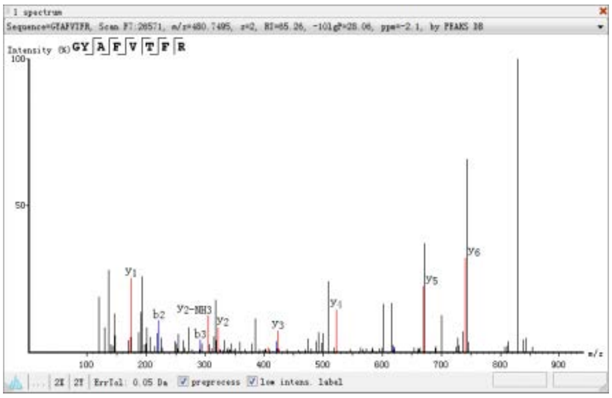

gi|225442434

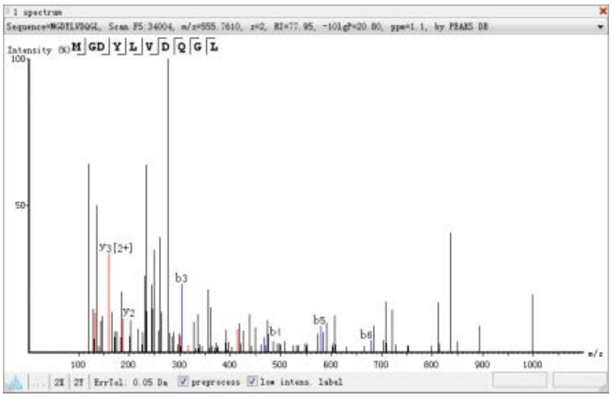

gi|225437515

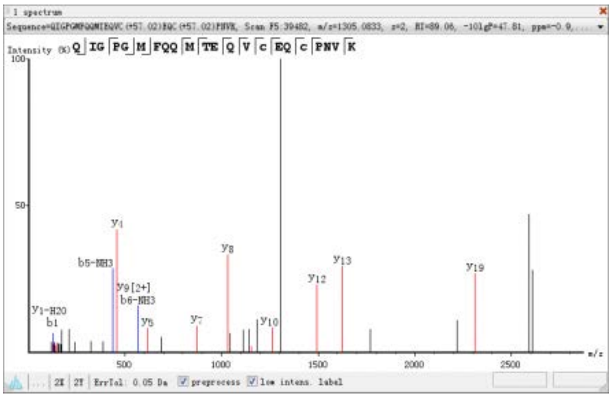

gi|566205979

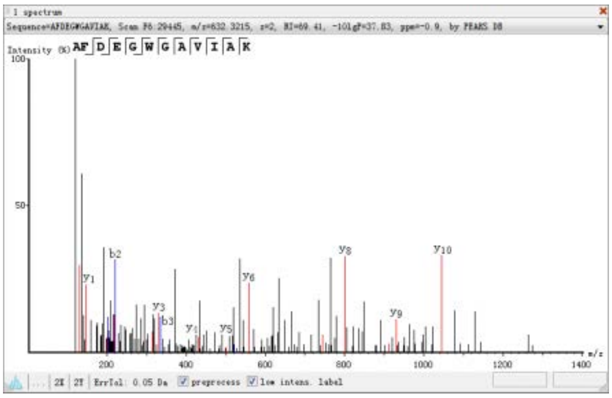

gi|526117828

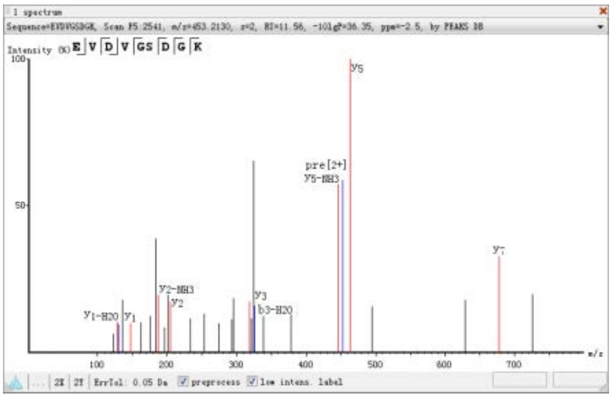

gi|731369373

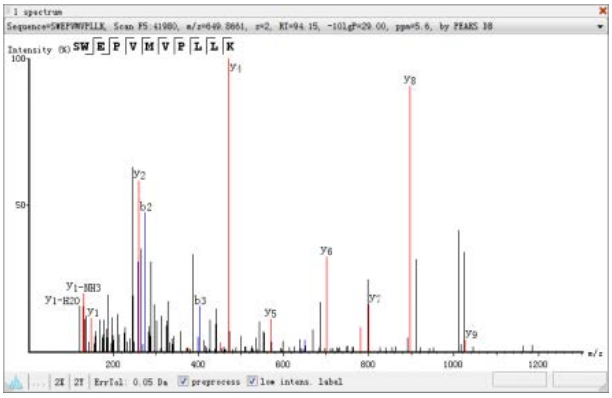

gi|566181279

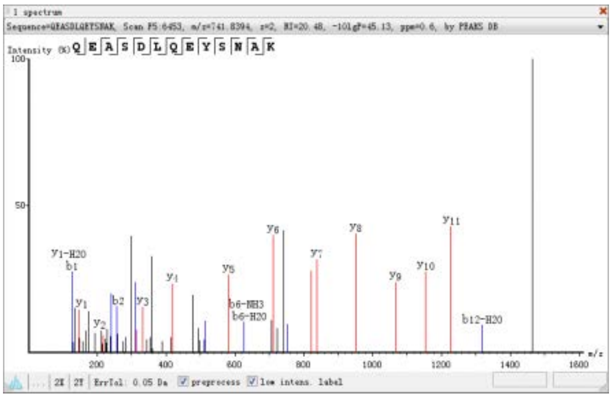

gi|224141563

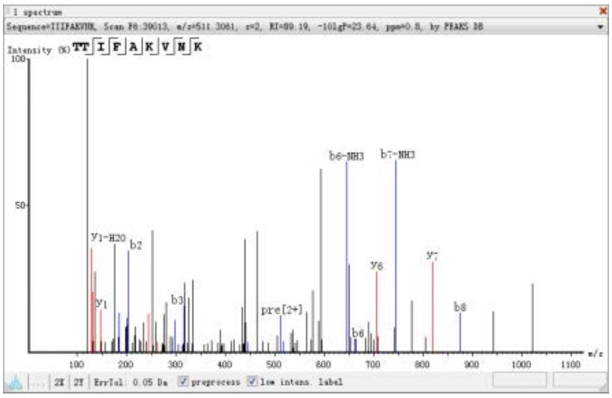

gi|731410110

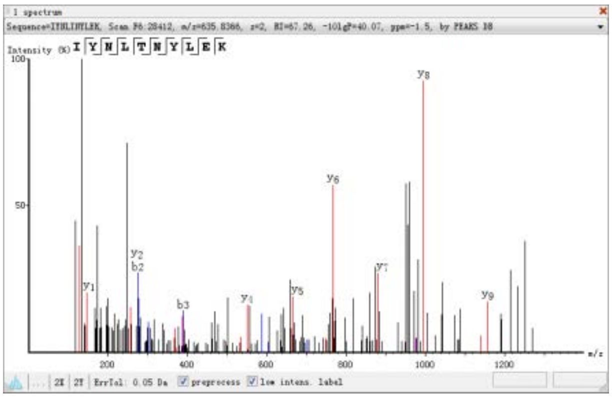

gi|225433676

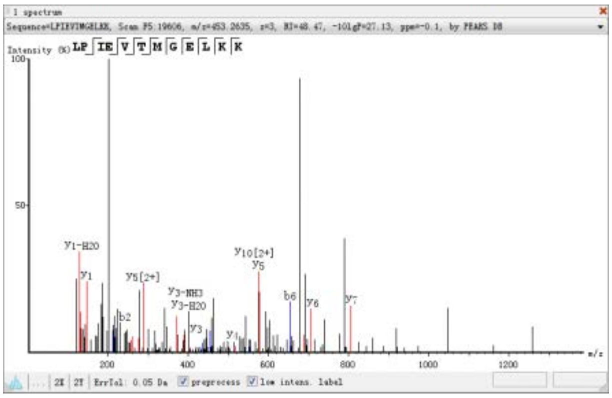

gi|566170295

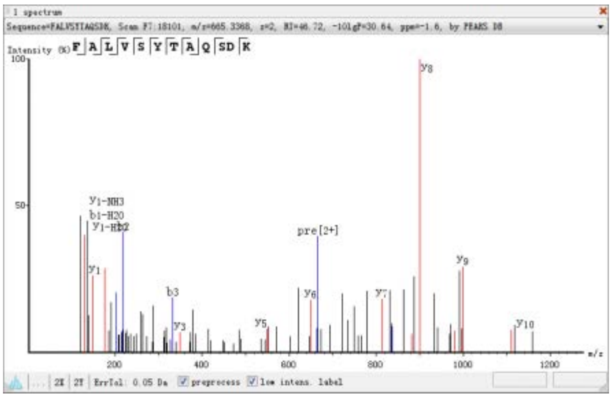

gi|731402065

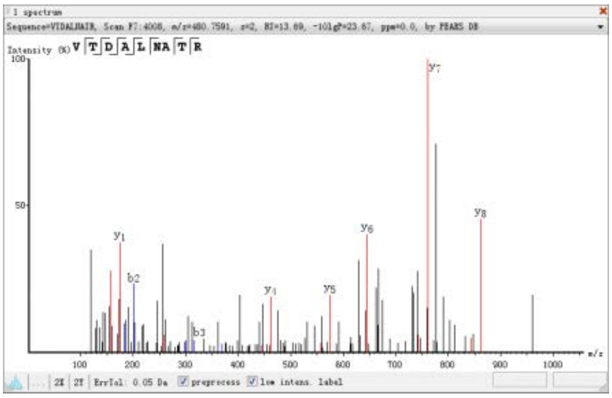

gi|224108087

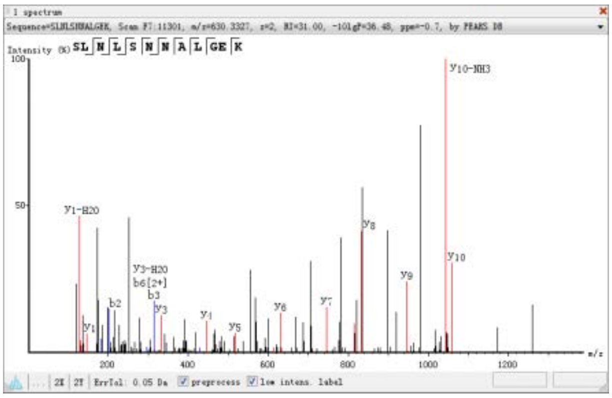

gi|731405420

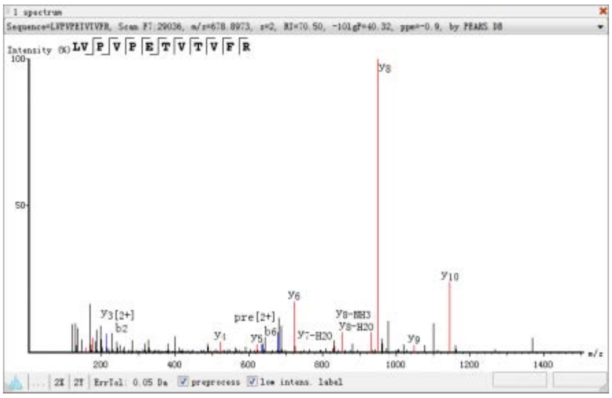

gi|225448433

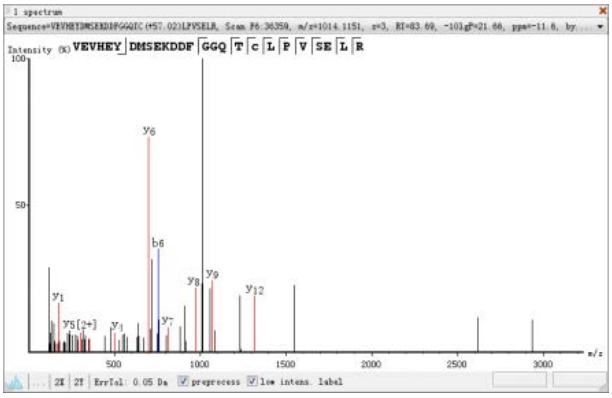

gi|225455386

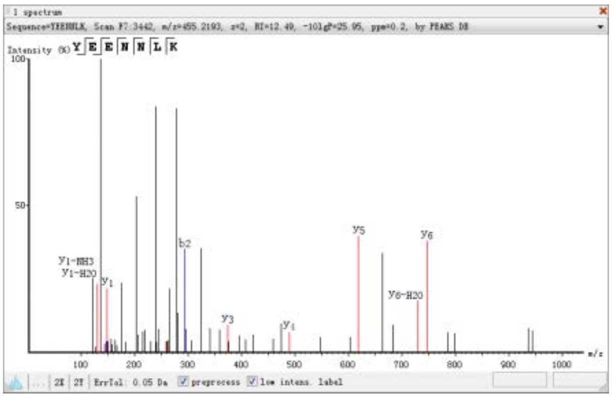

gi|225449198

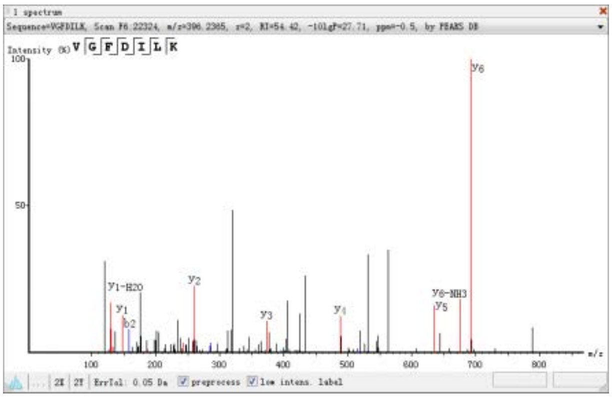

gi|566204168

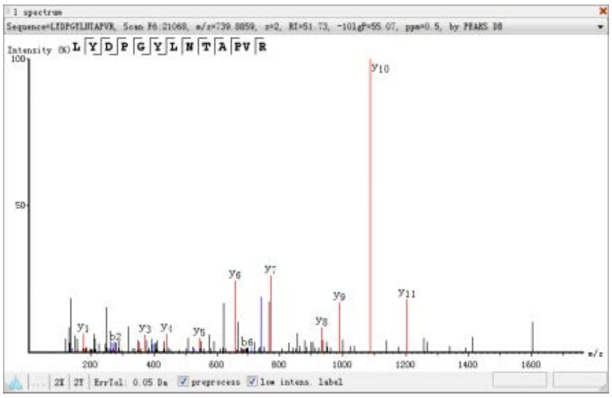

gi|224086130

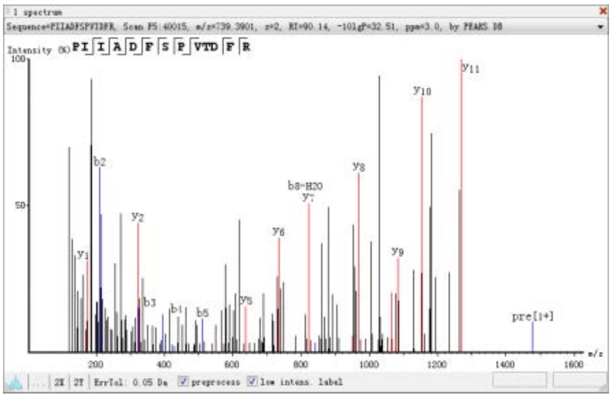

gi|731383113

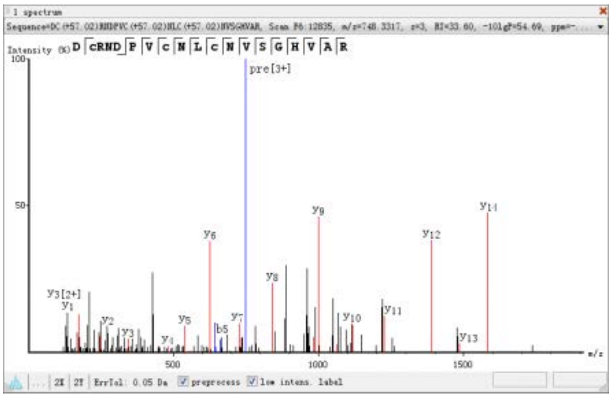

gi|731438722

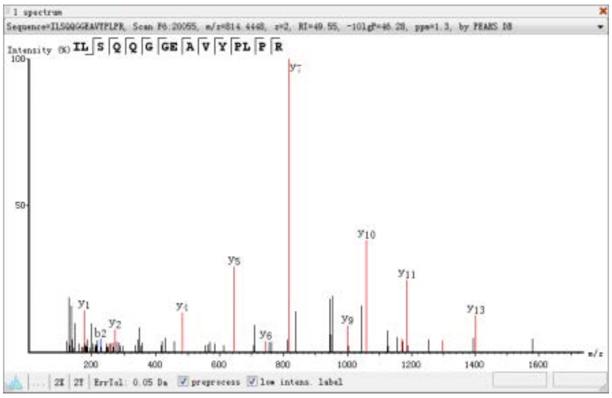

gi|224075888

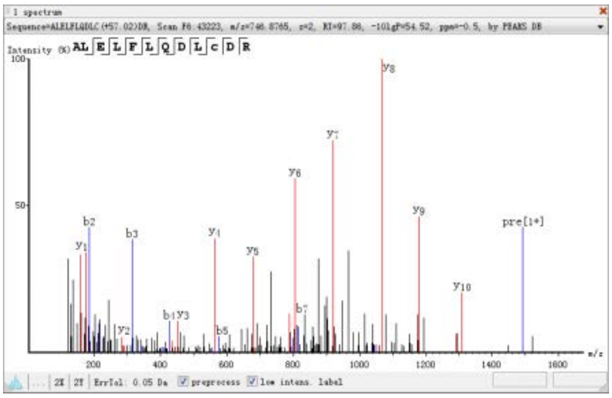

gi|731400520

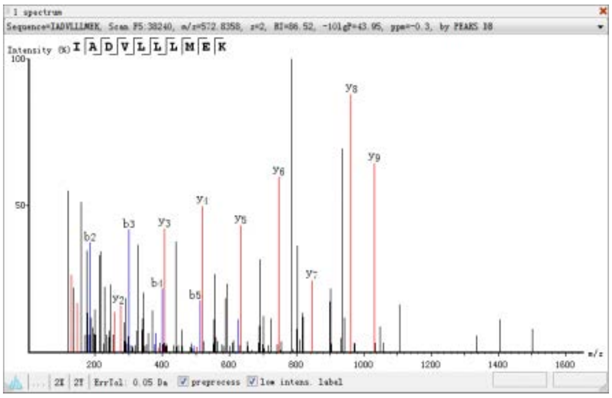

gi|359489456

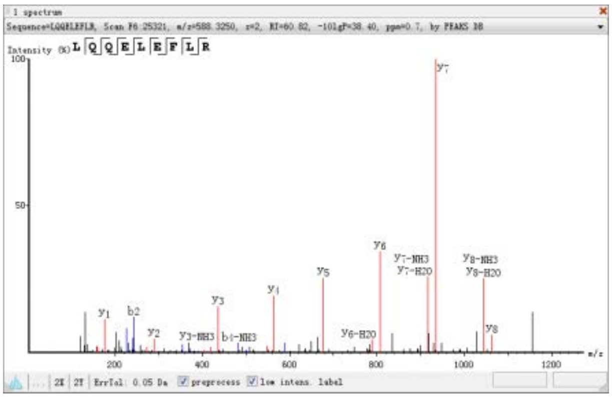

gi|359476827

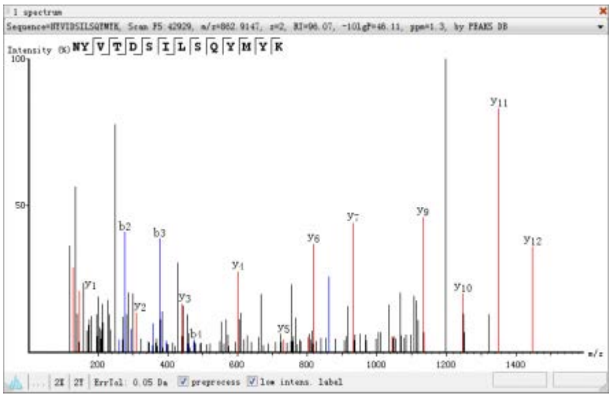

gi|566159086

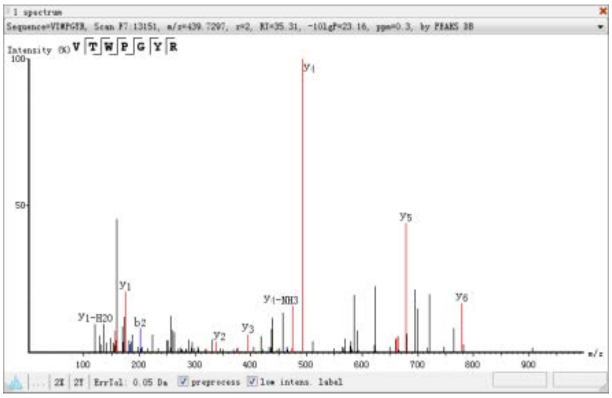

gi|566153022

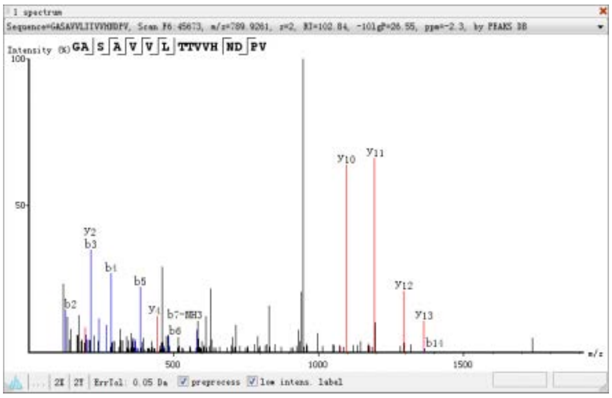

gi|225435594

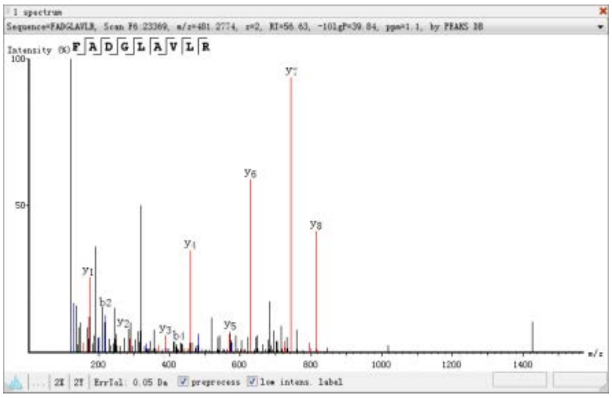

gi|731378986

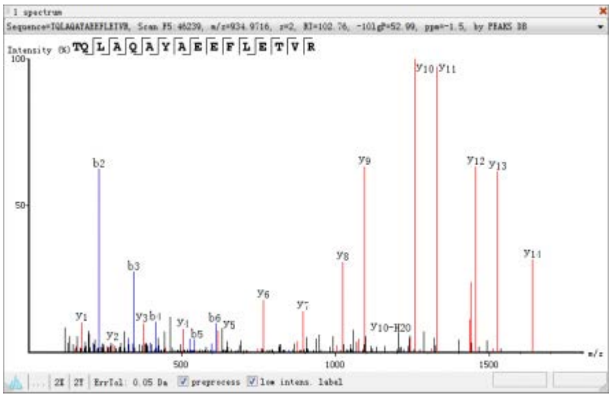

gi|566160921

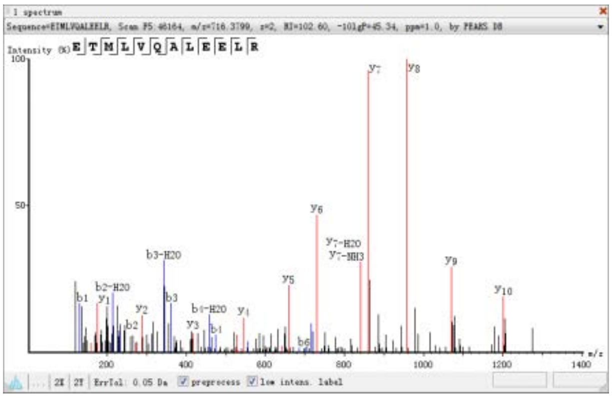

gi|731397329

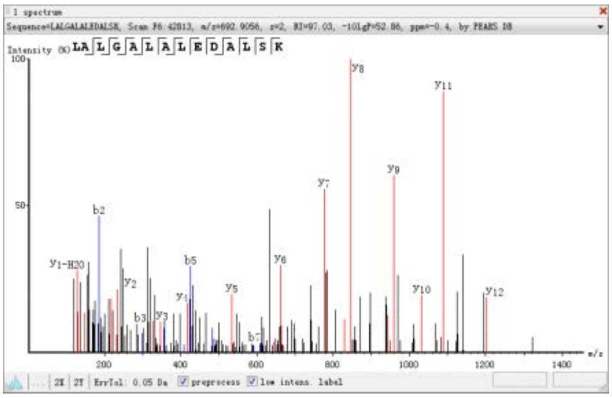

gi|224117526

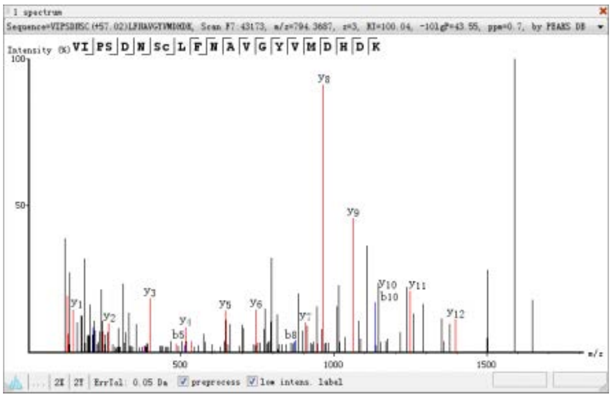

gi|225434197

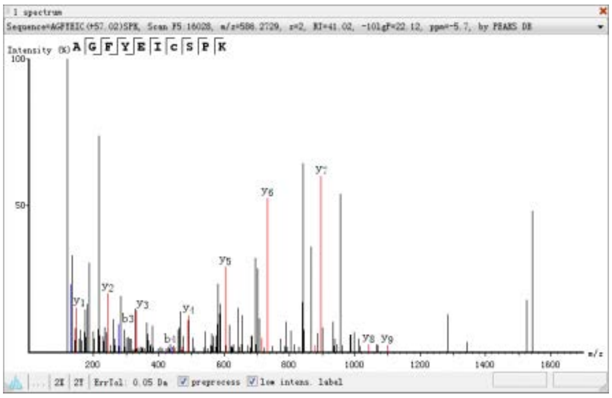

gi|731403895

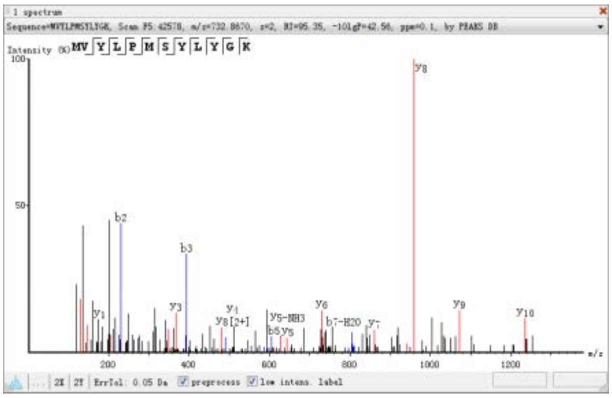

gi|566168456

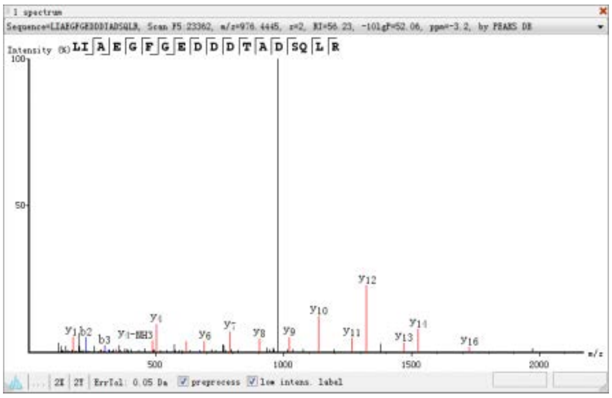

gi|225434156

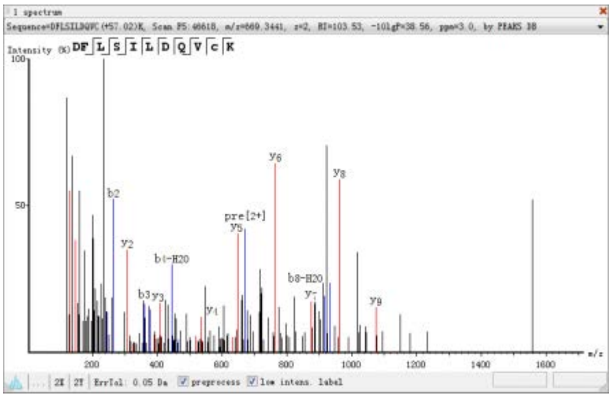

gi|359488959

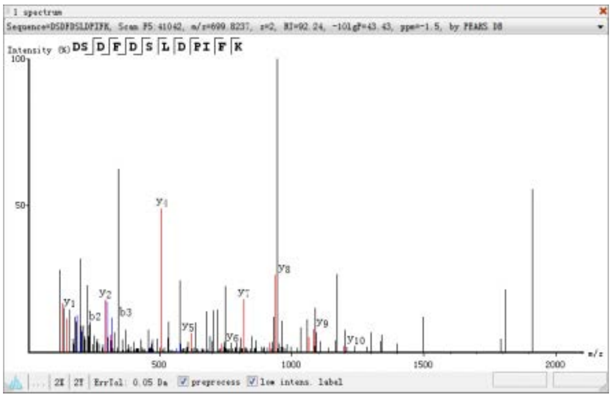

gi|731393417

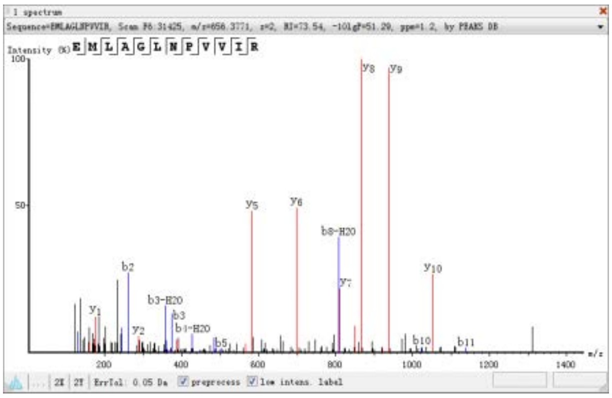

gi|224104085

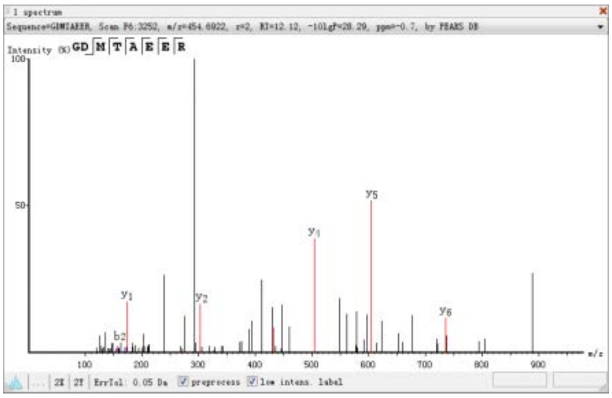

gi|566206225

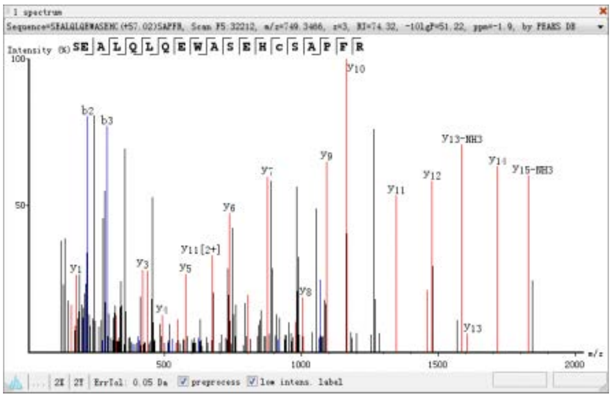

gi|224108744

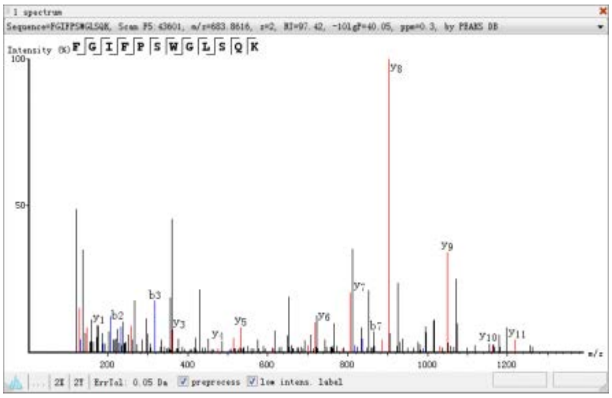

gi|225437581

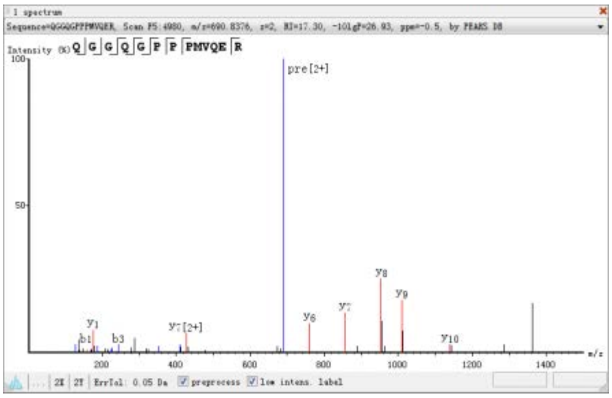

gi|224062053

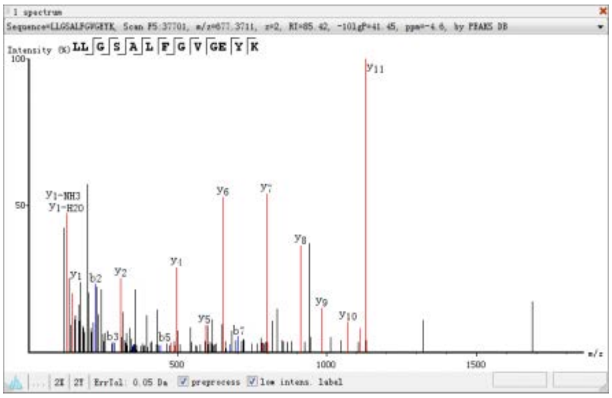

gi|225430537

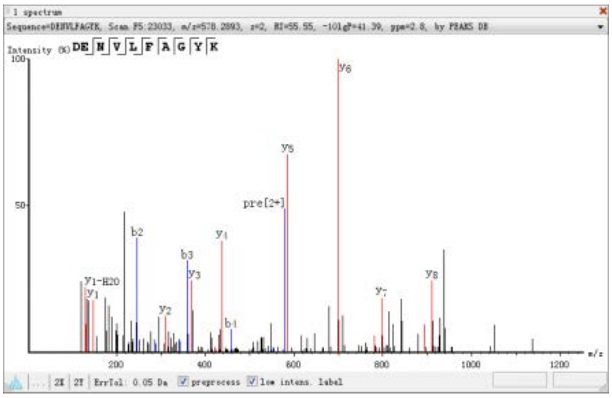

gi|224117704

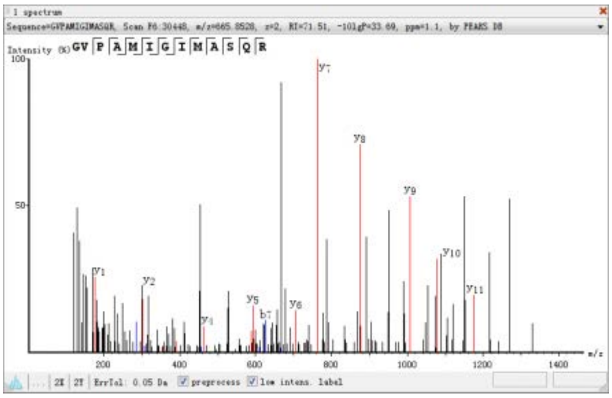

gi|225435776

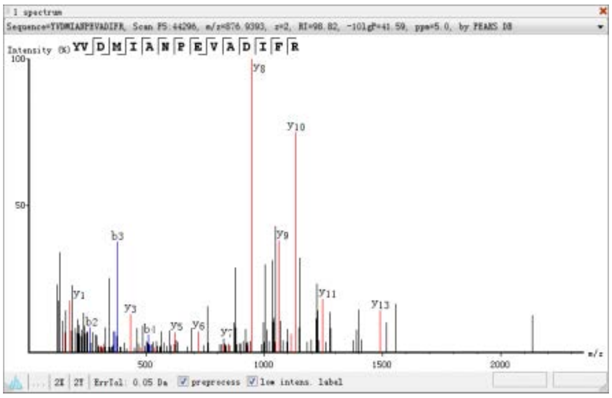

gi|224118638

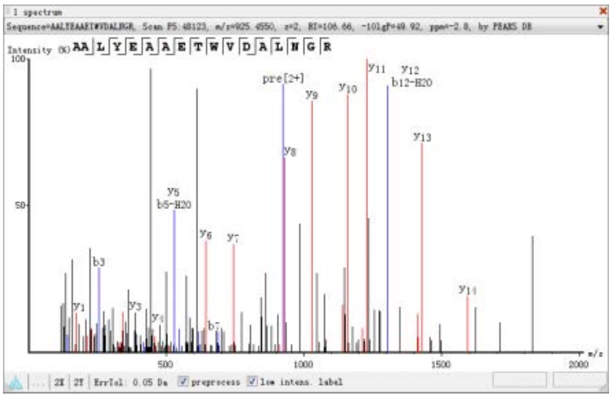

gi|225436003

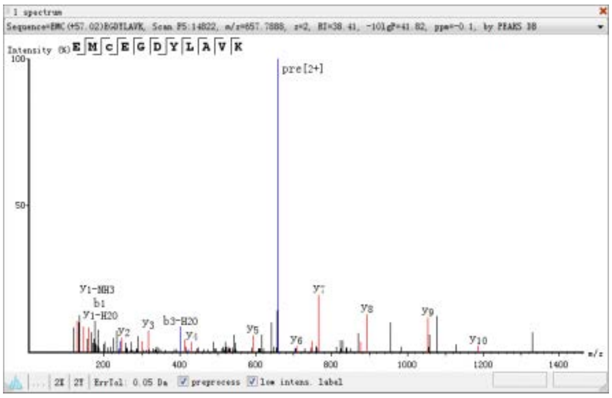

gi|224099925

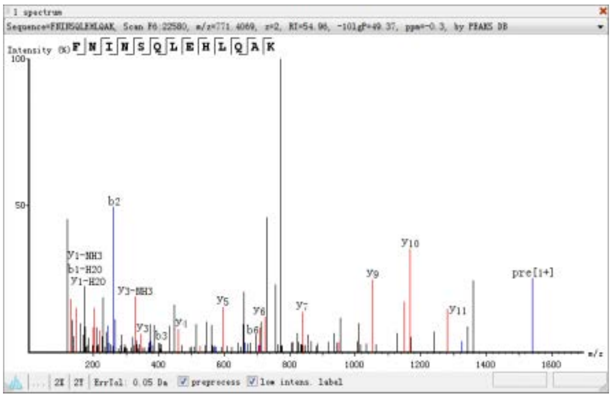

gi|731394805

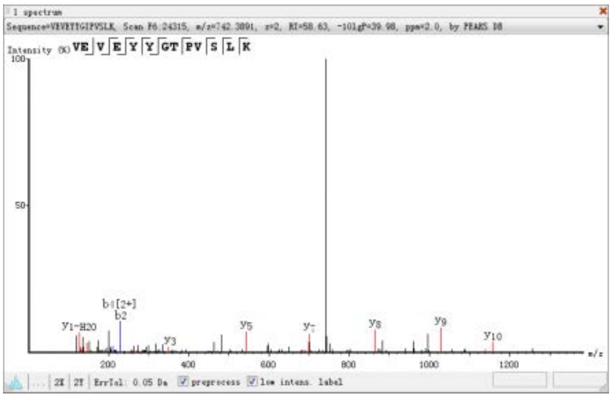

gi|566151879

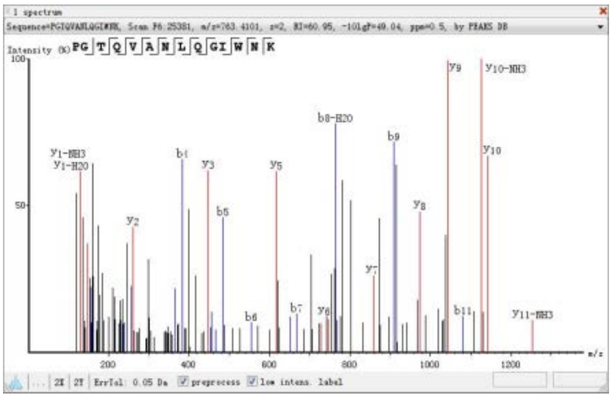

gi|731392112

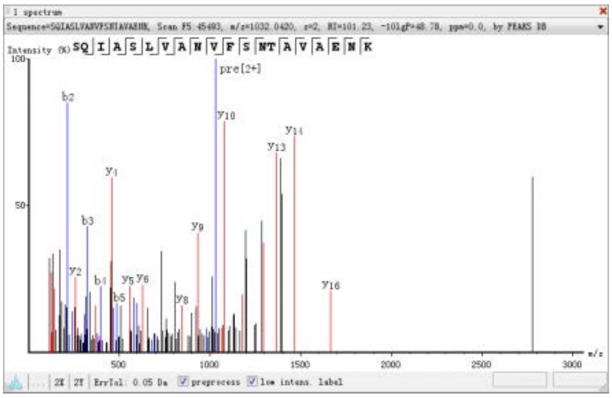

gi|224055587

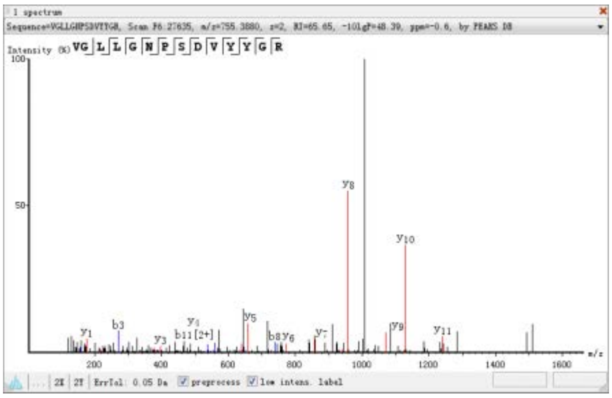

gi|566182607

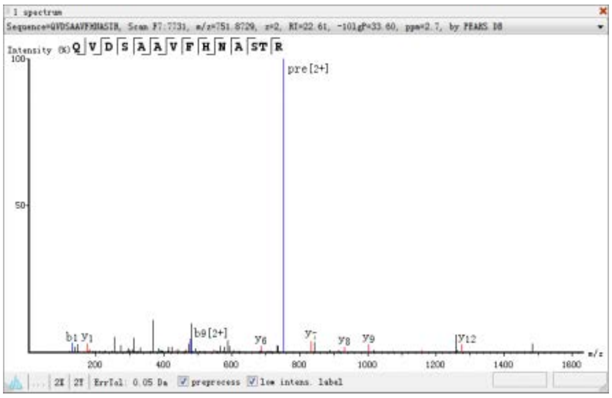

gi|566150079

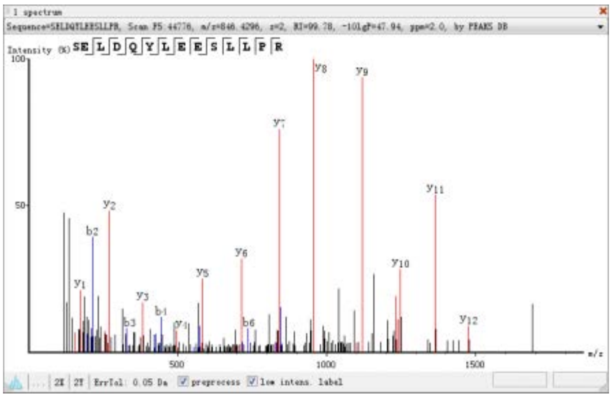

gi|225424148

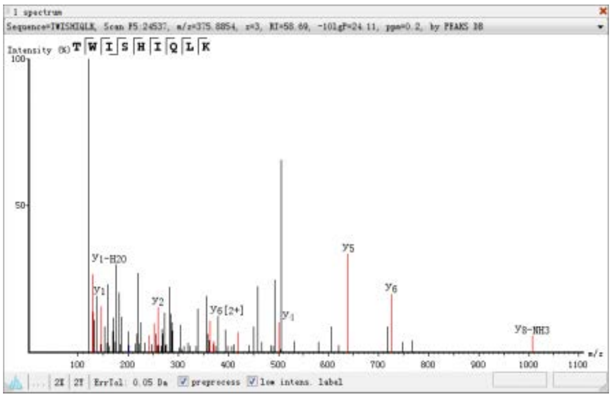

gi|225452863

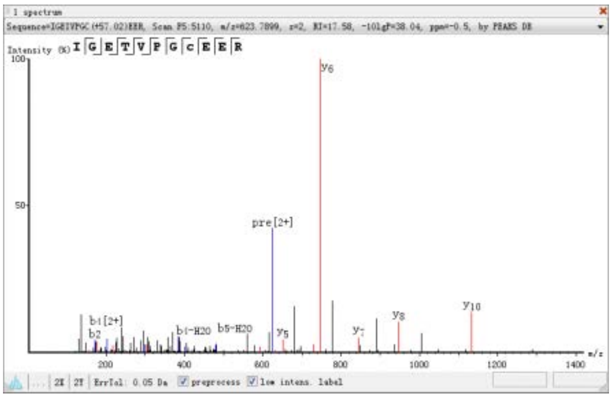

gi|566155559

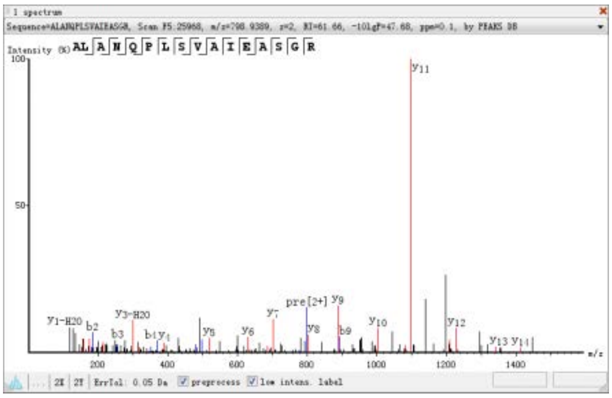

gi|224077096

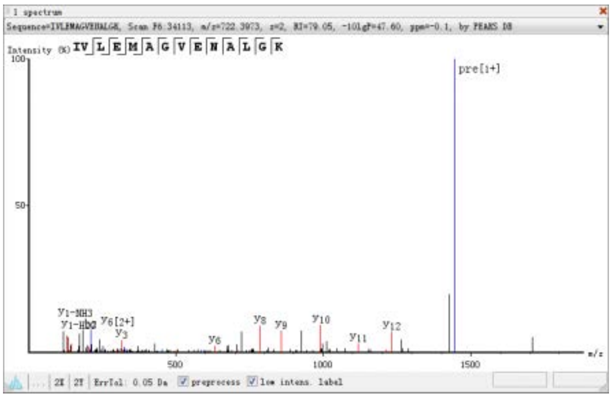

gi|224146385

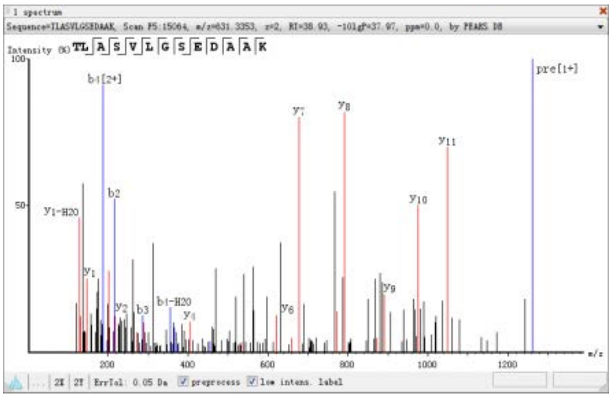

gi|731422518

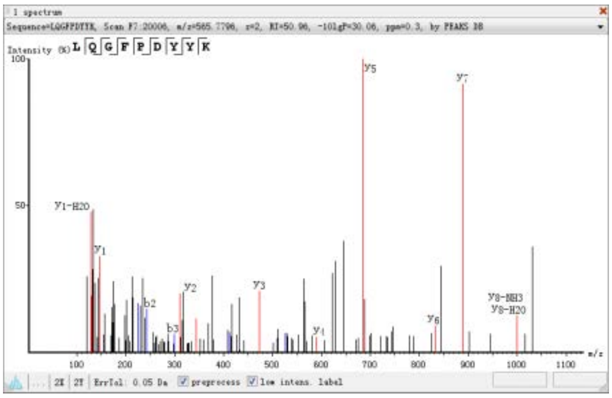

gi|224094580

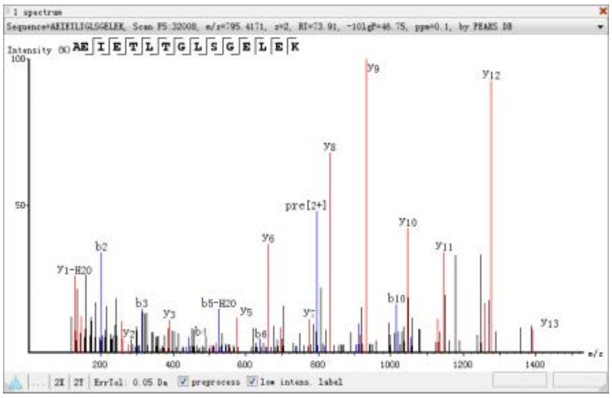

gi|225458227

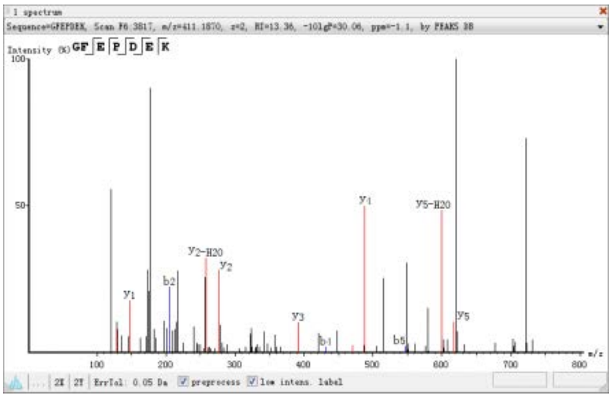

gi|566206658

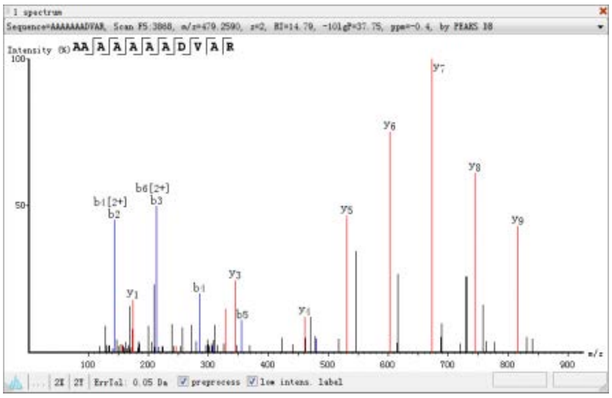

gi|224098848

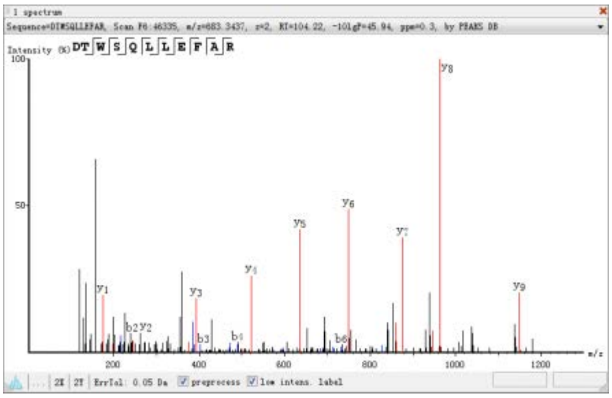

gi|566210253

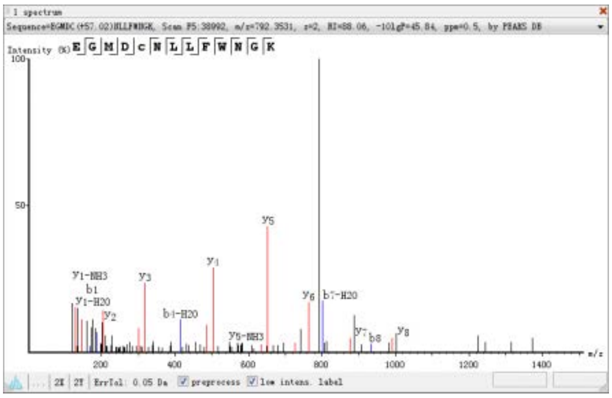

gi|566193348

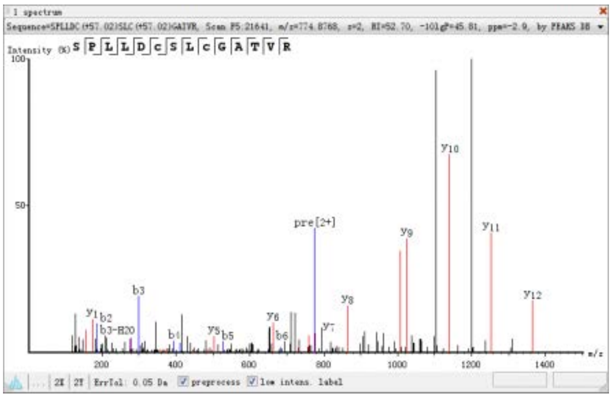

gi|225437358

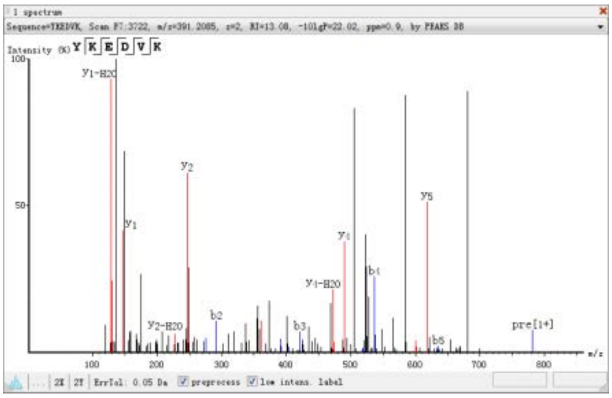

gi|224118700

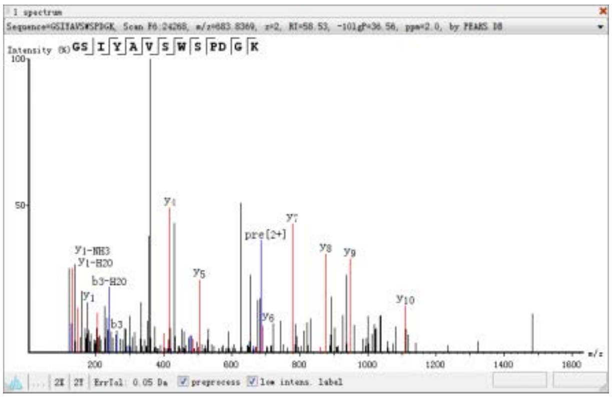

gi|224079549

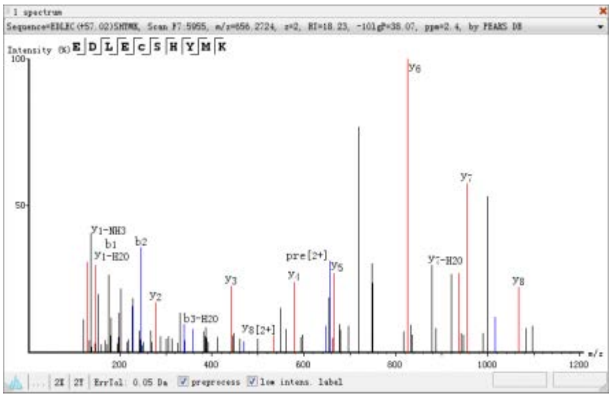

gi|225456794

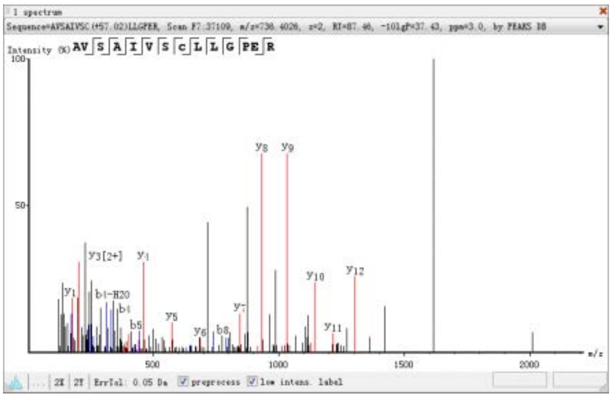

gi|225444537

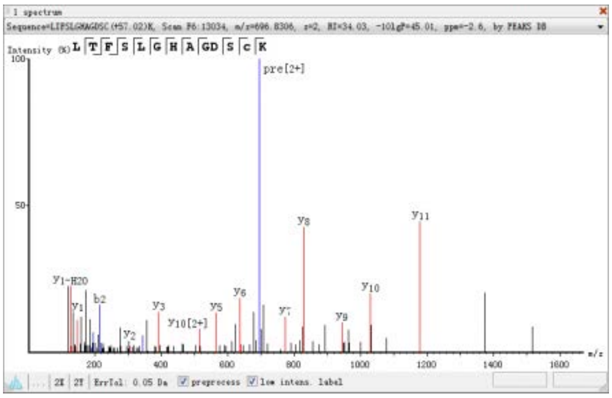

gi|224064766

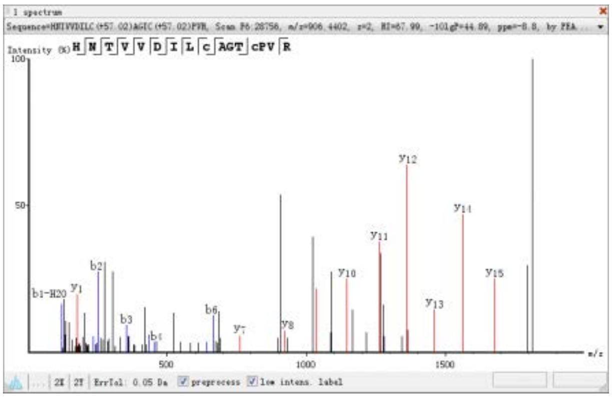

gi|566179360

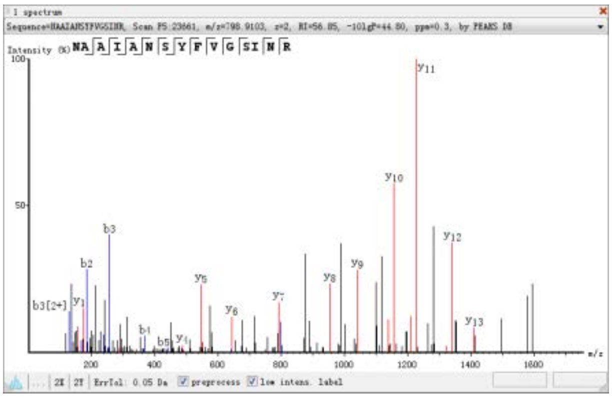

gi|566189493

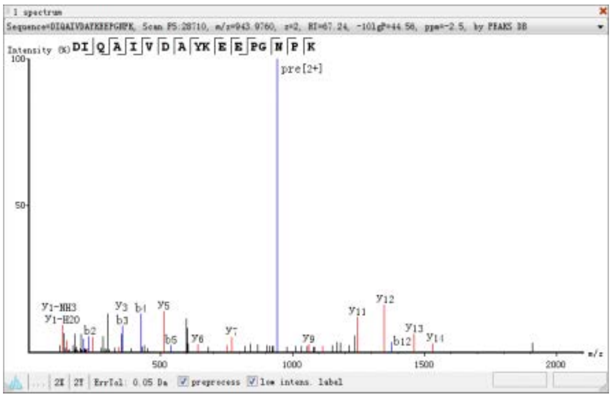

gi|359490488

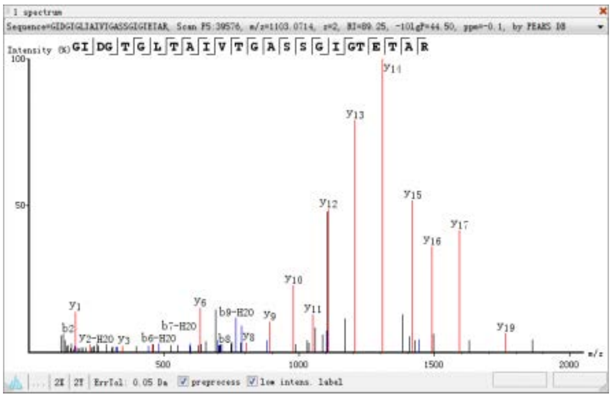

gi|566183391

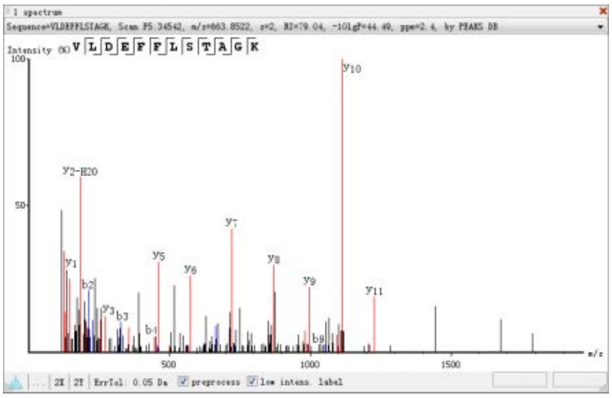

gi|225429824

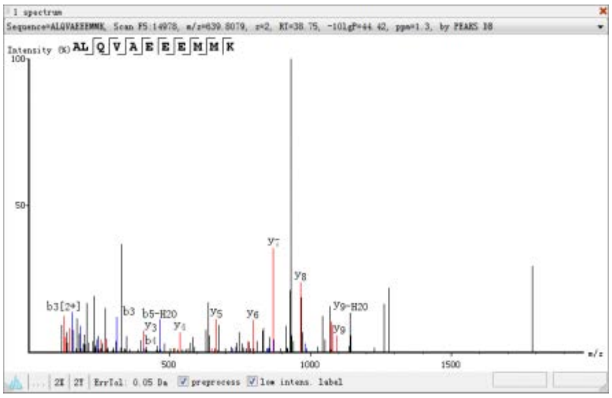

gi|359487063

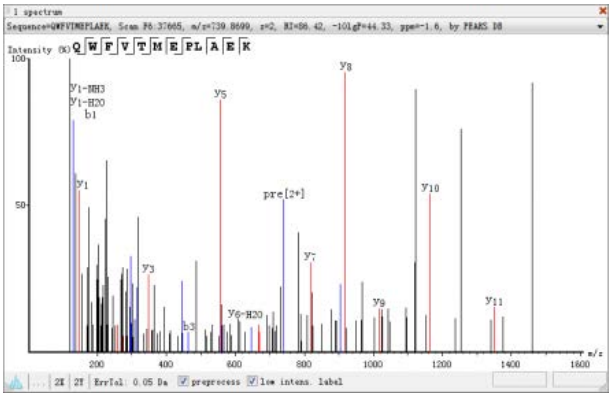

gi|224103873

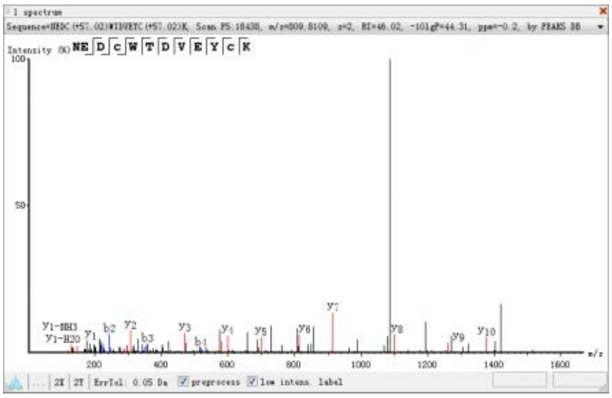

gi|225443254

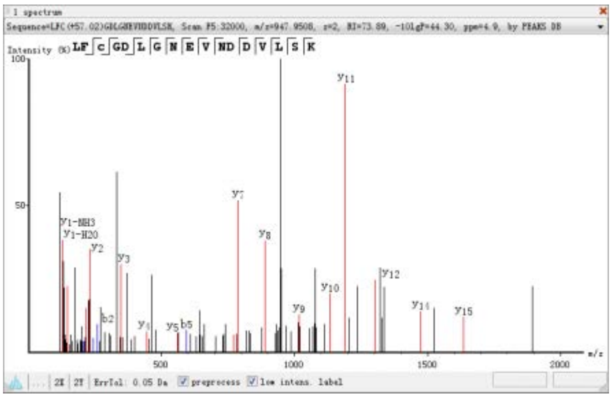

gi|731386977

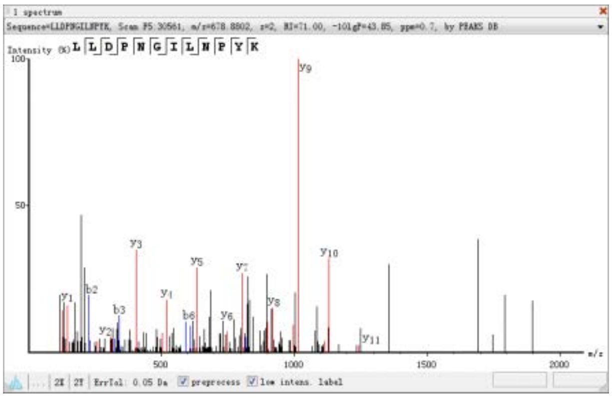

gi|731389767

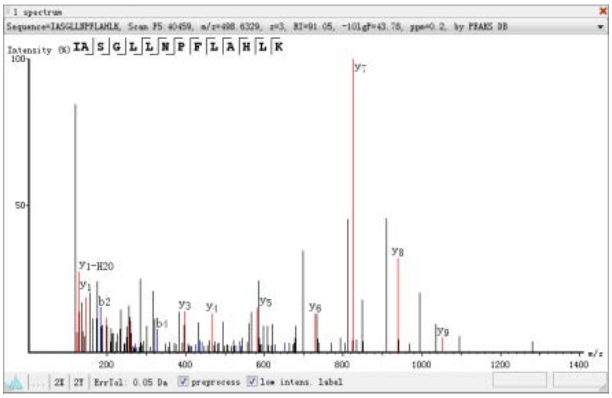

gi|566161892

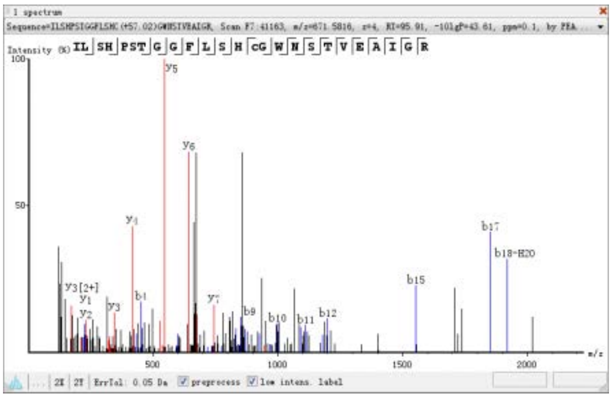

gi|566173634

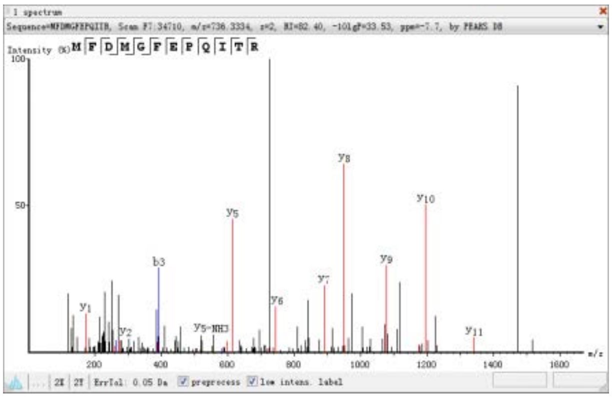

gi|566213840

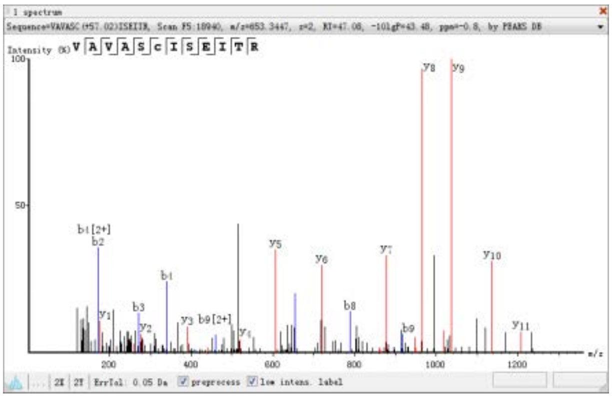

gi|224085976

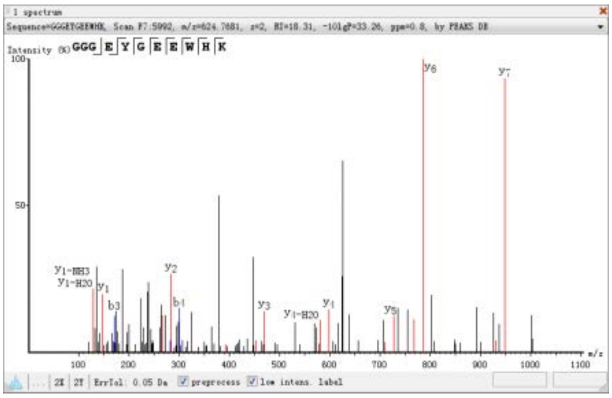

gi|224100879

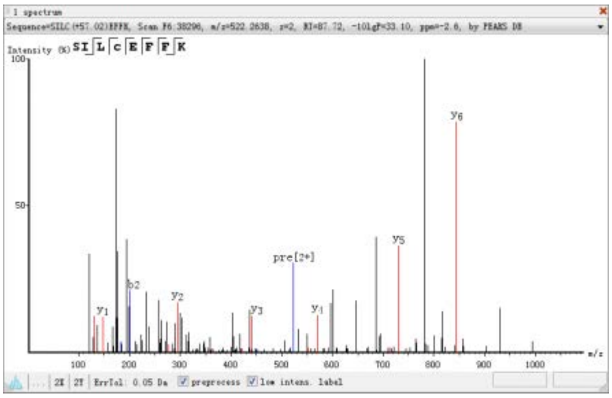

gi|566157213

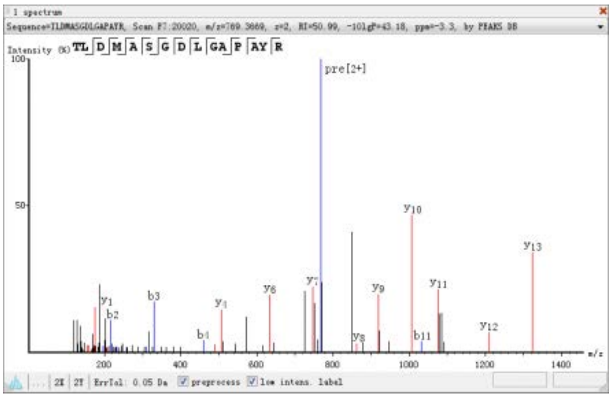

gi|566154461

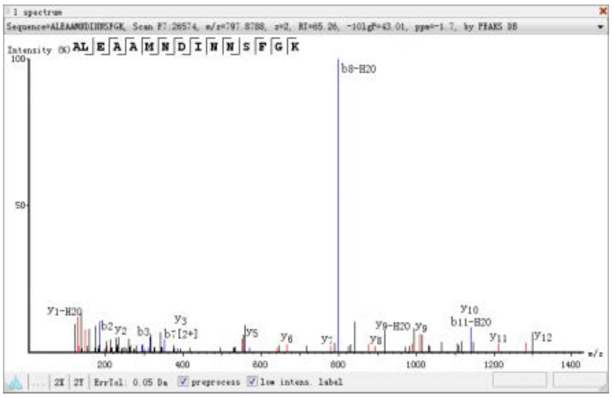

gi|224109026

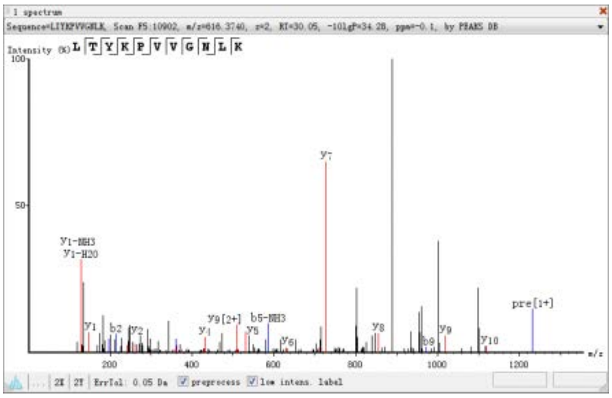

gi|566152935

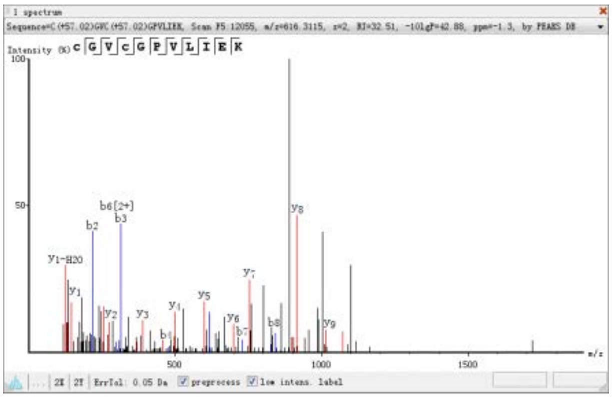

gi|225458607

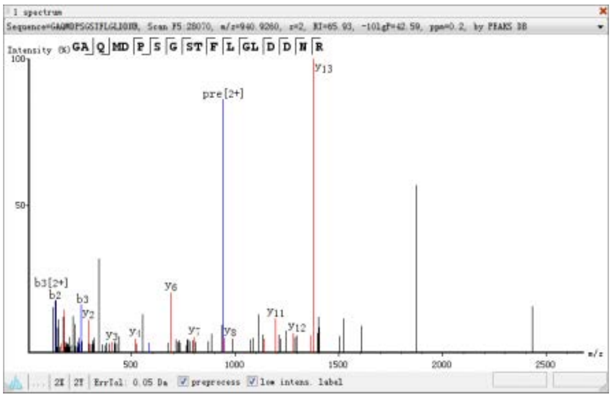

gi|224078604

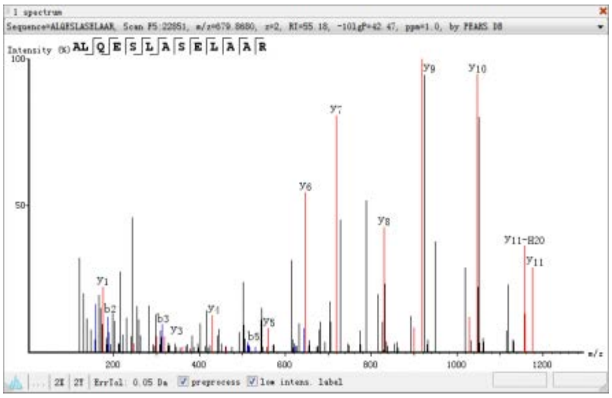

gi|566147174

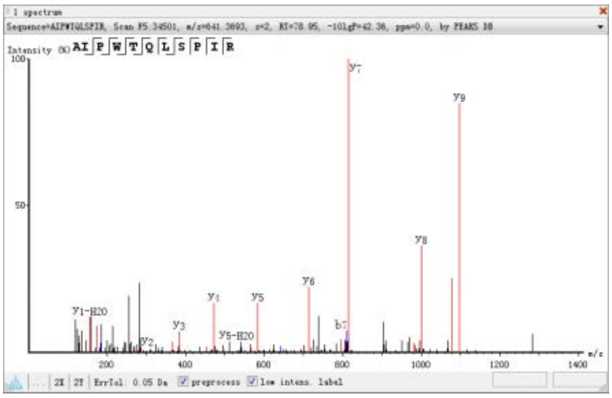

gi|224145402

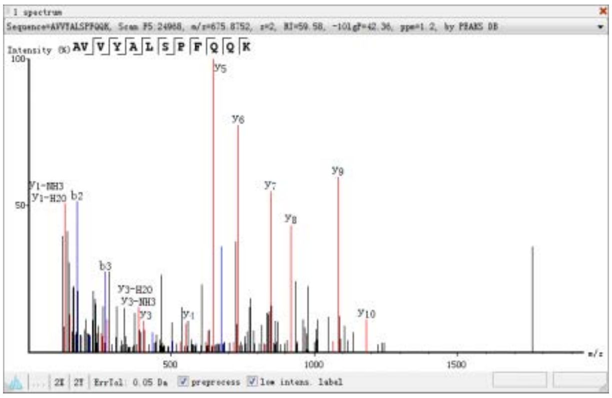

gi|225459538

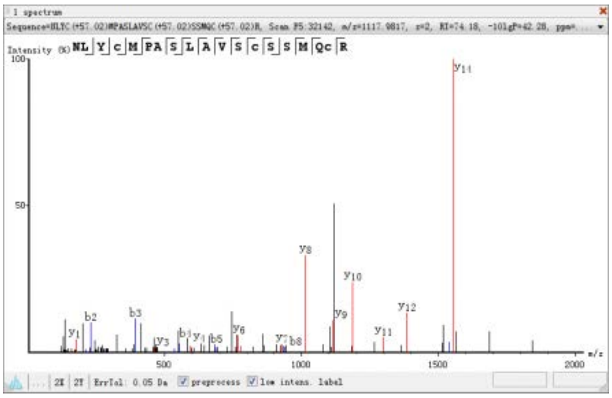

gi|731399255

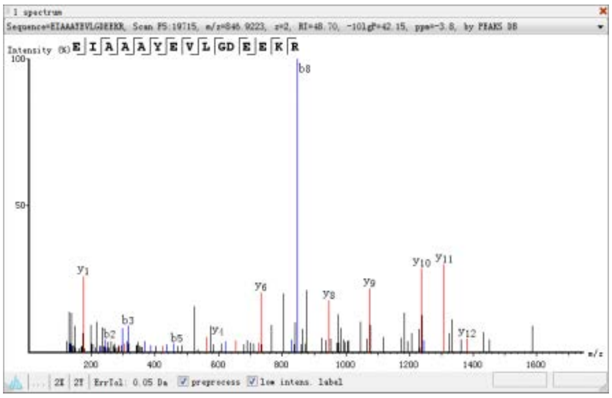

gi|731433331

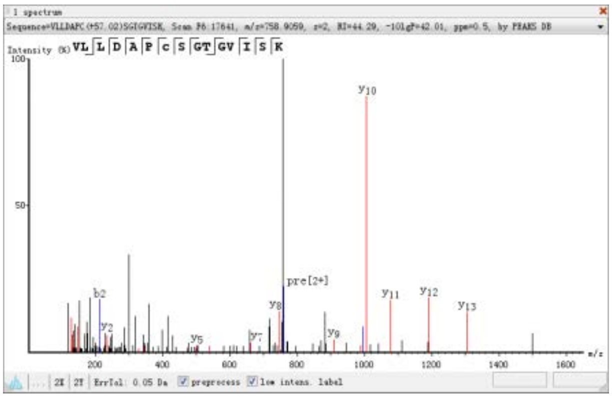

gi|731417029

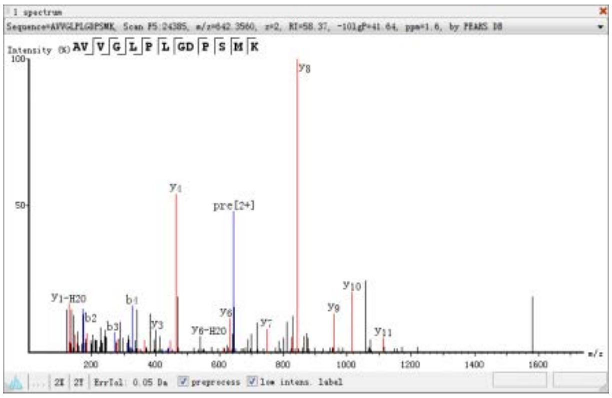

gi|224120424

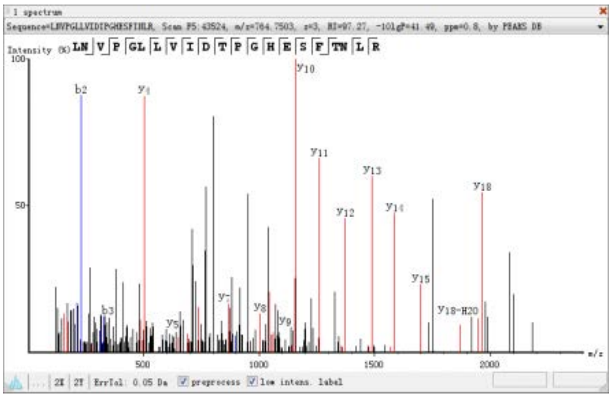

gi|731420110

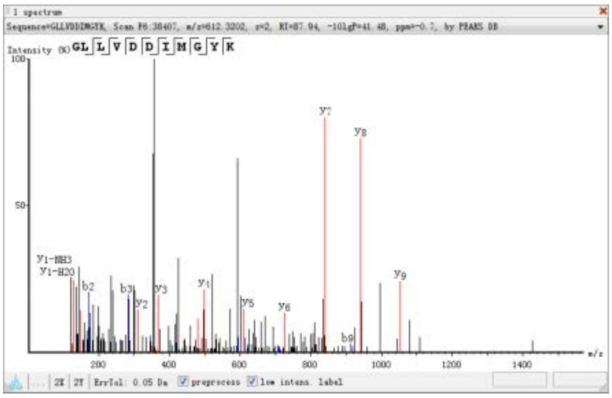

gi|225450051

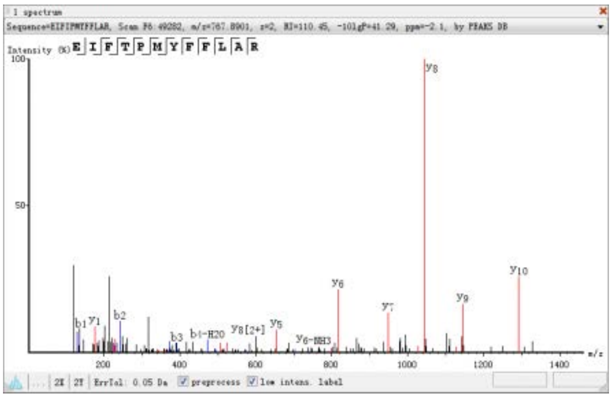

gi|566192632

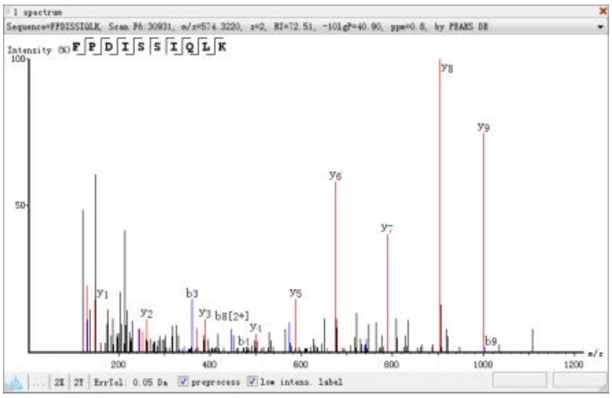

gi|731427762

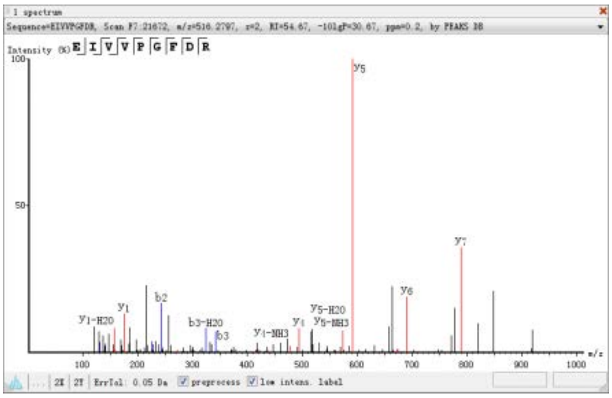

gi|224115158

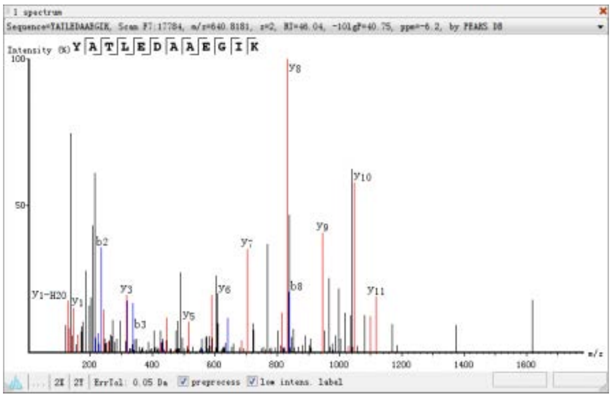

gi|566214148

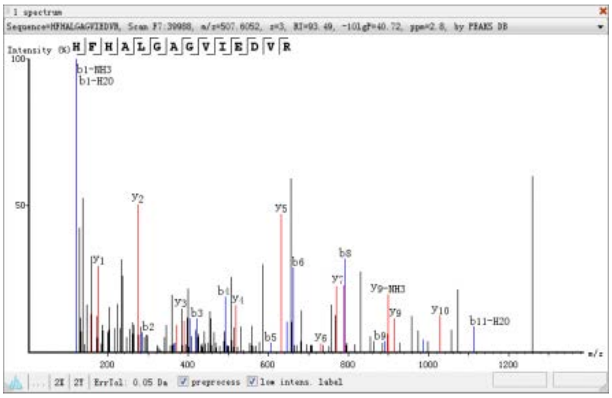

gi|225436041

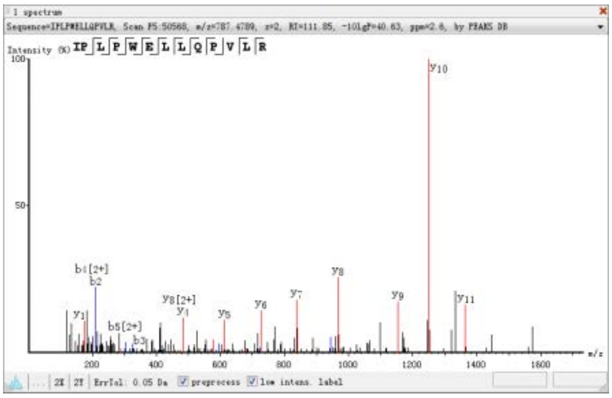

gi|731425417

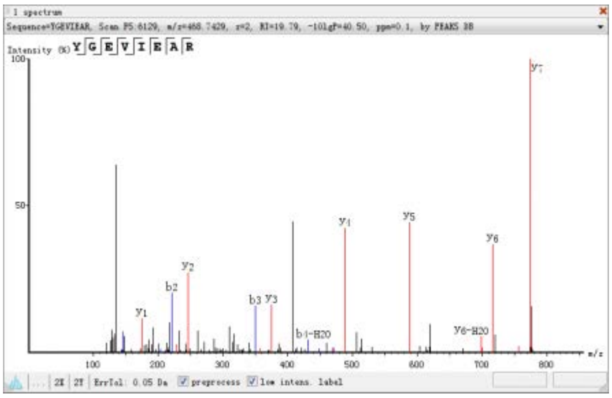

gi|731386510

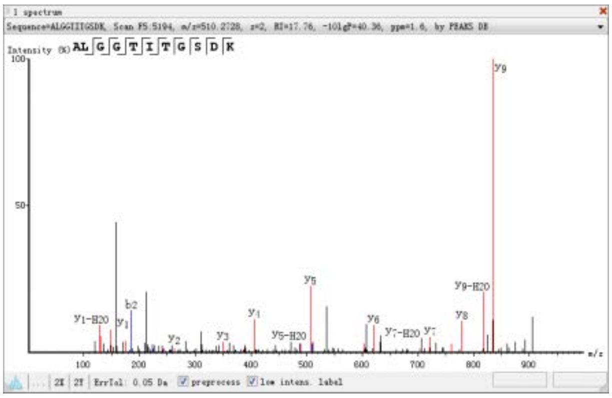

gi|566215395

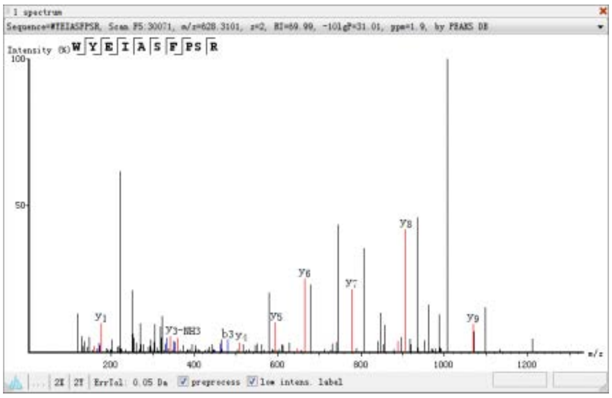

gi|566155359

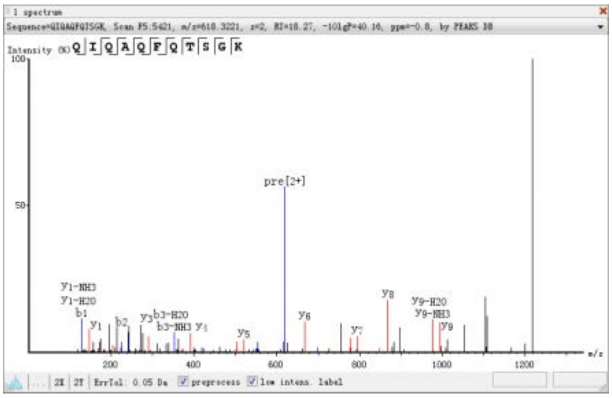

gi|731428902

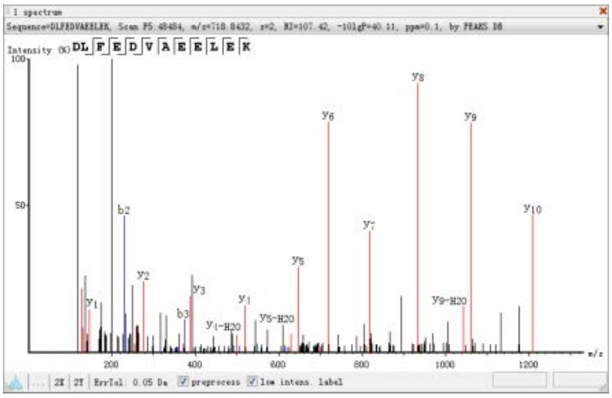

gi|225456479

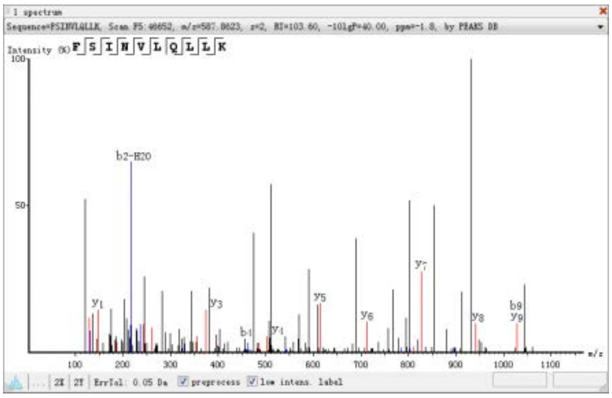

gi|731438931

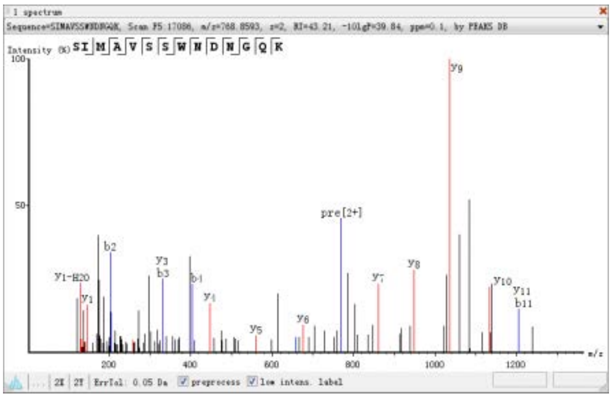

gi|225457574

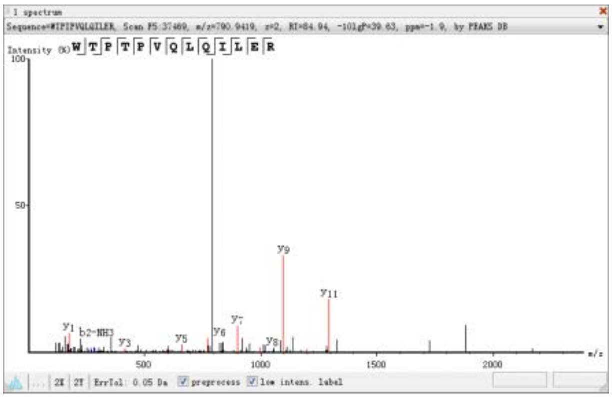

gi|225446724

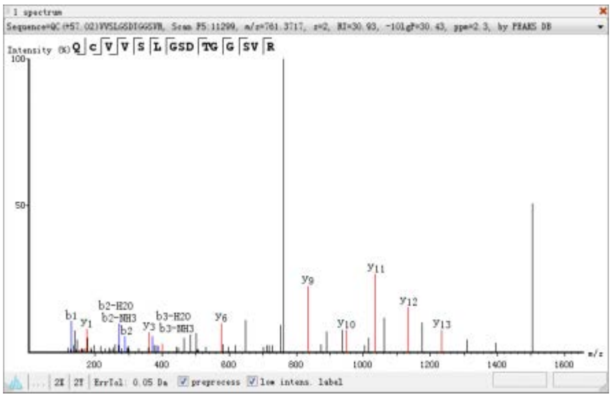

gi|566202901

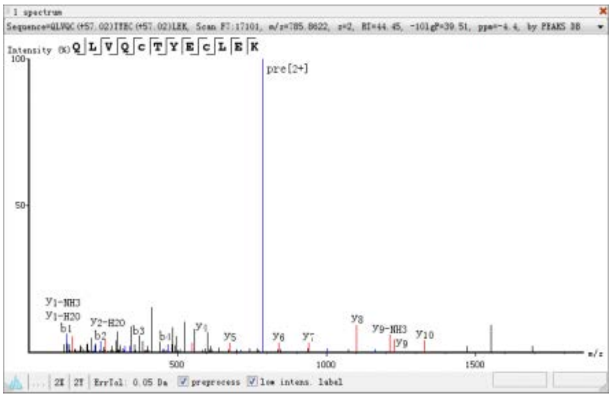

gi|731430482

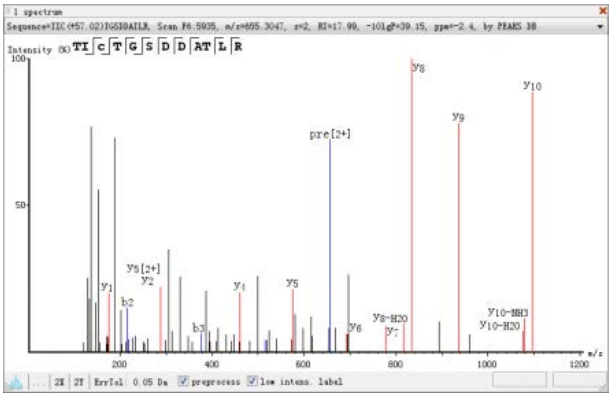

gi|224137970

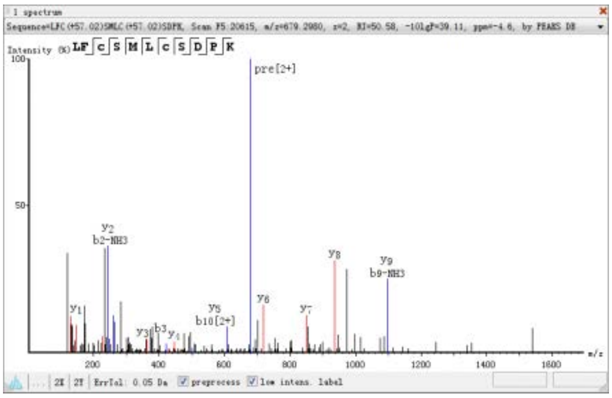

gi|225459083

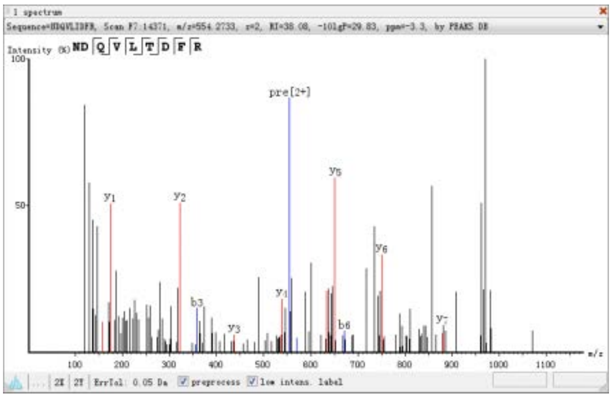

gi|225426544

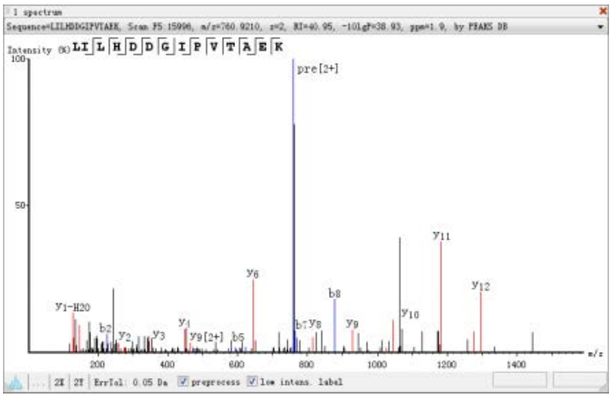

gi|224066789

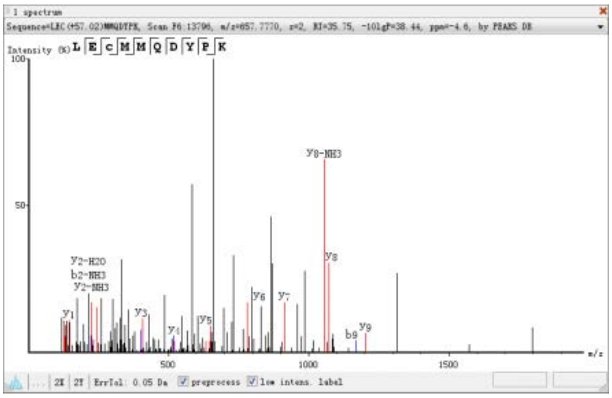

gi|566174766

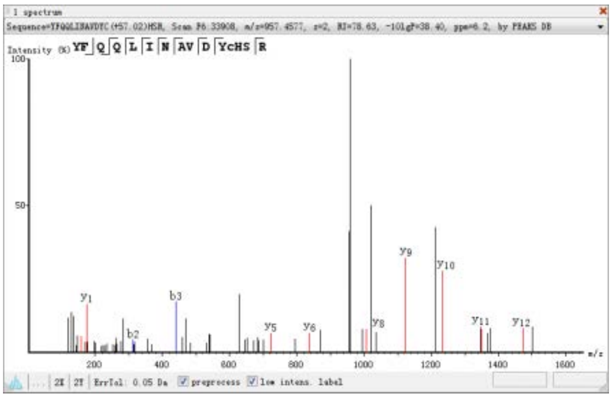

gi|224126789

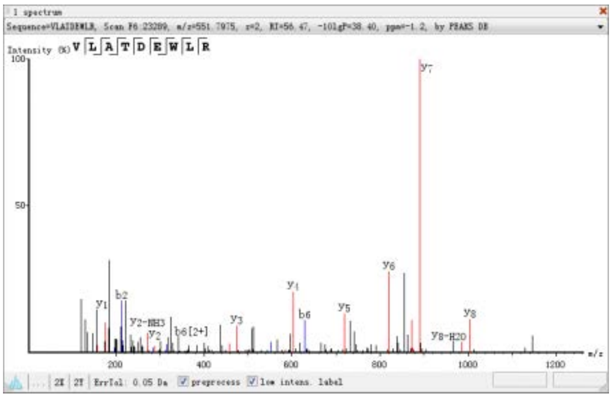

gi|225428560

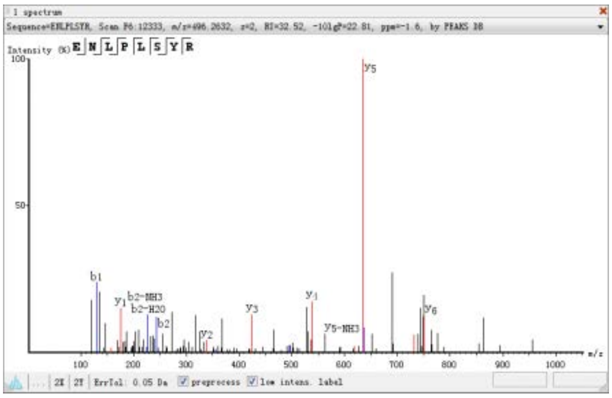

gi|731406152

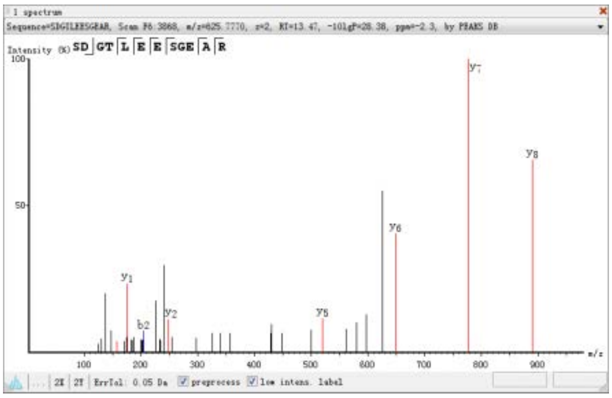

gi|225459530

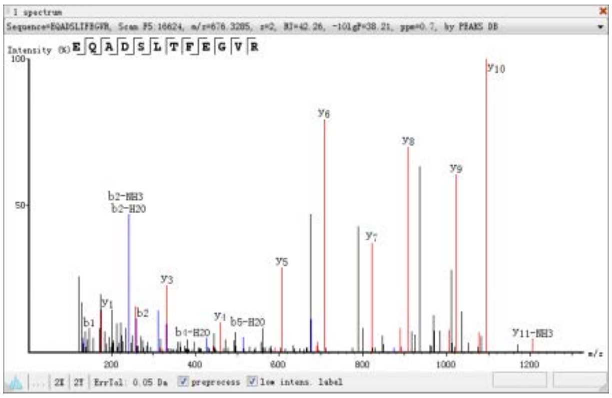

gi|225424272

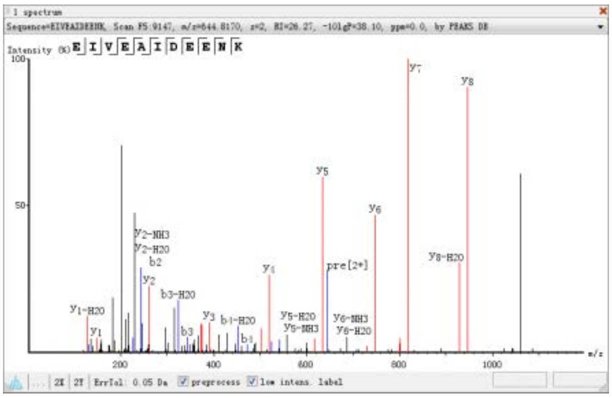

gi|224073306

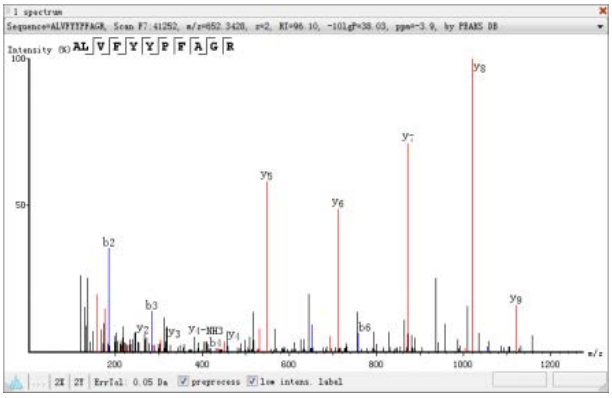

gi|225435429

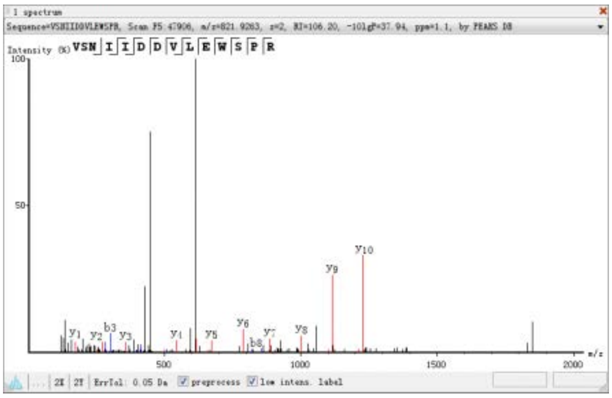

gi|359475077

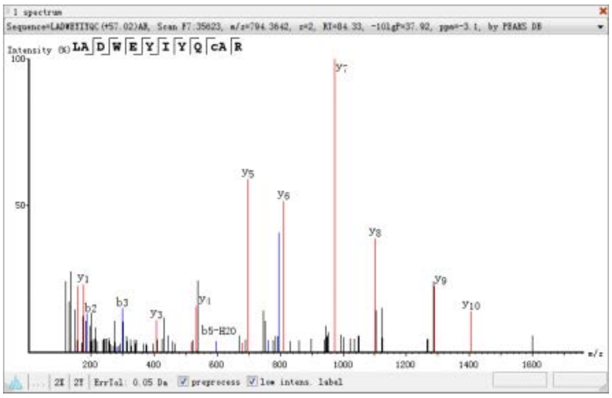

gi|566205509

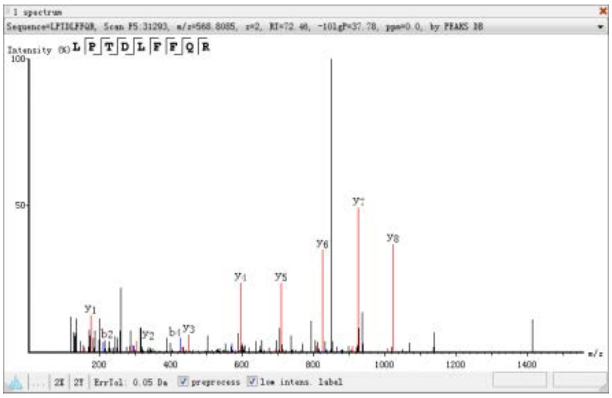

gi|225436347

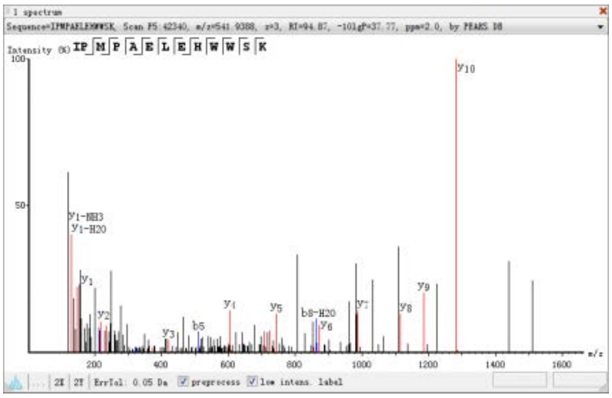

gi|566176657

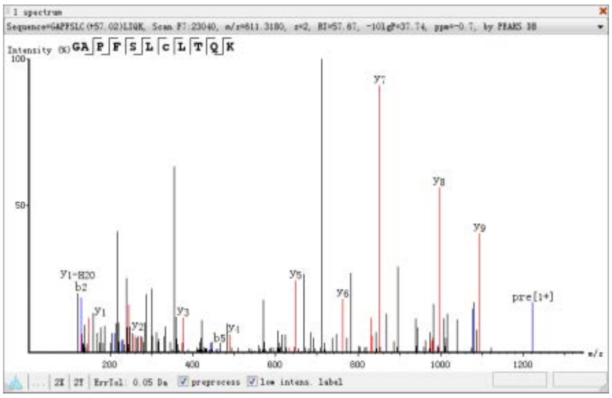

gi|566211614

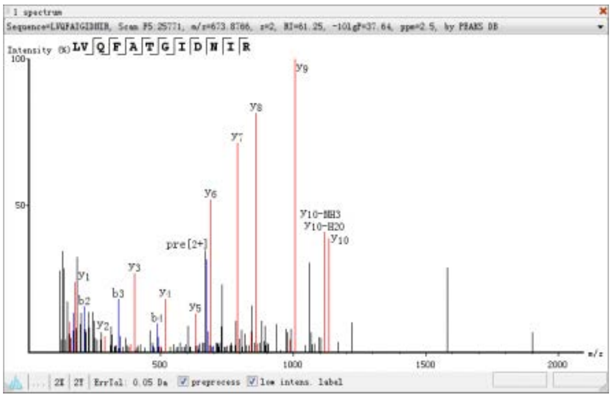

gi|566168047

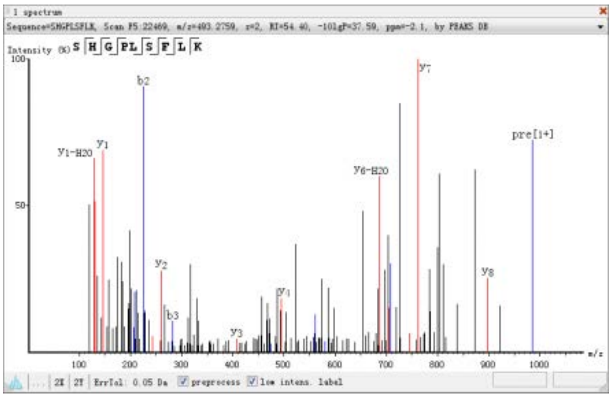

gi|731436255

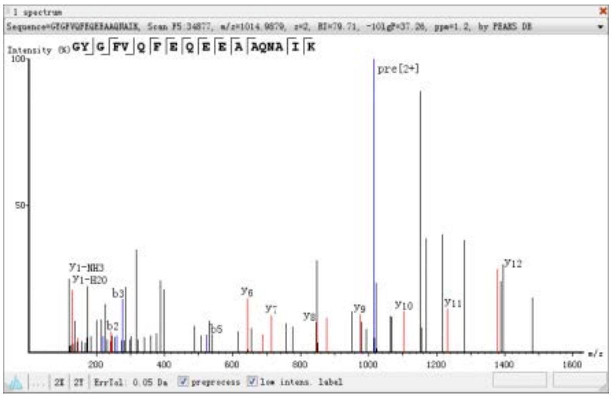

gi|225447488

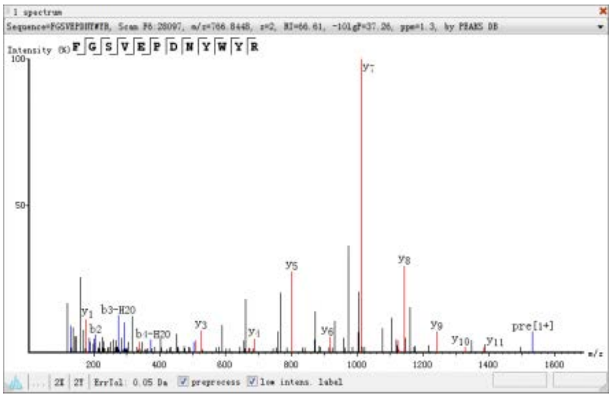

gi|224142329

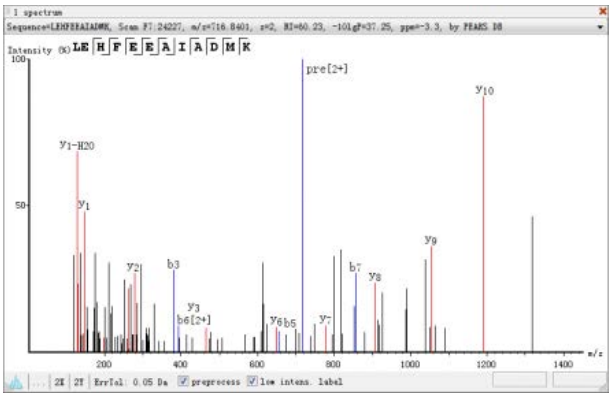

gi|225429195

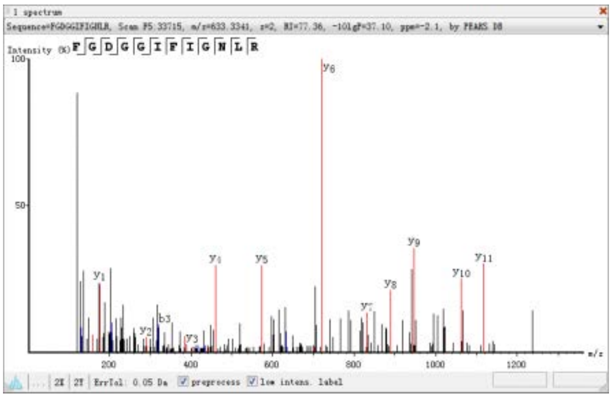

gi|225431489

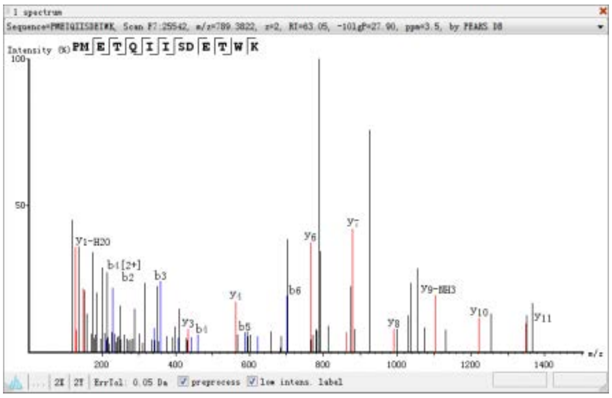

gi|731393379

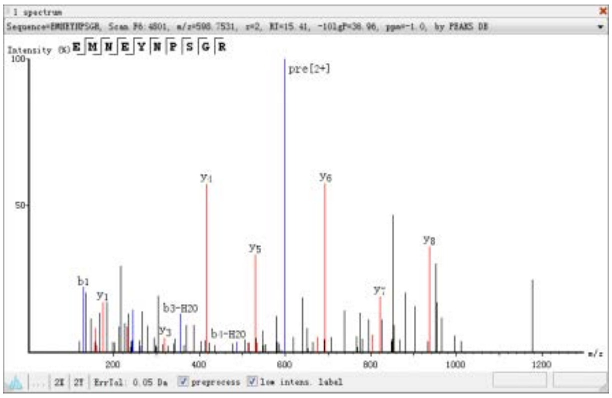

gi|224090669

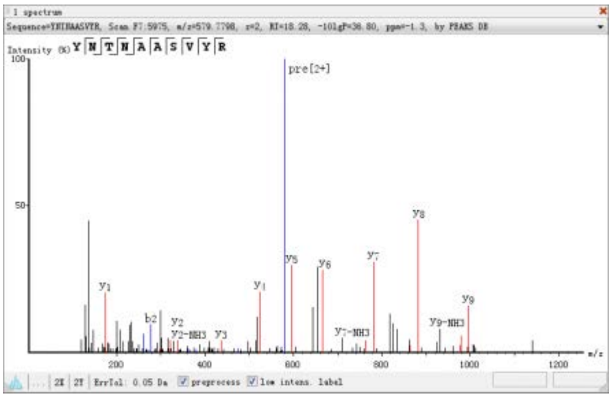

gi|224114830

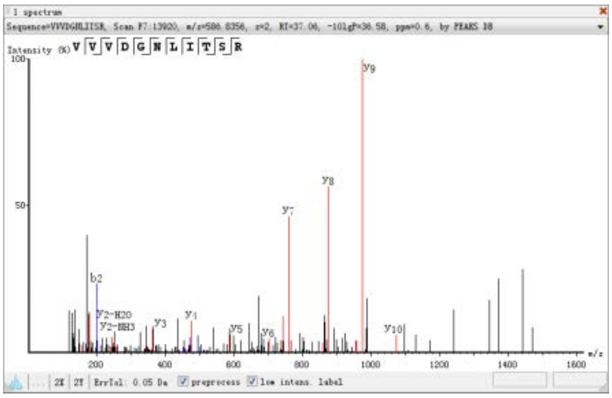

gi|566156450

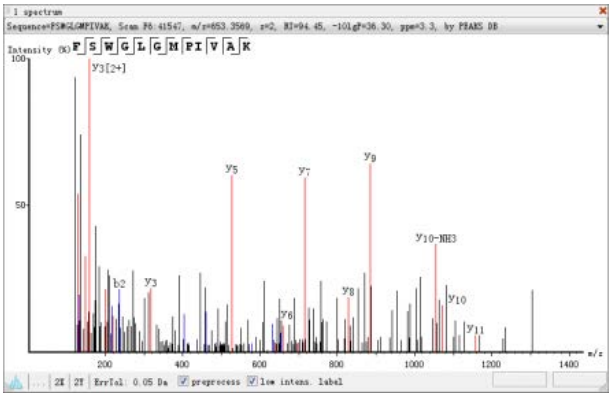

gi|225469438

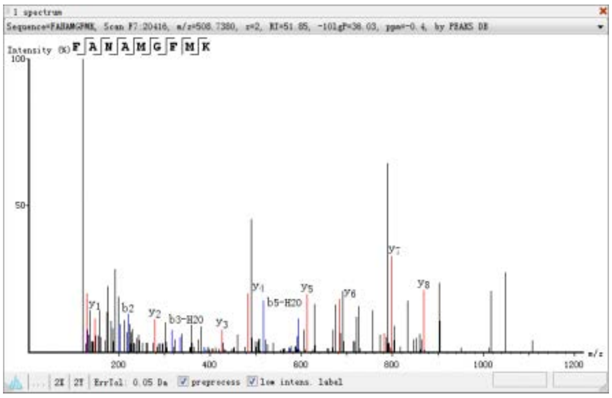

gi|731397495

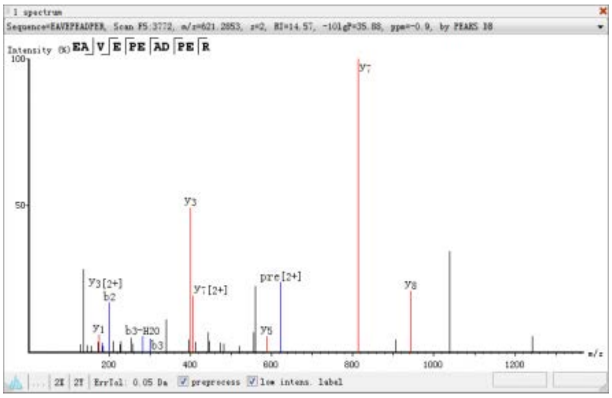

gi|225442841

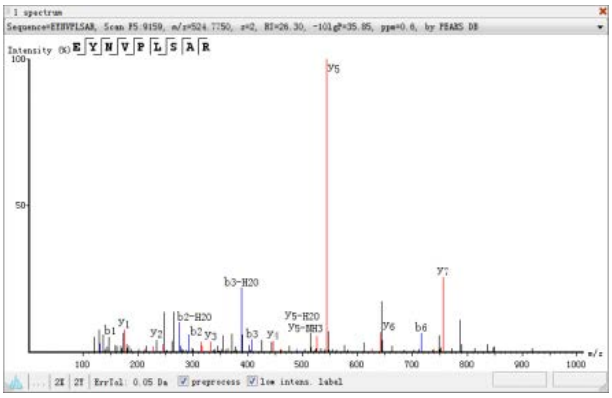

gi|731395888

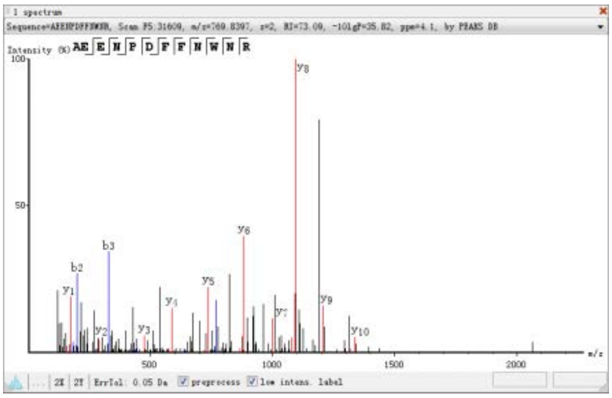

gi|566160273

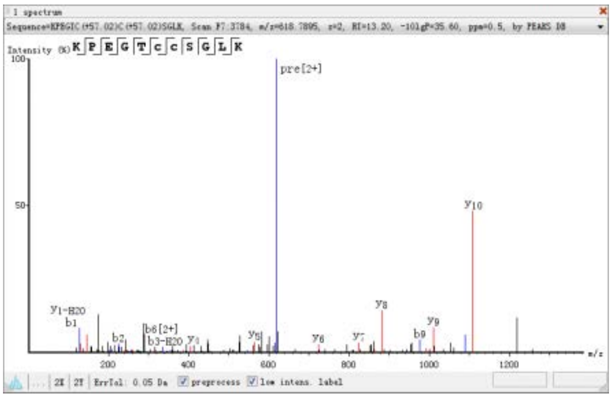

gi|225470348

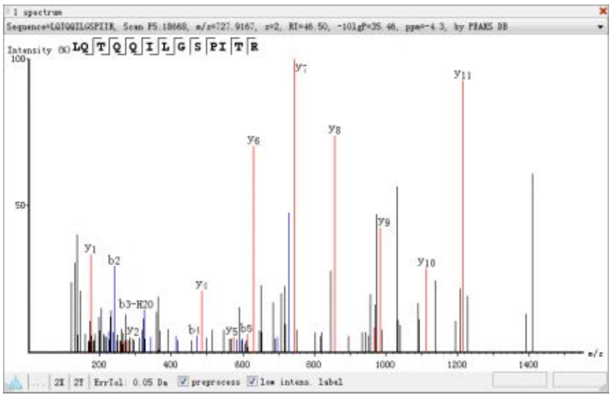

gi|225430161

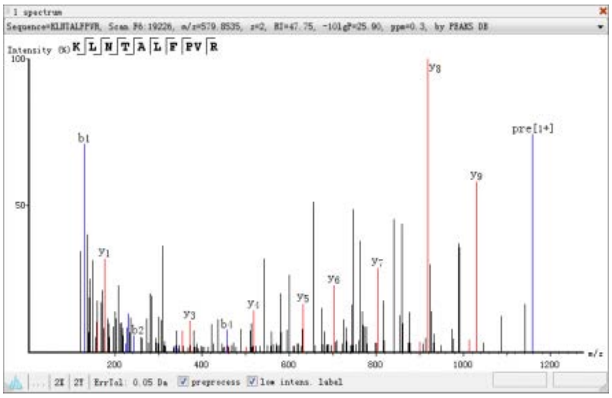

gi|731383801

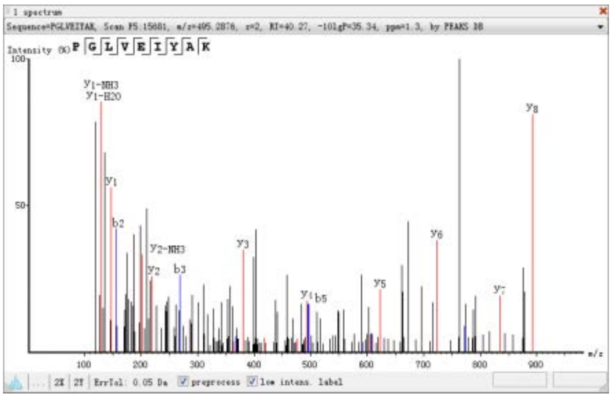

gi|731383208

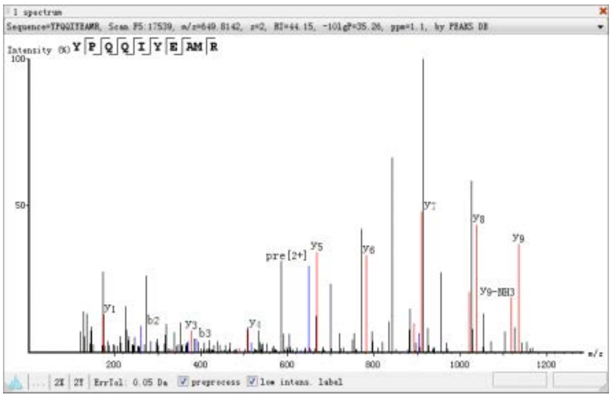

gi|359475014

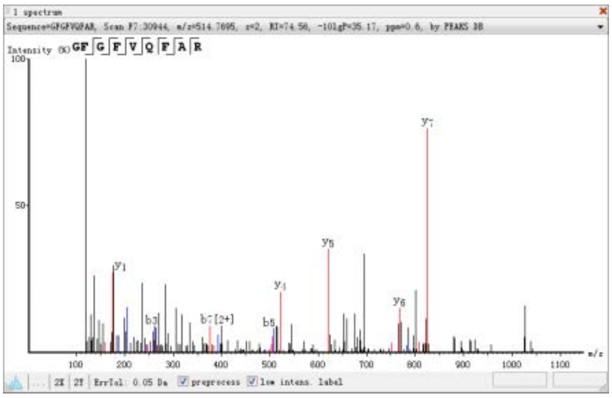

gi|566214618

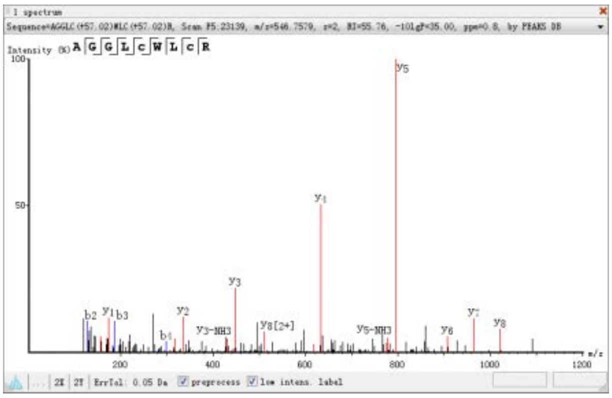

gi|225462062

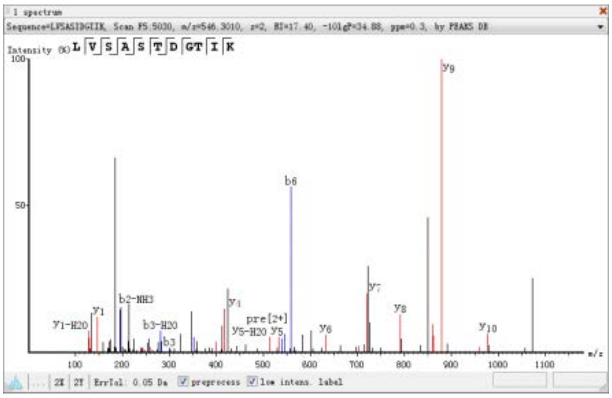

gi|224062736

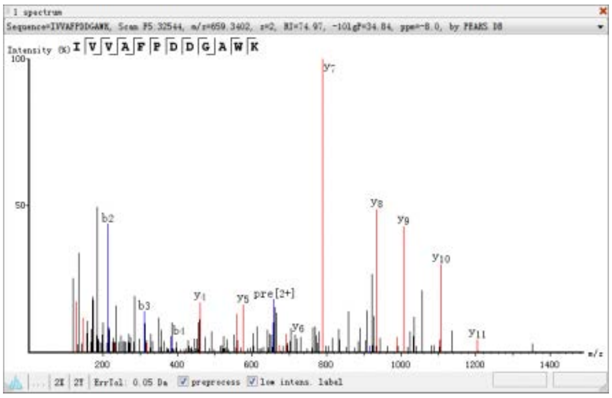

gi|225444159

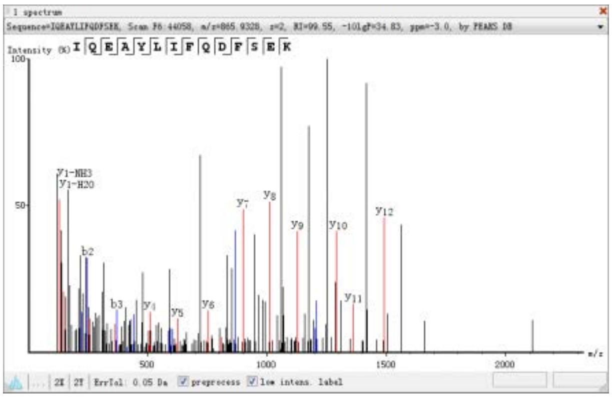

gi|225447221

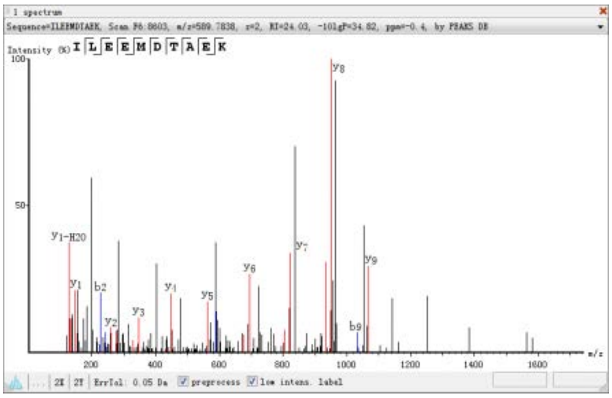

gi|225467859

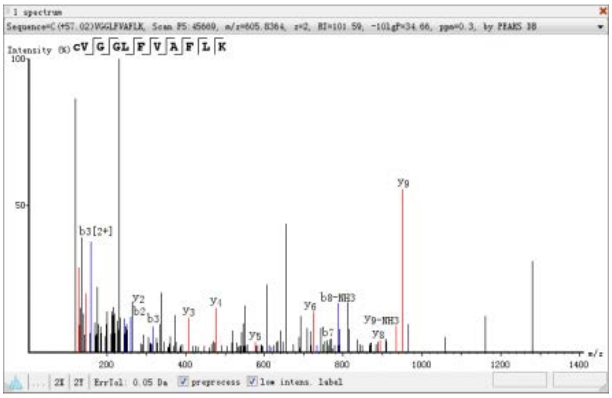

gi|225451663

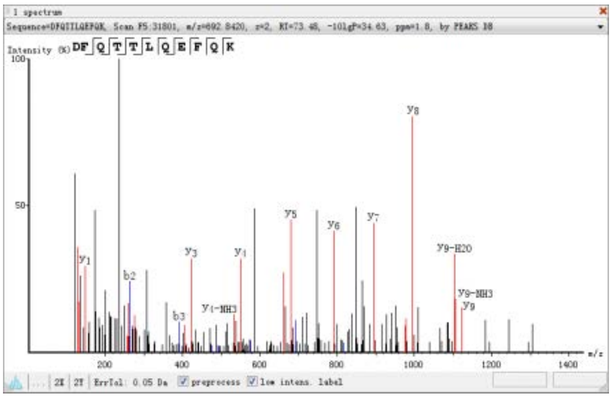

gi|225445842

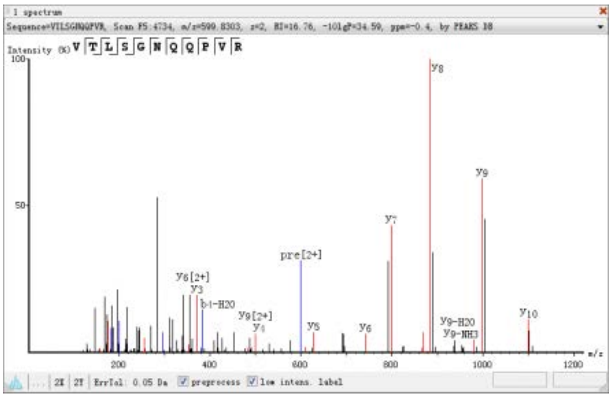

gi|566187444

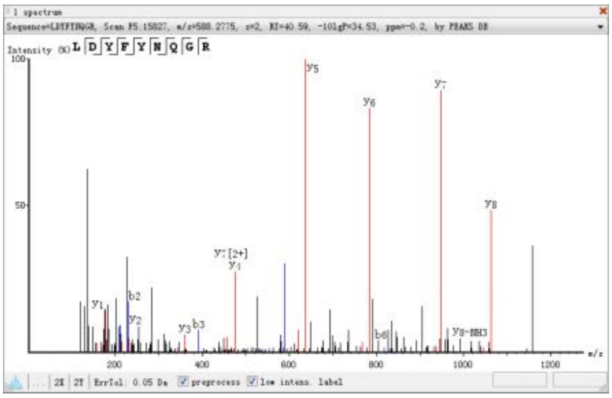

gi|566209115

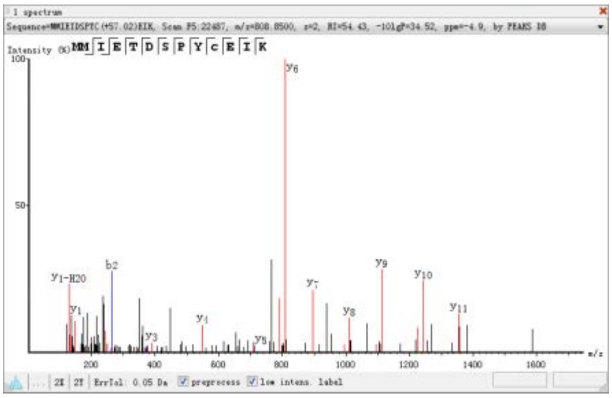

gi|566195508

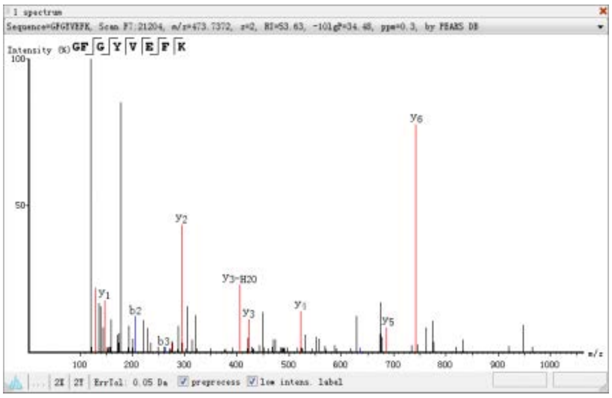

gi|224067716

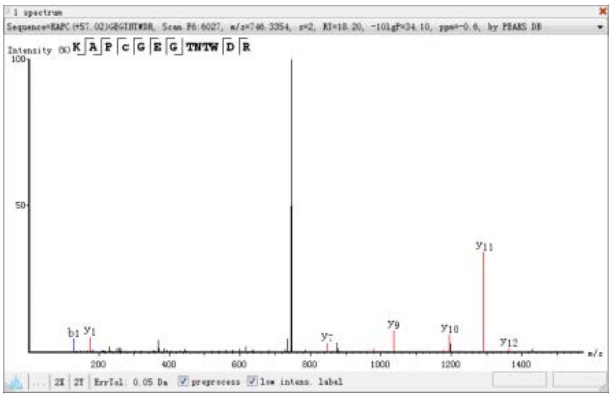

gi|566155545

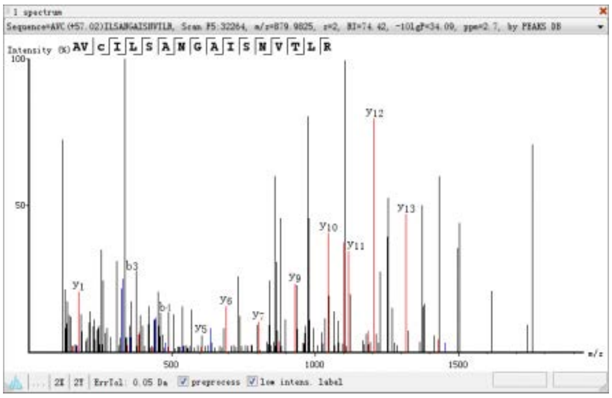

gi|566209274

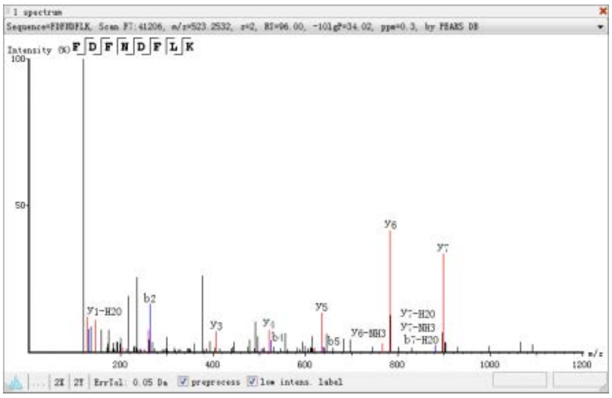

gi|225443988

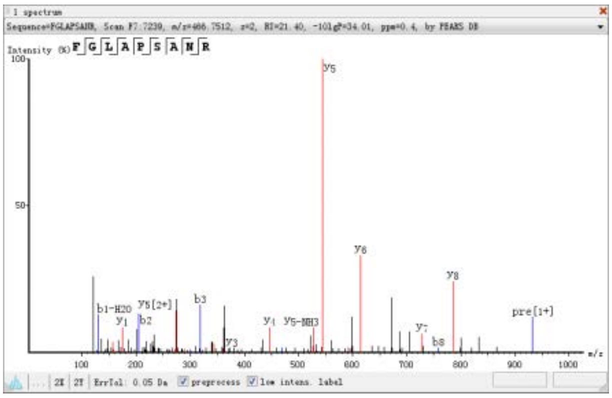

gi|224117028

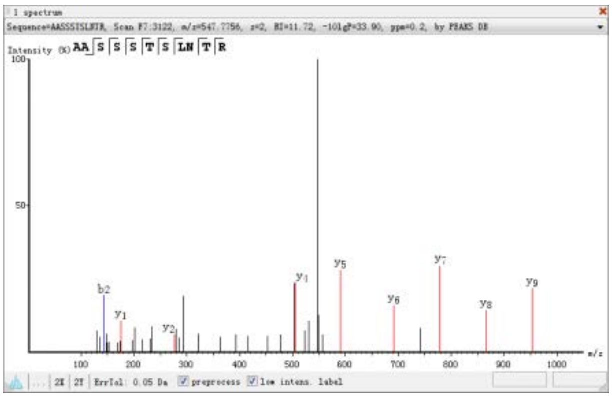

gi|731428892

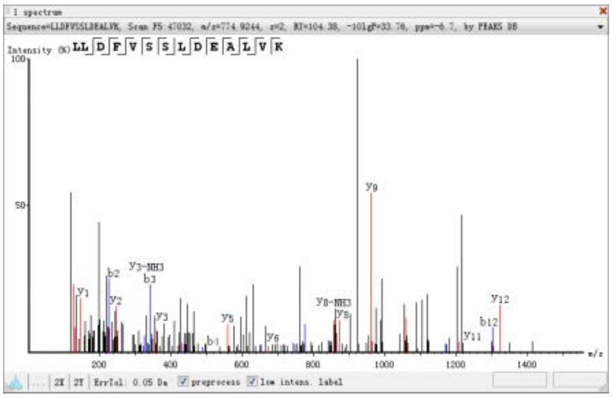

gi|731396112

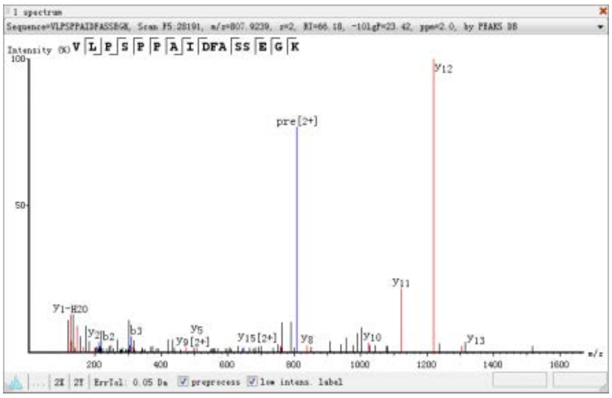

gi|566164165

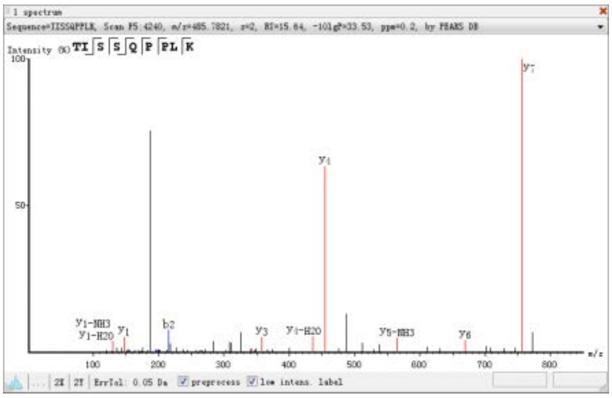

gi|225434205

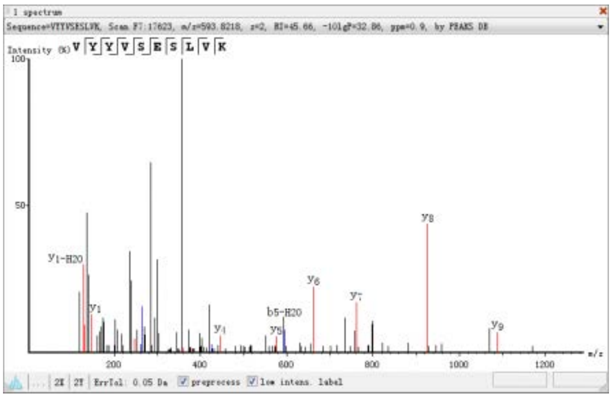

gi|566209622

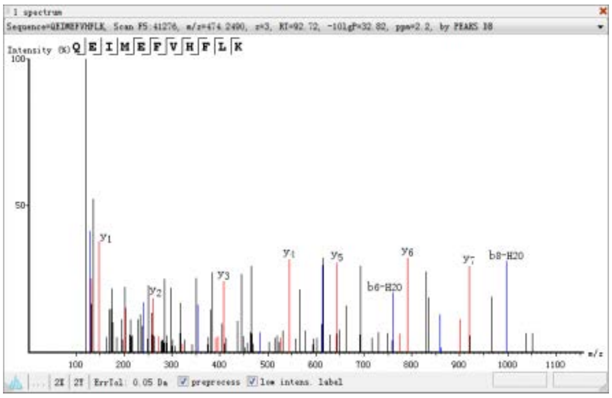

gi|566202105

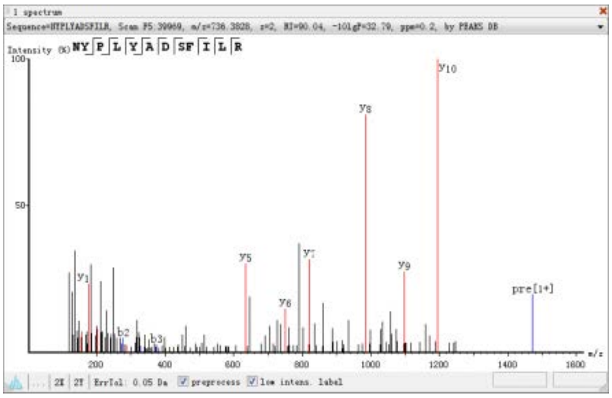

gi|224102881

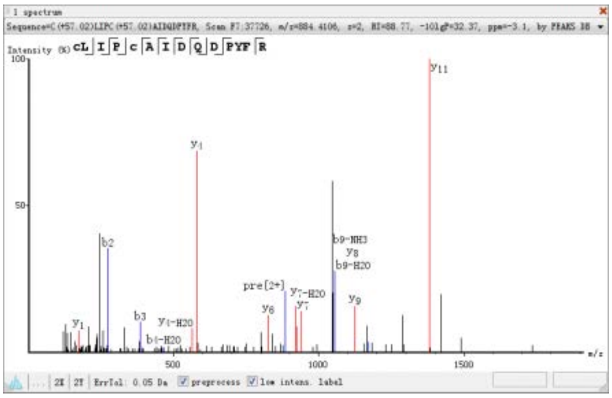

gi|225438579

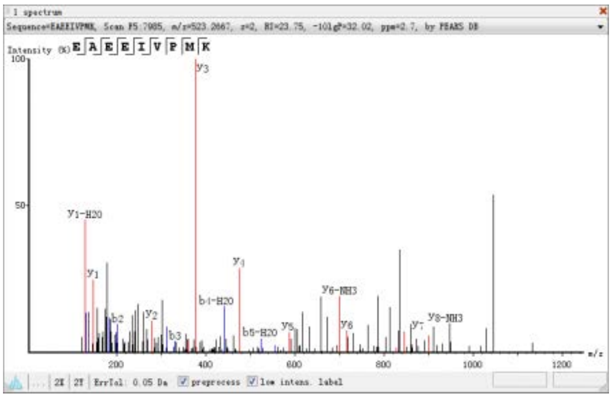

gi|224095855

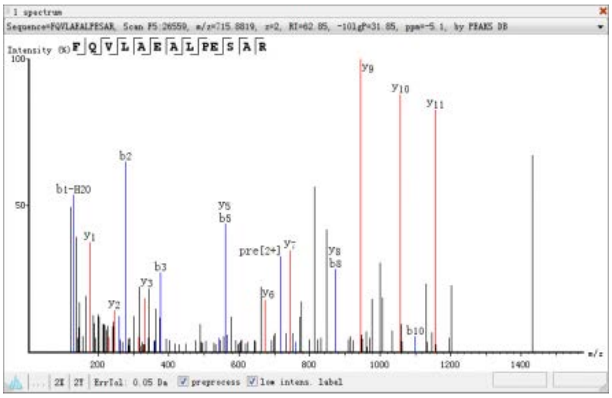

gi|225439352

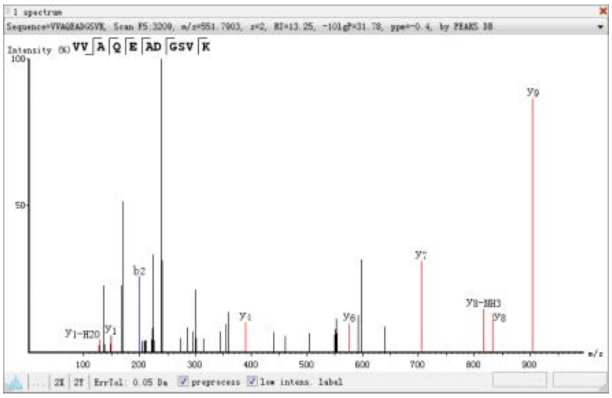

gi|566188099

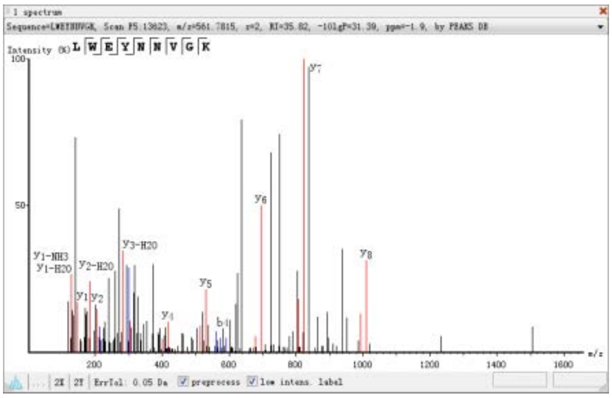

gi|731417542

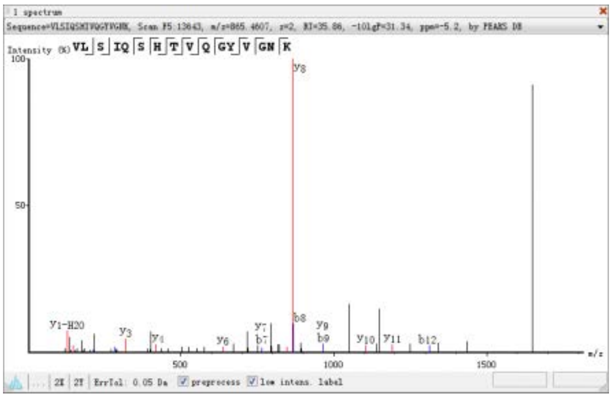

gi|224077516

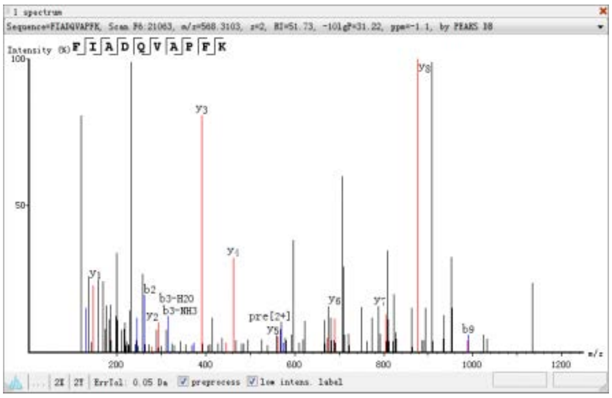

gi|225464240

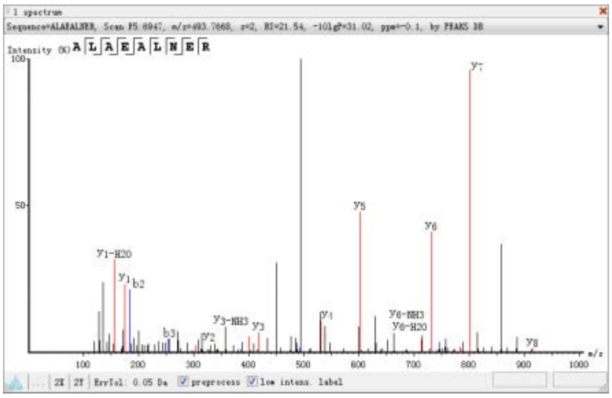

gi|225448041

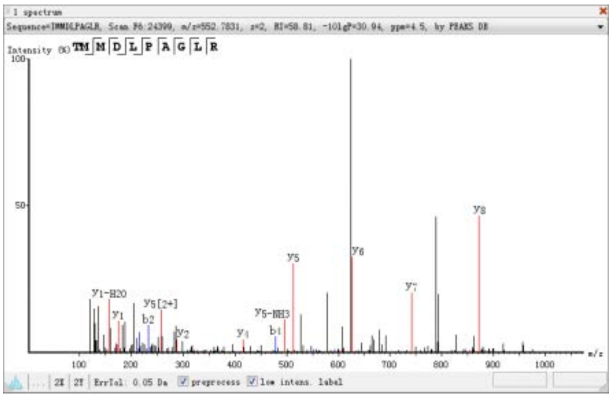

gi|224100723

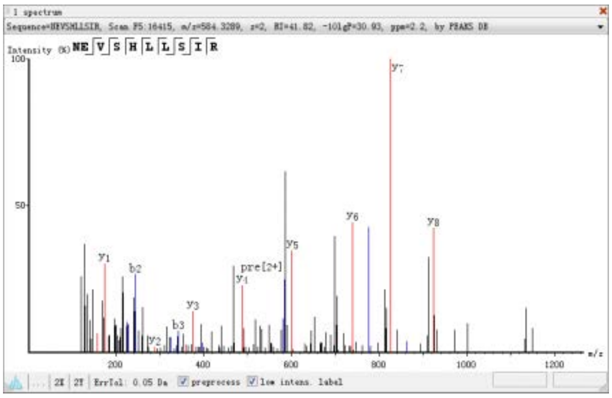

gi|731423901

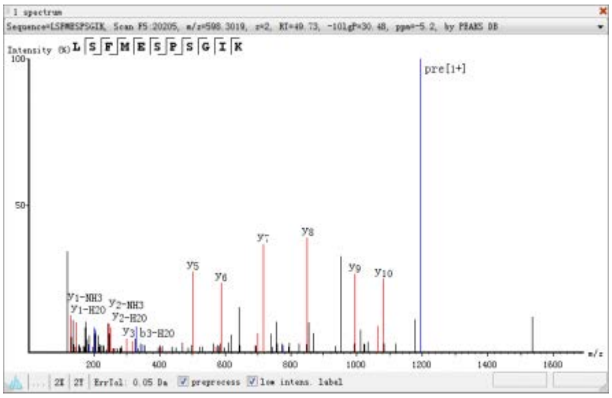

gi|359487708

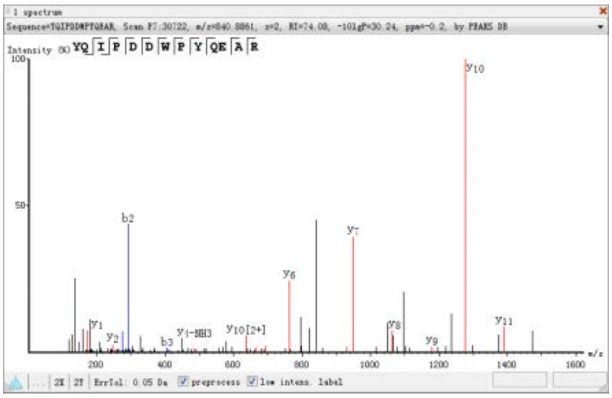

gi|566146741

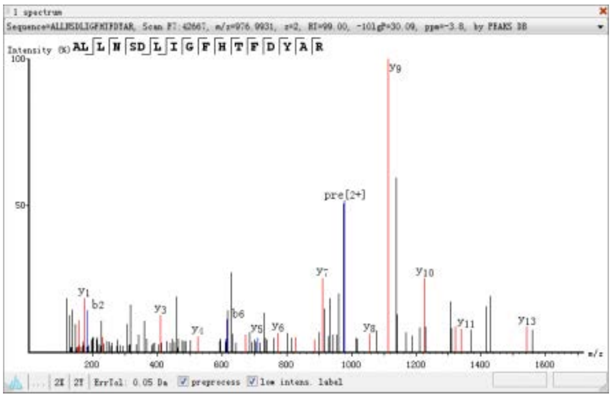

gi|224087965

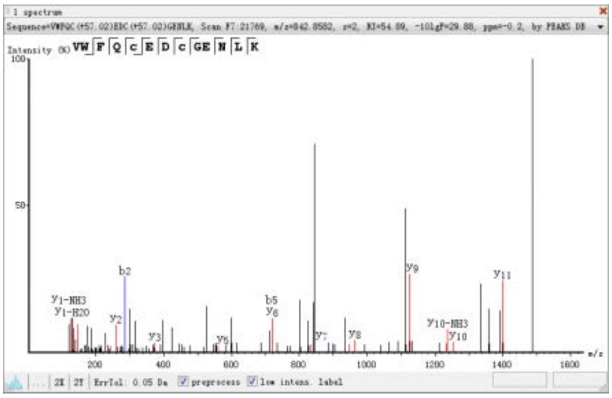

gi|566177300

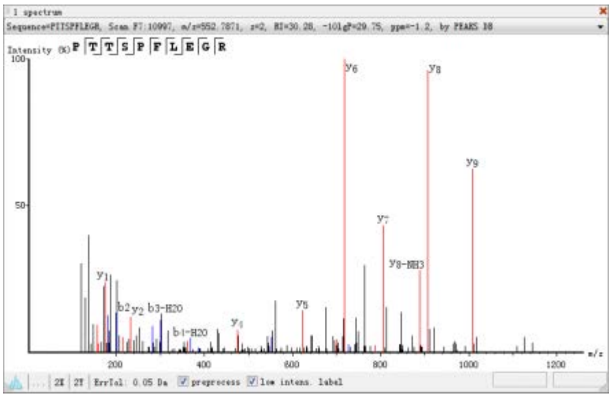

gi|225458599

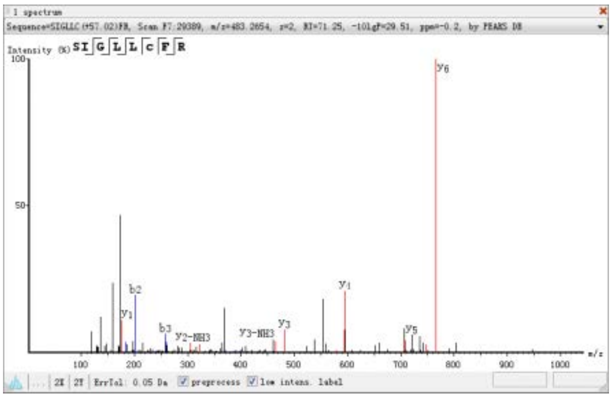

gi|359478392

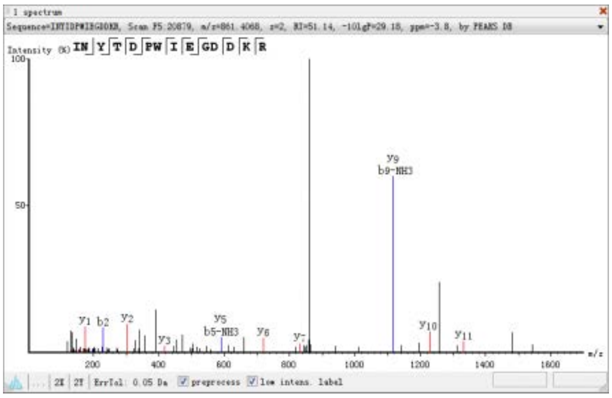

gi|225423716

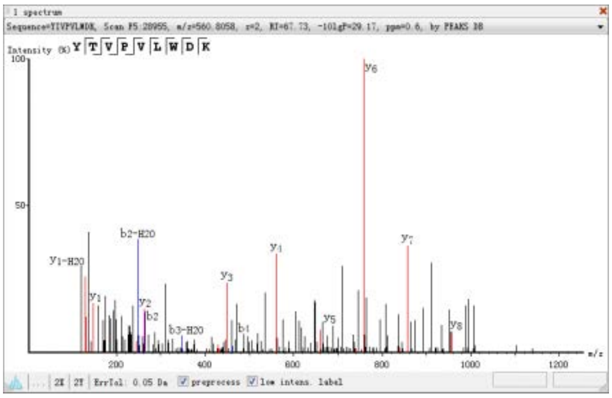

gi|225454892

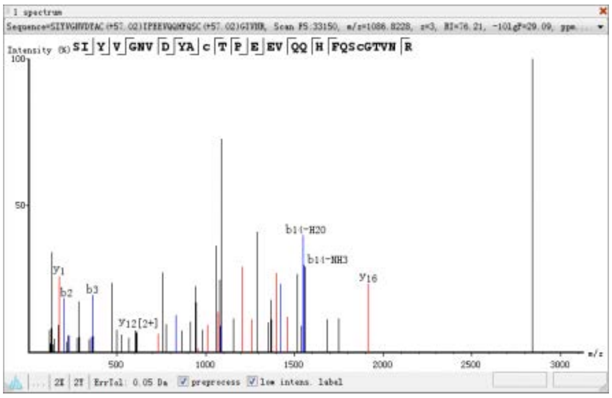

gi|225454791

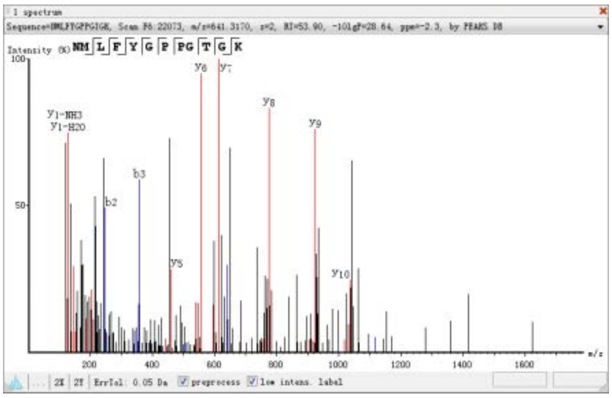

gi|225433514

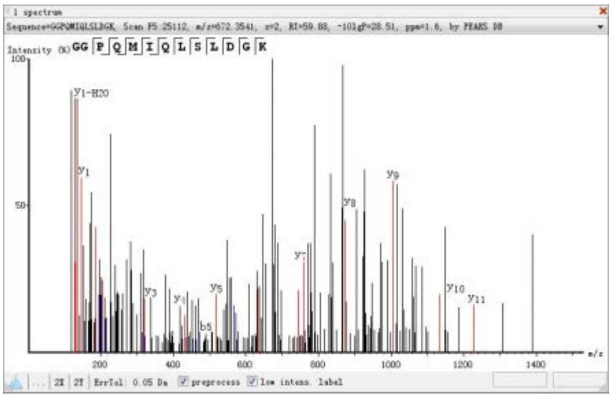

gi|731440611

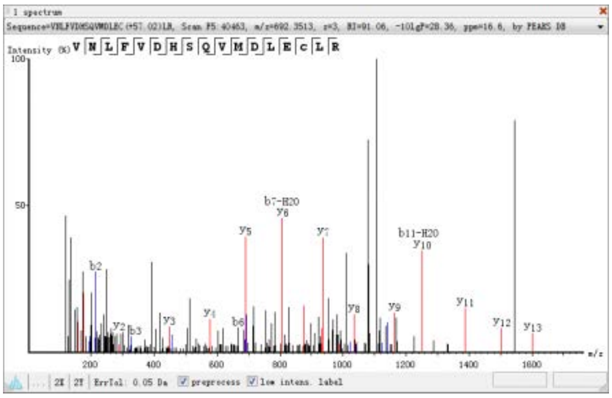

gi|359482034

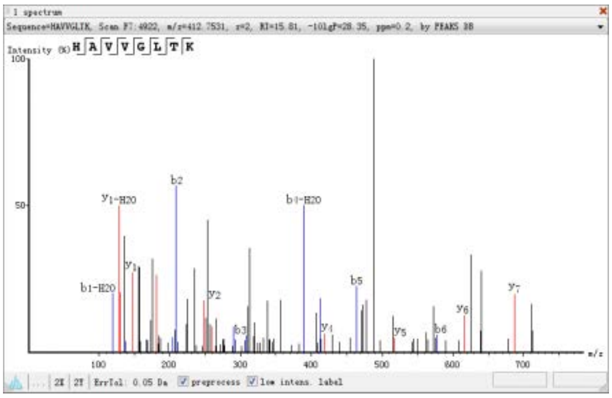

gi|566207206

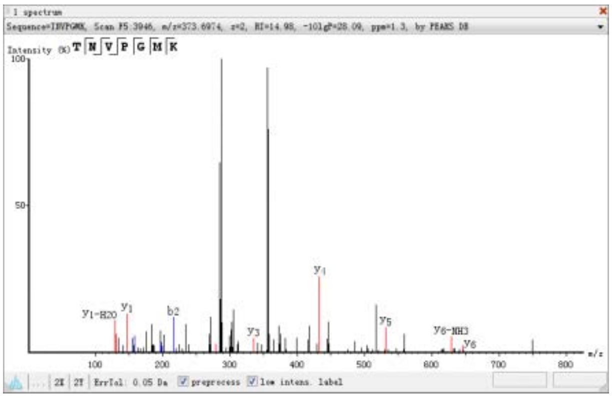

gi|225452543

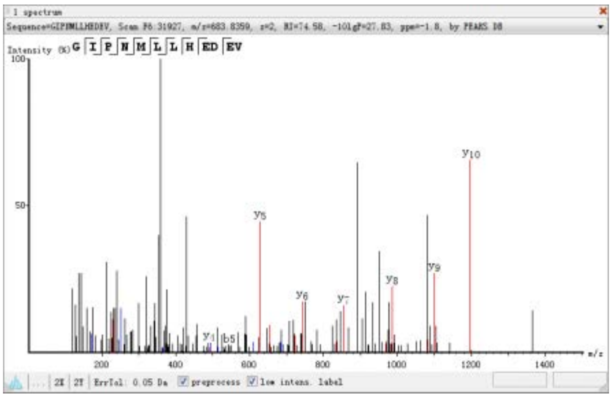

gi|731408402

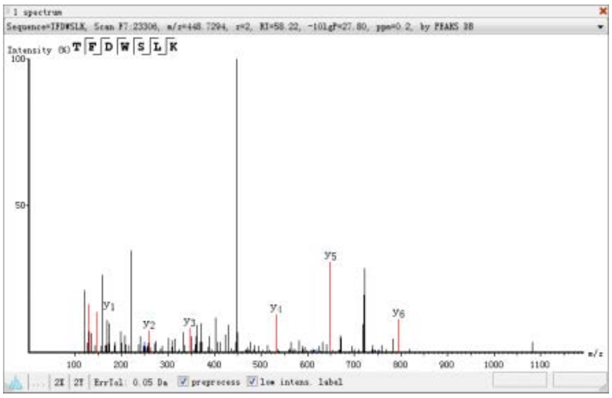

gi|225433902

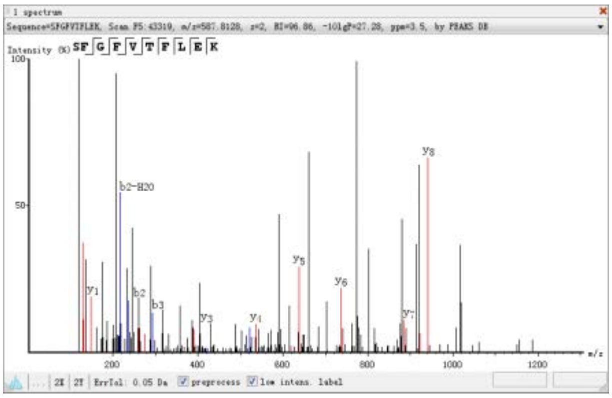

gi|566152952

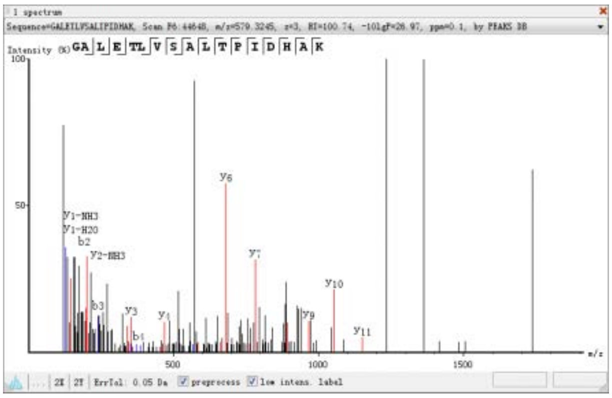

gi|225435189

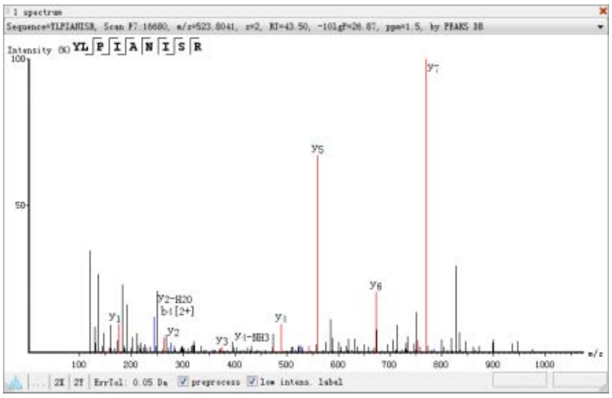

gi|566197842

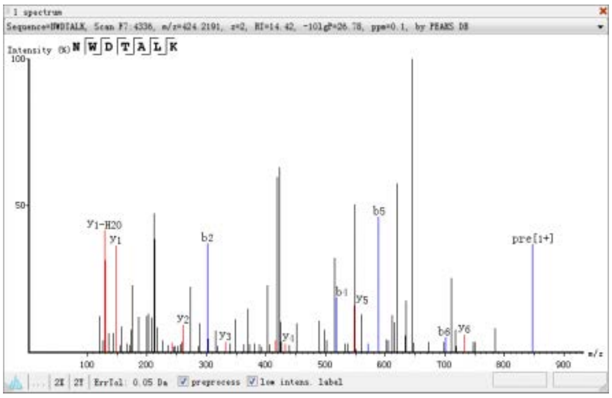

gi|225468608

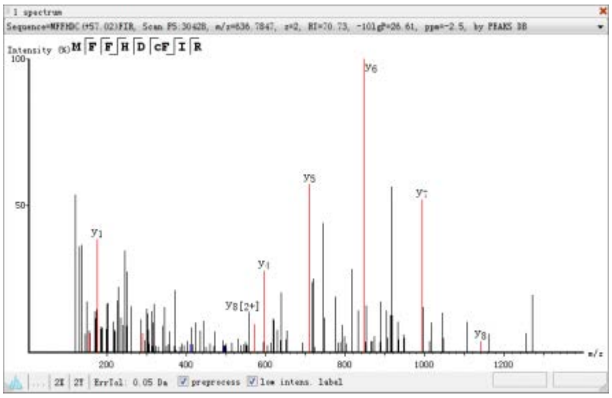

gi|225457225

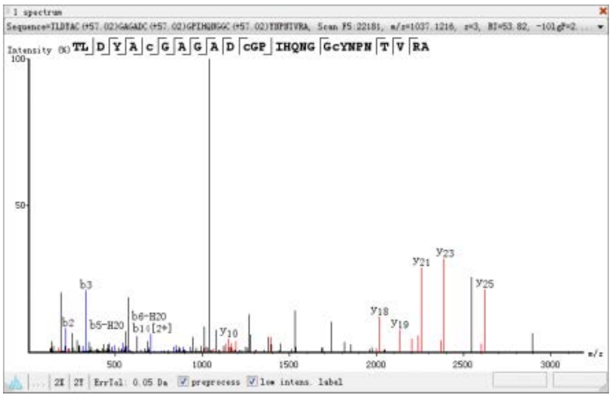

gi|566190046

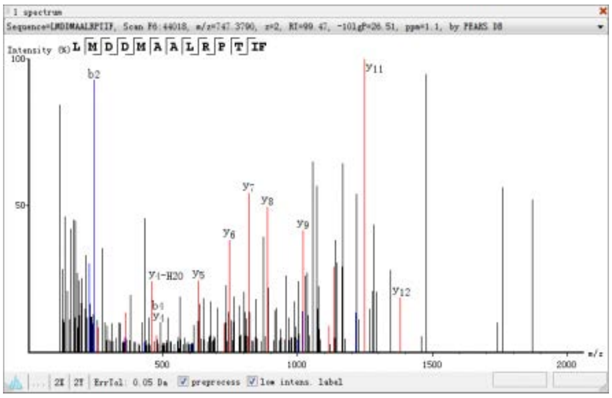

gi|731393820

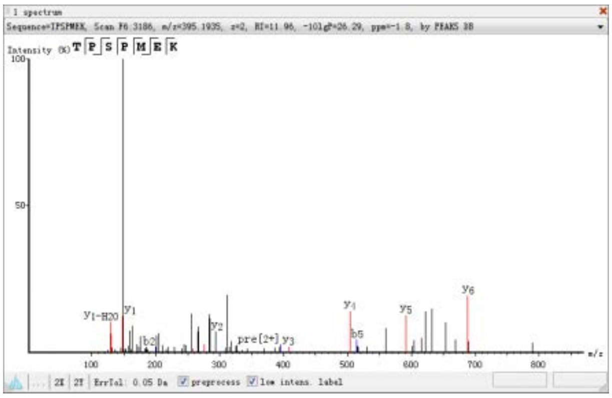

gi|566198361

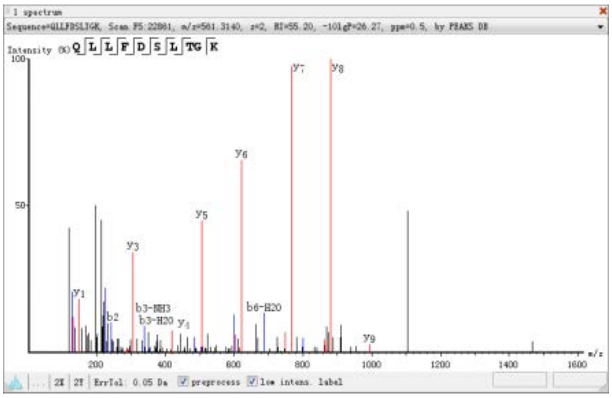

gi|566148044

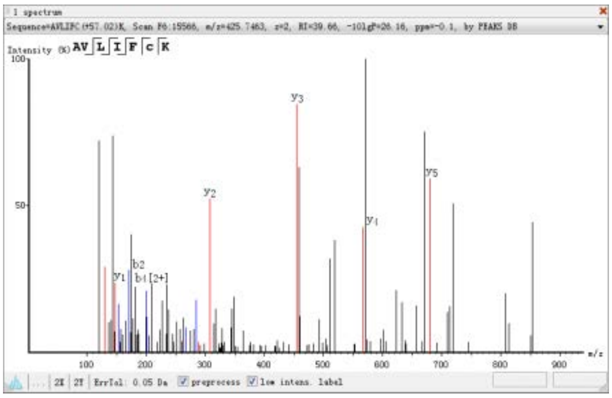

gi|224088794

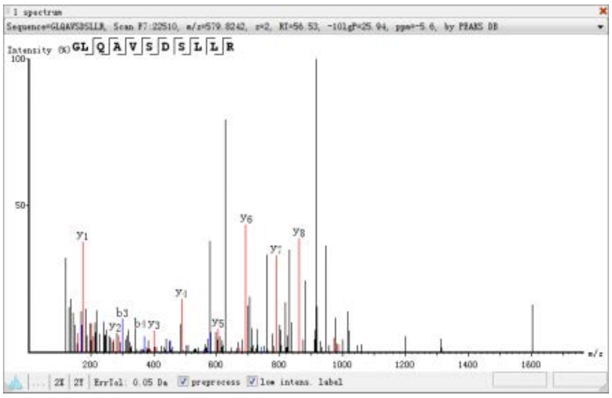

gi|566153219

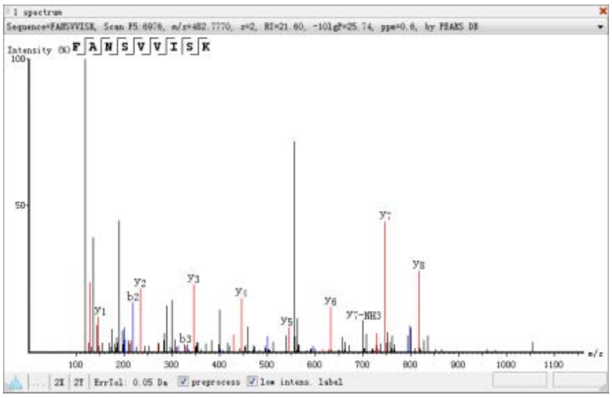

gi|225456165

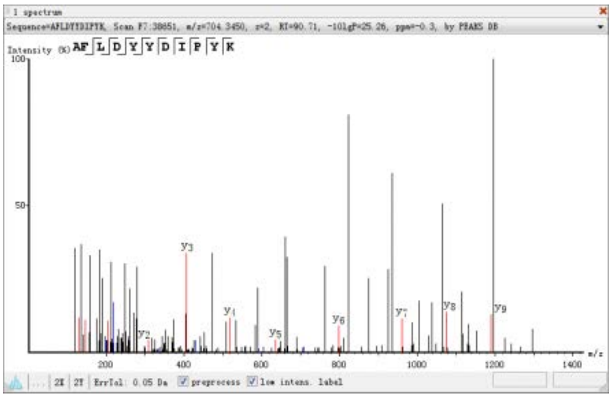

gi|224101571

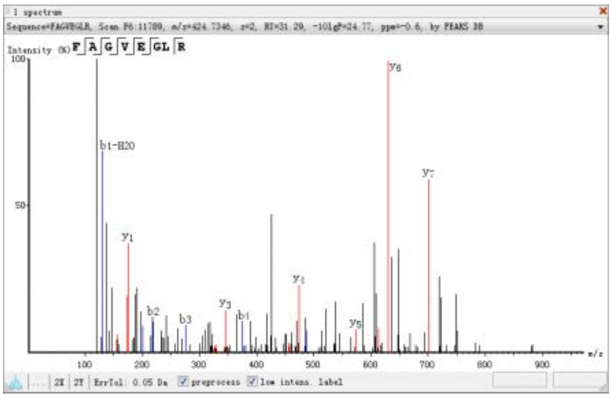

gi|225427112

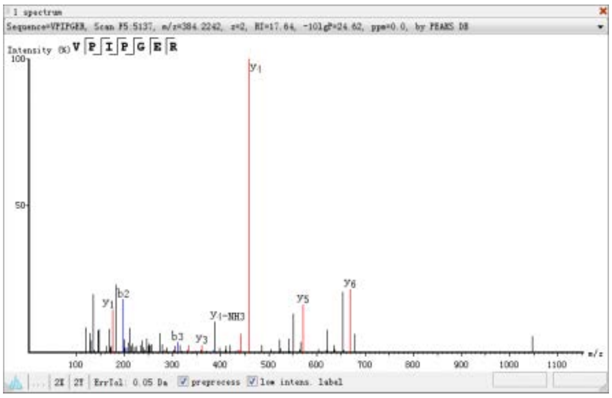

gi|224057220

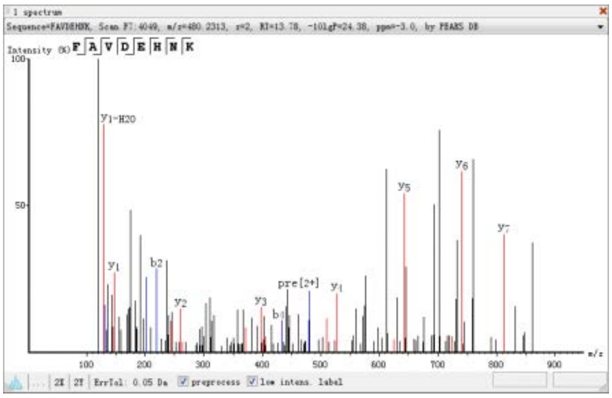

gi|731413580

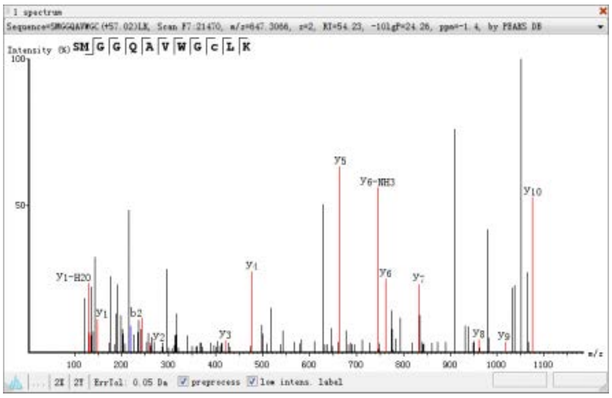

gi|225442791

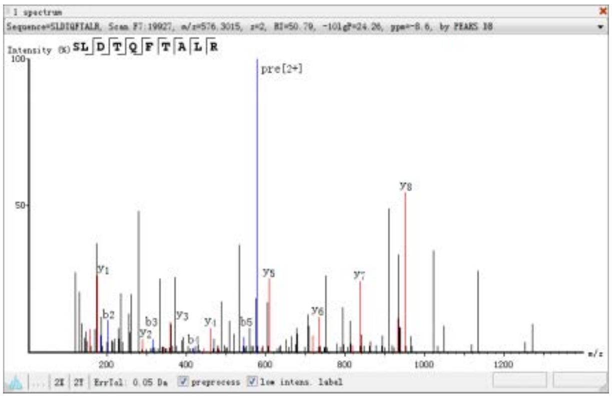

gi|225437318

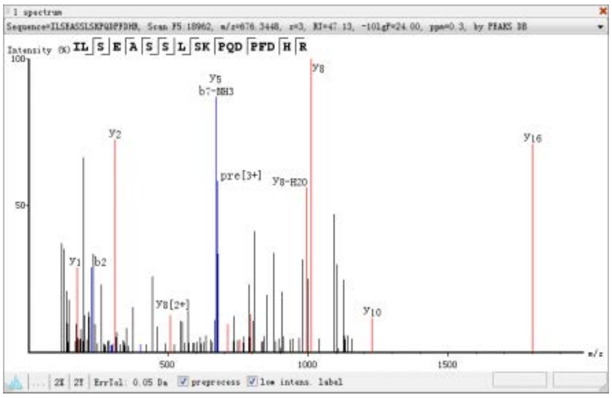

gi|225440807

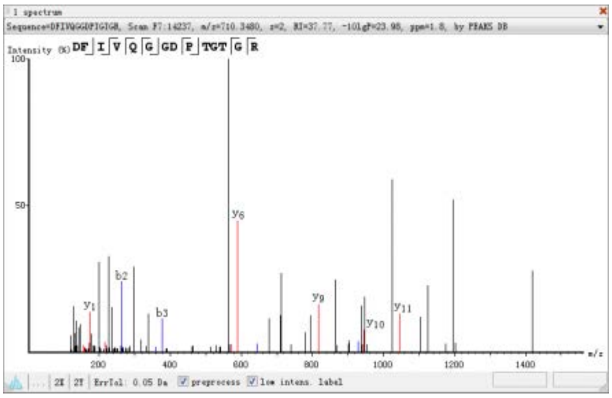

gi|225447498

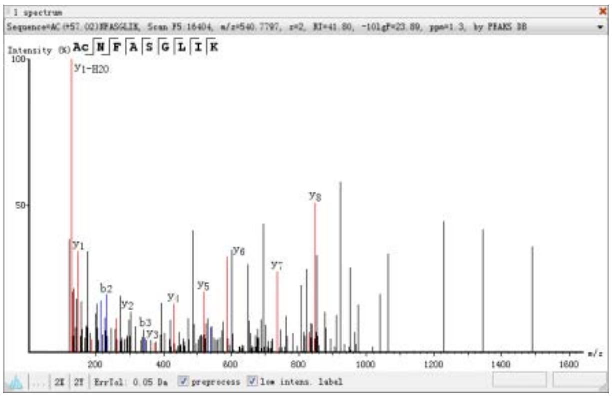

gi|731425525

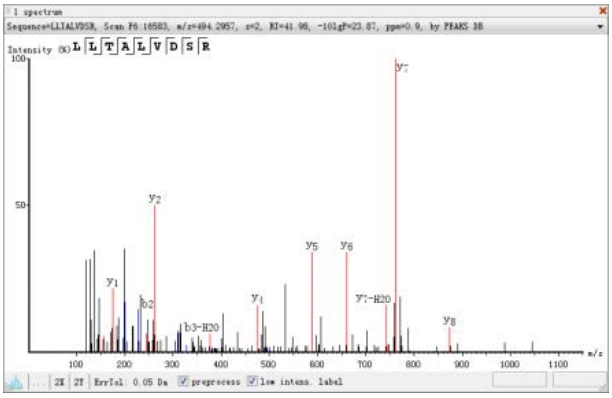

gi|566185108

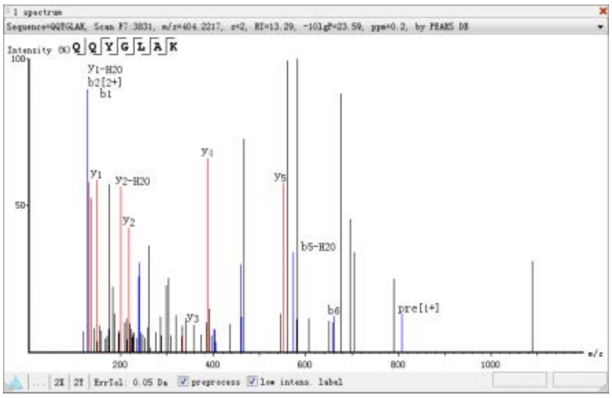

gi|731423435

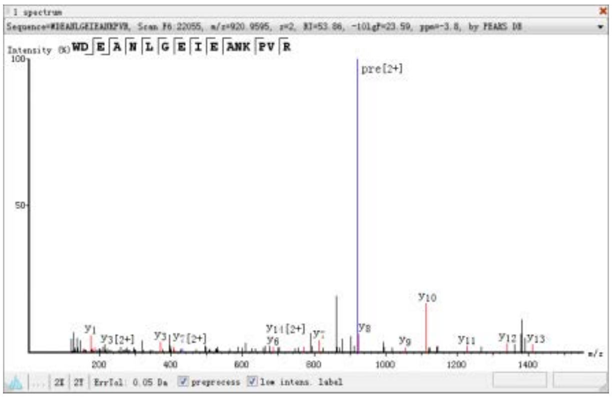

gi|225451245

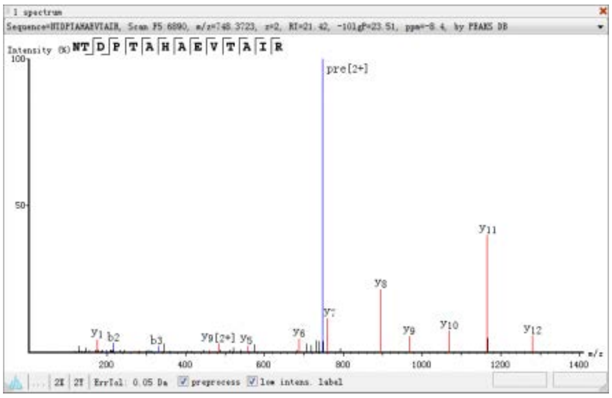

gi|225458143

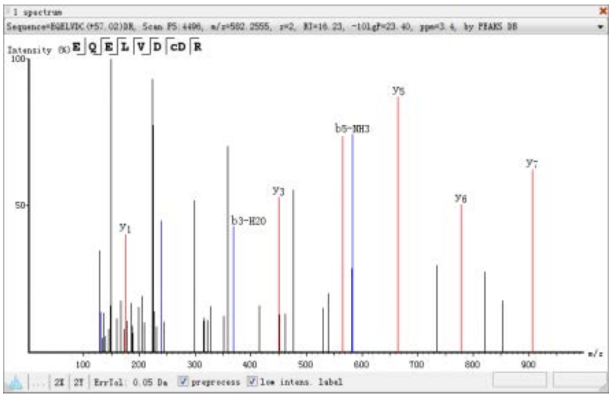

gi|225440692

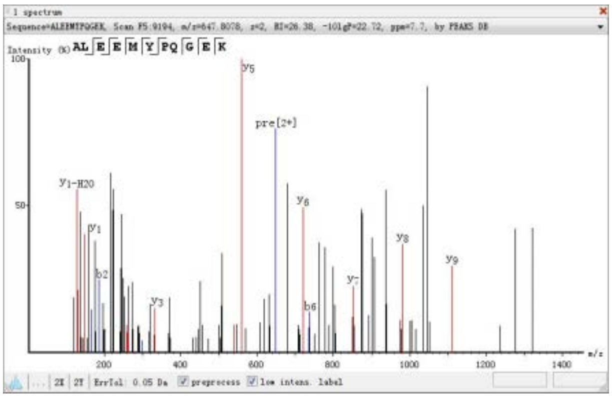

gi|566166782

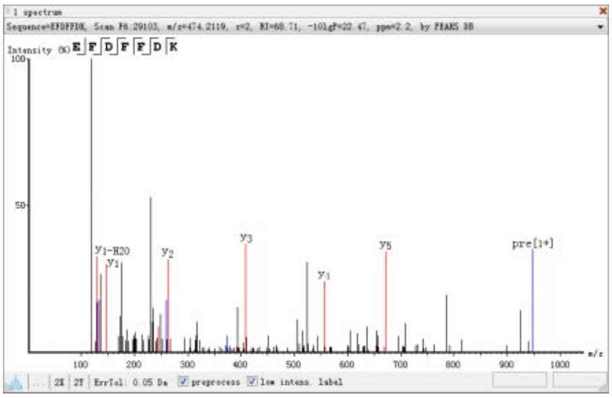

gi|566187962

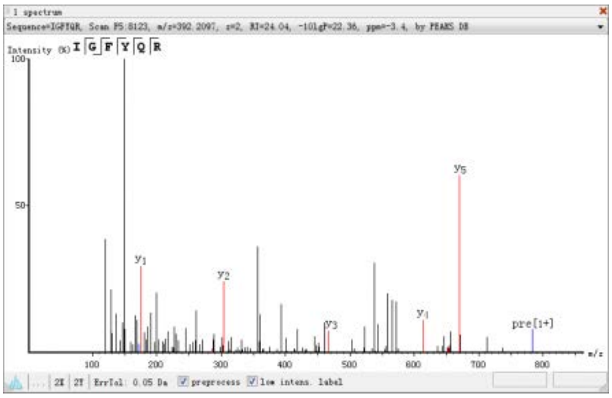

gi|566188564

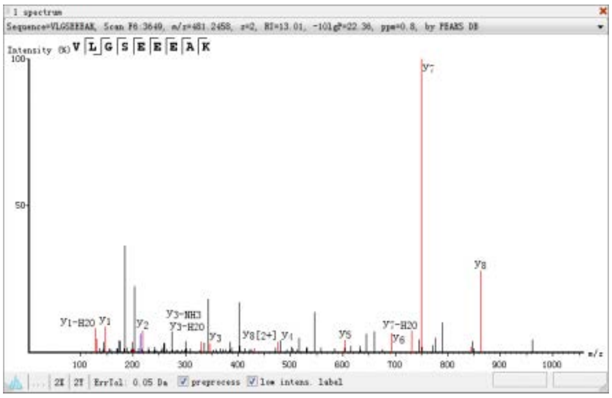

gi|566195960

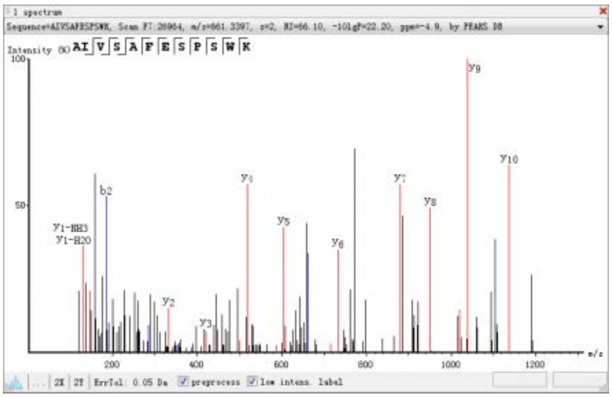

gi|731384376

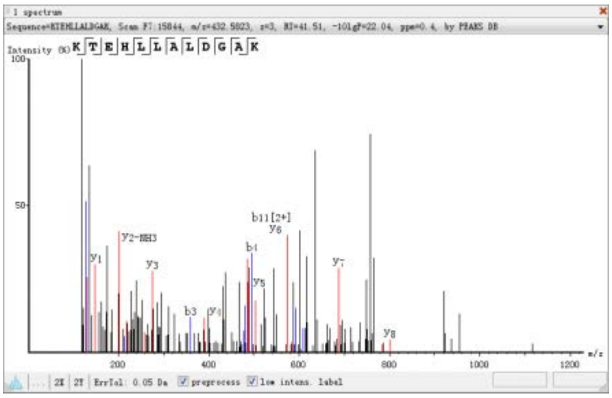

gi|566180229

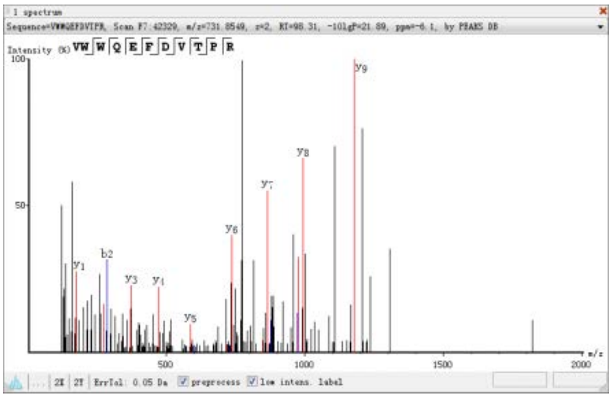

gi|359473301

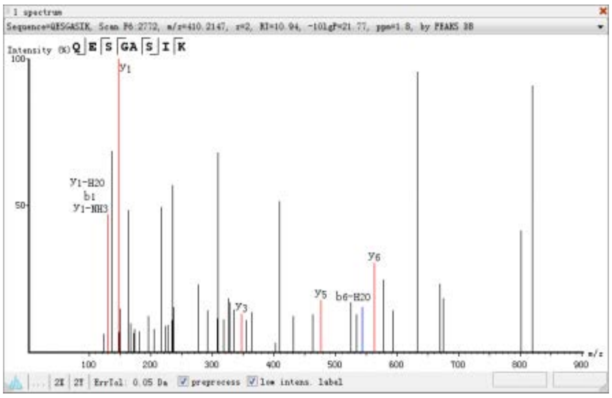

gi|225430352

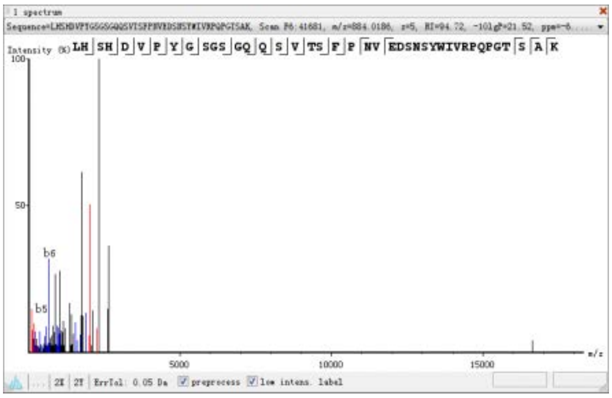

gi|225449250

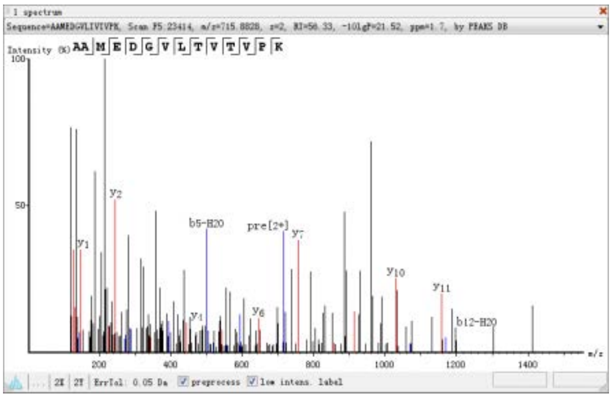

Fig.S5 The spectra of the proteins identified by one unique peptide with only one spectrum in HY2. Upper is the accession of protein, and under is its corresponding spectrum.

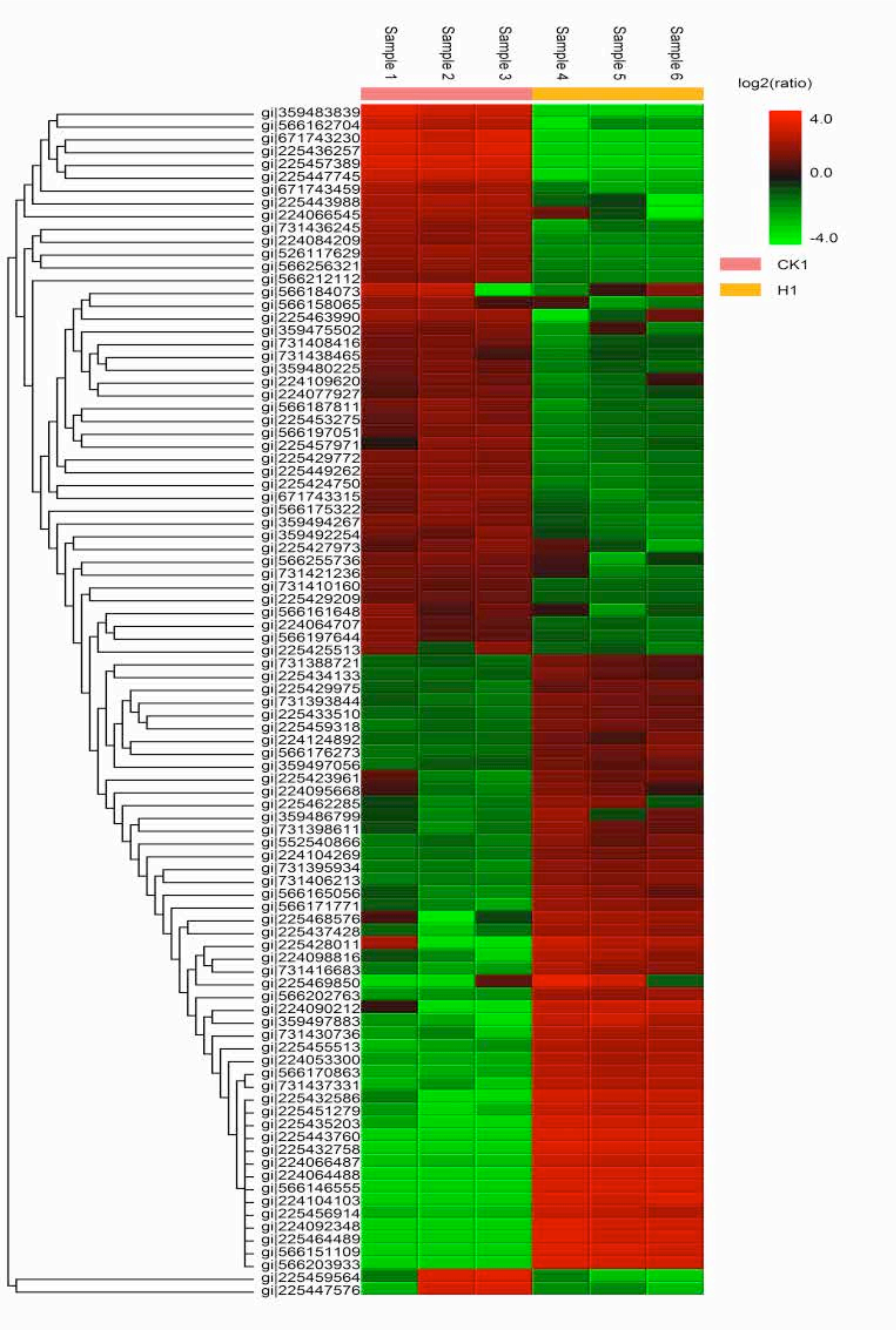

Fig. S6 Heat map of the differentially expressed proteins in the comparison of CK1 and HY1. Top, sample; left, protein tree. The color scale bar in the right indicates increased (red) and decreased (green) levels.

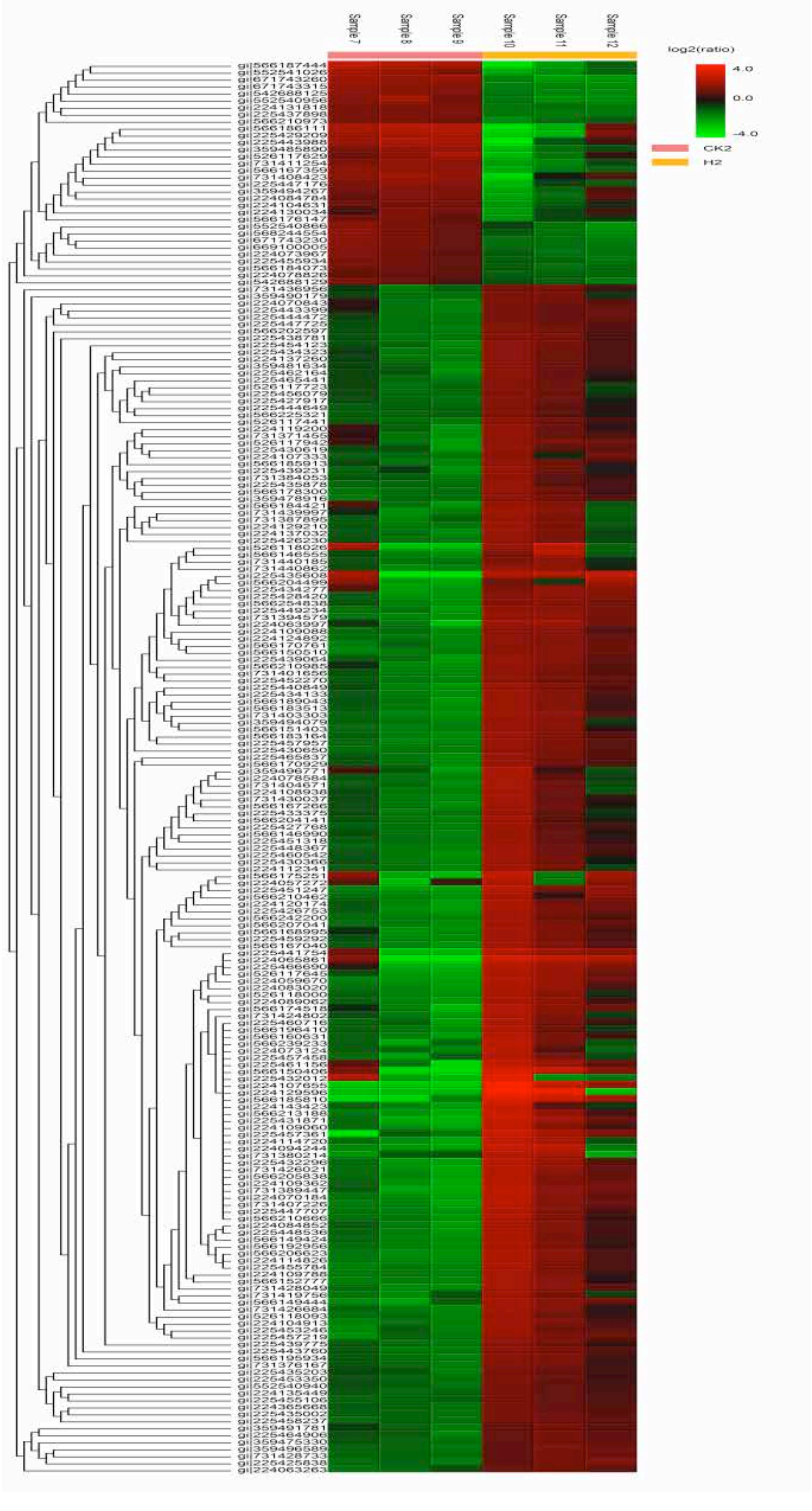
Sample 7
Sample 8
Sample 9
Sample 10
Sample 11
Sample 12
log2(ratio)
4.0
0.0
-4.0
CK2
H2

Fig. S7 Heat map of the differentially expressed proteins in the comparison of CK2 and HY2. Top, sample; left, protein tree. The color scale bar in the right indicates increased (red) and decreased (green) levels.

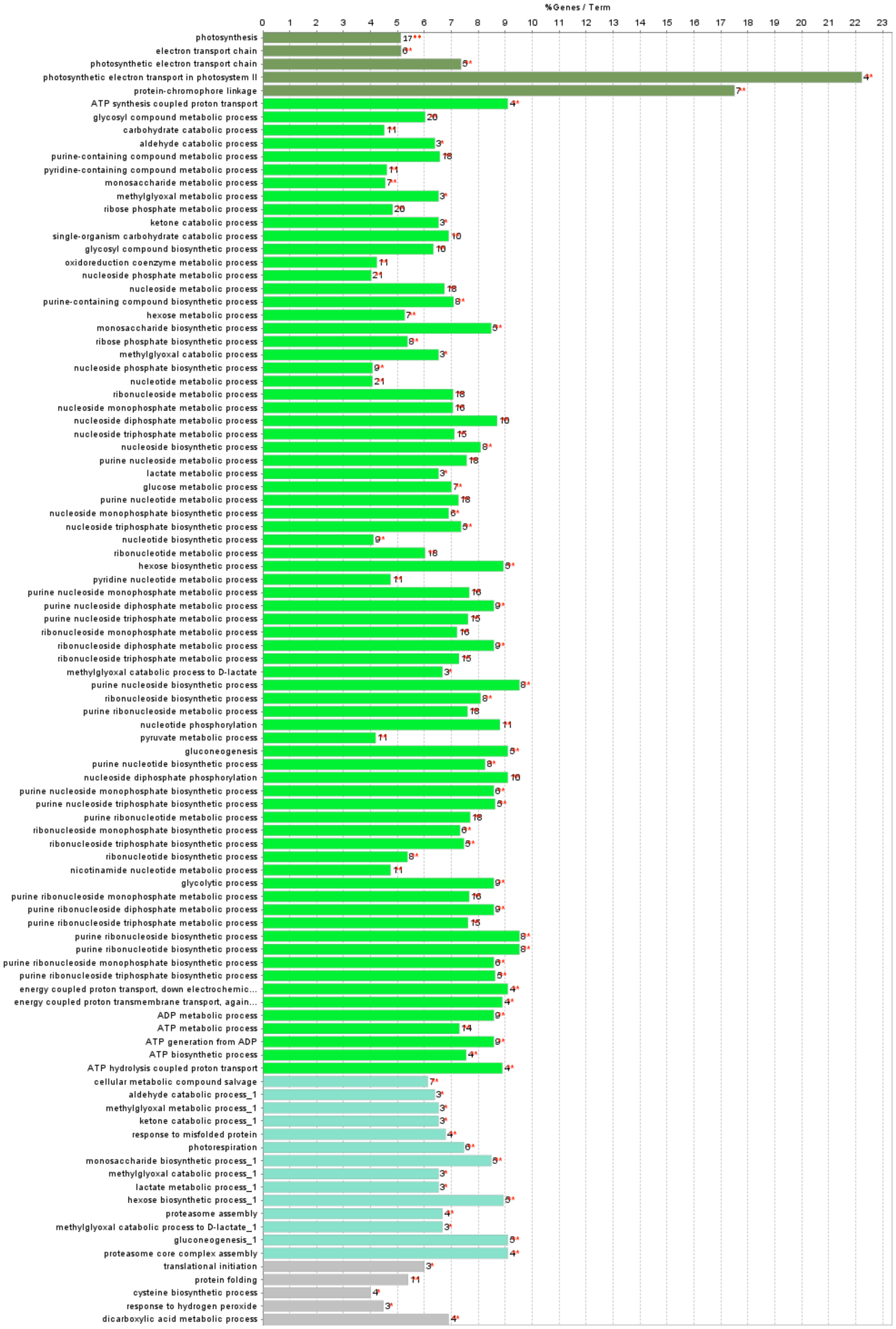

Fig. S8 Enriched GO terms of differentially expressed proteins identified from the comparison of HY2 and CK2.
